# Milk and mucin glycans orchestrate a synthetic infant gut microbiota structure

**DOI:** 10.1093/femsec/fiaf069

**Published:** 2025-06-25

**Authors:** Maryse D Berkhout, Athanasia Ioannou, Yuvashankar Kavanal Jayaprakash, Caroline M Plugge, Clara Belzer

**Affiliations:** Laboratory of Microbiology, Wageningen University and Research, Stippeneng 4, 6708 WE, Wageningen, The Netherlands; Laboratory of Microbiology, Wageningen University and Research, Stippeneng 4, 6708 WE, Wageningen, The Netherlands; Laboratory of Microbiology, Wageningen University and Research, Stippeneng 4, 6708 WE, Wageningen, The Netherlands; Laboratory of Microbiology, Wageningen University and Research, Stippeneng 4, 6708 WE, Wageningen, The Netherlands; Laboratory of Microbiology, Wageningen University and Research, Stippeneng 4, 6708 WE, Wageningen, The Netherlands

**Keywords:** human milk oligosaccharides, mucin, infant gut microbiota synthetic community, *Akkermansia muciniphila*, niche segregation, cross-feeding interactions

## Abstract

Glycans are crucial for infant gut microbiota development. Human milk contains prebiotic human milk oligosaccharides (HMOs) that stimulate gut microbes. Simultaneously, the glycan-rich mucus layer develops and attracts mucin glycan-degrading bacteria. As HMOs and mucin are degraded by homologous enzymes, bacterial glycan-degrading abilities overlap. However, less is known about how infant gut microbial communities form when both types of glycans are available. To study this, we created a synthetic community with specialist glycan degraders and cross-feeders from the infant gut (BabyBac). We evaluated it in different *in vitro* conditions including combinations of diet-derived [HMOs, galactooligosaccharides (GOS), and fructooligosaccharides (FOS)] and mucus glycans. Glycan combinations significantly affected the community composition and metabolic output. The glycan type affected the overall community, with mucin and HMOs being the top drivers of variation. HMOs favoured glycan degraders and cross-feeders, whereas mucin glycan degrader *Akkermansia muciniphila* was outcompeted. Conversely, when mucin was present, *A. muciniphila* thrived. Addition of mucin monomers and 2′-FL to GOS/FOS did not reinstate *A. muciniphila* abundance. This suggests that *A. muciniphila* cannot compete with infant-related bacteria without the complete mucin structure. Overall, our findings suggest that the interplay between dietary and mucus glycans creates niche differentiation in the infant gut microbiota.

## Teaser

Dietary and gut glycans drive the composition of an *in vitro* synthetic community of infant gut microbes.

## Introduction

The first months of life form a critical window for the establishment of the gut microbiota and consequently for infant health. Many factors such as the birth mode (Bäckhed et al. 2015, Martin et al. 2016) and gestational age (Korpela et al. 2018a, Hill et al. 2017, Aguilar-Lopez et al. 2021) affect the composition of the gut microbiota in the first days of life, mainly through alternate seeding routes (Korpela et al. 2018b). As time passes, both diet-derived and host-derived glycans play an important role in this process, as feeding mode is a key factor that shapes the infant gut microbiota (Bäckhed et al. 2015, Martin et al. 2016). The development of the gut mucus layer occurs simultaneously with the establishment and maturation of the mucosal microbiome (Rokhsefat et al. 2016). Therefore, the developing glycan landscape of the infant gut plays a role in shaping the succession of microbial communities that occurs at the infant gut mucus layer.

A major source of dietary glycans in the first months of life is human milk. Human milk oligosaccharides (HMOs) are a key component of human milk. They are not digested by the infant, and therefore reach the lower gut (Engfer et al. 2000). HMOs are structurally diverse glycans consisting of glucose (Glc), galactose (Gal), *N*-acetylglucosamine (GlcNAc), fucose (Fuc), and sialic acid (Neu5Ac) (Wu et al. 2010, Urashima et al. 2017). The core structure of HMOs is lactose (Gal-β1,4-Glc), which can be extended with lacto-*N*-biose (Gal-β1,3-GlcNAc) or *N*-acetyllactosamine (Gal-β1,4-GlcNAc). HMO structural profiles vary greatly among mothers and are determined by a range of factors including genetics, stage of lactation, and geographic location (Thurl et al. 2017, Azad et al. 2018, Soyyilmaz et al. 2021). In the developing infant gut, HMOs function as prebiotics, which results in stimulation of glycan-degrading microbiota including *Bifidobacterium* spp. and *Bacteroides* spp. (De Leoz et al. 2015, Jost et al. 2015). When exclusive breastfeeding is not possible, infants are also introduced to infant formula, which contains added glycans like galactooligosaccharides (GOS) and fructooligosaccharides (FOS). These are added to steer gut microbiota composition to be more similar to that of breastfed infants because these glycans stimulate bifidobacterial growth (Sims and Tannock 2020) and lower fecal pH (Bakker-Zierikzee et al. 2005), among others.

**Figure 1.**
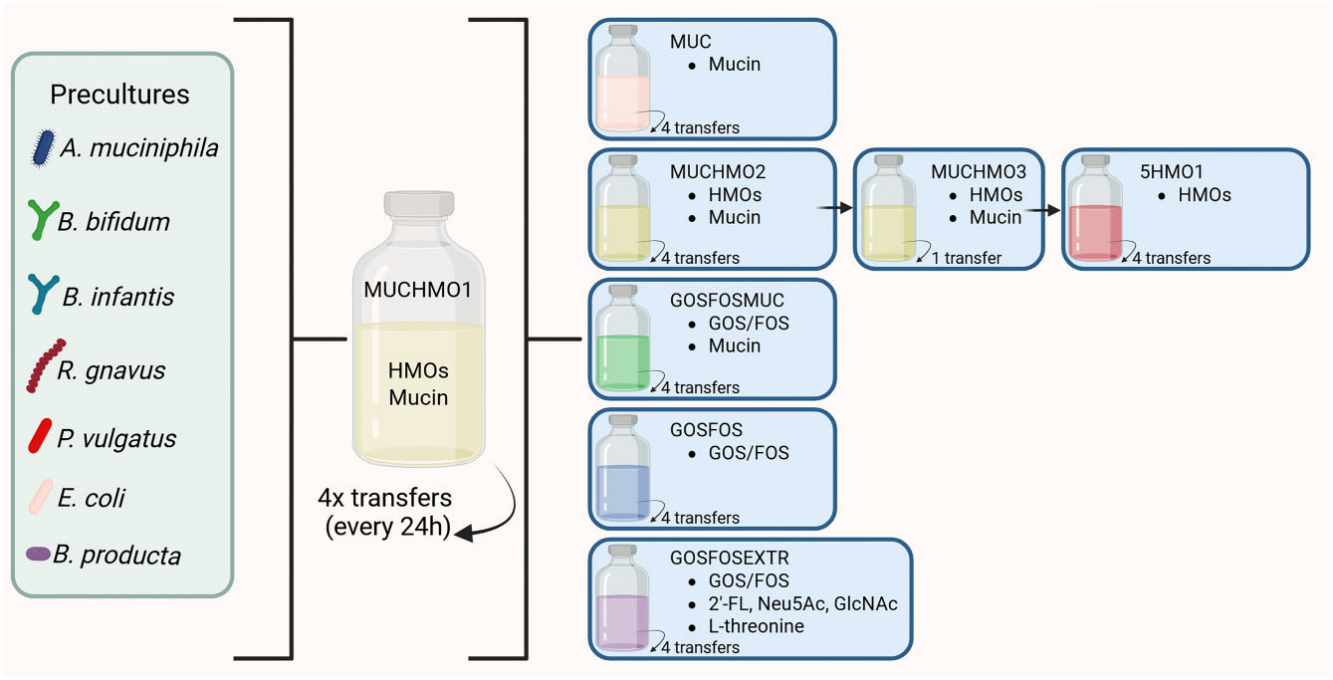
Study set-up and conditions. Precultures of each strain were mixed together in normalized OD:1 to prepare the inoculum for the *in vitro* fermentations. The community inoculum was first grown in mucin and HMOs (MUCHMO1) in triplicate. After four transfers every 24 h, the culture was used to inoculate triplicates of the other conditions (MUC, MUCHMO2, GOSFOS, GOSFOSMUC, and GOSFOSEXTR). The MUCHMO2 culture was revived in mucin and HMO (MUCHMO3) and was used as inoculum for 5HMO1. Each condition (apart from MUCHMO3) was cultured for 120 h, which included four transfers every 24 h. Created in BioRender. Microbiology, W. (2024) https://BioRender.com/l51lex2

In addition to diet-derived glycans, the abundance of host-derived mucin glycans increases during infant development (Wells et al. 2022). Mucin glycans form the mucus layer that shields the intestinal epithelium from contact with the gut microbiota. Interestingly, human milk also contains mucins, which share structural similarities with HMOs (Fig. 1), namely MUC1 and MUC4 (Liu and Newburg 2013). Human intestinal mucin glycans are glycoproteins that are extensively *O*-glycosylated at serine (Ser) or threonine (Thr) residues of the core protein. The glycan extensions are structurally diverse and consist of Gal, GlcNAc, *N*-acetylgalactosamine (GalNAc), Fuc, Neu5Ac, and sulfate (Brockhausen et al. 2009). Certain bacteria such as *Akkermansia muciniphila* and *Bacteroides thetaiotaomicron* can use mucin glycans as a sole carbon source and inhabit the outer mucus layer (Tailford et al. 2015).

The dietary and gut-derived glycans, available in the infant gut, have structural similarities. Mucin and HMOs share four monomers (Gal, GlcNAc, Neu5Ac, and Fuc) and multiple intermediate structures. Therefore, some gut bacteria employ similar enzymatic machineries to target both glycans, including galactosidases, sialidases, and fucosidases. Bacteria that degrade both glycans include *A. muciniphila* and *Bifidobacterium bifidum*, among others (Ruas-Madiedo et al. 2008, Marcobal et al. 2011, Kostopoulos et al. 2020). Additionally, some of those enzymes are utilized to degrade other indigestible glycans from complimentary feeding, such as beta-galactosidases acting on GOS (Ioannou et al. 2021). The ability to degrade a wide range of glycan structures aids bacteria to compete and adapt to the developing infant gut environment. However, it is not completely understood how the glycan landscape of the developing gut, from the lumen to the mucosal layer, steers the establishment of microbial communities.

To address this in the context of a complex community, we designed BabyBac, a synthetic community of seven individual strains. BabyBac includes bacteria that are commonly found in the gut of infants within the first year of life. All strains included can degrade dietary glycans and mucin glycans either as specialists or as generalists, or cross-feed on the products of glycan degradation. We hypothesized that this would create trophic interactions between bacteria that would lead to cooperation or competition within BabyBac. Therefore, we subjected BabyBac to sequential batch fermentations with different combinations of diet- and host-derived glycans. The results of our experiments showed differences in terms of microbial composition and metabolite production that were driven by the presence and amount of mucin but also by the presence of HMOs. With this, we propose that gradients of dietary and mucin glycans, along the mucus layer and throughout milk feeding period drive competition and cooperation, and create specific niches for the maturing infant gut microbiota.

## Materials and methods

### Anaerobic fermentations

#### Medium composition

Strains were cultivated in a bicarbonate-buffered anoxic medium as described before (Plugge 2005), and contained per l 0.5 mg resazurin, 0.408 g KH_2_PO_4_, 0.534 g Na_2_HPO_4_.2H_2_O, 0.3 g NH_4_Cl, 0.3 g NaCl, and 0.1 g MgCl_2_.6H_2_O, modified by the addition of 1 g/l yeast extract. To each l, 1 ml of acidic trace elements (50 mM HCl, 1 mM H_3_BO_3_, 0.5 mM MnCl_2_, 7.5 mM FeCl_2_, 0.5 mM CoCl_2_, 0.1 mM NiCl_2_, 0.5 mM ZnCl_2_, and 0.1 mM CuCl_2_) and 1 ml of basic trace elements (10 mM NaOH, 0.1 mM Na_2_SeO_3_, 0.1 mM Na_2_WO_4_, and 0.1 mM Na_2_MoO_4_) were added. The medium was then dispersed in anoxic vials. Anoxic vials were gas exchanged to a 80/20 N_2_/CO_2_ headspace pressurized at 1.6–1.7 atm and subsequently autoclaved. The reducing agent was 5% (v/v) of a stock solution containing 80 g/l NaHCO_3_ and 10 g/l l-cysteine hydrochloride hydrate. Carbon sources were added as described in the following section. The vitamin solution was prepared by combining 10 ml of a 11 g/l CaCl_2_.2H_2_O solution with 1 ml of our in-house standard vitamin solution (20 mg/l biotin, 200 mg/l nicotinamide, 100 mg/l *p*-aminobenzoic acid, 200 mg/l thiamin HCl, 100 mg/l panthotenic acid, 500 mg/l pyridoxamine HCl, 100 mg/l cyanocobalamine, and 100 mg/l riboflavin). The resulting solution was filter sterilized and 1% (v/v) was added to the medium after autoclavation.

#### Carbon source conditions

The 5HMO mix was a kind contribution of Danone Global Research and Innovation Center, purchased from Chr. Hansen HMO GmbH, Rheinbreitbach, Germany. The mix consists of 52% w/w 2′-FL, 13% 3-FL, 26% w/w LNT, 4% w/w 3′-SL, and 5% w/w 6′-SL (Parschat et al. 2021). Mucin from porcine stomach type III (Sigma-Aldrich, St. Louis, MO, USA) was purified as described previously (Belzer et al. 2017). GOS and FOS were kindly provided by Danone Global Research and Innovation Center. These glycan sources were added to the medium in different compositions (Table 1).

**Table 1.** Conditions of this experiment and their respective carbon source concentrations.

Condition	Carbon source	Concentration (g/l)
MUCHMO1 MUCHMO2 MUCHMO3	5HMO Mucin	2 2.5
MUC	Mucin	5
GOSFOS	GOS/FOS	4
GOSFOSMUC	GOS/FOS	2
	Mucin	2.5
GOSFOSEXTR	Threonine	1
	GlcNAc	1
	Sialic acid	0.4
	2′-FL	1
	GOS/FOS	1
5HMO1	5HMO	4

#### Design of BabyBac

To study the complex interaction of the gut microbiota with the glycan landscape of the maturing infant gut, we created a synthetic community (BabyBac). BabyBac consisted of seven bacterial strains of the infant gut (Table 2) that are well-characterized, highly prevalent and abundant (Collado et al. 2007, Bäckhed et al. 2015). We selected infant gut bacteria with a function of interest: BabyBac entailed two trophic levels: glycan degraders and cross-feeders. Glycan degraders were microbes that are able to degrade HMOs and/or mucin glycans, and we included both specialists and generalists. The glycan degraders in BabyBac were specialist HMO degraders *Bifidobacterium infantis* and *Phocaeicola vulgatus*, specialist mucin glycan degrader *A. muciniphila* and the HMO and mucin glycan degraders *B. bifidum* and *Ruminococcus gnavus. Escherichia coli* and *Blautia producta* represented cross-feeders, which are unable to participate in the primary degradation of HMOs and mucin glycans, but are able to cross-feed on the products of glycan degradation by others.

**Table 2.** Strains that were included in the BabyBac community and their predicted copy number of 16S rRNA genes.

Species	Strain	Predicted 16S copy number
*B. infantis*	ATCC15697 (JCM 1222/ DSM 20088)	4 (rrnDB, NCBI)
*B. bifidum*	JCM1254 (DSM 20082)	1 (GTDB, ContEst16S, IMG/JGI)
*P. vulgatus*	ATCC8482	7 (rrnDB, NCBI)
*R. gnavus*	ATCC 29 149	5 (rrnDB, NCBI)
*E. coli*	K-12 substrain MG1655 (DSM 18039)	7 (rrnDB, ContEst16S)
*B. producta*	JCM1471 (DSM2950)	5 (rrnDB, NCBI)
*A. muciniphila*	ATCC BAA 835 (DSM 22959)	3 (rrnDB)

#### Precultures

First, the bacteria were precultured individually in their preferred carbon sources being either 20 mM lactose (*Bifidobacterium* spp., *P. vulgatus, E. coli*, and *B. producta*), 20 mM glucose (*R. gnavus*), or 5 g/l crude mucin (*A. muciniphila*). Precultures were incubated in the dark at 37°C without shaking for 24 h (*R. gnavus, A. muciniphila, E. coli*, and *B. producta*) or 48 h (*Bifidobacterium* spp. and *P. vulgatus*). ΔOD600nm was used to normalize to OD:1 and combine the cultures in a community inoculum for condition MUCHMO1.

#### Sequential batch fermentation

After assembly of the community inoculum, 1% (v/v) was added to anoxic vials containing both mucin (2.5 g/l) and HMOs (2 g/l) in triplicate (MUCHMO1) and incubated at 37°C without shaking for 24 h. After 24 h, 1% (v/v) of the culture was transferred to fresh medium for a total of four consecutive transfers (t24, t48, t96, and t120) to adapt the community to the condition and to achieve a stable microbial community composition. Next, the inoculum (1% (v/v)) for triplicate (r1, r2, and r3) sequential batch conditions MUCHMO2, MUC, GOSFOS, GOSFOSMUC, and GOSFOSEXTR (Table 1, Fig. 1) was taken from t120 of MUCHMO1. To create MUCHMO3, BabyBac was revived from a glycerol stock made from MUCHMO2 t120. After one transfer (t48), this community was used as the inoculum for 5HMO1, which was transferred four times as described before. At the end point of each batch fermentation (t24, t48, t72, t96, and t120) samples were taken for OD600 measurements and were stored at −20°C for subsequent analysis.

### Sample analysis

#### 16S rRNA gene amplicon library preparation and sequencing

DNA was isolated from 1 ml culture using the FastDNA™ SPIN Kit for Soil (MP Biomedicals, California, USA) according to the manufacturer’s instructions. The concentration of extracted genomic DNA was quantified using the Qubit™ dsDNA BR Assay Kit (ThermoFisher Scientific, Massachusetts, USA). A triplicate barcoded polymerase chain reaction (PCR) was performed to amplify the V4 region of the 16S rRNA. For each replicate, we used 7μl of 5X Phusion HF Buffer (ThermoFisher Scientific), 0.7μl dNTPs, 0.7μl forward primer (10 μM), 0.7μl reverse primer (10 μM), 0.35μl Phusion Hot Start II High-Fidelity DNA Polymerase (ThermoFisher Scientific), 25.5μl nuclease free water, and 0.7μl of DNA or no template. The cycling program consisted of 1 cycle at 98°C for 30 s, 25 cycles at 98°C for 10 s, then at 50°C for 10 s, and at 72°C for 10 s followed by 1 cycle at 72°C for 7 min. The 515F-806R primers [515F: 5′-GTGYCAGCMGCCGCGGTAA-3′, 806R: 5′-GGACTACNVGGGTWTCTAAT-3′ (Apprill et al. 2015, Parada et al. 2016)] were barcoded to allow for multiplexing of samples into a library. For each library, negative and positive controls were included to assess the library preparation and sequencing process. As negative controls we included a no template sample that was carried throughout each DNA extraction process (DNA extraction negative control) and a no template PCR sample (PCR negative control). As positive controls, we used two different in-house mock communities, namely mock3 and mock4 (Ramiro-Garcia et al. 2018) that are also embedded in the NG-Tax pipeline.

The triplicate amplicon PCR products were pooled and purified with the CleanNGS beads (CleanNA, Waddinxveen, the Netherlands) without deviation from the manufacturer’s instructions. 200 ng of barcoded PCR products from 70 samples were pooled to create libraries followed by a purification as mentioned before. The libraries were sequenced in HiSeq platform (Illumina, California, USA).

#### Quantitative polymerase chain reaction

DNA originating from 1 ml culture was diluted to 1 ng/µl. For samples with a concentration <1 ng/µl, there was no further dilution. Universal bacterial primers were used for total bacterial abundance (1048F: 5′-GTGSTGCAYGGYYGTCGTCA-3′, 1175R:5′-ACGTCRTCCMCNCCTTCCTC-3′) (Maeda et al. 2003) while species-specific primers were used for *B. infantis* (BiINF-1: 5′-TTCCAGTTGATCGCATGGTC-3′, BiINF-2: 5′-GGAAACCCCATCTCTGGGAT-3′) (Matsuki et al. 1999, Martin et al. 2016) and *B. bifidum* (BiBIF-1: 5′-CCACATGATCGCATGTGATTG-3′, BiBIF-2: 5′-CCGAAGGCTTGCTCCCAAA-3′) (Matsuki et al. 1999, Martin et al. 2016) quantification. A standard curve was prepared using gradient concentrations 16S rRNA gene copies ranging from 10^1^ to 10^8^ cell copies/µl in 10-fold increase. The 16S rRNA gene PCR product was retrieved using the 27F-1492R primer pair in a PCR comprising 10 μl of 5X Phusion HF Buffer (ThermoFisher Scientific), 1 μl dNTPs, 0.5 μl forward primer (10 μM), 0.5 μl reverse primer (10 μM), 0.5 μl Phusion Hot Start II High-Fidelity DNA Polymerase (ThermoFisher Scientific), 36.5 μl nuclease-free water, and 1 μl DNA template. The cycling program consisted of 1 cycle at 95°C for 5 min, 35 cycles starting at 95°C for 30 s, then at 52°C for 20 s, and at 72°C for 30 s and 1 cycle at 72°C for 7 min. For the total bacteria qPCR, *E. coli* was used as a template and for the species-specific qPCR the respective species 16S rRNA gene copies. Each sample was run in triplicate and we also included negative and no template controls. For every reaction, we added 6.25 μl iQ SYBR Green Supermix (Bio-Rad Laboratories, California, USA), 0.25 μl forward primer (10uM), 0.25 μl reverse primer (10uM), 1 μl DNA template (1 ng/ul), and 4.75 μl nuclease-free water. The cycling program for the total bacteria qPCR consisted of 1 cycle at 95°C for 3 min, 40 cycles starting at 95°C for 15 s, then at 52°C for 30 s, and at 72°C for 30 s followed by a melt curve. For the species-specific qPCR, the cycling program started with 1 cycle at 94°C for 5 min, followed by 40 cycles at 94°C for 20 s, then at 55°C for 20 s, and at 72°C for 50 s and finally a melt curve. The reactions took place in an iQ Bio-Rad iCycler (Bio-Rad Laboratories) and the results were retrieved and preprocessed with CFX Manager™ Software (Bio-Rad Laboratories).

#### High performance liquid chromatography

Liquid metabolites were quantified using high-performance liquid chromatography (HPLC). Cell-free supernatants were obtained by centrifugation at 21 300 × *g* for 5 min. Samples that contained mucin were treated with the Carrez method prior to HPLC (Carrez 1908, Acker et al. 1967). External standards were selected based on predicted metabolisms of BabyBac. Included standards were lactate, acetate, propionate, 1,2-propanediol, butyrate, glucose, fructose, formate, ethanol, and succinate. For each standard, a calibration curve was created by running three distinct concentrations. Calibration was validated by running stock samples with a known concentration. Samples were analysed on a Shimadzu LC_2030 HPLC (Kyoto, Japan) equipped with a HI-PLEX H column (Agilent, Santa Clara, CA, USA) at 45°C for 20 min and detected using a refractive index detector. 0.01 N sulphuric acid was used as eluent. Data were analysed with Chromeleon (ThermoFisher Scientific). In Chromeleon, it is possible to discern the peaks generated in the chromatogram based on retention time by comparing to the retention time of the standards. The area under the curve of each peak can then be correlated to the concentration of a specific compound through the relevant calibration curve.

### Data analysis

#### 16S amplicon sequencing analysis

The quality of the raw reads was checked with FastQC (Andrews 2010). The 150 bp paired reads were clustered to ASVs and assigned to taxonomy using the NG-Tax 2.0 pipeline (Poncheewin et al. 2020). Default settings were chosen except for a 100 bp read length and the database SILVA138. ASVs unassigned to genus level were manually assigned by subjecting the forward and reverse read to BLASTn. *B. infantis* and *B. breve* were distinguished using qPCR inferred relative abundances. Relative abundances were transformed to cell numbers by multiplying with qPCR-inferred total bacteria 16S rRNA gene copies and subsequent division by the species 16S RNA gene copy number per cell (Jian et al. 2020).

#### Glycoside hydrolase prediction

The proteomes of each species in BabyBac were retrieved from UniProt in their isoform and canonical form. Namely *B. infantis* ATCC15697 (UP000001360), *B. bifidum* (UP000070092), *Bacteroides vulgatus* ATCC8482 (UP000002861), *E. coli K12* (UP000000625), *B. producta* (UP000515789), *A. muciniphila* (UP000001031), and *R. gnavus* ATCC29149 (UP000004410). Prediction of glycoside hydrolase (GH) was performed against the dbCAN HMM database (version 12) as proposed by the authors of the dbCAN2 meta server (Zhang et al. 2018). Matching of the proteomes against the dbCAN V12 database was performed using the -hmmscan function of the hmmer module (version 3.3). The output was further parsed with the hmmscan-parser.py tool to avoid duplicate hits. To increase prediction sensitivity, the parsed output was filtered for e-value <1e-15 and coverage >0.35.

#### Data processing and visualization

Further analysis took place in R (version 4.2.1; R Development Core Team 2008) using Rstudio (version 2022.12.0+353; RStudio Team 2021). Bacterial composition data were analysed using the packages ‘phyloseq’ (version 1.42.0; McMurdie and Holmes 2013) and ‘microbiome’ (version 1.20.0; Lahti et al. 2020). Beta-diversity was calculated using the Bray–Curtis dissimilarity, creating a principal coordinates analysis (PCoA) ordination plot. Bray–Curtis similarity (1-Bray–Curtis dissimilarity) was used to assess stable composition based on the vegdist() function of the ‘vegan’ package (version 2.6–4; Oksanen et al. 2022). Differential abundance per species between conditions was performed using the wilcox.test() function and subsequent FDR correction using the p.adjust() function, both from the ‘stats’ package (version 4.2.1; R Development Core Team 2008). We further assessed the differential abundance of each species with the linda() function of the ‘MicrobiomeStat’ package (version 1.1; Zhang and Chen 2022). Redundancy Analysis (RDA) and permutation testing to assess significance (annova.cca()) were performed with the ‘vegan’ package using mucin, HMO and GOS/FOS presence as explanatory variables. For LinDA, mucin-adjusted grouping as set as fixed effect and replicate was set as random effect. Spearman correlation coefficient between replicates was calculated with the function cor() (‘stats’ package, version 4.2.1) based on relative abundance of corrected 16S rRNA genes counts. Any further data analysis and visualization was facilitated by packages ‘dplyr’ (version 1.1.2; Wickham et al. 2023) and ggplot2 (version 3.4.2; Wickham 2016). Minor aesthetic processing and combining of figures was performed in Adobe Illustrator (April 2024 release, version 28.5).

## Results

### BabyBac members possess overlapping GH profiles for glycan degradation

**Figure 2.**
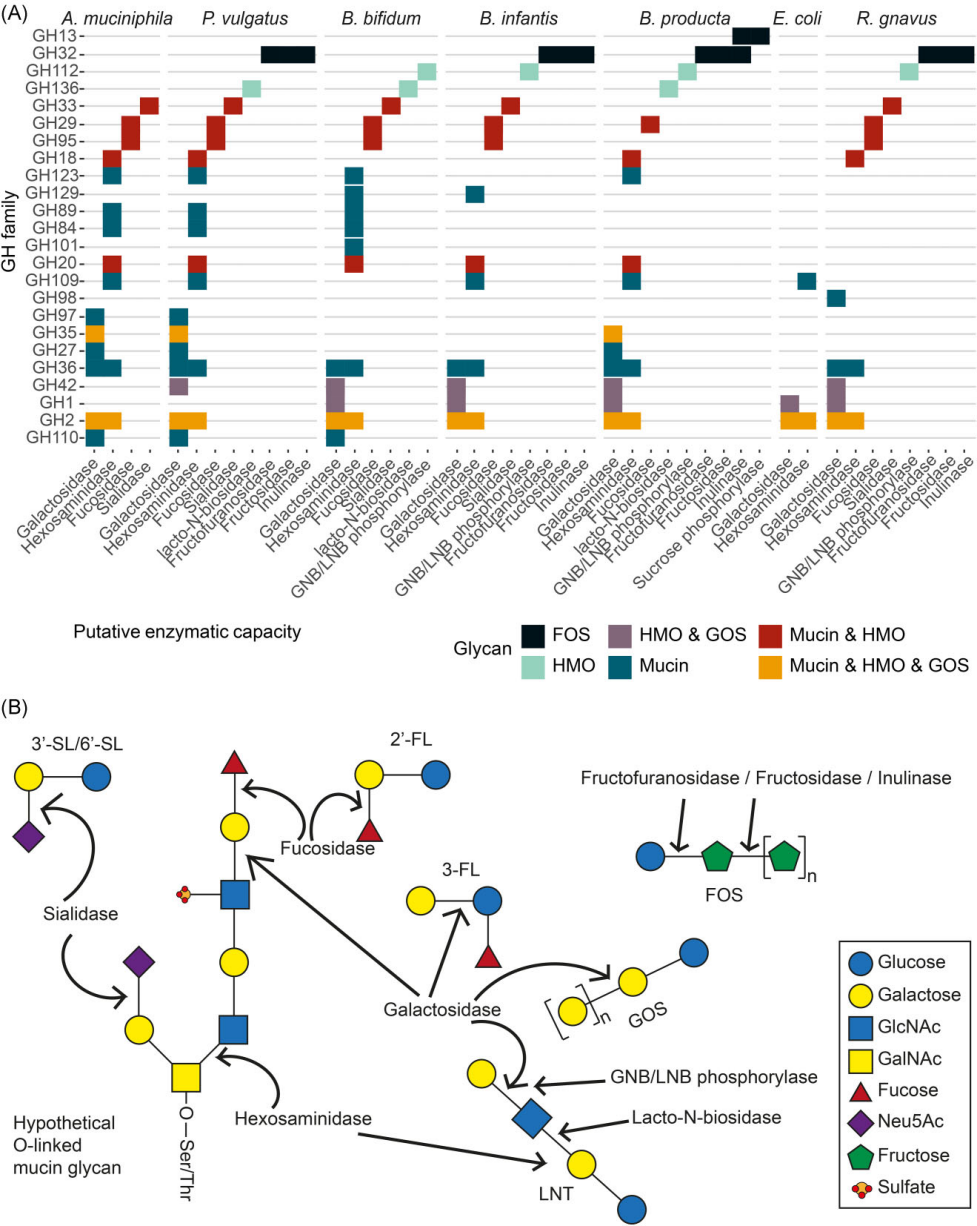
(A) Predicted glycoside hydrolases (GH) related to mucin, HMO, GOS, and FOS degradation, as well as those with overlapping activities, encoded within BabyBac. (B) Overlap in structure and enzymatic degradation between a hypothetical mucin glycan, HMOs, GOS, and FOS. Ser = serine, Thr = threonine, 2′-FL = 2′-fucosyllactose, 3-FL = 3-fucosyllactose, 3′-SL = 3′-sialyllactose, 6′-SL = 6′-sialyllactose, LNT = lacto-*N*-tetraose, GOS = galactooligosaccharides, FOS = fructooligosaccharides, GlcNAc = *N*-acetylglucosamine, GalNAc = *N*-acetylgalactosamine, and Neu5Ac = sialic acid.

We performed GH prediction on the proteomes of the BabyBac members (Fig. 2). GH families were mapped to categories based on their respective association to degradation of milk or mucin glycans based on our previous work (Ioannou et al. 2021, Berkhout et al. 2022) (Tables S1 and S2, Fig. 2, Fig. S1). This resulted in distinct GH profiles, such as the characteristic mucin glycan specialist profile of *A. muciniphila*, the glycan generalist profile of *P. vulgatus*, and the low number of relevant GHs that cross-feeder *E. coli* possesses. Therefore, we hypothesized that BabyBac community composition would be shaped by substrate-driven competition and cooperation.

### Glycan combinations determine BabyBac species composition and metabolite production

To investigate the response of BabyBac to different glycans available in the infant gut, we selected four conditions, namely HMOs (5HMO), GOS/FOS (GOSFOS), mucins (MUC), and their combinations (MUCHMO and GOSFOSMUC) as a carbon source. We also included mucin monomers and 2′-FL (GOSFOSEXTR) in one of the conditions to test whether those would induce similar community dynamics as complete mucin structures (Fig. 1). BabyBac was transferred four times for each of these conditions to adapt and stabilize community composition (Table S3). Growing BabyBac in each of the conditions led to a reproducible community (Fig. S2). To assess day-to-day community reproducibility, we calculated the Bray–Curtis similarity of each timepoint as compared to the final timepoint (t120). This analysis revealed that for most conditions the community composition was stable from timepoint t72 onwards (Fig. S2). Interestingly, conditions with mucin had a more reproducible Bray–Curtis similarity between replicates. Additionally, we detected high reproducibility between replicates using Spearman correlation coefficient based on corrected 16S rRNA gene counts (Fig. S3).

**Figure 3.**
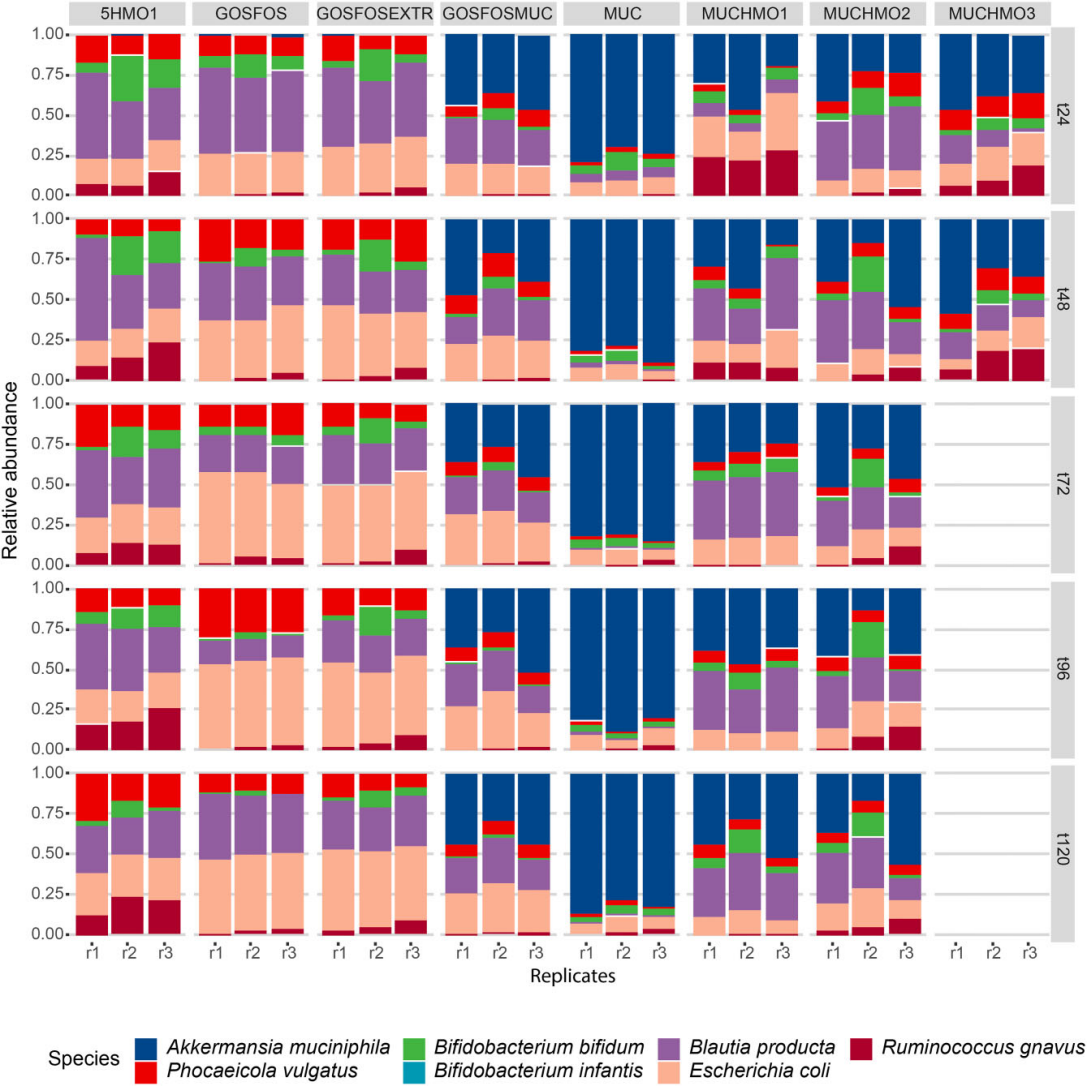
Relative abundances of BabyBac species grown in sequential batch in various conditions with glycans from the infant gut. Values were obtained from a combination of 16S rRNA gene amplicon sequencing and qPCR, normalized for 16S rRNA gene copy number. Each timepoint represents the endpoint of a 24 h batch fermentation.

In the initial condition (MUCHMO1), *A. muciniphila, B. bifidum*, and *P. vulgatus* were prominent glycan degraders (Fig. 3, Fig. S4). However, *R. gnavus* decreased to low relative abundance and *B. infantis* was not detected. The presence of *B. producta* and *E. coli* suggests that they are able to cross-feed on the products of glycan degradation. BabyBac was then used to study six conditions of glycans: MUCHMO2, MUC, 5HMO1, GOSFOS, GOSFOSMUC, and GOSFOSEXTR. This led to distinct community compositions. *B. bifidum* was the most consistently observed glycan degrader of BabyBac and remained present in all conditions until the end of the experiment. Although *R. gnavus* was reduced to a low relative abundance during the initial MUCHMO1 condition, this species remained present in all conditions. Interestingly, it performed best in HMOs only (5HMO1). In the MUC condition, *A. muciniphila* was dominant with low relative abundance of other glycan degraders and cross-feeders. However, *A. muciniphila* was lost in conditions without mucin. Conversely, *P. vulgatus* reached higher relative abundances as the predominant glycan degrader in the presence of HMOs or GOS/FOS.

In general, acetate and formate were the dominant SCFAs that BabyBac produced in all conditions (Table S3, Fig. S5). In GOSFOS and GOSFOSEXTR, the highest acetate and formate levels (>30 mM) were observed in a community rich in *E. coli* and *B. producta*. The other metabolites such as propionate, succinate, ethanol, and 1,2-propanediol were present in all conditions albeit in lower concentrations (<10 mM). Lactate was only observed in low amounts (<2 mM) in GOSFOSMUC and GOSFOSEXTR.

**Figure 4.**
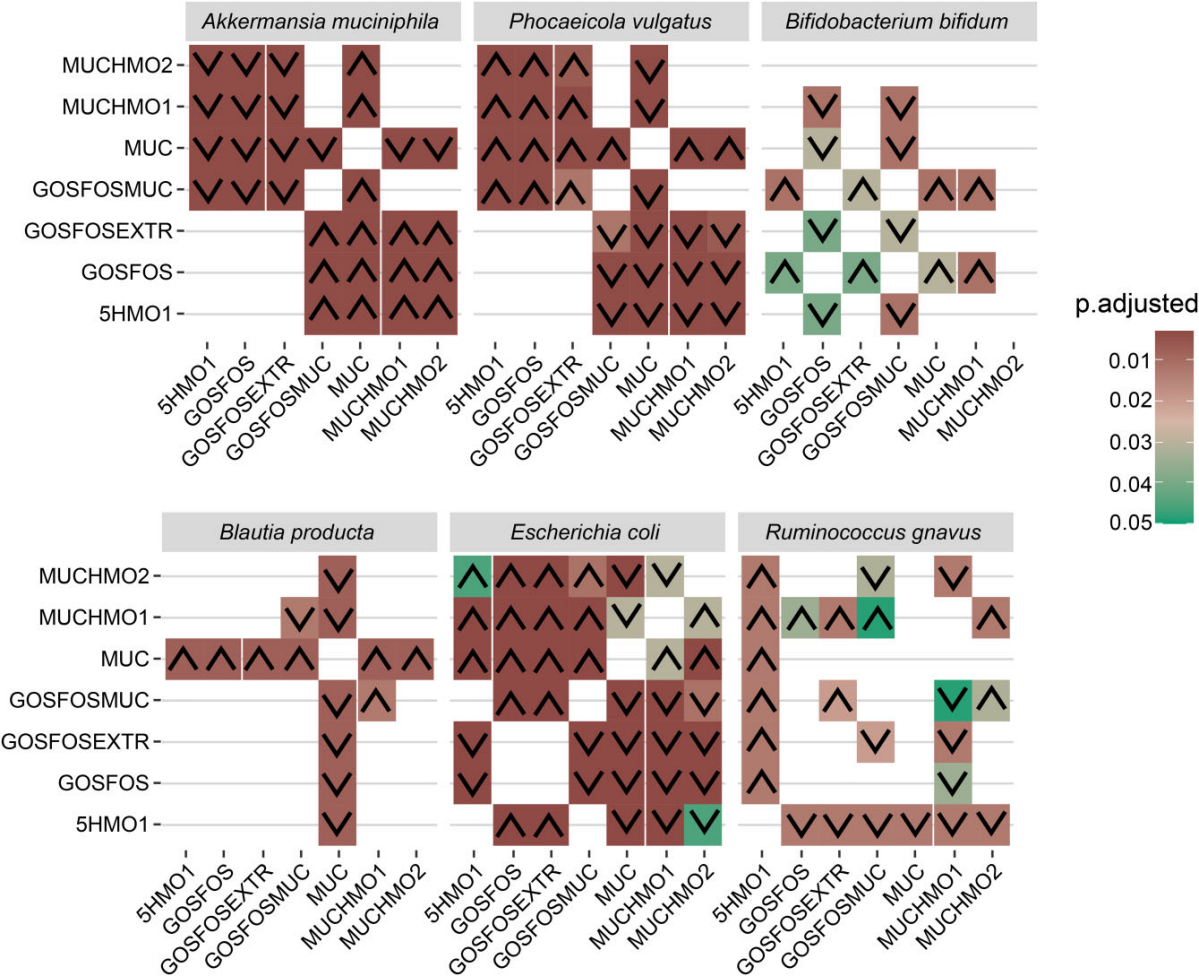
Statistically different relative abundance of species calculated by Wilcoxon testing of t96 and t120 replicates between conditions. Values are shown as *P*.adjusted, i.e. FDR corrected *P*-values. Arrows indicate increased (∧) or decreased (∨) relative abundance in the condition in the *x*-axis compared to the condition in the *y*-axis.

To better understand how each condition affected the BabyBac species, we statistically assessed the difference in relative abundance through FDR-corrected Wilcoxon testing (Fig. 4). *A. muciniphila* abundance was significantly higher in conditions containing mucin compared to the conditions that did not contain mucin. *P. vulgatus*, however, was lower abundant in conditions that contain mucin. *B. bifidum* was significantly lower in conditions GOSFOS and GOSFOSMUC compared to the other conditions (Fig. 4). The HMO-degrader, *R. gnavus*, was significantly higher in the 5HMO1 condition compared to all other conditions. As for *B. producta*, it was significantly lower, and almost absent in the presence of sole mucin as carbon source (MUC) compared to the other conditions. *E. coli* was statistically significantly higher in conditions with GOS/FOS (GOSFOS and GOSFOSEXTR) followed by 5HMO1 and GOSFOSMUC and being the lowest in the mucin-containing conditions (MUC, MUCHMO1, and MUCHMO2).

### Mucin and HMO presence and amount impact overall community structure

**Figure 5.**
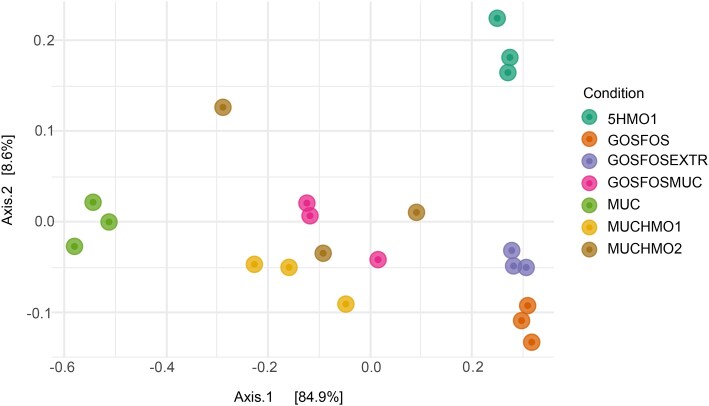
PCoA plot based on Bray–Curtis dissimilarity for t120 replicates, color-coded per condition.

From our results, it was suggested that the provided glycans had an effect on individual bacterial species and thus community structure. We, thereon, investigated the effect on the community composition at the final transfer (t120) using beta-diversity, as PCoA ordination of Bray–Curtis dissimilarity (Fig. 5). Axis 1 shows that there is a clear separation between MUC, the mucin-containing conditions MUCHMO1, MUCHMO2, and GOSFOSMUC and nonmucin conditions 5HMO1, GOSFOS, and GOSFOSEXTR. The axis that separates these conditions explains 84.9% of the variance. Moreover, Axis 2 separated the 5HMO condition from the rest of the conditions. These results show that community structure differed and that variation could be explained by the presence and amount of mucin and HMOs.

**Figure 6.**
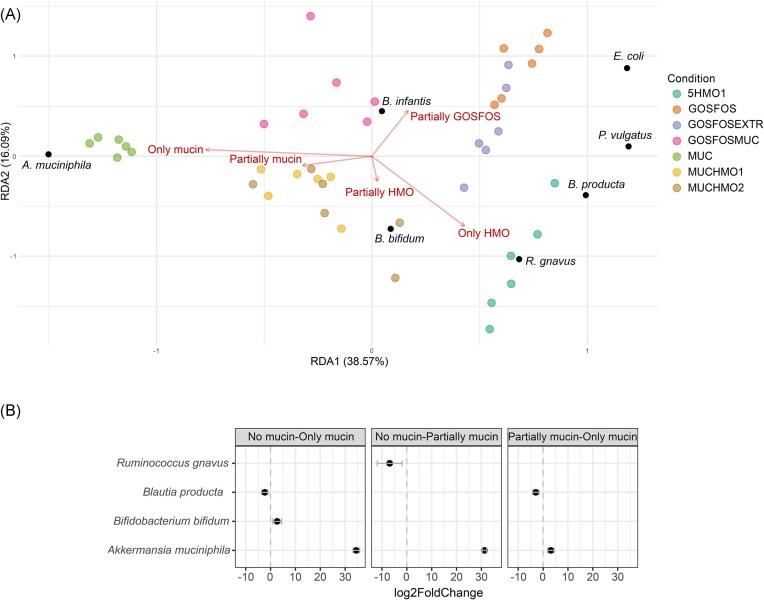
(A) RDA plot of the final two timepoints using the species composition as dependent variable and the mucin, HMO, and GOSFOS presence as explanatory variables. Samples (sites) are coloured based on condition. (B) Differentially abundant species based on the presence of mucin. Significantly different results are shown as log2 fold change from −10 (higher in the condition to the left) to 40 (higher in the condition to the right).

To verify this, we further stratified our data based on the presence and amount of each type of glycan. These were namely mucin (MUC = ‘Only mucin’; MUCHMO1, MUCHMO2, and GOSFOSMUC = ‘Partially mucin’; and 5HMO1, GOSFOS and GOSFOSEXTR = ‘No mucin’), HMOs (5HMO1 = ‘Only HMOs’; MUCHHMO1, MUCHMO2, and GOSFOSEXTR = ‘Partially HMOs’; and MUC, GOSFOS = ‘No HMOs’), and GOS/FOS (GOSFOS = ‘Only GOSFOS’; GOSFOSMUC and GOSFOSEXTR = ‘Partially GOSFOS’, and 5HMO1, MUCHMO1, MUCHMO2, and MUC = ‘No GOSFOS’). RDA using the composition of the two final (t96 and t120) timepoints’ replicates (Fig. 6A) showed that mucin and HMOs significantly affected the variation in community composition (p_mucin_=0.001, p_hmo_=0.001), while GOS/FOS did not (p_gosfos_=0.19). It also demonstrates that changes in composition are driven by *A. muciniphila, B. bifidum, R. gnavus*, and *B. producta*. To understand the effect of mucin on species relative abundance, we used LinDA on the aforementioned mucin-adjusted stratification. *A. muciniphila* was highly associated with the presence of mucin (Fig. 6B). Even partial presence of mucin translated to >30 log2 fold increase in *A. muciniphila* relative abundance. *R. gnavus* had a significantly lower relative abundance in conditions, where there was a combination of mucin with another glycan source compared to conditions without mucin. *B. bifidum* was significantly higher in the ‘Only mucin’ group when compared to conditions without mucin. Lastly, the cross-feeder *B. producta* had a 2.33 and a 2.93 log2 fold decrease in ‘Only mucin’ compared to the absence of mucin or partial presence of mucin, respectively.

The effect of mucin as a carbon source was also observed in the metabolic profile of BabyBac. For all conditions containing mucin, despite the comparable amount of cells (Table S3, Fig. S5), the total concentration of metabolites measured was lower compared to the conditions containing no mucin. In terms of relative proportions of metabolites, the conditions with HMOs had the highest relative acetate concentration, the conditions with GOS/FOS had the highest relative concentration of formate, and the condition with only mucin had the highest relative concentration of ethanol (Fig. S6).

### Mucin monomers and 2′-FL do not recapitulate a mucin- or HMO-driven synthetic community composition

In the previous analysis, we established that mucin and HMO presence and amount significantly affected community composition, and *A. muciniphila* was a major driver of community changes. *A. muciniphila* did not grow in the absence of mucin (GOSFOS and 5HMO1) despite its ability to utilize HMOs. We, therefore, created a condition including mucin monomers and 2′-FL together with GOSFOS to test whether this would allow *A. muciniphila* to claim a niche. However, the addition of these components did not lead to the growth of *A. muciniphila* (Figs 3 and 4, Fig. S4). *A. muciniphila* was, therefore, only able to grow in the presence of the complete mucin glycans. When considering the entire community, addition of 2′-FL, sialic acid, GlcNAc, and l-threonine reduced the distance with the conditions containing mucin and HMOs (Fig. 5), but multiple species remained differentially abundant. *B. bifidum* levels were increased in GOSFOSEXTR and resembled those of 5HMO1, but *E. coli* and *R. gnavus* remained significantly different between these conditions (Figs 3 and 4, Fig. S4). Moreover, apart from *A. muciniphila, P. vulgatus*, and *E. coli* remained significantly different (Fig. 4) between GOSFOSEXTR and mucin-containing conditions (MUC, MUCHMO1, MUCHMO2, and GOSFOSMUC).

## Discussion

In this study, we took a closer look at the microbial niche differentiation driven by the glycan landscape in the infant gut microbiota. For this, we employed a 7-strain synthetic community (BabyBac) that included bacteria known as important residents of the infant gut. BabyBac included HMO specialists, mucin specialists, glycan generalists, and cross-feeders. *B. infantis* is a dominant species in the breastfed infant gut due to its ability to consume HMOs (Sela et al. 2008) and *B. bifidum* is a prominent HMO degrader that has the unique property that it can apply its extracellular enzymes to degrade mucin (Ruas-Madiedo et al. 2008). *R. gnavus* also degrades both HMOs and mucin (Png et al. 2010, Crost et al. 2013, Wu et al. 2020), while *P. vulgatus* is an HMO degrader, that does not show high mucin glycan degradation capacity (Png et al. 2010, Sato et al. 2020). *A. muciniphila* is a well-known mucin glycan forager that can cross-feed other mucosal residents, but it has also been found to degrade and grow on HMOs (Belzer et al. 2017, Kostopoulos et al. 2020). Furthermore, we included cross-feeders *E. coli* and *B. producta* that consume glycans, gases, and metabolites released by the primary degraders (Chang et al. 2004, Ose et al. 2018). We, therefore, designed a synthetic community where members form clear trophic levels. We hypothesized that complex glycans, either dietary or mucin, could be degraded by specialists or generalists, releasing simpler glycans, metabolites, and gases that could then be consumed by cross-feeders.

By providing different ratios of dietary and mucin glycans, we observed that they affected BabyBac composition and metabolite production. Through sequential batch fermentation, BabyBac achieved a reproducible community composition after 72 h in most conditions and was highly reproducible between replicates. The interplay of different glycans paints a picture of niche differentiation from mucus to lumen which is driven by competition for specific glycans. Although the primary degraders are capable of primary degradation of the glycans to some extent in monoculture (Hoskins et al. 1992, Derrien et al. 2004, Crost et al. 2013, Garrido et al. 2013, Van Bueren et al. 2017, Kostopoulos et al. 2020, Ioannou et al. 2024), their potential to dominate in the community depended on the presence of other primary degraders. Cross-feeding interactions sustained BabyBac and affected its compositional and metabolic profile in a carbon-source dependent manner.

*B. infantis* was outcompeted in the initial phase even though it is an efficient HMO utilizer (Sela et al. 2008). This phenomenon was also observed in a recent study where *B. bifidum* outcompeted *B. infantis* when cocultured on HMOs (Ojima et al. 2022). This inefficiency could be a result of *B. infantis*’s intracellular degradation of HMOs while other HMO and mucin degraders have extracellular glycosidases to degrade HMOs (Sela et al. 2008, Lawson et al. 2019, Kostopoulos et al. 2020, Ojima et al. 2022). It can also be that *B. infantis* is able to prevail in the infant gut through other means of pressure, such as a lower pH (Henrick et al. 2018), the utilization of urea (You et al. 2023), or the stimulating potential of other compounds, including lactoferrin (Kim et al. 2004).

*A. muciniphila* displayed a strong association with the presence of mucin as a carbon source and dominated over other primary glycan degraders in the MUC condition. Even though *A. muciniphila* is capable of HMO degradation (Kostopoulos et al. 2020, Luna et al. 2022), it was rapidly outcompeted by other glycan degraders of BabyBac in the absence of mucin in our batch fermentations. This suggests that *A. muciniphila* relies on degradation of mucus to sustain itself in early life, rather than the degradation of HMOs. The inability of *A. muciniphila* to compete with infant gut bacteria for HMOs is confirmed by its low relative abundance in infants, which increases over time during the transition to a mature gut (Collado et al. 2007, Derrien et al. 2008, Bäckhed et al. 2015). Therefore, we hypothesize that *A. muciniphila* survives in the infant gut through utilization of mucus glycans from breast milk and the developing mucus layer, as opposed to through degradation of HMOs. Supplementation of GOS/FOS conditions with sialic acid, GlcNAc, 2′-FL, and the amino acid l-threonine, which is essential for *A. muciniphila* growth, did not enable *A. muciniphila* to compete with the other BabyBac members in sequential batch fermentation. This suggests a highly specific adaptation of *A. muciniphila* to the utilization of the complex mucin. This adaptation could, for example, be explained by the specific binding of *A. muciniphila* to *N*-acetyllactosamine (LacNAc and Galβ1–4GlcNAc), which is highly abundant in pig gastric mucin (Berkhout et al. 2024) and in human colonic mucus (Elzinga et al. 2024). Overall, we provide evidence for microbial ecological dynamics driven by the glycan landscape from lumen to mucus. Our results are in line with experiments in mice models, where a fiber-free diet increases the relative abundance of *A. muciniphila* (Desai et al. 2016, Grant et al. 2023, Kuffa et al. 2023, Parrish et al. 2023, Holmberg et al. 2024). These further highlight that the ratio of mucin and dietary fiber is crucial for the regulation of bacterial composition at the mucus-lumen interface. As recently shown through the use of a synthetic community in mice, dietary fiber and the presence of *A. muciniphila* have direct effects on immune modulation, even as parameters of the maternal microbiome (Grant et al. 2023). In terms of the effect of 2′-FL on *Akkermansia* spp., evidence is still controversial. 2′-FL supplementation in an *in vitro* model using formula fed infant feces led to a decrease in *Akkermansia* spp. (Salli et al. 2019), while 2′-FL feeding to mice increased *Akkermansia* spp. relative abundance (Ge et al. 2024). This association needs to be further researched, especially at strain level, since HMO utilization capacities of *Akkermansia* are strain-dependent (Luna et al. 2022).

Even though *R. gnavus* is capable of both mucin and HMO degradation (Png et al. 2010, Crost et al. 2013, Wu et al. 2020), its relative abundance was positively associated with HMOs and negatively associated with the partial presence of mucin. *R. gnavus* ATCC 29149 is an ample degrader of HMOs, which was also shown when 2′-FL was supplied. Furthermore, this strain is capable of 3-FL utilization (Crost et al. 2013). Of note, the inoculum for the 5HMO condition was MUCHMO3, which had a significantly higher relative abundance of *R. gnavus* compared to MUCHMO1, which was the inoculum for all other conditions. However, its HMO-degrading capacity justifies its high relative abundance in 5HMO. On the other hand, during growth on only mucin in BabyBac, *R. gnavus* was nearly completely suppressed. A probable explanation for this observation could be that *R. gnavus* is not able to compete with the highly specialized *A. muciniphila* that dominates in this condition, as it is not able to utilize mucin as efficiently (Crost et al. 2013). Interestingly, an inverse relationship between *R. gnavus* and *A. muciniphila* is often observed *in vivo*. Although both species are mucin glycan degraders, *A. muciniphila* is associated with a healthy mucus layer, whereas *R. gnavus* is associated with inflammatory bowel diseases and a range of other disorders (Png et al. 2010, Crost et al. 2013, Qiu et al. 2022).

In most conditions, cross-feeders *B. producta* and *E. coli* were able to thrive. However, these decreased during growth on only mucin. The near loss of *B. producta* in the mucin condition may be explained by the lack of H_2_ production by *A. muciniphila* during mucin degradation (Derrien et al. 2004). Similar associations between *Blautia* and the fiber–mucin ratio have been reported in studies where donor feces were transplanted in mice (Holmberg et al. 2024). There, *Blautia* spp. were significantly lower in mice fed a fiber-free diet, especially when donors did not consume a fiber-rich diet. Furthermore, degradation of mucin by *A. muciniphila* may produce fewer substrates for cross-feeding compared to HMO degradation by *Bifidobacterium* spp. and *Bacteroides* spp. The lower relative abundance of *E. coli* as a cross-feeder that scavenges the products of mucin degradation is in accordance with previous coculture studies that show a dominance of *A. muciniphila* over cross-feeders during mucin degradation (Belzer et al. 2017, Pichler et al. 2020). On the other hand, cocultures with *Bifidobacterium* spp. and cross-feeders growing on human milk carbohydrates show a more equal cell ratio or even dominance of the cross-feeder (Schwab et al. 2017, Katoh et al. 2020, Chia et al. 2021). Future experiments with cocultures could shed light on the exact by-products of HMO or mucin degradation that are utilized by each of the cross-feeders.

Mucin glycans and HMOs share many structural similarities in terms of glycan building blocks and glycosidic bonds (Pruss et al. 2020). As shown in our functional annotation of BabyBac proteomes, microbes share the potential to access both glycan types (Ruas-Madiedo et al. 2008, Marcobal et al. 2011, Kostopoulos et al. 2020, Luna et al. 2022). These observations have led to the hypothesis that HMOs can stimulate proper seeding of the gut mucosal layer with beneficial microbes (Koropatkin et al. 2014, Belzer 2022). This hypothesis is strengthened by the fact that human milk contains MUC1 and MUC4 (Liu and Newburg 2013). However, in our results, the compositional and metabolic profile of BabyBac was very distinct between the two conditions. This could signify that there is a possible regulation mechanism of the gut microbiota by the ratio between HMOs and mucin across the gut canal. Through ‘imperfect’ utilization of HMOs *A. muciniphila* could be retained in low enough numbers in infancy until the mucin layer matures and it is able to colonize its preferred niche. There is evidence from observational studies that strengthen this hypothesis. Infants that are breastfed for a longer period demonstrate signs of mucin degradation later than infants breastfed for <1 month (Midtvedt et al. 1994) and HMOs like 3-FL and LNT2 affect the production of MUC2 as well as that of other proteins that hold mucins together (Cheng et al. 2020). Additionally, freed mucin *O*-glycans in feces were associated with an increased relative abundance of Bacteroidaceae and a lower relative abundance of Bifidobacteriaceae (Karav et al. 2018).

Despite differences in terms of composition in BabyBac dependent on the type of glycan provided, the metabolic profiles were more aligned. In all conditions, the main metabolites produced by BabyBac were acetate and formate, whereas metabolites in lower concentrations included ethanol, propionate, succinate, and 1,2-propanediol and lactate was observed in a limited number of conditions. During infant development, three distinct phases of infant faecal SCFA profiles are described in the transition to adult-like gut microbiota. The first phase is characterized by low acetate and high succinate, the second phase by high lactate and formate, and the final phase by high propionate and butyrate (Tsukuda et al. 2021). We observed a SCFA profile that is similar to the second phase described by Tsukuda et al. (2021) with high acetate and formate and lower levels of succinate and propionate. However, lactate concentration was low in our experiments. This could be an indication of stress in *Bifidobacterium* spp., as a shift from lactate production to ethanol production increases ATP yield (Schöpping et al. 2022). In general, conditions containing mucin yielded a lower total metabolite concentration, although total carbon content cannot be calculated due to the heterogeneous and variable composition, and therefore no definitive conclusions can be drawn. However, apparent differences in metabolite concentrations may be explained by the differences in substrate concentration between mucin and other glycans. As mucin is a viscous substance, it cannot be added in high concentrations.

This is where one of the limitations of our experiments lies. The carbon sources used for the different conditions were balanced in terms of concentration (% w/v) but not in terms of carbon content and were not based on actual *in vivo* ratios in the infant gut. Apart from the heterogeneous nature of mucin, GOS and FOS are also contaminated with lactose, glucose, and galactose. The presence of these readily available sugars might have affected our results by allowing for the fast growing *E. coli* and the versatile *B. producta* to thrive in these conditions. The increase of *E. coli* in batch cultivation has been reported in a recent study with infant fecal inocula and could be associated with the nature of the setup, as batch fermentations favour fast-growing microorganisms (Rachmühl et al. 2023). Nevertheless, the sequential batch fermentation of BabyBac allowed us to model specific glycan-driven microbe–microbe interactions in a stable bacterial community of the infant gut. In this controlled system, the changes in community composition that were induced by the presence of specific glycans could be closely monitored so that ecological interactions could be tracked. However, this study does not include a host component, which could influence community composition and behaviour through host–microbe interactions. Furthermore, fresh glycans were supplied every 24 h, which does not perfectly recapitulate the glycan landscape *in vivo*, where breastfeeding and/or formula feeding have a certain periodicity, whereas mucin is constantly produced. A limitation of this work is that we did not perform experiments assessing growth of individual strains in our medium without carbon source, which we and others have done in previous studies (Table S1).

In summary, we created BabyBac, a synthetic community that was used to study gut microbiota adaptation to the glycan landscape in the infant gut. Glycan types significantly affected community composition, and the addition of simpler structures did not recapitulate the results of mucin and HMOs. We also found that specialist mucin degrader *A. muciniphila* is driven towards a niche of mucin glycan degradation, and is unable to compete with specialist HMO degraders in the absence of mucin in sequential batch cultures. On the other hand, *B. infantis* was not able to claim a niche under our conditions. Experiments in bioreactors, where a constant lower pH is maintained can recapitulate the conditions in the infant gut, which pose an additional pressure in microbial dynamics. Future experiments can also focus on adding spatial organization by placing mucin on beads or on a membrane in bioreactors. This would further verify the niche differentiation of *A. muciniphila* in the infant gut and its restriction to mucolysis and whether attachment is required. It still remains unclarified what ecological advantage HMO utilization confers to *A. muciniphila*. More work on the engraftment of *A. muciniphila* and other glycan degrading bacteria in the gut will be facilitated by the improvement of DNA isolation techniques from mucosal samples.

## Supplementary Material

fiaf069_Supplemental_Files

## Data Availability

The 16S rRNA gene amplicon sequencing dataset that was generated in this study is available in ENA at https://www.ebi.ac.uk/ena/browser/view/PRJEB72734 and can be accessed with project number PRJEB72734.
