# Supplementary material for: Milk and mucin glycans orchestrate a synthetic infant gut microbiota structure
Source: FEMS Microbiol Ecol. 2025 Jun 25;101(8):fiaf069. doi: 10.1093/femsec/fiaf069 (PMC12268331; doi:10.1093/femsec/fiaf069)
Supplement: fiaf069_Supplemental_Files [file fiaf069_supplemental_files.zip › Supplementary material Belzer Revision 3.docx]

**Supplementary Materials**

**Milk and mucin glycans orchestrate a synthetic infant gut microbiota structure**

Maryse D. Berkhout^1*^, Athanasia Ioannou^1*^, Yuvashankar Kavanal Jayaprakash^1^, Caroline M. Plugge ^1+^, Clara Belzer^1+#^

^1^Laboratory of Microbiology, Wageningen University and Research, Wageningen, The Netherlands

*Authors contributed equally

^+^Authors contributed equally

# Corresponding author, email: clara.belzer@wur.nl; Stippeneng 4, 6708 WE Wageningen, The Netherlands.

**Supplementary Table 1:** Growth of individual strains used in this study on carbon sources used in this study.

|  | **Lactose** | **5HMO** | **Mucin** | **GOS/FOS*** | **2-FL** | **GlcNAc** | **Sialic acid** |
| --- | --- | --- | --- | --- | --- | --- | --- |
| ***B. infantis* ATCC15697** | Yes | Yes (Ioannou et al., 2024) | No** | GOS: Yes (Garrido et al 2013), FOS: Yes (Perrin et al 2001) | Yes (Ioannou et al., 2024) | Yes (Turroni et al., 2012) | Yes (Turroni et al., 2012) |
| ***B. bifidum* JCM1254** | Yes | Yes (Ioannou et al., 2024) | Yes (Ruas-Madiedo 2008), minor** | GOS & FOS: Minor growth (Morozumi et al 2023) | Yes (Ioannou et al., 2024) | Yes (Turroni et al., 2012) | No (Turroni et al., 2012) |
| ***P. vulgatus* ATCC8482** | Yes | Yes (Ioannou et al., 2024) | No** | GOS: Yes Salli et al 2020, FOS: NA | Yes (Ioannou et al., 2024) | Yes (You et al., 2023) | NA |
| ***E. coli* Κ-12 substrain MG1655** | Yes | No (Ioannou et al., 2024) | Minor** | GOS: NA but other strains grow (Salli et al 2020), FOS: NA | No (Ioannou et al., 2024) | Yes (Huang et al., 2015) | Yes (Huang et al., 2015) |
| ***B. producta* JCM1471** | Yes | No (Ioannou et al., 2024) | No** | GOS: NA, FOS: NA | No (Ioannou et al., 2024) | Yes (Ioannou et al., 2024) | Possibly based on bioinformatic analysis and genus level (Coker et al., 2021) |
| ***A. muciniphila* ATCC BAA 835** | No (Derrien et al., 2004) | Expected (Padilla et al., 2024, Kostopoulos et al., 2020) | Yes (Derrien et al., 2004) | NA | Yes (Padilla et al., 2024) | Yes (Derrien et al., 2004) | No (Shuoker et al., 2023) |
| ***R. gnavus* ATCC 29149** | No | Yes (Ioannou et al., 2024) | Minor** (Crost et al., 2013) | NA | Yes  (Ioannou et al., 2024) | Yes (Crost et al., 2013) | No (monosaccharide, Crost et al., 2016) |

**Supplementary Table 2:** List of GH families and the respective enzyme types that are associated with the degradation of HMOs, GOS, FOS, mucin as well as those with overlapping acitvities.

| **GH family** | **Enzyme type** | **Glycan** |
| --- | --- | --- |
| GH29 | Fucosidase | Mucin & HMO |
| GH95 | Fucosidase | Mucin & HMO |
| GH27 | Galactosidase | Mucin |
| GH35 | Galactosidase | Mucin & HMO & GOS |
| GH97 | Galactosidase | Mucin |
| GH98 | Galactosidase | Mucin |
| GH110 | Galactosidase | Mucin |
| GH16 | Galactosidase | Mucin |
| GH2 | Galactosidase | Mucin & HMO & GOS |
| GH36 | Galactosidase | Mucin |
| GH2 | Hexosaminidase | Mucin & HMO & GOS |
| GH36 | Hexosaminidase | Mucin |
| GH18 | Hexosaminidase | Mucin & HMO |
| GH20 | Hexosaminidase | Mucin & HMO |
| GH84 | Hexosaminidase | Mucin |
| GH85 | Hexosaminidase | Mucin & HMO |
| GH89 | Hexosaminidase | Mucin |
| GH101 | Hexosaminidase | Mucin |
| GH109 | Hexosaminidase | Mucin |
| GH123 | Hexosaminidase | Mucin |
| GH129 | Hexosaminidase | Mucin |
| GH33 | Sialidase | Mucin & HMO |
| GH112 | GNB/LNB phosphorylase | HMO |
| GH136 | lacto-N-biosidase | HMO |
| GH1 | Galactosidase | HMO & GOS |
| GH42 | Galactosidase | HMO & GOS |
| GH53 | Galactanase | GOS |
| GH32 | Fructofuranosidase | FOS |
| GH32 | Fructofuranosidase | FOS |
| GH32 | Fructosidase | FOS |
| GH32 | Inulinase | FOS |
| GH13 | Inulinase | FOS |
| GH13 | Sucrose phosphorylase | FOS |

**Supplementary Table 3:** Composition and metabolites per sample. Compositional results include qPCR and copy number corrected abundances per species, total bacteria 16S rRNA gene copies, and total bacterial cell numbers (sum of the corrected abundances of species). Metabolites are provided as absolute concentrations (mM) and as relative concentrations that were used for the comparison between conditions. Available as a separate .xlsx file.

**Supplementary Data 1:** Raw HPLC data of conditions GOSFOS, GOSFOSEXTRA, GOSFOSMUC, MUCHMO1, MUCHMO2 and MUC transfers t72-t120. Available as a separate .pdf file.

**Supplementary Data 2:** Raw HPLC data of conditions 5HMO1 (all transfers), GOSFOS, GOSFOSEXTRA, GOSFOSMUC, MUCHMO1, MUCHMO2, MUCHMO3 and MUC (t24 and t48). Available as a separate .pdf file.

**Supplementary Figure 1:** Growth curves of monocultures of community members on mucin (porcine gastric mucin, 5 g/L) (OD600nm) in minimal medium supplemented with yeast extract (1 g/L).


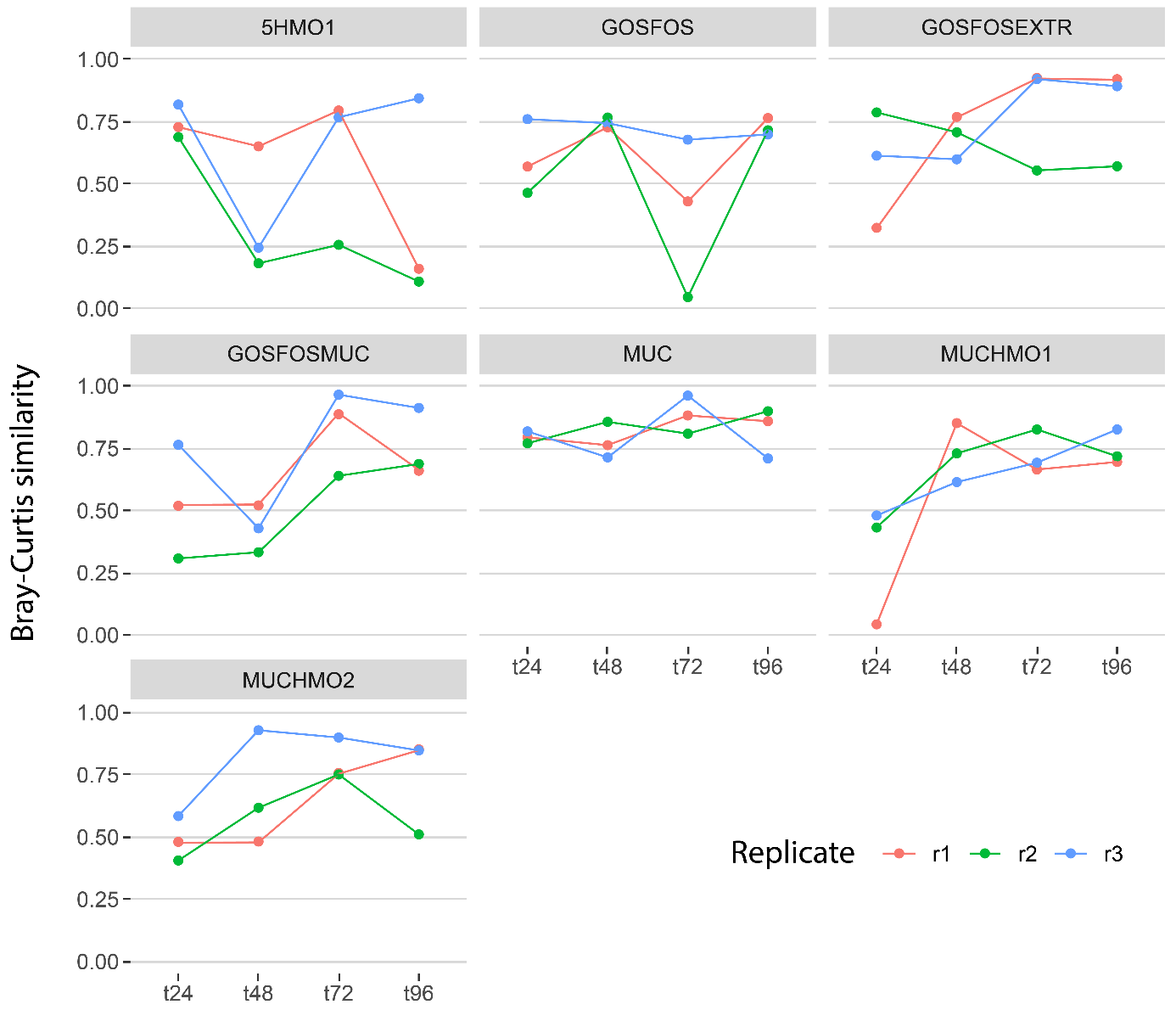


**Supplementary Figure 2:** Bray-Curtis similarity calculated between t120 and each previous timepoint. Values are grouped per condition and separated per replicate.


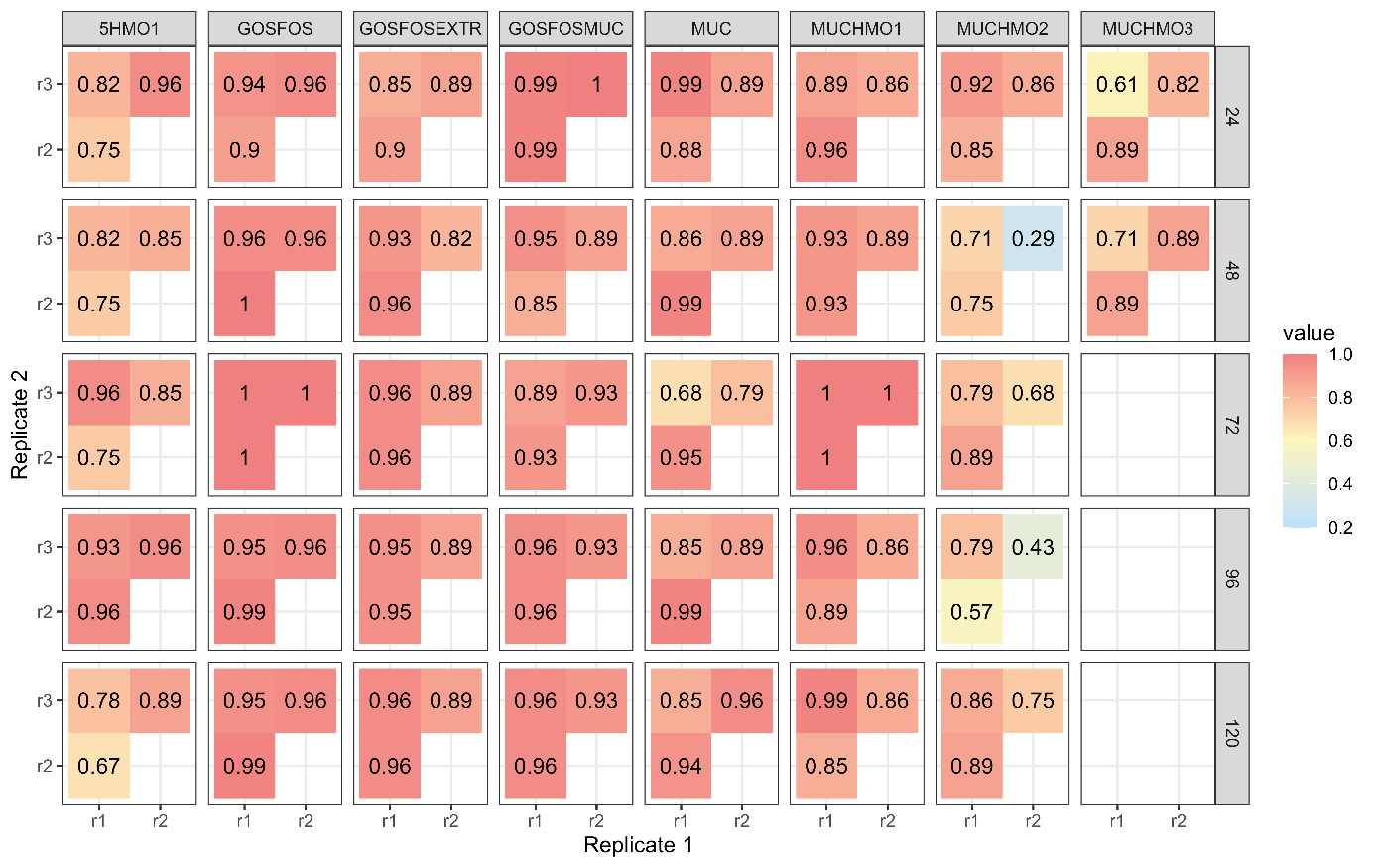


**Supplementary Figure 3:** Spearman correlation coefficient between replicates based on corrected 16S rRNA gene counts, stratified by condition and timepoint.


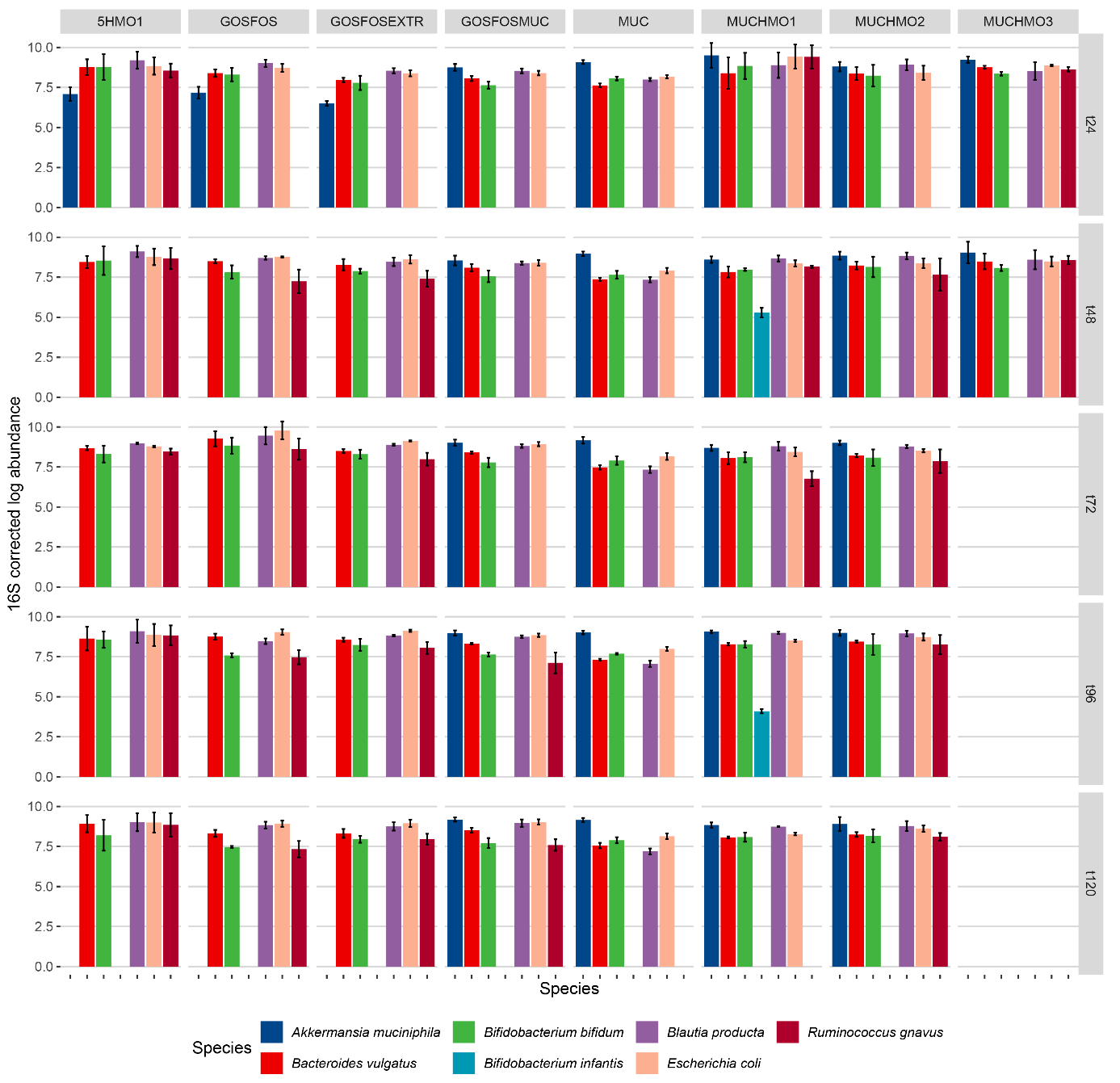


**Supplementary Figure 4:** Log mean absolute abundances of species per condition and timepoint. Error bars represent standard deviation between replicates. The ratio between species was determined through 16S rRNA gene amplicon sequencing and corrected for 16S rRNA gene copy number (Figure 3), and subsequently integrated in total bacterial cell count as determined by qPCR (Supplementary Table 2).


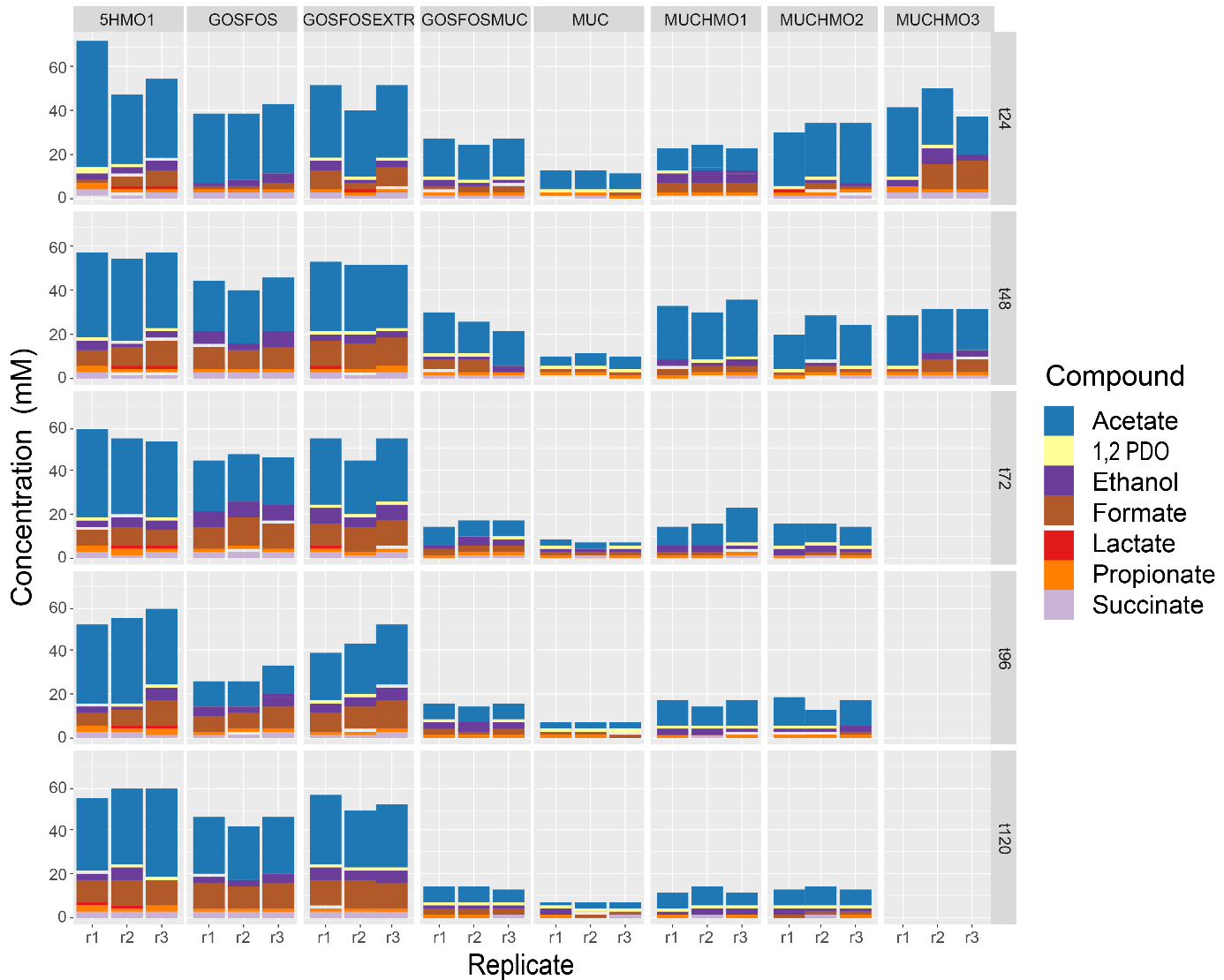


**Supplementary Figure 5:** Metabolite production by BabyBac in different conditions.


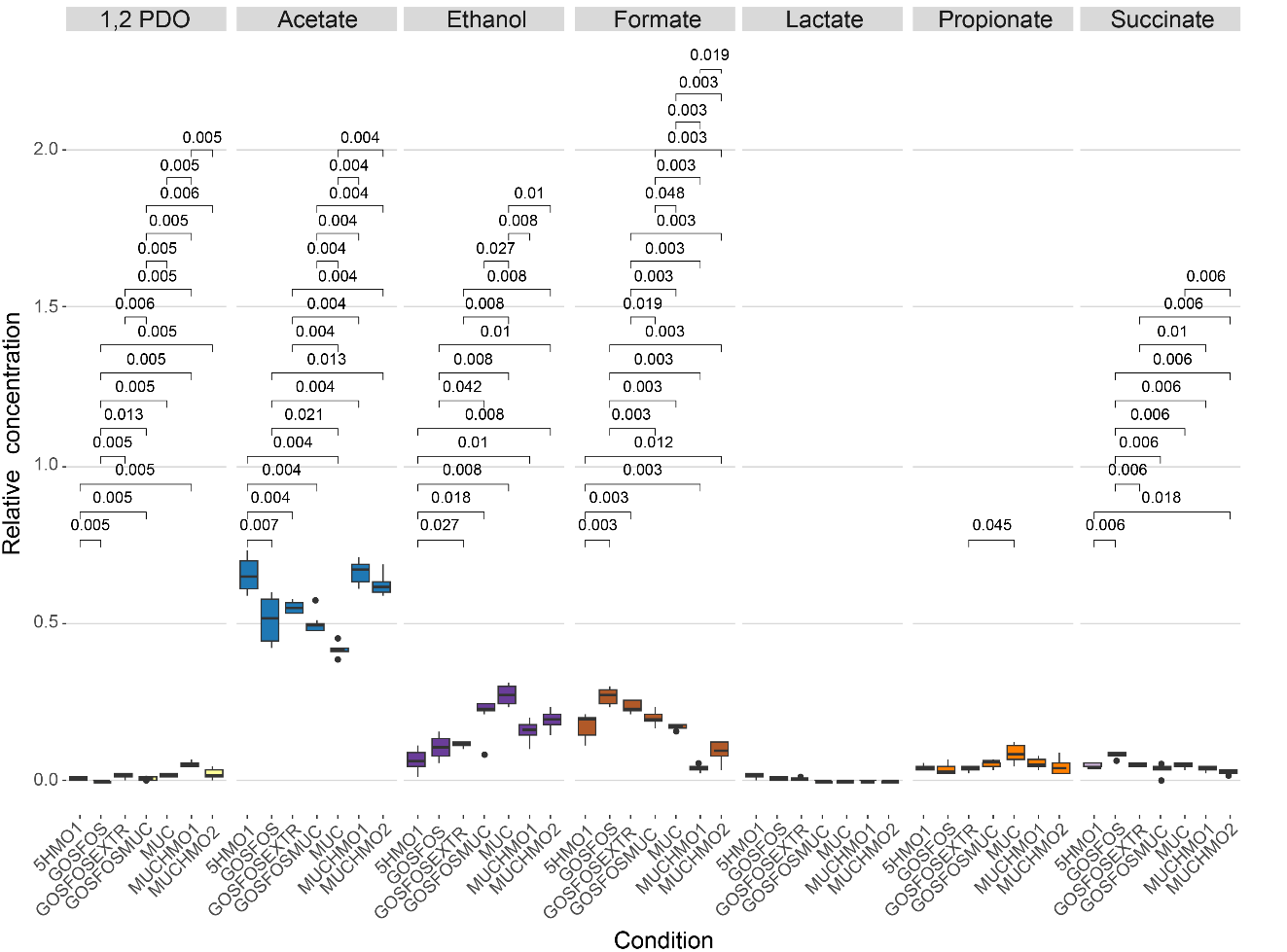


**Supplementary Figure 6:** Statistically significant FDR-corrected Wilcoxon test p-values for comparison of relative concentration of compounds between conditions. The relative concentrations used are from the final timepoints t96 and t120.

**Supplementary Materials References**

Coker JK, Moyne O, Rodionov DA, Zengler K. Carbohydrates great and small, from dietary fiber to sialic acids: How glycans influence the gut microbiome and affect human health. *Gut Microbes* 2021;13(1):1-18. https://doi.org/10.1080/19490976.2020.1869502

Crost EH, Tailford LE, Le Gall G, Fons M, Henrissat B, Juge N*.* Utilisation of mucin glycans by the human gut symbiont *Ruminococcus gnavus* is strain-dependent. *PLoS One* 2013;8(10): e76341. https://doi.org/10.1371/journal.pone.0076341

Crost EH, Tailford LE, Monestier M, Swarbreck D, Henrissat B, Crossman LC, Juge N. The mucin-degradation strategy of *Ruminococcus gnavus*: The importance of intramolecular trans-sialidases. *Gut Microbes* 2016;7(4):302-312. https://doi.org/10.1080/19490976.2016.1186334

Derrien M, Vaughan EE, Plugge CM, de Vos WM. *Akkermansia municiphila* gen. nov., sp. nov., a human intestinal mucin-degrading bacterium. *International Journal of Systematic and Evolutionary Microbiology* 2004;54:1469–76. https://doi.org/10.1099/ijs.0.02873-0

Garrido D, Ruiz-Moyano S, Jimenez-Espinoza R, Eom HJ, Block DE, Mills DA. Utilization of galactooligosaccharides by *Bifidobacterium longum* subsp. *infantis* isolates. *Food Microbiology* 2013;33(2):262-270. https://doi.org/10.1016/j.fm.2012.10.003

Huang YL, Chassard C, Hausmann M, von Itzstein M, Hennet T. Sialic acid catabolism drives intestinal inflammation and microbial dysbiosis in mice. *Nature Communications* 2015;6:8141. https://doi.org/10.1038/ncomms9141

Ioannou A, Berkhout MD, Scott WT, Blijenberg B, Boeren S, Mank M, Knol J, Belzer C. Resource sharing of an infant gut microbiota synthetic community in combinations of human milk oligosaccharides. *The ISME Journal* 2024;18(1): wrae209. https://doi.org/10.1093/ismejo/wrae209

Kostopoulos I, Elzinga J, Ottman N, Klievink JT, Blijenberg B, Aalvink S, Boeren S, Mank M, Knol J, de Vos WM, Belzer C. *Akkermansia muciniphila* uses human milk oligosaccharides to thrive in the early life conditions *in vitro*. *Scientific Reports* 2020;10(1):14330. https://doi.org/10.1038/s41598-020-71113-8

Morozumi M, Wada Y, Tsuda M, Tabata F, Ehara T, Nakamura H, Miyaji K. Cross-feeding among bifidobacteria on glycomacropeptide. *Journal of Functional Foods* 2023;103:105463. https://doi.org/10.1016/j.jff.2023.105463

Padilla L, Fricker AD, Luna E, Choudhury B, Hughes ER, Panzetta ME, Valdivia RH, Flores GE. Mechanism of 2'-fucosyllactose degradation by human-associated *Akkermansia*. *Journal of Bacteriology* 2024;206(2):e0033423. https://doi.org/10.1128/jb.00334-23

Perrin S, Warchol M, Grill JP, Schneider F. Fermentations of fructo‐oligosaccharides and their components by *Bifidobacterium infantis* ATCC 15697 on batch culture in semi‐synthetic medium. *Journal of Applied Microbiology* 2001;90(6):859–865. https://doi.org/10.1046/j.1365-2672.2001.01317.x

Salli K, Hirvonen J, Siitonen J, Ahonen I, Anglenius H, Maukonen J. Selective Utilization of the Human Milk Oligosaccharides 2′-Fucosyllactose, 3-Fucosyllactose, and Difucosyllactose by Various Probiotic and Pathogenic Bacteria. *Journal of Agricultural and Food Chemistry* 2021;69(1):170-182. https://doi.org/10.1021/acs.jafc.0c06041

Shuoker B, Pichler MJ, Jin C, Sakanaka H, Wu H, Martínez Gascueña A, Liu J, Nielsen TS, Holgersson J, Nordberg Karlsson E, Juge N, Meier S, Morth JP, Karlsson NG, Abou Hachem M. Sialidases and fucosidases of *Akkermansia muciniphila* are crucial for growth on mucin and nutrient sharing with mucus-associated gut bacteria. *Nature Communications* 2023;14:1833. https://doi.org/10.1038/s41467-023-37533-6

Turroni F, Strati F, Foroni E, Serafini F, Duranti S, van Sinderen D, Ventura M. Analysis of Predicted Carbohydrate Transport Systems Encoded by *Bifidobacterium bifidum* PRL2010. *Applied and Environmental Microbiology* 2012;78(14). https://doi.org/10.1128/AEM.00629-12

You HJ, Si J, Kim J, Yoon S, Cha KH, Yoon HS, Lee G, Yu J, Choi JS, Jung M, Kim DJ, Lee Y, Kim M, Vázquez-Castellanos JF, Sung J, Park JM, Ko G. *Bacteroides vulgatus* SNUG 40005 Restores *Akkermansia* Depletion by Metabolite Modulation. Gastroenterology 2023;164(1):103-116. https://doi.org/10.1053/j.gastro.2022.09.040
