# Supplementary material for: Milk and mucin glycans orchestrate a synthetic infant gut microbiota structure
Source: FEMS Microbiol Ecol. 2025 Jun 25;101(8):fiaf069. doi: 10.1093/femsec/fiaf069 (PMC12268331; doi:10.1093/femsec/fiaf069)
Supplement: fiaf069_Supplemental_Files [file fiaf069_supplemental_files.zip › Supplementary_Data_1_Belzer_HPLC_run1.pdf]

Sequence Overview

Sequence Details

Name:	**20230116 Synthetic community**	*Created On:*	**08/Dec/17 07:28:29**
Directory:	**HPLC-DATA\8_LC-2030C\Data2023\Maryse**	*Created By:*	**HPLC install**
Data Vault:	**AZR_CHROM_DATA_MIB-SSB**	*Updated On:*	**11/Apr/24 13:16:59**
No. of Injections:	**78**	*Updated By:*	**mib007**

Injection Details

No.	Injection Name	Position	Type	Level	Amount<br>Lactate RI	Amount<br>Succinate RI
1	water 1	3:1	Unknown		n.a.	n.a.
2	no injection	3:1	Blank		n.a.	n.a.
3	VFA 10	3:2	Calibration Standard	1	9,648616443	n.a.
4	VFA 20	3:2	Calibration Standard	1	19,77831766	n.a.
5	VFA 30	3:2	Calibration Standard	1	30,26491608	n.a.
6	1,2-Prop & 1- propane	3:3	Calibration Standard	1	n.a.	n.a.
7	1,2-Prop & 1- propane	3:3	Calibration Standard	1	n.a.	n.a.
8	1,2-Prop & 1- propane	3:3	Calibration Standard	1	n.a.	n.a.
9	Meth & Eth 100	3:4	Calibration Standard	3	n.a.	n.a.
10	Meth & Eth 200	3:4	Calibration Standard	3	n.a.	n.a.
11	Meth & Eth 300	3:4	Calibration Standard	3	n.a.	n.a.
12	16.93 acetate	3:5	Unknown		n.a.	n.a.
13	13.68 propionate	3:6	Unknown		n.a.	n.a.
14	GOSFOS t72 r1	3:7	Unknown		n.a.	2,891586975
15	GOSFOS t72 r2	3:8	Unknown		n.a.	3,592677347
16	GOSFOS t72 r3	3:9	Unknown		n.a.	3,419510607
17	GOSFOS t96 r1	3:10	Unknown		n.a.	2,284566741
18	GOSFOS t96 r2	3:11	Unknown		n.a.	2,141761071
19	GOSFOS t96 r3	3:12	Unknown		n.a.	2,539020404
20	GOSFOS t120 r1	3:13	Unknown		n.a.	3,000964627
21	GOSFOS t120 r2	3:14	Unknown		n.a.	6,452393118
22	GOSFOS t120 r3	3:15	Unknown		n.a.	3,020505797
23	GOSFOSEXTR t72 r1	3:16	Unknown		0,09004758702	2,60212514
24	GOSFOSEXTR t72 r2	3:17	Unknown		0,9074970478	3,766777953
25	GOSFOSEXTR t72 r3	3:18	Unknown		0,3260237694	3,018879189
26	GOSFOSEXTR t96 r	3:19	Unknown		0,08503662541	1,809464203
27	GOSFOSEXTR t96 r	3:20	Unknown		0,6102553782	4,603889086
28	GOSFOSEXTR t96 r	3:21	Unknown		0,250773452	2,732408969
29	GOSFOSEXTR t120	3:22	Unknown		0,17735526	2,691534003
30	GOSFOSEXTR t120	3:23	Unknown		0,2420446547	2,444979998
31	GOSFOSEXTR t120	3:24	Unknown		0,3201779102	2,522212354
32	MUCHMO1 t24 r1	3:25	Unknown		0,02610286264	0,5714063409
33	MUCHMO1 t24 r2	3:26	Unknown		0,2408460901	0,7092764961
34	MUCHMO1 t24 r3	3:27	Unknown		n.a.	0,6387855769
35	MUCHMO1 t72 r1	3:28	Unknown		n.a.	0,5132840776
36	MUCHMO1 t72 r2	3:29	Unknown		n.a.	0,5378882975
37	MUCHMO1 t72 r3	3:30	Unknown		n.a.	0,8654249769
38	MUCHMO1 t96 r1	3:31	Unknown		n.a.	0,6871676799
39	MUCHMO1 t96 r2	3:32	Unknown		n.a.	0,4491885162
40	MUCHMO1 t96 r3	3:33	Unknown		n.a.	0,8142585022
41	MUCHMO1 t120 r1	3:34	Unknown		n.a.	0,4606908244
42	MUCHMO1 t120 r2	3:35	Unknown		n.a.	0,3593753752

Chromeleon (c) Dionex
Version 7.2.10.25868

43	MUCHMO1 t120 r3	3:36	Unknown		n.a.	0,4281002565
44	MUCHMO2 t72 r1	3:37	Unknown		n.a.	0,5687139388
45	MUCHMO2 t72 r2	3:38	Unknown		n.a.	0,2736347354
46	MUCHMO2 t72 r3	3:39	Unknown		n.a.	0,4210811172
47	MUCHMO2 t96 r1	3:40	Unknown		n.a.	0,6647962921
48	MUCHMO2 t96 r2	3:41	Unknown		n.a.	0,1934783986
49	MUCHMO2 t96 r3	3:42	Unknown		n.a.	0,6614837207
50	MUCHMO2 t120 r1	3:43	Unknown		n.a.	0,3729852034
51	MUCHMO2 t120 r2	3:44	Unknown		n.a.	0,4053896093
52	no injection (after disc	3:1	Blank		n.a.	n.a.
53	no injection	3:1	Blank		n.a.	n.a.
54	MUCHMO2 t120 r3	3:45	Unknown		n.a.	0,3927650502
55	MUC t72 r1	3:49	Unknown		n.a.	0,5122248994
56	MUC t72 r2	3:50	Unknown		n.a.	0,2609587733
57	MUC t72 r3	3:51	Unknown		n.a.	0,354399016
58	MUC t96 r1	3:52	Unknown		n.a.	0,3343218659
59	MUC t96 r2	3:53	Unknown		n.a.	0,2641693724
60	MUC t96 r3	3:54	Unknown		n.a.	0,3489853152
61	MUC t120 r1	3:55	Unknown		n.a.	0,3271615769
62	MUC t120 r2	3:56	Unknown		n.a.	0,3177412263
63	MUC t120 r3	3:57	Unknown		n.a.	0,4192772912
64	GOSFOSMUC t72 r1	3:61	Unknown		n.a.	4,121086818
65	GOSFOSMUC t72 r2	3:62	Unknown		n.a.	1,106742275
66	GOSFOSMUC t72 r3	3:63	Unknown		n.a.	0,8064069134
67	GOSFOSMUC t96 r1	3:64	Unknown		n.a.	0,6612867224
68	GOSFOSMUC t96 r2	3:65	Unknown		n.a.	n.a.
69	GOSFOSMUC t96 r3	3:66	Unknown		n.a.	0,7207279571
70	GOSFOSMUC t120 r1	3:67	Unknown		n.a.	0,5692732583
71	GOSFOSMUC t120 r2	3:68	Unknown		n.a.	0,778419825
72	GOSFOSMUC t120 r3	3:69	Unknown		n.a.	0,5785752433
73	Glucose, fructose,glyc	3:85	Calibration Standard	2	n.a.	n.a.
74	Glucose, fructose,glyc	3:85	Calibration Standard	2	n.a.	n.a.
75	Glucose, fructose,glyc	3:85	Calibration Standard	2	n.a.	n.a.
76	citrate,succinate, malt	3:86	Calibration Standard	1	n.a.	9,472404891
77	citrate,succinate, malt	3:86	Calibration Standard	1	n.a.	19,80473965
78	citrate,succinate, malt	3:86	Calibration Standard	1	n.a.	30,30603861

Chromatogram and Results

Injection Details

Injection Name:	**water 1**	*Run Time (min):*	**20,00**
Vial Number:	**3:1**	*Injection Volume:*	**10,00**
Injection Type:	**Unknown**	*Channel:*	**RI_CH_1**
Calibration Level:		*Wavelength:*	**n.a.**
Instrument Method:	**Default method LC2030C 45 gr 20 min**	*Bandwidth:*	**n.a.**
Processing Method:	**Processing Method LC2030 45 gr**	*Dilution Factor:*	**1,0000**
Injection Date/Time:	**16/Jan/23 15:12**	*Sample Weight:*	**1,0000**

Chromatogram

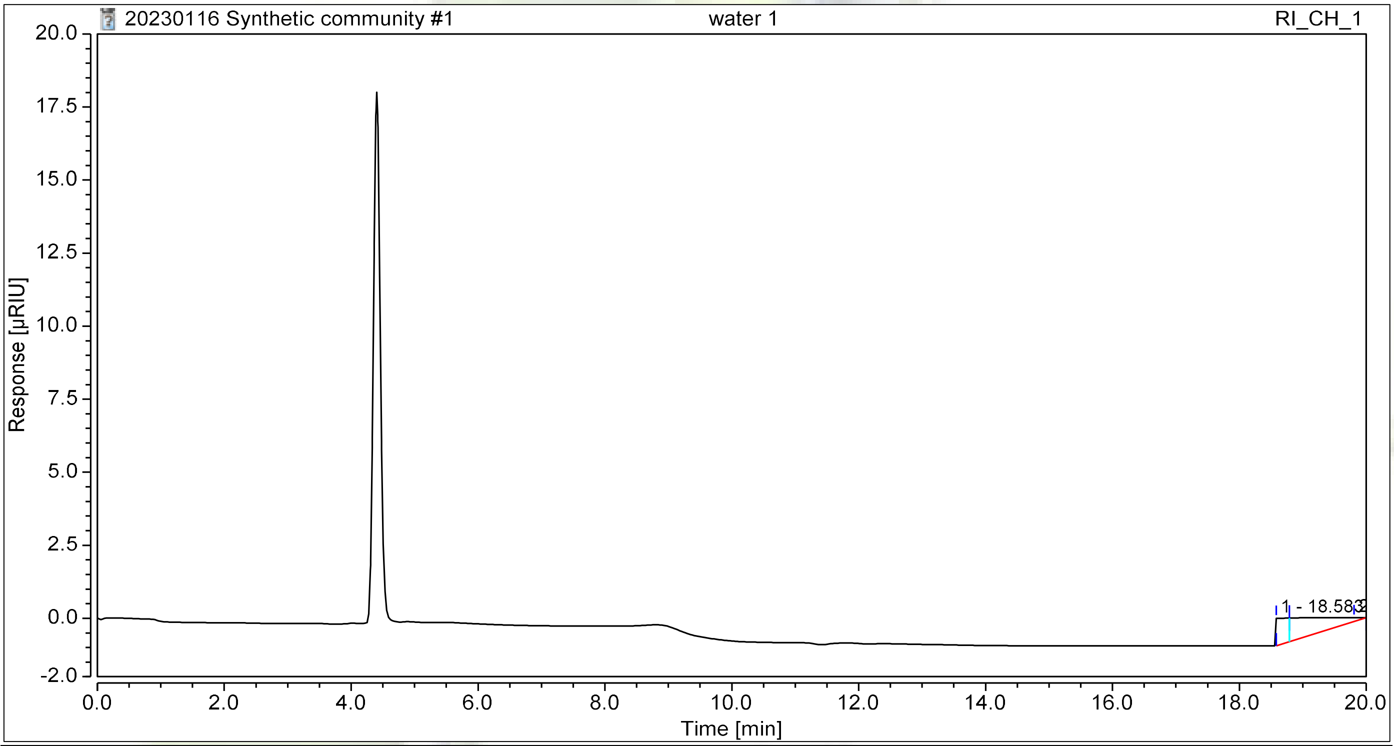

Integration Results

No.	Peak Name	Retention Time<br>min	Area<br>µRIU*min	Height<br>µRIU	Relative Area<br>%	Relative Height<br>%	Amount
n.a.	GlcNAc	n.a.	n.a.	n.a.	n.a.	n.a.	n.a.
n.a.	Citrate	n.a.	n.a.	n.a.	n.a.	n.a.	n.a.
n.a.	Glucose	n.a.	n.a.	n.a.	n.a.	n.a.	n.a.
n.a.	Galactose	n.a.	n.a.	n.a.	n.a.	n.a.	n.a.
n.a.	Fucose	n.a.	n.a.	n.a.	n.a.	n.a.	n.a.
n.a.	Succinate RI	n.a.	n.a.	n.a.	n.a.	n.a.	n.a.
n.a.	Lactate RI	n.a.	n.a.	n.a.	n.a.	n.a.	n.a.
n.a.	glycerol	n.a.	n.a.	n.a.	n.a.	n.a.	n.a.
n.a.	Formate RI	n.a.	n.a.	n.a.	n.a.	n.a.	n.a.
n.a.	Acetate RI	n.a.	n.a.	n.a.	n.a.	n.a.	n.a.
n.a.	1,2 PDO RI	n.a.	n.a.	n.a.	n.a.	n.a.	n.a.
n.a.	1,3-PDO	n.a.	n.a.	n.a.	n.a.	n.a.	n.a.
n.a.	Propionate RI	n.a.	n.a.	n.a.	n.a.	n.a.	n.a.
n.a.	1,3-PDO	n.a.	n.a.	n.a.	n.a.	n.a.	n.a.
n.a.	2-3 BDO	n.a.	n.a.	n.a.	n.a.	n.a.	n.a.
n.a.	Ethanol	n.a.	n.a.	n.a.	n.a.	n.a.	n.a.
n.a.	Isobutyrate RI	n.a.	n.a.	n.a.	n.a.	n.a.	n.a.
n.a.	Butyrate RI	n.a.	n.a.	n.a.	n.a.	n.a.	n.a.
1		18,583	0,180	0,941	26,55	87,70	n.a.
2		19,807	0,497	0,132	73,45	12,30	n.a.
**Total:**			**0,677**	**1,073**	**100,00**	**100,00**	

Chromeleon (c) Dionex
Version 7.2.10.25868

Peak Analysis

Injection Details

Injection Name:	**water 1**	*Run Time (min):*	**20,00**
Vial Number:	**3:1**	*Injection Volume:*	**10,00**
Injection Type:	**Unknown**	*Channel:*	**RI_CH_1**
Calibration Level:		*Wavelength:*	**n.a.**
Instrument Method:	**Default method LC2030C 45 gr 20 min**	*Bandwidth:*	**n.a.**
Processing Method:	**Processing Method LC2030 45 gr**	*Dilution Factor:*	**1,0000**
Injection Date/Time:	**16/Jan/23 15:12**	*Sample Weight:*	**1,0000**

Chromatogram

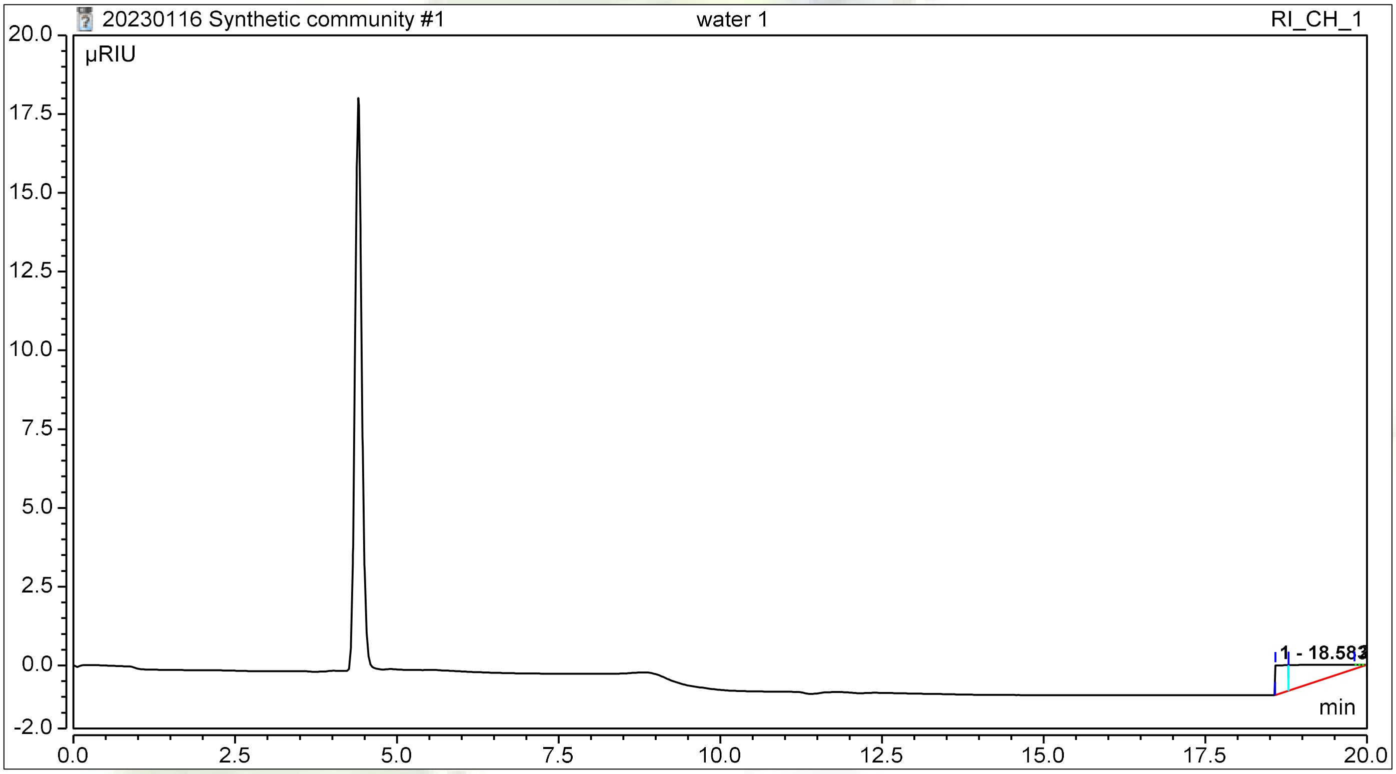

Peak Results

No.	Peak Name	Retention Time min	Width (50%) min	Type	Resolution (EP)	Asymmetry (EP)	Plates (EP)
n.a.	GlcNAc	n.a.	n.a.	n.a.	n.a.	n.a.	n.a.
n.a.	Citrate	n.a.	n.a.	n.a.	n.a.	n.a.	n.a.
n.a.	Glucose	n.a.	n.a.	n.a.	n.a.	n.a.	n.a.
n.a.	Galactose	n.a.	n.a.	n.a.	n.a.	n.a.	n.a.
n.a.	Fucose	n.a.	n.a.	n.a.	n.a.	n.a.	n.a.
n.a.	Succinate RI	n.a.	n.a.	n.a.	n.a.	n.a.	n.a.
n.a.	Lactate RI	n.a.	n.a.	n.a.	n.a.	n.a.	n.a.
n.a.	glycerol	n.a.	n.a.	n.a.	n.a.	n.a.	n.a.
n.a.	Formate RI	n.a.	n.a.	n.a.	n.a.	n.a.	n.a.
n.a.	Acetate RI	n.a.	n.a.	n.a.	n.a.	n.a.	n.a.
n.a.	1,2 PDO RI	n.a.	n.a.	n.a.	n.a.	n.a.	n.a.
n.a.	1,3-PDO	n.a.	n.a.	n.a.	n.a.	n.a.	n.a.
n.a.	Propionate RI	n.a.	n.a.	n.a.	n.a.	n.a.	n.a.
n.a.	1,3-PDO	n.a.	n.a.	n.a.	n.a.	n.a.	n.a.
n.a.	2-3 BDO	n.a.	n.a.	n.a.	n.a.	n.a.	n.a.
n.a.	Ethanol	n.a.	n.a.	n.a.	n.a.	n.a.	n.a.
n.a.	Isobutyrate RI	n.a.	n.a.	n.a.	n.a.	n.a.	n.a.
n.a.	Butyrate RI	n.a.	n.a.	n.a.	n.a.	n.a.	n.a.

Chromeleon (c) Dionex
Version 7.2.10.25868

1		18,583	n.a.	BM	n.a.	n.a.	n.a.
2		19,807	n.a.	MB	n.a.	n.a.	n.a.

Chromatogram and SST Results

Injection Details

Injection Name:	**water 1**	*Run Time (min):*	**20,00**
Vial Number:	**3:1**	*Injection Volume:*	**10,00**
Injection Type:	**Unknown**	*Channel:*	**RI_CH_1**
Calibration Level:		*Wavelength:*	**n.a.**
Instrument Method:	**Default method LC2030C 45 gr 20 min**	*Bandwidth:*	**n.a.**
Processing Method:	**Processing Method LC2030 45 gr**	*Dilution Factor:*	**1,0000**
Injection Date/Time:	**16/Jan/23 15:12**	*Sample Weight:*	**1,0000**

Chromatogram

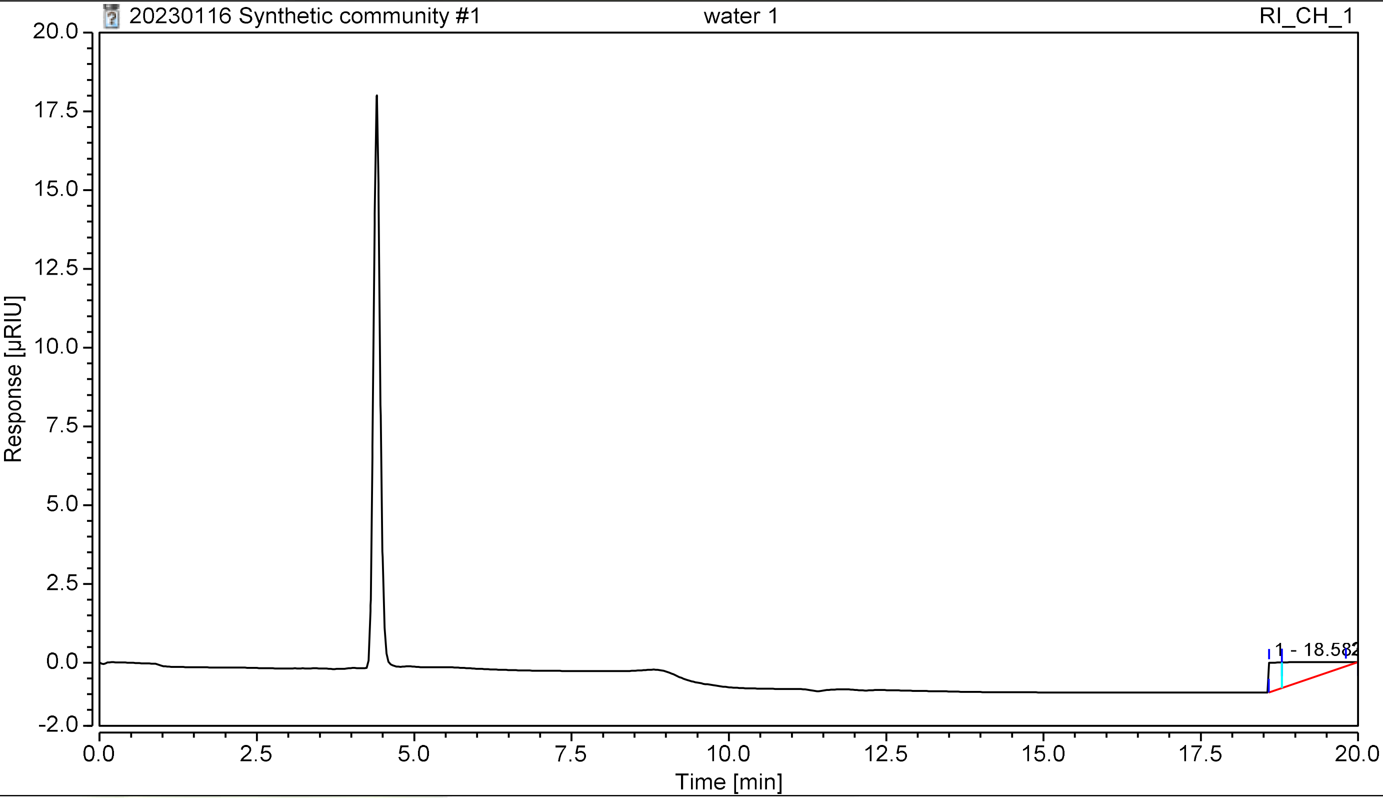

SST Results

No.	Name	Inj.Condition	Peak	Test Result	Injection

Number of executed test cases: **n.a.** **Total Result:** **Passed**

Chromeleon (c) Dionex
Version 7.2.10.25868

Chromatogram and Results

Injection Details

Injection Name:	**no injection**	*Run Time (min):*	**20,00**
Vial Number:	**3:1**	*Injection Volume:*	**10,00**
Injection Type:	**Blank**	*Channel:*	**RI_CH_1**
Calibration Level:		*Wavelength:*	**n.a.**
Instrument Method:	**Default method LC2030C 45 gr 20 min**	*Bandwidth:*	**n.a.**
Processing Method:	**Processing Method LC2030 45 gr**	*Dilution Factor:*	**1,0000**
Injection Date/Time:	**16/Jan/23 15:32**	*Sample Weight:*	**1,0000**

Chromatogram

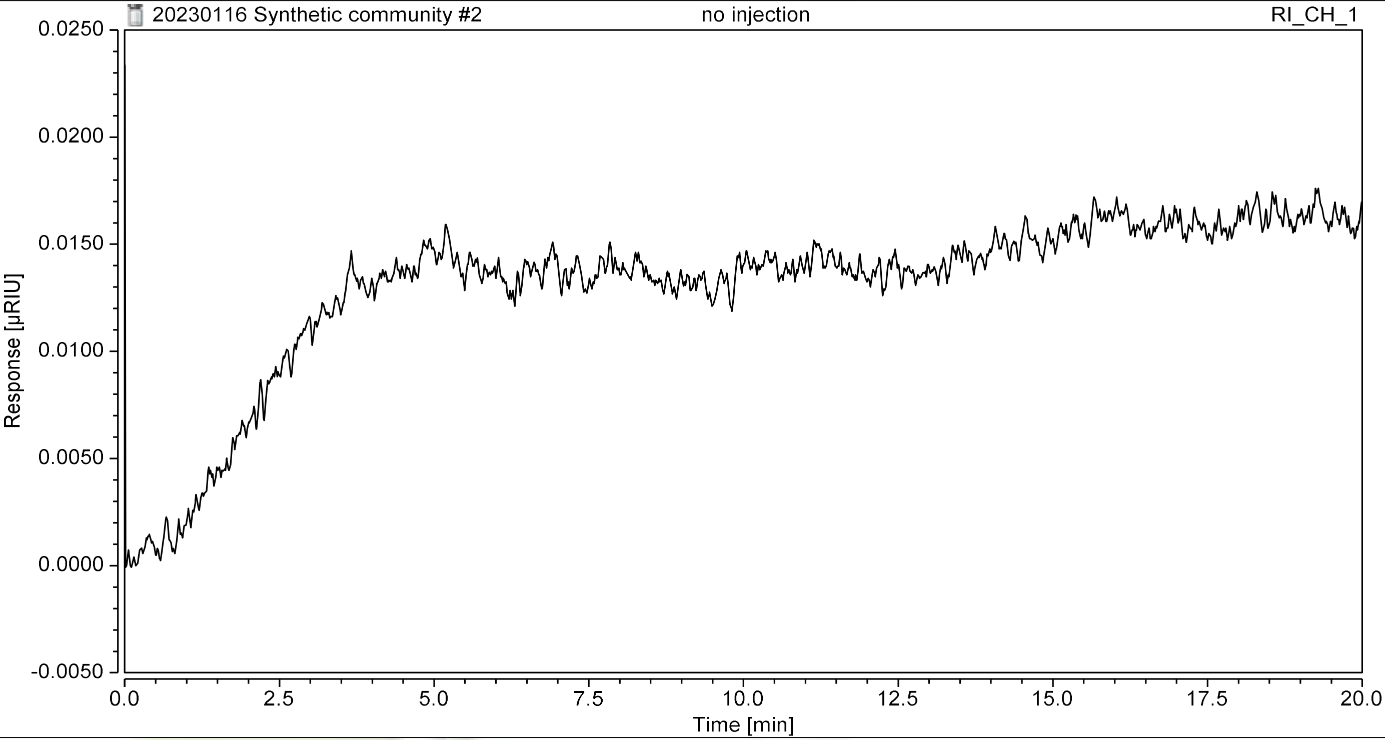

Integration Results

No.	Peak Name	Retention Time min	Area µRIU*min	Height µRIU	Relative Area %	Relative Height %	Amount
n.a.	GlcNAc	n.a.	n.a.	n.a.	n.a.	n.a.	n.a.
n.a.	Citrate	n.a.	n.a.	n.a.	n.a.	n.a.	n.a.
n.a.	Glucose	n.a.	n.a.	n.a.	n.a.	n.a.	n.a.
n.a.	Galactose	n.a.	n.a.	n.a.	n.a.	n.a.	n.a.
n.a.	Fucose	n.a.	n.a.	n.a.	n.a.	n.a.	n.a.
n.a.	Succinate RI	n.a.	n.a.	n.a.	n.a.	n.a.	n.a.
n.a.	Lactate RI	n.a.	n.a.	n.a.	n.a.	n.a.	n.a.
n.a.	glycerol	n.a.	n.a.	n.a.	n.a.	n.a.	n.a.
n.a.	Formate RI	n.a.	n.a.	n.a.	n.a.	n.a.	n.a.
n.a.	Acetate RI	n.a.	n.a.	n.a.	n.a.	n.a.	n.a.
n.a.	1,2 PDO RI	n.a.	n.a.	n.a.	n.a.	n.a.	n.a.
n.a.	1,3-PDO	n.a.	n.a.	n.a.	n.a.	n.a.	n.a.
n.a.	Propionate RI	n.a.	n.a.	n.a.	n.a.	n.a.	n.a.
n.a.	1,3-PDO	n.a.	n.a.	n.a.	n.a.	n.a.	n.a.
n.a.	2-3 BDO	n.a.	n.a.	n.a.	n.a.	n.a.	n.a.
n.a.	Ethanol	n.a.	n.a.	n.a.	n.a.	n.a.	n.a.
n.a.	Isobutyrate RI	n.a.	n.a.	n.a.	n.a.	n.a.	n.a.
n.a.	Butyrate RI	n.a.	n.a.	n.a.	n.a.	n.a.	n.a.
**Total:**			**0,000**	**0,000**	**0,00**	**0,00**	

Peak Analysis

Injection Details

Injection Name:	**no injection**	*Run Time (min):*	**20,00**
Vial Number:	**3:1**	*Injection Volume:*	**10,00**
Injection Type:	**Blank**	*Channel:*	**RI_CH_1**
Calibration Level:		*Wavelength:*	**n.a.**
Instrument Method:	**Default method LC2030C 45 gr 20 min**	*Bandwidth:*	**n.a.**
Processing Method:	**Processing Method LC2030 45 gr**	*Dilution Factor:*	**1,0000**
Injection Date/Time:	**16/Jan/23 15:32**	*Sample Weight:*	**1,0000**

Chromatogram

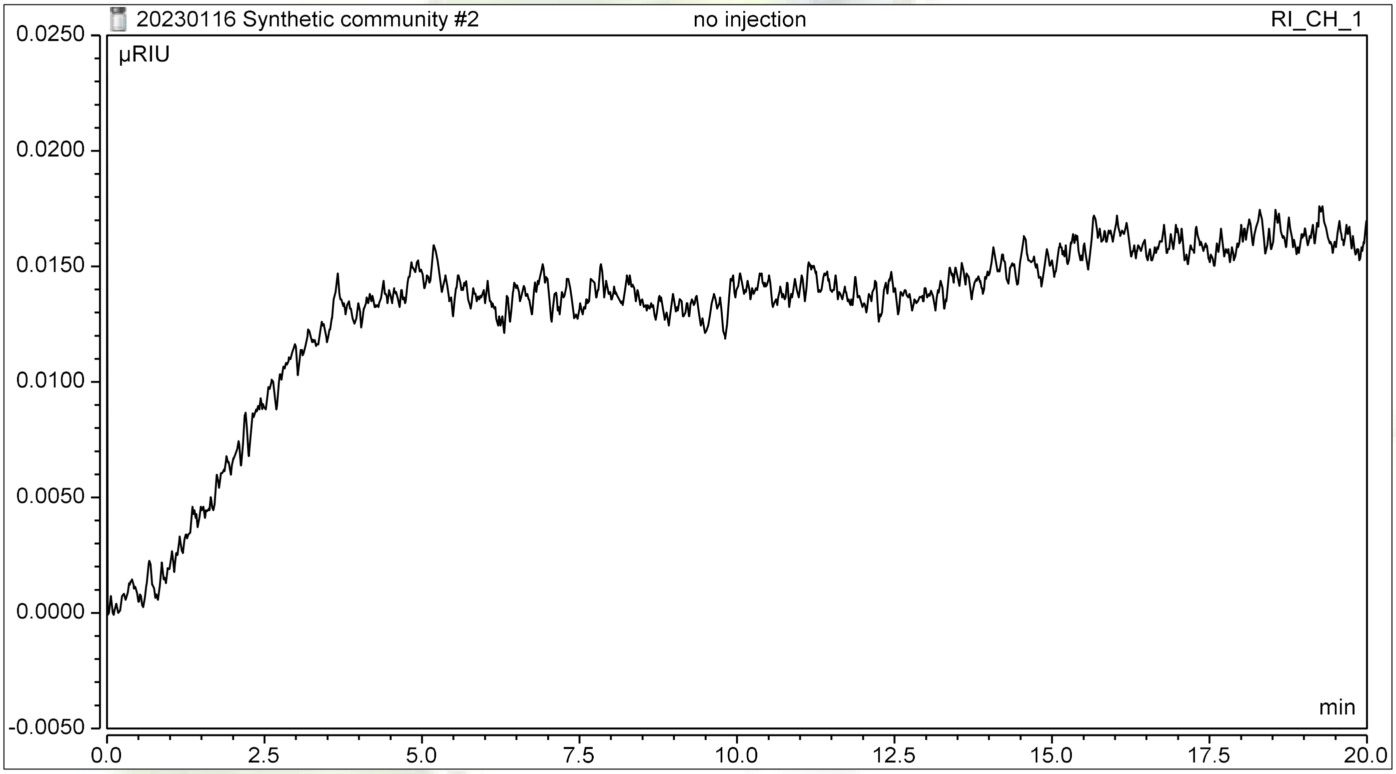

Peak Results

No.	Peak Name	Retention Time min	Width (50%) min	Type	Resolution (EP)	Asymmetry (EP)	Plates (EP)
n.a.	GlcNAc	n.a.	n.a.	n.a.	n.a.	n.a.	n.a.
n.a.	Citrate	n.a.	n.a.	n.a.	n.a.	n.a.	n.a.
n.a.	Glucose	n.a.	n.a.	n.a.	n.a.	n.a.	n.a.
n.a.	Galactose	n.a.	n.a.	n.a.	n.a.	n.a.	n.a.
n.a.	Fucose	n.a.	n.a.	n.a.	n.a.	n.a.	n.a.
n.a.	Succinate RI	n.a.	n.a.	n.a.	n.a.	n.a.	n.a.
n.a.	Lactate RI	n.a.	n.a.	n.a.	n.a.	n.a.	n.a.
n.a.	glycerol	n.a.	n.a.	n.a.	n.a.	n.a.	n.a.
n.a.	Formate RI	n.a.	n.a.	n.a.	n.a.	n.a.	n.a.
n.a.	Acetate RI	n.a.	n.a.	n.a.	n.a.	n.a.	n.a.
n.a.	1,2 PDO RI	n.a.	n.a.	n.a.	n.a.	n.a.	n.a.
n.a.	1,3-PDO	n.a.	n.a.	n.a.	n.a.	n.a.	n.a.
n.a.	Propionate RI	n.a.	n.a.	n.a.	n.a.	n.a.	n.a.
n.a.	1,3-PDO	n.a.	n.a.	n.a.	n.a.	n.a.	n.a.
n.a.	2-3 BDO	n.a.	n.a.	n.a.	n.a.	n.a.	n.a.
n.a.	Ethanol	n.a.	n.a.	n.a.	n.a.	n.a.	n.a.
n.a.	Isobutyrate RI	n.a.	n.a.	n.a.	n.a.	n.a.	n.a.
n.a.	Butyrate RI	n.a.	n.a.	n.a.	n.a.	n.a.	n.a.

Chromatogram and SST Results

Injection Details

Injection Name:	**no injection**	*Run Time (min):*	**20,00**
Vial Number:	**3:1**	*Injection Volume:*	**10,00**
Injection Type:	**Blank**	*Channel:*	**RI_CH_1**
Calibration Level:		*Wavelength:*	**n.a.**
Instrument Method:	**Default method LC2030C 45 gr 20 min**	*Bandwidth:*	**n.a.**
Processing Method:	**Processing Method LC2030 45 gr**	*Dilution Factor:*	**1,0000**
Injection Date/Time:	**16/Jan/23 15:32**	*Sample Weight:*	**1,0000**

Chromatogram

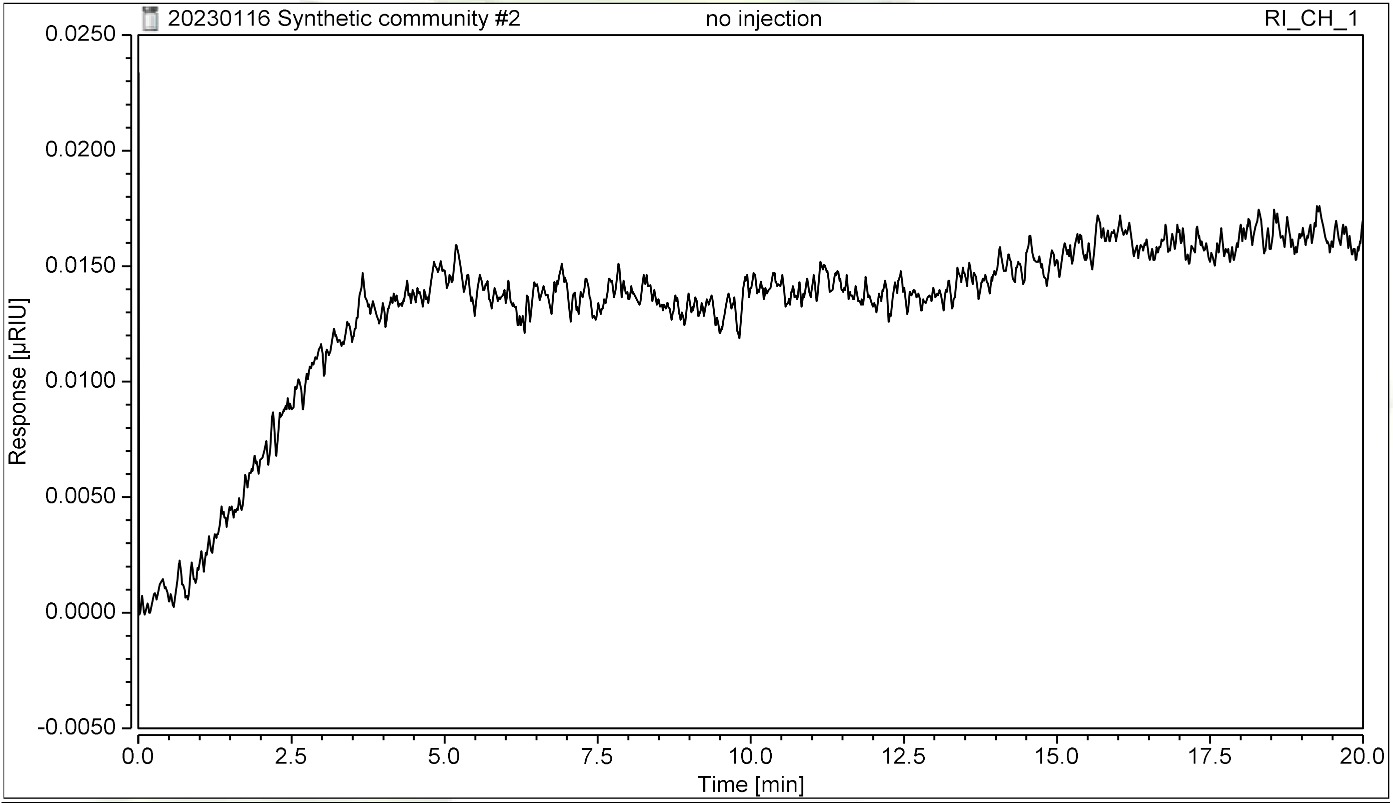

SST Results

No.	Name	Inj.Condition	Peak	Test Result	Injection

Number of executed test cases: **n.a.** **Total Result:** **Passed**

Chromeleon (c) Dionex
Version 7.2.10.25868

Chromatogram and Results

Injection Details

Injection Name:	**VFA 10**	*Run Time (min):*	**20,00**
Vial Number:	**3:2**	*Injection Volume:*	**1,00**
Injection Type:	**Calibration Standard**	*Channel:*	**RI_CH_1**
Calibration Level:	**1**	*Wavelength:*	**n.a.**
Instrument Method:	**Default method LC2030C 45 gr 20 min**	*Bandwidth:*	**n.a.**
Processing Method:	**Processing Method LC2030 45 gr**	*Dilution Factor:*	**1,0000**
Injection Date/Time:	**16/Jan/23 15:53**	*Sample Weight:*	**1,0000**

Chromatogram

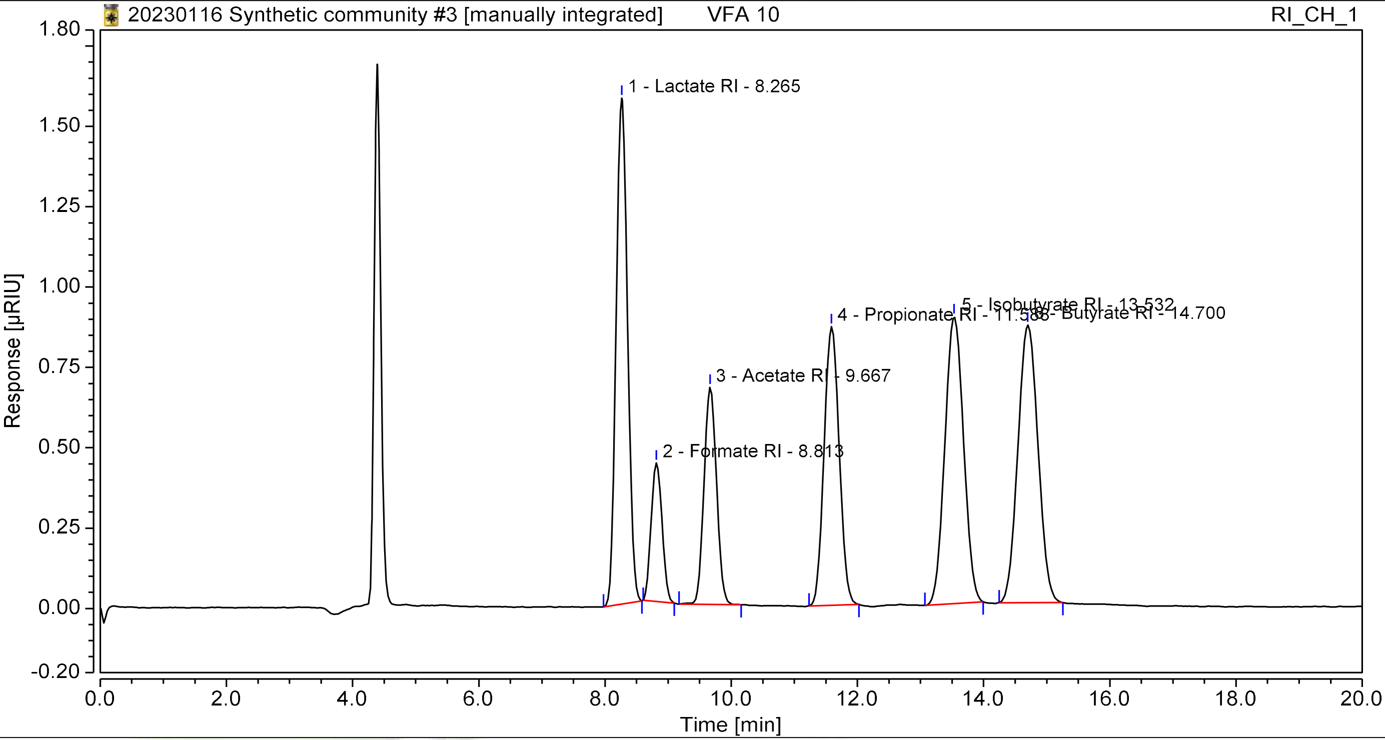

Integration Results

No.	Peak Name	Retention Time min	Area µRIU*min	Height µRIU	Relative Area %	Relative Height %	Amount
n.a.	GlcNAc	n.a.	n.a.	n.a.	n.a.	n.a.	n.a.
n.a.	Citrate	n.a.	n.a.	n.a.	n.a.	n.a.	n.a.
n.a.	Glucose	n.a.	n.a.	n.a.	n.a.	n.a.	n.a.
n.a.	Galactose	n.a.	n.a.	n.a.	n.a.	n.a.	n.a.
n.a.	Fucose	n.a.	n.a.	n.a.	n.a.	n.a.	n.a.
n.a.	Succinate RI	n.a.	n.a.	n.a.	n.a.	n.a.	n.a.
1	Lactate RI	8,265	0,330	1,574	22,89	29,67	9,6486
n.a.	glycerol	n.a.	n.a.	n.a.	n.a.	n.a.	n.a.
2	Formate RI	8,813	0,087	0,431	6,02	8,13	8,8665
3	Acetate RI	9,667	0,156	0,676	10,85	12,74	9,6905
n.a.	1,2 PDO RI	n.a.	n.a.	n.a.	n.a.	n.a.	n.a.
n.a.	1,3-PDO	n.a.	n.a.	n.a.	n.a.	n.a.	n.a.
4	Propionate RI	11,588	0,241	0,867	16,76	16,35	10,0914
n.a.	1,3-PDO	n.a.	n.a.	n.a.	n.a.	n.a.	n.a.
n.a.	2-3 BDO	n.a.	n.a.	n.a.	n.a.	n.a.	n.a.
n.a.	Ethanol	n.a.	n.a.	n.a.	n.a.	n.a.	n.a.
5	Isobutyrate RI	13,532	0,307	0,893	21,34	16,83	9,9941
6	Butyrate RI	14,700	0,319	0,864	22,15	16,28	9,8228
**Total:**			**1,440**	**5,306**	**100,00**	**100,00**	

Peak Analysis

Injection Details

Injection Name:	**VFA 10**	*Run Time (min):*	**20,00**
Vial Number:	**3:2**	*Injection Volume:*	**1,00**
Injection Type:	**Calibration Standard**	*Channel:*	**RI_CH_1**
Calibration Level:	**1**	*Wavelength:*	**n.a.**
Instrument Method:	**Default method LC2030C 45 gr 20 min**	*Bandwidth:*	**n.a.**
Processing Method:	**Processing Method LC2030 45 gr**	*Dilution Factor:*	**1,0000**
Injection Date/Time:	**16/Jan/23 15:53**	*Sample Weight:*	**1,0000**

Chromatogram

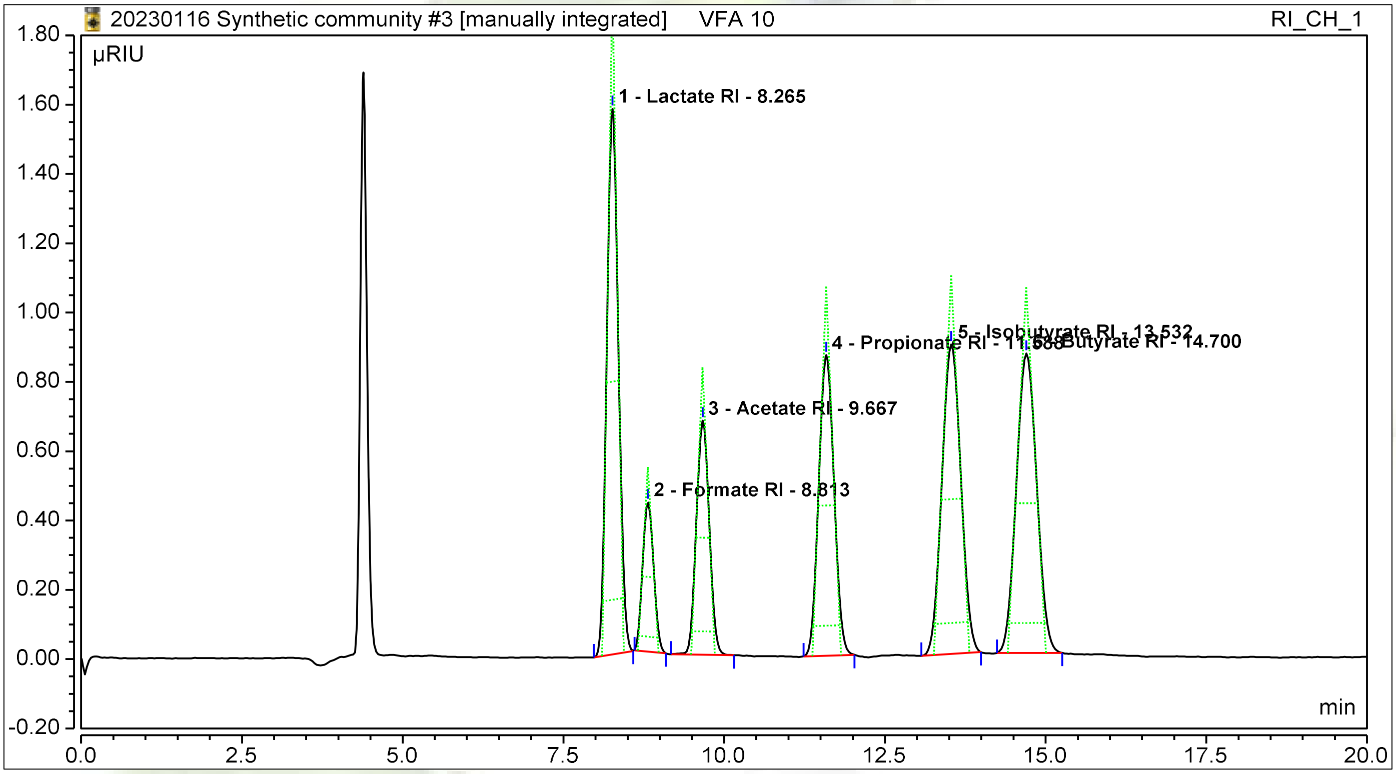

Peak Results

No.	Peak Name	Retention Time min	Width (50%) min	Type	Resolution (EP)	Asymmetry (EP)	Plates (EP)
n.a.	GlcNAc	n.a.	n.a.	n.a.	n.a.	n.a.	n.a.
n.a.	Citrate	n.a.	n.a.	n.a.	n.a.	n.a.	n.a.
n.a.	Glucose	n.a.	n.a.	n.a.	n.a.	n.a.	n.a.
n.a.	Galactose	n.a.	n.a.	n.a.	n.a.	n.a.	n.a.
n.a.	Fucose	n.a.	n.a.	n.a.	n.a.	n.a.	n.a.
n.a.	Succinate RI	n.a.	n.a.	n.a.	n.a.	n.a.	n.a.
1	Lactate RI	8,265	0,199	BMB	1,65	1,07	9596
n.a.	glycerol	n.a.	n.a.	n.a.	n.a.	n.a.	n.a.
2	Formate RI	8,813	0,192	BMB*	2,45	1,09	11624
3	Acetate RI	9,667	0,218	BMB	4,70	1,06	10886
n.a.	1,2 PDO RI	n.a.	n.a.	n.a.	n.a.	n.a.	n.a.
n.a.	1,3-PDO	n.a.	n.a.	n.a.	n.a.	n.a.	n.a.
4	Propionate RI	11,588	0,264	BMB*	3,88	1,06	10659
n.a.	1,3-PDO	n.a.	n.a.	n.a.	n.a.	n.a.	n.a.
n.a.	2-3 BDO	n.a.	n.a.	n.a.	n.a.	n.a.	n.a.
n.a.	Ethanol	n.a.	n.a.	n.a.	n.a.	n.a.	n.a.
5	Isobutyrate RI	13,532	0,327	BMB*	2,03	1,04	9480
6	Butyrate RI	14,700	0,350	BMB*	n.a.	1,05	9751

Chromeleon (c) Dionex
Version 7.2.10.25868

Chromatogram and SST Results

Injection Details

Injection Name:	**VFA 10**	*Run Time (min):*	**20,00**
Vial Number:	**3:2**	*Injection Volume:*	**1,00**
Injection Type:	**Calibration Standard**	*Channel:*	**RI_CH_1**
Calibration Level:	**1**	*Wavelength:*	**n.a.**
Instrument Method:	**Default method LC2030C 45 gr 20 min**	*Bandwidth:*	**n.a.**
Processing Method:	**Processing Method LC2030 45 gr**	*Dilution Factor:*	**1,0000**
Injection Date/Time:	**16/Jan/23 15:53**	*Sample Weight:*	**1,0000**

Chromatogram

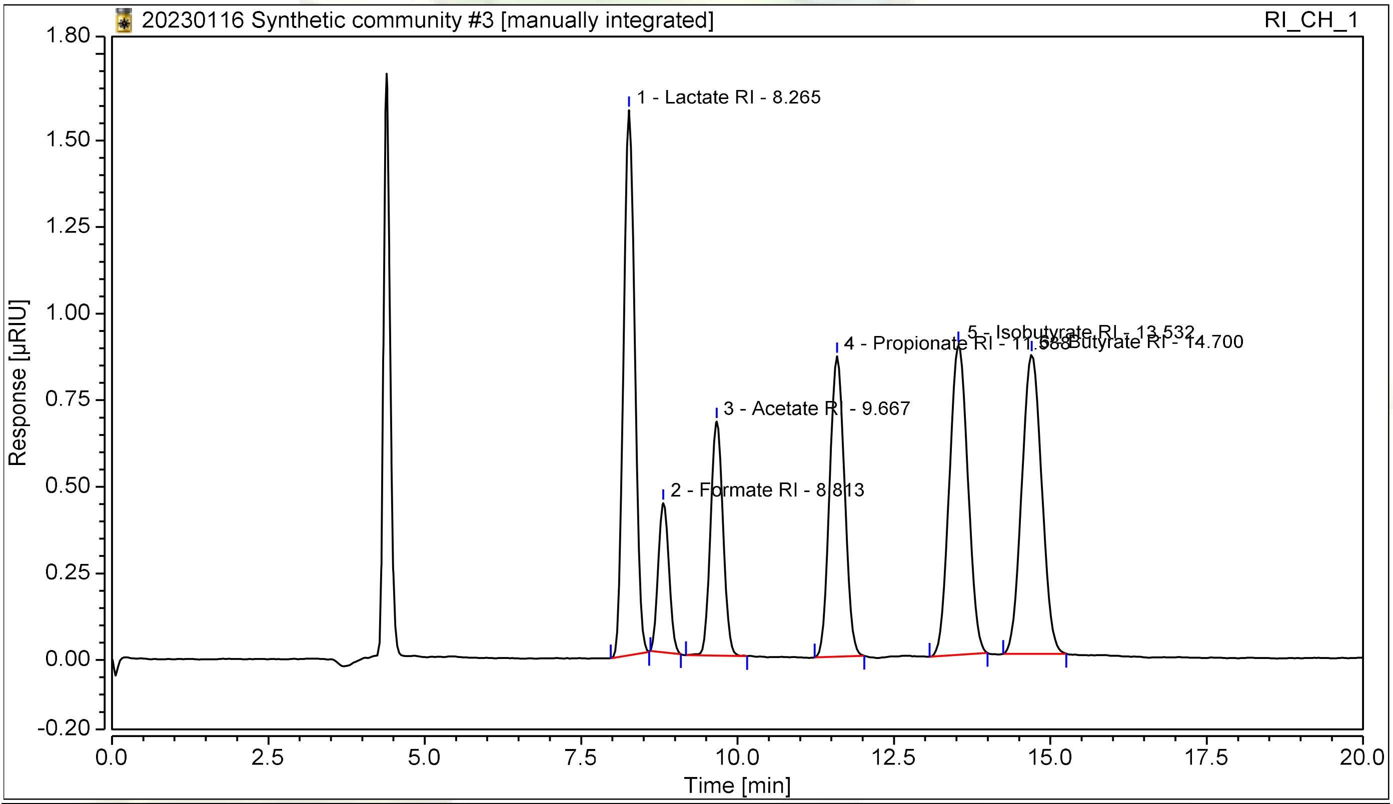

SST Results

No.	Name	Inj.Condition	Peak	Test Result	Injection

Number of executed test cases: **n.a.** **Total Result:** **Passed**

Chromatogram and Results

Injection Details

Injection Name:	**VFA 20**	*Run Time (min):*	**20,00**
Vial Number:	**3:2**	*Injection Volume:*	**2,00**
Injection Type:	**Calibration Standard**	*Channel:*	**RI_CH_1**
Calibration Level:	**1**	*Wavelength:*	**n.a.**
Instrument Method:	**Default method LC2030C 45 gr 20 min**	*Bandwidth:*	**n.a.**
Processing Method:	**Processing Method LC2030 45 gr**	*Dilution Factor:*	**1,0000**
Injection Date/Time:	**16/Jan/23 16:13**	*Sample Weight:*	**1,0000**

Chromatogram

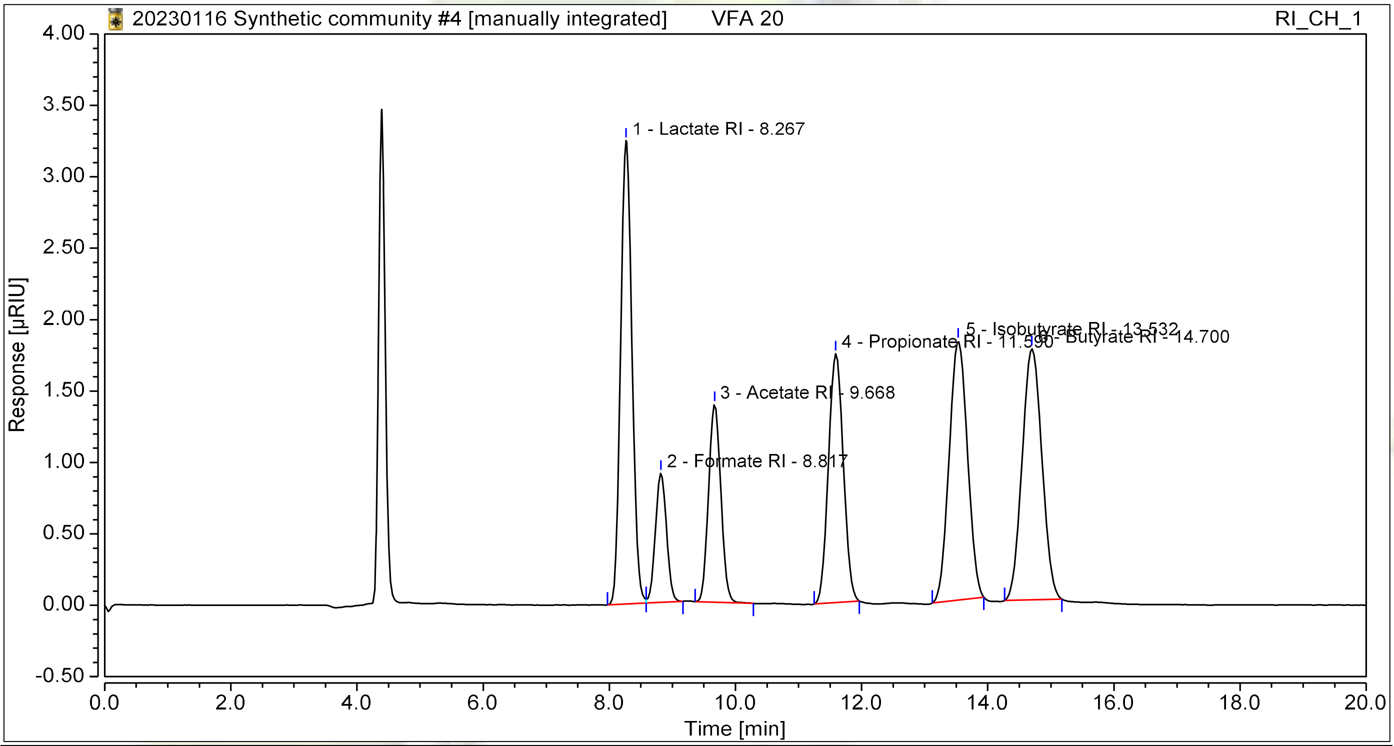

Integration Results

No.	Peak Name	Retention Time min	Area µRIU*min	Height µRIU	Relative Area %	Relative Height %	Amount
n.a.	GlcNAc	n.a.	n.a.	n.a.	n.a.	n.a.	n.a.
n.a.	Citrate	n.a.	n.a.	n.a.	n.a.	n.a.	n.a.
n.a.	Glucose	n.a.	n.a.	n.a.	n.a.	n.a.	n.a.
n.a.	Galactose	n.a.	n.a.	n.a.	n.a.	n.a.	n.a.
n.a.	Fucose	n.a.	n.a.	n.a.	n.a.	n.a.	n.a.
n.a.	Succinate RI	n.a.	n.a.	n.a.	n.a.	n.a.	n.a.
1	Lactate RI	8,267	0,676	3,244	23,28	29,89	19,7783
n.a.	glycerol	n.a.	n.a.	n.a.	n.a.	n.a.	n.a.
2	Formate RI	8,817	0,185	0,906	6,37	8,35	18,9179
3	Acetate RI	9,668	0,315	1,386	10,84	12,77	19,5177
n.a.	1,2 PDO RI	n.a.	n.a.	n.a.	n.a.	n.a.	n.a.
n.a.	1,3-PDO	n.a.	n.a.	n.a.	n.a.	n.a.	n.a.
4	Propionate RI	11,590	0,477	1,742	16,44	16,06	19,9545
n.a.	1,3-PDO	n.a.	n.a.	n.a.	n.a.	n.a.	n.a.
n.a.	2-3 BDO	n.a.	n.a.	n.a.	n.a.	n.a.	n.a.
n.a.	Ethanol	n.a.	n.a.	n.a.	n.a.	n.a.	n.a.
5	Isobutyrate RI	13,532	0,611	1,815	21,06	16,72	19,8709
6	Butyrate RI	14,700	0,639	1,759	22,00	16,21	19,6667
**Total:**			**2,902**	**10,852**	**100,00**	**100,00**	

Chromeleon (c) Dionex
Version 7.2.10.25868

Peak Analysis

Injection Details

Injection Name:	**VFA 20**	*Run Time (min):*	**20,00**
Vial Number:	**3:2**	*Injection Volume:*	**2,00**
Injection Type:	**Calibration Standard**	*Channel:*	**RI_CH_1**
Calibration Level:	**1**	*Wavelength:*	**n.a.**
Instrument Method:	**Default method LC2030C 45 gr 20 min**	*Bandwidth:*	**n.a.**
Processing Method:	**Processing Method LC2030 45 gr**	*Dilution Factor:*	**1,0000**
Injection Date/Time:	**16/Jan/23 16:13**	*Sample Weight:*	**1,0000**

Chromatogram

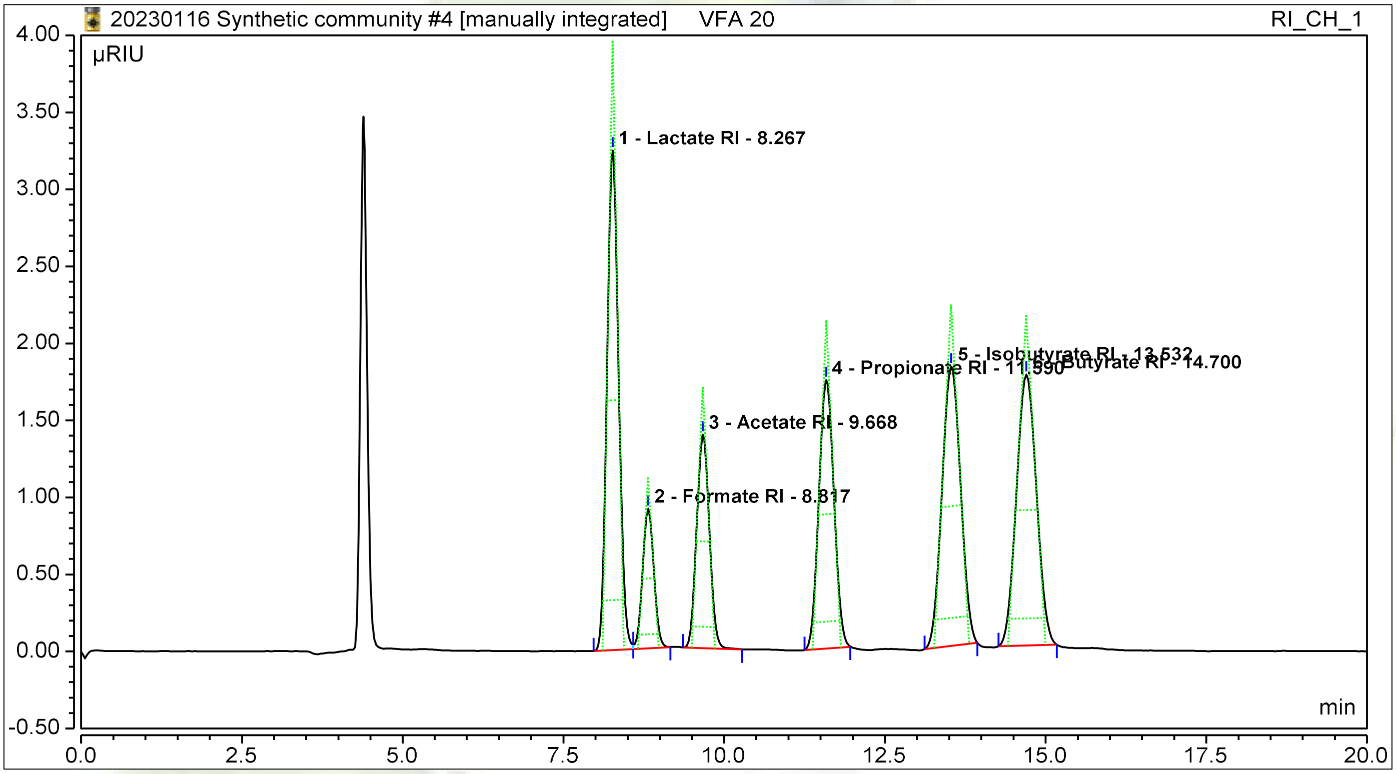

Peak Results

No.	Peak Name	Retention Time min	Width (50%) min	Type	Resolution (EP)	Asymmetry (EP)	Plates (EP)
n.a.	GlcNAc	n.a.	n.a.	n.a.	n.a.	n.a.	n.a.
n.a.	Citrate	n.a.	n.a.	n.a.	n.a.	n.a.	n.a.
n.a.	Glucose	n.a.	n.a.	n.a.	n.a.	n.a.	n.a.
n.a.	Galactose	n.a.	n.a.	n.a.	n.a.	n.a.	n.a.
n.a.	Fucose	n.a.	n.a.	n.a.	n.a.	n.a.	n.a.
n.a.	Succinate RI	n.a.	n.a.	n.a.	n.a.	n.a.	n.a.
1	Lactate RI	8,267	0,196	BM	1,67	1,09	9869
n.a.	glycerol	n.a.	n.a.	n.a.	n.a.	n.a.	n.a.
2	Formate RI	8,817	0,192	MB	2,48	1,04	11717
3	Acetate RI	9,668	0,214	BMB	4,78	1,07	11324
n.a.	1,2 PDO RI	n.a.	n.a.	n.a.	n.a.	n.a.	n.a.
n.a.	1,3-PDO	n.a.	n.a.	n.a.	n.a.	n.a.	n.a.
4	Propionate RI	11,590	0,260	BMB*	3,94	1,05	10988
n.a.	1,3-PDO	n.a.	n.a.	n.a.	n.a.	n.a.	n.a.
n.a.	2-3 BDO	n.a.	n.a.	n.a.	n.a.	n.a.	n.a.
n.a.	Ethanol	n.a.	n.a.	n.a.	n.a.	n.a.	n.a.
5	Isobutyrate RI	13,532	0,321	BMB*	2,07	1,03	9816
6	Butyrate RI	14,700	0,346	BMB*	n.a.	1,04	10019

Chromeleon (c) Dionex
Version 7.2.10.25868

Chromatogram and SST Results

Injection Details

Injection Name:	**VFA 20**	*Run Time (min):*	**20,00**
Vial Number:	**3:2**	*Injection Volume:*	**2,00**
Injection Type:	**Calibration Standard**	*Channel:*	**RI_CH_1**
Calibration Level:	**1**	*Wavelength:*	**n.a.**
Instrument Method:	**Default method LC2030C 45 gr 20 min**	*Bandwidth:*	**n.a.**
Processing Method:	**Processing Method LC2030 45 gr**	*Dilution Factor:*	**1,0000**
Injection Date/Time:	**16/Jan/23 16:13**	*Sample Weight:*	**1,0000**

Chromatogram

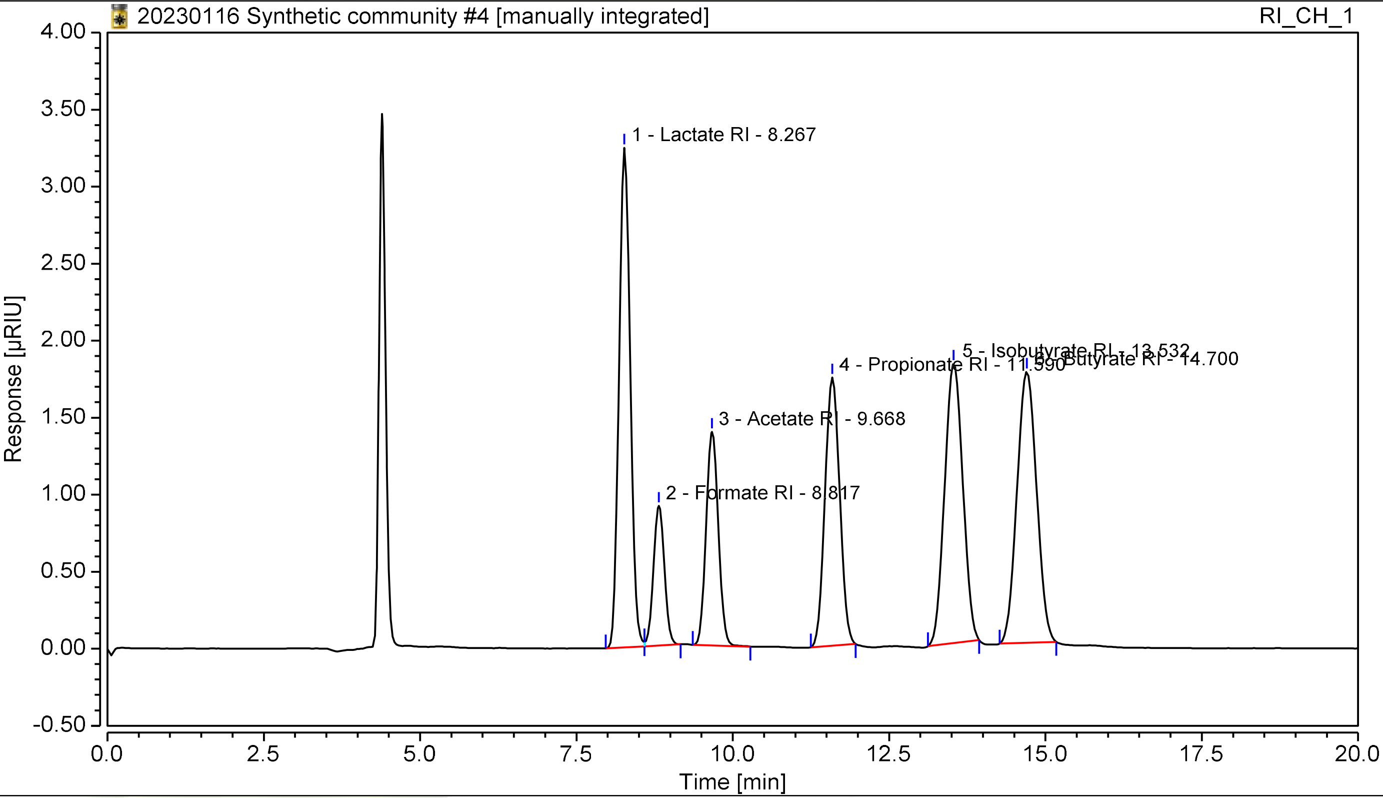

SST Results

No.	Name	Inj.Condition	Peak	Test Result	Injection

Number of executed test cases: **n.a.** **Total Result:** **Passed**

Chromatogram and Results

Injection Details

Injection Name:	**VFA 30**	*Run Time (min):*	**20,00**
Vial Number:	**3:2**	*Injection Volume:*	**3,00**
Injection Type:	**Calibration Standard**	*Channel:*	**RI_CH_1**
Calibration Level:	**1**	*Wavelength:*	**n.a.**
Instrument Method:	**Default method LC2030C 45 gr 20 min**	*Bandwidth:*	**n.a.**
Processing Method:	**Processing Method LC2030 45 gr**	*Dilution Factor:*	**1,0000**
Injection Date/Time:	**16/Jan/23 16:34**	*Sample Weight:*	**1,0000**

Chromatogram

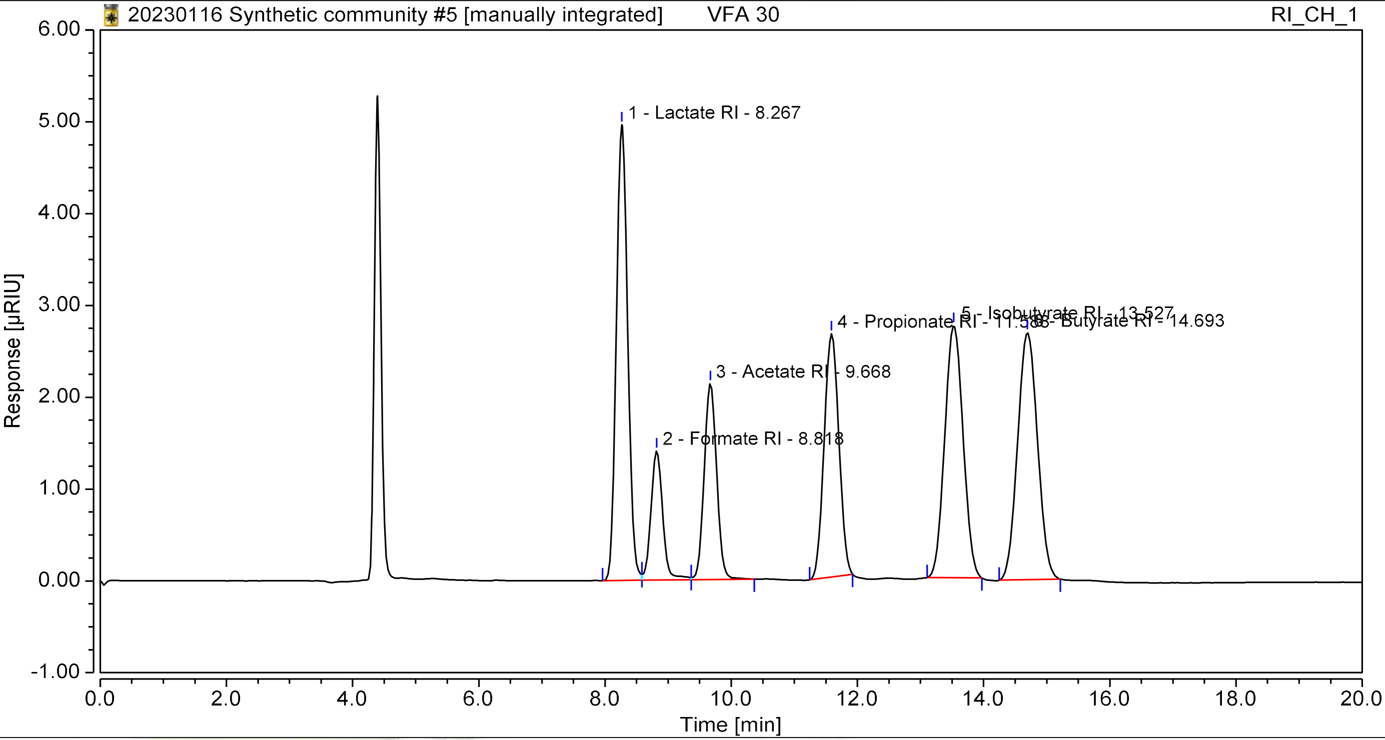

Integration Results

No.	Peak Name	Retention Time min	Area µRIU*min	Height µRIU	Relative Area %	Relative Height %	Amount
n.a.	GlcNAc	n.a.	n.a.	n.a.	n.a.	n.a.	n.a.
n.a.	Citrate	n.a.	n.a.	n.a.	n.a.	n.a.	n.a.
n.a.	Glucose	n.a.	n.a.	n.a.	n.a.	n.a.	n.a.
n.a.	Galactose	n.a.	n.a.	n.a.	n.a.	n.a.	n.a.
n.a.	Fucose	n.a.	n.a.	n.a.	n.a.	n.a.	n.a.
n.a.	Succinate RI	n.a.	n.a.	n.a.	n.a.	n.a.	n.a.
1	Lactate RI	8,267	1,034	4,963	23,21	29,91	30,2649
n.a.	glycerol	n.a.	n.a.	n.a.	n.a.	n.a.	n.a.
2	Formate RI	8,818	0,304	1,408	6,82	8,48	31,0992
3	Acetate RI	9,668	0,491	2,136	11,01	12,87	30,4247
n.a.	1,2 PDO RI	n.a.	n.a.	n.a.	n.a.	n.a.	n.a.
n.a.	1,3-PDO	n.a.	n.a.	n.a.	n.a.	n.a.	n.a.
4	Propionate RI	11,588	0,717	2,649	16,10	15,96	29,9998
n.a.	1,3-PDO	n.a.	n.a.	n.a.	n.a.	n.a.	n.a.
n.a.	2-3 BDO	n.a.	n.a.	n.a.	n.a.	n.a.	n.a.
n.a.	Ethanol	n.a.	n.a.	n.a.	n.a.	n.a.	n.a.
5	Isobutyrate RI	13,527	0,925	2,748	20,77	16,56	30,0880
6	Butyrate RI	14,693	0,983	2,692	22,07	16,22	30,2813
**Total:**			**4,454**	**16,596**	**100,00**	**100,00**	

Peak Analysis

Injection Details

Injection Name:	**VFA 30**	*Run Time (min):*	**20,00**
Vial Number:	**3:2**	*Injection Volume:*	**3,00**
Injection Type:	**Calibration Standard**	*Channel:*	**RI_CH_1**
Calibration Level:	**1**	*Wavelength:*	**n.a.**
Instrument Method:	**Default method LC2030C 45 gr 20 min**	*Bandwidth:*	**n.a.**
Processing Method:	**Processing Method LC2030 45 gr**	*Dilution Factor:*	**1,0000**
Injection Date/Time:	**16/Jan/23 16:34**	*Sample Weight:*	**1,0000**

Chromatogram

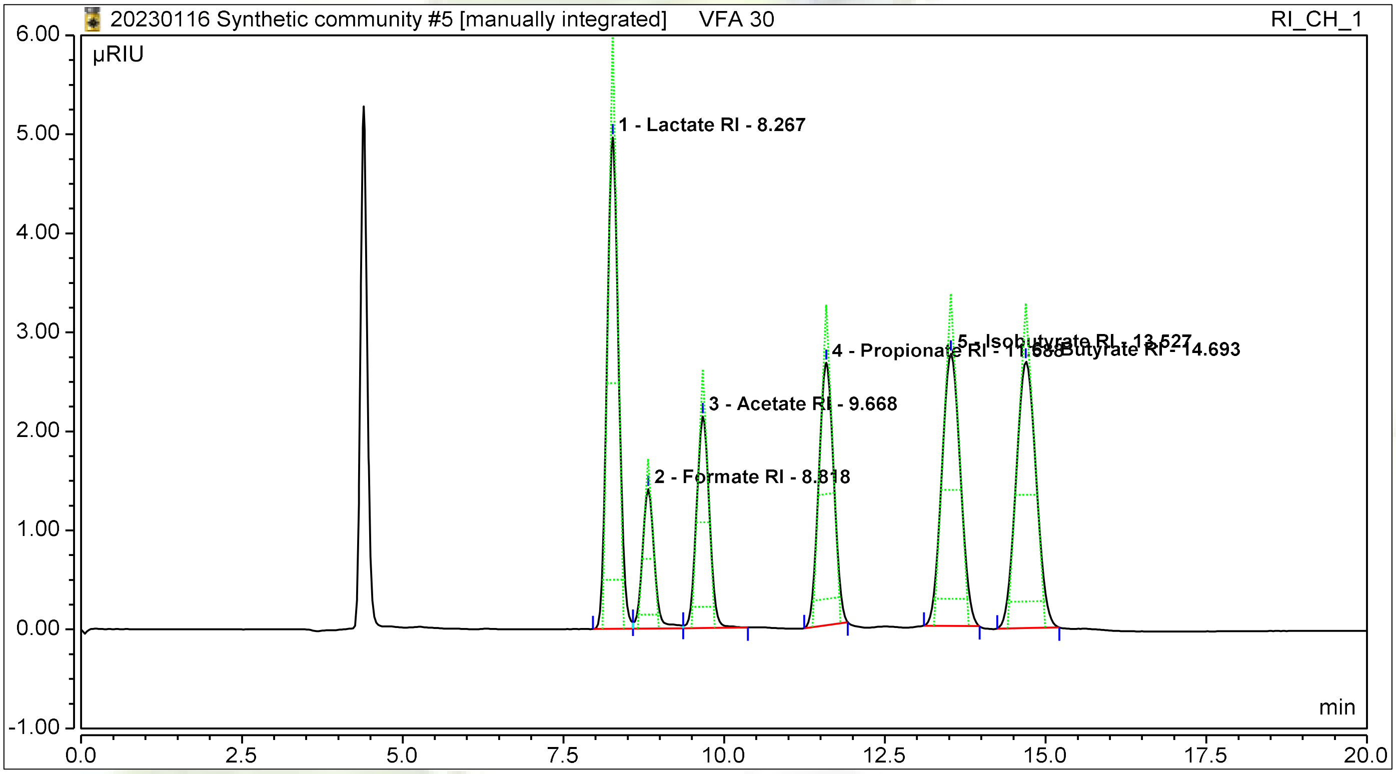

Peak Results

No.	Peak Name	Retention Time min	Width (50%) min	Type	Resolution (EP)	Asymmetry (EP)	Plates (EP)
n.a.	GlcNAc	n.a.	n.a.	n.a.	n.a.	n.a.	n.a.
n.a.	Citrate	n.a.	n.a.	n.a.	n.a.	n.a.	n.a.
n.a.	Glucose	n.a.	n.a.	n.a.	n.a.	n.a.	n.a.
n.a.	Galactose	n.a.	n.a.	n.a.	n.a.	n.a.	n.a.
n.a.	Fucose	n.a.	n.a.	n.a.	n.a.	n.a.	n.a.
n.a.	Succinate RI	n.a.	n.a.	n.a.	n.a.	n.a.	n.a.
1	Lactate RI	8,267	0,195	BM	1,68	1,09	9926
n.a.	glycerol	n.a.	n.a.	n.a.	n.a.	n.a.	n.a.
2	Formate RI	8,818	0,193	M	2,46	1,04	11533
3	Acetate RI	9,668	0,214	MB	4,80	1,06	11326
n.a.	1,2 PDO RI	n.a.	n.a.	n.a.	n.a.	n.a.	n.a.
n.a.	1,3-PDO	n.a.	n.a.	n.a.	n.a.	n.a.	n.a.
4	Propionate RI	11,588	0,258	BMB*	3,95	1,03	11148
n.a.	1,3-PDO	n.a.	n.a.	n.a.	n.a.	n.a.	n.a.
n.a.	2-3 BDO	n.a.	n.a.	n.a.	n.a.	n.a.	n.a.
n.a.	Ethanol	n.a.	n.a.	n.a.	n.a.	n.a.	n.a.
5	Isobutyrate RI	13,527	0,320	BMB*	2,07	1,05	9897
6	Butyrate RI	14,693	0,346	BMB*	n.a.	1,05	9980

Chromatogram and SST Results

Injection Details

Injection Name:	**VFA 30**	*Run Time (min):*	**20,00**
Vial Number:	**3:2**	*Injection Volume:*	**3,00**
Injection Type:	**Calibration Standard**	*Channel:*	**RI_CH_1**
Calibration Level:	**1**	*Wavelength:*	**n.a.**
Instrument Method:	**Default method LC2030C 45 gr 20 min**	*Bandwidth:*	**n.a.**
Processing Method:	**Processing Method LC2030 45 gr**	*Dilution Factor:*	**1,0000**
Injection Date/Time:	**16/Jan/23 16:34**	*Sample Weight:*	**1,0000**

Chromatogram

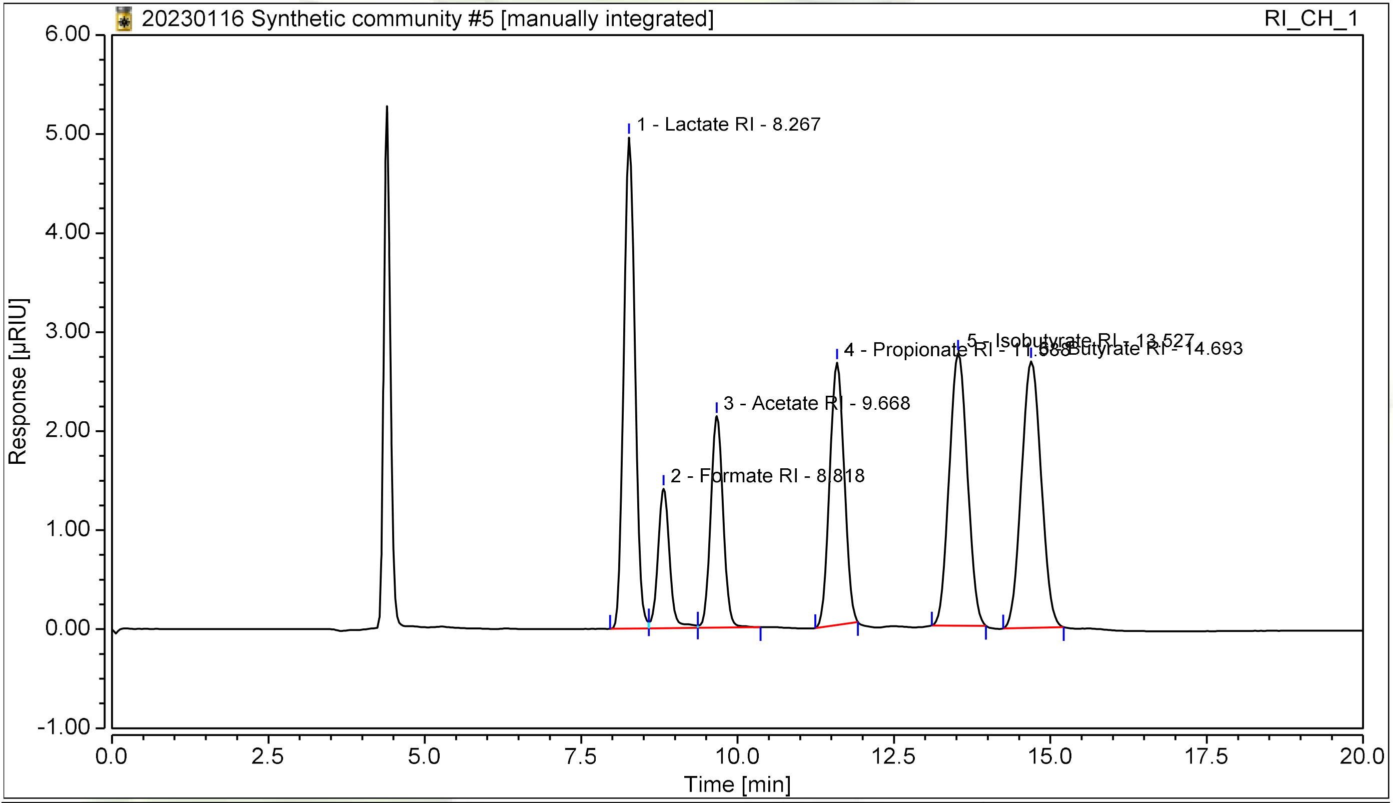

SST Results

No.	Name	Inj.Condition	Peak	Test Result	Injection
**Number of executed test cases:**	**n.a.**		**Total Result:**	**Passed**	

Chromatogram and Results

Injection Details

Injection Name:	**1,2-Prop & 1- propane 10**	*Run Time (min):*	**20,00**
Vial Number:	**3:3**	*Injection Volume:*	**1,00**
Injection Type:	**Calibration Standard**	*Channel:*	**RI_CH_1**
Calibration Level:	**1**	*Wavelength:*	**n.a.**
Instrument Method:	**Default method LC2030C 45 gr 20 min**	*Bandwidth:*	**n.a.**
Processing Method:	**Processing Method LC2030 45 gr**	*Dilution Factor:*	**1,0000**
Injection Date/Time:	**16/Jan/23 16:54**	*Sample Weight:*	**1,0000**

Chromatogram

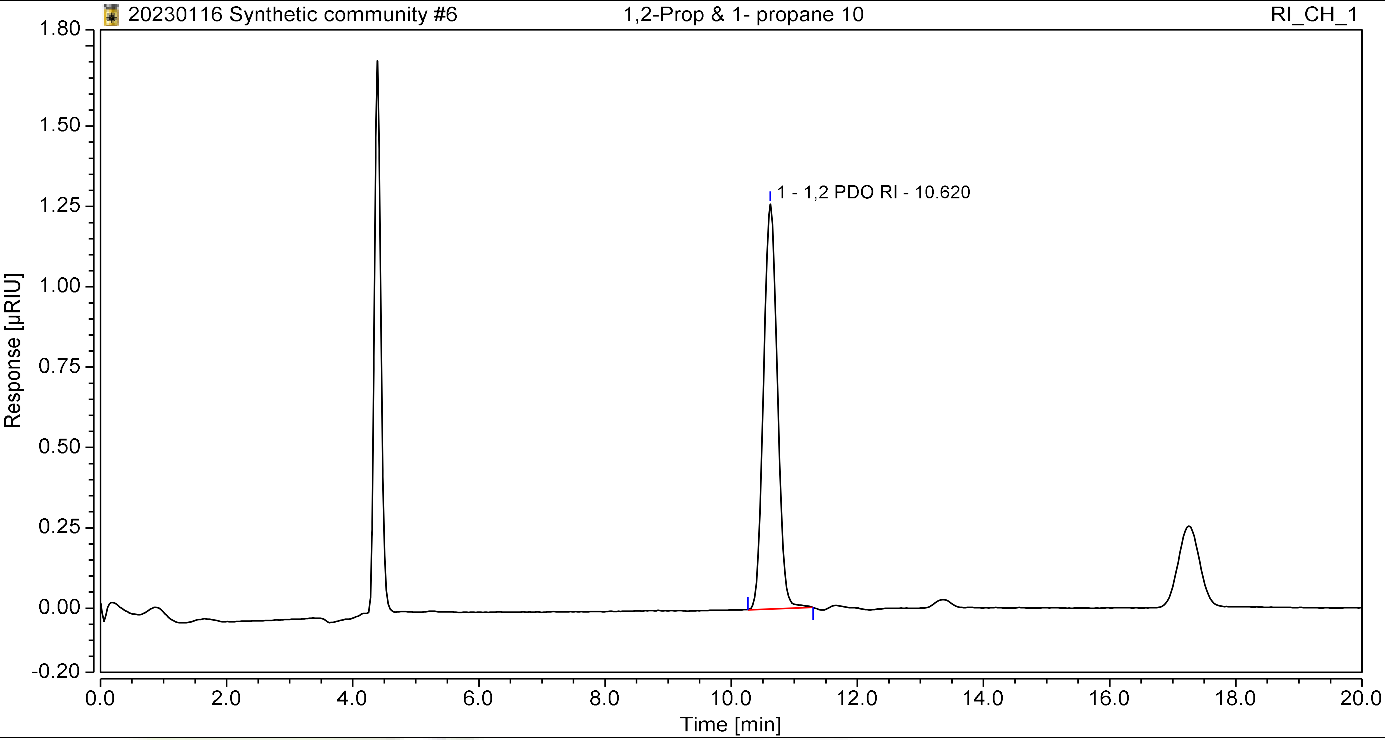

Integration Results

No.	Peak Name	Retention Time min	Area µRIU*min	Height µRIU	Relative Area %	Relative Height %	Amount
n.a.	GlcNAc	n.a.	n.a.	n.a.	n.a.	n.a.	n.a.
n.a.	Citrate	n.a.	n.a.	n.a.	n.a.	n.a.	n.a.
n.a.	Glucose	n.a.	n.a.	n.a.	n.a.	n.a.	n.a.
n.a.	Galactose	n.a.	n.a.	n.a.	n.a.	n.a.	n.a.
n.a.	Fucose	n.a.	n.a.	n.a.	n.a.	n.a.	n.a.
n.a.	Succinate RI	n.a.	n.a.	n.a.	n.a.	n.a.	n.a.
n.a.	Lactate RI	n.a.	n.a.	n.a.	n.a.	n.a.	n.a.
n.a.	glycerol	n.a.	n.a.	n.a.	n.a.	n.a.	n.a.
n.a.	Formate RI	n.a.	n.a.	n.a.	n.a.	n.a.	n.a.
n.a.	Acetate RI	n.a.	n.a.	n.a.	n.a.	n.a.	n.a.
1	1,2 PDO RI	10,620	0,321	1,260	100,00	100,00	9,7842
n.a.	1,3-PDO	n.a.	n.a.	n.a.	n.a.	n.a.	n.a.
n.a.	Propionate RI	n.a.	n.a.	n.a.	n.a.	n.a.	n.a.
n.a.	1,3-PDO	n.a.	n.a.	n.a.	n.a.	n.a.	n.a.
n.a.	2-3 BDO	n.a.	n.a.	n.a.	n.a.	n.a.	n.a.
n.a.	Ethanol	n.a.	n.a.	n.a.	n.a.	n.a.	n.a.
n.a.	Isobutyrate RI	n.a.	n.a.	n.a.	n.a.	n.a.	n.a.
n.a.	Butyrate RI	n.a.	n.a.	n.a.	n.a.	n.a.	n.a.
**Total:**			**0,321**	**1,260**	**100,00**	**100,00**	

Peak Analysis

Injection Details

Injection Name:	**1,2-Prop & 1- propane 10**	*Run Time (min):*	**20,00**
Vial Number:	**3:3**	*Injection Volume:*	**1,00**
Injection Type:	**Calibration Standard**	*Channel:*	**RI_CH_1**
Calibration Level:	**1**	*Wavelength:*	**n.a.**
Instrument Method:	**Default method LC2030C 45 gr 20 min**	*Bandwidth:*	**n.a.**
Processing Method:	**Processing Method LC2030 45 gr**	*Dilution Factor:*	**1,0000**
Injection Date/Time:	**16/Jan/23 16:54**	*Sample Weight:*	**1,0000**

Chromatogram

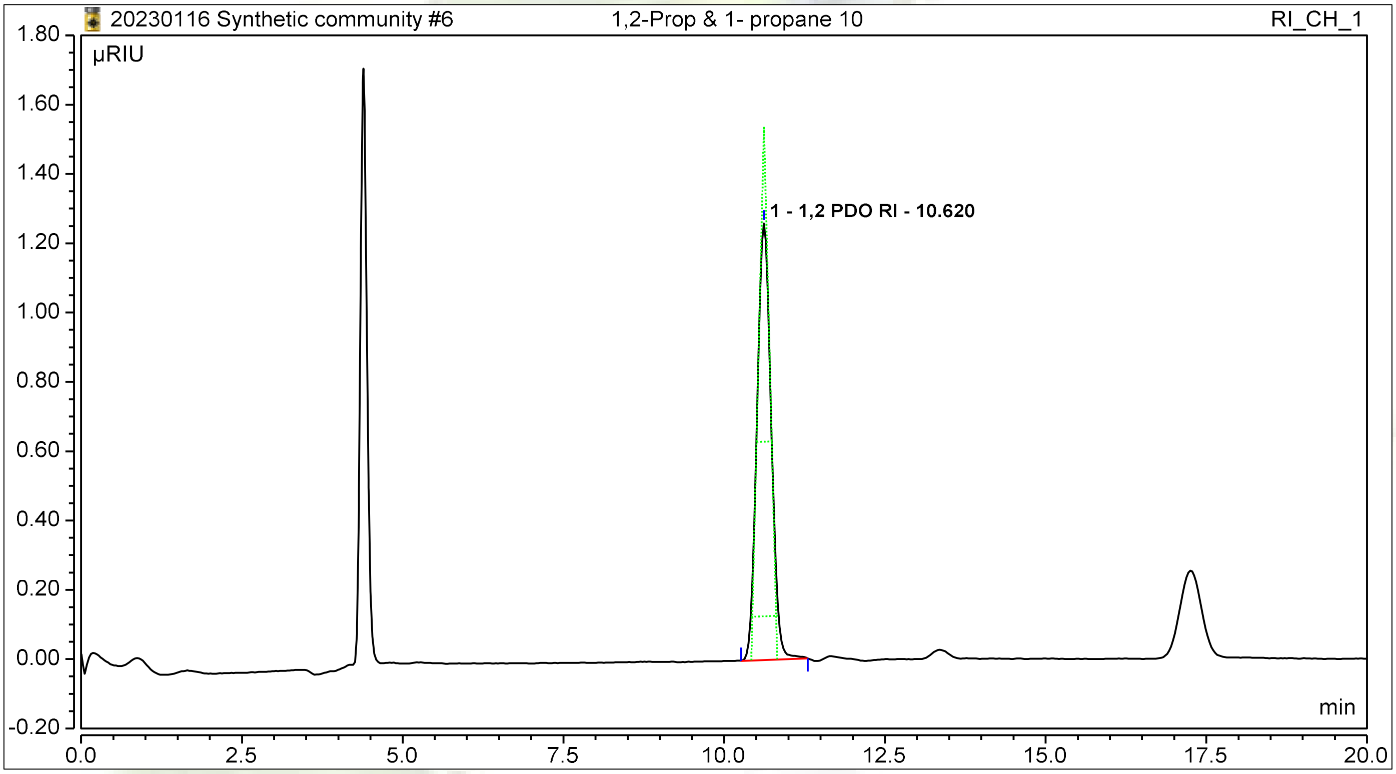

Peak Results

No.	Peak Name	Retention Time min	Width (50%) min	Type	Resolution (EP)	Asymmetry (EP)	Plates (EP)
n.a.	GlcNAc	n.a.	n.a.	n.a.	n.a.	n.a.	n.a.
n.a.	Citrate	n.a.	n.a.	n.a.	n.a.	n.a.	n.a.
n.a.	Glucose	n.a.	n.a.	n.a.	n.a.	n.a.	n.a.
n.a.	Galactose	n.a.	n.a.	n.a.	n.a.	n.a.	n.a.
n.a.	Fucose	n.a.	n.a.	n.a.	n.a.	n.a.	n.a.
n.a.	Succinate RI	n.a.	n.a.	n.a.	n.a.	n.a.	n.a.
n.a.	Lactate RI	n.a.	n.a.	n.a.	n.a.	n.a.	n.a.
n.a.	glycerol	n.a.	n.a.	n.a.	n.a.	n.a.	n.a.
n.a.	Formate RI	n.a.	n.a.	n.a.	n.a.	n.a.	n.a.
n.a.	Acetate RI	n.a.	n.a.	n.a.	n.a.	n.a.	n.a.
1	1,2 PDO RI	10,620	0,239	BMB	n.a.	1,06	10970
n.a.	1,3-PDO	n.a.	n.a.	n.a.	n.a.	n.a.	n.a.
n.a.	Propionate RI	n.a.	n.a.	n.a.	n.a.	n.a.	n.a.
n.a.	1,3-PDO	n.a.	n.a.	n.a.	n.a.	n.a.	n.a.
n.a.	2-3 BDO	n.a.	n.a.	n.a.	n.a.	n.a.	n.a.
n.a.	Ethanol	n.a.	n.a.	n.a.	n.a.	n.a.	n.a.
n.a.	Isobutyrate RI	n.a.	n.a.	n.a.	n.a.	n.a.	n.a.
n.a.	Butyrate RI	n.a.	n.a.	n.a.	n.a.	n.a.	n.a.

Chromeleon (c) Dionex
Version 7.2.10.25868

Chromatogram and SST Results

Injection Details

Injection Name:	**1,2-Prop & 1- propane 10**	*Run Time (min):*	**20,00**
Vial Number:	**3:3**	*Injection Volume:*	**1,00**
Injection Type:	**Calibration Standard**	*Channel:*	**RI_CH_1**
Calibration Level:	**1**	*Wavelength:*	**n.a.**
Instrument Method:	**Default method LC2030C 45 gr 20 min**	*Bandwidth:*	**n.a.**
Processing Method:	**Processing Method LC2030 45 gr**	*Dilution Factor:*	**1,0000**
Injection Date/Time:	**16/Jan/23 16:54**	*Sample Weight:*	**1,0000**

Chromatogram

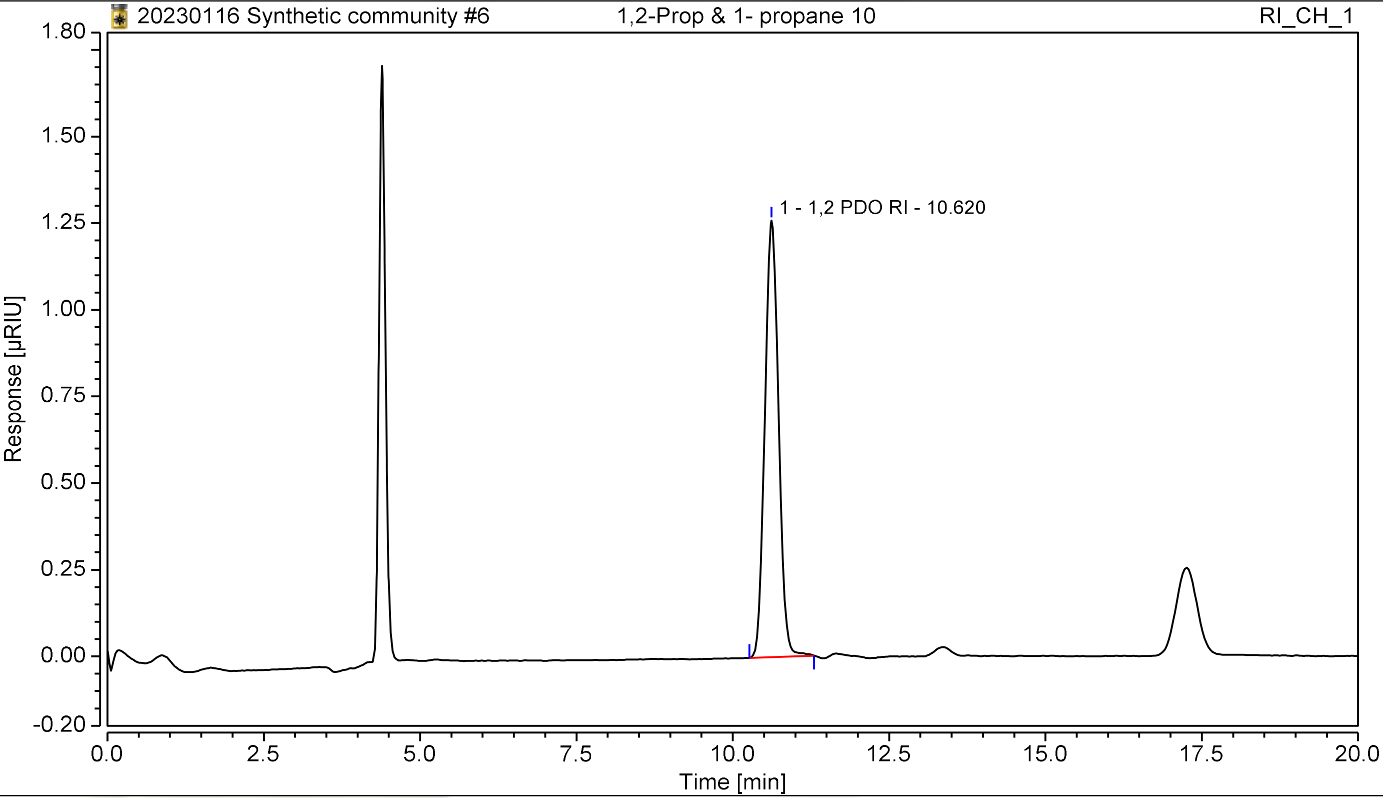

SST Results

No.	Name	Inj.Condition	Peak	Test Result	Injection

Number of executed test cases: **n.a.** **Total Result:** **Passed**

Chromatogram and Results

Injection Details

Injection Name:	**1,2-Prop & 1- propane 20**	*Run Time (min):*	**20,00**
Vial Number:	**3:3**	*Injection Volume:*	**2,00**
Injection Type:	**Calibration Standard**	*Channel:*	**RI_CH_1**
Calibration Level:	**1**	*Wavelength:*	**n.a.**
Instrument Method:	**Default method LC2030C 45 gr 20 min**	*Bandwidth:*	**n.a.**
Processing Method:	**Processing Method LC2030 45 gr**	*Dilution Factor:*	**1,0000**
Injection Date/Time:	**16/Jan/23 17:14**	*Sample Weight:*	**1,0000**

Chromatogram

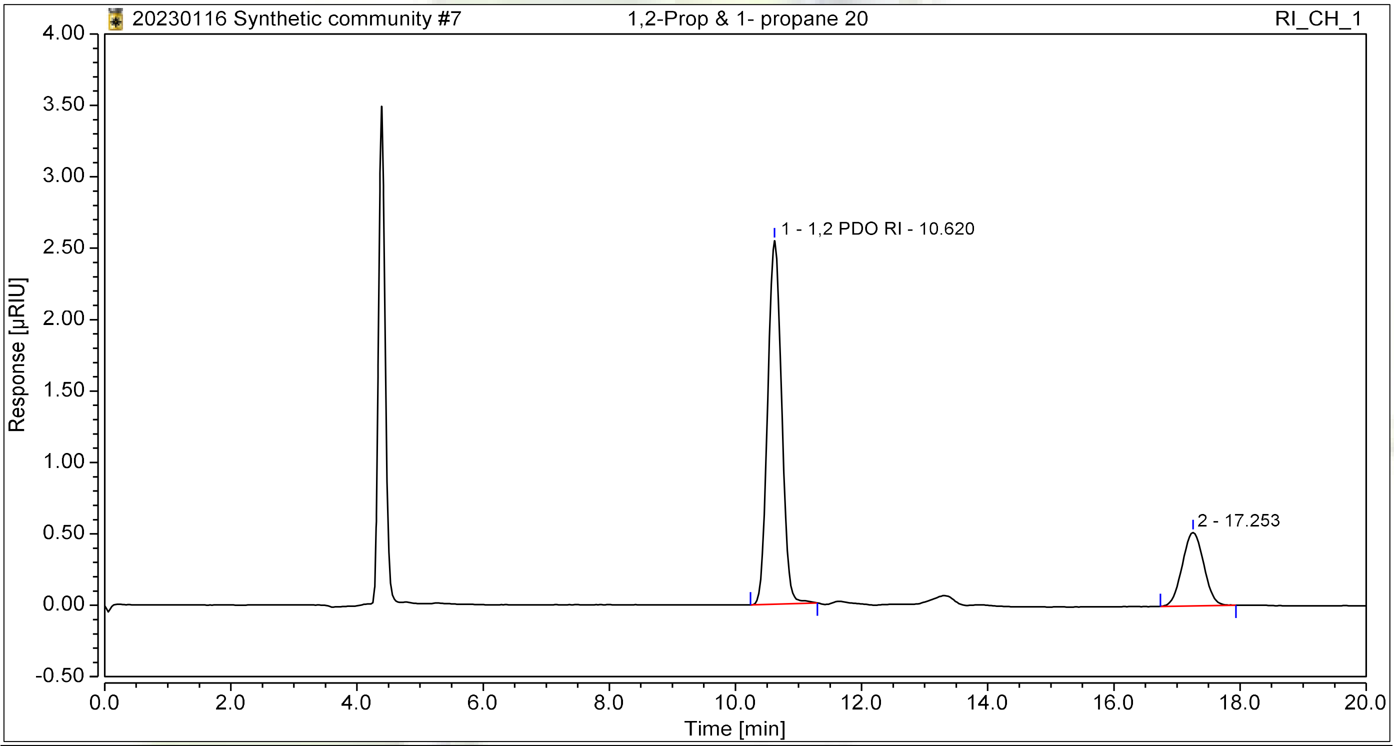

Integration Results

No.	Peak Name	Retention Time min	Area µRIU*min	Height µRIU	Relative Area %	Relative Height %	Amount
n.a.	GlcNAc	n.a.	n.a.	n.a.	n.a.	n.a.	n.a.
n.a.	Citrate	n.a.	n.a.	n.a.	n.a.	n.a.	n.a.
n.a.	Glucose	n.a.	n.a.	n.a.	n.a.	n.a.	n.a.
n.a.	Galactose	n.a.	n.a.	n.a.	n.a.	n.a.	n.a.
n.a.	Fucose	n.a.	n.a.	n.a.	n.a.	n.a.	n.a.
n.a.	Succinate RI	n.a.	n.a.	n.a.	n.a.	n.a.	n.a.
n.a.	Lactate RI	n.a.	n.a.	n.a.	n.a.	n.a.	n.a.
n.a.	glycerol	n.a.	n.a.	n.a.	n.a.	n.a.	n.a.
n.a.	Formate RI	n.a.	n.a.	n.a.	n.a.	n.a.	n.a.
n.a.	Acetate RI	n.a.	n.a.	n.a.	n.a.	n.a.	n.a.
1	1,2 PDO RI	10,620	0,651	2,545	76,26	83,20	19,8219
n.a.	1,3-PDO	n.a.	n.a.	n.a.	n.a.	n.a.	n.a.
n.a.	Propionate RI	n.a.	n.a.	n.a.	n.a.	n.a.	n.a.
n.a.	1,3-PDO	n.a.	n.a.	n.a.	n.a.	n.a.	n.a.
n.a.	2-3 BDO	n.a.	n.a.	n.a.	n.a.	n.a.	n.a.
n.a.	Ethanol	n.a.	n.a.	n.a.	n.a.	n.a.	n.a.
n.a.	Isobutyrate RI	n.a.	n.a.	n.a.	n.a.	n.a.	n.a.
n.a.	Butyrate RI	n.a.	n.a.	n.a.	n.a.	n.a.	n.a.
2		17,253	0,203	0,514	23,74	16,80	n.a.
**Total:**			**0,854**	**3,059**	**100,00**	**100,00**	

Chromeleon (c) Dionex
Version 7.2.10.25868

Peak Analysis

Injection Details

Injection Name:	**1,2-Prop & 1- propane 20**	*Run Time (min):*	**20,00**
Vial Number:	**3:3**	*Injection Volume:*	**2,00**
Injection Type:	**Calibration Standard**	*Channel:*	**RI_CH_1**
Calibration Level:	**1**	*Wavelength:*	**n.a.**
Instrument Method:	**Default method LC2030C 45 gr 20 min**	*Bandwidth:*	**n.a.**
Processing Method:	**Processing Method LC2030 45 gr**	*Dilution Factor:*	**1,0000**
Injection Date/Time:	**16/Jan/23 17:14**	*Sample Weight:*	**1,0000**

Chromatogram

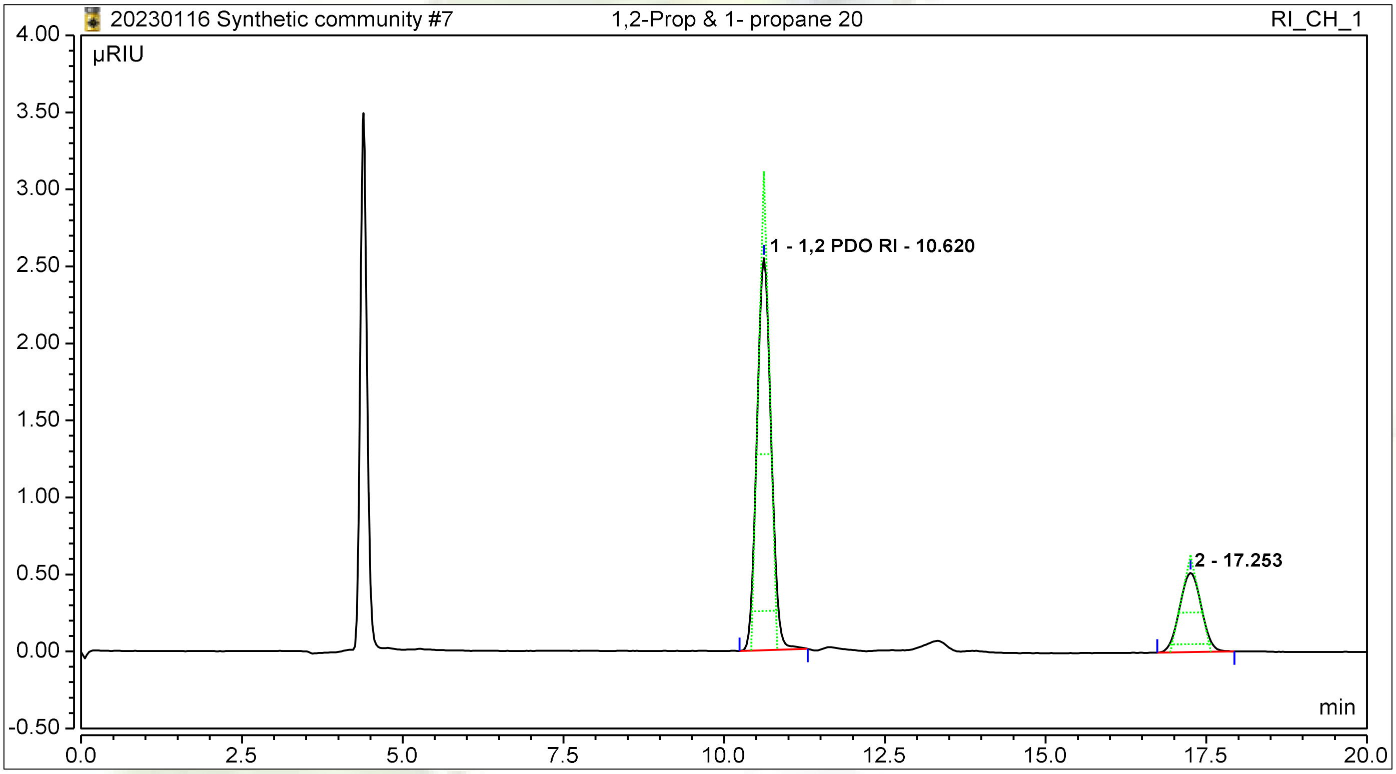

Peak Results

No.	Peak Name	Retention Time min	Width (50%) min	Type	Resolution (EP)	Asymmetry (EP)	Plates (EP)
n.a.	GlcNAc	n.a.	n.a.	n.a.	n.a.	n.a.	n.a.
n.a.	Citrate	n.a.	n.a.	n.a.	n.a.	n.a.	n.a.
n.a.	Glucose	n.a.	n.a.	n.a.	n.a.	n.a.	n.a.
n.a.	Galactose	n.a.	n.a.	n.a.	n.a.	n.a.	n.a.
n.a.	Fucose	n.a.	n.a.	n.a.	n.a.	n.a.	n.a.
n.a.	Succinate RI	n.a.	n.a.	n.a.	n.a.	n.a.	n.a.
n.a.	Lactate RI	n.a.	n.a.	n.a.	n.a.	n.a.	n.a.
n.a.	glycerol	n.a.	n.a.	n.a.	n.a.	n.a.	n.a.
n.a.	Formate RI	n.a.	n.a.	n.a.	n.a.	n.a.	n.a.
n.a.	Acetate RI	n.a.	n.a.	n.a.	n.a.	n.a.	n.a.
1	1,2 PDO RI	10,620	0,239	BMB	12,77	1,06	10898
n.a.	1,3-PDO	n.a.	n.a.	n.a.	n.a.	n.a.	n.a.
n.a.	Propionate RI	n.a.	n.a.	n.a.	n.a.	n.a.	n.a.
n.a.	1,3-PDO	n.a.	n.a.	n.a.	n.a.	n.a.	n.a.
n.a.	2-3 BDO	n.a.	n.a.	n.a.	n.a.	n.a.	n.a.
n.a.	Ethanol	n.a.	n.a.	n.a.	n.a.	n.a.	n.a.
n.a.	Isobutyrate RI	n.a.	n.a.	n.a.	n.a.	n.a.	n.a.
n.a.	Butyrate RI	n.a.	n.a.	n.a.	n.a.	n.a.	n.a.

Chromeleon (c) Dionex
Version 7.2.10.25868

2		17,253	0,373	BMB	n.a.	1,02	11837

Chromatogram and SST Results

Injection Details

Injection Name:	**1,2-Prop & 1- propane 20**	*Run Time (min):*	**20,00**
Vial Number:	**3:3**	*Injection Volume:*	**2,00**
Injection Type:	**Calibration Standard**	*Channel:*	**RI_CH_1**
Calibration Level:	**1**	*Wavelength:*	**n.a.**
Instrument Method:	**Default method LC2030C 45 gr 20 min**	*Bandwidth:*	**n.a.**
Processing Method:	**Processing Method LC2030 45 gr**	*Dilution Factor:*	**1,0000**
Injection Date/Time:	**16/Jan/23 17:14**	*Sample Weight:*	**1,0000**

Chromatogram

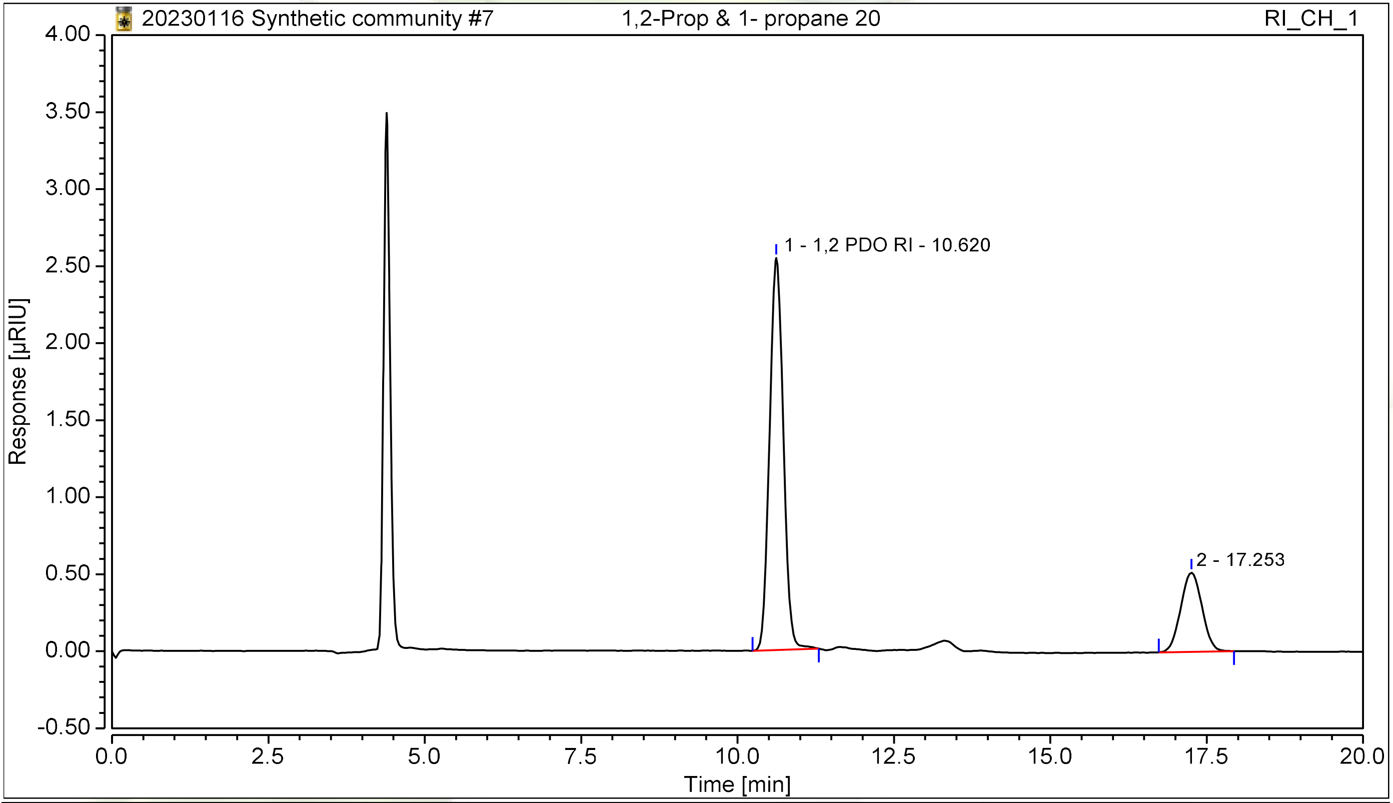

SST Results

No.	Name	Inj.Condition	Peak	Test Result	Injection

Number of executed test cases: **n.a.** **Total Result:** **Passed**

Chromatogram and Results

Injection Details

Injection Name:	**1,2-Prop & 1- propane 30**	*Run Time (min):*	**20,00**
Vial Number:	**3:3**	*Injection Volume:*	**3,00**
Injection Type:	**Calibration Standard**	*Channel:*	**RI_CH_1**
Calibration Level:	**1**	*Wavelength:*	**n.a.**
Instrument Method:	**Default method LC2030C 45 gr 20 min**	*Bandwidth:*	**n.a.**
Processing Method:	**Processing Method LC2030 45 gr**	*Dilution Factor:*	**1,0000**
Injection Date/Time:	**16/Jan/23 17:35**	*Sample Weight:*	**1,0000**

Chromatogram

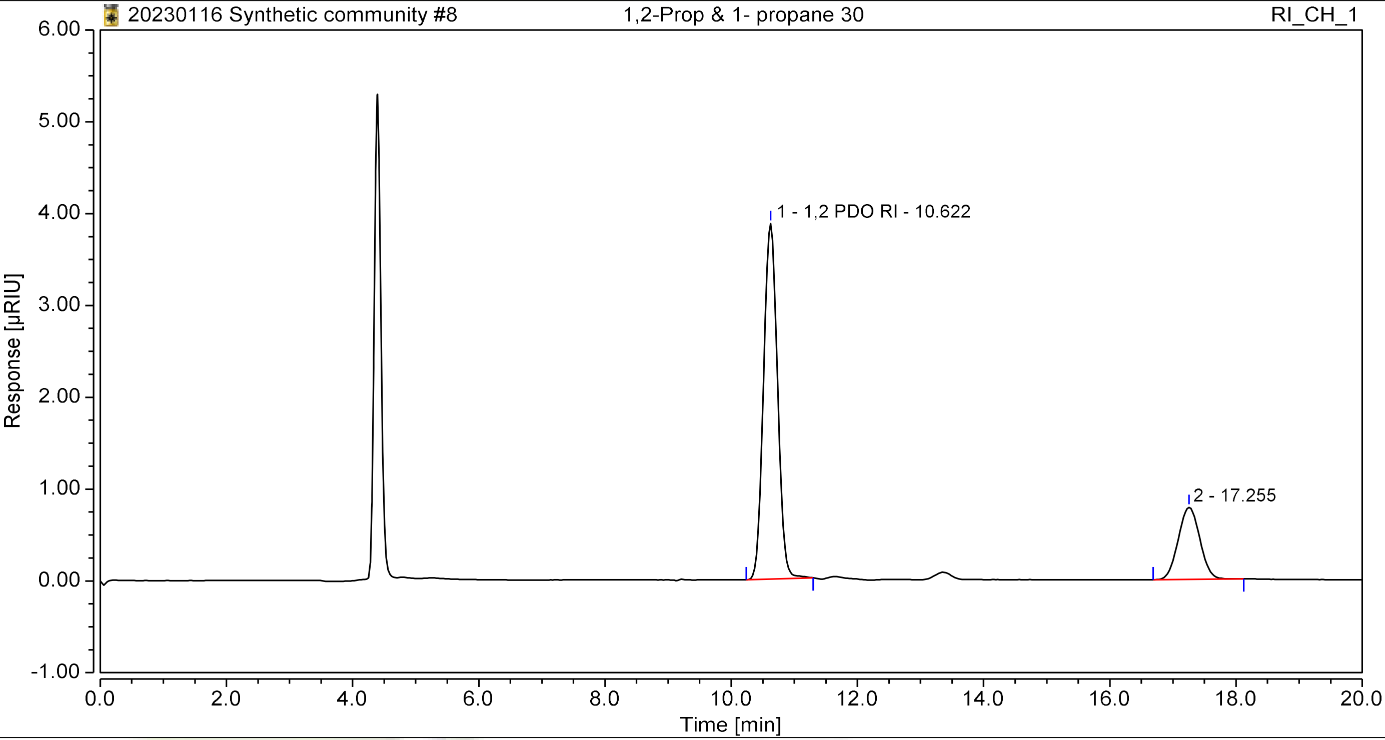

Integration Results

No.	Peak Name	Retention Time min	Area µRIU*min	Height µRIU	Relative Area %	Relative Height %	Amount
n.a.	GlcNAc	n.a.	n.a.	n.a.	n.a.	n.a.	n.a.
n.a.	Citrate	n.a.	n.a.	n.a.	n.a.	n.a.	n.a.
n.a.	Glucose	n.a.	n.a.	n.a.	n.a.	n.a.	n.a.
n.a.	Galactose	n.a.	n.a.	n.a.	n.a.	n.a.	n.a.
n.a.	Fucose	n.a.	n.a.	n.a.	n.a.	n.a.	n.a.
n.a.	Succinate RI	n.a.	n.a.	n.a.	n.a.	n.a.	n.a.
n.a.	Lactate RI	n.a.	n.a.	n.a.	n.a.	n.a.	n.a.
n.a.	glycerol	n.a.	n.a.	n.a.	n.a.	n.a.	n.a.
n.a.	Formate RI	n.a.	n.a.	n.a.	n.a.	n.a.	n.a.
n.a.	Acetate RI	n.a.	n.a.	n.a.	n.a.	n.a.	n.a.
1	1,2 PDO RI	10,622	0,992	3,872	76,15	83,18	30,1907
n.a.	1,3-PDO	n.a.	n.a.	n.a.	n.a.	n.a.	n.a.
n.a.	Propionate RI	n.a.	n.a.	n.a.	n.a.	n.a.	n.a.
n.a.	1,3-PDO	n.a.	n.a.	n.a.	n.a.	n.a.	n.a.
n.a.	2-3 BDO	n.a.	n.a.	n.a.	n.a.	n.a.	n.a.
n.a.	Ethanol	n.a.	n.a.	n.a.	n.a.	n.a.	n.a.
n.a.	Isobutyrate RI	n.a.	n.a.	n.a.	n.a.	n.a.	n.a.
n.a.	Butyrate RI	n.a.	n.a.	n.a.	n.a.	n.a.	n.a.
2		17,255	0,311	0,783	23,85	16,82	n.a.
**Total:**			**1,303**	**4,656**	**100,00**	**100,00**	

Chromeleon (c) Dionex
Version 7.2.10.25868

Peak Analysis

Injection Details

Injection Name:	**1,2-Prop & 1- propane 30**	*Run Time (min):*	**20,00**
Vial Number:	**3:3**	*Injection Volume:*	**3,00**
Injection Type:	**Calibration Standard**	*Channel:*	**RI_CH_1**
Calibration Level:	**1**	*Wavelength:*	**n.a.**
Instrument Method:	**Default method LC2030C 45 gr 20 min**	*Bandwidth:*	**n.a.**
Processing Method:	**Processing Method LC2030 45 gr**	*Dilution Factor:*	**1,0000**
Injection Date/Time:	**16/Jan/23 17:35**	*Sample Weight:*	**1,0000**

Chromatogram

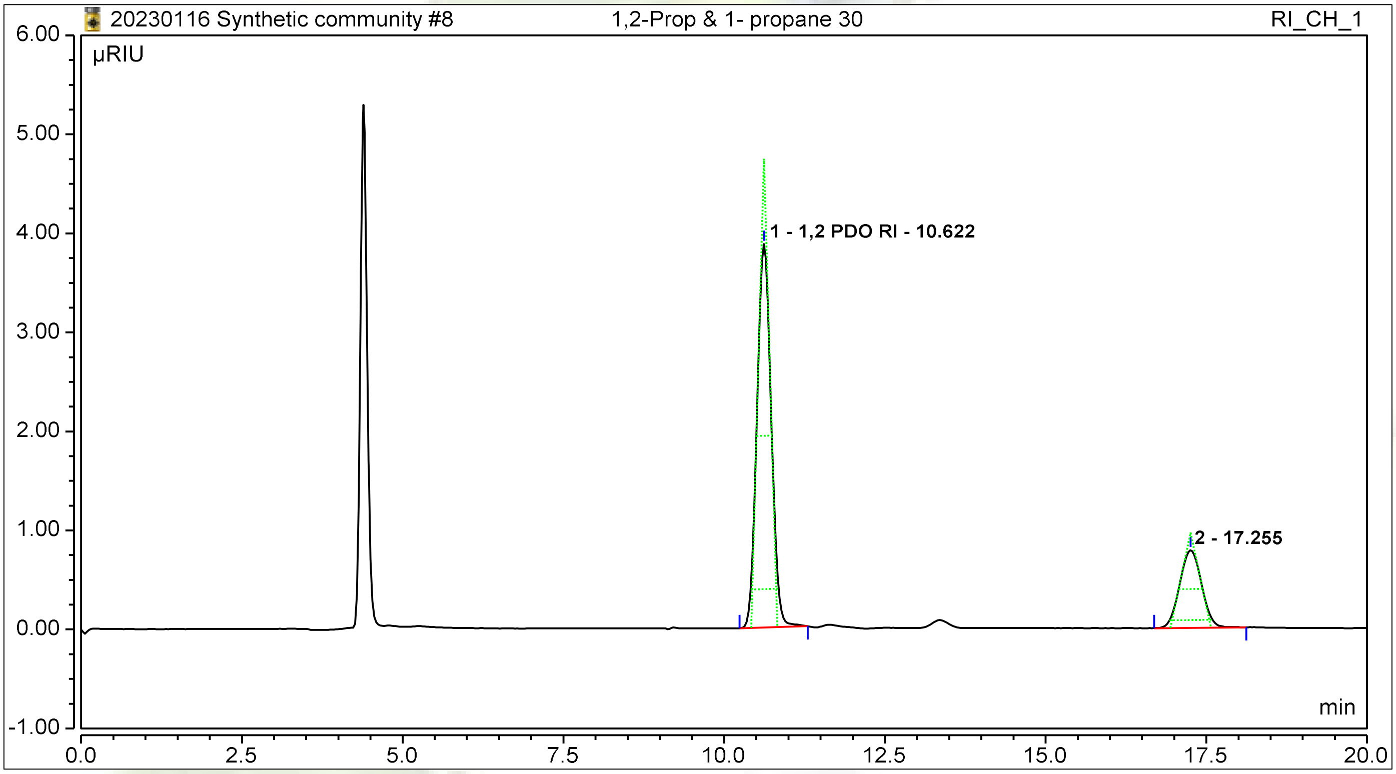

Peak Results

No.	Peak Name	Retention Time min	Width (50%) min	Type	Resolution (EP)	Asymmetry (EP)	Plates (EP)
n.a.	GlcNAc	n.a.	n.a.	n.a.	n.a.	n.a.	n.a.
n.a.	Citrate	n.a.	n.a.	n.a.	n.a.	n.a.	n.a.
n.a.	Glucose	n.a.	n.a.	n.a.	n.a.	n.a.	n.a.
n.a.	Galactose	n.a.	n.a.	n.a.	n.a.	n.a.	n.a.
n.a.	Fucose	n.a.	n.a.	n.a.	n.a.	n.a.	n.a.
n.a.	Succinate RI	n.a.	n.a.	n.a.	n.a.	n.a.	n.a.
n.a.	Lactate RI	n.a.	n.a.	n.a.	n.a.	n.a.	n.a.
n.a.	glycerol	n.a.	n.a.	n.a.	n.a.	n.a.	n.a.
n.a.	Formate RI	n.a.	n.a.	n.a.	n.a.	n.a.	n.a.
n.a.	Acetate RI	n.a.	n.a.	n.a.	n.a.	n.a.	n.a.
1	1,2 PDO RI	10,622	0,240	BMB	12,74	1,05	10853
n.a.	1,3-PDO	n.a.	n.a.	n.a.	n.a.	n.a.	n.a.
n.a.	Propionate RI	n.a.	n.a.	n.a.	n.a.	n.a.	n.a.
n.a.	1,3-PDO	n.a.	n.a.	n.a.	n.a.	n.a.	n.a.
n.a.	2-3 BDO	n.a.	n.a.	n.a.	n.a.	n.a.	n.a.
n.a.	Ethanol	n.a.	n.a.	n.a.	n.a.	n.a.	n.a.
n.a.	Isobutyrate RI	n.a.	n.a.	n.a.	n.a.	n.a.	n.a.
n.a.	Butyrate RI	n.a.	n.a.	n.a.	n.a.	n.a.	n.a.

2		17,255	0,374	BMB	n.a.	1,03	11775

Chromatogram and SST Results

Injection Details

Injection Name:	**1,2-Prop & 1- propane 30**	*Run Time (min):*	**20,00**
Vial Number:	**3:3**	*Injection Volume:*	**3,00**
Injection Type:	**Calibration Standard**	*Channel:*	**RI_CH_1**
Calibration Level:	**1**	*Wavelength:*	**n.a.**
Instrument Method:	**Default method LC2030C 45 gr 20 min**	*Bandwidth:*	**n.a.**
Processing Method:	**Processing Method LC2030 45 gr**	*Dilution Factor:*	**1,0000**
Injection Date/Time:	**16/Jan/23 17:35**	*Sample Weight:*	**1,0000**

Chromatogram

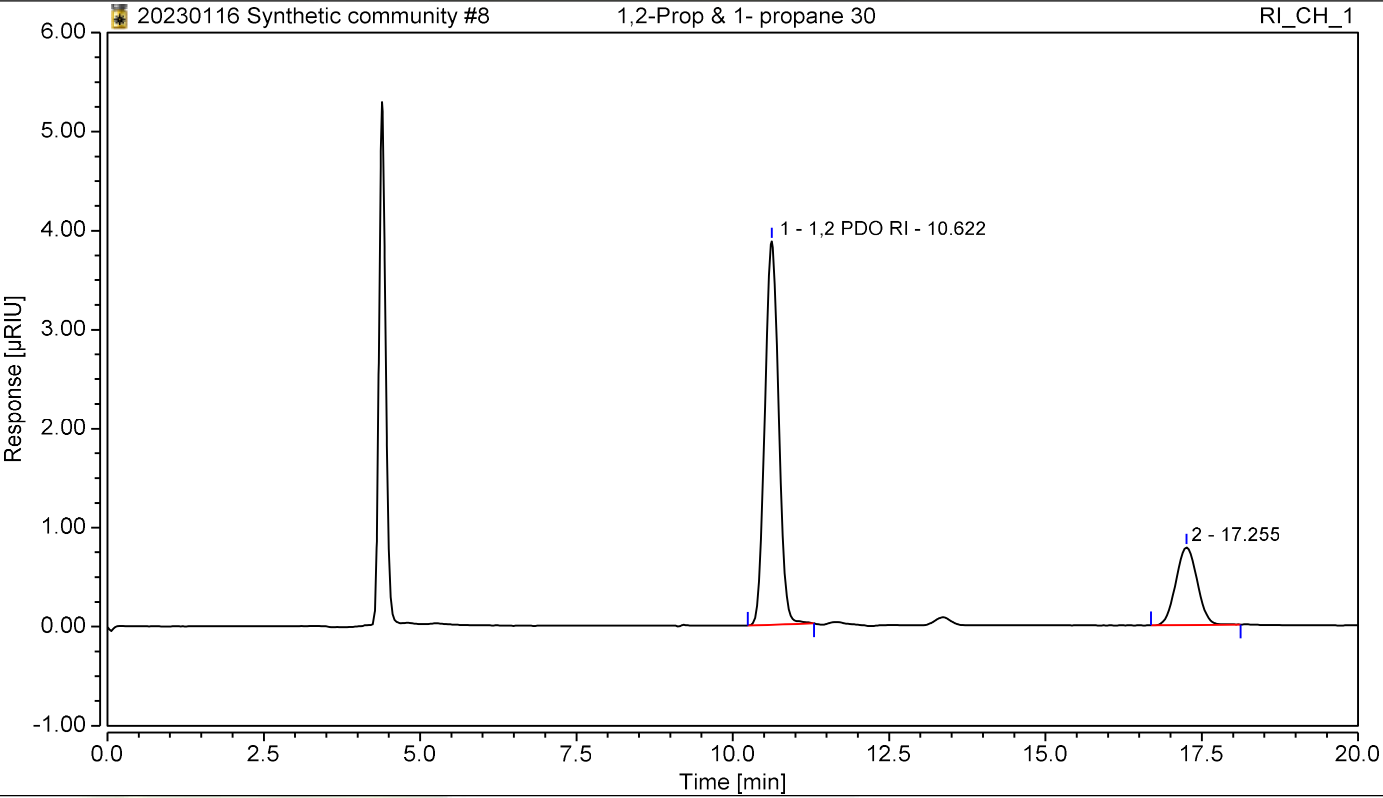

SST Results

No.	Name	Inj.Condition	Peak	Test Result	Injection
**Number of executed test cases:**	**n.a.**		**Total Result:**	**Passed**	

Chromatogram and Results

Injection Details

Injection Name:	**Meth & Eth 100**	*Run Time (min):*	**20,00**
Vial Number:	**3:4**	*Injection Volume:*	**1,00**
Injection Type:	**Calibration Standard**	*Channel:*	**RI_CH_1**
Calibration Level:	**3**	*Wavelength:*	**n.a.**
Instrument Method:	**Default method LC2030C 45 gr 20 min**	*Bandwidth:*	**n.a.**
Processing Method:	**Processing Method LC2030 45 gr**	*Dilution Factor:*	**1,0000**
Injection Date/Time:	**16/Jan/23 17:55**	*Sample Weight:*	**1,0000**

Chromatogram

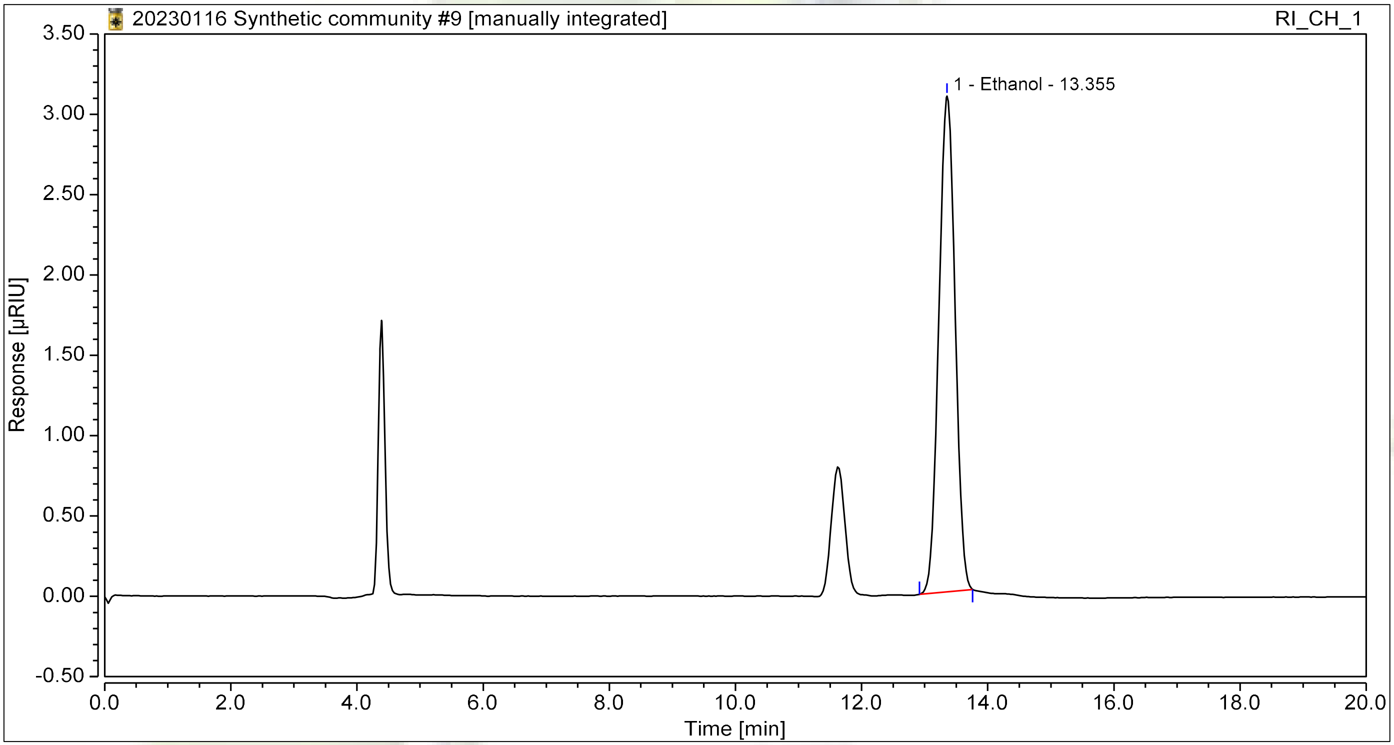

Integration Results

No.	Peak Name	Retention Time min	Area µRIU*min	Height µRIU	Relative Area %	Relative Height %	Amount
n.a.	GlcNAc	n.a.	n.a.	n.a.	n.a.	n.a.	n.a.
n.a.	Citrate	n.a.	n.a.	n.a.	n.a.	n.a.	n.a.
n.a.	Glucose	n.a.	n.a.	n.a.	n.a.	n.a.	n.a.
n.a.	Galactose	n.a.	n.a.	n.a.	n.a.	n.a.	n.a.
n.a.	Fucose	n.a.	n.a.	n.a.	n.a.	n.a.	n.a.
n.a.	Succinate RI	n.a.	n.a.	n.a.	n.a.	n.a.	n.a.
n.a.	Lactate RI	n.a.	n.a.	n.a.	n.a.	n.a.	n.a.
n.a.	glycerol	n.a.	n.a.	n.a.	n.a.	n.a.	n.a.
n.a.	Formate RI	n.a.	n.a.	n.a.	n.a.	n.a.	n.a.
n.a.	Acetate RI	n.a.	n.a.	n.a.	n.a.	n.a.	n.a.
n.a.	1,2 PDO RI	n.a.	n.a.	n.a.	n.a.	n.a.	n.a.
n.a.	1,3-PDO	n.a.	n.a.	n.a.	n.a.	n.a.	n.a.
n.a.	Propionate RI	n.a.	n.a.	n.a.	n.a.	n.a.	n.a.
n.a.	1,3-PDO	n.a.	n.a.	n.a.	n.a.	n.a.	n.a.
n.a.	2-3 BDO	n.a.	n.a.	n.a.	n.a.	n.a.	n.a.
1	Ethanol	13,355	0,921	3,086	100,00	100,00	96,6305
n.a.	Isobutyrate RI	n.a.	n.a.	n.a.	n.a.	n.a.	n.a.
n.a.	Butyrate RI	n.a.	n.a.	n.a.	n.a.	n.a.	n.a.
**Total:**			**0,921**	**3,086**	**100,00**	**100,00**	

Peak Analysis

Injection Details

Injection Name:	**Meth & Eth 100**	*Run Time (min):*	**20,00**
Vial Number:	**3:4**	*Injection Volume:*	**1,00**
Injection Type:	**Calibration Standard**	*Channel:*	**RI_CH_1**
Calibration Level:	**3**	*Wavelength:*	**n.a.**
Instrument Method:	**Default method LC2030C 45 gr 20 min**	*Bandwidth:*	**n.a.**
Processing Method:	**Processing Method LC2030 45 gr**	*Dilution Factor:*	**1,0000**
Injection Date/Time:	**16/Jan/23 17:55**	*Sample Weight:*	**1,0000**

Chromatogram

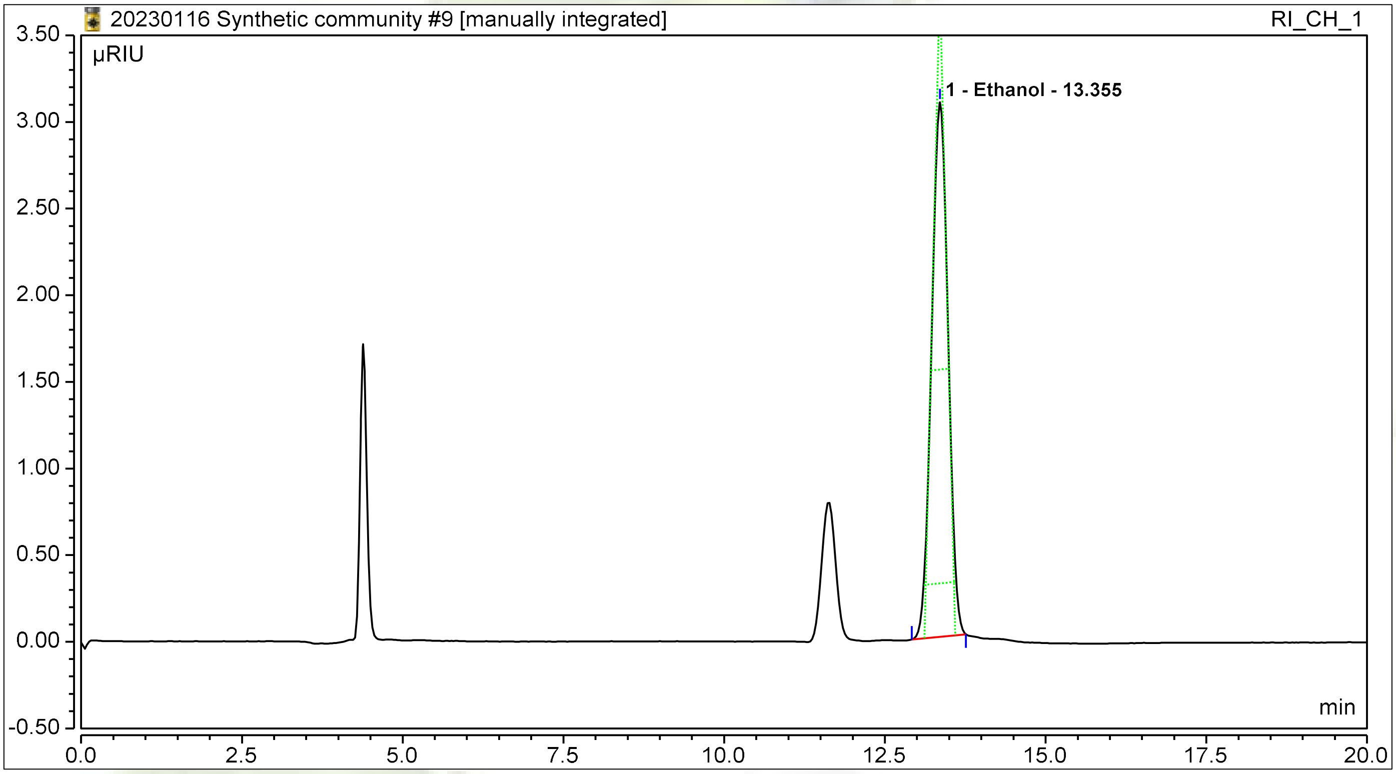

Peak Results

No.	Peak Name	Retention Time min	Width (50%) min	Type	Resolution (EP)	Asymmetry (EP)	Plates (EP)
n.a.	GlcNAc	n.a.	n.a.	n.a.	n.a.	n.a.	n.a.
n.a.	Citrate	n.a.	n.a.	n.a.	n.a.	n.a.	n.a.
n.a.	Glucose	n.a.	n.a.	n.a.	n.a.	n.a.	n.a.
n.a.	Galactose	n.a.	n.a.	n.a.	n.a.	n.a.	n.a.
n.a.	Fucose	n.a.	n.a.	n.a.	n.a.	n.a.	n.a.
n.a.	Succinate RI	n.a.	n.a.	n.a.	n.a.	n.a.	n.a.
n.a.	Lactate RI	n.a.	n.a.	n.a.	n.a.	n.a.	n.a.
n.a.	glycerol	n.a.	n.a.	n.a.	n.a.	n.a.	n.a.
n.a.	Formate RI	n.a.	n.a.	n.a.	n.a.	n.a.	n.a.
n.a.	Acetate RI	n.a.	n.a.	n.a.	n.a.	n.a.	n.a.
n.a.	1,2 PDO RI	n.a.	n.a.	n.a.	n.a.	n.a.	n.a.
n.a.	1,3-PDO	n.a.	n.a.	n.a.	n.a.	n.a.	n.a.
n.a.	Propionate RI	n.a.	n.a.	n.a.	n.a.	n.a.	n.a.
n.a.	1,3-PDO	n.a.	n.a.	n.a.	n.a.	n.a.	n.a.
n.a.	2-3 BDO	n.a.	n.a.	n.a.	n.a.	n.a.	n.a.
1	Ethanol	13,355	0,283	BMB*	n.a.	1,01	12314
n.a.	Isobutyrate RI	n.a.	n.a.	n.a.	n.a.	n.a.	n.a.
n.a.	Butyrate RI	n.a.	n.a.	n.a.	n.a.	n.a.	n.a.

Chromatogram and SST Results

Injection Details

Injection Name:	**Meth & Eth 100**	*Run Time (min):*	**20,00**
Vial Number:	**3:4**	*Injection Volume:*	**1,00**
Injection Type:	**Calibration Standard**	*Channel:*	**RI_CH_1**
Calibration Level:	**3**	*Wavelength:*	**n.a.**
Instrument Method:	**Default method LC2030C 45 gr 20 min**	*Bandwidth:*	**n.a.**
Processing Method:	**Processing Method LC2030 45 gr**	*Dilution Factor:*	**1,0000**
Injection Date/Time:	**16/Jan/23 17:55**	*Sample Weight:*	**1,0000**

Chromatogram

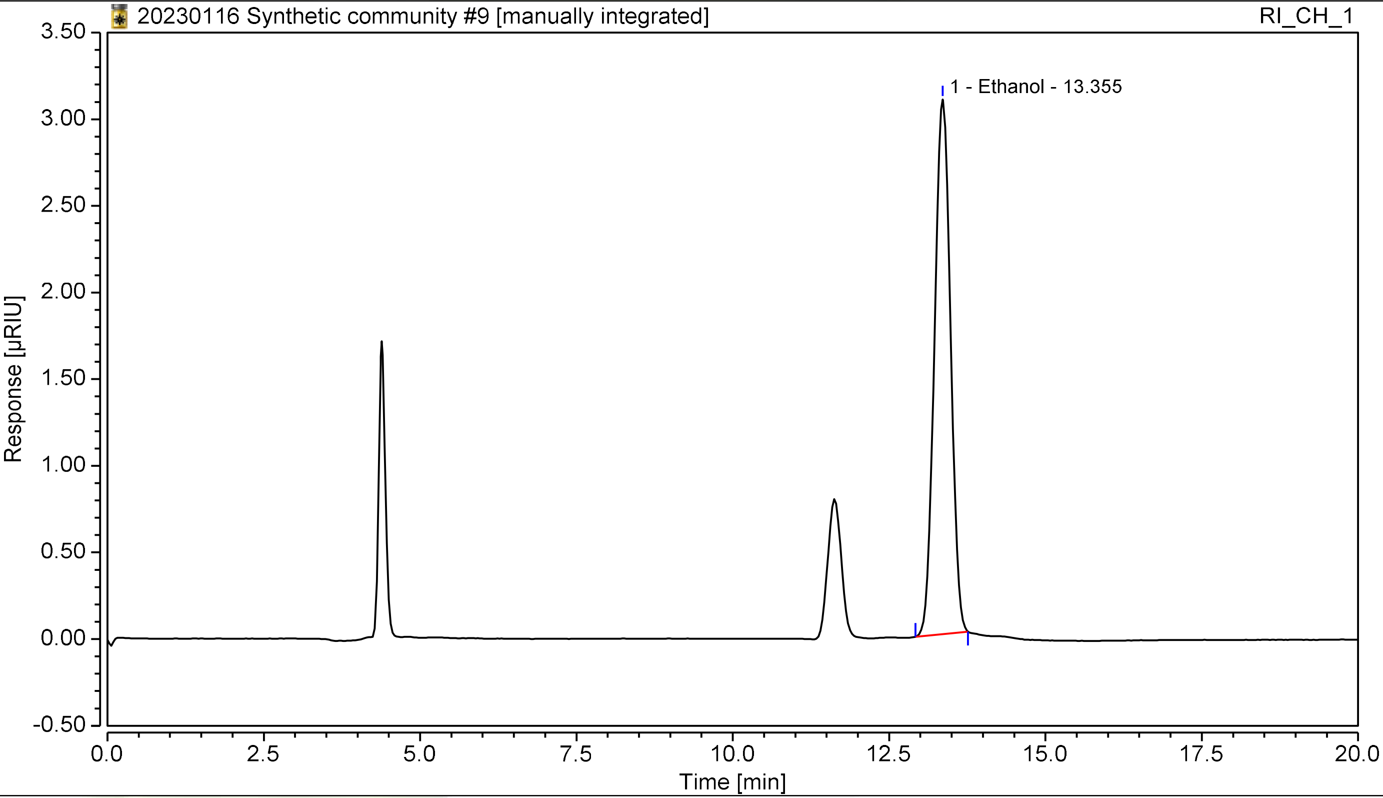

SST Results

No.	Name	Inj.Condition	Peak	Test Result	Injection
**Number of executed test cases:**	**n.a.**		**Total Result:**	**Passed**	

Chromatogram and Results

Injection Details

Injection Name:	**Meth & Eth 200**	*Run Time (min):*	**20,00**
Vial Number:	**3:4**	*Injection Volume:*	**2,00**
Injection Type:	**Calibration Standard**	*Channel:*	**RI_CH_1**
Calibration Level:	**3**	*Wavelength:*	**n.a.**
Instrument Method:	**Default method LC2030C 45 gr 20 min**	*Bandwidth:*	**n.a.**
Processing Method:	**Processing Method LC2030 45 gr**	*Dilution Factor:*	**1,0000**
Injection Date/Time:	**16/Jan/23 18:16**	*Sample Weight:*	**1,0000**

Chromatogram

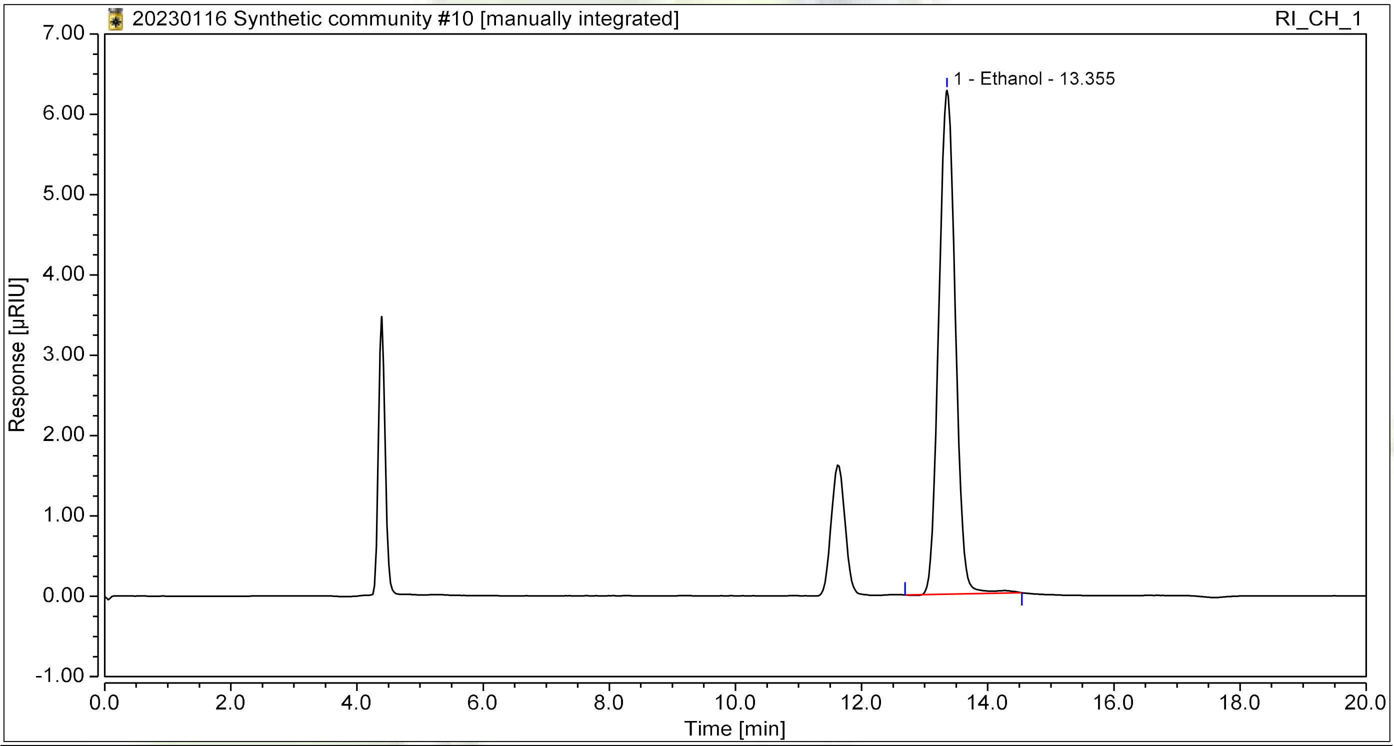

Integration Results

No.	Peak Name	Retention Time min	Area µRIU*min	Height µRIU	Relative Area %	Relative Height %	Amount
n.a.	GlcNAc	n.a.	n.a.	n.a.	n.a.	n.a.	n.a.
n.a.	Citrate	n.a.	n.a.	n.a.	n.a.	n.a.	n.a.
n.a.	Glucose	n.a.	n.a.	n.a.	n.a.	n.a.	n.a.
n.a.	Galactose	n.a.	n.a.	n.a.	n.a.	n.a.	n.a.
n.a.	Fucose	n.a.	n.a.	n.a.	n.a.	n.a.	n.a.
n.a.	Succinate RI	n.a.	n.a.	n.a.	n.a.	n.a.	n.a.
n.a.	Lactate RI	n.a.	n.a.	n.a.	n.a.	n.a.	n.a.
n.a.	glycerol	n.a.	n.a.	n.a.	n.a.	n.a.	n.a.
n.a.	Formate RI	n.a.	n.a.	n.a.	n.a.	n.a.	n.a.
n.a.	Acetate RI	n.a.	n.a.	n.a.	n.a.	n.a.	n.a.
n.a.	1,2 PDO RI	n.a.	n.a.	n.a.	n.a.	n.a.	n.a.
n.a.	1,3-PDO	n.a.	n.a.	n.a.	n.a.	n.a.	n.a.
n.a.	Propionate RI	n.a.	n.a.	n.a.	n.a.	n.a.	n.a.
n.a.	1,3-PDO	n.a.	n.a.	n.a.	n.a.	n.a.	n.a.
n.a.	2-3 BDO	n.a.	n.a.	n.a.	n.a.	n.a.	n.a.
1	Ethanol	13,355	1,909	6,269	100,00	100,00	200,2342
n.a.	Isobutyrate RI	n.a.	n.a.	n.a.	n.a.	n.a.	n.a.
n.a.	Butyrate RI	n.a.	n.a.	n.a.	n.a.	n.a.	n.a.
**Total:**			**1,909**	**6,269**	**100,00**	**100,00**	

Chromeleon (c) Dionex
Version 7.2.10.25868

Peak Analysis

Injection Details

Injection Name:	**Meth & Eth 200**	*Run Time (min):*	**20,00**
Vial Number:	**3:4**	*Injection Volume:*	**2,00**
Injection Type:	**Calibration Standard**	*Channel:*	**RI_CH_1**
Calibration Level:	**3**	*Wavelength:*	**n.a.**
Instrument Method:	**Default method LC2030C 45 gr 20 min**	*Bandwidth:*	**n.a.**
Processing Method:	**Processing Method LC2030 45 gr**	*Dilution Factor:*	**1,0000**
Injection Date/Time:	**16/Jan/23 18:16**	*Sample Weight:*	**1,0000**

Chromatogram

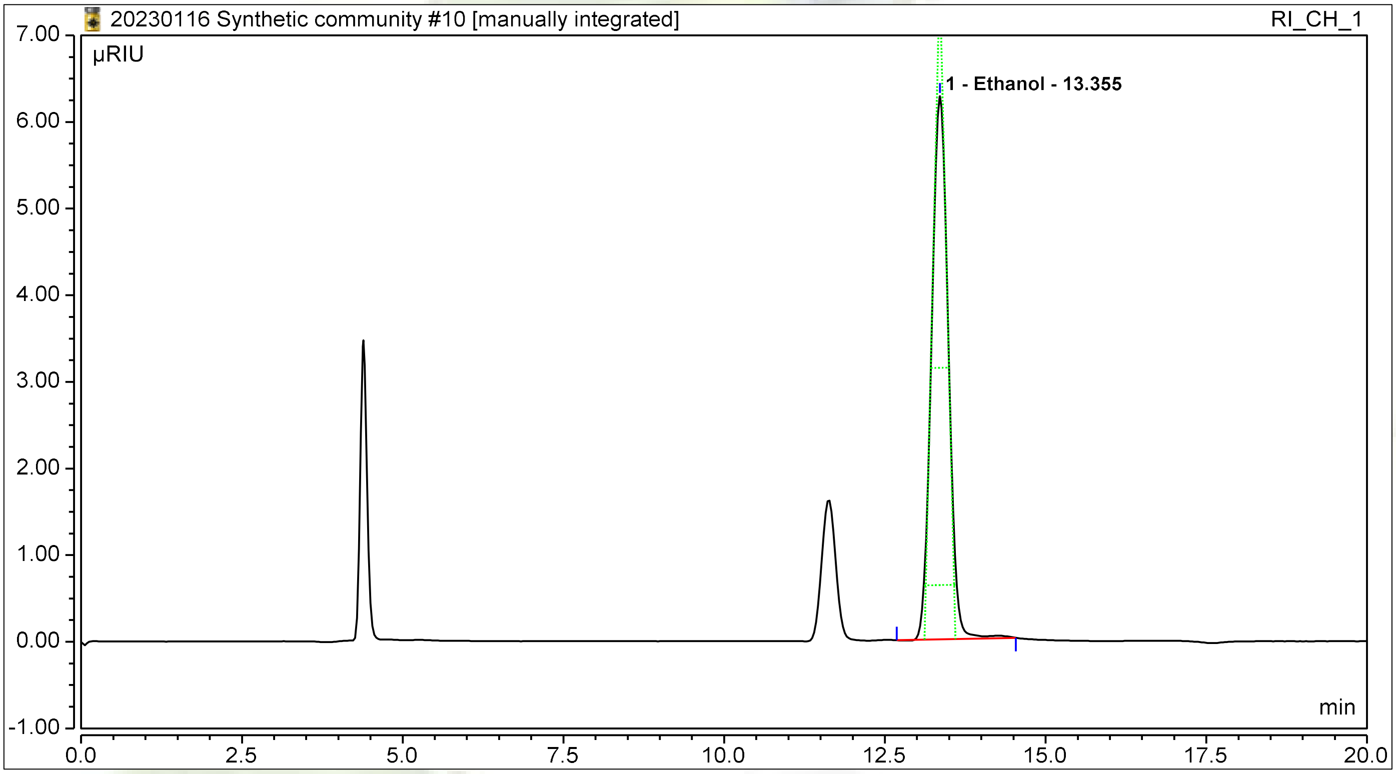

Peak Results

No.	Peak Name	Retention Time min	Width (50%) min	Type	Resolution (EP)	Asymmetry (EP)	Plates (EP)
n.a.	GlcNAc	n.a.	n.a.	n.a.	n.a.	n.a.	n.a.
n.a.	Citrate	n.a.	n.a.	n.a.	n.a.	n.a.	n.a.
n.a.	Glucose	n.a.	n.a.	n.a.	n.a.	n.a.	n.a.
n.a.	Galactose	n.a.	n.a.	n.a.	n.a.	n.a.	n.a.
n.a.	Fucose	n.a.	n.a.	n.a.	n.a.	n.a.	n.a.
n.a.	Succinate RI	n.a.	n.a.	n.a.	n.a.	n.a.	n.a.
n.a.	Lactate RI	n.a.	n.a.	n.a.	n.a.	n.a.	n.a.
n.a.	glycerol	n.a.	n.a.	n.a.	n.a.	n.a.	n.a.
n.a.	Formate RI	n.a.	n.a.	n.a.	n.a.	n.a.	n.a.
n.a.	Acetate RI	n.a.	n.a.	n.a.	n.a.	n.a.	n.a.
n.a.	1,2 PDO RI	n.a.	n.a.	n.a.	n.a.	n.a.	n.a.
n.a.	1,3-PDO	n.a.	n.a.	n.a.	n.a.	n.a.	n.a.
n.a.	Propionate RI	n.a.	n.a.	n.a.	n.a.	n.a.	n.a.
n.a.	1,3-PDO	n.a.	n.a.	n.a.	n.a.	n.a.	n.a.
n.a.	2-3 BDO	n.a.	n.a.	n.a.	n.a.	n.a.	n.a.
1	Ethanol	13,355	0,285	BMB*	n.a.	1,04	12201
n.a.	Isobutyrate RI	n.a.	n.a.	n.a.	n.a.	n.a.	n.a.
n.a.	Butyrate RI	n.a.	n.a.	n.a.	n.a.	n.a.	n.a.

Chromeleon (c) Dionex
Version 7.2.10.25868

Chromatogram and SST Results

Injection Details

Injection Name:	**Meth & Eth 200**	*Run Time (min):*	**20,00**
Vial Number:	**3:4**	*Injection Volume:*	**2,00**
Injection Type:	**Calibration Standard**	*Channel:*	**RI_CH_1**
Calibration Level:	**3**	*Wavelength:*	**n.a.**
Instrument Method:	**Default method LC2030C 45 gr 20 min**	*Bandwidth:*	**n.a.**
Processing Method:	**Processing Method LC2030 45 gr**	*Dilution Factor:*	**1,0000**
Injection Date/Time:	**16/Jan/23 18:16**	*Sample Weight:*	**1,0000**

Chromatogram

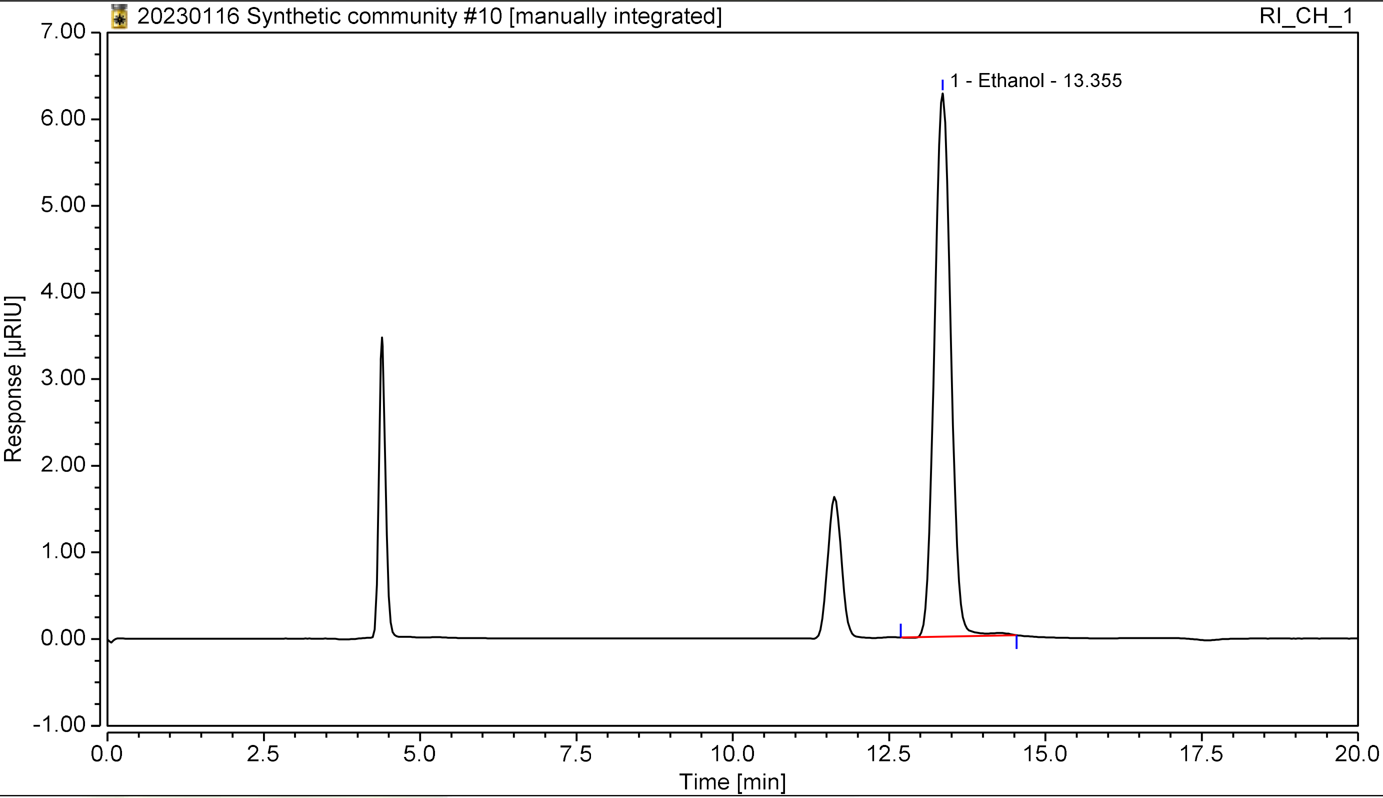

SST Results

No.	Name	Inj.Condition	Peak	Test Result	Injection

Number of executed test cases: **n.a.** **Total Result:** **Passed**

Chromatogram and Results

Injection Details

Injection Name:	**Meth & Eth 300**	*Run Time (min):*	**20,00**
Vial Number:	**3:4**	*Injection Volume:*	**3,00**
Injection Type:	**Calibration Standard**	*Channel:*	**RI_CH_1**
Calibration Level:	**3**	*Wavelength:*	**n.a.**
Instrument Method:	**Default method LC2030C 45 gr 20 min**	*Bandwidth:*	**n.a.**
Processing Method:	**Processing Method LC2030 45 gr**	*Dilution Factor:*	**1,0000**
Injection Date/Time:	**16/Jan/23 18:36**	*Sample Weight:*	**1,0000**

Chromatogram

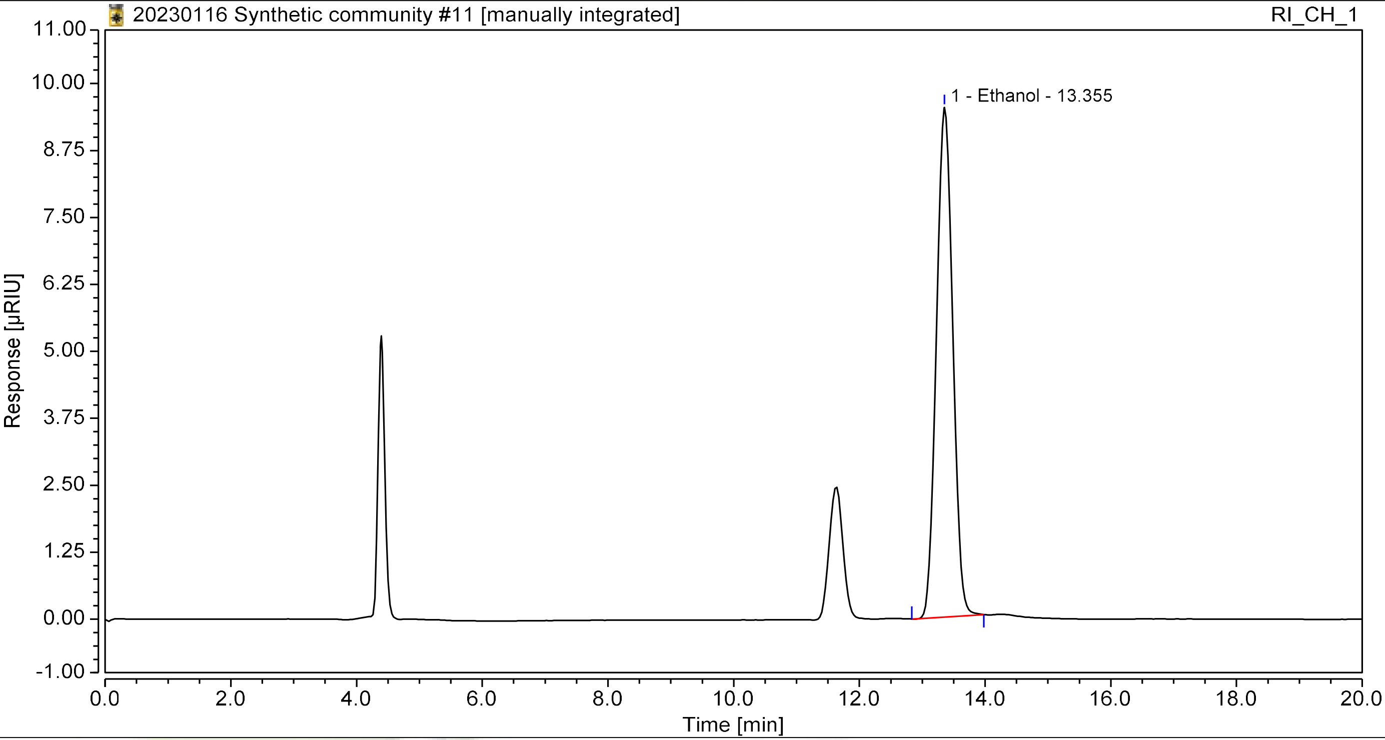

Integration Results

No.	Peak Name	Retention Time min	Area µRIU*min	Height µRIU	Relative Area %	Relative Height %	Amount
n.a.	GlcNAc	n.a.	n.a.	n.a.	n.a.	n.a.	n.a.
n.a.	Citrate	n.a.	n.a.	n.a.	n.a.	n.a.	n.a.
n.a.	Glucose	n.a.	n.a.	n.a.	n.a.	n.a.	n.a.
n.a.	Galactose	n.a.	n.a.	n.a.	n.a.	n.a.	n.a.
n.a.	Fucose	n.a.	n.a.	n.a.	n.a.	n.a.	n.a.
n.a.	Succinate RI	n.a.	n.a.	n.a.	n.a.	n.a.	n.a.
n.a.	Lactate RI	n.a.	n.a.	n.a.	n.a.	n.a.	n.a.
n.a.	glycerol	n.a.	n.a.	n.a.	n.a.	n.a.	n.a.
n.a.	Formate RI	n.a.	n.a.	n.a.	n.a.	n.a.	n.a.
n.a.	Acetate RI	n.a.	n.a.	n.a.	n.a.	n.a.	n.a.
n.a.	1,2 PDO RI	n.a.	n.a.	n.a.	n.a.	n.a.	n.a.
n.a.	1,3-PDO	n.a.	n.a.	n.a.	n.a.	n.a.	n.a.
n.a.	Propionate RI	n.a.	n.a.	n.a.	n.a.	n.a.	n.a.
n.a.	1,3-PDO	n.a.	n.a.	n.a.	n.a.	n.a.	n.a.
n.a.	2-3 BDO	n.a.	n.a.	n.a.	n.a.	n.a.	n.a.
1	Ethanol	13,355	2,869	9,516	100,00	100,00	300,9671
n.a.	Isobutyrate RI	n.a.	n.a.	n.a.	n.a.	n.a.	n.a.
n.a.	Butyrate RI	n.a.	n.a.	n.a.	n.a.	n.a.	n.a.
**Total:**			**2,869**	**9,516**	**100,00**	**100,00**	

Calibration

Calibration Details	Lactate RI		
Calibration Type	Lin	*Offset (C0)*	0,0000
Evaluation Type	Area	*Slope (C1)*	0,0342
Number of Calibration Points	3	*Curve (C2)*	0,0000
Number of disabled Calibration Points	0	*R-Square*	0,9989

Calibration Plot — Lactate RI

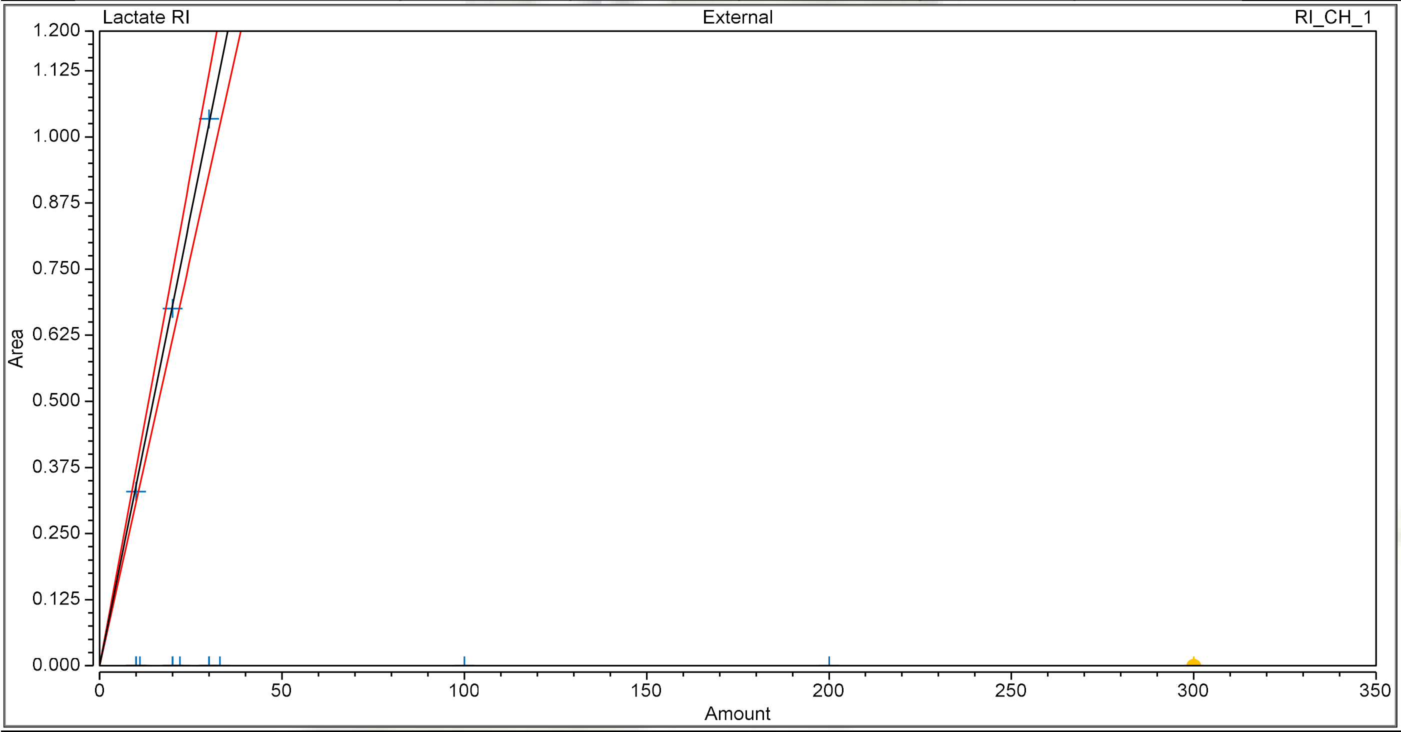

Calibration Results — Lactate RI

No.	Injection Name	Calibration Level	X Value RI_CH_1 Lactate RI	Y Value RI_CH_1 Lactate RI	Y Value RI_CH_1 Lactate RI	Area µRIU*min RI_CH_1 Lactate RI	Height µRIU RI_CH_1 Lactate RI
3	VFA 10	1	10,0000	0,3296	0,3296	0,330	1,574
4	VFA 20	1	20,0000	0,6757	0,6757	0,676	3,244
5	VFA 30	1	30,0000	1,0340	1,0340	1,034	4,963
6	1,2-Prop & 1- propane 10	1	10,0000	n.a.	n.a.	n.a.	n.a.
7	1,2-Prop & 1- propane 20	1	20,0000	n.a.	n.a.	n.a.	n.a.
8	1,2-Prop & 1- propane 30	1	30,0000	n.a.	n.a.	n.a.	n.a.
9	Meth & Eth 100	3	100,0000	n.a.	n.a.	n.a.	n.a.
10	Meth & Eth 200	3	200,0000	n.a.	n.a.	n.a.	n.a.
11	Meth & Eth 300	3	300,0000	n.a.	n.a.	n.a.	n.a.
73	Glucose, fructose,glycerol,2,3-bu	2	11,0000	n.a.	n.a.	n.a.	n.a.
74	Glucose, fructose,glycerol,2,3-bu	2	22,0000	n.a.	n.a.	n.a.	n.a.
75	Glucose, fructose,glycerol,2,3-bu	2	33,0000	n.a.	n.a.	n.a.	n.a.
76	citrate,succinate, maltate,fumarat	1	10,0000	n.a.	n.a.	n.a.	n.a.
77	citrate,succinate, maltate,fumarat	1	20,0000	n.a.	n.a.	n.a.	n.a.
78	citrate,succinate, maltate,fumarat	1	30,0000	n.a.	n.a.	n.a.	n.a.

Chromeleon (c) Dionex
Version 7.2.10.25868

Peak Analysis

Injection Details

Injection Name:	**Meth & Eth 300**	*Run Time (min):*	**20,00**
Vial Number:	**3:4**	*Injection Volume:*	**3,00**
Injection Type:	**Calibration Standard**	*Channel:*	**RI_CH_1**
Calibration Level:	**3**	*Wavelength:*	**n.a.**
Instrument Method:	**Default method LC2030C 45 gr 20 min**	*Bandwidth:*	**n.a.**
Processing Method:	**Processing Method LC2030 45 gr**	*Dilution Factor:*	**1,0000**
Injection Date/Time:	**16/Jan/23 18:36**	*Sample Weight:*	**1,0000**

Chromatogram

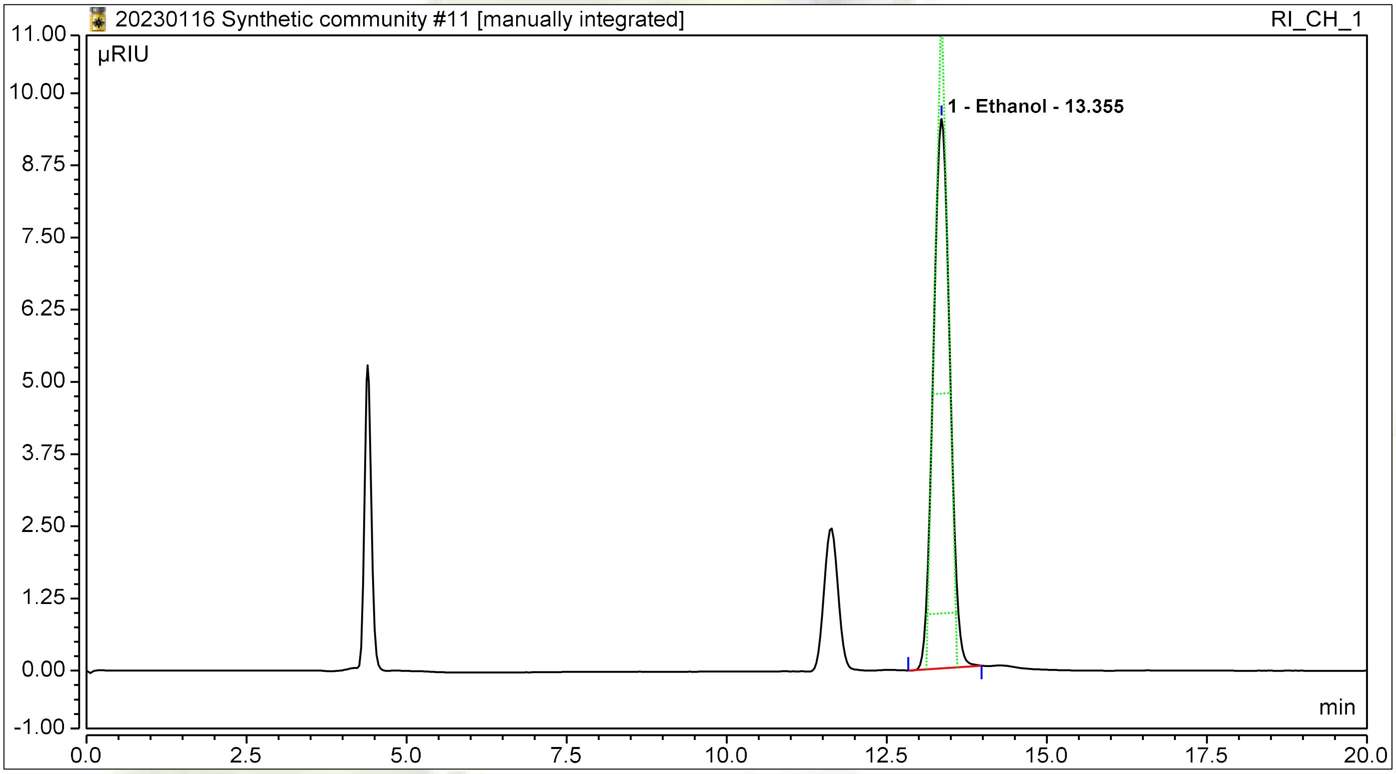

Peak Results

No.	Peak Name	Retention Time min	Width (50%) min	Type	Resolution (EP)	Asymmetry (EP)	Plates (EP)
n.a.	GlcNAc	n.a.	n.a.	n.a.	n.a.	n.a.	n.a.
n.a.	Citrate	n.a.	n.a.	n.a.	n.a.	n.a.	n.a.
n.a.	Glucose	n.a.	n.a.	n.a.	n.a.	n.a.	n.a.
n.a.	Galactose	n.a.	n.a.	n.a.	n.a.	n.a.	n.a.
n.a.	Fucose	n.a.	n.a.	n.a.	n.a.	n.a.	n.a.
n.a.	Succinate RI	n.a.	n.a.	n.a.	n.a.	n.a.	n.a.
n.a.	Lactate RI	n.a.	n.a.	n.a.	n.a.	n.a.	n.a.
n.a.	glycerol	n.a.	n.a.	n.a.	n.a.	n.a.	n.a.
n.a.	Formate RI	n.a.	n.a.	n.a.	n.a.	n.a.	n.a.
n.a.	Acetate RI	n.a.	n.a.	n.a.	n.a.	n.a.	n.a.
n.a.	1,2 PDO RI	n.a.	n.a.	n.a.	n.a.	n.a.	n.a.
n.a.	1,3-PDO	n.a.	n.a.	n.a.	n.a.	n.a.	n.a.
n.a.	Propionate RI	n.a.	n.a.	n.a.	n.a.	n.a.	n.a.
n.a.	1,3-PDO	n.a.	n.a.	n.a.	n.a.	n.a.	n.a.
n.a.	2-3 BDO	n.a.	n.a.	n.a.	n.a.	n.a.	n.a.
1	Ethanol	13,355	0,285	BMB*	n.a.	1,05	12172
n.a.	Isobutyrate RI	n.a.	n.a.	n.a.	n.a.	n.a.	n.a.
n.a.	Butyrate RI	n.a.	n.a.	n.a.	n.a.	n.a.	n.a.

Chromeleon (c) Dionex
Version 7.2.10.25868

Chromatogram and SST Results

Injection Details

Injection Name:	**Meth & Eth 300**	*Run Time (min):*	**20,00**
Vial Number:	**3:4**	*Injection Volume:*	**3,00**
Injection Type:	**Calibration Standard**	*Channel:*	**RI_CH_1**
Calibration Level:	**3**	*Wavelength:*	**n.a.**
Instrument Method:	**Default method LC2030C 45 gr 20 min**	*Bandwidth:*	**n.a.**
Processing Method:	**Processing Method LC2030 45 gr**	*Dilution Factor:*	**1,0000**
Injection Date/Time:	**16/Jan/23 18:36**	*Sample Weight:*	**1,0000**

Chromatogram

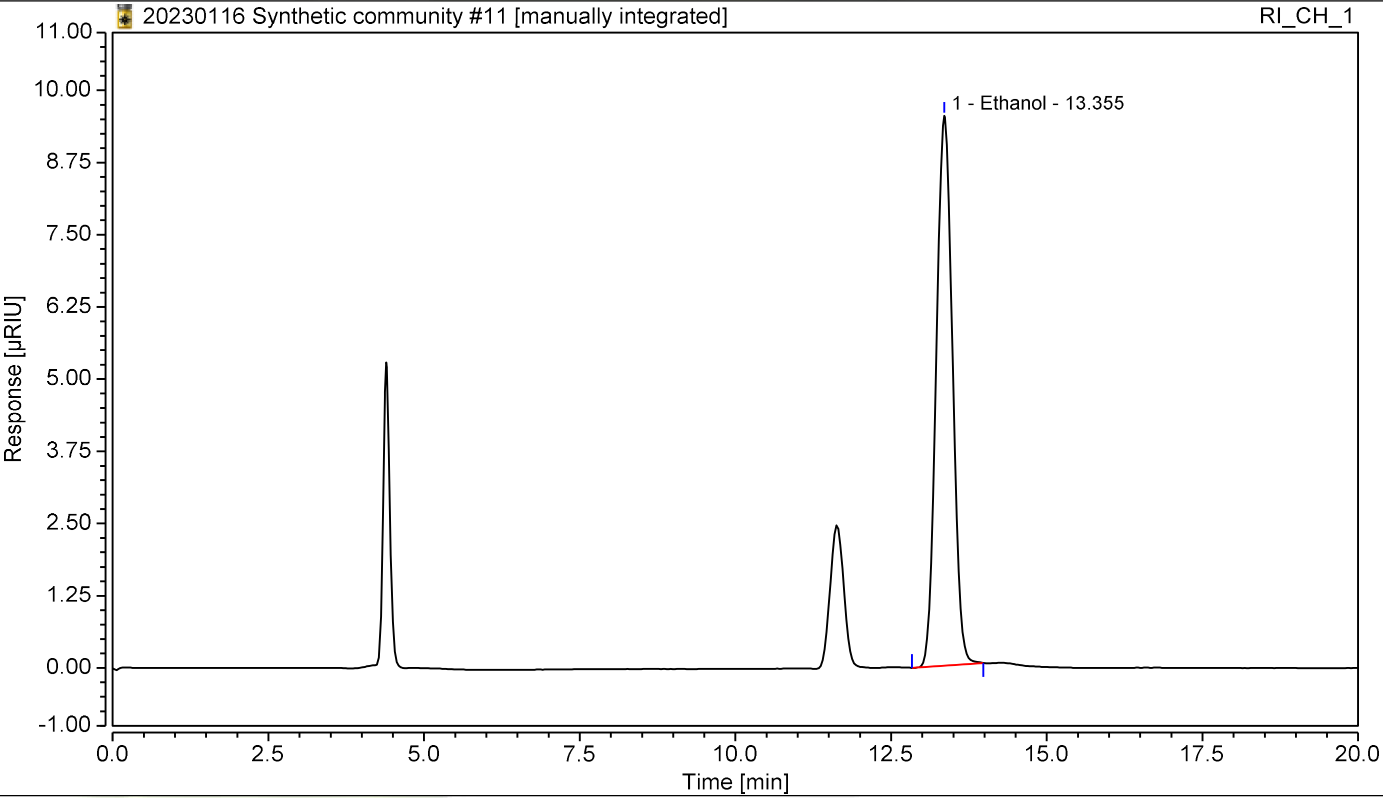

SST Results

No.	Name	Inj.Condition	Peak	Test Result	Injection
**Number of executed test cases:**	**n.a.**		**Total Result:**	**Passed**	

Chromatogram and Results

Injection Details

Injection Name:	**16.93 acetate**	*Run Time (min):*	**20,00**
Vial Number:	**3:5**	*Injection Volume:*	**10,00**
Injection Type:	**Unknown**	*Channel:*	**RI_CH_1**
Calibration Level:		*Wavelength:*	**n.a.**
Instrument Method:	**Default method LC2030C 45 gr 20 min**	*Bandwidth:*	**n.a.**
Processing Method:	**Processing Method LC2030 45 gr**	*Dilution Factor:*	**1,0000**
Injection Date/Time:	**16/Jan/23 18:57**	*Sample Weight:*	**1,0000**

Chromatogram

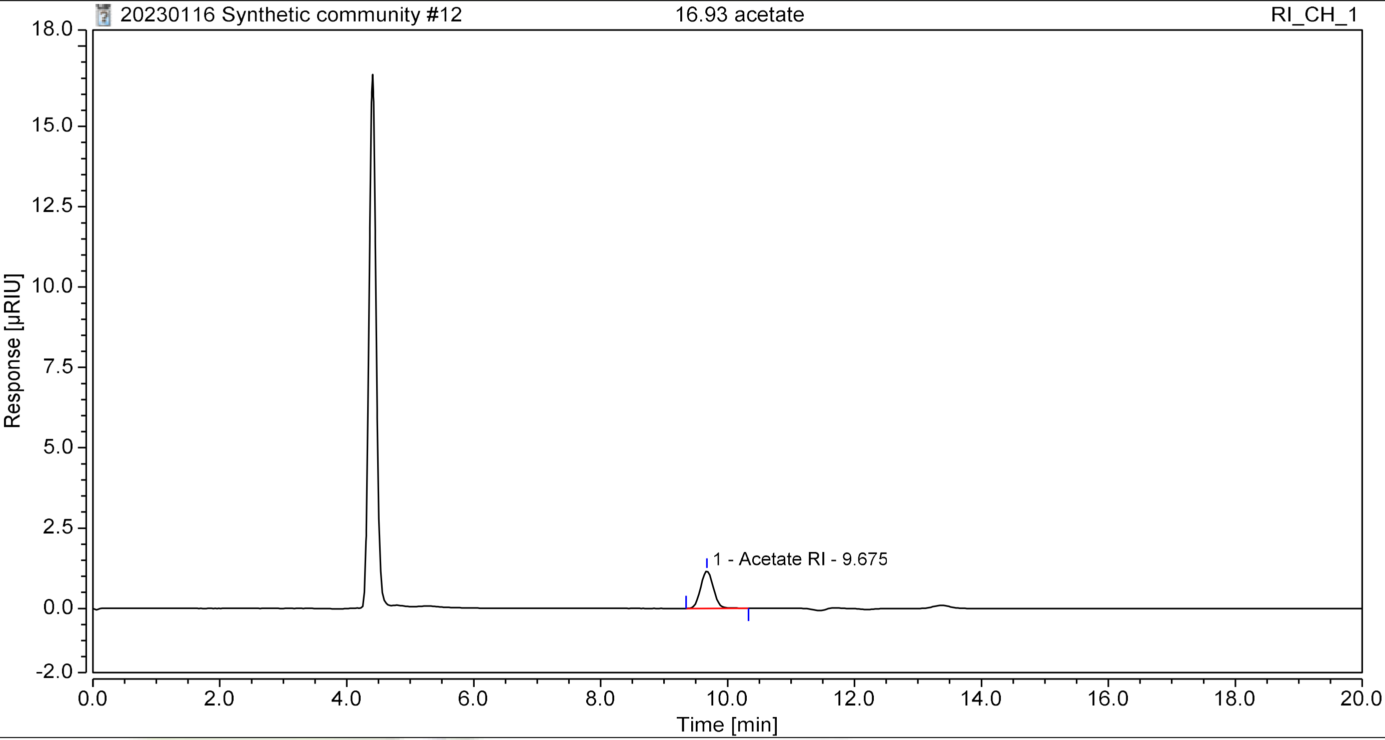

Integration Results

No.	Peak Name	Retention Time min	Area µRIU*min	Height µRIU	Relative Area %	Relative Height %	Amount
n.a.	GlcNAc	n.a.	n.a.	n.a.	n.a.	n.a.	n.a.
n.a.	Citrate	n.a.	n.a.	n.a.	n.a.	n.a.	n.a.
n.a.	Glucose	n.a.	n.a.	n.a.	n.a.	n.a.	n.a.
n.a.	Galactose	n.a.	n.a.	n.a.	n.a.	n.a.	n.a.
n.a.	Fucose	n.a.	n.a.	n.a.	n.a.	n.a.	n.a.
n.a.	Succinate RI	n.a.	n.a.	n.a.	n.a.	n.a.	n.a.
n.a.	Lactate RI	n.a.	n.a.	n.a.	n.a.	n.a.	n.a.
n.a.	glycerol	n.a.	n.a.	n.a.	n.a.	n.a.	n.a.
n.a.	Formate RI	n.a.	n.a.	n.a.	n.a.	n.a.	n.a.
1	Acetate RI	9,675	0,269	1,158	100,00	100,00	16,7074
n.a.	1,2 PDO RI	n.a.	n.a.	n.a.	n.a.	n.a.	n.a.
n.a.	1,3-PDO	n.a.	n.a.	n.a.	n.a.	n.a.	n.a.
n.a.	Propionate RI	n.a.	n.a.	n.a.	n.a.	n.a.	n.a.
n.a.	1,3-PDO	n.a.	n.a.	n.a.	n.a.	n.a.	n.a.
n.a.	2-3 BDO	n.a.	n.a.	n.a.	n.a.	n.a.	n.a.
n.a.	Ethanol	n.a.	n.a.	n.a.	n.a.	n.a.	n.a.
n.a.	Isobutyrate RI	n.a.	n.a.	n.a.	n.a.	n.a.	n.a.
n.a.	Butyrate RI	n.a.	n.a.	n.a.	n.a.	n.a.	n.a.
**Total:**			**0,269**	**1,158**	**100,00**	**100,00**	

Peak Analysis

Injection Details

Injection Name:	**16.93 acetate**	*Run Time (min):*	**20,00**
Vial Number:	**3:5**	*Injection Volume:*	**10,00**
Injection Type:	**Unknown**	*Channel:*	**RI_CH_1**
Calibration Level:		*Wavelength:*	**n.a.**
Instrument Method:	**Default method LC2030C 45 gr 20 min**	*Bandwidth:*	**n.a.**
Processing Method:	**Processing Method LC2030 45 gr**	*Dilution Factor:*	**1,0000**
Injection Date/Time:	**16/Jan/23 18:57**	*Sample Weight:*	**1,0000**

Chromatogram

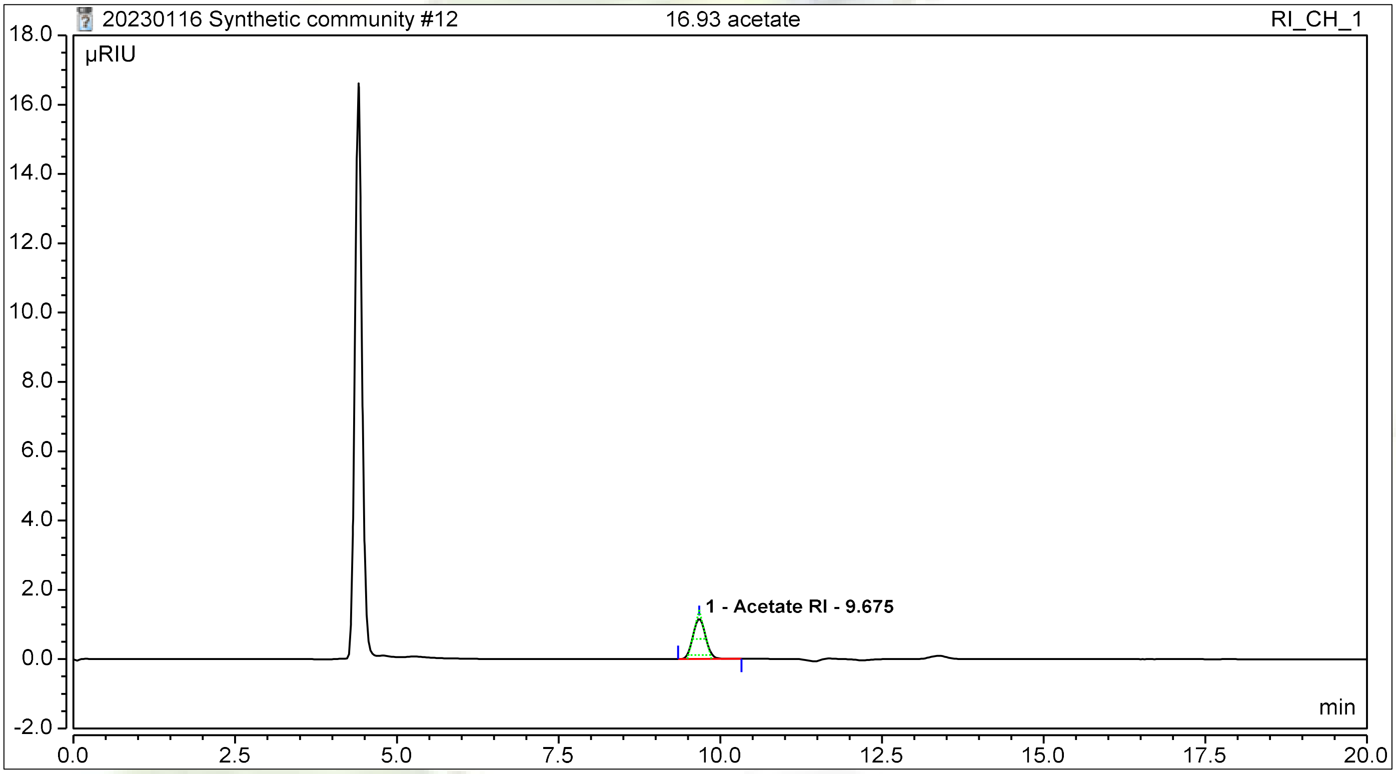

Peak Results

No.	Peak Name	Retention Time min	Width (50%) min	Type	Resolution (EP)	Asymmetry (EP)	Plates (EP)
n.a.	GlcNAc	n.a.	n.a.	n.a.	n.a.	n.a.	n.a.
n.a.	Citrate	n.a.	n.a.	n.a.	n.a.	n.a.	n.a.
n.a.	Glucose	n.a.	n.a.	n.a.	n.a.	n.a.	n.a.
n.a.	Galactose	n.a.	n.a.	n.a.	n.a.	n.a.	n.a.
n.a.	Fucose	n.a.	n.a.	n.a.	n.a.	n.a.	n.a.
n.a.	Succinate RI	n.a.	n.a.	n.a.	n.a.	n.a.	n.a.
n.a.	Lactate RI	n.a.	n.a.	n.a.	n.a.	n.a.	n.a.
n.a.	glycerol	n.a.	n.a.	n.a.	n.a.	n.a.	n.a.
n.a.	Formate RI	n.a.	n.a.	n.a.	n.a.	n.a.	n.a.
1	Acetate RI	9,675	0,218	BMB	n.a.	1,05	10892
n.a.	1,2 PDO RI	n.a.	n.a.	n.a.	n.a.	n.a.	n.a.
n.a.	1,3-PDO	n.a.	n.a.	n.a.	n.a.	n.a.	n.a.
n.a.	Propionate RI	n.a.	n.a.	n.a.	n.a.	n.a.	n.a.
n.a.	1,3-PDO	n.a.	n.a.	n.a.	n.a.	n.a.	n.a.
n.a.	2-3 BDO	n.a.	n.a.	n.a.	n.a.	n.a.	n.a.
n.a.	Ethanol	n.a.	n.a.	n.a.	n.a.	n.a.	n.a.
n.a.	Isobutyrate RI	n.a.	n.a.	n.a.	n.a.	n.a.	n.a.
n.a.	Butyrate RI	n.a.	n.a.	n.a.	n.a.	n.a.	n.a.

Chromatogram and SST Results

Injection Details

Injection Name:	**16.93 acetate**	*Run Time (min):*	**20,00**
Vial Number:	**3:5**	*Injection Volume:*	**10,00**
Injection Type:	**Unknown**	*Channel:*	**RI_CH_1**
Calibration Level:		*Wavelength:*	**n.a.**
Instrument Method:	**Default method LC2030C 45 gr 20 min**	*Bandwidth:*	**n.a.**
Processing Method:	**Processing Method LC2030 45 gr**	*Dilution Factor:*	**1,0000**
Injection Date/Time:	**16/Jan/23 18:57**	*Sample Weight:*	**1,0000**

Chromatogram

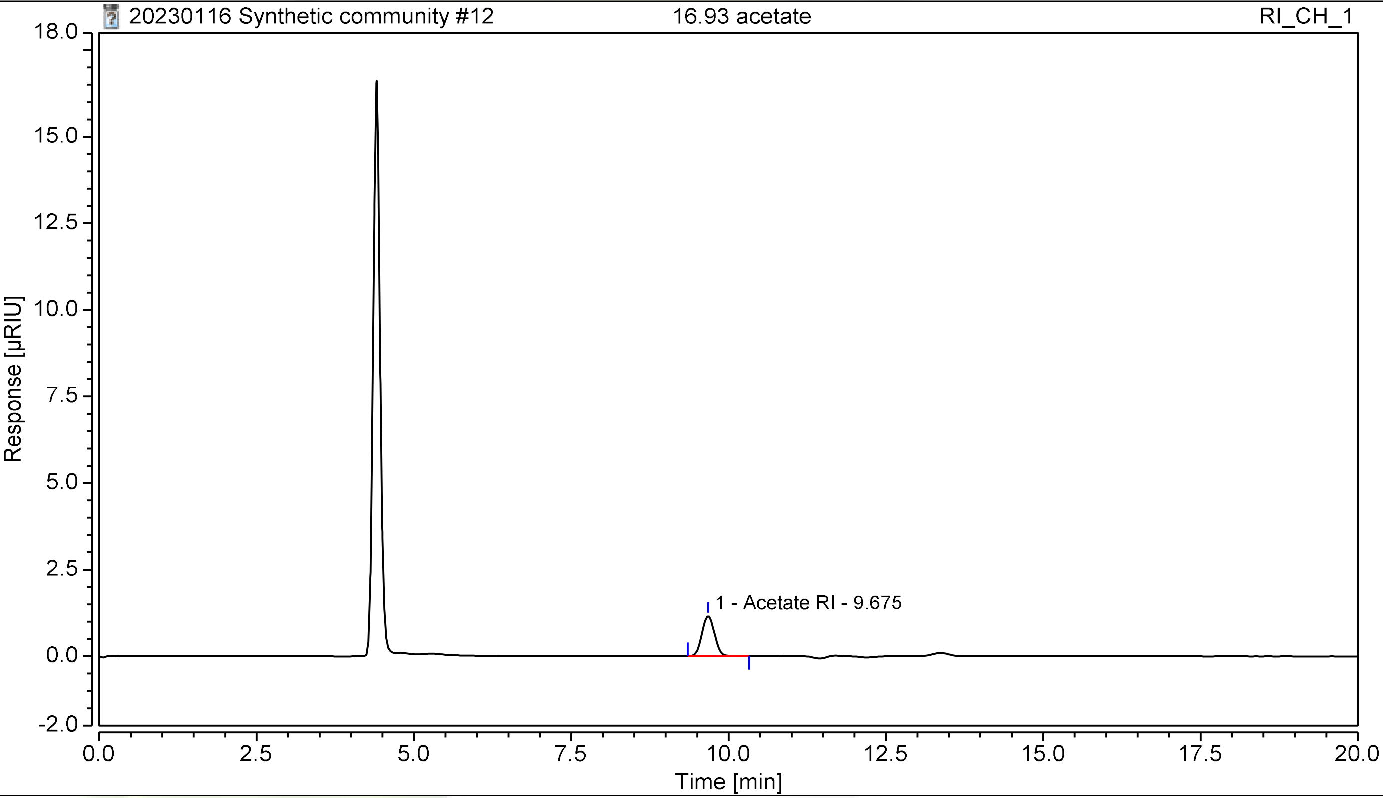

SST Results

No.	Name	Inj.Condition	Peak	Test Result	Injection

Number of executed test cases: **n.a.** **Total Result:** **Passed**

Chromatogram and Results

Injection Details

Injection Name:	**13.68 propionate**	*Run Time (min):*	**20,00**
Vial Number:	**3:6**	*Injection Volume:*	**10,00**
Injection Type:	**Unknown**	*Channel:*	**RI_CH_1**
Calibration Level:		*Wavelength:*	**n.a.**
Instrument Method:	**Default method LC2030C 45 gr 20 min**	*Bandwidth:*	**n.a.**
Processing Method:	**Processing Method LC2030 45 gr**	*Dilution Factor:*	**1,0000**
Injection Date/Time:	**16/Jan/23 19:17**	*Sample Weight:*	**1,0000**

Chromatogram

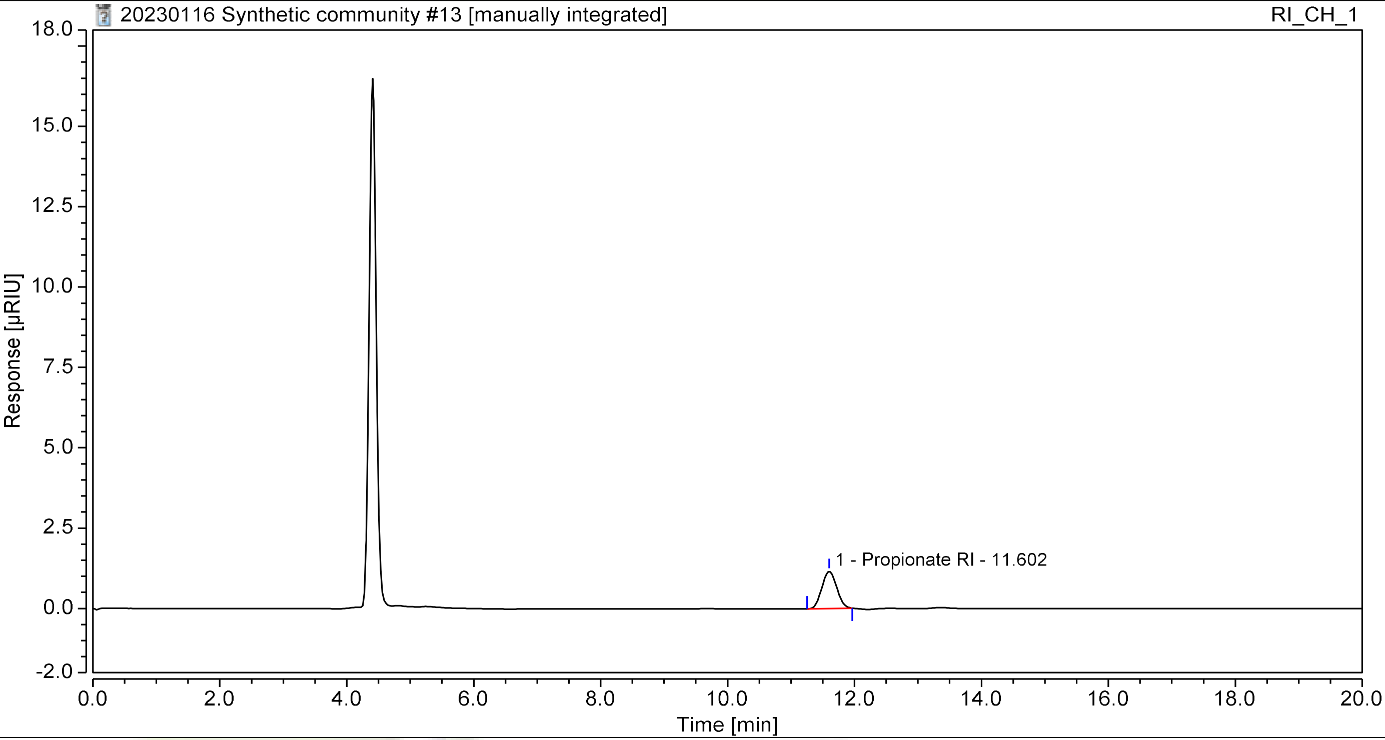

Integration Results

No.	Peak Name	Retention Time min	Area µRIU*min	Height µRIU	Relative Area %	Relative Height %	Amount
n.a.	GlcNAc	n.a.	n.a.	n.a.	n.a.	n.a.	n.a.
n.a.	Citrate	n.a.	n.a.	n.a.	n.a.	n.a.	n.a.
n.a.	Glucose	n.a.	n.a.	n.a.	n.a.	n.a.	n.a.
n.a.	Galactose	n.a.	n.a.	n.a.	n.a.	n.a.	n.a.
n.a.	Fucose	n.a.	n.a.	n.a.	n.a.	n.a.	n.a.
n.a.	Succinate RI	n.a.	n.a.	n.a.	n.a.	n.a.	n.a.
n.a.	Lactate RI	n.a.	n.a.	n.a.	n.a.	n.a.	n.a.
n.a.	glycerol	n.a.	n.a.	n.a.	n.a.	n.a.	n.a.
n.a.	Formate RI	n.a.	n.a.	n.a.	n.a.	n.a.	n.a.
n.a.	Acetate RI	n.a.	n.a.	n.a.	n.a.	n.a.	n.a.
n.a.	1,2 PDO RI	n.a.	n.a.	n.a.	n.a.	n.a.	n.a.
n.a.	1,3-PDO	n.a.	n.a.	n.a.	n.a.	n.a.	n.a.
1	Propionate RI	11,602	0,312	1,148	100,00	100,00	13,0483
n.a.	1,3-PDO	n.a.	n.a.	n.a.	n.a.	n.a.	n.a.
n.a.	2-3 BDO	n.a.	n.a.	n.a.	n.a.	n.a.	n.a.
n.a.	Ethanol	n.a.	n.a.	n.a.	n.a.	n.a.	n.a.
n.a.	Isobutyrate RI	n.a.	n.a.	n.a.	n.a.	n.a.	n.a.
n.a.	Butyrate RI	n.a.	n.a.	n.a.	n.a.	n.a.	n.a.
**Total:**			**0,312**	**1,148**	**100,00**	**100,00**	

Peak Analysis

Injection Details

Injection Name:	**13.68 propionate**	*Run Time (min):*	**20,00**
Vial Number:	**3:6**	*Injection Volume:*	**10,00**
Injection Type:	**Unknown**	*Channel:*	**RI_CH_1**
Calibration Level:		*Wavelength:*	**n.a.**
Instrument Method:	**Default method LC2030C 45 gr 20 min**	*Bandwidth:*	**n.a.**
Processing Method:	**Processing Method LC2030 45 gr**	*Dilution Factor:*	**1,0000**
Injection Date/Time:	**16/Jan/23 19:17**	*Sample Weight:*	**1,0000**

Chromatogram

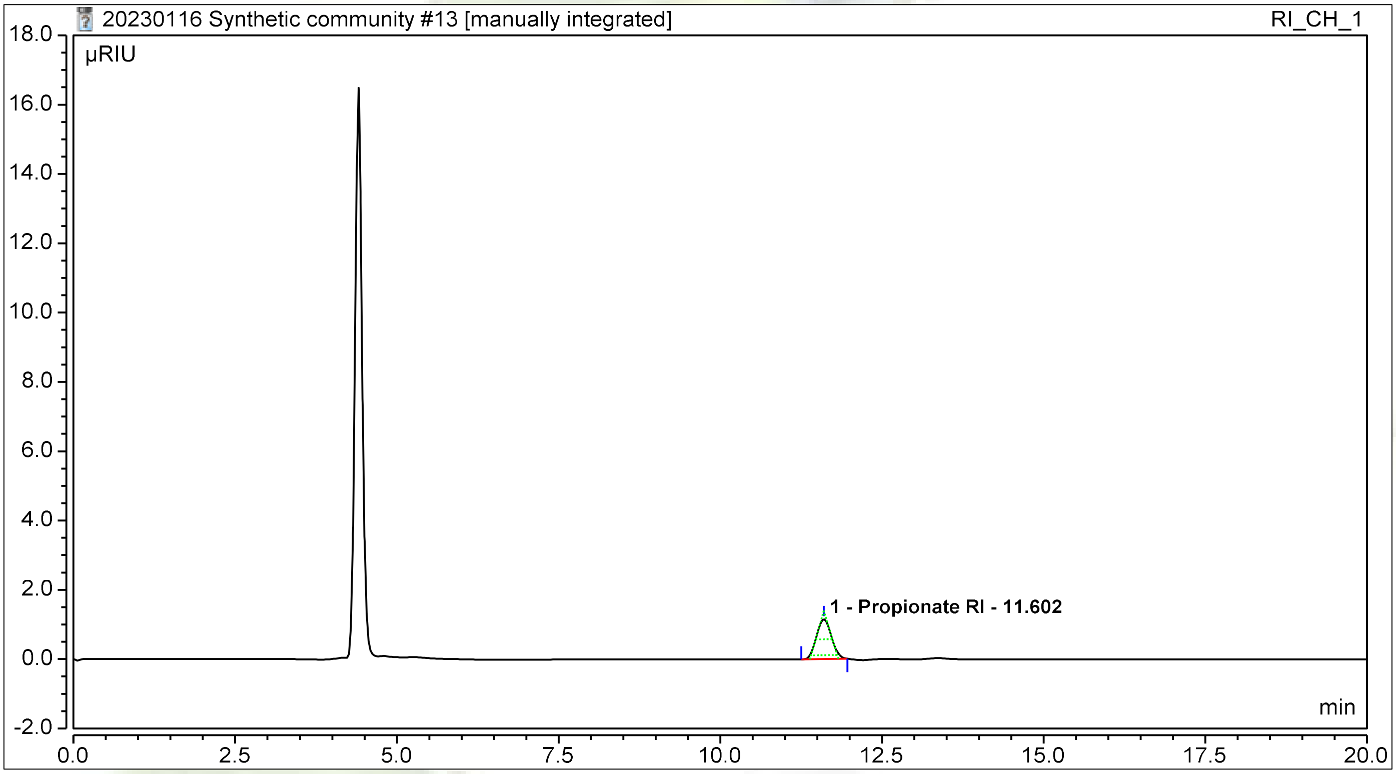

Peak Results

No.	Peak Name	Retention Time min	Width (50%) min	Type	Resolution (EP)	Asymmetry (EP)	Plates (EP)
n.a.	GlcNAc	n.a.	n.a.	n.a.	n.a.	n.a.	n.a.
n.a.	Citrate	n.a.	n.a.	n.a.	n.a.	n.a.	n.a.
n.a.	Glucose	n.a.	n.a.	n.a.	n.a.	n.a.	n.a.
n.a.	Galactose	n.a.	n.a.	n.a.	n.a.	n.a.	n.a.
n.a.	Fucose	n.a.	n.a.	n.a.	n.a.	n.a.	n.a.
n.a.	Succinate RI	n.a.	n.a.	n.a.	n.a.	n.a.	n.a.
n.a.	Lactate RI	n.a.	n.a.	n.a.	n.a.	n.a.	n.a.
n.a.	glycerol	n.a.	n.a.	n.a.	n.a.	n.a.	n.a.
n.a.	Formate RI	n.a.	n.a.	n.a.	n.a.	n.a.	n.a.
n.a.	Acetate RI	n.a.	n.a.	n.a.	n.a.	n.a.	n.a.
n.a.	1,2 PDO RI	n.a.	n.a.	n.a.	n.a.	n.a.	n.a.
n.a.	1,3-PDO	n.a.	n.a.	n.a.	n.a.	n.a.	n.a.
1	Propionate RI	11,602	0,259	BMB*	n.a.	1,06	11131
n.a.	1,3-PDO	n.a.	n.a.	n.a.	n.a.	n.a.	n.a.
n.a.	2-3 BDO	n.a.	n.a.	n.a.	n.a.	n.a.	n.a.
n.a.	Ethanol	n.a.	n.a.	n.a.	n.a.	n.a.	n.a.
n.a.	Isobutyrate RI	n.a.	n.a.	n.a.	n.a.	n.a.	n.a.
n.a.	Butyrate RI	n.a.	n.a.	n.a.	n.a.	n.a.	n.a.

Chromatogram and SST Results

Injection Details

Injection Name:	**13.68 propionate**	*Run Time (min):*	**20,00**
Vial Number:	**3:6**	*Injection Volume:*	**10,00**
Injection Type:	**Unknown**	*Channel:*	**RI_CH_1**
Calibration Level:		*Wavelength:*	**n.a.**
Instrument Method:	**Default method LC2030C 45 gr 20 min**	*Bandwidth:*	**n.a.**
Processing Method:	**Processing Method LC2030 45 gr**	*Dilution Factor:*	**1,0000**
Injection Date/Time:	**16/Jan/23 19:17**	*Sample Weight:*	**1,0000**

Chromatogram

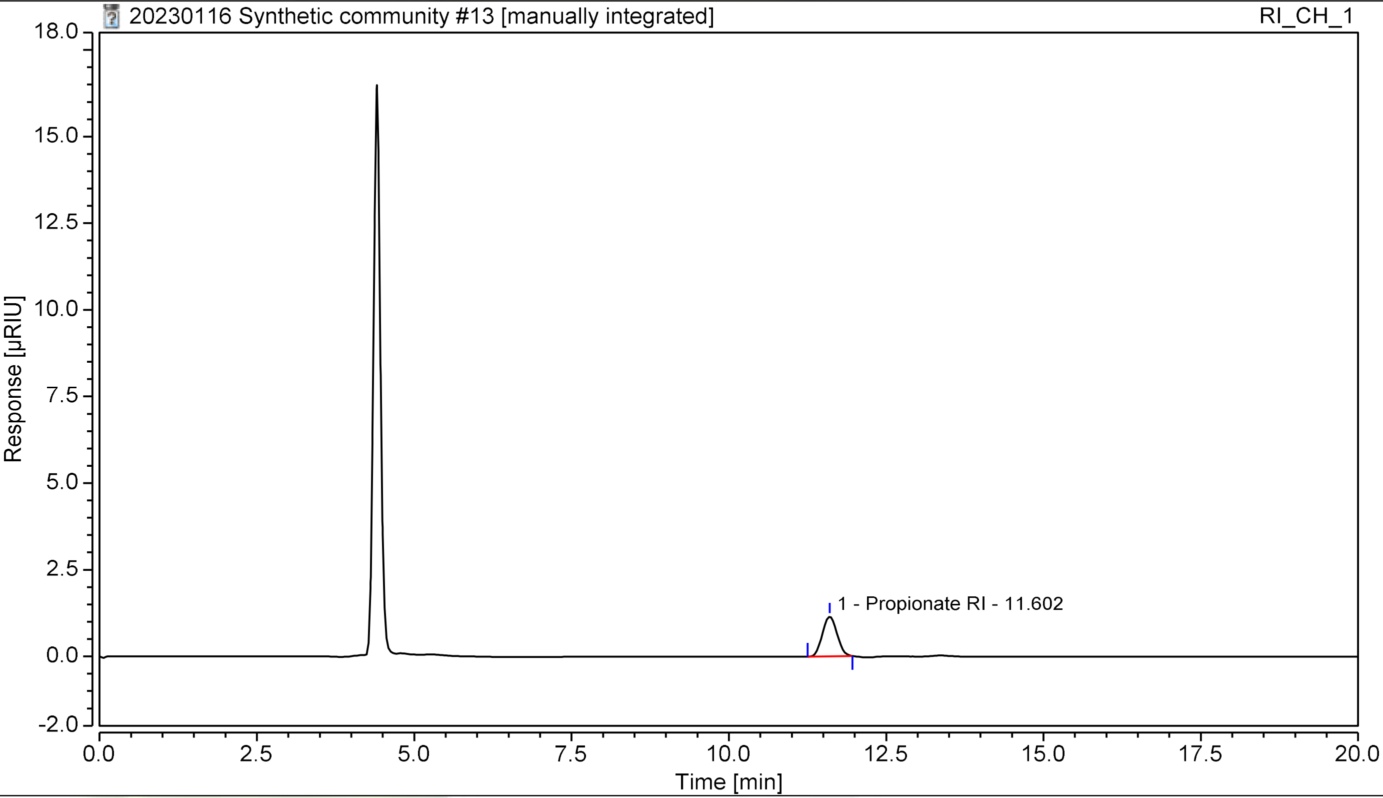

SST Results

No.	Name	Inj.Condition	Peak	Test Result	Injection
**Number of executed test cases:**	**n.a.**		**Total Result:**	**Passed**	

Chromeleon (c) Dionex
Version 7.2.10.25868

Chromatogram and Results

Injection Details

Injection Name:	**GOSFOS t72 r1**	*Run Time (min):*	**20,00**
Vial Number:	**3:7**	*Injection Volume:*	**10,00**
Injection Type:	**Unknown**	*Channel:*	**RI_CH_1**
Calibration Level:		*Wavelength:*	**n.a.**
Instrument Method:	**Default method LC2030C 45 gr 20 min**	*Bandwidth:*	**n.a.**
Processing Method:	**Processing Method LC2030 45 gr**	*Dilution Factor:*	**1,0000**
Injection Date/Time:	**16/Jan/23 19:38**	*Sample Weight:*	**1,0000**

Chromatogram

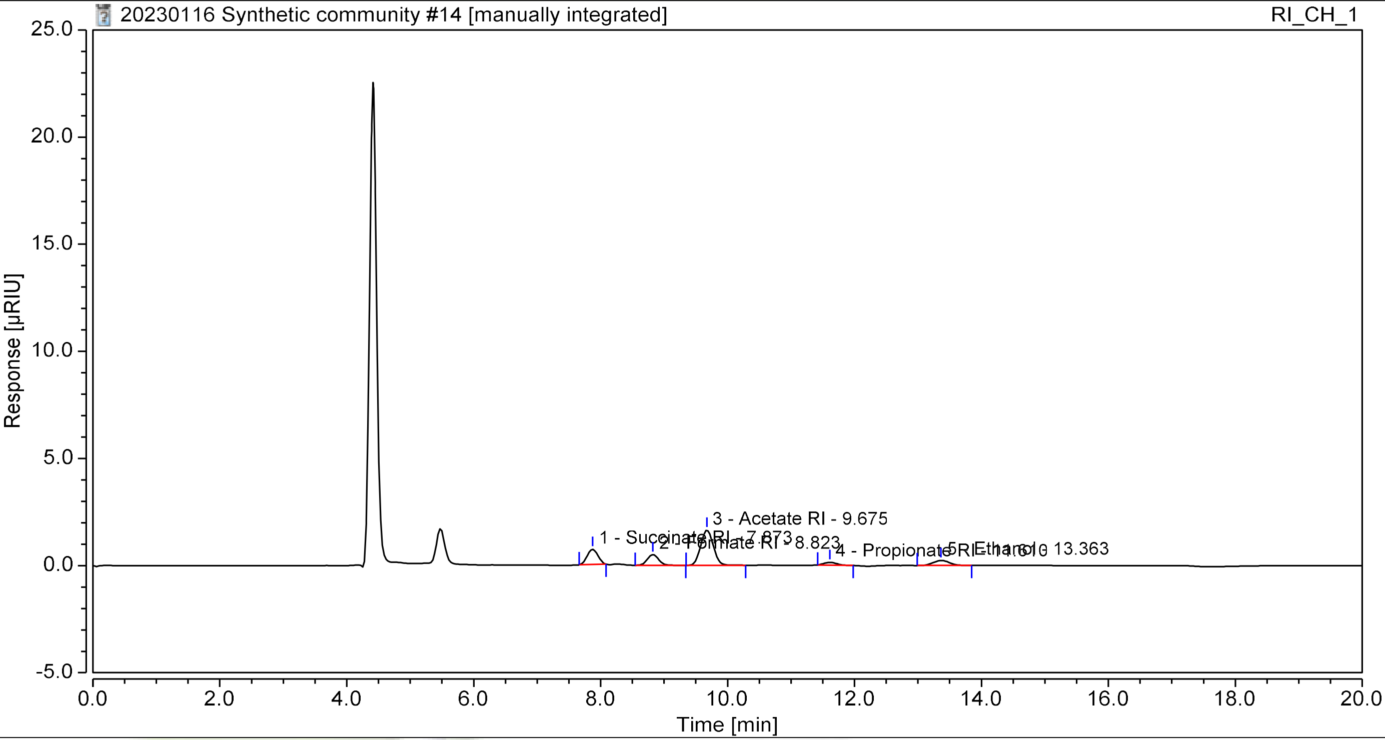

Integration Results

No.	Peak Name	Retention Time min	Area µRIU*min	Height µRIU	Relative Area %	Relative Height %	Amount
n.a.	GlcNAc	n.a.	n.a.	n.a.	n.a.	n.a.	n.a.
n.a.	Citrate	n.a.	n.a.	n.a.	n.a.	n.a.	n.a.
n.a.	Glucose	n.a.	n.a.	n.a.	n.a.	n.a.	n.a.
n.a.	Galactose	n.a.	n.a.	n.a.	n.a.	n.a.	n.a.
n.a.	Fucose	n.a.	n.a.	n.a.	n.a.	n.a.	n.a.
1	Succinate RI	7,873	0,140	0,704	19,40	22,08	2,8916
n.a.	Lactate RI	n.a.	n.a.	n.a.	n.a.	n.a.	n.a.
n.a.	glycerol	n.a.	n.a.	n.a.	n.a.	n.a.	n.a.
2	Formate RI	8,823	0,103	0,492	14,23	15,44	10,5137
3	Acetate RI	9,675	0,379	1,634	52,51	51,24	23,5150
n.a.	1,2 PDO RI	n.a.	n.a.	n.a.	n.a.	n.a.	n.a.
n.a.	1,3-PDO	n.a.	n.a.	n.a.	n.a.	n.a.	n.a.
4	Propionate RI	11,610	0,029	0,122	3,96	3,81	1,1967
n.a.	1,3-PDO	n.a.	n.a.	n.a.	n.a.	n.a.	n.a.
n.a.	2-3 BDO	n.a.	n.a.	n.a.	n.a.	n.a.	n.a.
5	Ethanol	13,363	0,072	0,237	9,90	7,42	7,5007
n.a.	Isobutyrate RI	n.a.	n.a.	n.a.	n.a.	n.a.	n.a.
n.a.	Butyrate RI	n.a.	n.a.	n.a.	n.a.	n.a.	n.a.
**Total:**			**0,722**	**3,188**	**100,00**	**100,00**	

Peak Analysis

Injection Details

Injection Name:	**GOSFOS t72 r1**	*Run Time (min):*	**20,00**
Vial Number:	**3:7**	*Injection Volume:*	**10,00**
Injection Type:	**Unknown**	*Channel:*	**RI_CH_1**
Calibration Level:		*Wavelength:*	**n.a.**
Instrument Method:	**Default method LC2030C 45 gr 20 min**	*Bandwidth:*	**n.a.**
Processing Method:	**Processing Method LC2030 45 gr**	*Dilution Factor:*	**1,0000**
Injection Date/Time:	**16/Jan/23 19:38**	*Sample Weight:*	**1,0000**

Chromatogram

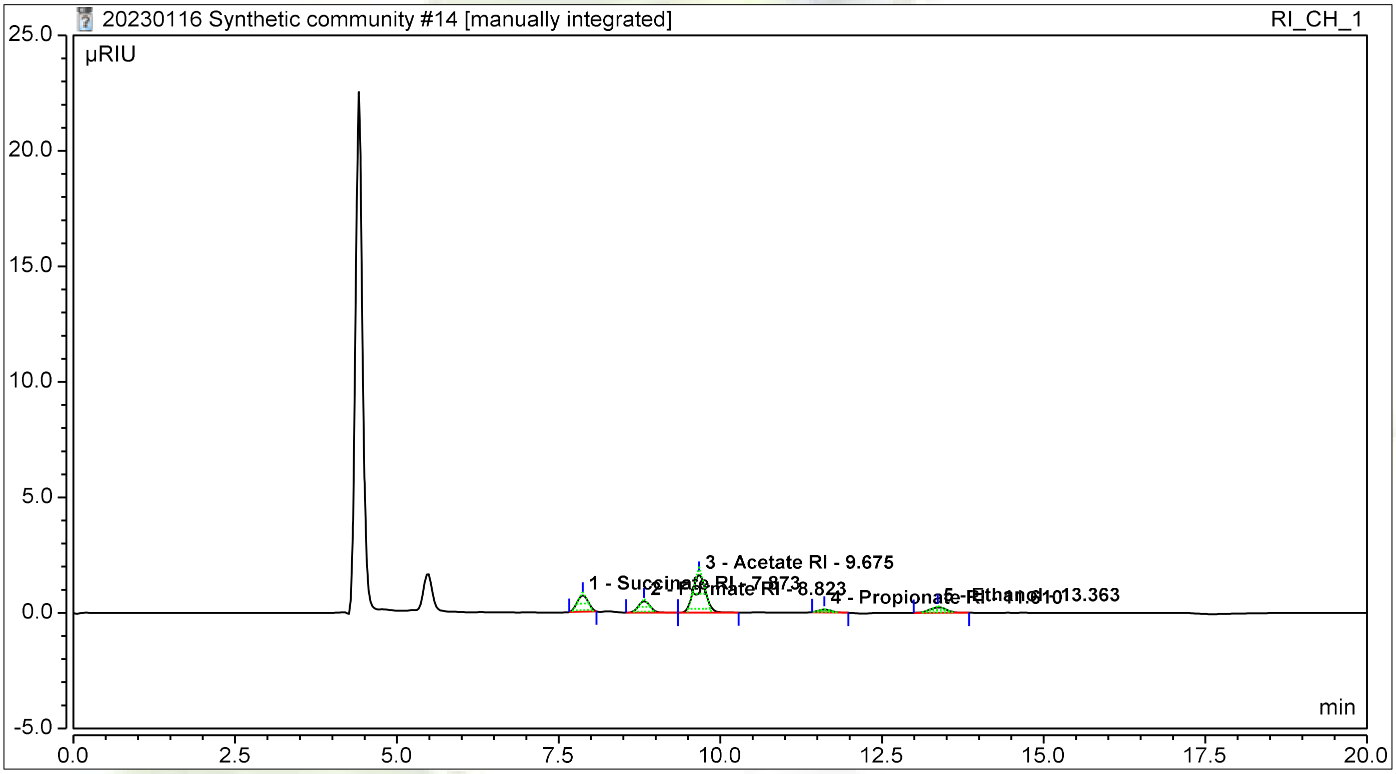

Peak Results

No.	Peak Name	Retention Time min	Width (50%) min	Type	Resolution (EP)	Asymmetry (EP)	Plates (EP)
n.a.	GlcNAc	n.a.	n.a.	n.a.	n.a.	n.a.	n.a.
n.a.	Citrate	n.a.	n.a.	n.a.	n.a.	n.a.	n.a.
n.a.	Glucose	n.a.	n.a.	n.a.	n.a.	n.a.	n.a.
n.a.	Galactose	n.a.	n.a.	n.a.	n.a.	n.a.	n.a.
n.a.	Fucose	n.a.	n.a.	n.a.	n.a.	n.a.	n.a.
1	Succinate RI	7,873	0,193	BMB*	2,89	1,02	9240
n.a.	Lactate RI	n.a.	n.a.	n.a.	n.a.	n.a.	n.a.
n.a.	glycerol	n.a.	n.a.	n.a.	n.a.	n.a.	n.a.
2	Formate RI	8,823	0,195	BMB	2,43	1,06	11303
3	Acetate RI	9,675	0,218	BMB	5,13	1,05	10919
n.a.	1,2 PDO RI	n.a.	n.a.	n.a.	n.a.	n.a.	n.a.
n.a.	1,3-PDO	n.a.	n.a.	n.a.	n.a.	n.a.	n.a.
4	Propionate RI	11,610	0,227	BMB*	4,02	1,22	14491
n.a.	1,3-PDO	n.a.	n.a.	n.a.	n.a.	n.a.	n.a.
n.a.	2-3 BDO	n.a.	n.a.	n.a.	n.a.	n.a.	n.a.
5	Ethanol	13,363	0,287	BMB*	n.a.	1,04	12008
n.a.	Isobutyrate RI	n.a.	n.a.	n.a.	n.a.	n.a.	n.a.
n.a.	Butyrate RI	n.a.	n.a.	n.a.	n.a.	n.a.	n.a.

Chromatogram and SST Results

Injection Details

Injection Name:	**GOSFOS t72 r1**	*Run Time (min):*	**20,00**
Vial Number:	**3:7**	*Injection Volume:*	**10,00**
Injection Type:	**Unknown**	*Channel:*	**RI_CH_1**
Calibration Level:		*Wavelength:*	**n.a.**
Instrument Method:	**Default method LC2030C 45 gr 20 min**	*Bandwidth:*	**n.a.**
Processing Method:	**Processing Method LC2030 45 gr**	*Dilution Factor:*	**1,0000**
Injection Date/Time:	**16/Jan/23 19:38**	*Sample Weight:*	**1,0000**

Chromatogram

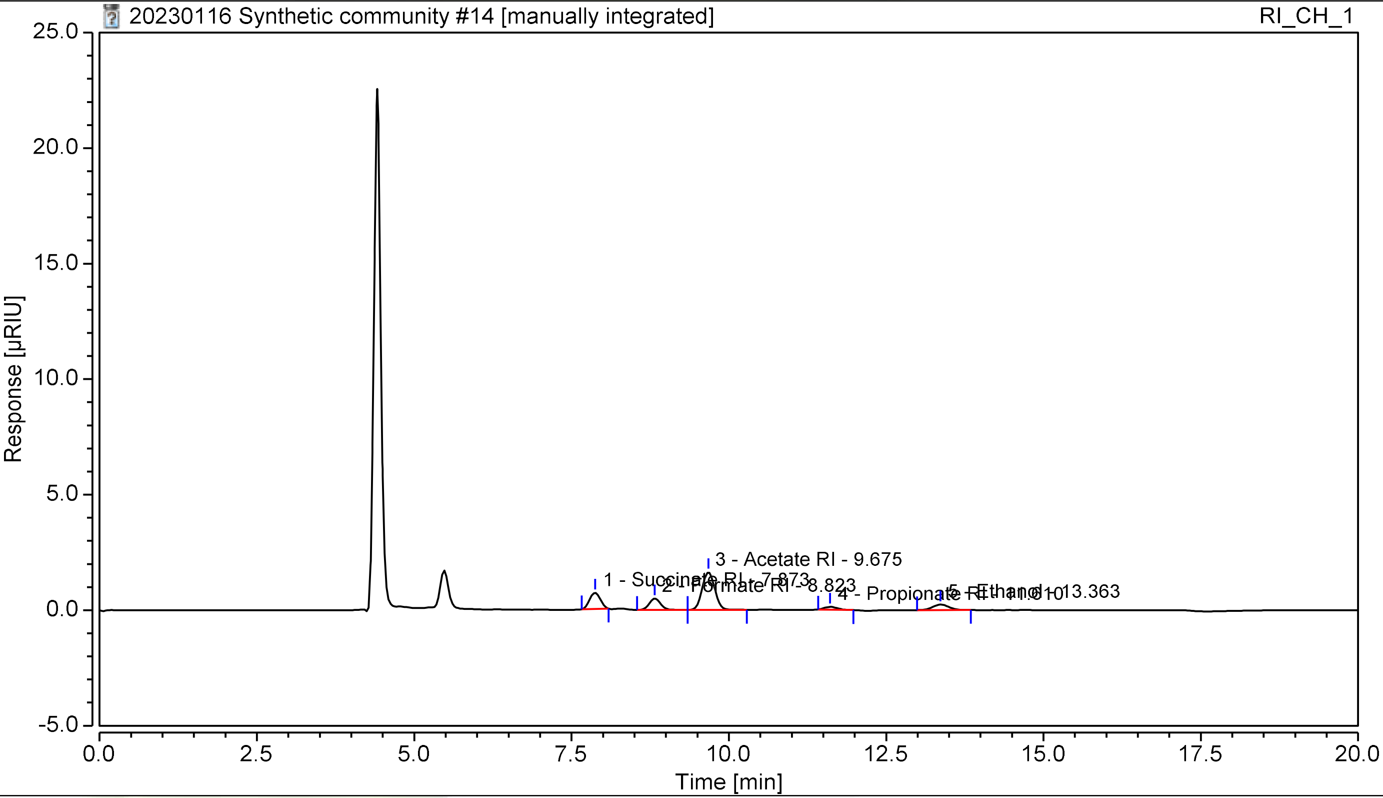

SST Results

No.	Name	Inj.Condition	Peak	Test Result	Injection

Number of executed test cases: n.a. **Total Result: Passed**

Chromatogram and Results

Injection Details

Injection Name:	**GOSFOS t72 r2**	*Run Time (min):*	**20,00**
Vial Number:	**3:8**	*Injection Volume:*	**10,00**
Injection Type:	**Unknown**	*Channel:*	**RI_CH_1**
Calibration Level:		*Wavelength:*	**n.a.**
Instrument Method:	**Default method LC2030C 45 gr 20 min**	*Bandwidth:*	**n.a.**
Processing Method:	**Processing Method LC2030 45 gr**	*Dilution Factor:*	**1,0000**
Injection Date/Time:	**16/Jan/23 19:58**	*Sample Weight:*	**1,0000**

Chromatogram

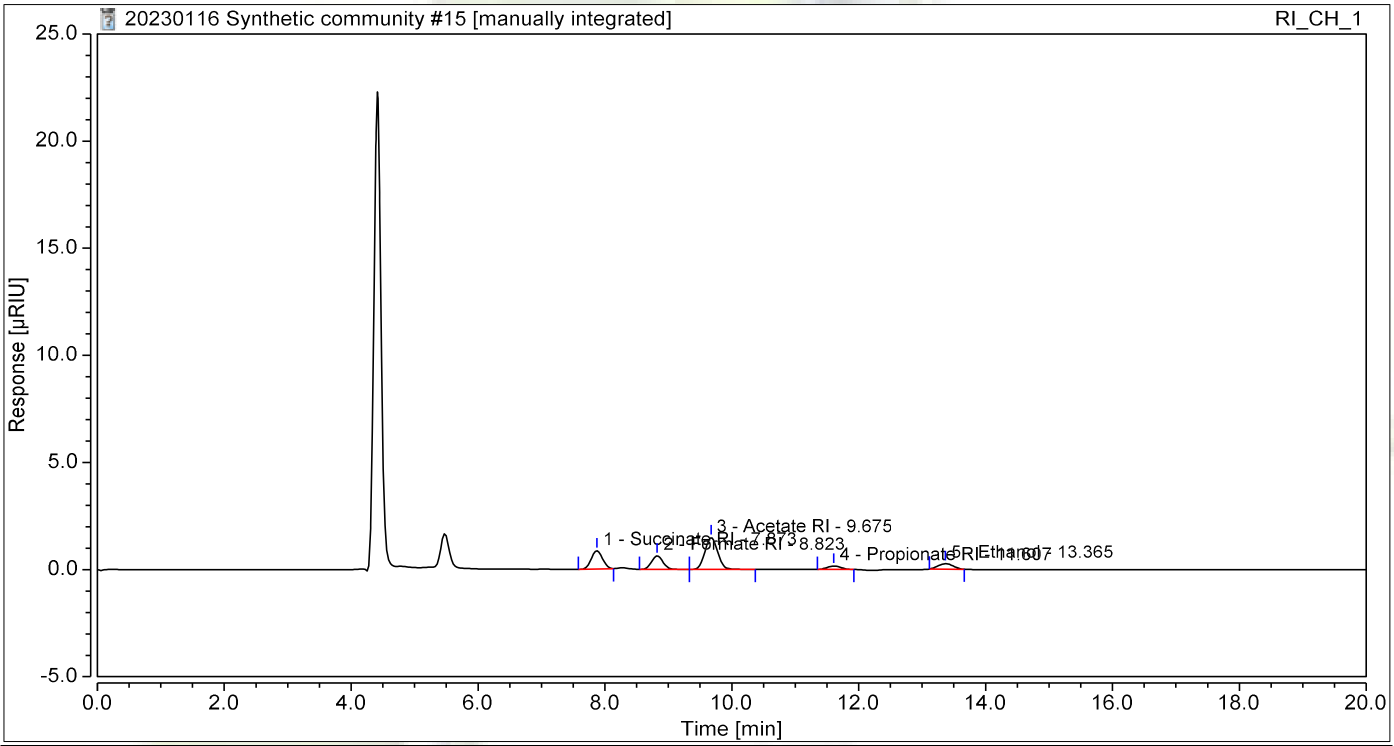

Integration Results

No.	Peak Name	Retention Time min	Area µRIU*min	Height µRIU	Relative Area %	Relative Height %	Amount
n.a.	GlcNAc	n.a.	n.a.	n.a.	n.a.	n.a.	n.a.
n.a.	Citrate	n.a.	n.a.	n.a.	n.a.	n.a.	n.a.
n.a.	Glucose	n.a.	n.a.	n.a.	n.a.	n.a.	n.a.
n.a.	Galactose	n.a.	n.a.	n.a.	n.a.	n.a.	n.a.
n.a.	Fucose	n.a.	n.a.	n.a.	n.a.	n.a.	n.a.
1	Succinate RI	7,873	0,174	0,845	23,05	25,21	3,5927
n.a.	Lactate RI	n.a.	n.a.	n.a.	n.a.	n.a.	n.a.
n.a.	glycerol	n.a.	n.a.	n.a.	n.a.	n.a.	n.a.
2	Formate RI	8,823	0,130	0,626	17,23	18,67	13,3194
3	Acetate RI	9,675	0,343	1,474	45,43	43,98	21,2769
n.a.	1,2 PDO RI	n.a.	n.a.	n.a.	n.a.	n.a.	n.a.
n.a.	1,3-PDO	n.a.	n.a.	n.a.	n.a.	n.a.	n.a.
4	Propionate RI	11,607	0,039	0,155	5,11	4,62	1,6135
n.a.	1,3-PDO	n.a.	n.a.	n.a.	n.a.	n.a.	n.a.
n.a.	2-3 BDO	n.a.	n.a.	n.a.	n.a.	n.a.	n.a.
5	Ethanol	13,365	0,069	0,252	9,17	7,52	7,2669
n.a.	Isobutyrate RI	n.a.	n.a.	n.a.	n.a.	n.a.	n.a.
n.a.	Butyrate RI	n.a.	n.a.	n.a.	n.a.	n.a.	n.a.
**Total:**			**0,755**	**3,351**	**100,00**	**100,00**	

Peak Analysis

Injection Details

Injection Name:	**GOSFOS t72 r2**	*Run Time (min):*	**20,00**
Vial Number:	**3:8**	*Injection Volume:*	**10,00**
Injection Type:	**Unknown**	*Channel:*	**RI_CH_1**
Calibration Level:		*Wavelength:*	**n.a.**
Instrument Method:	**Default method LC2030C 45 gr 20 min**	*Bandwidth:*	**n.a.**
Processing Method:	**Processing Method LC2030 45 gr**	*Dilution Factor:*	**1,0000**
Injection Date/Time:	**16/Jan/23 19:58**	*Sample Weight:*	**1,0000**

Chromatogram

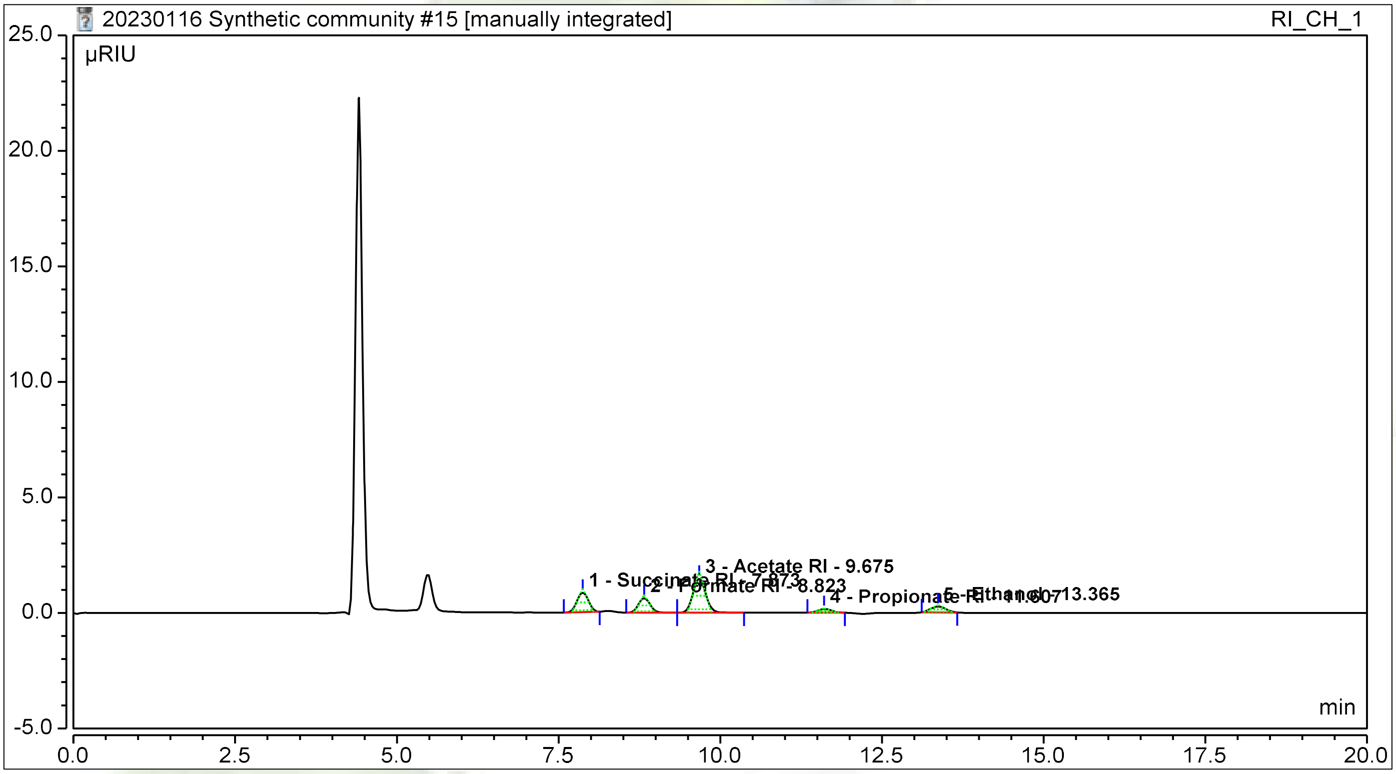

Peak Results

No.	Peak Name	Retention Time min	Width (50%) min	Type	Resolution (EP)	Asymmetry (EP)	Plates (EP)
n.a.	GlcNAc	n.a.	n.a.	n.a.	n.a.	n.a.	n.a.
n.a.	Citrate	n.a.	n.a.	n.a.	n.a.	n.a.	n.a.
n.a.	Glucose	n.a.	n.a.	n.a.	n.a.	n.a.	n.a.
n.a.	Galactose	n.a.	n.a.	n.a.	n.a.	n.a.	n.a.
n.a.	Fucose	n.a.	n.a.	n.a.	n.a.	n.a.	n.a.
1	Succinate RI	7,873	0,197	BMB	2,86	1,02	8879
n.a.	Lactate RI	n.a.	n.a.	n.a.	n.a.	n.a.	n.a.
n.a.	glycerol	n.a.	n.a.	n.a.	n.a.	n.a.	n.a.
2	Formate RI	8,823	0,195	BMB	2,43	1,07	11312
3	Acetate RI	9,675	0,218	BMB	4,98	1,05	10883
n.a.	1,2 PDO RI	n.a.	n.a.	n.a.	n.a.	n.a.	n.a.
n.a.	1,3-PDO	n.a.	n.a.	n.a.	n.a.	n.a.	n.a.
4	Propionate RI	11,607	0,239	BMB*	4,07	1,10	13063
n.a.	1,3-PDO	n.a.	n.a.	n.a.	n.a.	n.a.	n.a.
n.a.	2-3 BDO	n.a.	n.a.	n.a.	n.a.	n.a.	n.a.
5	Ethanol	13,365	0,270	BMB*	n.a.	1,06	13553
n.a.	Isobutyrate RI	n.a.	n.a.	n.a.	n.a.	n.a.	n.a.
n.a.	Butyrate RI	n.a.	n.a.	n.a.	n.a.	n.a.	n.a.

Chromatogram and SST Results

Injection Details

Injection Name:	**GOSFOS t72 r2**	*Run Time (min):*	**20,00**
Vial Number:	**3:8**	*Injection Volume:*	**10,00**
Injection Type:	**Unknown**	*Channel:*	**RI_CH_1**
Calibration Level:		*Wavelength:*	**n.a.**
Instrument Method:	**Default method LC2030C 45 gr 20 min**	*Bandwidth:*	**n.a.**
Processing Method:	**Processing Method LC2030 45 gr**	*Dilution Factor:*	**1,0000**
Injection Date/Time:	**16/Jan/23 19:58**	*Sample Weight:*	**1,0000**

Chromatogram

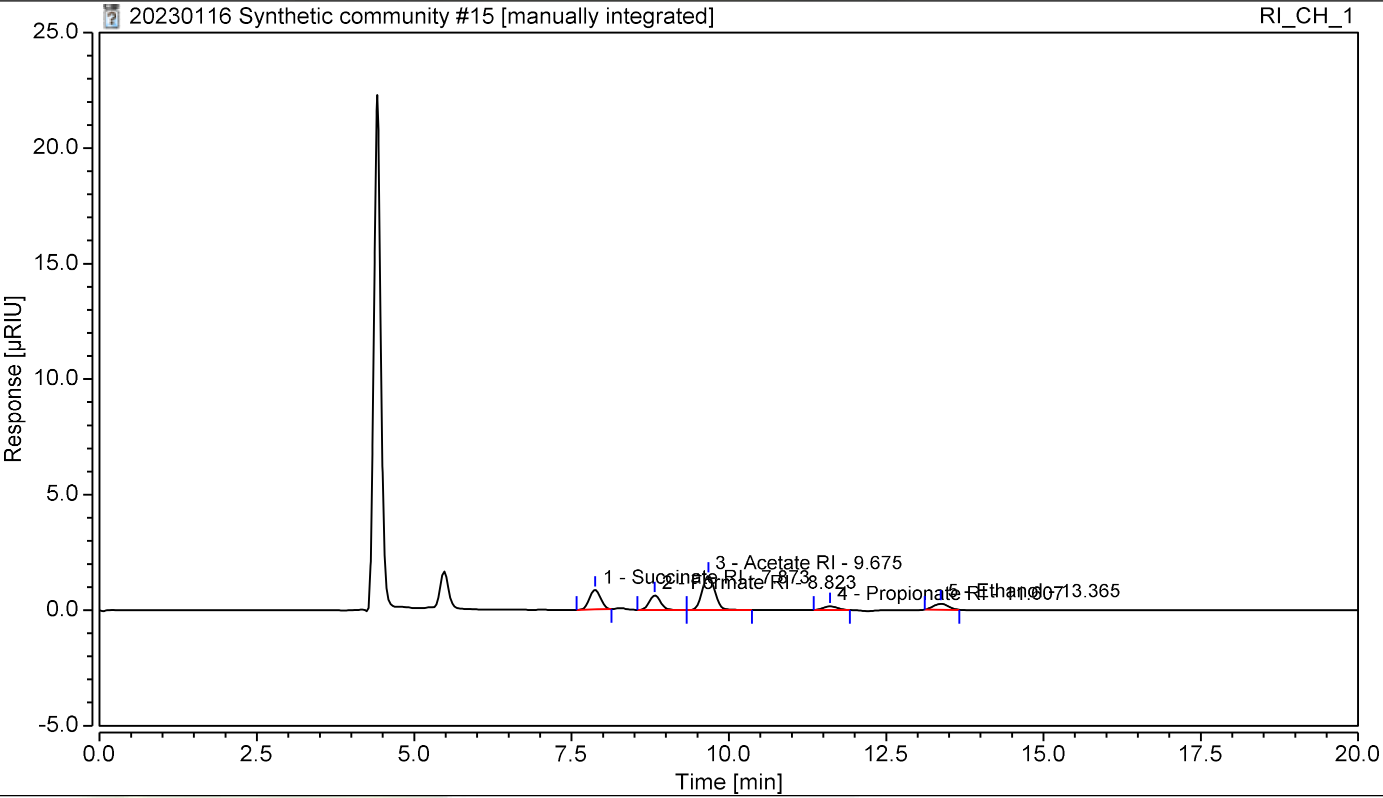

SST Results

No.	Name	Inj.Condition	Peak	Test Result	Injection

Number of executed test cases: **n.a.** **Total Result:** **Passed**

Chromatogram and Results

Injection Details

Injection Name:	**GOSFOS t72 r3**	*Run Time (min):*	**20,00**
Vial Number:	**3:9**	*Injection Volume:*	**10,00**
Injection Type:	**Unknown**	*Channel:*	**RI_CH_1**
Calibration Level:		*Wavelength:*	**n.a.**
Instrument Method:	**Default method LC2030C 45 gr 20 min**	*Bandwidth:*	**n.a.**
Processing Method:	**Processing Method LC2030 45 gr**	*Dilution Factor:*	**1,0000**
Injection Date/Time:	**16/Jan/23 20:18**	*Sample Weight:*	**1,0000**

Chromatogram

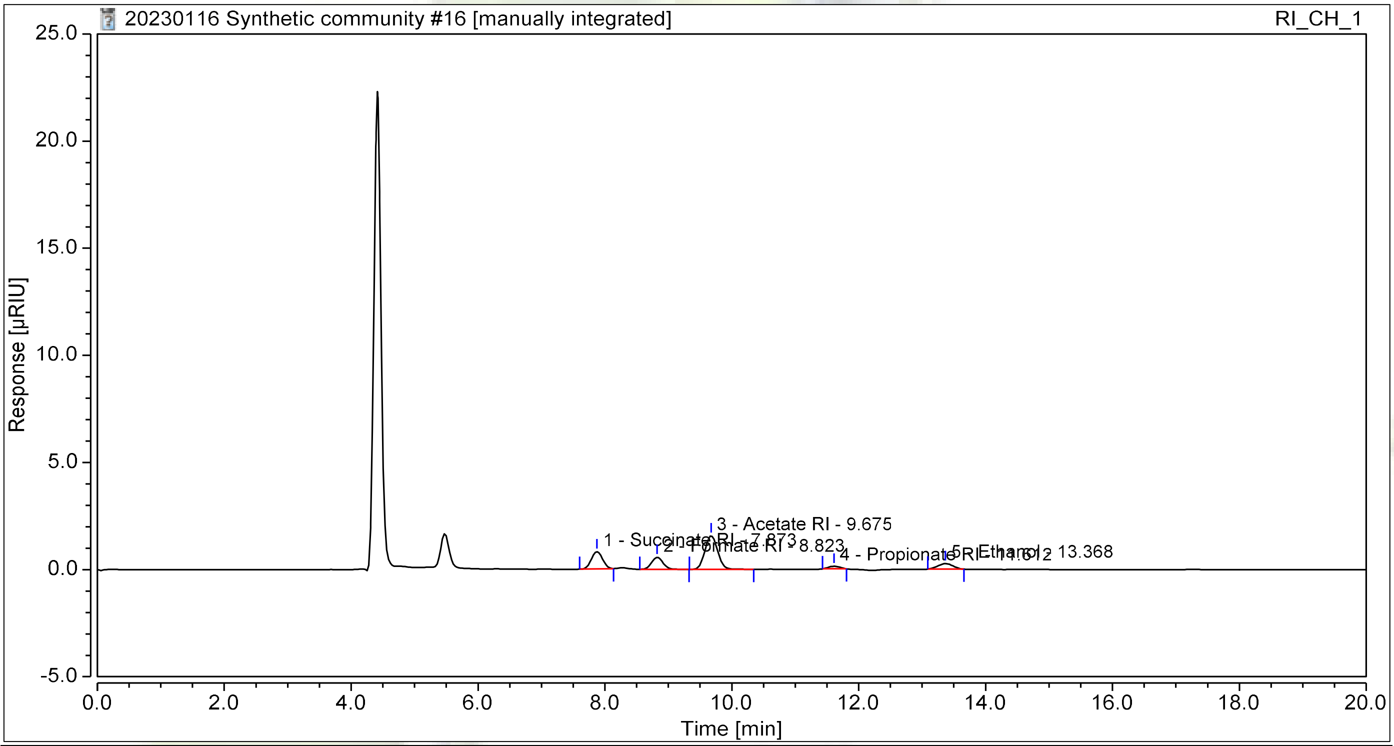

Integration Results

No.	Peak Name	Retention Time min	Area µRIU*min	Height µRIU	Relative Area %	Relative Height %	Amount
n.a.	GlcNAc	n.a.	n.a.	n.a.	n.a.	n.a.	n.a.
n.a.	Citrate	n.a.	n.a.	n.a.	n.a.	n.a.	n.a.
n.a.	Glucose	n.a.	n.a.	n.a.	n.a.	n.a.	n.a.
n.a.	Galactose	n.a.	n.a.	n.a.	n.a.	n.a.	n.a.
n.a.	Fucose	n.a.	n.a.	n.a.	n.a.	n.a.	n.a.
1	Succinate RI	7,873	0,166	0,805	22,28	24,35	3,4195
n.a.	Lactate RI	n.a.	n.a.	n.a.	n.a.	n.a.	n.a.
n.a.	glycerol	n.a.	n.a.	n.a.	n.a.	n.a.	n.a.
2	Formate RI	8,823	0,116	0,559	15,65	16,91	11,9045
3	Acetate RI	9,675	0,364	1,562	48,92	47,27	22,5537
n.a.	1,2 PDO RI	n.a.	n.a.	n.a.	n.a.	n.a.	n.a.
n.a.	1,3-PDO	n.a.	n.a.	n.a.	n.a.	n.a.	n.a.
4	Propionate RI	11,612	0,026	0,122	3,45	3,68	1,0738
n.a.	1,3-PDO	n.a.	n.a.	n.a.	n.a.	n.a.	n.a.
n.a.	2-3 BDO	n.a.	n.a.	n.a.	n.a.	n.a.	n.a.
5	Ethanol	13,368	0,072	0,258	9,70	7,79	7,5637
n.a.	Isobutyrate RI	n.a.	n.a.	n.a.	n.a.	n.a.	n.a.
n.a.	Butyrate RI	n.a.	n.a.	n.a.	n.a.	n.a.	n.a.
**Total:**			**0,743**	**3,304**	**100,00**	**100,00**	

Chromeleon (c) Dionex
Version 7.2.10.25868

Peak Analysis

Injection Details

Injection Name:	**GOSFOS t72 r3**	*Run Time (min):*	**20,00**
Vial Number:	**3:9**	*Injection Volume:*	**10,00**
Injection Type:	**Unknown**	*Channel:*	**RI_CH_1**
Calibration Level:		*Wavelength:*	**n.a.**
Instrument Method:	**Default method LC2030C 45 gr 20 min**	*Bandwidth:*	**n.a.**
Processing Method:	**Processing Method LC2030 45 gr**	*Dilution Factor:*	**1,0000**
Injection Date/Time:	**16/Jan/23 20:18**	*Sample Weight:*	**1,0000**

Chromatogram

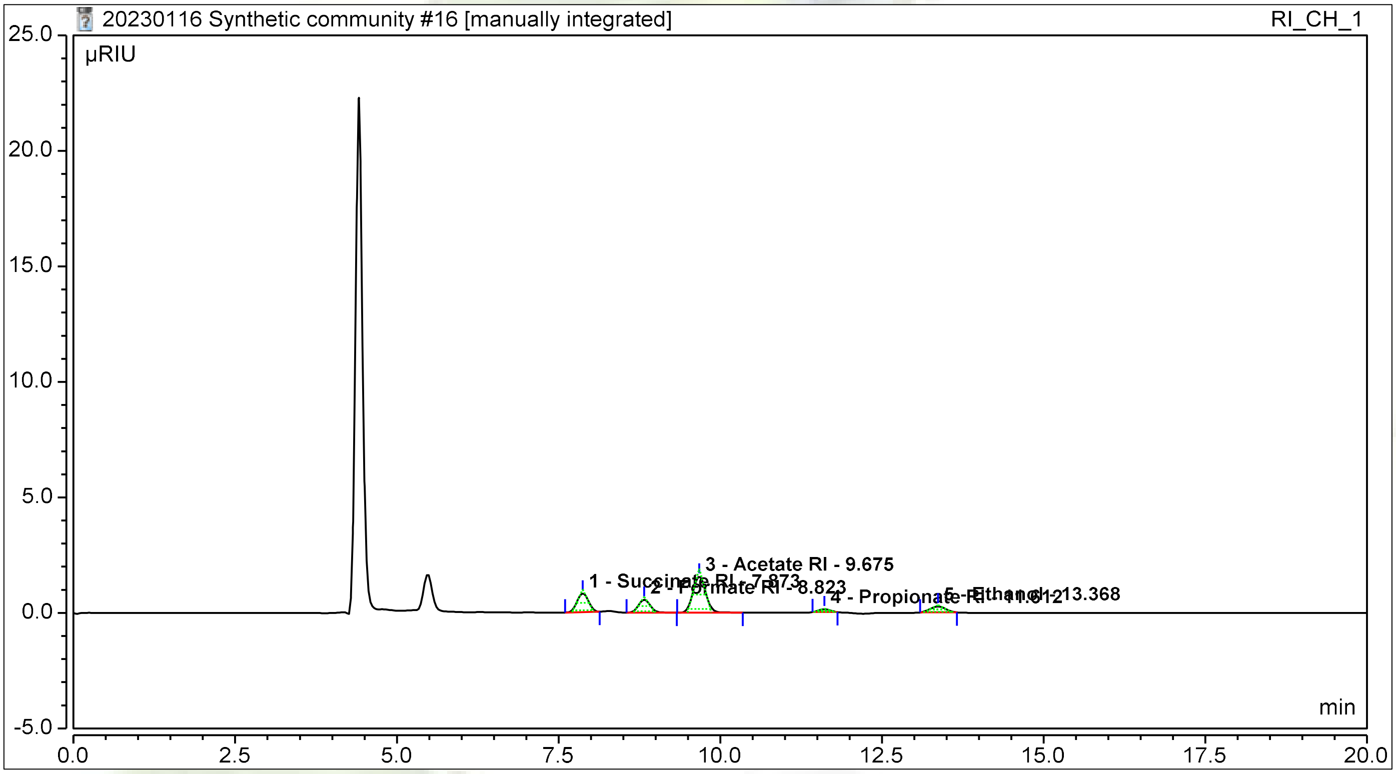

Peak Results

No.	Peak Name	Retention Time min	Width (50%) min	Type	Resolution (EP)	Asymmetry (EP)	Plates (EP)
n.a.	GlcNAc	n.a.	n.a.	n.a.	n.a.	n.a.	n.a.
n.a.	Citrate	n.a.	n.a.	n.a.	n.a.	n.a.	n.a.
n.a.	Glucose	n.a.	n.a.	n.a.	n.a.	n.a.	n.a.
n.a.	Galactose	n.a.	n.a.	n.a.	n.a.	n.a.	n.a.
n.a.	Fucose	n.a.	n.a.	n.a.	n.a.	n.a.	n.a.
1	Succinate RI	7,873	0,197	BMB	2,86	1,03	8887
n.a.	Lactate RI	n.a.	n.a.	n.a.	n.a.	n.a.	n.a.
n.a.	glycerol	n.a.	n.a.	n.a.	n.a.	n.a.	n.a.
2	Formate RI	8,823	0,195	BMB	2,43	1,07	11326
3	Acetate RI	9,675	0,218	BMB	5,31	1,05	10877
n.a.	1,2 PDO RI	n.a.	n.a.	n.a.	n.a.	n.a.	n.a.
n.a.	1,3-PDO	n.a.	n.a.	n.a.	n.a.	n.a.	n.a.
4	Propionate RI	11,612	0,212	BMB*	4,27	1,04	16577
n.a.	1,3-PDO	n.a.	n.a.	n.a.	n.a.	n.a.	n.a.
n.a.	2-3 BDO	n.a.	n.a.	n.a.	n.a.	n.a.	n.a.
5	Ethanol	13,368	0,273	BMB*	n.a.	1,01	13247
n.a.	Isobutyrate RI	n.a.	n.a.	n.a.	n.a.	n.a.	n.a.
n.a.	Butyrate RI	n.a.	n.a.	n.a.	n.a.	n.a.	n.a.

Chromatogram and SST Results

Injection Details

Injection Name:	**GOSFOS t72 r3**	*Run Time (min):*	**20,00**
Vial Number:	**3:9**	*Injection Volume:*	**10,00**
Injection Type:	**Unknown**	*Channel:*	**RI_CH_1**
Calibration Level:		*Wavelength:*	**n.a.**
Instrument Method:	**Default method LC2030C 45 gr 20 min**	*Bandwidth:*	**n.a.**
Processing Method:	**Processing Method LC2030 45 gr**	*Dilution Factor:*	**1,0000**
Injection Date/Time:	**16/Jan/23 20:18**	*Sample Weight:*	**1,0000**

Chromatogram

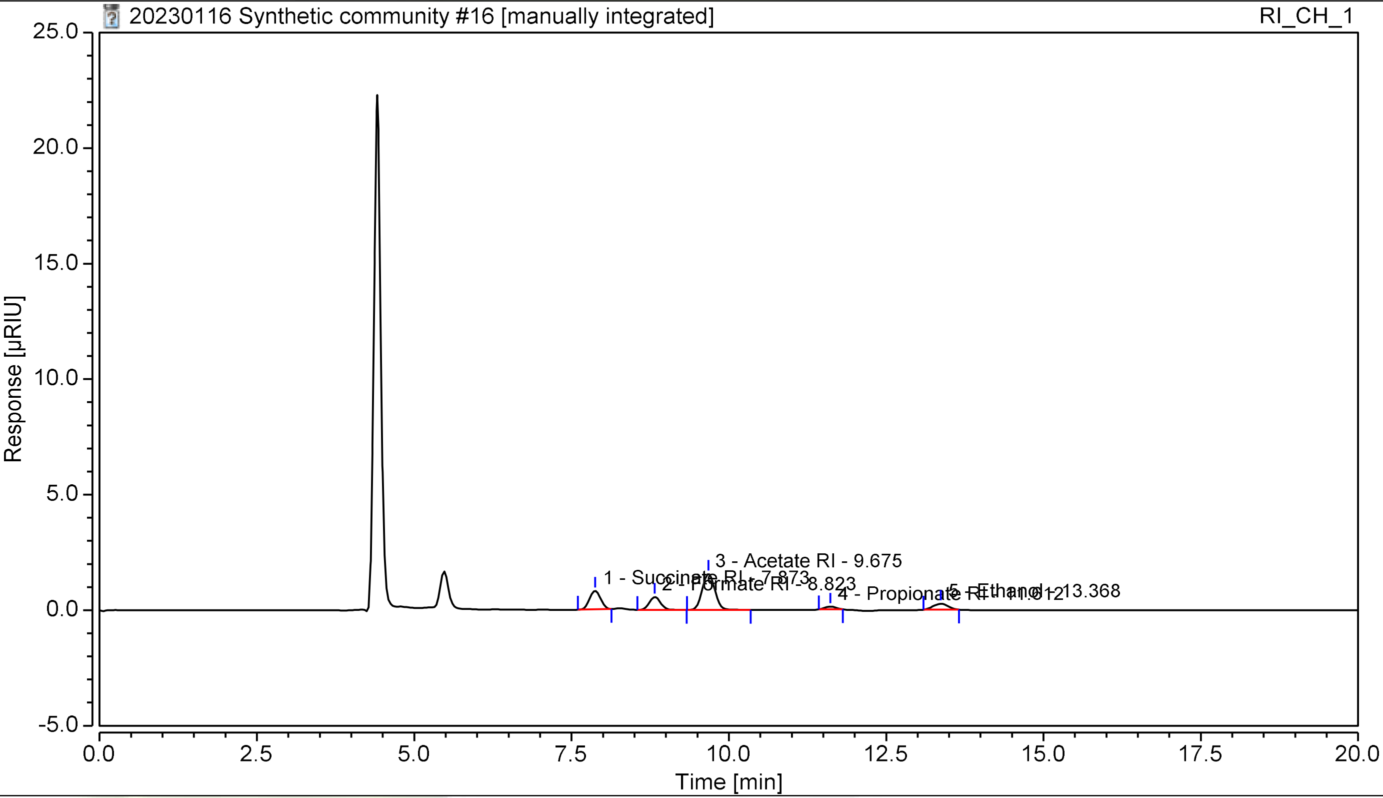

SST Results

No.	Name	Inj.Condition	Peak	Test Result	Injection

Number of executed test cases: **n.a.** **Total Result:** **Passed**

Chromatogram and Results

Injection Details

Injection Name:	**GOSFOS t96 r1**	*Run Time (min):*	**20,00**
Vial Number:	**3:10**	*Injection Volume:*	**10,00**
Injection Type:	**Unknown**	*Channel:*	**RI_CH_1**
Calibration Level:		*Wavelength:*	**n.a.**
Instrument Method:	**Default method LC2030C 45 gr 20 min**	*Bandwidth:*	**n.a.**
Processing Method:	**Processing Method LC2030 45 gr**	*Dilution Factor:*	**1,0000**
Injection Date/Time:	**16/Jan/23 20:39**	*Sample Weight:*	**1,0000**

Chromatogram

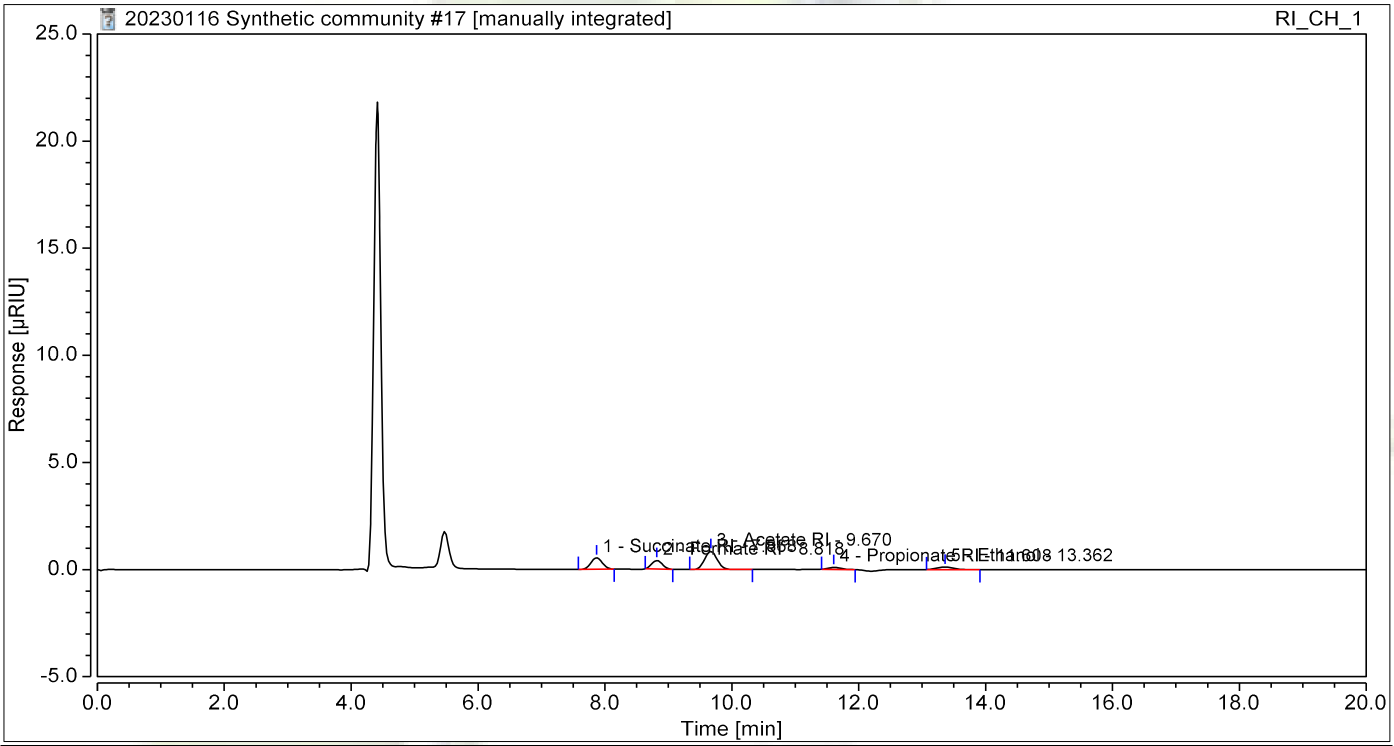

Integration Results

No.	Peak Name	Retention Time<br>min	Area<br>µRIU*min	Height<br>µRIU	Relative Area<br>%	Relative Height<br>%	Amount
n.a.	GlcNAc	n.a.	n.a.	n.a.	n.a.	n.a.	n.a.
n.a.	Citrate	n.a.	n.a.	n.a.	n.a.	n.a.	n.a.
n.a.	Glucose	n.a.	n.a.	n.a.	n.a.	n.a.	n.a.
n.a.	Galactose	n.a.	n.a.	n.a.	n.a.	n.a.	n.a.
n.a.	Fucose	n.a.	n.a.	n.a.	n.a.	n.a.	n.a.
1	Succinate RI	7,868	0,111	0,531	25,17	26,91	2,2846
n.a.	Lactate RI	n.a.	n.a.	n.a.	n.a.	n.a.	n.a.
n.a.	glycerol	n.a.	n.a.	n.a.	n.a.	n.a.	n.a.
2	Formate RI	8,818	0,077	0,393	17,44	19,93	7,8455
3	Acetate RI	9,670	0,195	0,837	44,39	42,40	12,1032
n.a.	1,2 PDO RI	n.a.	n.a.	n.a.	n.a.	n.a.	n.a.
n.a.	1,3-PDO	n.a.	n.a.	n.a.	n.a.	n.a.	n.a.
4	Propionate RI	11,608	0,022	0,092	4,92	4,65	0,9037
n.a.	1,3-PDO	n.a.	n.a.	n.a.	n.a.	n.a.	n.a.
n.a.	2-3 BDO	n.a.	n.a.	n.a.	n.a.	n.a.	n.a.
5	Ethanol	13,362	0,036	0,121	8,08	6,11	3,7267
n.a.	Isobutyrate RI	n.a.	n.a.	n.a.	n.a.	n.a.	n.a.
n.a.	Butyrate RI	n.a.	n.a.	n.a.	n.a.	n.a.	n.a.
**Total:**			**0,440**	**1,975**	**100,00**	**100,00**	

Peak Analysis

Injection Details

Injection Name:	**GOSFOS t96 r1**	*Run Time (min):*	**20,00**
Vial Number:	**3:10**	*Injection Volume:*	**10,00**
Injection Type:	**Unknown**	*Channel:*	**RI_CH_1**
Calibration Level:		*Wavelength:*	**n.a.**
Instrument Method:	**Default method LC2030C 45 gr 20 min**	*Bandwidth:*	**n.a.**
Processing Method:	**Processing Method LC2030 45 gr**	*Dilution Factor:*	**1,0000**
Injection Date/Time:	**16/Jan/23 20:39**	*Sample Weight:*	**1,0000**

Chromatogram

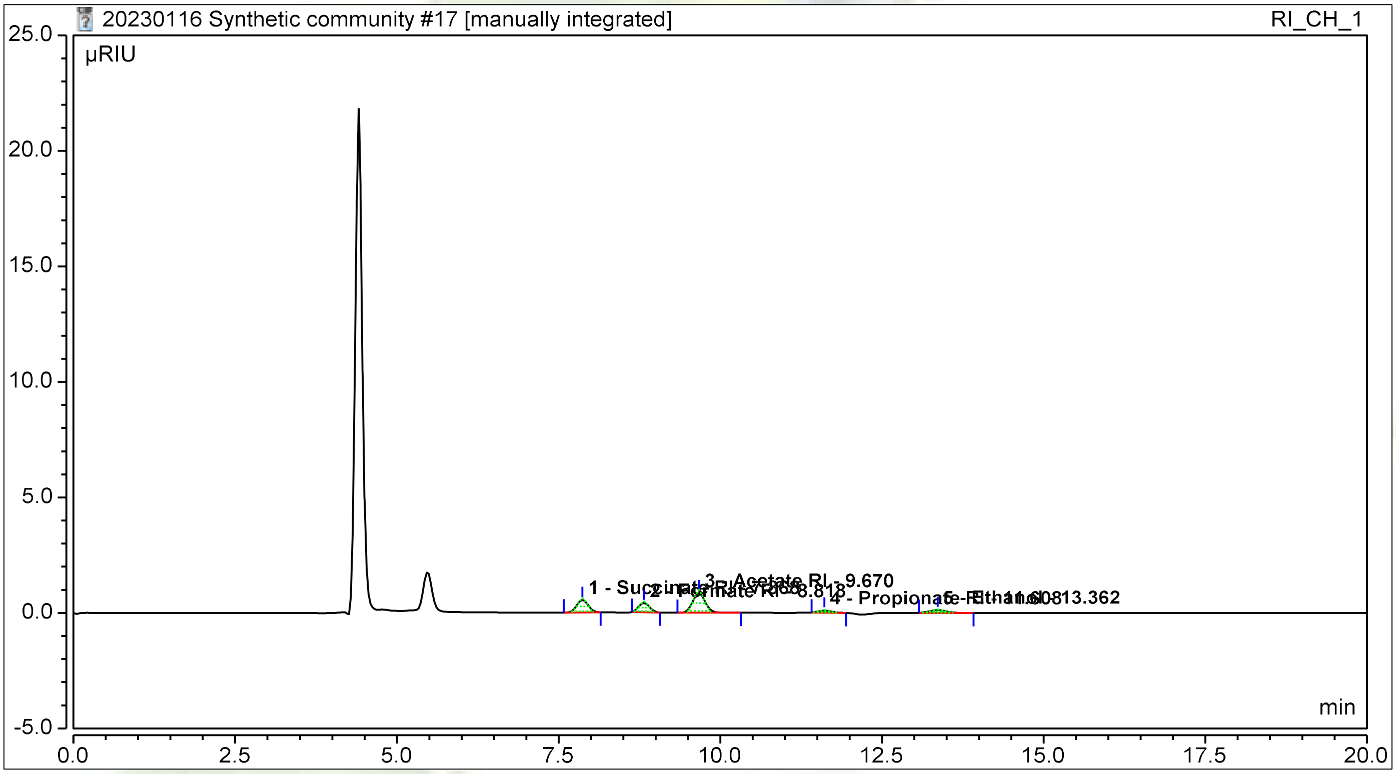

Peak Results

No.	Peak Name	Retention Time min	Width (50%) min	Type	Resolution (EP)	Asymmetry (EP)	Plates (EP)
n.a.	GlcNAc	n.a.	n.a.	n.a.	n.a.	n.a.	n.a.
n.a.	Citrate	n.a.	n.a.	n.a.	n.a.	n.a.	n.a.
n.a.	Glucose	n.a.	n.a.	n.a.	n.a.	n.a.	n.a.
n.a.	Galactose	n.a.	n.a.	n.a.	n.a.	n.a.	n.a.
n.a.	Fucose	n.a.	n.a.	n.a.	n.a.	n.a.	n.a.
1	Succinate RI	7,868	0,198	BMB	2,90	1,03	8764
n.a.	Lactate RI	n.a.	n.a.	n.a.	n.a.	n.a.	n.a.
n.a.	glycerol	n.a.	n.a.	n.a.	n.a.	n.a.	n.a.
2	Formate RI	8,818	0,188	BMB*	2,47	1,09	12130
3	Acetate RI	9,670	0,218	BMB	5,16	1,05	10879
n.a.	1,2 PDO RI	n.a.	n.a.	n.a.	n.a.	n.a.	n.a.
n.a.	1,3-PDO	n.a.	n.a.	n.a.	n.a.	n.a.	n.a.
4	Propionate RI	11,608	0,225	BMB*	4,08	1,23	14716
n.a.	1,3-PDO	n.a.	n.a.	n.a.	n.a.	n.a.	n.a.
n.a.	2-3 BDO	n.a.	n.a.	n.a.	n.a.	n.a.	n.a.
5	Ethanol	13,362	0,281	BMB*	n.a.	1,07	12502
n.a.	Isobutyrate RI	n.a.	n.a.	n.a.	n.a.	n.a.	n.a.
n.a.	Butyrate RI	n.a.	n.a.	n.a.	n.a.	n.a.	n.a.

Chromatogram and SST Results

Injection Details

Injection Name:	**GOSFOS t96 r1**	*Run Time (min):*	**20,00**
Vial Number:	**3:10**	*Injection Volume:*	**10,00**
Injection Type:	**Unknown**	*Channel:*	**RI_CH_1**
Calibration Level:		*Wavelength:*	**n.a.**
Instrument Method:	**Default method LC2030C 45 gr 20 min**	*Bandwidth:*	**n.a.**
Processing Method:	**Processing Method LC2030 45 gr**	*Dilution Factor:*	**1,0000**
Injection Date/Time:	**16/Jan/23 20:39**	*Sample Weight:*	**1,0000**

Chromatogram

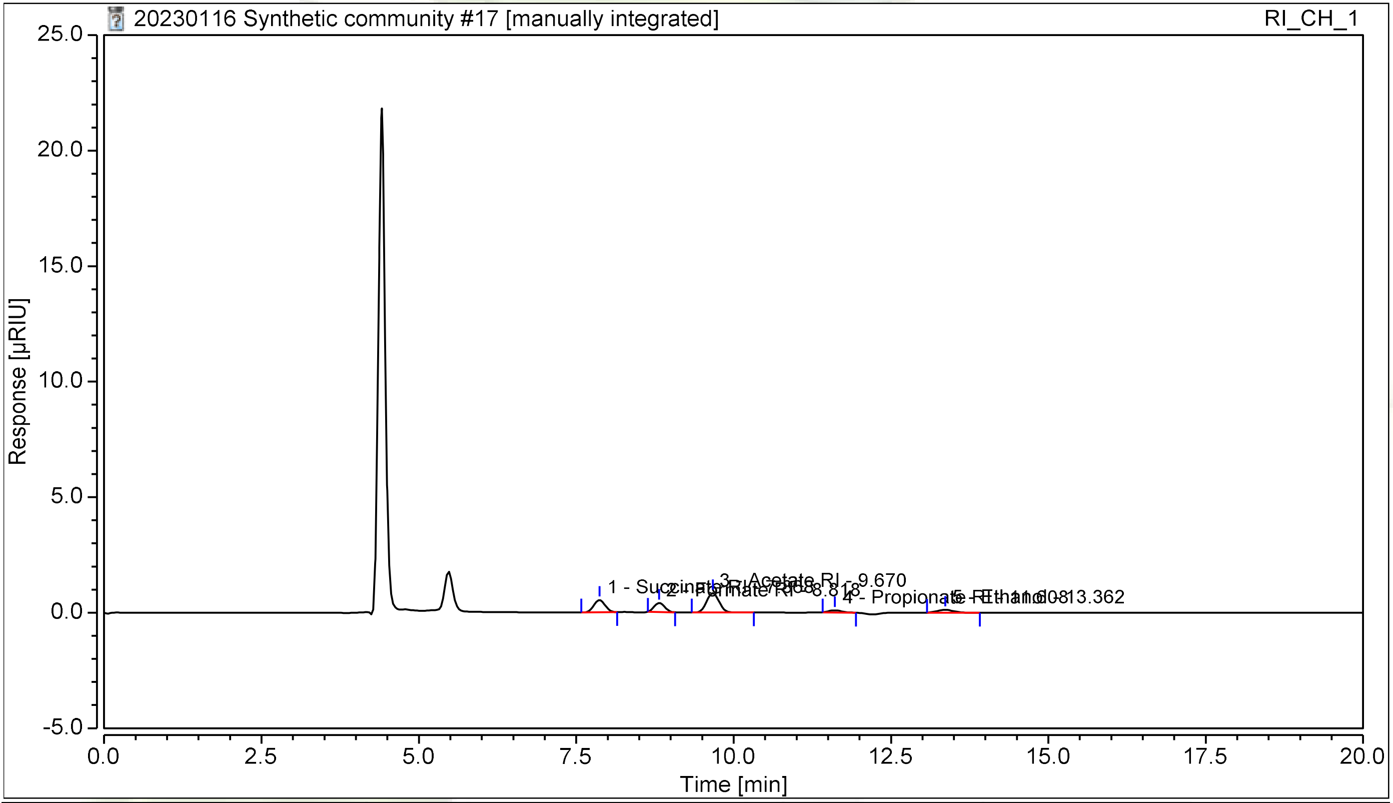

SST Results

No.	Name	Inj.Condition	Peak	Test Result	Injection

Number of executed test cases: **n.a.** **Total Result:** **Passed**

Chromatogram and Results

Injection Details

Injection Name:	**GOSFOS t96 r2**	*Run Time (min):*	**20,00**
Vial Number:	**3:11**	*Injection Volume:*	**10,00**
Injection Type:	**Unknown**	*Channel:*	**RI_CH_1**
Calibration Level:		*Wavelength:*	**n.a.**
Instrument Method:	**Default method LC2030C 45 gr 20 min**	*Bandwidth:*	**n.a.**
Processing Method:	**Processing Method LC2030 45 gr**	*Dilution Factor:*	**1,0000**
Injection Date/Time:	**16/Jan/23 20:59**	*Sample Weight:*	**1,0000**

Chromatogram

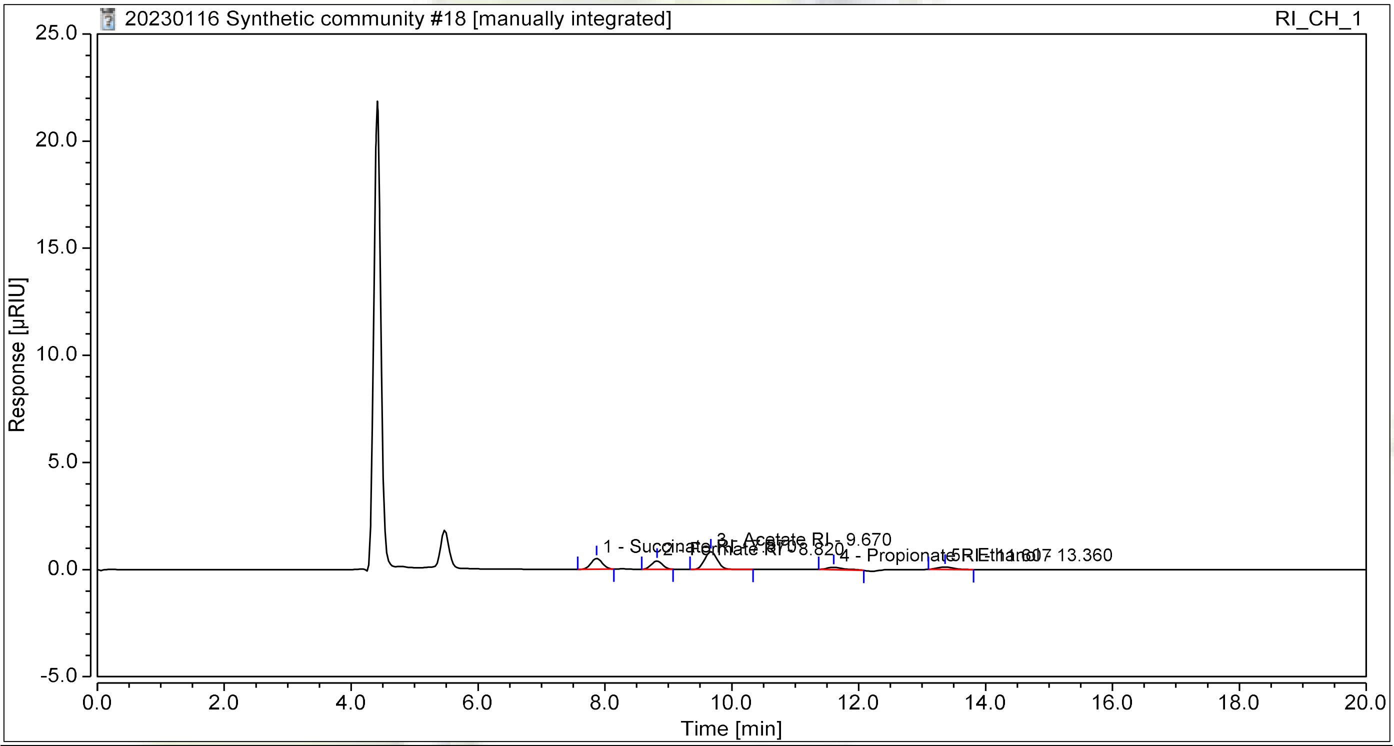

Integration Results

No.	Peak Name	Retention Time min	Area µRIU*min	Height µRIU	Relative Area %	Relative Height %	Amount
n.a.	GlcNAc	n.a.	n.a.	n.a.	n.a.	n.a.	n.a.
n.a.	Citrate	n.a.	n.a.	n.a.	n.a.	n.a.	n.a.
n.a.	Glucose	n.a.	n.a.	n.a.	n.a.	n.a.	n.a.
n.a.	Galactose	n.a.	n.a.	n.a.	n.a.	n.a.	n.a.
n.a.	Fucose	n.a.	n.a.	n.a.	n.a.	n.a.	n.a.
1	Succinate RI	7,870	0,104	0,499	23,59	25,96	2,1418
n.a.	Lactate RI	n.a.	n.a.	n.a.	n.a.	n.a.	n.a.
n.a.	glycerol	n.a.	n.a.	n.a.	n.a.	n.a.	n.a.
2	Formate RI	8,820	0,078	0,384	17,70	19,97	7,9678
3	Acetate RI	9,670	0,192	0,822	43,60	42,76	11,8916
n.a.	1,2 PDO RI	n.a.	n.a.	n.a.	n.a.	n.a.	n.a.
n.a.	1,3-PDO	n.a.	n.a.	n.a.	n.a.	n.a.	n.a.
4	Propionate RI	11,607	0,035	0,108	7,91	5,63	1,4543
n.a.	1,3-PDO	n.a.	n.a.	n.a.	n.a.	n.a.	n.a.
n.a.	2-3 BDO	n.a.	n.a.	n.a.	n.a.	n.a.	n.a.
5	Ethanol	13,360	0,032	0,109	7,21	5,68	3,3250
n.a.	Isobutyrate RI	n.a.	n.a.	n.a.	n.a.	n.a.	n.a.
n.a.	Butyrate RI	n.a.	n.a.	n.a.	n.a.	n.a.	n.a.
**Total:**			**0,440**	**1,923**	**100,00**	**100,00**	

Peak Analysis

Injection Details

Injection Name:	**GOSFOS t96 r2**	*Run Time (min):*	**20,00**
Vial Number:	**3:11**	*Injection Volume:*	**10,00**
Injection Type:	**Unknown**	*Channel:*	**RI_CH_1**
Calibration Level:		*Wavelength:*	**n.a.**
Instrument Method:	**Default method LC2030C 45 gr 20 min**	*Bandwidth:*	**n.a.**
Processing Method:	**Processing Method LC2030 45 gr**	*Dilution Factor:*	**1,0000**
Injection Date/Time:	**16/Jan/23 20:59**	*Sample Weight:*	**1,0000**

Chromatogram

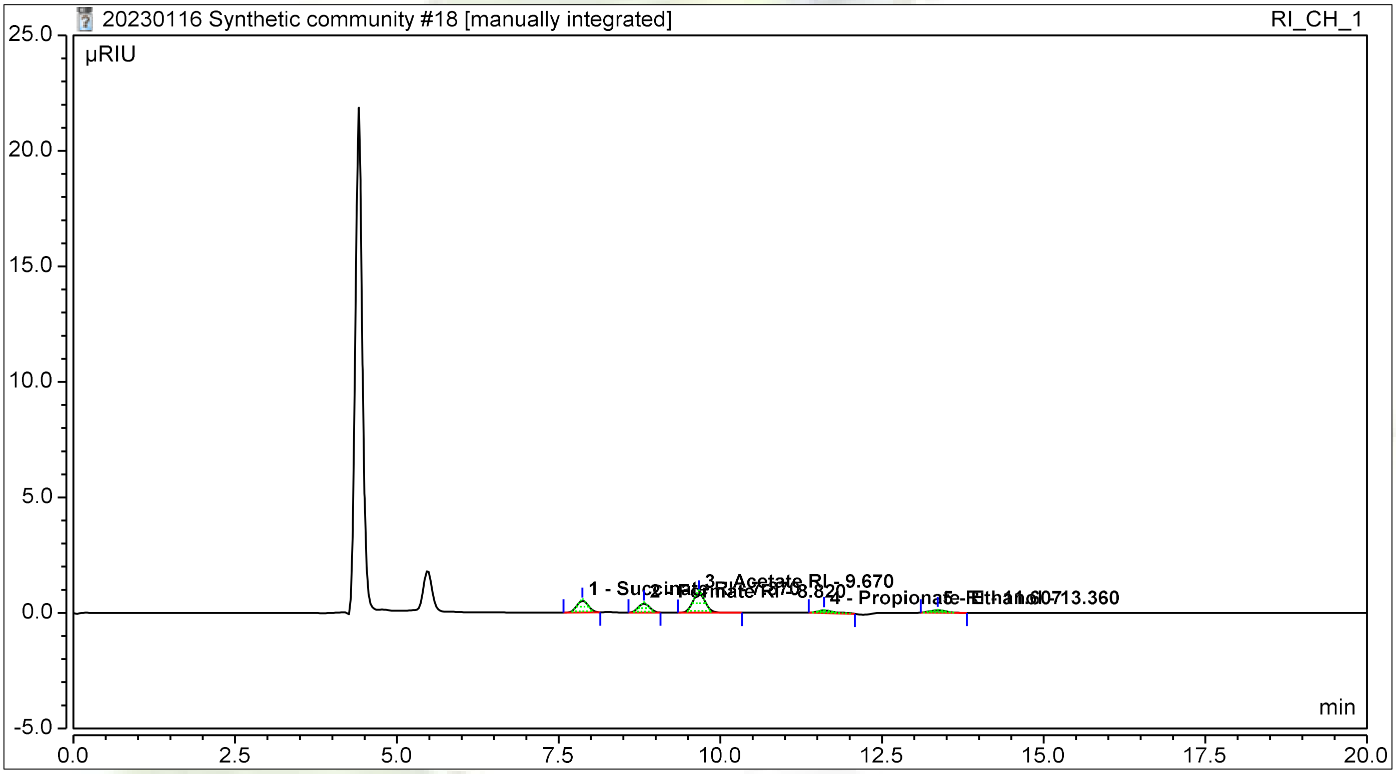

Peak Results

No.	Peak Name	Retention Time min	Width (50%) min	Type	Resolution (EP)	Asymmetry (EP)	Plates (EP)
n.a.	GlcNAc	n.a.	n.a.	n.a.	n.a.	n.a.	n.a.
n.a.	Citrate	n.a.	n.a.	n.a.	n.a.	n.a.	n.a.
n.a.	Glucose	n.a.	n.a.	n.a.	n.a.	n.a.	n.a.
n.a.	Galactose	n.a.	n.a.	n.a.	n.a.	n.a.	n.a.
n.a.	Fucose	n.a.	n.a.	n.a.	n.a.	n.a.	n.a.
1	Succinate RI	7,870	0,198	BMB	2,87	1,03	8778
n.a.	Lactate RI	n.a.	n.a.	n.a.	n.a.	n.a.	n.a.
n.a.	glycerol	n.a.	n.a.	n.a.	n.a.	n.a.	n.a.
2	Formate RI	8,820	0,193	BMB*	2,44	1,03	11512
3	Acetate RI	9,670	0,218	BMB	4,76	1,06	10863
n.a.	1,2 PDO RI	n.a.	n.a.	n.a.	n.a.	n.a.	n.a.
n.a.	1,3-PDO	n.a.	n.a.	n.a.	n.a.	n.a.	n.a.
4	Propionate RI	11,607	0,262	BMB*	3,81	1,62	10890
n.a.	1,3-PDO	n.a.	n.a.	n.a.	n.a.	n.a.	n.a.
n.a.	2-3 BDO	n.a.	n.a.	n.a.	n.a.	n.a.	n.a.
5	Ethanol	13,360	0,281	BMB*	n.a.	1,11	12528
n.a.	Isobutyrate RI	n.a.	n.a.	n.a.	n.a.	n.a.	n.a.
n.a.	Butyrate RI	n.a.	n.a.	n.a.	n.a.	n.a.	n.a.

Chromeleon (c) Dionex
Version 7.2.10.25868

Chromatogram and SST Results

Injection Details

Injection Name:	**GOSFOS t96 r2**	*Run Time (min):*	**20,00**
Vial Number:	**3:11**	*Injection Volume:*	**10,00**
Injection Type:	**Unknown**	*Channel:*	**RI_CH_1**
Calibration Level:		*Wavelength:*	**n.a.**
Instrument Method:	**Default method LC2030C 45 gr 20 min**	*Bandwidth:*	**n.a.**
Processing Method:	**Processing Method LC2030 45 gr**	*Dilution Factor:*	**1,0000**
Injection Date/Time:	**16/Jan/23 20:59**	*Sample Weight:*	**1,0000**

Chromatogram

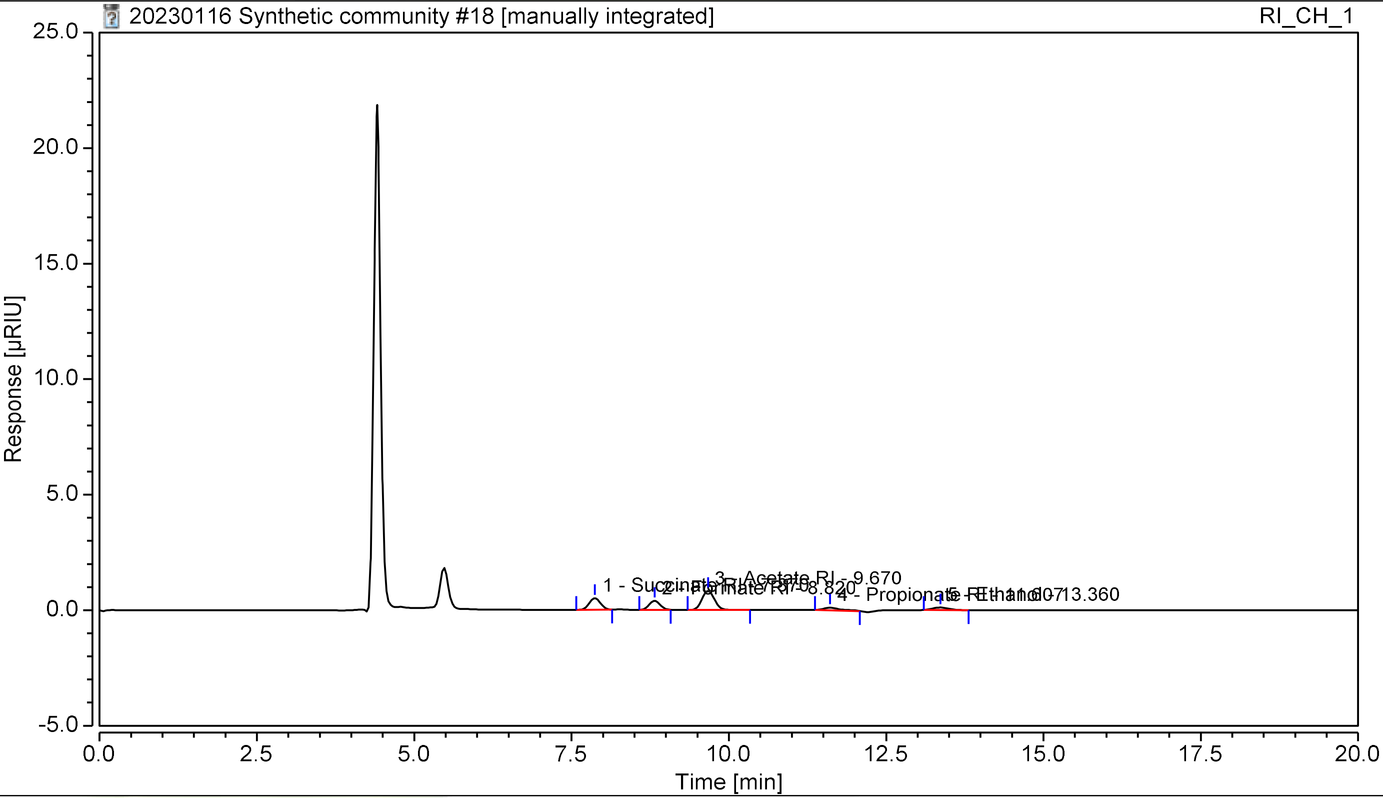

SST Results

No.	Name	Inj.Condition	Peak	Test Result	Injection
**Number of executed test cases:**	**n.a.**		**Total Result:**	**Passed**	

Chromatogram and Results

Injection Details

Injection Name:	**GOSFOS t96 r3**	*Run Time (min):*	**20,00**
Vial Number:	**3:12**	*Injection Volume:*	**10,00**
Injection Type:	**Unknown**	*Channel:*	**RI_CH_1**
Calibration Level:		*Wavelength:*	**n.a.**
Instrument Method:	**Default method LC2030C 45 gr 20 min**	*Bandwidth:*	**n.a.**
Processing Method:	**Processing Method LC2030 45 gr**	*Dilution Factor:*	**1,0000**
Injection Date/Time:	**16/Jan/23 21:20**	*Sample Weight:*	**1,0000**

Chromatogram

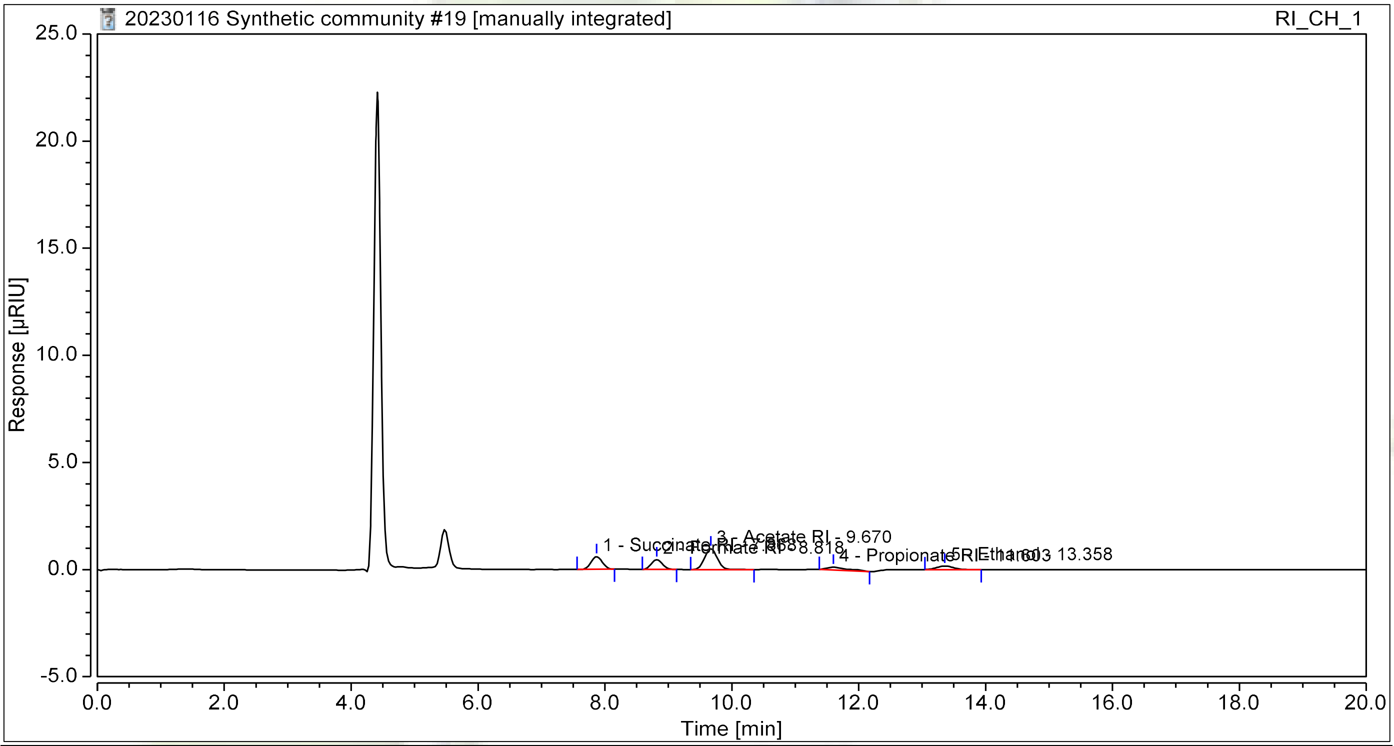

Integration Results

No.	Peak Name	Retention Time<br>min	Area<br>µRIU*min	Height<br>µRIU	Relative Area<br>%	Relative Height<br>%	Amount
n.a.	GlcNAc	n.a.	n.a.	n.a.	n.a.	n.a.	n.a.
n.a.	Citrate	n.a.	n.a.	n.a.	n.a.	n.a.	n.a.
n.a.	Glucose	n.a.	n.a.	n.a.	n.a.	n.a.	n.a.
n.a.	Galactose	n.a.	n.a.	n.a.	n.a.	n.a.	n.a.
n.a.	Fucose	n.a.	n.a.	n.a.	n.a.	n.a.	n.a.
1	Succinate RI	7,868	0,123	0,591	22,71	25,62	2,5390
n.a.	Lactate RI	n.a.	n.a.	n.a.	n.a.	n.a.	n.a.
n.a.	glycerol	n.a.	n.a.	n.a.	n.a.	n.a.	n.a.
2	Formate RI	8,818	0,093	0,456	17,21	19,77	9,5374
3	Acetate RI	9,670	0,226	0,968	41,68	41,93	14,0022
n.a.	1,2 PDO RI	n.a.	n.a.	n.a.	n.a.	n.a.	n.a.
n.a.	1,3-PDO	n.a.	n.a.	n.a.	n.a.	n.a.	n.a.
4	Propionate RI	11,603	0,051	0,127	9,33	5,51	2,1133
n.a.	1,3-PDO	n.a.	n.a.	n.a.	n.a.	n.a.	n.a.
n.a.	2-3 BDO	n.a.	n.a.	n.a.	n.a.	n.a.	n.a.
5	Ethanol	13,358	0,049	0,165	9,07	7,16	5,1553
n.a.	Isobutyrate RI	n.a.	n.a.	n.a.	n.a.	n.a.	n.a.
n.a.	Butyrate RI	n.a.	n.a.	n.a.	n.a.	n.a.	n.a.
**Total:**			**0,542**	**2,308**	**100,00**	**100,00**	

Peak Analysis

Injection Details

Injection Name:	**GOSFOS t96 r3**	*Run Time (min):*	**20,00**
Vial Number:	**3:12**	*Injection Volume:*	**10,00**
Injection Type:	**Unknown**	*Channel:*	**RI_CH_1**
Calibration Level:		*Wavelength:*	**n.a.**
Instrument Method:	**Default method LC2030C 45 gr 20 min**	*Bandwidth:*	**n.a.**
Processing Method:	**Processing Method LC2030 45 gr**	*Dilution Factor:*	**1,0000**
Injection Date/Time:	**16/Jan/23 21:20**	*Sample Weight:*	**1,0000**

Chromatogram

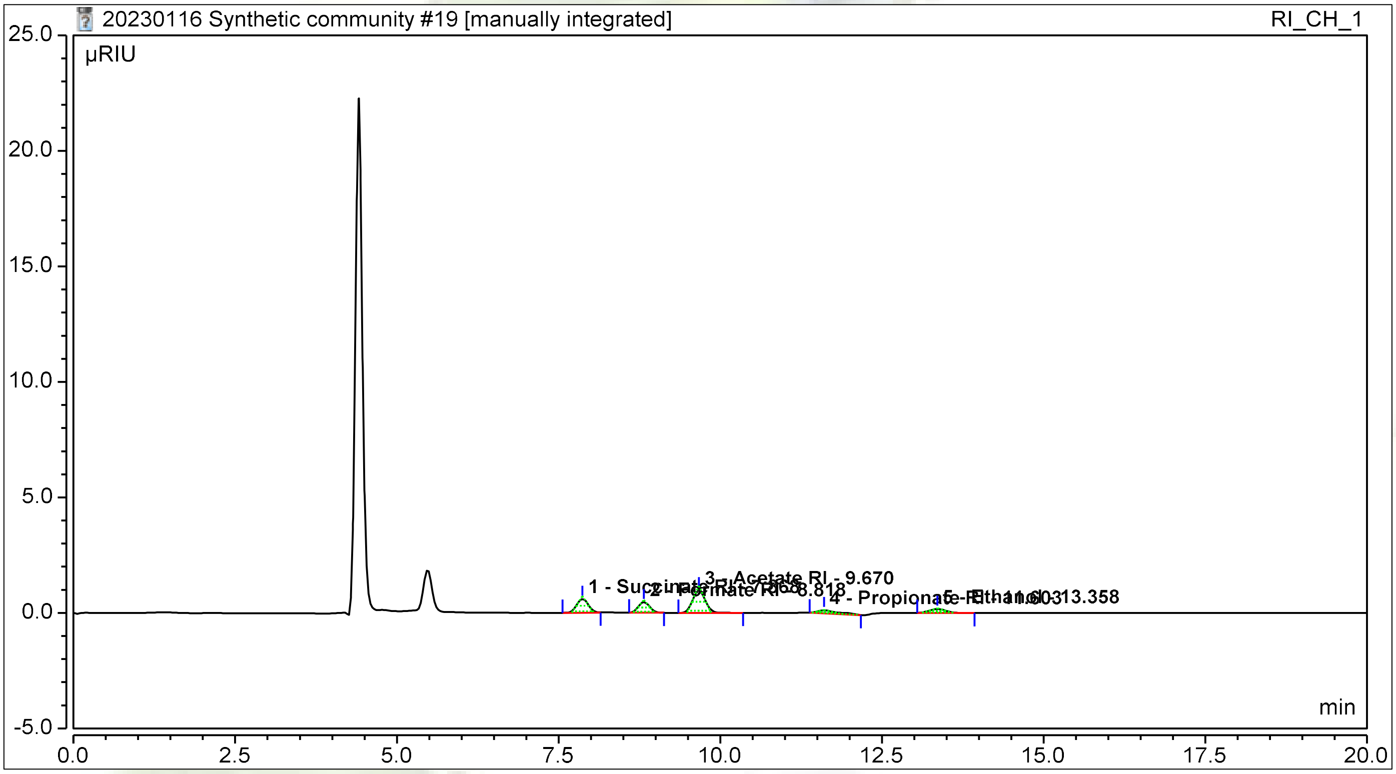

Peak Results

No.	Peak Name	Retention Time min	Width (50%) min	Type	Resolution (EP)	Asymmetry (EP)	Plates (EP)
n.a.	GlcNAc	n.a.	n.a.	n.a.	n.a.	n.a.	n.a.
n.a.	Citrate	n.a.	n.a.	n.a.	n.a.	n.a.	n.a.
n.a.	Glucose	n.a.	n.a.	n.a.	n.a.	n.a.	n.a.
n.a.	Galactose	n.a.	n.a.	n.a.	n.a.	n.a.	n.a.
n.a.	Fucose	n.a.	n.a.	n.a.	n.a.	n.a.	n.a.
1	Succinate RI	7,868	0,198	BMB	2,86	1,03	8775
n.a.	Lactate RI	n.a.	n.a.	n.a.	n.a.	n.a.	n.a.
n.a.	glycerol	n.a.	n.a.	n.a.	n.a.	n.a.	n.a.
2	Formate RI	8,818	0,194	BMB*	2,44	1,06	11465
3	Acetate RI	9,670	0,219	BMB	4,32	1,05	10837
n.a.	1,2 PDO RI	n.a.	n.a.	n.a.	n.a.	n.a.	n.a.
n.a.	1,3-PDO	n.a.	n.a.	n.a.	n.a.	n.a.	n.a.
4	Propionate RI	11,603	0,309	BMB*	3,49	1,83	7820
n.a.	1,3-PDO	n.a.	n.a.	n.a.	n.a.	n.a.	n.a.
n.a.	2-3 BDO	n.a.	n.a.	n.a.	n.a.	n.a.	n.a.
5	Ethanol	13,358	0,285	BMB*	n.a.	1,05	12192
n.a.	Isobutyrate RI	n.a.	n.a.	n.a.	n.a.	n.a.	n.a.
n.a.	Butyrate RI	n.a.	n.a.	n.a.	n.a.	n.a.	n.a.

Chromeleon (c) Dionex
Version 7.2.10.25868

Chromatogram and SST Results

Injection Details

Injection Name:	**GOSFOS t96 r3**	*Run Time (min):*	**20,00**
Vial Number:	**3:12**	*Injection Volume:*	**10,00**
Injection Type:	**Unknown**	*Channel:*	**RI_CH_1**
Calibration Level:		*Wavelength:*	**n.a.**
Instrument Method:	**Default method LC2030C 45 gr 20 min**	*Bandwidth:*	**n.a.**
Processing Method:	**Processing Method LC2030 45 gr**	*Dilution Factor:*	**1,0000**
Injection Date/Time:	**16/Jan/23 21:20**	*Sample Weight:*	**1,0000**

Chromatogram

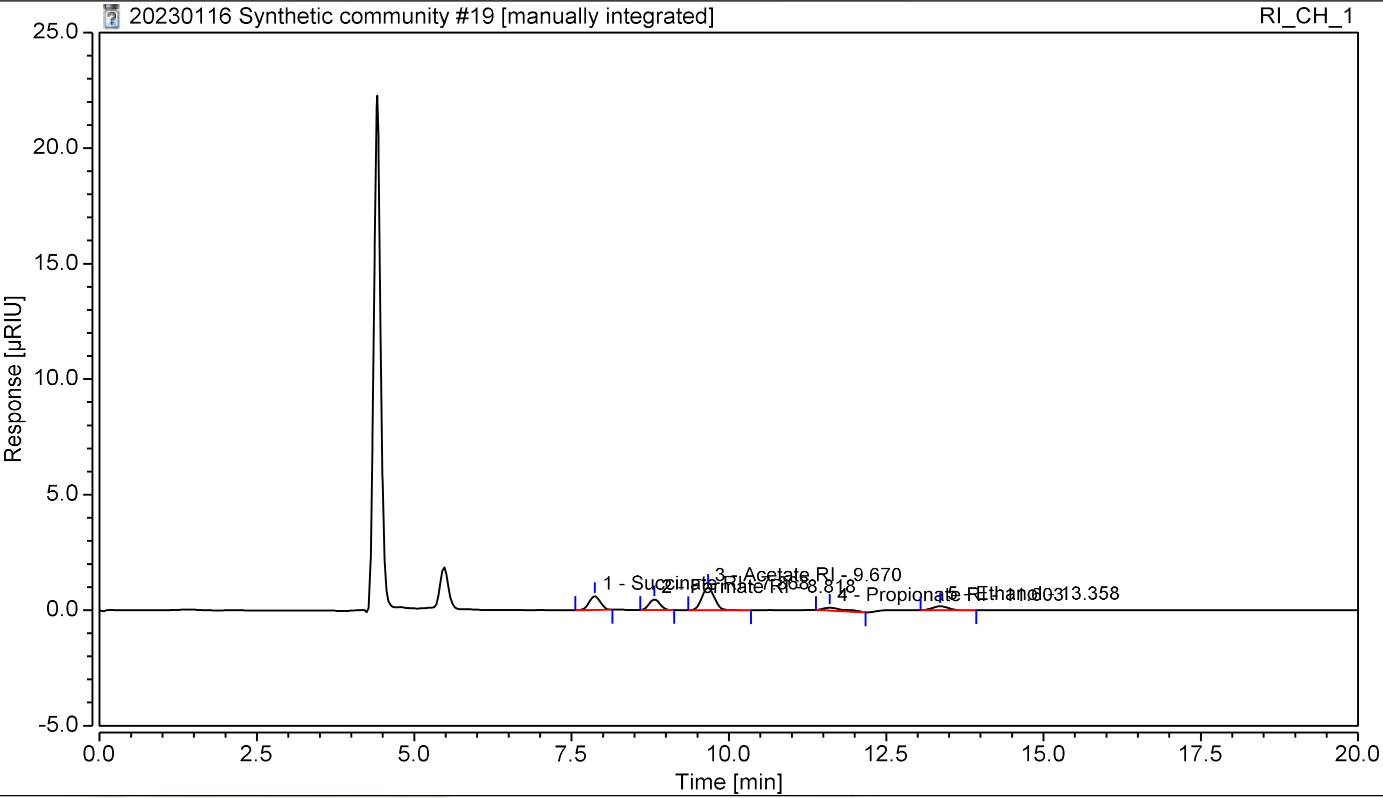

SST Results

No.	Name	Inj.Condition	Peak	Test Result	Injection
**Number of executed test cases:**	**n.a.**		**Total Result:**	**Passed**	

Chromatogram and Results

Injection Details

Injection Name:	**GOSFOS t120 r1**	*Run Time (min):*	**20,00**
Vial Number:	**3:13**	*Injection Volume:*	**10,00**
Injection Type:	**Unknown**	*Channel:*	**RI_CH_1**
Calibration Level:		*Wavelength:*	**n.a.**
Instrument Method:	**Default method LC2030C 45 gr 20 min**	*Bandwidth:*	**n.a.**
Processing Method:	**Processing Method LC2030 45 gr**	*Dilution Factor:*	**1,0000**
Injection Date/Time:	**16/Jan/23 21:40**	*Sample Weight:*	**1,0000**

Chromatogram

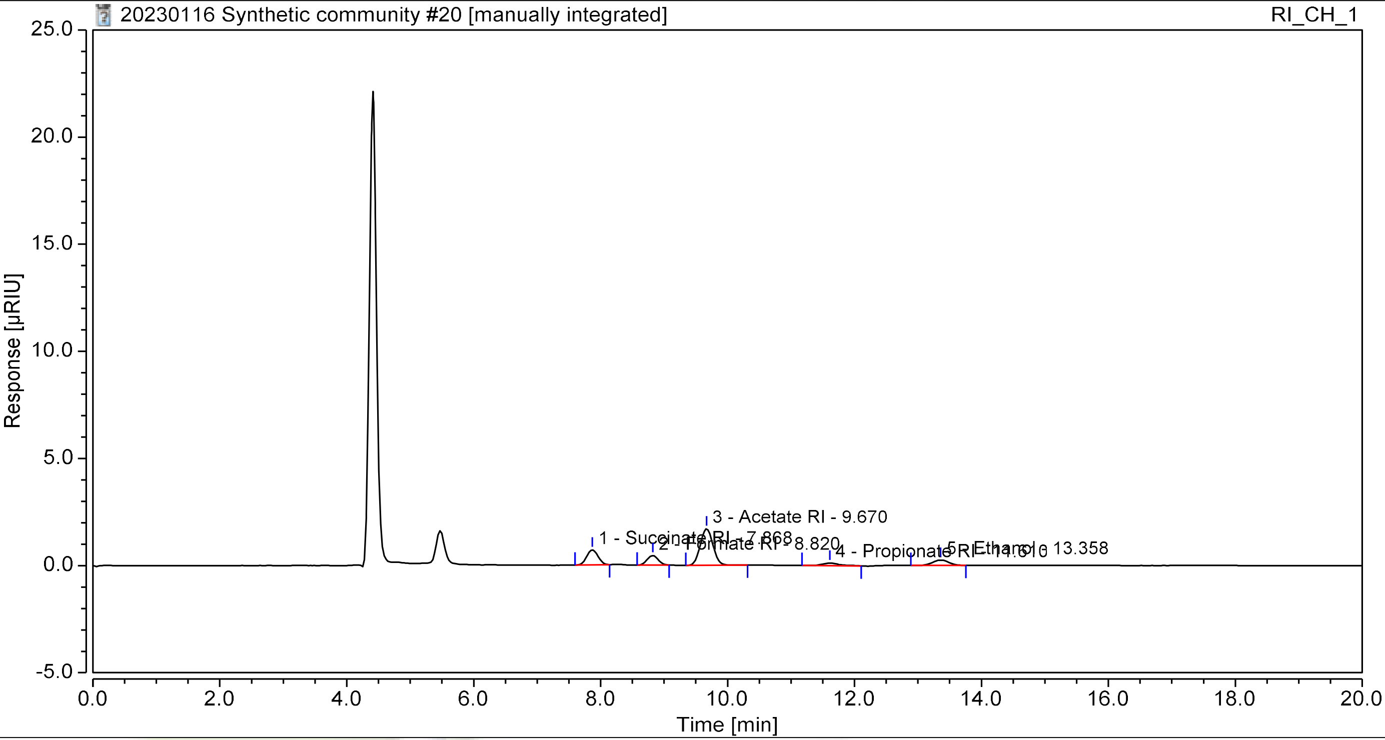

Integration Results

No.	Peak Name	Retention Time min	Area µRIU*min	Height µRIU	Relative Area %	Relative Height %	Amount
n.a.	GlcNAc	n.a.	n.a.	n.a.	n.a.	n.a.	n.a.
n.a.	Citrate	n.a.	n.a.	n.a.	n.a.	n.a.	n.a.
n.a.	Glucose	n.a.	n.a.	n.a.	n.a.	n.a.	n.a.
n.a.	Galactose	n.a.	n.a.	n.a.	n.a.	n.a.	n.a.
n.a.	Fucose	n.a.	n.a.	n.a.	n.a.	n.a.	n.a.
1	Succinate RI	7,868	0,145	0,701	19,55	21,82	3,0010
n.a.	Lactate RI	n.a.	n.a.	n.a.	n.a.	n.a.	n.a.
n.a.	glycerol	n.a.	n.a.	n.a.	n.a.	n.a.	n.a.
2	Formate RI	8,820	0,090	0,444	12,16	13,82	9,2536
3	Acetate RI	9,670	0,396	1,698	53,19	52,82	24,5323
n.a.	1,2 PDO RI	n.a.	n.a.	n.a.	n.a.	n.a.	n.a.
n.a.	1,3-PDO	n.a.	n.a.	n.a.	n.a.	n.a.	n.a.
4	Propionate RI	11,610	0,037	0,121	4,97	3,75	1,5453
n.a.	1,3-PDO	n.a.	n.a.	n.a.	n.a.	n.a.	n.a.
n.a.	2-3 BDO	n.a.	n.a.	n.a.	n.a.	n.a.	n.a.
5	Ethanol	13,358	0,075	0,250	10,14	7,79	7,9083
n.a.	Isobutyrate RI	n.a.	n.a.	n.a.	n.a.	n.a.	n.a.
n.a.	Butyrate RI	n.a.	n.a.	n.a.	n.a.	n.a.	n.a.
**Total:**			**0,744**	**3,215**	**100,00**	**100,00**	

Peak Analysis

Injection Details

Injection Name:	**GOSFOS t120 r1**	*Run Time (min):*	**20,00**
Vial Number:	**3:13**	*Injection Volume:*	**10,00**
Injection Type:	**Unknown**	*Channel:*	**RI_CH_1**
Calibration Level:		*Wavelength:*	**n.a.**
Instrument Method:	**Default method LC2030C 45 gr 20 min**	*Bandwidth:*	**n.a.**
Processing Method:	**Processing Method LC2030 45 gr**	*Dilution Factor:*	**1,0000**
Injection Date/Time:	**16/Jan/23 21:40**	*Sample Weight:*	**1,0000**

Chromatogram

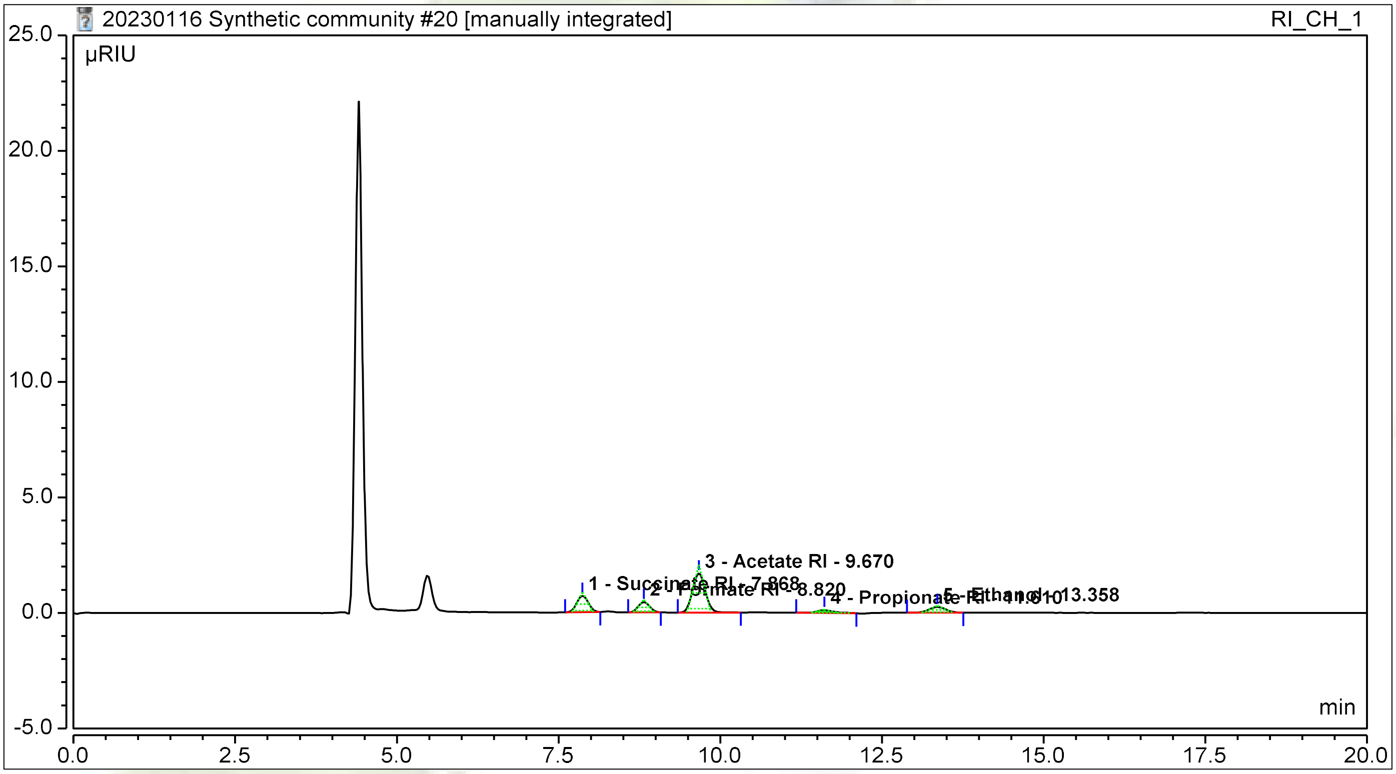

Peak Results

No.	Peak Name	Retention Time min	Width (50%) min	Type	Resolution (EP)	Asymmetry (EP)	Plates (EP)
n.a.	GlcNAc	n.a.	n.a.	n.a.	n.a.	n.a.	n.a.
n.a.	Citrate	n.a.	n.a.	n.a.	n.a.	n.a.	n.a.
n.a.	Glucose	n.a.	n.a.	n.a.	n.a.	n.a.	n.a.
n.a.	Galactose	n.a.	n.a.	n.a.	n.a.	n.a.	n.a.
n.a.	Fucose	n.a.	n.a.	n.a.	n.a.	n.a.	n.a.
1	Succinate RI	7,868	0,197	BMB	2,87	1,03	8799
n.a.	Lactate RI	n.a.	n.a.	n.a.	n.a.	n.a.	n.a.
n.a.	glycerol	n.a.	n.a.	n.a.	n.a.	n.a.	n.a.
2	Formate RI	8,820	0,194	BMB*	2,43	1,03	11499
3	Acetate RI	9,670	0,218	BMB	4,86	1,05	10868
n.a.	1,2 PDO RI	n.a.	n.a.	n.a.	n.a.	n.a.	n.a.
n.a.	1,3-PDO	n.a.	n.a.	n.a.	n.a.	n.a.	n.a.
4	Propionate RI	11,610	0,252	BMB*	3,83	1,13	11732
n.a.	1,3-PDO	n.a.	n.a.	n.a.	n.a.	n.a.	n.a.
n.a.	2-3 BDO	n.a.	n.a.	n.a.	n.a.	n.a.	n.a.
5	Ethanol	13,358	0,286	BMB*	n.a.	1,04	12093
n.a.	Isobutyrate RI	n.a.	n.a.	n.a.	n.a.	n.a.	n.a.
n.a.	Butyrate RI	n.a.	n.a.	n.a.	n.a.	n.a.	n.a.

Chromatogram and SST Results

Injection Details

Injection Name:	**GOSFOS t120 r1**	*Run Time (min):*	**20,00**
Vial Number:	**3:13**	*Injection Volume:*	**10,00**
Injection Type:	**Unknown**	*Channel:*	**RI_CH_1**
Calibration Level:		*Wavelength:*	**n.a.**
Instrument Method:	**Default method LC2030C 45 gr 20 min**	*Bandwidth:*	**n.a.**
Processing Method:	**Processing Method LC2030 45 gr**	*Dilution Factor:*	**1,0000**
Injection Date/Time:	**16/Jan/23 21:40**	*Sample Weight:*	**1,0000**

Chromatogram

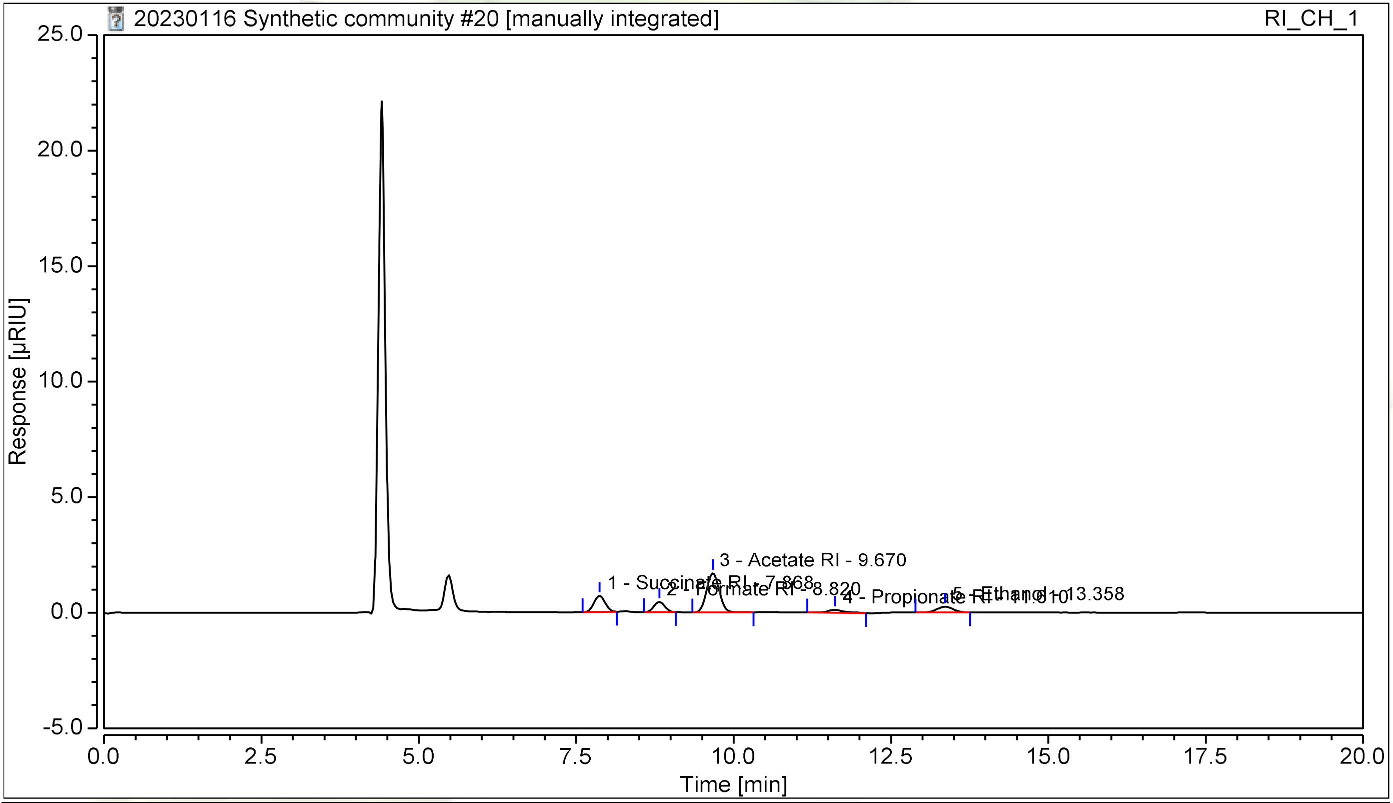

SST Results

No.	Name	Inj.Condition	Peak	Test Result	Injection

Number of executed test cases: **n.a.** **Total Result:** **Passed**

Chromatogram and Results

Injection Details

Injection Name:	**GOSFOS t120 r2**	*Run Time (min):*	**20,00**
Vial Number:	**3:14**	*Injection Volume:*	**10,00**
Injection Type:	**Unknown**	*Channel:*	**RI_CH_1**
Calibration Level:		*Wavelength:*	**n.a.**
Instrument Method:	**Default method LC2030C 45 gr 20 min**	*Bandwidth:*	**n.a.**
Processing Method:	**Processing Method LC2030 45 gr**	*Dilution Factor:*	**1,0000**
Injection Date/Time:	**16/Jan/23 22:01**	*Sample Weight:*	**1,0000**

Chromatogram

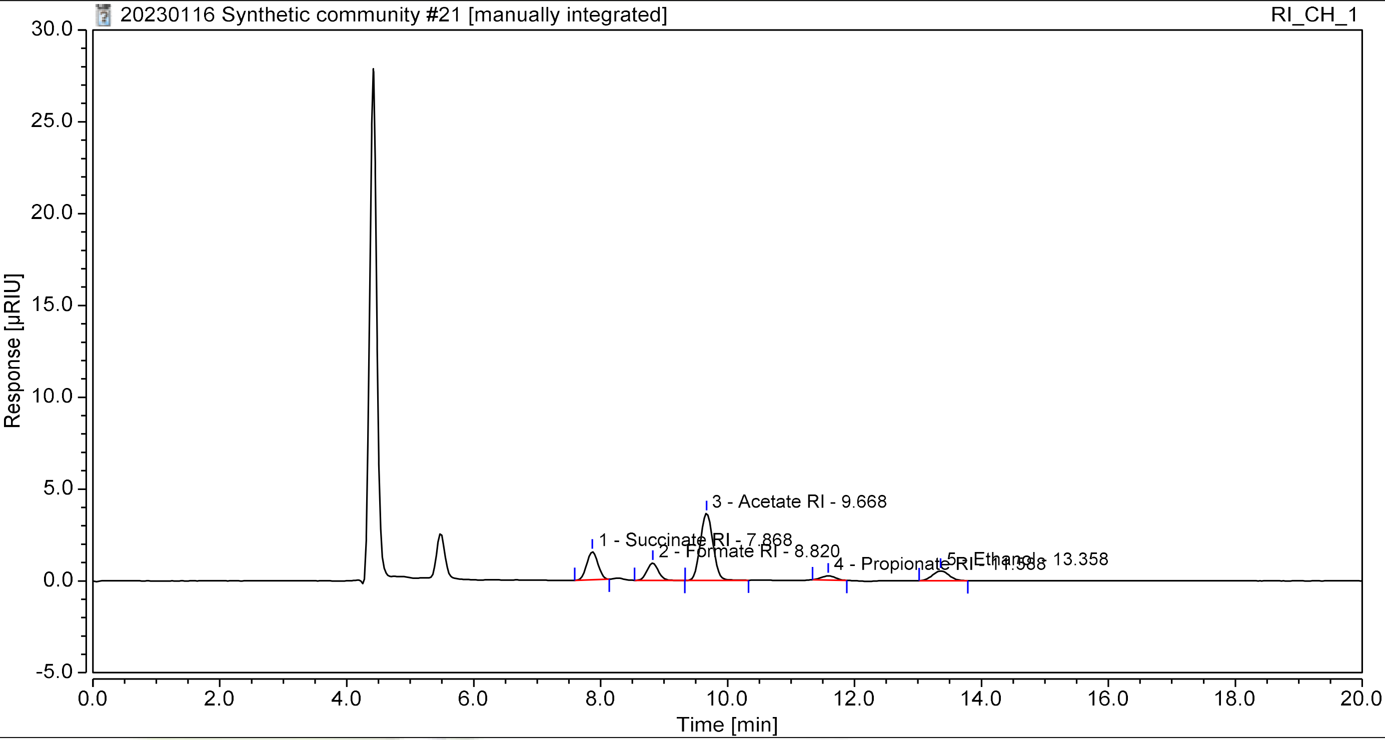

Integration Results

No.	Peak Name	Retention Time min	Area µRIU*min	Height µRIU	Relative Area %	Relative Height %	Amount
n.a.	GlcNAc	n.a.	n.a.	n.a.	n.a.	n.a.	n.a.
n.a.	Citrate	n.a.	n.a.	n.a.	n.a.	n.a.	n.a.
n.a.	Glucose	n.a.	n.a.	n.a.	n.a.	n.a.	n.a.
n.a.	Galactose	n.a.	n.a.	n.a.	n.a.	n.a.	n.a.
n.a.	Fucose	n.a.	n.a.	n.a.	n.a.	n.a.	n.a.
1	Succinate RI	7,868	0,313	1,517	19,91	22,16	6,4524
n.a.	Lactate RI	n.a.	n.a.	n.a.	n.a.	n.a.	n.a.
n.a.	glycerol	n.a.	n.a.	n.a.	n.a.	n.a.	n.a.
2	Formate RI	8,820	0,195	0,937	12,43	13,68	19,9589
3	Acetate RI	9,668	0,848	3,644	54,01	53,22	52,5682
n.a.	1,2 PDO RI	n.a.	n.a.	n.a.	n.a.	n.a.	n.a.
n.a.	1,3-PDO	n.a.	n.a.	n.a.	n.a.	n.a.	n.a.
4	Propionate RI	11,588	0,057	0,224	3,64	3,27	2,3870
n.a.	1,3-PDO	n.a.	n.a.	n.a.	n.a.	n.a.	n.a.
n.a.	2-3 BDO	n.a.	n.a.	n.a.	n.a.	n.a.	n.a.
5	Ethanol	13,358	0,157	0,526	10,02	7,68	16,4918
n.a.	Isobutyrate RI	n.a.	n.a.	n.a.	n.a.	n.a.	n.a.
n.a.	Butyrate RI	n.a.	n.a.	n.a.	n.a.	n.a.	n.a.
**Total:**			**1,570**	**6,847**	**100,00**	**100,00**	

Peak Analysis

Injection Details

Injection Name:	**GOSFOS t120 r2**	*Run Time (min):*	**20,00**
Vial Number:	**3:14**	*Injection Volume:*	**10,00**
Injection Type:	**Unknown**	*Channel:*	**RI_CH_1**
Calibration Level:		*Wavelength:*	**n.a.**
Instrument Method:	**Default method LC2030C 45 gr 20 min**	*Bandwidth:*	**n.a.**
Processing Method:	**Processing Method LC2030 45 gr**	*Dilution Factor:*	**1,0000**
Injection Date/Time:	**16/Jan/23 22:01**	*Sample Weight:*	**1,0000**

Chromatogram

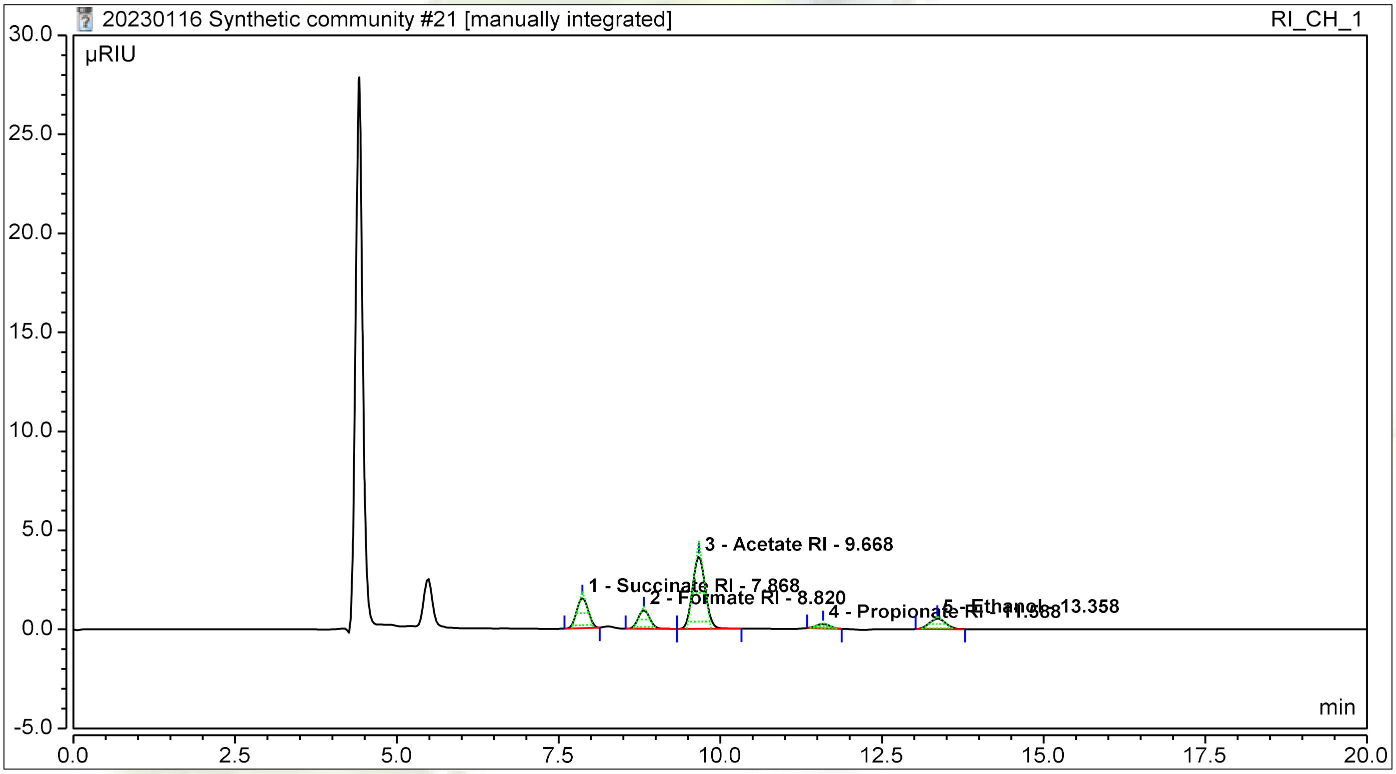

Peak Results

No.	Peak Name	Retention Time min	Width (50%) min	Type	Resolution (EP)	Asymmetry (EP)	Plates (EP)
n.a.	GlcNAc	n.a.	n.a.	n.a.	n.a.	n.a.	n.a.
n.a.	Citrate	n.a.	n.a.	n.a.	n.a.	n.a.	n.a.
n.a.	Glucose	n.a.	n.a.	n.a.	n.a.	n.a.	n.a.
n.a.	Galactose	n.a.	n.a.	n.a.	n.a.	n.a.	n.a.
n.a.	Fucose	n.a.	n.a.	n.a.	n.a.	n.a.	n.a.
1	Succinate RI	7,868	0,197	BMB	2,87	1,03	8867
n.a.	Lactate RI	n.a.	n.a.	n.a.	n.a.	n.a.	n.a.
n.a.	glycerol	n.a.	n.a.	n.a.	n.a.	n.a.	n.a.
2	Formate RI	8,820	0,195	BMB	2,42	1,06	11340
3	Acetate RI	9,668	0,218	BMB	4,87	1,06	10882
n.a.	1,2 PDO RI	n.a.	n.a.	n.a.	n.a.	n.a.	n.a.
n.a.	1,3-PDO	n.a.	n.a.	n.a.	n.a.	n.a.	n.a.
4	Propionate RI	11,588	0,247	BMB*	3,93	1,03	12173
n.a.	1,3-PDO	n.a.	n.a.	n.a.	n.a.	n.a.	n.a.
n.a.	2-3 BDO	n.a.	n.a.	n.a.	n.a.	n.a.	n.a.
5	Ethanol	13,358	0,285	BMB*	n.a.	1,04	12208
n.a.	Isobutyrate RI	n.a.	n.a.	n.a.	n.a.	n.a.	n.a.
n.a.	Butyrate RI	n.a.	n.a.	n.a.	n.a.	n.a.	n.a.

Chromeleon (c) Dionex
Version 7.2.10.25868

Chromatogram and SST Results

Injection Details

Injection Name:	**GOSFOS t120 r2**	*Run Time (min):*	**20,00**
Vial Number:	**3:14**	*Injection Volume:*	**10,00**
Injection Type:	**Unknown**	*Channel:*	**RI_CH_1**
Calibration Level:		*Wavelength:*	**n.a.**
Instrument Method:	**Default method LC2030C 45 gr 20 min**	*Bandwidth:*	**n.a.**
Processing Method:	**Processing Method LC2030 45 gr**	*Dilution Factor:*	**1,0000**
Injection Date/Time:	**16/Jan/23 22:01**	*Sample Weight:*	**1,0000**

Chromatogram

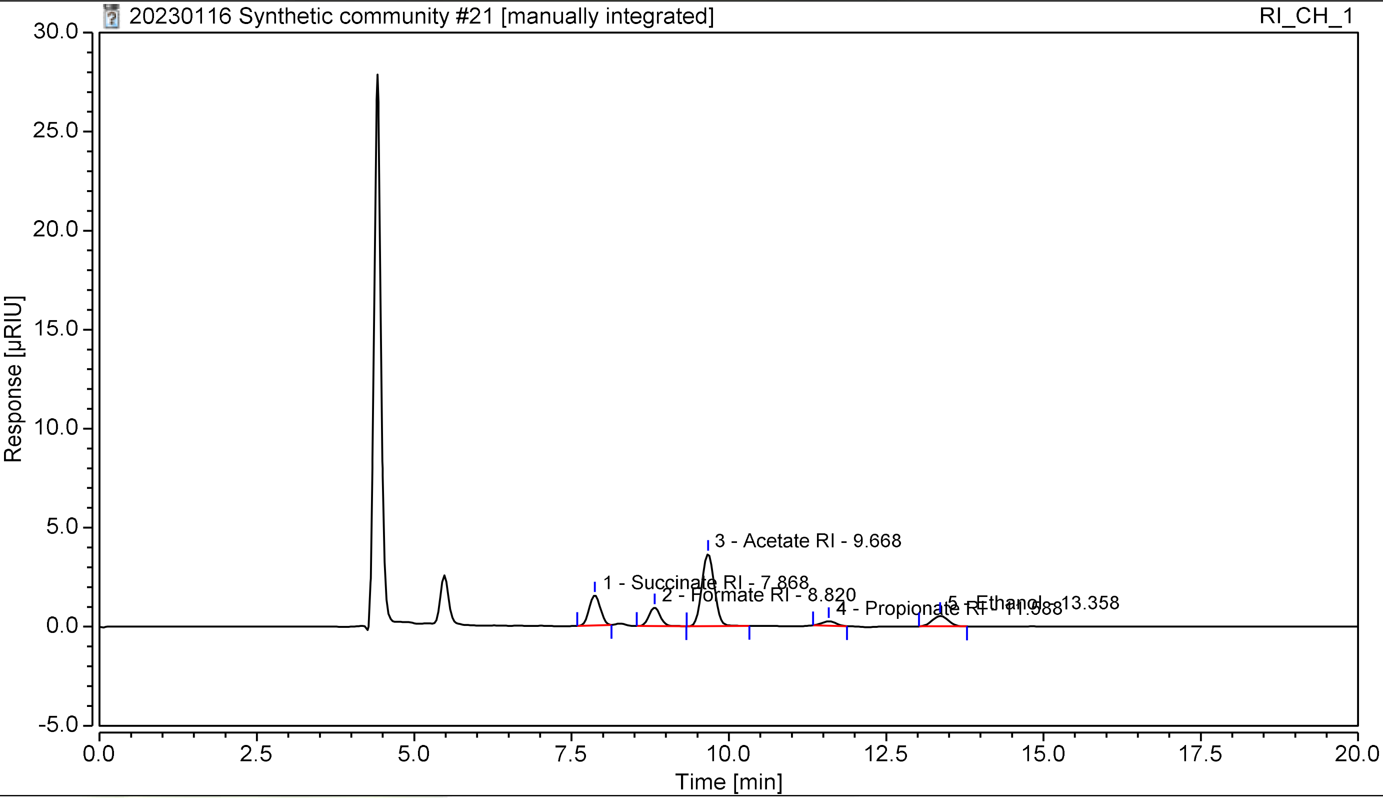

SST Results

No.	Name	Inj.Condition	Peak	Test Result	Injection

Number of executed test cases: **n.a.** **Total Result:** **Passed**

Chromatogram and Results

Injection Details

Injection Name:	**GOSFOS t120 r3**	*Run Time (min):*	**20,00**
Vial Number:	**3:15**	*Injection Volume:*	**10,00**
Injection Type:	**Unknown**	*Channel:*	**RI_CH_1**
Calibration Level:		*Wavelength:*	**n.a.**
Instrument Method:	**Default method LC2030C 45 gr 20 min**	*Bandwidth:*	**n.a.**
Processing Method:	**Processing Method LC2030 45 gr**	*Dilution Factor:*	**1,0000**
Injection Date/Time:	**16/Jan/23 22:21**	*Sample Weight:*	**1,0000**

Chromatogram

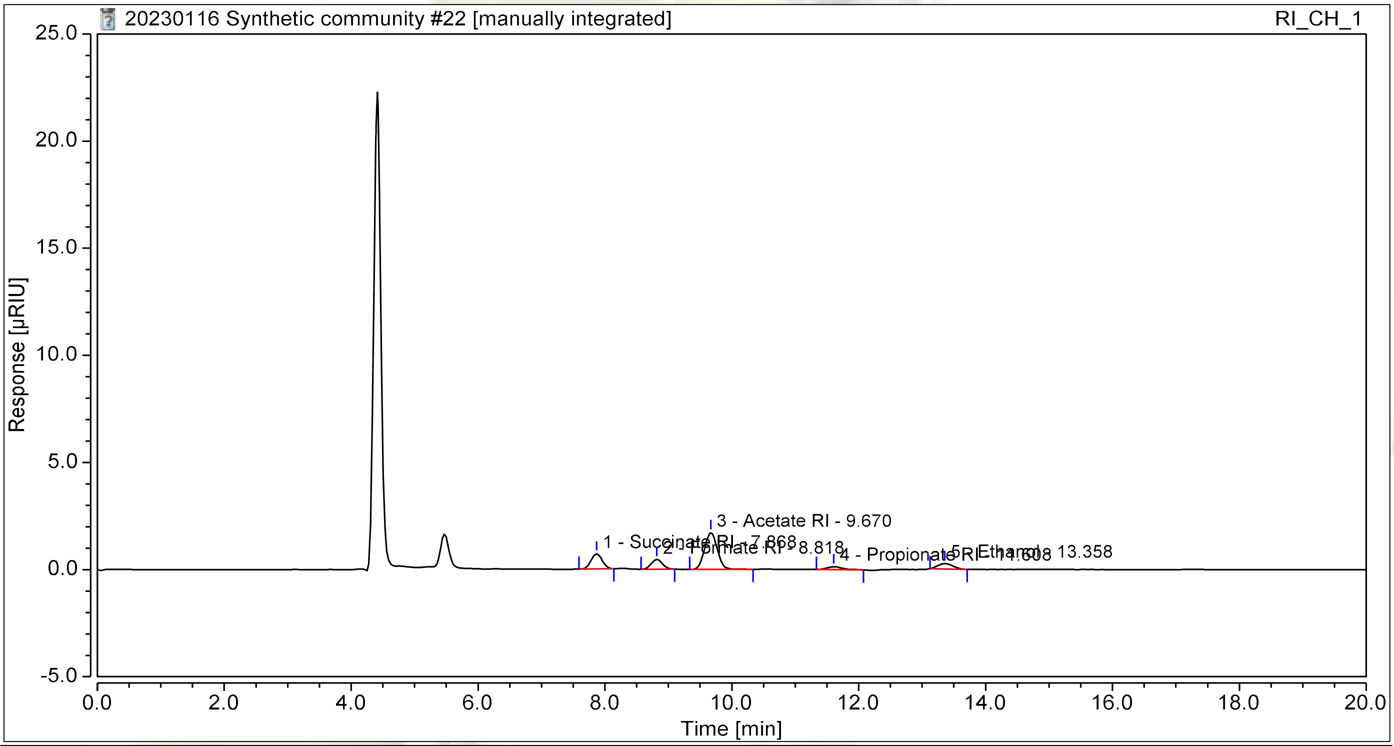

Integration Results

No.	Peak Name	Retention Time min	Area µRIU*min	Height µRIU	Relative Area %	Relative Height %	Amount
n.a.	GlcNAc	n.a.	n.a.	n.a.	n.a.	n.a.	n.a.
n.a.	Citrate	n.a.	n.a.	n.a.	n.a.	n.a.	n.a.
n.a.	Glucose	n.a.	n.a.	n.a.	n.a.	n.a.	n.a.
n.a.	Galactose	n.a.	n.a.	n.a.	n.a.	n.a.	n.a.
n.a.	Fucose	n.a.	n.a.	n.a.	n.a.	n.a.	n.a.
1	Succinate RI	7,868	0,146	0,706	19,68	21,70	3,0205
n.a.	Lactate RI	n.a.	n.a.	n.a.	n.a.	n.a.	n.a.
n.a.	glycerol	n.a.	n.a.	n.a.	n.a.	n.a.	n.a.
2	Formate RI	8,818	0,093	0,456	12,55	14,02	9,5514
3	Acetate RI	9,670	0,398	1,709	53,50	52,52	24,6748
n.a.	1,2 PDO RI	n.a.	n.a.	n.a.	n.a.	n.a.	n.a.
n.a.	1,3-PDO	n.a.	n.a.	n.a.	n.a.	n.a.	n.a.
4	Propionate RI	11,608	0,036	0,129	4,84	3,97	1,5062
n.a.	1,3-PDO	n.a.	n.a.	n.a.	n.a.	n.a.	n.a.
n.a.	2-3 BDO	n.a.	n.a.	n.a.	n.a.	n.a.	n.a.
5	Ethanol	13,358	0,070	0,254	9,43	7,79	7,3549
n.a.	Isobutyrate RI	n.a.	n.a.	n.a.	n.a.	n.a.	n.a.
n.a.	Butyrate RI	n.a.	n.a.	n.a.	n.a.	n.a.	n.a.
**Total:**			**0,744**	**3,255**	**100,00**	**100,00**	

Chromeleon (c) Dionex
Version 7.2.10.25868

Peak Analysis

Injection Details

Injection Name:	**GOSFOS t120 r3**	*Run Time (min):*	**20,00**
Vial Number:	**3:15**	*Injection Volume:*	**10,00**
Injection Type:	**Unknown**	*Channel:*	**RI_CH_1**
Calibration Level:		*Wavelength:*	**n.a.**
Instrument Method:	**Default method LC2030C 45 gr 20 min**	*Bandwidth:*	**n.a.**
Processing Method:	**Processing Method LC2030 45 gr**	*Dilution Factor:*	**1,0000**
Injection Date/Time:	**16/Jan/23 22:21**	*Sample Weight:*	**1,0000**

Chromatogram

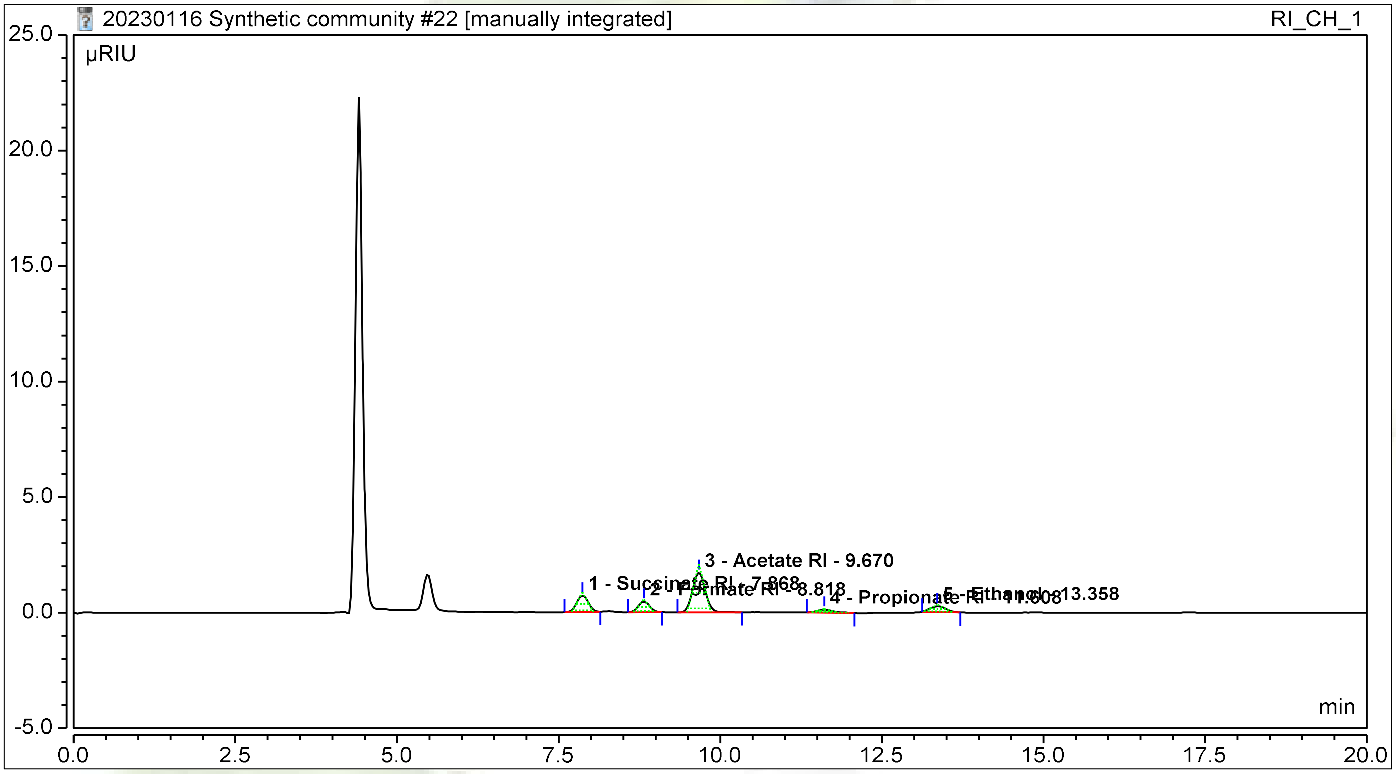

Peak Results

No.	Peak Name	Retention Time min	Width (50%) min	Type	Resolution (EP)	Asymmetry (EP)	Plates (EP)
n.a.	GlcNAc	n.a.	n.a.	n.a.	n.a.	n.a.	n.a.
n.a.	Citrate	n.a.	n.a.	n.a.	n.a.	n.a.	n.a.
n.a.	Glucose	n.a.	n.a.	n.a.	n.a.	n.a.	n.a.
n.a.	Galactose	n.a.	n.a.	n.a.	n.a.	n.a.	n.a.
n.a.	Fucose	n.a.	n.a.	n.a.	n.a.	n.a.	n.a.
1	Succinate RI	7,868	0,197	BMB	2,86	1,03	8798
n.a.	Lactate RI	n.a.	n.a.	n.a.	n.a.	n.a.	n.a.
n.a.	glycerol	n.a.	n.a.	n.a.	n.a.	n.a.	n.a.
2	Formate RI	8,818	0,194	BMB*	2,44	1,04	11446
3	Acetate RI	9,670	0,218	BMB	4,93	1,05	10871
n.a.	1,2 PDO RI	n.a.	n.a.	n.a.	n.a.	n.a.	n.a.
n.a.	1,3-PDO	n.a.	n.a.	n.a.	n.a.	n.a.	n.a.
4	Propionate RI	11,608	0,246	BMB*	4,01	1,49	12362
n.a.	1,3-PDO	n.a.	n.a.	n.a.	n.a.	n.a.	n.a.
n.a.	2-3 BDO	n.a.	n.a.	n.a.	n.a.	n.a.	n.a.
5	Ethanol	13,358	0,270	BMB*	n.a.	1,14	13607
n.a.	Isobutyrate RI	n.a.	n.a.	n.a.	n.a.	n.a.	n.a.
n.a.	Butyrate RI	n.a.	n.a.	n.a.	n.a.	n.a.	n.a.

Chromeleon (c) Dionex
Version 7.2.10.25868

Chromatogram and SST Results

Injection Details

Injection Name:	**GOSFOS t120 r3**	*Run Time (min):*	**20,00**
Vial Number:	**3:15**	*Injection Volume:*	**10,00**
Injection Type:	**Unknown**	*Channel:*	**RI_CH_1**
Calibration Level:		*Wavelength:*	**n.a.**
Instrument Method:	**Default method LC2030C 45 gr 20 min**	*Bandwidth:*	**n.a.**
Processing Method:	**Processing Method LC2030 45 gr**	*Dilution Factor:*	**1,0000**
Injection Date/Time:	**16/Jan/23 22:21**	*Sample Weight:*	**1,0000**

Chromatogram

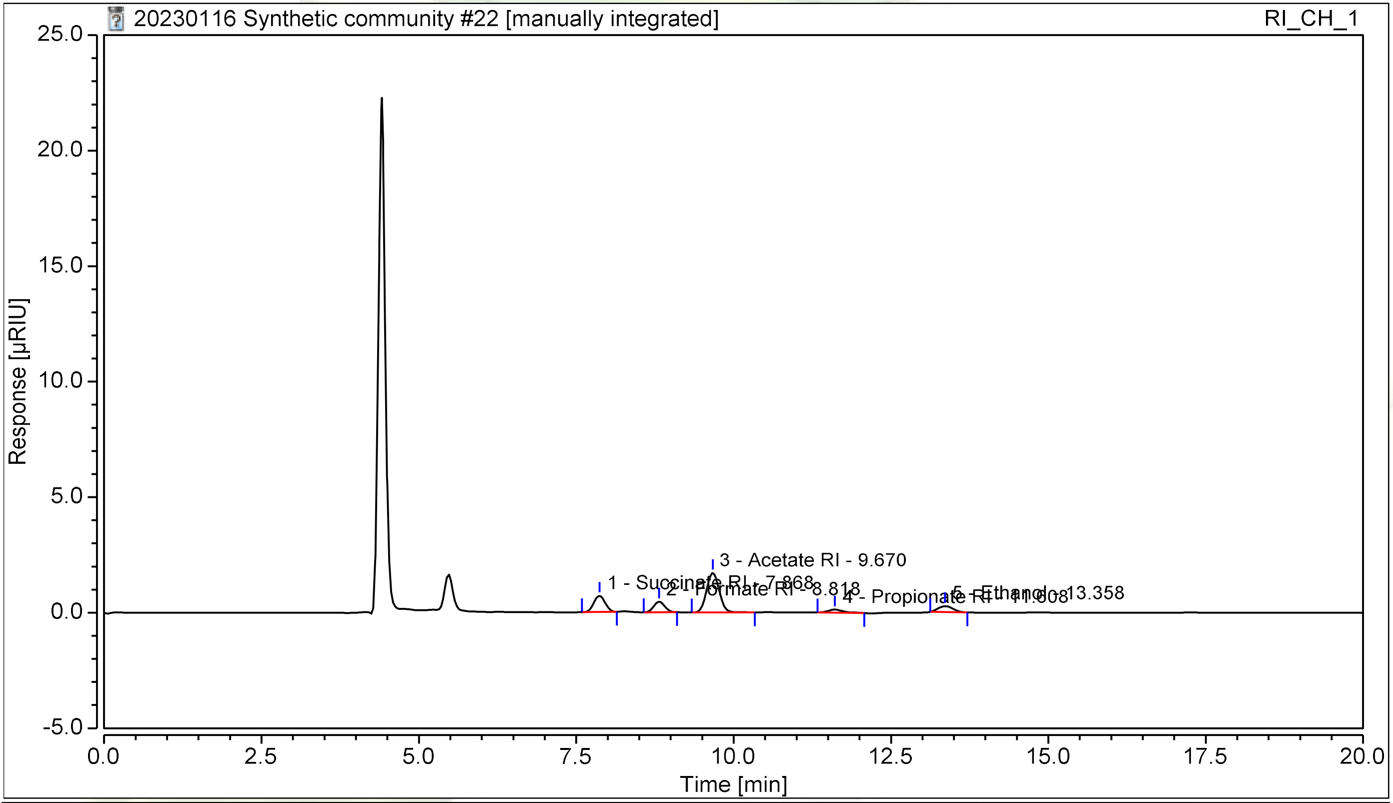

SST Results

No.	Name	Inj.Condition	Peak	Test Result	Injection

Number of executed test cases: **n.a.**   **Total Result:** **Passed**

Chromatogram and Results

Injection Details

Injection Name:	**GOSFOSEXTR t72 r1**	*Run Time (min):*	**20,00**
Vial Number:	**3:16**	*Injection Volume:*	**10,00**
Injection Type:	**Unknown**	*Channel:*	**RI_CH_1**
Calibration Level:		*Wavelength:*	**n.a.**
Instrument Method:	**Default method LC2030C 45 gr 20 min**	*Bandwidth:*	**n.a.**
Processing Method:	**Processing Method LC2030 45 gr**	*Dilution Factor:*	**1,0000**
Injection Date/Time:	**16/Jan/23 22:42**	*Sample Weight:*	**1,0000**

Chromatogram

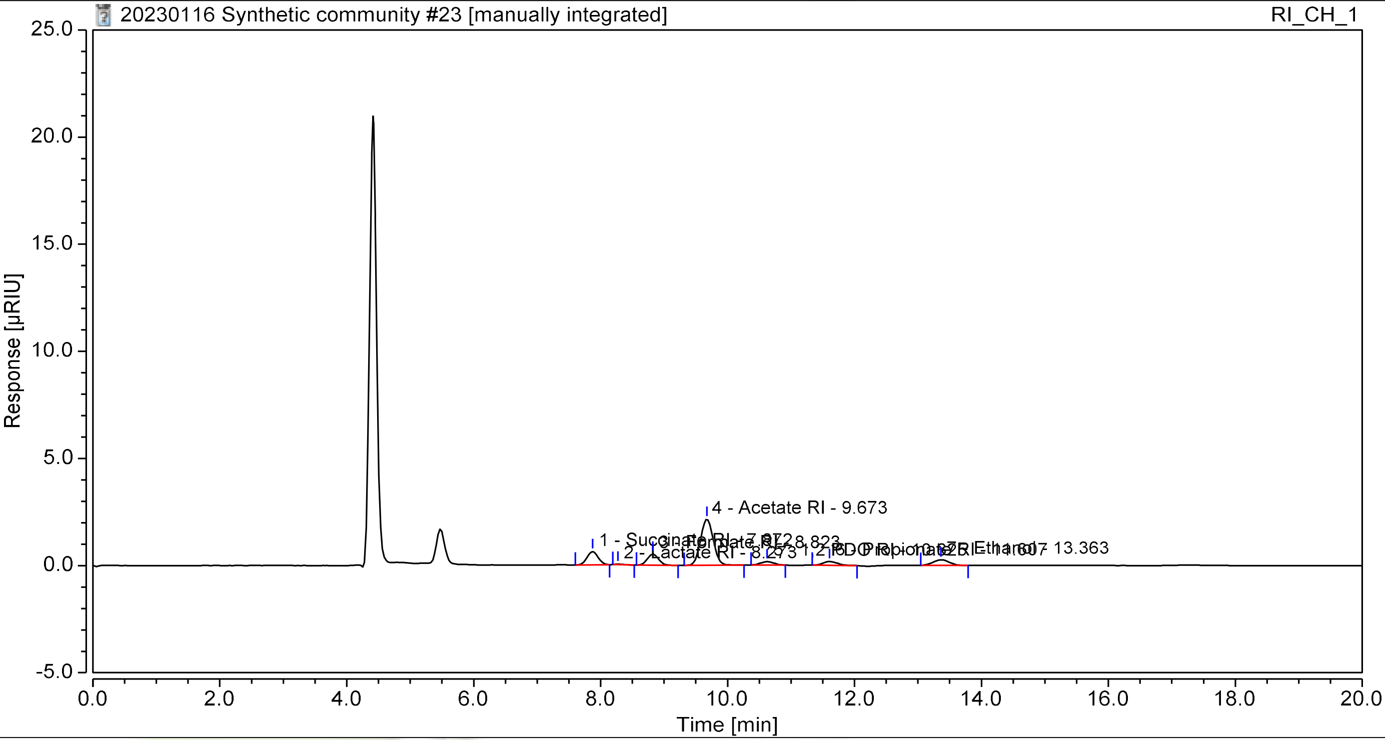

Integration Results

No.	Peak Name	Retention Time min	Area µRIU*min	Height µRIU	Relative Area %	Relative Height %	Amount
n.a.	GlcNAc	n.a.	n.a.	n.a.	n.a.	n.a.	n.a.
n.a.	Citrate	n.a.	n.a.	n.a.	n.a.	n.a.	n.a.
n.a.	Glucose	n.a.	n.a.	n.a.	n.a.	n.a.	n.a.
n.a.	Galactose	n.a.	n.a.	n.a.	n.a.	n.a.	n.a.
n.a.	Fucose	n.a.	n.a.	n.a.	n.a.	n.a.	n.a.
1	Succinate RI	7,872	0,126	0,613	14,06	15,79	2,6021
2	Lactate RI	8,273	0,003	0,021	0,34	0,54	0,0900
n.a.	glycerol	n.a.	n.a.	n.a.	n.a.	n.a.	n.a.
3	Formate RI	8,823	0,105	0,513	11,76	13,22	10,7846
4	Acetate RI	9,673	0,498	2,140	55,52	55,10	30,8602
5	1,2 PDO RI	10,625	0,039	0,159	4,40	4,09	1,2016
n.a.	1,3-PDO	n.a.	n.a.	n.a.	n.a.	n.a.	n.a.
6	Propionate RI	11,607	0,047	0,178	5,25	4,57	1,9690
n.a.	1,3-PDO	n.a.	n.a.	n.a.	n.a.	n.a.	n.a.
n.a.	2-3 BDO	n.a.	n.a.	n.a.	n.a.	n.a.	n.a.
7	Ethanol	13,363	0,078	0,260	8,66	6,69	8,1376
n.a.	Isobutyrate RI	n.a.	n.a.	n.a.	n.a.	n.a.	n.a.
n.a.	Butyrate RI	n.a.	n.a.	n.a.	n.a.	n.a.	n.a.
**Total:**			**0,896**	**3,884**	**100,00**	**100,00**	

Peak Analysis

Injection Details

Injection Name:	**GOSFOSEXTR t72 r1**	*Run Time (min):*	**20,00**
Vial Number:	**3:16**	*Injection Volume:*	**10,00**
Injection Type:	**Unknown**	*Channel:*	**RI_CH_1**
Calibration Level:		*Wavelength:*	**n.a.**
Instrument Method:	**Default method LC2030C 45 gr 20 min**	*Bandwidth:*	**n.a.**
Processing Method:	**Processing Method LC2030 45 gr**	*Dilution Factor:*	**1,0000**
Injection Date/Time:	**16/Jan/23 22:42**	*Sample Weight:*	**1,0000**

Chromatogram

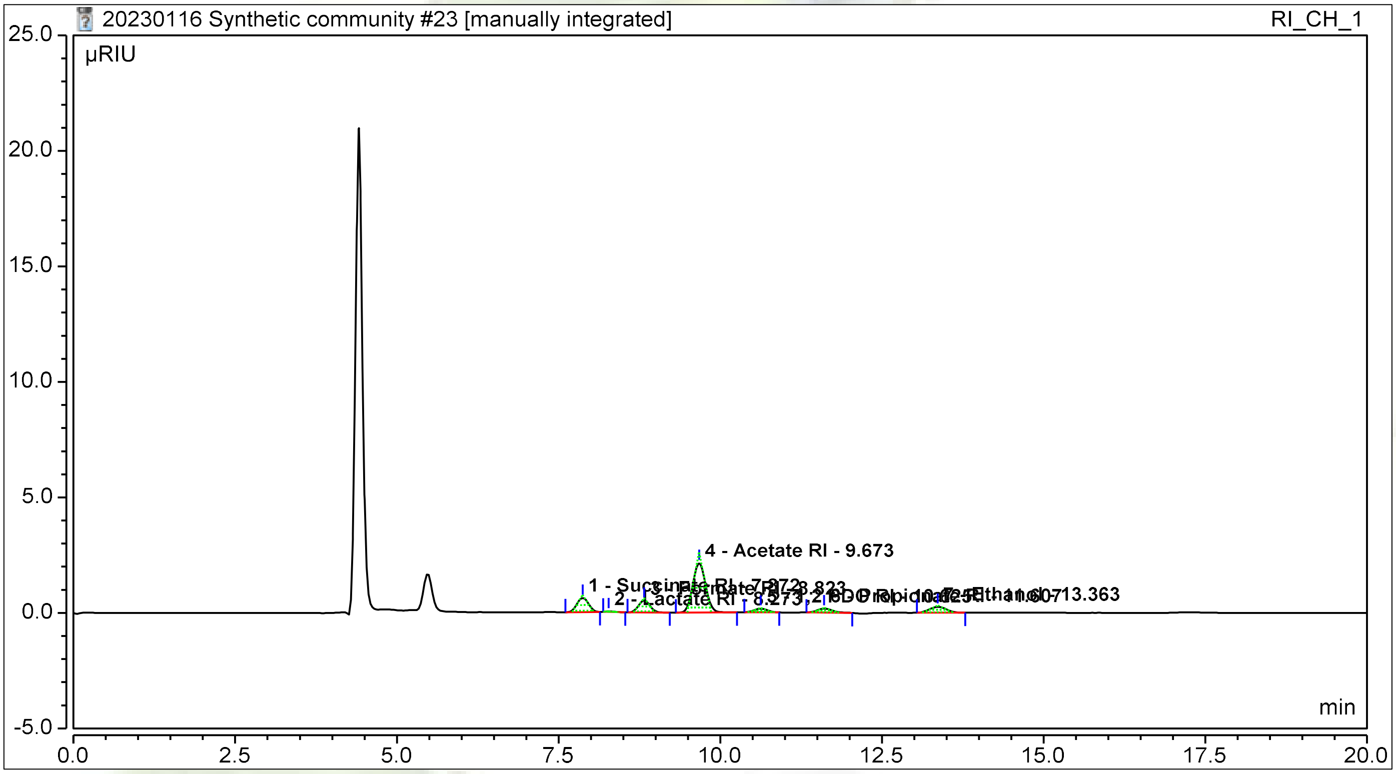

Peak Results

No.	Peak Name	Retention Time min	Width (50%) min	Type	Resolution (EP)	Asymmetry (EP)	Plates (EP)
n.a.	GlcNAc	n.a.	n.a.	n.a.	n.a.	n.a.	n.a.
n.a.	Citrate	n.a.	n.a.	n.a.	n.a.	n.a.	n.a.
n.a.	Glucose	n.a.	n.a.	n.a.	n.a.	n.a.	n.a.
n.a.	Galactose	n.a.	n.a.	n.a.	n.a.	n.a.	n.a.
n.a.	Fucose	n.a.	n.a.	n.a.	n.a.	n.a.	n.a.
1	Succinate RI	7,872	0,196	BMB	1,39	1,03	8911
2	Lactate RI	8,273	0,144	BMB*	1,92	1,62	18393
n.a.	glycerol	n.a.	n.a.	n.a.	n.a.	n.a.	n.a.
3	Formate RI	8,823	0,194	BMB	2,43	1,05	11444
4	Acetate RI	9,673	0,218	BMB	2,45	1,05	10868
5	1,2 PDO RI	10,625	0,241	BMB*	2,37	1,04	10778
n.a.	1,3-PDO	n.a.	n.a.	n.a.	n.a.	n.a.	n.a.
6	Propionate RI	11,607	0,247	BMB*	3,89	1,23	12196
n.a.	1,3-PDO	n.a.	n.a.	n.a.	n.a.	n.a.	n.a.
n.a.	2-3 BDO	n.a.	n.a.	n.a.	n.a.	n.a.	n.a.
7	Ethanol	13,363	0,285	BMB*	n.a.	1,05	12152
n.a.	Isobutyrate RI	n.a.	n.a.	n.a.	n.a.	n.a.	n.a.
n.a.	Butyrate RI	n.a.	n.a.	n.a.	n.a.	n.a.	n.a.

Chromatogram and SST Results

Injection Details

Injection Name:	**GOSFOSEXTR t72 r1**	*Run Time (min):*	**20,00**
Vial Number:	**3:16**	*Injection Volume:*	**10,00**
Injection Type:	**Unknown**	*Channel:*	**RI_CH_1**
Calibration Level:		*Wavelength:*	**n.a.**
Instrument Method:	**Default method LC2030C 45 gr 20 min**	*Bandwidth:*	**n.a.**
Processing Method:	**Processing Method LC2030 45 gr**	*Dilution Factor:*	**1,0000**
Injection Date/Time:	**16/Jan/23 22:42**	*Sample Weight:*	**1,0000**

Chromatogram

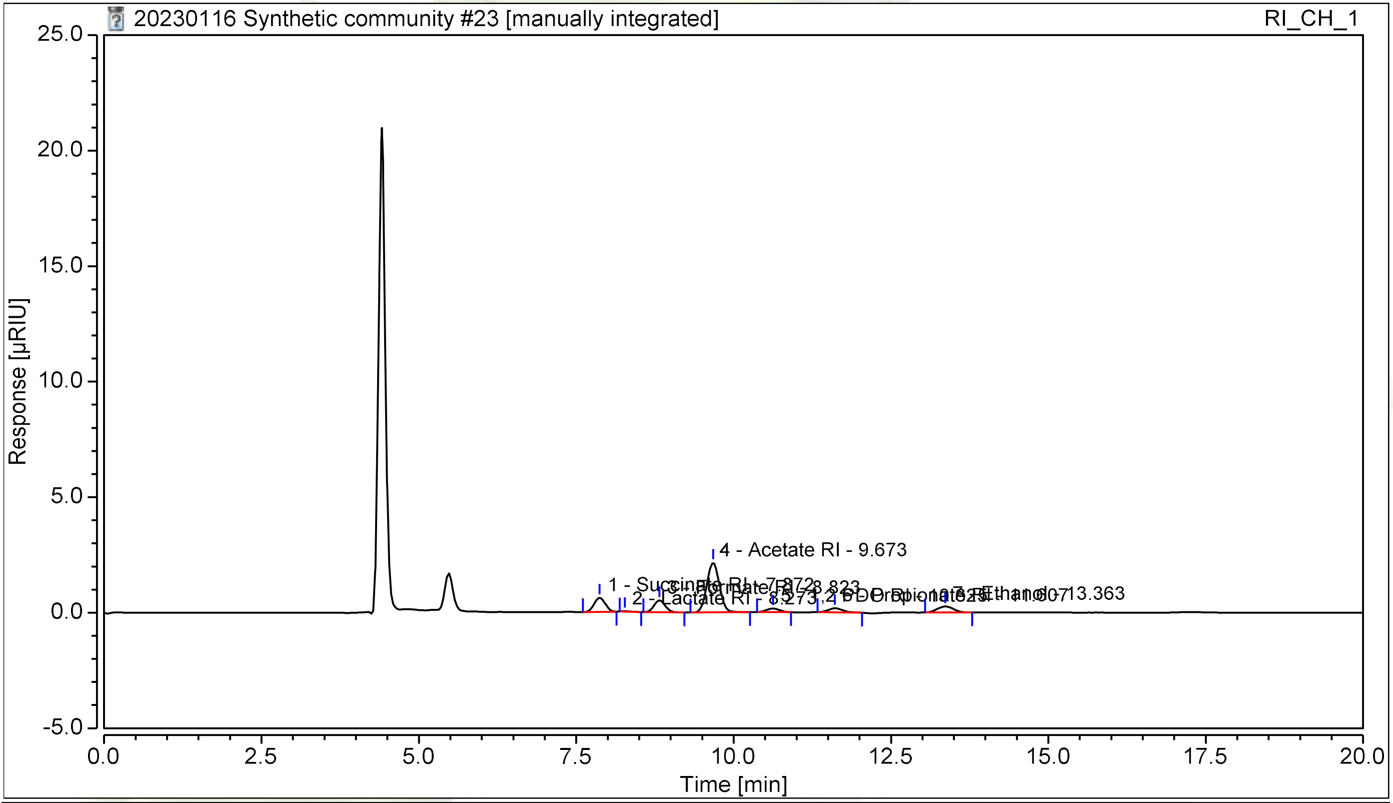

SST Results

No.	Name	Inj.Condition	Peak	Test Result	Injection

Number of executed test cases: **n.a.** **Total Result:** **Passed**

Chromatogram and Results

Injection Details

Injection Name:	**GOSFOSEXTR t72 r2**	*Run Time (min):*	**20,00**
Vial Number:	**3:17**	*Injection Volume:*	**10,00**
Injection Type:	**Unknown**	*Channel:*	**RI_CH_1**
Calibration Level:		*Wavelength:*	**n.a.**
Instrument Method:	**Default method LC2030C 45 gr 20 min**	*Bandwidth:*	**n.a.**
Processing Method:	**Processing Method LC2030 45 gr**	*Dilution Factor:*	**1,0000**
Injection Date/Time:	**16/Jan/23 23:02**	*Sample Weight:*	**1,0000**

Chromatogram

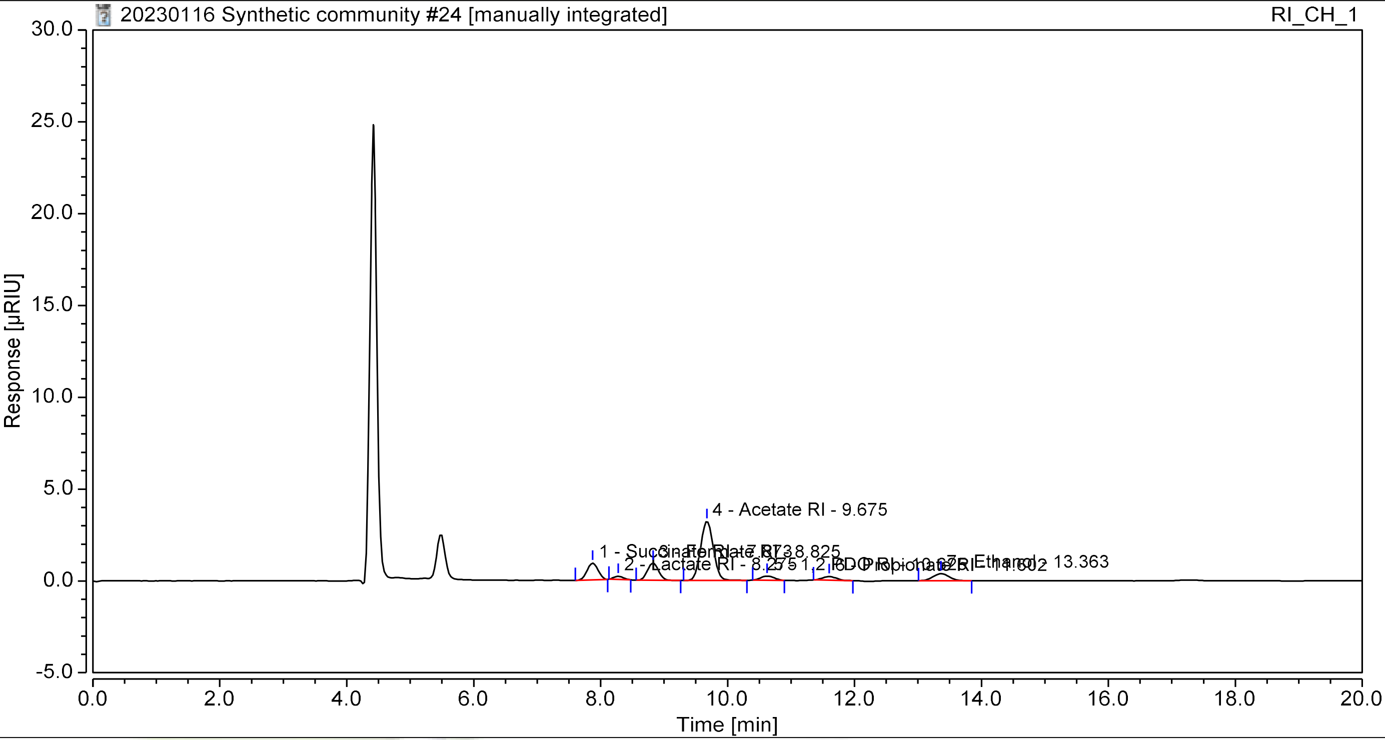

Integration Results

No.	Peak Name	Retention Time min	Area µRIU*min	Height µRIU	Relative Area %	Relative Height %	Amount
n.a.	GlcNAc	n.a.	n.a.	n.a.	n.a.	n.a.	n.a.
n.a.	Citrate	n.a.	n.a.	n.a.	n.a.	n.a.	n.a.
n.a.	Glucose	n.a.	n.a.	n.a.	n.a.	n.a.	n.a.
n.a.	Galactose	n.a.	n.a.	n.a.	n.a.	n.a.	n.a.
n.a.	Fucose	n.a.	n.a.	n.a.	n.a.	n.a.	n.a.
1	Succinate RI	7,873	0,182	0,903	13,30	15,00	3,7668
2	Lactate RI	8,275	0,031	0,176	2,26	2,93	0,9075
n.a.	glycerol	n.a.	n.a.	n.a.	n.a.	n.a.	n.a.
3	Formate RI	8,825	0,192	0,932	13,97	15,48	19,6288
4	Acetate RI	9,675	0,750	3,213	54,63	53,36	46,4992
5	1,2 PDO RI	10,625	0,054	0,222	3,94	3,69	1,6457
n.a.	1,3-PDO	n.a.	n.a.	n.a.	n.a.	n.a.	n.a.
6	Propionate RI	11,602	0,048	0,192	3,52	3,19	2,0197
n.a.	1,3-PDO	n.a.	n.a.	n.a.	n.a.	n.a.	n.a.
n.a.	2-3 BDO	n.a.	n.a.	n.a.	n.a.	n.a.	n.a.
7	Ethanol	13,363	0,115	0,382	8,38	6,35	12,0656
n.a.	Isobutyrate RI	n.a.	n.a.	n.a.	n.a.	n.a.	n.a.
n.a.	Butyrate RI	n.a.	n.a.	n.a.	n.a.	n.a.	n.a.
**Total:**			**1,373**	**6,021**	**100,00**	**100,00**	

Peak Analysis

Injection Details

Injection Name:	**GOSFOSEXTR t72 r2**	*Run Time (min):*	**20,00**
Vial Number:	**3:17**	*Injection Volume:*	**10,00**
Injection Type:	**Unknown**	*Channel:*	**RI_CH_1**
Calibration Level:		*Wavelength:*	**n.a.**
Instrument Method:	**Default method LC2030C 45 gr 20 min**	*Bandwidth:*	**n.a.**
Processing Method:	**Processing Method LC2030 45 gr**	*Dilution Factor:*	**1,0000**
Injection Date/Time:	**16/Jan/23 23:02**	*Sample Weight:*	**1,0000**

Chromatogram

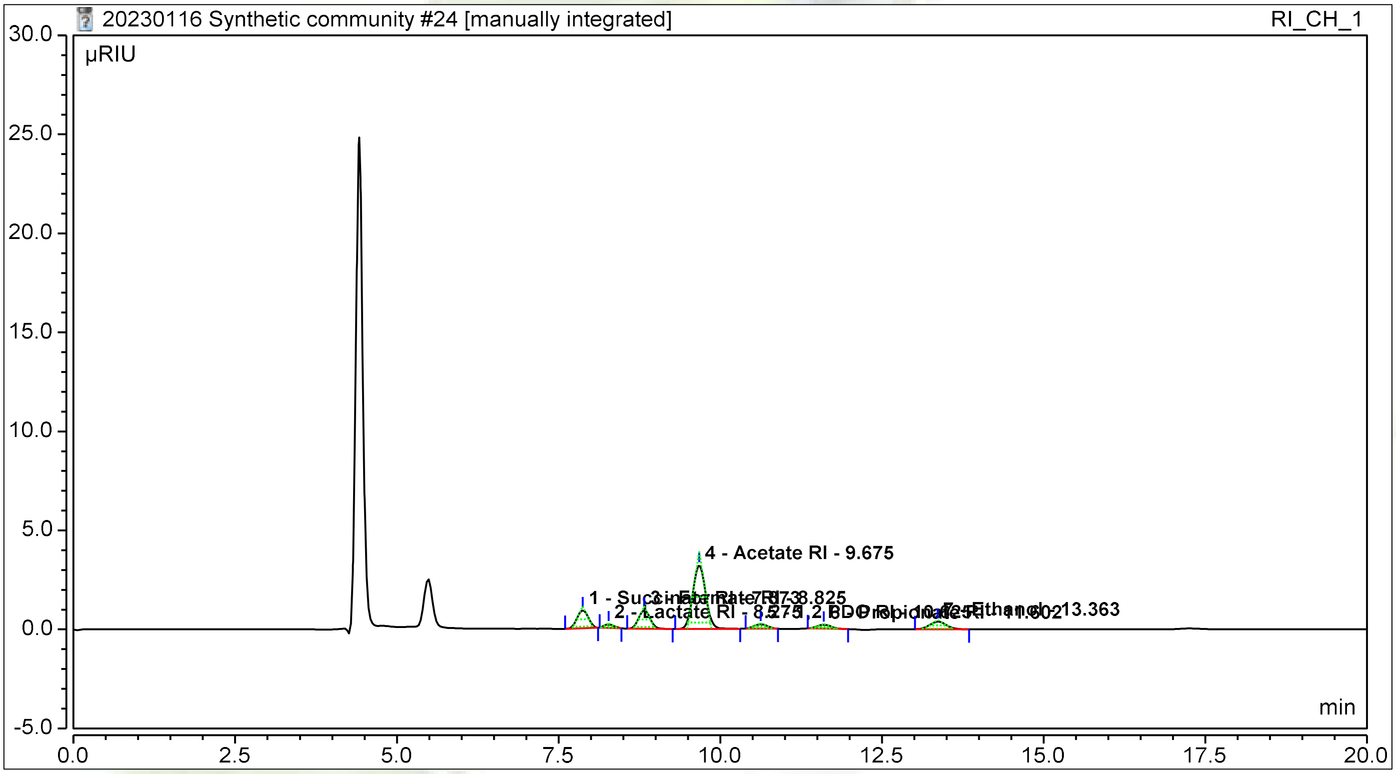

Peak Results

No.	Peak Name	Retention Time min	Width (50%) min	Type	Resolution (EP)	Asymmetry (EP)	Plates (EP)
n.a.	GlcNAc	n.a.	n.a.	n.a.	n.a.	n.a.	n.a.
n.a.	Citrate	n.a.	n.a.	n.a.	n.a.	n.a.	n.a.
n.a.	Glucose	n.a.	n.a.	n.a.	n.a.	n.a.	n.a.
n.a.	Galactose	n.a.	n.a.	n.a.	n.a.	n.a.	n.a.
n.a.	Fucose	n.a.	n.a.	n.a.	n.a.	n.a.	n.a.
1	Succinate RI	7,873	0,194	BMB	1,29	1,01	9090
2	Lactate RI	8,275	0,174	BMB*	1,76	1,16	12475
n.a.	glycerol	n.a.	n.a.	n.a.	n.a.	n.a.	n.a.
3	Formate RI	8,825	0,194	BMB	2,43	1,05	11431
4	Acetate RI	9,675	0,219	BMB	2,46	1,04	10836
5	1,2 PDO RI	10,625	0,237	BMB*	2,40	1,06	11088
n.a.	1,3-PDO	n.a.	n.a.	n.a.	n.a.	n.a.	n.a.
6	Propionate RI	11,602	0,242	BMB*	3,93	1,03	12680
n.a.	1,3-PDO	n.a.	n.a.	n.a.	n.a.	n.a.	n.a.
n.a.	2-3 BDO	n.a.	n.a.	n.a.	n.a.	n.a.	n.a.
7	Ethanol	13,363	0,286	BMB*	n.a.	1,04	12104
n.a.	Isobutyrate RI	n.a.	n.a.	n.a.	n.a.	n.a.	n.a.
n.a.	Butyrate RI	n.a.	n.a.	n.a.	n.a.	n.a.	n.a.

Chromatogram and SST Results

Injection Details

Injection Name:	**GOSFOSEXTR t72 r2**	*Run Time (min):*	**20,00**
Vial Number:	**3:17**	*Injection Volume:*	**10,00**
Injection Type:	**Unknown**	*Channel:*	**RI_CH_1**
Calibration Level:		*Wavelength:*	**n.a.**
Instrument Method:	**Default method LC2030C 45 gr 20 min**	*Bandwidth:*	**n.a.**
Processing Method:	**Processing Method LC2030 45 gr**	*Dilution Factor:*	**1,0000**
Injection Date/Time:	**16/Jan/23 23:02**	*Sample Weight:*	**1,0000**

Chromatogram

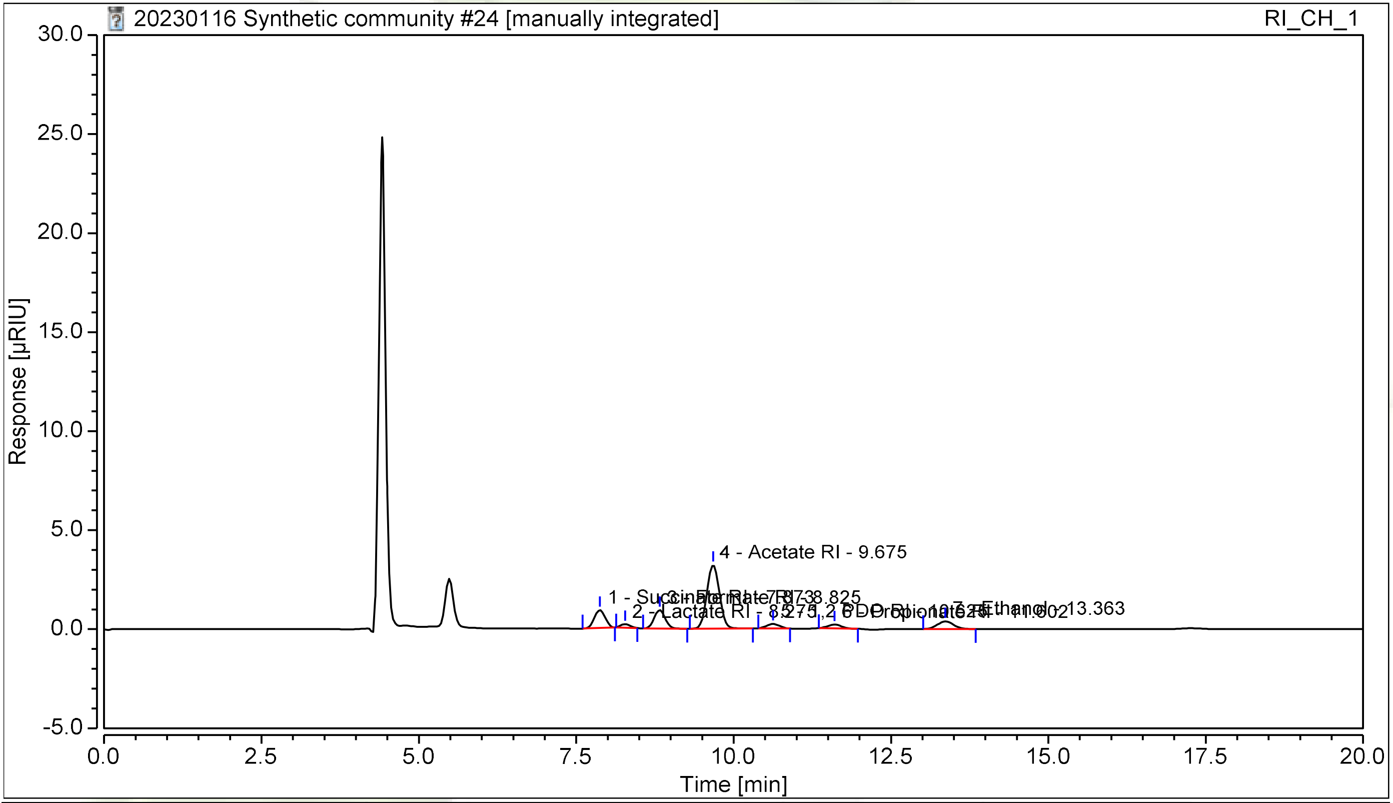

SST Results

No.	Name	Inj.Condition	Peak	Test Result	Injection

Number of executed test cases: **n.a.** **Total Result:** **Passed**

Chromatogram and Results

Injection Details

Injection Name:	**GOSFOSEXTR t72 r3**	*Run Time (min):*	**20,00**
Vial Number:	**3:18**	*Injection Volume:*	**10,00**
Injection Type:	**Unknown**	*Channel:*	**RI_CH_1**
Calibration Level:		*Wavelength:*	**n.a.**
Instrument Method:	**Default method LC2030C 45 gr 20 min**	*Bandwidth:*	**n.a.**
Processing Method:	**Processing Method LC2030 45 gr**	*Dilution Factor:*	**1,0000**
Injection Date/Time:	**16/Jan/23 23:22**	*Sample Weight:*	**1,0000**

Chromatogram

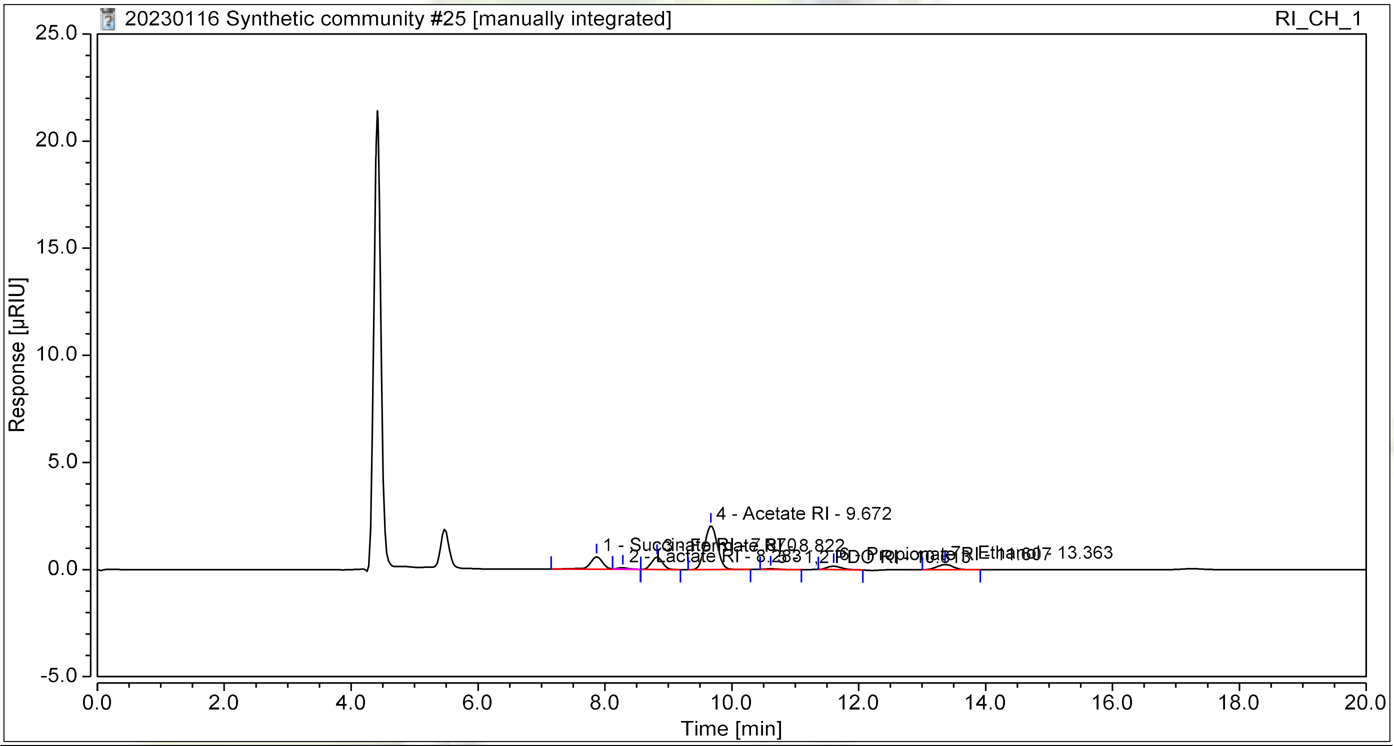

Integration Results

No.	Peak Name	Retention Time min	Area µRIU*min	Height µRIU	Relative Area %	Relative Height %	Amount
n.a.	GlcNAc	n.a.	n.a.	n.a.	n.a.	n.a.	n.a.
n.a.	Citrate	n.a.	n.a.	n.a.	n.a.	n.a.	n.a.
n.a.	Glucose	n.a.	n.a.	n.a.	n.a.	n.a.	n.a.
n.a.	Galactose	n.a.	n.a.	n.a.	n.a.	n.a.	n.a.
n.a.	Fucose	n.a.	n.a.	n.a.	n.a.	n.a.	n.a.
1	Succinate RI	7,870	0,146	0,590	16,73	15,96	3,0189
2	Lactate RI	8,283	0,011	0,057	1,27	1,54	0,3260
n.a.	glycerol	n.a.	n.a.	n.a.	n.a.	n.a.	n.a.
3	Formate RI	8,822	0,119	0,574	13,59	15,52	12,1608
4	Acetate RI	9,672	0,476	2,044	54,44	55,26	29,5198
5	1,2 PDO RI	10,613	0,006	0,029	0,72	0,80	0,1910
n.a.	1,3-PDO	n.a.	n.a.	n.a.	n.a.	n.a.	n.a.
6	Propionate RI	11,607	0,043	0,164	4,96	4,43	1,8156
n.a.	1,3-PDO	n.a.	n.a.	n.a.	n.a.	n.a.	n.a.
n.a.	2-3 BDO	n.a.	n.a.	n.a.	n.a.	n.a.	n.a.
7	Ethanol	13,363	0,072	0,240	8,29	6,50	7,6037
n.a.	Isobutyrate RI	n.a.	n.a.	n.a.	n.a.	n.a.	n.a.
n.a.	Butyrate RI	n.a.	n.a.	n.a.	n.a.	n.a.	n.a.
**Total:**			**0,874**	**3,698**	**100,00**	**100,00**	

Chromeleon (c) Dionex
Version 7.2.10.25868

Peak Analysis

Injection Details

Injection Name:	**GOSFOSEXTR t72 r3**	*Run Time (min):*	**20,00**
Vial Number:	**3:18**	*Injection Volume:*	**10,00**
Injection Type:	**Unknown**	*Channel:*	**RI_CH_1**
Calibration Level:		*Wavelength:*	**n.a.**
Instrument Method:	**Default method LC2030C 45 gr 20 min**	*Bandwidth:*	**n.a.**
Processing Method:	**Processing Method LC2030 45 gr**	*Dilution Factor:*	**1,0000**
Injection Date/Time:	**16/Jan/23 23:22**	*Sample Weight:*	**1,0000**

Chromatogram

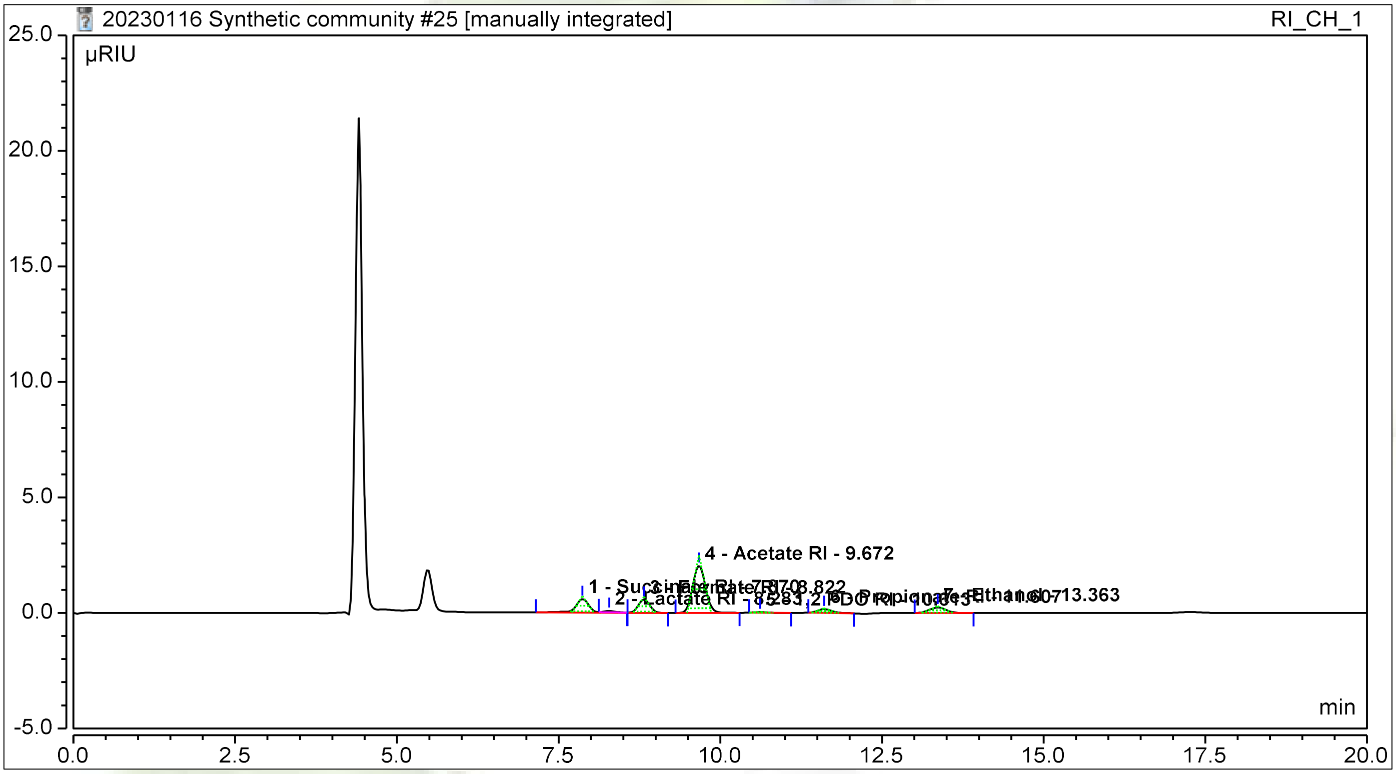

Peak Results

No.	Peak Name	Retention Time min	Width (50%) min	Type	Resolution (EP)	Asymmetry (EP)	Plates (EP)
n.a.	GlcNAc	n.a.	n.a.	n.a.	n.a.	n.a.	n.a.
n.a.	Citrate	n.a.	n.a.	n.a.	n.a.	n.a.	n.a.
n.a.	Glucose	n.a.	n.a.	n.a.	n.a.	n.a.	n.a.
n.a.	Galactose	n.a.	n.a.	n.a.	n.a.	n.a.	n.a.
n.a.	Fucose	n.a.	n.a.	n.a.	n.a.	n.a.	n.a.
1	Succinate RI	7,870	0,203	BM	2,83	0,81	8357
2	Lactate RI	8,283	n.a.	Rd	n.a.	n.a.	n.a.
n.a.	glycerol	n.a.	n.a.	n.a.	n.a.	n.a.	n.a.
3	Formate RI	8,822	0,195	MB	2,43	1,03	11392
4	Acetate RI	9,672	0,218	BMB	2,54	1,05	10857
5	1,2 PDO RI	10,613	0,220	BMB*	2,54	1,22	12941
n.a.	1,3-PDO	n.a.	n.a.	n.a.	n.a.	n.a.	n.a.
6	Propionate RI	11,607	0,242	BMB*	3,92	1,47	12693
n.a.	1,3-PDO	n.a.	n.a.	n.a.	n.a.	n.a.	n.a.
n.a.	2-3 BDO	n.a.	n.a.	n.a.	n.a.	n.a.	n.a.
7	Ethanol	13,363	0,286	BMB*	n.a.	1,02	12103
n.a.	Isobutyrate RI	n.a.	n.a.	n.a.	n.a.	n.a.	n.a.
n.a.	Butyrate RI	n.a.	n.a.	n.a.	n.a.	n.a.	n.a.

Chromatogram and SST Results

Injection Details

Injection Name:	**GOSFOSEXTR t72 r3**	*Run Time (min):*	**20,00**
Vial Number:	**3:18**	*Injection Volume:*	**10,00**
Injection Type:	**Unknown**	*Channel:*	**RI_CH_1**
Calibration Level:		*Wavelength:*	**n.a.**
Instrument Method:	**Default method LC2030C 45 gr 20 min**	*Bandwidth:*	**n.a.**
Processing Method:	**Processing Method LC2030 45 gr**	*Dilution Factor:*	**1,0000**
Injection Date/Time:	**16/Jan/23 23:22**	*Sample Weight:*	**1,0000**

Chromatogram

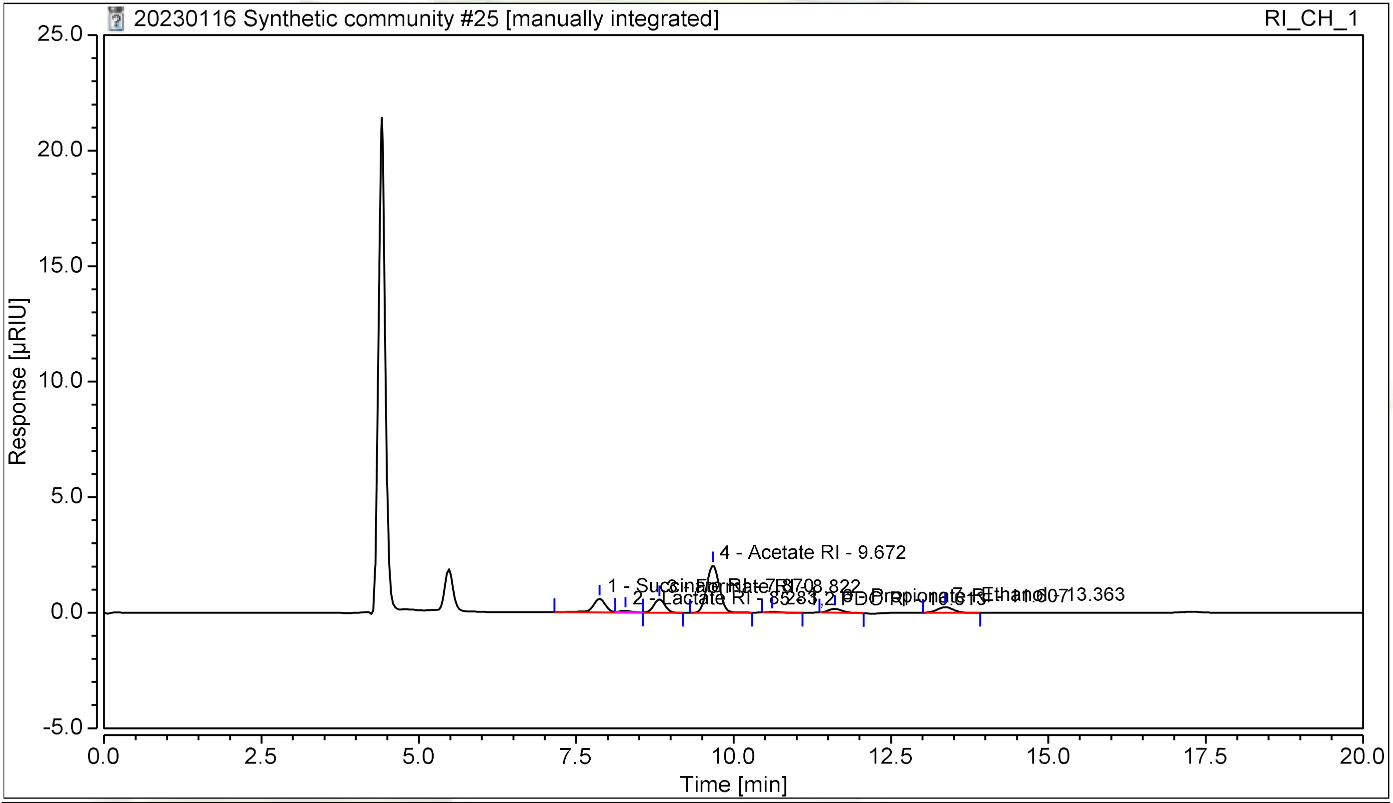

SST Results

No.	Name	Inj.Condition	Peak	Test Result	Injection

Number of executed test cases: **n.a.** **Total Result:** **Passed**

Chromeleon (c) Dionex
Version 7.2.10.25868

Chromatogram and Results

Injection Details

Injection Name:	**GOSFOSEXTR t96 r1**	*Run Time (min):*	**20,00**
Vial Number:	**3:19**	*Injection Volume:*	**10,00**
Injection Type:	**Unknown**	*Channel:*	**RI_CH_1**
Calibration Level:		*Wavelength:*	**n.a.**
Instrument Method:	**Default method LC2030C 45 gr 20 min**	*Bandwidth:*	**n.a.**
Processing Method:	**Processing Method LC2030 45 gr**	*Dilution Factor:*	**1,0000**
Injection Date/Time:	**16/Jan/23 23:43**	*Sample Weight:*	**1,0000**

Chromatogram

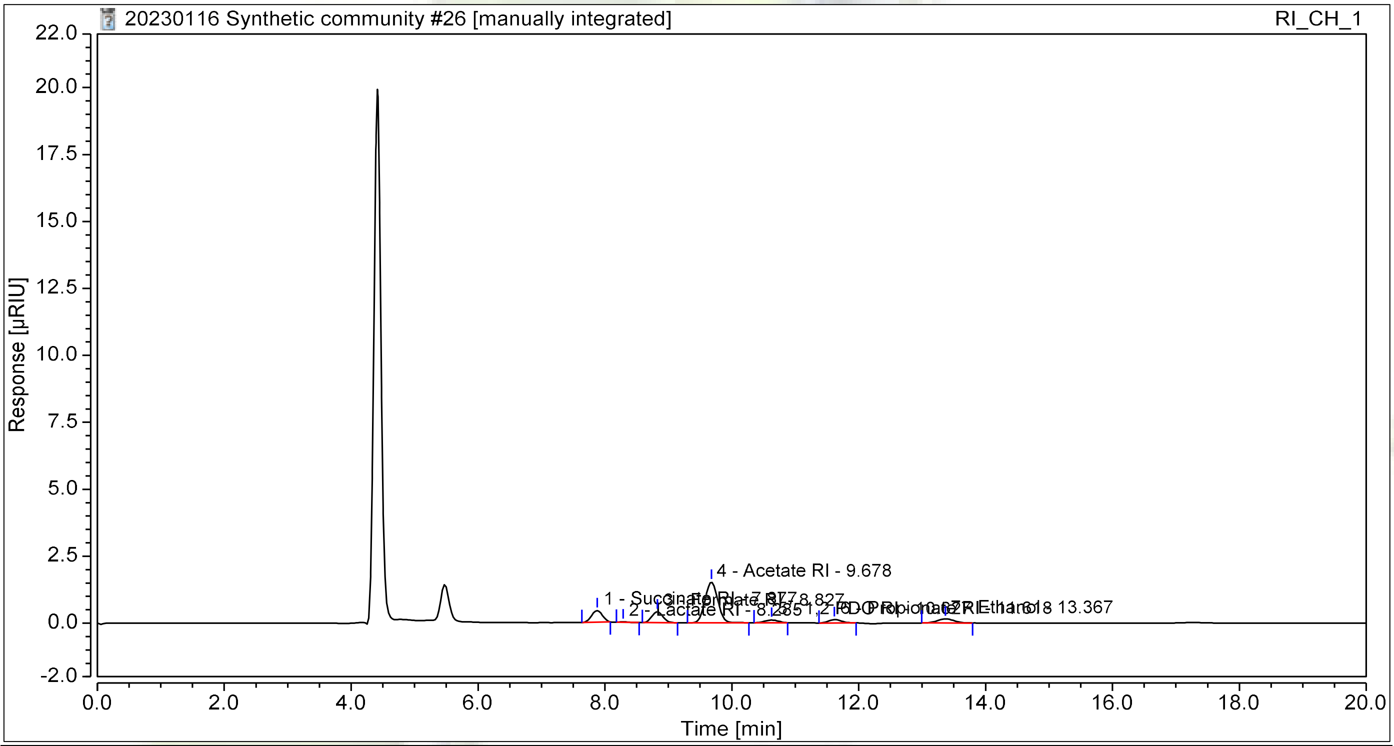

Integration Results

No.	Peak Name	Retention Time min	Area µRIU*min	Height µRIU	Relative Area %	Relative Height %	Amount
n.a.	GlcNAc	n.a.	n.a.	n.a.	n.a.	n.a.	n.a.
n.a.	Citrate	n.a.	n.a.	n.a.	n.a.	n.a.	n.a.
n.a.	Glucose	n.a.	n.a.	n.a.	n.a.	n.a.	n.a.
n.a.	Galactose	n.a.	n.a.	n.a.	n.a.	n.a.	n.a.
n.a.	Fucose	n.a.	n.a.	n.a.	n.a.	n.a.	n.a.
1	Succinate RI	7,877	0,088	0,435	13,98	15,82	1,8095
2	Lactate RI	8,285	0,003	0,019	0,46	0,69	0,0850
n.a.	glycerol	n.a.	n.a.	n.a.	n.a.	n.a.	n.a.
3	Formate RI	8,827	0,083	0,406	13,26	14,77	8,5044
4	Acetate RI	9,678	0,354	1,515	56,40	55,11	21,9264
5	1,2 PDO RI	10,627	0,024	0,097	3,80	3,52	0,7254
n.a.	1,3-PDO	n.a.	n.a.	n.a.	n.a.	n.a.	n.a.
6	Propionate RI	11,618	0,030	0,124	4,78	4,52	1,2539
n.a.	1,3-PDO	n.a.	n.a.	n.a.	n.a.	n.a.	n.a.
n.a.	2-3 BDO	n.a.	n.a.	n.a.	n.a.	n.a.	n.a.
7	Ethanol	13,367	0,046	0,153	7,32	5,58	4,8123
n.a.	Isobutyrate RI	n.a.	n.a.	n.a.	n.a.	n.a.	n.a.
n.a.	Butyrate RI	n.a.	n.a.	n.a.	n.a.	n.a.	n.a.
**Total:**			**0,627**	**2,749**	**100,00**	**100,00**	

Chromeleon (c) Dionex
Version 7.2.10.25868

Peak Analysis

Injection Details

Injection Name:	**GOSFOSEXTR t96 r1**	*Run Time (min):*	**20,00**
Vial Number:	**3:19**	*Injection Volume:*	**10,00**
Injection Type:	**Unknown**	*Channel:*	**RI_CH_1**
Calibration Level:		*Wavelength:*	**n.a.**
Instrument Method:	**Default method LC2030C 45 gr 20 min**	*Bandwidth:*	**n.a.**
Processing Method:	**Processing Method LC2030 45 gr**	*Dilution Factor:*	**1,0000**
Injection Date/Time:	**16/Jan/23 23:43**	*Sample Weight:*	**1,0000**

Chromatogram

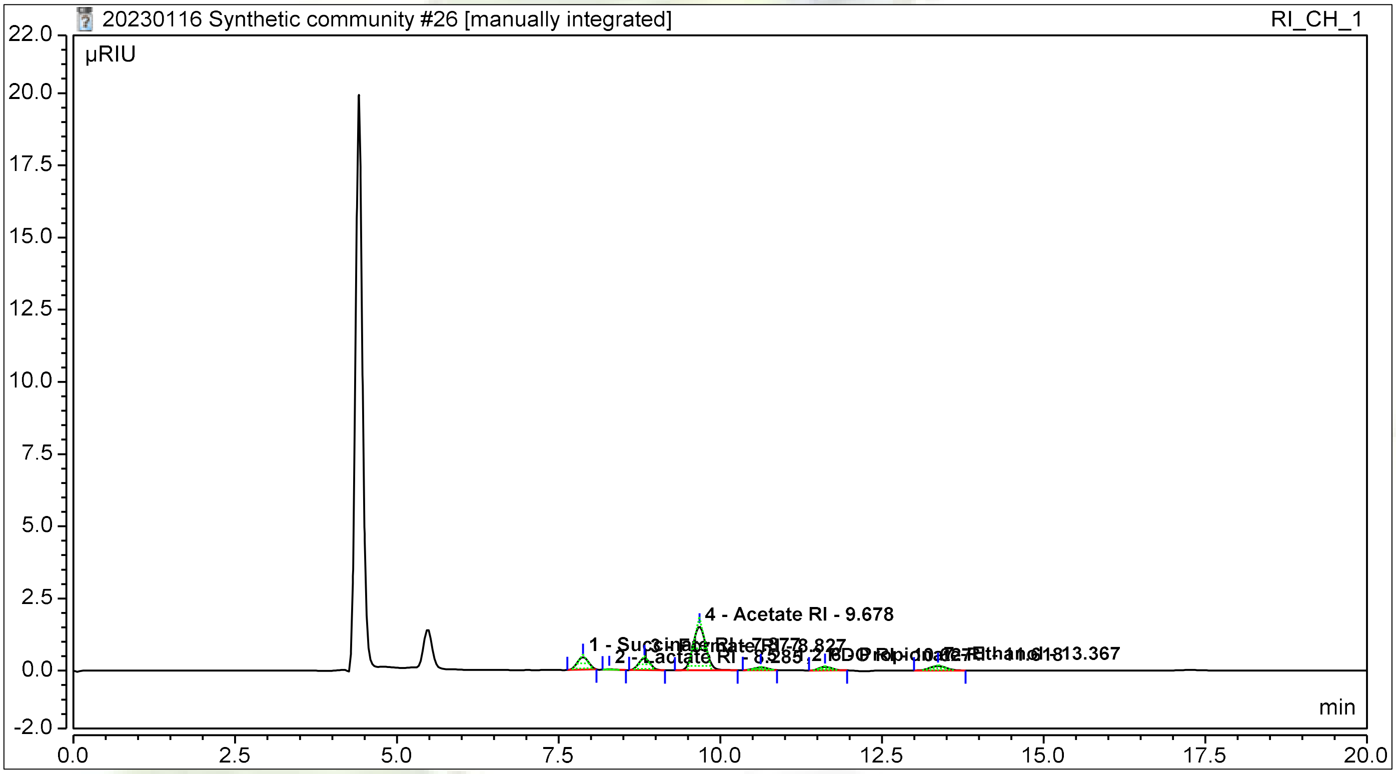

Peak Results

No.	Peak Name	Retention Time<br>min	Width (50%)<br>min	Type	Resolution (EP)	Asymmetry (EP)	Plates (EP)
n.a.	GlcNAc	n.a.	n.a.	n.a.	n.a.	n.a.	n.a.
n.a.	Citrate	n.a.	n.a.	n.a.	n.a.	n.a.	n.a.
n.a.	Glucose	n.a.	n.a.	n.a.	n.a.	n.a.	n.a.
n.a.	Galactose	n.a.	n.a.	n.a.	n.a.	n.a.	n.a.
n.a.	Fucose	n.a.	n.a.	n.a.	n.a.	n.a.	n.a.
1	Succinate RI	7,877	0,195	BMB*	1,39	0,99	9082
2	Lactate RI	8,285	0,151	BMB*	1,85	1,36	16644
n.a.	glycerol	n.a.	n.a.	n.a.	n.a.	n.a.	n.a.
3	Formate RI	8,827	0,194	BMB*	2,43	1,05	11445
4	Acetate RI	9,678	0,219	BMB	2,45	1,04	10823
5	1,2 PDO RI	10,627	0,238	BMB*	2,49	0,98	11024
n.a.	1,3-PDO	n.a.	n.a.	n.a.	n.a.	n.a.	n.a.
6	Propionate RI	11,618	0,231	BMB*	4,00	1,16	14025
n.a.	1,3-PDO	n.a.	n.a.	n.a.	n.a.	n.a.	n.a.
n.a.	2-3 BDO	n.a.	n.a.	n.a.	n.a.	n.a.	n.a.
7	Ethanol	13,367	0,285	BMB*	n.a.	1,04	12175
n.a.	Isobutyrate RI	n.a.	n.a.	n.a.	n.a.	n.a.	n.a.
n.a.	Butyrate RI	n.a.	n.a.	n.a.	n.a.	n.a.	n.a.

Chromatogram and SST Results

Injection Details

Injection Name:	**GOSFOSEXTR t96 r1**	*Run Time (min):*	**20,00**
Vial Number:	**3:19**	*Injection Volume:*	**10,00**
Injection Type:	**Unknown**	*Channel:*	**RI_CH_1**
Calibration Level:		*Wavelength:*	**n.a.**
Instrument Method:	**Default method LC2030C 45 gr 20 min**	*Bandwidth:*	**n.a.**
Processing Method:	**Processing Method LC2030 45 gr**	*Dilution Factor:*	**1,0000**
Injection Date/Time:	**16/Jan/23 23:43**	*Sample Weight:*	**1,0000**

Chromatogram

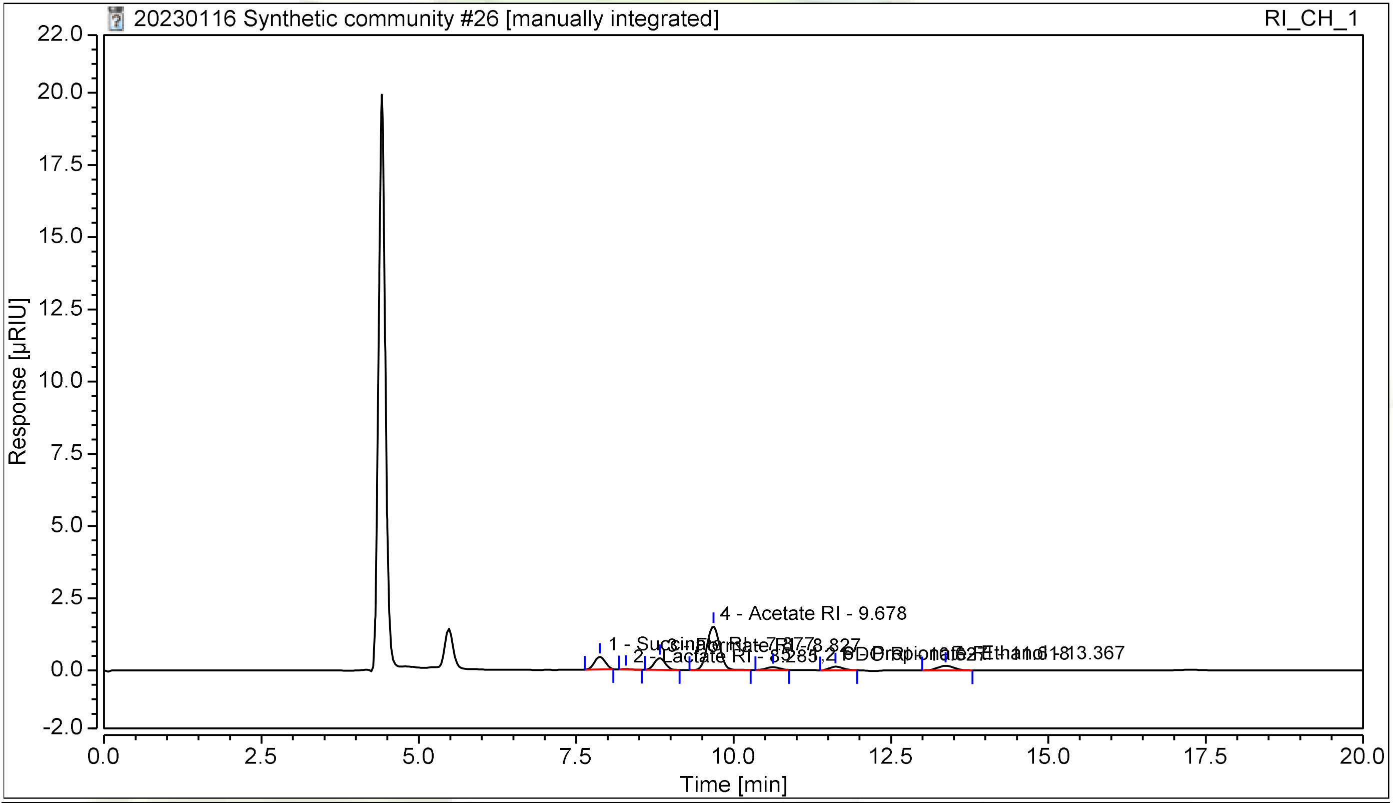

SST Results

No.	Name	Inj.Condition	Peak	Test Result	Injection

Number of executed test cases: **n.a.** **Total Result:** **Passed**

Chromatogram and Results

Injection Details

Injection Name:	**GOSFOSEXTR t96 r2**	*Run Time (min):*	**20,00**
Vial Number:	**3:20**	*Injection Volume:*	**10,00**
Injection Type:	**Unknown**	*Channel:*	**RI_CH_1**
Calibration Level:		*Wavelength:*	**n.a.**
Instrument Method:	**Default method LC2030C 45 gr 20 min**	*Bandwidth:*	**n.a.**
Processing Method:	**Processing Method LC2030 45 gr**	*Dilution Factor:*	**1,0000**
Injection Date/Time:	**17/Jan/23 00:03**	*Sample Weight:*	**1,0000**

Chromatogram

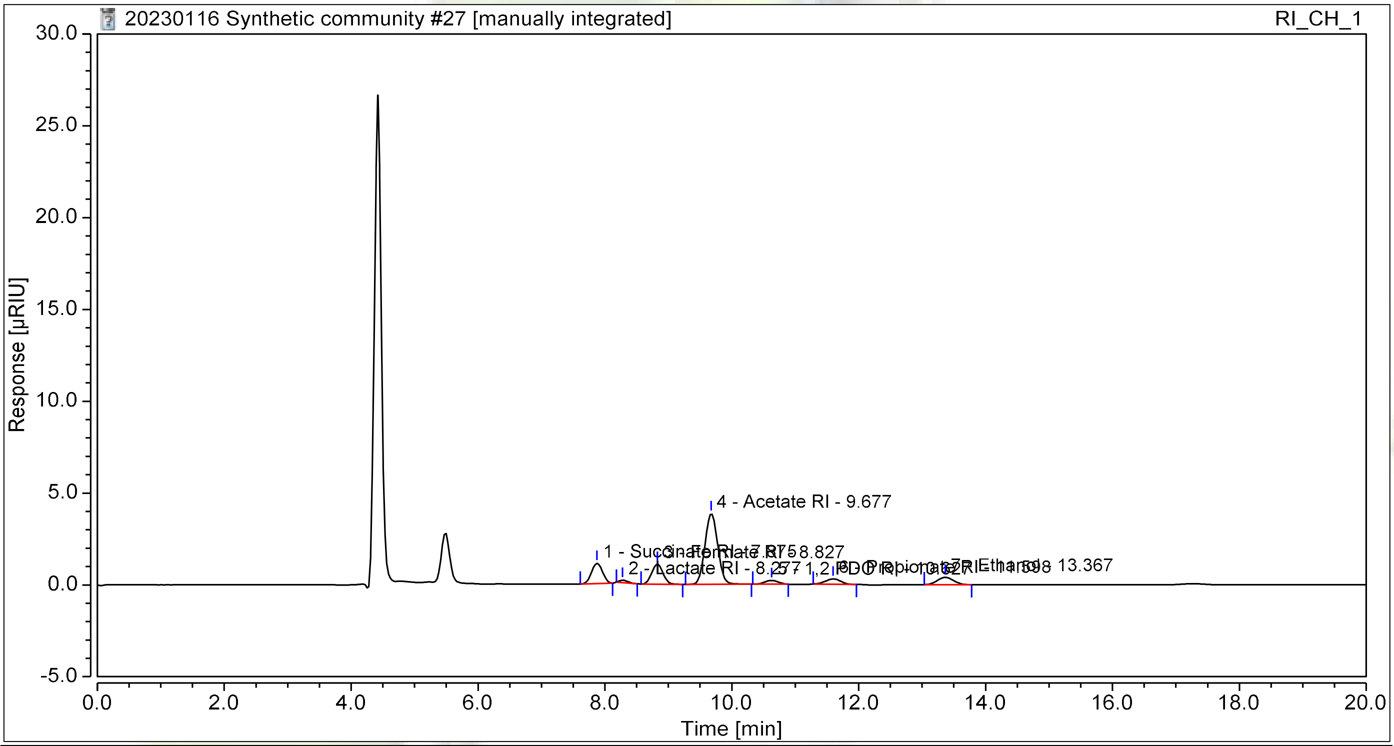

Integration Results

No.	Peak Name	Retention Time min	Area µRIU*min	Height µRIU	Relative Area %	Relative Height %	Amount
n.a.	GlcNAc	n.a.	n.a.	n.a.	n.a.	n.a.	n.a.
n.a.	Citrate	n.a.	n.a.	n.a.	n.a.	n.a.	n.a.
n.a.	Glucose	n.a.	n.a.	n.a.	n.a.	n.a.	n.a.
n.a.	Galactose	n.a.	n.a.	n.a.	n.a.	n.a.	n.a.
n.a.	Fucose	n.a.	n.a.	n.a.	n.a.	n.a.	n.a.
1	Succinate RI	7,875	0,223	1,100	13,83	15,61	4,6039
2	Lactate RI	8,277	0,021	0,133	1,29	1,89	0,6103
n.a.	glycerol	n.a.	n.a.	n.a.	n.a.	n.a.	n.a.
3	Formate RI	8,827	0,223	1,083	13,80	15,37	22,7724
4	Acetate RI	9,677	0,898	3,846	55,70	54,59	55,7151
5	1,2 PDO RI	10,627	0,050	0,200	3,12	2,84	1,5304
n.a.	1,3-PDO	n.a.	n.a.	n.a.	n.a.	n.a.	n.a.
6	Propionate RI	11,598	0,079	0,289	4,92	4,09	3,3184
n.a.	1,3-PDO	n.a.	n.a.	n.a.	n.a.	n.a.	n.a.
n.a.	2-3 BDO	n.a.	n.a.	n.a.	n.a.	n.a.	n.a.
7	Ethanol	13,367	0,118	0,396	7,34	5,62	12,4270
n.a.	Isobutyrate RI	n.a.	n.a.	n.a.	n.a.	n.a.	n.a.
n.a.	Butyrate RI	n.a.	n.a.	n.a.	n.a.	n.a.	n.a.
**Total:**			**1,613**	**7,046**	**100,00**	**100,00**	

Peak Analysis

Injection Details

Injection Name:	**GOSFOSEXTR t96 r2**	*Run Time (min):*	**20,00**
Vial Number:	**3:20**	*Injection Volume:*	**10,00**
Injection Type:	**Unknown**	*Channel:*	**RI_CH_1**
Calibration Level:		*Wavelength:*	**n.a.**
Instrument Method:	**Default method LC2030C 45 gr 20 min**	*Bandwidth:*	**n.a.**
Processing Method:	**Processing Method LC2030 45 gr**	*Dilution Factor:*	**1,0000**
Injection Date/Time:	**17/Jan/23 00:03**	*Sample Weight:*	**1,0000**

Chromatogram

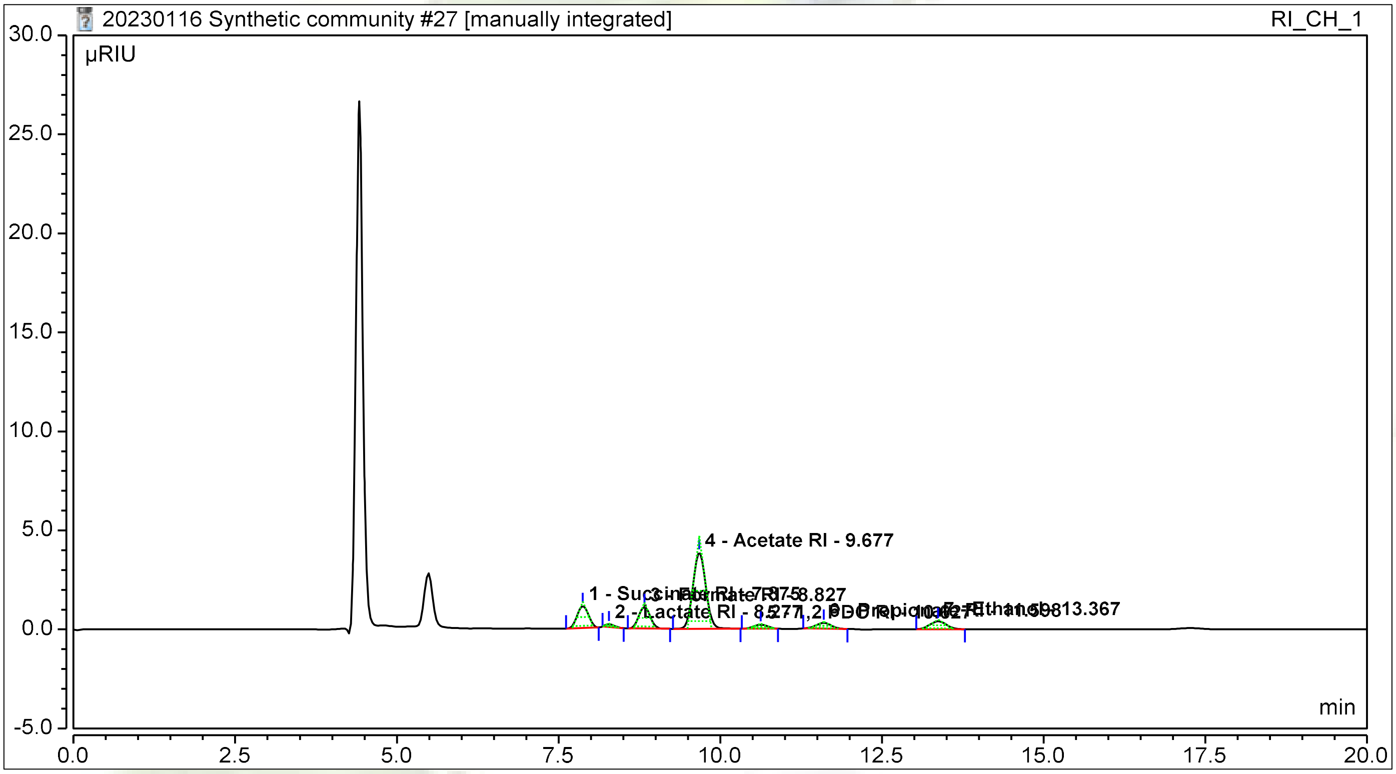

Peak Results

No.	Peak Name	Retention Time min	Width (50%) min	Type	Resolution (EP)	Asymmetry (EP)	Plates (EP)
n.a.	GlcNAc	n.a.	n.a.	n.a.	n.a.	n.a.	n.a.
n.a.	Citrate	n.a.	n.a.	n.a.	n.a.	n.a.	n.a.
n.a.	Glucose	n.a.	n.a.	n.a.	n.a.	n.a.	n.a.
n.a.	Galactose	n.a.	n.a.	n.a.	n.a.	n.a.	n.a.
n.a.	Fucose	n.a.	n.a.	n.a.	n.a.	n.a.	n.a.
1	Succinate RI	7,875	0,195	BMB	1,35	1,01	9055
2	Lactate RI	8,277	0,155	BMB*	1,86	1,45	15717
n.a.	glycerol	n.a.	n.a.	n.a.	n.a.	n.a.	n.a.
3	Formate RI	8,827	0,194	BMB	2,43	1,05	11435
4	Acetate RI	9,677	0,219	BMB	2,43	1,04	10829
5	1,2 PDO RI	10,627	0,242	BMB*	2,28	0,99	10710
n.a.	1,3-PDO	n.a.	n.a.	n.a.	n.a.	n.a.	n.a.
6	Propionate RI	11,598	0,261	BMB*	3,82	0,96	10950
n.a.	1,3-PDO	n.a.	n.a.	n.a.	n.a.	n.a.	n.a.
n.a.	2-3 BDO	n.a.	n.a.	n.a.	n.a.	n.a.	n.a.
7	Ethanol	13,367	0,286	BMB*	n.a.	1,04	12124
n.a.	Isobutyrate RI	n.a.	n.a.	n.a.	n.a.	n.a.	n.a.
n.a.	Butyrate RI	n.a.	n.a.	n.a.	n.a.	n.a.	n.a.

Chromatogram and SST Results

Injection Details

Injection Name:	**GOSFOSEXTR t96 r2**	*Run Time (min):*	**20,00**
Vial Number:	**3:20**	*Injection Volume:*	**10,00**
Injection Type:	**Unknown**	*Channel:*	**RI_CH_1**
Calibration Level:		*Wavelength:*	**n.a.**
Instrument Method:	**Default method LC2030C 45 gr 20 min**	*Bandwidth:*	**n.a.**
Processing Method:	**Processing Method LC2030 45 gr**	*Dilution Factor:*	**1,0000**
Injection Date/Time:	**17/Jan/23 00:03**	*Sample Weight:*	**1,0000**

Chromatogram

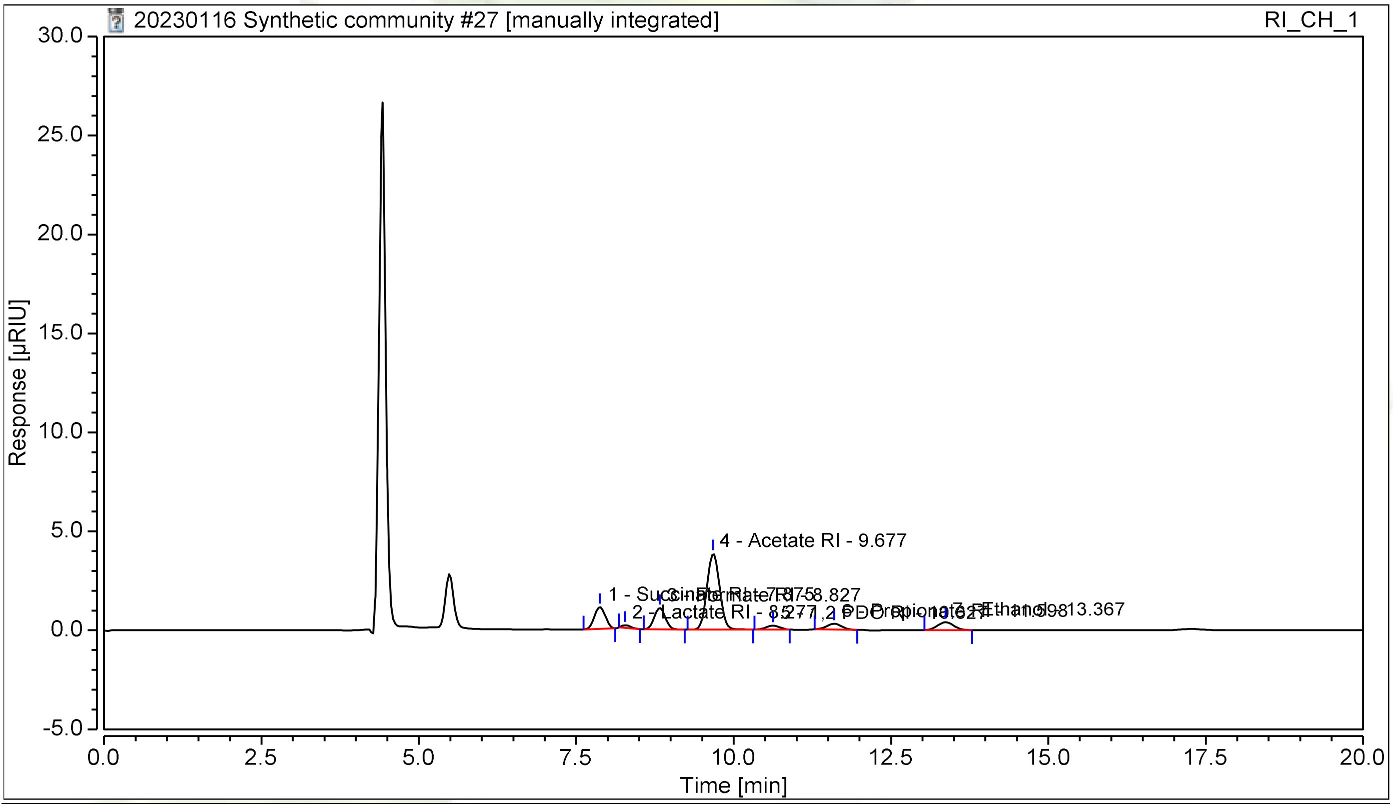

SST Results

No.	Name	Inj.Condition	Peak	Test Result	Injection

Number of executed test cases: **n.a.**   **Total Result:** **Passed**

Chromatogram and Results

Injection Details

Injection Name:	**GOSFOSEXTR t96 r3**	*Run Time (min):*	**20,00**
Vial Number:	**3:21**	*Injection Volume:*	**10,00**
Injection Type:	**Unknown**	*Channel:*	**RI_CH_1**
Calibration Level:		*Wavelength:*	**n.a.**
Instrument Method:	**Default method LC2030C 45 gr 20 min**	*Bandwidth:*	**n.a.**
Processing Method:	**Processing Method LC2030 45 gr**	*Dilution Factor:*	**1,0000**
Injection Date/Time:	**17/Jan/23 00:24**	*Sample Weight:*	**1,0000**

Chromatogram

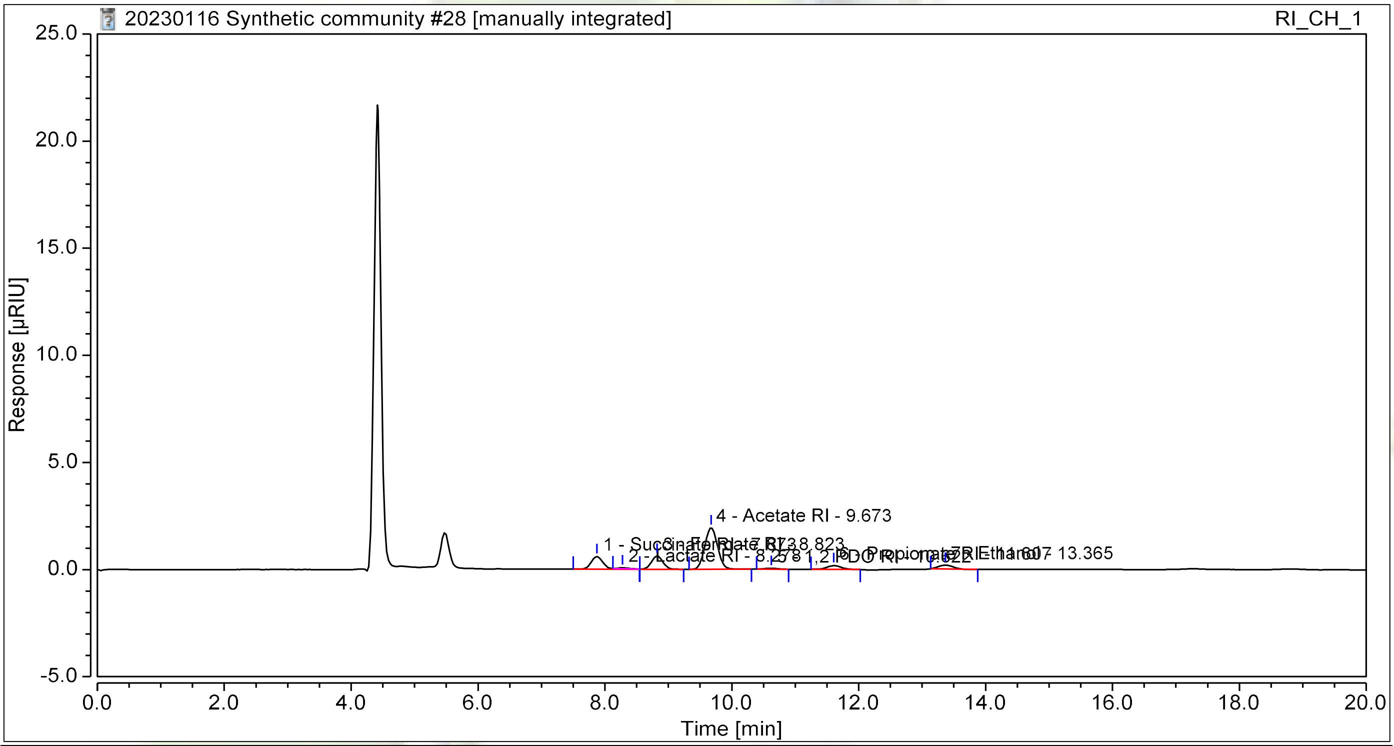

Integration Results

No.	Peak Name	Retention Time min	Area µRIU*min	Height µRIU	Relative Area %	Relative Height %	Amount
n.a.	GlcNAc	n.a.	n.a.	n.a.	n.a.	n.a.	n.a.
n.a.	Citrate	n.a.	n.a.	n.a.	n.a.	n.a.	n.a.
n.a.	Glucose	n.a.	n.a.	n.a.	n.a.	n.a.	n.a.
n.a.	Galactose	n.a.	n.a.	n.a.	n.a.	n.a.	n.a.
n.a.	Fucose	n.a.	n.a.	n.a.	n.a.	n.a.	n.a.
1	Succinate RI	7,873	0,132	0,588	16,07	16,43	2,7324
2	Lactate RI	8,278	0,009	0,047	1,04	1,32	0,2508
n.a.	glycerol	n.a.	n.a.	n.a.	n.a.	n.a.	n.a.
3	Formate RI	8,823	0,128	0,614	15,58	17,17	13,1346
4	Acetate RI	9,673	0,452	1,937	54,83	54,15	28,0181
5	1,2 PDO RI	10,622	0,010	0,040	1,18	1,11	0,2957
n.a.	1,3-PDO	n.a.	n.a.	n.a.	n.a.	n.a.	n.a.
6	Propionate RI	11,607	0,045	0,173	5,49	4,82	1,8929
n.a.	1,3-PDO	n.a.	n.a.	n.a.	n.a.	n.a.	n.a.
n.a.	2-3 BDO	n.a.	n.a.	n.a.	n.a.	n.a.	n.a.
7	Ethanol	13,365	0,048	0,179	5,81	5,00	5,0225
n.a.	Isobutyrate RI	n.a.	n.a.	n.a.	n.a.	n.a.	n.a.
n.a.	Butyrate RI	n.a.	n.a.	n.a.	n.a.	n.a.	n.a.
**Total:**			**0,824**	**3,578**	**100,00**	**100,00**	

Peak Analysis

Injection Details

Injection Name:	**GOSFOSEXTR t96 r3**	*Run Time (min):*	**20,00**
Vial Number:	**3:21**	*Injection Volume:*	**10,00**
Injection Type:	**Unknown**	*Channel:*	**RI_CH_1**
Calibration Level:		*Wavelength:*	**n.a.**
Instrument Method:	**Default method LC2030C 45 gr 20 min**	*Bandwidth:*	**n.a.**
Processing Method:	**Processing Method LC2030 45 gr**	*Dilution Factor:*	**1,0000**
Injection Date/Time:	**17/Jan/23 00:24**	*Sample Weight:*	**1,0000**

Chromatogram

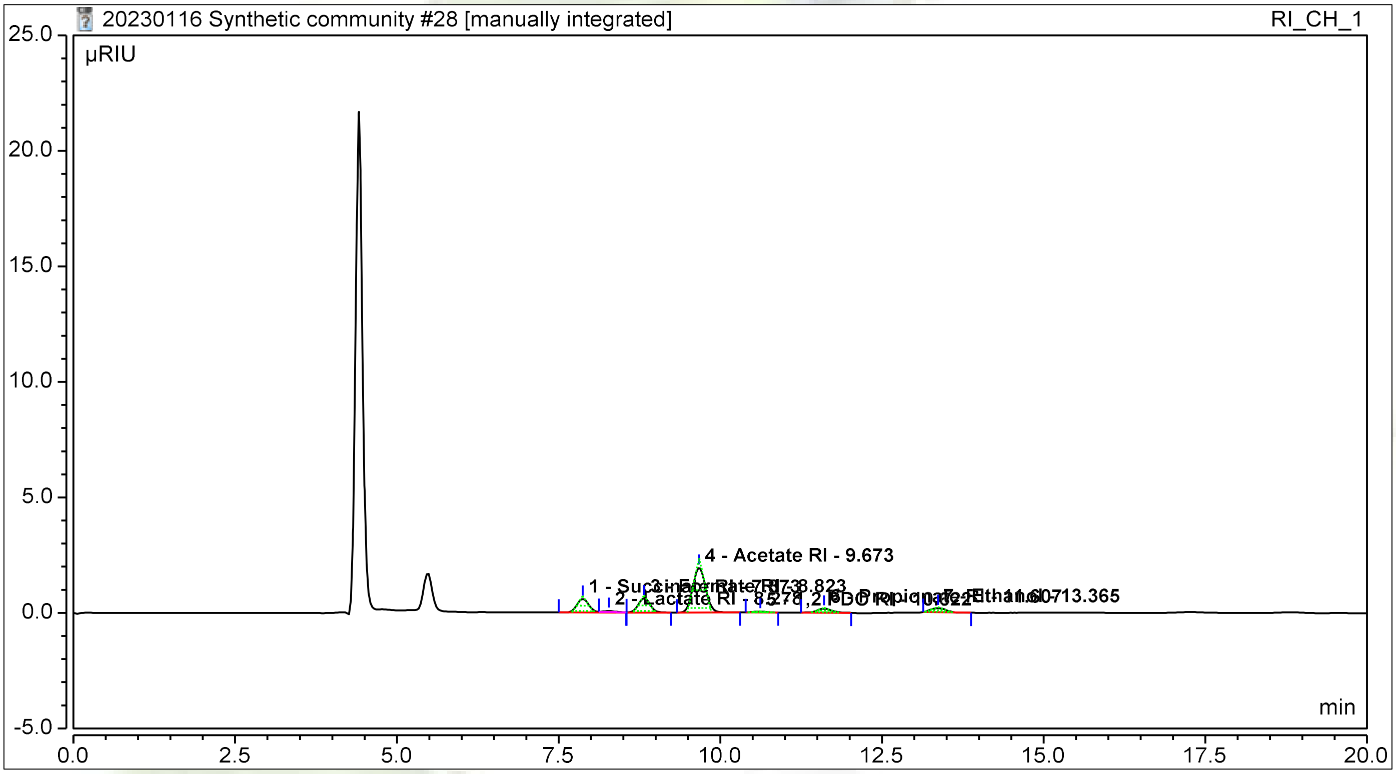

Peak Results

No.	Peak Name	Retention Time min	Width (50%) min	Type	Resolution (EP)	Asymmetry (EP)	Plates (EP)
n.a.	GlcNAc	n.a.	n.a.	n.a.	n.a.	n.a.	n.a.
n.a.	Citrate	n.a.	n.a.	n.a.	n.a.	n.a.	n.a.
n.a.	Glucose	n.a.	n.a.	n.a.	n.a.	n.a.	n.a.
n.a.	Galactose	n.a.	n.a.	n.a.	n.a.	n.a.	n.a.
n.a.	Fucose	n.a.	n.a.	n.a.	n.a.	n.a.	n.a.
1	Succinate RI	7,873	0,200	BM	2,84	1,09	8615
2	Lactate RI	8,278	n.a.	Rd	n.a.	n.a.	n.a.
n.a.	glycerol	n.a.	n.a.	n.a.	n.a.	n.a.	n.a.
3	Formate RI	8,823	0,196	MB	2,42	1,04	11264
4	Acetate RI	9,673	0,219	BMB	2,46	1,05	10827
5	1,2 PDO RI	10,622	0,237	BMB*	2,42	1,06	11172
n.a.	1,3-PDO	n.a.	n.a.	n.a.	n.a.	n.a.	n.a.
6	Propionate RI	11,607	0,245	BMB*	4,06	1,12	12461
n.a.	1,3-PDO	n.a.	n.a.	n.a.	n.a.	n.a.	n.a.
n.a.	2-3 BDO	n.a.	n.a.	n.a.	n.a.	n.a.	n.a.
7	Ethanol	13,365	0,267	BMB*	n.a.	1,10	13891
n.a.	Isobutyrate RI	n.a.	n.a.	n.a.	n.a.	n.a.	n.a.
n.a.	Butyrate RI	n.a.	n.a.	n.a.	n.a.	n.a.	n.a.

Chromeleon (c) Dionex
Version 7.2.10.25868

Chromatogram and SST Results

Injection Details

Injection Name:	**GOSFOSEXTR t96 r3**	*Run Time (min):*	**20,00**
Vial Number:	**3:21**	*Injection Volume:*	**10,00**
Injection Type:	**Unknown**	*Channel:*	**RI_CH_1**
Calibration Level:		*Wavelength:*	**n.a.**
Instrument Method:	**Default method LC2030C 45 gr 20 min**	*Bandwidth:*	**n.a.**
Processing Method:	**Processing Method LC2030 45 gr**	*Dilution Factor:*	**1,0000**
Injection Date/Time:	**17/Jan/23 00:24**	*Sample Weight:*	**1,0000**

Chromatogram

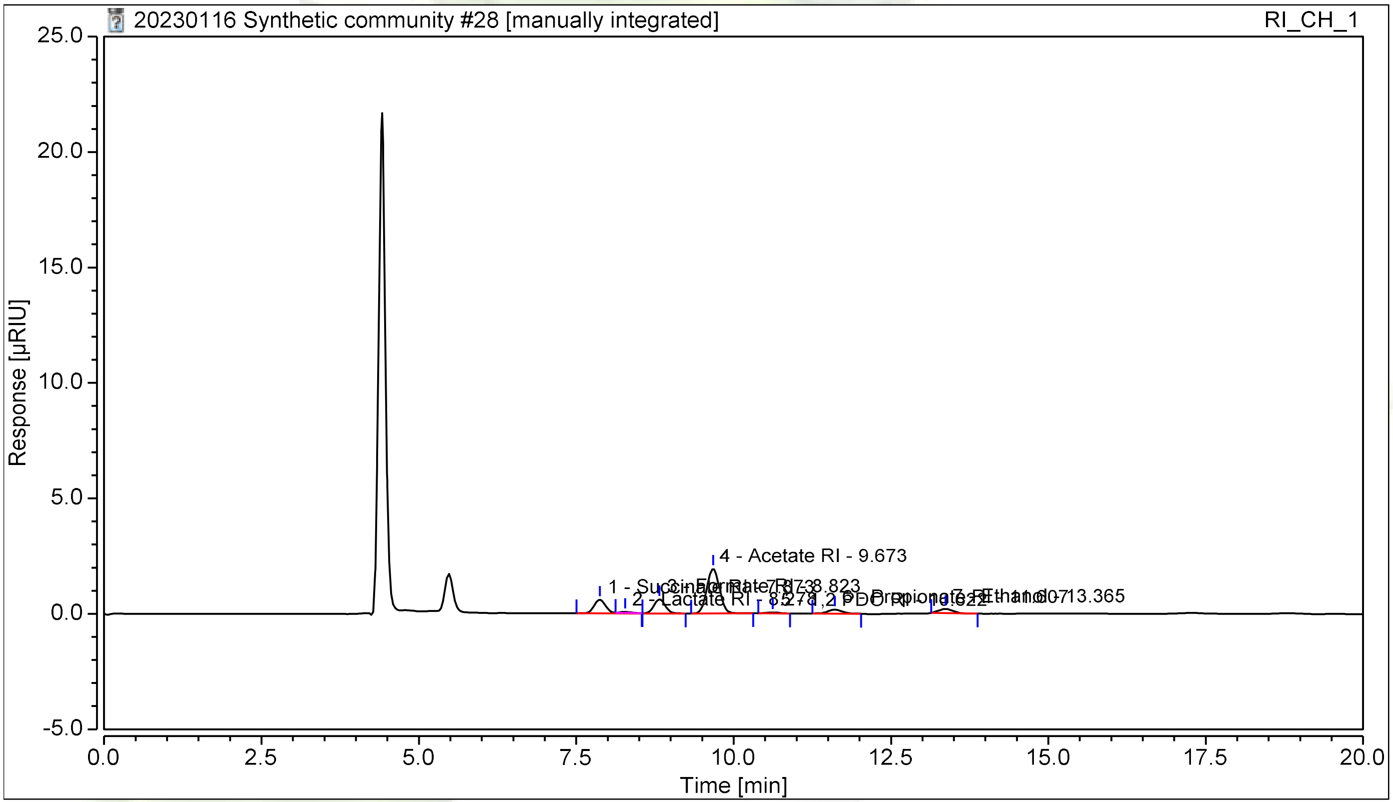

SST Results

No.	Name	Inj.Condition	Peak	Test Result	Injection
**Number of executed test cases:**	**n.a.**		**Total Result:**	**Passed**	

Chromatogram and Results

Injection Details

Injection Name:	**GOSFOSEXTR t120 r1**	*Run Time (min):*	**20,00**
Vial Number:	**3:22**	*Injection Volume:*	**10,00**
Injection Type:	**Unknown**	*Channel:*	**RI_CH_1**
Calibration Level:		*Wavelength:*	**n.a.**
Instrument Method:	**Default method LC2030C 45 gr 20 min**	*Bandwidth:*	**n.a.**
Processing Method:	**Processing Method LC2030 45 gr**	*Dilution Factor:*	**1,0000**
Injection Date/Time:	**17/Jan/23 00:44**	*Sample Weight:*	**1,0000**

Chromatogram

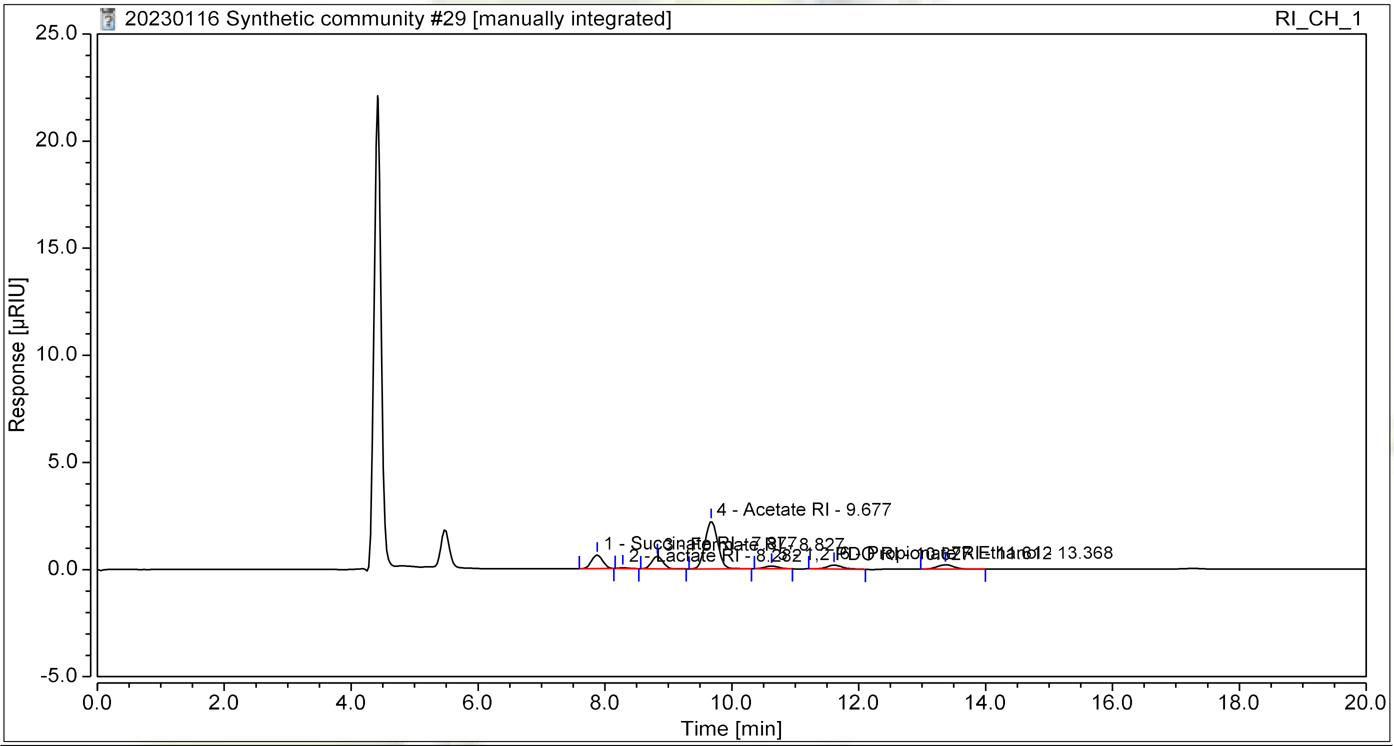

Integration Results

No.	Peak Name	Retention Time min	Area µRIU*min	Height µRIU	Relative Area %	Relative Height %	Amount
n.a.	GlcNAc	n.a.	n.a.	n.a.	n.a.	n.a.	n.a.
n.a.	Citrate	n.a.	n.a.	n.a.	n.a.	n.a.	n.a.
n.a.	Glucose	n.a.	n.a.	n.a.	n.a.	n.a.	n.a.
n.a.	Galactose	n.a.	n.a.	n.a.	n.a.	n.a.	n.a.
n.a.	Fucose	n.a.	n.a.	n.a.	n.a.	n.a.	n.a.
1	Succinate RI	7,877	0,130	0,632	14,27	15,97	2,6915
2	Lactate RI	8,282	0,006	0,035	0,66	0,88	0,1774
n.a.	glycerol	n.a.	n.a.	n.a.	n.a.	n.a.	n.a.
3	Formate RI	8,827	0,119	0,577	12,98	14,58	12,1352
4	Acetate RI	9,677	0,515	2,210	56,38	55,87	31,9440
5	1,2 PDO RI	10,627	0,032	0,125	3,50	3,16	0,9746
n.a.	1,3-PDO	n.a.	n.a.	n.a.	n.a.	n.a.	n.a.
6	Propionate RI	11,612	0,052	0,182	5,69	4,59	2,1727
n.a.	1,3-PDO	n.a.	n.a.	n.a.	n.a.	n.a.	n.a.
n.a.	2-3 BDO	n.a.	n.a.	n.a.	n.a.	n.a.	n.a.
7	Ethanol	13,368	0,060	0,196	6,52	4,95	6,2443
n.a.	Isobutyrate RI	n.a.	n.a.	n.a.	n.a.	n.a.	n.a.
n.a.	Butyrate RI	n.a.	n.a.	n.a.	n.a.	n.a.	n.a.
**Total:**			**0,914**	**3,955**	**100,00**	**100,00**	

Chromeleon (c) Dionex
Version 7.2.10.25868

Peak Analysis

Injection Details

Injection Name:	**GOSFOSEXTR t120 r1**	*Run Time (min):*	**20,00**
Vial Number:	**3:22**	*Injection Volume:*	**10,00**
Injection Type:	**Unknown**	*Channel:*	**RI_CH_1**
Calibration Level:		*Wavelength:*	**n.a.**
Instrument Method:	**Default method LC2030C 45 gr 20 min**	*Bandwidth:*	**n.a.**
Processing Method:	**Processing Method LC2030 45 gr**	*Dilution Factor:*	**1,0000**
Injection Date/Time:	**17/Jan/23 00:44**	*Sample Weight:*	**1,0000**

Chromatogram

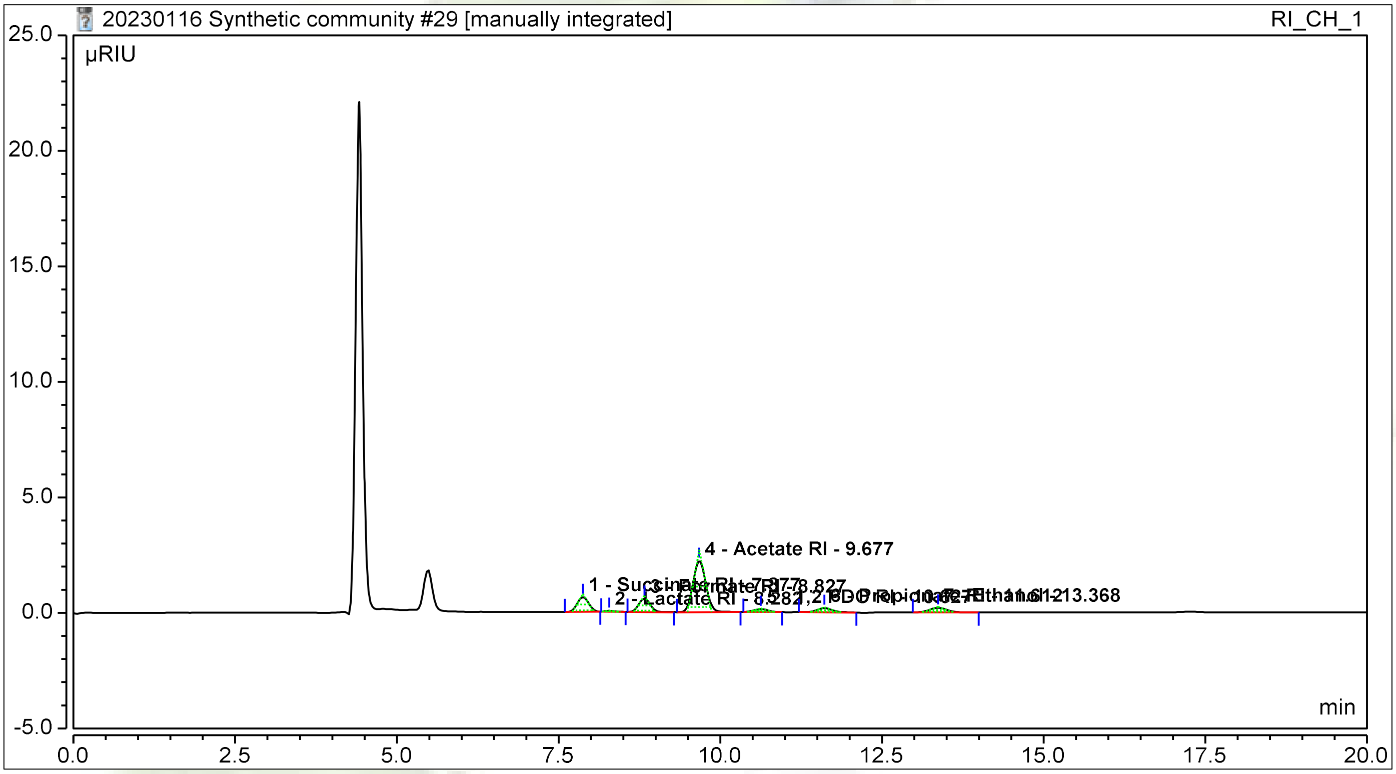

Peak Results

No.	Peak Name	Retention Time min	Width (50%) min	Type	Resolution (EP)	Asymmetry (EP)	Plates (EP)
n.a.	GlcNAc	n.a.	n.a.	n.a.	n.a.	n.a.	n.a.
n.a.	Citrate	n.a.	n.a.	n.a.	n.a.	n.a.	n.a.
n.a.	Glucose	n.a.	n.a.	n.a.	n.a.	n.a.	n.a.
n.a.	Galactose	n.a.	n.a.	n.a.	n.a.	n.a.	n.a.
n.a.	Fucose	n.a.	n.a.	n.a.	n.a.	n.a.	n.a.
1	Succinate RI	7,877	0,197	BMB	1,31	1,02	8860
2	Lactate RI	8,282	0,168	BMB*	1,77	1,42	13490
n.a.	glycerol	n.a.	n.a.	n.a.	n.a.	n.a.	n.a.
3	Formate RI	8,827	0,195	BMB	2,43	1,06	11400
4	Acetate RI	9,677	0,219	BMB	2,42	1,05	10846
5	1,2 PDO RI	10,627	0,244	BMB*	2,34	1,05	10507
n.a.	1,3-PDO	n.a.	n.a.	n.a.	n.a.	n.a.	n.a.
6	Propionate RI	11,612	0,253	BMB*	3,85	1,30	11688
n.a.	1,3-PDO	n.a.	n.a.	n.a.	n.a.	n.a.	n.a.
n.a.	2-3 BDO	n.a.	n.a.	n.a.	n.a.	n.a.	n.a.
7	Ethanol	13,368	0,286	BMB*	n.a.	1,03	12079
n.a.	Isobutyrate RI	n.a.	n.a.	n.a.	n.a.	n.a.	n.a.
n.a.	Butyrate RI	n.a.	n.a.	n.a.	n.a.	n.a.	n.a.

Chromatogram and SST Results

Injection Details

Injection Name:	**GOSFOSEXTR t120 r1**	*Run Time (min):*	**20,00**
Vial Number:	**3:22**	*Injection Volume:*	**10,00**
Injection Type:	**Unknown**	*Channel:*	**RI_CH_1**
Calibration Level:		*Wavelength:*	**n.a.**
Instrument Method:	**Default method LC2030C 45 gr 20 min**	*Bandwidth:*	**n.a.**
Processing Method:	**Processing Method LC2030 45 gr**	*Dilution Factor:*	**1,0000**
Injection Date/Time:	**17/Jan/23 00:44**	*Sample Weight:*	**1,0000**

Chromatogram

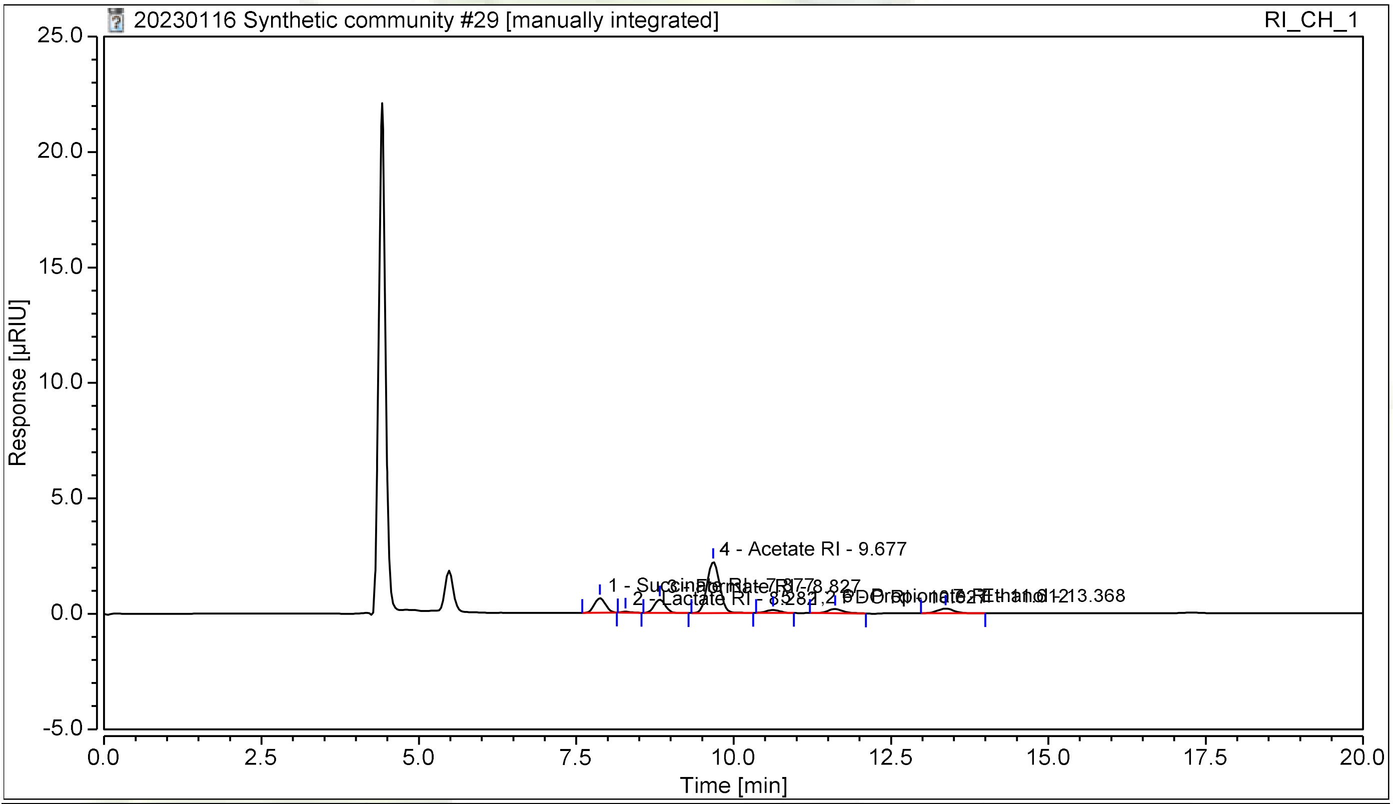

SST Results

No.	Name	Inj.Condition	Peak	Test Result	Injection

**Number of executed test cases:**	**n.a.**	**Total Result:**	**Passed**

Chromatogram and Results

Injection Details

Injection Name:	**GOSFOSEXTR t120 r2**	*Run Time (min):*	**20,00**
Vial Number:	**3:23**	*Injection Volume:*	**10,00**
Injection Type:	**Unknown**	*Channel:*	**RI_CH_1**
Calibration Level:		*Wavelength:*	**n.a.**
Instrument Method:	**Default method LC2030C 45 gr 20 min**	*Bandwidth:*	**n.a.**
Processing Method:	**Processing Method LC2030 45 gr**	*Dilution Factor:*	**1,0000**
Injection Date/Time:	**17/Jan/23 01:05**	*Sample Weight:*	**1,0000**

Chromatogram

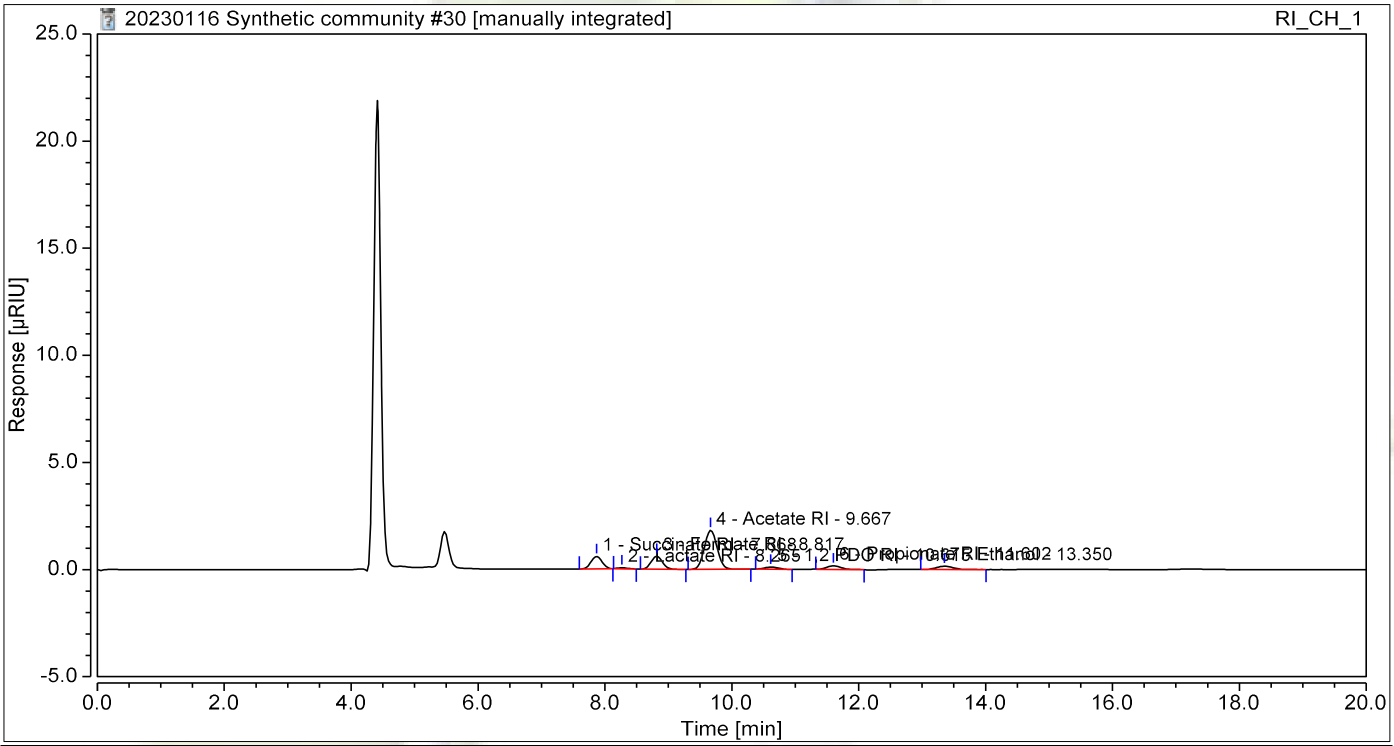

Integration Results

No.	Peak Name	Retention Time min	Area µRIU*min	Height µRIU	Relative Area %	Relative Height %	Amount
n.a.	GlcNAc	n.a.	n.a.	n.a.	n.a.	n.a.	n.a.
n.a.	Citrate	n.a.	n.a.	n.a.	n.a.	n.a.	n.a.
n.a.	Glucose	n.a.	n.a.	n.a.	n.a.	n.a.	n.a.
n.a.	Galactose	n.a.	n.a.	n.a.	n.a.	n.a.	n.a.
n.a.	Fucose	n.a.	n.a.	n.a.	n.a.	n.a.	n.a.
1	Succinate RI	7,868	0,118	0,577	14,99	16,70	2,4450
2	Lactate RI	8,265	0,008	0,047	1,05	1,35	0,2420
n.a.	glycerol	n.a.	n.a.	n.a.	n.a.	n.a.	n.a.
3	Formate RI	8,817	0,124	0,602	15,70	17,43	12,6989
4	Acetate RI	9,667	0,423	1,812	53,52	52,43	26,2278
5	1,2 PDO RI	10,615	0,024	0,098	3,07	2,84	0,7389
n.a.	1,3-PDO	n.a.	n.a.	n.a.	n.a.	n.a.	n.a.
6	Propionate RI	11,602	0,045	0,165	5,68	4,78	1,8779
n.a.	1,3-PDO	n.a.	n.a.	n.a.	n.a.	n.a.	n.a.
n.a.	2-3 BDO	n.a.	n.a.	n.a.	n.a.	n.a.	n.a.
7	Ethanol	13,350	0,047	0,154	5,99	4,47	4,9624
n.a.	Isobutyrate RI	n.a.	n.a.	n.a.	n.a.	n.a.	n.a.
n.a.	Butyrate RI	n.a.	n.a.	n.a.	n.a.	n.a.	n.a.
**Total:**			**0,790**	**3,456**	**100,00**	**100,00**	

Chromeleon (c) Dionex
Version 7.2.10.25868

Peak Analysis

Injection Details

Injection Name:	**GOSFOSEXTR t120 r2**	*Run Time (min):*	**20,00**
Vial Number:	**3:23**	*Injection Volume:*	**10,00**
Injection Type:	**Unknown**	*Channel:*	**RI_CH_1**
Calibration Level:		*Wavelength:*	**n.a.**
Instrument Method:	**Default method LC2030C 45 gr 20 min**	*Bandwidth:*	**n.a.**
Processing Method:	**Processing Method LC2030 45 gr**	*Dilution Factor:*	**1,0000**
Injection Date/Time:	**17/Jan/23 01:05**	*Sample Weight:*	**1,0000**

Chromatogram

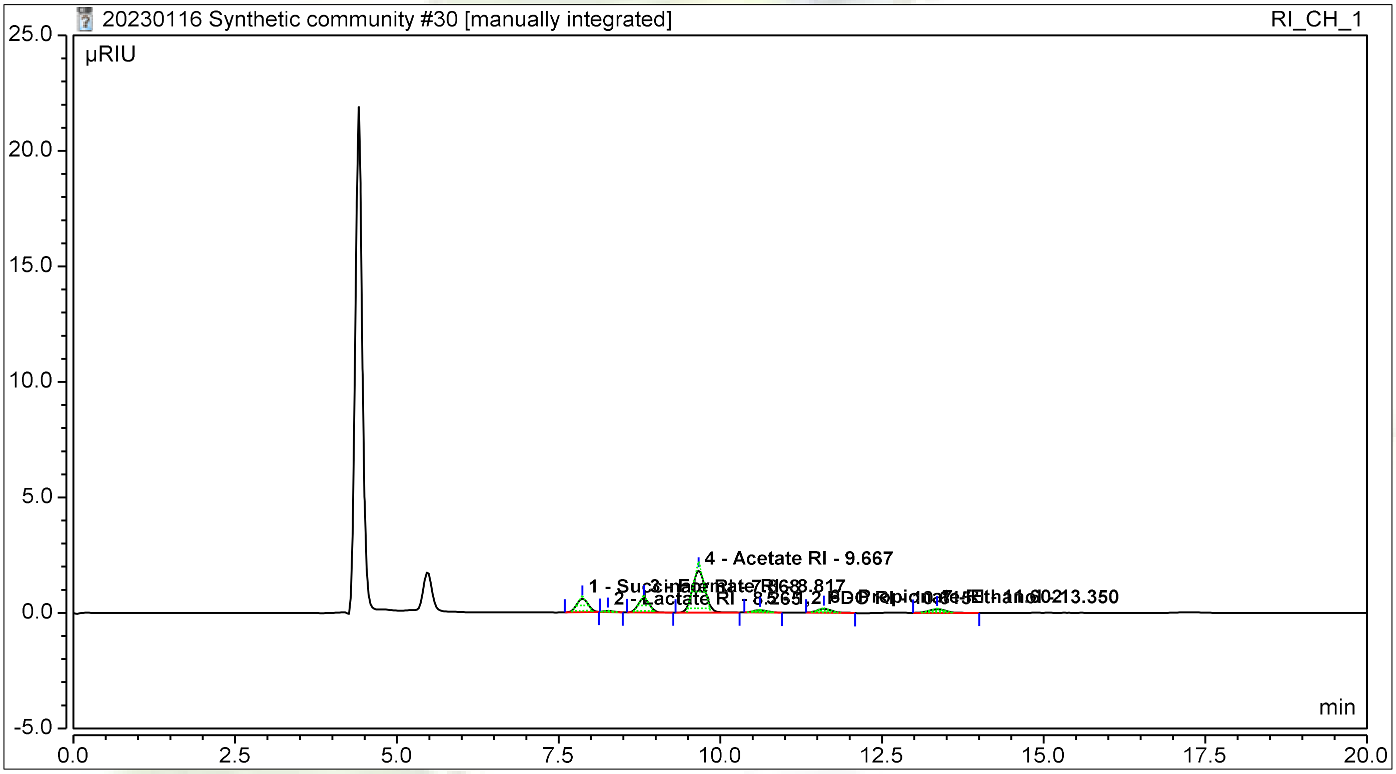

Peak Results

No.	Peak Name	Retention Time<br>min	Width (50%)<br>min	Type	Resolution (EP)	Asymmetry (EP)	Plates (EP)
n.a.	GlcNAc	n.a.	n.a.	n.a.	n.a.	n.a.	n.a.
n.a.	Citrate	n.a.	n.a.	n.a.	n.a.	n.a.	n.a.
n.a.	Glucose	n.a.	n.a.	n.a.	n.a.	n.a.	n.a.
n.a.	Galactose	n.a.	n.a.	n.a.	n.a.	n.a.	n.a.
n.a.	Fucose	n.a.	n.a.	n.a.	n.a.	n.a.	n.a.
1	Succinate RI	7,868	0,196	BMB	1,26	1,01	8911
2	Lactate RI	8,265	0,176	BMB*	1,76	1,30	12281
n.a.	glycerol	n.a.	n.a.	n.a.	n.a.	n.a.	n.a.
3	Formate RI	8,817	0,194	BMB	2,43	1,06	11393
4	Acetate RI	9,667	0,219	BMB	2,44	1,05	10817
5	1,2 PDO RI	10,615	0,239	BMB*	2,41	1,08	10904
n.a.	1,3-PDO	n.a.	n.a.	n.a.	n.a.	n.a.	n.a.
6	Propionate RI	11,602	0,245	BMB*	3,87	1,43	12440
n.a.	1,3-PDO	n.a.	n.a.	n.a.	n.a.	n.a.	n.a.
n.a.	2-3 BDO	n.a.	n.a.	n.a.	n.a.	n.a.	n.a.
7	Ethanol	13,350	0,289	BMB*	n.a.	1,06	11846
n.a.	Isobutyrate RI	n.a.	n.a.	n.a.	n.a.	n.a.	n.a.
n.a.	Butyrate RI	n.a.	n.a.	n.a.	n.a.	n.a.	n.a.

Chromeleon (c) Dionex
Version 7.2.10.25868

Chromatogram and SST Results

Injection Details

Injection Name:	**GOSFOSEXTR t120 r2**	*Run Time (min):*	**20,00**
Vial Number:	**3:23**	*Injection Volume:*	**10,00**
Injection Type:	**Unknown**	*Channel:*	**RI_CH_1**
Calibration Level:		*Wavelength:*	**n.a.**
Instrument Method:	**Default method LC2030C 45 gr 20 min**	*Bandwidth:*	**n.a.**
Processing Method:	**Processing Method LC2030 45 gr**	*Dilution Factor:*	**1,0000**
Injection Date/Time:	**17/Jan/23 01:05**	*Sample Weight:*	**1,0000**

Chromatogram

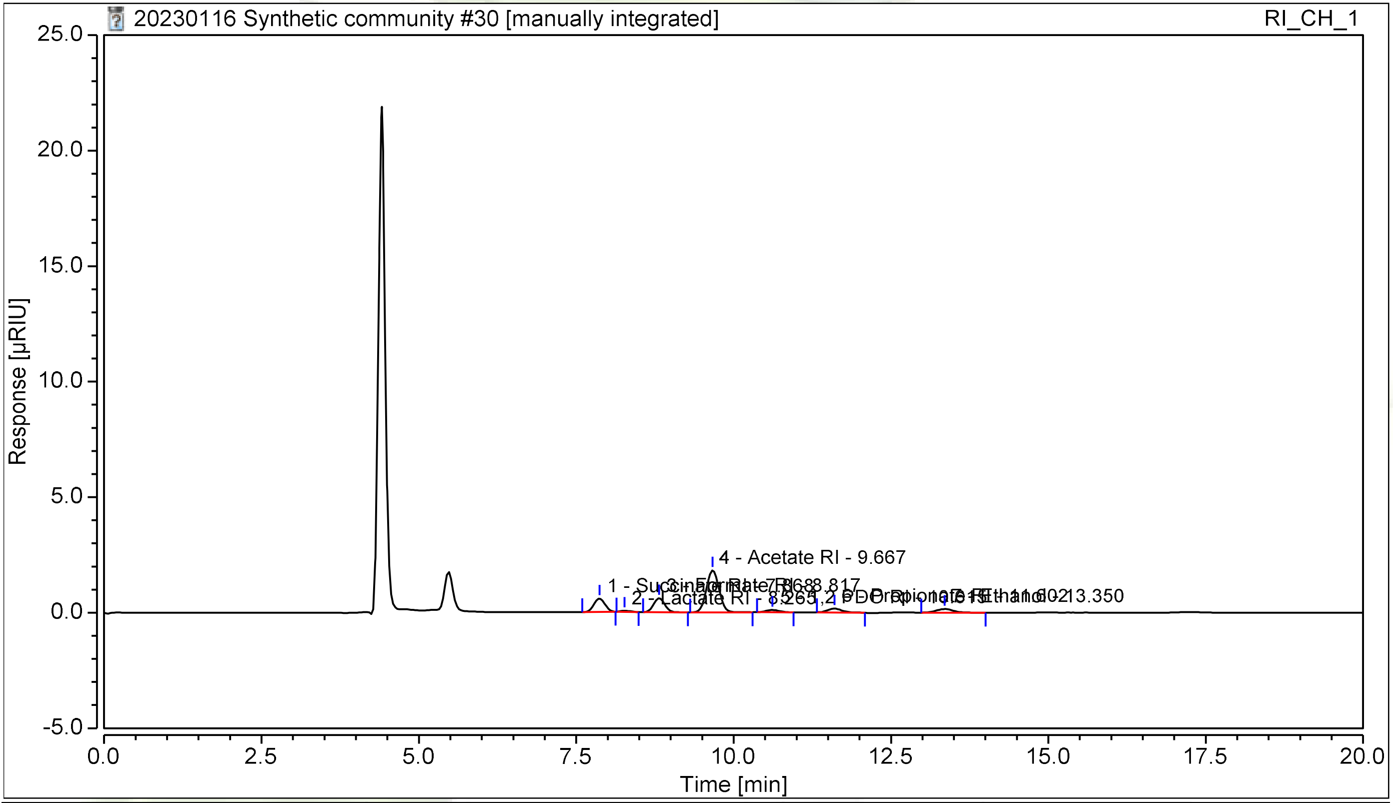

SST Results

No.	Name	Inj.Condition	Peak	Test Result	Injection

Number of executed test cases: **n.a.** **Total Result:** **Passed**

Chromatogram and Results

Injection Details

Injection Name:	**GOSFOSEXTR t120 r3**	*Run Time (min):*	**20,00**
Vial Number:	**3:24**	*Injection Volume:*	**10,00**
Injection Type:	**Unknown**	*Channel:*	**RI_CH_1**
Calibration Level:		*Wavelength:*	**n.a.**
Instrument Method:	**Default method LC2030C 45 gr 20 min**	*Bandwidth:*	**n.a.**
Processing Method:	**Processing Method LC2030 45 gr**	*Dilution Factor:*	**1,0000**
Injection Date/Time:	**17/Jan/23 01:25**	*Sample Weight:*	**1,0000**

Chromatogram

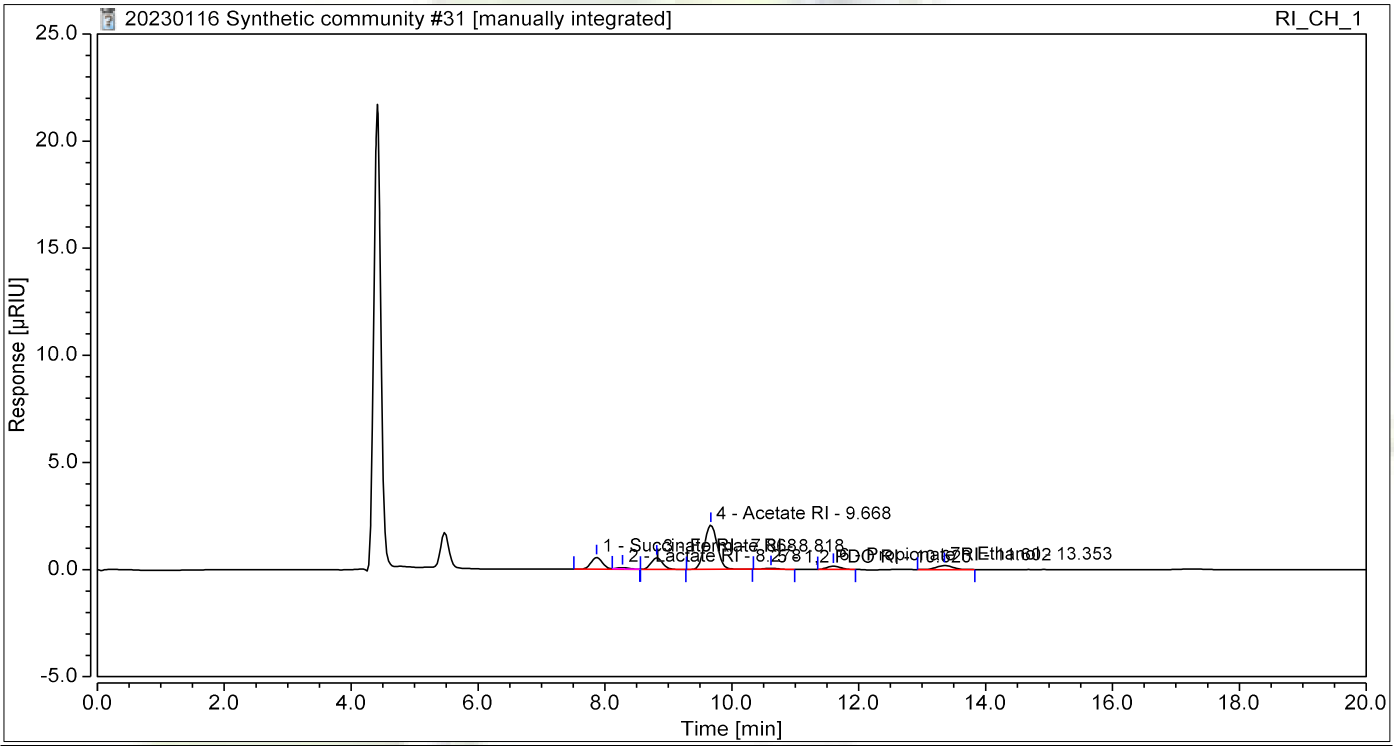

Integration Results

No.	Peak Name	Retention Time min	Area µRIU*min	Height µRIU	Relative Area %	Relative Height %	Amount
n.a.	GlcNAc	n.a.	n.a.	n.a.	n.a.	n.a.	n.a.
n.a.	Citrate	n.a.	n.a.	n.a.	n.a.	n.a.	n.a.
n.a.	Glucose	n.a.	n.a.	n.a.	n.a.	n.a.	n.a.
n.a.	Galactose	n.a.	n.a.	n.a.	n.a.	n.a.	n.a.
n.a.	Fucose	n.a.	n.a.	n.a.	n.a.	n.a.	n.a.
1	Succinate RI	7,868	0,122	0,544	14,75	15,23	2,5222
2	Lactate RI	8,278	0,011	0,058	1,32	1,62	0,3202
n.a.	glycerol	n.a.	n.a.	n.a.	n.a.	n.a.	n.a.
3	Formate RI	8,818	0,112	0,538	13,55	15,07	11,4919
4	Acetate RI	9,668	0,479	2,057	57,85	57,59	29,7258
5	1,2 PDO RI	10,620	0,012	0,044	1,43	1,24	0,3605
n.a.	1,3-PDO	n.a.	n.a.	n.a.	n.a.	n.a.	n.a.
6	Propionate RI	11,602	0,035	0,144	4,26	4,03	1,4775
n.a.	1,3-PDO	n.a.	n.a.	n.a.	n.a.	n.a.	n.a.
n.a.	2-3 BDO	n.a.	n.a.	n.a.	n.a.	n.a.	n.a.
7	Ethanol	13,353	0,057	0,187	6,84	5,23	5,9421
n.a.	Isobutyrate RI	n.a.	n.a.	n.a.	n.a.	n.a.	n.a.
n.a.	Butyrate RI	n.a.	n.a.	n.a.	n.a.	n.a.	n.a.
**Total:**			**0,829**	**3,572**	**100,00**	**100,00**	

Chromeleon (c) Dionex
Version 7.2.10.25868

Peak Analysis

Injection Details

Injection Name:	**GOSFOSEXTR t120 r3**	*Run Time (min):*	**20,00**
Vial Number:	**3:24**	*Injection Volume:*	**10,00**
Injection Type:	**Unknown**	*Channel:*	**RI_CH_1**
Calibration Level:		*Wavelength:*	**n.a.**
Instrument Method:	**Default method LC2030C 45 gr 20 min**	*Bandwidth:*	**n.a.**
Processing Method:	**Processing Method LC2030 45 gr**	*Dilution Factor:*	**1,0000**
Injection Date/Time:	**17/Jan/23 01:25**	*Sample Weight:*	**1,0000**

Chromatogram

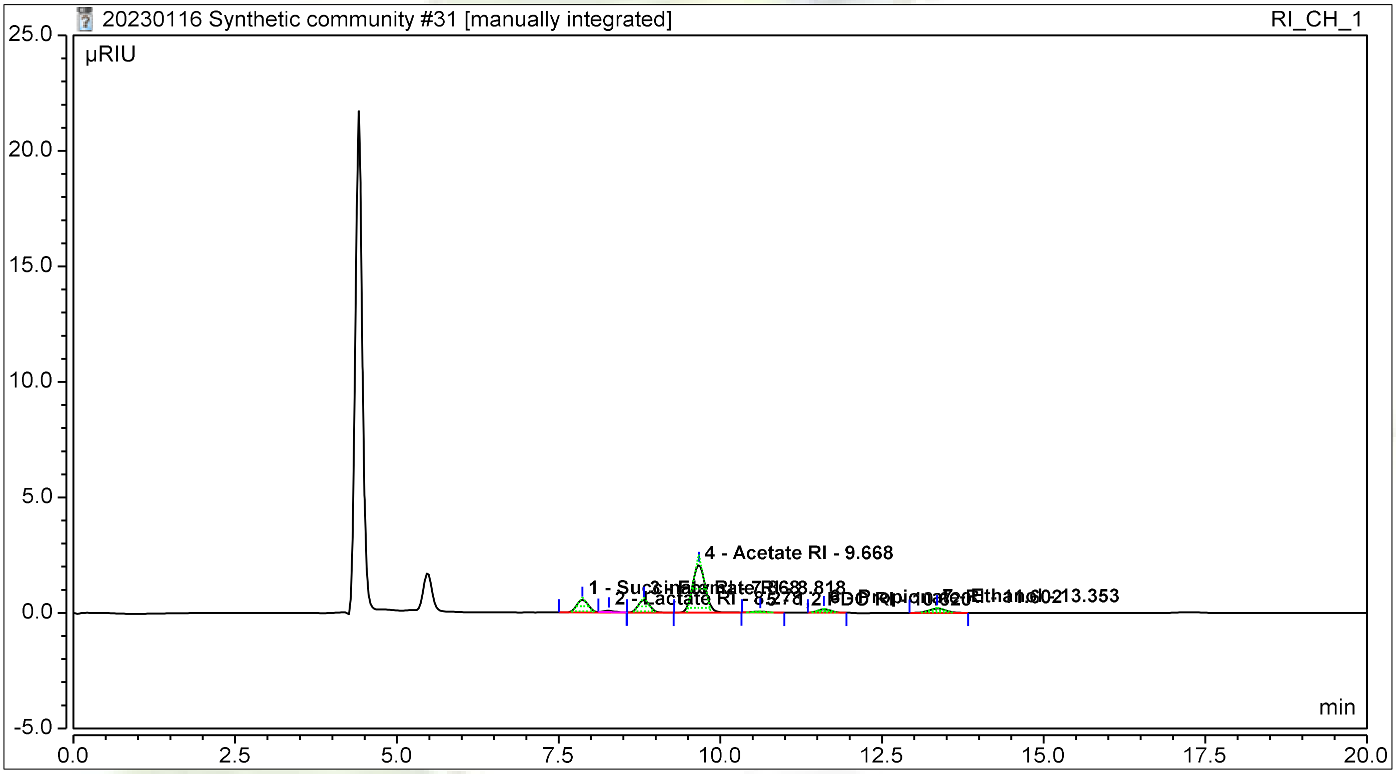

Peak Results

No.	Peak Name	Retention Time min	Width (50%) min	Type	Resolution (EP)	Asymmetry (EP)	Plates (EP)
n.a.	GlcNAc	n.a.	n.a.	n.a.	n.a.	n.a.	n.a.
n.a.	Citrate	n.a.	n.a.	n.a.	n.a.	n.a.	n.a.
n.a.	Glucose	n.a.	n.a.	n.a.	n.a.	n.a.	n.a.
n.a.	Galactose	n.a.	n.a.	n.a.	n.a.	n.a.	n.a.
n.a.	Fucose	n.a.	n.a.	n.a.	n.a.	n.a.	n.a.
1	Succinate RI	7,868	0,200	BM	2,84	1,10	8616
2	Lactate RI	8,278	n.a.	Rd	n.a.	n.a.	n.a.
n.a.	glycerol	n.a.	n.a.	n.a.	n.a.	n.a.	n.a.
3	Formate RI	8,818	0,195	MB	2,42	1,05	11274
4	Acetate RI	9,668	0,219	BMB	2,39	1,05	10847
5	1,2 PDO RI	10,620	0,252	BMB*	2,37	1,05	9858
n.a.	1,3-PDO	n.a.	n.a.	n.a.	n.a.	n.a.	n.a.
6	Propionate RI	11,602	0,236	BMB*	3,95	1,10	13339
n.a.	1,3-PDO	n.a.	n.a.	n.a.	n.a.	n.a.	n.a.
n.a.	2-3 BDO	n.a.	n.a.	n.a.	n.a.	n.a.	n.a.
7	Ethanol	13,353	0,287	BMB*	n.a.	1,03	11992
n.a.	Isobutyrate RI	n.a.	n.a.	n.a.	n.a.	n.a.	n.a.
n.a.	Butyrate RI	n.a.	n.a.	n.a.	n.a.	n.a.	n.a.

Chromatogram and SST Results

Injection Details

Injection Name:	**GOSFOSEXTR t120 r3**	*Run Time (min):*	**20,00**
Vial Number:	**3:24**	*Injection Volume:*	**10,00**
Injection Type:	**Unknown**	*Channel:*	**RI_CH_1**
Calibration Level:		*Wavelength:*	**n.a.**
Instrument Method:	**Default method LC2030C 45 gr 20 min**	*Bandwidth:*	**n.a.**
Processing Method:	**Processing Method LC2030 45 gr**	*Dilution Factor:*	**1,0000**
Injection Date/Time:	**17/Jan/23 01:25**	*Sample Weight:*	**1,0000**

Chromatogram

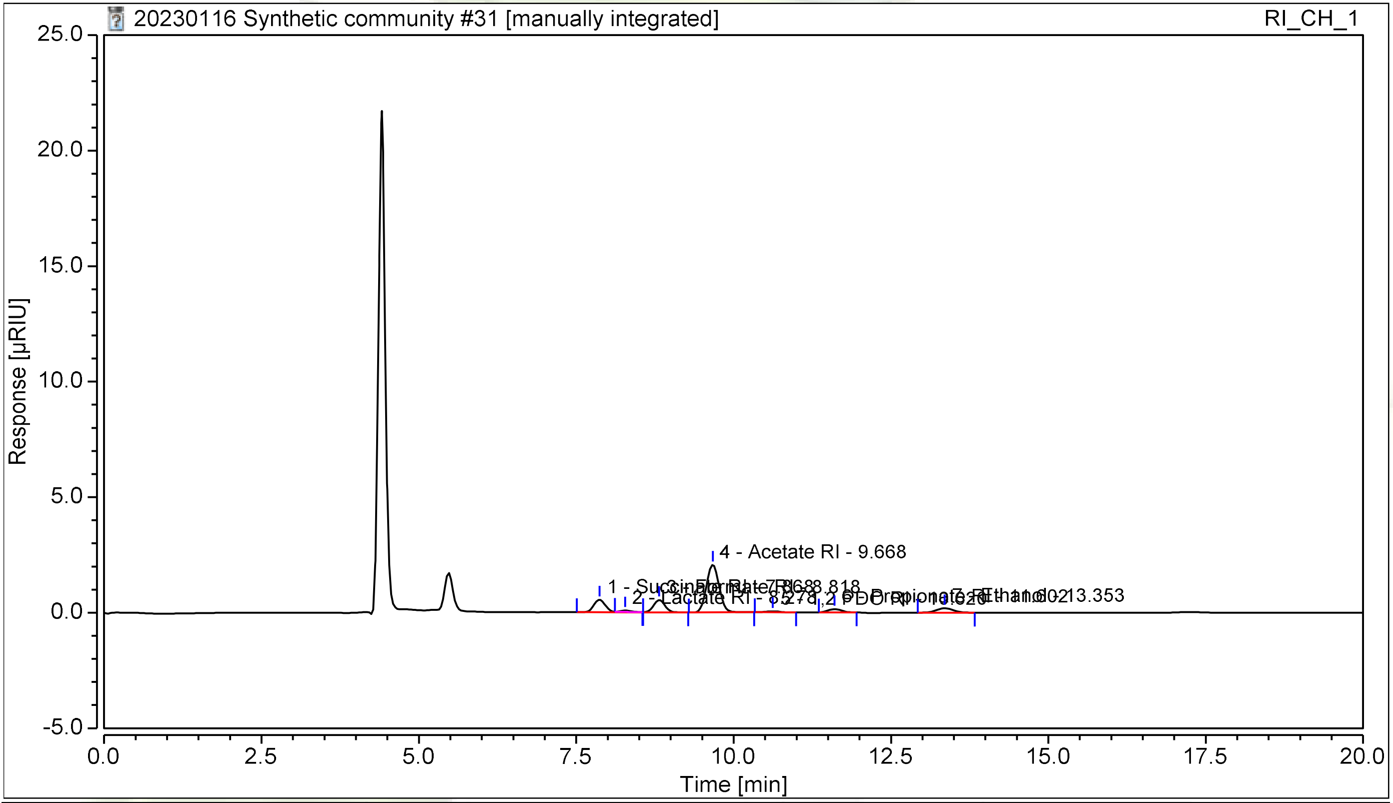

SST Results

No.	Name	Inj.Condition	Peak	Test Result	Injection
**Number of executed test cases:**	**n.a.**		**Total Result:**	**Passed**	

Chromeleon (c) Dionex
Version 7.2.10.25868

Chromatogram and Results

Injection Details

Injection Name:	**MUCHMO1 t24 r1**	*Run Time (min):*	**20,00**
Vial Number:	**3:25**	*Injection Volume:*	**10,00**
Injection Type:	**Unknown**	*Channel:*	**RI_CH_1**
Calibration Level:		*Wavelength:*	**n.a.**
Instrument Method:	**Default method LC2030C 45 gr 20 min**	*Bandwidth:*	**n.a.**
Processing Method:	**Processing Method LC2030 45 gr**	*Dilution Factor:*	**1,0000**
Injection Date/Time:	**17/Jan/23 01:45**	*Sample Weight:*	**1,0000**

Chromatogram

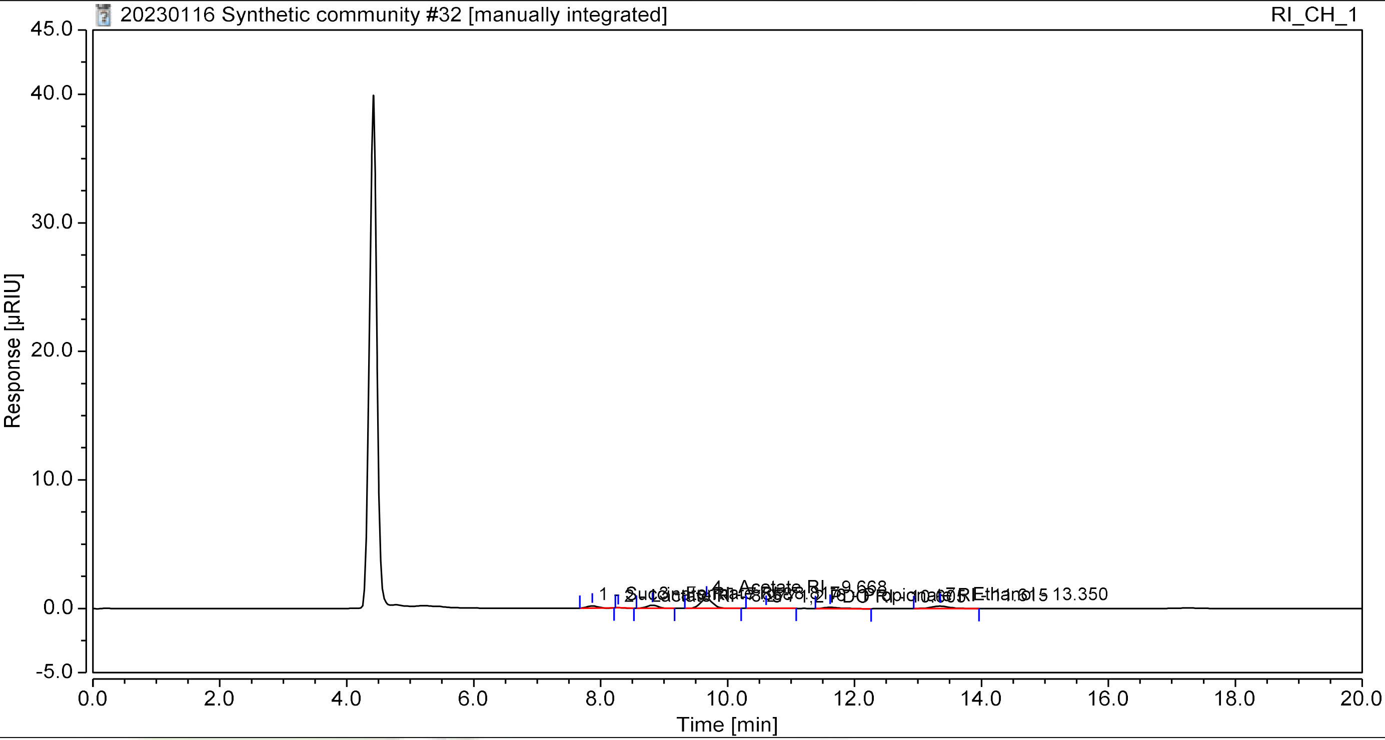

Integration Results

No.	Peak Name	Retention Time min	Area µRIU*min	Height µRIU	Relative Area %	Relative Height %	Amount
n.a.	GlcNAc	n.a.	n.a.	n.a.	n.a.	n.a.	n.a.
n.a.	Citrate	n.a.	n.a.	n.a.	n.a.	n.a.	n.a.
n.a.	Glucose	n.a.	n.a.	n.a.	n.a.	n.a.	n.a.
n.a.	Galactose	n.a.	n.a.	n.a.	n.a.	n.a.	n.a.
n.a.	Fucose	n.a.	n.a.	n.a.	n.a.	n.a.	n.a.
1	Succinate RI	7,868	0,028	0,161	8,25	11,28	0,5714
2	Lactate RI	8,277	0,001	0,011	0,27	0,76	0,0261
n.a.	glycerol	n.a.	n.a.	n.a.	n.a.	n.a.	n.a.
3	Formate RI	8,817	0,048	0,236	14,44	16,54	4,9589
4	Acetate RI	9,668	0,170	0,732	50,70	51,21	10,5491
5	1,2 PDO RI	10,605	0,002	0,006	0,70	0,39	0,0714
n.a.	1,3-PDO	n.a.	n.a.	n.a.	n.a.	n.a.	n.a.
6	Propionate RI	11,615	0,031	0,105	9,37	7,35	1,3145
n.a.	1,3-PDO	n.a.	n.a.	n.a.	n.a.	n.a.	n.a.
n.a.	2-3 BDO	n.a.	n.a.	n.a.	n.a.	n.a.	n.a.
7	Ethanol	13,350	0,055	0,178	16,28	12,47	5,7311
n.a.	Isobutyrate RI	n.a.	n.a.	n.a.	n.a.	n.a.	n.a.
n.a.	Butyrate RI	n.a.	n.a.	n.a.	n.a.	n.a.	n.a.
**Total:**			**0,336**	**1,429**	**100,00**	**100,00**	

Chromeleon (c) Dionex
Version 7.2.10.25868

Peak Analysis

Injection Details

Injection Name:	**MUCHMO1 t24 r1**	*Run Time (min):*	**20,00**
Vial Number:	**3:25**	*Injection Volume:*	**10,00**
Injection Type:	**Unknown**	*Channel:*	**RI_CH_1**
Calibration Level:		*Wavelength:*	**n.a.**
Instrument Method:	**Default method LC2030C 45 gr 20 min**	*Bandwidth:*	**n.a.**
Processing Method:	**Processing Method LC2030 45 gr**	*Dilution Factor:*	**1,0000**
Injection Date/Time:	**17/Jan/23 01:45**	*Sample Weight:*	**1,0000**

Chromatogram

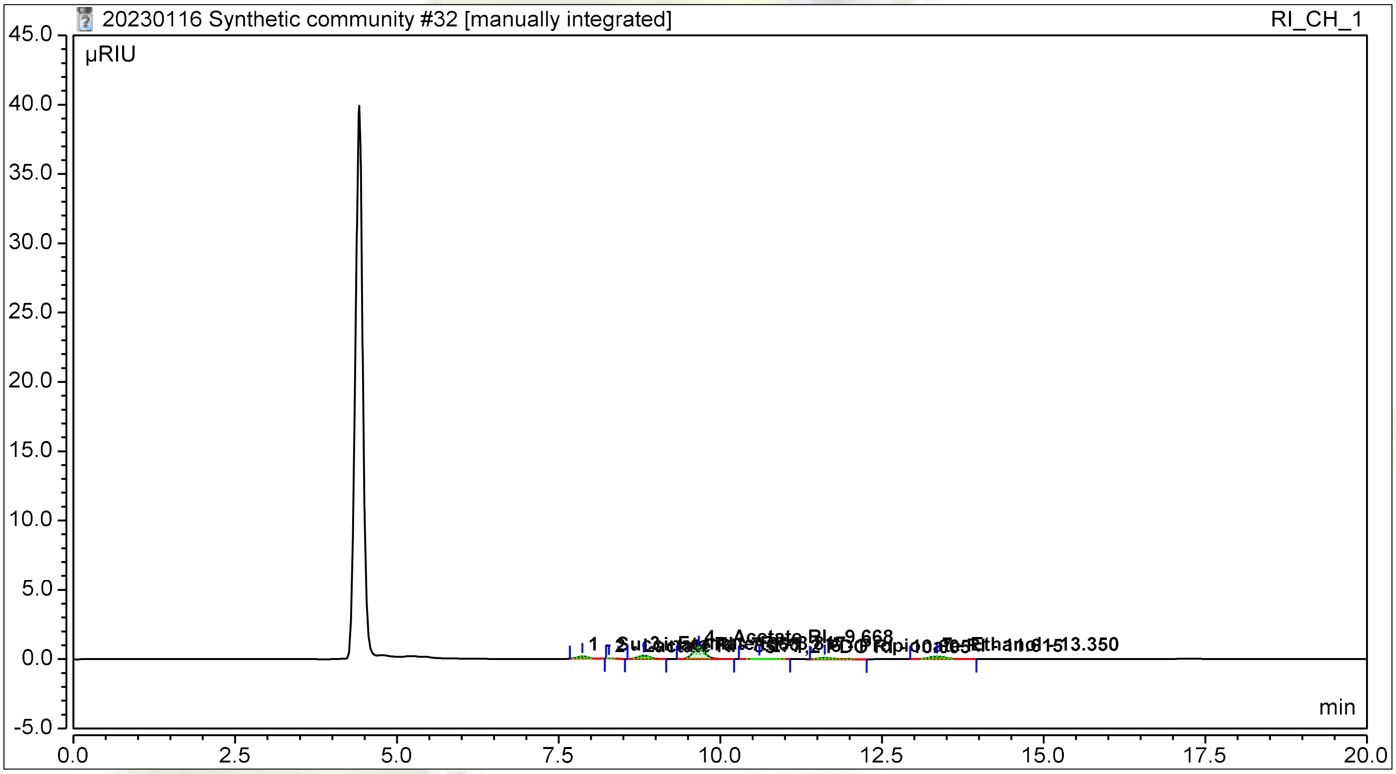

Peak Results

No.	Peak Name	Retention Time min	Width (50%) min	Type	Resolution (EP)	Asymmetry (EP)	Plates (EP)
n.a.	GlcNAc	n.a.	n.a.	n.a.	n.a.	n.a.	n.a.
n.a.	Citrate	n.a.	n.a.	n.a.	n.a.	n.a.	n.a.
n.a.	Glucose	n.a.	n.a.	n.a.	n.a.	n.a.	n.a.
n.a.	Galactose	n.a.	n.a.	n.a.	n.a.	n.a.	n.a.
n.a.	Fucose	n.a.	n.a.	n.a.	n.a.	n.a.	n.a.
1	Succinate RI	7,868	0,184	BMB*	1,68	0,95	10111
2	Lactate RI	8,277	0,103	BMB*	2,15	1,77	35987
n.a.	glycerol	n.a.	n.a.	n.a.	n.a.	n.a.	n.a.
3	Formate RI	8,817	0,194	BMB*	2,44	1,06	11451
4	Acetate RI	9,668	0,218	BMB	1,66	1,04	10864
5	1,2 PDO RI	10,605	0,447	BMB*	1,71	1,56	3124
n.a.	1,3-PDO	n.a.	n.a.	n.a.	n.a.	n.a.	n.a.
6	Propionate RI	11,615	0,251	BMB*	3,80	1,75	11868
n.a.	1,3-PDO	n.a.	n.a.	n.a.	n.a.	n.a.	n.a.
n.a.	2-3 BDO	n.a.	n.a.	n.a.	n.a.	n.a.	n.a.
7	Ethanol	13,350	0,289	BMB*	n.a.	1,04	11862
n.a.	Isobutyrate RI	n.a.	n.a.	n.a.	n.a.	n.a.	n.a.
n.a.	Butyrate RI	n.a.	n.a.	n.a.	n.a.	n.a.	n.a.

Chromeleon (c) Dionex
Version 7.2.10.25868

Chromatogram and SST Results

Injection Details

Injection Name:	**MUCHMO1 t24 r1**	*Run Time (min):*	**20,00**
Vial Number:	**3:25**	*Injection Volume:*	**10,00**
Injection Type:	**Unknown**	*Channel:*	**RI_CH_1**
Calibration Level:		*Wavelength:*	**n.a.**
Instrument Method:	**Default method LC2030C 45 gr 20 min**	*Bandwidth:*	**n.a.**
Processing Method:	**Processing Method LC2030 45 gr**	*Dilution Factor:*	**1,0000**
Injection Date/Time:	**17/Jan/23 01:45**	*Sample Weight:*	**1,0000**

Chromatogram

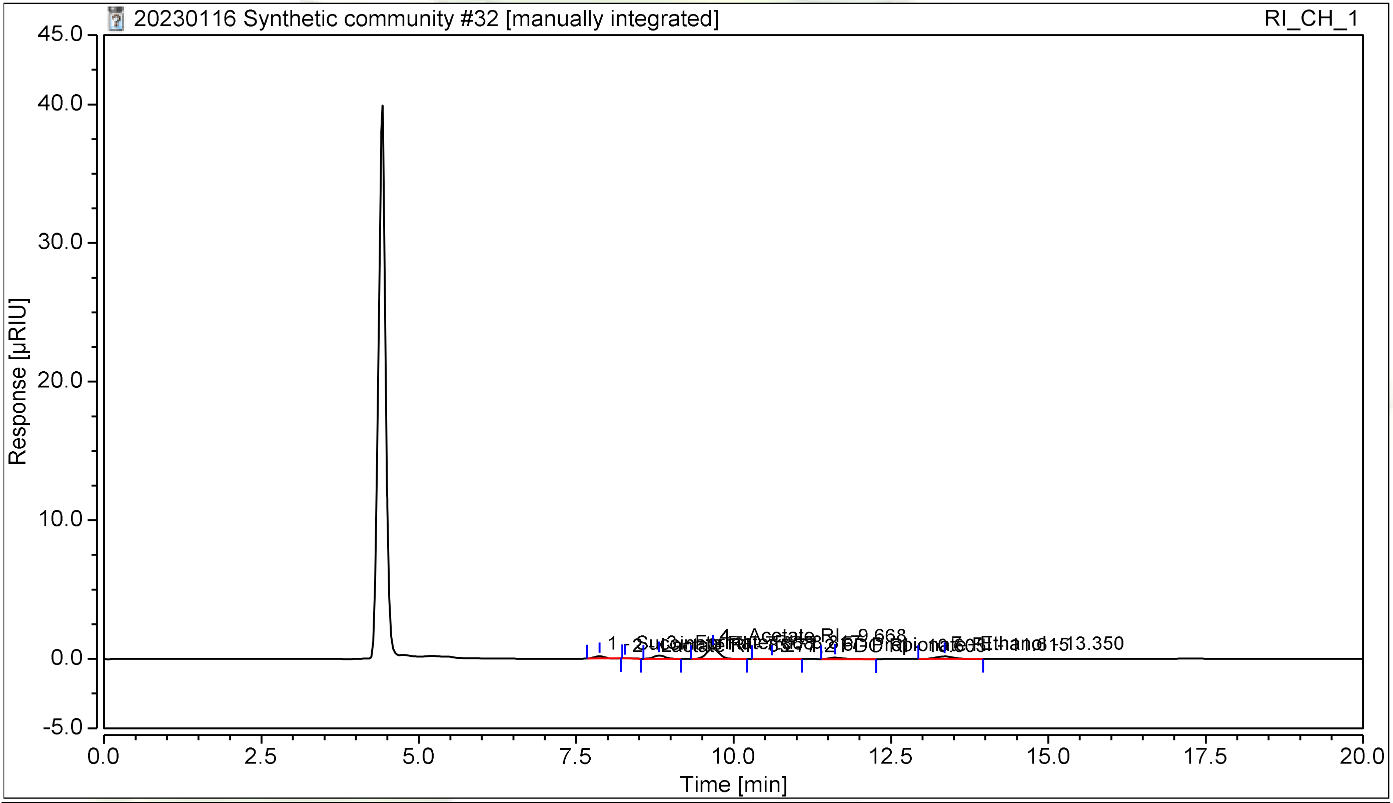

SST Results

No.	Name	Inj.Condition	Peak	Test Result	Injection

Number of executed test cases: **n.a.** **Total Result:** **Passed**

Chromatogram and Results

Injection Details

Injection Name:	**MUCHMO1 t24 r2**	*Run Time (min):*	**20,00**
Vial Number:	**3:26**	*Injection Volume:*	**10,00**
Injection Type:	**Unknown**	*Channel:*	**RI_CH_1**
Calibration Level:		*Wavelength:*	**n.a.**
Instrument Method:	**Default method LC2030C 45 gr 20 min**	*Bandwidth:*	**n.a.**
Processing Method:	**Processing Method LC2030 45 gr**	*Dilution Factor:*	**1,0000**
Injection Date/Time:	**17/Jan/23 02:06**	*Sample Weight:*	**1,0000**

Chromatogram

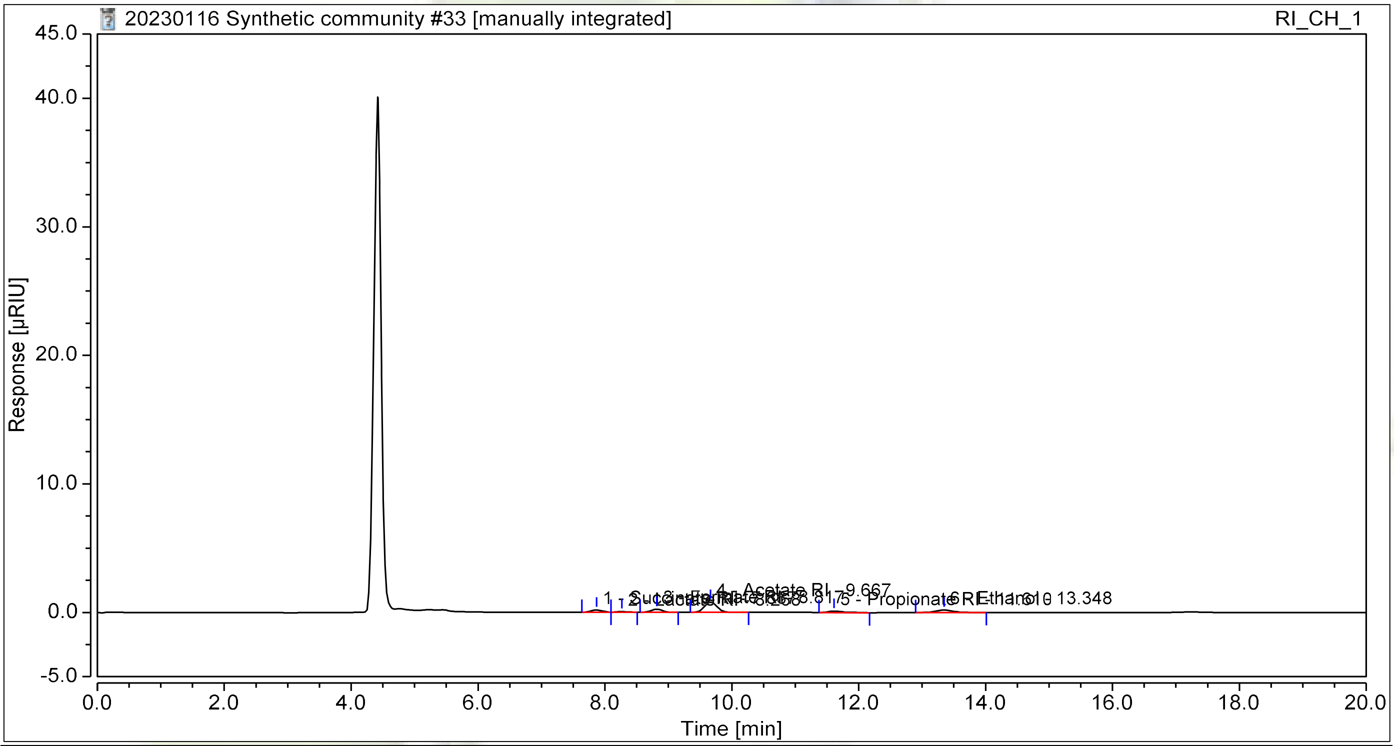

Integration Results

No.	Peak Name	Retention Time min	Area µRIU*min	Height µRIU	Relative Area %	Relative Height %	Amount
n.a.	GlcNAc	n.a.	n.a.	n.a.	n.a.	n.a.	n.a.
n.a.	Citrate	n.a.	n.a.	n.a.	n.a.	n.a.	n.a.
n.a.	Glucose	n.a.	n.a.	n.a.	n.a.	n.a.	n.a.
n.a.	Galactose	n.a.	n.a.	n.a.	n.a.	n.a.	n.a.
n.a.	Fucose	n.a.	n.a.	n.a.	n.a.	n.a.	n.a.
1	Succinate RI	7,867	0,034	0,170	9,32	11,00	0,7093
2	Lactate RI	8,268	0,008	0,042	2,23	2,74	0,2408
n.a.	glycerol	n.a.	n.a.	n.a.	n.a.	n.a.	n.a.
3	Formate RI	8,817	0,049	0,239	13,23	15,41	4,9940
4	Acetate RI	9,667	0,185	0,794	50,04	51,28	11,4424
n.a.	1,2 PDO RI	n.a.	n.a.	n.a.	n.a.	n.a.	n.a.
n.a.	1,3-PDO	n.a.	n.a.	n.a.	n.a.	n.a.	n.a.
5	Propionate RI	11,610	0,036	0,115	9,69	7,45	1,4937
n.a.	1,3-PDO	n.a.	n.a.	n.a.	n.a.	n.a.	n.a.
n.a.	2-3 BDO	n.a.	n.a.	n.a.	n.a.	n.a.	n.a.
6	Ethanol	13,348	0,057	0,187	15,49	12,11	5,9914
n.a.	Isobutyrate RI	n.a.	n.a.	n.a.	n.a.	n.a.	n.a.
n.a.	Butyrate RI	n.a.	n.a.	n.a.	n.a.	n.a.	n.a.
**Total:**			**0,369**	**1,548**	**100,00**	**100,00**	

Chromeleon (c) Dionex
Version 7.2.10.25868

Peak Analysis

Injection Details

Injection Name:	**MUCHMO1 t24 r2**	*Run Time (min):*	**20,00**
Vial Number:	**3:26**	*Injection Volume:*	**10,00**
Injection Type:	**Unknown**	*Channel:*	**RI_CH_1**
Calibration Level:		*Wavelength:*	**n.a.**
Instrument Method:	**Default method LC2030C 45 gr 20 min**	*Bandwidth:*	**n.a.**
Processing Method:	**Processing Method LC2030 45 gr**	*Dilution Factor:*	**1,0000**
Injection Date/Time:	**17/Jan/23 02:06**	*Sample Weight:*	**1,0000**

Chromatogram

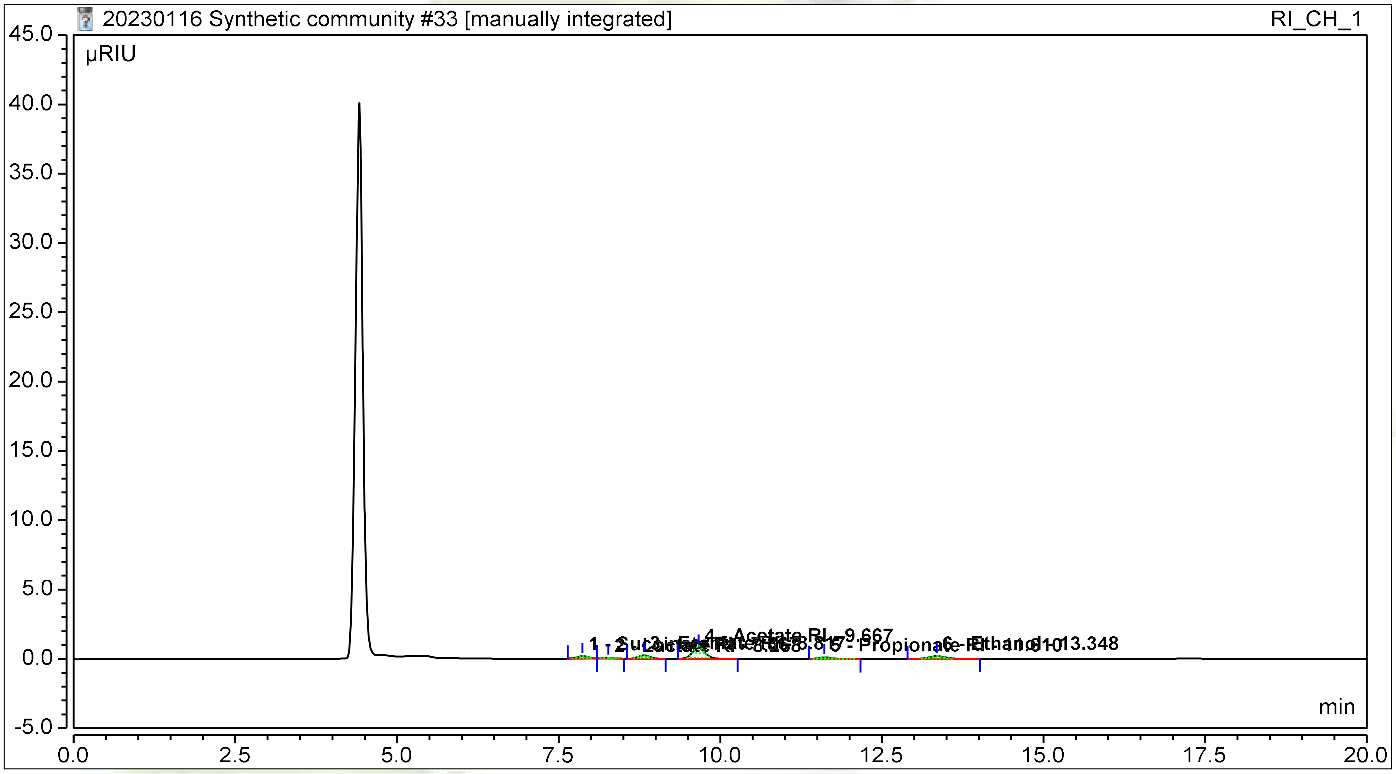

Peak Results

No.	Peak Name	Retention Time min	Width (50%) min	Type	Resolution (EP)	Asymmetry (EP)	Plates (EP)
n.a.	GlcNAc	n.a.	n.a.	n.a.	n.a.	n.a.	n.a.
n.a.	Citrate	n.a.	n.a.	n.a.	n.a.	n.a.	n.a.
n.a.	Glucose	n.a.	n.a.	n.a.	n.a.	n.a.	n.a.
n.a.	Galactose	n.a.	n.a.	n.a.	n.a.	n.a.	n.a.
n.a.	Fucose	n.a.	n.a.	n.a.	n.a.	n.a.	n.a.
1	Succinate RI	7,867	0,195	BMB*	1,24	1,00	9038
2	Lactate RI	8,268	0,187	BMB*	1,70	1,24	10803
n.a.	glycerol	n.a.	n.a.	n.a.	n.a.	n.a.	n.a.
3	Formate RI	8,817	0,194	BMB*	2,43	1,06	11466
4	Acetate RI	9,667	0,218	BMB	4,88	1,05	10868
n.a.	1,2 PDO RI	n.a.	n.a.	n.a.	n.a.	n.a.	n.a.
n.a.	1,3-PDO	n.a.	n.a.	n.a.	n.a.	n.a.	n.a.
5	Propionate RI	11,610	0,252	BMB*	3,82	1,80	11792
n.a.	1,3-PDO	n.a.	n.a.	n.a.	n.a.	n.a.	n.a.
n.a.	2-3 BDO	n.a.	n.a.	n.a.	n.a.	n.a.	n.a.
6	Ethanol	13,348	0,285	BMB*	n.a.	1,04	12127
n.a.	Isobutyrate RI	n.a.	n.a.	n.a.	n.a.	n.a.	n.a.
n.a.	Butyrate RI	n.a.	n.a.	n.a.	n.a.	n.a.	n.a.

Chromeleon (c) Dionex
Version 7.2.10.25868

Chromatogram and SST Results

Injection Details

Injection Name:	**MUCHMO1 t24 r2**	*Run Time (min):*	**20,00**
Vial Number:	**3:26**	*Injection Volume:*	**10,00**
Injection Type:	**Unknown**	*Channel:*	**RI_CH_1**
Calibration Level:		*Wavelength:*	**n.a.**
Instrument Method:	**Default method LC2030C 45 gr 20 min**	*Bandwidth:*	**n.a.**
Processing Method:	**Processing Method LC2030 45 gr**	*Dilution Factor:*	**1,0000**
Injection Date/Time:	**17/Jan/23 02:06**	*Sample Weight:*	**1,0000**

Chromatogram

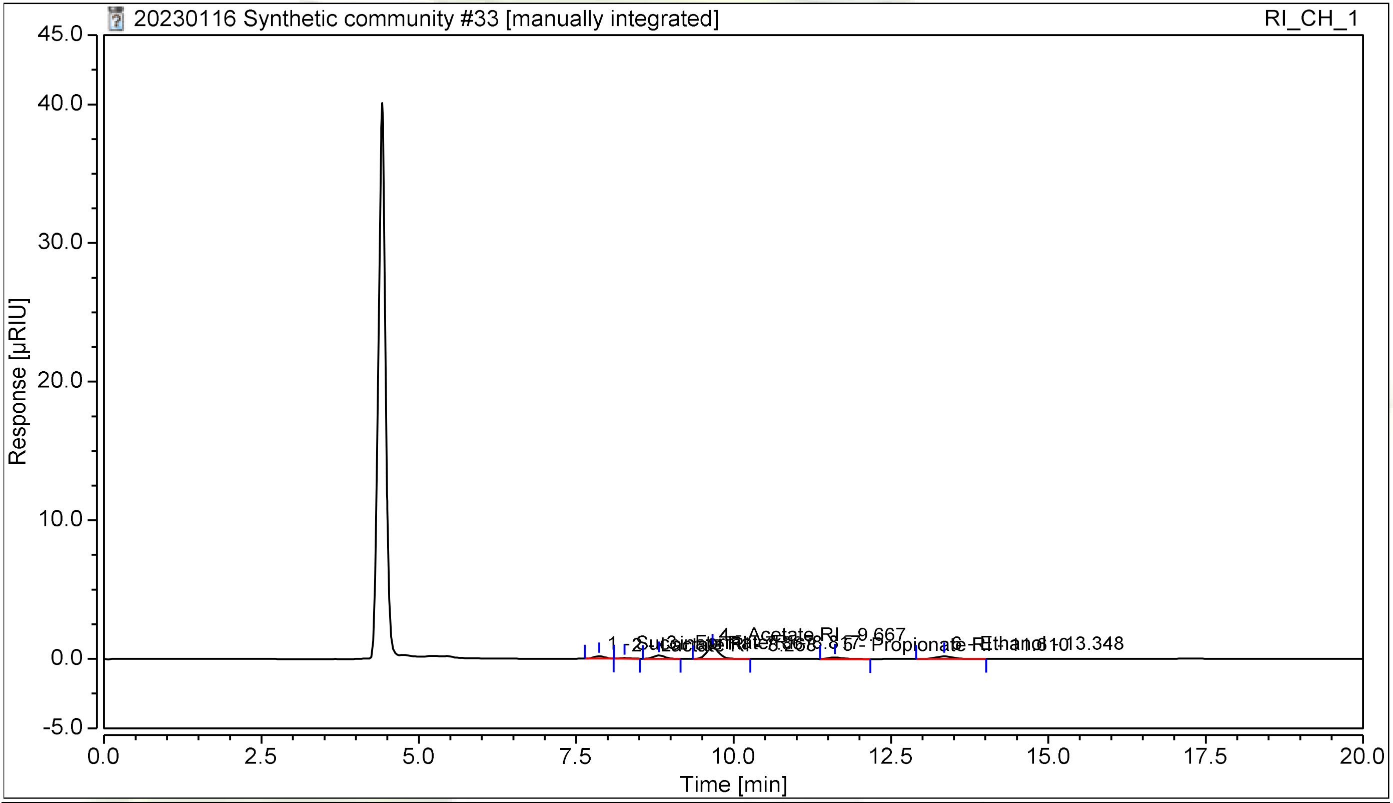

SST Results

No.	Name	Inj.Condition	Peak	Test Result	Injection
**Number of executed test cases:**	**n.a.**		**Total Result:**	**Passed**	

Chromatogram and Results

Injection Details

Injection Name:	**MUCHMO1 t24 r3**	*Run Time (min):*	**20,00**
Vial Number:	**3:27**	*Injection Volume:*	**10,00**
Injection Type:	**Unknown**	*Channel:*	**RI_CH_1**
Calibration Level:		*Wavelength:*	**n.a.**
Instrument Method:	**Default method LC2030C 45 gr 20 min**	*Bandwidth:*	**n.a.**
Processing Method:	**Processing Method LC2030 45 gr**	*Dilution Factor:*	**1,0000**
Injection Date/Time:	**17/Jan/23 02:26**	*Sample Weight:*	**1,0000**

Chromatogram

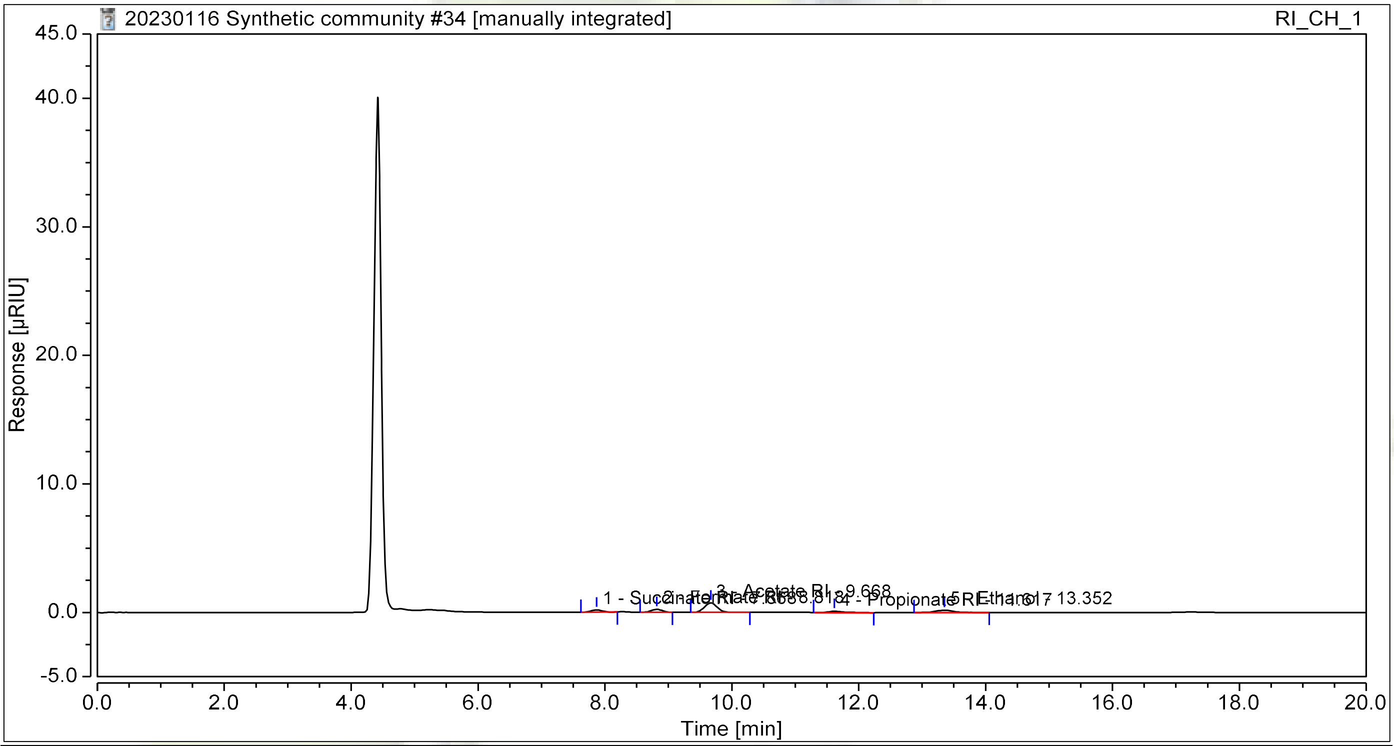

Integration Results

No.	Peak Name	Retention Time min	Area µRIU*min	Height µRIU	Relative Area %	Relative Height %	Amount
n.a.	GlcNAc	n.a.	n.a.	n.a.	n.a.	n.a.	n.a.
n.a.	Citrate	n.a.	n.a.	n.a.	n.a.	n.a.	n.a.
n.a.	Glucose	n.a.	n.a.	n.a.	n.a.	n.a.	n.a.
n.a.	Galactose	n.a.	n.a.	n.a.	n.a.	n.a.	n.a.
n.a.	Fucose	n.a.	n.a.	n.a.	n.a.	n.a.	n.a.
1	Succinate RI	7,868	0,031	0,169	9,45	12,06	0,6388
n.a.	Lactate RI	n.a.	n.a.	n.a.	n.a.	n.a.	n.a.
n.a.	glycerol	n.a.	n.a.	n.a.	n.a.	n.a.	n.a.
2	Formate RI	8,818	0,046	0,228	14,05	16,21	4,7071
3	Acetate RI	9,668	0,173	0,741	52,70	52,80	10,7005
n.a.	1,2 PDO RI	n.a.	n.a.	n.a.	n.a.	n.a.	n.a.
n.a.	1,3-PDO	n.a.	n.a.	n.a.	n.a.	n.a.	n.a.
4	Propionate RI	11,617	0,026	0,094	8,03	6,68	1,0992
n.a.	1,3-PDO	n.a.	n.a.	n.a.	n.a.	n.a.	n.a.
n.a.	2-3 BDO	n.a.	n.a.	n.a.	n.a.	n.a.	n.a.
5	Ethanol	13,352	0,052	0,172	15,77	12,25	5,4170
n.a.	Isobutyrate RI	n.a.	n.a.	n.a.	n.a.	n.a.	n.a.
n.a.	Butyrate RI	n.a.	n.a.	n.a.	n.a.	n.a.	n.a.
**Total:**			**0,327**	**1,403**	**100,00**	**100,00**	

Peak Analysis

Injection Details

Injection Name:	**MUCHMO1 t24 r3**	*Run Time (min):*	**20,00**
Vial Number:	**3:27**	*Injection Volume:*	**10,00**
Injection Type:	**Unknown**	*Channel:*	**RI_CH_1**
Calibration Level:		*Wavelength:*	**n.a.**
Instrument Method:	**Default method LC2030C 45 gr 20 min**	*Bandwidth:*	**n.a.**
Processing Method:	**Processing Method LC2030 45 gr**	*Dilution Factor:*	**1,0000**
Injection Date/Time:	**17/Jan/23 02:26**	*Sample Weight:*	**1,0000**

Chromatogram

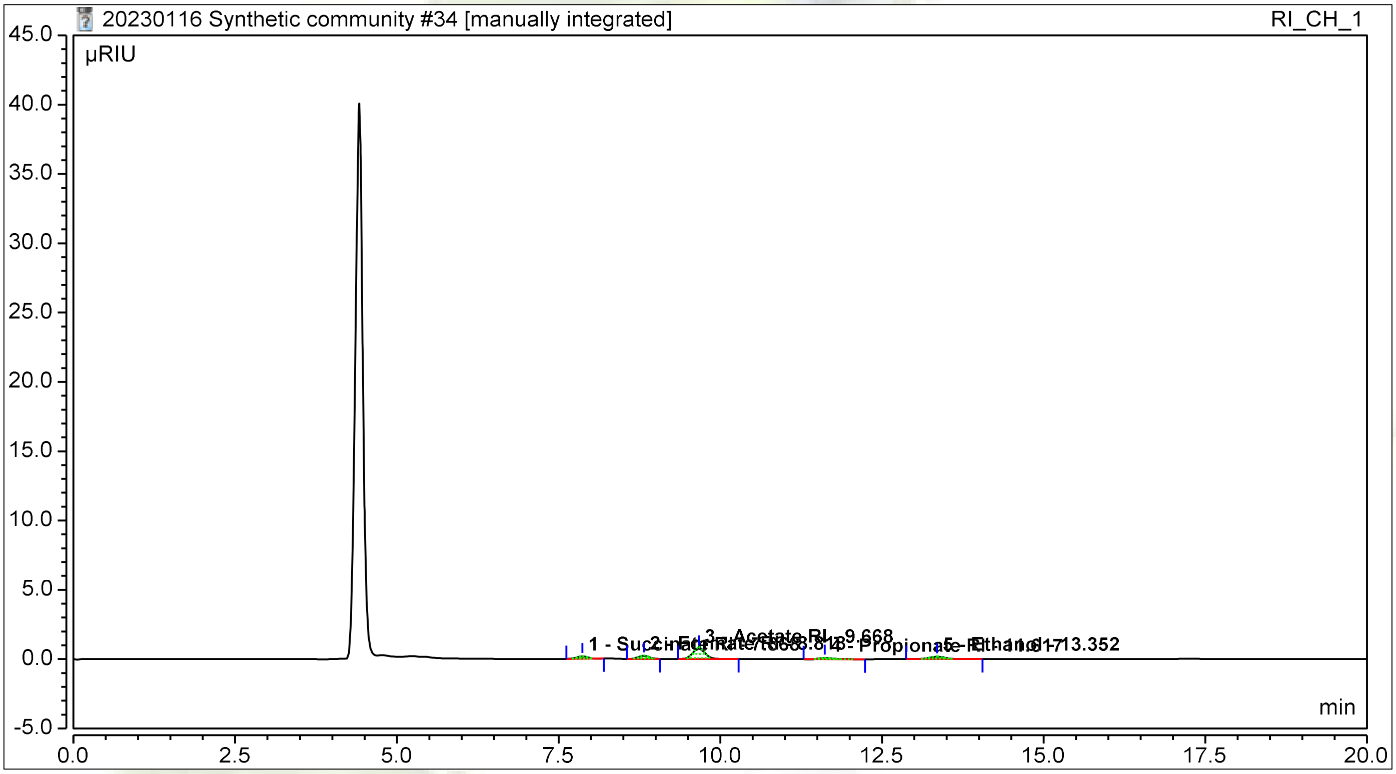

Peak Results

No.	Peak Name	Retention Time min	Width (50%) min	Type	Resolution (EP)	Asymmetry (EP)	Plates (EP)
n.a.	GlcNAc	n.a.	n.a.	n.a.	n.a.	n.a.	n.a.
n.a.	Citrate	n.a.	n.a.	n.a.	n.a.	n.a.	n.a.
n.a.	Glucose	n.a.	n.a.	n.a.	n.a.	n.a.	n.a.
n.a.	Galactose	n.a.	n.a.	n.a.	n.a.	n.a.	n.a.
n.a.	Fucose	n.a.	n.a.	n.a.	n.a.	n.a.	n.a.
1	Succinate RI	7,868	0,188	BMB*	2,94	0,94	9718
n.a.	Lactate RI	n.a.	n.a.	n.a.	n.a.	n.a.	n.a.
n.a.	glycerol	n.a.	n.a.	n.a.	n.a.	n.a.	n.a.
2	Formate RI	8,818	0,193	BMB*	2,44	1,04	11592
3	Acetate RI	9,668	0,218	BMB	5,02	1,05	10872
n.a.	1,2 PDO RI	n.a.	n.a.	n.a.	n.a.	n.a.	n.a.
n.a.	1,3-PDO	n.a.	n.a.	n.a.	n.a.	n.a.	n.a.
4	Propionate RI	11,617	0,240	BMB*	3,90	1,99	12962
n.a.	1,3-PDO	n.a.	n.a.	n.a.	n.a.	n.a.	n.a.
n.a.	2-3 BDO	n.a.	n.a.	n.a.	n.a.	n.a.	n.a.
5	Ethanol	13,352	0,285	BMB*	n.a.	1,02	12123
n.a.	Isobutyrate RI	n.a.	n.a.	n.a.	n.a.	n.a.	n.a.
n.a.	Butyrate RI	n.a.	n.a.	n.a.	n.a.	n.a.	n.a.

Chromatogram and SST Results

Injection Details

Injection Name:	**MUCHMO1 t24 r3**	*Run Time (min):*	**20,00**
Vial Number:	**3:27**	*Injection Volume:*	**10,00**
Injection Type:	**Unknown**	*Channel:*	**RI_CH_1**
Calibration Level:		*Wavelength:*	**n.a.**
Instrument Method:	**Default method LC2030C 45 gr 20 min**	*Bandwidth:*	**n.a.**
Processing Method:	**Processing Method LC2030 45 gr**	*Dilution Factor:*	**1,0000**
Injection Date/Time:	**17/Jan/23 02:26**	*Sample Weight:*	**1,0000**

Chromatogram

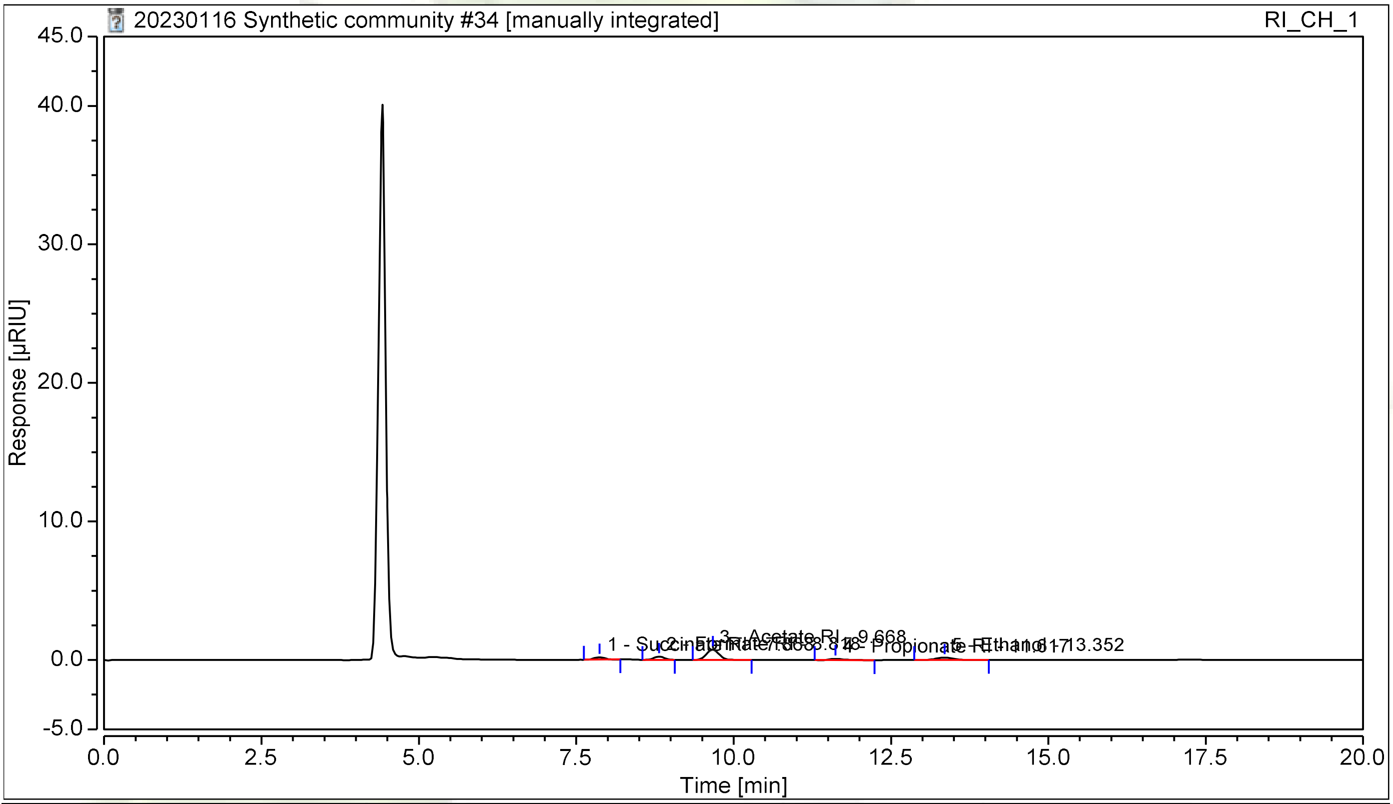

SST Results

No.	Name	Inj.Condition	Peak	Test Result	Injection

Number of executed test cases: **n.a.** **Total Result:** **Passed**

Chromatogram and Results

Injection Details

Injection Name:	**MUCHMO1 t72 r1**	*Run Time (min):*	**20,00**
Vial Number:	**3:28**	*Injection Volume:*	**10,00**
Injection Type:	**Unknown**	*Channel:*	**RI_CH_1**
Calibration Level:		*Wavelength:*	**n.a.**
Instrument Method:	**Default method LC2030C 45 gr 20 min**	*Bandwidth:*	**n.a.**
Processing Method:	**Processing Method LC2030 45 gr**	*Dilution Factor:*	**1,0000**
Injection Date/Time:	**17/Jan/23 02:47**	*Sample Weight:*	**1,0000**

Chromatogram

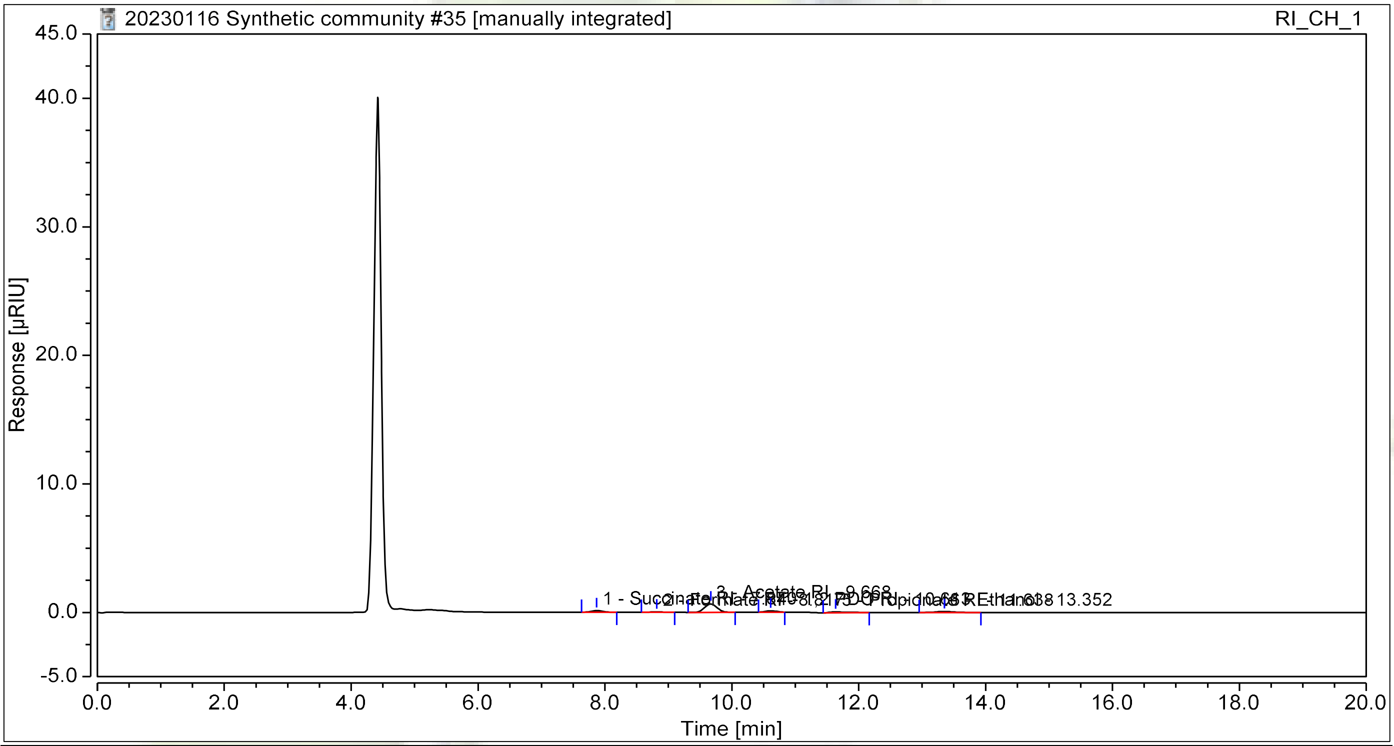

Integration Results

No.	Peak Name	Retention Time min	Area µRIU*min	Height µRIU	Relative Area %	Relative Height %	Amount
n.a.	GlcNAc	n.a.	n.a.	n.a.	n.a.	n.a.	n.a.
n.a.	Citrate	n.a.	n.a.	n.a.	n.a.	n.a.	n.a.
n.a.	Glucose	n.a.	n.a.	n.a.	n.a.	n.a.	n.a.
n.a.	Galactose	n.a.	n.a.	n.a.	n.a.	n.a.	n.a.
n.a.	Fucose	n.a.	n.a.	n.a.	n.a.	n.a.	n.a.
1	Succinate RI	7,870	0,025	0,124	9,92	11,76	0,5133
n.a.	Lactate RI	n.a.	n.a.	n.a.	n.a.	n.a.	n.a.
n.a.	glycerol	n.a.	n.a.	n.a.	n.a.	n.a.	n.a.
2	Formate RI	8,817	0,009	0,045	3,66	4,28	0,9390
3	Acetate RI	9,668	0,150	0,647	59,72	61,59	9,2806
4	1,2 PDO RI	10,613	0,021	0,096	8,40	9,13	0,6411
n.a.	1,3-PDO	n.a.	n.a.	n.a.	n.a.	n.a.	n.a.
5	Propionate RI	11,638	0,023	0,065	9,25	6,21	0,9699
n.a.	1,3-PDO	n.a.	n.a.	n.a.	n.a.	n.a.	n.a.
n.a.	2-3 BDO	n.a.	n.a.	n.a.	n.a.	n.a.	n.a.
6	Ethanol	13,352	0,023	0,074	9,04	7,03	2,3764
n.a.	Isobutyrate RI	n.a.	n.a.	n.a.	n.a.	n.a.	n.a.
n.a.	Butyrate RI	n.a.	n.a.	n.a.	n.a.	n.a.	n.a.
**Total:**			**0,251**	**1,051**	**100,00**	**100,00**	

Chromeleon (c) Dionex
Version 7.2.10.25868

Peak Analysis

Injection Details

Injection Name:	**MUCHMO1 t72 r1**	*Run Time (min):*	**20,00**
Vial Number:	**3:28**	*Injection Volume:*	**10,00**
Injection Type:	**Unknown**	*Channel:*	**RI_CH_1**
Calibration Level:		*Wavelength:*	**n.a.**
Instrument Method:	**Default method LC2030C 45 gr 20 min**	*Bandwidth:*	**n.a.**
Processing Method:	**Processing Method LC2030 45 gr**	*Dilution Factor:*	**1,0000**
Injection Date/Time:	**17/Jan/23 02:47**	*Sample Weight:*	**1,0000**

Chromatogram

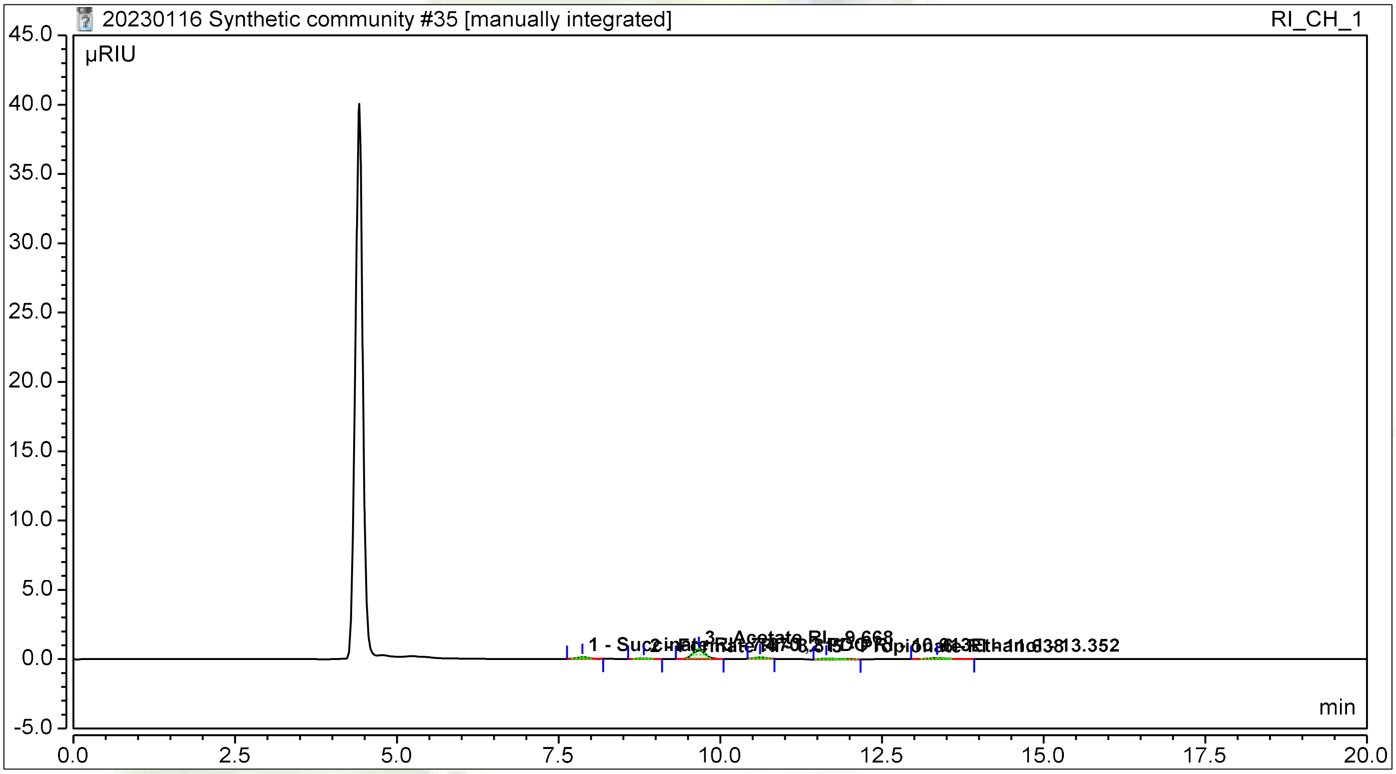

Peak Results

No.	Peak Name	Retention Time min	Width (50%) min	Type	Resolution (EP)	Asymmetry (EP)	Plates (EP)
n.a.	GlcNAc	n.a.	n.a.	n.a.	n.a.	n.a.	n.a.
n.a.	Citrate	n.a.	n.a.	n.a.	n.a.	n.a.	n.a.
n.a.	Glucose	n.a.	n.a.	n.a.	n.a.	n.a.	n.a.
n.a.	Galactose	n.a.	n.a.	n.a.	n.a.	n.a.	n.a.
n.a.	Fucose	n.a.	n.a.	n.a.	n.a.	n.a.	n.a.
1	Succinate RI	7,870	0,195	BMB*	2,86	0,99	8998
n.a.	Lactate RI	n.a.	n.a.	n.a.	n.a.	n.a.	n.a.
n.a.	glycerol	n.a.	n.a.	n.a.	n.a.	n.a.	n.a.
2	Formate RI	8,817	0,196	BMB*	2,43	1,03	11259
3	Acetate RI	9,668	0,218	BMB	2,55	1,04	10870
4	1,2 PDO RI	10,613	0,218	BMB*	2,39	1,07	13091
n.a.	1,3-PDO	n.a.	n.a.	n.a.	n.a.	n.a.	n.a.
5	Propionate RI	11,638	0,288	BMB*	3,51	1,93	9020
n.a.	1,3-PDO	n.a.	n.a.	n.a.	n.a.	n.a.	n.a.
n.a.	2-3 BDO	n.a.	n.a.	n.a.	n.a.	n.a.	n.a.
6	Ethanol	13,352	0,287	BMB*	n.a.	1,06	11952
n.a.	Isobutyrate RI	n.a.	n.a.	n.a.	n.a.	n.a.	n.a.
n.a.	Butyrate RI	n.a.	n.a.	n.a.	n.a.	n.a.	n.a.

Chromatogram and SST Results

Injection Details

Injection Name:	**MUCHMO1 t72 r1**	*Run Time (min):*	**20,00**
Vial Number:	**3:28**	*Injection Volume:*	**10,00**
Injection Type:	**Unknown**	*Channel:*	**RI_CH_1**
Calibration Level:		*Wavelength:*	**n.a.**
Instrument Method:	**Default method LC2030C 45 gr 20 min**	*Bandwidth:*	**n.a.**
Processing Method:	**Processing Method LC2030 45 gr**	*Dilution Factor:*	**1,0000**
Injection Date/Time:	**17/Jan/23 02:47**	*Sample Weight:*	**1,0000**

Chromatogram

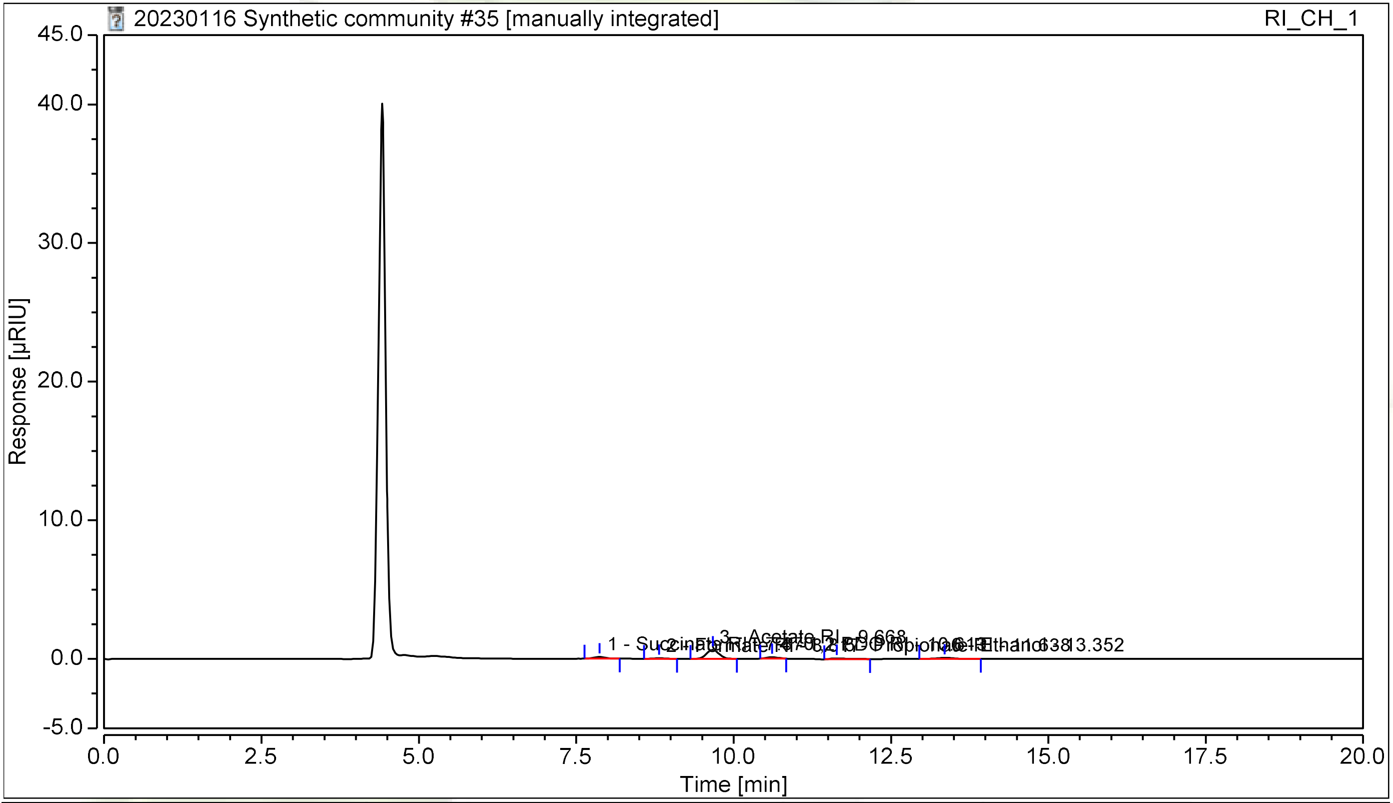

SST Results

No.	Name	Inj.Condition	Peak	Test Result	Injection
**Number of executed test cases:**	**n.a.**		**Total Result:**	**Passed**	

Chromatogram and Results

Injection Details

Injection Name:	**MUCHMO1 t72 r2**	*Run Time (min):*	**20,00**
Vial Number:	**3:29**	*Injection Volume:*	**10,00**
Injection Type:	**Unknown**	*Channel:*	**RI_CH_1**
Calibration Level:		*Wavelength:*	**n.a.**
Instrument Method:	**Default method LC2030C 45 gr 20 min**	*Bandwidth:*	**n.a.**
Processing Method:	**Processing Method LC2030 45 gr**	*Dilution Factor:*	**1,0000**
Injection Date/Time:	**17/Jan/23 03:07**	*Sample Weight:*	**1,0000**

Chromatogram

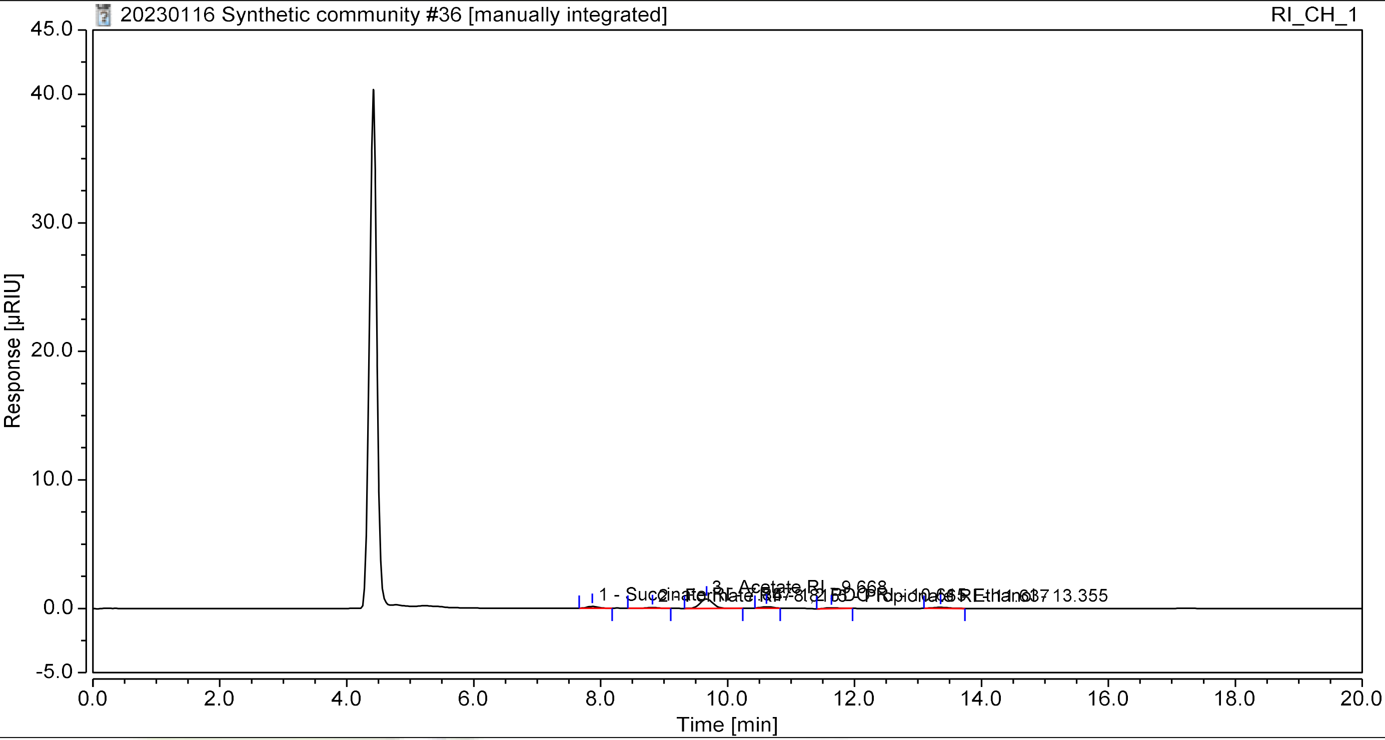

Integration Results

No.	Peak Name	Retention Time min	Area µRIU*min	Height µRIU	Relative Area %	Relative Height %	Amount
n.a.	GlcNAc	n.a.	n.a.	n.a.	n.a.	n.a.	n.a.
n.a.	Citrate	n.a.	n.a.	n.a.	n.a.	n.a.	n.a.
n.a.	Glucose	n.a.	n.a.	n.a.	n.a.	n.a.	n.a.
n.a.	Galactose	n.a.	n.a.	n.a.	n.a.	n.a.	n.a.
n.a.	Fucose	n.a.	n.a.	n.a.	n.a.	n.a.	n.a.
1	Succinate RI	7,867	0,026	0,134	9,69	11,41	0,5379
n.a.	Lactate RI	n.a.	n.a.	n.a.	n.a.	n.a.	n.a.
n.a.	glycerol	n.a.	n.a.	n.a.	n.a.	n.a.	n.a.
2	Formate RI	8,815	0,012	0,062	4,56	5,32	1,2558
3	Acetate RI	9,668	0,171	0,733	63,60	62,66	10,6091
4	1,2 PDO RI	10,615	0,022	0,100	8,12	8,51	0,6652
n.a.	1,3-PDO	n.a.	n.a.	n.a.	n.a.	n.a.	n.a.
5	Propionate RI	11,637	0,016	0,064	5,78	5,50	0,6504
n.a.	1,3-PDO	n.a.	n.a.	n.a.	n.a.	n.a.	n.a.
n.a.	2-3 BDO	n.a.	n.a.	n.a.	n.a.	n.a.	n.a.
6	Ethanol	13,355	0,022	0,077	8,24	6,59	2,3244
n.a.	Isobutyrate RI	n.a.	n.a.	n.a.	n.a.	n.a.	n.a.
n.a.	Butyrate RI	n.a.	n.a.	n.a.	n.a.	n.a.	n.a.
**Total:**			**0,269**	**1,171**	**100,00**	**100,00**	

Chromeleon (c) Dionex
Version 7.2.10.25868

Peak Analysis

Injection Details

Injection Name:	**MUCHMO1 t72 r2**	*Run Time (min):*	**20,00**
Vial Number:	**3:29**	*Injection Volume:*	**10,00**
Injection Type:	**Unknown**	*Channel:*	**RI_CH_1**
Calibration Level:		*Wavelength:*	**n.a.**
Instrument Method:	**Default method LC2030C 45 gr 20 min**	*Bandwidth:*	**n.a.**
Processing Method:	**Processing Method LC2030 45 gr**	*Dilution Factor:*	**1,0000**
Injection Date/Time:	**17/Jan/23 03:07**	*Sample Weight:*	**1,0000**

Chromatogram

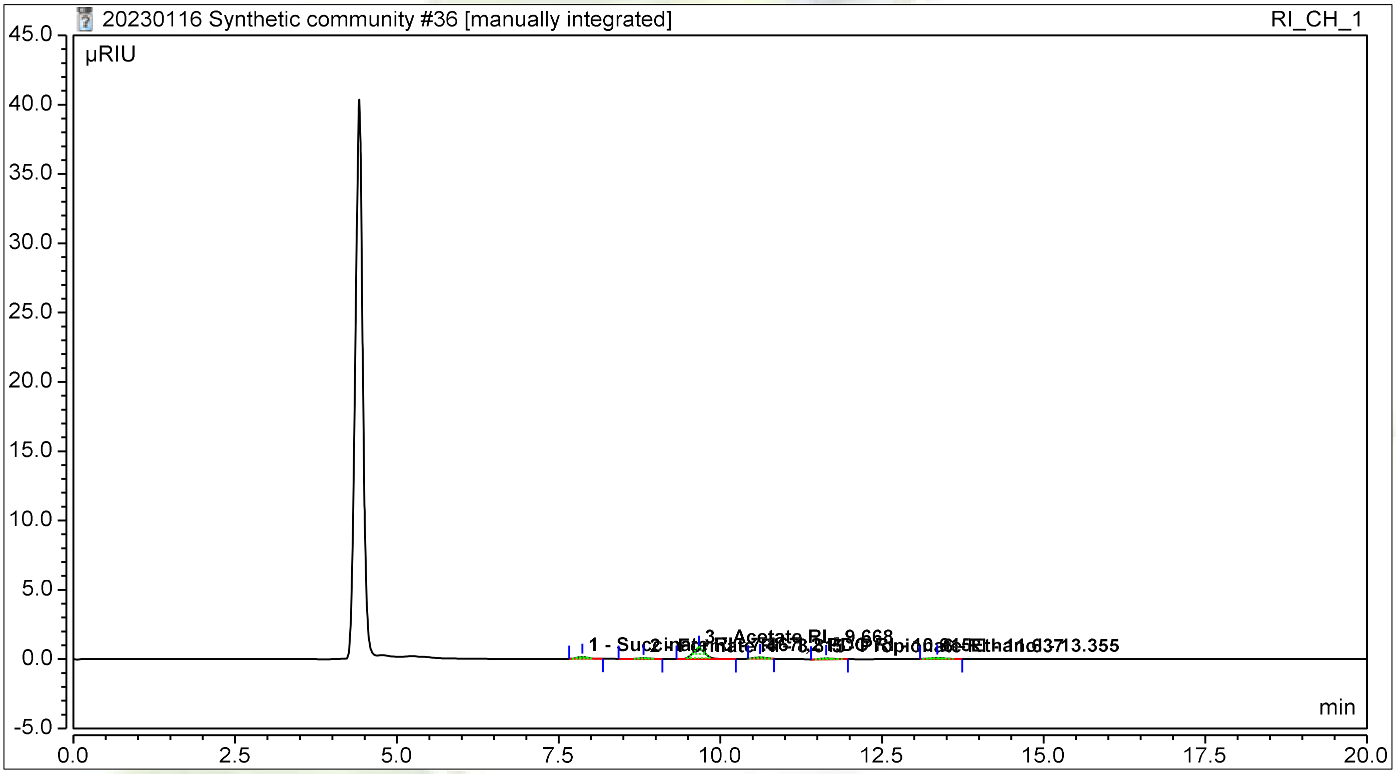

Peak Results

No.	Peak Name	Retention Time min	Width (50%) min	Type	Resolution (EP)	Asymmetry (EP)	Plates (EP)
n.a.	GlcNAc	n.a.	n.a.	n.a.	n.a.	n.a.	n.a.
n.a.	Citrate	n.a.	n.a.	n.a.	n.a.	n.a.	n.a.
n.a.	Glucose	n.a.	n.a.	n.a.	n.a.	n.a.	n.a.
n.a.	Galactose	n.a.	n.a.	n.a.	n.a.	n.a.	n.a.
n.a.	Fucose	n.a.	n.a.	n.a.	n.a.	n.a.	n.a.
1	Succinate RI	7,867	0,192	BMB*	2,91	1,01	9303
n.a.	Lactate RI	n.a.	n.a.	n.a.	n.a.	n.a.	n.a.
n.a.	glycerol	n.a.	n.a.	n.a.	n.a.	n.a.	n.a.
2	Formate RI	8,815	0,192	BMB*	2,45	1,10	11668
3	Acetate RI	9,668	0,219	BMB	2,56	1,05	10840
4	1,2 PDO RI	10,615	0,219	BMB*	2,71	1,09	13070
n.a.	1,3-PDO	n.a.	n.a.	n.a.	n.a.	n.a.	n.a.
5	Propionate RI	11,637	0,226	BMB*	4,03	1,32	14634
n.a.	1,3-PDO	n.a.	n.a.	n.a.	n.a.	n.a.	n.a.
n.a.	2-3 BDO	n.a.	n.a.	n.a.	n.a.	n.a.	n.a.
6	Ethanol	13,355	0,277	BMB*	n.a.	1,10	12894
n.a.	Isobutyrate RI	n.a.	n.a.	n.a.	n.a.	n.a.	n.a.
n.a.	Butyrate RI	n.a.	n.a.	n.a.	n.a.	n.a.	n.a.

Chromeleon (c) Dionex
Version 7.2.10.25868

Chromatogram and SST Results

Injection Details

Injection Name:	**MUCHMO1 t72 r2**	*Run Time (min):*	**20,00**
Vial Number:	**3:29**	*Injection Volume:*	**10,00**
Injection Type:	**Unknown**	*Channel:*	**RI_CH_1**
Calibration Level:		*Wavelength:*	**n.a.**
Instrument Method:	**Default method LC2030C 45 gr 20 min**	*Bandwidth:*	**n.a.**
Processing Method:	**Processing Method LC2030 45 gr**	*Dilution Factor:*	**1,0000**
Injection Date/Time:	**17/Jan/23 03:07**	*Sample Weight:*	**1,0000**

Chromatogram

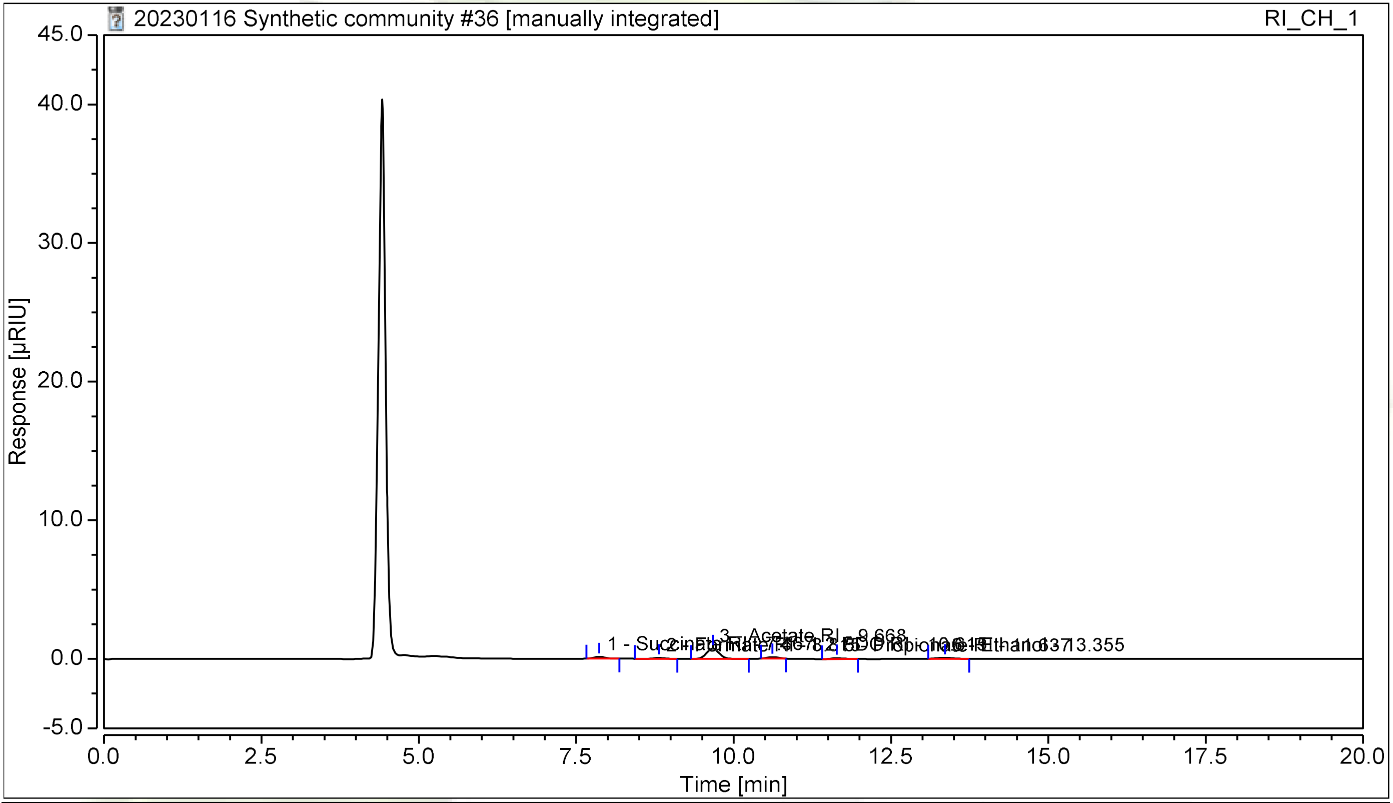

SST Results

No.	Name	Inj.Condition	Peak	Test Result	Injection
**Number of executed test cases:**	**n.a.**		**Total Result:**	**Passed**	

Chromatogram and Results

Injection Details

Injection Name:	**MUCHMO1 t72 r3**	*Run Time (min):*	**20,00**
Vial Number:	**3:30**	*Injection Volume:*	**10,00**
Injection Type:	**Unknown**	*Channel:*	**RI_CH_1**
Calibration Level:		*Wavelength:*	**n.a.**
Instrument Method:	**Default method LC2030C 45 gr 20 min**	*Bandwidth:*	**n.a.**
Processing Method:	**Processing Method LC2030 45 gr**	*Dilution Factor:*	**1,0000**
Injection Date/Time:	**17/Jan/23 03:28**	*Sample Weight:*	**1,0000**

Chromatogram

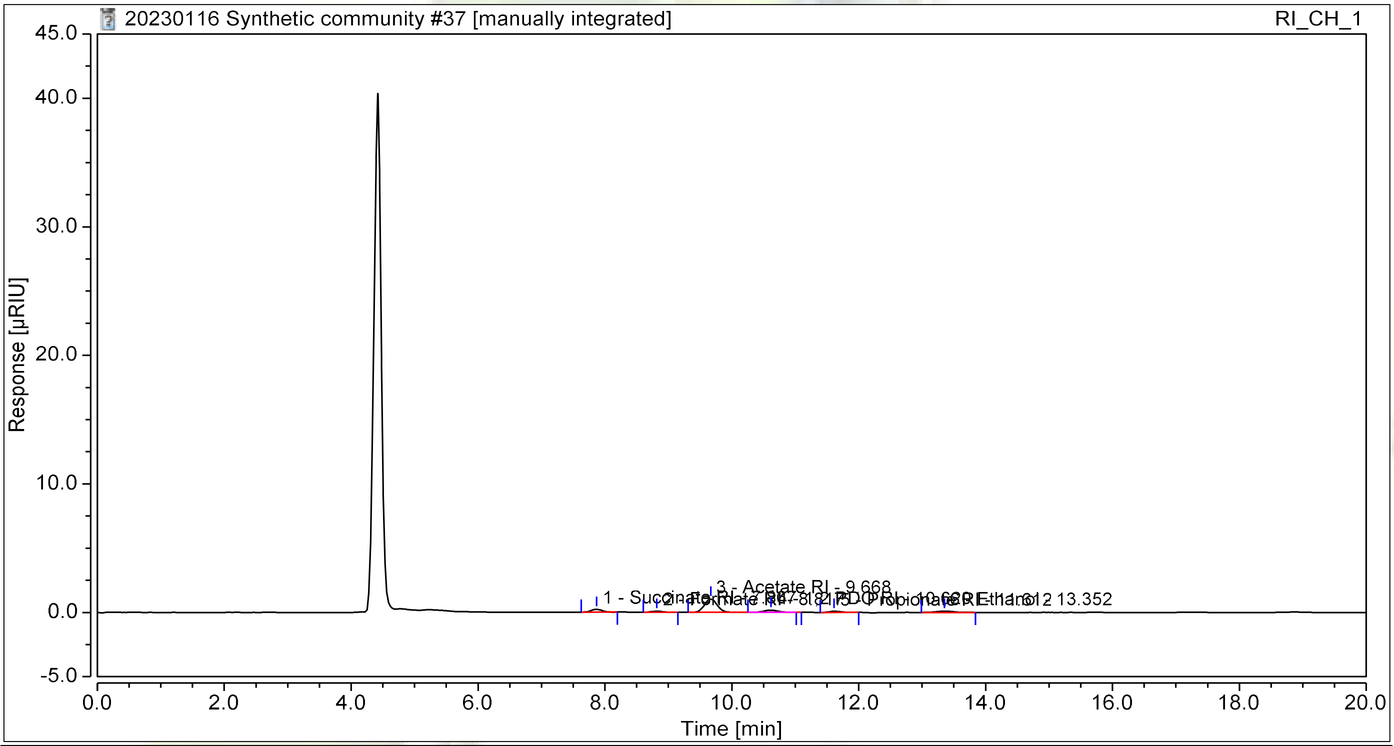

Integration Results

No.	Peak Name	Retention Time min	Area µRIU*min	Height µRIU	Relative Area %	Relative Height %	Amount
n.a.	GlcNAc	n.a.	n.a.	n.a.	n.a.	n.a.	n.a.
n.a.	Citrate	n.a.	n.a.	n.a.	n.a.	n.a.	n.a.
n.a.	Glucose	n.a.	n.a.	n.a.	n.a.	n.a.	n.a.
n.a.	Galactose	n.a.	n.a.	n.a.	n.a.	n.a.	n.a.
n.a.	Fucose	n.a.	n.a.	n.a.	n.a.	n.a.	n.a.
1	Succinate RI	7,867	0,042	0,210	10,61	12,57	0,8654
n.a.	Lactate RI	n.a.	n.a.	n.a.	n.a.	n.a.	n.a.
n.a.	glycerol	n.a.	n.a.	n.a.	n.a.	n.a.	n.a.
2	Formate RI	8,817	0,016	0,080	4,12	4,80	1,6654
3	Acetate RI	9,668	0,246	1,040	62,25	62,08	15,2500
4	1,2 PDO RI	10,620	0,042	0,163	10,70	9,75	1,2862
n.a.	1,3-PDO	n.a.	n.a.	n.a.	n.a.	n.a.	n.a.
5	Propionate RI	11,612	0,020	0,085	5,04	5,09	0,8331
n.a.	1,3-PDO	n.a.	n.a.	n.a.	n.a.	n.a.	n.a.
n.a.	2-3 BDO	n.a.	n.a.	n.a.	n.a.	n.a.	n.a.
6	Ethanol	13,352	0,029	0,096	7,28	5,72	3,0152
n.a.	Isobutyrate RI	n.a.	n.a.	n.a.	n.a.	n.a.	n.a.
n.a.	Butyrate RI	n.a.	n.a.	n.a.	n.a.	n.a.	n.a.
**Total:**			**0,395**	**1,675**	**100,00**	**100,00**	

Peak Analysis

Injection Details

Injection Name:	**MUCHMO1 t72 r3**	*Run Time (min):*	**20,00**
Vial Number:	**3:30**	*Injection Volume:*	**10,00**
Injection Type:	**Unknown**	*Channel:*	**RI_CH_1**
Calibration Level:		*Wavelength:*	**n.a.**
Instrument Method:	**Default method LC2030C 45 gr 20 min**	*Bandwidth:*	**n.a.**
Processing Method:	**Processing Method LC2030 45 gr**	*Dilution Factor:*	**1,0000**
Injection Date/Time:	**17/Jan/23 03:28**	*Sample Weight:*	**1,0000**

Chromatogram

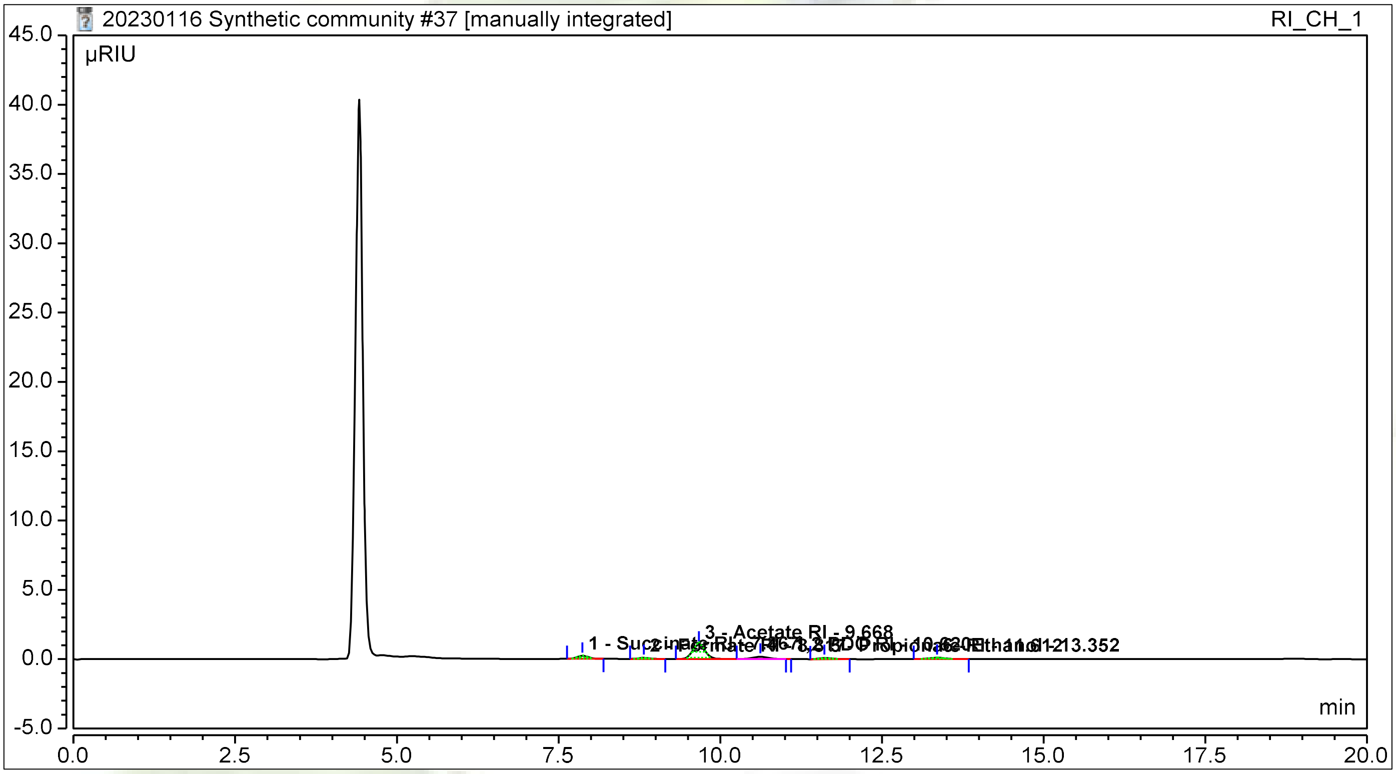

Peak Results

No.	Peak Name	Retention Time min	Width (50%) min	Type	Resolution (EP)	Asymmetry (EP)	Plates (EP)
n.a.	GlcNAc	n.a.	n.a.	n.a.	n.a.	n.a.	n.a.
n.a.	Citrate	n.a.	n.a.	n.a.	n.a.	n.a.	n.a.
n.a.	Glucose	n.a.	n.a.	n.a.	n.a.	n.a.	n.a.
n.a.	Galactose	n.a.	n.a.	n.a.	n.a.	n.a.	n.a.
n.a.	Fucose	n.a.	n.a.	n.a.	n.a.	n.a.	n.a.
1	Succinate RI	7,867	0,195	BMB*	2,89	1,01	9034
n.a.	Lactate RI	n.a.	n.a.	n.a.	n.a.	n.a.	n.a.
n.a.	glycerol	n.a.	n.a.	n.a.	n.a.	n.a.	n.a.
2	Formate RI	8,817	0,193	BMB*	2,44	1,09	11598
3	Acetate RI	9,668	0,219	BMB	5,18	1,05	10828
4	1,2 PDO RI	10,620	n.a.	Rd	n.a.	n.a.	n.a.
n.a.	1,3-PDO	n.a.	n.a.	n.a.	n.a.	n.a.	n.a.
5	Propionate RI	11,612	0,224	BMB*	4,03	1,20	14878
n.a.	1,3-PDO	n.a.	n.a.	n.a.	n.a.	n.a.	n.a.
n.a.	2-3 BDO	n.a.	n.a.	n.a.	n.a.	n.a.	n.a.
6	Ethanol	13,352	0,285	BMB*	n.a.	1,02	12142
n.a.	Isobutyrate RI	n.a.	n.a.	n.a.	n.a.	n.a.	n.a.
n.a.	Butyrate RI	n.a.	n.a.	n.a.	n.a.	n.a.	n.a.

Chromatogram and SST Results

Injection Details

Injection Name:	**MUCHMO1 t72 r3**	*Run Time (min):*	**20,00**
Vial Number:	**3:30**	*Injection Volume:*	**10,00**
Injection Type:	**Unknown**	*Channel:*	**RI_CH_1**
Calibration Level:		*Wavelength:*	**n.a.**
Instrument Method:	**Default method LC2030C 45 gr 20 min**	*Bandwidth:*	**n.a.**
Processing Method:	**Processing Method LC2030 45 gr**	*Dilution Factor:*	**1,0000**
Injection Date/Time:	**17/Jan/23 03:28**	*Sample Weight:*	**1,0000**

Chromatogram

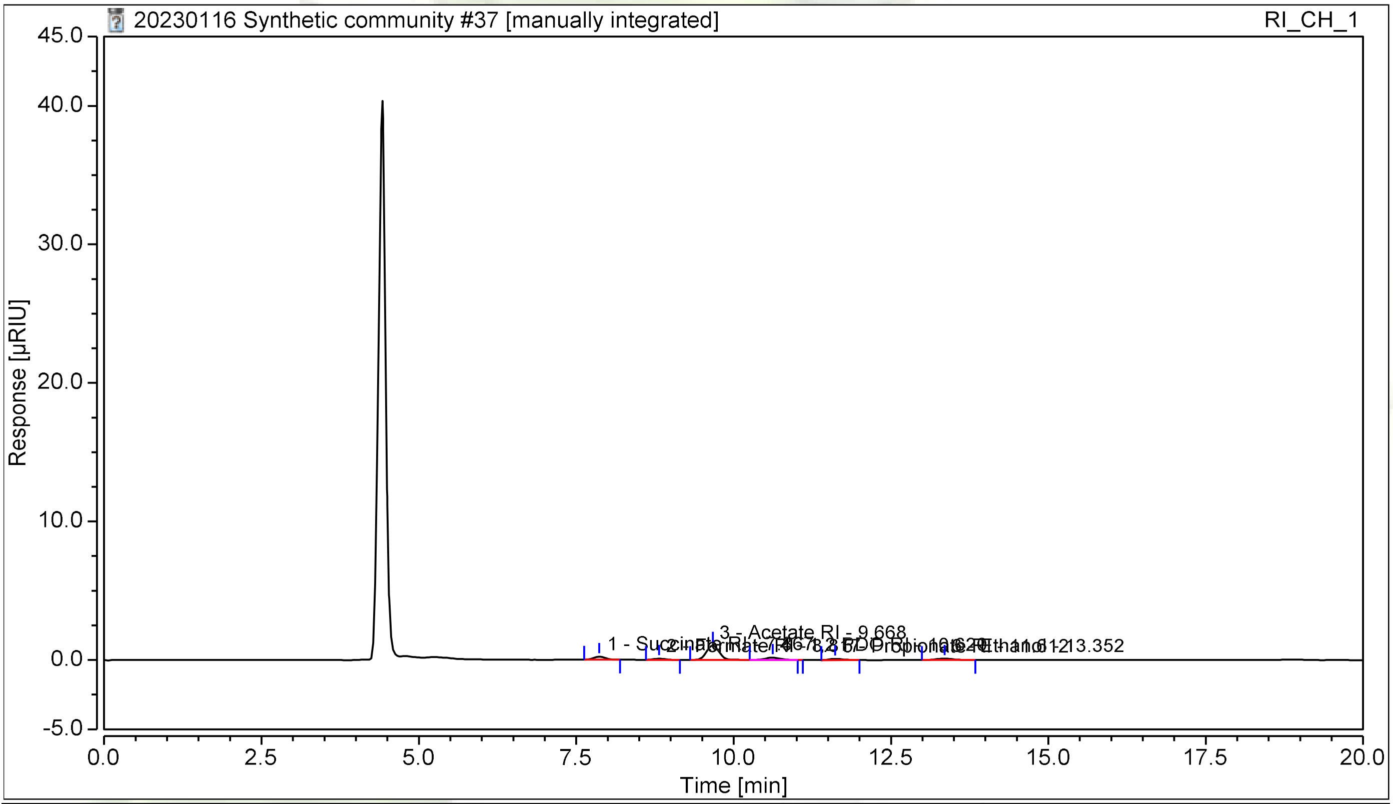

SST Results

No.	Name	Inj.Condition	Peak	Test Result	Injection

Number of executed test cases: **n.a.** **Total Result:** **Passed**

Chromatogram and Results

Injection Details

Injection Name:	**MUCHMO1 t96 r1**	*Run Time (min):*	**20,00**
Vial Number:	**3:31**	*Injection Volume:*	**10,00**
Injection Type:	**Unknown**	*Channel:*	**RI_CH_1**
Calibration Level:		*Wavelength:*	**n.a.**
Instrument Method:	**Default method LC2030C 45 gr 20 min**	*Bandwidth:*	**n.a.**
Processing Method:	**Processing Method LC2030 45 gr**	*Dilution Factor:*	**1,0000**
Injection Date/Time:	**17/Jan/23 03:48**	*Sample Weight:*	**1,0000**

Chromatogram

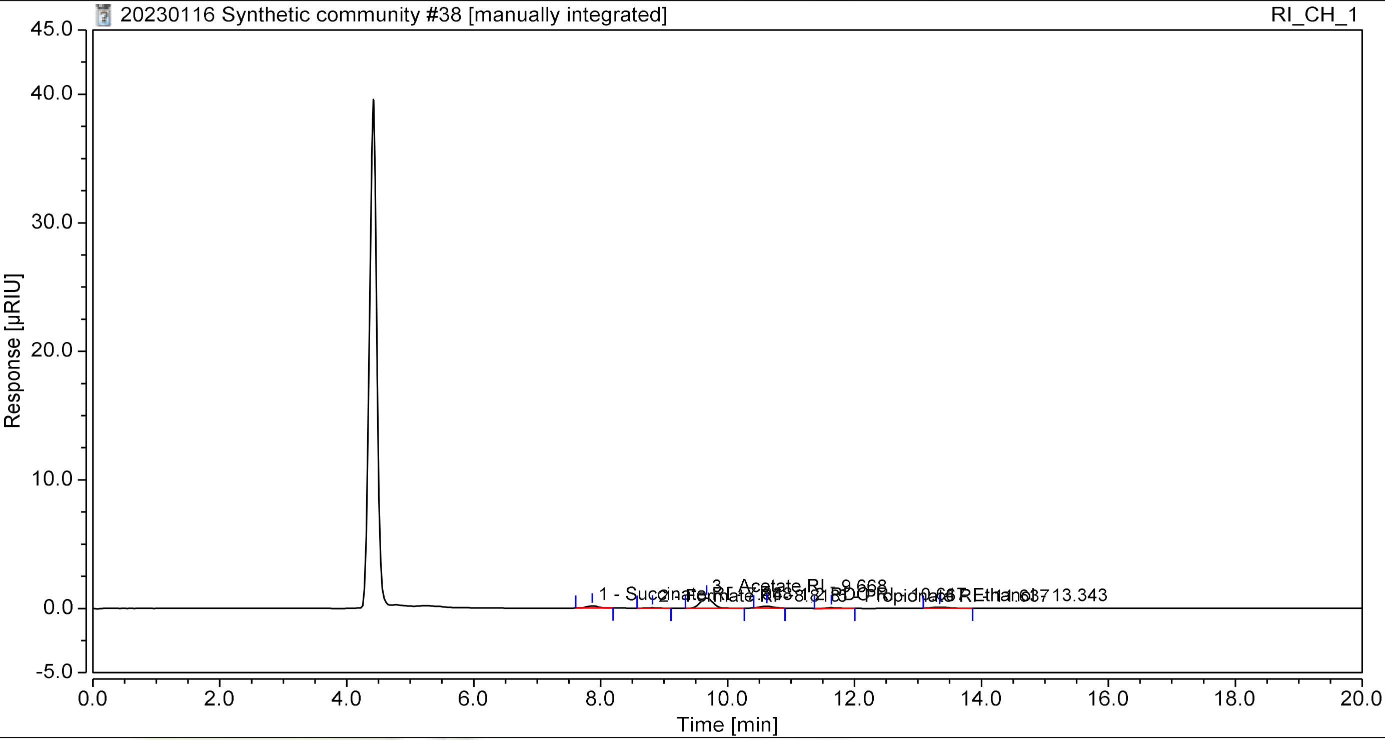

Integration Results

No.	Peak Name	Retention Time min	Area µRIU*min	Height µRIU	Relative Area %	Relative Height %	Amount
n.a.	GlcNAc	n.a.	n.a.	n.a.	n.a.	n.a.	n.a.
n.a.	Citrate	n.a.	n.a.	n.a.	n.a.	n.a.	n.a.
n.a.	Glucose	n.a.	n.a.	n.a.	n.a.	n.a.	n.a.
n.a.	Galactose	n.a.	n.a.	n.a.	n.a.	n.a.	n.a.
n.a.	Fucose	n.a.	n.a.	n.a.	n.a.	n.a.	n.a.
1	Succinate RI	7,868	0,033	0,166	11,54	13,27	0,6872
n.a.	Lactate RI	n.a.	n.a.	n.a.	n.a.	n.a.	n.a.
n.a.	glycerol	n.a.	n.a.	n.a.	n.a.	n.a.	n.a.
2	Formate RI	8,818	0,007	0,032	2,25	2,54	0,6657
3	Acetate RI	9,668	0,182	0,784	63,21	62,86	11,3121
4	1,2 PDO RI	10,617	0,032	0,138	11,24	11,03	0,9875
n.a.	1,3-PDO	n.a.	n.a.	n.a.	n.a.	n.a.	n.a.
5	Propionate RI	11,637	0,011	0,050	3,98	4,04	0,4807
n.a.	1,3-PDO	n.a.	n.a.	n.a.	n.a.	n.a.	n.a.
n.a.	2-3 BDO	n.a.	n.a.	n.a.	n.a.	n.a.	n.a.
6	Ethanol	13,343	0,022	0,078	7,77	6,26	2,3516
n.a.	Isobutyrate RI	n.a.	n.a.	n.a.	n.a.	n.a.	n.a.
n.a.	Butyrate RI	n.a.	n.a.	n.a.	n.a.	n.a.	n.a.
**Total:**			**0,289**	**1,248**	**100,00**	**100,00**	

Chromeleon (c) Dionex
Version 7.2.10.25868

Peak Analysis

Injection Details

Injection Name:	**MUCHMO1 t96 r1**	*Run Time (min):*	**20,00**
Vial Number:	**3:31**	*Injection Volume:*	**10,00**
Injection Type:	**Unknown**	*Channel:*	**RI_CH_1**
Calibration Level:		*Wavelength:*	**n.a.**
Instrument Method:	**Default method LC2030C 45 gr 20 min**	*Bandwidth:*	**n.a.**
Processing Method:	**Processing Method LC2030 45 gr**	*Dilution Factor:*	**1,0000**
Injection Date/Time:	**17/Jan/23 03:48**	*Sample Weight:*	**1,0000**

Chromatogram

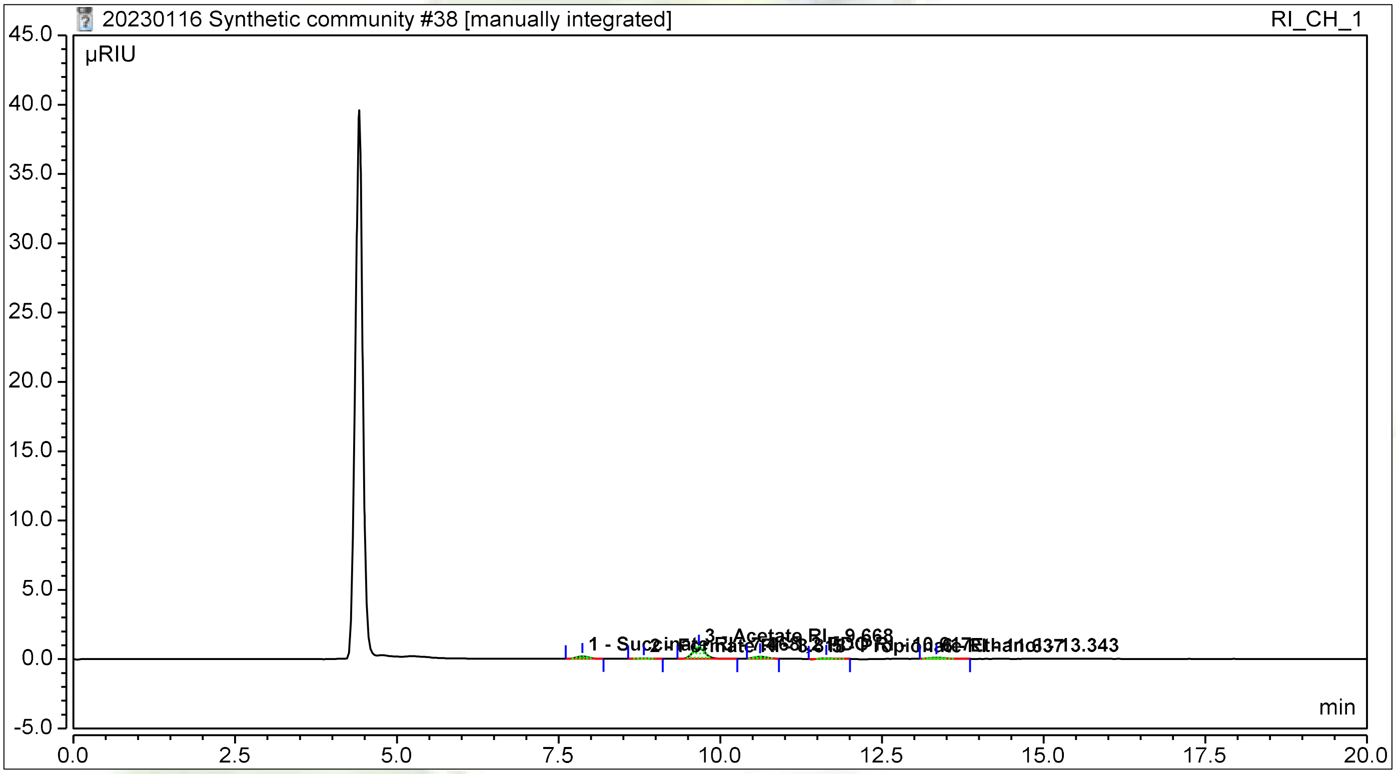

Peak Results

No.	Peak Name	Retention Time min	Width (50%) min	Type	Resolution (EP)	Asymmetry (EP)	Plates (EP)
n.a.	GlcNAc	n.a.	n.a.	n.a.	n.a.	n.a.	n.a.
n.a.	Citrate	n.a.	n.a.	n.a.	n.a.	n.a.	n.a.
n.a.	Glucose	n.a.	n.a.	n.a.	n.a.	n.a.	n.a.
n.a.	Galactose	n.a.	n.a.	n.a.	n.a.	n.a.	n.a.
n.a.	Fucose	n.a.	n.a.	n.a.	n.a.	n.a.	n.a.
1	Succinate RI	7,868	0,195	BMB*	2,87	1,00	9009
n.a.	Lactate RI	n.a.	n.a.	n.a.	n.a.	n.a.	n.a.
n.a.	glycerol	n.a.	n.a.	n.a.	n.a.	n.a.	n.a.
2	Formate RI	8,818	0,196	BMB*	2,42	1,07	11229
3	Acetate RI	9,668	0,218	BMB	2,49	1,05	10860
4	1,2 PDO RI	10,617	0,230	BMB*	2,66	1,12	11777
n.a.	1,3-PDO	n.a.	n.a.	n.a.	n.a.	n.a.	n.a.
5	Propionate RI	11,637	0,222	BMB*	4,06	1,64	15256
n.a.	1,3-PDO	n.a.	n.a.	n.a.	n.a.	n.a.	n.a.
n.a.	2-3 BDO	n.a.	n.a.	n.a.	n.a.	n.a.	n.a.
6	Ethanol	13,343	0,275	BMB*	n.a.	1,13	13072
n.a.	Isobutyrate RI	n.a.	n.a.	n.a.	n.a.	n.a.	n.a.
n.a.	Butyrate RI	n.a.	n.a.	n.a.	n.a.	n.a.	n.a.

Chromatogram and SST Results

Injection Details

Injection Name:	**MUCHMO1 t96 r1**	*Run Time (min):*	**20,00**
Vial Number:	**3:31**	*Injection Volume:*	**10,00**
Injection Type:	**Unknown**	*Channel:*	**RI_CH_1**
Calibration Level:		*Wavelength:*	**n.a.**
Instrument Method:	**Default method LC2030C 45 gr 20 min**	*Bandwidth:*	**n.a.**
Processing Method:	**Processing Method LC2030 45 gr**	*Dilution Factor:*	**1,0000**
Injection Date/Time:	**17/Jan/23 03:48**	*Sample Weight:*	**1,0000**

Chromatogram

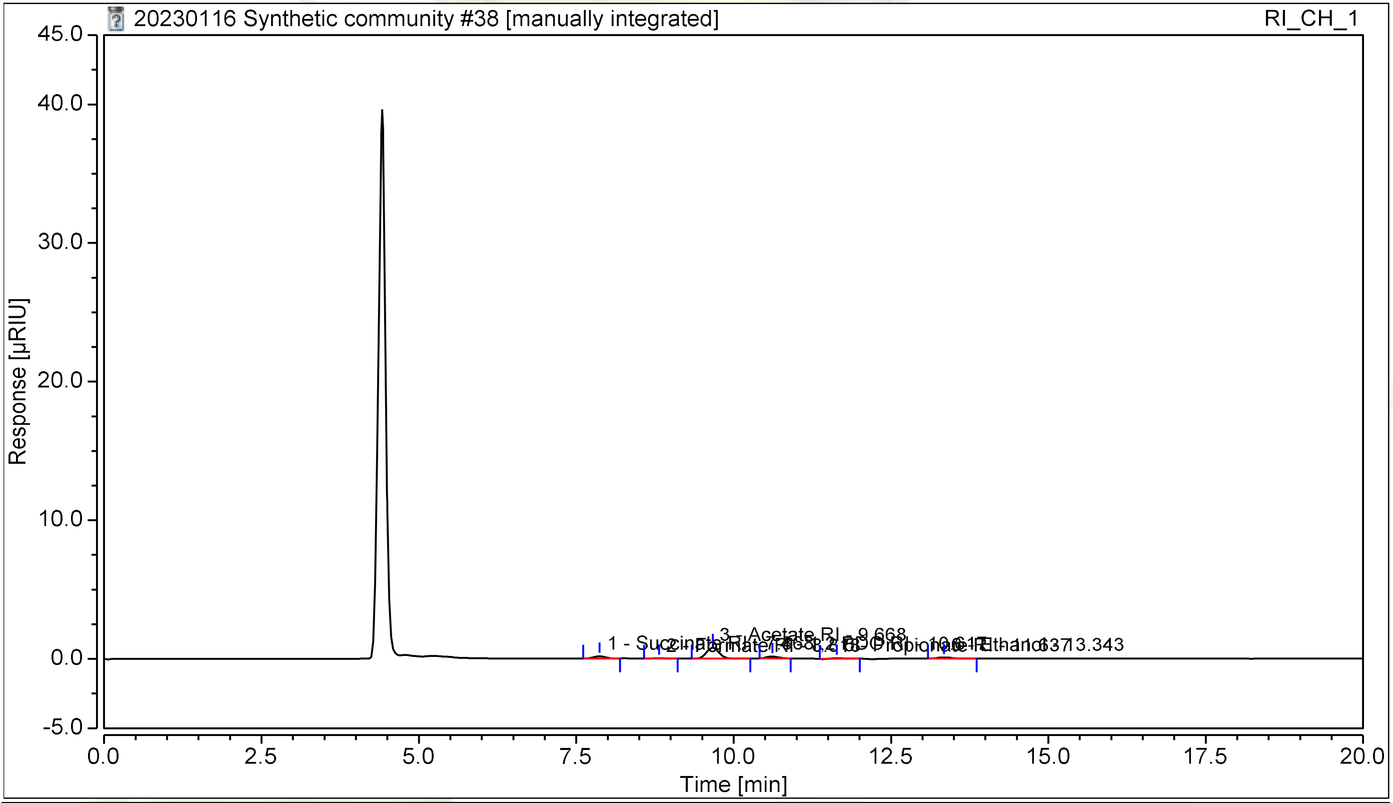

SST Results

No.	Name	Inj.Condition	Peak	Test Result	Injection
**Number of executed test cases:**	**n.a.**		**Total Result:**	**Passed**	

Chromatogram and Results

Injection Details

Injection Name:	**MUCHMO1 t96 r2**	*Run Time (min):*	**20,00**
Vial Number:	**3:32**	*Injection Volume:*	**10,00**
Injection Type:	**Unknown**	*Channel:*	**RI_CH_1**
Calibration Level:		*Wavelength:*	**n.a.**
Instrument Method:	**Default method LC2030C 45 gr 20 min**	*Bandwidth:*	**n.a.**
Processing Method:	**Processing Method LC2030 45 gr**	*Dilution Factor:*	**1,0000**
Injection Date/Time:	**17/Jan/23 04:08**	*Sample Weight:*	**1,0000**

Chromatogram

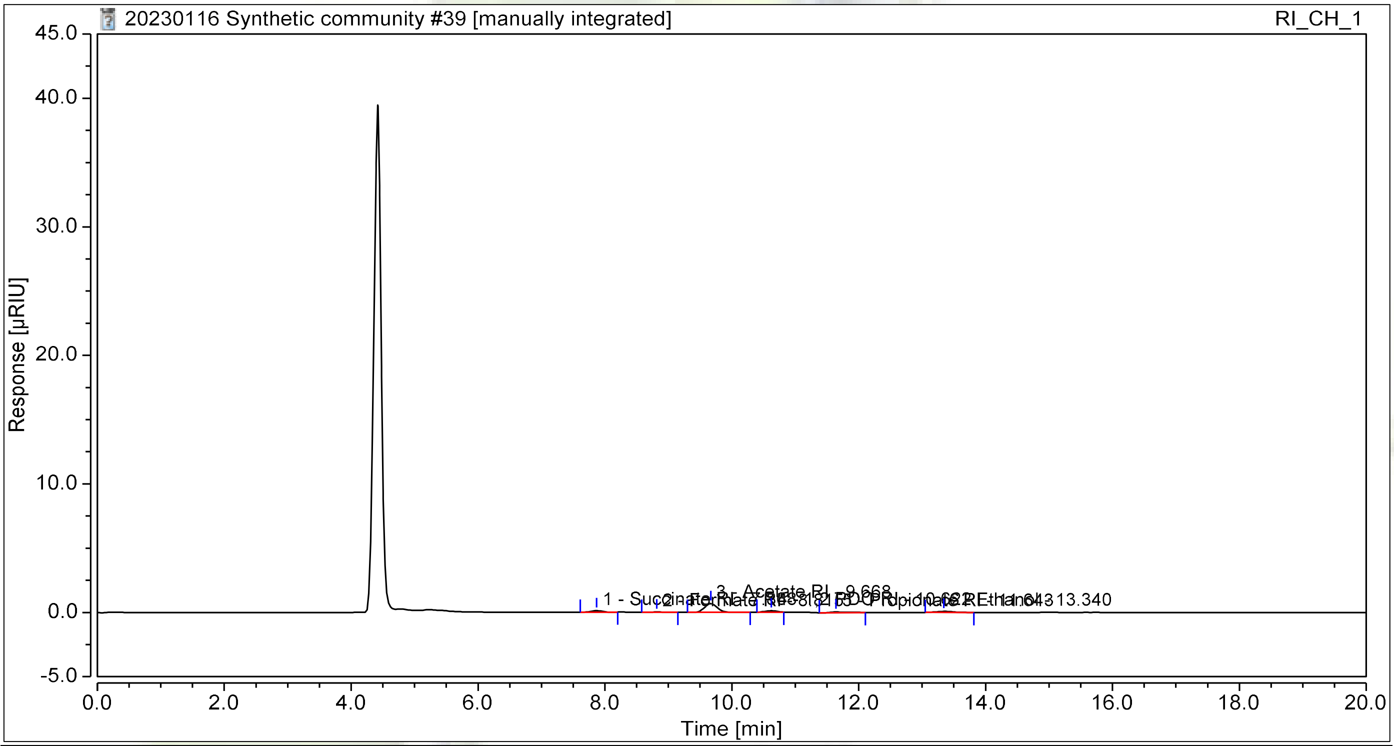

Integration Results

No.	Peak Name	Retention Time min	Area µRIU*min	Height µRIU	Relative Area %	Relative Height %	Amount
n.a.	GlcNAc	n.a.	n.a.	n.a.	n.a.	n.a.	n.a.
n.a.	Citrate	n.a.	n.a.	n.a.	n.a.	n.a.	n.a.
n.a.	Glucose	n.a.	n.a.	n.a.	n.a.	n.a.	n.a.
n.a.	Galactose	n.a.	n.a.	n.a.	n.a.	n.a.	n.a.
n.a.	Fucose	n.a.	n.a.	n.a.	n.a.	n.a.	n.a.
1	Succinate RI	7,868	0,022	0,113	8,76	10,69	0,4492
n.a.	Lactate RI	n.a.	n.a.	n.a.	n.a.	n.a.	n.a.
n.a.	glycerol	n.a.	n.a.	n.a.	n.a.	n.a.	n.a.
2	Formate RI	8,815	0,005	0,026	2,03	2,42	0,5165
3	Acetate RI	9,668	0,158	0,679	63,44	64,15	9,7773
4	1,2 PDO RI	10,622	0,024	0,108	9,81	10,21	0,7418
n.a.	1,3-PDO	n.a.	n.a.	n.a.	n.a.	n.a.	n.a.
5	Propionate RI	11,643	0,017	0,055	6,81	5,22	0,7073
n.a.	1,3-PDO	n.a.	n.a.	n.a.	n.a.	n.a.	n.a.
n.a.	2-3 BDO	n.a.	n.a.	n.a.	n.a.	n.a.	n.a.
6	Ethanol	13,340	0,023	0,077	9,15	7,31	2,3864
n.a.	Isobutyrate RI	n.a.	n.a.	n.a.	n.a.	n.a.	n.a.
n.a.	Butyrate RI	n.a.	n.a.	n.a.	n.a.	n.a.	n.a.
**Total:**			**0,249**	**1,058**	**100,00**	**100,00**	

Chromeleon (c) Dionex
Version 7.2.10.25868

Peak Analysis

Injection Details

Injection Name:	**MUCHMO1 t96 r2**	*Run Time (min):*	**20,00**
Vial Number:	**3:32**	*Injection Volume:*	**10,00**
Injection Type:	**Unknown**	*Channel:*	**RI_CH_1**
Calibration Level:		*Wavelength:*	**n.a.**
Instrument Method:	**Default method LC2030C 45 gr 20 min**	*Bandwidth:*	**n.a.**
Processing Method:	**Processing Method LC2030 45 gr**	*Dilution Factor:*	**1,0000**
Injection Date/Time:	**17/Jan/23 04:08**	*Sample Weight:*	**1,0000**

Chromatogram

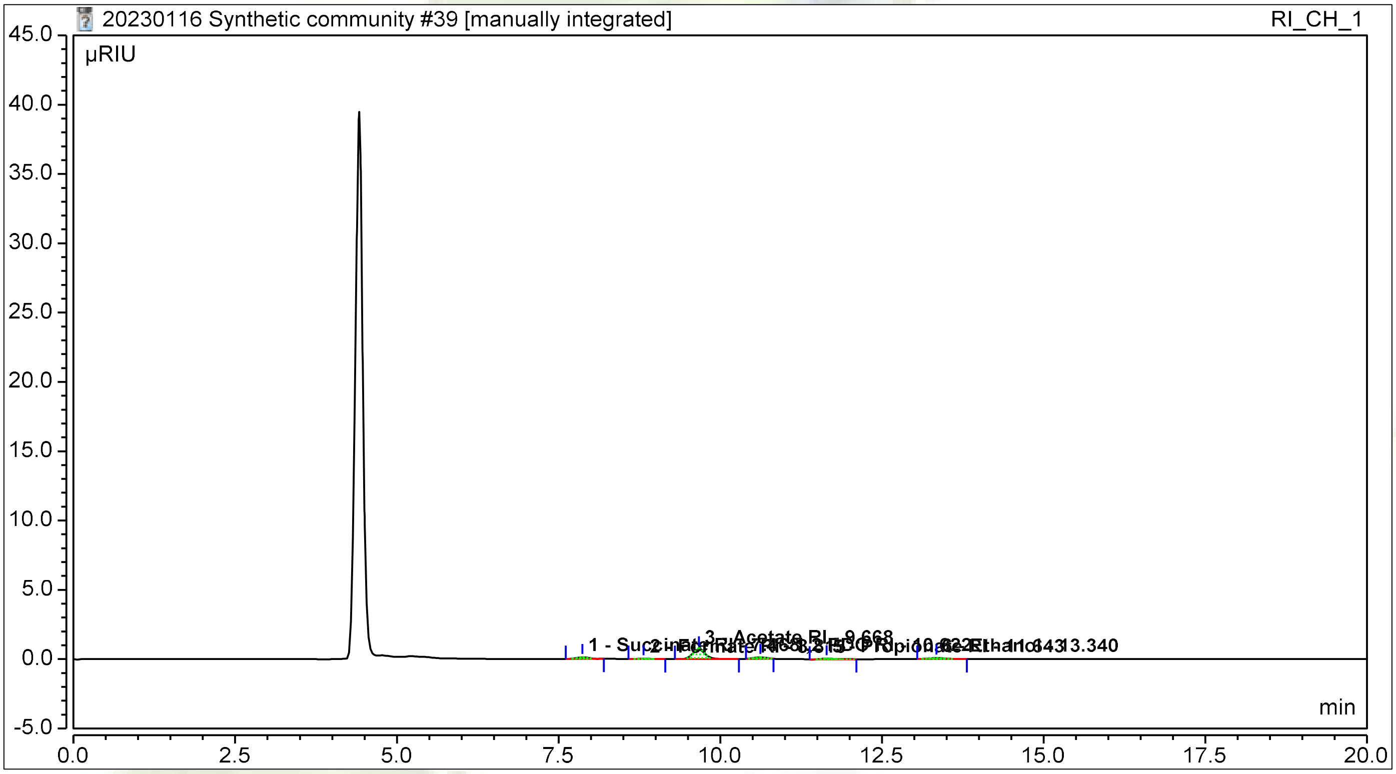

Peak Results

No.	Peak Name	Retention Time min	Width (50%) min	Type	Resolution (EP)	Asymmetry (EP)	Plates (EP)
n.a.	GlcNAc	n.a.	n.a.	n.a.	n.a.	n.a.	n.a.
n.a.	Citrate	n.a.	n.a.	n.a.	n.a.	n.a.	n.a.
n.a.	Glucose	n.a.	n.a.	n.a.	n.a.	n.a.	n.a.
n.a.	Galactose	n.a.	n.a.	n.a.	n.a.	n.a.	n.a.
n.a.	Fucose	n.a.	n.a.	n.a.	n.a.	n.a.	n.a.
1	Succinate RI	7,868	0,192	BMB*	2,94	0,95	9324
n.a.	Lactate RI	n.a.	n.a.	n.a.	n.a.	n.a.	n.a.
n.a.	glycerol	n.a.	n.a.	n.a.	n.a.	n.a.	n.a.
2	Formate RI	8,815	0,188	BMB*	2,48	1,05	12152
3	Acetate RI	9,668	0,218	BMB	2,54	1,05	10893
4	1,2 PDO RI	10,622	0,224	BMB*	2,49	0,95	12424
n.a.	1,3-PDO	n.a.	n.a.	n.a.	n.a.	n.a.	n.a.
5	Propionate RI	11,643	0,259	BMB*	3,70	1,84	11161
n.a.	1,3-PDO	n.a.	n.a.	n.a.	n.a.	n.a.	n.a.
n.a.	2-3 BDO	n.a.	n.a.	n.a.	n.a.	n.a.	n.a.
6	Ethanol	13,340	0,282	BMB*	n.a.	1,10	12415
n.a.	Isobutyrate RI	n.a.	n.a.	n.a.	n.a.	n.a.	n.a.
n.a.	Butyrate RI	n.a.	n.a.	n.a.	n.a.	n.a.	n.a.

Chromatogram and SST Results

Injection Details

Injection Name:	**MUCHMO1 t96 r2**	*Run Time (min):*	**20,00**
Vial Number:	**3:32**	*Injection Volume:*	**10,00**
Injection Type:	**Unknown**	*Channel:*	**RI_CH_1**
Calibration Level:		*Wavelength:*	**n.a.**
Instrument Method:	**Default method LC2030C 45 gr 20 min**	*Bandwidth:*	**n.a.**
Processing Method:	**Processing Method LC2030 45 gr**	*Dilution Factor:*	**1,0000**
Injection Date/Time:	**17/Jan/23 04:08**	*Sample Weight:*	**1,0000**

Chromatogram

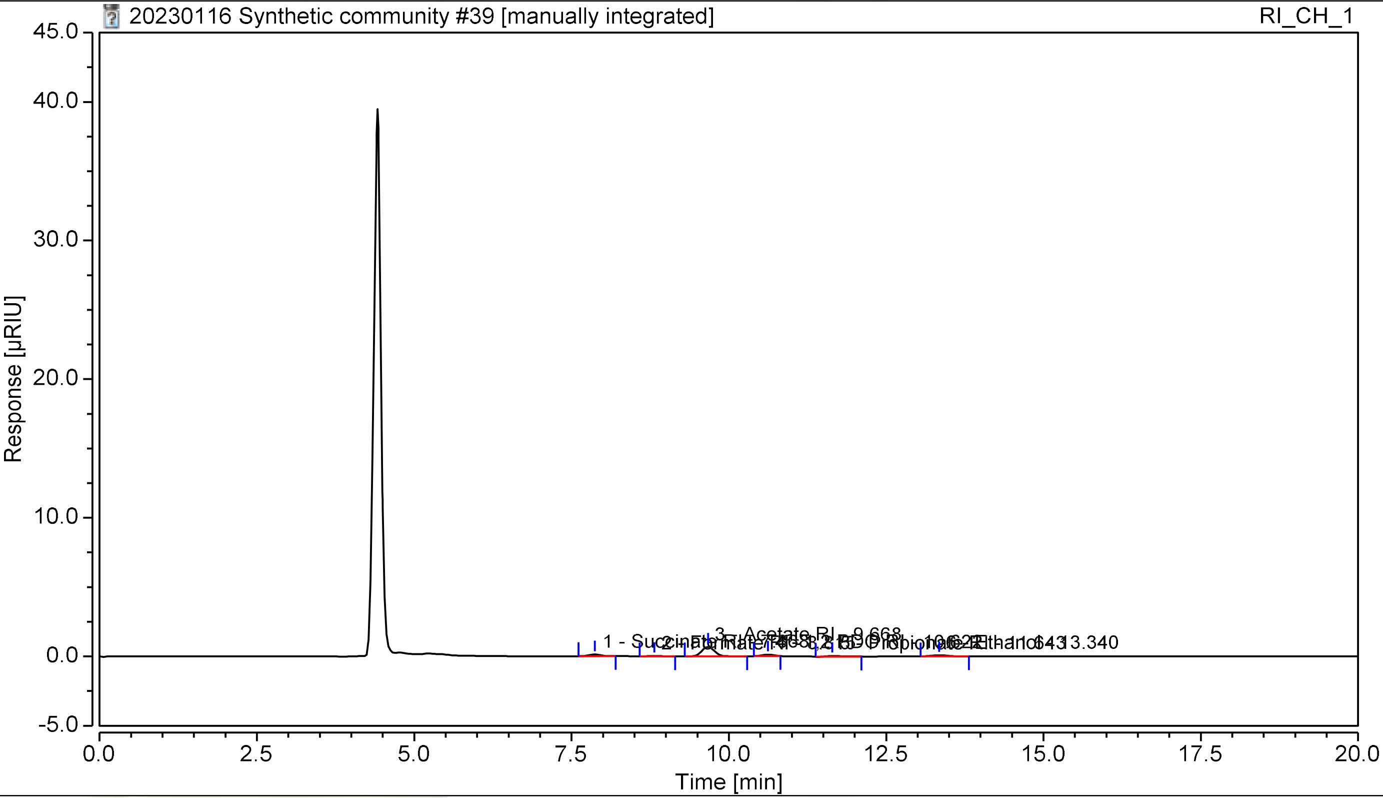

SST Results

No.	Name	Inj.Condition	Peak	Test Result	Injection

Number of executed test cases: **n.a.** **Total Result:** **Passed**

Chromatogram and Results

Injection Details

Injection Name:	**MUCHMO1 t96 r3**	*Run Time (min):*	**20,00**
Vial Number:	**3:33**	*Injection Volume:*	**10,00**
Injection Type:	**Unknown**	*Channel:*	**RI_CH_1**
Calibration Level:		*Wavelength:*	**n.a.**
Instrument Method:	**Default method LC2030C 45 gr 20 min**	*Bandwidth:*	**n.a.**
Processing Method:	**Processing Method LC2030 45 gr**	*Dilution Factor:*	**1,0000**
Injection Date/Time:	**17/Jan/23 04:29**	*Sample Weight:*	**1,0000**

Chromatogram

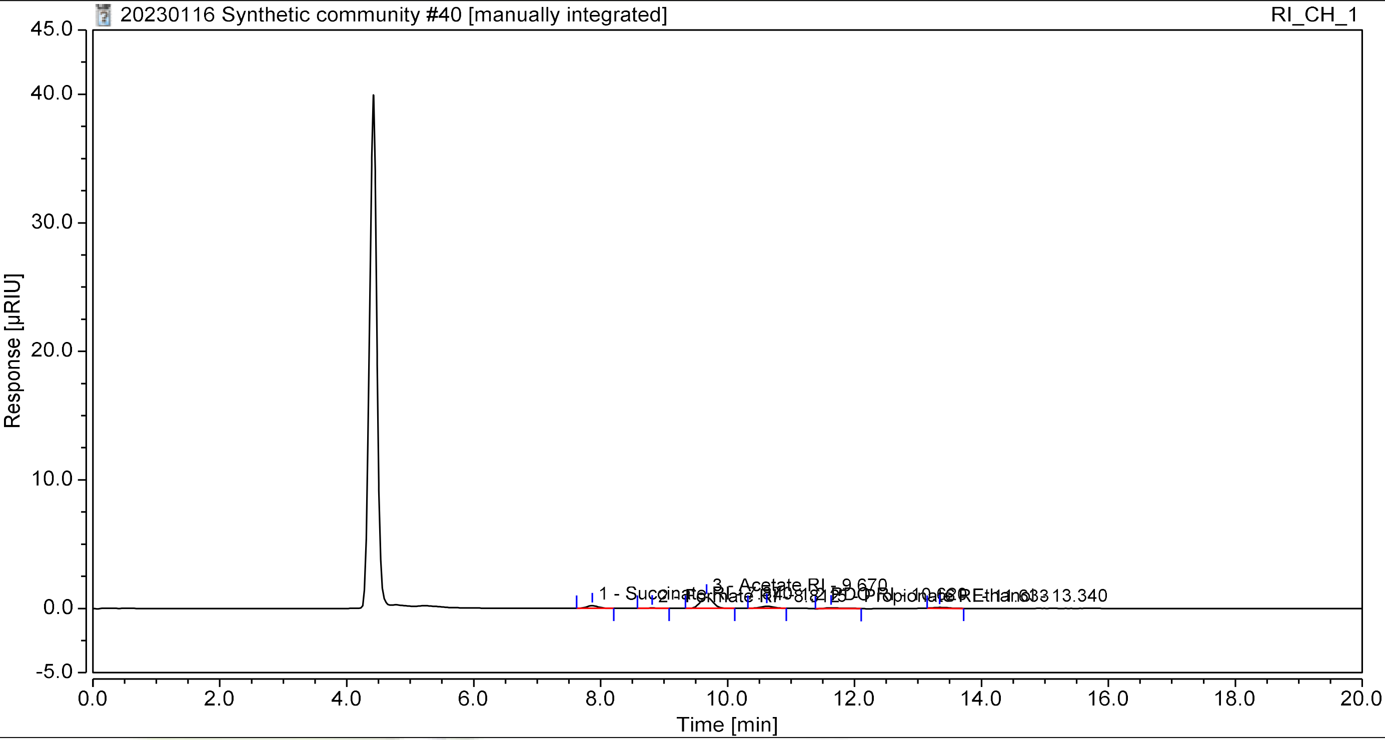

Integration Results

No.	Peak Name	Retention Time min	Area µRIU*min	Height µRIU	Relative Area %	Relative Height %	Amount
n.a.	GlcNAc	n.a.	n.a.	n.a.	n.a.	n.a.	n.a.
n.a.	Citrate	n.a.	n.a.	n.a.	n.a.	n.a.	n.a.
n.a.	Glucose	n.a.	n.a.	n.a.	n.a.	n.a.	n.a.
n.a.	Galactose	n.a.	n.a.	n.a.	n.a.	n.a.	n.a.
n.a.	Fucose	n.a.	n.a.	n.a.	n.a.	n.a.	n.a.
1	Succinate RI	7,870	0,039	0,194	12,21	14,14	0,8143
n.a.	Lactate RI	n.a.	n.a.	n.a.	n.a.	n.a.	n.a.
n.a.	glycerol	n.a.	n.a.	n.a.	n.a.	n.a.	n.a.
2	Formate RI	8,812	0,005	0,022	1,43	1,59	0,4739
3	Acetate RI	9,670	0,200	0,868	62,06	63,16	12,4332
4	1,2 PDO RI	10,620	0,039	0,156	12,23	11,37	1,2021
n.a.	1,3-PDO	n.a.	n.a.	n.a.	n.a.	n.a.	n.a.
5	Propionate RI	11,633	0,022	0,069	6,77	5,04	0,9141
n.a.	1,3-PDO	n.a.	n.a.	n.a.	n.a.	n.a.	n.a.
n.a.	2-3 BDO	n.a.	n.a.	n.a.	n.a.	n.a.	n.a.
6	Ethanol	13,340	0,017	0,065	5,30	4,69	1,7968
n.a.	Isobutyrate RI	n.a.	n.a.	n.a.	n.a.	n.a.	n.a.
n.a.	Butyrate RI	n.a.	n.a.	n.a.	n.a.	n.a.	n.a.
**Total:**			**0,323**	**1,375**	**100,00**	**100,00**	

Chromeleon (c) Dionex
Version 7.2.10.25868

Peak Analysis

Injection Details

Injection Name:	**MUCHMO1 t96 r3**	*Run Time (min):*	**20,00**
Vial Number:	**3:33**	*Injection Volume:*	**10,00**
Injection Type:	**Unknown**	*Channel:*	**RI_CH_1**
Calibration Level:		*Wavelength:*	**n.a.**
Instrument Method:	**Default method LC2030C 45 gr 20 min**	*Bandwidth:*	**n.a.**
Processing Method:	**Processing Method LC2030 45 gr**	*Dilution Factor:*	**1,0000**
Injection Date/Time:	**17/Jan/23 04:29**	*Sample Weight:*	**1,0000**

Chromatogram

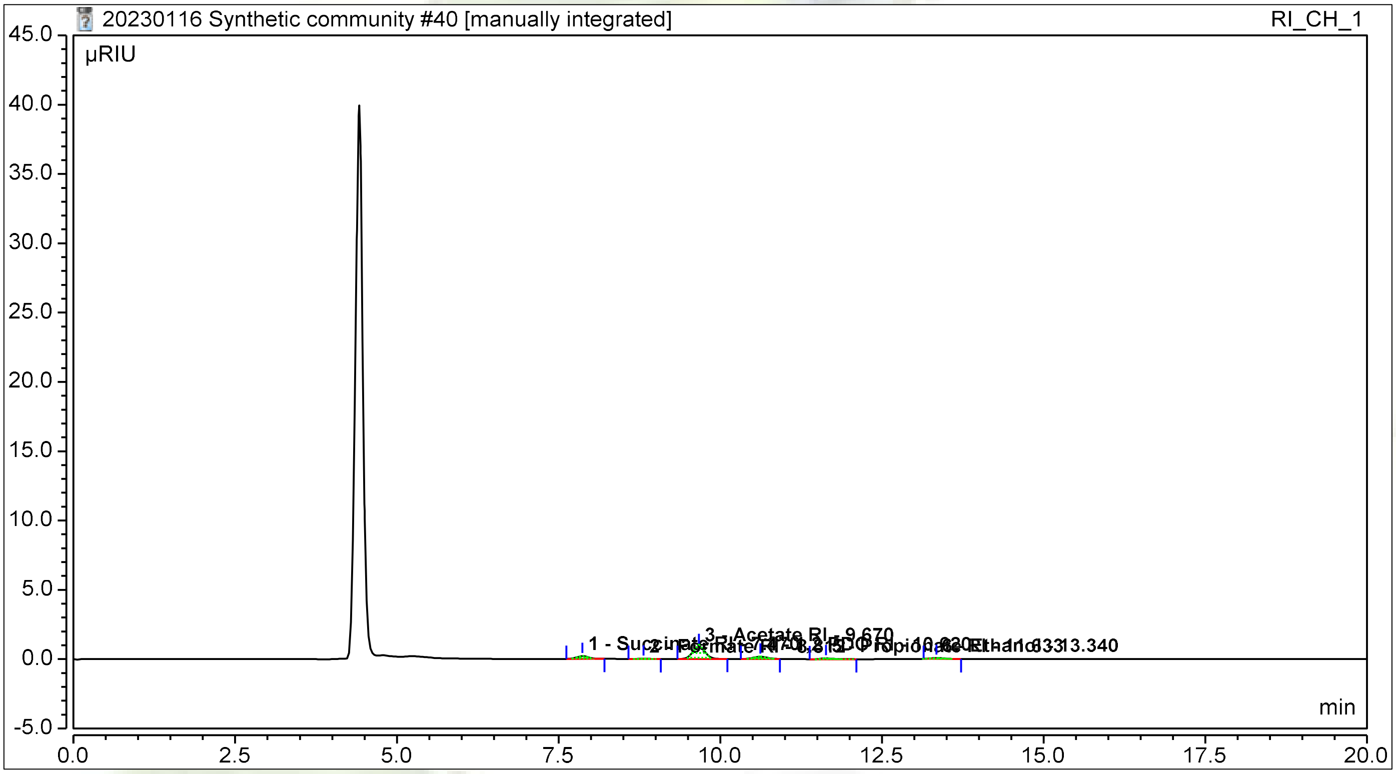

Peak Results

No.	Peak Name	Retention Time min	Width (50%) min	Type	Resolution (EP)	Asymmetry (EP)	Plates (EP)
n.a.	GlcNAc	n.a.	n.a.	n.a.	n.a.	n.a.	n.a.
n.a.	Citrate	n.a.	n.a.	n.a.	n.a.	n.a.	n.a.
n.a.	Glucose	n.a.	n.a.	n.a.	n.a.	n.a.	n.a.
n.a.	Galactose	n.a.	n.a.	n.a.	n.a.	n.a.	n.a.
n.a.	Fucose	n.a.	n.a.	n.a.	n.a.	n.a.	n.a.
1	Succinate RI	7,870	0,196	BMB*	2,80	1,01	8937
n.a.	Lactate RI	n.a.	n.a.	n.a.	n.a.	n.a.	n.a.
n.a.	glycerol	n.a.	n.a.	n.a.	n.a.	n.a.	n.a.
2	Formate RI	8,812	0,201	BMB*	2,42	1,13	10616
3	Acetate RI	9,670	0,218	BMB	2,44	1,04	10904
4	1,2 PDO RI	10,620	0,242	BMB*	2,39	1,01	10708
n.a.	1,3-PDO	n.a.	n.a.	n.a.	n.a.	n.a.	n.a.
5	Propionate RI	11,633	0,258	BMB*	3,88	1,78	11287
n.a.	1,3-PDO	n.a.	n.a.	n.a.	n.a.	n.a.	n.a.
n.a.	2-3 BDO	n.a.	n.a.	n.a.	n.a.	n.a.	n.a.
6	Ethanol	13,340	0,261	BMB*	n.a.	1,26	14432
n.a.	Isobutyrate RI	n.a.	n.a.	n.a.	n.a.	n.a.	n.a.
n.a.	Butyrate RI	n.a.	n.a.	n.a.	n.a.	n.a.	n.a.

Chromatogram and SST Results

Injection Details

Injection Name:	**MUCHMO1 t96 r3**	*Run Time (min):*	**20,00**
Vial Number:	**3:33**	*Injection Volume:*	**10,00**
Injection Type:	**Unknown**	*Channel:*	**RI_CH_1**
Calibration Level:		*Wavelength:*	**n.a.**
Instrument Method:	**Default method LC2030C 45 gr 20 min**	*Bandwidth:*	**n.a.**
Processing Method:	**Processing Method LC2030 45 gr**	*Dilution Factor:*	**1,0000**
Injection Date/Time:	**17/Jan/23 04:29**	*Sample Weight:*	**1,0000**

Chromatogram

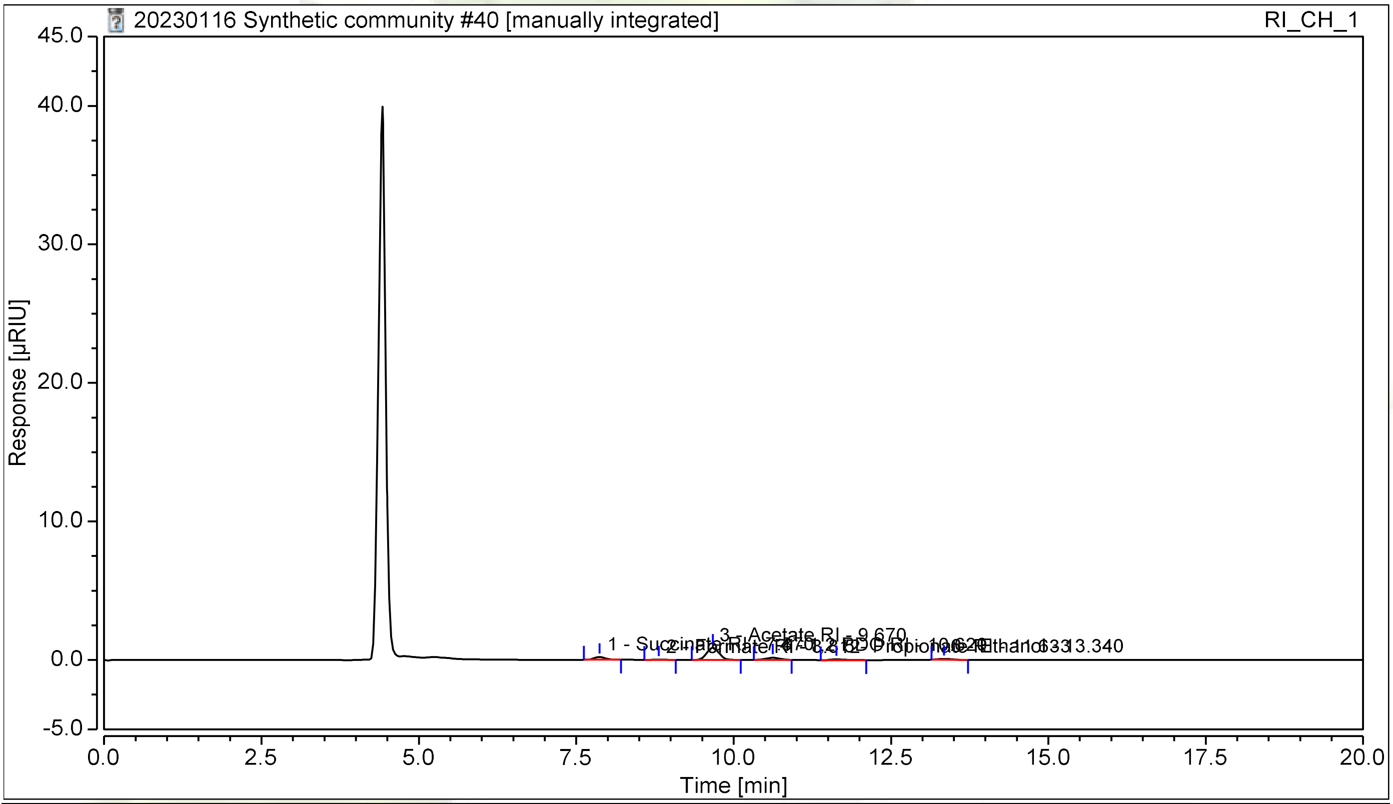

SST Results

No.	Name	Inj.Condition	Peak	Test Result	Injection
**Number of executed test cases:**	**n.a.**		**Total Result:**	**Passed**	

Chromatogram and Results

Injection Details

Injection Name:	**MUCHMO1 t120 r1**	*Run Time (min):*	**20,00**
Vial Number:	**3:34**	*Injection Volume:*	**10,00**
Injection Type:	**Unknown**	*Channel:*	**RI_CH_1**
Calibration Level:		*Wavelength:*	**n.a.**
Instrument Method:	**Default method LC2030C 45 gr 20 min**	*Bandwidth:*	**n.a.**
Processing Method:	**Processing Method LC2030 45 gr**	*Dilution Factor:*	**1,0000**
Injection Date/Time:	**17/Jan/23 04:49**	*Sample Weight:*	**1,0000**

Chromatogram

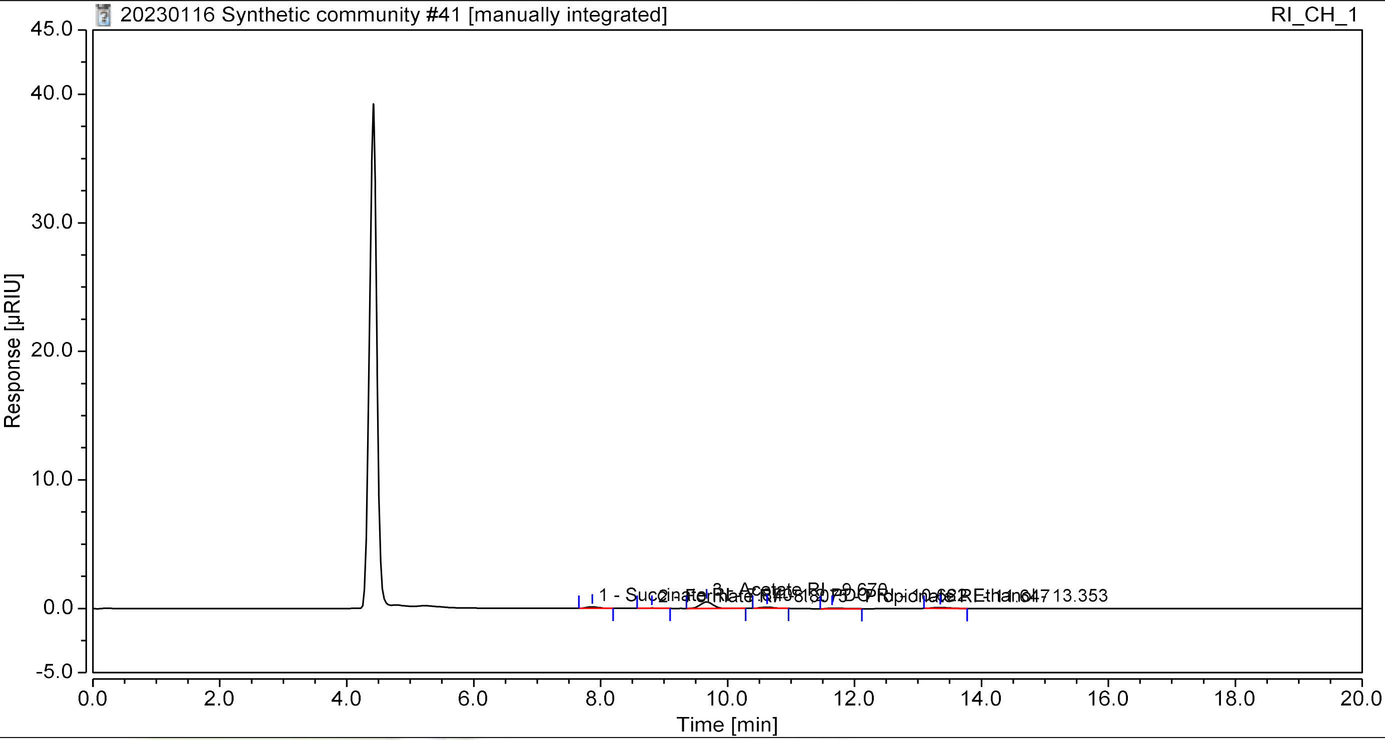

Integration Results

No.	Peak Name	Retention Time min	Area µRIU*min	Height µRIU	Relative Area %	Relative Height %	Amount
n.a.	GlcNAc	n.a.	n.a.	n.a.	n.a.	n.a.	n.a.
n.a.	Citrate	n.a.	n.a.	n.a.	n.a.	n.a.	n.a.
n.a.	Glucose	n.a.	n.a.	n.a.	n.a.	n.a.	n.a.
n.a.	Galactose	n.a.	n.a.	n.a.	n.a.	n.a.	n.a.
n.a.	Fucose	n.a.	n.a.	n.a.	n.a.	n.a.	n.a.
1	Succinate RI	7,870	0,022	0,112	10,78	13,03	0,4607
n.a.	Lactate RI	n.a.	n.a.	n.a.	n.a.	n.a.	n.a.
n.a.	glycerol	n.a.	n.a.	n.a.	n.a.	n.a.	n.a.
2	Formate RI	8,807	0,004	0,023	2,17	2,66	0,4604
3	Acetate RI	9,670	0,118	0,508	57,10	59,33	7,3279
4	1,2 PDO RI	10,622	0,021	0,088	10,33	10,30	0,6507
n.a.	1,3-PDO	n.a.	n.a.	n.a.	n.a.	n.a.	n.a.
5	Propionate RI	11,647	0,021	0,056	9,94	6,53	0,8602
n.a.	1,3-PDO	n.a.	n.a.	n.a.	n.a.	n.a.	n.a.
n.a.	2-3 BDO	n.a.	n.a.	n.a.	n.a.	n.a.	n.a.
6	Ethanol	13,353	0,020	0,070	9,68	8,15	2,1015
n.a.	Isobutyrate RI	n.a.	n.a.	n.a.	n.a.	n.a.	n.a.
n.a.	Butyrate RI	n.a.	n.a.	n.a.	n.a.	n.a.	n.a.
**Total:**			**0,207**	**0,857**	**100,00**	**100,00**	

Chromeleon (c) Dionex
Version 7.2.10.25868

Peak Analysis

Injection Details

Injection Name:	**MUCHMO1 t120 r1**	*Run Time (min):*	**20,00**
Vial Number:	**3:34**	*Injection Volume:*	**10,00**
Injection Type:	**Unknown**	*Channel:*	**RI_CH_1**
Calibration Level:		*Wavelength:*	**n.a.**
Instrument Method:	**Default method LC2030C 45 gr 20 min**	*Bandwidth:*	**n.a.**
Processing Method:	**Processing Method LC2030 45 gr**	*Dilution Factor:*	**1,0000**
Injection Date/Time:	**17/Jan/23 04:49**	*Sample Weight:*	**1,0000**

Chromatogram

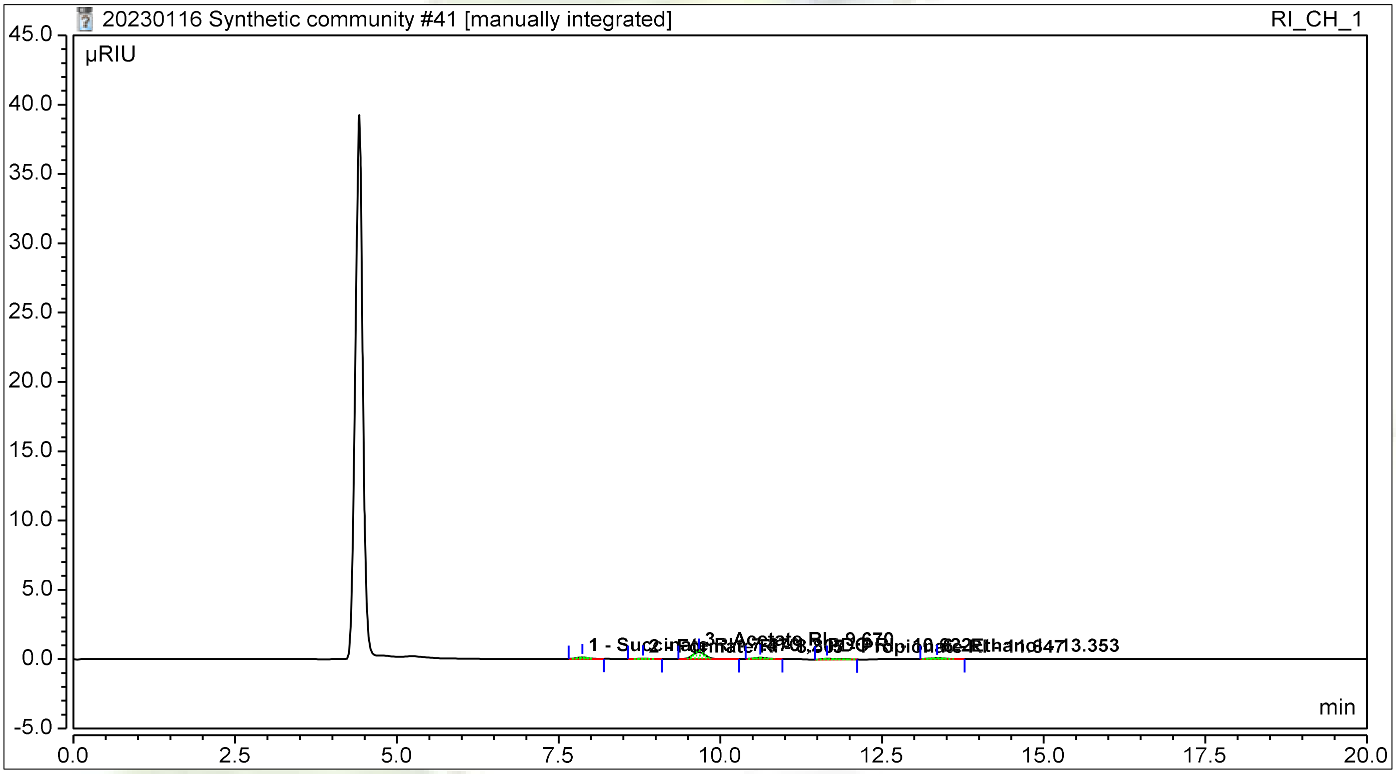

Peak Results

No.	Peak Name	Retention Time min	Width (50%) min	Type	Resolution (EP)	Asymmetry (EP)	Plates (EP)
n.a.	GlcNAc	n.a.	n.a.	n.a.	n.a.	n.a.	n.a.
n.a.	Citrate	n.a.	n.a.	n.a.	n.a.	n.a.	n.a.
n.a.	Glucose	n.a.	n.a.	n.a.	n.a.	n.a.	n.a.
n.a.	Galactose	n.a.	n.a.	n.a.	n.a.	n.a.	n.a.
n.a.	Fucose	n.a.	n.a.	n.a.	n.a.	n.a.	n.a.
1	Succinate RI	7,870	0,195	BMB*	2,88	1,01	9045
n.a.	Lactate RI	n.a.	n.a.	n.a.	n.a.	n.a.	n.a.
n.a.	glycerol	n.a.	n.a.	n.a.	n.a.	n.a.	n.a.
2	Formate RI	8,807	0,189	BMB*	2,50	1,19	12039
3	Acetate RI	9,670	0,218	BMB	2,49	1,05	10889
4	1,2 PDO RI	10,622	0,233	BMB*	1,94	1,09	11539
n.a.	1,3-PDO	n.a.	n.a.	n.a.	n.a.	n.a.	n.a.
5	Propionate RI	11,647	0,392	BMB*	3,01	1,81	4902
n.a.	1,3-PDO	n.a.	n.a.	n.a.	n.a.	n.a.	n.a.
n.a.	2-3 BDO	n.a.	n.a.	n.a.	n.a.	n.a.	n.a.
6	Ethanol	13,353	0,276	BMB*	n.a.	1,12	12929
n.a.	Isobutyrate RI	n.a.	n.a.	n.a.	n.a.	n.a.	n.a.
n.a.	Butyrate RI	n.a.	n.a.	n.a.	n.a.	n.a.	n.a.

Chromatogram and SST Results

Injection Details

Injection Name:	**MUCHMO1 t120 r1**	*Run Time (min):*	**20,00**
Vial Number:	**3:34**	*Injection Volume:*	**10,00**
Injection Type:	**Unknown**	*Channel:*	**RI_CH_1**
Calibration Level:		*Wavelength:*	**n.a.**
Instrument Method:	**Default method LC2030C 45 gr 20 min**	*Bandwidth:*	**n.a.**
Processing Method:	**Processing Method LC2030 45 gr**	*Dilution Factor:*	**1,0000**
Injection Date/Time:	**17/Jan/23 04:49**	*Sample Weight:*	**1,0000**

Chromatogram

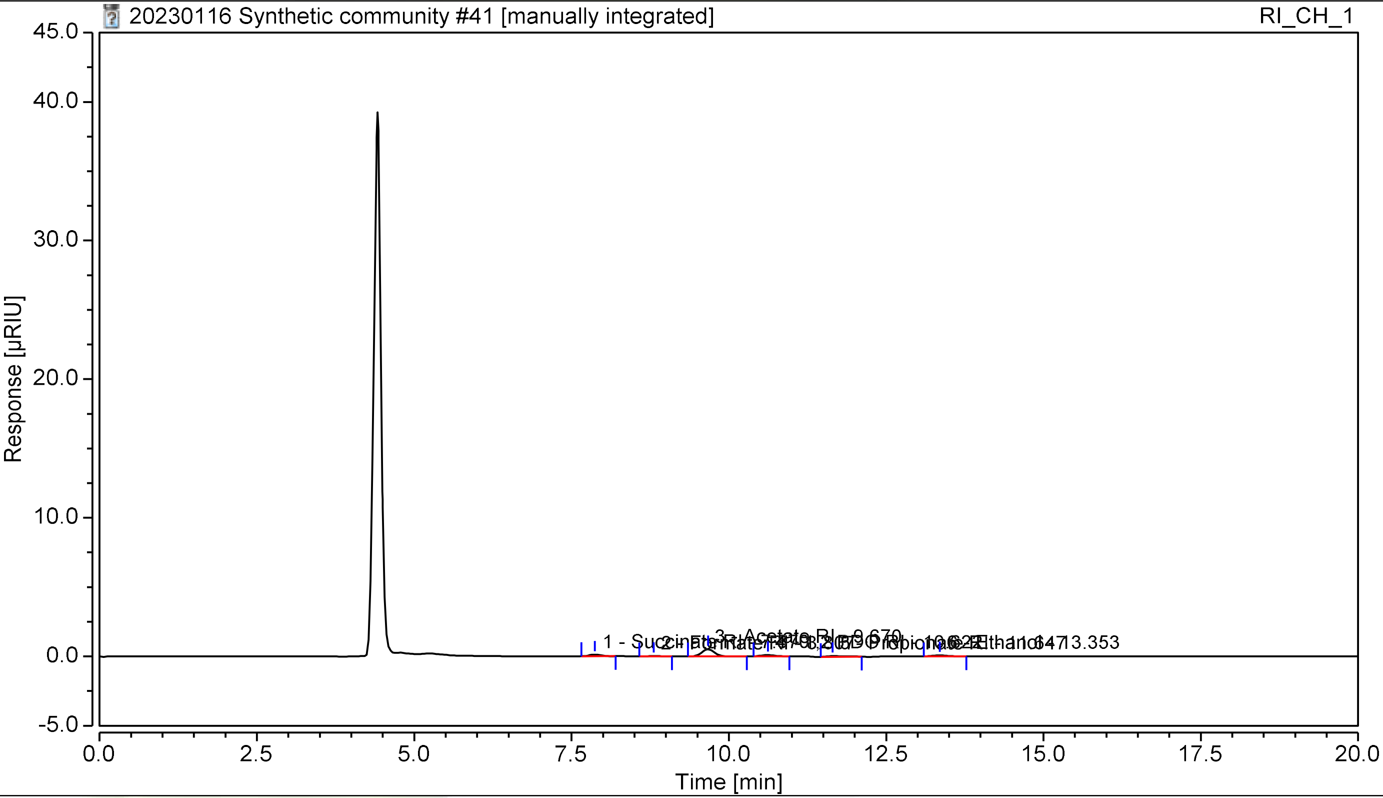

SST Results

No.	Name	Inj.Condition	Peak	Test Result	Injection
**Number of executed test cases:**	**n.a.**		**Total Result:**	**Passed**	

Chromatogram and Results

Injection Details

Injection Name:	**MUCHMO1 t120 r2**	*Run Time (min):*	**20,00**
Vial Number:	**3:35**	*Injection Volume:*	**10,00**
Injection Type:	**Unknown**	*Channel:*	**RI_CH_1**
Calibration Level:		*Wavelength:*	**n.a.**
Instrument Method:	**Default method LC2030C 45 gr 20 min**	*Bandwidth:*	**n.a.**
Processing Method:	**Processing Method LC2030 45 gr**	*Dilution Factor:*	**1,0000**
Injection Date/Time:	**17/Jan/23 05:10**	*Sample Weight:*	**1,0000**

Chromatogram

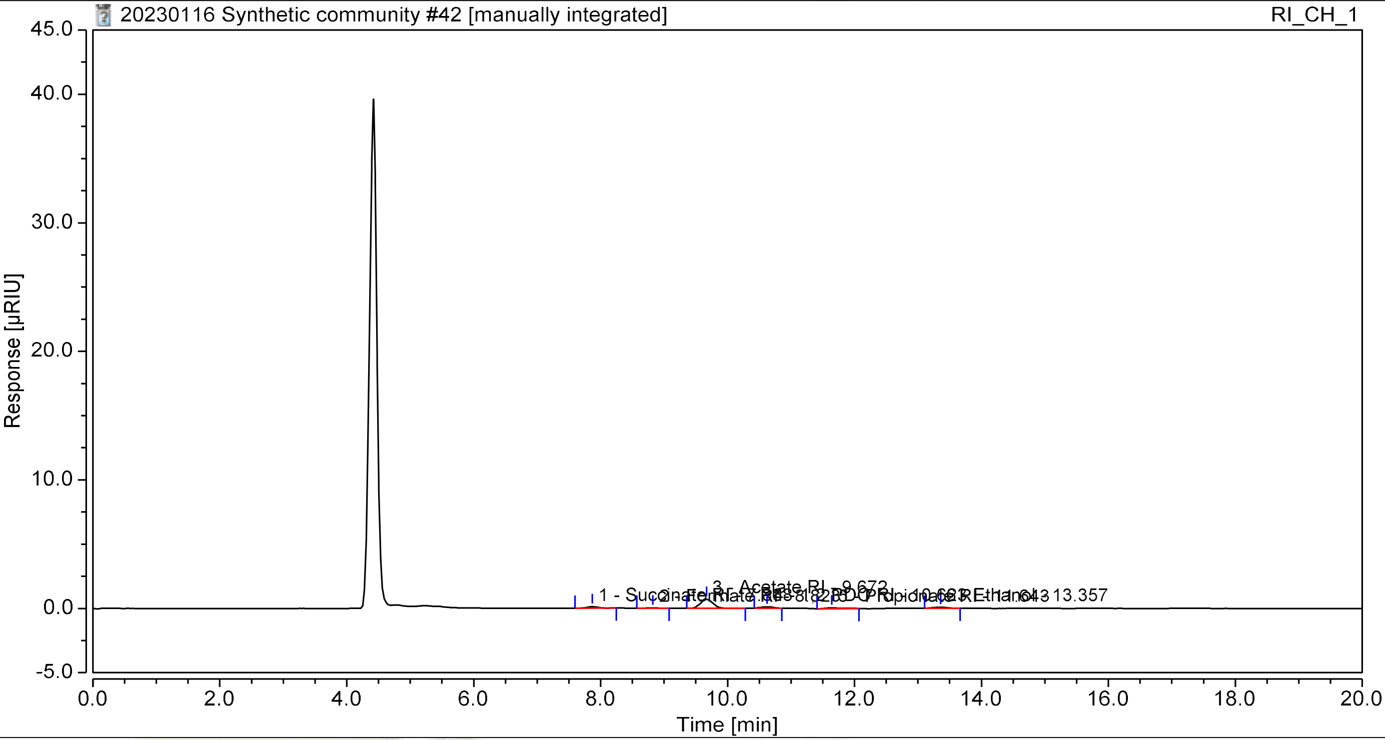

Integration Results

No.	Peak Name	Retention Time min	Area µRIU*min	Height µRIU	Relative Area %	Relative Height %	Amount
n.a.	GlcNAc	n.a.	n.a.	n.a.	n.a.	n.a.	n.a.
n.a.	Citrate	n.a.	n.a.	n.a.	n.a.	n.a.	n.a.
n.a.	Glucose	n.a.	n.a.	n.a.	n.a.	n.a.	n.a.
n.a.	Galactose	n.a.	n.a.	n.a.	n.a.	n.a.	n.a.
n.a.	Fucose	n.a.	n.a.	n.a.	n.a.	n.a.	n.a.
1	Succinate RI	7,868	0,017	0,107	6,92	9,77	0,3594
n.a.	Lactate RI	n.a.	n.a.	n.a.	n.a.	n.a.	n.a.
n.a.	glycerol	n.a.	n.a.	n.a.	n.a.	n.a.	n.a.
2	Formate RI	8,823	0,008	0,040	3,18	3,70	0,8185
3	Acetate RI	9,672	0,164	0,702	65,01	64,34	10,1417
4	1,2 PDO RI	10,623	0,024	0,105	9,55	9,66	0,7312
n.a.	1,3-PDO	n.a.	n.a.	n.a.	n.a.	n.a.	n.a.
5	Propionate RI	11,643	0,017	0,057	6,68	5,25	0,7031
n.a.	1,3-PDO	n.a.	n.a.	n.a.	n.a.	n.a.	n.a.
n.a.	2-3 BDO	n.a.	n.a.	n.a.	n.a.	n.a.	n.a.
6	Ethanol	13,357	0,022	0,079	8,66	7,27	2,2845
n.a.	Isobutyrate RI	n.a.	n.a.	n.a.	n.a.	n.a.	n.a.
n.a.	Butyrate RI	n.a.	n.a.	n.a.	n.a.	n.a.	n.a.
**Total:**			**0,252**	**1,091**	**100,00**	**100,00**	

Chromeleon (c) Dionex
Version 7.2.10.25868

Peak Analysis

Injection Details

Injection Name:	**MUCHMO1 t120 r2**	*Run Time (min):*	**20,00**
Vial Number:	**3:35**	*Injection Volume:*	**10,00**
Injection Type:	**Unknown**	*Channel:*	**RI_CH_1**
Calibration Level:		*Wavelength:*	**n.a.**
Instrument Method:	**Default method LC2030C 45 gr 20 min**	*Bandwidth:*	**n.a.**
Processing Method:	**Processing Method LC2030 45 gr**	*Dilution Factor:*	**1,0000**
Injection Date/Time:	**17/Jan/23 05:10**	*Sample Weight:*	**1,0000**

Chromatogram

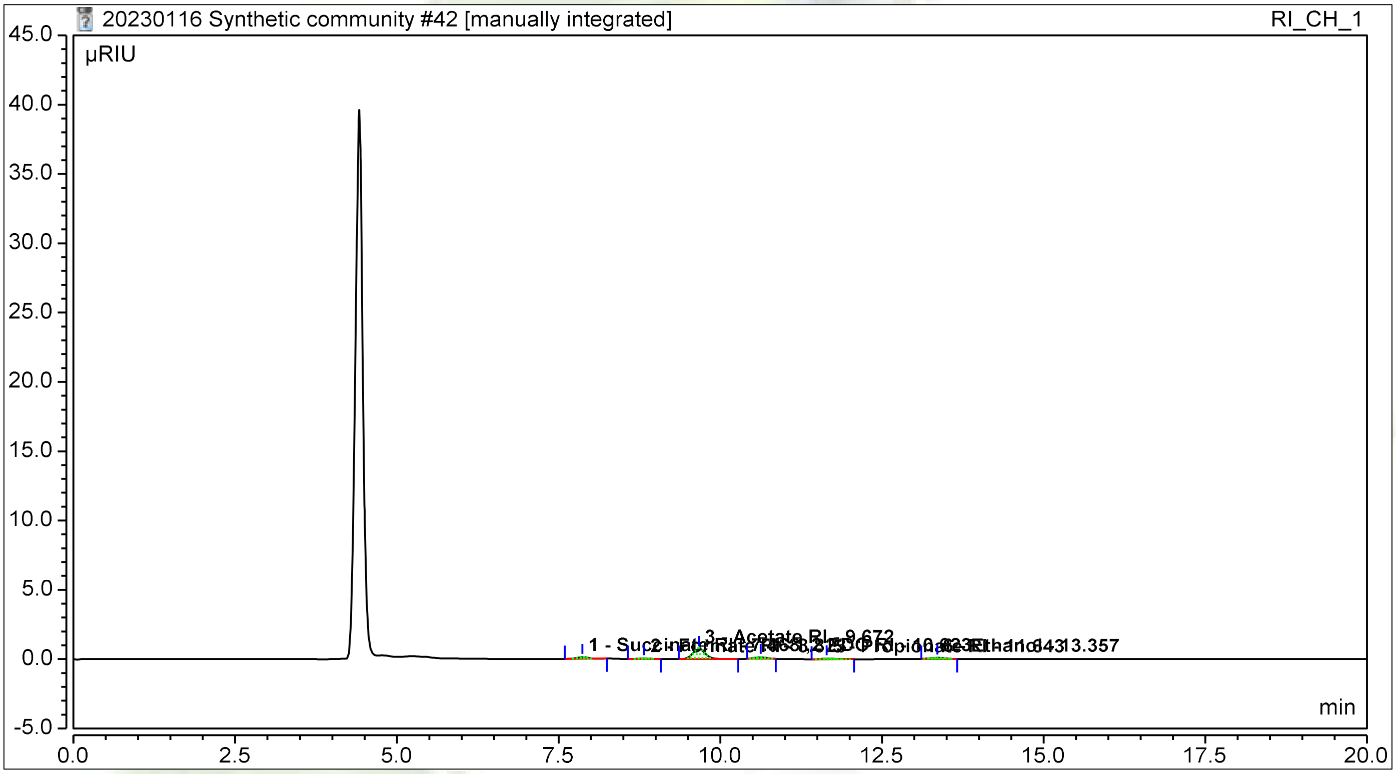

Peak Results

No.	Peak Name	Retention Time min	Width (50%) min	Type	Resolution (EP)	Asymmetry (EP)	Plates (EP)
n.a.	GlcNAc	n.a.	n.a.	n.a.	n.a.	n.a.	n.a.
n.a.	Citrate	n.a.	n.a.	n.a.	n.a.	n.a.	n.a.
n.a.	Glucose	n.a.	n.a.	n.a.	n.a.	n.a.	n.a.
n.a.	Galactose	n.a.	n.a.	n.a.	n.a.	n.a.	n.a.
n.a.	Fucose	n.a.	n.a.	n.a.	n.a.	n.a.	n.a.
1	Succinate RI	7,868	0,182	BMB*	3,01	0,94	10308
n.a.	Lactate RI	n.a.	n.a.	n.a.	n.a.	n.a.	n.a.
n.a.	glycerol	n.a.	n.a.	n.a.	n.a.	n.a.	n.a.
2	Formate RI	8,823	0,192	BMB*	2,44	1,03	11649
3	Acetate RI	9,672	0,218	BMB	2,53	1,05	10865
4	1,2 PDO RI	10,623	0,226	BMB*	2,53	1,04	12212
n.a.	1,3-PDO	n.a.	n.a.	n.a.	n.a.	n.a.	n.a.
5	Propionate RI	11,643	0,250	BMB*	3,88	1,63	11985
n.a.	1,3-PDO	n.a.	n.a.	n.a.	n.a.	n.a.	n.a.
n.a.	2-3 BDO	n.a.	n.a.	n.a.	n.a.	n.a.	n.a.
6	Ethanol	13,357	0,271	BMB*	n.a.	1,07	13489
n.a.	Isobutyrate RI	n.a.	n.a.	n.a.	n.a.	n.a.	n.a.
n.a.	Butyrate RI	n.a.	n.a.	n.a.	n.a.	n.a.	n.a.

Chromatogram and SST Results

Injection Details

Injection Name:	**MUCHMO1 t120 r2**	*Run Time (min):*	**20,00**
Vial Number:	**3:35**	*Injection Volume:*	**10,00**
Injection Type:	**Unknown**	*Channel:*	**RI_CH_1**
Calibration Level:		*Wavelength:*	**n.a.**
Instrument Method:	**Default method LC2030C 45 gr 20 min**	*Bandwidth:*	**n.a.**
Processing Method:	**Processing Method LC2030 45 gr**	*Dilution Factor:*	**1,0000**
Injection Date/Time:	**17/Jan/23 05:10**	*Sample Weight:*	**1,0000**

Chromatogram

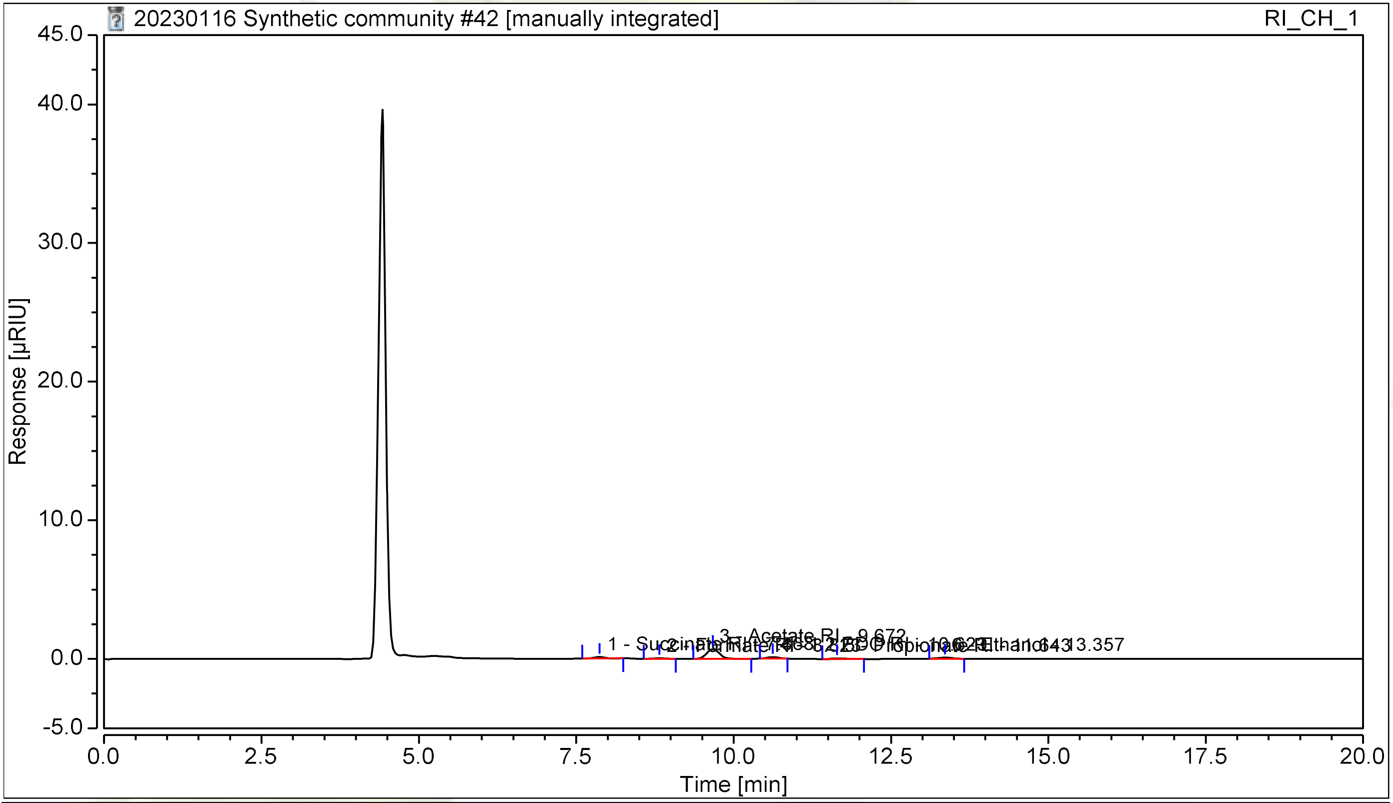

SST Results

No.	Name	Inj.Condition	Peak	Test Result	Injection
**Number of executed test cases:**	**n.a.**		**Total Result:**	**Passed**	

Chromatogram and Results

Injection Details

Injection Name:	**MUCHMO1 t120 r3**	*Run Time (min):*	**20,00**
Vial Number:	**3:36**	*Injection Volume:*	**10,00**
Injection Type:	**Unknown**	*Channel:*	**RI_CH_1**
Calibration Level:		*Wavelength:*	**n.a.**
Instrument Method:	**Default method LC2030C 45 gr 20 min**	*Bandwidth:*	**n.a.**
Processing Method:	**Processing Method LC2030 45 gr**	*Dilution Factor:*	**1,0000**
Injection Date/Time:	**17/Jan/23 05:30**	*Sample Weight:*	**1,0000**

Chromatogram

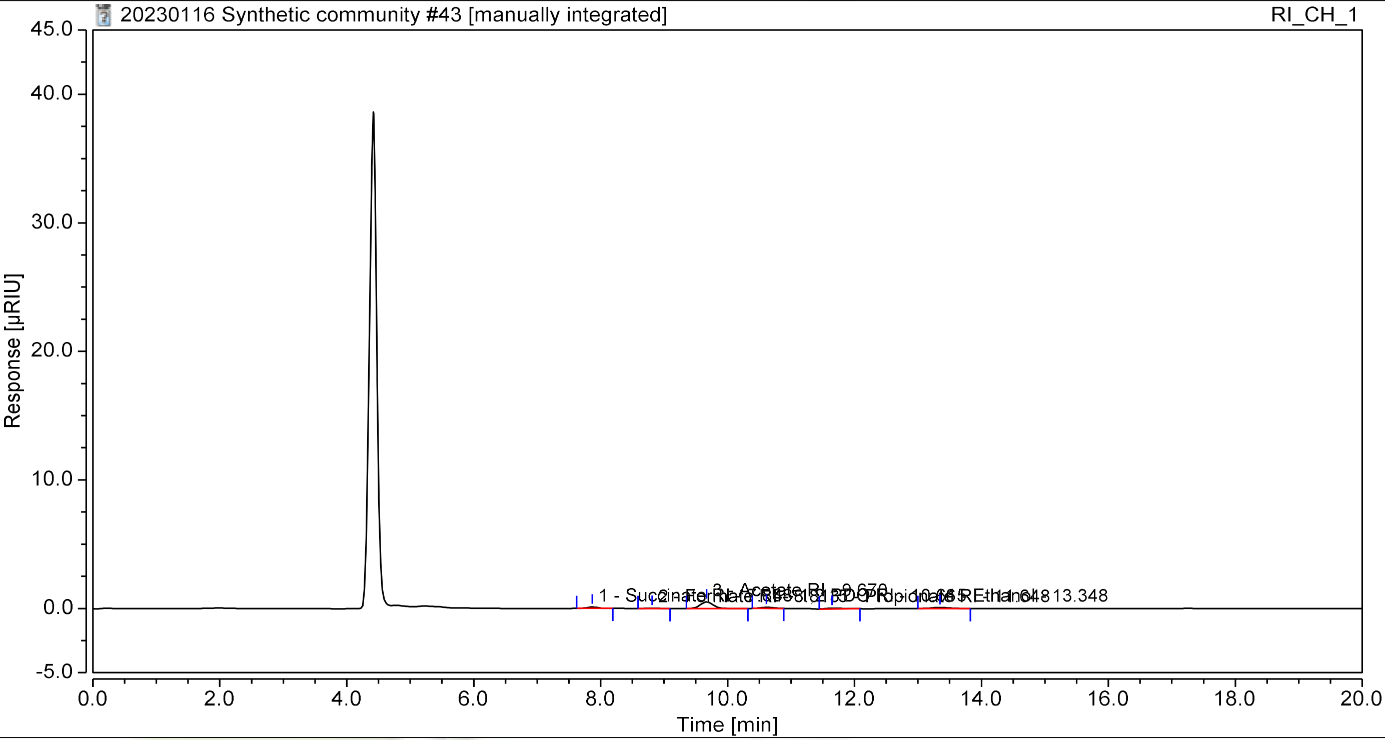

Integration Results

No.	Peak Name	Retention Time min	Area µRIU*min	Height µRIU	Relative Area %	Relative Height %	Amount
n.a.	GlcNAc	n.a.	n.a.	n.a.	n.a.	n.a.	n.a.
n.a.	Citrate	n.a.	n.a.	n.a.	n.a.	n.a.	n.a.
n.a.	Glucose	n.a.	n.a.	n.a.	n.a.	n.a.	n.a.
n.a.	Galactose	n.a.	n.a.	n.a.	n.a.	n.a.	n.a.
n.a.	Fucose	n.a.	n.a.	n.a.	n.a.	n.a.	n.a.
1	Succinate RI	7,868	0,021	0,103	10,06	12,07	0,4281
n.a.	Lactate RI	n.a.	n.a.	n.a.	n.a.	n.a.	n.a.
n.a.	glycerol	n.a.	n.a.	n.a.	n.a.	n.a.	n.a.
2	Formate RI	8,813	0,004	0,019	1,79	2,22	0,3779
3	Acetate RI	9,670	0,119	0,513	57,82	60,32	7,3926
4	1,2 PDO RI	10,615	0,019	0,080	9,33	9,42	0,5857
n.a.	1,3-PDO	n.a.	n.a.	n.a.	n.a.	n.a.	n.a.
5	Propionate RI	11,648	0,020	0,059	9,59	6,90	0,8270
n.a.	1,3-PDO	n.a.	n.a.	n.a.	n.a.	n.a.	n.a.
n.a.	2-3 BDO	n.a.	n.a.	n.a.	n.a.	n.a.	n.a.
6	Ethanol	13,348	0,024	0,077	11,40	9,07	2,4661
n.a.	Isobutyrate RI	n.a.	n.a.	n.a.	n.a.	n.a.	n.a.
n.a.	Butyrate RI	n.a.	n.a.	n.a.	n.a.	n.a.	n.a.
**Total:**			**0,206**	**0,850**	**100,00**	**100,00**	

Chromeleon (c) Dionex
Version 7.2.10.25868

Peak Analysis

Injection Details

Injection Name:	**MUCHMO1 t120 r3**	*Run Time (min):*	**20,00**
Vial Number:	**3:36**	*Injection Volume:*	**10,00**
Injection Type:	**Unknown**	*Channel:*	**RI_CH_1**
Calibration Level:		*Wavelength:*	**n.a.**
Instrument Method:	**Default method LC2030C 45 gr 20 min**	*Bandwidth:*	**n.a.**
Processing Method:	**Processing Method LC2030 45 gr**	*Dilution Factor:*	**1,0000**
Injection Date/Time:	**17/Jan/23 05:30**	*Sample Weight:*	**1,0000**

Chromatogram

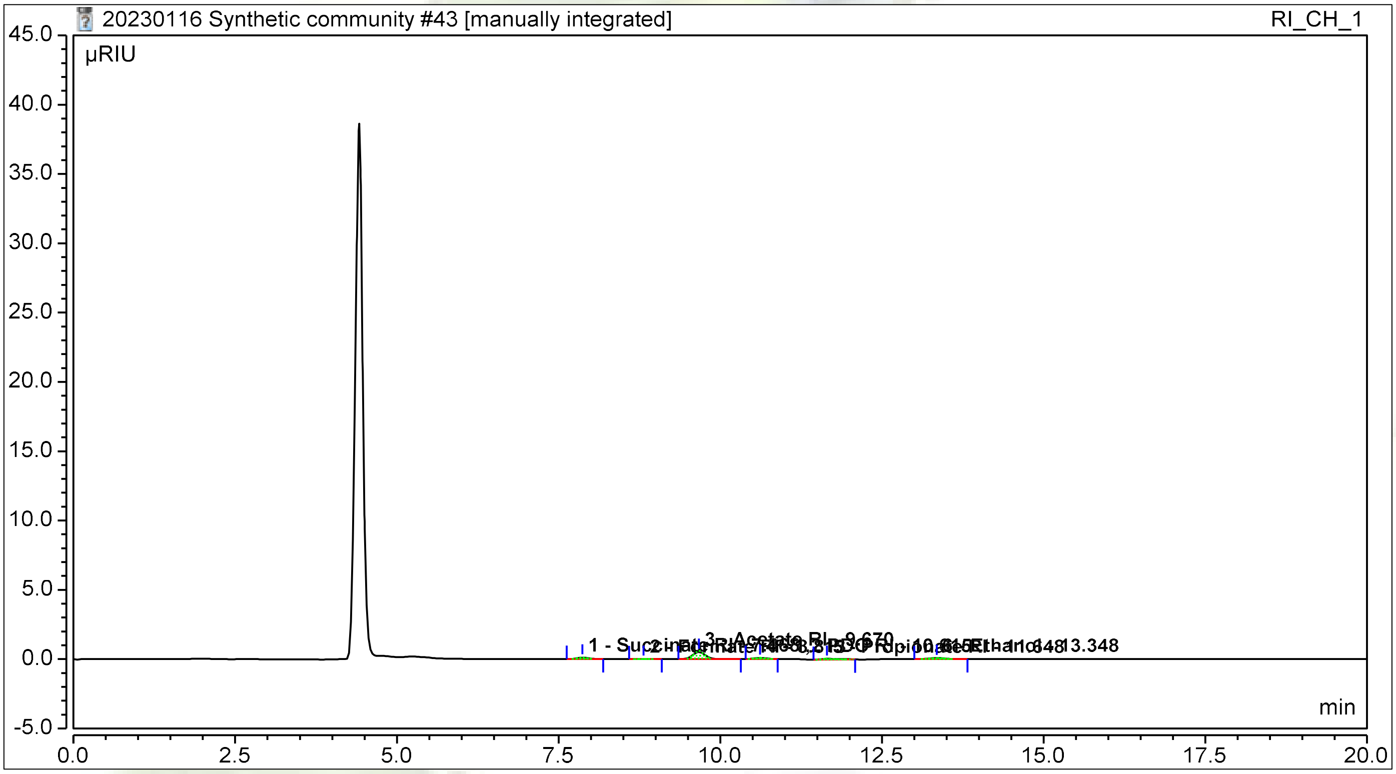

Peak Results

No.	Peak Name	Retention Time min	Width (50%) min	Type	Resolution (EP)	Asymmetry (EP)	Plates (EP)
n.a.	GlcNAc	n.a.	n.a.	n.a.	n.a.	n.a.	n.a.
n.a.	Citrate	n.a.	n.a.	n.a.	n.a.	n.a.	n.a.
n.a.	Glucose	n.a.	n.a.	n.a.	n.a.	n.a.	n.a.
n.a.	Galactose	n.a.	n.a.	n.a.	n.a.	n.a.	n.a.
n.a.	Fucose	n.a.	n.a.	n.a.	n.a.	n.a.	n.a.
1	Succinate RI	7,868	0,195	BMB*	2,92	1,03	9055
n.a.	Lactate RI	n.a.	n.a.	n.a.	n.a.	n.a.	n.a.
n.a.	glycerol	n.a.	n.a.	n.a.	n.a.	n.a.	n.a.
2	Formate RI	8,813	0,188	BMB*	2,49	1,03	12221
3	Acetate RI	9,670	0,218	BMB	2,46	1,05	10854
4	1,2 PDO RI	10,615	0,235	BMB*	2,24	1,08	11332
n.a.	1,3-PDO	n.a.	n.a.	n.a.	n.a.	n.a.	n.a.
5	Propionate RI	11,648	0,310	BMB*	3,36	1,65	7840
n.a.	1,3-PDO	n.a.	n.a.	n.a.	n.a.	n.a.	n.a.
n.a.	2-3 BDO	n.a.	n.a.	n.a.	n.a.	n.a.	n.a.
6	Ethanol	13,348	0,288	BMB*	n.a.	1,07	11930
n.a.	Isobutyrate RI	n.a.	n.a.	n.a.	n.a.	n.a.	n.a.
n.a.	Butyrate RI	n.a.	n.a.	n.a.	n.a.	n.a.	n.a.

Chromatogram and SST Results

Injection Details

Injection Name:	**MUCHMO1 t120 r3**	*Run Time (min):*	**20,00**
Vial Number:	**3:36**	*Injection Volume:*	**10,00**
Injection Type:	**Unknown**	*Channel:*	**RI_CH_1**
Calibration Level:		*Wavelength:*	**n.a.**
Instrument Method:	**Default method LC2030C 45 gr 20 min**	*Bandwidth:*	**n.a.**
Processing Method:	**Processing Method LC2030 45 gr**	*Dilution Factor:*	**1,0000**
Injection Date/Time:	**17/Jan/23 05:30**	*Sample Weight:*	**1,0000**

Chromatogram

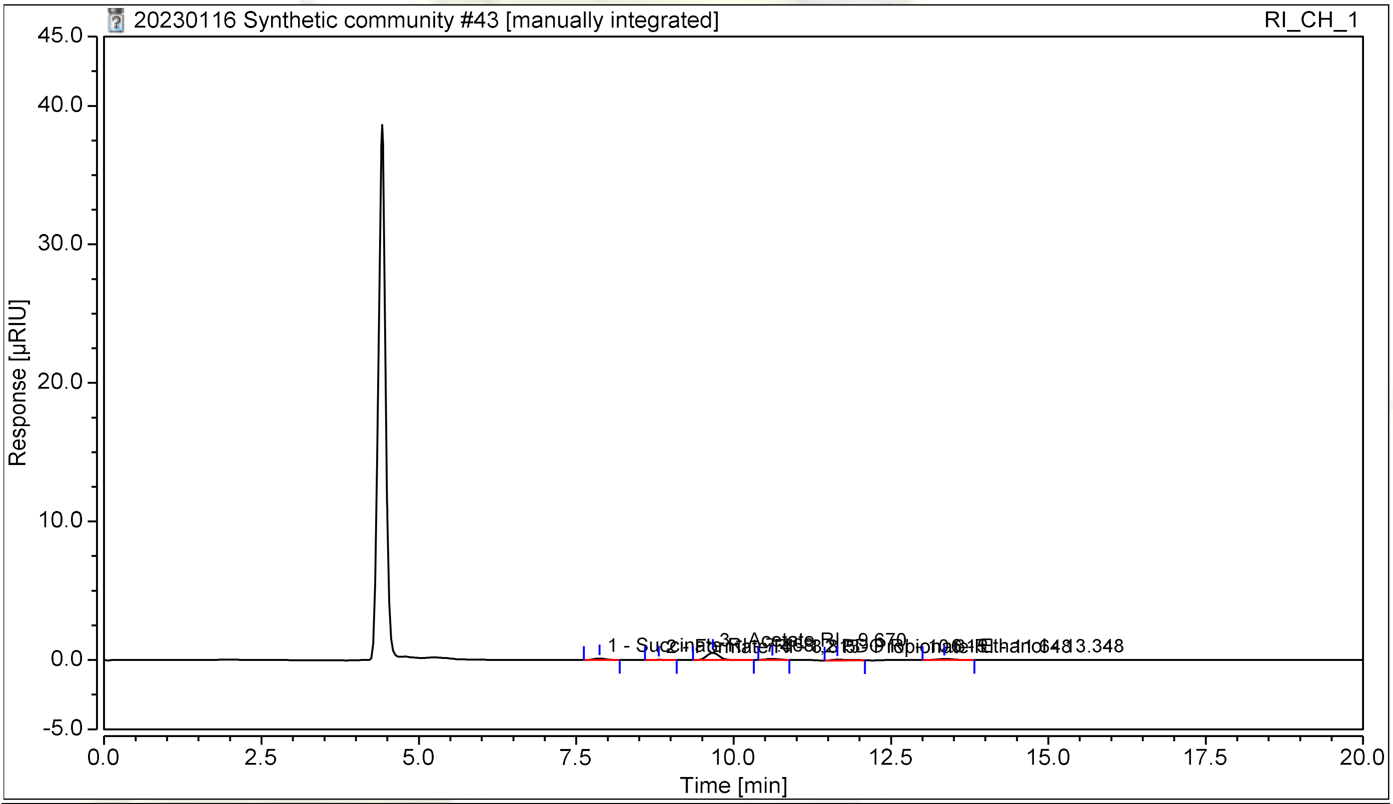

SST Results

No.	Name	Inj.Condition	Peak	Test Result	Injection
**Number of executed test cases:**	**n.a.**		**Total Result:**	**Passed**	

Chromatogram and Results

Injection Details

Injection Name:	**MUCHMO2 t72 r1**	*Run Time (min):*	**20,00**
Vial Number:	**3:37**	*Injection Volume:*	**10,00**
Injection Type:	**Unknown**	*Channel:*	**RI_CH_1**
Calibration Level:		*Wavelength:*	**n.a.**
Instrument Method:	**Default method LC2030C 45 gr 20 min**	*Bandwidth:*	**n.a.**
Processing Method:	**Processing Method LC2030 45 gr**	*Dilution Factor:*	**1,0000**
Injection Date/Time:	**17/Jan/23 05:51**	*Sample Weight:*	**1,0000**

Chromatogram

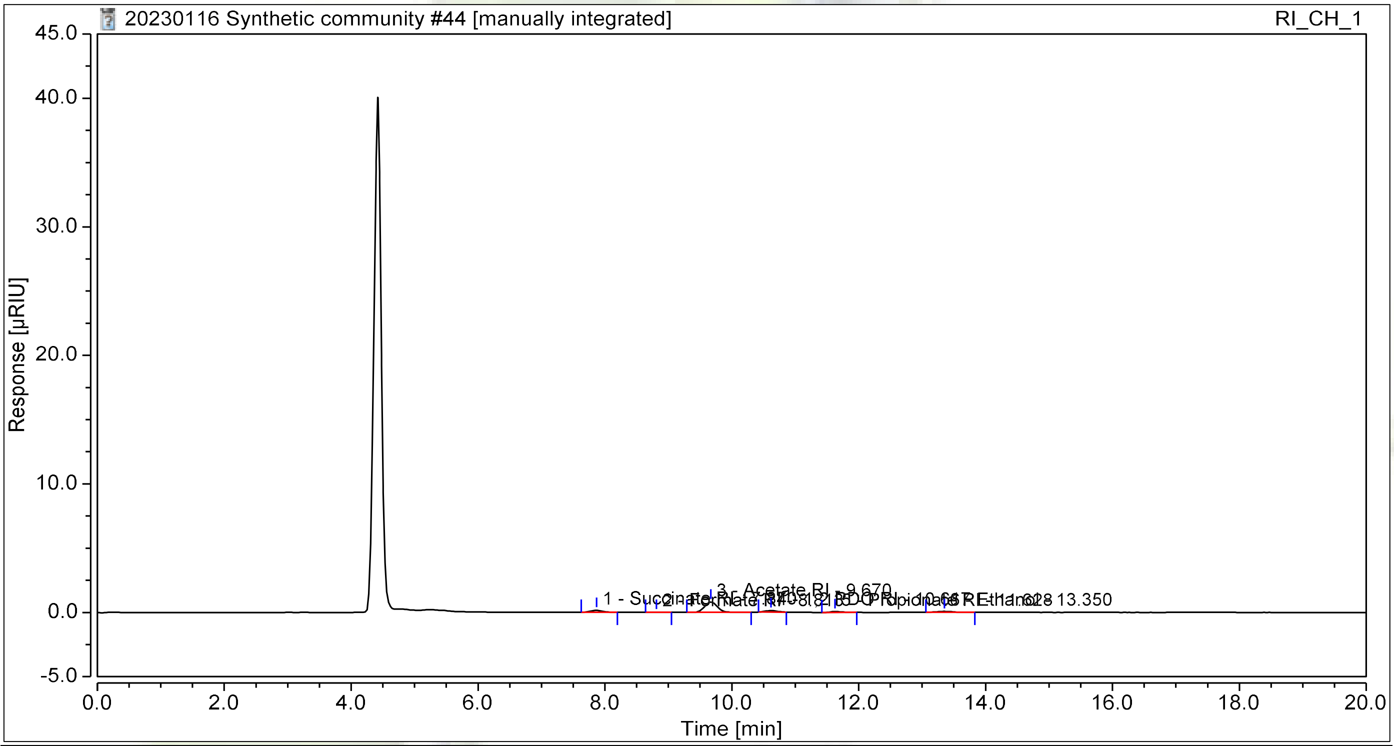

Integration Results

No.	Peak Name	Retention Time min	Area µRIU*min	Height µRIU	Relative Area %	Relative Height %	Amount
n.a.	GlcNAc	n.a.	n.a.	n.a.	n.a.	n.a.	n.a.
n.a.	Citrate	n.a.	n.a.	n.a.	n.a.	n.a.	n.a.
n.a.	Glucose	n.a.	n.a.	n.a.	n.a.	n.a.	n.a.
n.a.	Galactose	n.a.	n.a.	n.a.	n.a.	n.a.	n.a.
n.a.	Fucose	n.a.	n.a.	n.a.	n.a.	n.a.	n.a.
1	Succinate RI	7,870	0,028	0,138	9,88	11,49	0,5687
n.a.	Lactate RI	n.a.	n.a.	n.a.	n.a.	n.a.	n.a.
n.a.	glycerol	n.a.	n.a.	n.a.	n.a.	n.a.	n.a.
2	Formate RI	8,813	0,003	0,017	1,19	1,43	0,3401
3	Acetate RI	9,670	0,185	0,796	66,51	66,32	11,5000
4	1,2 PDO RI	10,617	0,025	0,110	8,98	9,15	0,7623
n.a.	1,3-PDO	n.a.	n.a.	n.a.	n.a.	n.a.	n.a.
5	Propionate RI	11,628	0,016	0,067	5,75	5,62	0,6706
n.a.	1,3-PDO	n.a.	n.a.	n.a.	n.a.	n.a.	n.a.
n.a.	2-3 BDO	n.a.	n.a.	n.a.	n.a.	n.a.	n.a.
6	Ethanol	13,350	0,021	0,072	7,68	6,00	2,2451
n.a.	Isobutyrate RI	n.a.	n.a.	n.a.	n.a.	n.a.	n.a.
n.a.	Butyrate RI	n.a.	n.a.	n.a.	n.a.	n.a.	n.a.
**Total:**			**0,279**	**1,200**	**100,00**	**100,00**	

Chromeleon (c) Dionex
Version 7.2.10.25868

Peak Analysis

Injection Details

Injection Name:	**MUCHMO2 t72 r1**	*Run Time (min):*	**20,00**
Vial Number:	**3:37**	*Injection Volume:*	**10,00**
Injection Type:	**Unknown**	*Channel:*	**RI_CH_1**
Calibration Level:		*Wavelength:*	**n.a.**
Instrument Method:	**Default method LC2030C 45 gr 20 min**	*Bandwidth:*	**n.a.**
Processing Method:	**Processing Method LC2030 45 gr**	*Dilution Factor:*	**1,0000**
Injection Date/Time:	**17/Jan/23 05:51**	*Sample Weight:*	**1,0000**

Chromatogram

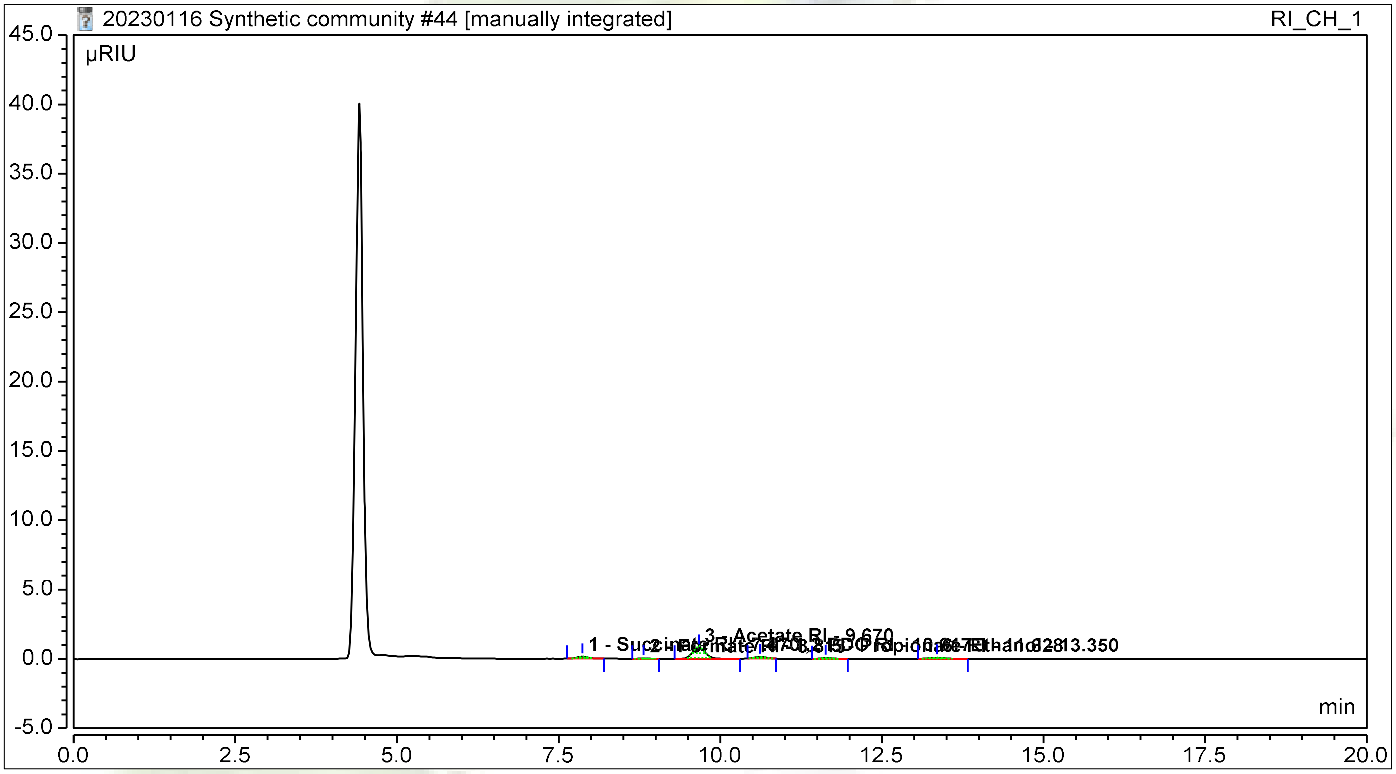

Peak Results

No.	Peak Name	Retention Time min	Width (50%) min	Type	Resolution (EP)	Asymmetry (EP)	Plates (EP)
n.a.	GlcNAc	n.a.	n.a.	n.a.	n.a.	n.a.	n.a.
n.a.	Citrate	n.a.	n.a.	n.a.	n.a.	n.a.	n.a.
n.a.	Glucose	n.a.	n.a.	n.a.	n.a.	n.a.	n.a.
n.a.	Galactose	n.a.	n.a.	n.a.	n.a.	n.a.	n.a.
n.a.	Fucose	n.a.	n.a.	n.a.	n.a.	n.a.	n.a.
1	Succinate RI	7,870	0,194	BMB*	2,90	1,00	9102
n.a.	Lactate RI	n.a.	n.a.	n.a.	n.a.	n.a.	n.a.
n.a.	glycerol	n.a.	n.a.	n.a.	n.a.	n.a.	n.a.
2	Formate RI	8,813	0,189	BMB*	2,48	1,08	11984
3	Acetate RI	9,670	0,218	BMB	2,52	1,05	10877
4	1,2 PDO RI	10,617	0,226	BMB*	2,63	1,09	12270
n.a.	1,3-PDO	n.a.	n.a.	n.a.	n.a.	n.a.	n.a.
5	Propionate RI	11,628	0,228	BMB*	3,97	1,26	14454
n.a.	1,3-PDO	n.a.	n.a.	n.a.	n.a.	n.a.	n.a.
n.a.	2-3 BDO	n.a.	n.a.	n.a.	n.a.	n.a.	n.a.
6	Ethanol	13,350	0,284	BMB*	n.a.	1,07	12232
n.a.	Isobutyrate RI	n.a.	n.a.	n.a.	n.a.	n.a.	n.a.
n.a.	Butyrate RI	n.a.	n.a.	n.a.	n.a.	n.a.	n.a.

Chromatogram and SST Results

Injection Details

Injection Name:	**MUCHMO2 t72 r1**	*Run Time (min):*	**20,00**
Vial Number:	**3:37**	*Injection Volume:*	**10,00**
Injection Type:	**Unknown**	*Channel:*	**RI_CH_1**
Calibration Level:		*Wavelength:*	**n.a.**
Instrument Method:	**Default method LC2030C 45 gr 20 min**	*Bandwidth:*	**n.a.**
Processing Method:	**Processing Method LC2030 45 gr**	*Dilution Factor:*	**1,0000**
Injection Date/Time:	**17/Jan/23 05:51**	*Sample Weight:*	**1,0000**

Chromatogram

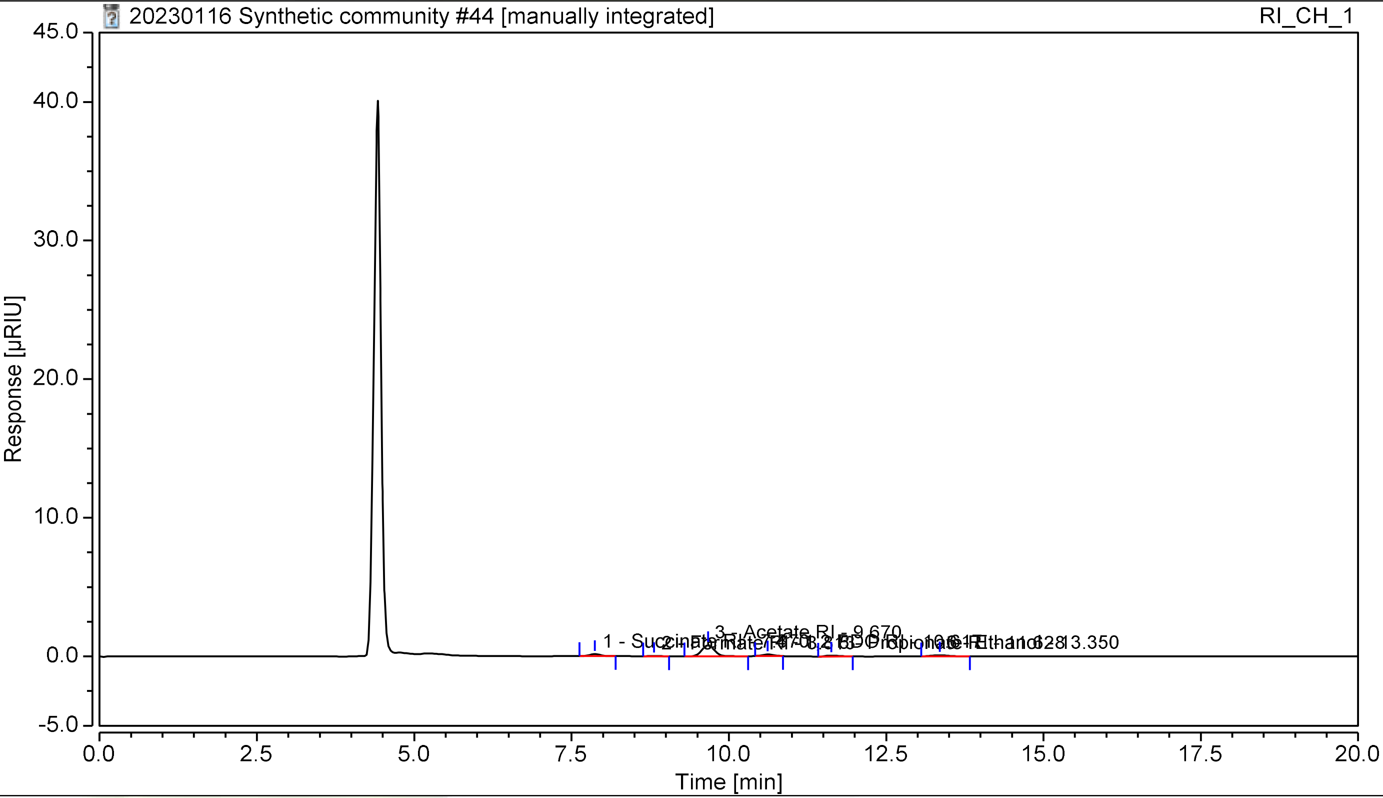

SST Results

No.	Name	Inj.Condition	Peak	Test Result	Injection

Number of executed test cases: **n.a.** **Total Result:** **Passed**

Chromatogram and Results

Injection Details

Injection Name:	**MUCHMO2 t72 r2**	*Run Time (min):*	**20,00**
Vial Number:	**3:38**	*Injection Volume:*	**10,00**
Injection Type:	**Unknown**	*Channel:*	**RI_CH_1**
Calibration Level:		*Wavelength:*	**n.a.**
Instrument Method:	**Default method LC2030C 45 gr 20 min**	*Bandwidth:*	**n.a.**
Processing Method:	**Processing Method LC2030 45 gr**	*Dilution Factor:*	**1,0000**
Injection Date/Time:	**17/Jan/23 06:11**	*Sample Weight:*	**1,0000**

Chromatogram

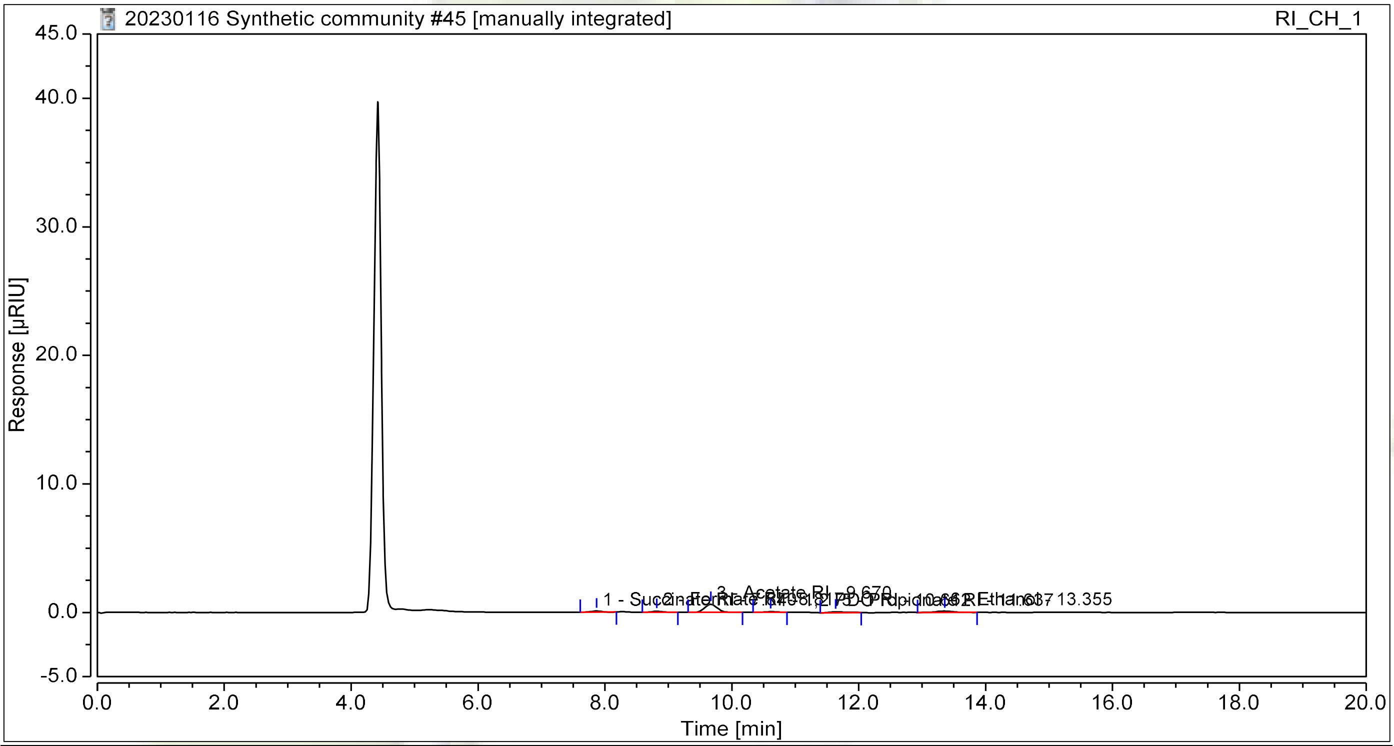

Integration Results

No.	Peak Name	Retention Time min	Area µRIU*min	Height µRIU	Relative Area %	Relative Height %	Amount
n.a.	GlcNAc	n.a.	n.a.	n.a.	n.a.	n.a.	n.a.
n.a.	Citrate	n.a.	n.a.	n.a.	n.a.	n.a.	n.a.
n.a.	Glucose	n.a.	n.a.	n.a.	n.a.	n.a.	n.a.
n.a.	Galactose	n.a.	n.a.	n.a.	n.a.	n.a.	n.a.
n.a.	Fucose	n.a.	n.a.	n.a.	n.a.	n.a.	n.a.
1	Succinate RI	7,870	0,013	0,082	5,66	8,21	0,2736
n.a.	Lactate RI	n.a.	n.a.	n.a.	n.a.	n.a.	n.a.
n.a.	glycerol	n.a.	n.a.	n.a.	n.a.	n.a.	n.a.
2	Formate RI	8,817	0,017	0,082	7,25	8,25	1,7357
3	Acetate RI	9,670	0,145	0,622	61,84	62,26	8,9748
4	1,2 PDO RI	10,612	0,012	0,049	5,16	4,94	0,3678
n.a.	1,3-PDO	n.a.	n.a.	n.a.	n.a.	n.a.	n.a.
5	Propionate RI	11,637	0,017	0,062	7,16	6,22	0,7010
n.a.	1,3-PDO	n.a.	n.a.	n.a.	n.a.	n.a.	n.a.
n.a.	2-3 BDO	n.a.	n.a.	n.a.	n.a.	n.a.	n.a.
6	Ethanol	13,355	0,030	0,101	12,92	10,12	3,1721
n.a.	Isobutyrate RI	n.a.	n.a.	n.a.	n.a.	n.a.	n.a.
n.a.	Butyrate RI	n.a.	n.a.	n.a.	n.a.	n.a.	n.a.
**Total:**			**0,234**	**1,000**	**100,00**	**100,00**	

Chromeleon (c) Dionex
Version 7.2.10.25868

Peak Analysis

Injection Details

Injection Name:	**MUCHMO2 t72 r2**	*Run Time (min):*	**20,00**
Vial Number:	**3:38**	*Injection Volume:*	**10,00**
Injection Type:	**Unknown**	*Channel:*	**RI_CH_1**
Calibration Level:		*Wavelength:*	**n.a.**
Instrument Method:	**Default method LC2030C 45 gr 20 min**	*Bandwidth:*	**n.a.**
Processing Method:	**Processing Method LC2030 45 gr**	*Dilution Factor:*	**1,0000**
Injection Date/Time:	**17/Jan/23 06:11**	*Sample Weight:*	**1,0000**

Chromatogram

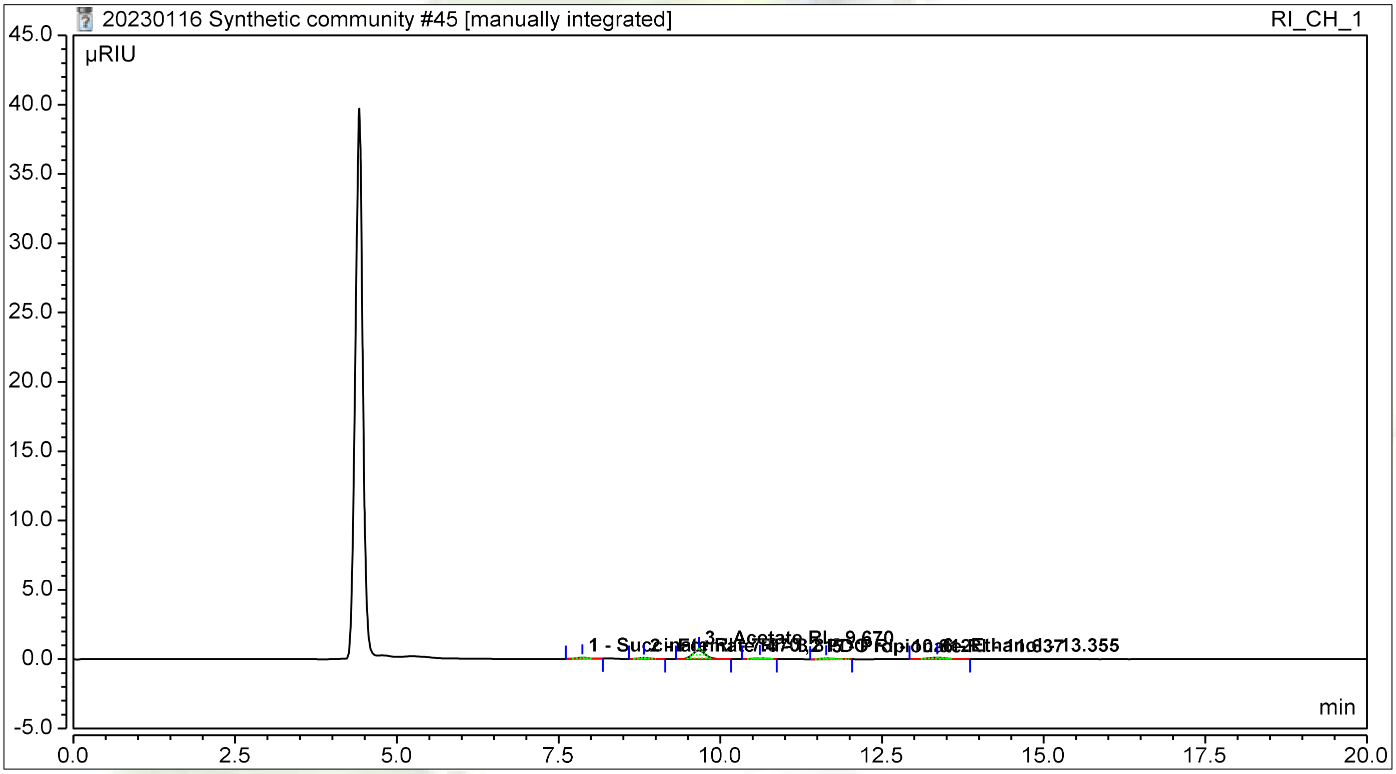

Peak Results

No.	Peak Name	Retention Time min	Width (50%) min	Type	Resolution (EP)	Asymmetry (EP)	Plates (EP)
n.a.	GlcNAc	n.a.	n.a.	n.a.	n.a.	n.a.	n.a.
n.a.	Citrate	n.a.	n.a.	n.a.	n.a.	n.a.	n.a.
n.a.	Glucose	n.a.	n.a.	n.a.	n.a.	n.a.	n.a.
n.a.	Galactose	n.a.	n.a.	n.a.	n.a.	n.a.	n.a.
n.a.	Fucose	n.a.	n.a.	n.a.	n.a.	n.a.	n.a.
1	Succinate RI	7,870	0,182	BMB*	2,98	0,90	10380
n.a.	Lactate RI	n.a.	n.a.	n.a.	n.a.	n.a.	n.a.
n.a.	glycerol	n.a.	n.a.	n.a.	n.a.	n.a.	n.a.
2	Formate RI	8,817	0,193	BMB*	2,45	1,09	11533
3	Acetate RI	9,670	0,219	BMB	2,45	1,04	10842
4	1,2 PDO RI	10,612	0,235	BMB*	2,54	1,01	11249
n.a.	1,3-PDO	n.a.	n.a.	n.a.	n.a.	n.a.	n.a.
5	Propionate RI	11,637	0,240	BMB*	3,85	1,56	12988
n.a.	1,3-PDO	n.a.	n.a.	n.a.	n.a.	n.a.	n.a.
n.a.	2-3 BDO	n.a.	n.a.	n.a.	n.a.	n.a.	n.a.
6	Ethanol	13,355	0,286	BMB*	n.a.	0,99	12069
n.a.	Isobutyrate RI	n.a.	n.a.	n.a.	n.a.	n.a.	n.a.
n.a.	Butyrate RI	n.a.	n.a.	n.a.	n.a.	n.a.	n.a.

Chromatogram and SST Results

Injection Details

Injection Name:	**MUCHMO2 t72 r2**	*Run Time (min):*	**20,00**
Vial Number:	**3:38**	*Injection Volume:*	**10,00**
Injection Type:	**Unknown**	*Channel:*	**RI_CH_1**
Calibration Level:		*Wavelength:*	**n.a.**
Instrument Method:	**Default method LC2030C 45 gr 20 min**	*Bandwidth:*	**n.a.**
Processing Method:	**Processing Method LC2030 45 gr**	*Dilution Factor:*	**1,0000**
Injection Date/Time:	**17/Jan/23 06:11**	*Sample Weight:*	**1,0000**

Chromatogram

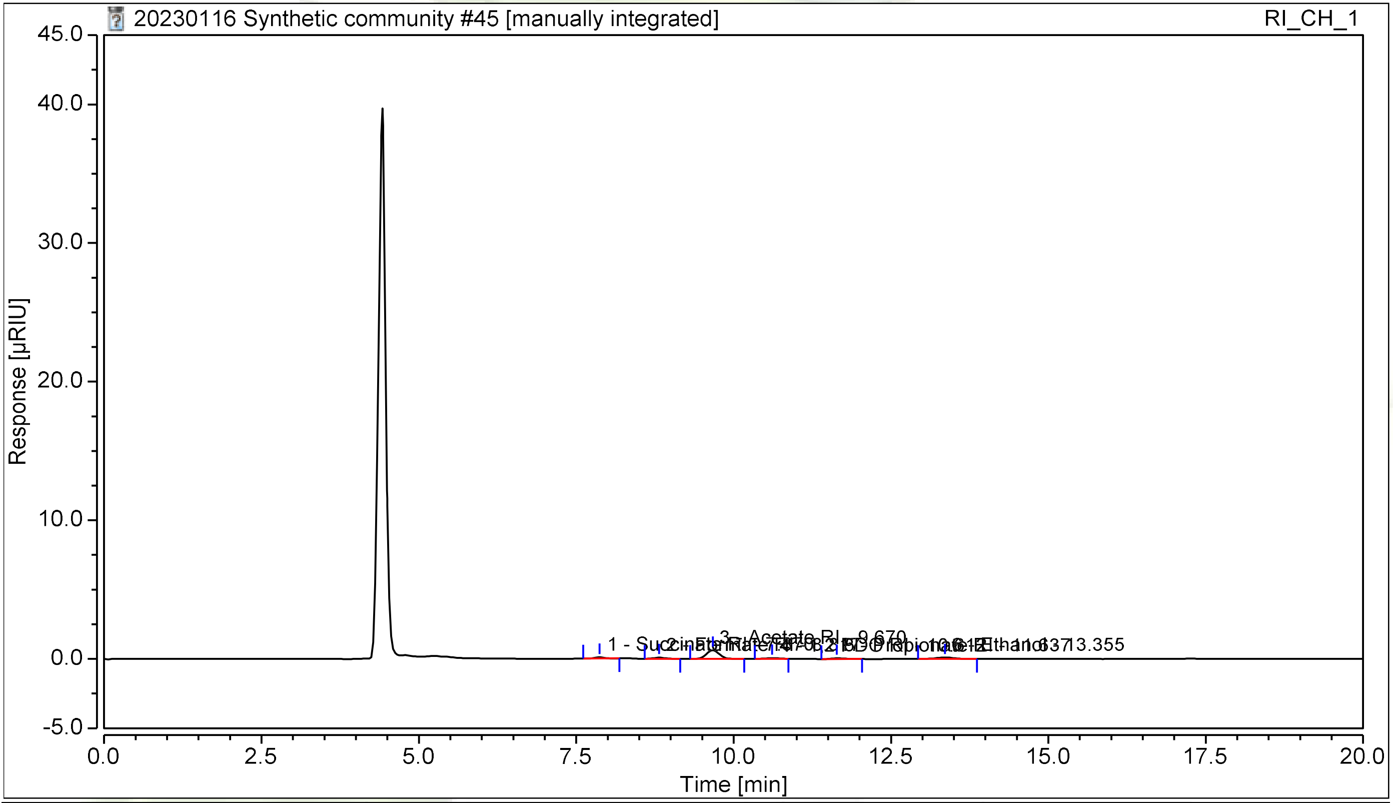

SST Results

No.	Name	Inj.Condition	Peak	Test Result	Injection
**Number of executed test cases:**	**n.a.**		**Total Result:**	**Passed**	

Chromatogram and Results

Injection Details

Injection Name:	**MUCHMO2 t72 r3**	*Run Time (min):*	**20,00**
Vial Number:	**3:39**	*Injection Volume:*	**10,00**
Injection Type:	**Unknown**	*Channel:*	**RI_CH_1**
Calibration Level:		*Wavelength:*	**n.a.**
Instrument Method:	**Default method LC2030C 45 gr 20 min**	*Bandwidth:*	**n.a.**
Processing Method:	**Processing Method LC2030 45 gr**	*Dilution Factor:*	**1,0000**
Injection Date/Time:	**17/Jan/23 06:32**	*Sample Weight:*	**1,0000**

Chromatogram

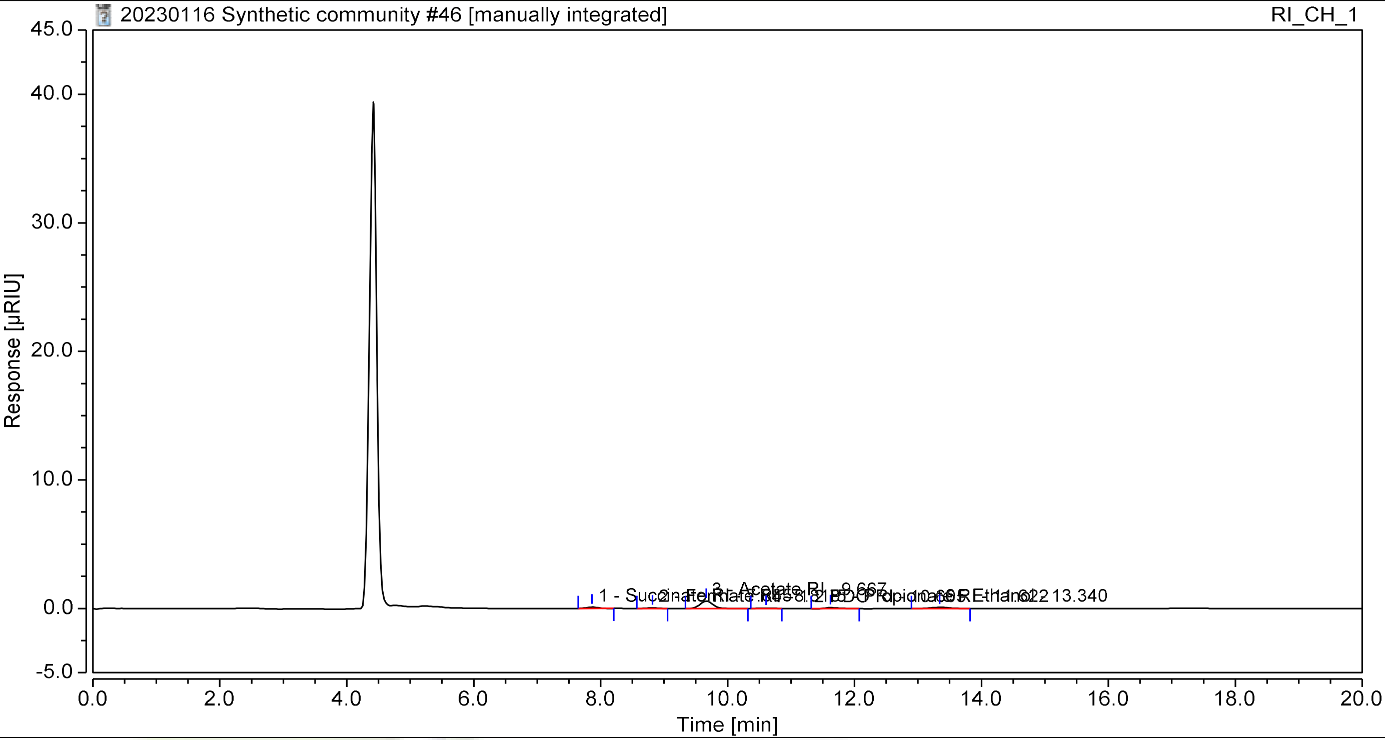

Integration Results

No.	Peak Name	Retention Time min	Area µRIU*min	Height µRIU	Relative Area %	Relative Height %	Amount
n.a.	GlcNAc	n.a.	n.a.	n.a.	n.a.	n.a.	n.a.
n.a.	Citrate	n.a.	n.a.	n.a.	n.a.	n.a.	n.a.
n.a.	Glucose	n.a.	n.a.	n.a.	n.a.	n.a.	n.a.
n.a.	Galactose	n.a.	n.a.	n.a.	n.a.	n.a.	n.a.
n.a.	Fucose	n.a.	n.a.	n.a.	n.a.	n.a.	n.a.
1	Succinate RI	7,865	0,020	0,102	9,44	11,15	0,4211
n.a.	Lactate RI	n.a.	n.a.	n.a.	n.a.	n.a.	n.a.
n.a.	glycerol	n.a.	n.a.	n.a.	n.a.	n.a.	n.a.
2	Formate RI	8,818	0,010	0,049	4,57	5,33	1,0101
3	Acetate RI	9,667	0,135	0,580	62,62	63,35	8,3945
4	1,2 PDO RI	10,605	0,005	0,022	2,41	2,35	0,1585
n.a.	1,3-PDO	n.a.	n.a.	n.a.	n.a.	n.a.	n.a.
5	Propionate RI	11,622	0,019	0,076	8,81	8,28	0,7966
n.a.	1,3-PDO	n.a.	n.a.	n.a.	n.a.	n.a.	n.a.
n.a.	2-3 BDO	n.a.	n.a.	n.a.	n.a.	n.a.	n.a.
6	Ethanol	13,340	0,026	0,087	12,16	9,54	2,7577
n.a.	Isobutyrate RI	n.a.	n.a.	n.a.	n.a.	n.a.	n.a.
n.a.	Butyrate RI	n.a.	n.a.	n.a.	n.a.	n.a.	n.a.
**Total:**			**0,216**	**0,916**	**100,00**	**100,00**	

Peak Analysis

Injection Details

Injection Name:	**MUCHMO2 t72 r3**	*Run Time (min):*	**20,00**
Vial Number:	**3:39**	*Injection Volume:*	**10,00**
Injection Type:	**Unknown**	*Channel:*	**RI_CH_1**
Calibration Level:		*Wavelength:*	**n.a.**
Instrument Method:	**Default method LC2030C 45 gr 20 min**	*Bandwidth:*	**n.a.**
Processing Method:	**Processing Method LC2030 45 gr**	*Dilution Factor:*	**1,0000**
Injection Date/Time:	**17/Jan/23 06:32**	*Sample Weight:*	**1,0000**

Chromatogram

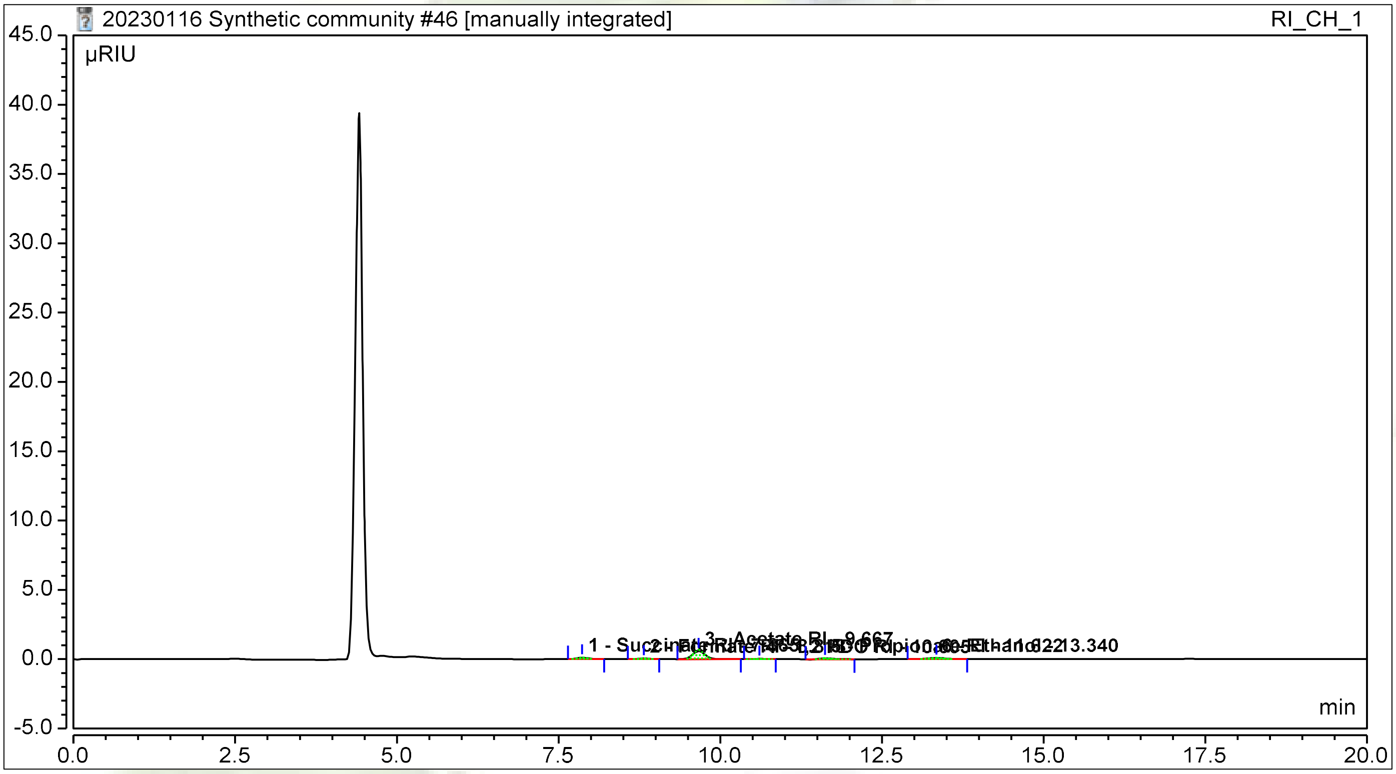

Peak Results

No.	Peak Name	Retention Time min	Width (50%) min	Type	Resolution (EP)	Asymmetry (EP)	Plates (EP)
n.a.	GlcNAc	n.a.	n.a.	n.a.	n.a.	n.a.	n.a.
n.a.	Citrate	n.a.	n.a.	n.a.	n.a.	n.a.	n.a.
n.a.	Glucose	n.a.	n.a.	n.a.	n.a.	n.a.	n.a.
n.a.	Galactose	n.a.	n.a.	n.a.	n.a.	n.a.	n.a.
n.a.	Fucose	n.a.	n.a.	n.a.	n.a.	n.a.	n.a.
1	Succinate RI	7,865	0,194	BMB*	2,90	1,03	9086
n.a.	Lactate RI	n.a.	n.a.	n.a.	n.a.	n.a.	n.a.
n.a.	glycerol	n.a.	n.a.	n.a.	n.a.	n.a.	n.a.
2	Formate RI	8,818	0,194	BMB*	2,43	1,02	11467
3	Acetate RI	9,667	0,219	BMB	2,44	1,05	10827
4	1,2 PDO RI	10,605	0,235	BMB*	2,58	1,00	11281
n.a.	1,3-PDO	n.a.	n.a.	n.a.	n.a.	n.a.	n.a.
5	Propionate RI	11,622	0,230	BMB*	3,91	1,89	14163
n.a.	1,3-PDO	n.a.	n.a.	n.a.	n.a.	n.a.	n.a.
n.a.	2-3 BDO	n.a.	n.a.	n.a.	n.a.	n.a.	n.a.
6	Ethanol	13,340	0,288	BMB*	n.a.	1,05	11845
n.a.	Isobutyrate RI	n.a.	n.a.	n.a.	n.a.	n.a.	n.a.
n.a.	Butyrate RI	n.a.	n.a.	n.a.	n.a.	n.a.	n.a.

Chromatogram and SST Results

Injection Details

Injection Name:	**MUCHMO2 t72 r3**	*Run Time (min):*	**20,00**
Vial Number:	**3:39**	*Injection Volume:*	**10,00**
Injection Type:	**Unknown**	*Channel:*	**RI_CH_1**
Calibration Level:		*Wavelength:*	**n.a.**
Instrument Method:	**Default method LC2030C 45 gr 20 min**	*Bandwidth:*	**n.a.**
Processing Method:	**Processing Method LC2030 45 gr**	*Dilution Factor:*	**1,0000**
Injection Date/Time:	**17/Jan/23 06:32**	*Sample Weight:*	**1,0000**

Chromatogram

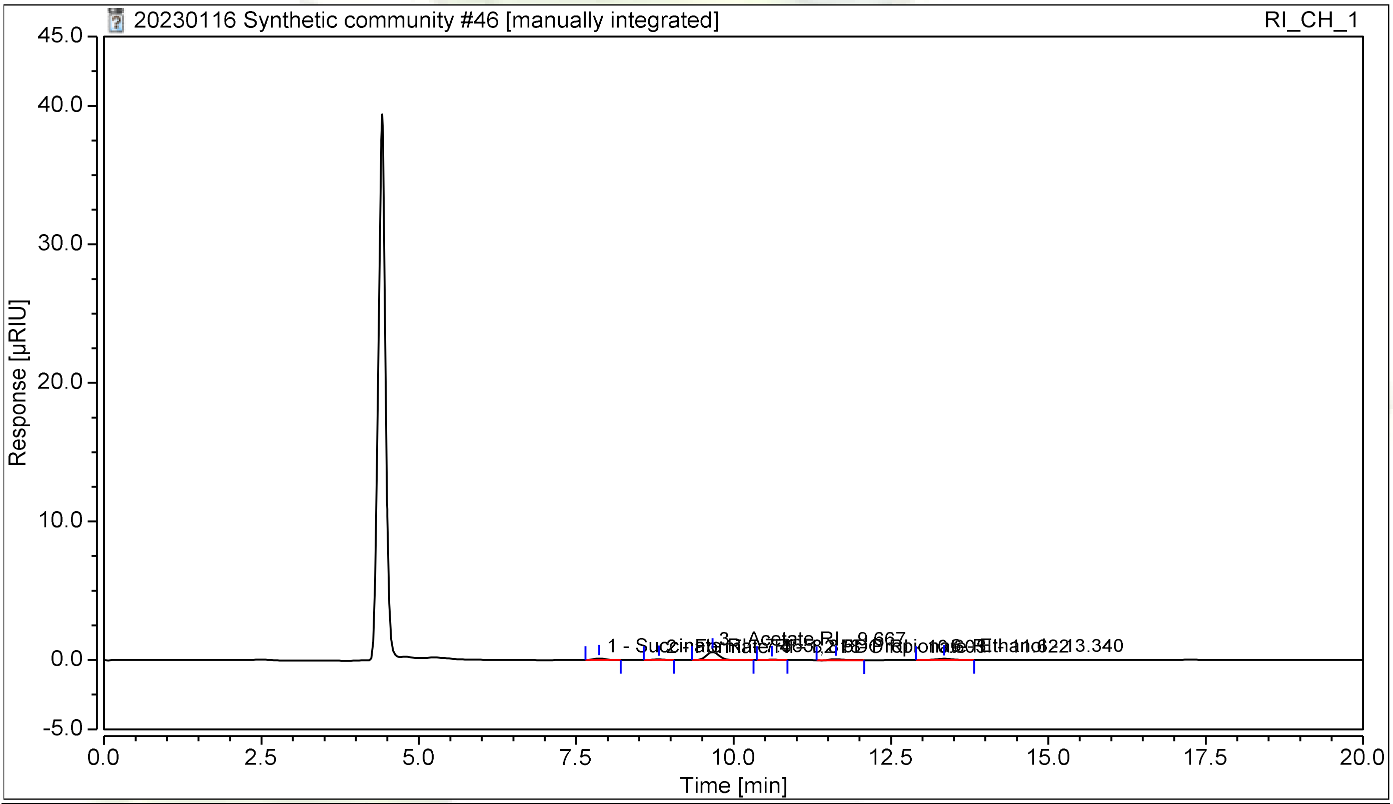

SST Results

No.	Name	Inj.Condition	Peak	Test Result	Injection

Number of executed test cases: **n.a.** **Total Result:** **Passed**

Chromatogram and Results

Injection Details

Injection Name:	**MUCHMO2 t96 r1**	*Run Time (min):*	**20,00**
Vial Number:	**3:40**	*Injection Volume:*	**10,00**
Injection Type:	**Unknown**	*Channel:*	**RI_CH_1**
Calibration Level:		*Wavelength:*	**n.a.**
Instrument Method:	**Default method LC2030C 45 gr 20 min**	*Bandwidth:*	**n.a.**
Processing Method:	**Processing Method LC2030 45 gr**	*Dilution Factor:*	**1,0000**
Injection Date/Time:	**17/Jan/23 06:52**	*Sample Weight:*	**1,0000**

Chromatogram

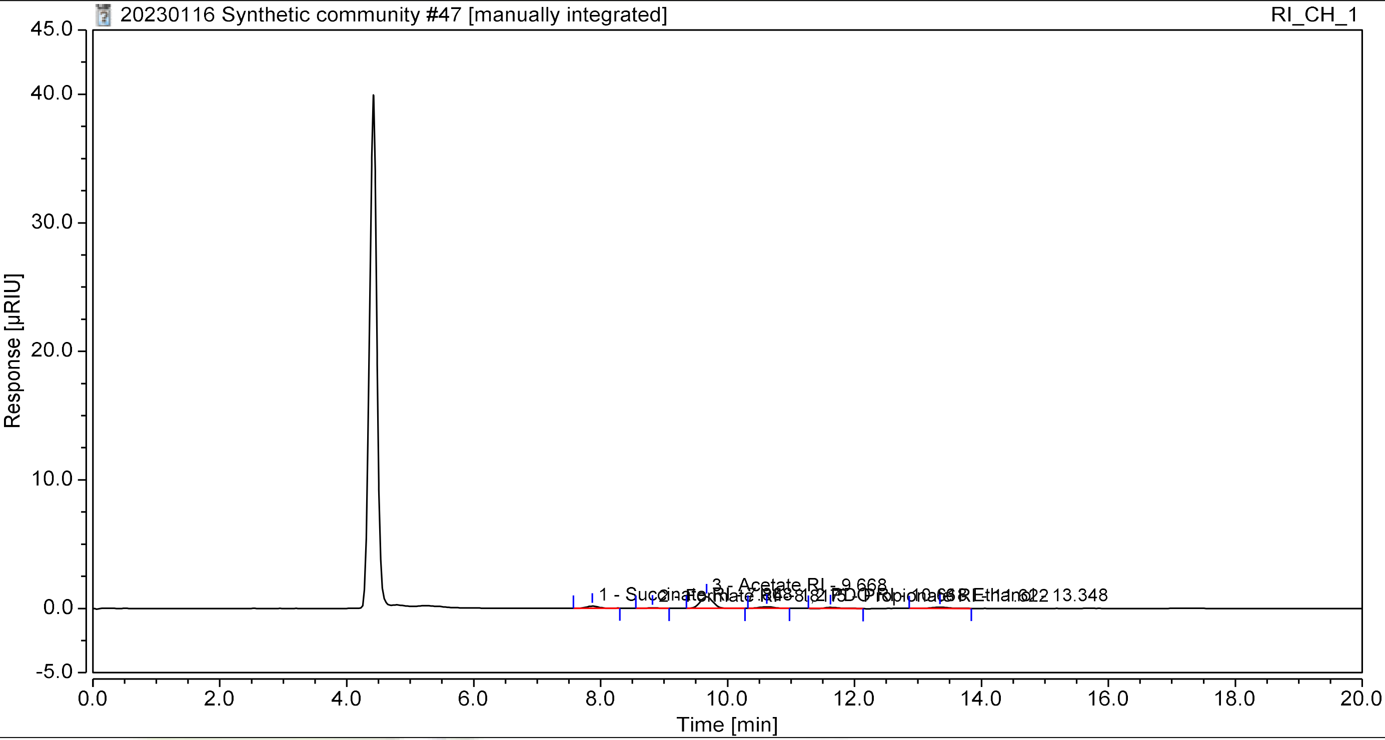

Integration Results

No.	Peak Name	Retention Time min	Area µRIU*min	Height µRIU	Relative Area %	Relative Height %	Amount
n.a.	GlcNAc	n.a.	n.a.	n.a.	n.a.	n.a.	n.a.
n.a.	Citrate	n.a.	n.a.	n.a.	n.a.	n.a.	n.a.
n.a.	Glucose	n.a.	n.a.	n.a.	n.a.	n.a.	n.a.
n.a.	Galactose	n.a.	n.a.	n.a.	n.a.	n.a.	n.a.
n.a.	Fucose	n.a.	n.a.	n.a.	n.a.	n.a.	n.a.
1	Succinate RI	7,868	0,032	0,161	9,85	11,67	0,6648
n.a.	Lactate RI	n.a.	n.a.	n.a.	n.a.	n.a.	n.a.
n.a.	glycerol	n.a.	n.a.	n.a.	n.a.	n.a.	n.a.
2	Formate RI	8,817	0,007	0,034	2,11	2,46	0,7072
3	Acetate RI	9,668	0,209	0,897	63,95	65,19	12,9668
4	1,2 PDO RI	10,618	0,029	0,115	8,99	8,33	0,8949
n.a.	1,3-PDO	n.a.	n.a.	n.a.	n.a.	n.a.	n.a.
5	Propionate RI	11,622	0,022	0,082	6,88	5,94	0,9404
n.a.	1,3-PDO	n.a.	n.a.	n.a.	n.a.	n.a.	n.a.
n.a.	2-3 BDO	n.a.	n.a.	n.a.	n.a.	n.a.	n.a.
6	Ethanol	13,348	0,027	0,088	8,21	6,42	2,8174
n.a.	Isobutyrate RI	n.a.	n.a.	n.a.	n.a.	n.a.	n.a.
n.a.	Butyrate RI	n.a.	n.a.	n.a.	n.a.	n.a.	n.a.
**Total:**			**0,327**	**1,377**	**100,00**	**100,00**	

Chromeleon (c) Dionex
Version 7.2.10.25868

Peak Analysis

Injection Details

Injection Name:	**MUCHMO2 t96 r1**	*Run Time (min):*	**20,00**
Vial Number:	**3:40**	*Injection Volume:*	**10,00**
Injection Type:	**Unknown**	*Channel:*	**RI_CH_1**
Calibration Level:		*Wavelength:*	**n.a.**
Instrument Method:	**Default method LC2030C 45 gr 20 min**	*Bandwidth:*	**n.a.**
Processing Method:	**Processing Method LC2030 45 gr**	*Dilution Factor:*	**1,0000**
Injection Date/Time:	**17/Jan/23 06:52**	*Sample Weight:*	**1,0000**

Chromatogram

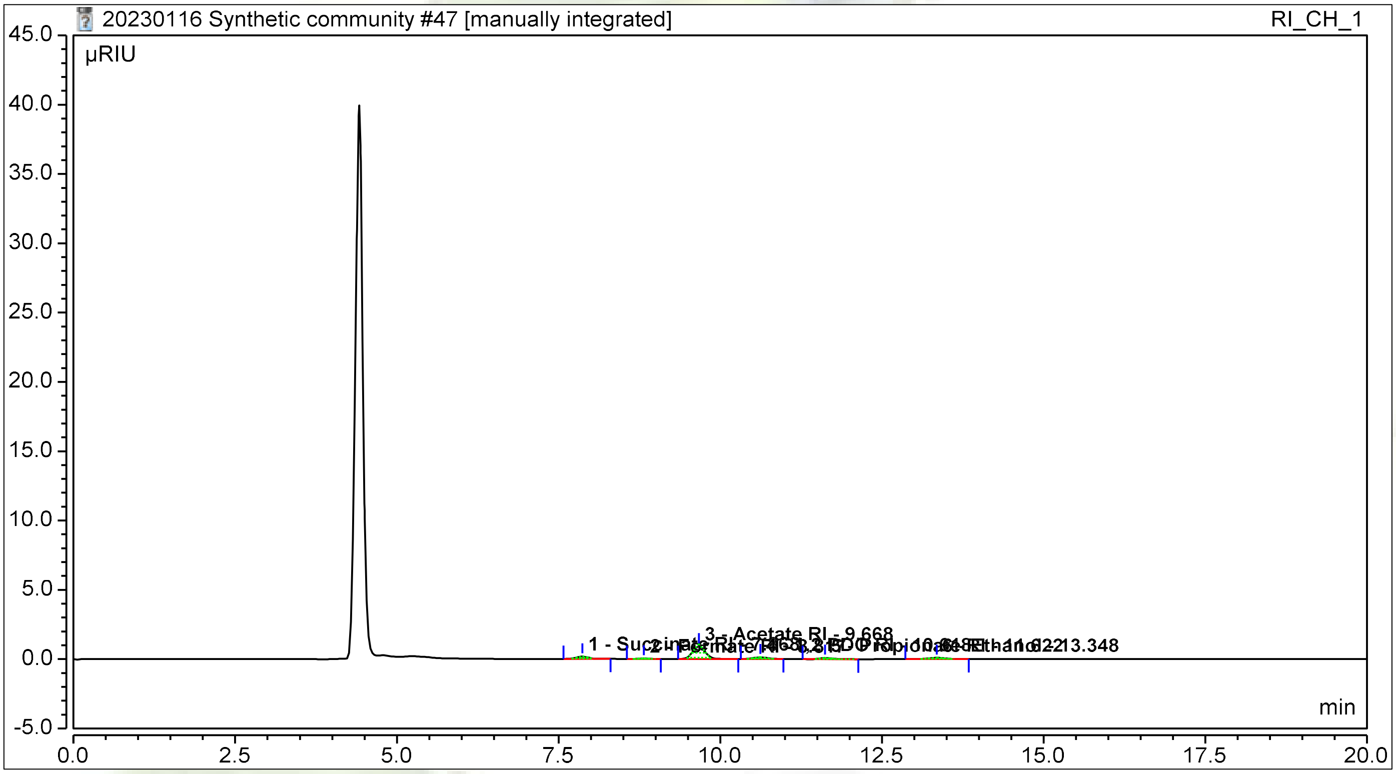

Peak Results

No.	Peak Name	Retention Time min	Width (50%) min	Type	Resolution (EP)	Asymmetry (EP)	Plates (EP)
n.a.	GlcNAc	n.a.	n.a.	n.a.	n.a.	n.a.	n.a.
n.a.	Citrate	n.a.	n.a.	n.a.	n.a.	n.a.	n.a.
n.a.	Glucose	n.a.	n.a.	n.a.	n.a.	n.a.	n.a.
n.a.	Galactose	n.a.	n.a.	n.a.	n.a.	n.a.	n.a.
n.a.	Fucose	n.a.	n.a.	n.a.	n.a.	n.a.	n.a.
1	Succinate RI	7,868	0,195	BMB*	2,90	0,99	9049
n.a.	Lactate RI	n.a.	n.a.	n.a.	n.a.	n.a.	n.a.
n.a.	glycerol	n.a.	n.a.	n.a.	n.a.	n.a.	n.a.
2	Formate RI	8,817	0,191	BMB*	2,45	1,04	11793
3	Acetate RI	9,668	0,219	BMB	2,43	1,05	10817
4	1,2 PDO RI	10,618	0,242	BMB*	2,48	1,04	10638
n.a.	1,3-PDO	n.a.	n.a.	n.a.	n.a.	n.a.	n.a.
5	Propionate RI	11,622	0,235	BMB*	3,90	2,03	13506
n.a.	1,3-PDO	n.a.	n.a.	n.a.	n.a.	n.a.	n.a.
n.a.	2-3 BDO	n.a.	n.a.	n.a.	n.a.	n.a.	n.a.
6	Ethanol	13,348	0,287	BMB*	n.a.	1,06	11995
n.a.	Isobutyrate RI	n.a.	n.a.	n.a.	n.a.	n.a.	n.a.
n.a.	Butyrate RI	n.a.	n.a.	n.a.	n.a.	n.a.	n.a.

Chromatogram and SST Results

Injection Details

Injection Name:	**MUCHMO2 t96 r1**	*Run Time (min):*	**20,00**
Vial Number:	**3:40**	*Injection Volume:*	**10,00**
Injection Type:	**Unknown**	*Channel:*	**RI_CH_1**
Calibration Level:		*Wavelength:*	**n.a.**
Instrument Method:	**Default method LC2030C 45 gr 20 min**	*Bandwidth:*	**n.a.**
Processing Method:	**Processing Method LC2030 45 gr**	*Dilution Factor:*	**1,0000**
Injection Date/Time:	**17/Jan/23 06:52**	*Sample Weight:*	**1,0000**

Chromatogram

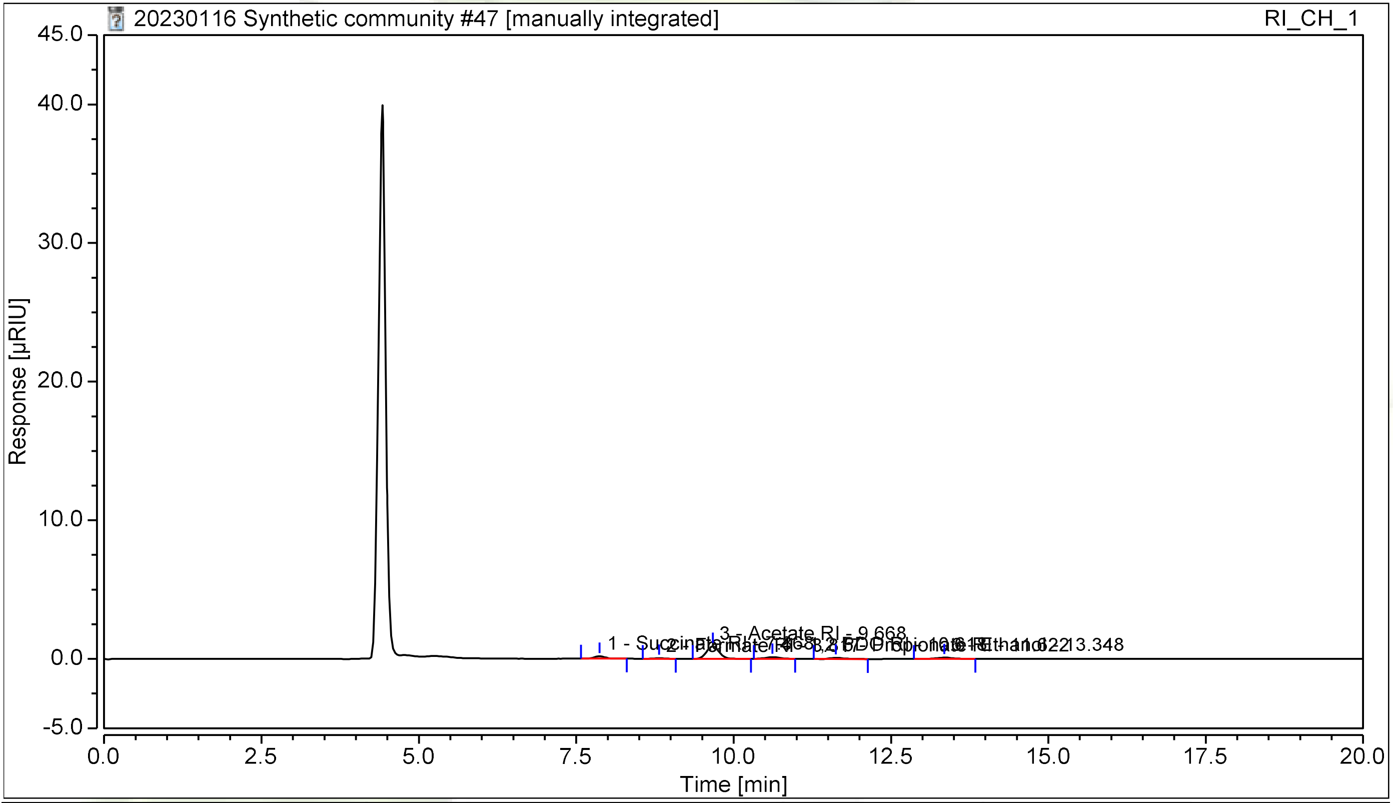

SST Results

No.	Name	Inj.Condition	Peak	Test Result	Injection
**Number of executed test cases:**	**n.a.**		**Total Result:**	**Passed**	

Chromatogram and Results

Injection Details

Injection Name:	**MUCHMO2 t96 r2**	*Run Time (min):*	**20,00**
Vial Number:	**3:41**	*Injection Volume:*	**10,00**
Injection Type:	**Unknown**	*Channel:*	**RI_CH_1**
Calibration Level:		*Wavelength:*	**n.a.**
Instrument Method:	**Default method LC2030C 45 gr 20 min**	*Bandwidth:*	**n.a.**
Processing Method:	**Processing Method LC2030 45 gr**	*Dilution Factor:*	**1,0000**
Injection Date/Time:	**17/Jan/23 07:12**	*Sample Weight:*	**1,0000**

Chromatogram

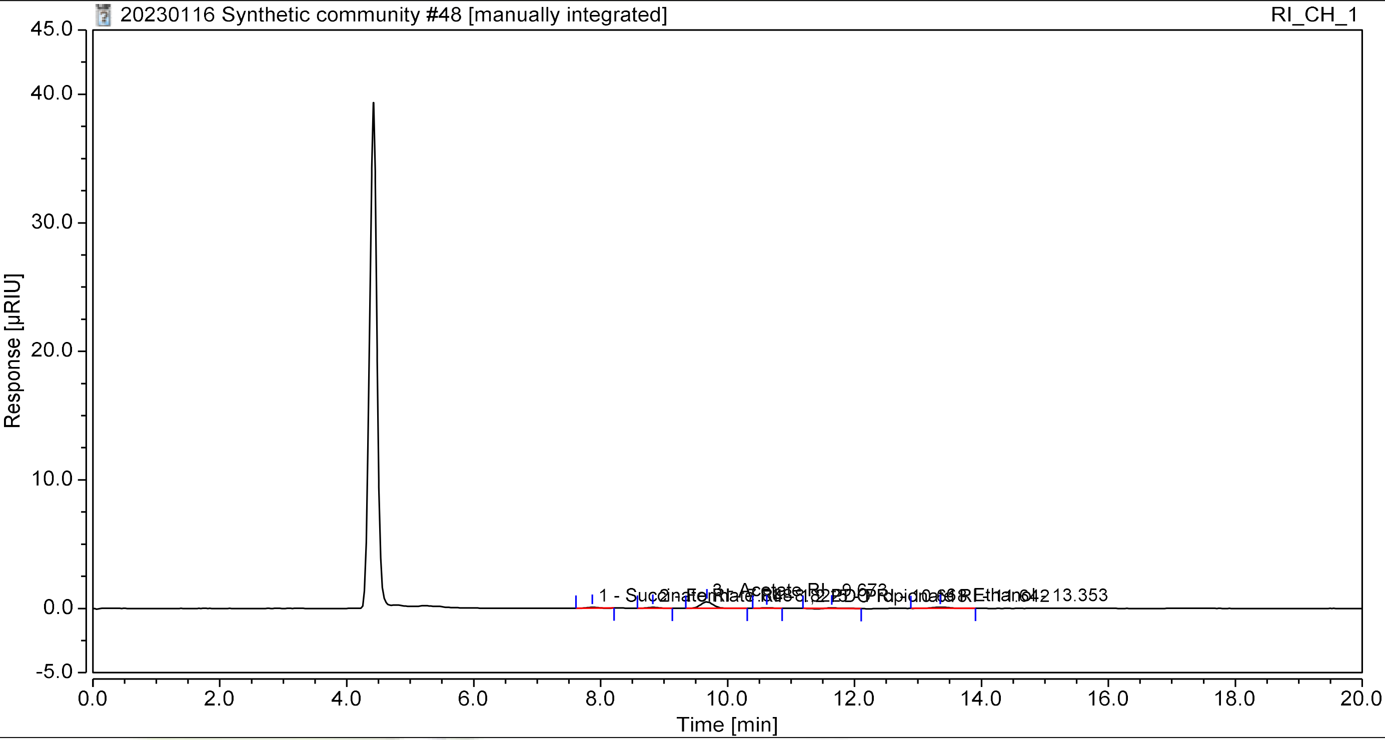

Integration Results

No.	Peak Name	Retention Time min	Area µRIU*min	Height µRIU	Relative Area %	Relative Height %	Amount
n.a.	GlcNAc	n.a.	n.a.	n.a.	n.a.	n.a.	n.a.
n.a.	Citrate	n.a.	n.a.	n.a.	n.a.	n.a.	n.a.
n.a.	Glucose	n.a.	n.a.	n.a.	n.a.	n.a.	n.a.
n.a.	Galactose	n.a.	n.a.	n.a.	n.a.	n.a.	n.a.
n.a.	Fucose	n.a.	n.a.	n.a.	n.a.	n.a.	n.a.
1	Succinate RI	7,868	0,009	0,062	5,09	7,71	0,1935
n.a.	Lactate RI	n.a.	n.a.	n.a.	n.a.	n.a.	n.a.
n.a.	glycerol	n.a.	n.a.	n.a.	n.a.	n.a.	n.a.
2	Formate RI	8,822	0,015	0,074	8,05	9,15	1,5166
3	Acetate RI	9,673	0,117	0,500	63,50	62,28	7,2455
4	1,2 PDO RI	10,618	0,007	0,030	3,82	3,71	0,2138
n.a.	1,3-PDO	n.a.	n.a.	n.a.	n.a.	n.a.	n.a.
5	Propionate RI	11,642	0,008	0,044	4,23	5,54	0,3254
n.a.	1,3-PDO	n.a.	n.a.	n.a.	n.a.	n.a.	n.a.
n.a.	2-3 BDO	n.a.	n.a.	n.a.	n.a.	n.a.	n.a.
6	Ethanol	13,353	0,028	0,093	15,30	11,60	2,9528
n.a.	Isobutyrate RI	n.a.	n.a.	n.a.	n.a.	n.a.	n.a.
n.a.	Butyrate RI	n.a.	n.a.	n.a.	n.a.	n.a.	n.a.
**Total:**			**0,184**	**0,803**	**100,00**	**100,00**	

Chromeleon (c) Dionex
Version 7.2.10.25868

Peak Analysis

Injection Details

Injection Name:	**MUCHMO2 t96 r2**	*Run Time (min):*	**20,00**
Vial Number:	**3:41**	*Injection Volume:*	**10,00**
Injection Type:	**Unknown**	*Channel:*	**RI_CH_1**
Calibration Level:		*Wavelength:*	**n.a.**
Instrument Method:	**Default method LC2030C 45 gr 20 min**	*Bandwidth:*	**n.a.**
Processing Method:	**Processing Method LC2030 45 gr**	*Dilution Factor:*	**1,0000**
Injection Date/Time:	**17/Jan/23 07:12**	*Sample Weight:*	**1,0000**

Chromatogram

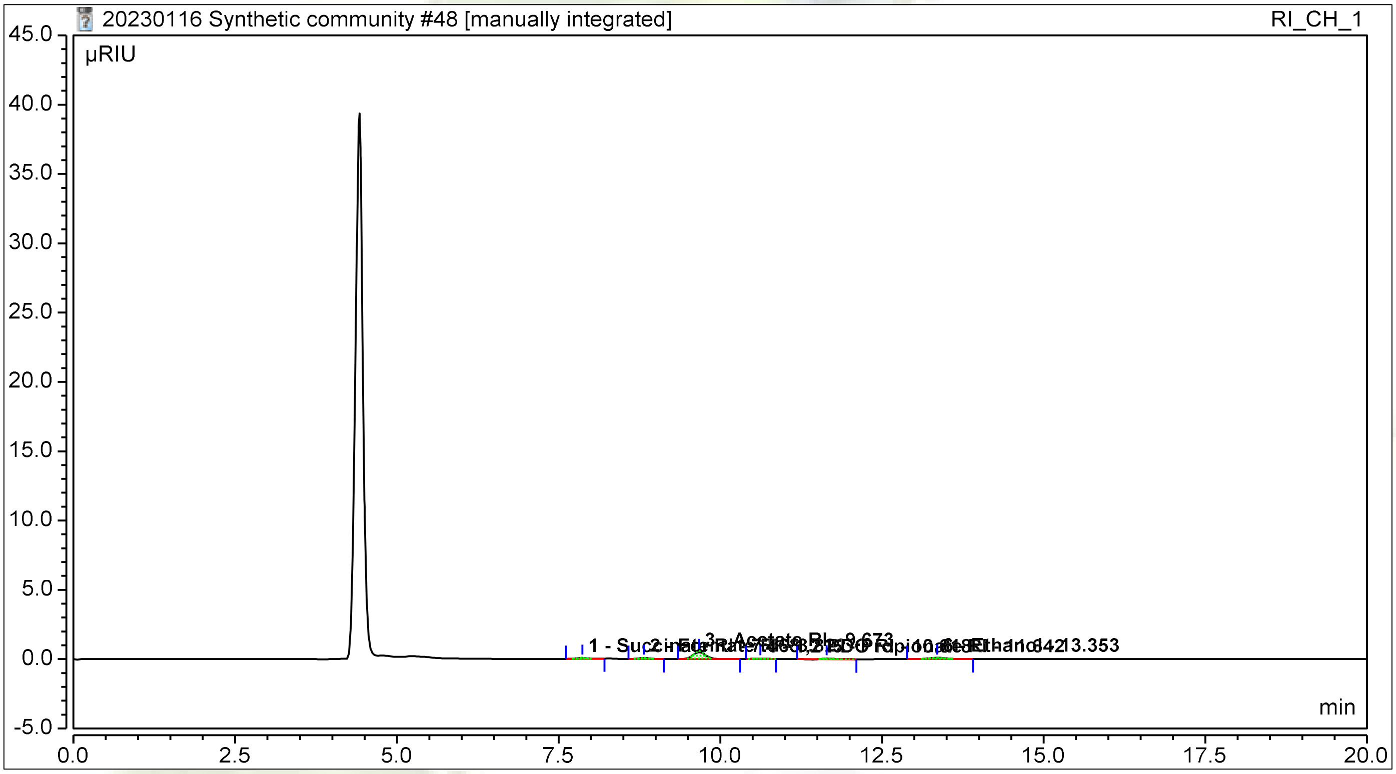

Peak Results

No.	Peak Name	Retention Time min	Width (50%) min	Type	Resolution (EP)	Asymmetry (EP)	Plates (EP)
n.a.	GlcNAc	n.a.	n.a.	n.a.	n.a.	n.a.	n.a.
n.a.	Citrate	n.a.	n.a.	n.a.	n.a.	n.a.	n.a.
n.a.	Glucose	n.a.	n.a.	n.a.	n.a.	n.a.	n.a.
n.a.	Galactose	n.a.	n.a.	n.a.	n.a.	n.a.	n.a.
n.a.	Fucose	n.a.	n.a.	n.a.	n.a.	n.a.	n.a.
1	Succinate RI	7,868	0,177	BMB*	3,05	0,91	10970
n.a.	Lactate RI	n.a.	n.a.	n.a.	n.a.	n.a.	n.a.
n.a.	glycerol	n.a.	n.a.	n.a.	n.a.	n.a.	n.a.
2	Formate RI	8,822	0,192	BMB*	2,44	1,06	11674
3	Acetate RI	9,673	0,219	BMB	2,46	1,05	10822
4	1,2 PDO RI	10,618	0,234	BMB*	2,64	1,06	11430
n.a.	1,3-PDO	n.a.	n.a.	n.a.	n.a.	n.a.	n.a.
5	Propionate RI	11,642	0,224	BMB*	3,95	2,40	14955
n.a.	1,3-PDO	n.a.	n.a.	n.a.	n.a.	n.a.	n.a.
n.a.	2-3 BDO	n.a.	n.a.	n.a.	n.a.	n.a.	n.a.
6	Ethanol	13,353	0,287	BMB*	n.a.	1,03	12012
n.a.	Isobutyrate RI	n.a.	n.a.	n.a.	n.a.	n.a.	n.a.
n.a.	Butyrate RI	n.a.	n.a.	n.a.	n.a.	n.a.	n.a.

Chromatogram and SST Results

Injection Details

Injection Name:	**MUCHMO2 t96 r2**	*Run Time (min):*	**20,00**
Vial Number:	**3:41**	*Injection Volume:*	**10,00**
Injection Type:	**Unknown**	*Channel:*	**RI_CH_1**
Calibration Level:		*Wavelength:*	**n.a.**
Instrument Method:	**Default method LC2030C 45 gr 20 min**	*Bandwidth:*	**n.a.**
Processing Method:	**Processing Method LC2030 45 gr**	*Dilution Factor:*	**1,0000**
Injection Date/Time:	**17/Jan/23 07:12**	*Sample Weight:*	**1,0000**

Chromatogram

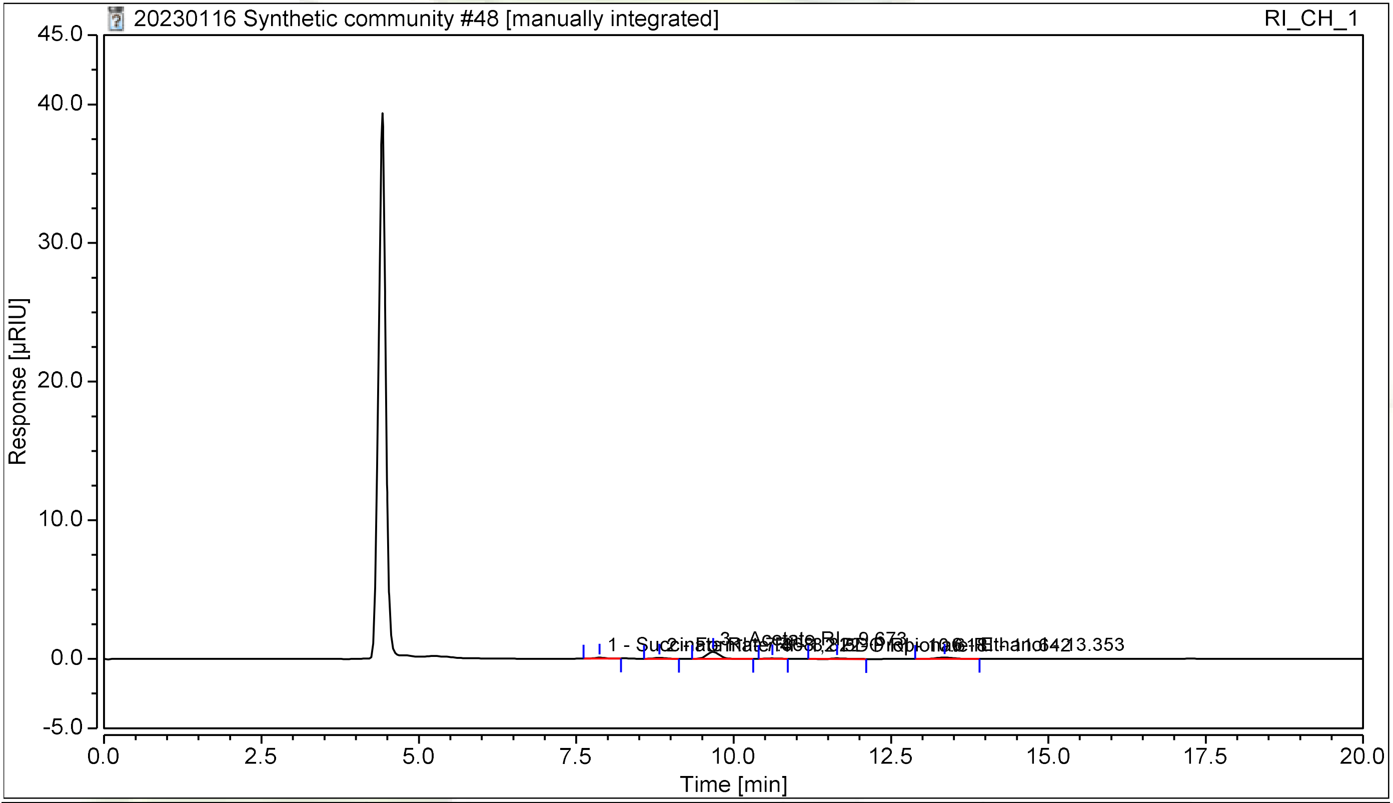

SST Results

No.	Name	Inj.Condition	Peak	Test Result	Injection
**Number of executed test cases:**	**n.a.**		**Total Result:**	**Passed**	

Chromatogram and Results

Injection Details

Injection Name:	**MUCHMO2 t96 r3**	*Run Time (min):*	**20,00**
Vial Number:	**3:42**	*Injection Volume:*	**10,00**
Injection Type:	**Unknown**	*Channel:*	**RI_CH_1**
Calibration Level:		*Wavelength:*	**n.a.**
Instrument Method:	**Default method LC2030C 45 gr 20 min**	*Bandwidth:*	**n.a.**
Processing Method:	**Processing Method LC2030 45 gr**	*Dilution Factor:*	**1,0000**
Injection Date/Time:	**17/Jan/23 07:33**	*Sample Weight:*	**1,0000**

Chromatogram

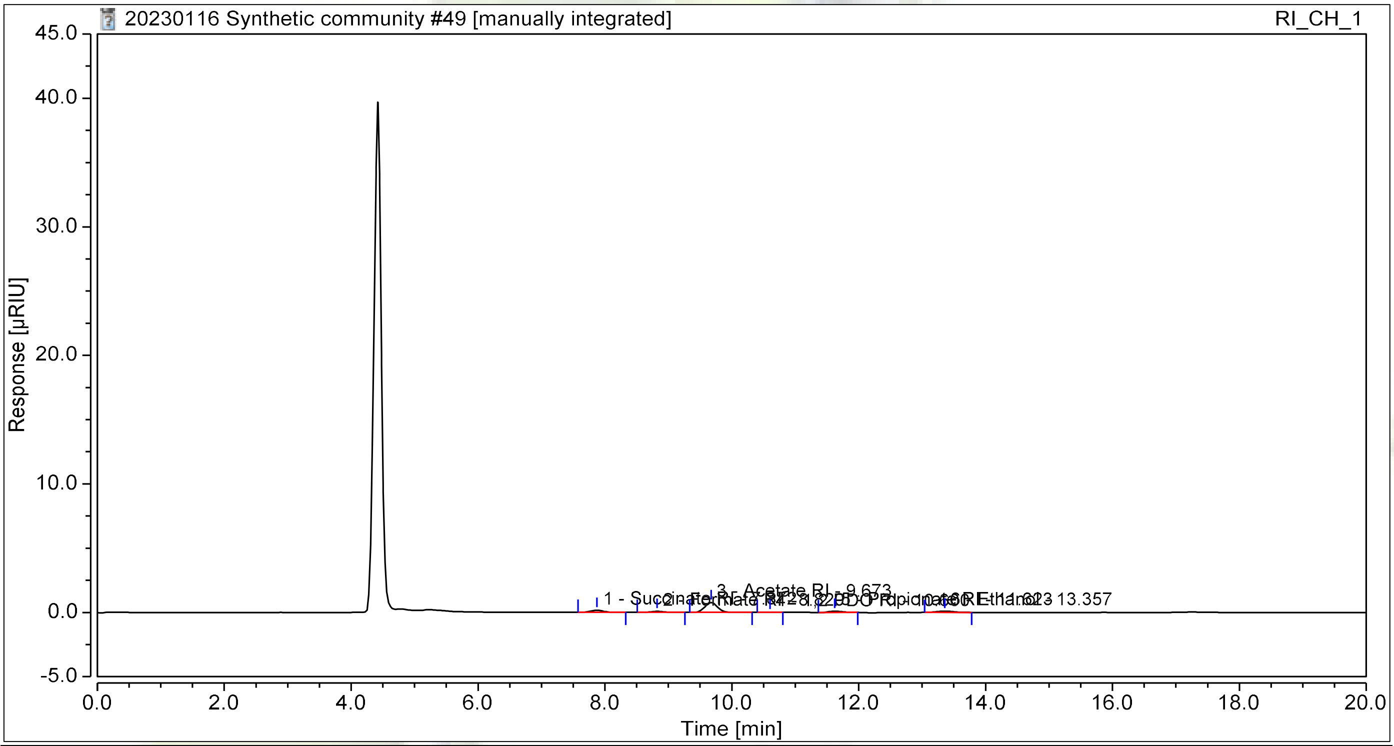

Integration Results

No.	Peak Name	Retention Time min	Area µRIU*min	Height µRIU	Relative Area %	Relative Height %	Amount
n.a.	GlcNAc	n.a.	n.a.	n.a.	n.a.	n.a.	n.a.
n.a.	Citrate	n.a.	n.a.	n.a.	n.a.	n.a.	n.a.
n.a.	Glucose	n.a.	n.a.	n.a.	n.a.	n.a.	n.a.
n.a.	Galactose	n.a.	n.a.	n.a.	n.a.	n.a.	n.a.
n.a.	Fucose	n.a.	n.a.	n.a.	n.a.	n.a.	n.a.
1	Succinate RI	7,872	0,032	0,155	11,40	12,88	0,6615
n.a.	Lactate RI	n.a.	n.a.	n.a.	n.a.	n.a.	n.a.
n.a.	glycerol	n.a.	n.a.	n.a.	n.a.	n.a.	n.a.
2	Formate RI	8,820	0,015	0,074	5,27	6,14	1,5167
3	Acetate RI	9,673	0,177	0,757	62,88	62,97	10,9622
4	1,2 PDO RI	10,600	0,003	0,012	0,94	1,02	0,0801
n.a.	1,3-PDO	n.a.	n.a.	n.a.	n.a.	n.a.	n.a.
5	Propionate RI	11,623	0,026	0,106	9,14	8,84	1,0746
n.a.	1,3-PDO	n.a.	n.a.	n.a.	n.a.	n.a.	n.a.
n.a.	2-3 BDO	n.a.	n.a.	n.a.	n.a.	n.a.	n.a.
6	Ethanol	13,357	0,029	0,098	10,38	8,14	3,0610
n.a.	Isobutyrate RI	n.a.	n.a.	n.a.	n.a.	n.a.	n.a.
n.a.	Butyrate RI	n.a.	n.a.	n.a.	n.a.	n.a.	n.a.
**Total:**			**0,281**	**1,202**	**100,00**	**100,00**	

Chromeleon (c) Dionex
Version 7.2.10.25868

Peak Analysis

Injection Details

Injection Name:	**MUCHMO2 t96 r3**	*Run Time (min):*	**20,00**
Vial Number:	**3:42**	*Injection Volume:*	**10,00**
Injection Type:	**Unknown**	*Channel:*	**RI_CH_1**
Calibration Level:		*Wavelength:*	**n.a.**
Instrument Method:	**Default method LC2030C 45 gr 20 min**	*Bandwidth:*	**n.a.**
Processing Method:	**Processing Method LC2030 45 gr**	*Dilution Factor:*	**1,0000**
Injection Date/Time:	**17/Jan/23 07:33**	*Sample Weight:*	**1,0000**

Chromatogram

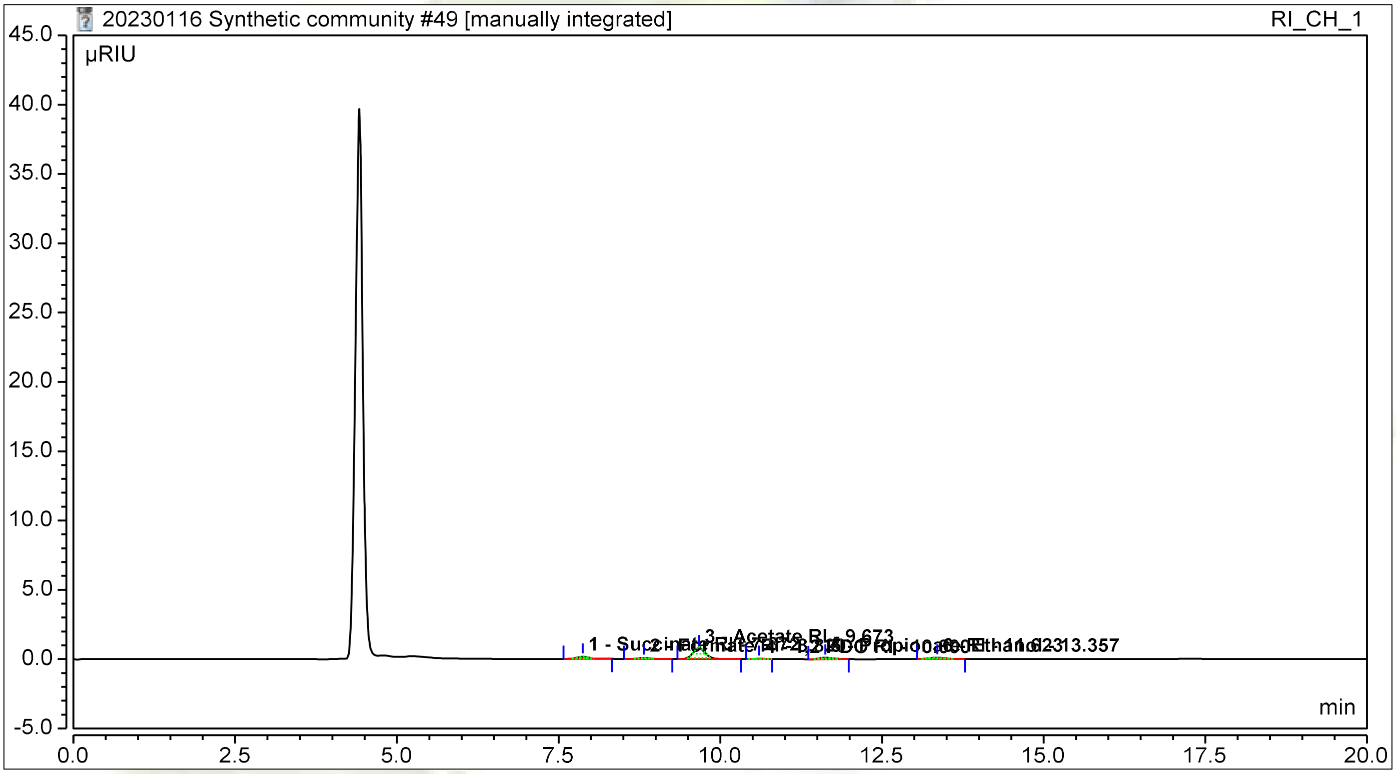

Peak Results

No.	Peak Name	Retention Time min	Width (50%) min	Type	Resolution (EP)	Asymmetry (EP)	Plates (EP)
n.a.	GlcNAc	n.a.	n.a.	n.a.	n.a.	n.a.	n.a.
n.a.	Citrate	n.a.	n.a.	n.a.	n.a.	n.a.	n.a.
n.a.	Glucose	n.a.	n.a.	n.a.	n.a.	n.a.	n.a.
n.a.	Galactose	n.a.	n.a.	n.a.	n.a.	n.a.	n.a.
n.a.	Fucose	n.a.	n.a.	n.a.	n.a.	n.a.	n.a.
1	Succinate RI	7,872	0,197	BMB*	2,87	1,02	8822
n.a.	Lactate RI	n.a.	n.a.	n.a.	n.a.	n.a.	n.a.
n.a.	glycerol	n.a.	n.a.	n.a.	n.a.	n.a.	n.a.
2	Formate RI	8,820	0,193	BMB*	2,45	1,13	11593
3	Acetate RI	9,673	0,219	BMB	2,52	1,04	10810
4	1,2 PDO RI	10,600	0,215	BMB*	2,70	1,04	13424
n.a.	1,3-PDO	n.a.	n.a.	n.a.	n.a.	n.a.	n.a.
5	Propionate RI	11,623	0,231	BMB*	3,97	1,24	13972
n.a.	1,3-PDO	n.a.	n.a.	n.a.	n.a.	n.a.	n.a.
n.a.	2-3 BDO	n.a.	n.a.	n.a.	n.a.	n.a.	n.a.
6	Ethanol	13,357	0,283	BMB*	n.a.	1,03	12309
n.a.	Isobutyrate RI	n.a.	n.a.	n.a.	n.a.	n.a.	n.a.
n.a.	Butyrate RI	n.a.	n.a.	n.a.	n.a.	n.a.	n.a.

Chromatogram and SST Results

Injection Details

Injection Name:	**MUCHMO2 t96 r3**	*Run Time (min):*	**20,00**
Vial Number:	**3:42**	*Injection Volume:*	**10,00**
Injection Type:	**Unknown**	*Channel:*	**RI_CH_1**
Calibration Level:		*Wavelength:*	**n.a.**
Instrument Method:	**Default method LC2030C 45 gr 20 min**	*Bandwidth:*	**n.a.**
Processing Method:	**Processing Method LC2030 45 gr**	*Dilution Factor:*	**1,0000**
Injection Date/Time:	**17/Jan/23 07:33**	*Sample Weight:*	**1,0000**

Chromatogram

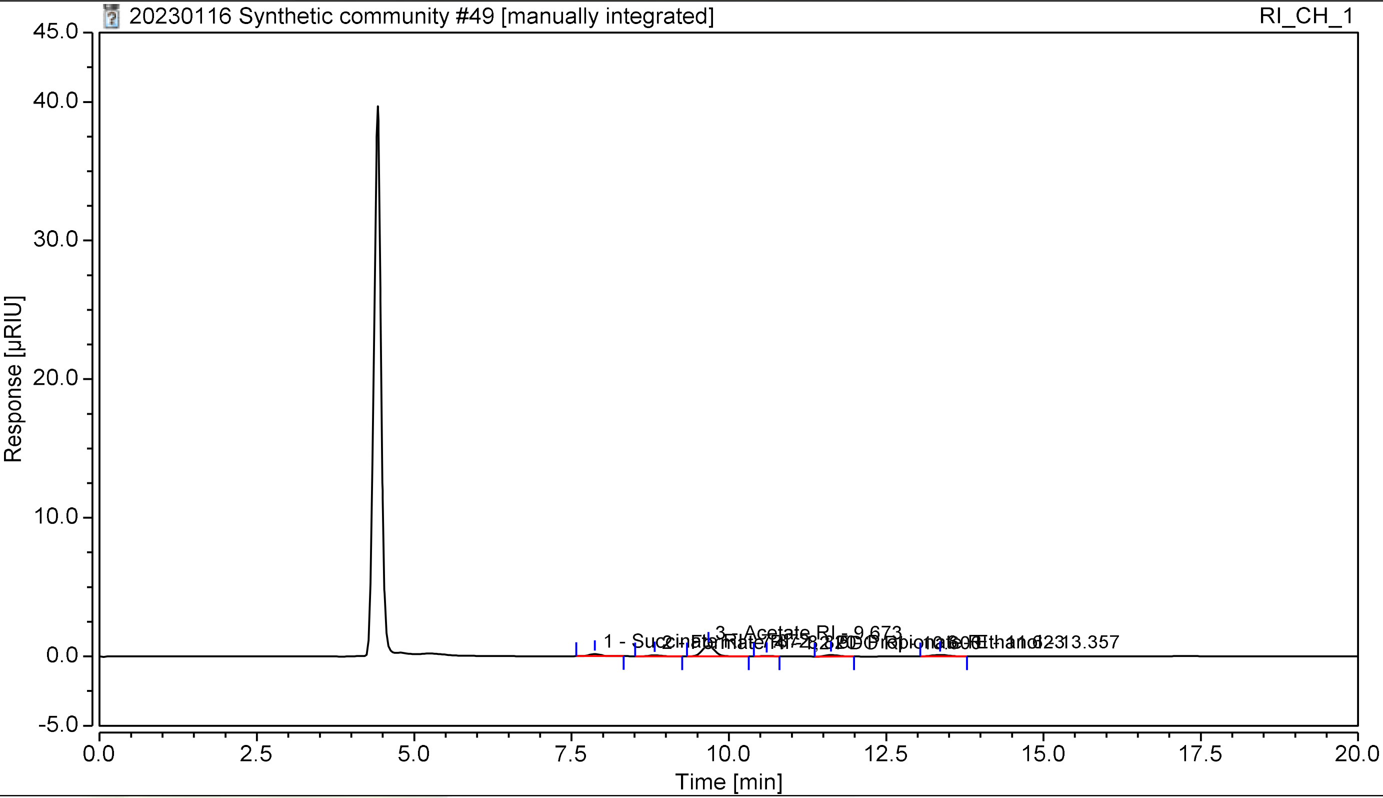

SST Results

No.	Name	Inj.Condition	Peak	Test Result	Injection

Number of executed test cases: **n.a.** **Total Result:** **Passed**

Chromatogram and Results

Injection Details

Injection Name:	**MUCHMO2 t120 r1**	*Run Time (min):*	**20,00**
Vial Number:	**3:43**	*Injection Volume:*	**10,00**
Injection Type:	**Unknown**	*Channel:*	**RI_CH_1**
Calibration Level:		*Wavelength:*	**n.a.**
Instrument Method:	**Default method LC2030C 45 gr 20 min**	*Bandwidth:*	**n.a.**
Processing Method:	**Processing Method LC2030 45 gr**	*Dilution Factor:*	**1,0000**
Injection Date/Time:	**17/Jan/23 07:53**	*Sample Weight:*	**1,0000**

Chromatogram

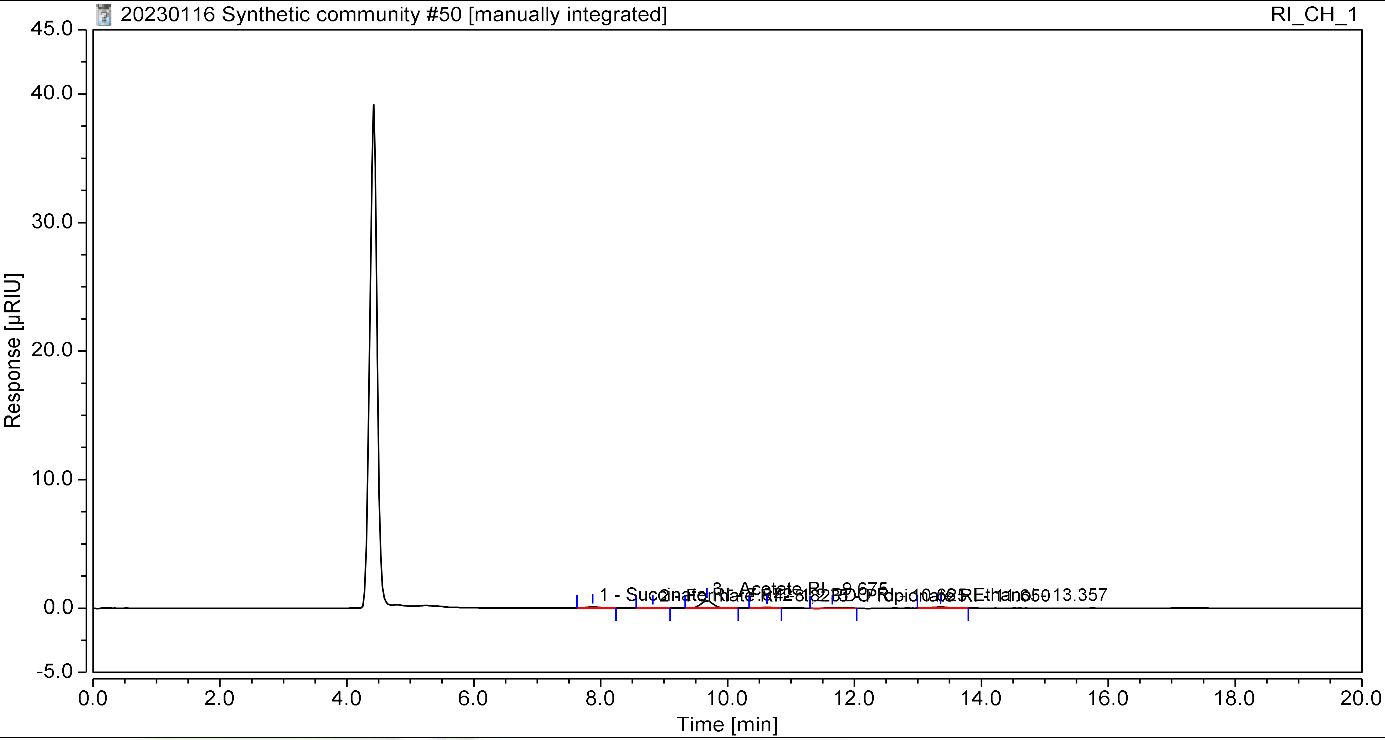

Integration Results

No.	Peak Name	Retention Time min	Area µRIU*min	Height µRIU	Relative Area %	Relative Height %	Amount
n.a.	GlcNAc	n.a.	n.a.	n.a.	n.a.	n.a.	n.a.
n.a.	Citrate	n.a.	n.a.	n.a.	n.a.	n.a.	n.a.
n.a.	Glucose	n.a.	n.a.	n.a.	n.a.	n.a.	n.a.
n.a.	Galactose	n.a.	n.a.	n.a.	n.a.	n.a.	n.a.
n.a.	Fucose	n.a.	n.a.	n.a.	n.a.	n.a.	n.a.
1	Succinate RI	7,872	0,018	0,094	8,89	10,65	0,3730
n.a.	Lactate RI	n.a.	n.a.	n.a.	n.a.	n.a.	n.a.
n.a.	glycerol	n.a.	n.a.	n.a.	n.a.	n.a.	n.a.
2	Formate RI	8,823	0,009	0,044	4,40	5,00	0,9142
3	Acetate RI	9,675	0,129	0,555	63,45	62,79	7,9978
4	1,2 PDO RI	10,625	0,013	0,054	6,41	6,07	0,3963
n.a.	1,3-PDO	n.a.	n.a.	n.a.	n.a.	n.a.	n.a.
5	Propionate RI	11,650	0,008	0,052	4,17	5,83	0,3545
n.a.	1,3-PDO	n.a.	n.a.	n.a.	n.a.	n.a.	n.a.
n.a.	2-3 BDO	n.a.	n.a.	n.a.	n.a.	n.a.	n.a.
6	Ethanol	13,357	0,026	0,085	12,69	9,66	2,7049
n.a.	Isobutyrate RI	n.a.	n.a.	n.a.	n.a.	n.a.	n.a.
n.a.	Butyrate RI	n.a.	n.a.	n.a.	n.a.	n.a.	n.a.
**Total:**			**0,203**	**0,884**	**100,00**	**100,00**	

Peak Analysis

Injection Details

Injection Name:	**MUCHMO2 t120 r1**	*Run Time (min):*	**20,00**
Vial Number:	**3:43**	*Injection Volume:*	**10,00**
Injection Type:	**Unknown**	*Channel:*	**RI_CH_1**
Calibration Level:		*Wavelength:*	**n.a.**
Instrument Method:	**Default method LC2030C 45 gr 20 min**	*Bandwidth:*	**n.a.**
Processing Method:	**Processing Method LC2030 45 gr**	*Dilution Factor:*	**1,0000**
Injection Date/Time:	**17/Jan/23 07:53**	*Sample Weight:*	**1,0000**

Chromatogram

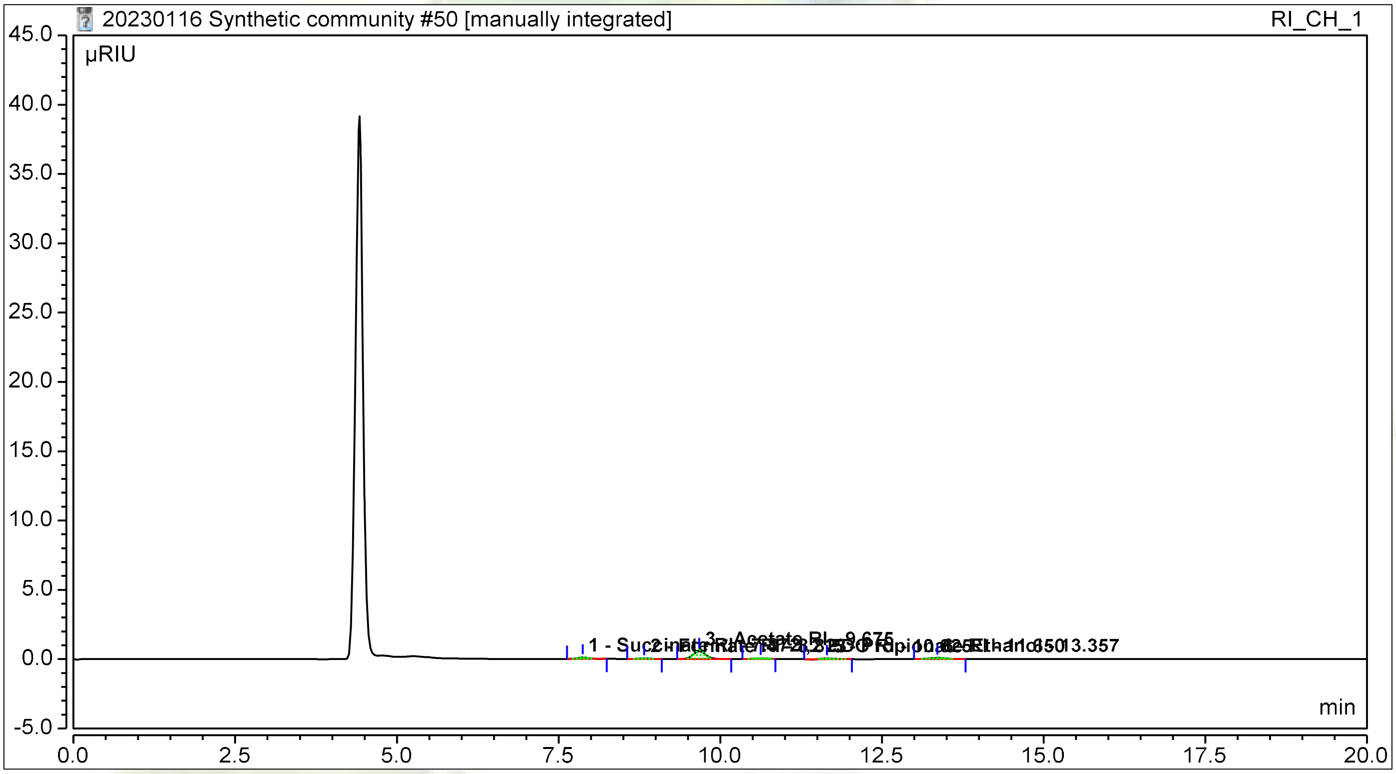

Peak Results

No.	Peak Name	Retention Time min	Width (50%) min	Type	Resolution (EP)	Asymmetry (EP)	Plates (EP)
n.a.	GlcNAc	n.a.	n.a.	n.a.	n.a.	n.a.	n.a.
n.a.	Citrate	n.a.	n.a.	n.a.	n.a.	n.a.	n.a.
n.a.	Glucose	n.a.	n.a.	n.a.	n.a.	n.a.	n.a.
n.a.	Galactose	n.a.	n.a.	n.a.	n.a.	n.a.	n.a.
n.a.	Fucose	n.a.	n.a.	n.a.	n.a.	n.a.	n.a.
1	Succinate RI	7,872	0,192	BMB*	2,92	0,99	9357
n.a.	Lactate RI	n.a.	n.a.	n.a.	n.a.	n.a.	n.a.
n.a.	glycerol	n.a.	n.a.	n.a.	n.a.	n.a.	n.a.
2	Formate RI	8,823	0,193	BMB*	2,44	1,09	11545
3	Acetate RI	9,675	0,218	BMB	2,46	1,05	10867
4	1,2 PDO RI	10,625	0,236	BMB*	2,72	0,94	11188
n.a.	1,3-PDO	n.a.	n.a.	n.a.	n.a.	n.a.	n.a.
5	Propionate RI	11,650	0,209	BMB*	4,07	1,82	17243
n.a.	1,3-PDO	n.a.	n.a.	n.a.	n.a.	n.a.	n.a.
n.a.	2-3 BDO	n.a.	n.a.	n.a.	n.a.	n.a.	n.a.
6	Ethanol	13,357	0,287	BMB*	n.a.	1,03	12037
n.a.	Isobutyrate RI	n.a.	n.a.	n.a.	n.a.	n.a.	n.a.
n.a.	Butyrate RI	n.a.	n.a.	n.a.	n.a.	n.a.	n.a.

Chromatogram and SST Results

Injection Details

Injection Name:	**MUCHMO2 t120 r1**	*Run Time (min):*	**20,00**
Vial Number:	**3:43**	*Injection Volume:*	**10,00**
Injection Type:	**Unknown**	*Channel:*	**RI_CH_1**
Calibration Level:		*Wavelength:*	**n.a.**
Instrument Method:	**Default method LC2030C 45 gr 20 min**	*Bandwidth:*	**n.a.**
Processing Method:	**Processing Method LC2030 45 gr**	*Dilution Factor:*	**1,0000**
Injection Date/Time:	**17/Jan/23 07:53**	*Sample Weight:*	**1,0000**

Chromatogram

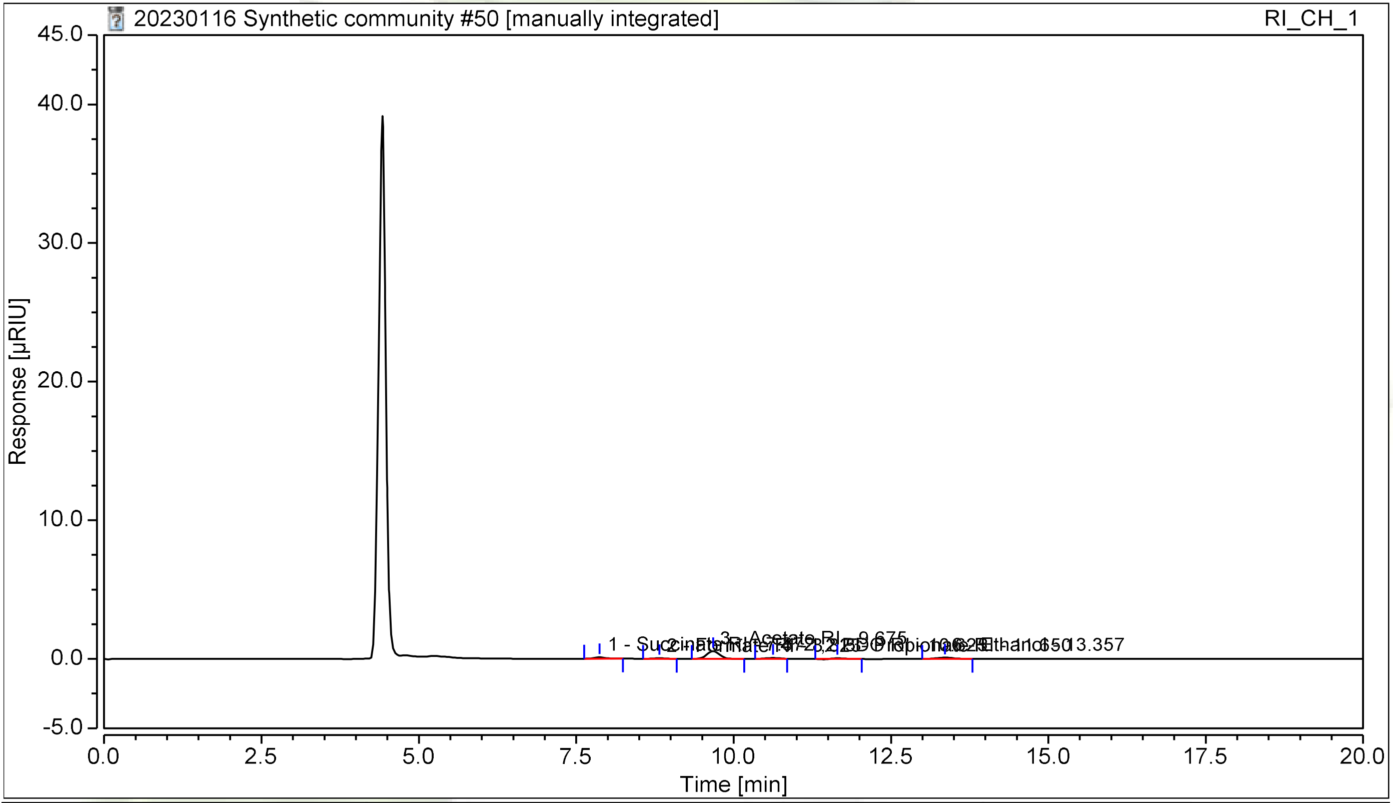

SST Results

No.	Name	Inj.Condition	Peak	Test Result	Injection
**Number of executed test cases:**	**n.a.**		**Total Result:**	**Passed**	

Chromatogram and Results

Injection Details

Injection Name:	**MUCHMO2 t120 r2**	*Run Time (min):*	**20,00**
Vial Number:	**3:44**	*Injection Volume:*	**10,00**
Injection Type:	**Unknown**	*Channel:*	**RI_CH_1**
Calibration Level:		*Wavelength:*	**n.a.**
Instrument Method:	**Default method LC2030C 45 gr 20 min**	*Bandwidth:*	**n.a.**
Processing Method:	**Processing Method LC2030 45 gr**	*Dilution Factor:*	**1,0000**
Injection Date/Time:	**17/Jan/23 08:14**	*Sample Weight:*	**1,0000**

Chromatogram

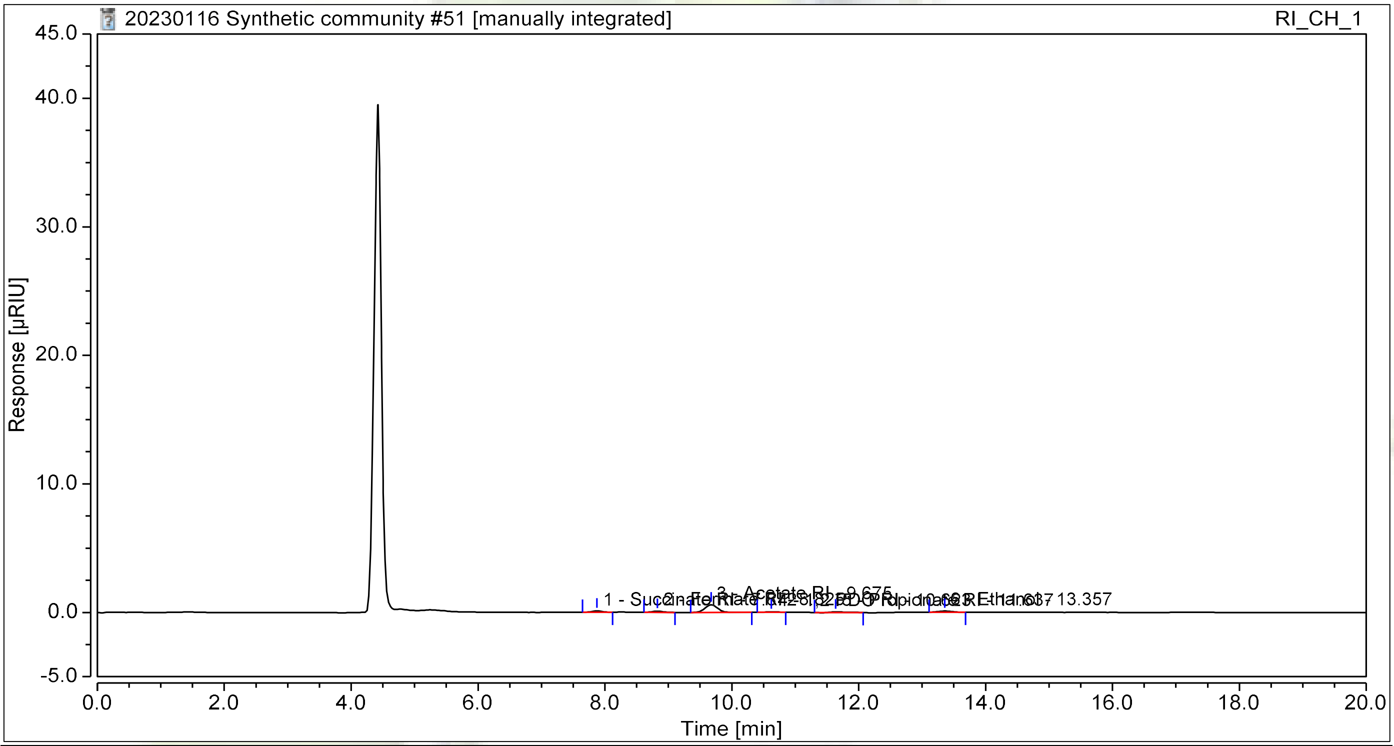

Integration Results

No.	Peak Name	Retention Time<br>min	Area<br>µRIU*min	Height<br>µRIU	Relative Area<br>%	Relative Height<br>%	Amount
n.a.	GlcNAc	n.a.	n.a.	n.a.	n.a.	n.a.	n.a.
n.a.	Citrate	n.a.	n.a.	n.a.	n.a.	n.a.	n.a.
n.a.	Glucose	n.a.	n.a.	n.a.	n.a.	n.a.	n.a.
n.a.	Galactose	n.a.	n.a.	n.a.	n.a.	n.a.	n.a.
n.a.	Fucose	n.a.	n.a.	n.a.	n.a.	n.a.	n.a.
1	Succinate RI	7,872	0,020	0,097	9,02	10,31	0,4054
n.a.	Lactate RI	n.a.	n.a.	n.a.	n.a.	n.a.	n.a.
n.a.	glycerol	n.a.	n.a.	n.a.	n.a.	n.a.	n.a.
2	Formate RI	8,825	0,017	0,085	7,95	9,01	1,7708
3	Acetate RI	9,675	0,136	0,581	62,25	61,53	8,4046
4	1,2 PDO RI	10,623	0,009	0,038	4,07	4,02	0,2695
n.a.	1,3-PDO	n.a.	n.a.	n.a.	n.a.	n.a.	n.a.
5	Propionate RI	11,637	0,010	0,050	4,82	5,32	0,4390
n.a.	1,3-PDO	n.a.	n.a.	n.a.	n.a.	n.a.	n.a.
n.a.	2-3 BDO	n.a.	n.a.	n.a.	n.a.	n.a.	n.a.
6	Ethanol	13,357	0,026	0,093	11,89	9,81	2,7150
n.a.	Isobutyrate RI	n.a.	n.a.	n.a.	n.a.	n.a.	n.a.
n.a.	Butyrate RI	n.a.	n.a.	n.a.	n.a.	n.a.	n.a.
**Total:**			**0,218**	**0,944**	**100,00**	**100,00**	

Chromeleon (c) Dionex
Version 7.2.10.25868

Peak Analysis

Injection Details

Injection Name:	**MUCHMO2 t120 r2**	*Run Time (min):*	**20,00**
Vial Number:	**3:44**	*Injection Volume:*	**10,00**
Injection Type:	**Unknown**	*Channel:*	**RI_CH_1**
Calibration Level:		*Wavelength:*	**n.a.**
Instrument Method:	**Default method LC2030C 45 gr 20 min**	*Bandwidth:*	**n.a.**
Processing Method:	**Processing Method LC2030 45 gr**	*Dilution Factor:*	**1,0000**
Injection Date/Time:	**17/Jan/23 08:14**	*Sample Weight:*	**1,0000**

Chromatogram

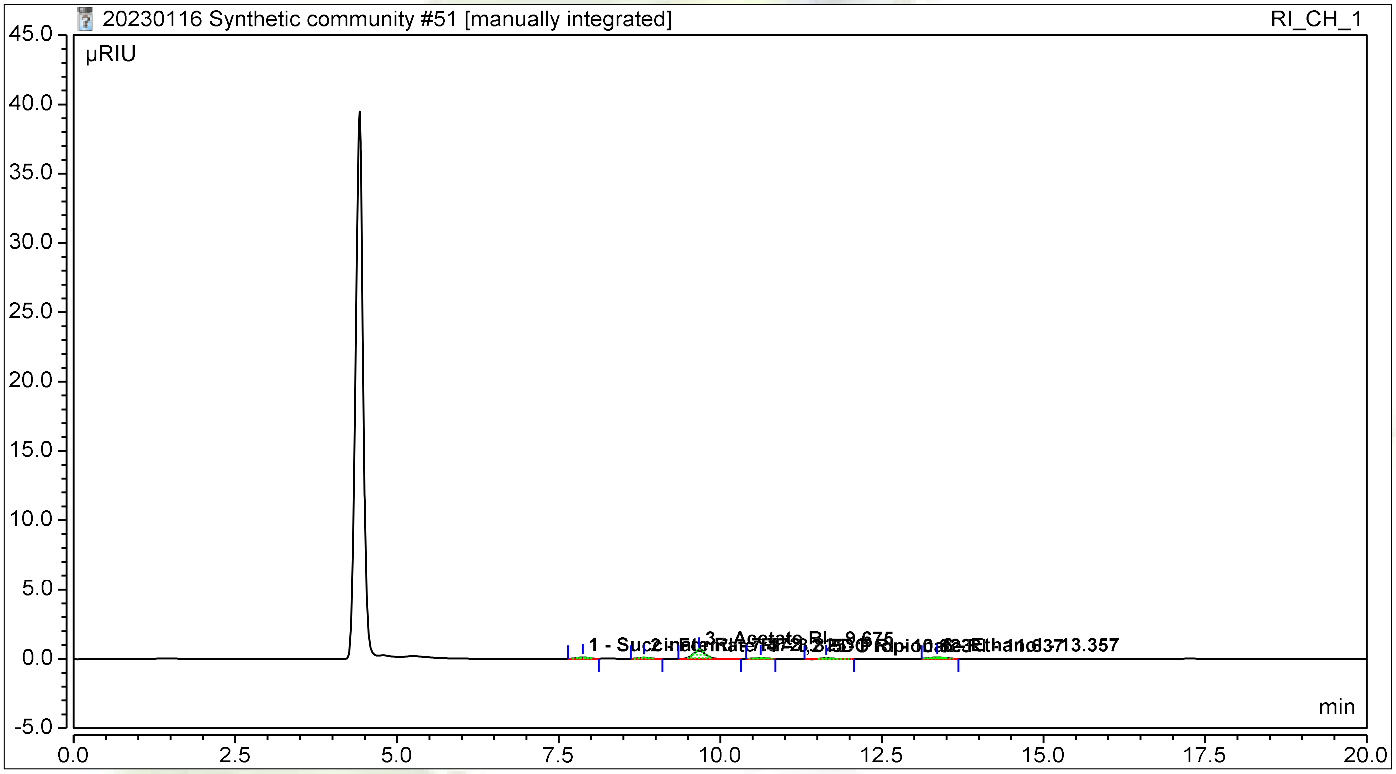

Peak Results

No.	Peak Name	Retention Time min	Width (50%) min	Type	Resolution (EP)	Asymmetry (EP)	Plates (EP)
n.a.	GlcNAc	n.a.	n.a.	n.a.	n.a.	n.a.	n.a.
n.a.	Citrate	n.a.	n.a.	n.a.	n.a.	n.a.	n.a.
n.a.	Glucose	n.a.	n.a.	n.a.	n.a.	n.a.	n.a.
n.a.	Galactose	n.a.	n.a.	n.a.	n.a.	n.a.	n.a.
n.a.	Fucose	n.a.	n.a.	n.a.	n.a.	n.a.	n.a.
1	Succinate RI	7,872	0,195	BMB*	2,89	1,02	9041
n.a.	Lactate RI	n.a.	n.a.	n.a.	n.a.	n.a.	n.a.
n.a.	glycerol	n.a.	n.a.	n.a.	n.a.	n.a.	n.a.
2	Formate RI	8,825	0,195	BMB*	2,43	1,07	11381
3	Acetate RI	9,675	0,219	BMB	2,48	1,05	10833
4	1,2 PDO RI	10,623	0,232	BMB*	2,61	1,01	11664
n.a.	1,3-PDO	n.a.	n.a.	n.a.	n.a.	n.a.	n.a.
5	Propionate RI	11,637	0,227	BMB*	4,05	2,14	14573
n.a.	1,3-PDO	n.a.	n.a.	n.a.	n.a.	n.a.	n.a.
n.a.	2-3 BDO	n.a.	n.a.	n.a.	n.a.	n.a.	n.a.
6	Ethanol	13,357	0,274	BMB*	n.a.	1,10	13191
n.a.	Isobutyrate RI	n.a.	n.a.	n.a.	n.a.	n.a.	n.a.
n.a.	Butyrate RI	n.a.	n.a.	n.a.	n.a.	n.a.	n.a.

Chromatogram and SST Results

Injection Details

Injection Name:	**MUCHMO2 t120 r2**	*Run Time (min):*	**20,00**
Vial Number:	**3:44**	*Injection Volume:*	**10,00**
Injection Type:	**Unknown**	*Channel:*	**RI_CH_1**
Calibration Level:		*Wavelength:*	**n.a.**
Instrument Method:	**Default method LC2030C 45 gr 20 min**	*Bandwidth:*	**n.a.**
Processing Method:	**Processing Method LC2030 45 gr**	*Dilution Factor:*	**1,0000**
Injection Date/Time:	**17/Jan/23 08:14**	*Sample Weight:*	**1,0000**

Chromatogram

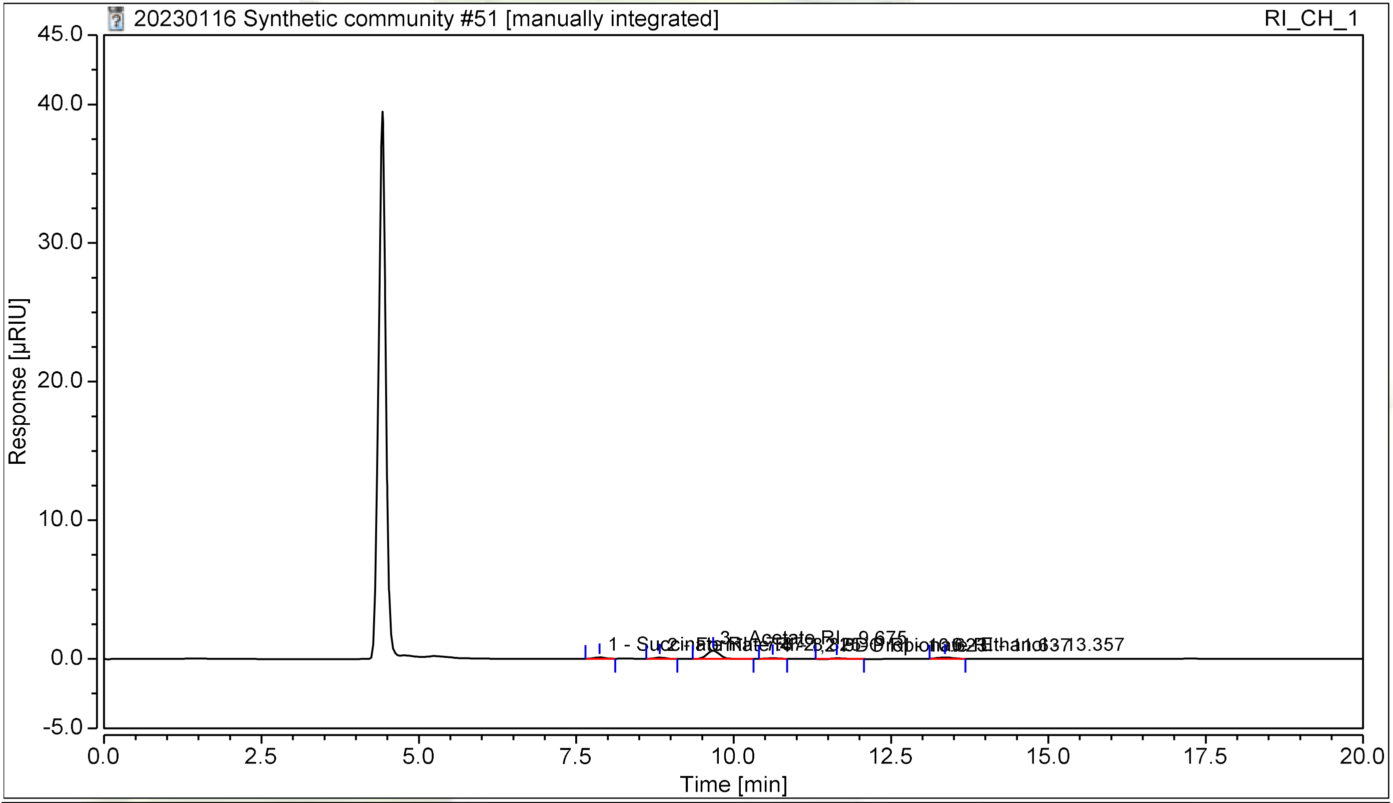

SST Results

No.	Name	Inj.Condition	Peak	Test Result	Injection
**Number of executed test cases:**	**n.a.**		**Total Result:**	**Passed**	

Chromatogram and Results

Injection Details

Injection Name:	**no injection (after disconect)**	*Run Time (min):*	**20,00**
Vial Number:	**3:1**	*Injection Volume:*	**10,00**
Injection Type:	**Blank**	*Channel:*	**RI_CH_1**
Calibration Level:		*Wavelength:*	**n.a.**
Instrument Method:	**Default method LC2030C 45 gr 20 min**	*Bandwidth:*	**n.a.**
Processing Method:	**Processing Method LC2030 45 gr**	*Dilution Factor:*	**1,0000**
Injection Date/Time:	**17/Jan/23 09:06**	*Sample Weight:*	**1,0000**

Chromatogram

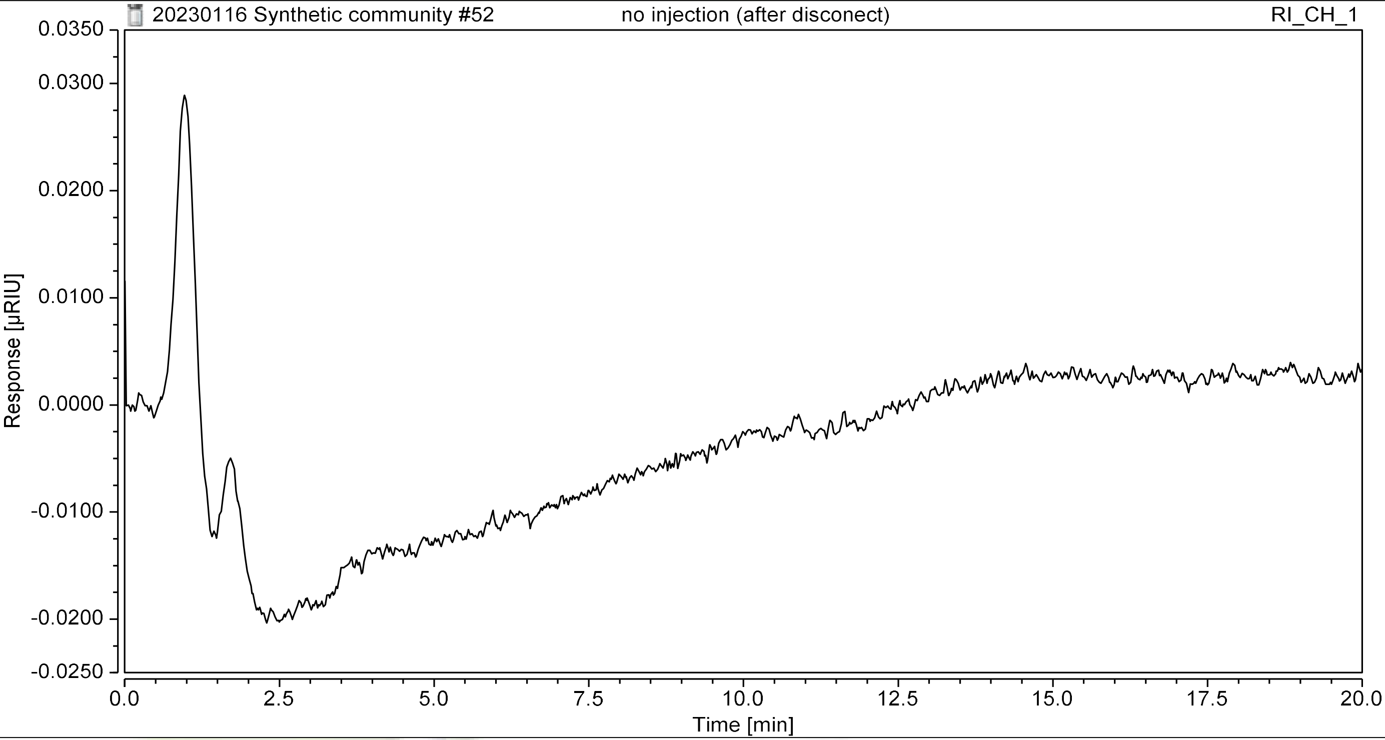

Integration Results

No.	Peak Name	Retention Time min	Area µRIU*min	Height µRIU	Relative Area %	Relative Height %	Amount
n.a.	GlcNAc	n.a.	n.a.	n.a.	n.a.	n.a.	n.a.
n.a.	Citrate	n.a.	n.a.	n.a.	n.a.	n.a.	n.a.
n.a.	Glucose	n.a.	n.a.	n.a.	n.a.	n.a.	n.a.
n.a.	Galactose	n.a.	n.a.	n.a.	n.a.	n.a.	n.a.
n.a.	Fucose	n.a.	n.a.	n.a.	n.a.	n.a.	n.a.
n.a.	Succinate RI	n.a.	n.a.	n.a.	n.a.	n.a.	n.a.
n.a.	Lactate RI	n.a.	n.a.	n.a.	n.a.	n.a.	n.a.
n.a.	glycerol	n.a.	n.a.	n.a.	n.a.	n.a.	n.a.
n.a.	Formate RI	n.a.	n.a.	n.a.	n.a.	n.a.	n.a.
n.a.	Acetate RI	n.a.	n.a.	n.a.	n.a.	n.a.	n.a.
n.a.	1,2 PDO RI	n.a.	n.a.	n.a.	n.a.	n.a.	n.a.
n.a.	1,3-PDO	n.a.	n.a.	n.a.	n.a.	n.a.	n.a.
n.a.	Propionate RI	n.a.	n.a.	n.a.	n.a.	n.a.	n.a.
n.a.	1,3-PDO	n.a.	n.a.	n.a.	n.a.	n.a.	n.a.
n.a.	2-3 BDO	n.a.	n.a.	n.a.	n.a.	n.a.	n.a.
n.a.	Ethanol	n.a.	n.a.	n.a.	n.a.	n.a.	n.a.
n.a.	Isobutyrate RI	n.a.	n.a.	n.a.	n.a.	n.a.	n.a.
n.a.	Butyrate RI	n.a.	n.a.	n.a.	n.a.	n.a.	n.a.
**Total:**			**0,000**	**0,000**	**0,00**	**0,00**	

Chromeleon (c) Dionex
Version 7.2.10.25868

Peak Analysis

Injection Details

Injection Name:	**no injection (after disconect)**	*Run Time (min):*	**20,00**
Vial Number:	**3:1**	*Injection Volume:*	**10,00**
Injection Type:	**Blank**	*Channel:*	**RI_CH_1**
Calibration Level:		*Wavelength:*	**n.a.**
Instrument Method:	**Default method LC2030C 45 gr 20 min**	*Bandwidth:*	**n.a.**
Processing Method:	**Processing Method LC2030 45 gr**	*Dilution Factor:*	**1,0000**
Injection Date/Time:	**17/Jan/23 09:06**	*Sample Weight:*	**1,0000**

Chromatogram

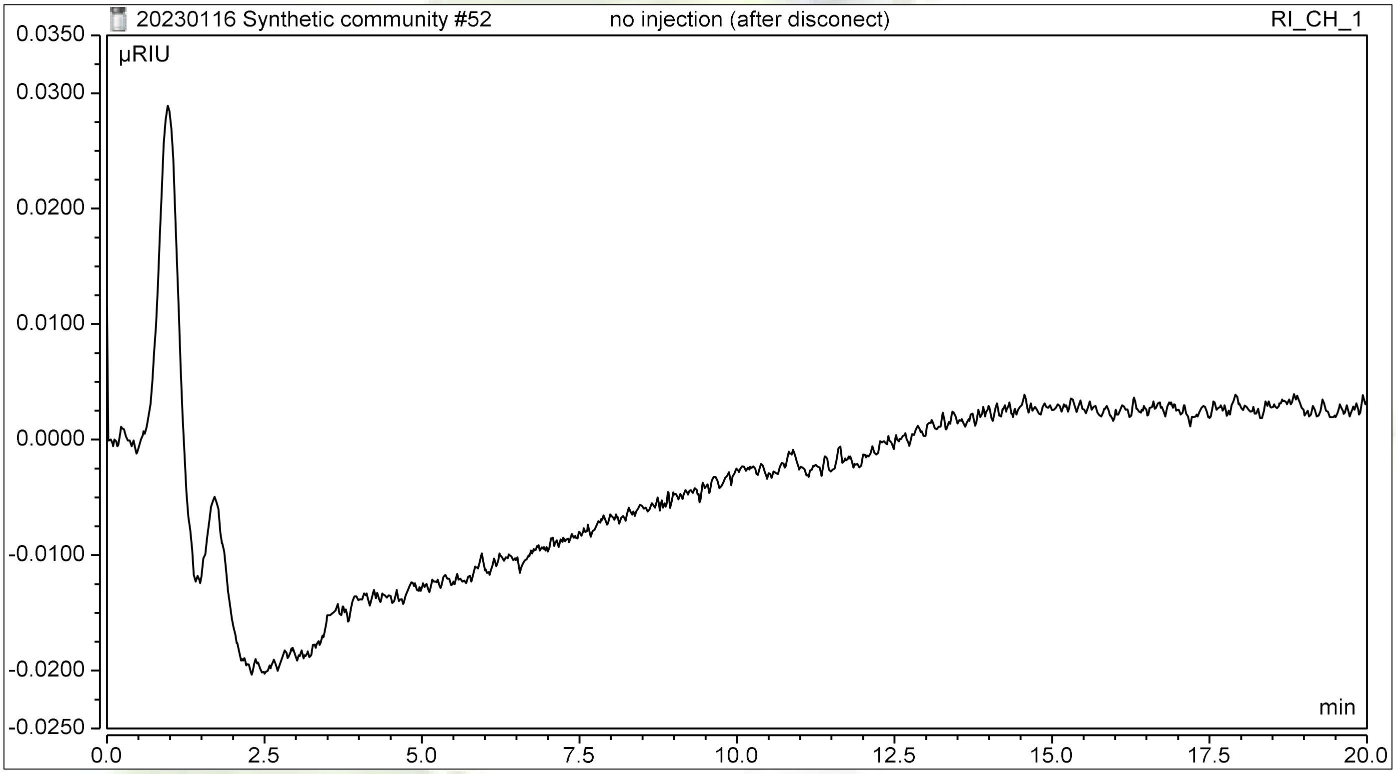

Peak Results

No.	Peak Name	Retention Time min	Width (50%) min	Type	Resolution (EP)	Asymmetry (EP)	Plates (EP)
n.a.	GlcNAc	n.a.	n.a.	n.a.	n.a.	n.a.	n.a.
n.a.	Citrate	n.a.	n.a.	n.a.	n.a.	n.a.	n.a.
n.a.	Glucose	n.a.	n.a.	n.a.	n.a.	n.a.	n.a.
n.a.	Galactose	n.a.	n.a.	n.a.	n.a.	n.a.	n.a.
n.a.	Fucose	n.a.	n.a.	n.a.	n.a.	n.a.	n.a.
n.a.	Succinate RI	n.a.	n.a.	n.a.	n.a.	n.a.	n.a.
n.a.	Lactate RI	n.a.	n.a.	n.a.	n.a.	n.a.	n.a.
n.a.	glycerol	n.a.	n.a.	n.a.	n.a.	n.a.	n.a.
n.a.	Formate RI	n.a.	n.a.	n.a.	n.a.	n.a.	n.a.
n.a.	Acetate RI	n.a.	n.a.	n.a.	n.a.	n.a.	n.a.
n.a.	1,2 PDO RI	n.a.	n.a.	n.a.	n.a.	n.a.	n.a.
n.a.	1,3-PDO	n.a.	n.a.	n.a.	n.a.	n.a.	n.a.
n.a.	Propionate RI	n.a.	n.a.	n.a.	n.a.	n.a.	n.a.
n.a.	1,3-PDO	n.a.	n.a.	n.a.	n.a.	n.a.	n.a.
n.a.	2-3 BDO	n.a.	n.a.	n.a.	n.a.	n.a.	n.a.
n.a.	Ethanol	n.a.	n.a.	n.a.	n.a.	n.a.	n.a.
n.a.	Isobutyrate RI	n.a.	n.a.	n.a.	n.a.	n.a.	n.a.
n.a.	Butyrate RI	n.a.	n.a.	n.a.	n.a.	n.a.	n.a.

Chromeleon (c) Dionex
Version 7.2.10.25868

Chromatogram and SST Results

Injection Details

Injection Name:	**no injection (after disconect)**	*Run Time (min):*	**20,00**
Vial Number:	**3:1**	*Injection Volume:*	**10,00**
Injection Type:	**Blank**	*Channel:*	**RI_CH_1**
Calibration Level:		*Wavelength:*	**n.a.**
Instrument Method:	**Default method LC2030C 45 gr 20 min**	*Bandwidth:*	**n.a.**
Processing Method:	**Processing Method LC2030 45 gr**	*Dilution Factor:*	**1,0000**
Injection Date/Time:	**17/Jan/23 09:06**	*Sample Weight:*	**1,0000**

Chromatogram

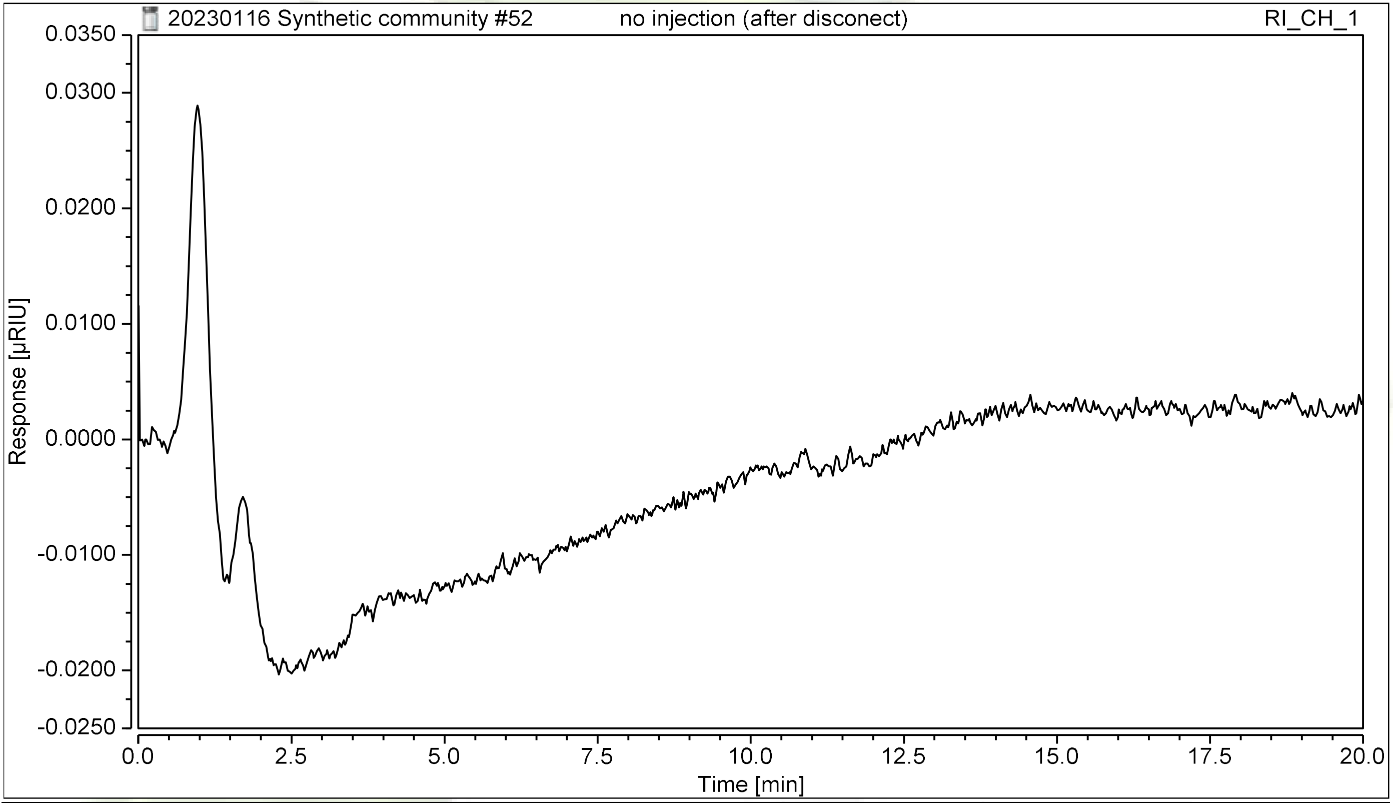

SST Results

No.	Name	Inj.Condition	Peak	Test Result	Injection

Number of executed test cases: **n.a.** **Total Result:** **Passed**

Chromatogram and Results

Injection Details

Injection Name:	**no injection**	*Run Time (min):*	**20,00**
Vial Number:	**3:1**	*Injection Volume:*	**10,00**
Injection Type:	**Blank**	*Channel:*	**RI_CH_1**
Calibration Level:		*Wavelength:*	**n.a.**
Instrument Method:	**Default method LC2030C 45 gr 20 min**	*Bandwidth:*	**n.a.**
Processing Method:	**Processing Method LC2030 45 gr**	*Dilution Factor:*	**1,0000**
Injection Date/Time:	**17/Jan/23 09:26**	*Sample Weight:*	**1,0000**

Chromatogram

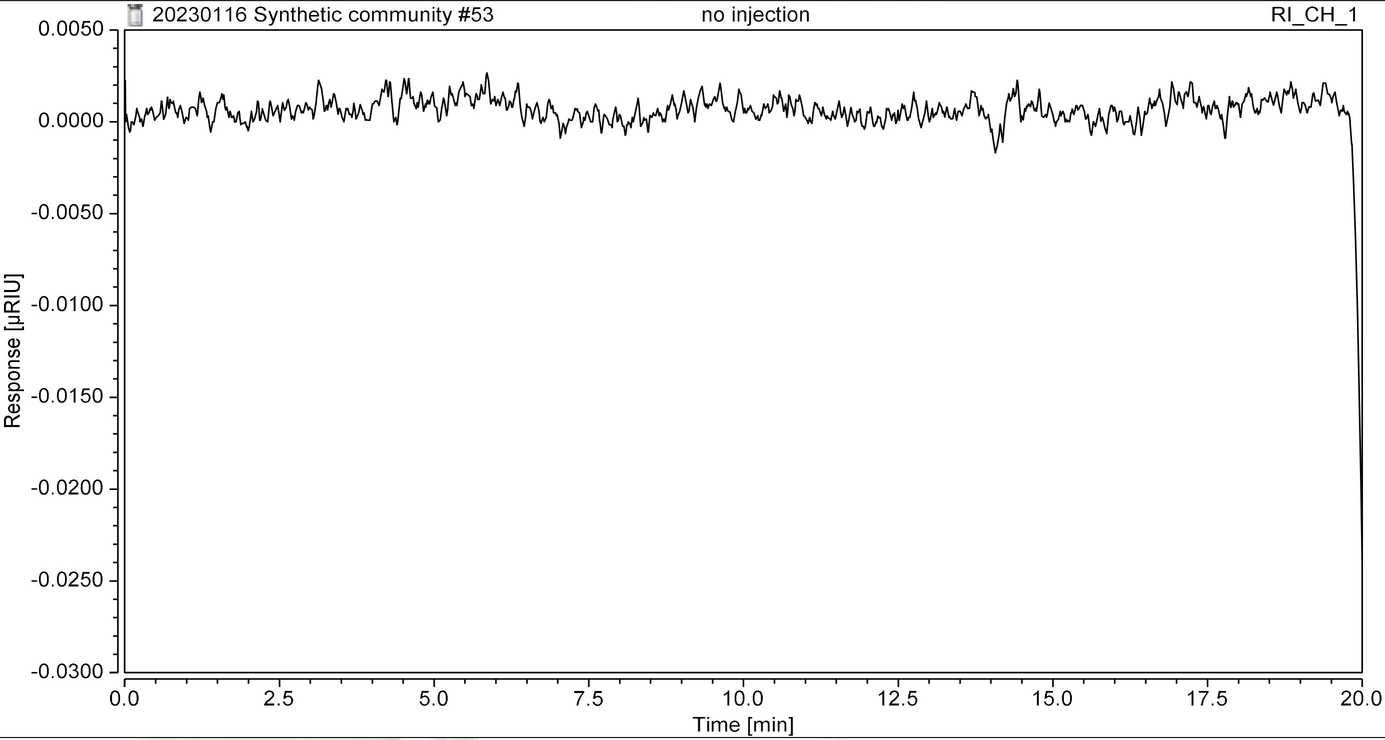

Integration Results

No.	Peak Name	Retention Time min	Area µRIU*min	Height µRIU	Relative Area %	Relative Height %	Amount
n.a.	GlcNAc	n.a.	n.a.	n.a.	n.a.	n.a.	n.a.
n.a.	Citrate	n.a.	n.a.	n.a.	n.a.	n.a.	n.a.
n.a.	Glucose	n.a.	n.a.	n.a.	n.a.	n.a.	n.a.
n.a.	Galactose	n.a.	n.a.	n.a.	n.a.	n.a.	n.a.
n.a.	Fucose	n.a.	n.a.	n.a.	n.a.	n.a.	n.a.
n.a.	Succinate RI	n.a.	n.a.	n.a.	n.a.	n.a.	n.a.
n.a.	Lactate RI	n.a.	n.a.	n.a.	n.a.	n.a.	n.a.
n.a.	glycerol	n.a.	n.a.	n.a.	n.a.	n.a.	n.a.
n.a.	Formate RI	n.a.	n.a.	n.a.	n.a.	n.a.	n.a.
n.a.	Acetate RI	n.a.	n.a.	n.a.	n.a.	n.a.	n.a.
n.a.	1,2 PDO RI	n.a.	n.a.	n.a.	n.a.	n.a.	n.a.
n.a.	1,3-PDO	n.a.	n.a.	n.a.	n.a.	n.a.	n.a.
n.a.	Propionate RI	n.a.	n.a.	n.a.	n.a.	n.a.	n.a.
n.a.	1,3-PDO	n.a.	n.a.	n.a.	n.a.	n.a.	n.a.
n.a.	2-3 BDO	n.a.	n.a.	n.a.	n.a.	n.a.	n.a.
n.a.	Ethanol	n.a.	n.a.	n.a.	n.a.	n.a.	n.a.
n.a.	Isobutyrate RI	n.a.	n.a.	n.a.	n.a.	n.a.	n.a.
n.a.	Butyrate RI	n.a.	n.a.	n.a.	n.a.	n.a.	n.a.
**Total:**			**0,000**	**0,000**	**0,00**	**0,00**	

Peak Analysis

Injection Details

Injection Name:	**no injection**	*Run Time (min):*	**20,00**
Vial Number:	**3:1**	*Injection Volume:*	**10,00**
Injection Type:	**Blank**	*Channel:*	**RI_CH_1**
Calibration Level:		*Wavelength:*	**n.a.**
Instrument Method:	**Default method LC2030C 45 gr 20 min**	*Bandwidth:*	**n.a.**
Processing Method:	**Processing Method LC2030 45 gr**	*Dilution Factor:*	**1,0000**
Injection Date/Time:	**17/Jan/23 09:26**	*Sample Weight:*	**1,0000**

Chromatogram

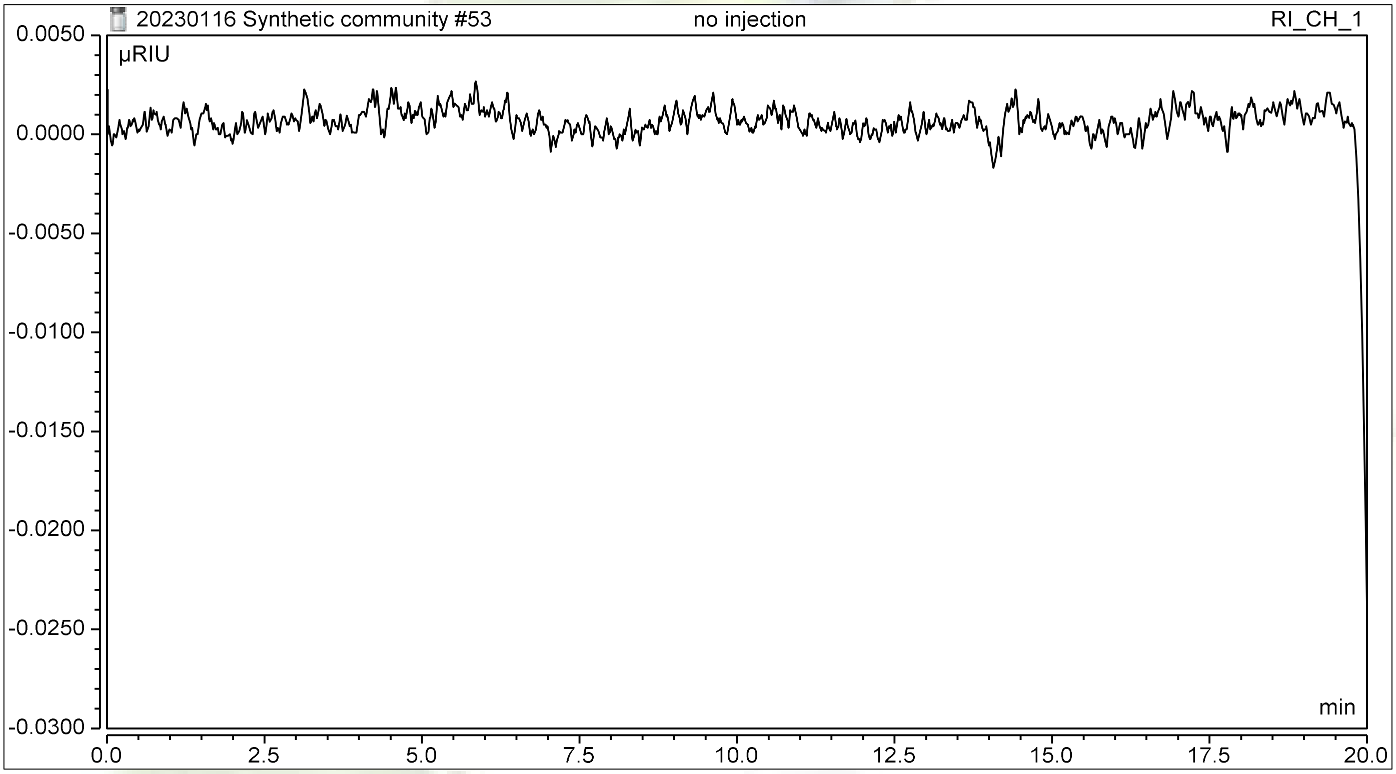

Peak Results

No.	Peak Name	Retention Time min	Width (50%) min	Type	Resolution (EP)	Asymmetry (EP)	Plates (EP)
n.a.	GlcNAc	n.a.	n.a.	n.a.	n.a.	n.a.	n.a.
n.a.	Citrate	n.a.	n.a.	n.a.	n.a.	n.a.	n.a.
n.a.	Glucose	n.a.	n.a.	n.a.	n.a.	n.a.	n.a.
n.a.	Galactose	n.a.	n.a.	n.a.	n.a.	n.a.	n.a.
n.a.	Fucose	n.a.	n.a.	n.a.	n.a.	n.a.	n.a.
n.a.	Succinate RI	n.a.	n.a.	n.a.	n.a.	n.a.	n.a.
n.a.	Lactate RI	n.a.	n.a.	n.a.	n.a.	n.a.	n.a.
n.a.	glycerol	n.a.	n.a.	n.a.	n.a.	n.a.	n.a.
n.a.	Formate RI	n.a.	n.a.	n.a.	n.a.	n.a.	n.a.
n.a.	Acetate RI	n.a.	n.a.	n.a.	n.a.	n.a.	n.a.
n.a.	1,2 PDO RI	n.a.	n.a.	n.a.	n.a.	n.a.	n.a.
n.a.	1,3-PDO	n.a.	n.a.	n.a.	n.a.	n.a.	n.a.
n.a.	Propionate RI	n.a.	n.a.	n.a.	n.a.	n.a.	n.a.
n.a.	1,3-PDO	n.a.	n.a.	n.a.	n.a.	n.a.	n.a.
n.a.	2-3 BDO	n.a.	n.a.	n.a.	n.a.	n.a.	n.a.
n.a.	Ethanol	n.a.	n.a.	n.a.	n.a.	n.a.	n.a.
n.a.	Isobutyrate RI	n.a.	n.a.	n.a.	n.a.	n.a.	n.a.
n.a.	Butyrate RI	n.a.	n.a.	n.a.	n.a.	n.a.	n.a.

Chromatogram and SST Results

Injection Details

Injection Name:	**no injection**	*Run Time (min):*	**20,00**
Vial Number:	**3:1**	*Injection Volume:*	**10,00**
Injection Type:	**Blank**	*Channel:*	**RI_CH_1**
Calibration Level:		*Wavelength:*	**n.a.**
Instrument Method:	**Default method LC2030C 45 gr 20 min**	*Bandwidth:*	**n.a.**
Processing Method:	**Processing Method LC2030 45 gr**	*Dilution Factor:*	**1,0000**
Injection Date/Time:	**17/Jan/23 09:26**	*Sample Weight:*	**1,0000**

Chromatogram

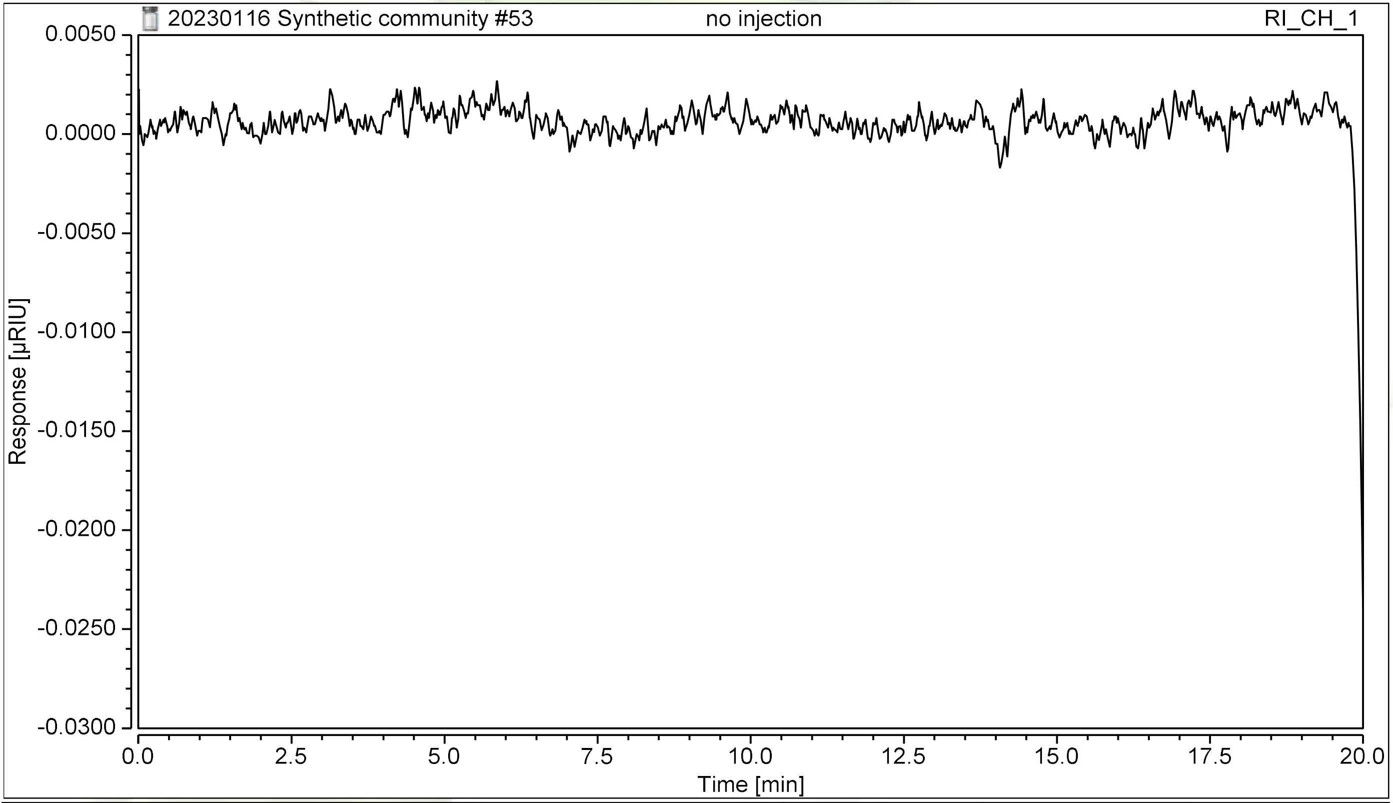

SST Results

No.	Name	Inj.Condition	Peak	Test Result	Injection

Number of executed test cases: **n.a.** **Total Result:** **Passed**

Chromatogram and Results

Injection Details

Injection Name:	**MUCHMO2 t120 r3**	*Run Time (min):*	**20,00**
Vial Number:	**3:45**	*Injection Volume:*	**10,00**
Injection Type:	**Unknown**	*Channel:*	**RI_CH_1**
Calibration Level:		*Wavelength:*	**n.a.**
Instrument Method:	**Default method LC2030C 45 gr 20 min**	*Bandwidth:*	**n.a.**
Processing Method:	**Processing Method LC2030 45 gr**	*Dilution Factor:*	**1,0000**
Injection Date/Time:	**17/Jan/23 09:47**	*Sample Weight:*	**1,0000**

Chromatogram

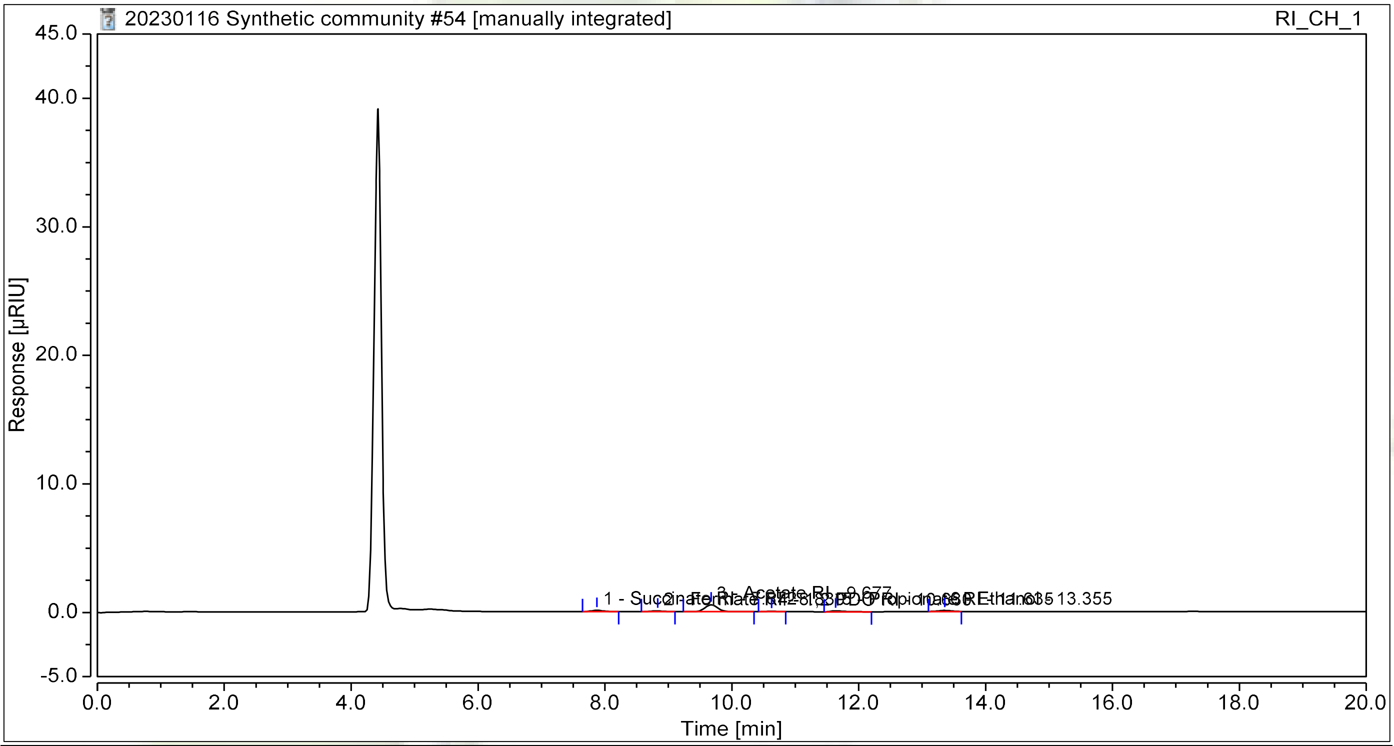

Integration Results

No.	Peak Name	Retention Time min	Area µRIU*min	Height µRIU	Relative Area %	Relative Height %	Amount
n.a.	GlcNAc	n.a.	n.a.	n.a.	n.a.	n.a.	n.a.
n.a.	Citrate	n.a.	n.a.	n.a.	n.a.	n.a.	n.a.
n.a.	Glucose	n.a.	n.a.	n.a.	n.a.	n.a.	n.a.
n.a.	Galactose	n.a.	n.a.	n.a.	n.a.	n.a.	n.a.
n.a.	Fucose	n.a.	n.a.	n.a.	n.a.	n.a.	n.a.
1	Succinate RI	7,872	0,019	0,094	8,95	10,64	0,3928
n.a.	Lactate RI	n.a.	n.a.	n.a.	n.a.	n.a.	n.a.
n.a.	glycerol	n.a.	n.a.	n.a.	n.a.	n.a.	n.a.
2	Formate RI	8,830	0,013	0,061	5,88	6,98	1,2797
3	Acetate RI	9,677	0,126	0,537	59,24	61,10	7,8078
4	1,2 PDO RI	10,630	0,004	0,017	1,78	1,94	0,1153
n.a.	1,3-PDO	n.a.	n.a.	n.a.	n.a.	n.a.	n.a.
5	Propionate RI	11,635	0,028	0,086	13,32	9,74	1,1840
n.a.	1,3-PDO	n.a.	n.a.	n.a.	n.a.	n.a.	n.a.
n.a.	2-3 BDO	n.a.	n.a.	n.a.	n.a.	n.a.	n.a.
6	Ethanol	13,355	0,023	0,084	10,82	9,60	2,4127
n.a.	Isobutyrate RI	n.a.	n.a.	n.a.	n.a.	n.a.	n.a.
n.a.	Butyrate RI	n.a.	n.a.	n.a.	n.a.	n.a.	n.a.
**Total:**			**0,213**	**0,879**	**100,00**	**100,00**	

Chromeleon (c) Dionex
Version 7.2.10.25868

Peak Analysis

Injection Details

Injection Name:	**MUCHMO2 t120 r3**	*Run Time (min):*	**20,00**
Vial Number:	**3:45**	*Injection Volume:*	**10,00**
Injection Type:	**Unknown**	*Channel:*	**RI_CH_1**
Calibration Level:		*Wavelength:*	**n.a.**
Instrument Method:	**Default method LC2030C 45 gr 20 min**	*Bandwidth:*	**n.a.**
Processing Method:	**Processing Method LC2030 45 gr**	*Dilution Factor:*	**1,0000**
Injection Date/Time:	**17/Jan/23 09:47**	*Sample Weight:*	**1,0000**

Chromatogram

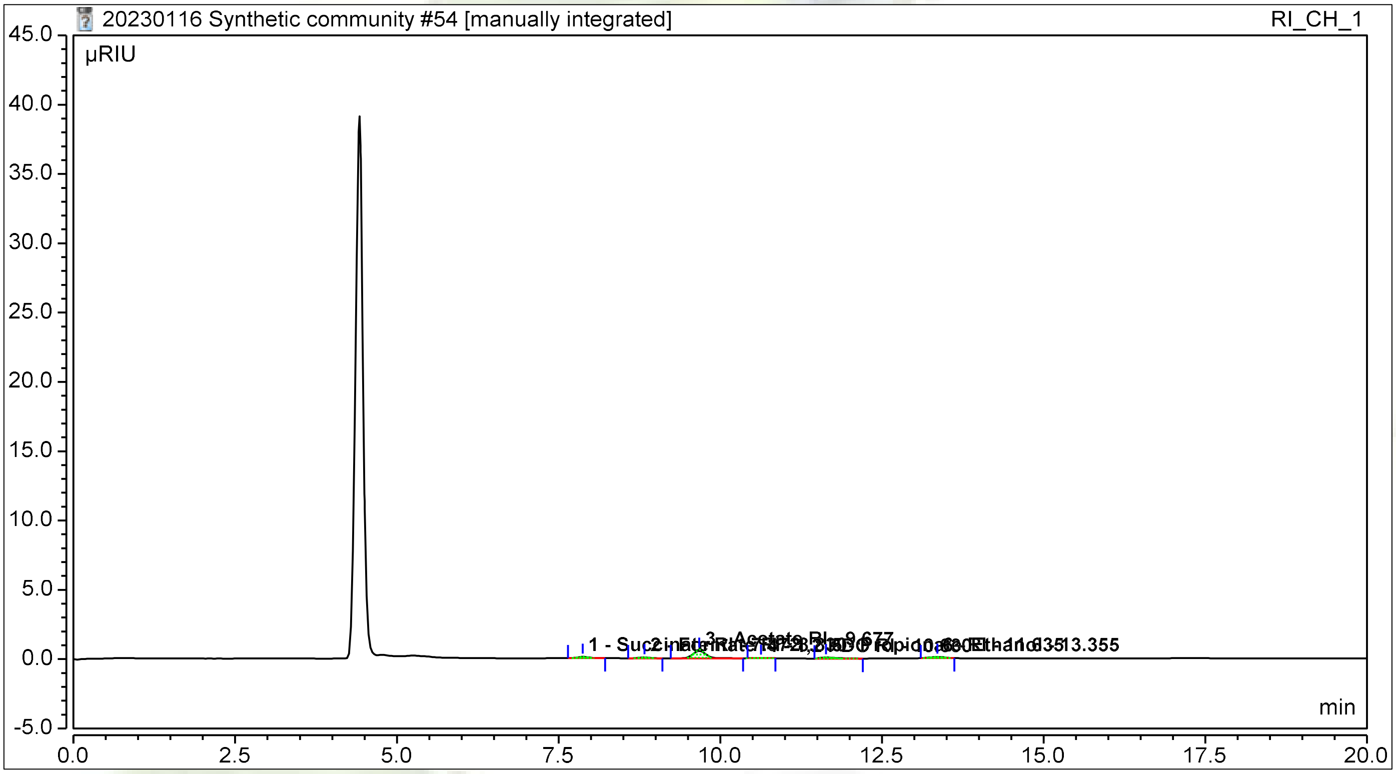

Peak Results

No.	Peak Name	Retention Time min	Width (50%) min	Type	Resolution (EP)	Asymmetry (EP)	Plates (EP)
n.a.	GlcNAc	n.a.	n.a.	n.a.	n.a.	n.a.	n.a.
n.a.	Citrate	n.a.	n.a.	n.a.	n.a.	n.a.	n.a.
n.a.	Glucose	n.a.	n.a.	n.a.	n.a.	n.a.	n.a.
n.a.	Galactose	n.a.	n.a.	n.a.	n.a.	n.a.	n.a.
n.a.	Fucose	n.a.	n.a.	n.a.	n.a.	n.a.	n.a.
1	Succinate RI	7,872	0,197	BMB*	2,89	1,02	8825
n.a.	Lactate RI	n.a.	n.a.	n.a.	n.a.	n.a.	n.a.
n.a.	glycerol	n.a.	n.a.	n.a.	n.a.	n.a.	n.a.
2	Formate RI	8,830	0,194	BMB*	2,42	1,02	11486
3	Acetate RI	9,677	0,219	BMB	2,54	1,04	10783
4	1,2 PDO RI	10,630	0,223	BMB*	2,45	1,04	12549
n.a.	1,3-PDO	n.a.	n.a.	n.a.	n.a.	n.a.	n.a.
5	Propionate RI	11,635	0,261	BMB*	3,82	2,03	10978
n.a.	1,3-PDO	n.a.	n.a.	n.a.	n.a.	n.a.	n.a.
n.a.	2-3 BDO	n.a.	n.a.	n.a.	n.a.	n.a.	n.a.
6	Ethanol	13,355	0,269	BMB*	n.a.	1,00	13629
n.a.	Isobutyrate RI	n.a.	n.a.	n.a.	n.a.	n.a.	n.a.
n.a.	Butyrate RI	n.a.	n.a.	n.a.	n.a.	n.a.	n.a.

Chromeleon (c) Dionex
Version 7.2.10.25868

Chromatogram and SST Results

Injection Details

Injection Name:	**MUCHMO2 t120 r3**	*Run Time (min):*	**20,00**
Vial Number:	**3:45**	*Injection Volume:*	**10,00**
Injection Type:	**Unknown**	*Channel:*	**RI_CH_1**
Calibration Level:		*Wavelength:*	**n.a.**
Instrument Method:	**Default method LC2030C 45 gr 20 min**	*Bandwidth:*	**n.a.**
Processing Method:	**Processing Method LC2030 45 gr**	*Dilution Factor:*	**1,0000**
Injection Date/Time:	**17/Jan/23 09:47**	*Sample Weight:*	**1,0000**

Chromatogram

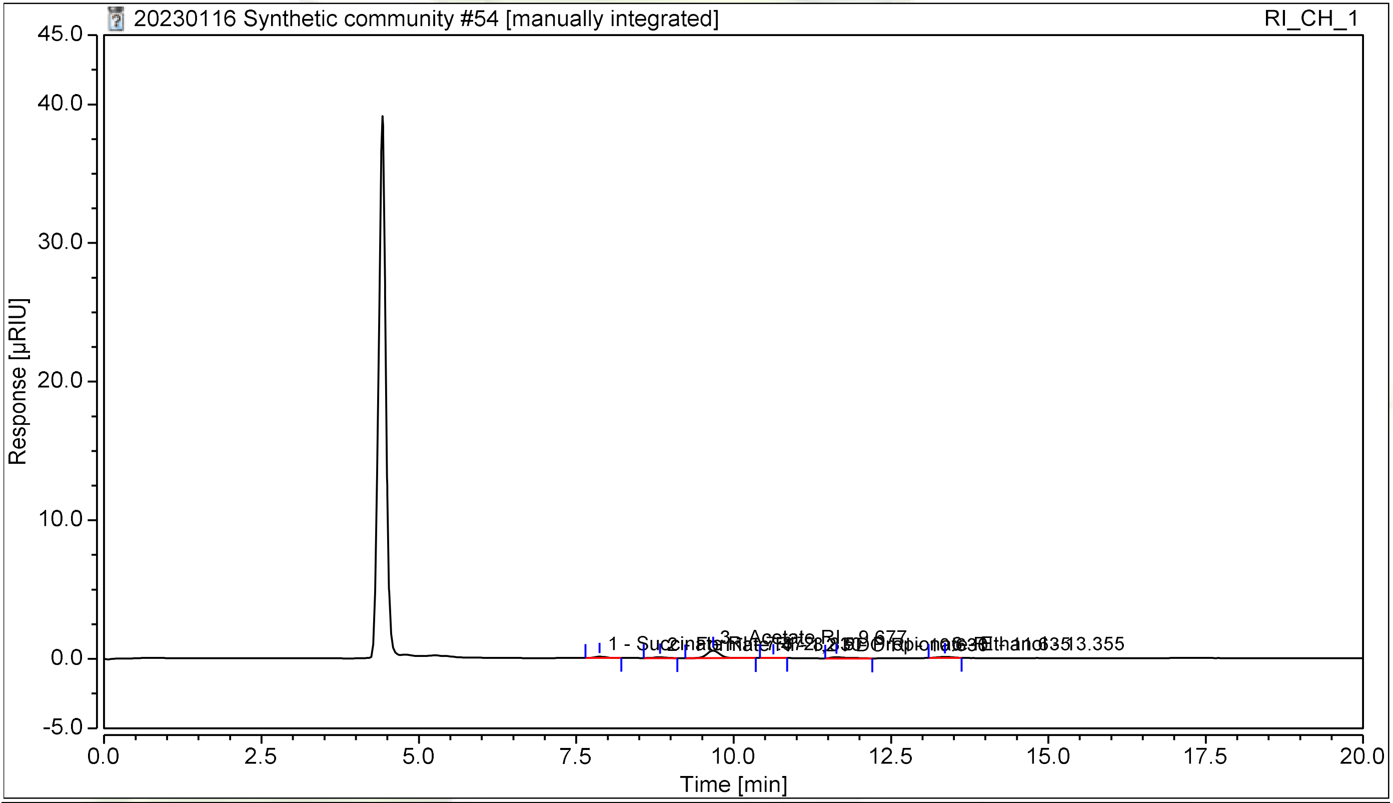

SST Results

No.	Name	Inj.Condition	Peak	Test Result	Injection

Number of executed test cases: n.a. **Total Result: Passed**

Chromeleon (c) Dionex
Version 7.2.10.25868

Chromatogram and Results

Injection Details

Injection Name:	**MUC t72 r1**	*Run Time (min):*	**20,00**
Vial Number:	**3:49**	*Injection Volume:*	**10,00**
Injection Type:	**Unknown**	*Channel:*	**RI_CH_1**
Calibration Level:		*Wavelength:*	**n.a.**
Instrument Method:	**Default method LC2030C 45 gr 20 min**	*Bandwidth:*	**n.a.**
Processing Method:	**Processing Method LC2030 45 gr**	*Dilution Factor:*	**1,0000**
Injection Date/Time:	**17/Jan/23 10:07**	*Sample Weight:*	**1,0000**

Chromatogram

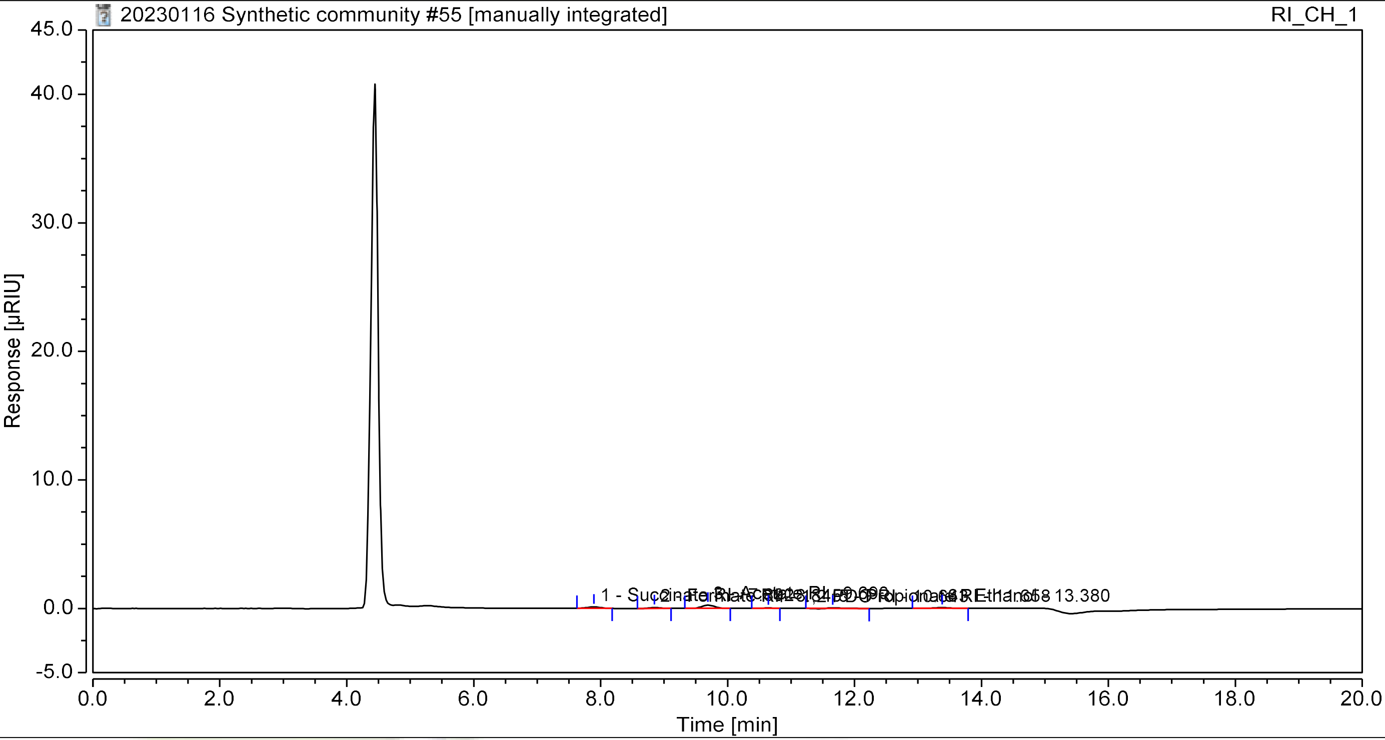

Integration Results

No.	Peak Name	Retention Time min	Area µRIU*min	Height µRIU	Relative Area %	Relative Height %	Amount
n.a.	GlcNAc	n.a.	n.a.	n.a.	n.a.	n.a.	n.a.
n.a.	Citrate	n.a.	n.a.	n.a.	n.a.	n.a.	n.a.
n.a.	Glucose	n.a.	n.a.	n.a.	n.a.	n.a.	n.a.
n.a.	Galactose	n.a.	n.a.	n.a.	n.a.	n.a.	n.a.
n.a.	Fucose	n.a.	n.a.	n.a.	n.a.	n.a.	n.a.
1	Succinate RI	7,892	0,025	0,112	18,55	19,61	0,5122
n.a.	Lactate RI	n.a.	n.a.	n.a.	n.a.	n.a.	n.a.
n.a.	glycerol	n.a.	n.a.	n.a.	n.a.	n.a.	n.a.
2	Formate RI	8,845	0,015	0,072	11,21	12,61	1,5345
3	Acetate RI	9,690	0,059	0,253	43,76	44,20	3,6314
4	1,2 PDO RI	10,643	0,005	0,021	3,60	3,64	0,1465
n.a.	1,3-PDO	n.a.	n.a.	n.a.	n.a.	n.a.	n.a.
5	Propionate RI	11,658	0,014	0,057	10,28	9,91	0,5752
n.a.	1,3-PDO	n.a.	n.a.	n.a.	n.a.	n.a.	n.a.
n.a.	2-3 BDO	n.a.	n.a.	n.a.	n.a.	n.a.	n.a.
6	Ethanol	13,380	0,017	0,058	12,61	10,04	1,7696
n.a.	Isobutyrate RI	n.a.	n.a.	n.a.	n.a.	n.a.	n.a.
n.a.	Butyrate RI	n.a.	n.a.	n.a.	n.a.	n.a.	n.a.
**Total:**			**0,134**	**0,573**	**100,00**	**100,00**	

Chromeleon (c) Dionex
Version 7.2.10.25868

Peak Analysis

Injection Details

Injection Name:	**MUC t72 r1**	*Run Time (min):*	**20,00**
Vial Number:	**3:49**	*Injection Volume:*	**10,00**
Injection Type:	**Unknown**	*Channel:*	**RI_CH_1**
Calibration Level:		*Wavelength:*	**n.a.**
Instrument Method:	**Default method LC2030C 45 gr 20 min**	*Bandwidth:*	**n.a.**
Processing Method:	**Processing Method LC2030 45 gr**	*Dilution Factor:*	**1,0000**
Injection Date/Time:	**17/Jan/23 10:07**	*Sample Weight:*	**1,0000**

Chromatogram

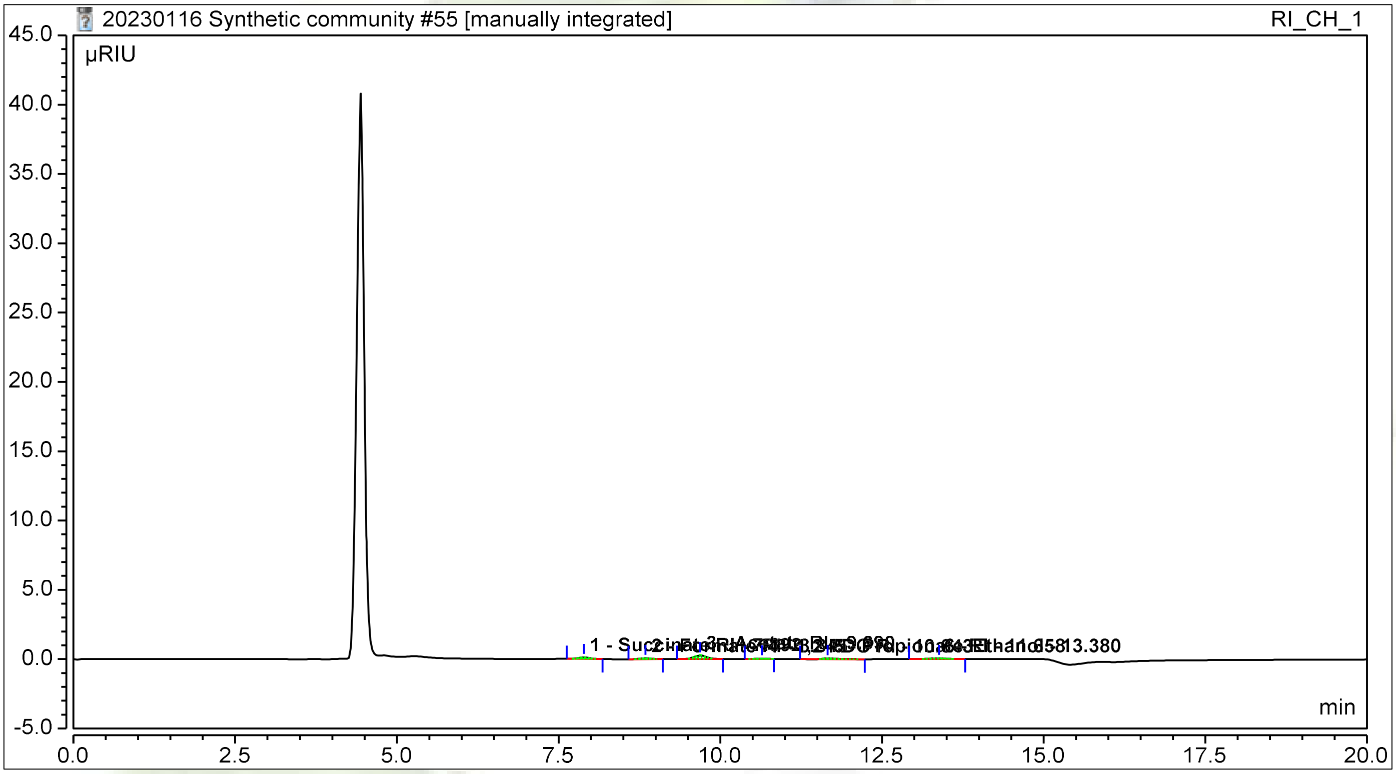

Peak Results

No.	Peak Name	Retention Time min	Width (50%) min	Type	Resolution (EP)	Asymmetry (EP)	Plates (EP)
n.a.	GlcNAc	n.a.	n.a.	n.a.	n.a.	n.a.	n.a.
n.a.	Citrate	n.a.	n.a.	n.a.	n.a.	n.a.	n.a.
n.a.	Glucose	n.a.	n.a.	n.a.	n.a.	n.a.	n.a.
n.a.	Galactose	n.a.	n.a.	n.a.	n.a.	n.a.	n.a.
n.a.	Fucose	n.a.	n.a.	n.a.	n.a.	n.a.	n.a.
1	Succinate RI	7,892	0,206	BMB*	2,80	1,05	8111
n.a.	Lactate RI	n.a.	n.a.	n.a.	n.a.	n.a.	n.a.
n.a.	glycerol	n.a.	n.a.	n.a.	n.a.	n.a.	n.a.
2	Formate RI	8,845	0,196	BMB*	2,40	1,05	11268
3	Acetate RI	9,690	0,219	BMB*	2,49	1,04	10849
4	1,2 PDO RI	10,643	0,233	BMB*	2,57	0,86	11552
n.a.	1,3-PDO	n.a.	n.a.	n.a.	n.a.	n.a.	n.a.
5	Propionate RI	11,658	0,233	BMB*	3,95	2,50	13908
n.a.	1,3-PDO	n.a.	n.a.	n.a.	n.a.	n.a.	n.a.
n.a.	2-3 BDO	n.a.	n.a.	n.a.	n.a.	n.a.	n.a.
6	Ethanol	13,380	0,281	BMB*	n.a.	1,01	12556
n.a.	Isobutyrate RI	n.a.	n.a.	n.a.	n.a.	n.a.	n.a.
n.a.	Butyrate RI	n.a.	n.a.	n.a.	n.a.	n.a.	n.a.

Chromeleon (c) Dionex
Version 7.2.10.25868

Chromatogram and SST Results

Injection Details

Injection Name:	**MUC t72 r1**	*Run Time (min):*	**20,00**
Vial Number:	**3:49**	*Injection Volume:*	**10,00**
Injection Type:	**Unknown**	*Channel:*	**RI_CH_1**
Calibration Level:		*Wavelength:*	**n.a.**
Instrument Method:	**Default method LC2030C 45 gr 20 min**	*Bandwidth:*	**n.a.**
Processing Method:	**Processing Method LC2030 45 gr**	*Dilution Factor:*	**1,0000**
Injection Date/Time:	**17/Jan/23 10:07**	*Sample Weight:*	**1,0000**

Chromatogram

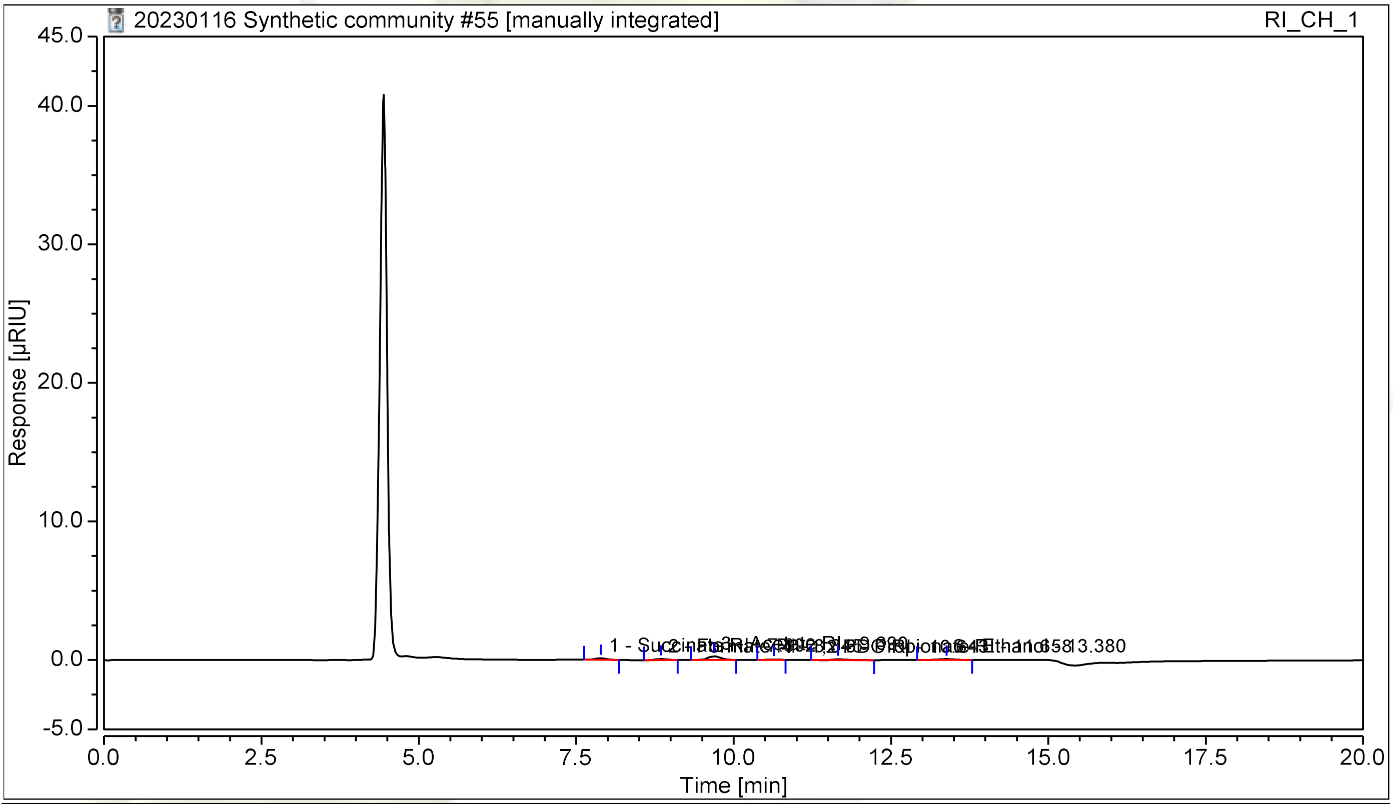

SST Results

No.	Name	Inj.Condition	Peak	Test Result	Injection
**Number of executed test cases:**	**n.a.**		**Total Result:**	**Passed**	

Chromatogram and Results

Injection Details

Injection Name:	**MUC t72 r2**	*Run Time (min):*	**20,00**
Vial Number:	**3:50**	*Injection Volume:*	**10,00**
Injection Type:	**Unknown**	*Channel:*	**RI_CH_1**
Calibration Level:		*Wavelength:*	**n.a.**
Instrument Method:	**Default method LC2030C 45 gr 20 min**	*Bandwidth:*	**n.a.**
Processing Method:	**Processing Method LC2030 45 gr**	*Dilution Factor:*	**1,0000**
Injection Date/Time:	**17/Jan/23 10:28**	*Sample Weight:*	**1,0000**

Chromatogram

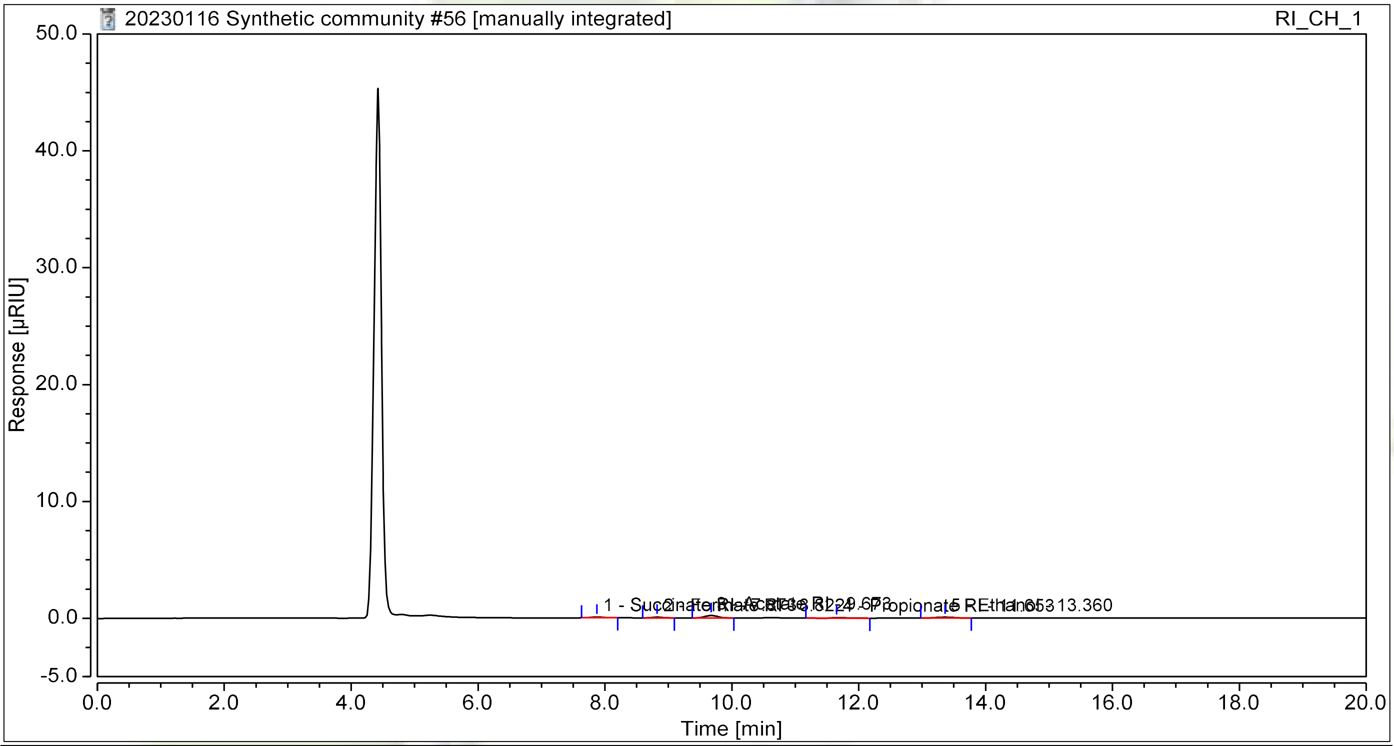

Integration Results

No.	Peak Name	Retention Time min	Area µRIU*min	Height µRIU	Relative Area %	Relative Height %	Amount
n.a.	GlcNAc	n.a.	n.a.	n.a.	n.a.	n.a.	n.a.
n.a.	Citrate	n.a.	n.a.	n.a.	n.a.	n.a.	n.a.
n.a.	Glucose	n.a.	n.a.	n.a.	n.a.	n.a.	n.a.
n.a.	Galactose	n.a.	n.a.	n.a.	n.a.	n.a.	n.a.
n.a.	Fucose	n.a.	n.a.	n.a.	n.a.	n.a.	n.a.
1	Succinate RI	7,873	0,013	0,067	12,12	14,61	0,2610
n.a.	Lactate RI	n.a.	n.a.	n.a.	n.a.	n.a.	n.a.
n.a.	glycerol	n.a.	n.a.	n.a.	n.a.	n.a.	n.a.
2	Formate RI	8,822	0,013	0,062	12,12	13,68	1,2942
3	Acetate RI	9,673	0,050	0,218	48,21	47,80	3,1189
n.a.	1,2 PDO RI	n.a.	n.a.	n.a.	n.a.	n.a.	n.a.
n.a.	1,3-PDO	n.a.	n.a.	n.a.	n.a.	n.a.	n.a.
4	Propionate RI	11,653	0,009	0,043	8,94	9,49	0,3900
n.a.	1,3-PDO	n.a.	n.a.	n.a.	n.a.	n.a.	n.a.
n.a.	2-3 BDO	n.a.	n.a.	n.a.	n.a.	n.a.	n.a.
5	Ethanol	13,360	0,019	0,066	18,61	14,42	2,0371
n.a.	Isobutyrate RI	n.a.	n.a.	n.a.	n.a.	n.a.	n.a.
n.a.	Butyrate RI	n.a.	n.a.	n.a.	n.a.	n.a.	n.a.
**Total:**			**0,104**	**0,456**	**100,00**	**100,00**	

Chromeleon (c) Dionex
Version 7.2.10.25868

Peak Analysis

Injection Details

Injection Name:	**MUC t72 r2**	*Run Time (min):*	**20,00**
Vial Number:	**3:50**	*Injection Volume:*	**10,00**
Injection Type:	**Unknown**	*Channel:*	**RI_CH_1**
Calibration Level:		*Wavelength:*	**n.a.**
Instrument Method:	**Default method LC2030C 45 gr 20 min**	*Bandwidth:*	**n.a.**
Processing Method:	**Processing Method LC2030 45 gr**	*Dilution Factor:*	**1,0000**
Injection Date/Time:	**17/Jan/23 10:28**	*Sample Weight:*	**1,0000**

Chromatogram

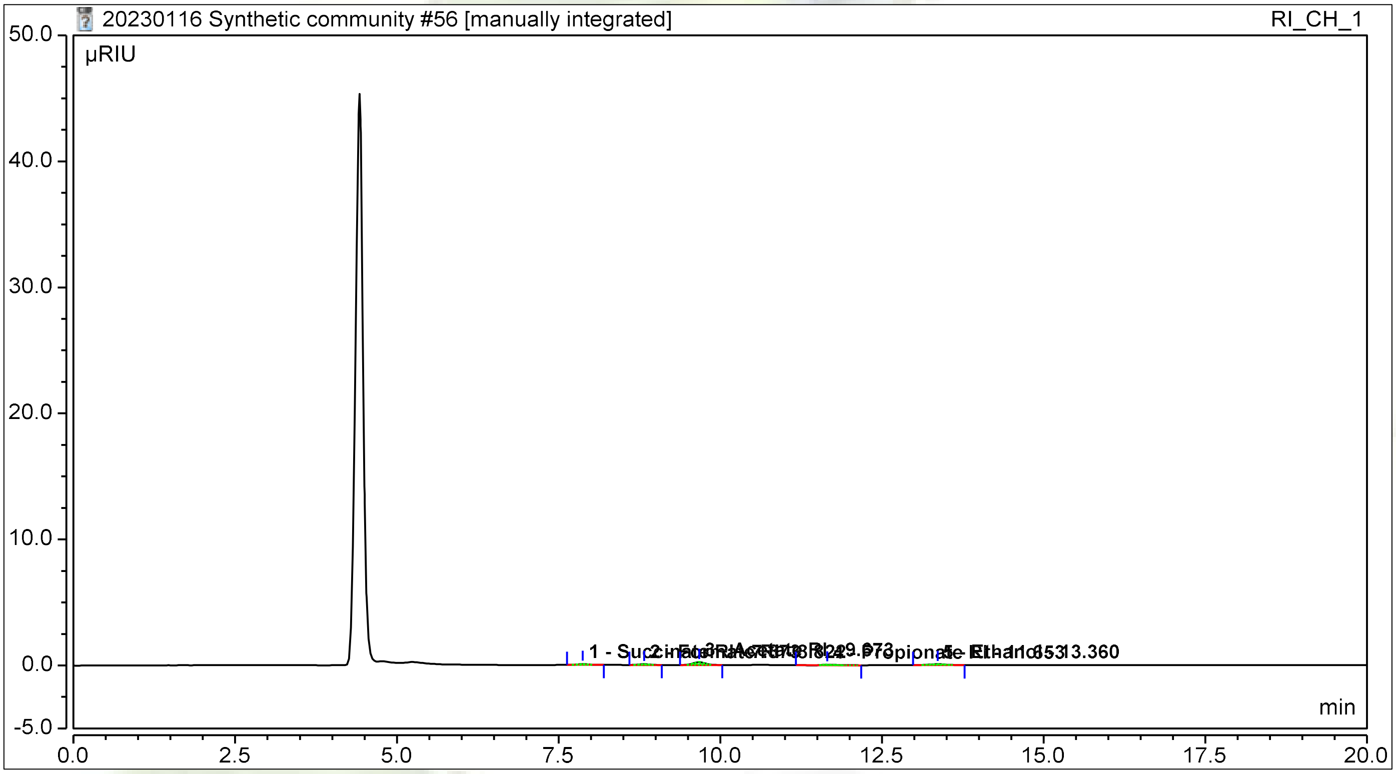

Peak Results

No.	Peak Name	Retention Time min	Width (50%) min	Type	Resolution (EP)	Asymmetry (EP)	Plates (EP)
n.a.	GlcNAc	n.a.	n.a.	n.a.	n.a.	n.a.	n.a.
n.a.	Citrate	n.a.	n.a.	n.a.	n.a.	n.a.	n.a.
n.a.	Glucose	n.a.	n.a.	n.a.	n.a.	n.a.	n.a.
n.a.	Galactose	n.a.	n.a.	n.a.	n.a.	n.a.	n.a.
n.a.	Fucose	n.a.	n.a.	n.a.	n.a.	n.a.	n.a.
1	Succinate RI	7,873	0,192	BMB*	2,91	0,96	9357
n.a.	Lactate RI	n.a.	n.a.	n.a.	n.a.	n.a.	n.a.
n.a.	glycerol	n.a.	n.a.	n.a.	n.a.	n.a.	n.a.
2	Formate RI	8,822	0,193	BMB*	2,44	1,05	11552
3	Acetate RI	9,673	0,218	BMB*	4,86	1,05	10860
n.a.	1,2 PDO RI	n.a.	n.a.	n.a.	n.a.	n.a.	n.a.
n.a.	1,3-PDO	n.a.	n.a.	n.a.	n.a.	n.a.	n.a.
4	Propionate RI	11,653	0,262	BMB*	3,70	2,52	10971
n.a.	1,3-PDO	n.a.	n.a.	n.a.	n.a.	n.a.	n.a.
n.a.	2-3 BDO	n.a.	n.a.	n.a.	n.a.	n.a.	n.a.
5	Ethanol	13,360	0,282	BMB*	n.a.	1,02	12394
n.a.	Isobutyrate RI	n.a.	n.a.	n.a.	n.a.	n.a.	n.a.
n.a.	Butyrate RI	n.a.	n.a.	n.a.	n.a.	n.a.	n.a.

Chromeleon (c) Dionex
Version 7.2.10.25868

Chromatogram and SST Results

Injection Details

Injection Name:	**MUC t72 r2**	*Run Time (min):*	**20,00**
Vial Number:	**3:50**	*Injection Volume:*	**10,00**
Injection Type:	**Unknown**	*Channel:*	**RI_CH_1**
Calibration Level:		*Wavelength:*	**n.a.**
Instrument Method:	**Default method LC2030C 45 gr 20 min**	*Bandwidth:*	**n.a.**
Processing Method:	**Processing Method LC2030 45 gr**	*Dilution Factor:*	**1,0000**
Injection Date/Time:	**17/Jan/23 10:28**	*Sample Weight:*	**1,0000**

Chromatogram

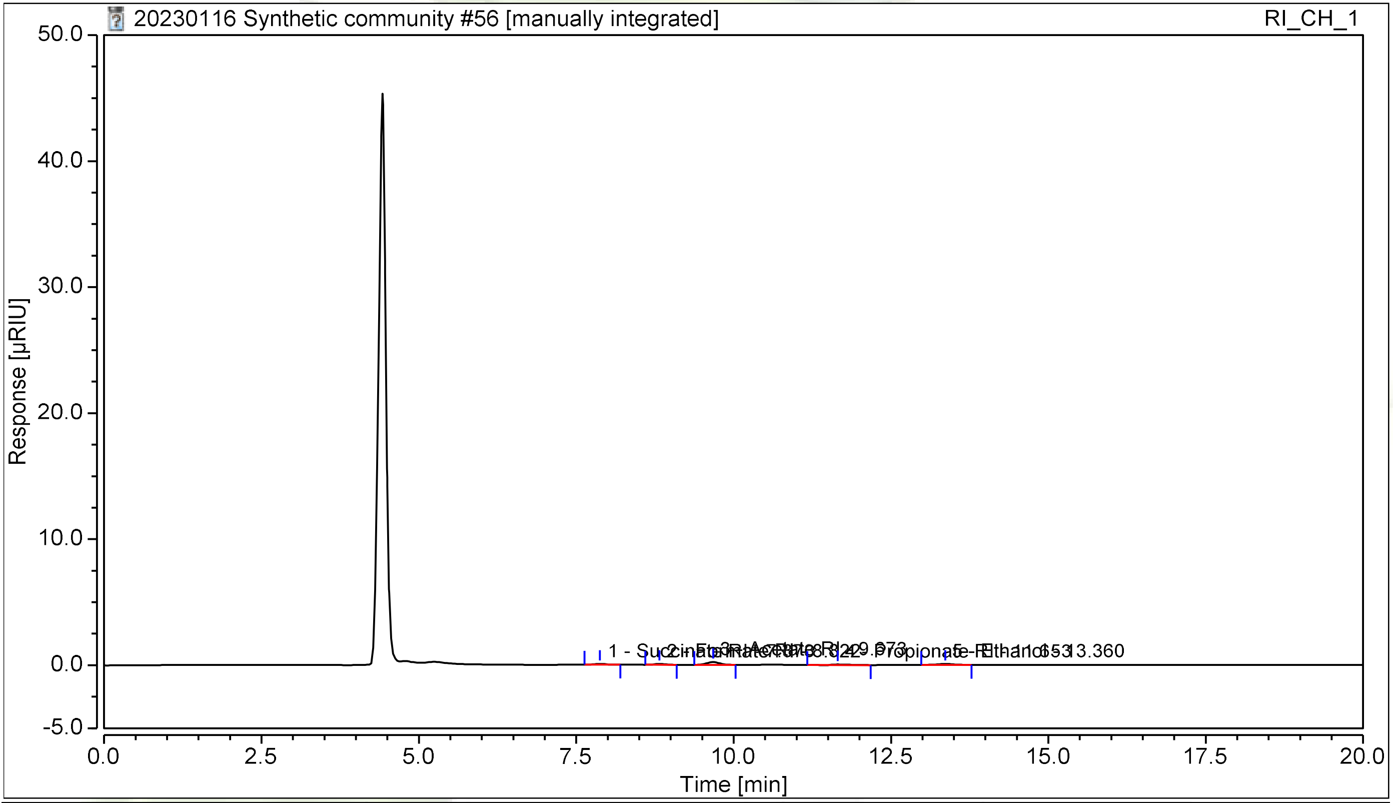

SST Results

No.	Name	Inj.Condition	Peak	Test Result	Injection
**Number of executed test cases:**	**n.a.**		**Total Result:**	**Passed**	

Chromatogram and Results

Injection Details

Injection Name:	**MUC t72 r3**	*Run Time (min):*	**20,00**
Vial Number:	**3:51**	*Injection Volume:*	**10,00**
Injection Type:	**Unknown**	*Channel:*	**RI_CH_1**
Calibration Level:		*Wavelength:*	**n.a.**
Instrument Method:	**Default method LC2030C 45 gr 20 min**	*Bandwidth:*	**n.a.**
Processing Method:	**Processing Method LC2030 45 gr**	*Dilution Factor:*	**1,0000**
Injection Date/Time:	**17/Jan/23 10:48**	*Sample Weight:*	**1,0000**

Chromatogram

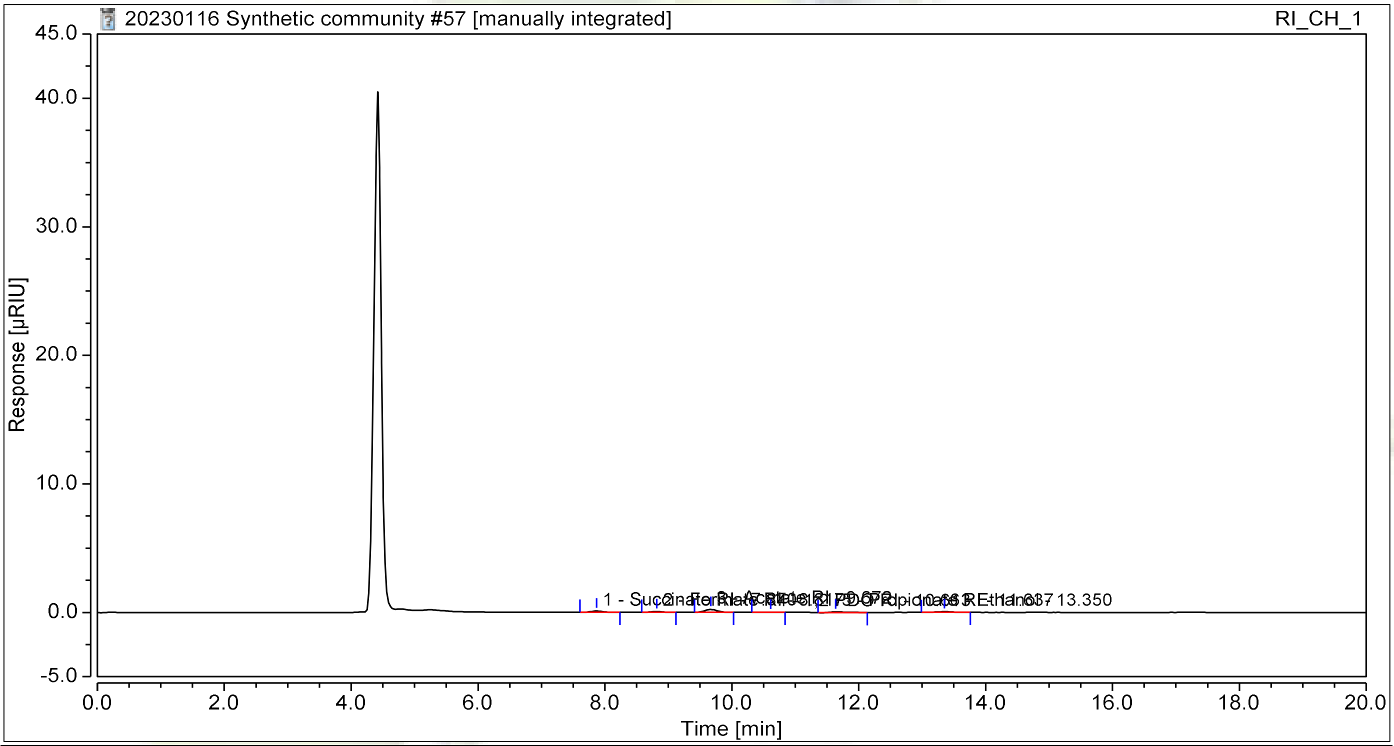

Integration Results

No.	Peak Name	Retention Time min	Area µRIU*min	Height µRIU	Relative Area %	Relative Height %	Amount
n.a.	GlcNAc	n.a.	n.a.	n.a.	n.a.	n.a.	n.a.
n.a.	Citrate	n.a.	n.a.	n.a.	n.a.	n.a.	n.a.
n.a.	Glucose	n.a.	n.a.	n.a.	n.a.	n.a.	n.a.
n.a.	Galactose	n.a.	n.a.	n.a.	n.a.	n.a.	n.a.
n.a.	Fucose	n.a.	n.a.	n.a.	n.a.	n.a.	n.a.
1	Succinate RI	7,870	0,017	0,089	14,40	17,81	0,3544
n.a.	Lactate RI	n.a.	n.a.	n.a.	n.a.	n.a.	n.a.
n.a.	glycerol	n.a.	n.a.	n.a.	n.a.	n.a.	n.a.
2	Formate RI	8,817	0,013	0,062	10,61	12,45	1,2947
3	Acetate RI	9,672	0,051	0,222	42,63	44,47	3,1512
4	1,2 PDO RI	10,613	0,002	0,007	1,42	1,37	0,0516
n.a.	1,3-PDO	n.a.	n.a.	n.a.	n.a.	n.a.	n.a.
5	Propionate RI	11,637	0,020	0,064	16,89	12,76	0,8421
n.a.	1,3-PDO	n.a.	n.a.	n.a.	n.a.	n.a.	n.a.
n.a.	2-3 BDO	n.a.	n.a.	n.a.	n.a.	n.a.	n.a.
6	Ethanol	13,350	0,017	0,056	14,03	11,14	1,7544
n.a.	Isobutyrate RI	n.a.	n.a.	n.a.	n.a.	n.a.	n.a.
n.a.	Butyrate RI	n.a.	n.a.	n.a.	n.a.	n.a.	n.a.
**Total:**			**0,119**	**0,500**	**100,00**	**100,00**	

Chromeleon (c) Dionex
Version 7.2.10.25868

Peak Analysis

Injection Details

Injection Name:	**MUC t72 r3**	*Run Time (min):*	**20,00**
Vial Number:	**3:51**	*Injection Volume:*	**10,00**
Injection Type:	**Unknown**	*Channel:*	**RI_CH_1**
Calibration Level:		*Wavelength:*	**n.a.**
Instrument Method:	**Default method LC2030C 45 gr 20 min**	*Bandwidth:*	**n.a.**
Processing Method:	**Processing Method LC2030 45 gr**	*Dilution Factor:*	**1,0000**
Injection Date/Time:	**17/Jan/23 10:48**	*Sample Weight:*	**1,0000**

Chromatogram

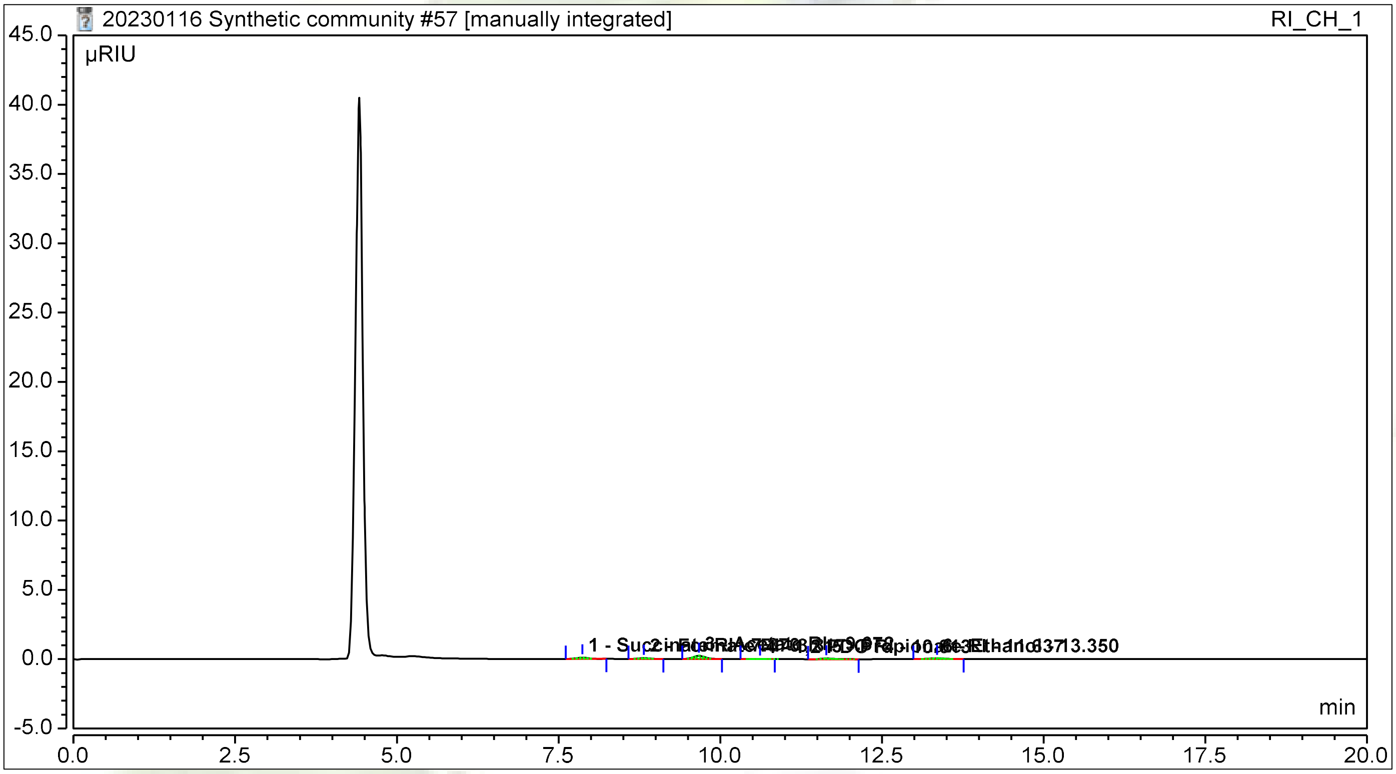

Peak Results

No.	Peak Name	Retention Time min	Width (50%) min	Type	Resolution (EP)	Asymmetry (EP)	Plates (EP)
n.a.	GlcNAc	n.a.	n.a.	n.a.	n.a.	n.a.	n.a.
n.a.	Citrate	n.a.	n.a.	n.a.	n.a.	n.a.	n.a.
n.a.	Glucose	n.a.	n.a.	n.a.	n.a.	n.a.	n.a.
n.a.	Galactose	n.a.	n.a.	n.a.	n.a.	n.a.	n.a.
n.a.	Fucose	n.a.	n.a.	n.a.	n.a.	n.a.	n.a.
1	Succinate RI	7,870	0,194	BMB*	2,88	0,98	9143
n.a.	Lactate RI	n.a.	n.a.	n.a.	n.a.	n.a.	n.a.
n.a.	glycerol	n.a.	n.a.	n.a.	n.a.	n.a.	n.a.
2	Formate RI	8,817	0,194	BMB*	2,45	1,10	11399
3	Acetate RI	9,672	0,217	BMB*	2,49	1,05	11005
4	1,2 PDO RI	10,613	0,230	BMB*	2,45	0,86	11784
n.a.	1,3-PDO	n.a.	n.a.	n.a.	n.a.	n.a.	n.a.
5	Propionate RI	11,637	0,262	BMB*	3,70	2,00	10934
n.a.	1,3-PDO	n.a.	n.a.	n.a.	n.a.	n.a.	n.a.
n.a.	2-3 BDO	n.a.	n.a.	n.a.	n.a.	n.a.	n.a.
6	Ethanol	13,350	0,284	BMB*	n.a.	1,05	12205
n.a.	Isobutyrate RI	n.a.	n.a.	n.a.	n.a.	n.a.	n.a.
n.a.	Butyrate RI	n.a.	n.a.	n.a.	n.a.	n.a.	n.a.

Chromatogram and SST Results

Injection Details

Injection Name:	**MUC t72 r3**	*Run Time (min):*	**20,00**
Vial Number:	**3:51**	*Injection Volume:*	**10,00**
Injection Type:	**Unknown**	*Channel:*	**RI_CH_1**
Calibration Level:		*Wavelength:*	**n.a.**
Instrument Method:	**Default method LC2030C 45 gr 20 min**	*Bandwidth:*	**n.a.**
Processing Method:	**Processing Method LC2030 45 gr**	*Dilution Factor:*	**1,0000**
Injection Date/Time:	**17/Jan/23 10:48**	*Sample Weight:*	**1,0000**

Chromatogram

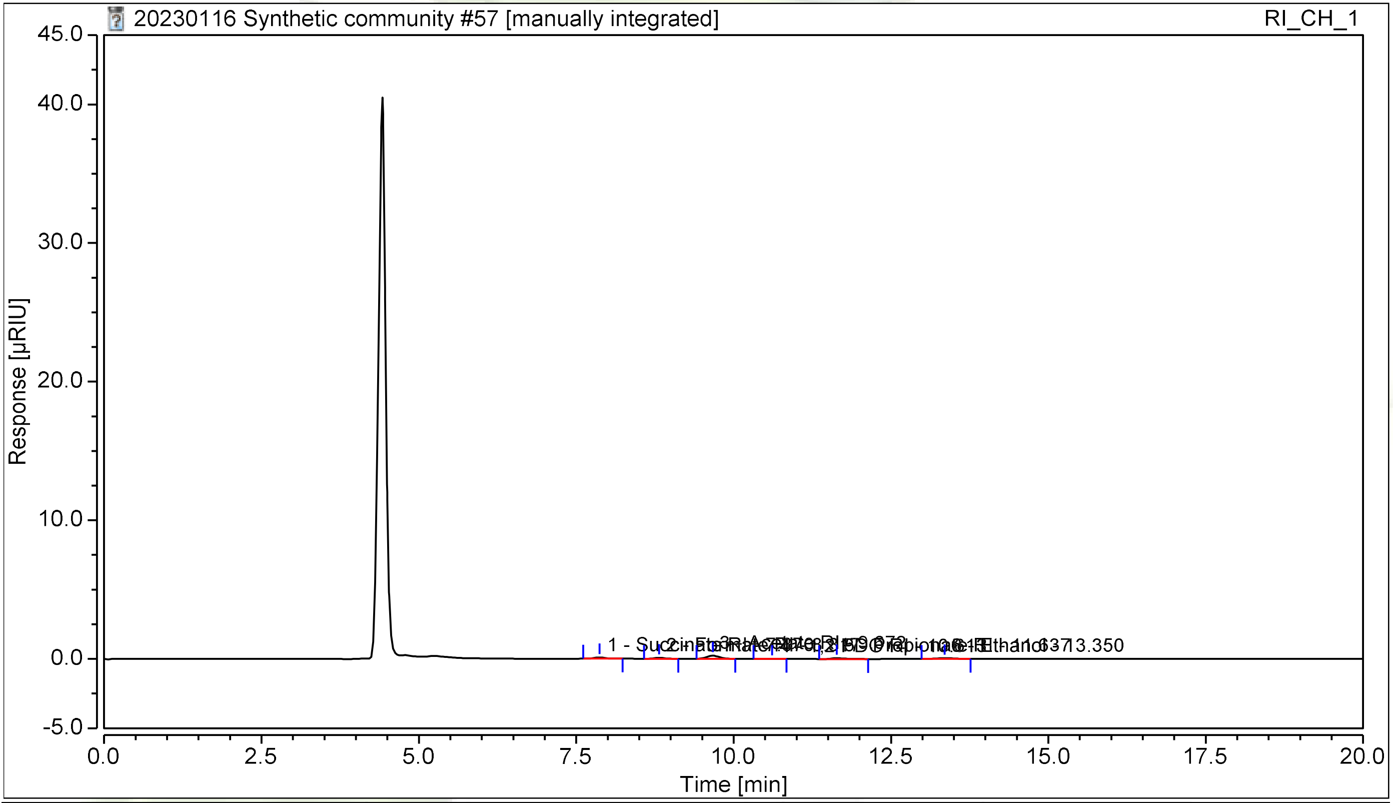

SST Results

No.	Name	Inj.Condition	Peak	Test Result	Injection
**Number of executed test cases:**	**n.a.**		**Total Result:**	**Passed**	

Chromatogram and Results

Injection Details

Injection Name:	**MUC t96 r1**	*Run Time (min):*	**20,00**
Vial Number:	**3:52**	*Injection Volume:*	**10,00**
Injection Type:	**Unknown**	*Channel:*	**RI_CH_1**
Calibration Level:		*Wavelength:*	**n.a.**
Instrument Method:	**Default method LC2030C 45 gr 20 min**	*Bandwidth:*	**n.a.**
Processing Method:	**Processing Method LC2030 45 gr**	*Dilution Factor:*	**1,0000**
Injection Date/Time:	**17/Jan/23 11:08**	*Sample Weight:*	**1,0000**

Chromatogram

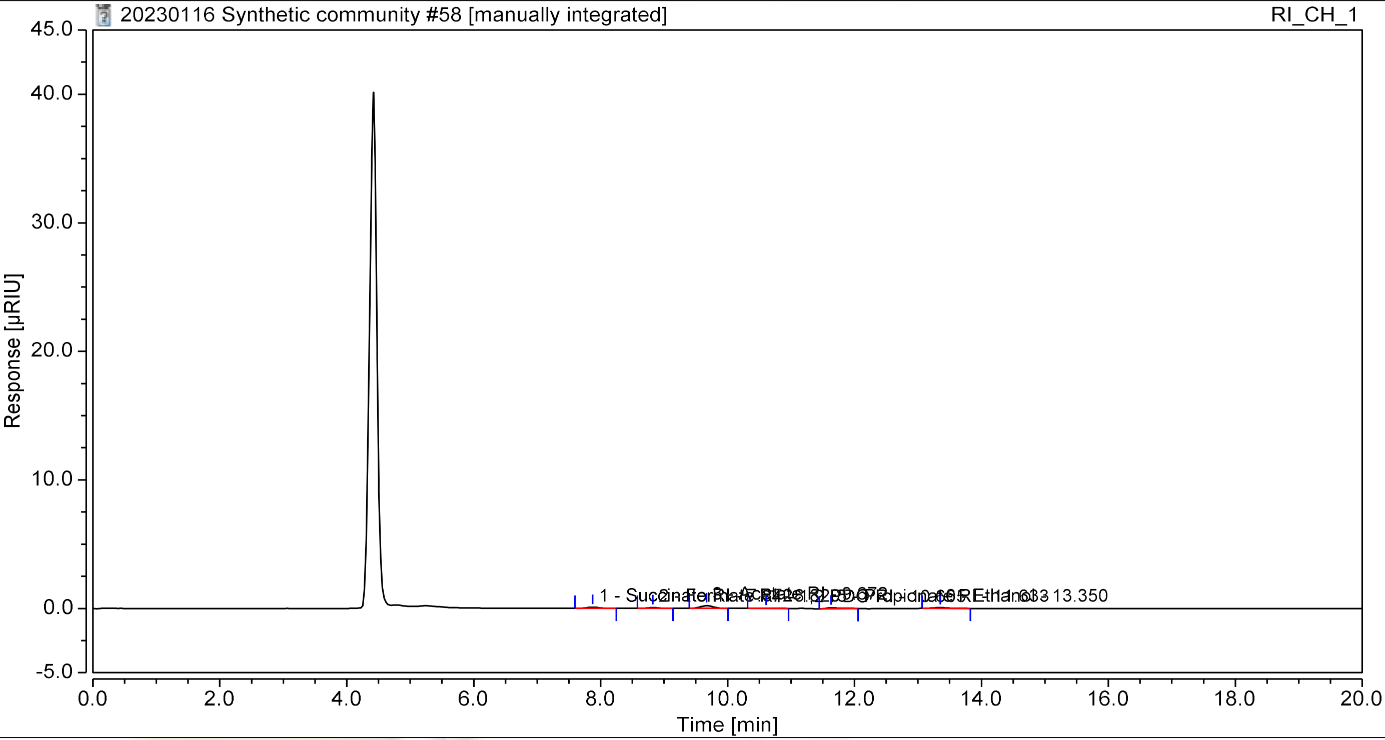

Integration Results

No.	Peak Name	Retention Time min	Area µRIU*min	Height µRIU	Relative Area %	Relative Height %	Amount
n.a.	GlcNAc	n.a.	n.a.	n.a.	n.a.	n.a.	n.a.
n.a.	Citrate	n.a.	n.a.	n.a.	n.a.	n.a.	n.a.
n.a.	Glucose	n.a.	n.a.	n.a.	n.a.	n.a.	n.a.
n.a.	Galactose	n.a.	n.a.	n.a.	n.a.	n.a.	n.a.
n.a.	Fucose	n.a.	n.a.	n.a.	n.a.	n.a.	n.a.
1	Succinate RI	7,872	0,016	0,083	13,91	16,81	0,3343
n.a.	Lactate RI	n.a.	n.a.	n.a.	n.a.	n.a.	n.a.
n.a.	glycerol	n.a.	n.a.	n.a.	n.a.	n.a.	n.a.
2	Formate RI	8,820	0,012	0,059	10,34	12,00	1,2321
3	Acetate RI	9,672	0,048	0,208	41,07	42,29	2,9655
4	1,2 PDO RI	10,605	0,005	0,019	4,14	3,89	0,1466
n.a.	1,3-PDO	n.a.	n.a.	n.a.	n.a.	n.a.	n.a.
5	Propionate RI	11,633	0,018	0,065	15,86	13,15	0,7725
n.a.	1,3-PDO	n.a.	n.a.	n.a.	n.a.	n.a.	n.a.
n.a.	2-3 BDO	n.a.	n.a.	n.a.	n.a.	n.a.	n.a.
6	Ethanol	13,350	0,017	0,058	14,68	11,87	1,7929
n.a.	Isobutyrate RI	n.a.	n.a.	n.a.	n.a.	n.a.	n.a.
n.a.	Butyrate RI	n.a.	n.a.	n.a.	n.a.	n.a.	n.a.
**Total:**			**0,116**	**0,492**	**100,00**	**100,00**	

Chromeleon (c) Dionex
Version 7.2.10.25868

Peak Analysis

Injection Details

Injection Name:	**MUC t96 r1**	*Run Time (min):*	**20,00**
Vial Number:	**3:52**	*Injection Volume:*	**10,00**
Injection Type:	**Unknown**	*Channel:*	**RI_CH_1**
Calibration Level:		*Wavelength:*	**n.a.**
Instrument Method:	**Default method LC2030C 45 gr 20 min**	*Bandwidth:*	**n.a.**
Processing Method:	**Processing Method LC2030 45 gr**	*Dilution Factor:*	**1,0000**
Injection Date/Time:	**17/Jan/23 11:08**	*Sample Weight:*	**1,0000**

Chromatogram

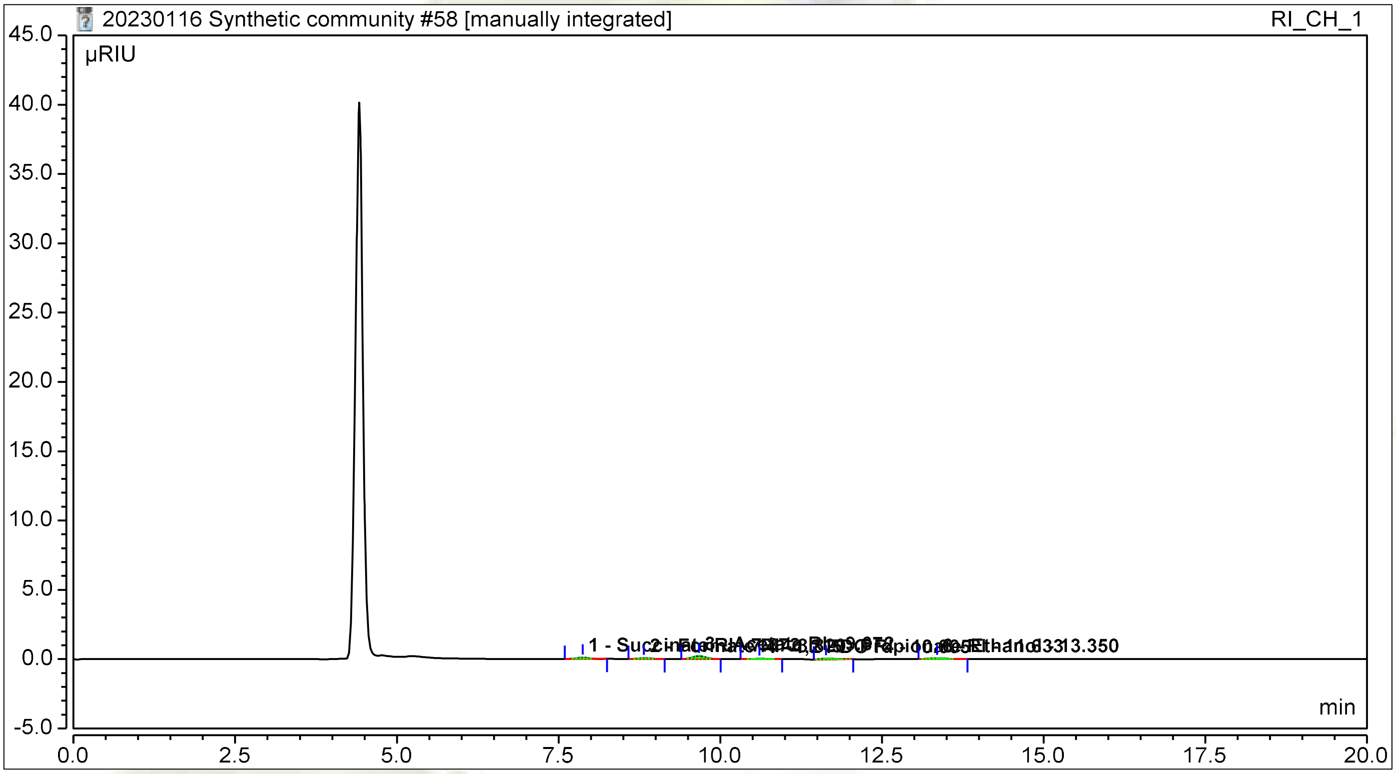

Peak Results

No.	Peak Name	Retention Time min	Width (50%) min	Type	Resolution (EP)	Asymmetry (EP)	Plates (EP)
n.a.	GlcNAc	n.a.	n.a.	n.a.	n.a.	n.a.	n.a.
n.a.	Citrate	n.a.	n.a.	n.a.	n.a.	n.a.	n.a.
n.a.	Glucose	n.a.	n.a.	n.a.	n.a.	n.a.	n.a.
n.a.	Galactose	n.a.	n.a.	n.a.	n.a.	n.a.	n.a.
n.a.	Fucose	n.a.	n.a.	n.a.	n.a.	n.a.	n.a.
1	Succinate RI	7,872	0,194	BMB*	2,90	0,97	9163
n.a.	Lactate RI	n.a.	n.a.	n.a.	n.a.	n.a.	n.a.
n.a.	glycerol	n.a.	n.a.	n.a.	n.a.	n.a.	n.a.
2	Formate RI	8,820	0,192	BMB*	2,45	1,08	11689
3	Acetate RI	9,672	0,218	BMB*	2,40	1,04	10881
4	1,2 PDO RI	10,605	0,241	BMB*	2,48	1,24	10763
n.a.	1,3-PDO	n.a.	n.a.	n.a.	n.a.	n.a.	n.a.
5	Propionate RI	11,633	0,250	BMB*	3,82	1,71	12028
n.a.	1,3-PDO	n.a.	n.a.	n.a.	n.a.	n.a.	n.a.
n.a.	2-3 BDO	n.a.	n.a.	n.a.	n.a.	n.a.	n.a.
6	Ethanol	13,350	0,281	BMB*	n.a.	1,10	12524
n.a.	Isobutyrate RI	n.a.	n.a.	n.a.	n.a.	n.a.	n.a.
n.a.	Butyrate RI	n.a.	n.a.	n.a.	n.a.	n.a.	n.a.

Chromatogram and SST Results

Injection Details

Injection Name:	**MUC t96 r1**	*Run Time (min):*	**20,00**
Vial Number:	**3:52**	*Injection Volume:*	**10,00**
Injection Type:	**Unknown**	*Channel:*	**RI_CH_1**
Calibration Level:		*Wavelength:*	**n.a.**
Instrument Method:	**Default method LC2030C 45 gr 20 min**	*Bandwidth:*	**n.a.**
Processing Method:	**Processing Method LC2030 45 gr**	*Dilution Factor:*	**1,0000**
Injection Date/Time:	**17/Jan/23 11:08**	*Sample Weight:*	**1,0000**

Chromatogram

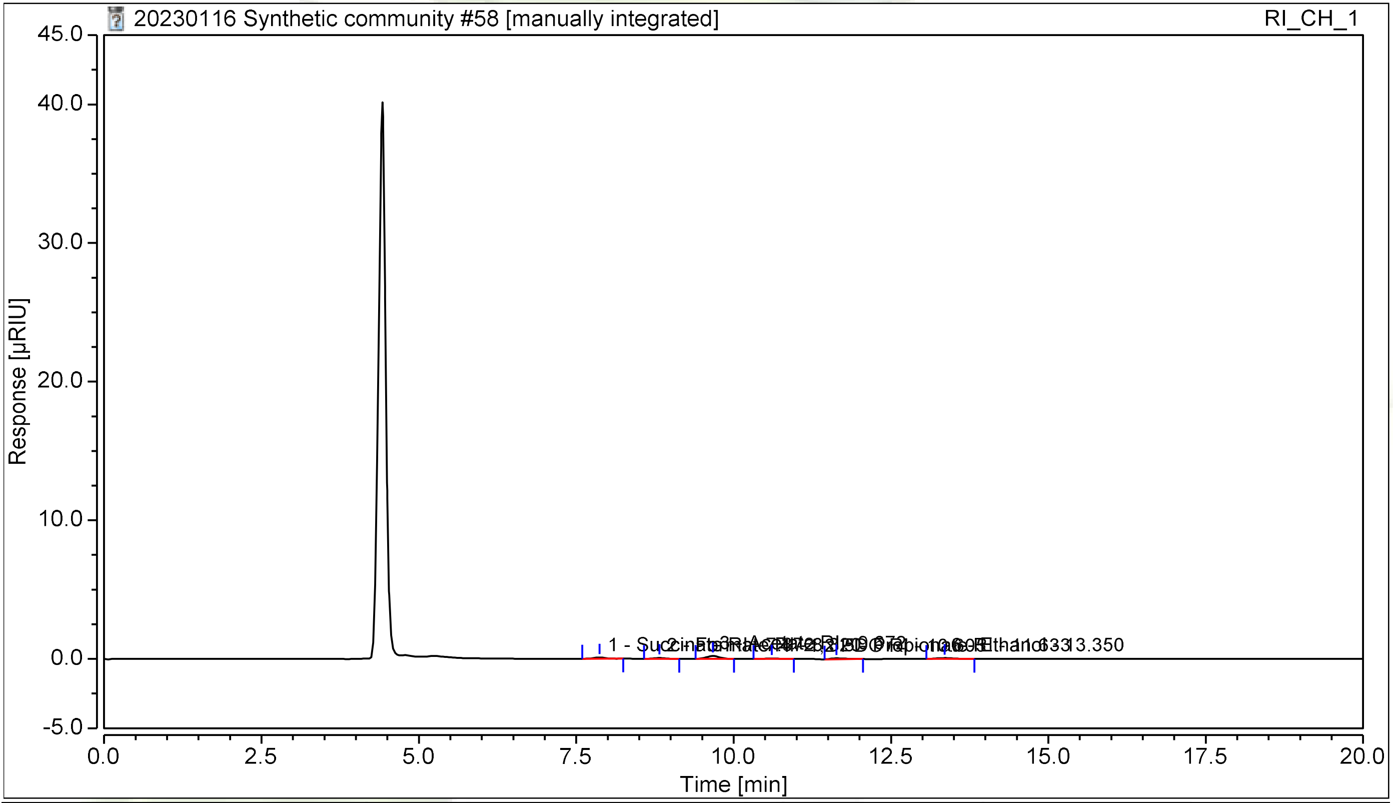

SST Results

No.	Name	Inj.Condition	Peak	Test Result	Injection

Number of executed test cases: **n.a.** **Total Result:** **Passed**

Chromeleon (c) Dionex
Version 7.2.10.25868

Chromatogram and Results

Injection Details

Injection Name:	**MUC t96 r2**	*Run Time (min):*	**20,00**
Vial Number:	**3:53**	*Injection Volume:*	**10,00**
Injection Type:	**Unknown**	*Channel:*	**RI_CH_1**
Calibration Level:		*Wavelength:*	**n.a.**
Instrument Method:	**Default method LC2030C 45 gr 20 min**	*Bandwidth:*	**n.a.**
Processing Method:	**Processing Method LC2030 45 gr**	*Dilution Factor:*	**1,0000**
Injection Date/Time:	**17/Jan/23 11:29**	*Sample Weight:*	**1,0000**

Chromatogram

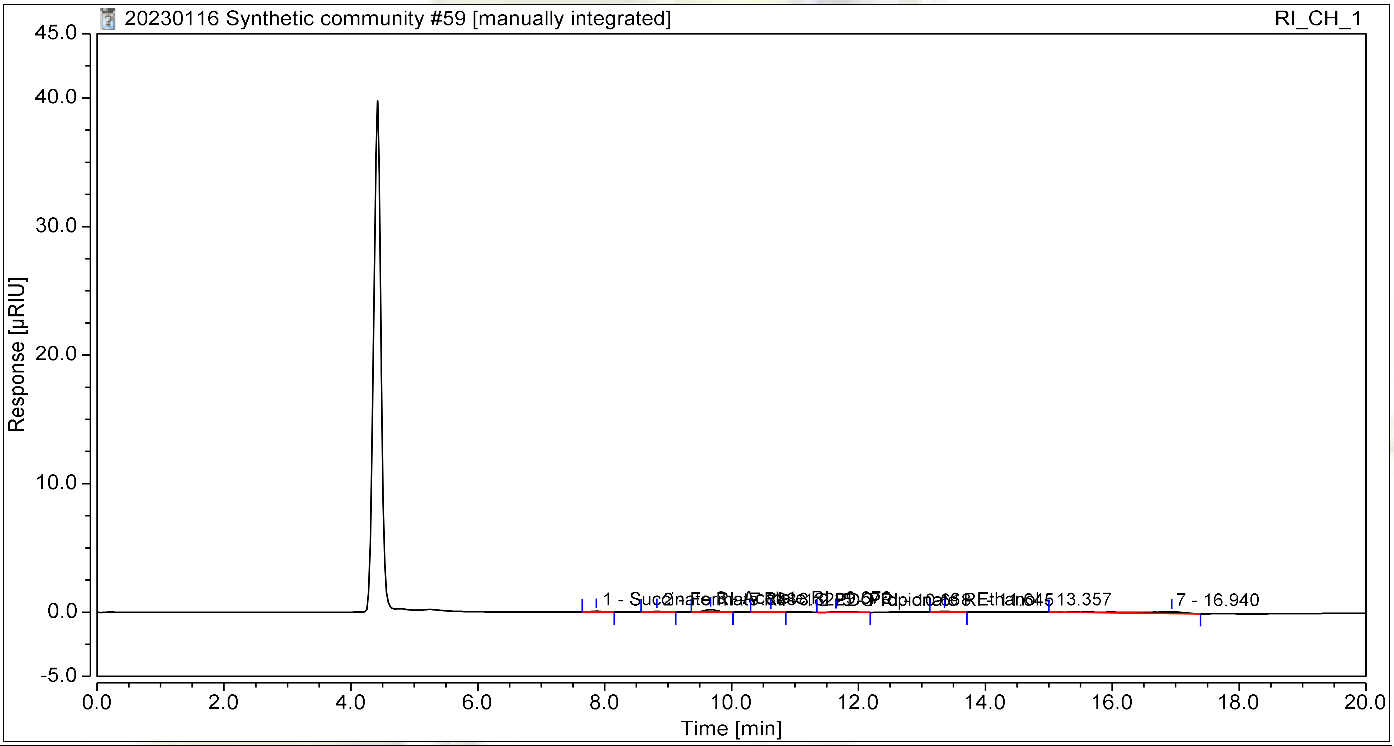

Integration Results

No.	Peak Name	Retention Time min	Area µRIU*min	Height µRIU	Relative Area %	Relative Height %	Amount
n.a.	GlcNAc	n.a.	n.a.	n.a.	n.a.	n.a.	n.a.
n.a.	Citrate	n.a.	n.a.	n.a.	n.a.	n.a.	n.a.
n.a.	Glucose	n.a.	n.a.	n.a.	n.a.	n.a.	n.a.
n.a.	Galactose	n.a.	n.a.	n.a.	n.a.	n.a.	n.a.
n.a.	Fucose	n.a.	n.a.	n.a.	n.a.	n.a.	n.a.
1	Succinate RI	7,868	0,013	0,062	5,47	11,13	0,2642
n.a.	Lactate RI	n.a.	n.a.	n.a.	n.a.	n.a.	n.a.
n.a.	glycerol	n.a.	n.a.	n.a.	n.a.	n.a.	n.a.
2	Formate RI	8,822	0,012	0,058	5,12	10,40	1,2247
3	Acetate RI	9,670	0,046	0,196	19,63	35,10	2,8460
4	1,2 PDO RI	10,618	0,003	0,013	1,17	2,31	0,0833
n.a.	1,3-PDO	n.a.	n.a.	n.a.	n.a.	n.a.	n.a.
5	Propionate RI	11,645	0,019	0,057	8,27	10,24	0,8089
n.a.	1,3-PDO	n.a.	n.a.	n.a.	n.a.	n.a.	n.a.
n.a.	2-3 BDO	n.a.	n.a.	n.a.	n.a.	n.a.	n.a.
6	Ethanol	13,357	0,016	0,059	6,76	10,51	1,6579
n.a.	Isobutyrate RI	n.a.	n.a.	n.a.	n.a.	n.a.	n.a.
n.a.	Butyrate RI	n.a.	n.a.	n.a.	n.a.	n.a.	n.a.
7		16,940	0,125	0,114	53,57	20,31	n.a.
**Total:**			**0,234**	**0,559**	**100,00**	**100,00**	

Chromeleon (c) Dionex
Version 7.2.10.25868

Peak Analysis

Injection Details

Injection Name:	**MUC t96 r2**	*Run Time (min):*	**20,00**
Vial Number:	**3:53**	*Injection Volume:*	**10,00**
Injection Type:	**Unknown**	*Channel:*	**RI_CH_1**
Calibration Level:		*Wavelength:*	**n.a.**
Instrument Method:	**Default method LC2030C 45 gr 20 min**	*Bandwidth:*	**n.a.**
Processing Method:	**Processing Method LC2030 45 gr**	*Dilution Factor:*	**1,0000**
Injection Date/Time:	**17/Jan/23 11:29**	*Sample Weight:*	**1,0000**

Chromatogram

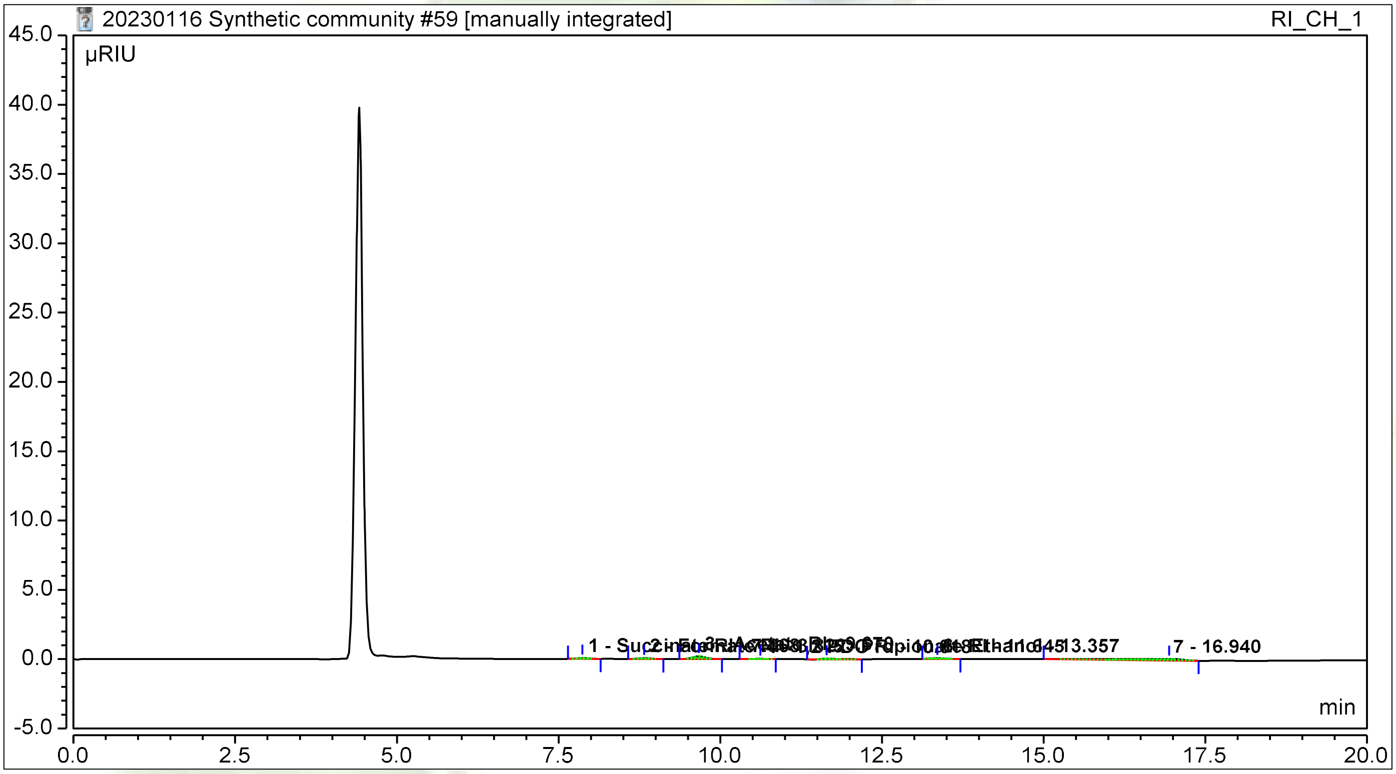

Peak Results

No.	Peak Name	Retention Time min	Width (50%) min	Type	Resolution (EP)	Asymmetry (EP)	Plates (EP)
n.a.	GlcNAc	n.a.	n.a.	n.a.	n.a.	n.a.	n.a.
n.a.	Citrate	n.a.	n.a.	n.a.	n.a.	n.a.	n.a.
n.a.	Glucose	n.a.	n.a.	n.a.	n.a.	n.a.	n.a.
n.a.	Galactose	n.a.	n.a.	n.a.	n.a.	n.a.	n.a.
n.a.	Fucose	n.a.	n.a.	n.a.	n.a.	n.a.	n.a.
1	Succinate RI	7,868	0,196	BMB*	2,89	1,03	8938
n.a.	Lactate RI	n.a.	n.a.	n.a.	n.a.	n.a.	n.a.
n.a.	glycerol	n.a.	n.a.	n.a.	n.a.	n.a.	n.a.
2	Formate RI	8,822	0,194	BMB*	2,41	1,03	11505
3	Acetate RI	9,670	0,221	BMB*	2,66	1,05	10590
4	1,2 PDO RI	10,618	0,199	BMB*	2,22	1,04	15760
n.a.	1,3-PDO	n.a.	n.a.	n.a.	n.a.	n.a.	n.a.
5	Propionate RI	11,645	0,346	BMB*	3,31	2,21	6270
n.a.	1,3-PDO	n.a.	n.a.	n.a.	n.a.	n.a.	n.a.
n.a.	2-3 BDO	n.a.	n.a.	n.a.	n.a.	n.a.	n.a.
6	Ethanol	13,357	0,265	BMB*	3,16	1,10	14097
n.a.	Isobutyrate RI	n.a.	n.a.	n.a.	n.a.	n.a.	n.a.
n.a.	Butyrate RI	n.a.	n.a.	n.a.	n.a.	n.a.	n.a.

7		16,940	1,074	BMB	n.a.	0,61	1378

Chromatogram and SST Results

Injection Details

Injection Name:	**MUC t96 r2**	*Run Time (min):*	**20,00**
Vial Number:	**3:53**	*Injection Volume:*	**10,00**
Injection Type:	**Unknown**	*Channel:*	**RI_CH_1**
Calibration Level:		*Wavelength:*	**n.a.**
Instrument Method:	**Default method LC2030C 45 gr 20 min**	*Bandwidth:*	**n.a.**
Processing Method:	**Processing Method LC2030 45 gr**	*Dilution Factor:*	**1,0000**
Injection Date/Time:	**17/Jan/23 11:29**	*Sample Weight:*	**1,0000**

Chromatogram

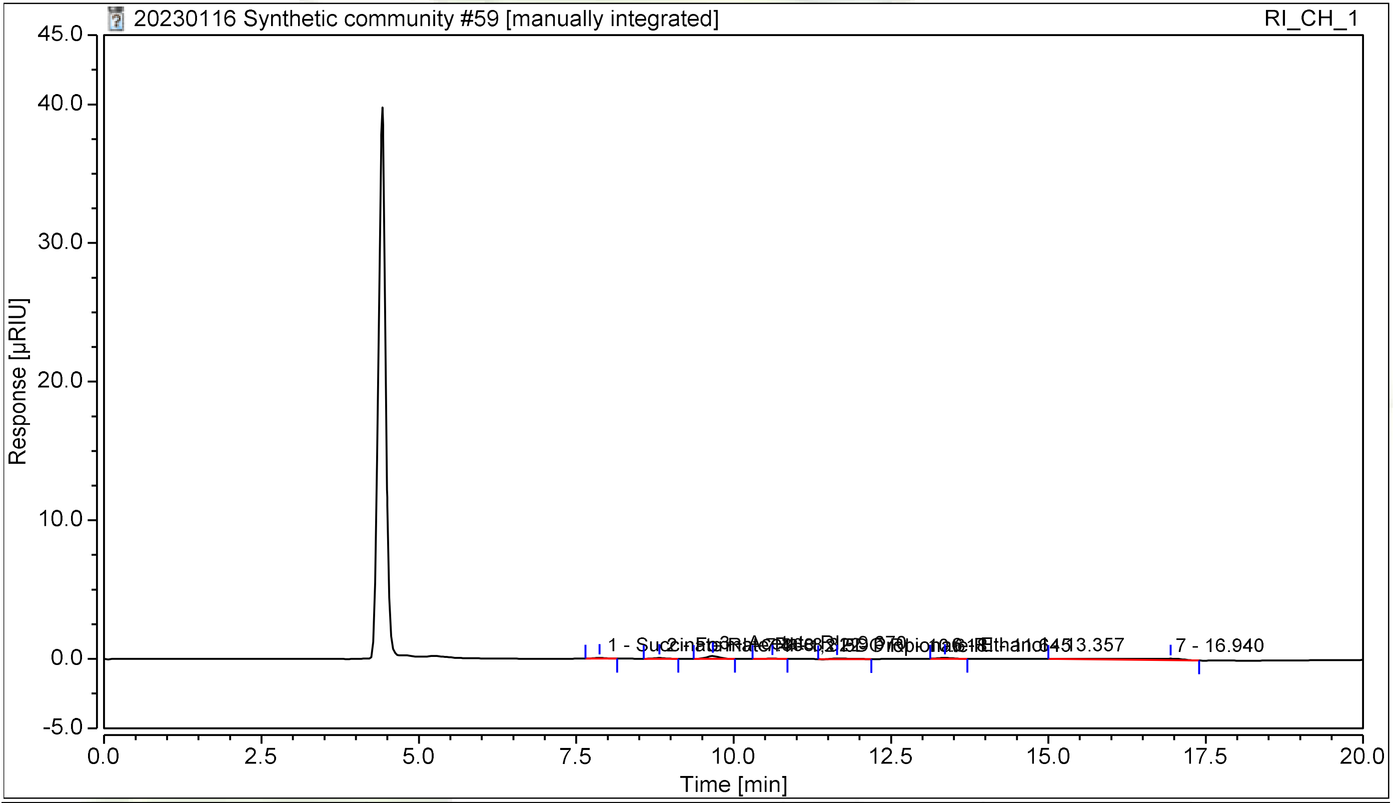

SST Results

No.	Name	Inj.Condition	Peak	Test Result	Injection
**Number of executed test cases:**	**n.a.**		**Total Result:**	**Passed**	

Chromatogram and Results

Injection Details

Injection Name:	**MUC t96 r3**	*Run Time (min):*	**20,00**
Vial Number:	**3:54**	*Injection Volume:*	**10,00**
Injection Type:	**Unknown**	*Channel:*	**RI_CH_1**
Calibration Level:		*Wavelength:*	**n.a.**
Instrument Method:	**Default method LC2030C 45 gr 20 min**	*Bandwidth:*	**n.a.**
Processing Method:	**Processing Method LC2030 45 gr**	*Dilution Factor:*	**1,0000**
Injection Date/Time:	**17/Jan/23 11:49**	*Sample Weight:*	**1,0000**

Chromatogram

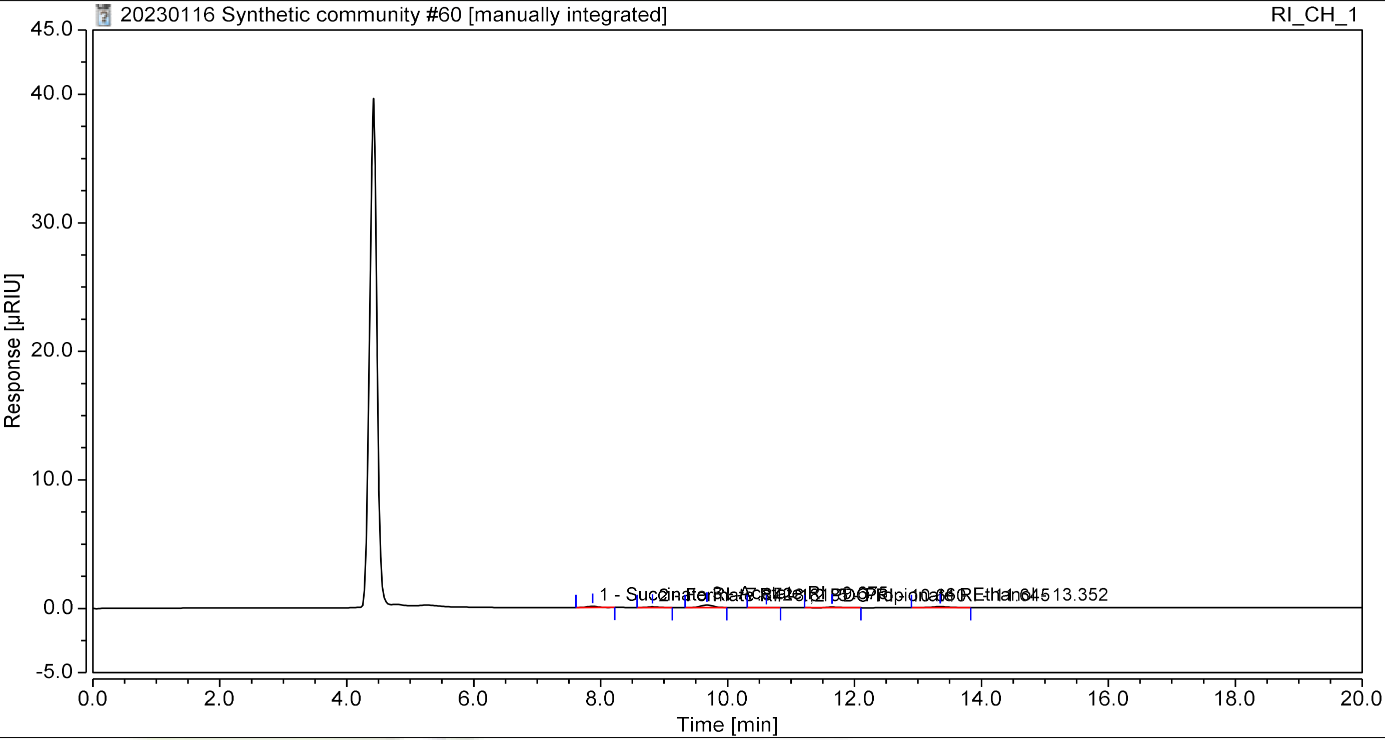

Integration Results

No.	Peak Name	Retention Time min	Area µRIU*min	Height µRIU	Relative Area %	Relative Height %	Amount
n.a.	GlcNAc	n.a.	n.a.	n.a.	n.a.	n.a.	n.a.
n.a.	Citrate	n.a.	n.a.	n.a.	n.a.	n.a.	n.a.
n.a.	Glucose	n.a.	n.a.	n.a.	n.a.	n.a.	n.a.
n.a.	Galactose	n.a.	n.a.	n.a.	n.a.	n.a.	n.a.
n.a.	Fucose	n.a.	n.a.	n.a.	n.a.	n.a.	n.a.
1	Succinate RI	7,872	0,017	0,086	16,31	18,74	0,3490
n.a.	Lactate RI	n.a.	n.a.	n.a.	n.a.	n.a.	n.a.
n.a.	glycerol	n.a.	n.a.	n.a.	n.a.	n.a.	n.a.
2	Formate RI	8,818	0,012	0,057	11,27	12,32	1,1954
3	Acetate RI	9,675	0,046	0,198	44,32	43,06	2,8490
4	1,2 PDO RI	10,610	0,001	0,006	1,31	1,34	0,0414
n.a.	1,3-PDO	n.a.	n.a.	n.a.	n.a.	n.a.	n.a.
5	Propionate RI	11,645	0,008	0,045	7,29	9,70	0,3160
n.a.	1,3-PDO	n.a.	n.a.	n.a.	n.a.	n.a.	n.a.
n.a.	2-3 BDO	n.a.	n.a.	n.a.	n.a.	n.a.	n.a.
6	Ethanol	13,352	0,020	0,068	19,50	14,84	2,1201
n.a.	Isobutyrate RI	n.a.	n.a.	n.a.	n.a.	n.a.	n.a.
n.a.	Butyrate RI	n.a.	n.a.	n.a.	n.a.	n.a.	n.a.
**Total:**			**0,104**	**0,461**	**100,00**	**100,00**	

Chromeleon (c) Dionex
Version 7.2.10.25868

Peak Analysis

Injection Details

Injection Name:	**MUC t96 r3**	*Run Time (min):*	**20,00**
Vial Number:	**3:54**	*Injection Volume:*	**10,00**
Injection Type:	**Unknown**	*Channel:*	**RI_CH_1**
Calibration Level:		*Wavelength:*	**n.a.**
Instrument Method:	**Default method LC2030C 45 gr 20 min**	*Bandwidth:*	**n.a.**
Processing Method:	**Processing Method LC2030 45 gr**	*Dilution Factor:*	**1,0000**
Injection Date/Time:	**17/Jan/23 11:49**	*Sample Weight:*	**1,0000**

Chromatogram

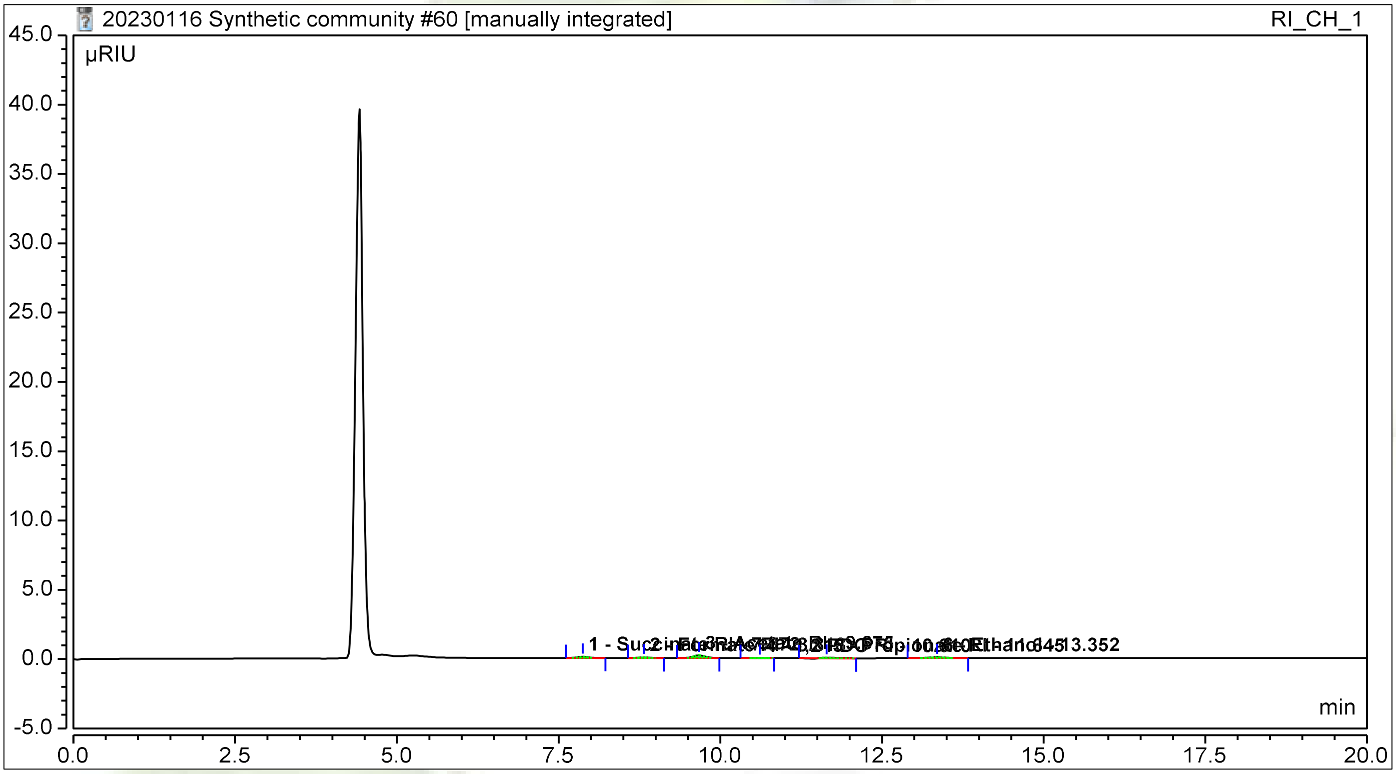

Peak Results

No.	Peak Name	Retention Time min	Width (50%) min	Type	Resolution (EP)	Asymmetry (EP)	Plates (EP)
n.a.	GlcNAc	n.a.	n.a.	n.a.	n.a.	n.a.	n.a.
n.a.	Citrate	n.a.	n.a.	n.a.	n.a.	n.a.	n.a.
n.a.	Glucose	n.a.	n.a.	n.a.	n.a.	n.a.	n.a.
n.a.	Galactose	n.a.	n.a.	n.a.	n.a.	n.a.	n.a.
n.a.	Fucose	n.a.	n.a.	n.a.	n.a.	n.a.	n.a.
1	Succinate RI	7,872	0,193	BMB*	2,89	0,99	9193
n.a.	Lactate RI	n.a.	n.a.	n.a.	n.a.	n.a.	n.a.
n.a.	glycerol	n.a.	n.a.	n.a.	n.a.	n.a.	n.a.
2	Formate RI	8,818	0,193	BMB*	2,45	1,07	11539
3	Acetate RI	9,675	0,220	BMB*	2,58	1,04	10714
4	1,2 PDO RI	10,610	0,207	BMB*	2,85	1,12	14534
n.a.	1,3-PDO	n.a.	n.a.	n.a.	n.a.	n.a.	n.a.
5	Propionate RI	11,645	0,222	BMB*	3,96	2,34	15243
n.a.	1,3-PDO	n.a.	n.a.	n.a.	n.a.	n.a.	n.a.
n.a.	2-3 BDO	n.a.	n.a.	n.a.	n.a.	n.a.	n.a.
6	Ethanol	13,352	0,286	BMB*	n.a.	1,05	12065
n.a.	Isobutyrate RI	n.a.	n.a.	n.a.	n.a.	n.a.	n.a.
n.a.	Butyrate RI	n.a.	n.a.	n.a.	n.a.	n.a.	n.a.

Chromatogram and SST Results

Injection Details

Injection Name:	**MUC t96 r3**	*Run Time (min):*	**20,00**
Vial Number:	**3:54**	*Injection Volume:*	**10,00**
Injection Type:	**Unknown**	*Channel:*	**RI_CH_1**
Calibration Level:		*Wavelength:*	**n.a.**
Instrument Method:	**Default method LC2030C 45 gr 20 min**	*Bandwidth:*	**n.a.**
Processing Method:	**Processing Method LC2030 45 gr**	*Dilution Factor:*	**1,0000**
Injection Date/Time:	**17/Jan/23 11:49**	*Sample Weight:*	**1,0000**

Chromatogram

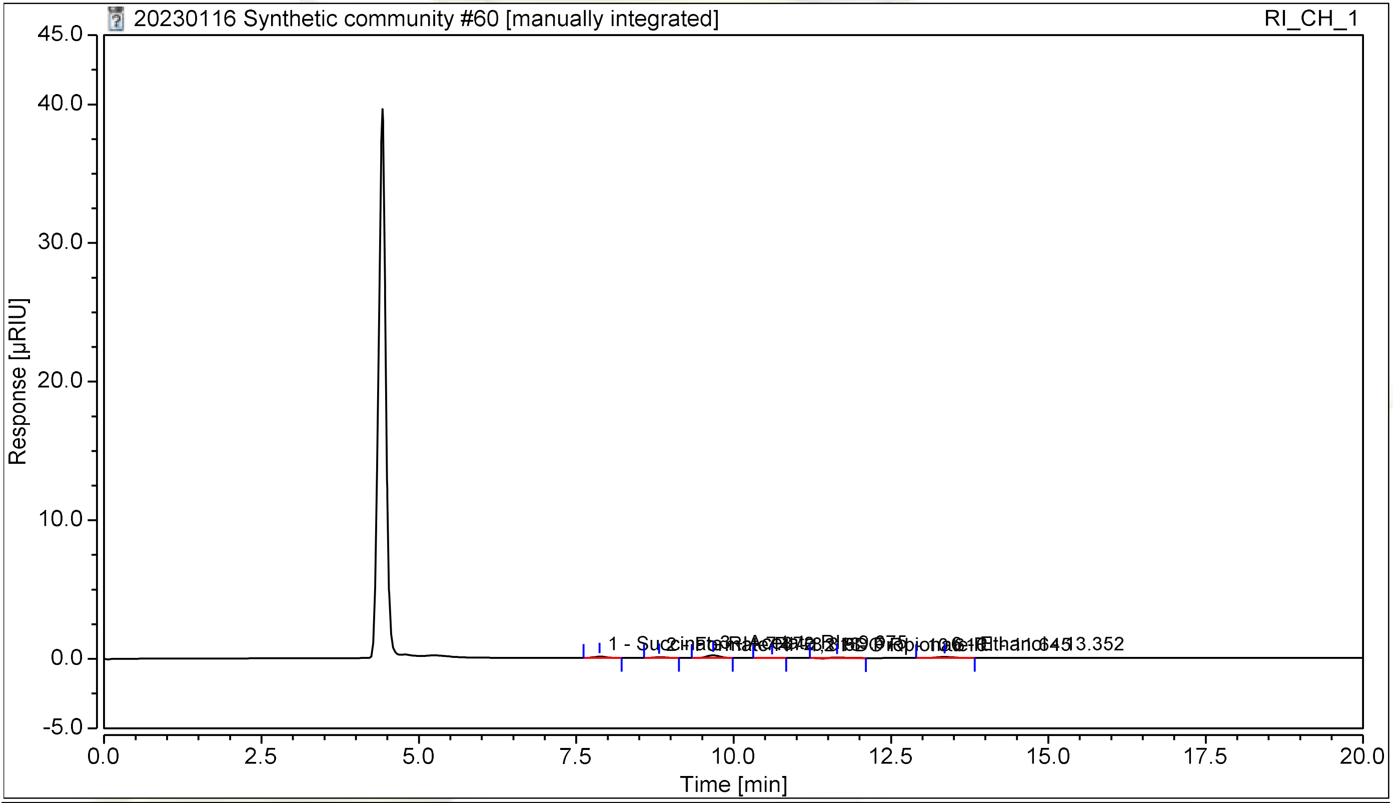

SST Results

No.	Name	Inj.Condition	Peak	Test Result	Injection

Number of executed test cases: n.a. **Total Result: Passed**

Chromatogram and Results

Injection Details

Injection Name:	**MUC t120 r1**	*Run Time (min):*	**20,00**
Vial Number:	**3:55**	*Injection Volume:*	**10,00**
Injection Type:	**Unknown**	*Channel:*	**RI_CH_1**
Calibration Level:		*Wavelength:*	**n.a.**
Instrument Method:	**Default method LC2030C 45 gr 20 min**	*Bandwidth:*	**n.a.**
Processing Method:	**Processing Method LC2030 45 gr**	*Dilution Factor:*	**1,0000**
Injection Date/Time:	**17/Jan/23 12:10**	*Sample Weight:*	**1,0000**

Chromatogram

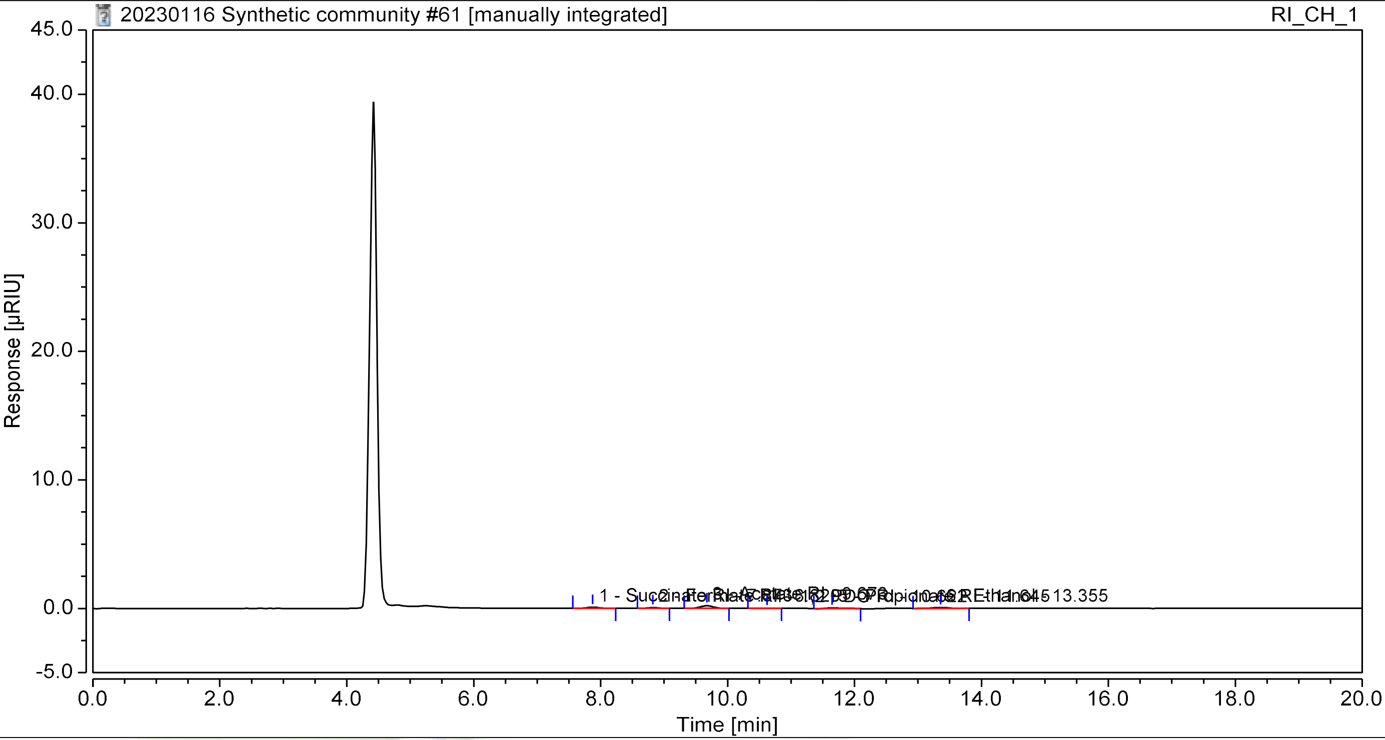

Integration Results

No.	Peak Name	Retention Time min	Area µRIU*min	Height µRIU	Relative Area %	Relative Height %	Amount
n.a.	GlcNAc	n.a.	n.a.	n.a.	n.a.	n.a.	n.a.
n.a.	Citrate	n.a.	n.a.	n.a.	n.a.	n.a.	n.a.
n.a.	Glucose	n.a.	n.a.	n.a.	n.a.	n.a.	n.a.
n.a.	Galactose	n.a.	n.a.	n.a.	n.a.	n.a.	n.a.
n.a.	Fucose	n.a.	n.a.	n.a.	n.a.	n.a.	n.a.
1	Succinate RI	7,873	0,016	0,079	13,23	15,97	0,3272
n.a.	Lactate RI	n.a.	n.a.	n.a.	n.a.	n.a.	n.a.
n.a.	glycerol	n.a.	n.a.	n.a.	n.a.	n.a.	n.a.
2	Formate RI	8,820	0,012	0,057	9,72	11,52	1,1919
3	Acetate RI	9,673	0,047	0,207	39,55	41,61	2,9377
4	1,2 PDO RI	10,622	0,004	0,018	3,48	3,61	0,1269
n.a.	1,3-PDO	n.a.	n.a.	n.a.	n.a.	n.a.	n.a.
5	Propionate RI	11,645	0,019	0,064	15,92	12,91	0,7975
n.a.	1,3-PDO	n.a.	n.a.	n.a.	n.a.	n.a.	n.a.
n.a.	2-3 BDO	n.a.	n.a.	n.a.	n.a.	n.a.	n.a.
6	Ethanol	13,355	0,022	0,071	18,10	14,38	2,2746
n.a.	Isobutyrate RI	n.a.	n.a.	n.a.	n.a.	n.a.	n.a.
n.a.	Butyrate RI	n.a.	n.a.	n.a.	n.a.	n.a.	n.a.
**Total:**			**0,120**	**0,497**	**100,00**	**100,00**	

Peak Analysis

Injection Details

Injection Name:	**MUC t120 r1**	*Run Time (min):*	**20,00**
Vial Number:	**3:55**	*Injection Volume:*	**10,00**
Injection Type:	**Unknown**	*Channel:*	**RI_CH_1**
Calibration Level:		*Wavelength:*	**n.a.**
Instrument Method:	**Default method LC2030C 45 gr 20 min**	*Bandwidth:*	**n.a.**
Processing Method:	**Processing Method LC2030 45 gr**	*Dilution Factor:*	**1,0000**
Injection Date/Time:	**17/Jan/23 12:10**	*Sample Weight:*	**1,0000**

Chromatogram

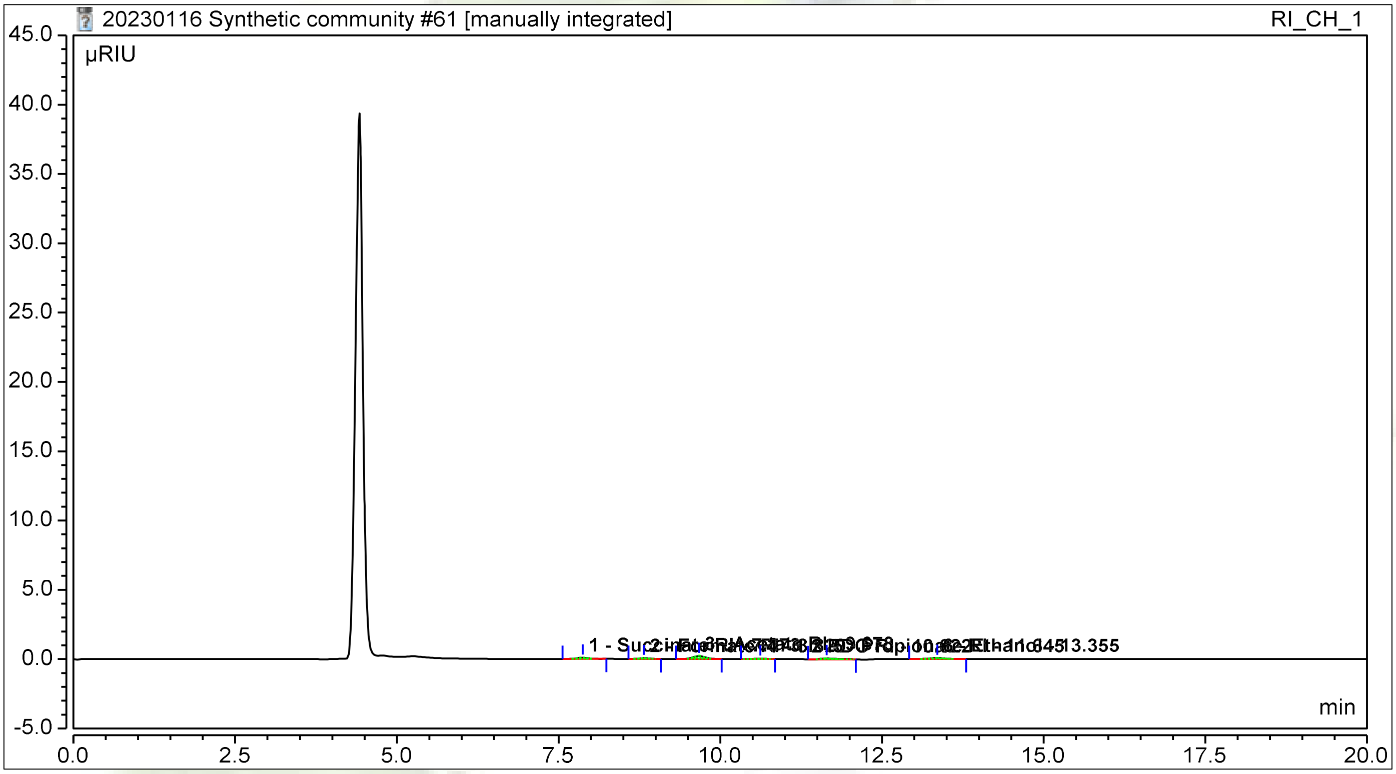

Peak Results

No.	Peak Name	Retention Time min	Width (50%) min	Type	Resolution (EP)	Asymmetry (EP)	Plates (EP)
n.a.	GlcNAc	n.a.	n.a.	n.a.	n.a.	n.a.	n.a.
n.a.	Citrate	n.a.	n.a.	n.a.	n.a.	n.a.	n.a.
n.a.	Glucose	n.a.	n.a.	n.a.	n.a.	n.a.	n.a.
n.a.	Galactose	n.a.	n.a.	n.a.	n.a.	n.a.	n.a.
n.a.	Fucose	n.a.	n.a.	n.a.	n.a.	n.a.	n.a.
1	Succinate RI	7,873	0,196	BMB*	2,88	0,97	8940
n.a.	Lactate RI	n.a.	n.a.	n.a.	n.a.	n.a.	n.a.
n.a.	glycerol	n.a.	n.a.	n.a.	n.a.	n.a.	n.a.
2	Formate RI	8,820	0,192	BMB*	2,46	1,06	11635
3	Acetate RI	9,673	0,217	BMB*	2,55	1,03	10975
4	1,2 PDO RI	10,622	0,222	BMB*	2,53	0,95	12715
n.a.	1,3-PDO	n.a.	n.a.	n.a.	n.a.	n.a.	n.a.
5	Propionate RI	11,645	0,256	BMB*	3,70	1,81	11438
n.a.	1,3-PDO	n.a.	n.a.	n.a.	n.a.	n.a.	n.a.
n.a.	2-3 BDO	n.a.	n.a.	n.a.	n.a.	n.a.	n.a.
6	Ethanol	13,355	0,289	BMB*	n.a.	1,02	11856
n.a.	Isobutyrate RI	n.a.	n.a.	n.a.	n.a.	n.a.	n.a.
n.a.	Butyrate RI	n.a.	n.a.	n.a.	n.a.	n.a.	n.a.

Chromatogram and SST Results

Injection Details

Injection Name:	**MUC t120 r1**	*Run Time (min):*	**20,00**
Vial Number:	**3:55**	*Injection Volume:*	**10,00**
Injection Type:	**Unknown**	*Channel:*	**RI_CH_1**
Calibration Level:		*Wavelength:*	**n.a.**
Instrument Method:	**Default method LC2030C 45 gr 20 min**	*Bandwidth:*	**n.a.**
Processing Method:	**Processing Method LC2030 45 gr**	*Dilution Factor:*	**1,0000**
Injection Date/Time:	**17/Jan/23 12:10**	*Sample Weight:*	**1,0000**

Chromatogram

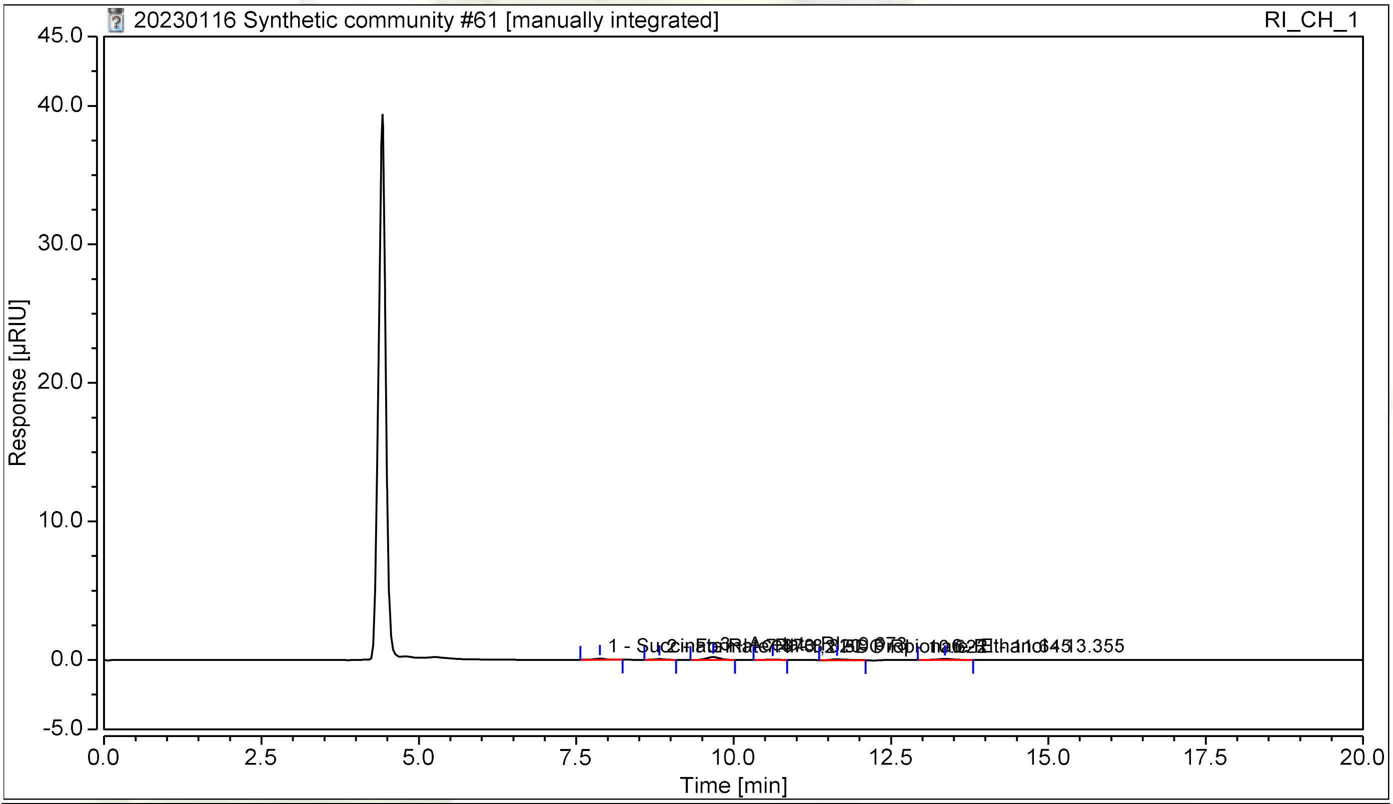

SST Results

No.	Name	Inj.Condition	Peak	Test Result	Injection
**Number of executed test cases:**	**n.a.**		**Total Result:**	**Passed**	

Chromatogram and Results

Injection Details

Injection Name:	**MUC t120 r2**	*Run Time (min):*	**20,00**
Vial Number:	**3:56**	*Injection Volume:*	**10,00**
Injection Type:	**Unknown**	*Channel:*	**RI_CH_1**
Calibration Level:		*Wavelength:*	**n.a.**
Instrument Method:	**Default method LC2030C 45 gr 20 min**	*Bandwidth:*	**n.a.**
Processing Method:	**Processing Method LC2030 45 gr**	*Dilution Factor:*	**1,0000**
Injection Date/Time:	**17/Jan/23 12:30**	*Sample Weight:*	**1,0000**

Chromatogram

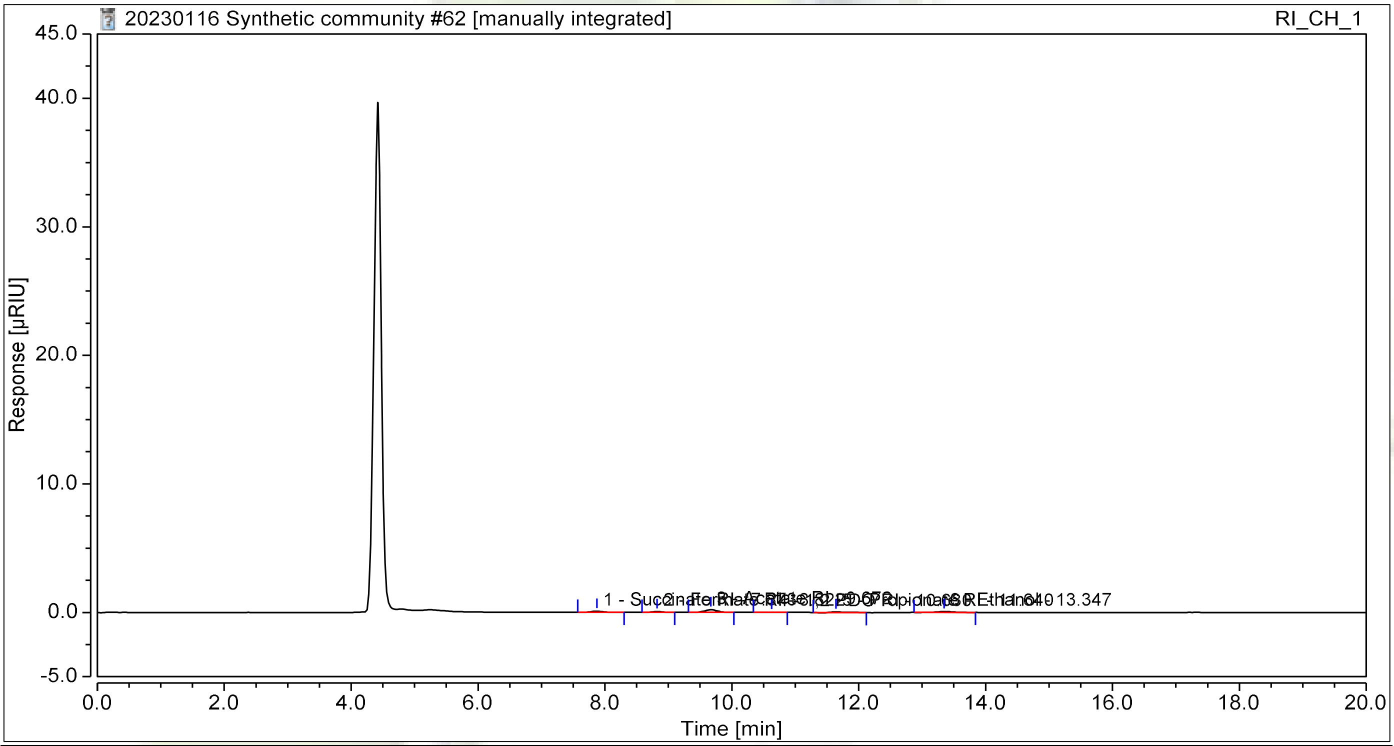

Integration Results

No.	Peak Name	Retention Time min	Area µRIU*min	Height µRIU	Relative Area %	Relative Height %	Amount
n.a.	GlcNAc	n.a.	n.a.	n.a.	n.a.	n.a.	n.a.
n.a.	Citrate	n.a.	n.a.	n.a.	n.a.	n.a.	n.a.
n.a.	Glucose	n.a.	n.a.	n.a.	n.a.	n.a.	n.a.
n.a.	Galactose	n.a.	n.a.	n.a.	n.a.	n.a.	n.a.
n.a.	Fucose	n.a.	n.a.	n.a.	n.a.	n.a.	n.a.
1	Succinate RI	7,873	0,015	0,079	14,61	17,22	0,3177
n.a.	Lactate RI	n.a.	n.a.	n.a.	n.a.	n.a.	n.a.
n.a.	glycerol	n.a.	n.a.	n.a.	n.a.	n.a.	n.a.
2	Formate RI	8,822	0,011	0,057	10,84	12,36	1,1688
3	Acetate RI	9,672	0,046	0,201	44,11	43,77	2,8827
4	1,2 PDO RI	10,630	0,003	0,012	2,75	2,52	0,0882
n.a.	1,3-PDO	n.a.	n.a.	n.a.	n.a.	n.a.	n.a.
5	Propionate RI	11,640	0,010	0,047	9,80	10,17	0,4318
n.a.	1,3-PDO	n.a.	n.a.	n.a.	n.a.	n.a.	n.a.
n.a.	2-3 BDO	n.a.	n.a.	n.a.	n.a.	n.a.	n.a.
6	Ethanol	13,347	0,019	0,064	17,89	13,95	1,9780
n.a.	Isobutyrate RI	n.a.	n.a.	n.a.	n.a.	n.a.	n.a.
n.a.	Butyrate RI	n.a.	n.a.	n.a.	n.a.	n.a.	n.a.
**Total:**			**0,105**	**0,459**	**100,00**	**100,00**	

Chromeleon (c) Dionex
Version 7.2.10.25868

Peak Analysis

Injection Details

Injection Name:	**MUC t120 r2**	*Run Time (min):*	**20,00**
Vial Number:	**3:56**	*Injection Volume:*	**10,00**
Injection Type:	**Unknown**	*Channel:*	**RI_CH_1**
Calibration Level:		*Wavelength:*	**n.a.**
Instrument Method:	**Default method LC2030C 45 gr 20 min**	*Bandwidth:*	**n.a.**
Processing Method:	**Processing Method LC2030 45 gr**	*Dilution Factor:*	**1,0000**
Injection Date/Time:	**17/Jan/23 12:30**	*Sample Weight:*	**1,0000**

Chromatogram

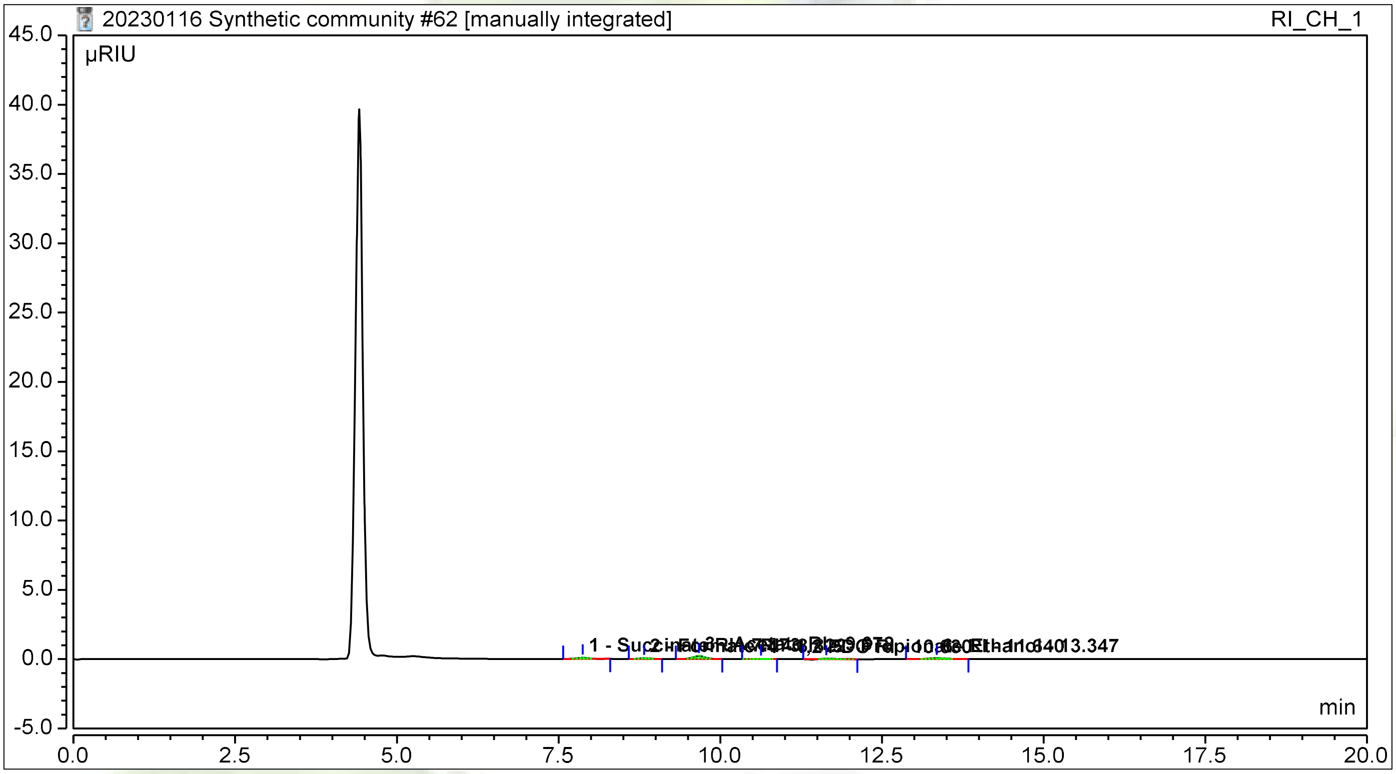

Peak Results

No.	Peak Name	Retention Time min	Width (50%) min	Type	Resolution (EP)	Asymmetry (EP)	Plates (EP)
n.a.	GlcNAc	n.a.	n.a.	n.a.	n.a.	n.a.	n.a.
n.a.	Citrate	n.a.	n.a.	n.a.	n.a.	n.a.	n.a.
n.a.	Glucose	n.a.	n.a.	n.a.	n.a.	n.a.	n.a.
n.a.	Galactose	n.a.	n.a.	n.a.	n.a.	n.a.	n.a.
n.a.	Fucose	n.a.	n.a.	n.a.	n.a.	n.a.	n.a.
1	Succinate RI	7,873	0,193	BMB*	2,90	0,97	9233
n.a.	Lactate RI	n.a.	n.a.	n.a.	n.a.	n.a.	n.a.
n.a.	glycerol	n.a.	n.a.	n.a.	n.a.	n.a.	n.a.
2	Formate RI	8,822	0,194	BMB*	2,43	1,05	11501
3	Acetate RI	9,672	0,219	BMB*	2,38	1,03	10801
4	1,2 PDO RI	10,630	0,257	BMB*	2,41	0,90	9484
n.a.	1,3-PDO	n.a.	n.a.	n.a.	n.a.	n.a.	n.a.
5	Propionate RI	11,640	0,237	BMB*	3,87	2,41	13386
n.a.	1,3-PDO	n.a.	n.a.	n.a.	n.a.	n.a.	n.a.
n.a.	2-3 BDO	n.a.	n.a.	n.a.	n.a.	n.a.	n.a.
6	Ethanol	13,347	0,284	BMB*	n.a.	1,05	12224
n.a.	Isobutyrate RI	n.a.	n.a.	n.a.	n.a.	n.a.	n.a.
n.a.	Butyrate RI	n.a.	n.a.	n.a.	n.a.	n.a.	n.a.

Chromatogram and SST Results

Injection Details

Injection Name:	**MUC t120 r2**	*Run Time (min):*	**20,00**
Vial Number:	**3:56**	*Injection Volume:*	**10,00**
Injection Type:	**Unknown**	*Channel:*	**RI_CH_1**
Calibration Level:		*Wavelength:*	**n.a.**
Instrument Method:	**Default method LC2030C 45 gr 20 min**	*Bandwidth:*	**n.a.**
Processing Method:	**Processing Method LC2030 45 gr**	*Dilution Factor:*	**1,0000**
Injection Date/Time:	**17/Jan/23 12:30**	*Sample Weight:*	**1,0000**

Chromatogram

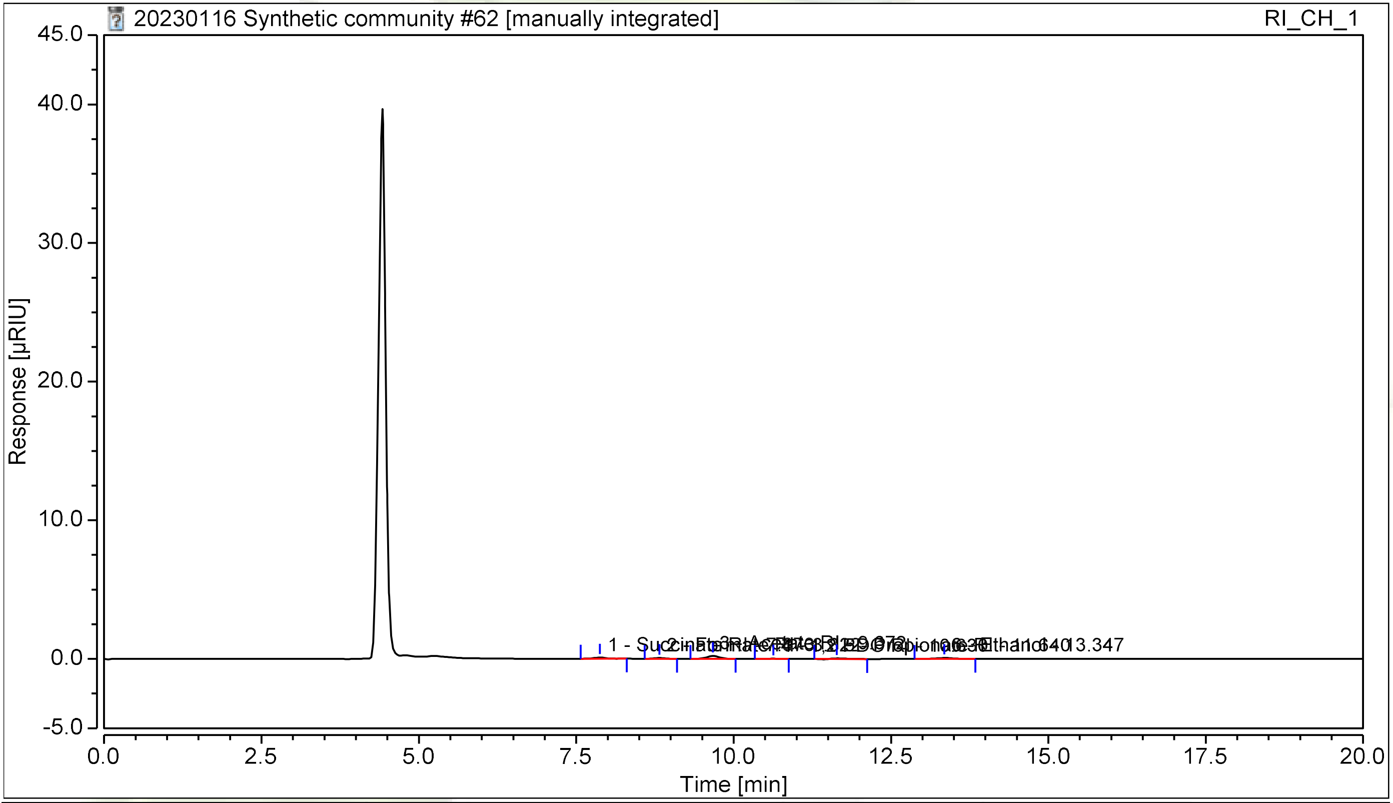

SST Results

No.	Name	Inj.Condition	Peak	Test Result	Injection
**Number of executed test cases:**	**n.a.**		**Total Result:**	**Passed**	

Chromeleon (c) Dionex
Version 7.2.10.25868

Chromatogram and Results

Injection Details

Injection Name:	**MUC t120 r3**	*Run Time (min):*	**20,00**
Vial Number:	**3:57**	*Injection Volume:*	**10,00**
Injection Type:	**Unknown**	*Channel:*	**RI_CH_1**
Calibration Level:		*Wavelength:*	**n.a.**
Instrument Method:	**Default method LC2030C 45 gr 20 min**	*Bandwidth:*	**n.a.**
Processing Method:	**Processing Method LC2030 45 gr**	*Dilution Factor:*	**1,0000**
Injection Date/Time:	**17/Jan/23 12:51**	*Sample Weight:*	**1,0000**

Chromatogram

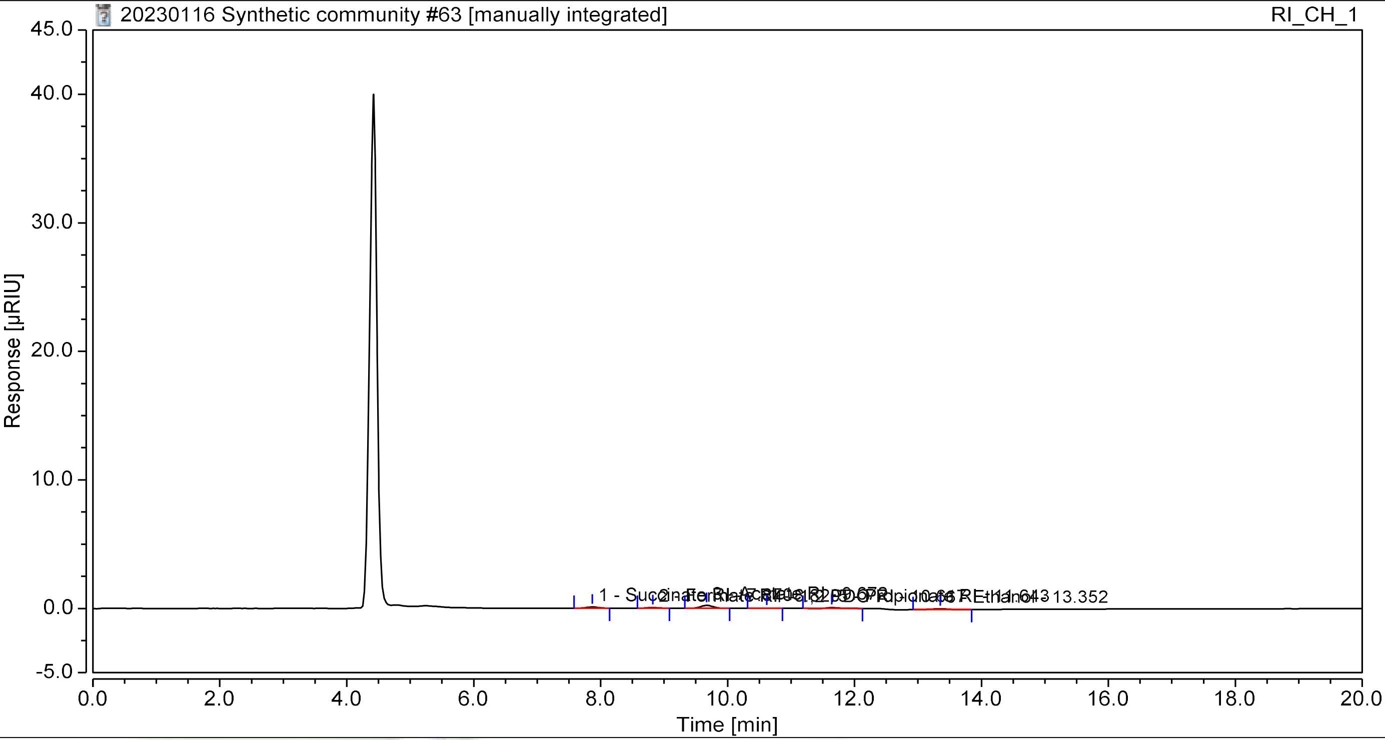

Integration Results

No.	Peak Name	Retention Time min	Area µRIU*min	Height µRIU	Relative Area %	Relative Height %	Amount
n.a.	GlcNAc	n.a.	n.a.	n.a.	n.a.	n.a.	n.a.
n.a.	Citrate	n.a.	n.a.	n.a.	n.a.	n.a.	n.a.
n.a.	Glucose	n.a.	n.a.	n.a.	n.a.	n.a.	n.a.
n.a.	Galactose	n.a.	n.a.	n.a.	n.a.	n.a.	n.a.
n.a.	Fucose	n.a.	n.a.	n.a.	n.a.	n.a.	n.a.
1	Succinate RI	7,870	0,020	0,097	17,79	19,36	0,4193
n.a.	Lactate RI	n.a.	n.a.	n.a.	n.a.	n.a.	n.a.
n.a.	glycerol	n.a.	n.a.	n.a.	n.a.	n.a.	n.a.
2	Formate RI	8,820	0,013	0,062	11,10	12,29	1,2968
3	Acetate RI	9,672	0,052	0,225	45,58	44,91	3,2264
4	1,2 PDO RI	10,617	0,002	0,008	1,71	1,62	0,0594
n.a.	1,3-PDO	n.a.	n.a.	n.a.	n.a.	n.a.	n.a.
5	Propionate RI	11,643	0,011	0,057	9,67	11,37	0,4617
n.a.	1,3-PDO	n.a.	n.a.	n.a.	n.a.	n.a.	n.a.
n.a.	2-3 BDO	n.a.	n.a.	n.a.	n.a.	n.a.	n.a.
6	Ethanol	13,352	0,016	0,052	14,15	10,44	1,6947
n.a.	Isobutyrate RI	n.a.	n.a.	n.a.	n.a.	n.a.	n.a.
n.a.	Butyrate RI	n.a.	n.a.	n.a.	n.a.	n.a.	n.a.
**Total:**			**0,114**	**0,502**	**100,00**	**100,00**	

Peak Analysis

Injection Details

Injection Name:	**MUC t120 r3**	*Run Time (min):*	**20,00**
Vial Number:	**3:57**	*Injection Volume:*	**10,00**
Injection Type:	**Unknown**	*Channel:*	**RI_CH_1**
Calibration Level:		*Wavelength:*	**n.a.**
Instrument Method:	**Default method LC2030C 45 gr 20 min**	*Bandwidth:*	**n.a.**
Processing Method:	**Processing Method LC2030 45 gr**	*Dilution Factor:*	**1,0000**
Injection Date/Time:	**17/Jan/23 12:51**	*Sample Weight:*	**1,0000**

Chromatogram

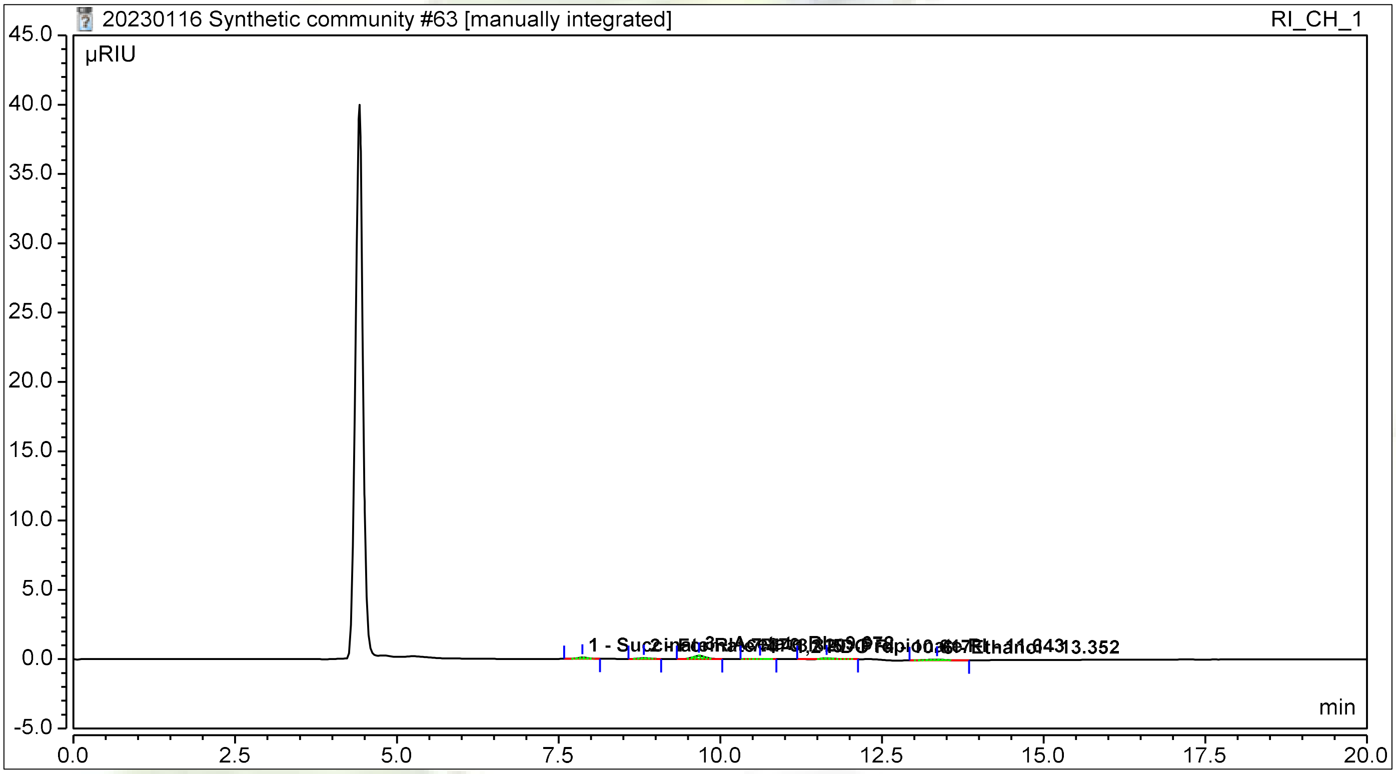

Peak Results

No.	Peak Name	Retention Time min	Width (50%) min	Type	Resolution (EP)	Asymmetry (EP)	Plates (EP)
n.a.	GlcNAc	n.a.	n.a.	n.a.	n.a.	n.a.	n.a.
n.a.	Citrate	n.a.	n.a.	n.a.	n.a.	n.a.	n.a.
n.a.	Glucose	n.a.	n.a.	n.a.	n.a.	n.a.	n.a.
n.a.	Galactose	n.a.	n.a.	n.a.	n.a.	n.a.	n.a.
n.a.	Fucose	n.a.	n.a.	n.a.	n.a.	n.a.	n.a.
1	Succinate RI	7,870	0,199	BMB*	2,85	1,02	8638
n.a.	Lactate RI	n.a.	n.a.	n.a.	n.a.	n.a.	n.a.
n.a.	glycerol	n.a.	n.a.	n.a.	n.a.	n.a.	n.a.
2	Formate RI	8,820	0,194	BMB*	2,44	1,03	11477
3	Acetate RI	9,672	0,219	BMB*	2,39	1,05	10815
4	1,2 PDO RI	10,617	0,248	BMB*	2,63	0,95	10188
n.a.	1,3-PDO	n.a.	n.a.	n.a.	n.a.	n.a.	n.a.
5	Propionate RI	11,643	0,213	BMB*	3,99	2,11	16542
n.a.	1,3-PDO	n.a.	n.a.	n.a.	n.a.	n.a.	n.a.
n.a.	2-3 BDO	n.a.	n.a.	n.a.	n.a.	n.a.	n.a.
6	Ethanol	13,352	0,292	BMB*	n.a.	0,80	11589
n.a.	Isobutyrate RI	n.a.	n.a.	n.a.	n.a.	n.a.	n.a.
n.a.	Butyrate RI	n.a.	n.a.	n.a.	n.a.	n.a.	n.a.

Chromatogram and SST Results

Injection Details

Injection Name:	**MUC t120 r3**	*Run Time (min):*	**20,00**
Vial Number:	**3:57**	*Injection Volume:*	**10,00**
Injection Type:	**Unknown**	*Channel:*	**RI_CH_1**
Calibration Level:		*Wavelength:*	**n.a.**
Instrument Method:	**Default method LC2030C 45 gr 20 min**	*Bandwidth:*	**n.a.**
Processing Method:	**Processing Method LC2030 45 gr**	*Dilution Factor:*	**1,0000**
Injection Date/Time:	**17/Jan/23 12:51**	*Sample Weight:*	**1,0000**

Chromatogram

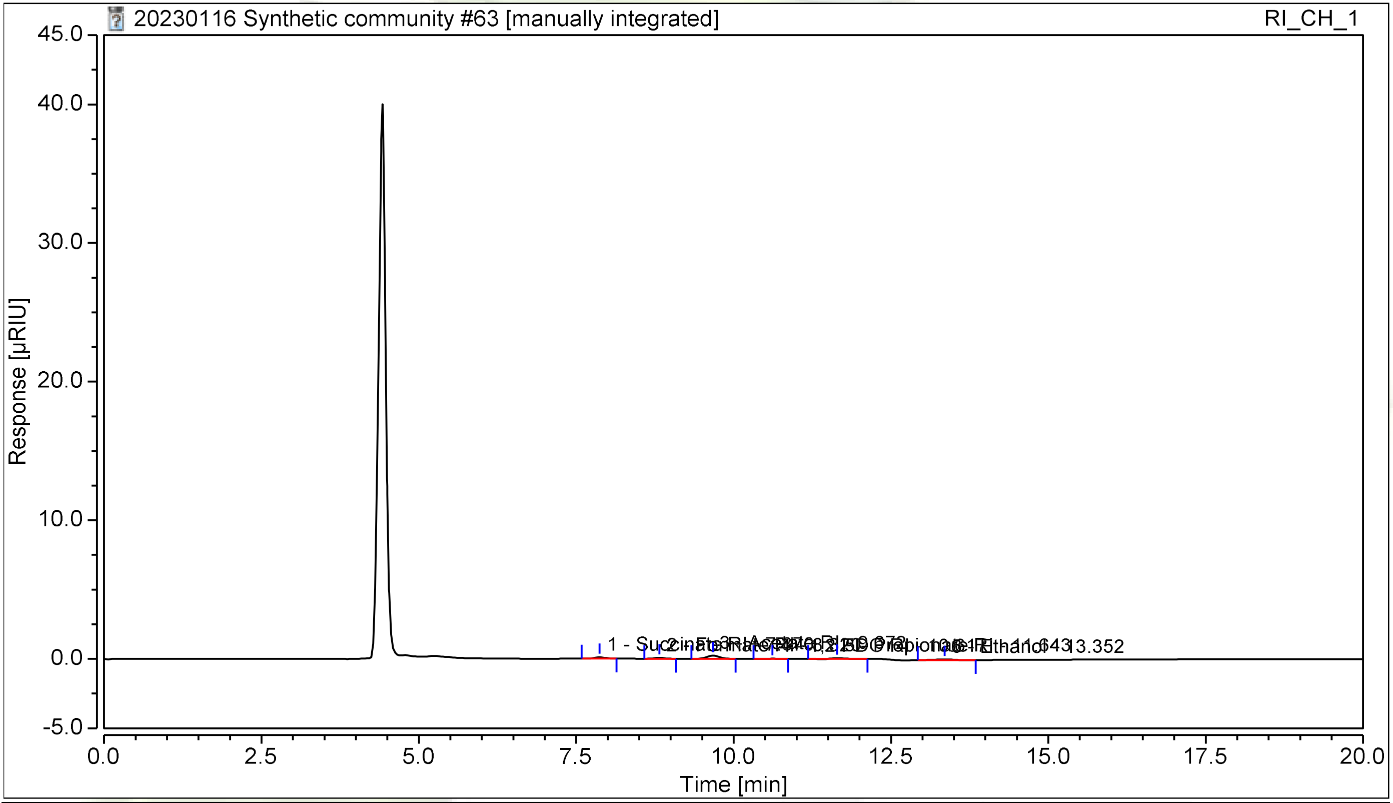

SST Results

No.	Name	Inj.Condition	Peak	Test Result	Injection

Number of executed test cases: **n.a.** **Total Result:** **Passed**

Chromatogram and Results

Injection Details

Injection Name:	**GOSFOSMUC t72 r1**	*Run Time (min):*	**11,31**
Vial Number:	**3:61**	*Injection Volume:*	**10,00**
Injection Type:	**Unknown**	*Channel:*	**RI_CH_1**
Calibration Level:		*Wavelength:*	**n.a.**
Instrument Method:	**Default method LC2030C 45 gr 20 min**	*Bandwidth:*	**n.a.**
Processing Method:	**Processing Method LC2030 45 gr**	*Dilution Factor:*	**1,0000**
Injection Date/Time:	**17/Jan/23 13:11**	*Sample Weight:*	**1,0000**

Chromatogram

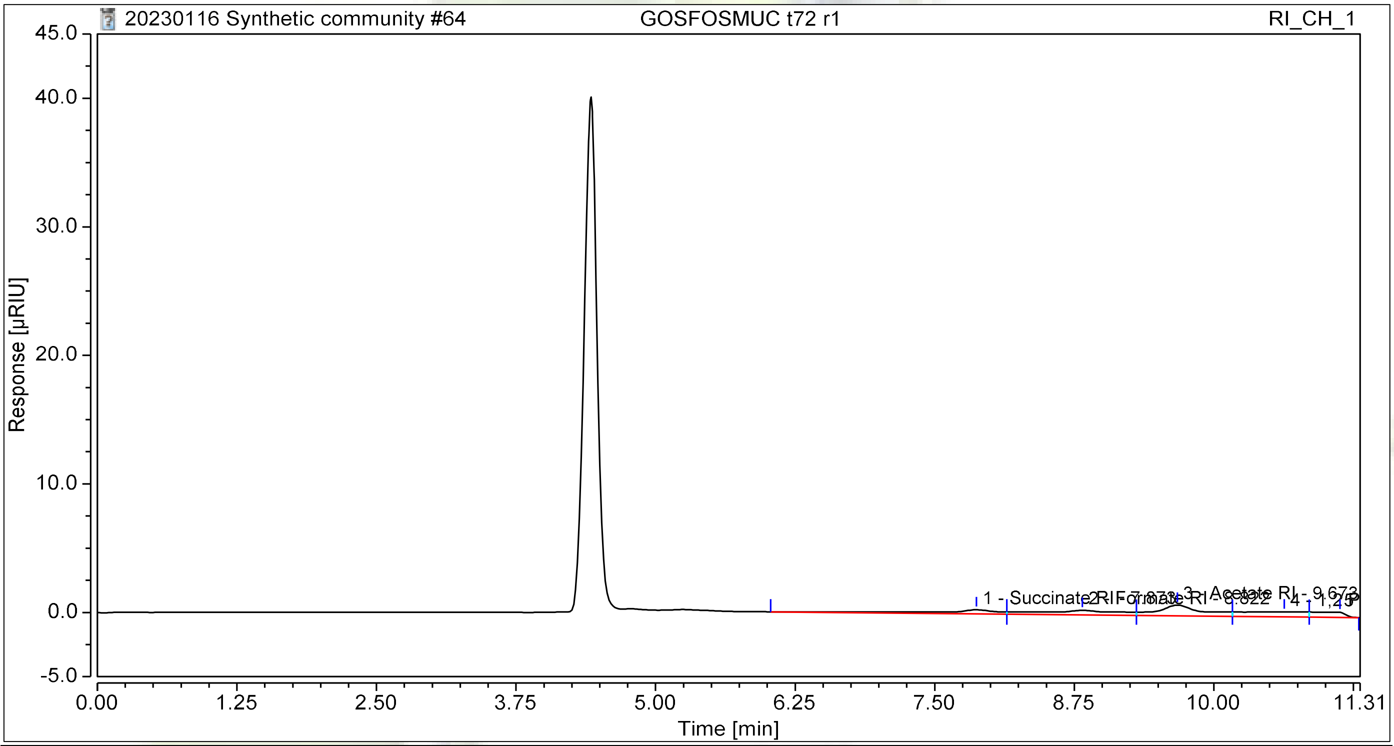

Integration Results

No.	Peak Name	Retention Time min	Area µRIU*min	Height µRIU	Relative Area %	Relative Height %	Amount
n.a.	GlcNAc	n.a.	n.a.	n.a.	n.a.	n.a.	n.a.
n.a.	Citrate	n.a.	n.a.	n.a.	n.a.	n.a.	n.a.
n.a.	Glucose	n.a.	n.a.	n.a.	n.a.	n.a.	n.a.
n.a.	Galactose	n.a.	n.a.	n.a.	n.a.	n.a.	n.a.
n.a.	Fucose	n.a.	n.a.	n.a.	n.a.	n.a.	n.a.
1	Succinate RI	7,873	0,200	0,321	15,85	13,78	4,1211
n.a.	Lactate RI	n.a.	n.a.	n.a.	n.a.	n.a.	n.a.
n.a.	glycerol	n.a.	n.a.	n.a.	n.a.	n.a.	n.a.
2	Formate RI	8,822	0,280	0,360	22,27	15,45	28,7043
3	Acetate RI	9,673	0,385	0,842	30,60	36,14	23,8981
4	1,2 PDO RI	10,630	0,257	0,391	20,37	16,77	7,8086
n.a.	1,3-PDO	n.a.	n.a.	n.a.	n.a.	n.a.	n.a.
5		11,130	0,137	0,416	10,91	17,86	n.a.
n.a.	Propionate RI	n.a.	n.a.	n.a.	n.a.	n.a.	n.a.
n.a.	1,3-PDO	n.a.	n.a.	n.a.	n.a.	n.a.	n.a.
n.a.	2-3 BDO	n.a.	n.a.	n.a.	n.a.	n.a.	n.a.
n.a.	Ethanol	n.a.	n.a.	n.a.	n.a.	n.a.	n.a.
n.a.	Isobutyrate RI	n.a.	n.a.	n.a.	n.a.	n.a.	n.a.
n.a.	Butyrate RI	n.a.	n.a.	n.a.	n.a.	n.a.	n.a.
**Total:**			**1,260**	**2,330**	**100,00**	**100,00**	

Chromeleon (c) Dionex
Version 7.2.10.25868

Peak Analysis

Injection Details

Injection Name:	**GOSFOSMUC t72 r1**	*Run Time (min):*	**11,31**
Vial Number:	**3:61**	*Injection Volume:*	**10,00**
Injection Type:	**Unknown**	*Channel:*	**RI_CH_1**
Calibration Level:		*Wavelength:*	**n.a.**
Instrument Method:	**Default method LC2030C 45 gr 20 min**	*Bandwidth:*	**n.a.**
Processing Method:	**Processing Method LC2030 45 gr**	*Dilution Factor:*	**1,0000**
Injection Date/Time:	**17/Jan/23 13:11**	*Sample Weight:*	**1,0000**

Chromatogram

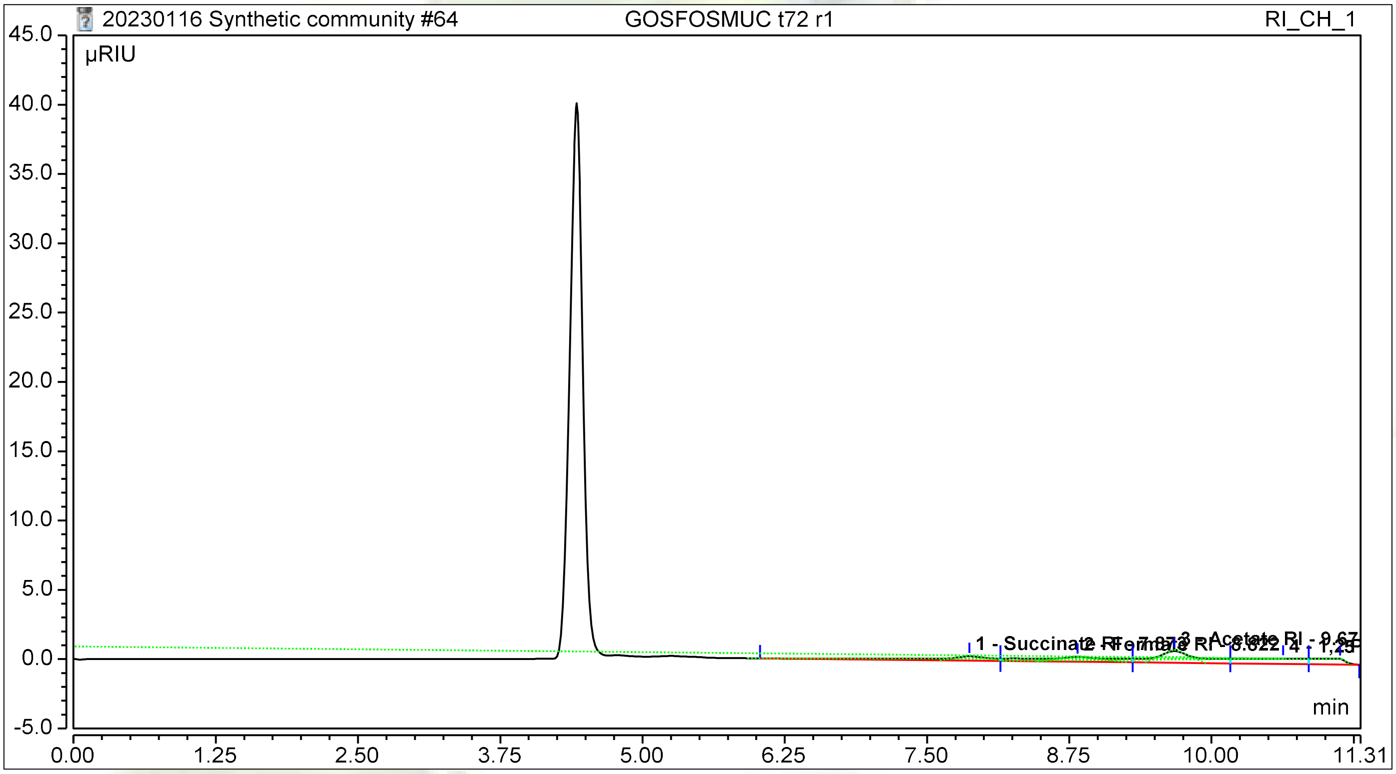

Peak Results

No.	Peak Name	Retention Time min	Width (50%) min	Type	Resolution (EP)	Asymmetry (EP)	Plates (EP)
n.a.	GlcNAc	n.a.	n.a.	n.a.	n.a.	n.a.	n.a.
n.a.	Citrate	n.a.	n.a.	n.a.	n.a.	n.a.	n.a.
n.a.	Glucose	n.a.	n.a.	n.a.	n.a.	n.a.	n.a.
n.a.	Galactose	n.a.	n.a.	n.a.	n.a.	n.a.	n.a.
n.a.	Fucose	n.a.	n.a.	n.a.	n.a.	n.a.	n.a.
1	Succinate RI	7,873	n.a.	BM	n.a.	n.a.	n.a.
n.a.	Lactate RI	n.a.	n.a.	n.a.	n.a.	n.a.	n.a.
n.a.	glycerol	n.a.	n.a.	n.a.	n.a.	n.a.	n.a.
2	Formate RI	8,822	n.a.	M	n.a.	n.a.	n.a.
3	Acetate RI	9,673	0,318	M	n.a.	n.a.	5123
4	1,2 PDO RI	10,630	n.a.	M	n.a.	n.a.	n.a.
n.a.	1,3-PDO	n.a.	n.a.	n.a.	n.a.	n.a.	n.a.
5		11,130	n.a.	MB	n.a.	n.a.	n.a.
n.a.	Propionate RI	n.a.	n.a.	n.a.	n.a.	n.a.	n.a.
n.a.	1,3-PDO	n.a.	n.a.	n.a.	n.a.	n.a.	n.a.
n.a.	2-3 BDO	n.a.	n.a.	n.a.	n.a.	n.a.	n.a.
n.a.	Ethanol	n.a.	n.a.	n.a.	n.a.	n.a.	n.a.
n.a.	Isobutyrate RI	n.a.	n.a.	n.a.	n.a.	n.a.	n.a.

n.a.	Butyrate RI	n.a.	n.a.	n.a.	n.a.	n.a.	n.a.

Chromatogram and SST Results

Injection Details

Injection Name:	**GOSFOSMUC t72 r1**	*Run Time (min):*	**11,31**
Vial Number:	**3:61**	*Injection Volume:*	**10,00**
Injection Type:	**Unknown**	*Channel:*	**RI_CH_1**
Calibration Level:		*Wavelength:*	**n.a.**
Instrument Method:	**Default method LC2030C 45 gr 20 min**	*Bandwidth:*	**n.a.**
Processing Method:	**Processing Method LC2030 45 gr**	*Dilution Factor:*	**1,0000**
Injection Date/Time:	**17/Jan/23 13:11**	*Sample Weight:*	**1,0000**

Chromatogram

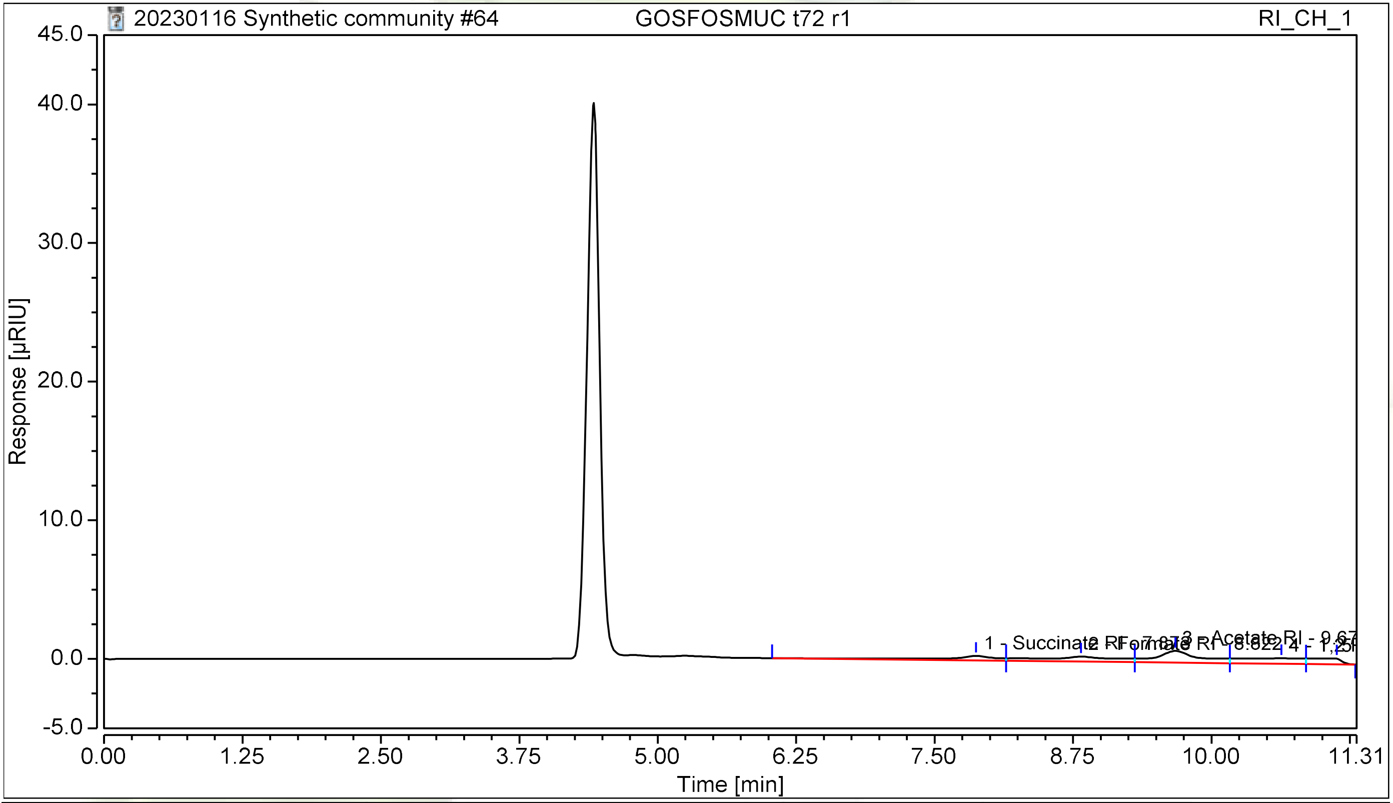

SST Results

No.	Name	Inj.Condition	Peak	Test Result	Injection
**Number of executed test cases:**	**n.a.**		**Total Result:**	**Passed**	

Chromatogram and Results

Injection Details

Injection Name:	**GOSFOSMUC t72 r2**	*Run Time (min):*	**20,00**
Vial Number:	**3:62**	*Injection Volume:*	**10,00**
Injection Type:	**Unknown**	*Channel:*	**RI_CH_1**
Calibration Level:		*Wavelength:*	**n.a.**
Instrument Method:	**Default method LC2030C 45 gr 20 min**	*Bandwidth:*	**n.a.**
Processing Method:	**Processing Method LC2030 45 gr**	*Dilution Factor:*	**1,0000**
Injection Date/Time:	**17/Jan/23 13:36**	*Sample Weight:*	**1,0000**

Chromatogram

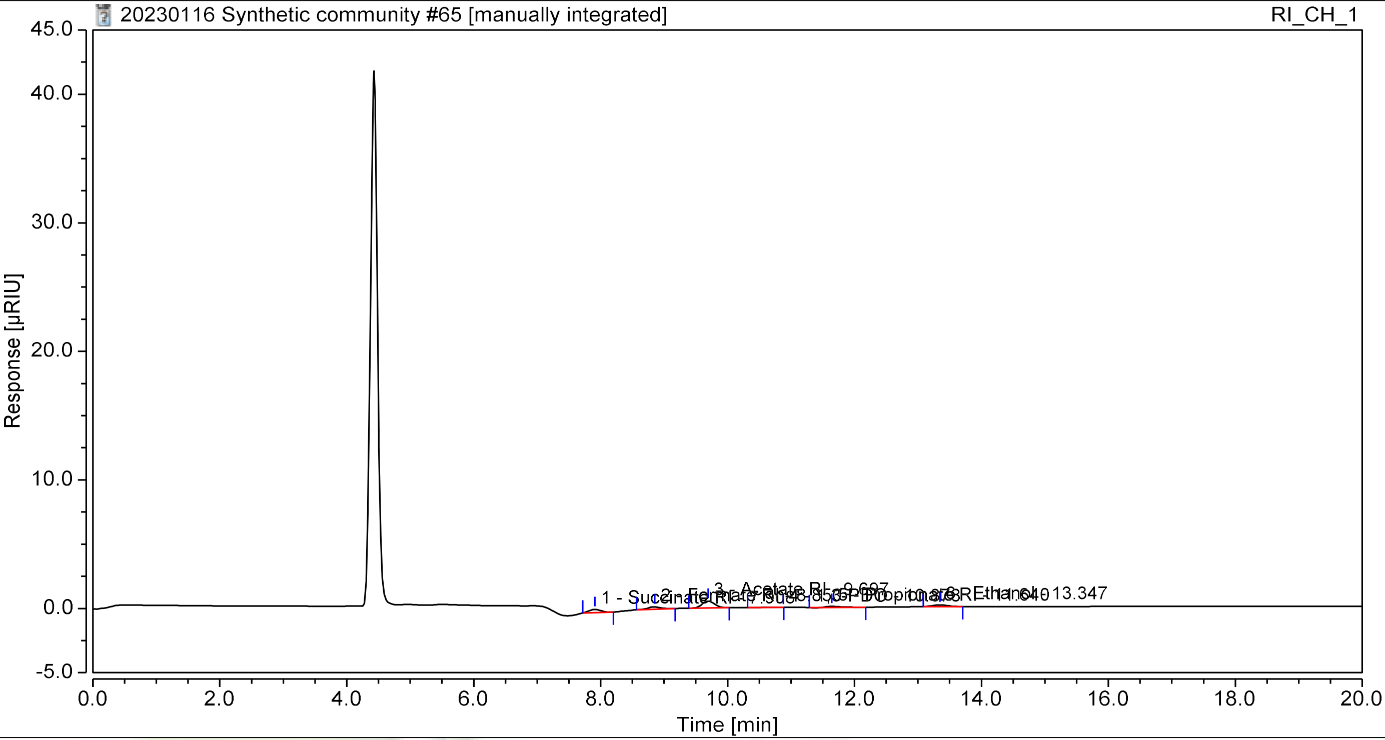

Integration Results

No.	Peak Name	Retention Time min	Area µRIU*min	Height µRIU	Relative Area %	Relative Height %	Amount
n.a.	GlcNAc	n.a.	n.a.	n.a.	n.a.	n.a.	n.a.
n.a.	Citrate	n.a.	n.a.	n.a.	n.a.	n.a.	n.a.
n.a.	Glucose	n.a.	n.a.	n.a.	n.a.	n.a.	n.a.
n.a.	Galactose	n.a.	n.a.	n.a.	n.a.	n.a.	n.a.
n.a.	Fucose	n.a.	n.a.	n.a.	n.a.	n.a.	n.a.
1	Succinate RI	7,908	0,054	0,247	19,13	21,03	1,1067
n.a.	Lactate RI	n.a.	n.a.	n.a.	n.a.	n.a.	n.a.
n.a.	glycerol	n.a.	n.a.	n.a.	n.a.	n.a.	n.a.
2	Formate RI	8,850	0,042	0,181	14,82	15,39	4,2525
3	Acetate RI	9,697	0,122	0,534	43,41	45,38	7,5468
n.a.	1,2 PDO RI	n.a.	n.a.	n.a.	n.a.	n.a.	n.a.
4	1,3-PDO	10,878	0,002	0,000	0,65	0,02	n.a.
5	Propionate RI	11,640	0,026	0,088	9,12	7,52	1,0691
n.a.	1,3-PDO	n.a.	n.a.	n.a.	n.a.	n.a.	n.a.
n.a.	2-3 BDO	n.a.	n.a.	n.a.	n.a.	n.a.	n.a.
6	Ethanol	13,347	0,036	0,125	12,87	10,65	3,7854
n.a.	Isobutyrate RI	n.a.	n.a.	n.a.	n.a.	n.a.	n.a.
n.a.	Butyrate RI	n.a.	n.a.	n.a.	n.a.	n.a.	n.a.
**Total:**			**0,280**	**1,176**	**100,00**	**100,00**	

Peak Analysis

Injection Details

Injection Name:	**GOSFOSMUC t72 r2**	*Run Time (min):*	**20,00**
Vial Number:	**3:62**	*Injection Volume:*	**10,00**
Injection Type:	**Unknown**	*Channel:*	**RI_CH_1**
Calibration Level:		*Wavelength:*	**n.a.**
Instrument Method:	**Default method LC2030C 45 gr 20 min**	*Bandwidth:*	**n.a.**
Processing Method:	**Processing Method LC2030 45 gr**	*Dilution Factor:*	**1,0000**
Injection Date/Time:	**17/Jan/23 13:36**	*Sample Weight:*	**1,0000**

Chromatogram

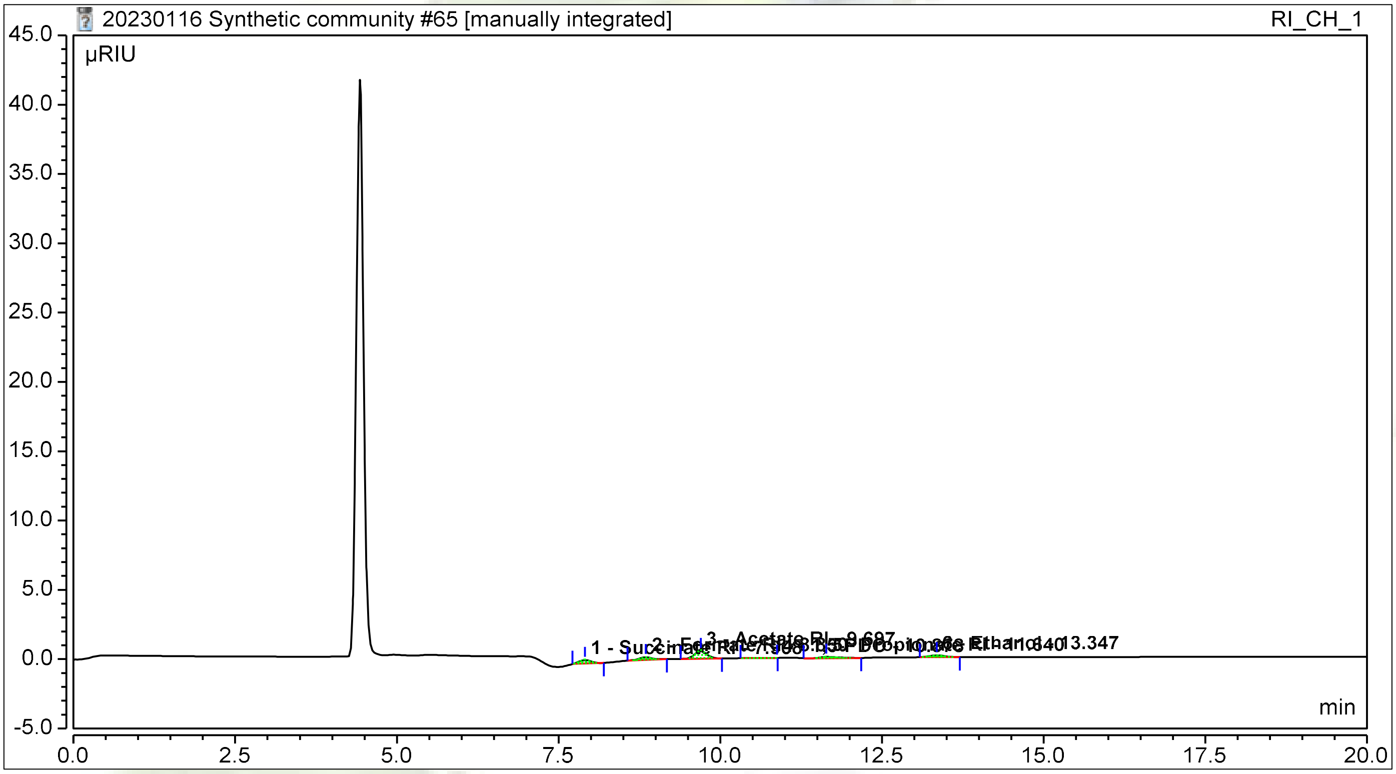

Peak Results

No.	Peak Name	Retention Time min	Width (50%) min	Type	Resolution (EP)	Asymmetry (EP)	Plates (EP)
n.a.	GlcNAc	n.a.	n.a.	n.a.	n.a.	n.a.	n.a.
n.a.	Citrate	n.a.	n.a.	n.a.	n.a.	n.a.	n.a.
n.a.	Glucose	n.a.	n.a.	n.a.	n.a.	n.a.	n.a.
n.a.	Galactose	n.a.	n.a.	n.a.	n.a.	n.a.	n.a.
n.a.	Fucose	n.a.	n.a.	n.a.	n.a.	n.a.	n.a.
1	Succinate RI	7,908	0,215	BMB*	2,63	1,05	7530
n.a.	Lactate RI	n.a.	n.a.	n.a.	n.a.	n.a.	n.a.
n.a.	glycerol	n.a.	n.a.	n.a.	n.a.	n.a.	n.a.
2	Formate RI	8,850	0,208	BMB*	2,35	1,01	10024
3	Acetate RI	9,697	0,216	BMB*	1,83	1,05	11114
n.a.	1,2 PDO RI	n.a.	n.a.	n.a.	n.a.	n.a.	n.a.
4	1,3-PDO	10,878	0,546	BMB*	1,07	0,51	2198
5	Propionate RI	11,640	0,291	BMB*	3,54	1,82	8886
n.a.	1,3-PDO	n.a.	n.a.	n.a.	n.a.	n.a.	n.a.
n.a.	2-3 BDO	n.a.	n.a.	n.a.	n.a.	n.a.	n.a.
6	Ethanol	13,347	0,278	BMB*	n.a.	1,10	12759
n.a.	Isobutyrate RI	n.a.	n.a.	n.a.	n.a.	n.a.	n.a.
n.a.	Butyrate RI	n.a.	n.a.	n.a.	n.a.	n.a.	n.a.

Chromatogram and SST Results

Injection Details

Injection Name:	**GOSFOSMUC t72 r2**	*Run Time (min):*	**20,00**
Vial Number:	**3:62**	*Injection Volume:*	**10,00**
Injection Type:	**Unknown**	*Channel:*	**RI_CH_1**
Calibration Level:		*Wavelength:*	**n.a.**
Instrument Method:	**Default method LC2030C 45 gr 20 min**	*Bandwidth:*	**n.a.**
Processing Method:	**Processing Method LC2030 45 gr**	*Dilution Factor:*	**1,0000**
Injection Date/Time:	**17/Jan/23 13:36**	*Sample Weight:*	**1,0000**

Chromatogram

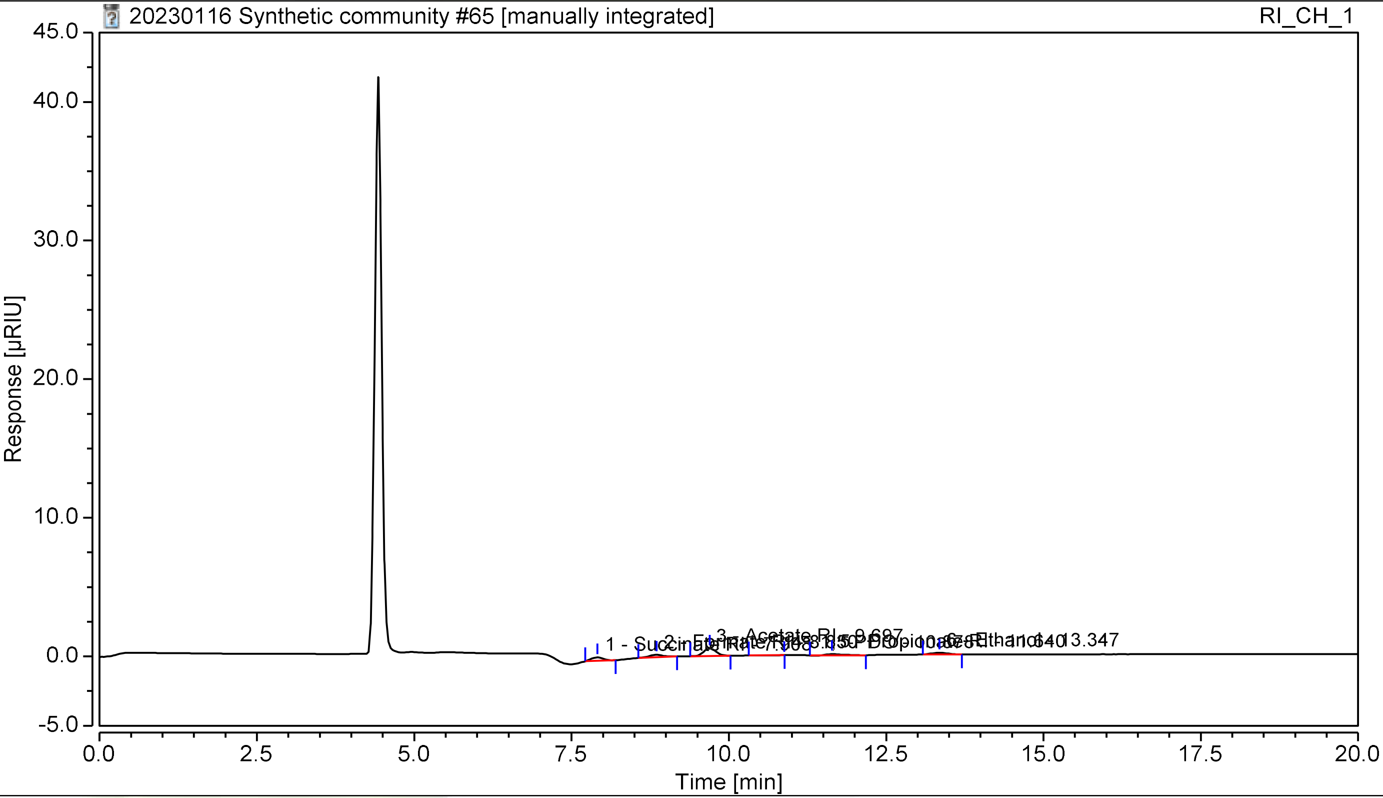

SST Results

No.	Name	Inj.Condition	Peak	Test Result	Injection

Number of executed test cases: **n.a.** **Total Result:** **Passed**

Chromatogram and Results

Injection Details

Injection Name:	**GOSFOSMUC t72 r3**	*Run Time (min):*	**20,00**
Vial Number:	**3:63**	*Injection Volume:*	**10,00**
Injection Type:	**Unknown**	*Channel:*	**RI_CH_1**
Calibration Level:		*Wavelength:*	**n.a.**
Instrument Method:	**Default method LC2030C 45 gr 20 min**	*Bandwidth:*	**n.a.**
Processing Method:	**Processing Method LC2030 45 gr**	*Dilution Factor:*	**1,0000**
Injection Date/Time:	**17/Jan/23 13:56**	*Sample Weight:*	**1,0000**

Chromatogram

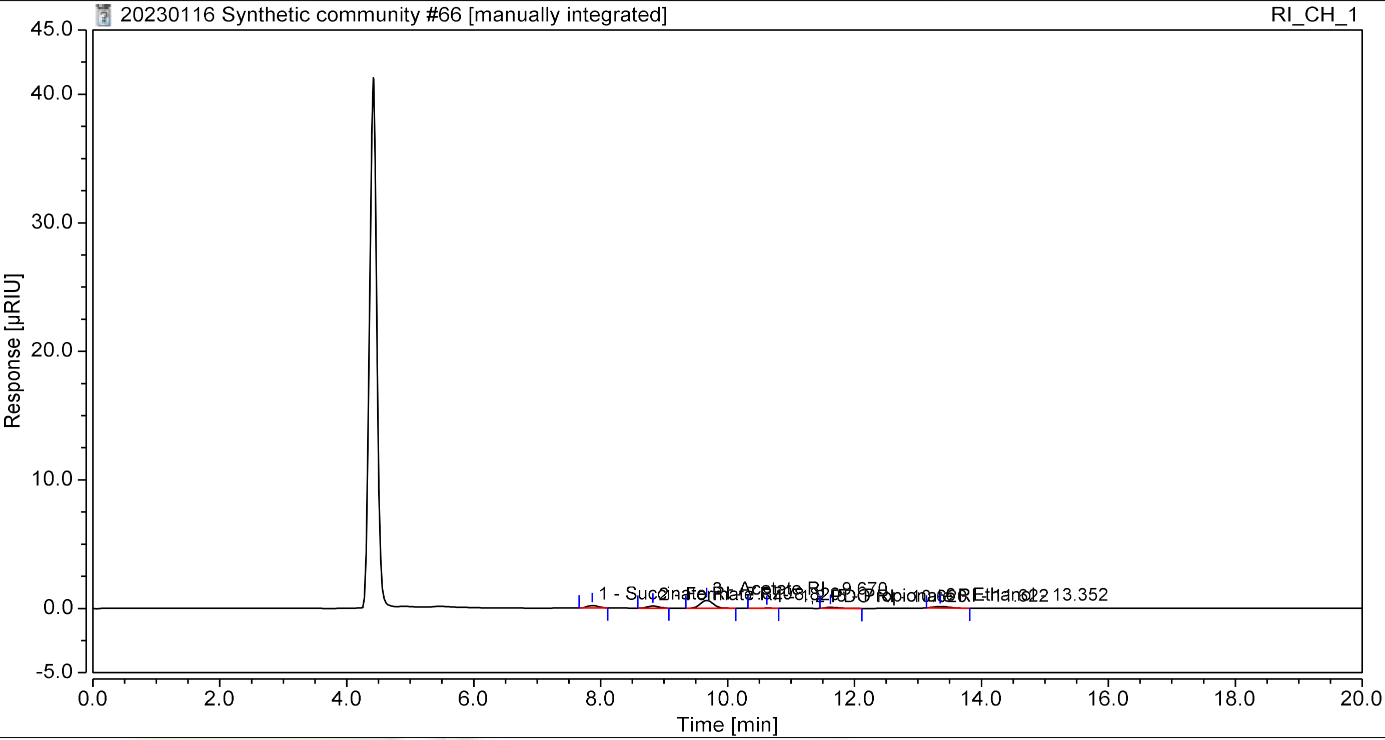

Integration Results

No.	Peak Name	Retention Time min	Area µRIU*min	Height µRIU	Relative Area %	Relative Height %	Amount
n.a.	GlcNAc	n.a.	n.a.	n.a.	n.a.	n.a.	n.a.
n.a.	Citrate	n.a.	n.a.	n.a.	n.a.	n.a.	n.a.
n.a.	Glucose	n.a.	n.a.	n.a.	n.a.	n.a.	n.a.
n.a.	Galactose	n.a.	n.a.	n.a.	n.a.	n.a.	n.a.
n.a.	Fucose	n.a.	n.a.	n.a.	n.a.	n.a.	n.a.
1	Succinate RI	7,870	0,039	0,192	14,56	16,68	0,8064
n.a.	Lactate RI	n.a.	n.a.	n.a.	n.a.	n.a.	n.a.
n.a.	glycerol	n.a.	n.a.	n.a.	n.a.	n.a.	n.a.
2	Formate RI	8,820	0,034	0,166	12,57	14,35	3,4506
3	Acetate RI	9,670	0,140	0,599	51,99	51,94	8,6511
4	1,2 PDO RI	10,620	0,001	0,004	0,19	0,33	0,0159
n.a.	1,3-PDO	n.a.	n.a.	n.a.	n.a.	n.a.	n.a.
5	Propionate RI	11,622	0,025	0,081	9,44	6,98	1,0588
n.a.	1,3-PDO	n.a.	n.a.	n.a.	n.a.	n.a.	n.a.
n.a.	2-3 BDO	n.a.	n.a.	n.a.	n.a.	n.a.	n.a.
6	Ethanol	13,352	0,030	0,112	11,25	9,72	3,1666
n.a.	Isobutyrate RI	n.a.	n.a.	n.a.	n.a.	n.a.	n.a.
n.a.	Butyrate RI	n.a.	n.a.	n.a.	n.a.	n.a.	n.a.
**Total:**			**0,268**	**1,154**	**100,00**	**100,00**	

Peak Analysis

Injection Details

Injection Name:	**GOSFOSMUC t72 r3**	*Run Time (min):*	**20,00**
Vial Number:	**3:63**	*Injection Volume:*	**10,00**
Injection Type:	**Unknown**	*Channel:*	**RI_CH_1**
Calibration Level:		*Wavelength:*	**n.a.**
Instrument Method:	**Default method LC2030C 45 gr 20 min**	*Bandwidth:*	**n.a.**
Processing Method:	**Processing Method LC2030 45 gr**	*Dilution Factor:*	**1,0000**
Injection Date/Time:	**17/Jan/23 13:56**	*Sample Weight:*	**1,0000**

Chromatogram

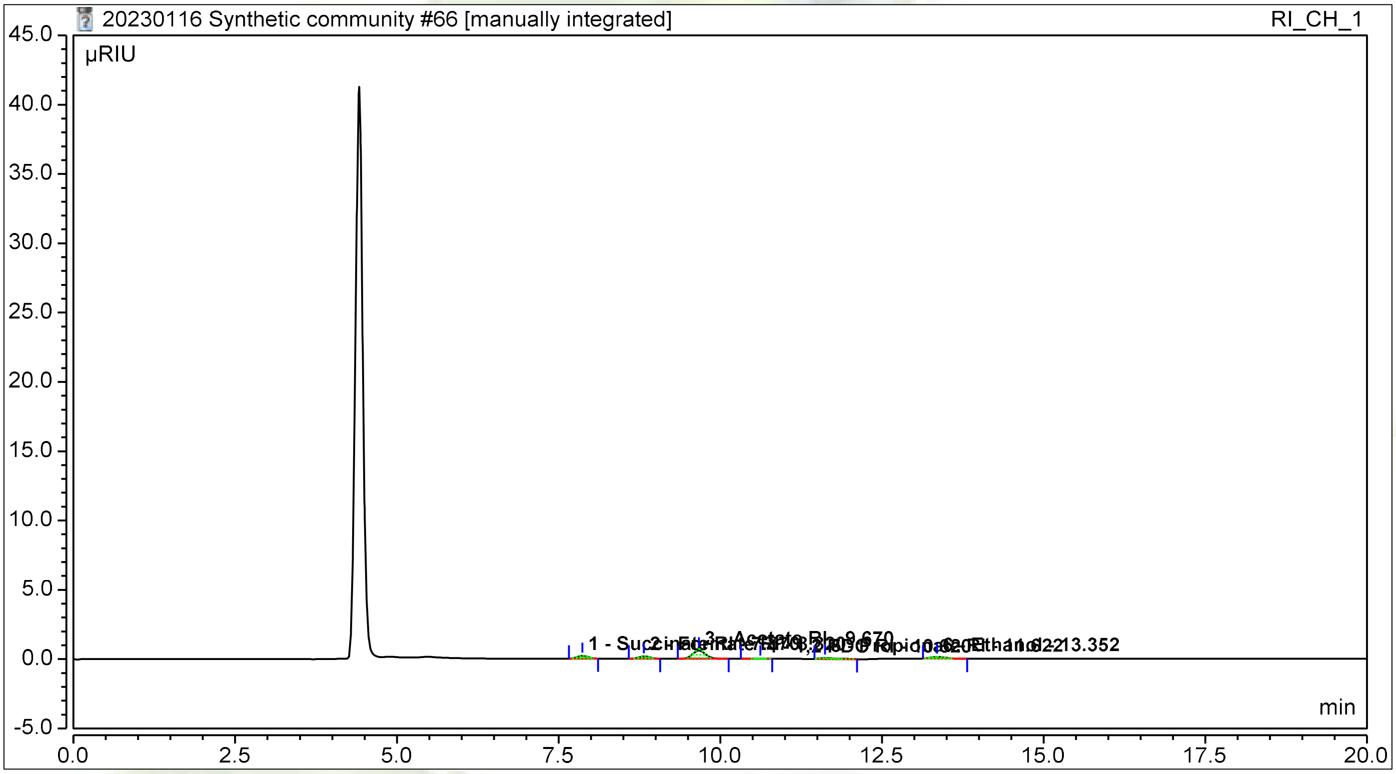

Peak Results

No.	Peak Name	Retention Time min	Width (50%) min	Type	Resolution (EP)	Asymmetry (EP)	Plates (EP)
n.a.	GlcNAc	n.a.	n.a.	n.a.	n.a.	n.a.	n.a.
n.a.	Citrate	n.a.	n.a.	n.a.	n.a.	n.a.	n.a.
n.a.	Glucose	n.a.	n.a.	n.a.	n.a.	n.a.	n.a.
n.a.	Galactose	n.a.	n.a.	n.a.	n.a.	n.a.	n.a.
n.a.	Fucose	n.a.	n.a.	n.a.	n.a.	n.a.	n.a.
1	Succinate RI	7,870	0,196	BMB*	2,86	1,07	8919
n.a.	Lactate RI	n.a.	n.a.	n.a.	n.a.	n.a.	n.a.
n.a.	glycerol	n.a.	n.a.	n.a.	n.a.	n.a.	n.a.
2	Formate RI	8,820	0,195	BMB*	2,41	1,06	11299
3	Acetate RI	9,670	0,220	BMB	2,97	1,07	10664
4	1,2 PDO RI	10,620	0,157	BMB*	2,87	1,08	25462
n.a.	1,3-PDO	n.a.	n.a.	n.a.	n.a.	n.a.	n.a.
5	Propionate RI	11,622	0,255	BMB*	3,91	2,00	11536
n.a.	1,3-PDO	n.a.	n.a.	n.a.	n.a.	n.a.	n.a.
n.a.	2-3 BDO	n.a.	n.a.	n.a.	n.a.	n.a.	n.a.
6	Ethanol	13,352	0,267	BMB*	n.a.	1,15	13865
n.a.	Isobutyrate RI	n.a.	n.a.	n.a.	n.a.	n.a.	n.a.
n.a.	Butyrate RI	n.a.	n.a.	n.a.	n.a.	n.a.	n.a.

Chromatogram and SST Results

Injection Details

Injection Name:	**GOSFOSMUC t72 r3**	*Run Time (min):*	**20,00**
Vial Number:	**3:63**	*Injection Volume:*	**10,00**
Injection Type:	**Unknown**	*Channel:*	**RI_CH_1**
Calibration Level:		*Wavelength:*	**n.a.**
Instrument Method:	**Default method LC2030C 45 gr 20 min**	*Bandwidth:*	**n.a.**
Processing Method:	**Processing Method LC2030 45 gr**	*Dilution Factor:*	**1,0000**
Injection Date/Time:	**17/Jan/23 13:56**	*Sample Weight:*	**1,0000**

Chromatogram

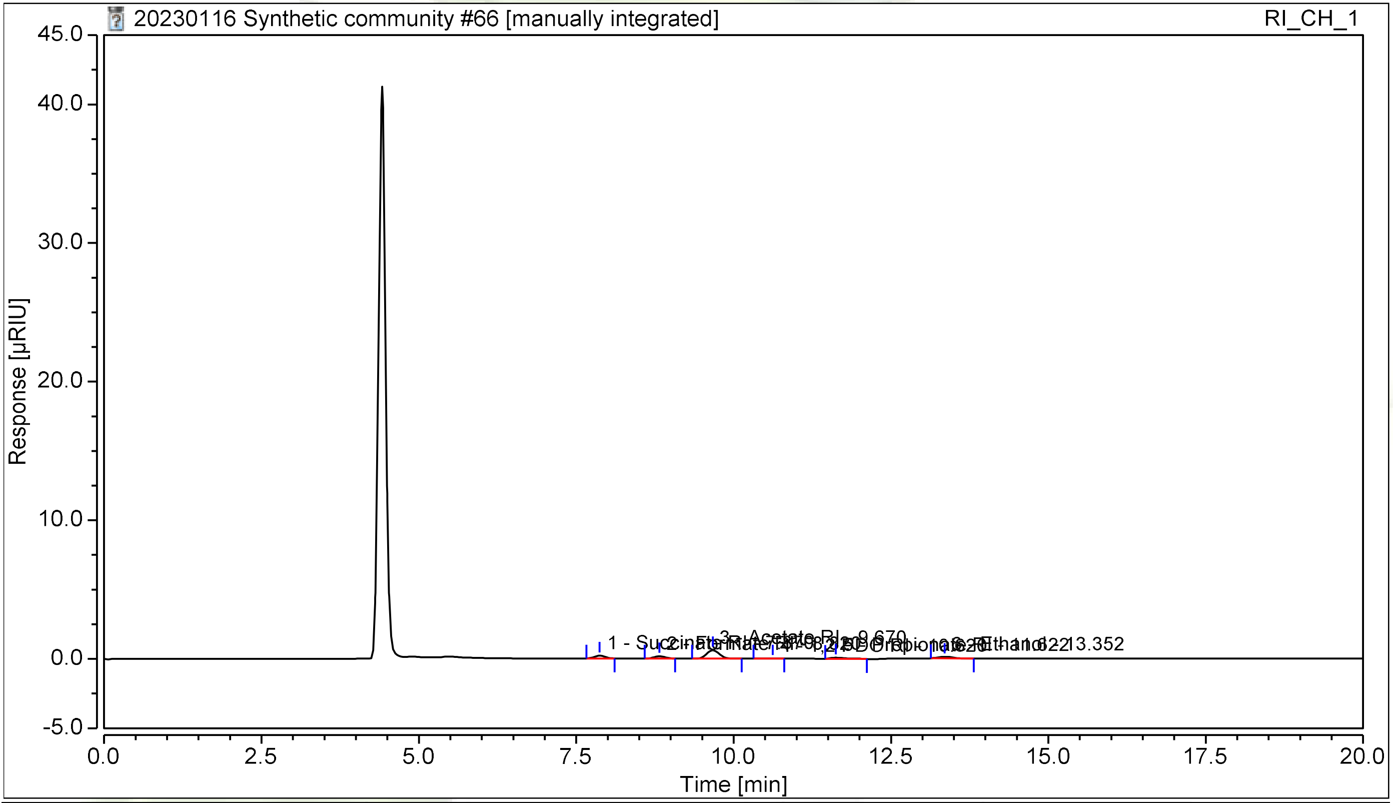

SST Results

No.	Name	Inj.Condition	Peak	Test Result	Injection
**Number of executed test cases:**	**n.a.**		**Total Result:**	**Passed**	

Chromeleon (c) Dionex
Version 7.2.10.25868

Chromatogram and Results

Injection Details

Injection Name:	**GOSFOSMUC t96 r1**	*Run Time (min):*	**20,00**
Vial Number:	**3:64**	*Injection Volume:*	**10,00**
Injection Type:	**Unknown**	*Channel:*	**RI_CH_1**
Calibration Level:		*Wavelength:*	**n.a.**
Instrument Method:	**Default method LC2030C 45 gr 20 min**	*Bandwidth:*	**n.a.**
Processing Method:	**Processing Method LC2030 45 gr**	*Dilution Factor:*	**1,0000**
Injection Date/Time:	**17/Jan/23 14:17**	*Sample Weight:*	**1,0000**

Chromatogram

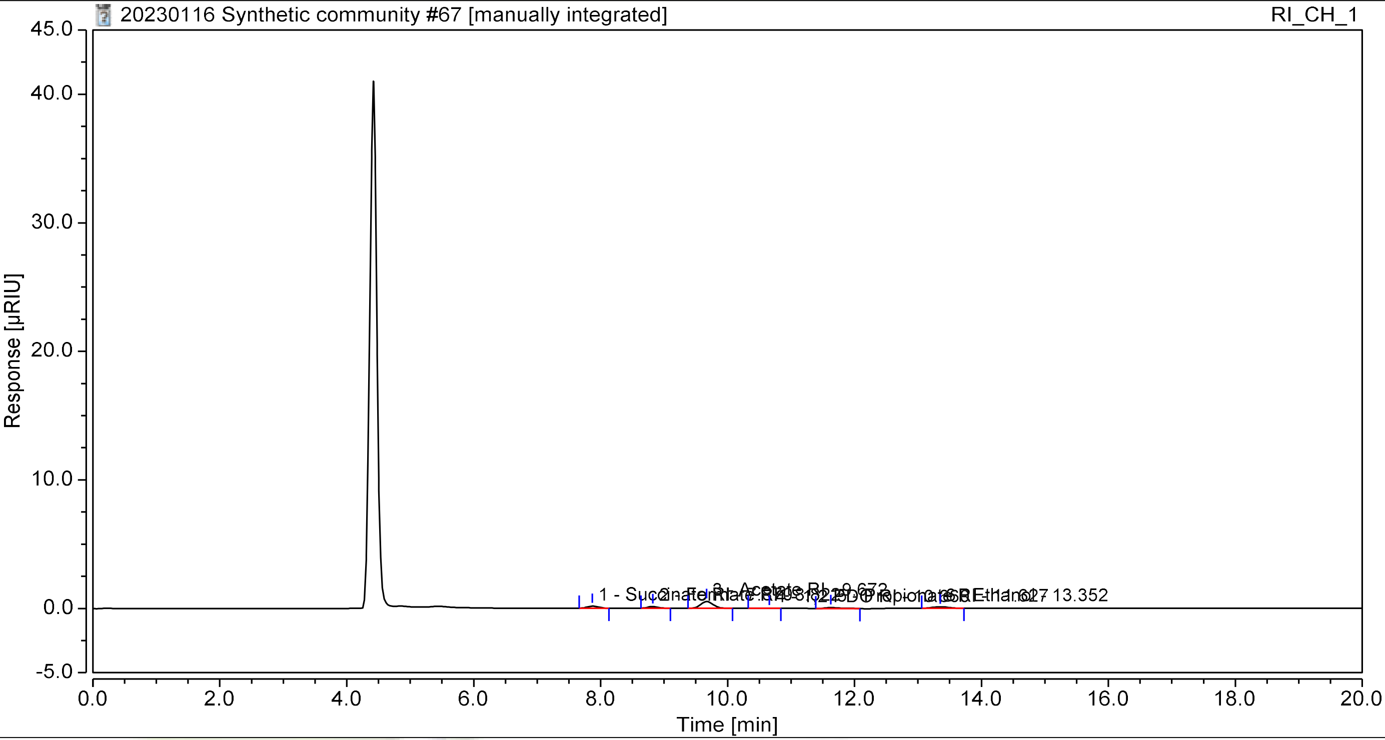

Integration Results

No.	Peak Name	Retention Time min	Area µRIU*min	Height µRIU	Relative Area %	Relative Height %	Amount
n.a.	GlcNAc	n.a.	n.a.	n.a.	n.a.	n.a.	n.a.
n.a.	Citrate	n.a.	n.a.	n.a.	n.a.	n.a.	n.a.
n.a.	Glucose	n.a.	n.a.	n.a.	n.a.	n.a.	n.a.
n.a.	Galactose	n.a.	n.a.	n.a.	n.a.	n.a.	n.a.
n.a.	Fucose	n.a.	n.a.	n.a.	n.a.	n.a.	n.a.
1	Succinate RI	7,870	0,032	0,159	13,32	15,52	0,6613
n.a.	Lactate RI	n.a.	n.a.	n.a.	n.a.	n.a.	n.a.
n.a.	glycerol	n.a.	n.a.	n.a.	n.a.	n.a.	n.a.
2	Formate RI	8,822	0,026	0,130	10,71	12,70	2,6353
3	Acetate RI	9,672	0,121	0,527	50,28	51,38	7,4990
4	1,2 PDO RI	10,660	0,002	0,009	0,77	0,84	0,0564
n.a.	1,3-PDO	n.a.	n.a.	n.a.	n.a.	n.a.	n.a.
5	Propionate RI	11,627	0,025	0,082	10,44	7,99	1,0497
n.a.	1,3-PDO	n.a.	n.a.	n.a.	n.a.	n.a.	n.a.
n.a.	2-3 BDO	n.a.	n.a.	n.a.	n.a.	n.a.	n.a.
6	Ethanol	13,352	0,035	0,119	14,48	11,57	3,6541
n.a.	Isobutyrate RI	n.a.	n.a.	n.a.	n.a.	n.a.	n.a.
n.a.	Butyrate RI	n.a.	n.a.	n.a.	n.a.	n.a.	n.a.
**Total:**			**0,241**	**1,026**	**100,00**	**100,00**	

Peak Analysis

Injection Details

Injection Name:	**GOSFOSMUC t96 r1**	*Run Time (min):*	**20,00**
Vial Number:	**3:64**	*Injection Volume:*	**10,00**
Injection Type:	**Unknown**	*Channel:*	**RI_CH_1**
Calibration Level:		*Wavelength:*	**n.a.**
Instrument Method:	**Default method LC2030C 45 gr 20 min**	*Bandwidth:*	**n.a.**
Processing Method:	**Processing Method LC2030 45 gr**	*Dilution Factor:*	**1,0000**
Injection Date/Time:	**17/Jan/23 14:17**	*Sample Weight:*	**1,0000**

Chromatogram

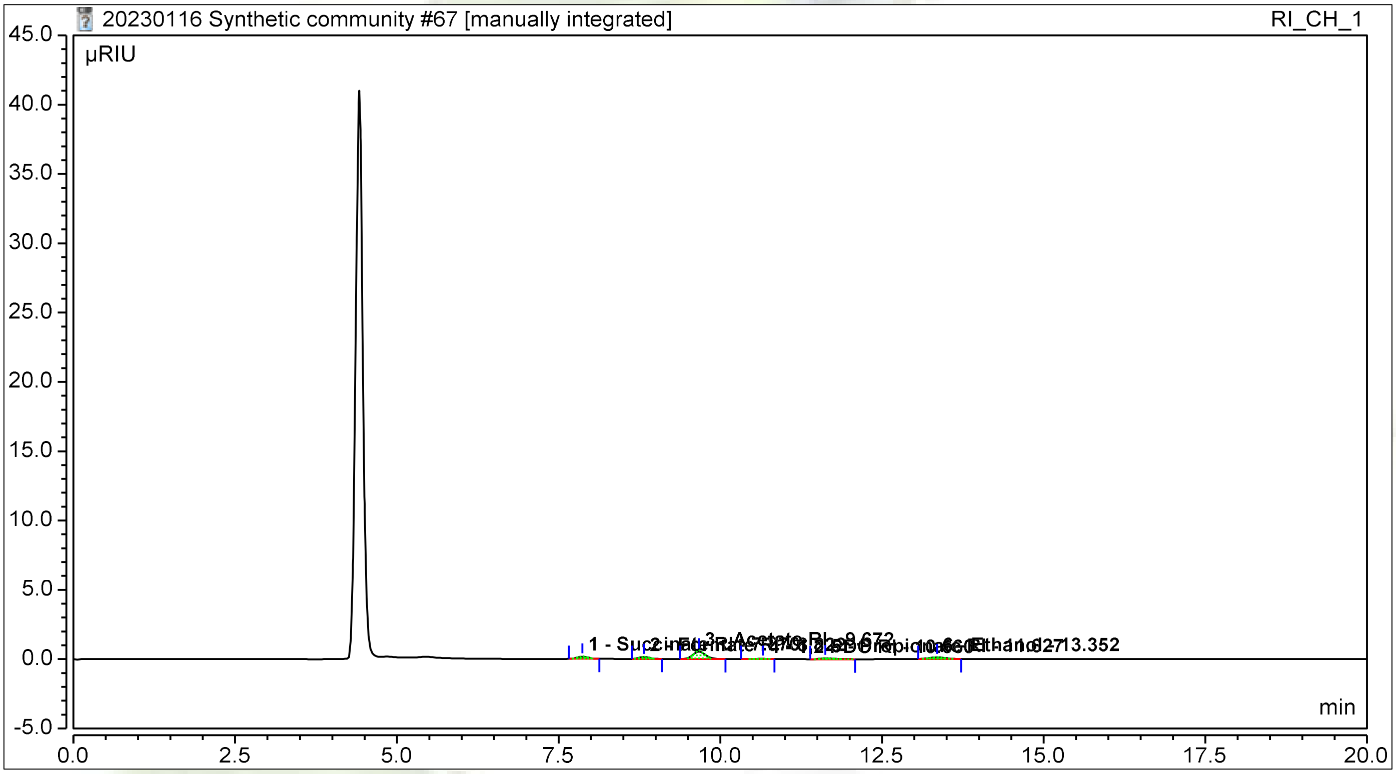

Peak Results

No.	Peak Name	Retention Time min	Width (50%) min	Type	Resolution (EP)	Asymmetry (EP)	Plates (EP)
n.a.	GlcNAc	n.a.	n.a.	n.a.	n.a.	n.a.	n.a.
n.a.	Citrate	n.a.	n.a.	n.a.	n.a.	n.a.	n.a.
n.a.	Glucose	n.a.	n.a.	n.a.	n.a.	n.a.	n.a.
n.a.	Galactose	n.a.	n.a.	n.a.	n.a.	n.a.	n.a.
n.a.	Fucose	n.a.	n.a.	n.a.	n.a.	n.a.	n.a.
1	Succinate RI	7,870	0,193	BMB*	2,93	1,07	9198
n.a.	Lactate RI	n.a.	n.a.	n.a.	n.a.	n.a.	n.a.
n.a.	glycerol	n.a.	n.a.	n.a.	n.a.	n.a.	n.a.
2	Formate RI	8,822	0,190	BMB*	2,47	1,11	11931
3	Acetate RI	9,672	0,216	BMB	2,63	1,07	11059
4	1,2 PDO RI	10,660	0,227	BMB*	2,35	0,83	12195
n.a.	1,3-PDO	n.a.	n.a.	n.a.	n.a.	n.a.	n.a.
5	Propionate RI	11,627	0,258	BMB*	3,77	1,69	11269
n.a.	1,3-PDO	n.a.	n.a.	n.a.	n.a.	n.a.	n.a.
n.a.	2-3 BDO	n.a.	n.a.	n.a.	n.a.	n.a.	n.a.
6	Ethanol	13,352	0,282	BMB*	n.a.	1,08	12460
n.a.	Isobutyrate RI	n.a.	n.a.	n.a.	n.a.	n.a.	n.a.
n.a.	Butyrate RI	n.a.	n.a.	n.a.	n.a.	n.a.	n.a.

Chromatogram and SST Results

Injection Details

Injection Name:	**GOSFOSMUC t96 r1**	*Run Time (min):*	**20,00**
Vial Number:	**3:64**	*Injection Volume:*	**10,00**
Injection Type:	**Unknown**	*Channel:*	**RI_CH_1**
Calibration Level:		*Wavelength:*	**n.a.**
Instrument Method:	**Default method LC2030C 45 gr 20 min**	*Bandwidth:*	**n.a.**
Processing Method:	**Processing Method LC2030 45 gr**	*Dilution Factor:*	**1,0000**
Injection Date/Time:	**17/Jan/23 14:17**	*Sample Weight:*	**1,0000**

Chromatogram

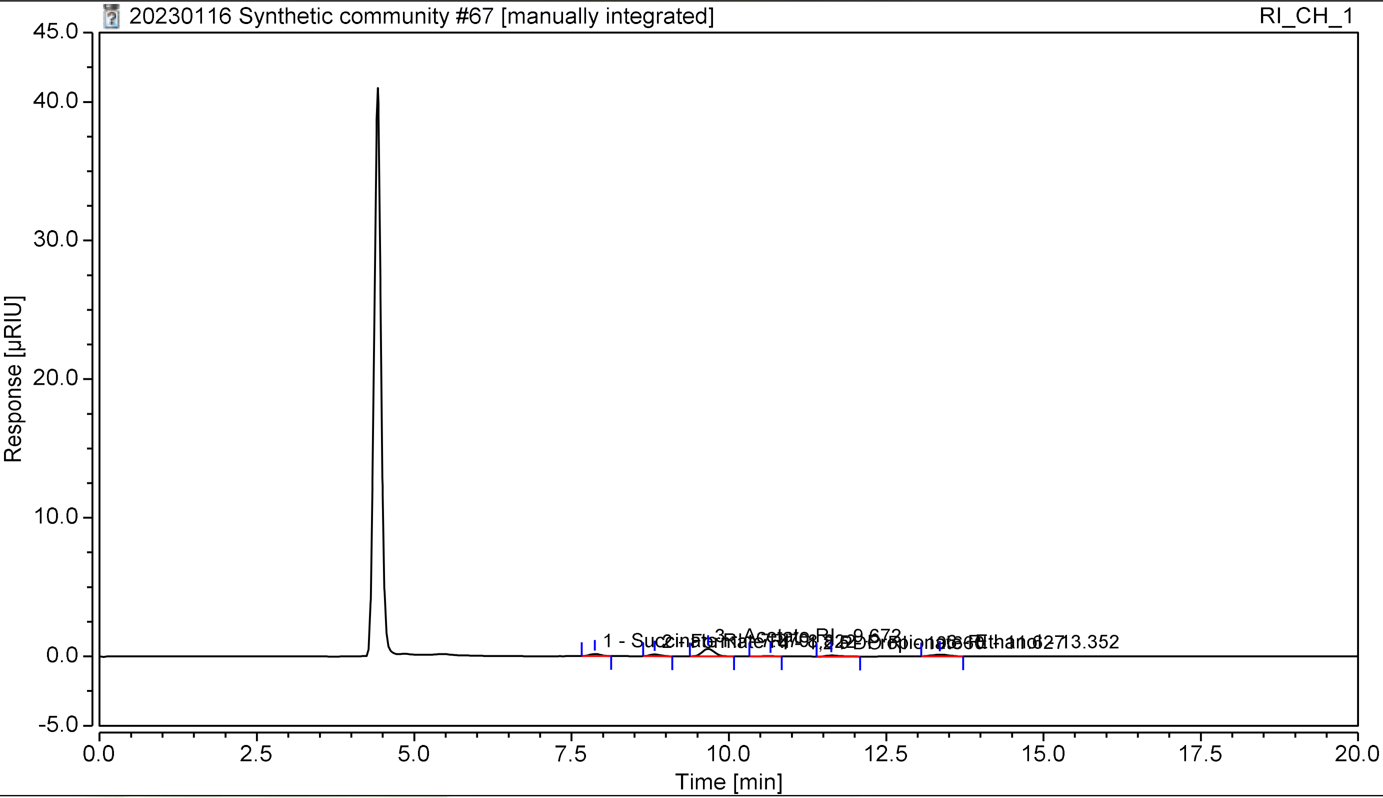

SST Results

No.	Name	Inj.Condition	Peak	Test Result	Injection
**Number of executed test cases:**	**n.a.**		**Total Result:**	**Passed**	

Chromatogram and Results

Injection Details

Injection Name:	**GOSFOSMUC t96 r2**	*Run Time (min):*	**20,00**
Vial Number:	**3:65**	*Injection Volume:*	**10,00**
Injection Type:	**Unknown**	*Channel:*	**RI_CH_1**
Calibration Level:		*Wavelength:*	**n.a.**
Instrument Method:	**Default method LC2030C 45 gr 20 min**	*Bandwidth:*	**n.a.**
Processing Method:	**Processing Method LC2030 45 gr**	*Dilution Factor:*	**1,0000**
Injection Date/Time:	**17/Jan/23 14:37**	*Sample Weight:*	**1,0000**

Chromatogram

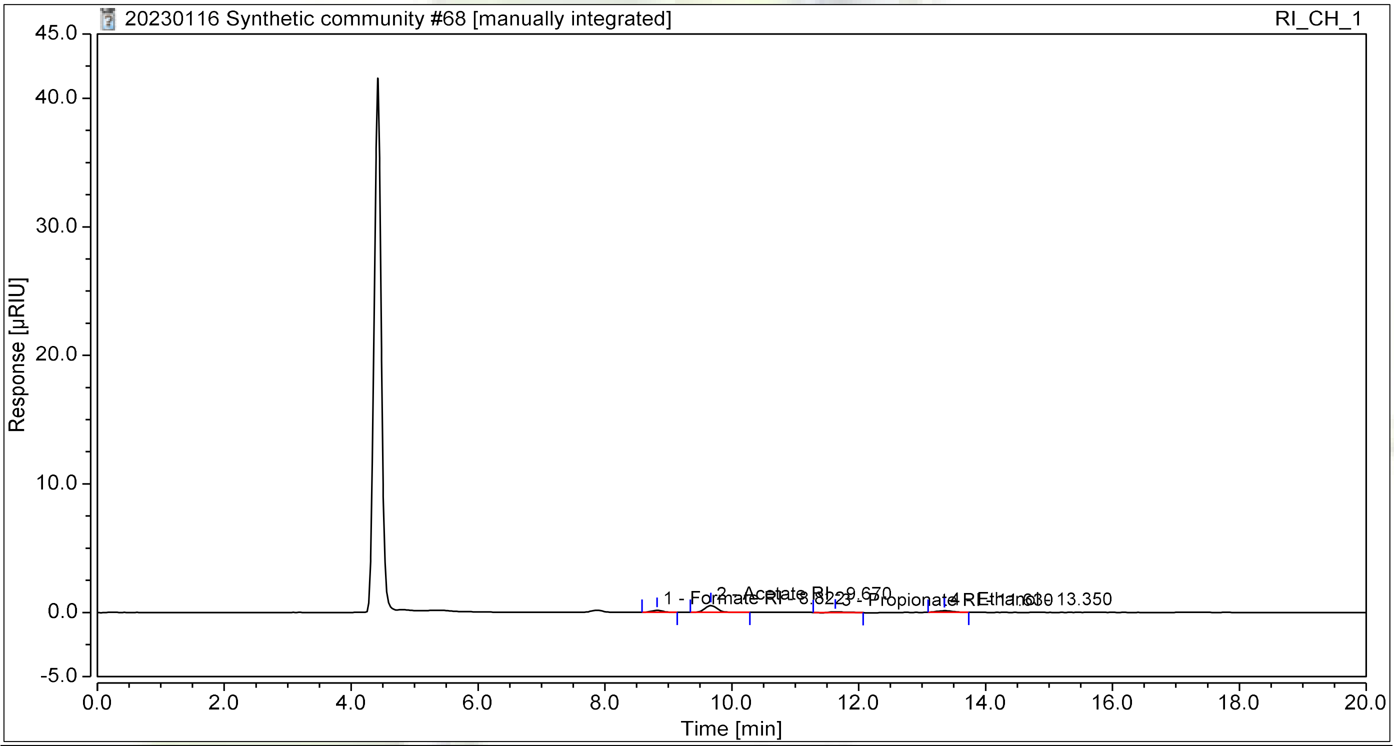

Integration Results

No.	Peak Name	Retention Time min	Area µRIU*min	Height µRIU	Relative Area %	Relative Height %	Amount
n.a.	GlcNAc	n.a.	n.a.	n.a.	n.a.	n.a.	n.a.
n.a.	Citrate	n.a.	n.a.	n.a.	n.a.	n.a.	n.a.
n.a.	Glucose	n.a.	n.a.	n.a.	n.a.	n.a.	n.a.
n.a.	Galactose	n.a.	n.a.	n.a.	n.a.	n.a.	n.a.
n.a.	Fucose	n.a.	n.a.	n.a.	n.a.	n.a.	n.a.
n.a.	Succinate RI	n.a.	n.a.	n.a.	n.a.	n.a.	n.a.
n.a.	Lactate RI	n.a.	n.a.	n.a.	n.a.	n.a.	n.a.
n.a.	glycerol	n.a.	n.a.	n.a.	n.a.	n.a.	n.a.
1	Formate RI	8,822	0,031	0,153	15,45	17,83	3,1421
2	Acetate RI	9,670	0,121	0,528	60,67	61,45	7,4748
n.a.	1,2 PDO RI	n.a.	n.a.	n.a.	n.a.	n.a.	n.a.
n.a.	1,3-PDO	n.a.	n.a.	n.a.	n.a.	n.a.	n.a.
3	Propionate RI	11,630	0,013	0,055	6,36	6,41	0,5287
n.a.	1,3-PDO	n.a.	n.a.	n.a.	n.a.	n.a.	n.a.
n.a.	2-3 BDO	n.a.	n.a.	n.a.	n.a.	n.a.	n.a.
4	Ethanol	13,350	0,035	0,123	17,52	14,31	3,6509
n.a.	Isobutyrate RI	n.a.	n.a.	n.a.	n.a.	n.a.	n.a.
n.a.	Butyrate RI	n.a.	n.a.	n.a.	n.a.	n.a.	n.a.
**Total:**			**0,199**	**0,858**	**100,00**	**100,00**	

Chromeleon (c) Dionex
Version 7.2.10.25868

Peak Analysis

Injection Details

Injection Name:	**GOSFOSMUC t96 r2**	*Run Time (min):*	**20,00**
Vial Number:	**3:65**	*Injection Volume:*	**10,00**
Injection Type:	**Unknown**	*Channel:*	**RI_CH_1**
Calibration Level:		*Wavelength:*	**n.a.**
Instrument Method:	**Default method LC2030C 45 gr 20 min**	*Bandwidth:*	**n.a.**
Processing Method:	**Processing Method LC2030 45 gr**	*Dilution Factor:*	**1,0000**
Injection Date/Time:	**17/Jan/23 14:37**	*Sample Weight:*	**1,0000**

Chromatogram

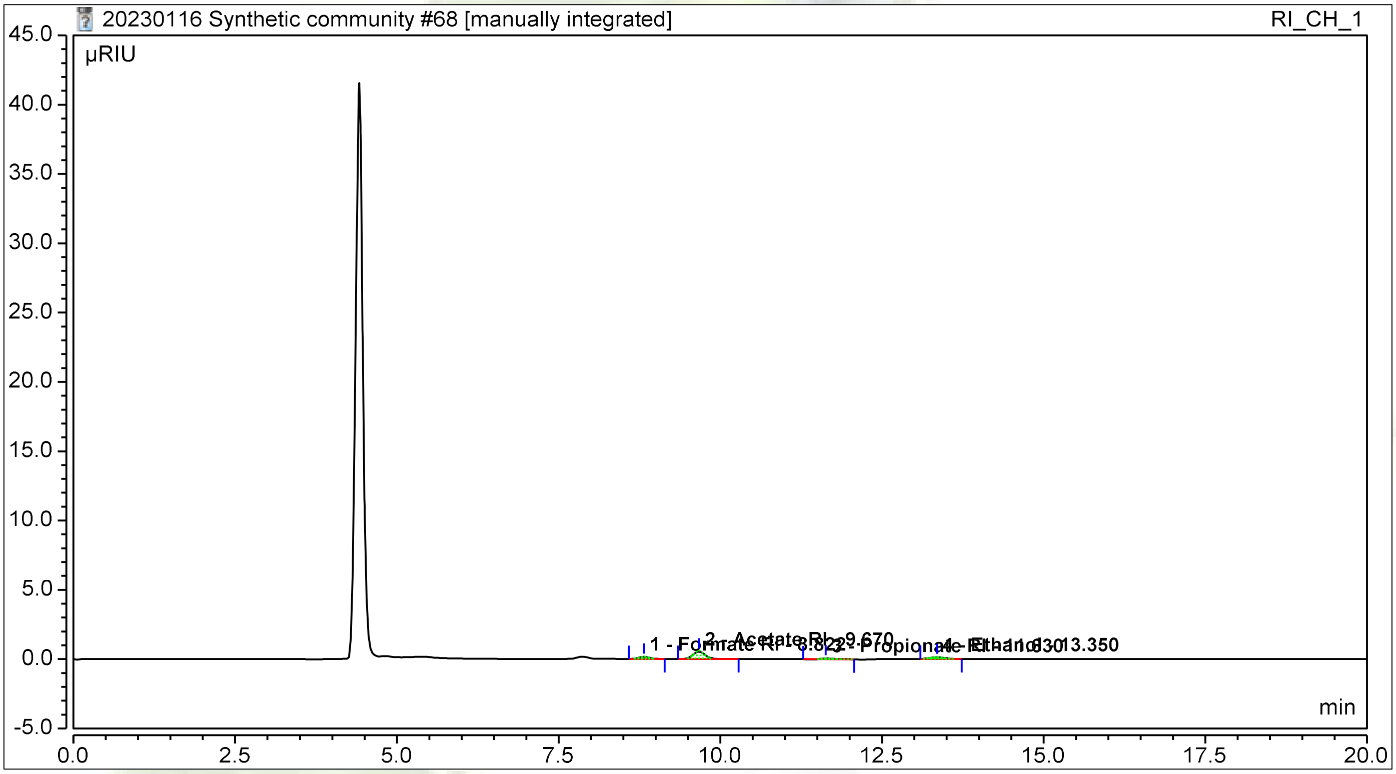

Peak Results

No.	Peak Name	Retention Time min	Width (50%) min	Type	Resolution (EP)	Asymmetry (EP)	Plates (EP)
n.a.	GlcNAc	n.a.	n.a.	n.a.	n.a.	n.a.	n.a.
n.a.	Citrate	n.a.	n.a.	n.a.	n.a.	n.a.	n.a.
n.a.	Glucose	n.a.	n.a.	n.a.	n.a.	n.a.	n.a.
n.a.	Galactose	n.a.	n.a.	n.a.	n.a.	n.a.	n.a.
n.a.	Fucose	n.a.	n.a.	n.a.	n.a.	n.a.	n.a.
n.a.	Succinate RI	n.a.	n.a.	n.a.	n.a.	n.a.	n.a.
n.a.	Lactate RI	n.a.	n.a.	n.a.	n.a.	n.a.	n.a.
n.a.	glycerol	n.a.	n.a.	n.a.	n.a.	n.a.	n.a.
1	Formate RI	8,822	0,190	BMB*	2,47	1,09	11925
2	Acetate RI	9,670	0,214	BMB	5,13	1,07	11271
n.a.	1,2 PDO RI	n.a.	n.a.	n.a.	n.a.	n.a.	n.a.
n.a.	1,3-PDO	n.a.	n.a.	n.a.	n.a.	n.a.	n.a.
3	Propionate RI	11,630	0,237	BMB*	3,98	2,14	13363
n.a.	1,3-PDO	n.a.	n.a.	n.a.	n.a.	n.a.	n.a.
n.a.	2-3 BDO	n.a.	n.a.	n.a.	n.a.	n.a.	n.a.
4	Ethanol	13,350	0,274	BMB*	n.a.	1,13	13181
n.a.	Isobutyrate RI	n.a.	n.a.	n.a.	n.a.	n.a.	n.a.
n.a.	Butyrate RI	n.a.	n.a.	n.a.	n.a.	n.a.	n.a.

Chromatogram and SST Results

Injection Details

Injection Name:	**GOSFOSMUC t96 r2**	*Run Time (min):*	**20,00**
Vial Number:	**3:65**	*Injection Volume:*	**10,00**
Injection Type:	**Unknown**	*Channel:*	**RI_CH_1**
Calibration Level:		*Wavelength:*	**n.a.**
Instrument Method:	**Default method LC2030C 45 gr 20 min**	*Bandwidth:*	**n.a.**
Processing Method:	**Processing Method LC2030 45 gr**	*Dilution Factor:*	**1,0000**
Injection Date/Time:	**17/Jan/23 14:37**	*Sample Weight:*	**1,0000**

Chromatogram

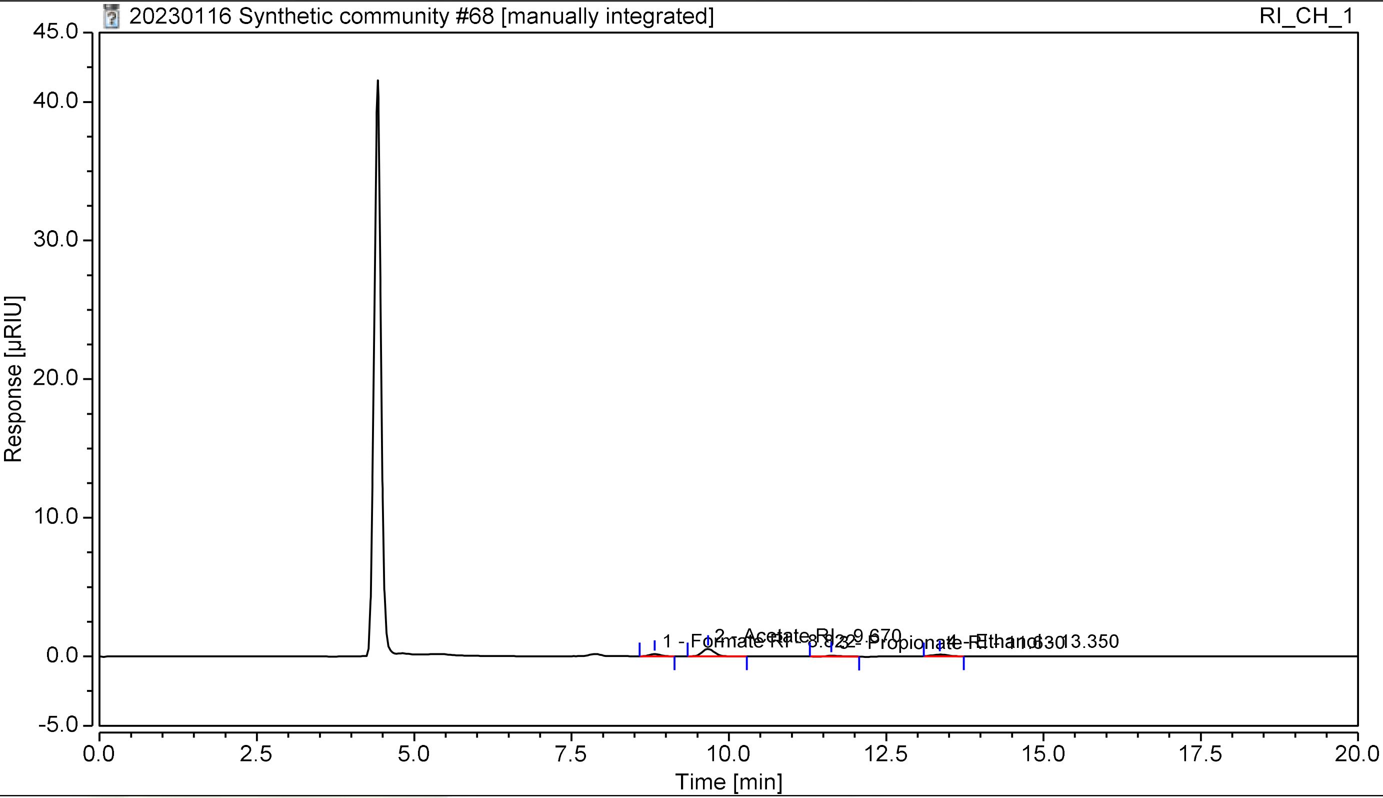

SST Results

No.	Name	Inj.Condition	Peak	Test Result	Injection
**Number of executed test cases:**	**n.a.**		**Total Result:**	**Passed**	

Chromatogram and Results

Injection Details

Injection Name:	**GOSFOSMUC t96 r3**	*Run Time (min):*	**20,00**
Vial Number:	**3:66**	*Injection Volume:*	**10,00**
Injection Type:	**Unknown**	*Channel:*	**RI_CH_1**
Calibration Level:		*Wavelength:*	**n.a.**
Instrument Method:	**Default method LC2030C 45 gr 20 min**	*Bandwidth:*	**n.a.**
Processing Method:	**Processing Method LC2030 45 gr**	*Dilution Factor:*	**1,0000**
Injection Date/Time:	**17/Jan/23 14:58**	*Sample Weight:*	**1,0000**

Chromatogram

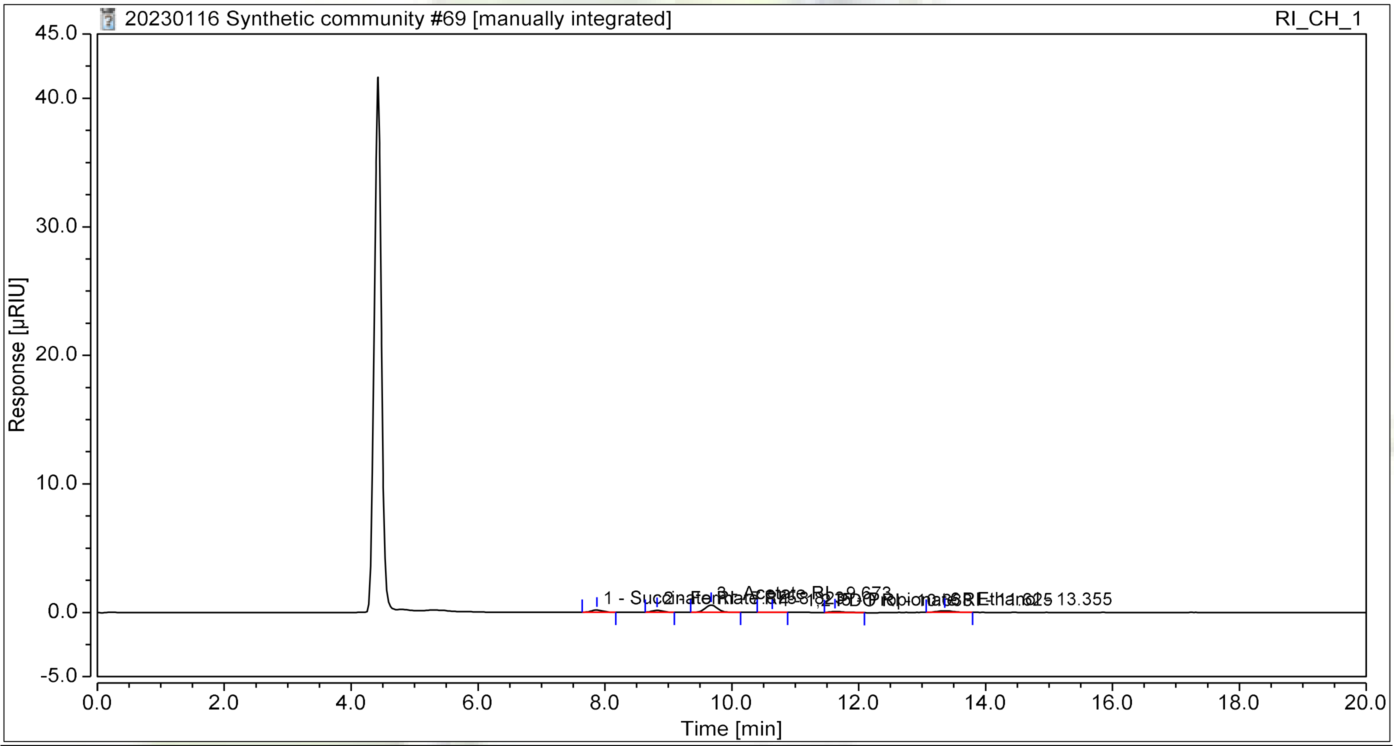

Integration Results

No.	Peak Name	Retention Time min	Area µRIU*min	Height µRIU	Relative Area %	Relative Height %	Amount
n.a.	GlcNAc	n.a.	n.a.	n.a.	n.a.	n.a.	n.a.
n.a.	Citrate	n.a.	n.a.	n.a.	n.a.	n.a.	n.a.
n.a.	Glucose	n.a.	n.a.	n.a.	n.a.	n.a.	n.a.
n.a.	Galactose	n.a.	n.a.	n.a.	n.a.	n.a.	n.a.
n.a.	Fucose	n.a.	n.a.	n.a.	n.a.	n.a.	n.a.
1	Succinate RI	7,875	0,035	0,172	13,89	15,73	0,7207
n.a.	Lactate RI	n.a.	n.a.	n.a.	n.a.	n.a.	n.a.
n.a.	glycerol	n.a.	n.a.	n.a.	n.a.	n.a.	n.a.
2	Formate RI	8,823	0,030	0,152	11,81	13,88	3,0397
3	Acetate RI	9,673	0,127	0,561	50,39	51,17	7,8571
4	1,2 PDO RI	10,638	0,001	0,006	0,53	0,53	0,0408
n.a.	1,3-PDO	n.a.	n.a.	n.a.	n.a.	n.a.	n.a.
5	Propionate RI	11,625	0,023	0,083	9,27	7,55	0,9751
n.a.	1,3-PDO	n.a.	n.a.	n.a.	n.a.	n.a.	n.a.
n.a.	2-3 BDO	n.a.	n.a.	n.a.	n.a.	n.a.	n.a.
6	Ethanol	13,355	0,035	0,122	14,10	11,14	3,7200
n.a.	Isobutyrate RI	n.a.	n.a.	n.a.	n.a.	n.a.	n.a.
n.a.	Butyrate RI	n.a.	n.a.	n.a.	n.a.	n.a.	n.a.
**Total:**			**0,251**	**1,096**	**100,00**	**100,00**	

Chromeleon (c) Dionex
Version 7.2.10.25868

Peak Analysis

Injection Details

Injection Name:	**GOSFOSMUC t96 r3**	*Run Time (min):*	**20,00**
Vial Number:	**3:66**	*Injection Volume:*	**10,00**
Injection Type:	**Unknown**	*Channel:*	**RI_CH_1**
Calibration Level:		*Wavelength:*	**n.a.**
Instrument Method:	**Default method LC2030C 45 gr 20 min**	*Bandwidth:*	**n.a.**
Processing Method:	**Processing Method LC2030 45 gr**	*Dilution Factor:*	**1,0000**
Injection Date/Time:	**17/Jan/23 14:58**	*Sample Weight:*	**1,0000**

Chromatogram

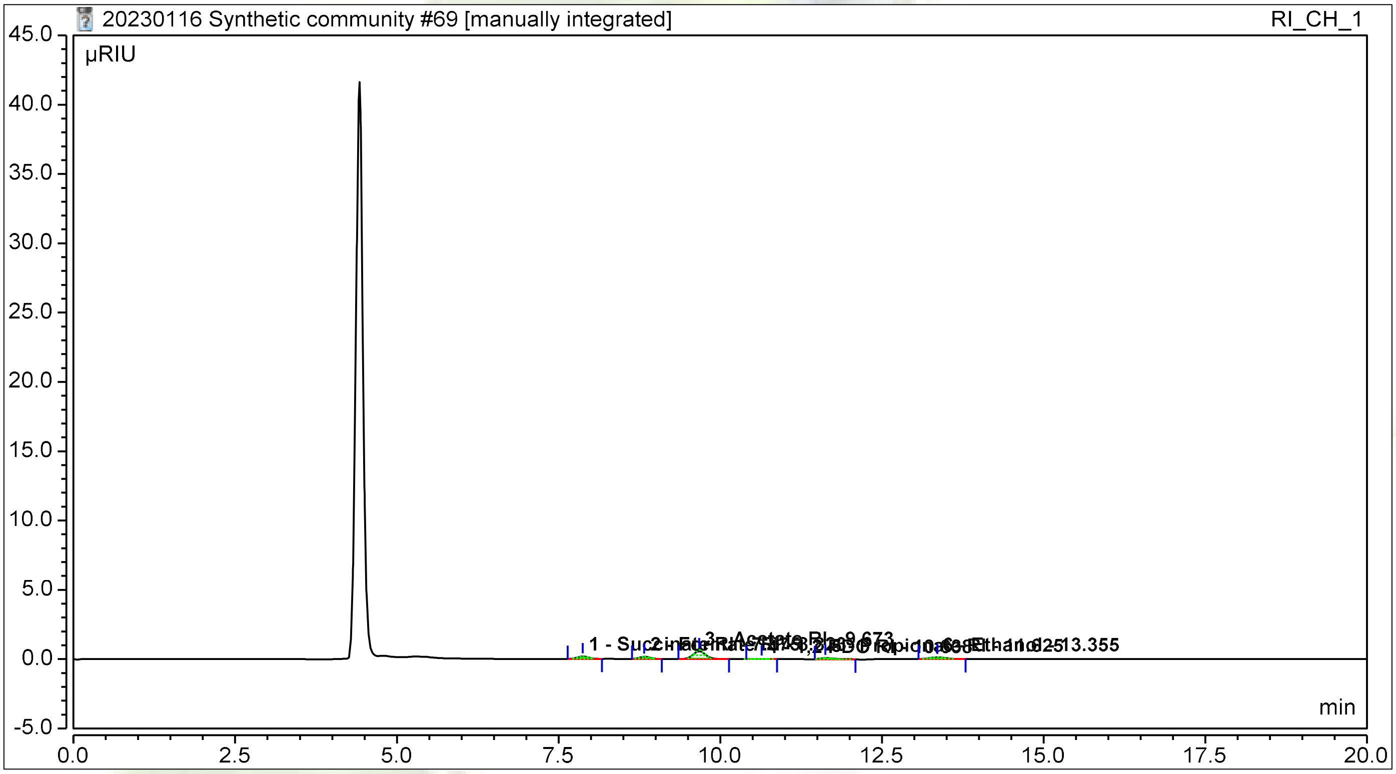

Peak Results

No.	Peak Name	Retention Time<br>min	Width (50%)<br>min	Type	Resolution (EP)	Asymmetry (EP)	Plates (EP)
n.a.	GlcNAc	n.a.	n.a.	n.a.	n.a.	n.a.	n.a.
n.a.	Citrate	n.a.	n.a.	n.a.	n.a.	n.a.	n.a.
n.a.	Glucose	n.a.	n.a.	n.a.	n.a.	n.a.	n.a.
n.a.	Galactose	n.a.	n.a.	n.a.	n.a.	n.a.	n.a.
n.a.	Fucose	n.a.	n.a.	n.a.	n.a.	n.a.	n.a.
1	Succinate RI	7,875	0,194	BMB*	2,94	1,05	9174
n.a.	Lactate RI	n.a.	n.a.	n.a.	n.a.	n.a.	n.a.
n.a.	glycerol	n.a.	n.a.	n.a.	n.a.	n.a.	n.a.
2	Formate RI	8,823	0,187	BMB*	2,51	1,12	12350
3	Acetate RI	9,673	0,213	BMB	2,45	1,07	11427
4	1,2 PDO RI	10,638	0,251	BMB*	2,40	1,01	9962
n.a.	1,3-PDO	n.a.	n.a.	n.a.	n.a.	n.a.	n.a.
5	Propionate RI	11,625	0,234	BMB*	3,99	1,94	13616
n.a.	1,3-PDO	n.a.	n.a.	n.a.	n.a.	n.a.	n.a.
n.a.	2-3 BDO	n.a.	n.a.	n.a.	n.a.	n.a.	n.a.
6	Ethanol	13,355	0,278	BMB*	n.a.	1,07	12813
n.a.	Isobutyrate RI	n.a.	n.a.	n.a.	n.a.	n.a.	n.a.
n.a.	Butyrate RI	n.a.	n.a.	n.a.	n.a.	n.a.	n.a.

Chromatogram and SST Results

Injection Details

Injection Name:	**GOSFOSMUC t96 r3**	*Run Time (min):*	**20,00**
Vial Number:	**3:66**	*Injection Volume:*	**10,00**
Injection Type:	**Unknown**	*Channel:*	**RI_CH_1**
Calibration Level:		*Wavelength:*	**n.a.**
Instrument Method:	**Default method LC2030C 45 gr 20 min**	*Bandwidth:*	**n.a.**
Processing Method:	**Processing Method LC2030 45 gr**	*Dilution Factor:*	**1,0000**
Injection Date/Time:	**17/Jan/23 14:58**	*Sample Weight:*	**1,0000**

Chromatogram

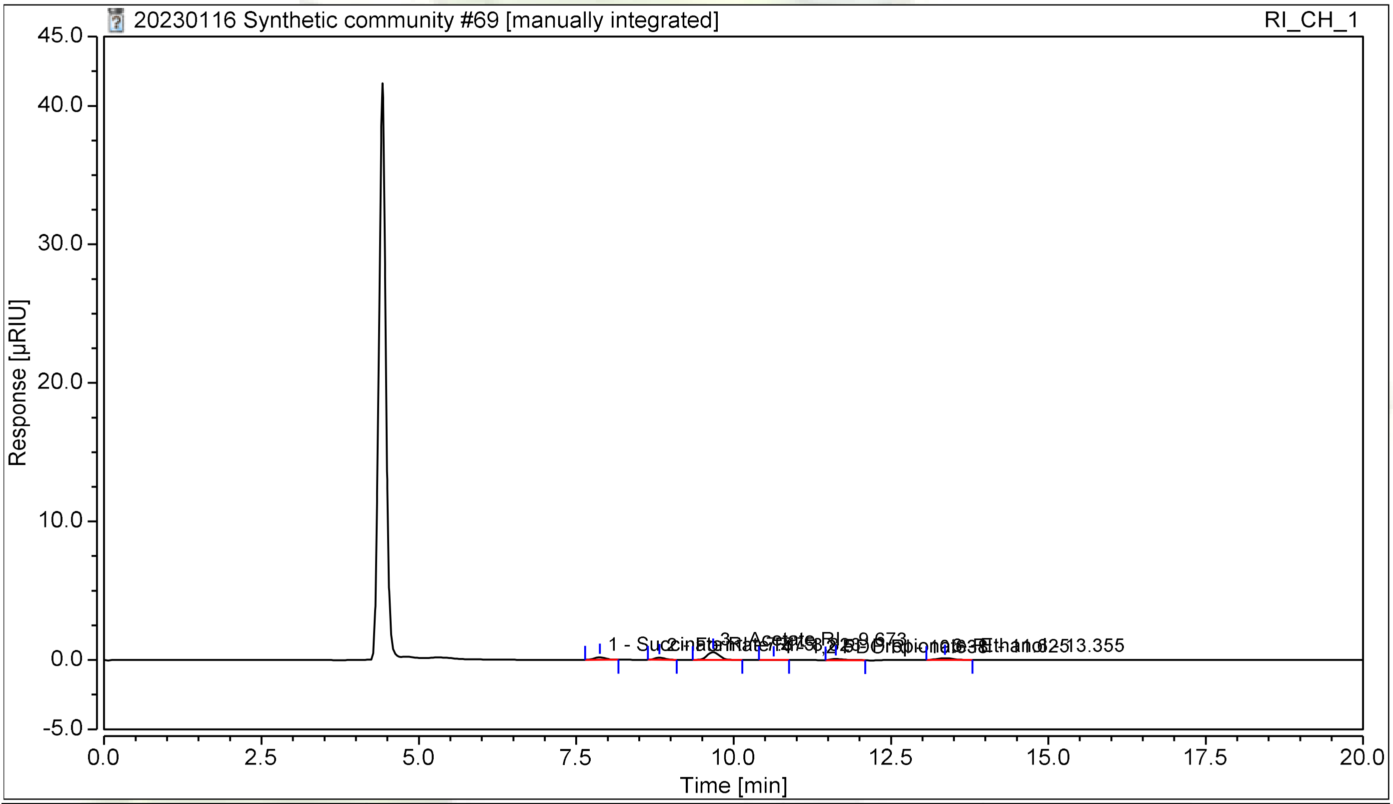

SST Results

No.	Name	Inj.Condition	Peak	Test Result	Injection
**Number of executed test cases:**	**n.a.**		**Total Result:**	**Passed**	

Chromatogram and Results

Injection Details

Injection Name:	**GOSFOSMUC t120 r1**	*Run Time (min):*	**20,00**
Vial Number:	**3:67**	*Injection Volume:*	**10,00**
Injection Type:	**Unknown**	*Channel:*	**RI_CH_1**
Calibration Level:		*Wavelength:*	**n.a.**
Instrument Method:	**Default method LC2030C 45 gr 20 min**	*Bandwidth:*	**n.a.**
Processing Method:	**Processing Method LC2030 45 gr**	*Dilution Factor:*	**1,0000**
Injection Date/Time:	**17/Jan/23 15:18**	*Sample Weight:*	**1,0000**

Chromatogram

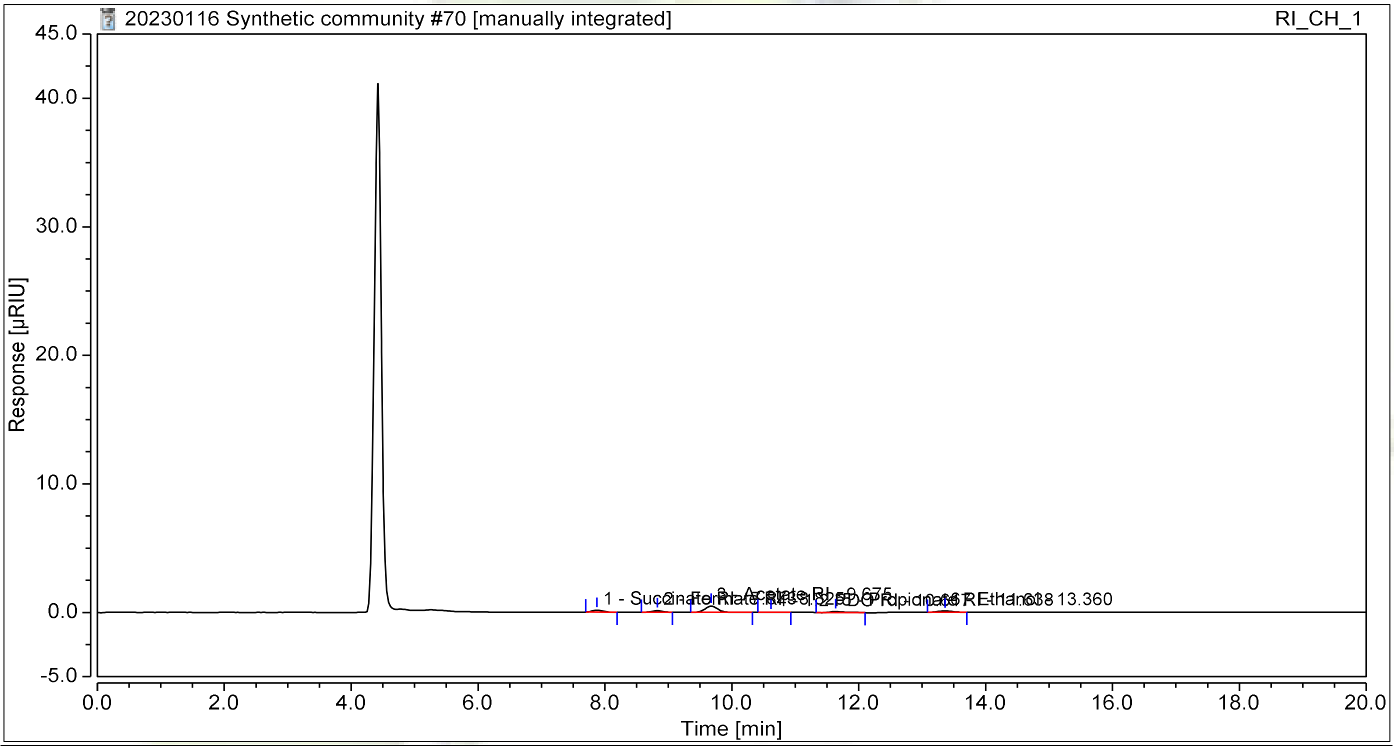

Integration Results

No.	Peak Name	Retention Time min	Area µRIU*min	Height µRIU	Relative Area %	Relative Height %	Amount
n.a.	GlcNAc	n.a.	n.a.	n.a.	n.a.	n.a.	n.a.
n.a.	Citrate	n.a.	n.a.	n.a.	n.a.	n.a.	n.a.
n.a.	Glucose	n.a.	n.a.	n.a.	n.a.	n.a.	n.a.
n.a.	Galactose	n.a.	n.a.	n.a.	n.a.	n.a.	n.a.
n.a.	Fucose	n.a.	n.a.	n.a.	n.a.	n.a.	n.a.
1	Succinate RI	7,873	0,028	0,144	12,86	15,30	0,5693
n.a.	Lactate RI	n.a.	n.a.	n.a.	n.a.	n.a.	n.a.
n.a.	glycerol	n.a.	n.a.	n.a.	n.a.	n.a.	n.a.
2	Formate RI	8,825	0,025	0,127	11,61	13,51	2,5488
3	Acetate RI	9,675	0,111	0,487	51,74	51,70	6,8834
4	1,2 PDO RI	10,617	0,002	0,009	1,05	0,99	0,0686
n.a.	1,3-PDO	n.a.	n.a.	n.a.	n.a.	n.a.	n.a.
5	Propionate RI	11,638	0,020	0,073	9,45	7,80	0,8479
n.a.	1,3-PDO	n.a.	n.a.	n.a.	n.a.	n.a.	n.a.
n.a.	2-3 BDO	n.a.	n.a.	n.a.	n.a.	n.a.	n.a.
6	Ethanol	13,360	0,029	0,101	13,29	10,70	2,9912
n.a.	Isobutyrate RI	n.a.	n.a.	n.a.	n.a.	n.a.	n.a.
n.a.	Butyrate RI	n.a.	n.a.	n.a.	n.a.	n.a.	n.a.
**Total:**			**0,215**	**0,942**	**100,00**	**100,00**	

Chromeleon (c) Dionex
Version 7.2.10.25868

Peak Analysis

Injection Details

Injection Name:	**GOSFOSMUC t120 r1**	*Run Time (min):*	**20,00**
Vial Number:	**3:67**	*Injection Volume:*	**10,00**
Injection Type:	**Unknown**	*Channel:*	**RI_CH_1**
Calibration Level:		*Wavelength:*	**n.a.**
Instrument Method:	**Default method LC2030C 45 gr 20 min**	*Bandwidth:*	**n.a.**
Processing Method:	**Processing Method LC2030 45 gr**	*Dilution Factor:*	**1,0000**
Injection Date/Time:	**17/Jan/23 15:18**	*Sample Weight:*	**1,0000**

Chromatogram

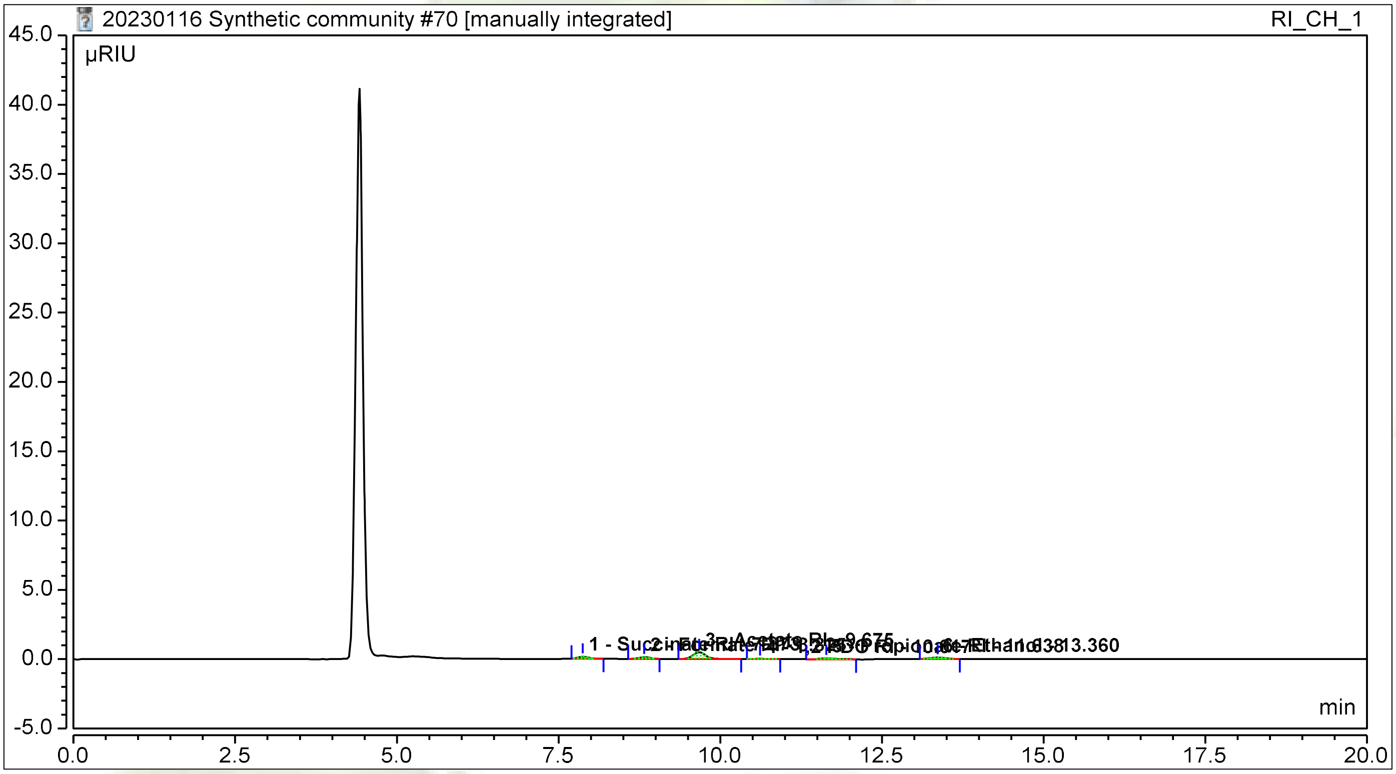

Peak Results

No.	Peak Name	Retention Time min	Width (50%) min	Type	Resolution (EP)	Asymmetry (EP)	Plates (EP)
n.a.	GlcNAc	n.a.	n.a.	n.a.	n.a.	n.a.	n.a.
n.a.	Citrate	n.a.	n.a.	n.a.	n.a.	n.a.	n.a.
n.a.	Glucose	n.a.	n.a.	n.a.	n.a.	n.a.	n.a.
n.a.	Galactose	n.a.	n.a.	n.a.	n.a.	n.a.	n.a.
n.a.	Fucose	n.a.	n.a.	n.a.	n.a.	n.a.	n.a.
1	Succinate RI	7,873	0,186	BMB*	3,00	1,11	9896
n.a.	Lactate RI	n.a.	n.a.	n.a.	n.a.	n.a.	n.a.
n.a.	glycerol	n.a.	n.a.	n.a.	n.a.	n.a.	n.a.
2	Formate RI	8,825	0,188	BMB*	2,50	1,04	12252
3	Acetate RI	9,675	0,213	BMB	2,45	1,06	11391
4	1,2 PDO RI	10,617	0,240	BMB*	2,51	1,35	10885
n.a.	1,3-PDO	n.a.	n.a.	n.a.	n.a.	n.a.	n.a.
5	Propionate RI	11,638	0,242	BMB*	3,94	1,90	12851
n.a.	1,3-PDO	n.a.	n.a.	n.a.	n.a.	n.a.	n.a.
n.a.	2-3 BDO	n.a.	n.a.	n.a.	n.a.	n.a.	n.a.
6	Ethanol	13,360	0,274	BMB*	n.a.	1,05	13150
n.a.	Isobutyrate RI	n.a.	n.a.	n.a.	n.a.	n.a.	n.a.
n.a.	Butyrate RI	n.a.	n.a.	n.a.	n.a.	n.a.	n.a.

Chromatogram and SST Results

Injection Details

Injection Name:	**GOSFOSMUC t120 r1**	*Run Time (min):*	**20,00**
Vial Number:	**3:67**	*Injection Volume:*	**10,00**
Injection Type:	**Unknown**	*Channel:*	**RI_CH_1**
Calibration Level:		*Wavelength:*	**n.a.**
Instrument Method:	**Default method LC2030C 45 gr 20 min**	*Bandwidth:*	**n.a.**
Processing Method:	**Processing Method LC2030 45 gr**	*Dilution Factor:*	**1,0000**
Injection Date/Time:	**17/Jan/23 15:18**	*Sample Weight:*	**1,0000**

Chromatogram

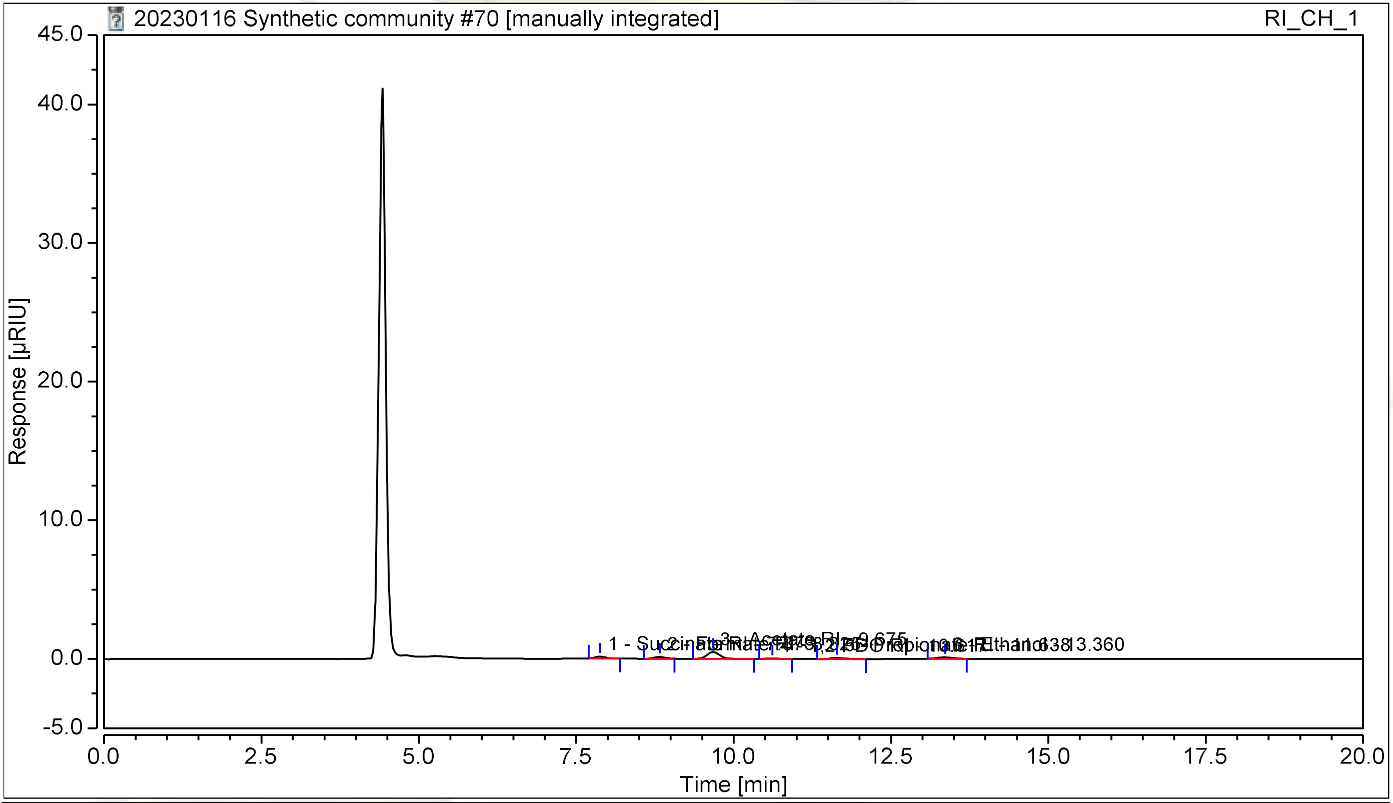

SST Results

No.	Name	Inj.Condition	Peak	Test Result	Injection
**Number of executed test cases:**	**n.a.**		**Total Result:**	**Passed**	

Chromeleon (c) Dionex
Version 7.2.10.25868

Chromatogram and Results

Injection Details

Injection Name:	**GOSFOSMUC t120 r2**	*Run Time (min):*	**20,00**
Vial Number:	**3:68**	*Injection Volume:*	**10,00**
Injection Type:	**Unknown**	*Channel:*	**RI_CH_1**
Calibration Level:		*Wavelength:*	**n.a.**
Instrument Method:	**Default method LC2030C 45 gr 20 min**	*Bandwidth:*	**n.a.**
Processing Method:	**Processing Method LC2030 45 gr**	*Dilution Factor:*	**1,0000**
Injection Date/Time:	**17/Jan/23 15:39**	*Sample Weight:*	**1,0000**

Chromatogram

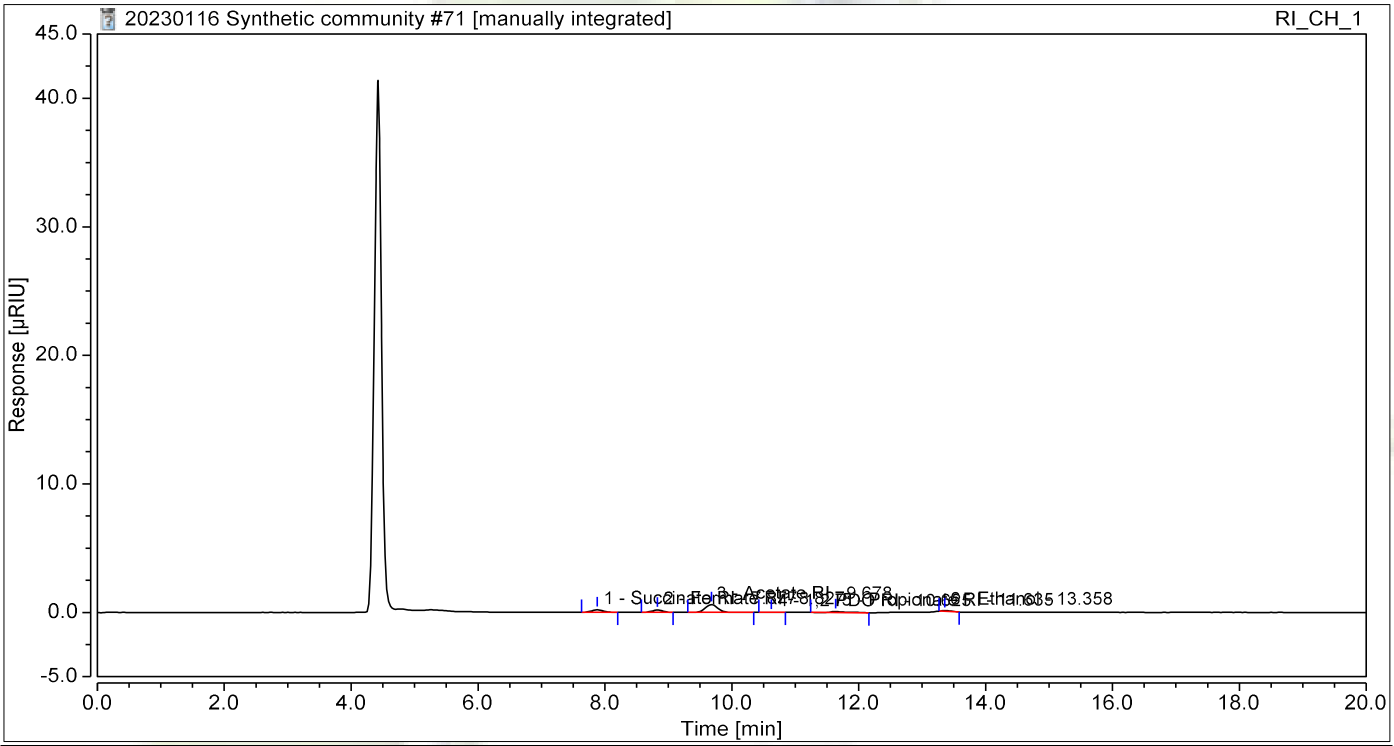

Integration Results

No.	Peak Name	Retention Time min	Area µRIU*min	Height µRIU	Relative Area %	Relative Height %	Amount
n.a.	GlcNAc	n.a.	n.a.	n.a.	n.a.	n.a.	n.a.
n.a.	Citrate	n.a.	n.a.	n.a.	n.a.	n.a.	n.a.
n.a.	Glucose	n.a.	n.a.	n.a.	n.a.	n.a.	n.a.
n.a.	Galactose	n.a.	n.a.	n.a.	n.a.	n.a.	n.a.
n.a.	Fucose	n.a.	n.a.	n.a.	n.a.	n.a.	n.a.
1	Succinate RI	7,877	0,038	0,188	15,60	17,13	0,7784
n.a.	Lactate RI	n.a.	n.a.	n.a.	n.a.	n.a.	n.a.
n.a.	glycerol	n.a.	n.a.	n.a.	n.a.	n.a.	n.a.
2	Formate RI	8,827	0,034	0,171	14,01	15,58	3,4651
3	Acetate RI	9,678	0,137	0,599	56,53	54,73	8,4736
4	1,2 PDO RI	10,625	0,001	0,007	0,59	0,64	0,0435
n.a.	1,3-PDO	n.a.	n.a.	n.a.	n.a.	n.a.	n.a.
5	Propionate RI	11,635	0,021	0,071	8,49	6,50	0,8586
n.a.	1,3-PDO	n.a.	n.a.	n.a.	n.a.	n.a.	n.a.
n.a.	2-3 BDO	n.a.	n.a.	n.a.	n.a.	n.a.	n.a.
6	Ethanol	13,358	0,012	0,059	4,78	5,42	1,2122
n.a.	Isobutyrate RI	n.a.	n.a.	n.a.	n.a.	n.a.	n.a.
n.a.	Butyrate RI	n.a.	n.a.	n.a.	n.a.	n.a.	n.a.
**Total:**			**0,242**	**1,095**	**100,00**	**100,00**	

Chromeleon (c) Dionex
Version 7.2.10.25868

Peak Analysis

Injection Details

Injection Name:	**GOSFOSMUC t120 r2**	*Run Time (min):*	**20,00**
Vial Number:	**3:68**	*Injection Volume:*	**10,00**
Injection Type:	**Unknown**	*Channel:*	**RI_CH_1**
Calibration Level:		*Wavelength:*	**n.a.**
Instrument Method:	**Default method LC2030C 45 gr 20 min**	*Bandwidth:*	**n.a.**
Processing Method:	**Processing Method LC2030 45 gr**	*Dilution Factor:*	**1,0000**
Injection Date/Time:	**17/Jan/23 15:39**	*Sample Weight:*	**1,0000**

Chromatogram

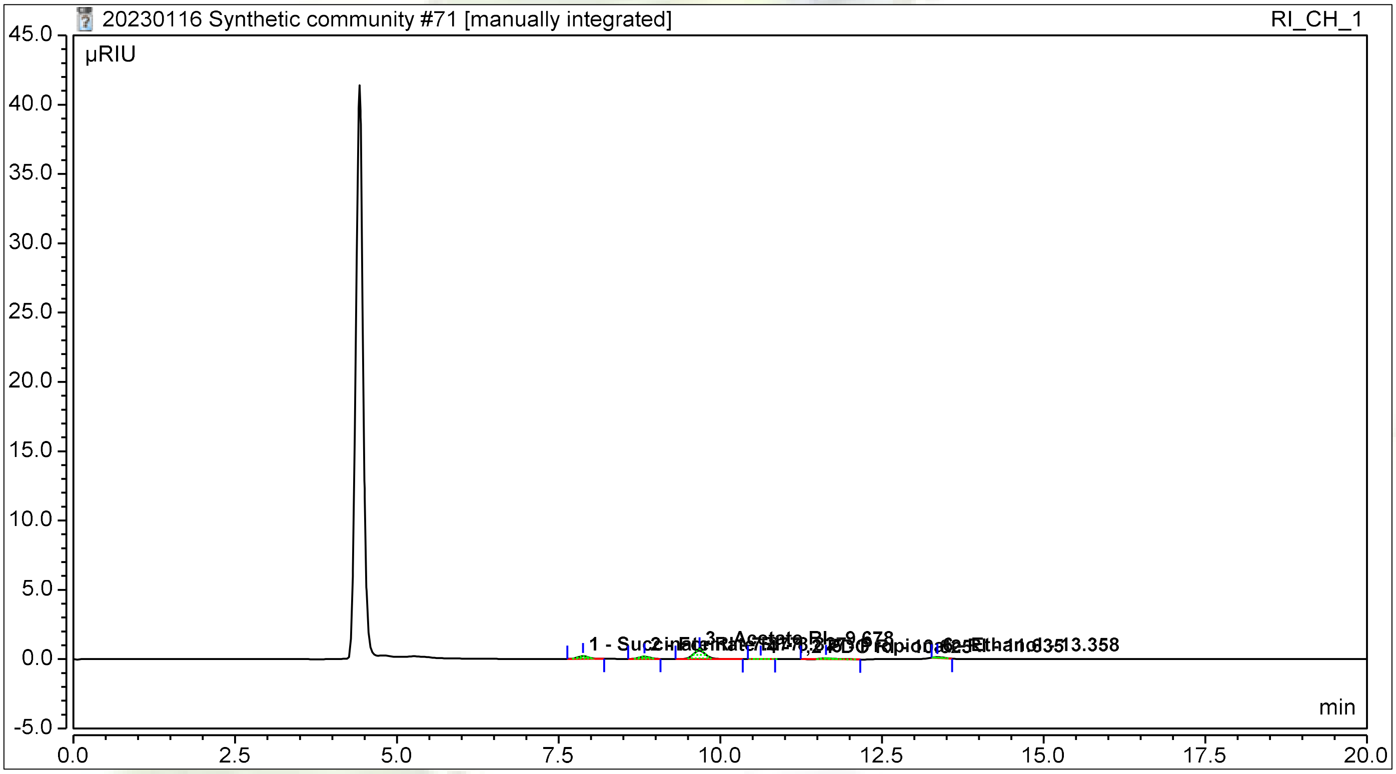

Peak Results

No.	Peak Name	Retention Time min	Width (50%) min	Type	Resolution (EP)	Asymmetry (EP)	Plates (EP)
n.a.	GlcNAc	n.a.	n.a.	n.a.	n.a.	n.a.	n.a.
n.a.	Citrate	n.a.	n.a.	n.a.	n.a.	n.a.	n.a.
n.a.	Glucose	n.a.	n.a.	n.a.	n.a.	n.a.	n.a.
n.a.	Galactose	n.a.	n.a.	n.a.	n.a.	n.a.	n.a.
n.a.	Fucose	n.a.	n.a.	n.a.	n.a.	n.a.	n.a.
1	Succinate RI	7,877	0,193	BMB*	2,94	1,03	9250
n.a.	Lactate RI	n.a.	n.a.	n.a.	n.a.	n.a.	n.a.
n.a.	glycerol	n.a.	n.a.	n.a.	n.a.	n.a.	n.a.
2	Formate RI	8,827	0,189	BMB*	2,50	1,04	12101
3	Acetate RI	9,678	0,214	BMB	2,66	1,06	11380
4	1,2 PDO RI	10,625	0,207	BMB*	2,62	0,97	14641
n.a.	1,3-PDO	n.a.	n.a.	n.a.	n.a.	n.a.	n.a.
5	Propionate RI	11,635	0,249	BMB*	4,61	2,22	12136
n.a.	1,3-PDO	n.a.	n.a.	n.a.	n.a.	n.a.	n.a.
n.a.	2-3 BDO	n.a.	n.a.	n.a.	n.a.	n.a.	n.a.
6	Ethanol	13,358	0,192	BMB*	n.a.	1,67	26697
n.a.	Isobutyrate RI	n.a.	n.a.	n.a.	n.a.	n.a.	n.a.
n.a.	Butyrate RI	n.a.	n.a.	n.a.	n.a.	n.a.	n.a.

Chromatogram and SST Results

Injection Details

Injection Name:	**GOSFOSMUC t120 r2**	*Run Time (min):*	**20,00**
Vial Number:	**3:68**	*Injection Volume:*	**10,00**
Injection Type:	**Unknown**	*Channel:*	**RI_CH_1**
Calibration Level:		*Wavelength:*	**n.a.**
Instrument Method:	**Default method LC2030C 45 gr 20 min**	*Bandwidth:*	**n.a.**
Processing Method:	**Processing Method LC2030 45 gr**	*Dilution Factor:*	**1,0000**
Injection Date/Time:	**17/Jan/23 15:39**	*Sample Weight:*	**1,0000**

Chromatogram

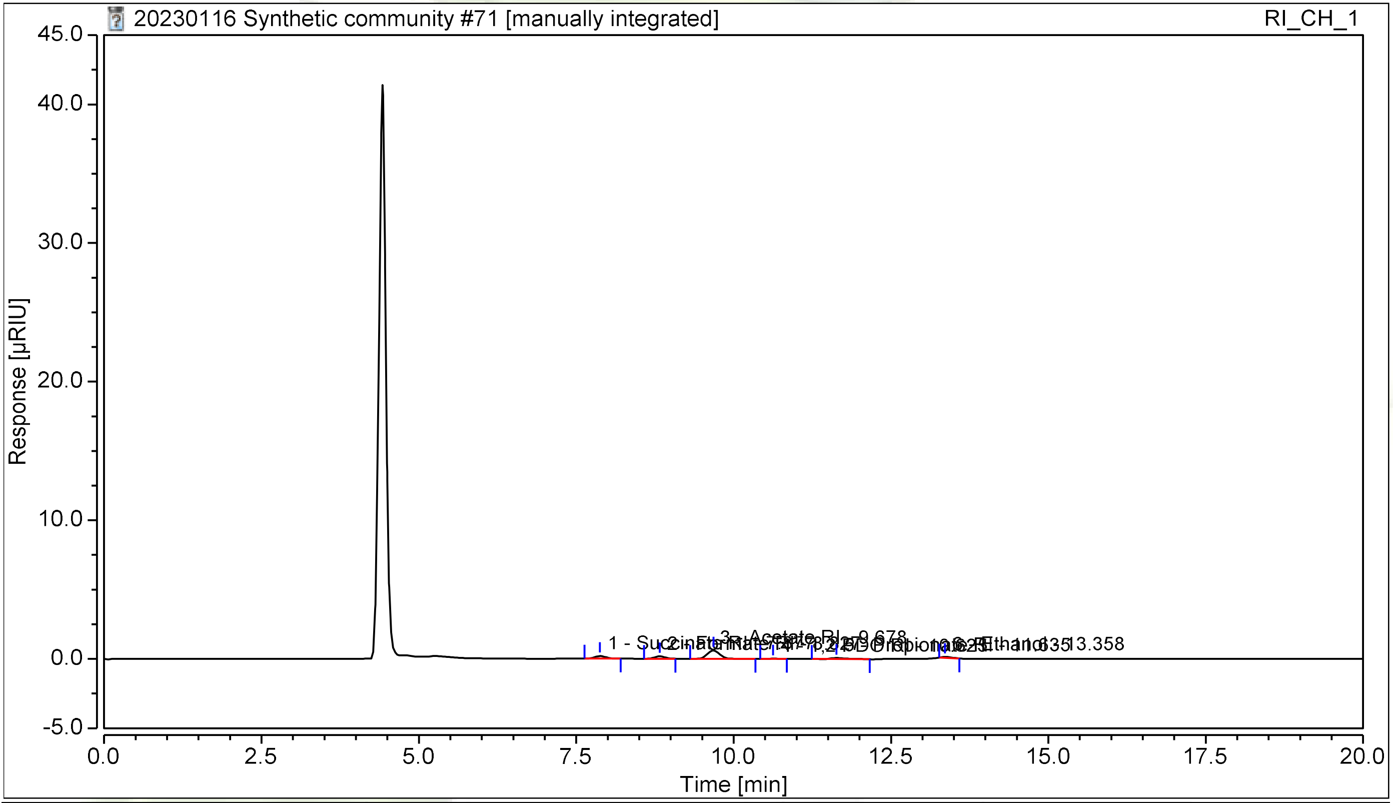

SST Results

No.	Name	Inj.Condition	Peak	Test Result	Injection
**Number of executed test cases:**	**n.a.**		**Total Result:**	**Passed**	

Chromatogram and Results

Injection Details

Injection Name:	**GOSFOSMUC t120 r3**	*Run Time (min):*	**20,00**
Vial Number:	**3:69**	*Injection Volume:*	**10,00**
Injection Type:	**Unknown**	*Channel:*	**RI_CH_1**
Calibration Level:		*Wavelength:*	**n.a.**
Instrument Method:	**Default method LC2030C 45 gr 20 min**	*Bandwidth:*	**n.a.**
Processing Method:	**Processing Method LC2030 45 gr**	*Dilution Factor:*	**1,0000**
Injection Date/Time:	**17/Jan/23 15:59**	*Sample Weight:*	**1,0000**

Chromatogram

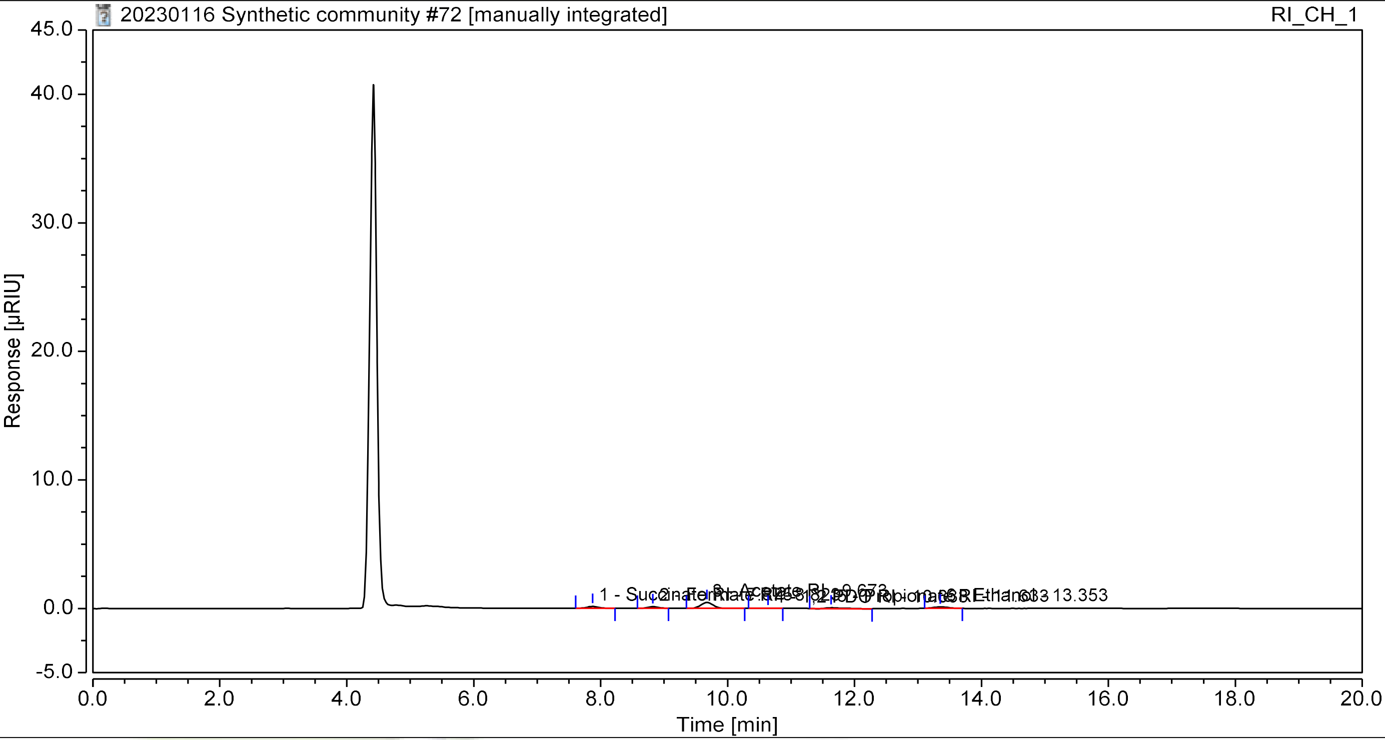

Integration Results

No.	Peak Name	Retention Time min	Area µRIU*min	Height µRIU	Relative Area %	Relative Height %	Amount
n.a.	GlcNAc	n.a.	n.a.	n.a.	n.a.	n.a.	n.a.
n.a.	Citrate	n.a.	n.a.	n.a.	n.a.	n.a.	n.a.
n.a.	Glucose	n.a.	n.a.	n.a.	n.a.	n.a.	n.a.
n.a.	Galactose	n.a.	n.a.	n.a.	n.a.	n.a.	n.a.
n.a.	Fucose	n.a.	n.a.	n.a.	n.a.	n.a.	n.a.
1	Succinate RI	7,875	0,028	0,140	13,68	15,51	0,5786
n.a.	Lactate RI	n.a.	n.a.	n.a.	n.a.	n.a.	n.a.
n.a.	glycerol	n.a.	n.a.	n.a.	n.a.	n.a.	n.a.
2	Formate RI	8,823	0,026	0,129	12,47	14,32	2,6149
3	Acetate RI	9,673	0,104	0,453	50,65	50,18	6,4351
4	1,2 PDO RI	10,638	0,001	0,005	0,64	0,59	0,0400
n.a.	1,3-PDO	n.a.	n.a.	n.a.	n.a.	n.a.	n.a.
5	Propionate RI	11,633	0,015	0,064	7,43	7,09	0,6365
n.a.	1,3-PDO	n.a.	n.a.	n.a.	n.a.	n.a.	n.a.
n.a.	2-3 BDO	n.a.	n.a.	n.a.	n.a.	n.a.	n.a.
6	Ethanol	13,353	0,031	0,111	15,13	12,30	3,2513
n.a.	Isobutyrate RI	n.a.	n.a.	n.a.	n.a.	n.a.	n.a.
n.a.	Butyrate RI	n.a.	n.a.	n.a.	n.a.	n.a.	n.a.
**Total:**			**0,205**	**0,903**	**100,00**	**100,00**	

Chromeleon (c) Dionex
Version 7.2.10.25868

Peak Analysis

Injection Details

Injection Name:	**GOSFOSMUC t120 r3**	*Run Time (min):*	**20,00**
Vial Number:	**3:69**	*Injection Volume:*	**10,00**
Injection Type:	**Unknown**	*Channel:*	**RI_CH_1**
Calibration Level:		*Wavelength:*	**n.a.**
Instrument Method:	**Default method LC2030C 45 gr 20 min**	*Bandwidth:*	**n.a.**
Processing Method:	**Processing Method LC2030 45 gr**	*Dilution Factor:*	**1,0000**
Injection Date/Time:	**17/Jan/23 15:59**	*Sample Weight:*	**1,0000**

Chromatogram

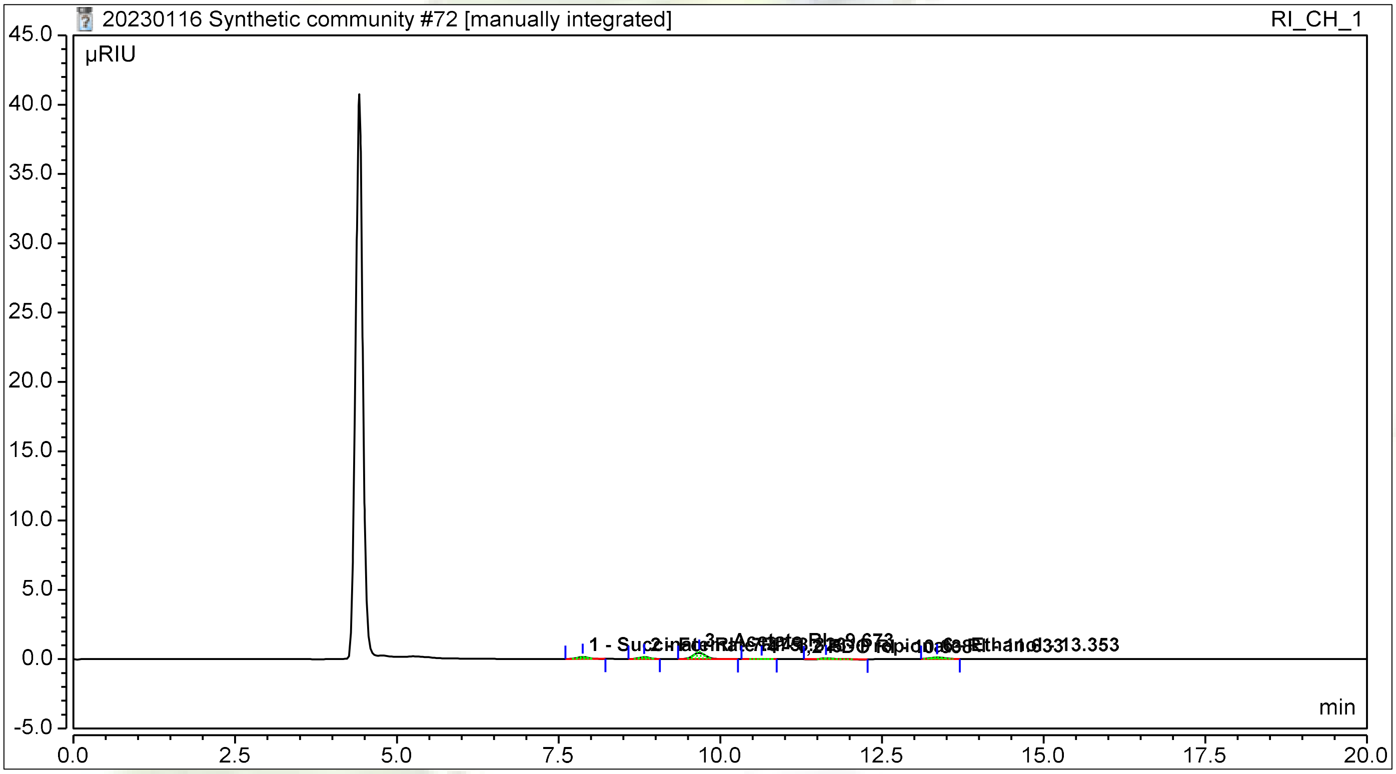

Peak Results

No.	Peak Name	Retention Time min	Width (50%) min	Type	Resolution (EP)	Asymmetry (EP)	Plates (EP)
n.a.	GlcNAc	n.a.	n.a.	n.a.	n.a.	n.a.	n.a.
n.a.	Citrate	n.a.	n.a.	n.a.	n.a.	n.a.	n.a.
n.a.	Glucose	n.a.	n.a.	n.a.	n.a.	n.a.	n.a.
n.a.	Galactose	n.a.	n.a.	n.a.	n.a.	n.a.	n.a.
n.a.	Fucose	n.a.	n.a.	n.a.	n.a.	n.a.	n.a.
1	Succinate RI	7,875	0,191	BMB*	2,94	1,02	9402
n.a.	Lactate RI	n.a.	n.a.	n.a.	n.a.	n.a.	n.a.
n.a.	glycerol	n.a.	n.a.	n.a.	n.a.	n.a.	n.a.
2	Formate RI	8,823	0,189	BMB*	2,48	1,04	12014
3	Acetate RI	9,673	0,214	BMB	2,52	1,07	11293
4	1,2 PDO RI	10,638	0,238	BMB*	2,49	0,98	11086
n.a.	1,3-PDO	n.a.	n.a.	n.a.	n.a.	n.a.	n.a.
5	Propionate RI	11,633	0,234	BMB*	4,04	2,15	13735
n.a.	1,3-PDO	n.a.	n.a.	n.a.	n.a.	n.a.	n.a.
n.a.	2-3 BDO	n.a.	n.a.	n.a.	n.a.	n.a.	n.a.
6	Ethanol	13,353	0,269	BMB*	n.a.	1,11	13617
n.a.	Isobutyrate RI	n.a.	n.a.	n.a.	n.a.	n.a.	n.a.
n.a.	Butyrate RI	n.a.	n.a.	n.a.	n.a.	n.a.	n.a.

Chromeleon (c) Dionex
Version 7.2.10.25868

Chromatogram and SST Results

Injection Details

Injection Name:	**GOSFOSMUC t120 r3**	*Run Time (min):*	**20,00**
Vial Number:	**3:69**	*Injection Volume:*	**10,00**
Injection Type:	**Unknown**	*Channel:*	**RI_CH_1**
Calibration Level:		*Wavelength:*	**n.a.**
Instrument Method:	**Default method LC2030C 45 gr 20 min**	*Bandwidth:*	**n.a.**
Processing Method:	**Processing Method LC2030 45 gr**	*Dilution Factor:*	**1,0000**
Injection Date/Time:	**17/Jan/23 15:59**	*Sample Weight:*	**1,0000**

Chromatogram

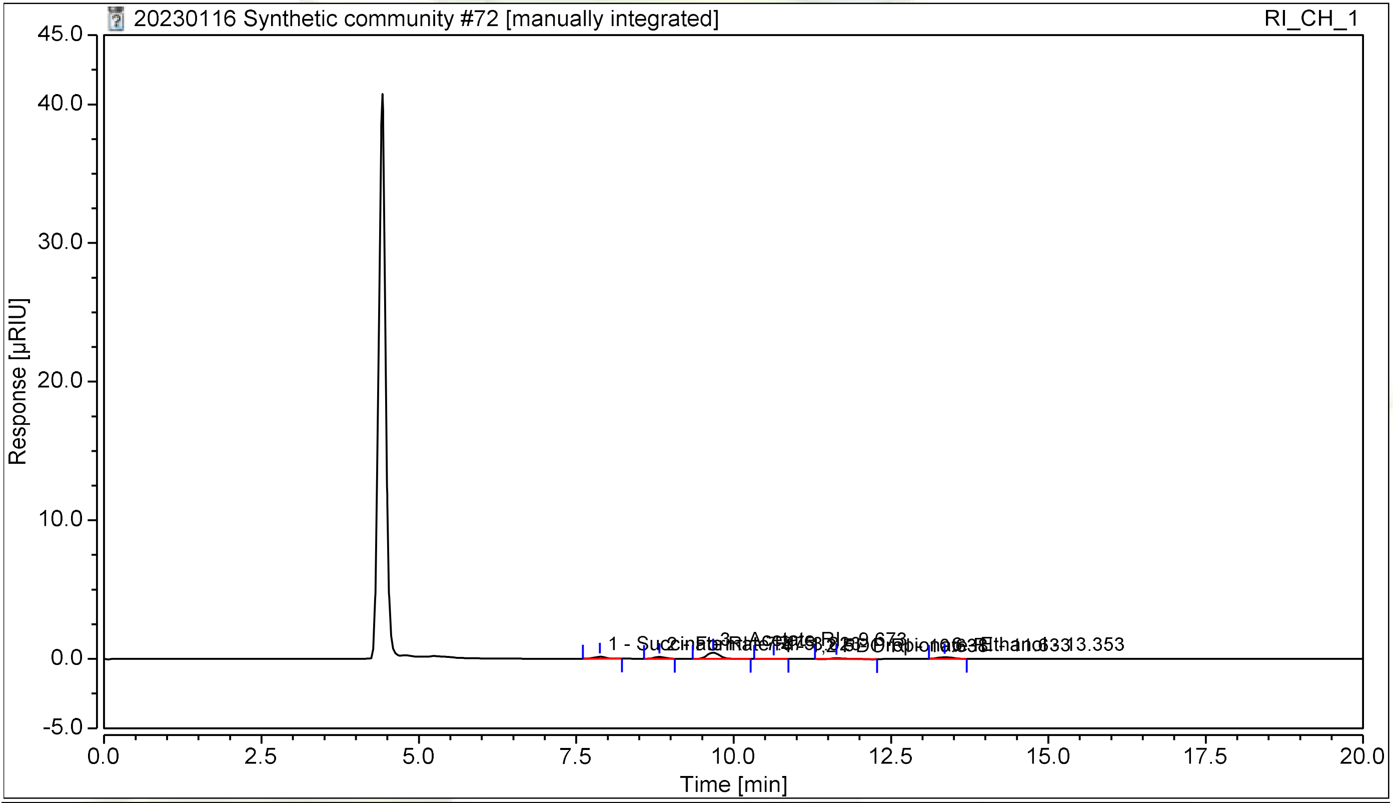

SST Results

No.	Name	Inj.Condition	Peak	Test Result	Injection
**Number of executed test cases:**	**n.a.**		**Total Result:**	**Passed**	

Chromatogram and Results

Injection Details

Injection Name:	**Glucose, fructose,glycerol,2,3-butandieol (11.1 mM**	*Run Time (min):*	**20,00**
Vial Number:	**3:85**	*Injection Volume:*	**1,00**
Injection Type:	**Calibration Standard**	*Channel:*	**RI_CH_1**
Calibration Level:	**2**	*Wavelength:*	**n.a.**
Instrument Method:	**Default method LC2030C 45 gr 20 min**	*Bandwidth:*	**n.a.**
Processing Method:	**Processing Method LC2030 45 gr**	*Dilution Factor:*	**1,0000**
Injection Date/Time:	**17/Jan/23 16:19**	*Sample Weight:*	**1,0000**

Chromatogram

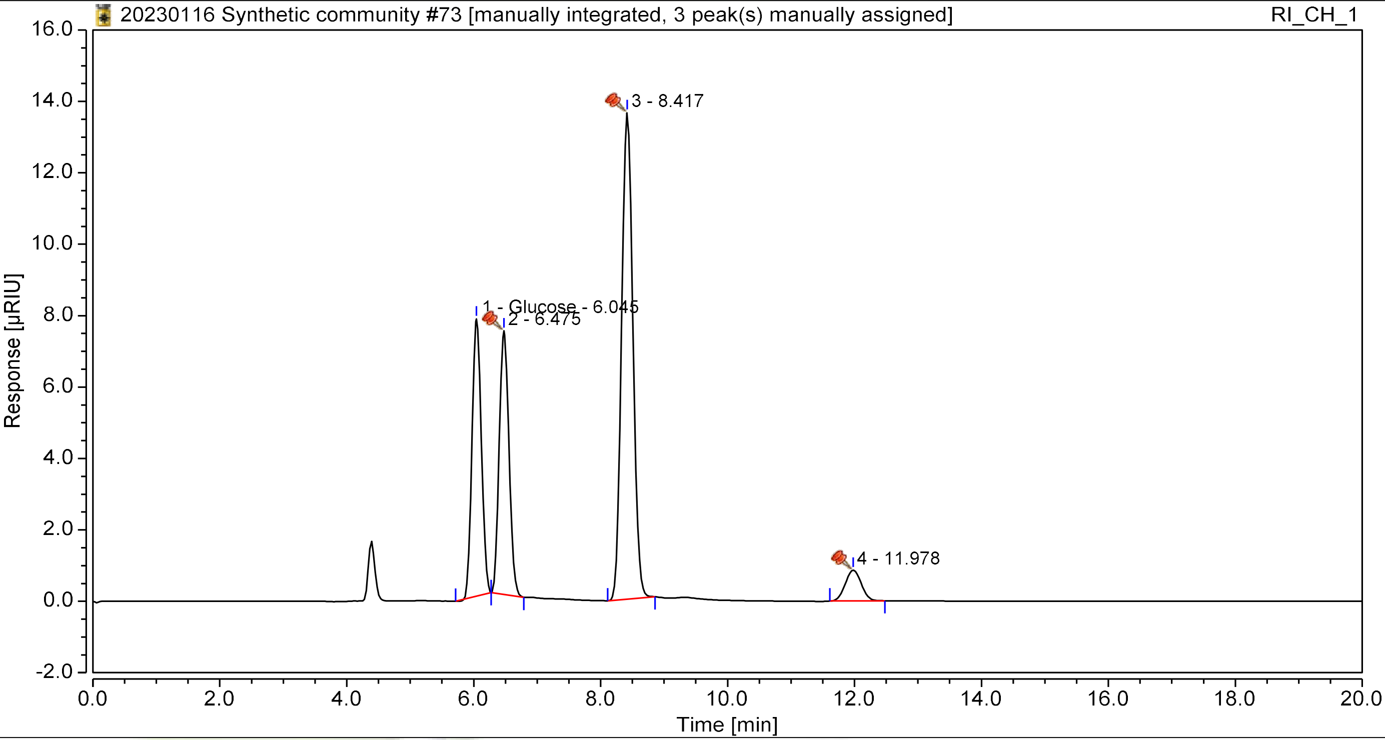

Integration Results

No.	Peak Name	Retention Time min	Area µRIU*min	Height µRIU	Relative Area %	Relative Height %	Amount
n.a.	GlcNAc	n.a.	n.a.	n.a.	n.a.	n.a.	n.a.
n.a.	Citrate	n.a.	n.a.	n.a.	n.a.	n.a.	n.a.
1	Glucose	6,045	1,320	7,763	23,19	26,20	10,6931
n.a.	Galactose	n.a.	n.a.	n.a.	n.a.	n.a.	n.a.
2		6,475	1,308	7,382	22,99	24,91	n.a.
n.a.	Fucose	n.a.	n.a.	n.a.	n.a.	n.a.	n.a.
n.a.	Succinate RI	n.a.	n.a.	n.a.	n.a.	n.a.	n.a.
n.a.	Lactate RI	n.a.	n.a.	n.a.	n.a.	n.a.	n.a.
3		8,417	2,811	13,623	49,40	45,98	n.a.
n.a.	glycerol	n.a.	n.a.	n.a.	n.a.	n.a.	n.a.
n.a.	Formate RI	n.a.	n.a.	n.a.	n.a.	n.a.	n.a.
n.a.	Acetate RI	n.a.	n.a.	n.a.	n.a.	n.a.	n.a.
n.a.	1,2 PDO RI	n.a.	n.a.	n.a.	n.a.	n.a.	n.a.
n.a.	1,3-PDO	n.a.	n.a.	n.a.	n.a.	n.a.	n.a.
n.a.	Propionate RI	n.a.	n.a.	n.a.	n.a.	n.a.	n.a.
n.a.	1,3-PDO	n.a.	n.a.	n.a.	n.a.	n.a.	n.a.
4		11,978	0,251	0,862	4,41	2,91	n.a.
n.a.	2-3 BDO	n.a.	n.a.	n.a.	n.a.	n.a.	n.a.
n.a.	Ethanol	n.a.	n.a.	n.a.	n.a.	n.a.	n.a.
n.a.	Isobutyrate RI	n.a.	n.a.	n.a.	n.a.	n.a.	n.a.
n.a.	Butyrate RI	n.a.	n.a.	n.a.	n.a.	n.a.	n.a.

**Total:**	**5,690**	**29,630**	**100,00**	**100,00**

Chromeleon (c) Dionex
Version 7.2.10.25868

Peak Analysis

Injection Details

Injection Name:	**Glucose, fructose,glycerol,2,3-butandieol (**	*Run Time (min):*	**20,00**
Vial Number:	**3:85**	*Injection Volume:*	**1,00**
Injection Type:	**Calibration Standard**	*Channel:*	**RI_CH_1**
Calibration Level:	**2**	*Wavelength:*	**n.a.**
Instrument Method:	**Default method LC2030C 45 gr 20 min**	*Bandwidth:*	**n.a.**
Processing Method:	**Processing Method LC2030 45 gr**	*Dilution Factor:*	**1,0000**
Injection Date/Time:	**17/Jan/23 16:19**	*Sample Weight:*	**1,0000**

Chromatogram

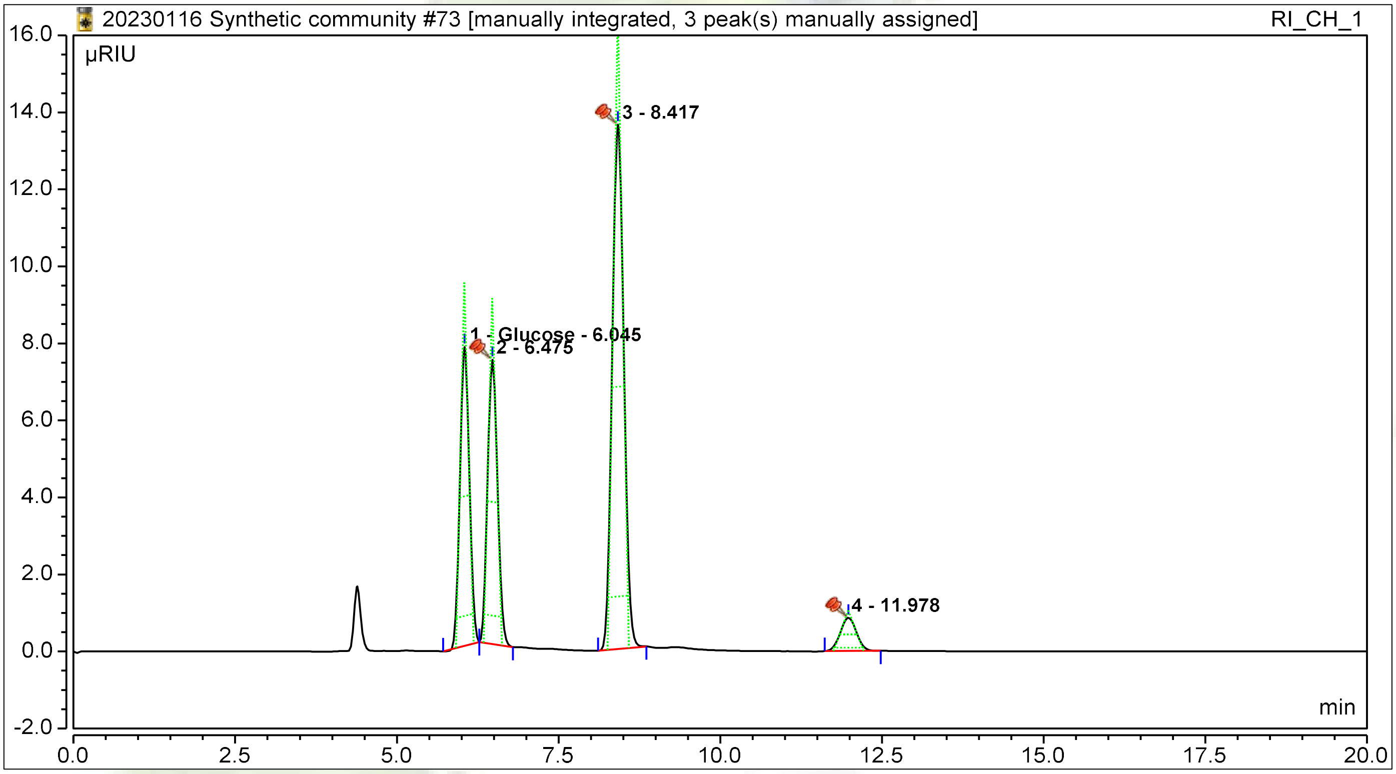

Peak Results

No.	Peak Name	Retention Time min	Width (50%) min	Type	Resolution (EP)	Asymmetry (EP)	Plates (EP)
n.a.	GlcNAc	n.a.	n.a.	n.a.	n.a.	n.a.	n.a.
n.a.	Citrate	n.a.	n.a.	n.a.	n.a.	n.a.	n.a.
1	Glucose	6,045	0,162	BMB*	1,54	1,07	7742
n.a.	Galactose	n.a.	n.a.	n.a.	n.a.	n.a.	n.a.
2		6,475	0,167	BMB^	6,33	1,12	8279
n.a.	Fucose	n.a.	n.a.	n.a.	n.a.	n.a.	n.a.
n.a.	Succinate RI	n.a.	n.a.	n.a.	n.a.	n.a.	n.a.
n.a.	Lactate RI	n.a.	n.a.	n.a.	n.a.	n.a.	n.a.
3		8,417	0,194	BMB*^	8,95	1,08	10399
n.a.	glycerol	n.a.	n.a.	n.a.	n.a.	n.a.	n.a.
n.a.	Formate RI	n.a.	n.a.	n.a.	n.a.	n.a.	n.a.
n.a.	Acetate RI	n.a.	n.a.	n.a.	n.a.	n.a.	n.a.
n.a.	1,2 PDO RI	n.a.	n.a.	n.a.	n.a.	n.a.	n.a.
n.a.	1,3-PDO	n.a.	n.a.	n.a.	n.a.	n.a.	n.a.
n.a.	Propionate RI	n.a.	n.a.	n.a.	n.a.	n.a.	n.a.
n.a.	1,3-PDO	n.a.	n.a.	n.a.	n.a.	n.a.	n.a.
4		11,978	0,275	BMB*^	n.a.	1,04	10489
n.a.	2-3 BDO	n.a.	n.a.	n.a.	n.a.	n.a.	n.a.

n.a.	Ethanol	n.a.	n.a.	n.a.	n.a.	n.a.	n.a.
n.a.	Isobutyrate RI	n.a.	n.a.	n.a.	n.a.	n.a.	n.a.
n.a.	Butyrate RI	n.a.	n.a.	n.a.	n.a.	n.a.	n.a.

Chromatogram and SST Results

Injection Details

Injection Name:	**Glucose, fructose,glycerol,2,3-butandieol (11.1 mM G**	*Run Time (min):*	**20,00**
Vial Number:	**3:85**	*Injection Volume:*	**1,00**
Injection Type:	**Calibration Standard**	*Channel:*	**RI_CH_1**
Calibration Level:	**2**	*Wavelength:*	**n.a.**
Instrument Method:	**Default method LC2030C 45 gr 20 min**	*Bandwidth:*	**n.a.**
Processing Method:	**Processing Method LC2030 45 gr**	*Dilution Factor:*	**1,0000**
Injection Date/Time:	**17/Jan/23 16:19**	*Sample Weight:*	**1,0000**

Chromatogram

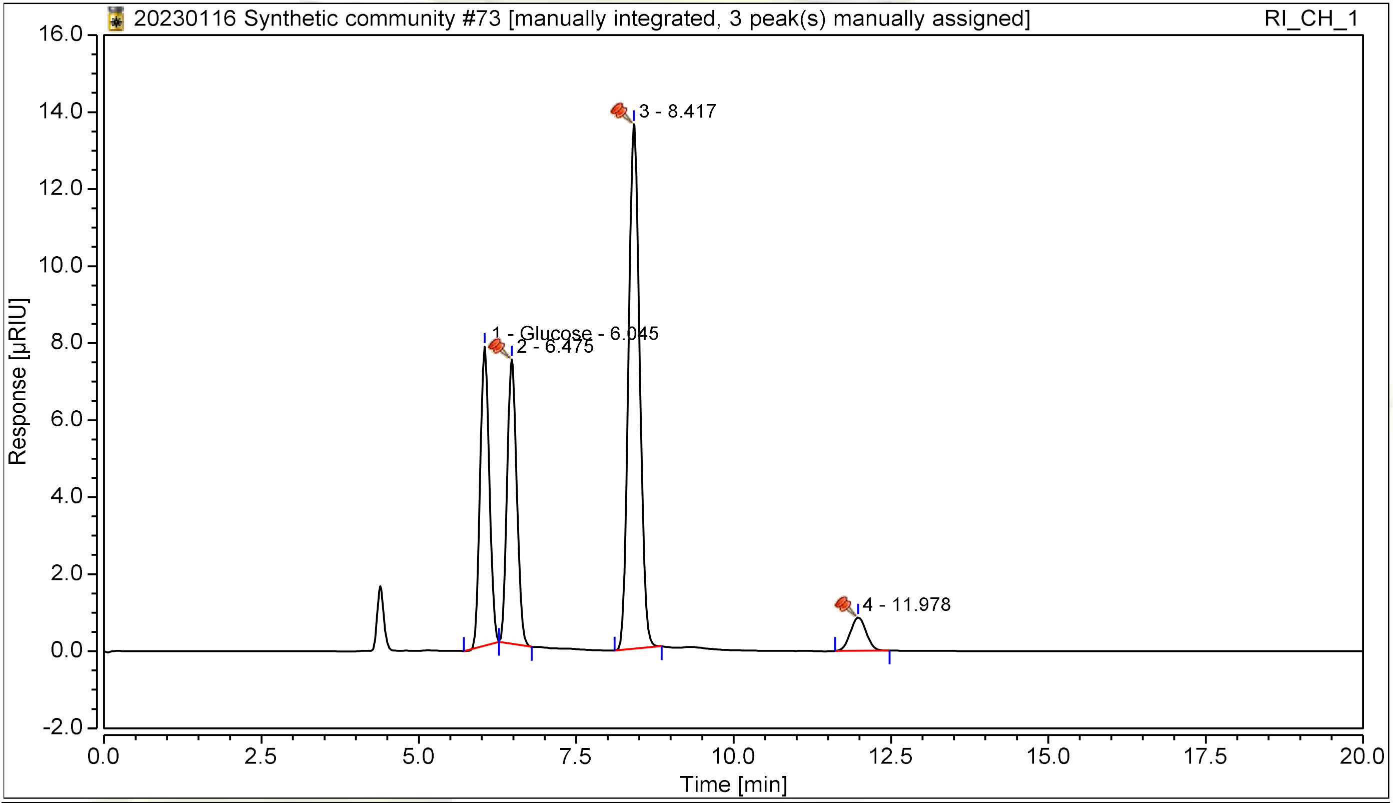

SST Results

No.	Name	Inj.Condition	Peak	Test Result	Injection
**Number of executed test cases:**	**n.a.**		**Total Result:**	**Passed**	

Chromatogram and Results

Injection Details

Injection Name:	**Glucose, fructose,glycerol,2,3-butandieol (22.2 ml**	*Run Time (min):*	**20,00**
Vial Number:	**3:85**	*Injection Volume:*	**2,00**
Injection Type:	**Calibration Standard**	*Channel:*	**RI_CH_1**
Calibration Level:	**2**	*Wavelength:*	**n.a.**
Instrument Method:	**Default method LC2030C 45 gr 20 min**	*Bandwidth:*	**n.a.**
Processing Method:	**Processing Method LC2030 45 gr**	*Dilution Factor:*	**1,0000**
Injection Date/Time:	**17/Jan/23 16:40**	*Sample Weight:*	**1,0000**

Chromatogram

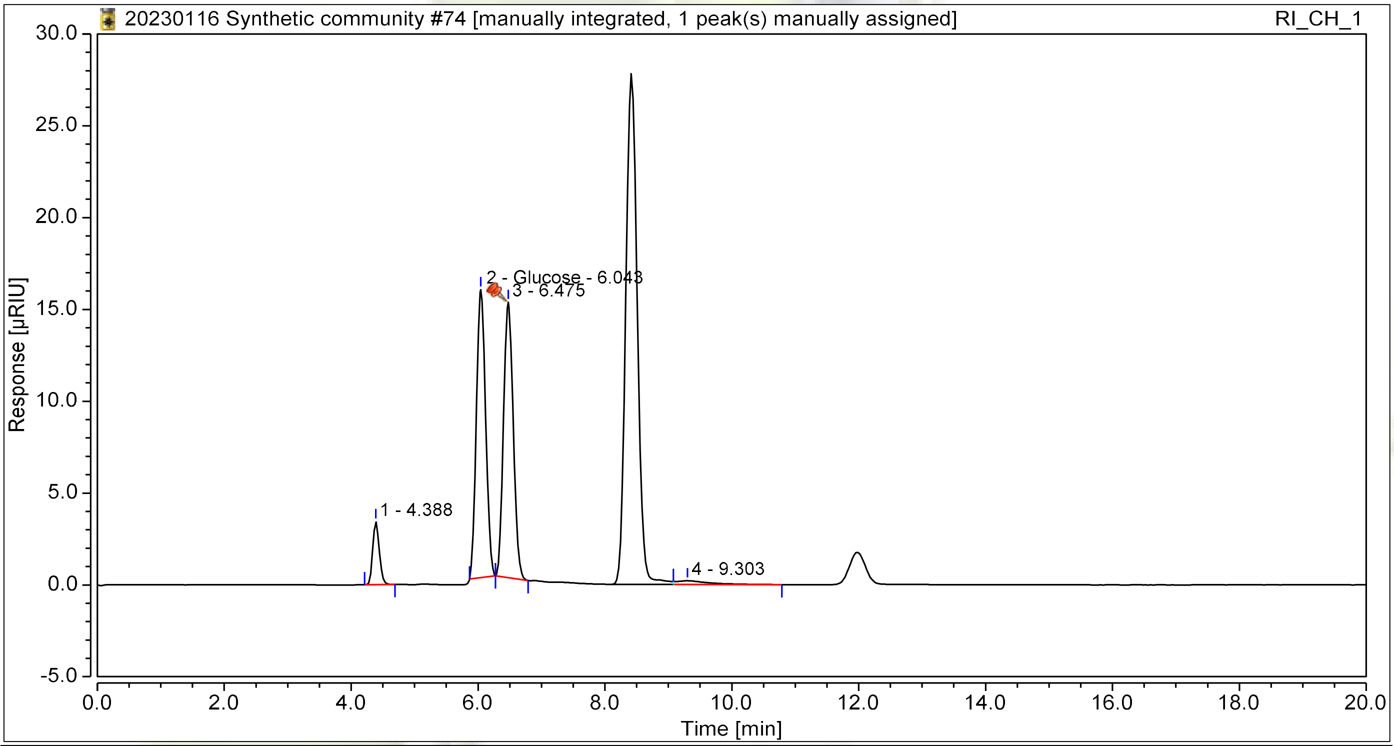

Integration Results

No.	Peak Name	Retention Time<br>min	Area<br>µRIU*min	Height<br>µRIU	Relative Area<br>%	Relative Height<br>%	Amount
1		4,388	0,412	3,425	7,06	9,98	n.a.
n.a.	GlcNAc	n.a.	n.a.	n.a.	n.a.	n.a.	n.a.
n.a.	Citrate	n.a.	n.a.	n.a.	n.a.	n.a.	n.a.
2	Glucose	6,043	2,650	15,679	45,35	45,71	21,4677
n.a.	Galactose	n.a.	n.a.	n.a.	n.a.	n.a.	n.a.
3		6,475	2,658	14,994	45,49	43,71	n.a.
n.a.	Fucose	n.a.	n.a.	n.a.	n.a.	n.a.	n.a.
n.a.	Succinate RI	n.a.	n.a.	n.a.	n.a.	n.a.	n.a.
n.a.	Lactate RI	n.a.	n.a.	n.a.	n.a.	n.a.	n.a.
n.a.	glycerol	n.a.	n.a.	n.a.	n.a.	n.a.	n.a.
n.a.	Formate RI	n.a.	n.a.	n.a.	n.a.	n.a.	n.a.
4		9,303	0,123	0,203	2,10	0,59	n.a.
n.a.	Acetate RI	n.a.	n.a.	n.a.	n.a.	n.a.	n.a.
n.a.	1,2 PDO RI	n.a.	n.a.	n.a.	n.a.	n.a.	n.a.
n.a.	1,3-PDO	n.a.	n.a.	n.a.	n.a.	n.a.	n.a.
n.a.	Propionate RI	n.a.	n.a.	n.a.	n.a.	n.a.	n.a.
n.a.	1,3-PDO	n.a.	n.a.	n.a.	n.a.	n.a.	n.a.
n.a.	2-3 BDO	n.a.	n.a.	n.a.	n.a.	n.a.	n.a.
n.a.	Ethanol	n.a.	n.a.	n.a.	n.a.	n.a.	n.a.
n.a.	Isobutyrate RI	n.a.	n.a.	n.a.	n.a.	n.a.	n.a.
n.a.	Butyrate RI	n.a.	n.a.	n.a.	n.a.	n.a.	n.a.

**Total:**	**5,843**	**34,301**	**100,00**	**100,00**

Peak Analysis

Injection Details

Injection Name:	**Glucose, fructose,glycerol,2,3-butandieol (**	*Run Time (min):*	**20,00**
Vial Number:	**3:85**	*Injection Volume:*	**2,00**
Injection Type:	**Calibration Standard**	*Channel:*	**RI_CH_1**
Calibration Level:	**2**	*Wavelength:*	**n.a.**
Instrument Method:	**Default method LC2030C 45 gr 20 min**	*Bandwidth:*	**n.a.**
Processing Method:	**Processing Method LC2030 45 gr**	*Dilution Factor:*	**1,0000**
Injection Date/Time:	**17/Jan/23 16:40**	*Sample Weight:*	**1,0000**

Chromatogram

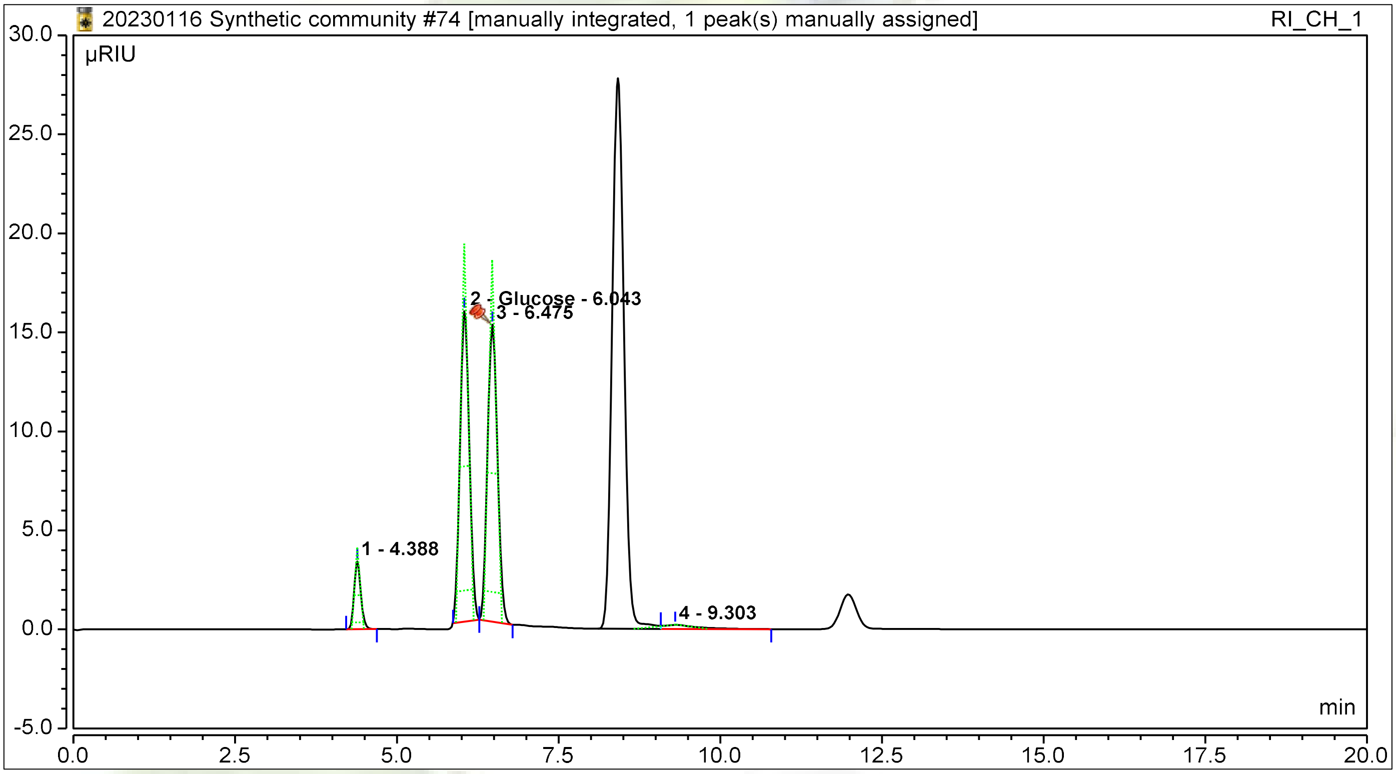

Peak Results

No.	Peak Name	Retention Time min	Width (50%) min	Type	Resolution (EP)	Asymmetry (EP)	Plates (EP)
1		4,388	0,111	BMB*	7,17	1,16	8592
n.a.	GlcNAc	n.a.	n.a.	n.a.	n.a.	n.a.	n.a.
n.a.	Citrate	n.a.	n.a.	n.a.	n.a.	n.a.	n.a.
2	Glucose	6,043	0,161	BMb*	1,55	1,09	7807
n.a.	Galactose	n.a.	n.a.	n.a.	n.a.	n.a.	n.a.
3		6,475	0,168	bMB*^	n.a.	1,11	8272
n.a.	Fucose	n.a.	n.a.	n.a.	n.a.	n.a.	n.a.
n.a.	Succinate RI	n.a.	n.a.	n.a.	n.a.	n.a.	n.a.
n.a.	Lactate RI	n.a.	n.a.	n.a.	n.a.	n.a.	n.a.
n.a.	glycerol	n.a.	n.a.	n.a.	n.a.	n.a.	n.a.
n.a.	Formate RI	n.a.	n.a.	n.a.	n.a.	n.a.	n.a.
4		9,303	n.a.	MB	n.a.	n.a.	n.a.
n.a.	Acetate RI	n.a.	n.a.	n.a.	n.a.	n.a.	n.a.
n.a.	1,2 PDO RI	n.a.	n.a.	n.a.	n.a.	n.a.	n.a.
n.a.	1,3-PDO	n.a.	n.a.	n.a.	n.a.	n.a.	n.a.
n.a.	Propionate RI	n.a.	n.a.	n.a.	n.a.	n.a.	n.a.
n.a.	1,3-PDO	n.a.	n.a.	n.a.	n.a.	n.a.	n.a.
n.a.	2-3 BDO	n.a.	n.a.	n.a.	n.a.	n.a.	n.a.

n.a.	Ethanol	n.a.	n.a.	n.a.	n.a.	n.a.	n.a.
n.a.	Isobutyrate RI	n.a.	n.a.	n.a.	n.a.	n.a.	n.a.
n.a.	Butyrate RI	n.a.	n.a.	n.a.	n.a.	n.a.	n.a.

Chromatogram and SST Results

Injection Details

Injection Name:	**Glucose, fructose,glycerol,2,3-butandieol (22.2 mM)**	*Run Time (min):*	**20,00**
Vial Number:	**3:85**	*Injection Volume:*	**2,00**
Injection Type:	**Calibration Standard**	*Channel:*	**RI_CH_1**
Calibration Level:	**2**	*Wavelength:*	**n.a.**
Instrument Method:	**Default method LC2030C 45 gr 20 min**	*Bandwidth:*	**n.a.**
Processing Method:	**Processing Method LC2030 45 gr**	*Dilution Factor:*	**1,0000**
Injection Date/Time:	**17/Jan/23 16:40**	*Sample Weight:*	**1,0000**

Chromatogram

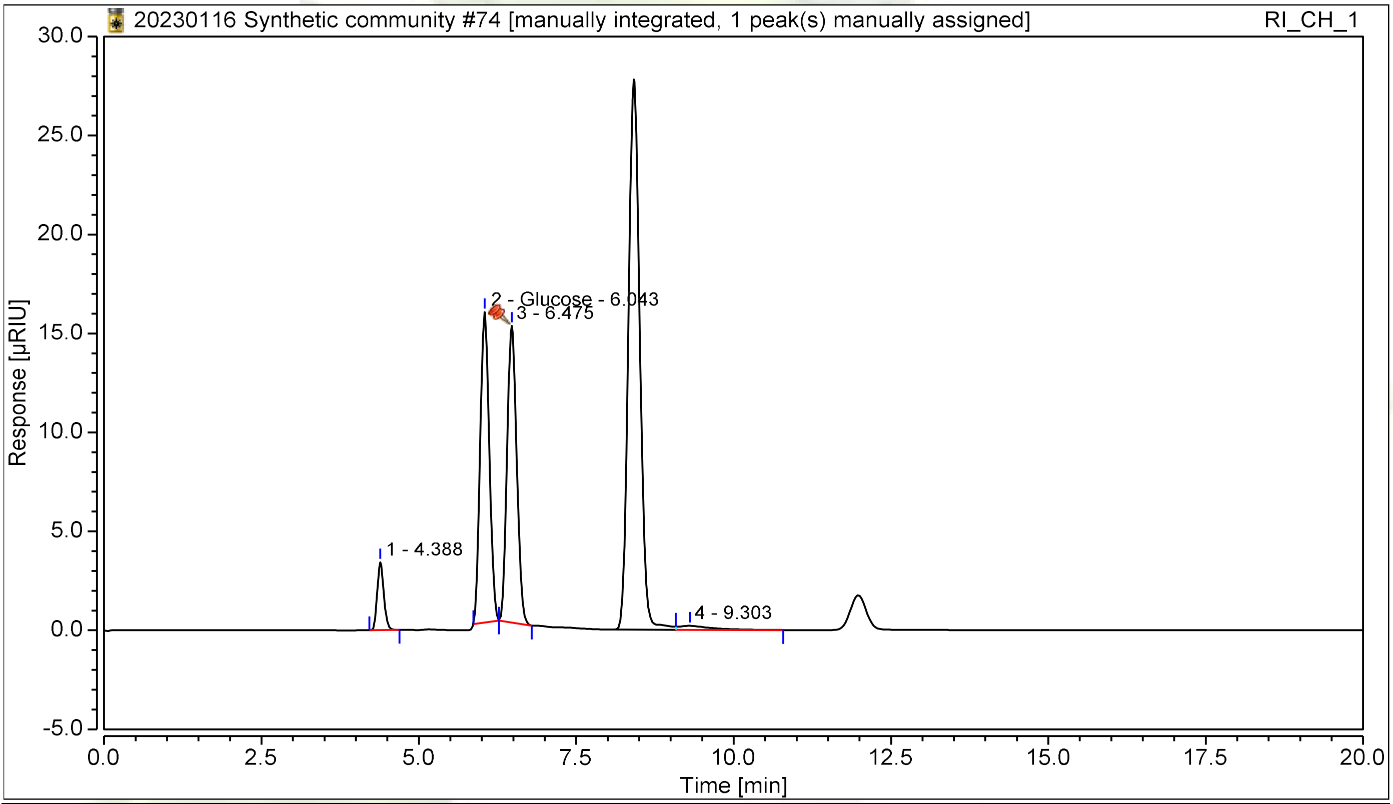

SST Results

No.	Name	Inj.Condition	Peak	Test Result	Injection

Number of executed test cases: **n.a.**   **Total Result:** **Passed**

Chromeleon (c) Dionex
Version 7.2.10.25868

Chromatogram and Results

Injection Details

Injection Name:	**Glucose, fructose,glycerol,2,3-butandieol (33.3 mM**	*Run Time (min):*	**20,00**
Vial Number:	**3:85**	*Injection Volume:*	**3,00**
Injection Type:	**Calibration Standard**	*Channel:*	**RI_CH_1**
Calibration Level:	**2**	*Wavelength:*	**n.a.**
Instrument Method:	**Default method LC2030C 45 gr 20 min**	*Bandwidth:*	**n.a.**
Processing Method:	**Processing Method LC2030 45 gr**	*Dilution Factor:*	**1,0000**
Injection Date/Time:	**17/Jan/23 17:00**	*Sample Weight:*	**1,0000**

Chromatogram

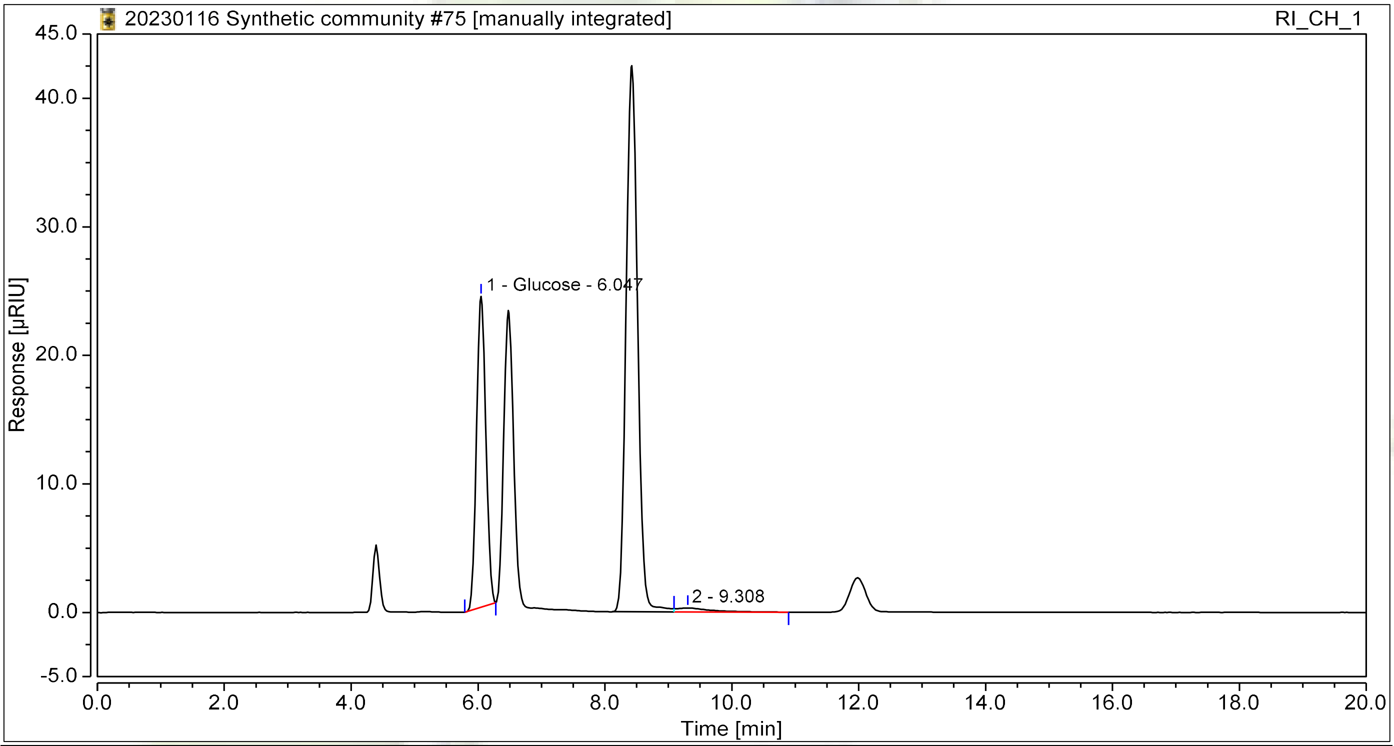

Integration Results

No.	Peak Name	Retention Time min	Area µRIU*min	Height µRIU	Relative Area %	Relative Height %	Amount
n.a.	GlcNAc	n.a.	n.a.	n.a.	n.a.	n.a.	n.a.
n.a.	Citrate	n.a.	n.a.	n.a.	n.a.	n.a.	n.a.
1	Glucose	6,047	4,129	24,161	95,61	98,73	33,4572
n.a.	Galactose	n.a.	n.a.	n.a.	n.a.	n.a.	n.a.
n.a.	Fucose	n.a.	n.a.	n.a.	n.a.	n.a.	n.a.
n.a.	Succinate RI	n.a.	n.a.	n.a.	n.a.	n.a.	n.a.
n.a.	Lactate RI	n.a.	n.a.	n.a.	n.a.	n.a.	n.a.
n.a.	glycerol	n.a.	n.a.	n.a.	n.a.	n.a.	n.a.
n.a.	Formate RI	n.a.	n.a.	n.a.	n.a.	n.a.	n.a.
2		9,308	0,190	0,312	4,39	1,27	n.a.
n.a.	Acetate RI	n.a.	n.a.	n.a.	n.a.	n.a.	n.a.
n.a.	1,2 PDO RI	n.a.	n.a.	n.a.	n.a.	n.a.	n.a.
n.a.	1,3-PDO	n.a.	n.a.	n.a.	n.a.	n.a.	n.a.
n.a.	Propionate RI	n.a.	n.a.	n.a.	n.a.	n.a.	n.a.
n.a.	1,3-PDO	n.a.	n.a.	n.a.	n.a.	n.a.	n.a.
n.a.	2-3 BDO	n.a.	n.a.	n.a.	n.a.	n.a.	n.a.
n.a.	Ethanol	n.a.	n.a.	n.a.	n.a.	n.a.	n.a.
n.a.	Isobutyrate RI	n.a.	n.a.	n.a.	n.a.	n.a.	n.a.
n.a.	Butyrate RI	n.a.	n.a.	n.a.	n.a.	n.a.	n.a.
**Total:**			**4,319**	**24,473**	**100,00**	**100,00**	

Peak Analysis

Injection Details

Injection Name:	**Glucose, fructose,glycerol,2,3-butandieol (**	*Run Time (min):*	**20,00**
Vial Number:	**3:85**	*Injection Volume:*	**3,00**
Injection Type:	**Calibration Standard**	*Channel:*	**RI_CH_1**
Calibration Level:	**2**	*Wavelength:*	**n.a.**
Instrument Method:	**Default method LC2030C 45 gr 20 min**	*Bandwidth:*	**n.a.**
Processing Method:	**Processing Method LC2030 45 gr**	*Dilution Factor:*	**1,0000**
Injection Date/Time:	**17/Jan/23 17:00**	*Sample Weight:*	**1,0000**

Chromatogram

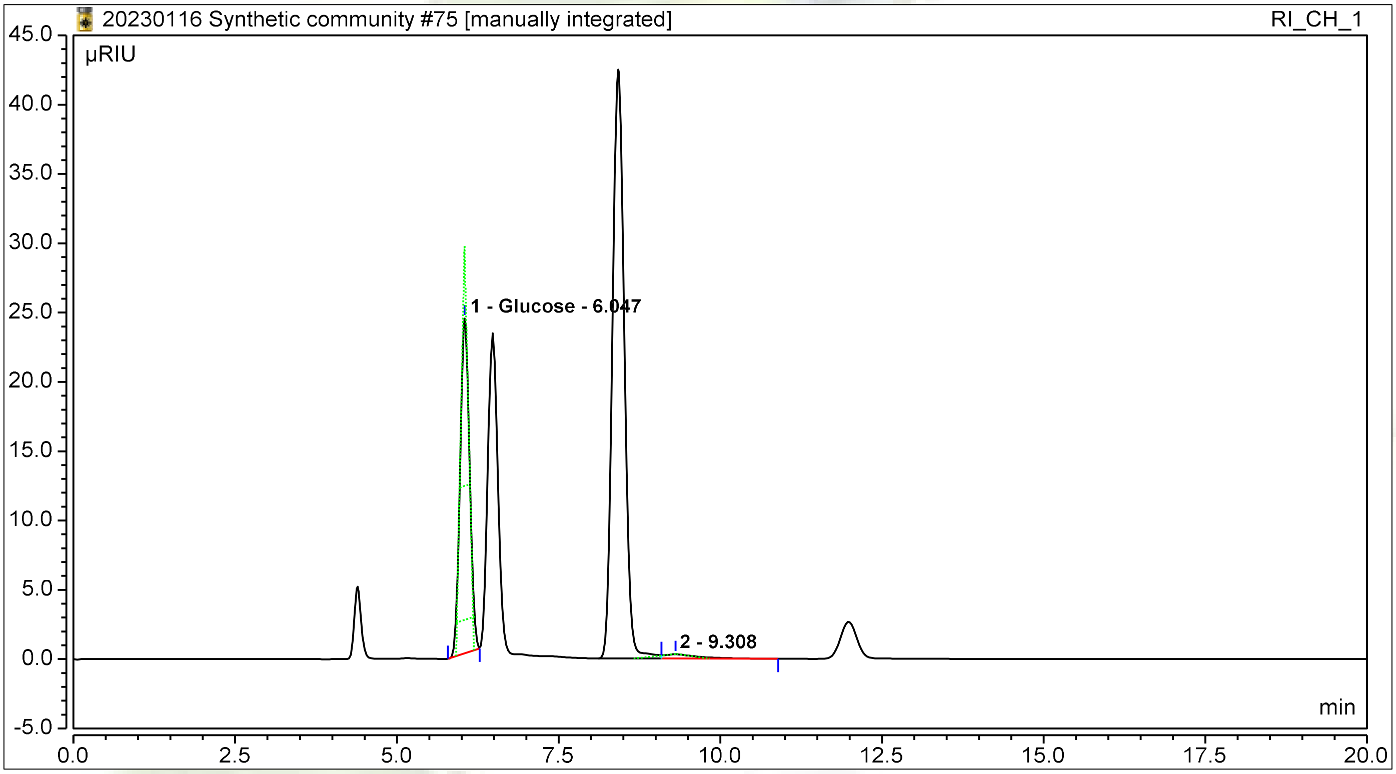

Peak Results

No.	Peak Name	Retention Time min	Width (50%) min	Type	Resolution (EP)	Asymmetry (EP)	Plates (EP)
n.a.	GlcNAc	n.a.	n.a.	n.a.	n.a.	n.a.	n.a.
n.a.	Citrate	n.a.	n.a.	n.a.	n.a.	n.a.	n.a.
1	Glucose	6,047	0,162	BMB*	n.a.	1,08	7713
n.a.	Galactose	n.a.	n.a.	n.a.	n.a.	n.a.	n.a.
n.a.	Fucose	n.a.	n.a.	n.a.	n.a.	n.a.	n.a.
n.a.	Succinate RI	n.a.	n.a.	n.a.	n.a.	n.a.	n.a.
n.a.	Lactate RI	n.a.	n.a.	n.a.	n.a.	n.a.	n.a.
n.a.	glycerol	n.a.	n.a.	n.a.	n.a.	n.a.	n.a.
n.a.	Formate RI	n.a.	n.a.	n.a.	n.a.	n.a.	n.a.
2		9,308	n.a.	MB	n.a.	n.a.	n.a.
n.a.	Acetate RI	n.a.	n.a.	n.a.	n.a.	n.a.	n.a.
n.a.	1,2 PDO RI	n.a.	n.a.	n.a.	n.a.	n.a.	n.a.
n.a.	1,3-PDO	n.a.	n.a.	n.a.	n.a.	n.a.	n.a.
n.a.	Propionate RI	n.a.	n.a.	n.a.	n.a.	n.a.	n.a.
n.a.	1,3-PDO	n.a.	n.a.	n.a.	n.a.	n.a.	n.a.
n.a.	2-3 BDO	n.a.	n.a.	n.a.	n.a.	n.a.	n.a.
n.a.	Ethanol	n.a.	n.a.	n.a.	n.a.	n.a.	n.a.
n.a.	Isobutyrate RI	n.a.	n.a.	n.a.	n.a.	n.a.	n.a.

Chromeleon (c) Dionex
Version 7.2.10.25868

n.a.	Butyrate RI	n.a.	n.a.	n.a.	n.a.	n.a.	n.a.

Chromatogram and SST Results

Injection Details

Injection Name:	**Glucose, fructose,glycerol,2,3-butandieol (33.3 mM)**	*Run Time (min):*	**20,00**
Vial Number:	**3:85**	*Injection Volume:*	**3,00**
Injection Type:	**Calibration Standard**	*Channel:*	**RI_CH_1**
Calibration Level:	**2**	*Wavelength:*	**n.a.**
Instrument Method:	**Default method LC2030C 45 gr 20 min**	*Bandwidth:*	**n.a.**
Processing Method:	**Processing Method LC2030 45 gr**	*Dilution Factor:*	**1,0000**
Injection Date/Time:	**17/Jan/23 17:00**	*Sample Weight:*	**1,0000**

Chromatogram

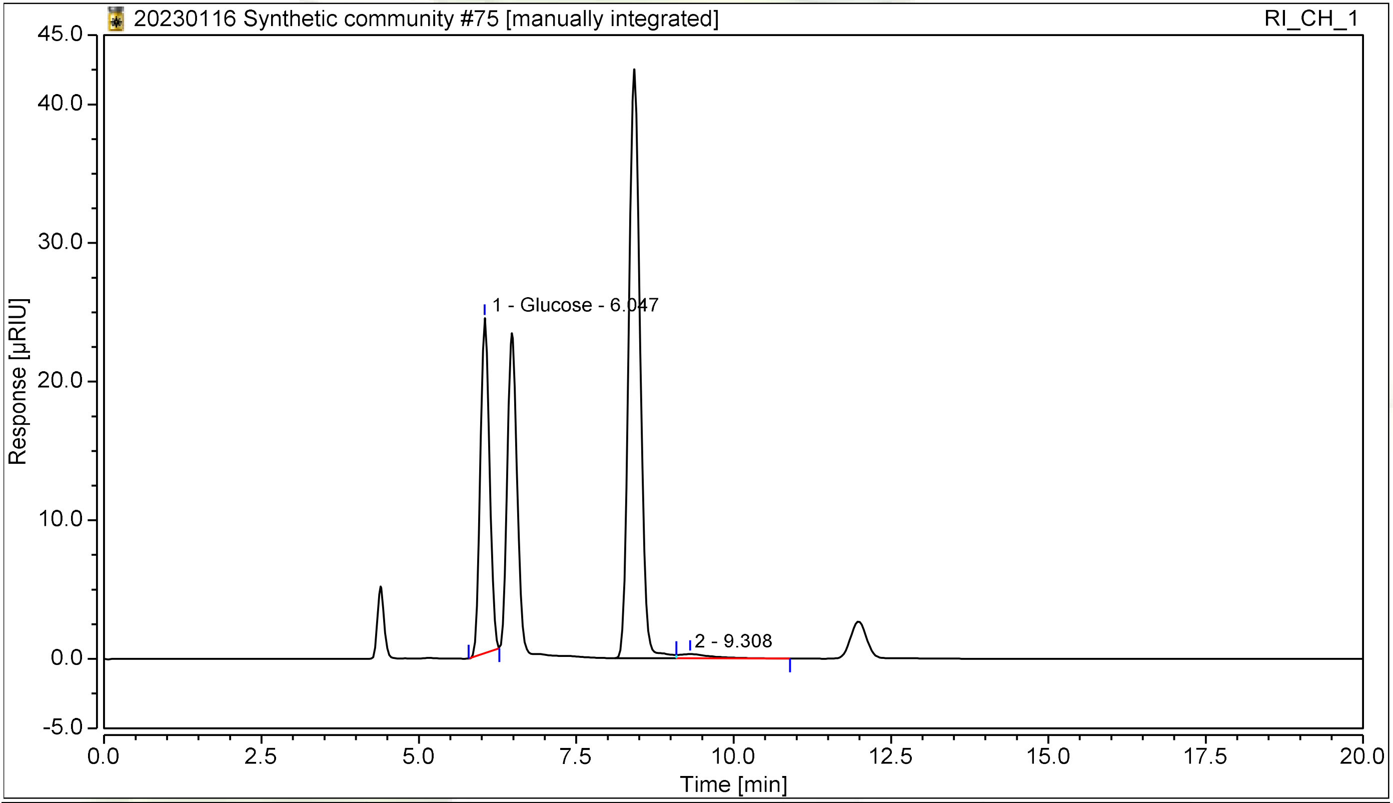

SST Results

No.	Name	Inj.Condition	Peak	Test Result	Injection

Number of executed test cases: n.a.   **Total Result: Passed**

Chromatogram and Results

Injection Details

Injection Name:	**citrate,succinate, maltate,fumarate 10**	*Run Time (min):*	**20,00**
Vial Number:	**3:86**	*Injection Volume:*	**1,00**
Injection Type:	**Calibration Standard**	*Channel:*	**RI_CH_1**
Calibration Level:	**1**	*Wavelength:*	**n.a.**
Instrument Method:	**Default method LC2030C 45 gr 20 min**	*Bandwidth:*	**n.a.**
Processing Method:	**Processing Method LC2030 45 gr**	*Dilution Factor:*	**1,0000**
Injection Date/Time:	**17/Jan/23 17:21**	*Sample Weight:*	**1,0000**

Chromatogram

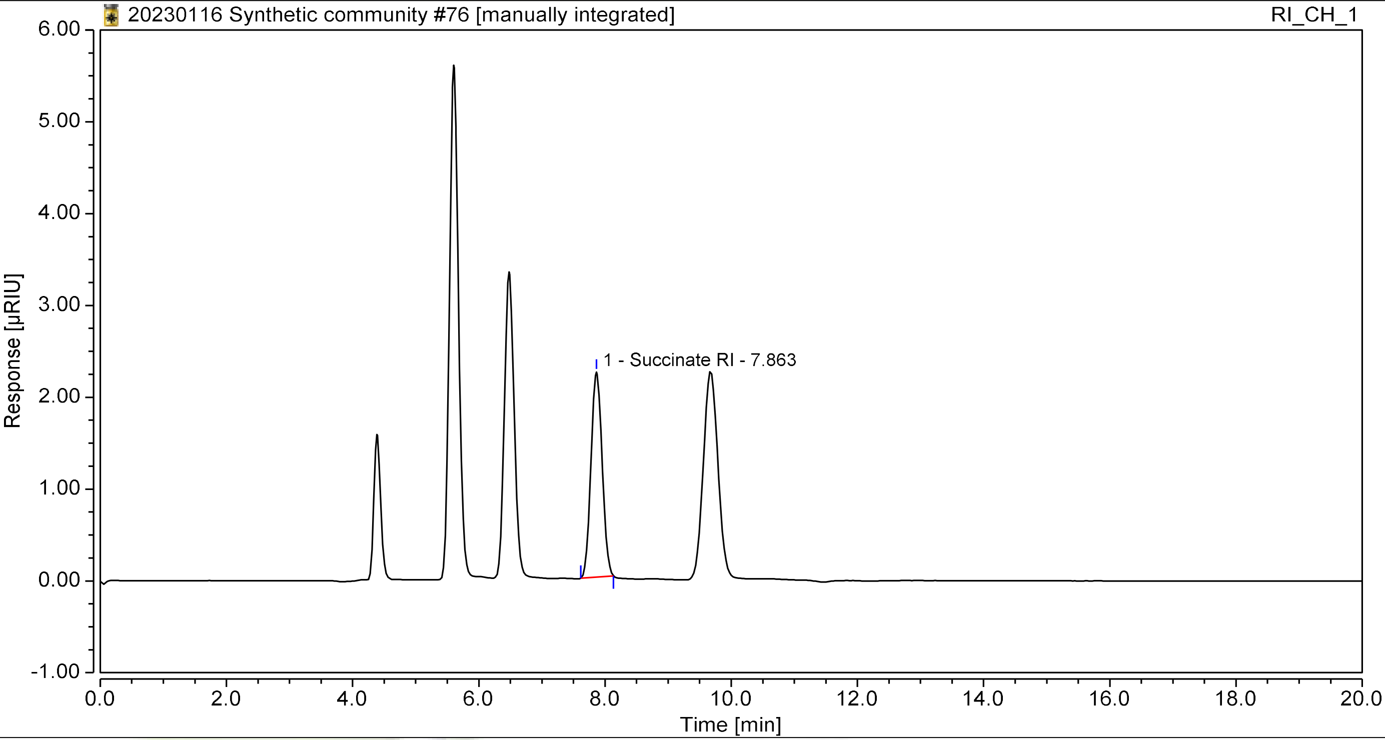

Integration Results

No.	Peak Name	Retention Time min	Area µRIU*min	Height µRIU	Relative Area %	Relative Height %	Amount
n.a.	GlcNAc	n.a.	n.a.	n.a.	n.a.	n.a.	n.a.
n.a.	Citrate	n.a.	n.a.	n.a.	n.a.	n.a.	n.a.
n.a.	Glucose	n.a.	n.a.	n.a.	n.a.	n.a.	n.a.
n.a.	Galactose	n.a.	n.a.	n.a.	n.a.	n.a.	n.a.
n.a.	Fucose	n.a.	n.a.	n.a.	n.a.	n.a.	n.a.
1	Succinate RI	7,863	0,459	2,234	100,00	100,00	9,4724
n.a.	Lactate RI	n.a.	n.a.	n.a.	n.a.	n.a.	n.a.
n.a.	glycerol	n.a.	n.a.	n.a.	n.a.	n.a.	n.a.
n.a.	Formate RI	n.a.	n.a.	n.a.	n.a.	n.a.	n.a.
n.a.	Acetate RI	n.a.	n.a.	n.a.	n.a.	n.a.	n.a.
n.a.	1,2 PDO RI	n.a.	n.a.	n.a.	n.a.	n.a.	n.a.
n.a.	1,3-PDO	n.a.	n.a.	n.a.	n.a.	n.a.	n.a.
n.a.	Propionate RI	n.a.	n.a.	n.a.	n.a.	n.a.	n.a.
n.a.	1,3-PDO	n.a.	n.a.	n.a.	n.a.	n.a.	n.a.
n.a.	2-3 BDO	n.a.	n.a.	n.a.	n.a.	n.a.	n.a.
n.a.	Ethanol	n.a.	n.a.	n.a.	n.a.	n.a.	n.a.
n.a.	Isobutyrate RI	n.a.	n.a.	n.a.	n.a.	n.a.	n.a.
n.a.	Butyrate RI	n.a.	n.a.	n.a.	n.a.	n.a.	n.a.
**Total:**			**0,459**	**2,234**	**100,00**	**100,00**	

Chromeleon (c) Dionex
Version 7.2.10.25868

Peak Analysis

Injection Details

Injection Name:	**citrate,succinate, maltate,fumarate 10**	*Run Time (min):*	**20,00**
Vial Number:	**3:86**	*Injection Volume:*	**1,00**
Injection Type:	**Calibration Standard**	*Channel:*	**RI_CH_1**
Calibration Level:	**1**	*Wavelength:*	**n.a.**
Instrument Method:	**Default method LC2030C 45 gr 20 min**	*Bandwidth:*	**n.a.**
Processing Method:	**Processing Method LC2030 45 gr**	*Dilution Factor:*	**1,0000**
Injection Date/Time:	**17/Jan/23 17:21**	*Sample Weight:*	**1,0000**

Chromatogram

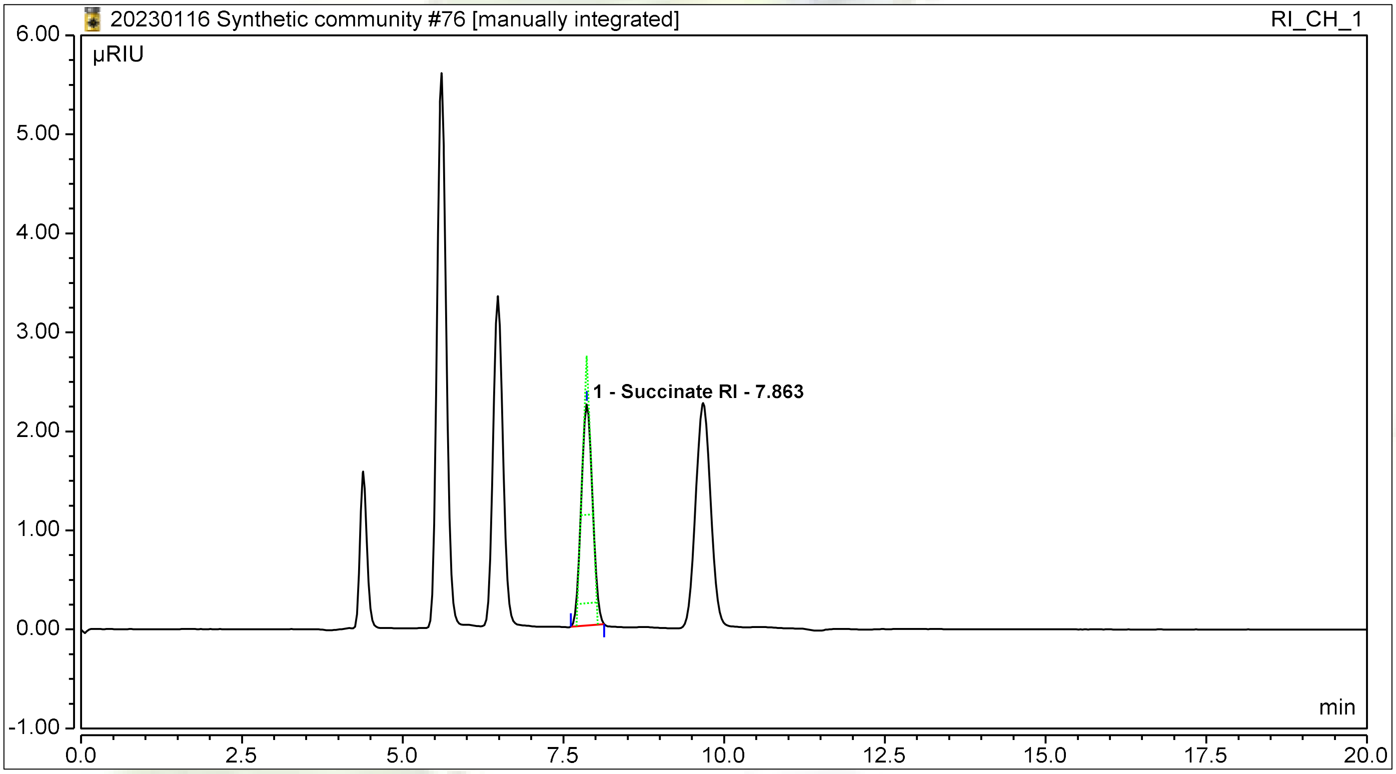

Peak Results

No.	Peak Name	Retention Time min	Width (50%) min	Type	Resolution (EP)	Asymmetry (EP)	Plates (EP)
n.a.	GlcNAc	n.a.	n.a.	n.a.	n.a.	n.a.	n.a.
n.a.	Citrate	n.a.	n.a.	n.a.	n.a.	n.a.	n.a.
n.a.	Glucose	n.a.	n.a.	n.a.	n.a.	n.a.	n.a.
n.a.	Galactose	n.a.	n.a.	n.a.	n.a.	n.a.	n.a.
n.a.	Fucose	n.a.	n.a.	n.a.	n.a.	n.a.	n.a.
1	Succinate RI	7,863	0,195	BMB*	n.a.	1,06	9017
n.a.	Lactate RI	n.a.	n.a.	n.a.	n.a.	n.a.	n.a.
n.a.	glycerol	n.a.	n.a.	n.a.	n.a.	n.a.	n.a.
n.a.	Formate RI	n.a.	n.a.	n.a.	n.a.	n.a.	n.a.
n.a.	Acetate RI	n.a.	n.a.	n.a.	n.a.	n.a.	n.a.
n.a.	1,2 PDO RI	n.a.	n.a.	n.a.	n.a.	n.a.	n.a.
n.a.	1,3-PDO	n.a.	n.a.	n.a.	n.a.	n.a.	n.a.
n.a.	Propionate RI	n.a.	n.a.	n.a.	n.a.	n.a.	n.a.
n.a.	1,3-PDO	n.a.	n.a.	n.a.	n.a.	n.a.	n.a.
n.a.	2-3 BDO	n.a.	n.a.	n.a.	n.a.	n.a.	n.a.
n.a.	Ethanol	n.a.	n.a.	n.a.	n.a.	n.a.	n.a.
n.a.	Isobutyrate RI	n.a.	n.a.	n.a.	n.a.	n.a.	n.a.
n.a.	Butyrate RI	n.a.	n.a.	n.a.	n.a.	n.a.	n.a.

Chromeleon (c) Dionex
Version 7.2.10.25868

Chromatogram and SST Results

Injection Details

Injection Name:	**citrate,succinate, maltate,fumarate 10**	*Run Time (min):*	**20,00**
Vial Number:	**3:86**	*Injection Volume:*	**1,00**
Injection Type:	**Calibration Standard**	*Channel:*	**RI_CH_1**
Calibration Level:	**1**	*Wavelength:*	**n.a.**
Instrument Method:	**Default method LC2030C 45 gr 20 min**	*Bandwidth:*	**n.a.**
Processing Method:	**Processing Method LC2030 45 gr**	*Dilution Factor:*	**1,0000**
Injection Date/Time:	**17/Jan/23 17:21**	*Sample Weight:*	**1,0000**

Chromatogram

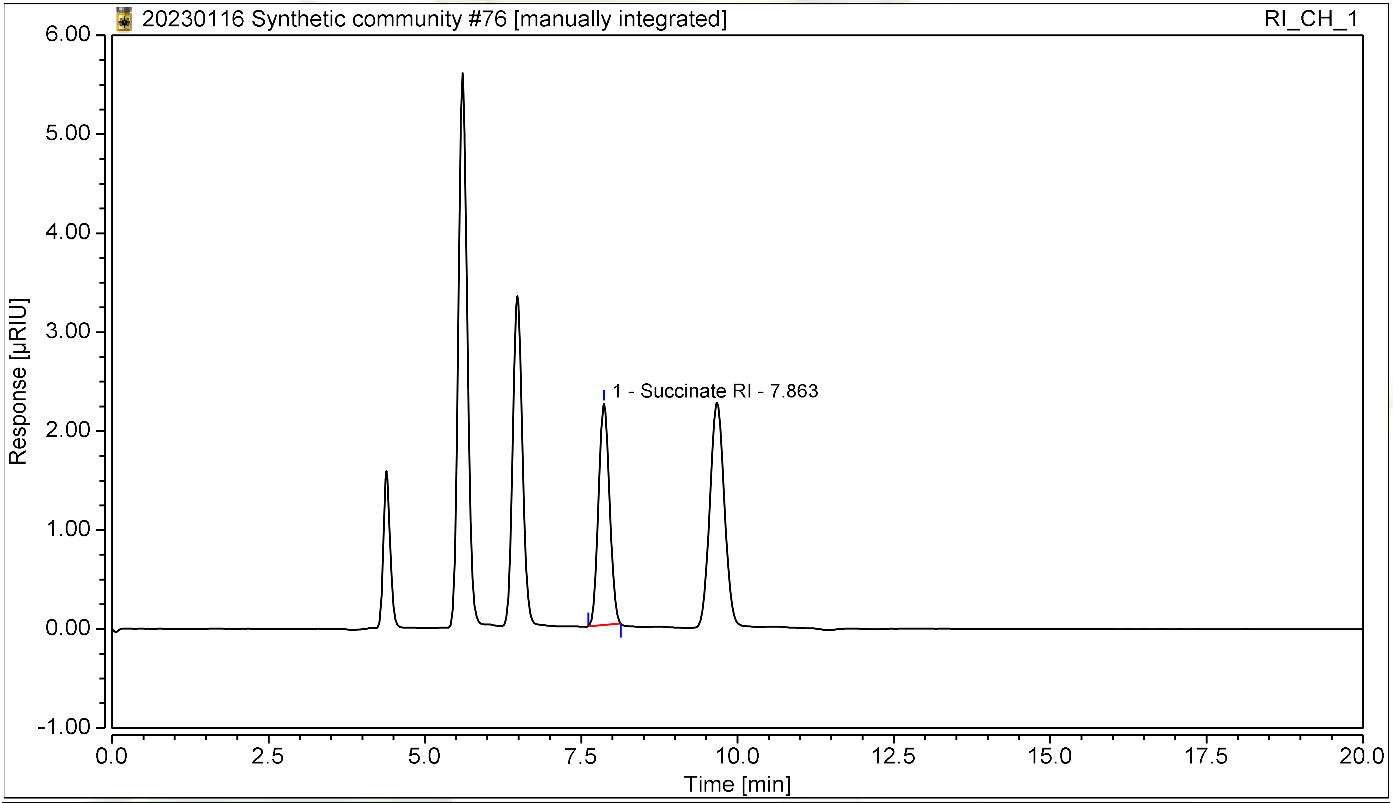

SST Results

No.	Name	Inj.Condition	Peak	Test Result	Injection
**Number of executed test cases:**	**n.a.**		**Total Result:**	**Passed**	

Chromatogram and Results

Injection Details

Injection Name:	**citrate,succinate, maltate,fumarate 20**	*Run Time (min):*	**20,00**
Vial Number:	**3:86**	*Injection Volume:*	**2,00**
Injection Type:	**Calibration Standard**	*Channel:*	**RI_CH_1**
Calibration Level:	**1**	*Wavelength:*	**n.a.**
Instrument Method:	**Default method LC2030C 45 gr 20 min**	*Bandwidth:*	**n.a.**
Processing Method:	**Processing Method LC2030 45 gr**	*Dilution Factor:*	**1,0000**
Injection Date/Time:	**17/Jan/23 17:41**	*Sample Weight:*	**1,0000**

Chromatogram

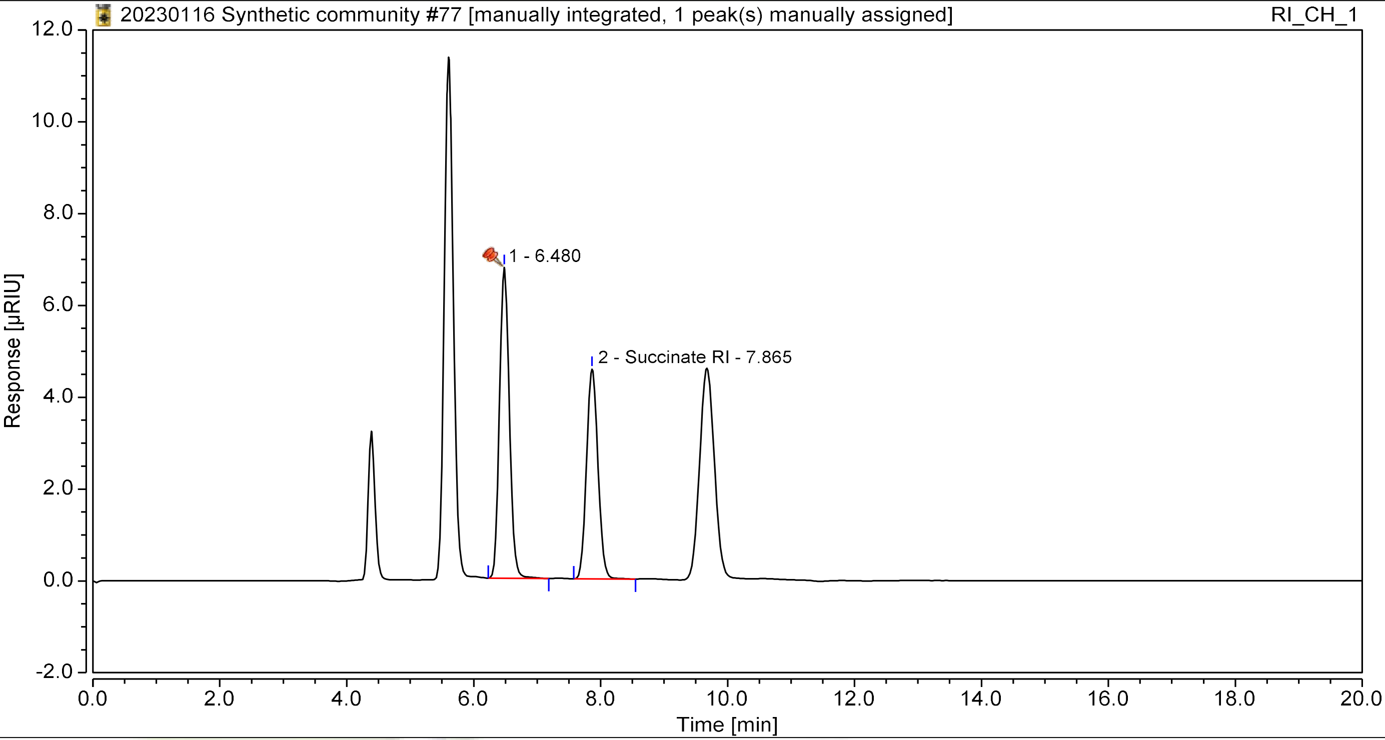

Integration Results

No.	Peak Name	Retention Time min	Area µRIU*min	Height µRIU	Relative Area %	Relative Height %	Amount
n.a.	GlcNAc	n.a.	n.a.	n.a.	n.a.	n.a.	n.a.
n.a.	Citrate	n.a.	n.a.	n.a.	n.a.	n.a.	n.a.
n.a.	Glucose	n.a.	n.a.	n.a.	n.a.	n.a.	n.a.
n.a.	Galactose	n.a.	n.a.	n.a.	n.a.	n.a.	n.a.
1		6,480	1,218	6,765	55,93	59,67	n.a.
n.a.	Fucose	n.a.	n.a.	n.a.	n.a.	n.a.	n.a.
2	Succinate RI	7,865	0,959	4,572	44,07	40,33	19,8047
n.a.	Lactate RI	n.a.	n.a.	n.a.	n.a.	n.a.	n.a.
n.a.	glycerol	n.a.	n.a.	n.a.	n.a.	n.a.	n.a.
n.a.	Formate RI	n.a.	n.a.	n.a.	n.a.	n.a.	n.a.
n.a.	Acetate RI	n.a.	n.a.	n.a.	n.a.	n.a.	n.a.
n.a.	1,2 PDO RI	n.a.	n.a.	n.a.	n.a.	n.a.	n.a.
n.a.	1,3-PDO	n.a.	n.a.	n.a.	n.a.	n.a.	n.a.
n.a.	Propionate RI	n.a.	n.a.	n.a.	n.a.	n.a.	n.a.
n.a.	1,3-PDO	n.a.	n.a.	n.a.	n.a.	n.a.	n.a.
n.a.	2-3 BDO	n.a.	n.a.	n.a.	n.a.	n.a.	n.a.
n.a.	Ethanol	n.a.	n.a.	n.a.	n.a.	n.a.	n.a.
n.a.	Isobutyrate RI	n.a.	n.a.	n.a.	n.a.	n.a.	n.a.
n.a.	Butyrate RI	n.a.	n.a.	n.a.	n.a.	n.a.	n.a.
**Total:**			**2,177**	**11,337**	**100,00**	**100,00**	

Chromeleon (c) Dionex
Version 7.2.10.25868

Peak Analysis

Injection Details

Injection Name:	**citrate,succinate, maltate,fumarate 20**	*Run Time (min):*	**20,00**
Vial Number:	**3:86**	*Injection Volume:*	**2,00**
Injection Type:	**Calibration Standard**	*Channel:*	**RI_CH_1**
Calibration Level:	**1**	*Wavelength:*	**n.a.**
Instrument Method:	**Default method LC2030C 45 gr 20 min**	*Bandwidth:*	**n.a.**
Processing Method:	**Processing Method LC2030 45 gr**	*Dilution Factor:*	**1,0000**
Injection Date/Time:	**17/Jan/23 17:41**	*Sample Weight:*	**1,0000**

Chromatogram

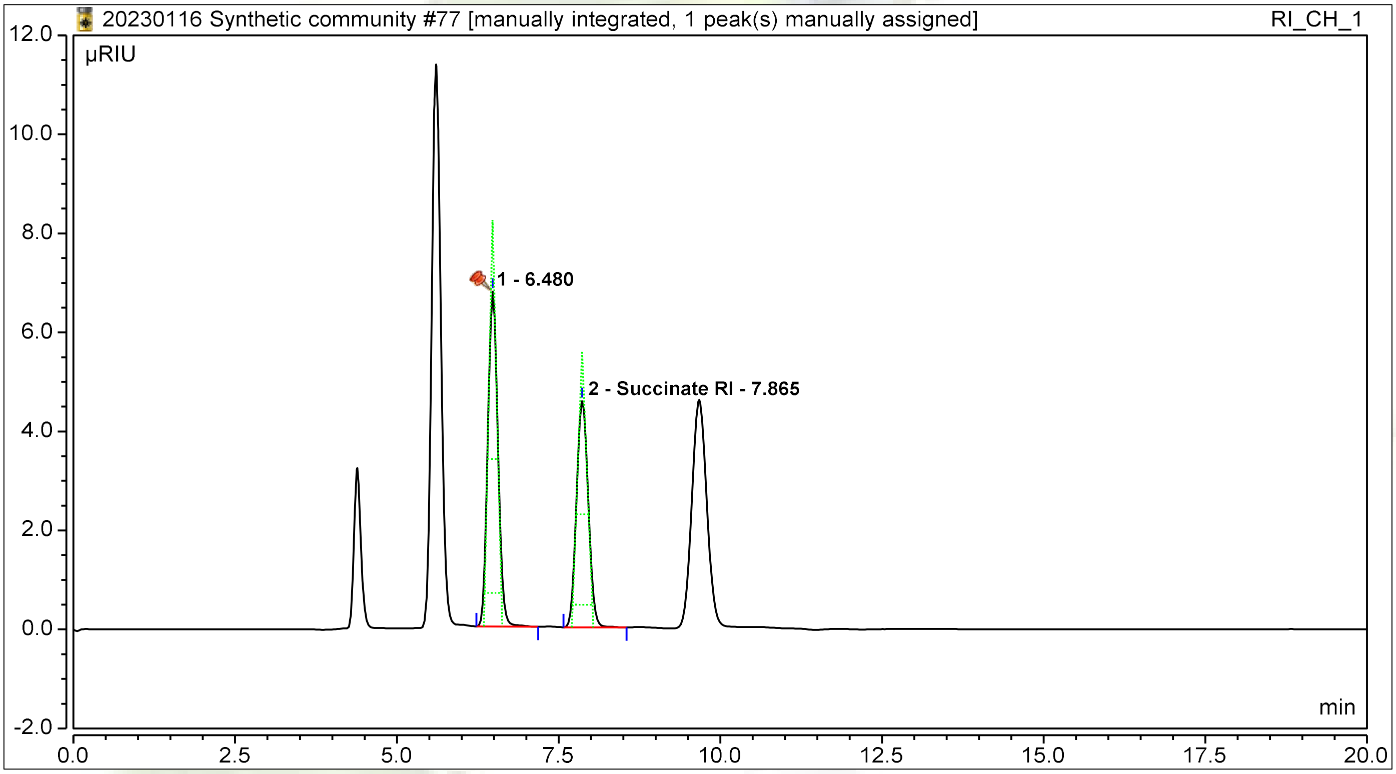

Peak Results

No.	Peak Name	Retention Time min	Width (50%) min	Type	Resolution (EP)	Asymmetry (EP)	Plates (EP)
n.a.	GlcNAc	n.a.	n.a.	n.a.	n.a.	n.a.	n.a.
n.a.	Citrate	n.a.	n.a.	n.a.	n.a.	n.a.	n.a.
n.a.	Glucose	n.a.	n.a.	n.a.	n.a.	n.a.	n.a.
n.a.	Galactose	n.a.	n.a.	n.a.	n.a.	n.a.	n.a.
1		6,480	0,167	BMB^	4,50	1,11	8334
n.a.	Fucose	n.a.	n.a.	n.a.	n.a.	n.a.	n.a.
2	Succinate RI	7,865	0,196	BMB	n.a.	1,08	8878
n.a.	Lactate RI	n.a.	n.a.	n.a.	n.a.	n.a.	n.a.
n.a.	glycerol	n.a.	n.a.	n.a.	n.a.	n.a.	n.a.
n.a.	Formate RI	n.a.	n.a.	n.a.	n.a.	n.a.	n.a.
n.a.	Acetate RI	n.a.	n.a.	n.a.	n.a.	n.a.	n.a.
n.a.	1,2 PDO RI	n.a.	n.a.	n.a.	n.a.	n.a.	n.a.
n.a.	1,3-PDO	n.a.	n.a.	n.a.	n.a.	n.a.	n.a.
n.a.	Propionate RI	n.a.	n.a.	n.a.	n.a.	n.a.	n.a.
n.a.	1,3-PDO	n.a.	n.a.	n.a.	n.a.	n.a.	n.a.
n.a.	2-3 BDO	n.a.	n.a.	n.a.	n.a.	n.a.	n.a.
n.a.	Ethanol	n.a.	n.a.	n.a.	n.a.	n.a.	n.a.
n.a.	Isobutyrate RI	n.a.	n.a.	n.a.	n.a.	n.a.	n.a.

n.a.	Butyrate RI	n.a.	n.a.	n.a.	n.a.	n.a.	n.a.

Chromatogram and SST Results

Injection Details

Injection Name:	**citrate,succinate, maltate,fumarate 20**	*Run Time (min):*	**20,00**
Vial Number:	**3:86**	*Injection Volume:*	**2,00**
Injection Type:	**Calibration Standard**	*Channel:*	**RI_CH_1**
Calibration Level:	**1**	*Wavelength:*	**n.a.**
Instrument Method:	**Default method LC2030C 45 gr 20 min**	*Bandwidth:*	**n.a.**
Processing Method:	**Processing Method LC2030 45 gr**	*Dilution Factor:*	**1,0000**
Injection Date/Time:	**17/Jan/23 17:41**	*Sample Weight:*	**1,0000**

Chromatogram

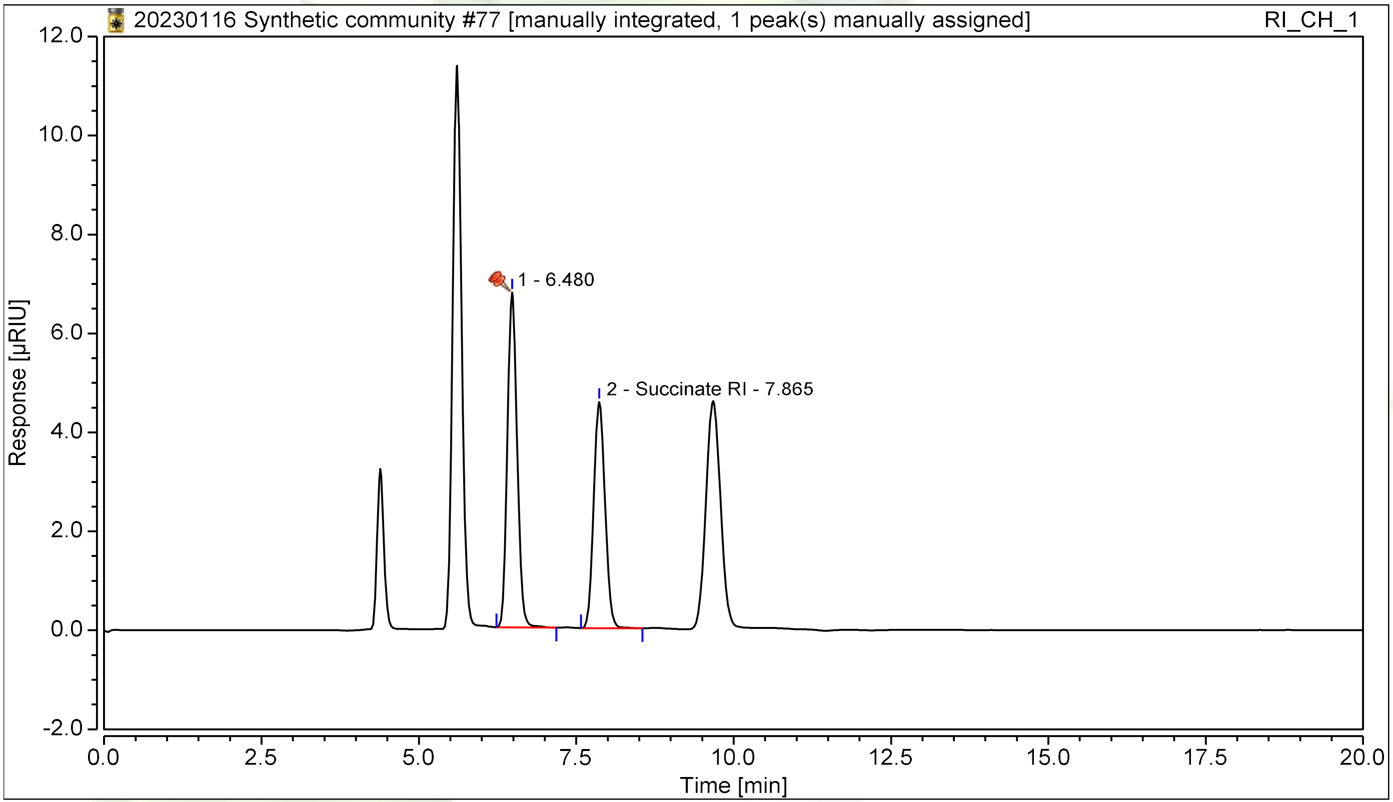

SST Results

No.	Name	Inj.Condition	Peak	Test Result	Injection

Number of executed test cases: **n.a.** **Total Result:** **Passed**

Chromatogram and Results

Injection Details

Injection Name:	**citrate,succinate, maltate,fumarate 30**	*Run Time (min):*	**20,00**
Vial Number:	**3:86**	*Injection Volume:*	**3,00**
Injection Type:	**Calibration Standard**	*Channel:*	**RI_CH_1**
Calibration Level:	**1**	*Wavelength:*	**n.a.**
Instrument Method:	**Default method LC2030C 45 gr 20 min**	*Bandwidth:*	**n.a.**
Processing Method:	**Processing Method LC2030 45 gr**	*Dilution Factor:*	**1,0000**
Injection Date/Time:	**17/Jan/23 18:02**	*Sample Weight:*	**1,0000**

Chromatogram

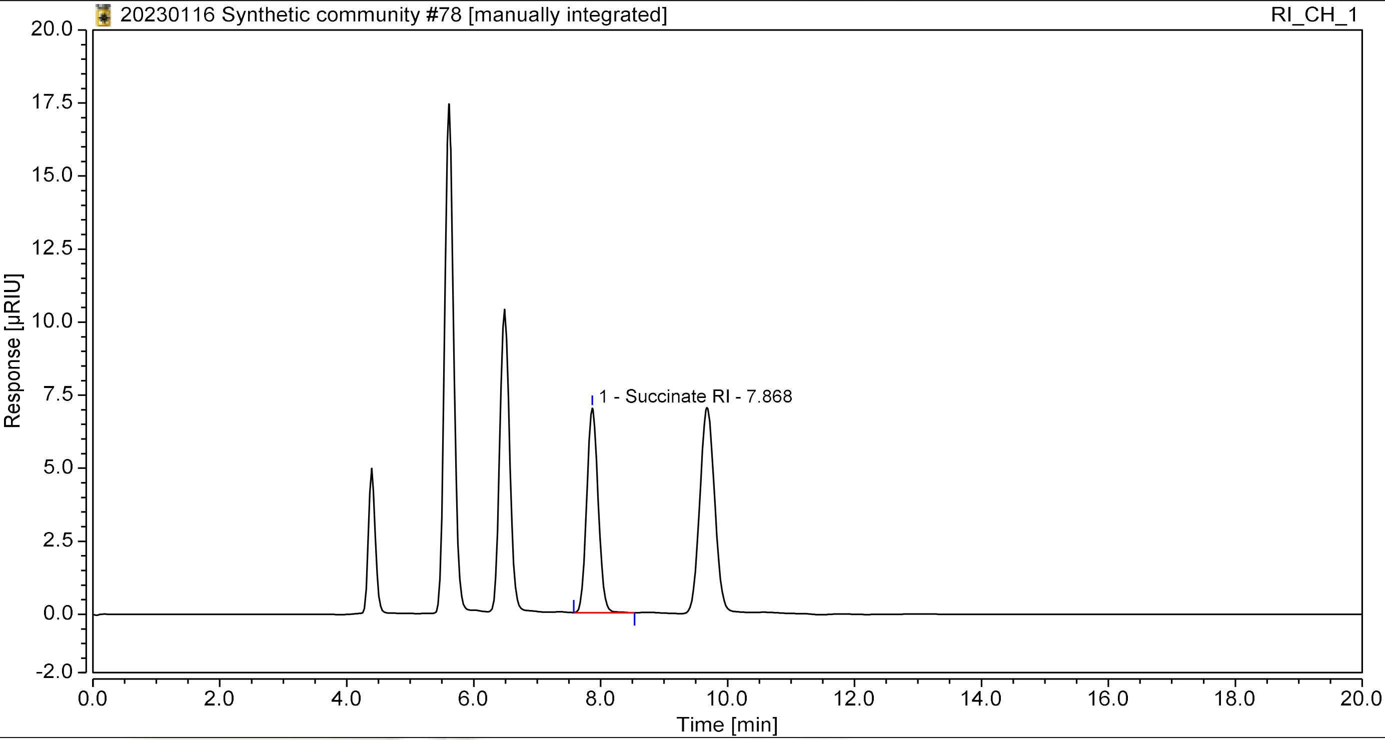

Integration Results

No.	Peak Name	Retention Time min	Area µRIU*min	Height µRIU	Relative Area %	Relative Height %	Amount
n.a.	GlcNAc	n.a.	n.a.	n.a.	n.a.	n.a.	n.a.
n.a.	Citrate	n.a.	n.a.	n.a.	n.a.	n.a.	n.a.
n.a.	Glucose	n.a.	n.a.	n.a.	n.a.	n.a.	n.a.
n.a.	Galactose	n.a.	n.a.	n.a.	n.a.	n.a.	n.a.
n.a.	Fucose	n.a.	n.a.	n.a.	n.a.	n.a.	n.a.
1	Succinate RI	7,868	1,468	6,990	100,00	100,00	30,3060
n.a.	Lactate RI	n.a.	n.a.	n.a.	n.a.	n.a.	n.a.
n.a.	glycerol	n.a.	n.a.	n.a.	n.a.	n.a.	n.a.
n.a.	Formate RI	n.a.	n.a.	n.a.	n.a.	n.a.	n.a.
n.a.	Acetate RI	n.a.	n.a.	n.a.	n.a.	n.a.	n.a.
n.a.	1,2 PDO RI	n.a.	n.a.	n.a.	n.a.	n.a.	n.a.
n.a.	1,3-PDO	n.a.	n.a.	n.a.	n.a.	n.a.	n.a.
n.a.	Propionate RI	n.a.	n.a.	n.a.	n.a.	n.a.	n.a.
n.a.	1,3-PDO	n.a.	n.a.	n.a.	n.a.	n.a.	n.a.
n.a.	2-3 BDO	n.a.	n.a.	n.a.	n.a.	n.a.	n.a.
n.a.	Ethanol	n.a.	n.a.	n.a.	n.a.	n.a.	n.a.
n.a.	Isobutyrate RI	n.a.	n.a.	n.a.	n.a.	n.a.	n.a.
n.a.	Butyrate RI	n.a.	n.a.	n.a.	n.a.	n.a.	n.a.
**Total:**			**1,468**	**6,990**	**100,00**	**100,00**	

Calibration

Calibration Details	Lactate RI		
Calibration Type	Lin	*Offset (C0)*	0,0000
Evaluation Type	Area	*Slope (C1)*	0,0342
Number of Calibration Points	3	*Curve (C2)*	0,0000
Number of disabled Calibration Points	0	*R-Square*	0,9989

Calibration Plot **Lactate RI**

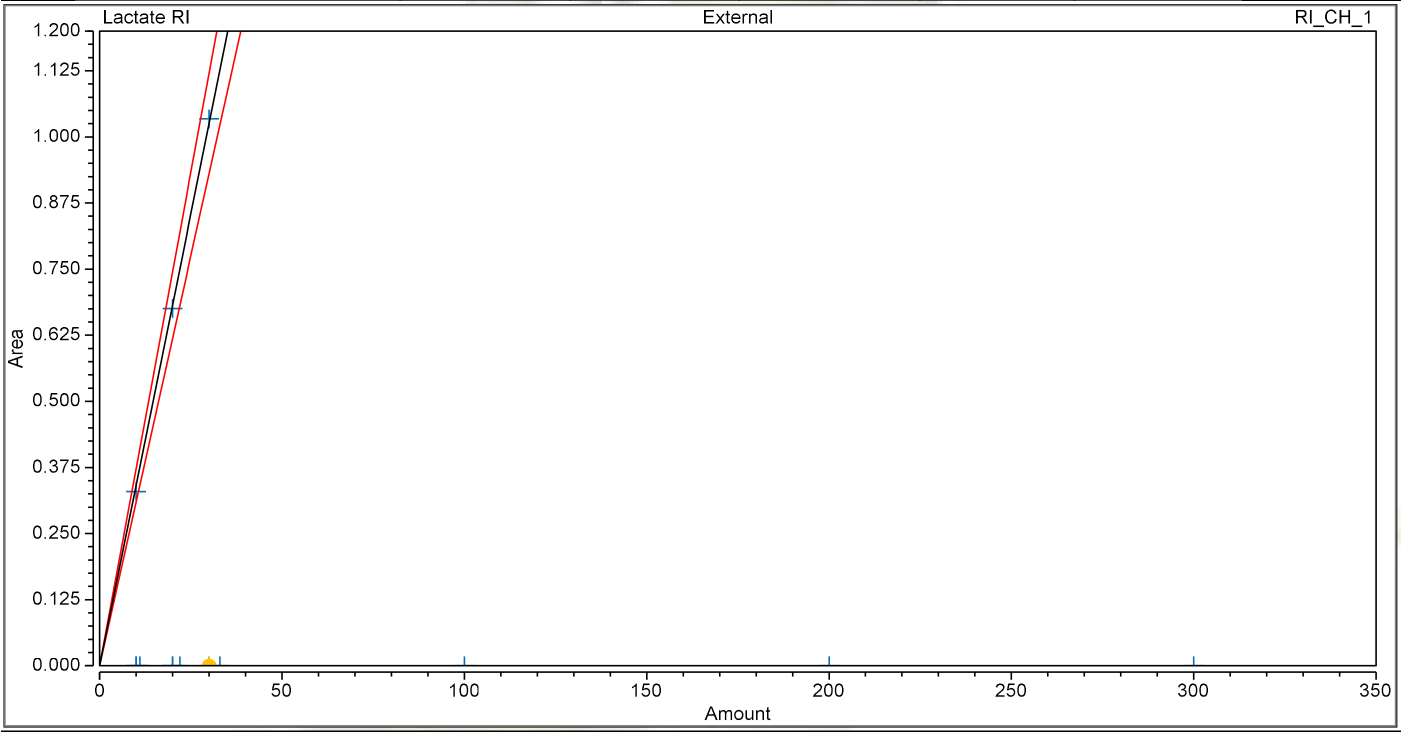

Calibration Results **Lactate RI**

No.	Injection Name	Calibration Level	X Value<br><br>RI_CH_1<br>Lactate RI	Y Value<br><br>RI_CH_1<br>Lactate RI	Y Value<br><br>RI_CH_1<br>Lactate RI	Area<br>µRIU*min<br>RI_CH_1<br>Lactate RI	Height<br>µRIU<br>RI_CH_1<br>Lactate RI
3	VFA 10	1	10,0000	0,3296	0,3296	0,330	1,574
4	VFA 20	1	20,0000	0,6757	0,6757	0,676	3,244
5	VFA 30	1	30,0000	1,0340	1,0340	1,034	4,963
6	1,2-Prop & 1- propane 10	1	10,0000	n.a.	n.a.	n.a.	n.a.
7	1,2-Prop & 1- propane 20	1	20,0000	n.a.	n.a.	n.a.	n.a.
8	1,2-Prop & 1- propane 30	1	30,0000	n.a.	n.a.	n.a.	n.a.
9	Meth & Eth 100	3	100,0000	n.a.	n.a.	n.a.	n.a.
10	Meth & Eth 200	3	200,0000	n.a.	n.a.	n.a.	n.a.
11	Meth & Eth 300	3	300,0000	n.a.	n.a.	n.a.	n.a.
73	Glucose, fructose,glycerol,2,3-bu	2	11,0000	n.a.	n.a.	n.a.	n.a.
74	Glucose, fructose,glycerol,2,3-bu	2	22,0000	n.a.	n.a.	n.a.	n.a.
75	Glucose, fructose,glycerol,2,3-bu	2	33,0000	n.a.	n.a.	n.a.	n.a.
76	citrate,succinate, maltate,fumarat	1	10,0000	n.a.	n.a.	n.a.	n.a.
77	citrate,succinate, maltate,fumarat	1	20,0000	n.a.	n.a.	n.a.	n.a.
78	citrate,succinate, maltate,fumarat	1	30,0000	n.a.	n.a.	n.a.	n.a.

Peak Analysis

Injection Details

Injection Name:	**citrate,succinate, maltate,fumarate 30**	*Run Time (min):*	**20,00**
Vial Number:	**3:86**	*Injection Volume:*	**3,00**
Injection Type:	**Calibration Standard**	*Channel:*	**RI_CH_1**
Calibration Level:	**1**	*Wavelength:*	**n.a.**
Instrument Method:	**Default method LC2030C 45 gr 20 min**	*Bandwidth:*	**n.a.**
Processing Method:	**Processing Method LC2030 45 gr**	*Dilution Factor:*	**1,0000**
Injection Date/Time:	**17/Jan/23 18:02**	*Sample Weight:*	**1,0000**

Chromatogram

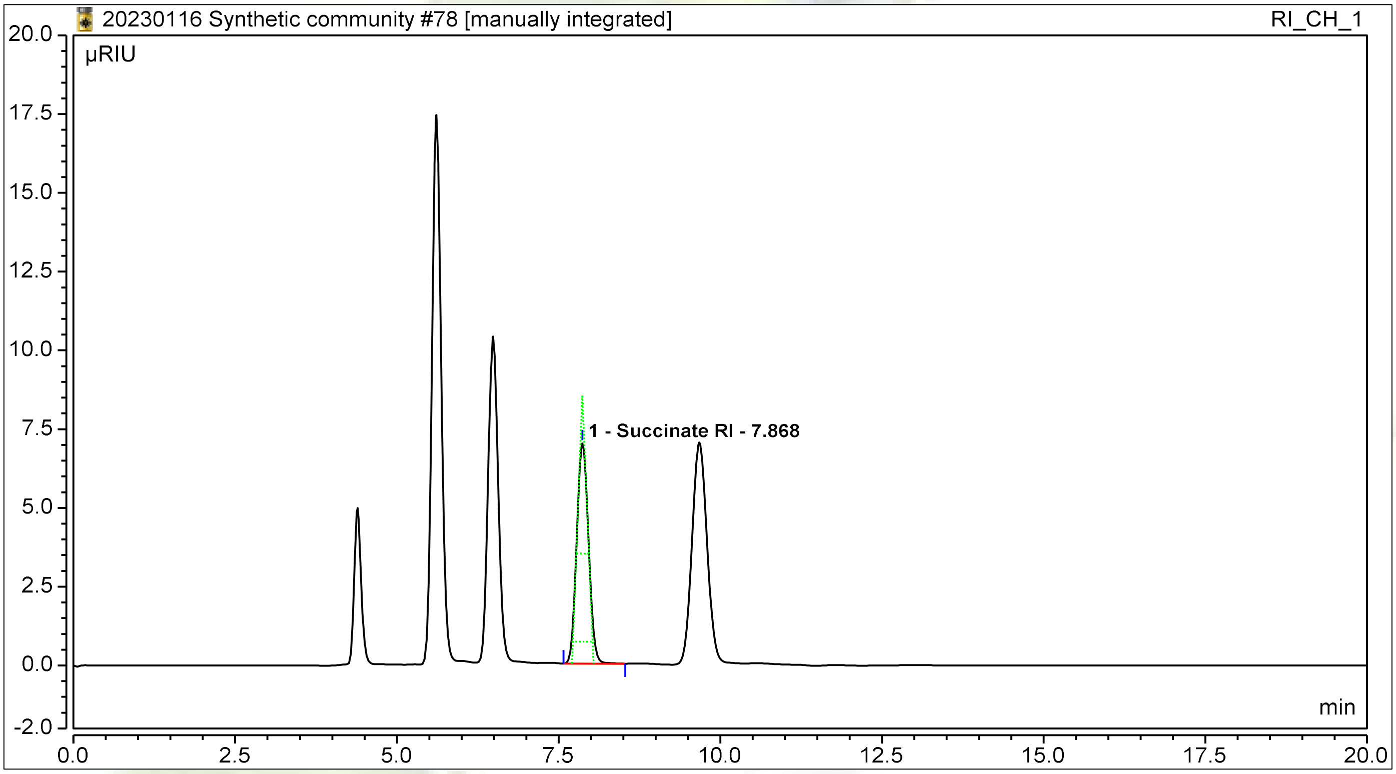

Peak Results

No.	Peak Name	Retention Time min	Width (50%) min	Type	Resolution (EP)	Asymmetry (EP)	Plates (EP)
n.a.	GlcNAc	n.a.	n.a.	n.a.	n.a.	n.a.	n.a.
n.a.	Citrate	n.a.	n.a.	n.a.	n.a.	n.a.	n.a.
n.a.	Glucose	n.a.	n.a.	n.a.	n.a.	n.a.	n.a.
n.a.	Galactose	n.a.	n.a.	n.a.	n.a.	n.a.	n.a.
n.a.	Fucose	n.a.	n.a.	n.a.	n.a.	n.a.	n.a.
1	Succinate RI	7,868	0,197	BMB	n.a.	1,08	8870
n.a.	Lactate RI	n.a.	n.a.	n.a.	n.a.	n.a.	n.a.
n.a.	glycerol	n.a.	n.a.	n.a.	n.a.	n.a.	n.a.
n.a.	Formate RI	n.a.	n.a.	n.a.	n.a.	n.a.	n.a.
n.a.	Acetate RI	n.a.	n.a.	n.a.	n.a.	n.a.	n.a.
n.a.	1,2 PDO RI	n.a.	n.a.	n.a.	n.a.	n.a.	n.a.
n.a.	1,3-PDO	n.a.	n.a.	n.a.	n.a.	n.a.	n.a.
n.a.	Propionate RI	n.a.	n.a.	n.a.	n.a.	n.a.	n.a.
n.a.	1,3-PDO	n.a.	n.a.	n.a.	n.a.	n.a.	n.a.
n.a.	2-3 BDO	n.a.	n.a.	n.a.	n.a.	n.a.	n.a.
n.a.	Ethanol	n.a.	n.a.	n.a.	n.a.	n.a.	n.a.
n.a.	Isobutyrate RI	n.a.	n.a.	n.a.	n.a.	n.a.	n.a.
n.a.	Butyrate RI	n.a.	n.a.	n.a.	n.a.	n.a.	n.a.

Chromeleon (c) Dionex
Version 7.2.10.25868

Chromatogram and SST Results

Injection Details

Injection Name:	**citrate,succinate, maltate,fumarate 30**	*Run Time (min):*	**20,00**
Vial Number:	**3:86**	*Injection Volume:*	**3,00**
Injection Type:	**Calibration Standard**	*Channel:*	**RI_CH_1**
Calibration Level:	**1**	*Wavelength:*	**n.a.**
Instrument Method:	**Default method LC2030C 45 gr 20 min**	*Bandwidth:*	**n.a.**
Processing Method:	**Processing Method LC2030 45 gr**	*Dilution Factor:*	**1,0000**
Injection Date/Time:	**17/Jan/23 18:02**	*Sample Weight:*	**1,0000**

Chromatogram

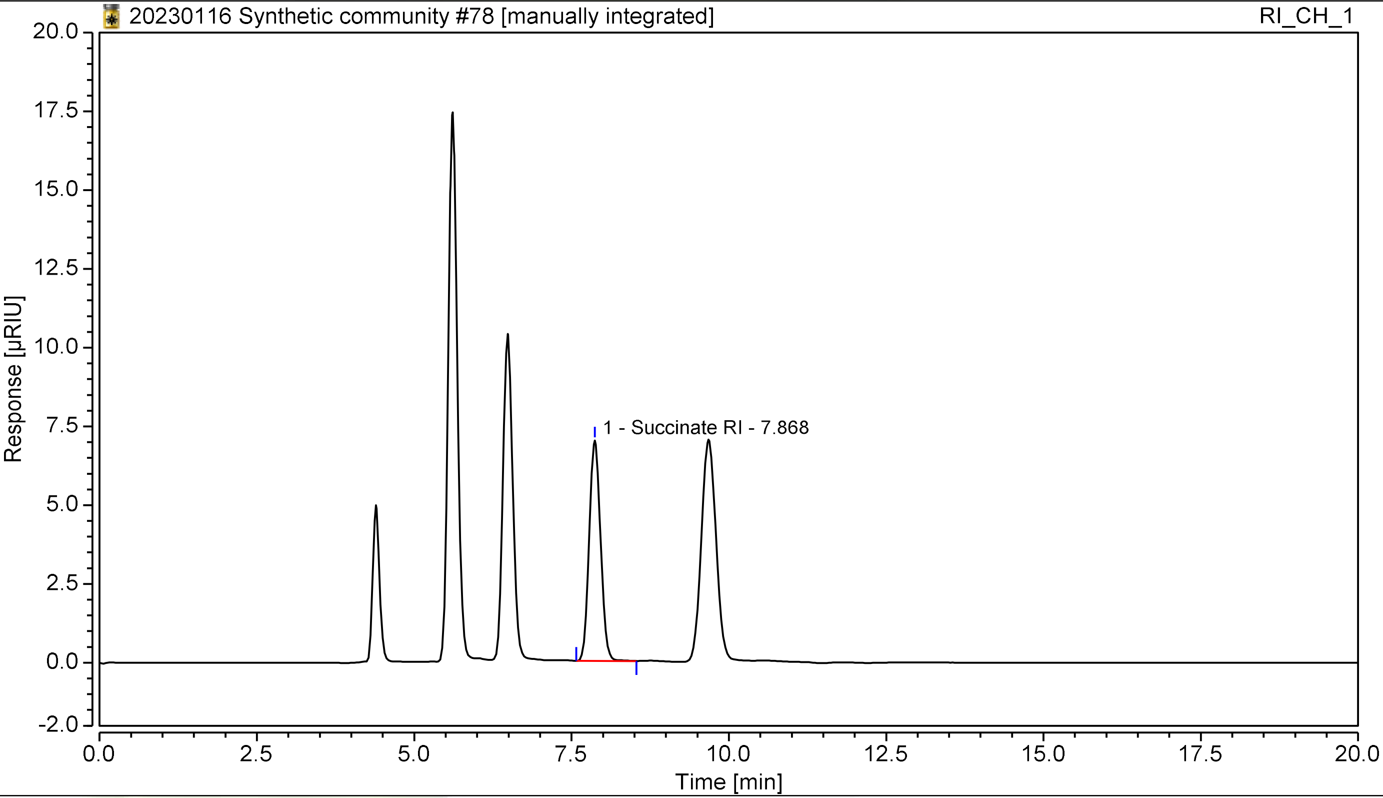

SST Results

No.	Name	Inj.Condition	Peak	Test Result	Injection

Number of executed test cases: **n.a.** **Total Result:** **Passed**

Summary

Sequence Details

Name:	**20230116 Synthetic community**	*Created On:*	**08/Dec/17 07:28:29**
Directory:	**HPLC-DATA\8_LC-2030C\Data2023\Maryse\Yu**	*Created By:*	**HPLC install**
Data Vault:	**AZR_CHROM_DATA_MIB-SSB**	*Updated On:*	**11/Apr/24 13:16:59**
No. of Injections:	**78**	*Updated By:*	**mib007**

By Component **Lactate RI**

No.	Injection Name	Ret.Time min RI_CH_1 Lactate RI	Area µRIU*min RI_CH_1 Lactate RI	Height µRIU RI_CH_1 Lactate RI	Amount RI_CH_1 Lactate RI	Rel.Area % RI_CH_1 Lactate RI	Peak Type RI_CH_1 Lactate RI
1	water 1	n.a.	n.a.	n.a.	n.a.	n.a.	n.a.
2	no injection	n.a.	n.a.	n.a.	n.a.	n.a.	n.a.
3	VFA 10	8,265	0,330	1,574	9,649	22,89	BMB
4	VFA 20	8,267	0,676	3,244	19,778	23,28	BM
5	VFA 30	8,267	1,034	4,963	30,265	23,21	BM
6	1,2-Prop & 1- propane 10	n.a.	n.a.	n.a.	n.a.	n.a.	n.a.
7	1,2-Prop & 1- propane 20	n.a.	n.a.	n.a.	n.a.	n.a.	n.a.
8	1,2-Prop & 1- propane 30	n.a.	n.a.	n.a.	n.a.	n.a.	n.a.
9	Meth & Eth 100	n.a.	n.a.	n.a.	n.a.	n.a.	n.a.
10	Meth & Eth 200	n.a.	n.a.	n.a.	n.a.	n.a.	n.a.
11	Meth & Eth 300	n.a.	n.a.	n.a.	n.a.	n.a.	n.a.
12	16.93 acetate	n.a.	n.a.	n.a.	n.a.	n.a.	n.a.
13	13.68 propionate	n.a.	n.a.	n.a.	n.a.	n.a.	n.a.
14	GOSFOS t72 r1	n.a.	n.a.	n.a.	n.a.	n.a.	n.a.
15	GOSFOS t72 r2	n.a.	n.a.	n.a.	n.a.	n.a.	n.a.
16	GOSFOS t72 r3	n.a.	n.a.	n.a.	n.a.	n.a.	n.a.
17	GOSFOS t96 r1	n.a.	n.a.	n.a.	n.a.	n.a.	n.a.
18	GOSFOS t96 r2	n.a.	n.a.	n.a.	n.a.	n.a.	n.a.
19	GOSFOS t96 r3	n.a.	n.a.	n.a.	n.a.	n.a.	n.a.
20	GOSFOS t120 r1	n.a.	n.a.	n.a.	n.a.	n.a.	n.a.
21	GOSFOS t120 r2	n.a.	n.a.	n.a.	n.a.	n.a.	n.a.
22	GOSFOS t120 r3	n.a.	n.a.	n.a.	n.a.	n.a.	n.a.
23	GOSFOSEXTR t72 r1	8,273	0,003	0,021	0,090	0,34	BMB*
24	GOSFOSEXTR t72 r2	8,275	0,031	0,176	0,907	2,26	BMB*
25	GOSFOSEXTR t72 r3	8,283	0,011	0,057	0,326	1,27	Rd
26	GOSFOSEXTR t96 r1	8,285	0,003	0,019	0,085	0,46	BMB*
27	GOSFOSEXTR t96 r2	8,277	0,021	0,133	0,610	1,29	BMB*
28	GOSFOSEXTR t96 r3	8,278	0,009	0,047	0,251	1,04	Rd
29	GOSFOSEXTR t120 r1	8,282	0,006	0,035	0,177	0,66	BMB*
30	GOSFOSEXTR t120 r2	8,265	0,008	0,047	0,242	1,05	BMB*
31	GOSFOSEXTR t120 r3	8,278	0,011	0,058	0,320	1,32	Rd
32	MUCHMO1 t24 r1	8,277	0,001	0,011	0,026	0,27	BMB*
33	MUCHMO1 t24 r2	8,268	0,008	0,042	0,241	2,23	BMB*
34	MUCHMO1 t24 r3	n.a.	n.a.	n.a.	n.a.	n.a.	n.a.
35	MUCHMO1 t72 r1	n.a.	n.a.	n.a.	n.a.	n.a.	n.a.
36	MUCHMO1 t72 r2	n.a.	n.a.	n.a.	n.a.	n.a.	n.a.
37	MUCHMO1 t72 r3	n.a.	n.a.	n.a.	n.a.	n.a.	n.a.
38	MUCHMO1 t96 r1	n.a.	n.a.	n.a.	n.a.	n.a.	n.a.
39	MUCHMO1 t96 r2	n.a.	n.a.	n.a.	n.a.	n.a.	n.a.
40	MUCHMO1 t96 r3	n.a.	n.a.	n.a.	n.a.	n.a.	n.a.
41	MUCHMO1 t120 r1	n.a.	n.a.	n.a.	n.a.	n.a.	n.a.
42	MUCHMO1 t120 r2	n.a.	n.a.	n.a.	n.a.	n.a.	n.a.
43	MUCHMO1 t120 r3	n.a.	n.a.	n.a.	n.a.	n.a.	n.a.

Chromeleon (c) Dionex
Version 7.2.10.25868

44	MUCHMO2 t72 r1	n.a.	n.a.	n.a.	n.a.	n.a.	n.a.
45	MUCHMO2 t72 r2	n.a.	n.a.	n.a.	n.a.	n.a.	n.a.
46	MUCHMO2 t72 r3	n.a.	n.a.	n.a.	n.a.	n.a.	n.a.
47	MUCHMO2 t96 r1	n.a.	n.a.	n.a.	n.a.	n.a.	n.a.
48	MUCHMO2 t96 r2	n.a.	n.a.	n.a.	n.a.	n.a.	n.a.
49	MUCHMO2 t96 r3	n.a.	n.a.	n.a.	n.a.	n.a.	n.a.
50	MUCHMO2 t120 r1	n.a.	n.a.	n.a.	n.a.	n.a.	n.a.
51	MUCHMO2 t120 r2	n.a.	n.a.	n.a.	n.a.	n.a.	n.a.
52	no injection (after disconec	n.a.	n.a.	n.a.	n.a.	n.a.	n.a.
53	no injection	n.a.	n.a.	n.a.	n.a.	n.a.	n.a.
54	MUCHMO2 t120 r3	n.a.	n.a.	n.a.	n.a.	n.a.	n.a.
55	MUC t72 r1	n.a.	n.a.	n.a.	n.a.	n.a.	n.a.
56	MUC t72 r2	n.a.	n.a.	n.a.	n.a.	n.a.	n.a.
57	MUC t72 r3	n.a.	n.a.	n.a.	n.a.	n.a.	n.a.
58	MUC t96 r1	n.a.	n.a.	n.a.	n.a.	n.a.	n.a.
59	MUC t96 r2	n.a.	n.a.	n.a.	n.a.	n.a.	n.a.
60	MUC t96 r3	n.a.	n.a.	n.a.	n.a.	n.a.	n.a.
61	MUC t120 r1	n.a.	n.a.	n.a.	n.a.	n.a.	n.a.
62	MUC t120 r2	n.a.	n.a.	n.a.	n.a.	n.a.	n.a.
63	MUC t120 r3	n.a.	n.a.	n.a.	n.a.	n.a.	n.a.
64	GOSFOSMUC t72 r1	n.a.	n.a.	n.a.	n.a.	n.a.	n.a.
65	GOSFOSMUC t72 r2	n.a.	n.a.	n.a.	n.a.	n.a.	n.a.
66	GOSFOSMUC t72 r3	n.a.	n.a.	n.a.	n.a.	n.a.	n.a.
67	GOSFOSMUC t96 r1	n.a.	n.a.	n.a.	n.a.	n.a.	n.a.
68	GOSFOSMUC t96 r2	n.a.	n.a.	n.a.	n.a.	n.a.	n.a.
69	GOSFOSMUC t96 r3	n.a.	n.a.	n.a.	n.a.	n.a.	n.a.
70	GOSFOSMUC t120 r1	n.a.	n.a.	n.a.	n.a.	n.a.	n.a.
71	GOSFOSMUC t120 r2	n.a.	n.a.	n.a.	n.a.	n.a.	n.a.
72	GOSFOSMUC t120 r3	n.a.	n.a.	n.a.	n.a.	n.a.	n.a.
73	Glucose, fructose,glycerol,2	n.a.	n.a.	n.a.	n.a.	n.a.	n.a.
74	Glucose, fructose,glycerol,2	n.a.	n.a.	n.a.	n.a.	n.a.	n.a.
75	Glucose, fructose,glycerol,2	n.a.	n.a.	n.a.	n.a.	n.a.	n.a.
76	citrate,succinate, maltate,fu	n.a.	n.a.	n.a.	n.a.	n.a.	n.a.
77	citrate,succinate, maltate,fu	n.a.	n.a.	n.a.	n.a.	n.a.	n.a.
78	citrate,succinate, maltate,fu	n.a.	n.a.	n.a.	n.a.	n.a.	n.a.

Chromeleon (c) Dionex
Version 7.2.10.25868
