# Supplementary material for: Milk and mucin glycans orchestrate a synthetic infant gut microbiota structure
Source: FEMS Microbiol Ecol. 2025 Jun 25;101(8):fiaf069. doi: 10.1093/femsec/fiaf069 (PMC12268331; doi:10.1093/femsec/fiaf069)
Supplement: fiaf069_Supplemental_Files [file fiaf069_supplemental_files.zip › Supplementary_Data_2_Belzer_HPLC_run2.pdf]

Sequence Overview

Sequence Details

Name:	**20230613 Mucin HMO Synthetic communit**	*Created On:*	**08-dec-17 07:28:29**
Directory:	**HPLC-DATA\8_LC-2030C\Data2023\Maryse**	*Created By:*	**HPLC install**
Data Vault:	**AZR_CHROM_DATA_MIB-SSB**	*Updated On:*	**13-sep-23 13:52:19**
No. of Injections:	**68**	*Updated By:*	**mib007**

Injection Details

No.	Injection Name	Position	Type	Level	Amount<br>Lactate RI	Amount<br>Succinate RI
1	water 1	3:A1	Unknown		n.a.	n.a.
2	no injection	3:A1	Blank		n.a.	n.a.
3	VFA 10	3:A2	Calibration Standard	1	9,608680617	n.a.
4	VFA 20	3:A2	Calibration Standard	1	19,86148218	n.a.
5	VFA 30	3:A2	Calibration Standard	1	30,222785	n.a.
6	1,2-Prop & 1- propane	3:A3	Calibration Standard	1	n.a.	n.a.
7	1,2-Prop & 1- propane	3:A3	Calibration Standard	1	n.a.	n.a.
8	1,2-Prop & 1- propane	3:A3	Calibration Standard	1	n.a.	n.a.
9	Meth & Eth 100	3:A4	Calibration Standard	1	n.a.	n.a.
10	Meth & Eth 200	3:A4	Calibration Standard	1	n.a.	n.a.
11	Meth & Eth 300	3:A4	Calibration Standard	1	n.a.	n.a.
12	16.93 acetate	3:A5	Unknown		n.a.	n.a.
13	13.68 propionate	3:A6	Unknown		n.a.	n.a.
14	40 5HMO1 t24 r1	3:A7	Unknown		0,5831076016	n.a.
15	41 5HMO1 t24 r2	3:A8	Unknown		1,737608502	n.a.
16	42 5HMO1 t24 r3	3:A9	Unknown		1,53232096	n.a.
17	43 5HMO1 t48 r1	3:A10	Unknown		0,4145481143	n.a.
18	44 5HMO1 t48 r2	3:A11	Unknown		2,034851795	n.a.
19	45 5HMO1 t48 r3	3:A12	Unknown		1,649949768	n.a.
20	46 5HMO1 t72 r1	3:B1	Unknown		0,2318090606	n.a.
21	47 5HMO1 t72 r2	3:B2	Unknown		1,859085279	n.a.
22	48 5HMO1 t72 r3	3:B3	Unknown		0,368837557	n.a.
23	49 5HMO1 t96 r1	3:B4	Unknown		0,4436150137	n.a.
24	50 5HMO1 t96 r2	3:B5	Unknown		0,8423620683	n.a.
25	51 5HMO1 t96 r3	3:B6	Unknown		1,094280538	n.a.
26	52 5HMO1 t120 r1	3:B7	Unknown		0,1669391665	n.a.
27	53 5HMO1 t120 r2	3:B8	Unknown		1,003014187	n.a.
28	54 5HMO1 t120 r3	3:B9	Unknown		0,734704135	n.a.
29	10 GOSFOS t24 r1	3:B10	Unknown		0,2340476515	n.a.
30	11 GOSFOS t24 r2	3:B11	Unknown		0,158441666	n.a.
31	12 GOSFOS t24 r3	3:B12	Unknown		0,32514478	n.a.
32	16 GOSFOSEXTR t24	3:C1	Unknown		0,03548213516	n.a.
33	17 GOSFOSEXTR t24	3:C2	Unknown		0,4006706483	n.a.
34	18 GOSFOSEXTR t24	3:C3	Unknown		0,2696908026	n.a.
35	25 GOSFOS t48 r1	3:C4	Unknown		n.a.	n.a.
36	26 GOSFOS t48 r2	3:C5	Unknown		0,2827713959	n.a.
37	27 GOSFOS t48 r3	3:C6	Unknown		0,07283314982	n.a.
38	31 GOSFOSEXTR t48	3:C7	Unknown		n.a.	n.a.
39	32 GOSFOSEXTR t48	3:C8	Unknown		0,6329439976	n.a.
40	33 GOSFOSEXTR t48	3:C9	Unknown		0,2735004717	n.a.
41	1 MUCHMO1 t48 r1	3:C10	Unknown		n.a.	n.a.
42	2 MUCHMO1 t48 r2	3:C11	Unknown		n.a.	n.a.

43	3 MUCHMO1 t48 r3	3:C12	Unknown		n.a.	n.a.
44	4 MUCHMO2 t24 r1	3:D1	Unknown		n.a.	n.a.
45	5 MUCHMO2 t24 r2	3:D2	Unknown		n.a.	n.a.
46	6 MUCHMO2 t24 r3	3:D3	Unknown		n.a.	n.a.
47	7 MUC t24 r1	3:D4	Unknown		n.a.	n.a.
48	8 MUC t24 r2	3:D5	Unknown		n.a.	n.a.
49	9 MUC t24 r3	3:D6	Unknown		n.a.	n.a.
50	13 GOSFOSMUC t24	3:D7	Unknown		n.a.	n.a.
51	14 GOSFOSMUC t24	3:D8	Unknown		n.a.	n.a.
52	15 GOSFOSMUC t24	3:D9	Unknown		n.a.	n.a.
53	19 MUCHMO2 t48 r1	3:D10	Unknown		n.a.	n.a.
54	20 MUCHMO2 t48 r2	3:D11	Unknown		n.a.	n.a.
55	21 MUCHMO2 t48 r3	3:D12	Unknown		n.a.	n.a.
56	22 MUC t48 r1	3:E1	Unknown		n.a.	n.a.
57	23 MUC t48 r2	3:E2	Unknown		n.a.	n.a.
58	24 MUC t48 r3	3:E3	Unknown		n.a.	n.a.
59	28 GOSFOSMUC t48	3:E4	Unknown		n.a.	n.a.
60	29 GOSFOSMUC t48	3:E5	Unknown		n.a.	n.a.
61	30 GOSFOSMUC t48	3:E6	Unknown		n.a.	n.a.
62	34 MUCHMO3 t24 r1	3:E7	Unknown		n.a.	n.a.
63	35 MUCHMO3 t24 r2	3:E8	Unknown		n.a.	n.a.
64	36 MUCHMO3 t24 r3	3:E9	Unknown		n.a.	n.a.
65	37 MUCHMO3 t48 r1	3:E10	Unknown		n.a.	n.a.
66	38 MUCHMO3 t48 r2	3:E11	Unknown		n.a.	n.a.
67	39 MUCHMO3 t48 r3	3:E12	Unknown		n.a.	n.a.
68	Shutdown	3:F1	Blank		n.a.	n.a.

Chromatogram and Results

Injection Details

Injection Name:	**water 1**	*Run Time (min):*	**20,00**
Vial Number:	**3:A1**	*Injection Volume:*	**10,00**
Injection Type:	**Unknown**	*Channel:*	**RI_CH_1**
Calibration Level:		*Wavelength:*	**n.a.**
Instrument Method:	**Default method LC2030C 45 gr 20 min**	*Bandwidth:*	**n.a.**
Processing Method:	**Processing Method LC2030 45 gr**	*Dilution Factor:*	**1,0000**
Injection Date/Time:	**13-jun-23 11:57**	*Sample Weight:*	**1,0000**

Chromatogram

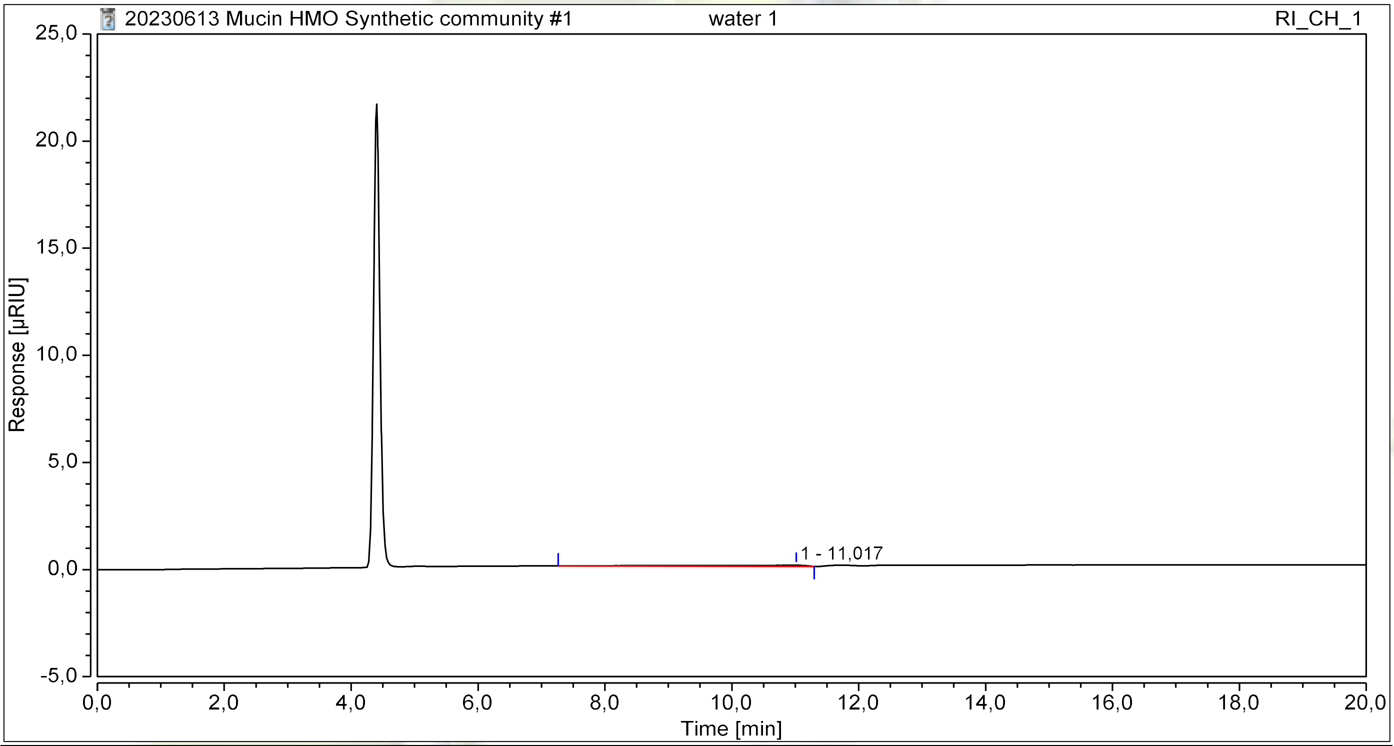

Integration Results

No.	Peak Name	Retention Time min	Area µRIU*min	Height µRIU	Relative Area %	Relative Height %	Amount
n.a.	GlcNAc	n.a.	n.a.	n.a.	n.a.	n.a.	n.a.
n.a.	Citrate	n.a.	n.a.	n.a.	n.a.	n.a.	n.a.
n.a.	Glucose	n.a.	n.a.	n.a.	n.a.	n.a.	n.a.
n.a.	Galactose	n.a.	n.a.	n.a.	n.a.	n.a.	n.a.
n.a.	Fucose	n.a.	n.a.	n.a.	n.a.	n.a.	n.a.
n.a.	Succinate RI	n.a.	n.a.	n.a.	n.a.	n.a.	n.a.
n.a.	Lactate RI	n.a.	n.a.	n.a.	n.a.	n.a.	n.a.
n.a.	glycerol	n.a.	n.a.	n.a.	n.a.	n.a.	n.a.
n.a.	Formate RI	n.a.	n.a.	n.a.	n.a.	n.a.	n.a.
n.a.	Acetate RI	n.a.	n.a.	n.a.	n.a.	n.a.	n.a.
n.a.	1,2 PDO RI	n.a.	n.a.	n.a.	n.a.	n.a.	n.a.
n.a.	1,3-PDO	n.a.	n.a.	n.a.	n.a.	n.a.	n.a.
1		11,017	0,115	0,054	100,00	100,00	n.a.
n.a.	Propionate RI	n.a.	n.a.	n.a.	n.a.	n.a.	n.a.
n.a.	1,3-PDO	n.a.	n.a.	n.a.	n.a.	n.a.	n.a.
n.a.	2-3 BDO	n.a.	n.a.	n.a.	n.a.	n.a.	n.a.
n.a.	Ethanol	n.a.	n.a.	n.a.	n.a.	n.a.	n.a.
n.a.	Isobutyrate RI	n.a.	n.a.	n.a.	n.a.	n.a.	n.a.
n.a.	Butyrate RI	n.a.	n.a.	n.a.	n.a.	n.a.	n.a.
**Total:**			**0,115**	**0,054**	**100,00**	**100,00**	

Peak Analysis

Injection Details

Injection Name:	**water 1**	*Run Time (min):*	**20,00**
Vial Number:	**3:A1**	*Injection Volume:*	**10,00**
Injection Type:	**Unknown**	*Channel:*	**RI_CH_1**
Calibration Level:		*Wavelength:*	**n.a.**
Instrument Method:	**Default method LC2030C 45 gr 20 min**	*Bandwidth:*	**n.a.**
Processing Method:	**Processing Method LC2030 45 gr**	*Dilution Factor:*	**1,0000**
Injection Date/Time:	**13-jun-23 11:57**	*Sample Weight:*	**1,0000**

Chromatogram

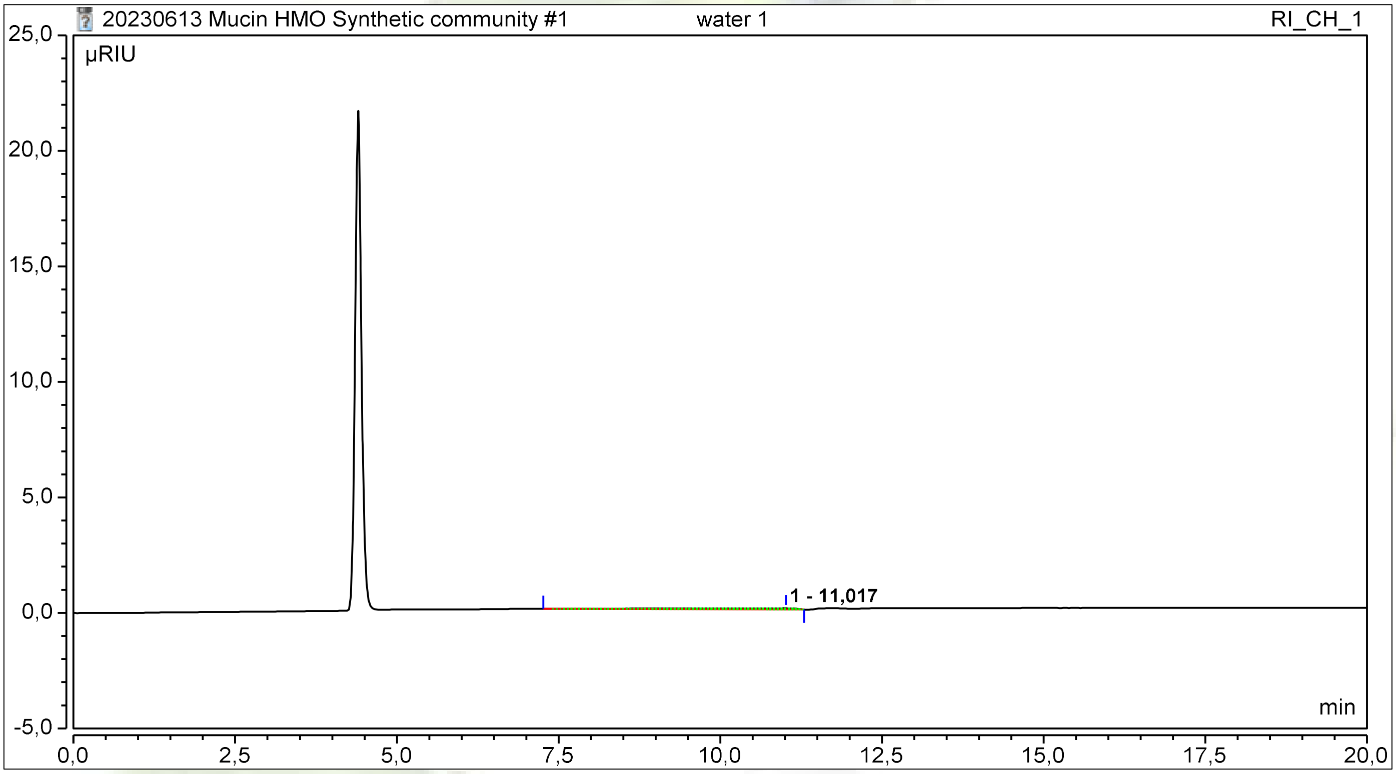

Peak Results

No.	Peak Name	Retention Time min	Width (50%) min	Type	Resolution (EP)	Asymmetry (EP)	Plates (EP)
n.a.	GlcNAc	n.a.	n.a.	n.a.	n.a.	n.a.	n.a.
n.a.	Citrate	n.a.	n.a.	n.a.	n.a.	n.a.	n.a.
n.a.	Glucose	n.a.	n.a.	n.a.	n.a.	n.a.	n.a.
n.a.	Galactose	n.a.	n.a.	n.a.	n.a.	n.a.	n.a.
n.a.	Fucose	n.a.	n.a.	n.a.	n.a.	n.a.	n.a.
n.a.	Succinate RI	n.a.	n.a.	n.a.	n.a.	n.a.	n.a.
n.a.	Lactate RI	n.a.	n.a.	n.a.	n.a.	n.a.	n.a.
n.a.	glycerol	n.a.	n.a.	n.a.	n.a.	n.a.	n.a.
n.a.	Formate RI	n.a.	n.a.	n.a.	n.a.	n.a.	n.a.
n.a.	Acetate RI	n.a.	n.a.	n.a.	n.a.	n.a.	n.a.
n.a.	1,2 PDO RI	n.a.	n.a.	n.a.	n.a.	n.a.	n.a.
n.a.	1,3-PDO	n.a.	n.a.	n.a.	n.a.	n.a.	n.a.
1		11,017	2,162	BMB	n.a.	0,54	144
n.a.	Propionate RI	n.a.	n.a.	n.a.	n.a.	n.a.	n.a.
n.a.	1,3-PDO	n.a.	n.a.	n.a.	n.a.	n.a.	n.a.
n.a.	2-3 BDO	n.a.	n.a.	n.a.	n.a.	n.a.	n.a.
n.a.	Ethanol	n.a.	n.a.	n.a.	n.a.	n.a.	n.a.
n.a.	Isobutyrate RI	n.a.	n.a.	n.a.	n.a.	n.a.	n.a.

n.a.	Butyrate RI	n.a.	n.a.	n.a.	n.a.	n.a.	n.a.

Chromeleon (c) Dionex
Version 7.2.10.25868

Chromatogram and SST Results

Injection Details

Injection Name:	**water 1**	*Run Time (min):*	**20,00**
Vial Number:	**3:A1**	*Injection Volume:*	**10,00**
Injection Type:	**Unknown**	*Channel:*	**RI_CH_1**
Calibration Level:		*Wavelength:*	**n.a.**
Instrument Method:	**Default method LC2030C 45 gr 20 min**	*Bandwidth:*	**n.a.**
Processing Method:	**Processing Method LC2030 45 gr**	*Dilution Factor:*	**1,0000**
Injection Date/Time:	**13-jun-23 11:57**	*Sample Weight:*	**1,0000**

Chromatogram

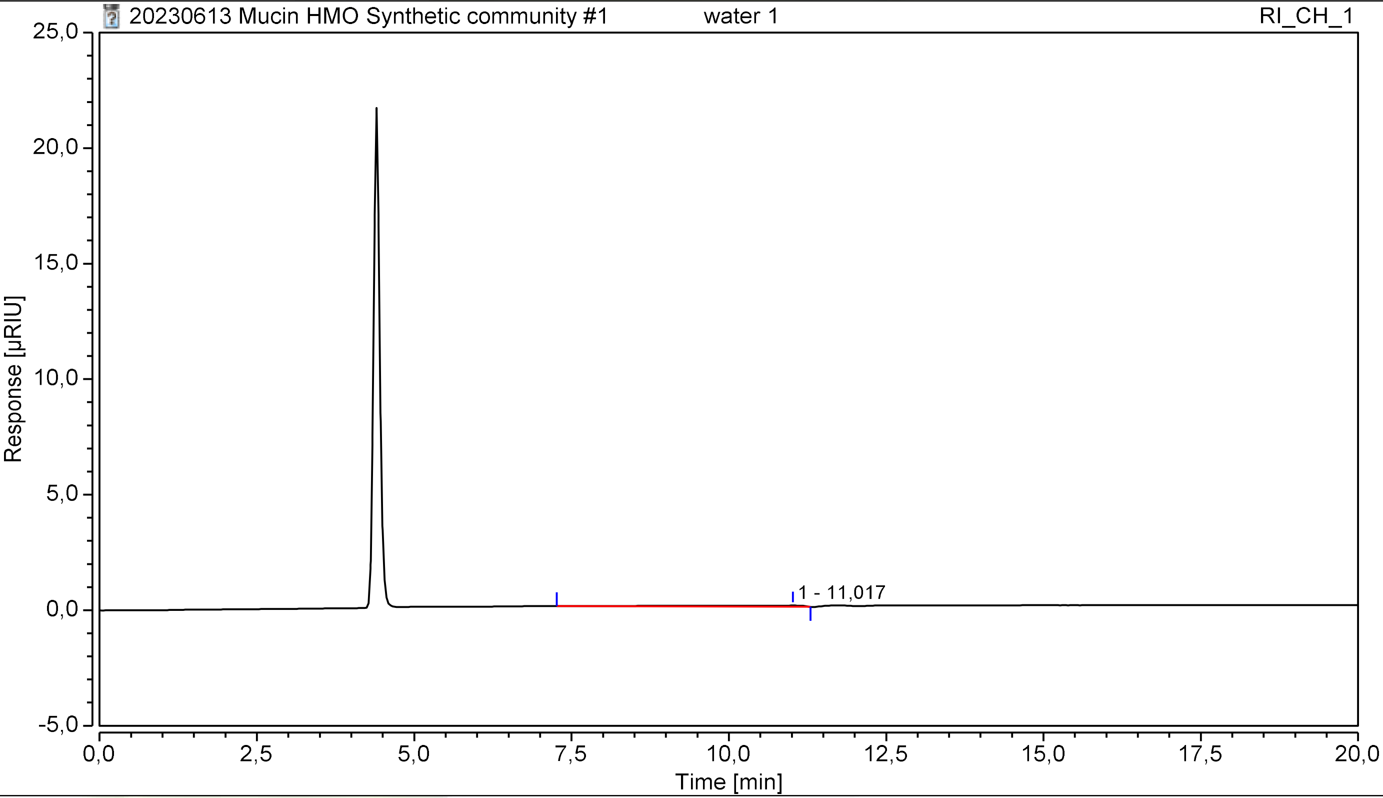

SST Results

No.	Name	Inj.Condition	Peak	Test Result	Injection

Number of executed test cases: **n.a.** **Total Result:** **Passed**

Chromatogram

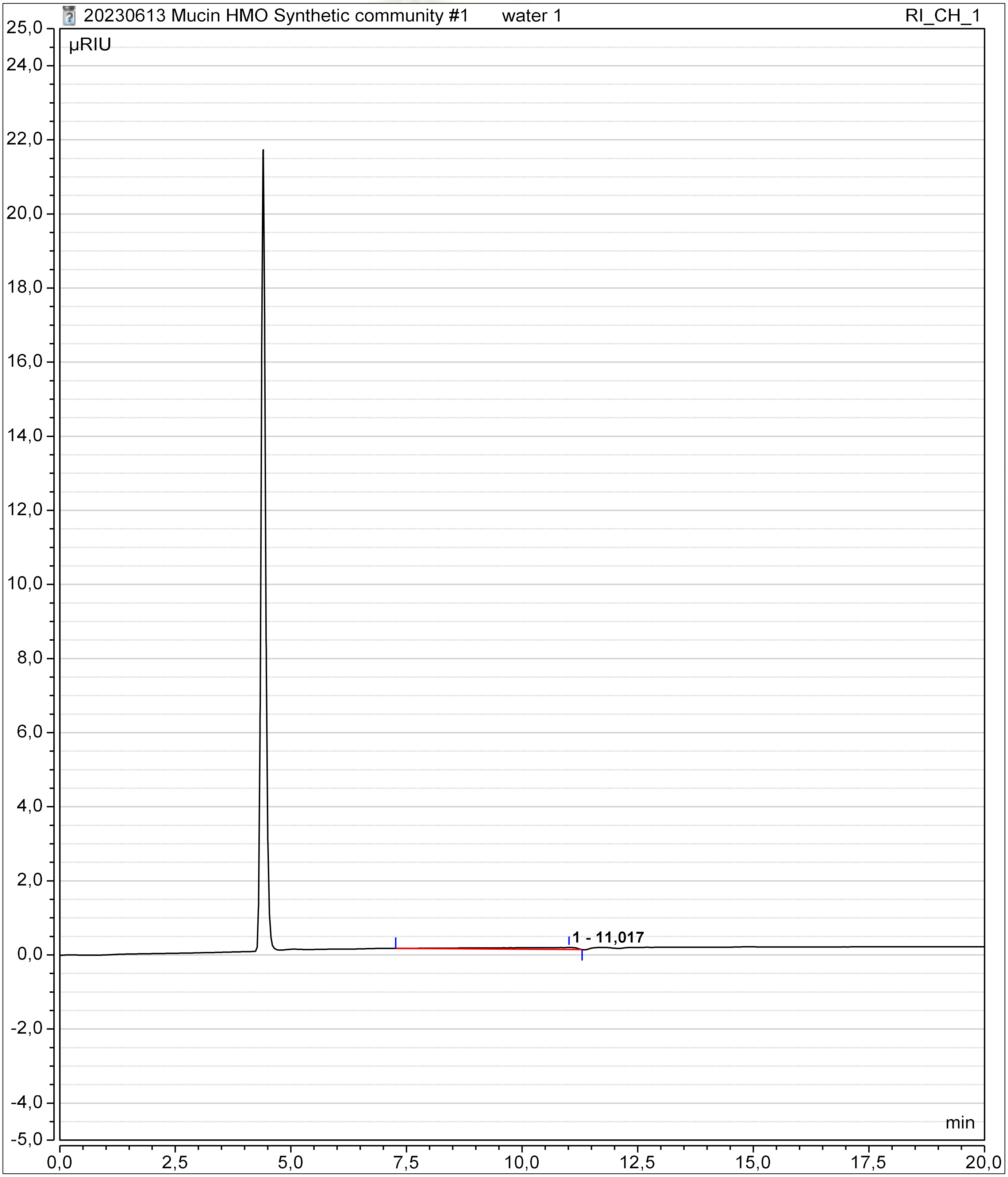

Chromatogram and Results

Injection Details

Injection Name:	**no injection**	*Run Time (min):*	**20,00**
Vial Number:	**3:A1**	*Injection Volume:*	**10,00**
Injection Type:	**Blank**	*Channel:*	**RI_CH_1**
Calibration Level:		*Wavelength:*	**n.a.**
Instrument Method:	**Default method LC2030C 45 gr 20 min**	*Bandwidth:*	**n.a.**
Processing Method:	**Processing Method LC2030 45 gr**	*Dilution Factor:*	**1,0000**
Injection Date/Time:	**13-jun-23 12:17**	*Sample Weight:*	**1,0000**

Chromatogram

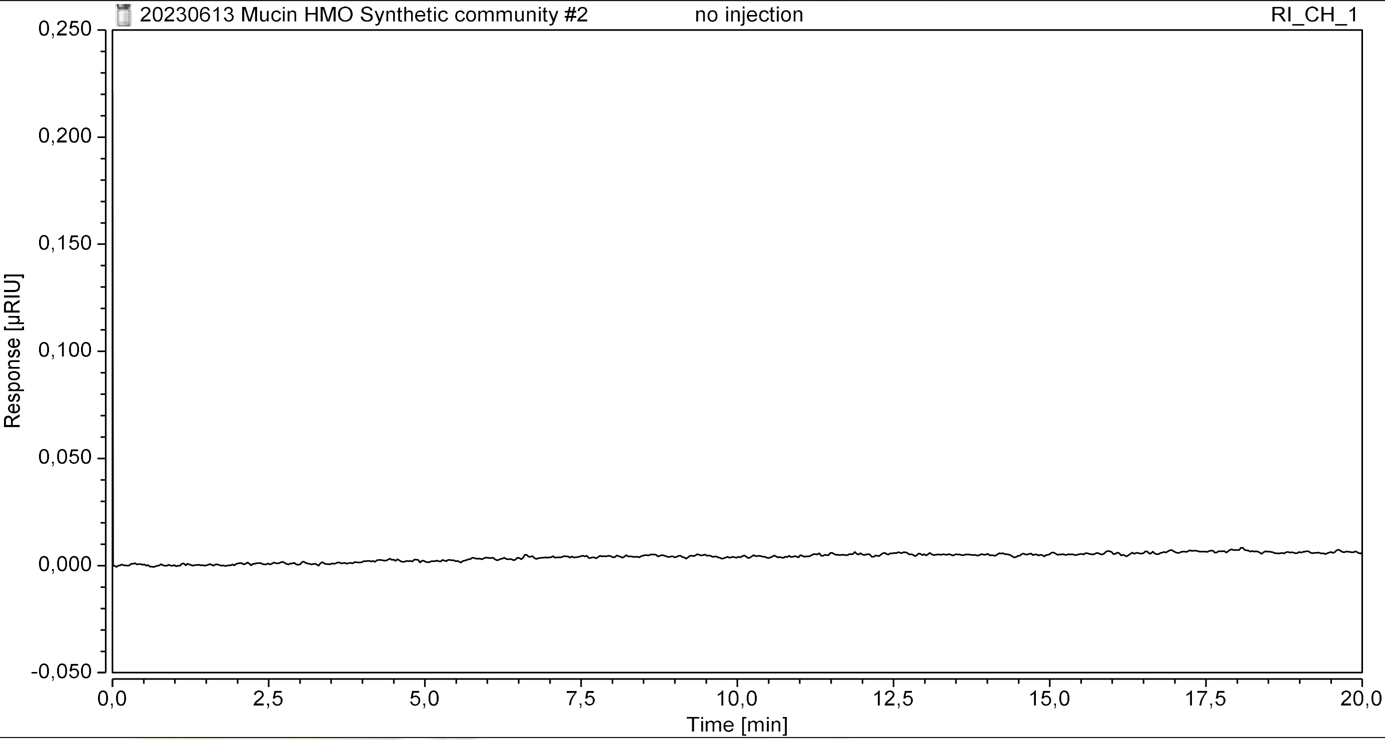

Integration Results

No.	Peak Name	Retention Time min	Area µRIU*min	Height µRIU	Relative Area %	Relative Height %	Amount
n.a.	GlcNAc	n.a.	n.a.	n.a.	n.a.	n.a.	n.a.
n.a.	Citrate	n.a.	n.a.	n.a.	n.a.	n.a.	n.a.
n.a.	Glucose	n.a.	n.a.	n.a.	n.a.	n.a.	n.a.
n.a.	Galactose	n.a.	n.a.	n.a.	n.a.	n.a.	n.a.
n.a.	Fucose	n.a.	n.a.	n.a.	n.a.	n.a.	n.a.
n.a.	Succinate RI	n.a.	n.a.	n.a.	n.a.	n.a.	n.a.
n.a.	Lactate RI	n.a.	n.a.	n.a.	n.a.	n.a.	n.a.
n.a.	glycerol	n.a.	n.a.	n.a.	n.a.	n.a.	n.a.
n.a.	Formate RI	n.a.	n.a.	n.a.	n.a.	n.a.	n.a.
n.a.	Acetate RI	n.a.	n.a.	n.a.	n.a.	n.a.	n.a.
n.a.	1,2 PDO RI	n.a.	n.a.	n.a.	n.a.	n.a.	n.a.
n.a.	1,3-PDO	n.a.	n.a.	n.a.	n.a.	n.a.	n.a.
n.a.	Propionate RI	n.a.	n.a.	n.a.	n.a.	n.a.	n.a.
n.a.	1,3-PDO	n.a.	n.a.	n.a.	n.a.	n.a.	n.a.
n.a.	2-3 BDO	n.a.	n.a.	n.a.	n.a.	n.a.	n.a.
n.a.	Ethanol	n.a.	n.a.	n.a.	n.a.	n.a.	n.a.
n.a.	Isobutyrate RI	n.a.	n.a.	n.a.	n.a.	n.a.	n.a.
n.a.	Butyrate RI	n.a.	n.a.	n.a.	n.a.	n.a.	n.a.
**Total:**			**0,000**	**0,000**	**0,00**	**0,00**	

Peak Analysis

Injection Details

Injection Name:	**no injection**	*Run Time (min):*	**20,00**
Vial Number:	**3:A1**	*Injection Volume:*	**10,00**
Injection Type:	**Blank**	*Channel:*	**RI_CH_1**
Calibration Level:		*Wavelength:*	**n.a.**
Instrument Method:	**Default method LC2030C 45 gr 20 min**	*Bandwidth:*	**n.a.**
Processing Method:	**Processing Method LC2030 45 gr**	*Dilution Factor:*	**1,0000**
Injection Date/Time:	**13-jun-23 12:17**	*Sample Weight:*	**1,0000**

Chromatogram

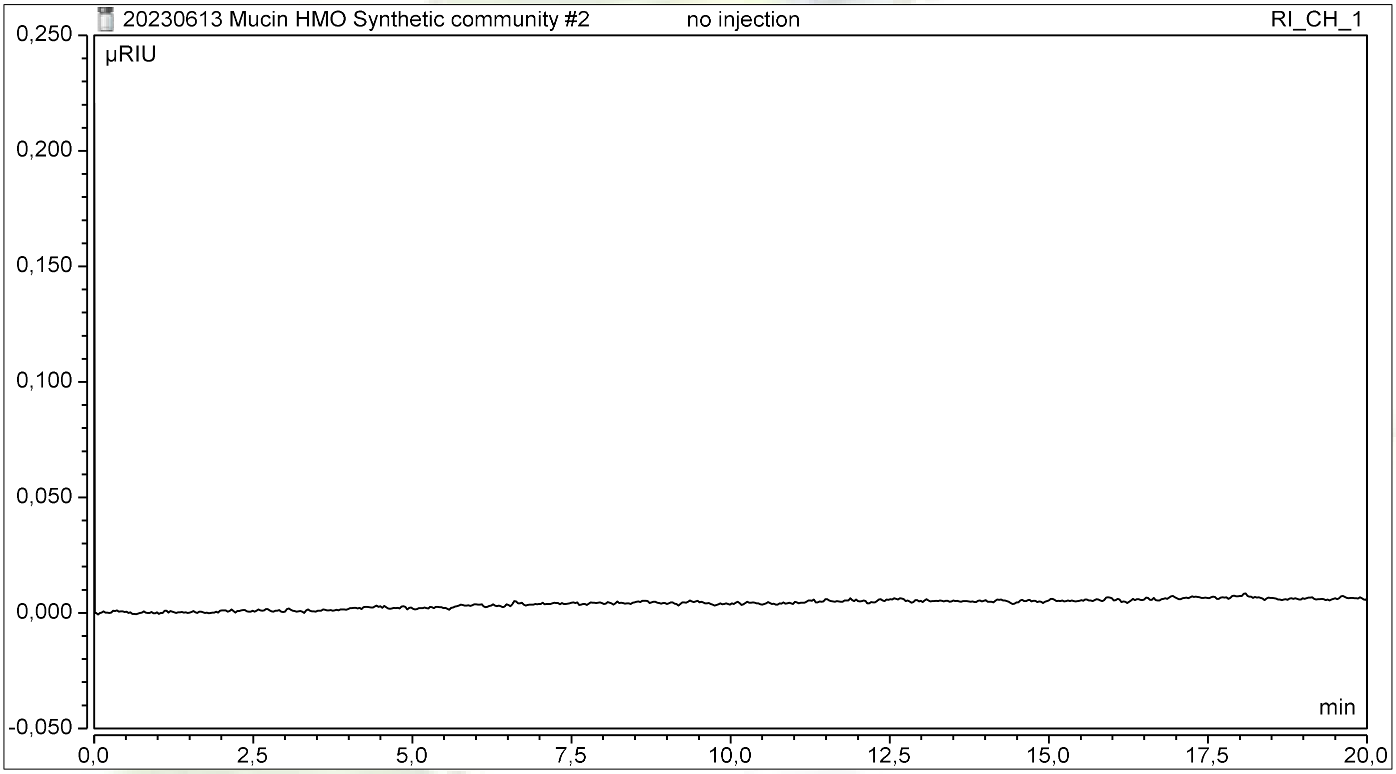

Peak Results

No.	Peak Name	Retention Time min	Width (50%) min	Type	Resolution (EP)	Asymmetry (EP)	Plates (EP)
n.a.	GlcNAc	n.a.	n.a.	n.a.	n.a.	n.a.	n.a.
n.a.	Citrate	n.a.	n.a.	n.a.	n.a.	n.a.	n.a.
n.a.	Glucose	n.a.	n.a.	n.a.	n.a.	n.a.	n.a.
n.a.	Galactose	n.a.	n.a.	n.a.	n.a.	n.a.	n.a.
n.a.	Fucose	n.a.	n.a.	n.a.	n.a.	n.a.	n.a.
n.a.	Succinate RI	n.a.	n.a.	n.a.	n.a.	n.a.	n.a.
n.a.	Lactate RI	n.a.	n.a.	n.a.	n.a.	n.a.	n.a.
n.a.	glycerol	n.a.	n.a.	n.a.	n.a.	n.a.	n.a.
n.a.	Formate RI	n.a.	n.a.	n.a.	n.a.	n.a.	n.a.
n.a.	Acetate RI	n.a.	n.a.	n.a.	n.a.	n.a.	n.a.
n.a.	1,2 PDO RI	n.a.	n.a.	n.a.	n.a.	n.a.	n.a.
n.a.	1,3-PDO	n.a.	n.a.	n.a.	n.a.	n.a.	n.a.
n.a.	Propionate RI	n.a.	n.a.	n.a.	n.a.	n.a.	n.a.
n.a.	1,3-PDO	n.a.	n.a.	n.a.	n.a.	n.a.	n.a.
n.a.	2-3 BDO	n.a.	n.a.	n.a.	n.a.	n.a.	n.a.
n.a.	Ethanol	n.a.	n.a.	n.a.	n.a.	n.a.	n.a.
n.a.	Isobutyrate RI	n.a.	n.a.	n.a.	n.a.	n.a.	n.a.
n.a.	Butyrate RI	n.a.	n.a.	n.a.	n.a.	n.a.	n.a.

Chromatogram and SST Results

Injection Details

Injection Name:	**no injection**	*Run Time (min):*	**20,00**
Vial Number:	**3:A1**	*Injection Volume:*	**10,00**
Injection Type:	**Blank**	*Channel:*	**RI_CH_1**
Calibration Level:		*Wavelength:*	**n.a.**
Instrument Method:	**Default method LC2030C 45 gr 20 min**	*Bandwidth:*	**n.a.**
Processing Method:	**Processing Method LC2030 45 gr**	*Dilution Factor:*	**1,0000**
Injection Date/Time:	**13-jun-23 12:17**	*Sample Weight:*	**1,0000**

Chromatogram

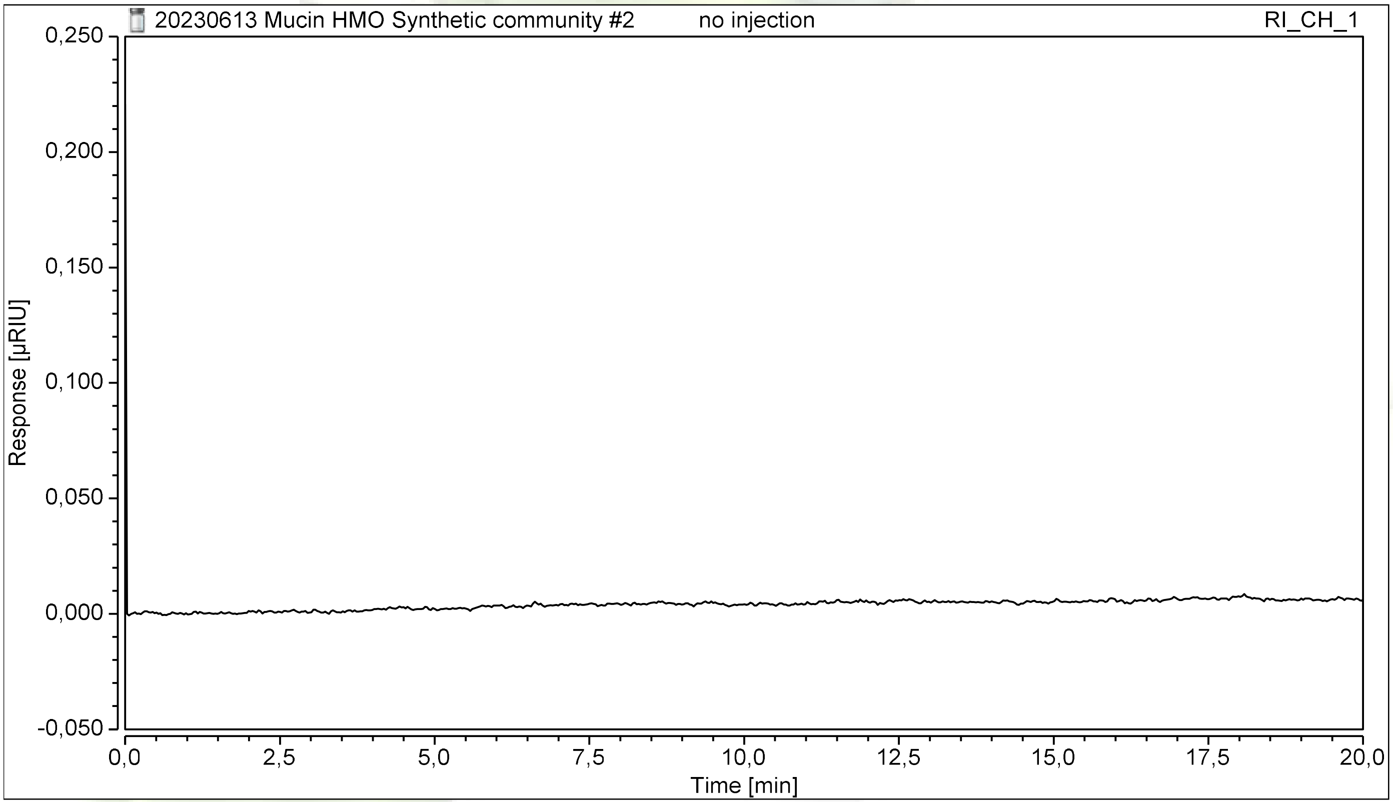

SST Results

No.	Name	Inj.Condition	Peak	Test Result	Injection

Number of executed test cases: **n.a.**   **Total Result:** **Passed**

Chromatogram

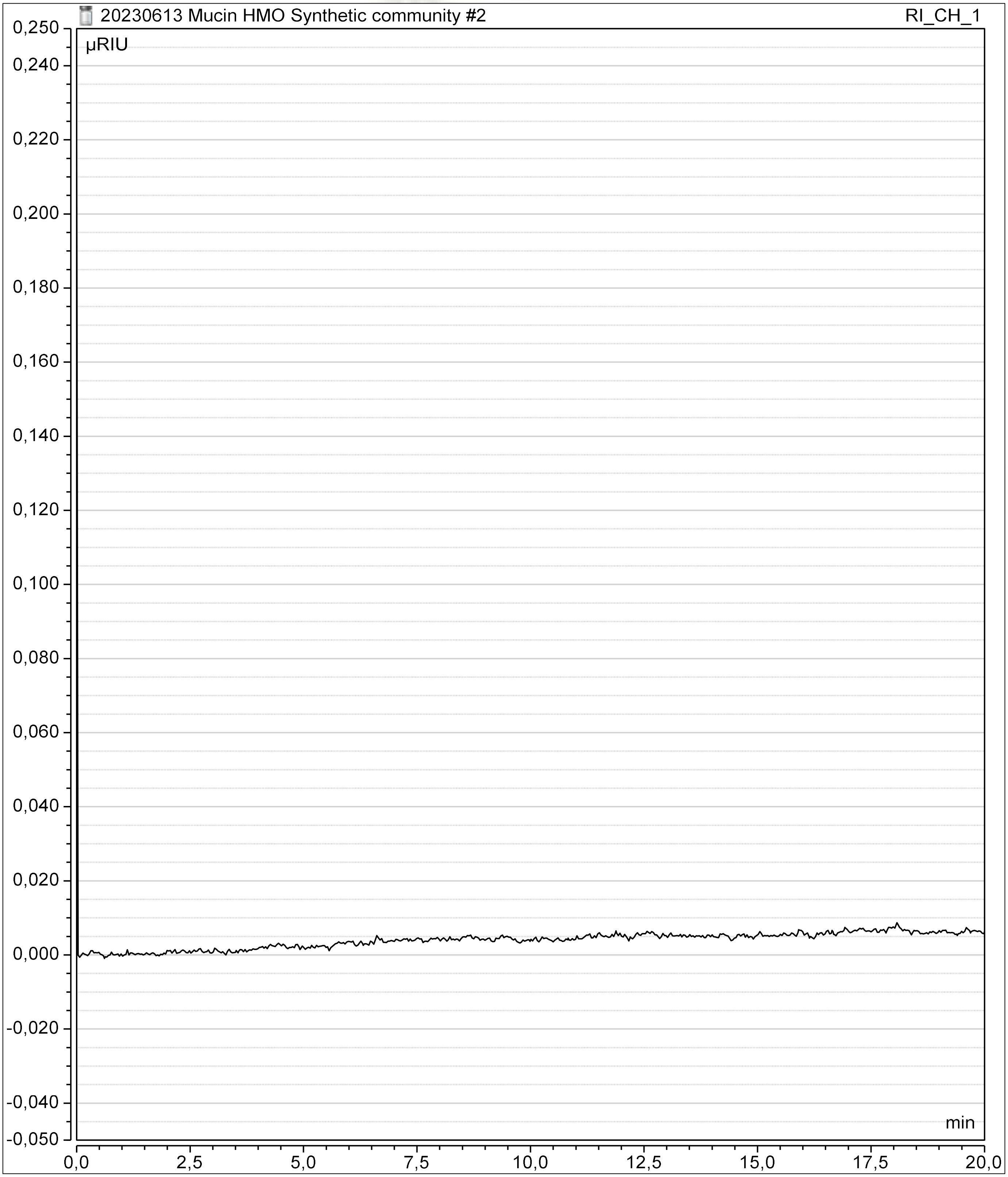

Chromatogram and Results

Injection Details

Injection Name:	**VFA 10**	*Run Time (min):*	**20,00**
Vial Number:	**3:A2**	*Injection Volume:*	**1,00**
Injection Type:	**Calibration Standard**	*Channel:*	**RI_CH_1**
Calibration Level:	**1**	*Wavelength:*	**n.a.**
Instrument Method:	**Default method LC2030C 45 gr 20 min**	*Bandwidth:*	**n.a.**
Processing Method:	**Processing Method LC2030 45 gr**	*Dilution Factor:*	**1,0000**
Injection Date/Time:	**13-jun-23 12:37**	*Sample Weight:*	**1,0000**

Chromatogram

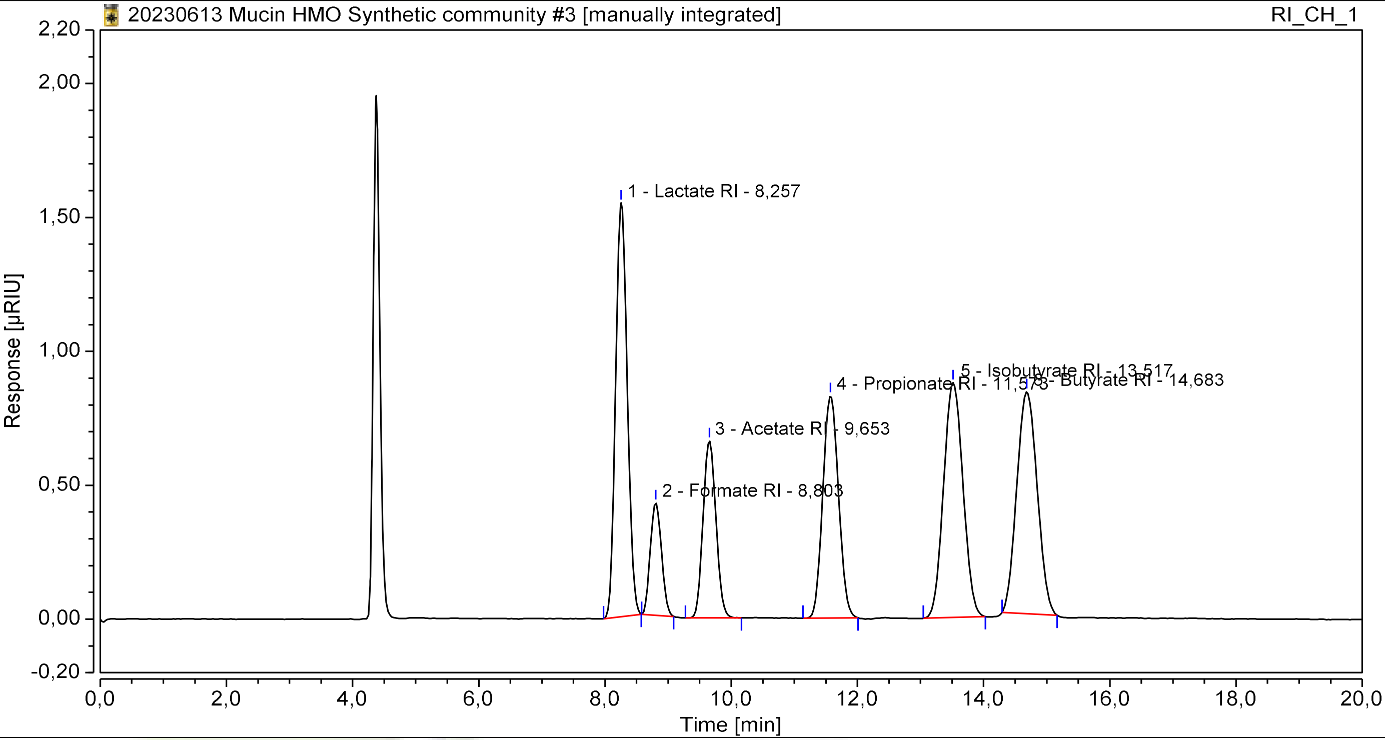

Integration Results

No.	Peak Name	Retention Time<br>min	Area<br>µRIU*min	Height<br>µRIU	Relative Area<br>%	Relative Height<br>%	Amount
n.a.	GlcNAc	n.a.	n.a.	n.a.	n.a.	n.a.	n.a.
n.a.	Citrate	n.a.	n.a.	n.a.	n.a.	n.a.	n.a.
n.a.	Glucose	n.a.	n.a.	n.a.	n.a.	n.a.	n.a.
n.a.	Galactose	n.a.	n.a.	n.a.	n.a.	n.a.	n.a.
n.a.	Fucose	n.a.	n.a.	n.a.	n.a.	n.a.	n.a.
n.a.	Succinate RI	n.a.	n.a.	n.a.	n.a.	n.a.	n.a.
1	Lactate RI	8,257	0,331	1,545	23,00	29,92	9,6087
n.a.	glycerol	n.a.	n.a.	n.a.	n.a.	n.a.	n.a.
2	Formate RI	8,803	0,087	0,420	6,04	8,14	9,1199
3	Acetate RI	9,653	0,158	0,662	10,95	12,83	9,6978
n.a.	1,2 PDO RI	n.a.	n.a.	n.a.	n.a.	n.a.	n.a.
n.a.	1,3-PDO	n.a.	n.a.	n.a.	n.a.	n.a.	n.a.
4	Propionate RI	11,573	0,240	0,831	16,67	16,08	9,6535
n.a.	1,3-PDO	n.a.	n.a.	n.a.	n.a.	n.a.	n.a.
n.a.	2-3 BDO	n.a.	n.a.	n.a.	n.a.	n.a.	n.a.
n.a.	Ethanol	n.a.	n.a.	n.a.	n.a.	n.a.	n.a.
5	Isobutyrate RI	13,517	0,313	0,877	21,75	16,99	9,7681
6	Butyrate RI	14,683	0,311	0,829	21,59	16,04	9,5323
**Total:**			**1,439**	**5,164**	**100,00**	**100,00**	

Peak Analysis

Injection Details

Injection Name:	**VFA 10**	*Run Time (min):*	**20,00**
Vial Number:	**3:A2**	*Injection Volume:*	**1,00**
Injection Type:	**Calibration Standard**	*Channel:*	**RI_CH_1**
Calibration Level:	**1**	*Wavelength:*	**n.a.**
Instrument Method:	**Default method LC2030C 45 gr 20 min**	*Bandwidth:*	**n.a.**
Processing Method:	**Processing Method LC2030 45 gr**	*Dilution Factor:*	**1,0000**
Injection Date/Time:	**13-jun-23 12:37**	*Sample Weight:*	**1,0000**

Chromatogram

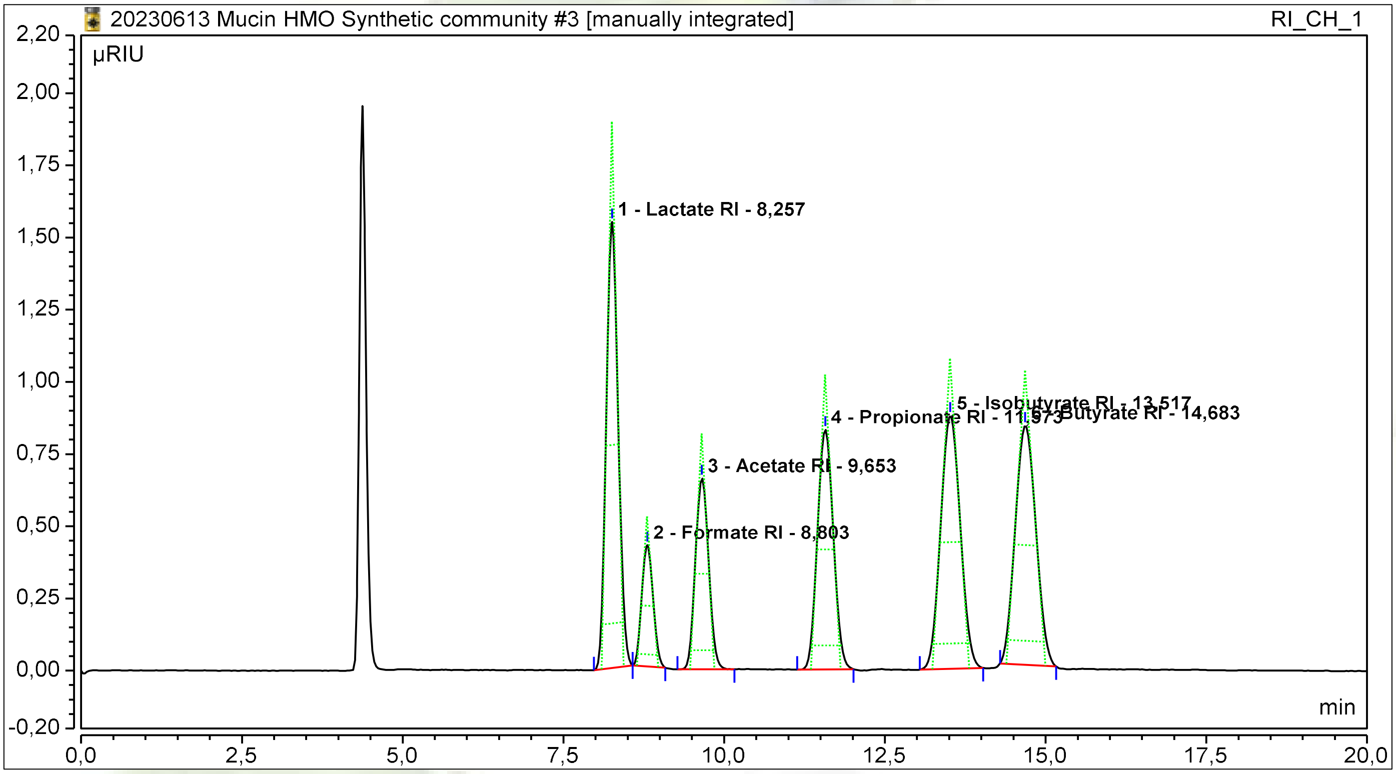

Peak Results

No.	Peak Name	Retention Time min	Width (50%) min	Type	Resolution (EP)	Asymmetry (EP)	Plates (EP)
n.a.	GlcNAc	n.a.	n.a.	n.a.	n.a.	n.a.	n.a.
n.a.	Citrate	n.a.	n.a.	n.a.	n.a.	n.a.	n.a.
n.a.	Glucose	n.a.	n.a.	n.a.	n.a.	n.a.	n.a.
n.a.	Galactose	n.a.	n.a.	n.a.	n.a.	n.a.	n.a.
n.a.	Fucose	n.a.	n.a.	n.a.	n.a.	n.a.	n.a.
n.a.	Succinate RI	n.a.	n.a.	n.a.	n.a.	n.a.	n.a.
1	Lactate RI	8,257	0,203	BMB	1,61	1,10	9170
n.a.	glycerol	n.a.	n.a.	n.a.	n.a.	n.a.	n.a.
2	Formate RI	8,803	0,198	BMB*	2,38	1,09	10996
3	Acetate RI	9,653	0,225	BMB	4,54	1,08	10236
n.a.	1,2 PDO RI	n.a.	n.a.	n.a.	n.a.	n.a.	n.a.
n.a.	1,3-PDO	n.a.	n.a.	n.a.	n.a.	n.a.	n.a.
4	Propionate RI	11,573	0,274	BMB*	3,74	1,07	9856
n.a.	1,3-PDO	n.a.	n.a.	n.a.	n.a.	n.a.	n.a.
n.a.	2-3 BDO	n.a.	n.a.	n.a.	n.a.	n.a.	n.a.
n.a.	Ethanol	n.a.	n.a.	n.a.	n.a.	n.a.	n.a.
5	Isobutyrate RI	13,517	0,339	BMB*	1,97	1,05	8825
6	Butyrate RI	14,683	0,360	BMB*	n.a.	1,07	9209

Chromeleon (c) Dionex
Version 7.2.10.25868

Chromatogram and SST Results

Injection Details

Injection Name:	**VFA 10**	*Run Time (min):*	**20,00**
Vial Number:	**3:A2**	*Injection Volume:*	**1,00**
Injection Type:	**Calibration Standard**	*Channel:*	**RI_CH_1**
Calibration Level:	**1**	*Wavelength:*	**n.a.**
Instrument Method:	**Default method LC2030C 45 gr 20 min**	*Bandwidth:*	**n.a.**
Processing Method:	**Processing Method LC2030 45 gr**	*Dilution Factor:*	**1,0000**
Injection Date/Time:	**13-jun-23 12:37**	*Sample Weight:*	**1,0000**

Chromatogram

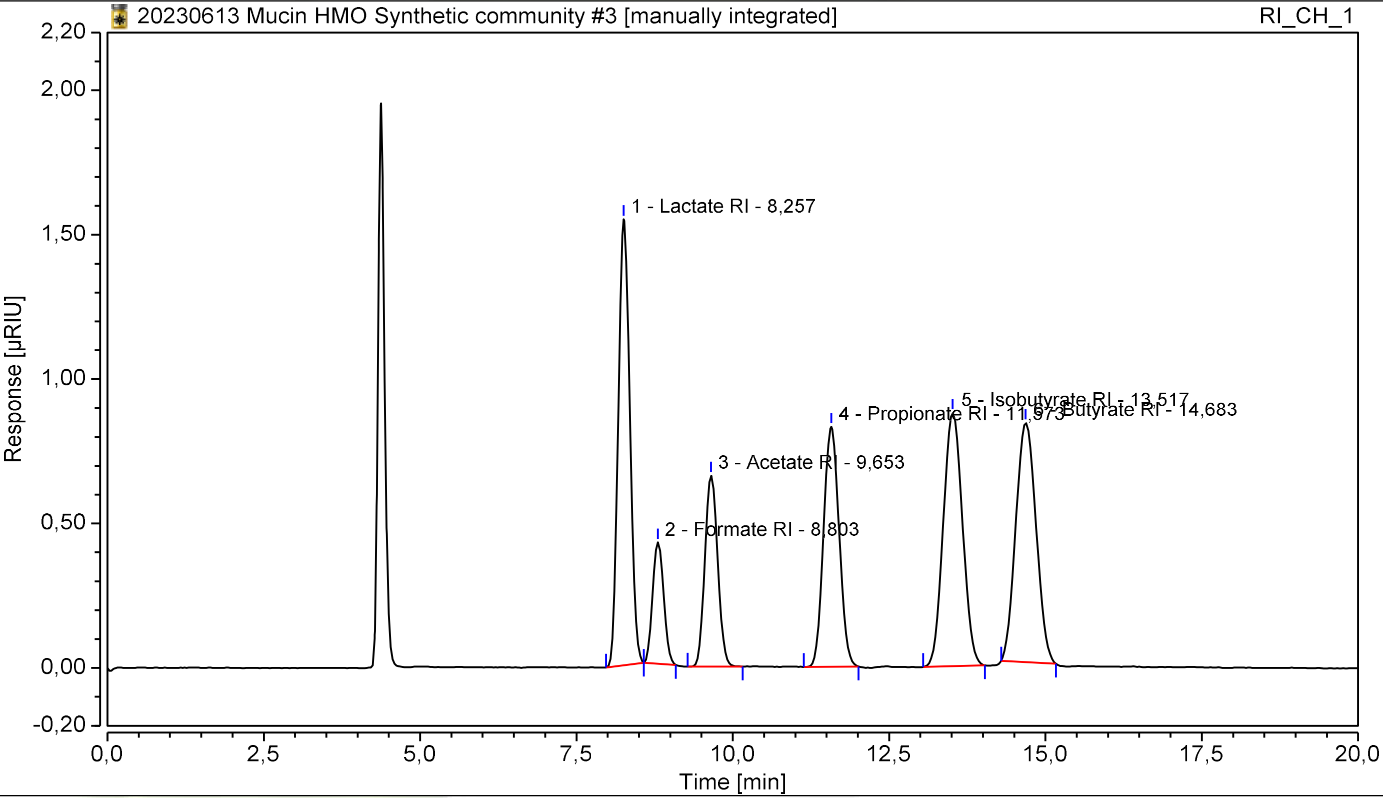

SST Results

No.	Name	Inj.Condition	Peak	Test Result	Injection
**Number of executed test cases:**	**n.a.**		**Total Result:**	**Passed**	

Chromatogram

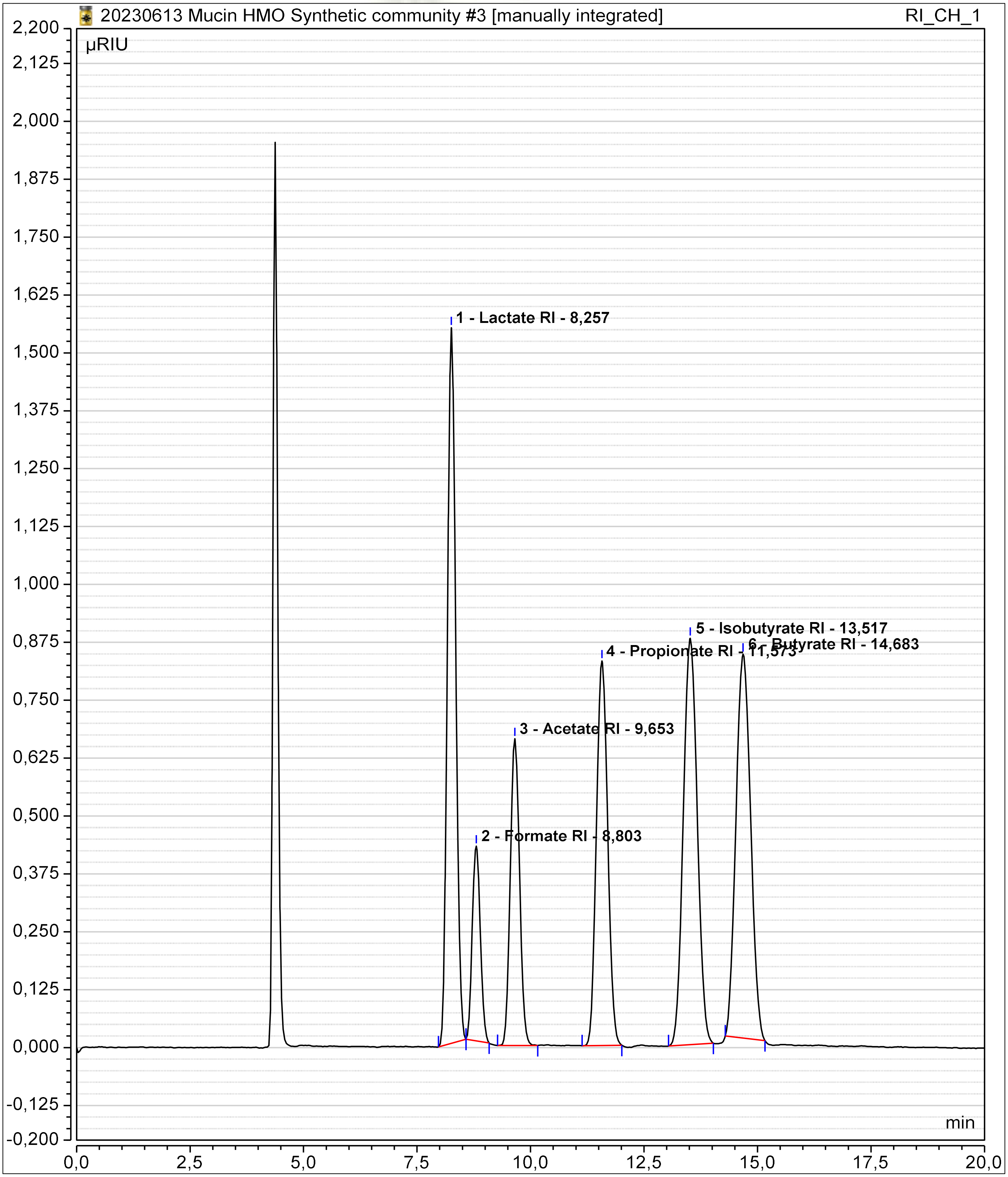

Chromeleon (c) Dionex
Version 7.2.10.25868

Chromatogram and Results

Injection Details

Injection Name:	**VFA 20**	*Run Time (min):*	**20,00**
Vial Number:	**3:A2**	*Injection Volume:*	**2,00**
Injection Type:	**Calibration Standard**	*Channel:*	**RI_CH_1**
Calibration Level:	**1**	*Wavelength:*	**n.a.**
Instrument Method:	**Default method LC2030C 45 gr 20 min**	*Bandwidth:*	**n.a.**
Processing Method:	**Processing Method LC2030 45 gr**	*Dilution Factor:*	**1,0000**
Injection Date/Time:	**13-jun-23 12:58**	*Sample Weight:*	**1,0000**

Chromatogram

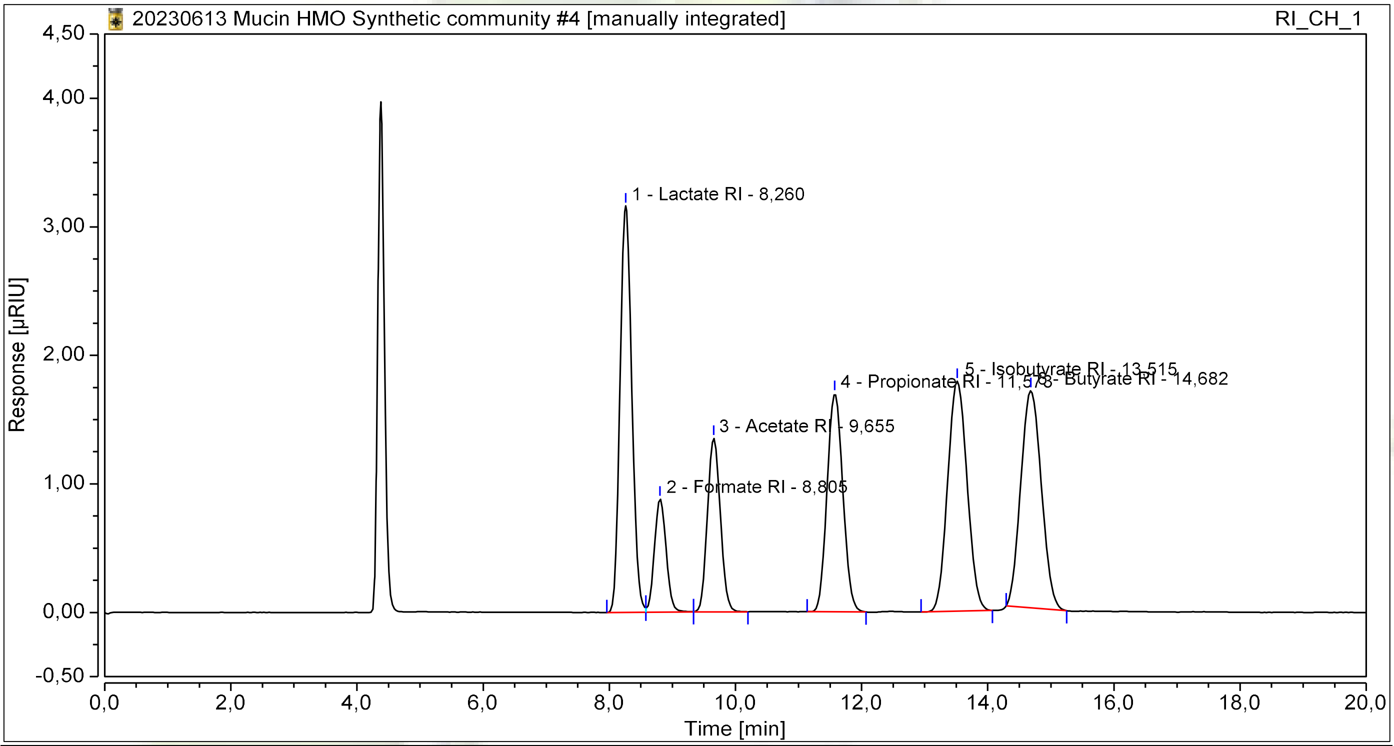

Integration Results

No.	Peak Name	Retention Time min	Area µRIU*min	Height µRIU	Relative Area %	Relative Height %	Amount
n.a.	GlcNAc	n.a.	n.a.	n.a.	n.a.	n.a.	n.a.
n.a.	Citrate	n.a.	n.a.	n.a.	n.a.	n.a.	n.a.
n.a.	Glucose	n.a.	n.a.	n.a.	n.a.	n.a.	n.a.
n.a.	Galactose	n.a.	n.a.	n.a.	n.a.	n.a.	n.a.
n.a.	Fucose	n.a.	n.a.	n.a.	n.a.	n.a.	n.a.
n.a.	Succinate RI	n.a.	n.a.	n.a.	n.a.	n.a.	n.a.
1	Lactate RI	8,260	0,684	3,162	23,03	29,91	19,8615
n.a.	glycerol	n.a.	n.a.	n.a.	n.a.	n.a.	n.a.
2	Formate RI	8,805	0,190	0,881	6,41	8,34	19,9907
3	Acetate RI	9,655	0,323	1,352	10,87	12,79	19,8709
n.a.	1,2 PDO RI	n.a.	n.a.	n.a.	n.a.	n.a.	n.a.
n.a.	1,3-PDO	n.a.	n.a.	n.a.	n.a.	n.a.	n.a.
4	Propionate RI	11,573	0,494	1,699	16,63	16,07	19,8687
n.a.	1,3-PDO	n.a.	n.a.	n.a.	n.a.	n.a.	n.a.
n.a.	2-3 BDO	n.a.	n.a.	n.a.	n.a.	n.a.	n.a.
n.a.	Ethanol	n.a.	n.a.	n.a.	n.a.	n.a.	n.a.
5	Isobutyrate RI	13,515	0,639	1,788	21,52	16,91	19,9478
6	Butyrate RI	14,682	0,640	1,690	21,54	15,99	19,6196
**Total:**			**2,970**	**10,572**	**100,00**	**100,00**	

Peak Analysis

Injection Details

Injection Name:	**VFA 20**	*Run Time (min):*	**20,00**
Vial Number:	**3:A2**	*Injection Volume:*	**2,00**
Injection Type:	**Calibration Standard**	*Channel:*	**RI_CH_1**
Calibration Level:	**1**	*Wavelength:*	**n.a.**
Instrument Method:	**Default method LC2030C 45 gr 20 min**	*Bandwidth:*	**n.a.**
Processing Method:	**Processing Method LC2030 45 gr**	*Dilution Factor:*	**1,0000**
Injection Date/Time:	**13-jun-23 12:58**	*Sample Weight:*	**1,0000**

Chromatogram

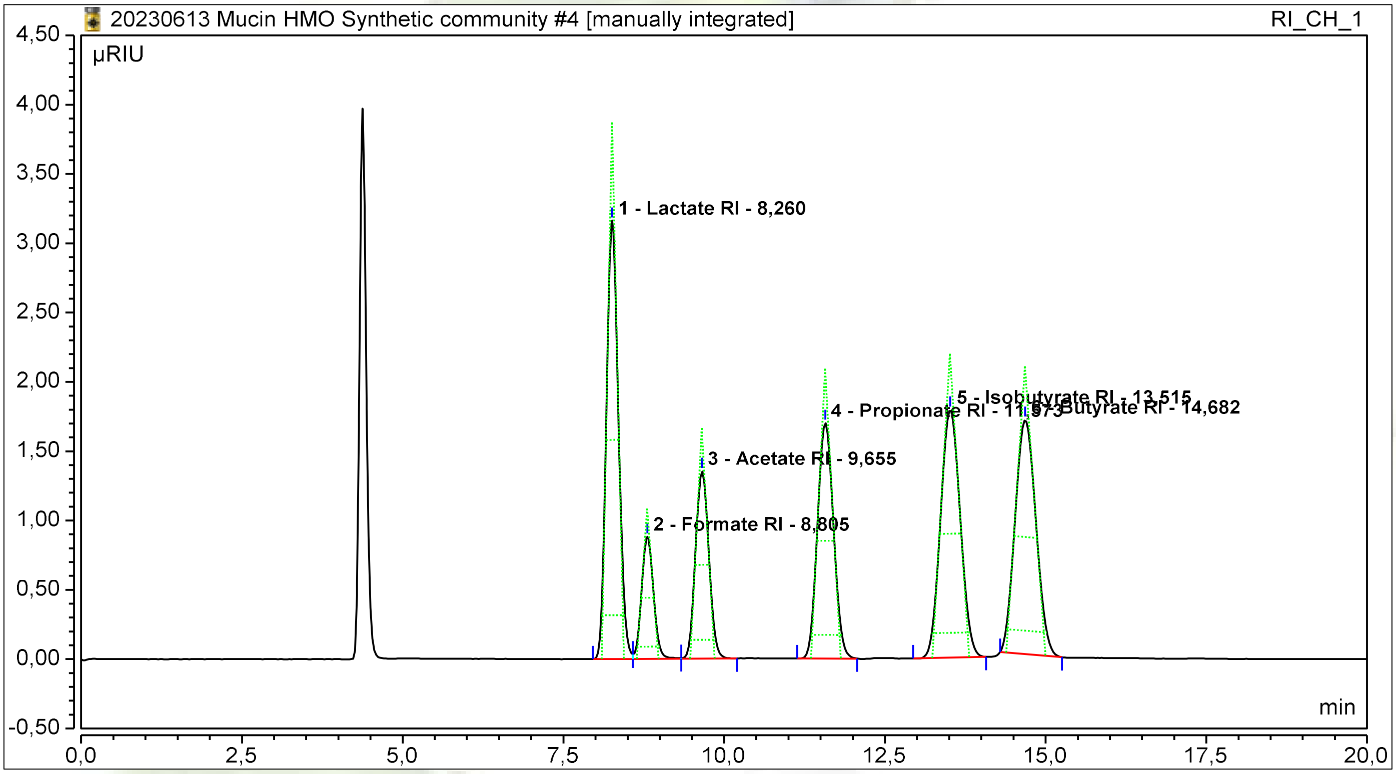

Peak Results

No.	Peak Name	Retention Time min	Width (50%) min	Type	Resolution (EP)	Asymmetry (EP)	Plates (EP)
n.a.	GlcNAc	n.a.	n.a.	n.a.	n.a.	n.a.	n.a.
n.a.	Citrate	n.a.	n.a.	n.a.	n.a.	n.a.	n.a.
n.a.	Glucose	n.a.	n.a.	n.a.	n.a.	n.a.	n.a.
n.a.	Galactose	n.a.	n.a.	n.a.	n.a.	n.a.	n.a.
n.a.	Fucose	n.a.	n.a.	n.a.	n.a.	n.a.	n.a.
n.a.	Succinate RI	n.a.	n.a.	n.a.	n.a.	n.a.	n.a.
1	Lactate RI	8,260	0,204	BM	1,59	1,11	9101
n.a.	glycerol	n.a.	n.a.	n.a.	n.a.	n.a.	n.a.
2	Formate RI	8,805	0,201	M	2,35	1,08	10604
3	Acetate RI	9,655	0,225	MB	4,52	1,09	10183
n.a.	1,2 PDO RI	n.a.	n.a.	n.a.	n.a.	n.a.	n.a.
n.a.	1,3-PDO	n.a.	n.a.	n.a.	n.a.	n.a.	n.a.
4	Propionate RI	11,573	0,275	BMB*	3,73	1,08	9786
n.a.	1,3-PDO	n.a.	n.a.	n.a.	n.a.	n.a.	n.a.
n.a.	2-3 BDO	n.a.	n.a.	n.a.	n.a.	n.a.	n.a.
n.a.	Ethanol	n.a.	n.a.	n.a.	n.a.	n.a.	n.a.
5	Isobutyrate RI	13,515	0,339	BMB*	1,96	1,06	8782
6	Butyrate RI	14,682	0,362	BMB*	n.a.	1,08	9121

Chromeleon (c) Dionex
Version 7.2.10.25868

Chromatogram and SST Results

Injection Details

Injection Name:	**VFA 20**	*Run Time (min):*	**20,00**
Vial Number:	**3:A2**	*Injection Volume:*	**2,00**
Injection Type:	**Calibration Standard**	*Channel:*	**RI_CH_1**
Calibration Level:	**1**	*Wavelength:*	**n.a.**
Instrument Method:	**Default method LC2030C 45 gr 20 min**	*Bandwidth:*	**n.a.**
Processing Method:	**Processing Method LC2030 45 gr**	*Dilution Factor:*	**1,0000**
Injection Date/Time:	**13-jun-23 12:58**	*Sample Weight:*	**1,0000**

Chromatogram

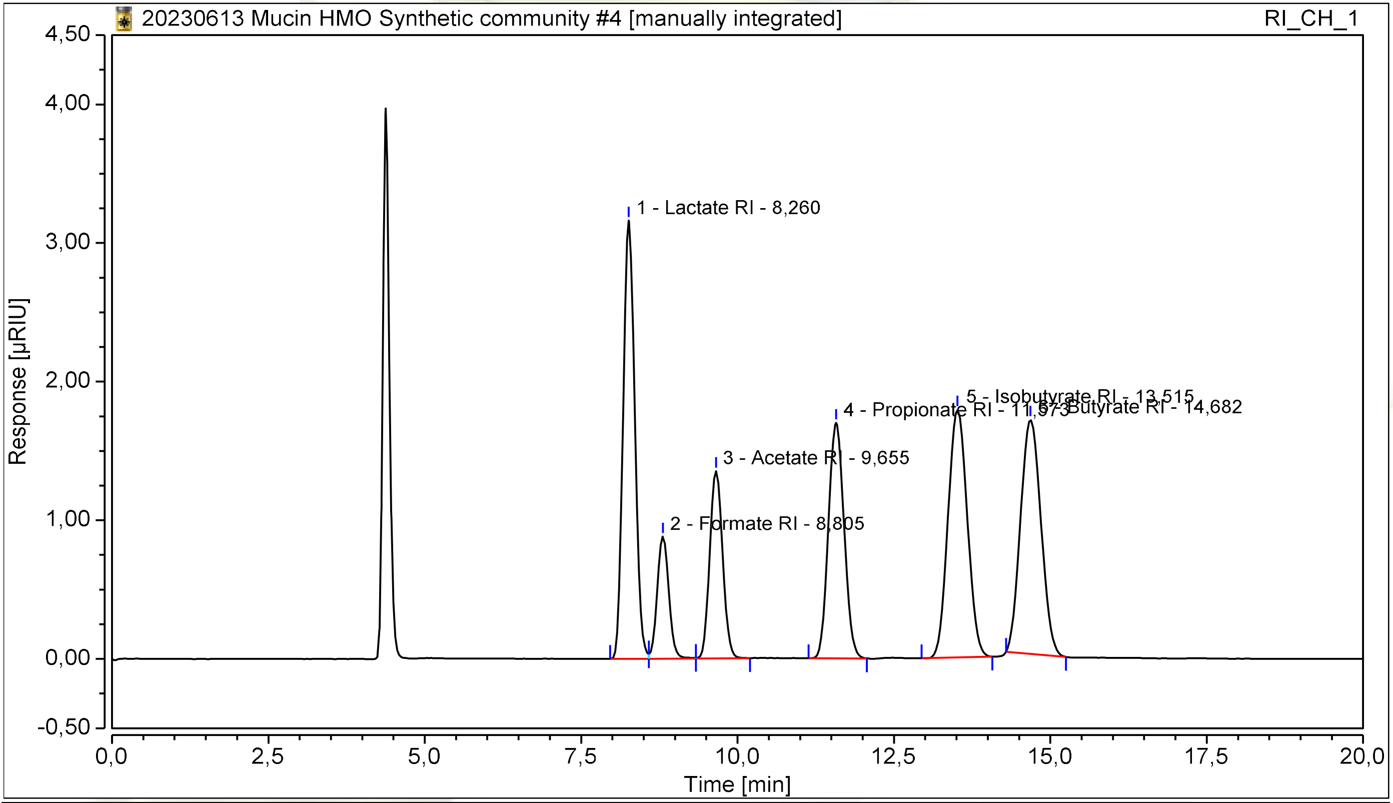

SST Results

No.	Name	Inj.Condition	Peak	Test Result	Injection

Number of executed test cases: **n.a.** **Total Result:** **Passed**

Chromeleon (c) Dionex
Version 7.2.10.25868

Chromatogram

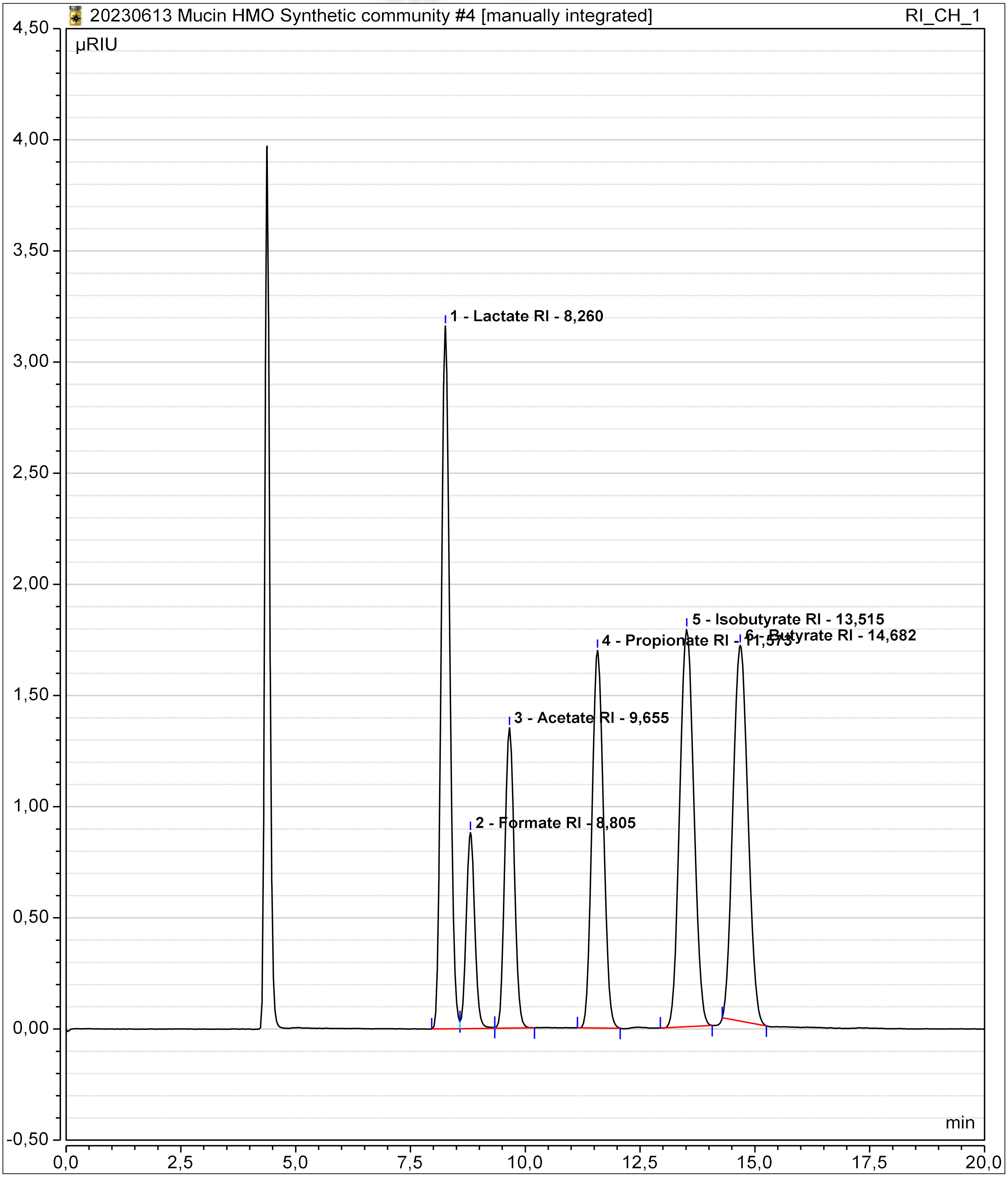

Chromatogram and Results

Injection Details

Injection Name:	**VFA 30**	*Run Time (min):*	**20,00**
Vial Number:	**3:A2**	*Injection Volume:*	**3,00**
Injection Type:	**Calibration Standard**	*Channel:*	**RI_CH_1**
Calibration Level:	**1**	*Wavelength:*	**n.a.**
Instrument Method:	**Default method LC2030C 45 gr 20 min**	*Bandwidth:*	**n.a.**
Processing Method:	**Processing Method LC2030 45 gr**	*Dilution Factor:*	**1,0000**
Injection Date/Time:	**13-jun-23 13:18**	*Sample Weight:*	**1,0000**

Chromatogram

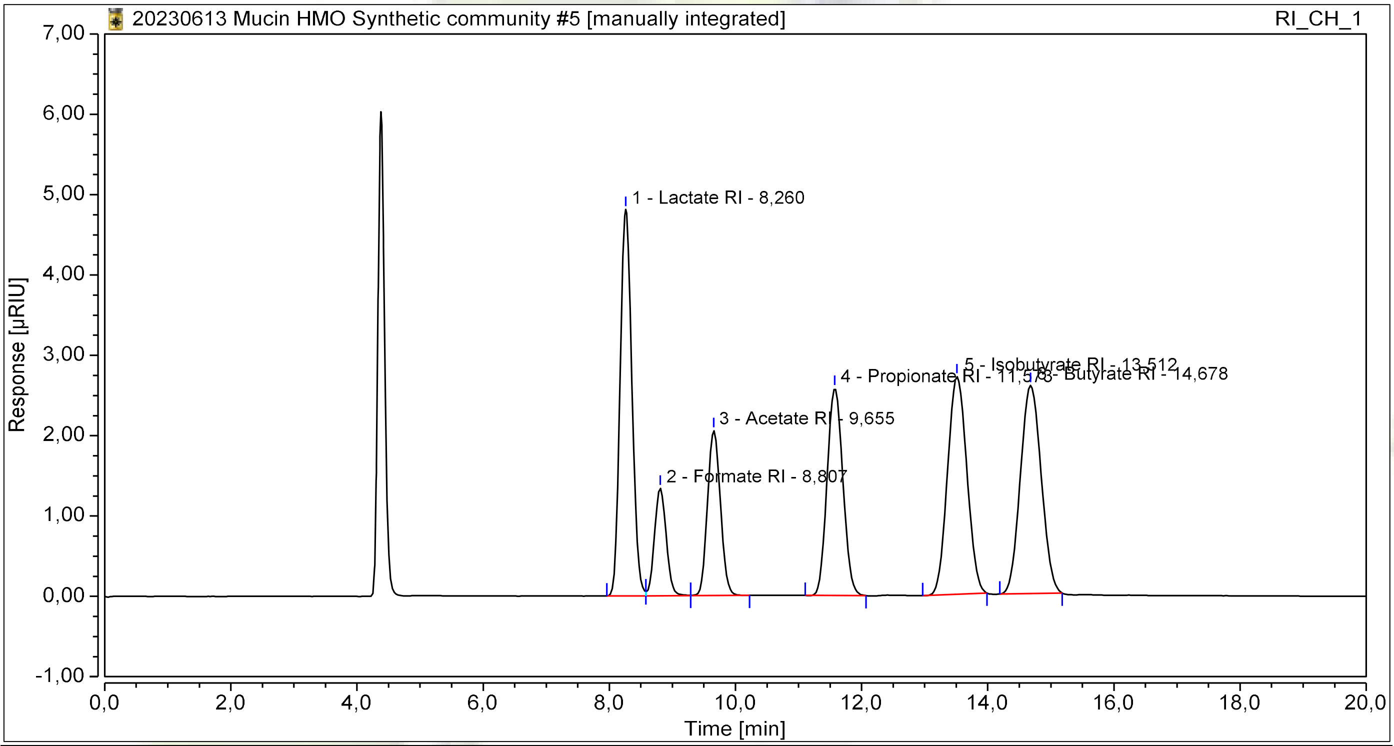

Integration Results

No.	Peak Name	Retention Time min	Area µRIU*min	Height µRIU	Relative Area %	Relative Height %	Amount
n.a.	GlcNAc	n.a.	n.a.	n.a.	n.a.	n.a.	n.a.
n.a.	Citrate	n.a.	n.a.	n.a.	n.a.	n.a.	n.a.
n.a.	Glucose	n.a.	n.a.	n.a.	n.a.	n.a.	n.a.
n.a.	Galactose	n.a.	n.a.	n.a.	n.a.	n.a.	n.a.
n.a.	Fucose	n.a.	n.a.	n.a.	n.a.	n.a.	n.a.
n.a.	Succinate RI	n.a.	n.a.	n.a.	n.a.	n.a.	n.a.
1	Lactate RI	8,260	1,041	4,810	22,99	29,91	30,2228
n.a.	glycerol	n.a.	n.a.	n.a.	n.a.	n.a.	n.a.
2	Formate RI	8,807	0,289	1,339	6,38	8,32	30,2996
3	Acetate RI	9,655	0,490	2,054	10,83	12,77	30,1868
n.a.	1,2 PDO RI	n.a.	n.a.	n.a.	n.a.	n.a.	n.a.
n.a.	1,3-PDO	n.a.	n.a.	n.a.	n.a.	n.a.	n.a.
4	Propionate RI	11,573	0,751	2,581	16,58	16,05	30,2030
n.a.	1,3-PDO	n.a.	n.a.	n.a.	n.a.	n.a.	n.a.
n.a.	2-3 BDO	n.a.	n.a.	n.a.	n.a.	n.a.	n.a.
n.a.	Ethanol	n.a.	n.a.	n.a.	n.a.	n.a.	n.a.
5	Isobutyrate RI	13,512	0,965	2,710	21,32	16,85	30,1121
6	Butyrate RI	14,678	0,991	2,590	21,90	16,10	30,4095
**Total:**			**4,527**	**16,083**	**100,00**	**100,00**	

Peak Analysis

Injection Details

Injection Name:	**VFA 30**	*Run Time (min):*	**20,00**
Vial Number:	**3:A2**	*Injection Volume:*	**3,00**
Injection Type:	**Calibration Standard**	*Channel:*	**RI_CH_1**
Calibration Level:	**1**	*Wavelength:*	**n.a.**
Instrument Method:	**Default method LC2030C 45 gr 20 min**	*Bandwidth:*	**n.a.**
Processing Method:	**Processing Method LC2030 45 gr**	*Dilution Factor:*	**1,0000**
Injection Date/Time:	**13-jun-23 13:18**	*Sample Weight:*	**1,0000**

Chromatogram

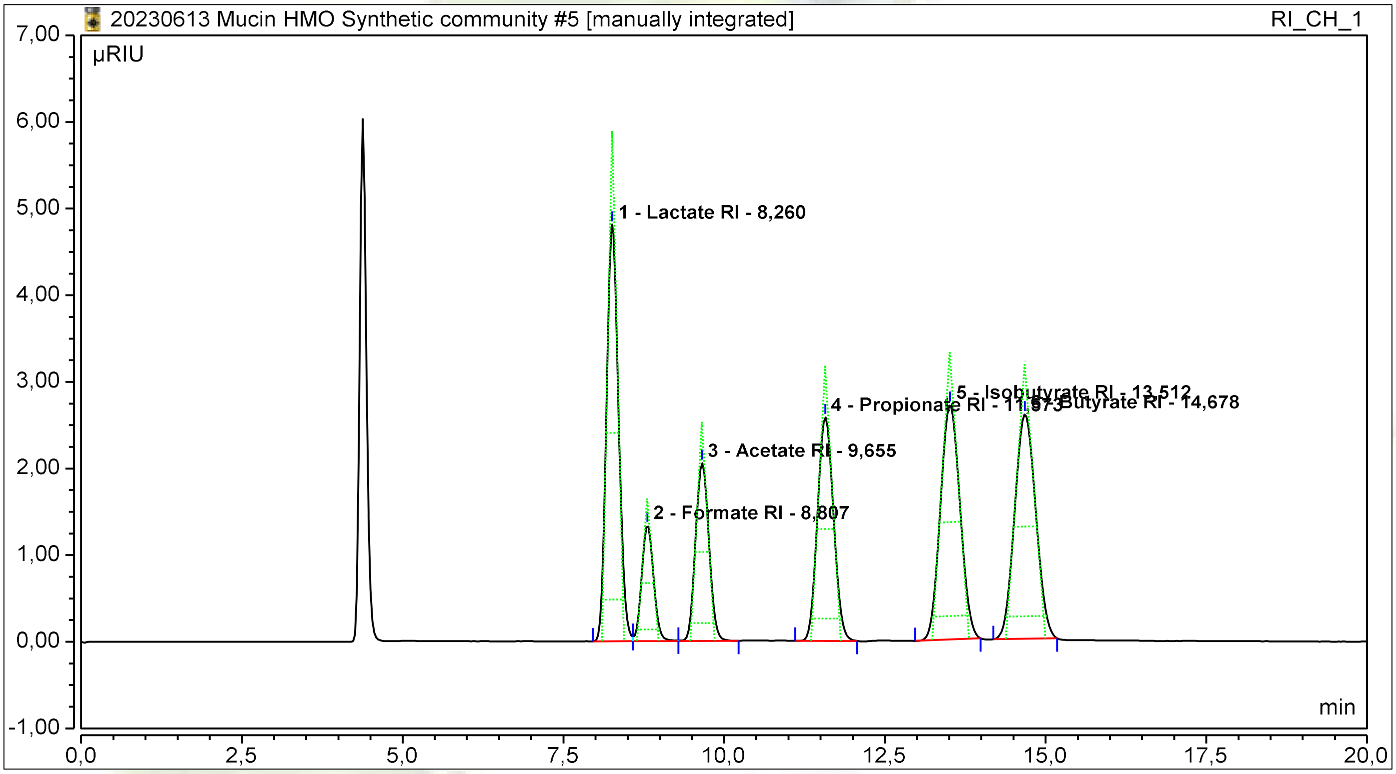

Peak Results

No.	Peak Name	Retention Time min	Width (50%) min	Type	Resolution (EP)	Asymmetry (EP)	Plates (EP)
n.a.	GlcNAc	n.a.	n.a.	n.a.	n.a.	n.a.	n.a.
n.a.	Citrate	n.a.	n.a.	n.a.	n.a.	n.a.	n.a.
n.a.	Glucose	n.a.	n.a.	n.a.	n.a.	n.a.	n.a.
n.a.	Galactose	n.a.	n.a.	n.a.	n.a.	n.a.	n.a.
n.a.	Fucose	n.a.	n.a.	n.a.	n.a.	n.a.	n.a.
n.a.	Succinate RI	n.a.	n.a.	n.a.	n.a.	n.a.	n.a.
1	Lactate RI	8,260	0,204	BM	1,59	1,11	9100
n.a.	glycerol	n.a.	n.a.	n.a.	n.a.	n.a.	n.a.
2	Formate RI	8,807	0,201	M	2,35	1,08	10612
3	Acetate RI	9,655	0,225	MB	4,52	1,09	10180
n.a.	1,2 PDO RI	n.a.	n.a.	n.a.	n.a.	n.a.	n.a.
n.a.	1,3-PDO	n.a.	n.a.	n.a.	n.a.	n.a.	n.a.
4	Propionate RI	11,573	0,276	BMB*	3,72	1,09	9769
n.a.	1,3-PDO	n.a.	n.a.	n.a.	n.a.	n.a.	n.a.
n.a.	2-3 BDO	n.a.	n.a.	n.a.	n.a.	n.a.	n.a.
n.a.	Ethanol	n.a.	n.a.	n.a.	n.a.	n.a.	n.a.
5	Isobutyrate RI	13,512	0,339	BMB*	1,96	1,06	8799
6	Butyrate RI	14,678	0,365	BMB*	n.a.	1,06	8972

Chromatogram and SST Results

Injection Details

Injection Name:	**VFA 30**	*Run Time (min):*	**20,00**
Vial Number:	**3:A2**	*Injection Volume:*	**3,00**
Injection Type:	**Calibration Standard**	*Channel:*	**RI_CH_1**
Calibration Level:	**1**	*Wavelength:*	**n.a.**
Instrument Method:	**Default method LC2030C 45 gr 20 min**	*Bandwidth:*	**n.a.**
Processing Method:	**Processing Method LC2030 45 gr**	*Dilution Factor:*	**1,0000**
Injection Date/Time:	**13-jun-23 13:18**	*Sample Weight:*	**1,0000**

Chromatogram

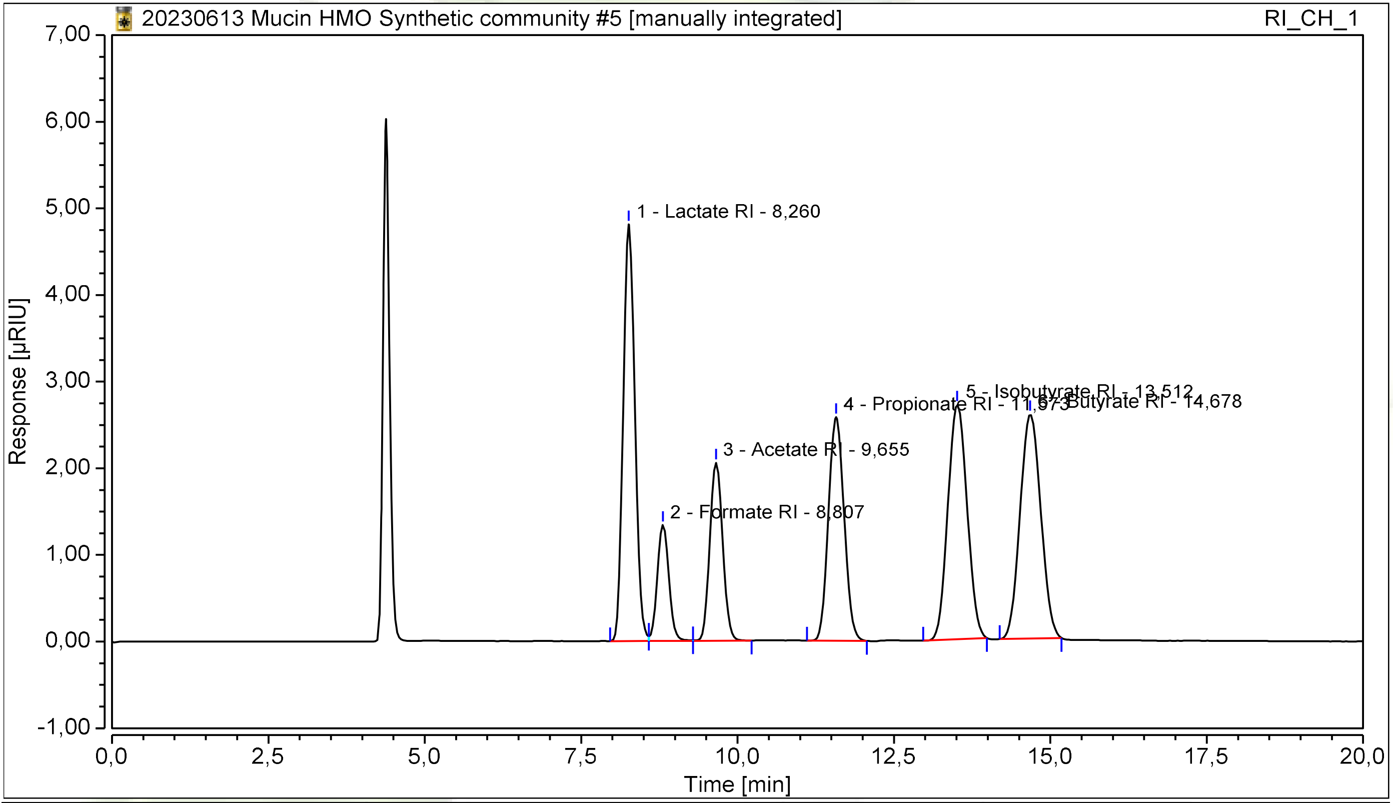

SST Results

No.	Name	Inj.Condition	Peak	Test Result	Injection
**Number of executed test cases:**	**n.a.**		**Total Result:**	**Passed**	

Chromatogram

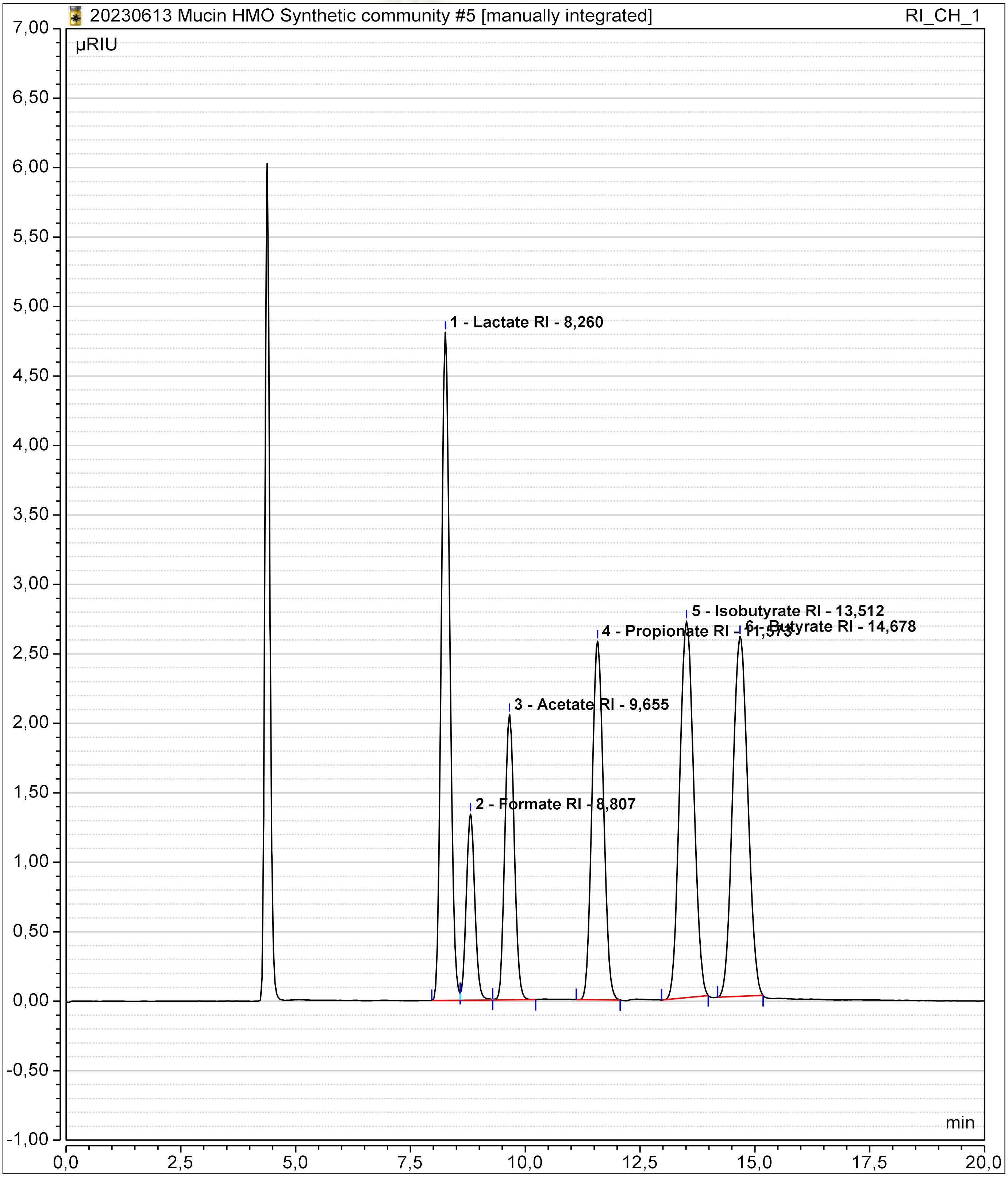

Chromatogram and Results

Injection Details

Injection Name:	**1,2-Prop & 1- propane 10**	*Run Time (min):*	**20,00**
Vial Number:	**3:A3**	*Injection Volume:*	**1,00**
Injection Type:	**Calibration Standard**	*Channel:*	**RI_CH_1**
Calibration Level:	**1**	*Wavelength:*	**n.a.**
Instrument Method:	**Default method LC2030C 45 gr 20 min**	*Bandwidth:*	**n.a.**
Processing Method:	**Processing Method LC2030 45 gr**	*Dilution Factor:*	**1,0000**
Injection Date/Time:	**13-jun-23 13:38**	*Sample Weight:*	**1,0000**

Chromatogram

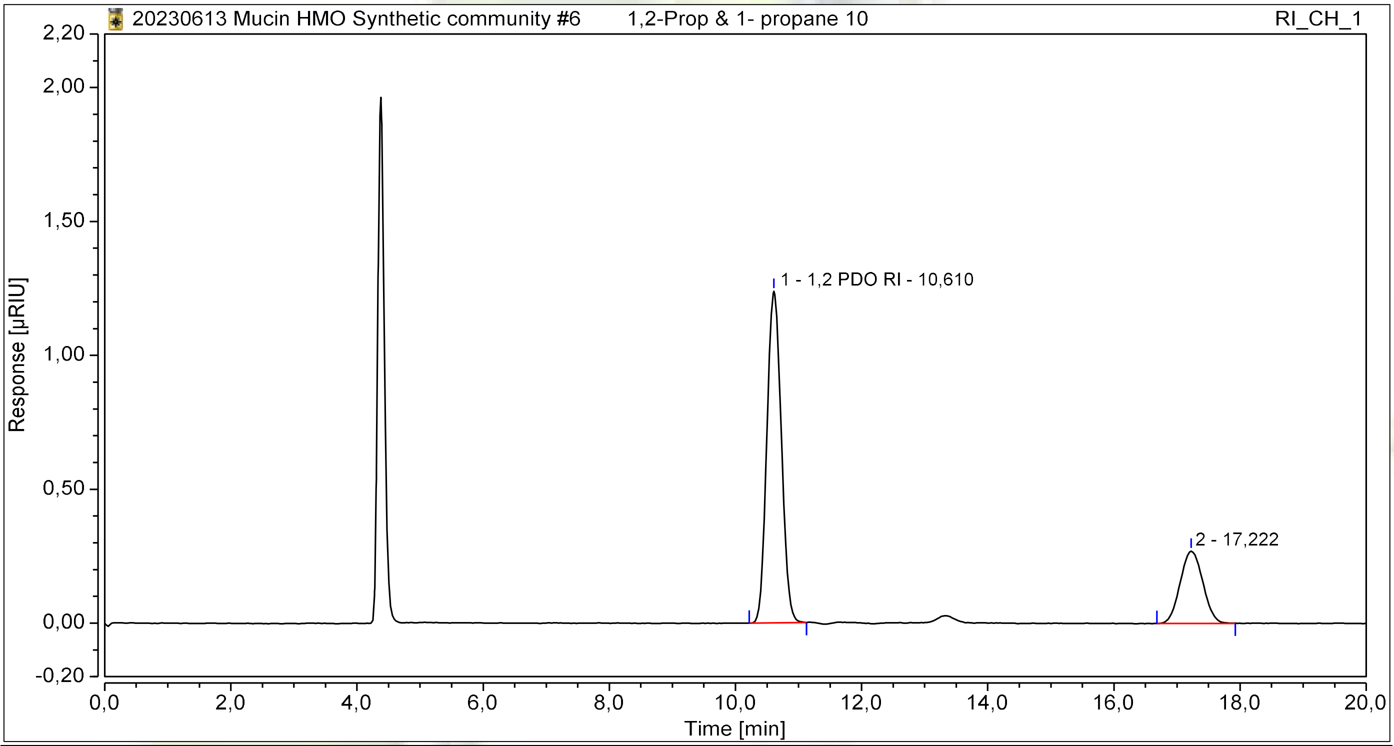

Integration Results

No.	Peak Name	Retention Time min	Area µRIU*min	Height µRIU	Relative Area %	Relative Height %	Amount
n.a.	GlcNAc	n.a.	n.a.	n.a.	n.a.	n.a.	n.a.
n.a.	Citrate	n.a.	n.a.	n.a.	n.a.	n.a.	n.a.
n.a.	Glucose	n.a.	n.a.	n.a.	n.a.	n.a.	n.a.
n.a.	Galactose	n.a.	n.a.	n.a.	n.a.	n.a.	n.a.
n.a.	Fucose	n.a.	n.a.	n.a.	n.a.	n.a.	n.a.
n.a.	Succinate RI	n.a.	n.a.	n.a.	n.a.	n.a.	n.a.
n.a.	Lactate RI	n.a.	n.a.	n.a.	n.a.	n.a.	n.a.
n.a.	glycerol	n.a.	n.a.	n.a.	n.a.	n.a.	n.a.
n.a.	Formate RI	n.a.	n.a.	n.a.	n.a.	n.a.	n.a.
n.a.	Acetate RI	n.a.	n.a.	n.a.	n.a.	n.a.	n.a.
1	1,2 PDO RI	10,610	0,329	1,238	74,54	82,10	9,7818
n.a.	1,3-PDO	n.a.	n.a.	n.a.	n.a.	n.a.	n.a.
n.a.	Propionate RI	n.a.	n.a.	n.a.	n.a.	n.a.	n.a.
n.a.	1,3-PDO	n.a.	n.a.	n.a.	n.a.	n.a.	n.a.
n.a.	2-3 BDO	n.a.	n.a.	n.a.	n.a.	n.a.	n.a.
n.a.	Ethanol	n.a.	n.a.	n.a.	n.a.	n.a.	n.a.
n.a.	Isobutyrate RI	n.a.	n.a.	n.a.	n.a.	n.a.	n.a.
n.a.	Butyrate RI	n.a.	n.a.	n.a.	n.a.	n.a.	n.a.
2		17,222	0,112	0,270	25,46	17,90	n.a.
**Total:**			**0,441**	**1,508**	**100,00**	**100,00**	

Peak Analysis

Injection Details

Injection Name:	**1,2-Prop & 1- propane 10**	*Run Time (min):*	**20,00**
Vial Number:	**3:A3**	*Injection Volume:*	**1,00**
Injection Type:	**Calibration Standard**	*Channel:*	**RI_CH_1**
Calibration Level:	**1**	*Wavelength:*	**n.a.**
Instrument Method:	**Default method LC2030C 45 gr 20 min**	*Bandwidth:*	**n.a.**
Processing Method:	**Processing Method LC2030 45 gr**	*Dilution Factor:*	**1,0000**
Injection Date/Time:	**13-jun-23 13:38**	*Sample Weight:*	**1,0000**

Chromatogram

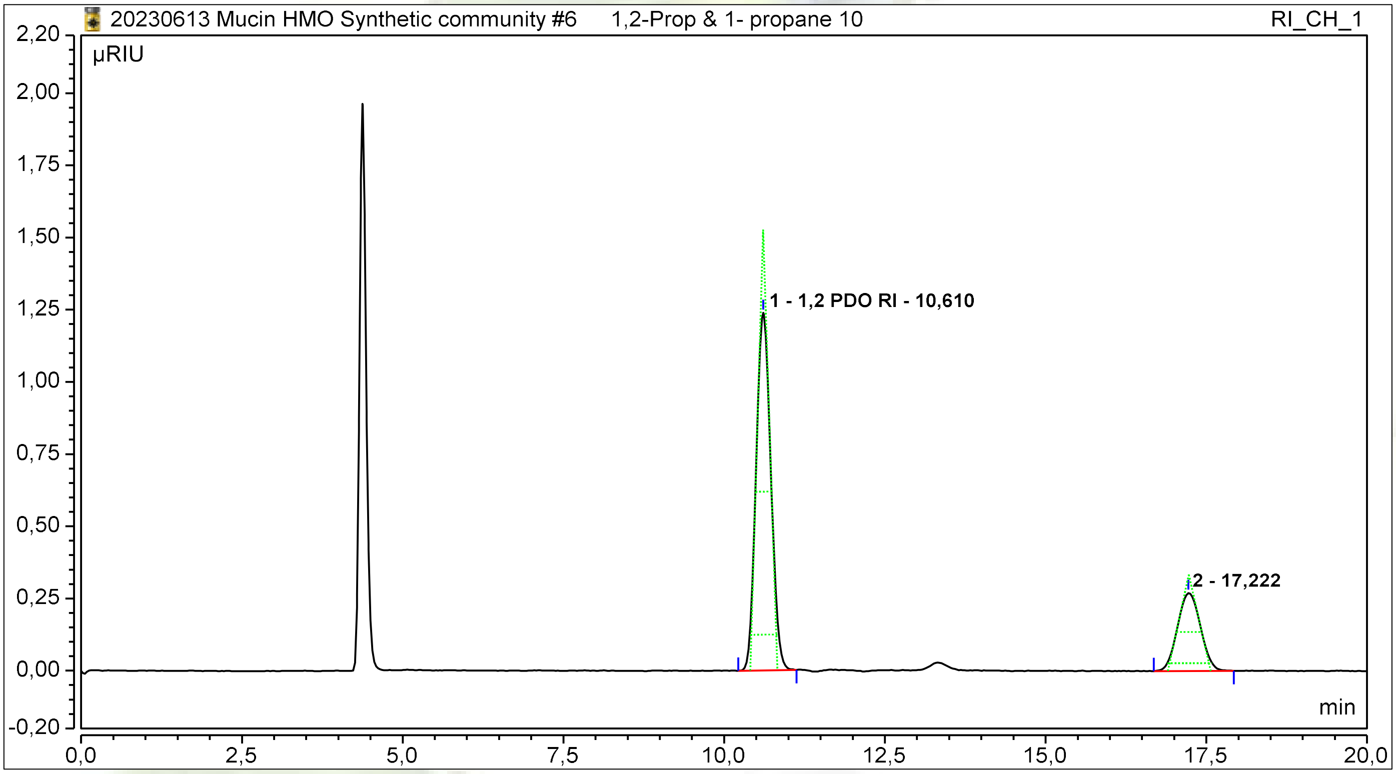

Peak Results

No.	Peak Name	Retention Time min	Width (50%) min	Type	Resolution (EP)	Asymmetry (EP)	Plates (EP)
n.a.	GlcNAc	n.a.	n.a.	n.a.	n.a.	n.a.	n.a.
n.a.	Citrate	n.a.	n.a.	n.a.	n.a.	n.a.	n.a.
n.a.	Glucose	n.a.	n.a.	n.a.	n.a.	n.a.	n.a.
n.a.	Galactose	n.a.	n.a.	n.a.	n.a.	n.a.	n.a.
n.a.	Fucose	n.a.	n.a.	n.a.	n.a.	n.a.	n.a.
n.a.	Succinate RI	n.a.	n.a.	n.a.	n.a.	n.a.	n.a.
n.a.	Lactate RI	n.a.	n.a.	n.a.	n.a.	n.a.	n.a.
n.a.	glycerol	n.a.	n.a.	n.a.	n.a.	n.a.	n.a.
n.a.	Formate RI	n.a.	n.a.	n.a.	n.a.	n.a.	n.a.
n.a.	Acetate RI	n.a.	n.a.	n.a.	n.a.	n.a.	n.a.
1	1,2 PDO RI	10,610	0,251	BMB	12,04	1,08	9874
n.a.	1,3-PDO	n.a.	n.a.	n.a.	n.a.	n.a.	n.a.
n.a.	Propionate RI	n.a.	n.a.	n.a.	n.a.	n.a.	n.a.
n.a.	1,3-PDO	n.a.	n.a.	n.a.	n.a.	n.a.	n.a.
n.a.	2-3 BDO	n.a.	n.a.	n.a.	n.a.	n.a.	n.a.
n.a.	Ethanol	n.a.	n.a.	n.a.	n.a.	n.a.	n.a.
n.a.	Isobutyrate RI	n.a.	n.a.	n.a.	n.a.	n.a.	n.a.
n.a.	Butyrate RI	n.a.	n.a.	n.a.	n.a.	n.a.	n.a.

2		17,222	0,397	BMB	n.a.	1,06	10446

Chromatogram and SST Results

Injection Details

Injection Name:	**1,2-Prop & 1- propane 10**	*Run Time (min):*	**20,00**
Vial Number:	**3:A3**	*Injection Volume:*	**1,00**
Injection Type:	**Calibration Standard**	*Channel:*	**RI_CH_1**
Calibration Level:	**1**	*Wavelength:*	**n.a.**
Instrument Method:	**Default method LC2030C 45 gr 20 min**	*Bandwidth:*	**n.a.**
Processing Method:	**Processing Method LC2030 45 gr**	*Dilution Factor:*	**1,0000**
Injection Date/Time:	**13-jun-23 13:38**	*Sample Weight:*	**1,0000**

Chromatogram

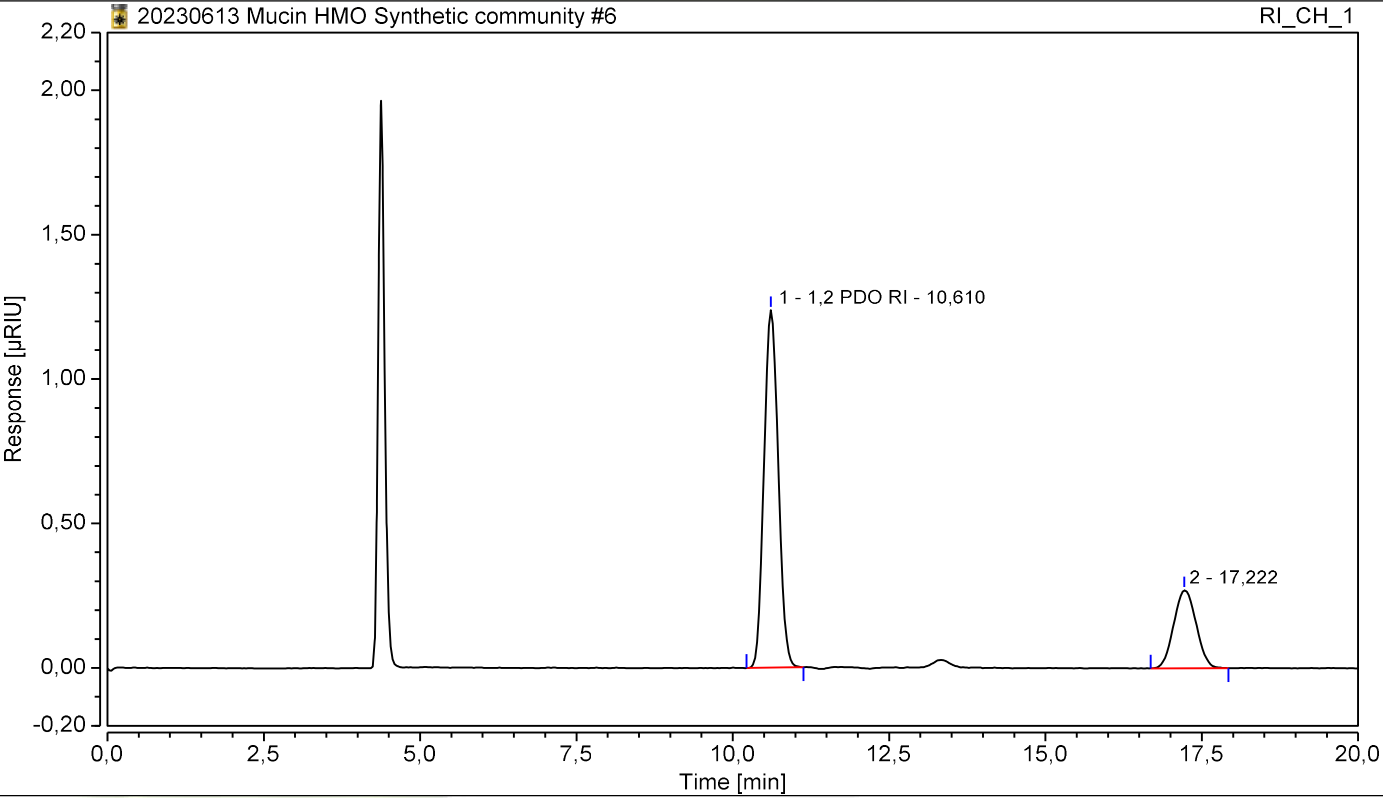

SST Results

No.	Name	Inj.Condition	Peak	Test Result	Injection

Number of executed test cases: **n.a.** **Total Result:** **Passed**

Chromatogram

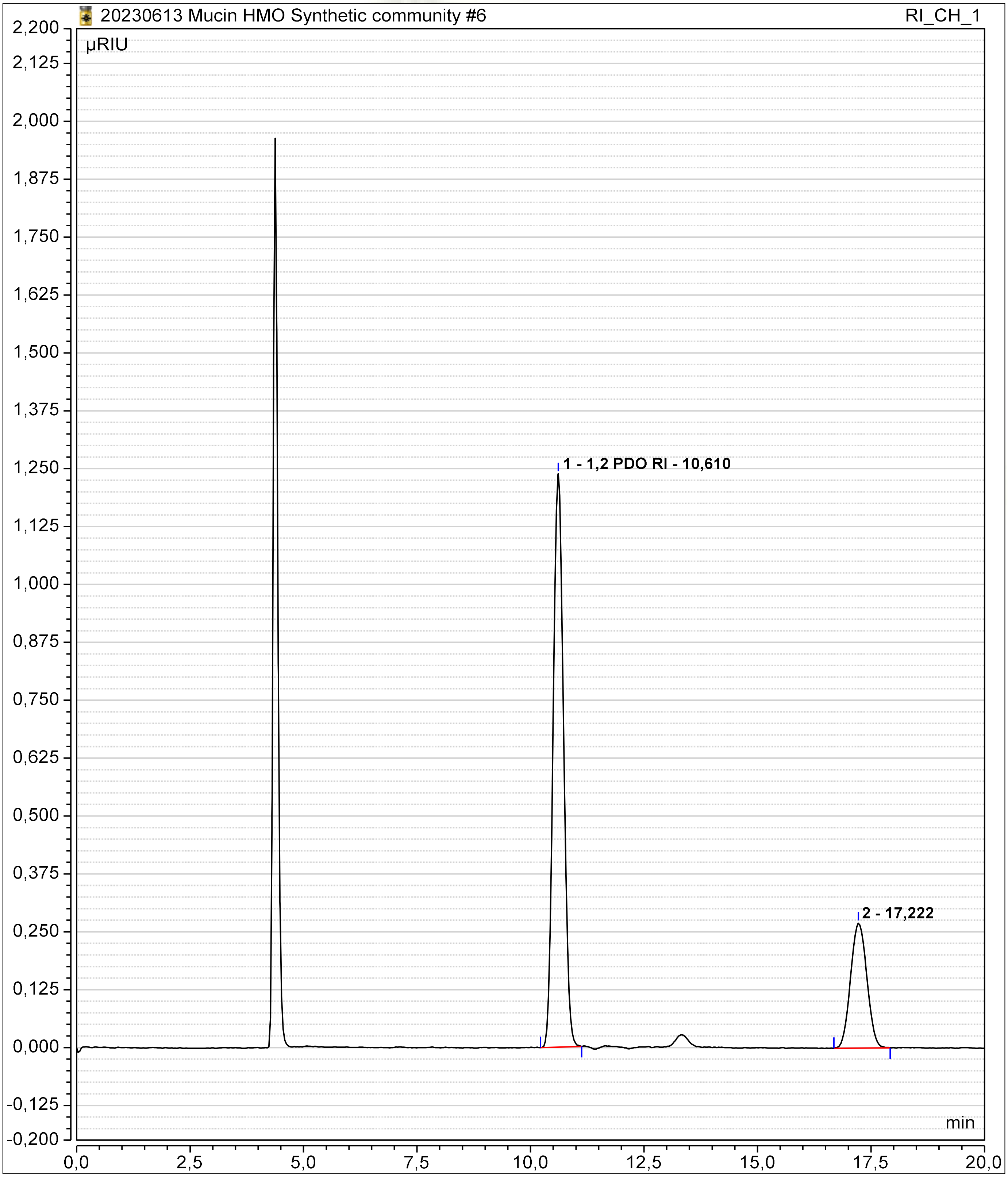

Chromatogram and Results

Injection Details

Injection Name:	**1,2-Prop & 1- propane 20**	*Run Time (min):*	**20,00**
Vial Number:	**3:A3**	*Injection Volume:*	**2,00**
Injection Type:	**Calibration Standard**	*Channel:*	**RI_CH_1**
Calibration Level:	**1**	*Wavelength:*	**n.a.**
Instrument Method:	**Default method LC2030C 45 gr 20 min**	*Bandwidth:*	**n.a.**
Processing Method:	**Processing Method LC2030 45 gr**	*Dilution Factor:*	**1,0000**
Injection Date/Time:	**13-jun-23 13:59**	*Sample Weight:*	**1,0000**

Chromatogram

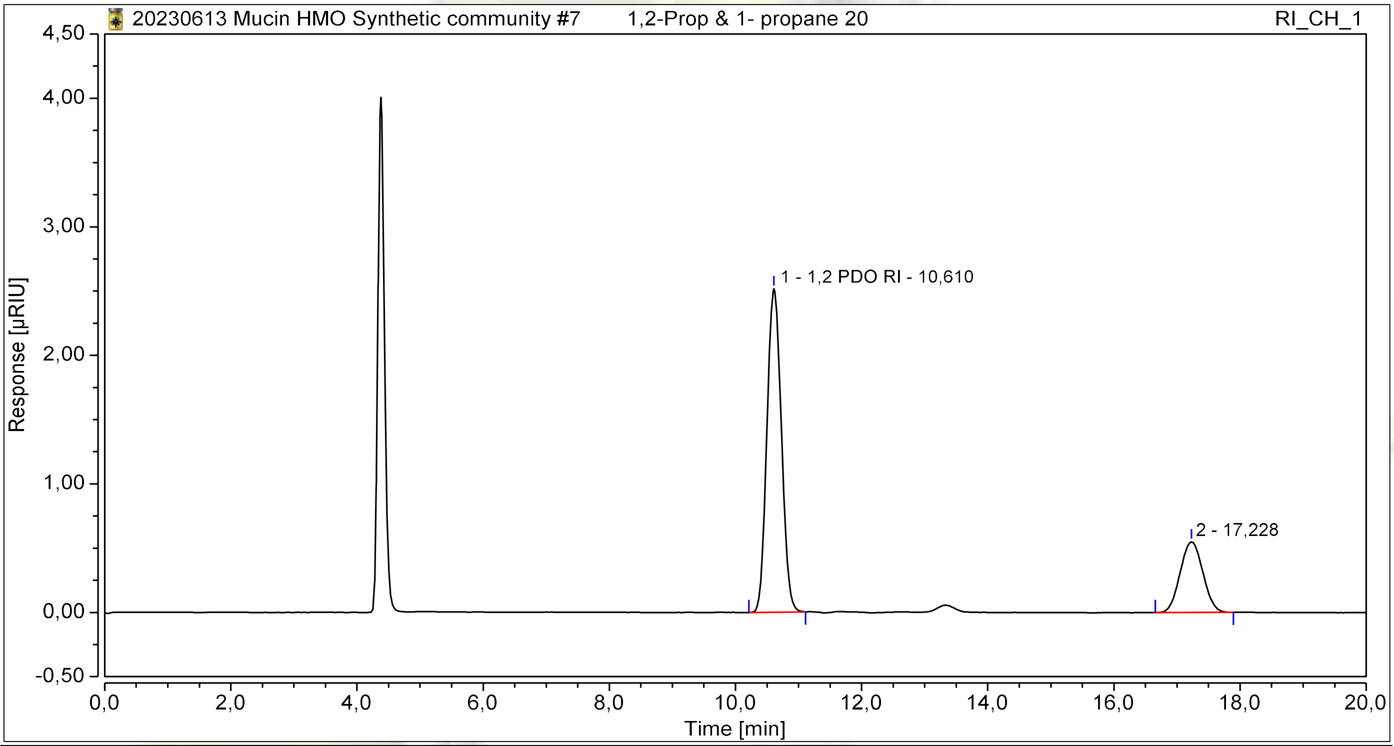

Integration Results

No.	Peak Name	Retention Time min	Area µRIU*min	Height µRIU	Relative Area %	Relative Height %	Amount
n.a.	GlcNAc	n.a.	n.a.	n.a.	n.a.	n.a.	n.a.
n.a.	Citrate	n.a.	n.a.	n.a.	n.a.	n.a.	n.a.
n.a.	Glucose	n.a.	n.a.	n.a.	n.a.	n.a.	n.a.
n.a.	Galactose	n.a.	n.a.	n.a.	n.a.	n.a.	n.a.
n.a.	Fucose	n.a.	n.a.	n.a.	n.a.	n.a.	n.a.
n.a.	Succinate RI	n.a.	n.a.	n.a.	n.a.	n.a.	n.a.
n.a.	Lactate RI	n.a.	n.a.	n.a.	n.a.	n.a.	n.a.
n.a.	glycerol	n.a.	n.a.	n.a.	n.a.	n.a.	n.a.
n.a.	Formate RI	n.a.	n.a.	n.a.	n.a.	n.a.	n.a.
n.a.	Acetate RI	n.a.	n.a.	n.a.	n.a.	n.a.	n.a.
1	1,2 PDO RI	10,610	0,669	2,518	74,45	82,07	19,8797
n.a.	1,3-PDO	n.a.	n.a.	n.a.	n.a.	n.a.	n.a.
n.a.	Propionate RI	n.a.	n.a.	n.a.	n.a.	n.a.	n.a.
n.a.	1,3-PDO	n.a.	n.a.	n.a.	n.a.	n.a.	n.a.
n.a.	2-3 BDO	n.a.	n.a.	n.a.	n.a.	n.a.	n.a.
n.a.	Ethanol	n.a.	n.a.	n.a.	n.a.	n.a.	n.a.
n.a.	Isobutyrate RI	n.a.	n.a.	n.a.	n.a.	n.a.	n.a.
n.a.	Butyrate RI	n.a.	n.a.	n.a.	n.a.	n.a.	n.a.
2		17,228	0,229	0,550	25,55	17,93	n.a.
**Total:**			**0,898**	**3,068**	**100,00**	**100,00**	

Peak Analysis

Injection Details

Injection Name:	**1,2-Prop & 1- propane 20**	*Run Time (min):*	**20,00**
Vial Number:	**3:A3**	*Injection Volume:*	**2,00**
Injection Type:	**Calibration Standard**	*Channel:*	**RI_CH_1**
Calibration Level:	**1**	*Wavelength:*	**n.a.**
Instrument Method:	**Default method LC2030C 45 gr 20 min**	*Bandwidth:*	**n.a.**
Processing Method:	**Processing Method LC2030 45 gr**	*Dilution Factor:*	**1,0000**
Injection Date/Time:	**13-jun-23 13:59**	*Sample Weight:*	**1,0000**

Chromatogram

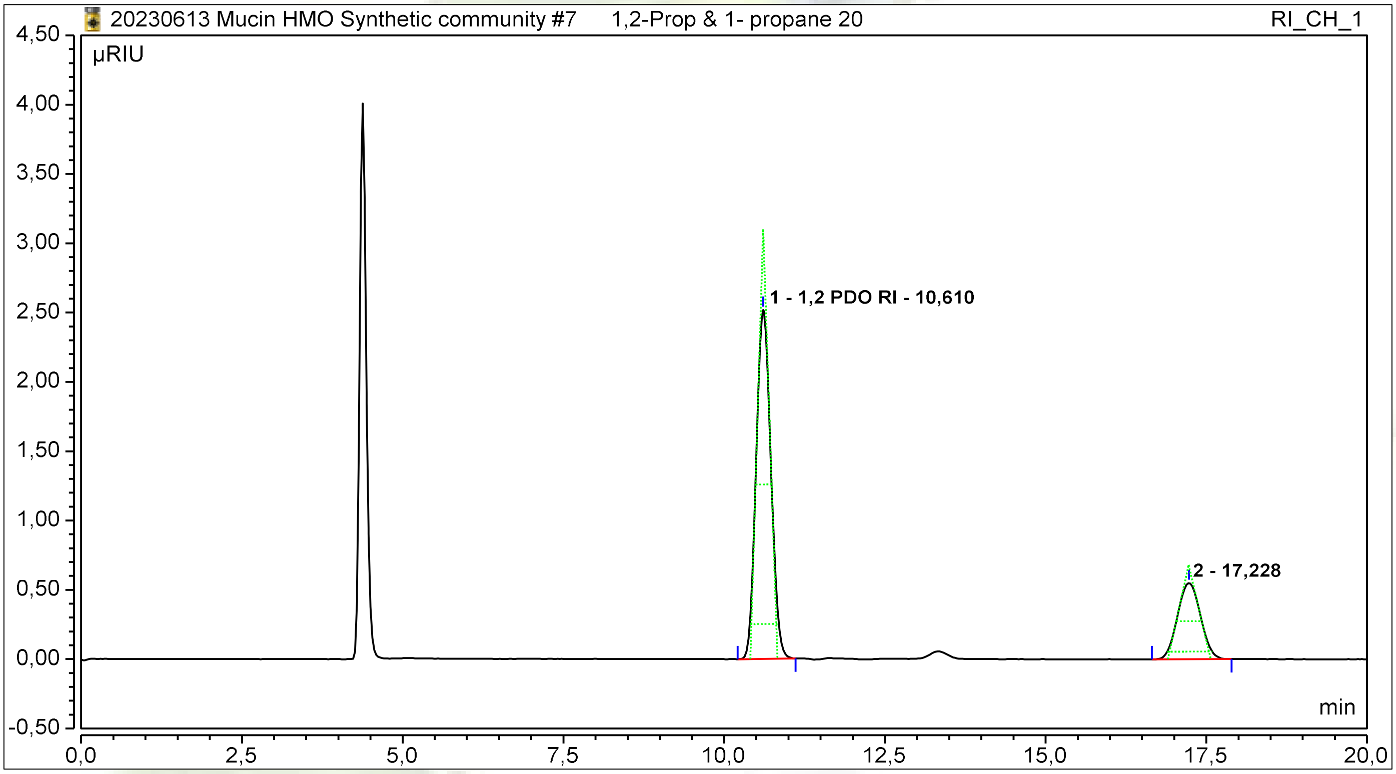

Peak Results

No.	Peak Name	Retention Time min	Width (50%) min	Type	Resolution (EP)	Asymmetry (EP)	Plates (EP)
n.a.	GlcNAc	n.a.	n.a.	n.a.	n.a.	n.a.	n.a.
n.a.	Citrate	n.a.	n.a.	n.a.	n.a.	n.a.	n.a.
n.a.	Glucose	n.a.	n.a.	n.a.	n.a.	n.a.	n.a.
n.a.	Galactose	n.a.	n.a.	n.a.	n.a.	n.a.	n.a.
n.a.	Fucose	n.a.	n.a.	n.a.	n.a.	n.a.	n.a.
n.a.	Succinate RI	n.a.	n.a.	n.a.	n.a.	n.a.	n.a.
n.a.	Lactate RI	n.a.	n.a.	n.a.	n.a.	n.a.	n.a.
n.a.	glycerol	n.a.	n.a.	n.a.	n.a.	n.a.	n.a.
n.a.	Formate RI	n.a.	n.a.	n.a.	n.a.	n.a.	n.a.
n.a.	Acetate RI	n.a.	n.a.	n.a.	n.a.	n.a.	n.a.
1	1,2 PDO RI	10,610	0,251	BMB	12,04	1,08	9879
n.a.	1,3-PDO	n.a.	n.a.	n.a.	n.a.	n.a.	n.a.
n.a.	Propionate RI	n.a.	n.a.	n.a.	n.a.	n.a.	n.a.
n.a.	1,3-PDO	n.a.	n.a.	n.a.	n.a.	n.a.	n.a.
n.a.	2-3 BDO	n.a.	n.a.	n.a.	n.a.	n.a.	n.a.
n.a.	Ethanol	n.a.	n.a.	n.a.	n.a.	n.a.	n.a.
n.a.	Isobutyrate RI	n.a.	n.a.	n.a.	n.a.	n.a.	n.a.
n.a.	Butyrate RI	n.a.	n.a.	n.a.	n.a.	n.a.	n.a.

2		17,228	0,397	BMB	n.a.	1,04	10425

Chromatogram and SST Results

Injection Details

Injection Name:	**1,2-Prop & 1- propane 20**	*Run Time (min):*	**20,00**
Vial Number:	**3:A3**	*Injection Volume:*	**2,00**
Injection Type:	**Calibration Standard**	*Channel:*	**RI_CH_1**
Calibration Level:	**1**	*Wavelength:*	**n.a.**
Instrument Method:	**Default method LC2030C 45 gr 20 min**	*Bandwidth:*	**n.a.**
Processing Method:	**Processing Method LC2030 45 gr**	*Dilution Factor:*	**1,0000**
Injection Date/Time:	**13-jun-23 13:59**	*Sample Weight:*	**1,0000**

Chromatogram

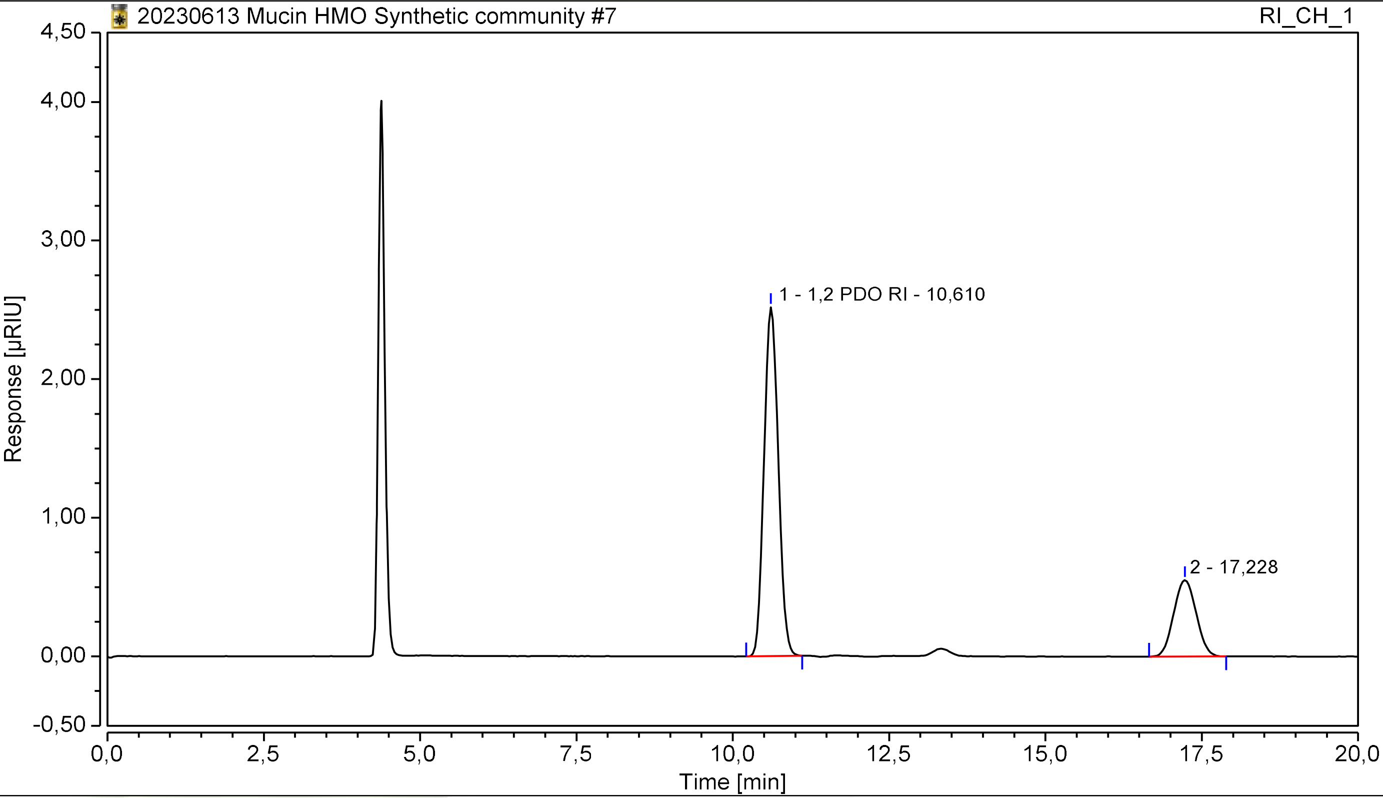

SST Results

No.	Name	Inj.Condition	Peak	Test Result	Injection

Number of executed test cases: n.a. **Total Result: Passed**

Chromeleon (c) Dionex
Version 7.2.10.25868

Chromatogram

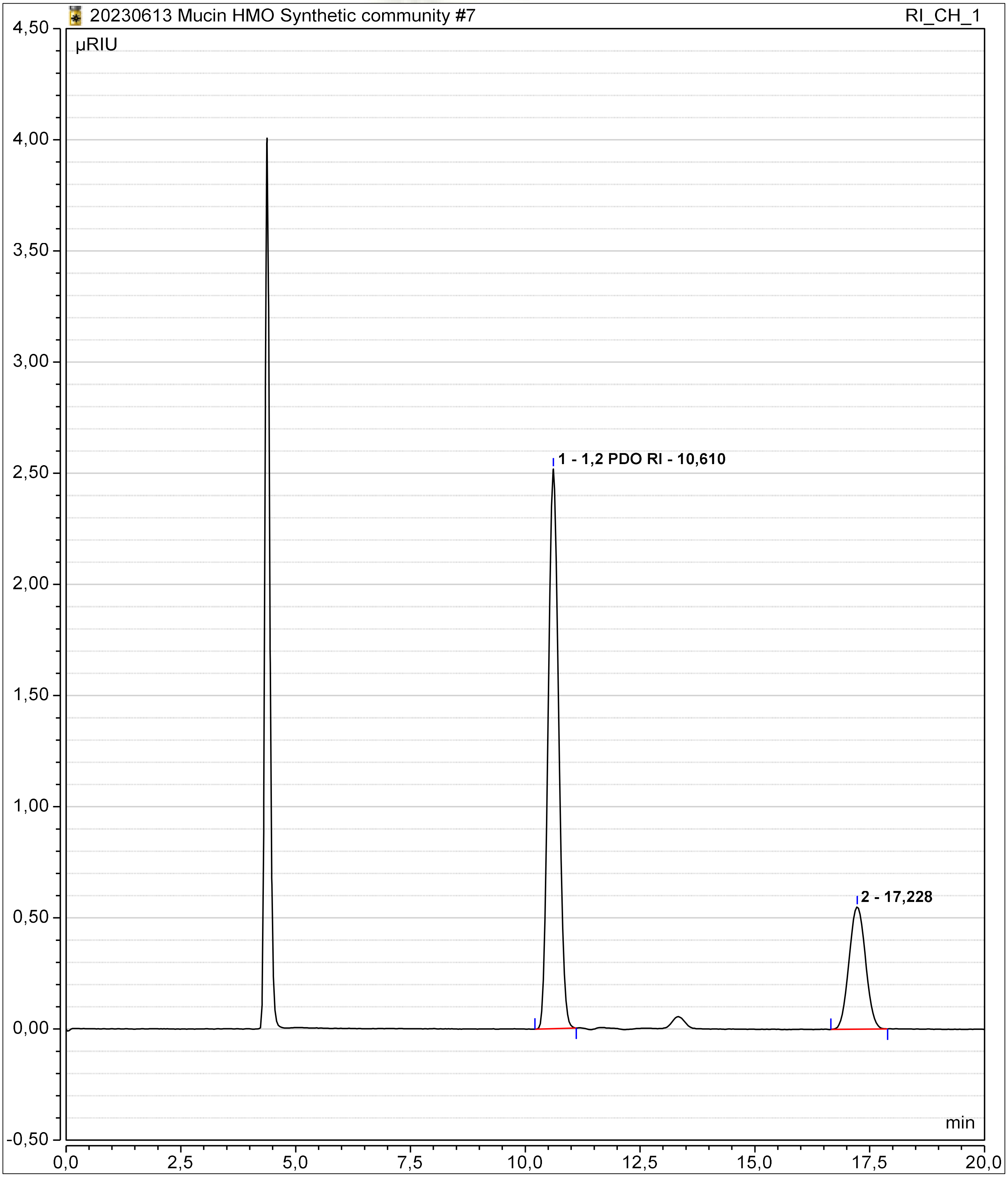

Chromatogram and Results

Injection Details

Injection Name:	**1,2-Prop & 1- propane 30**	*Run Time (min):*	**20,00**
Vial Number:	**3:A3**	*Injection Volume:*	**3,00**
Injection Type:	**Calibration Standard**	*Channel:*	**RI_CH_1**
Calibration Level:	**1**	*Wavelength:*	**n.a.**
Instrument Method:	**Default method LC2030C 45 gr 20 min**	*Bandwidth:*	**n.a.**
Processing Method:	**Processing Method LC2030 45 gr**	*Dilution Factor:*	**1,0000**
Injection Date/Time:	**13-jun-23 14:19**	*Sample Weight:*	**1,0000**

Chromatogram

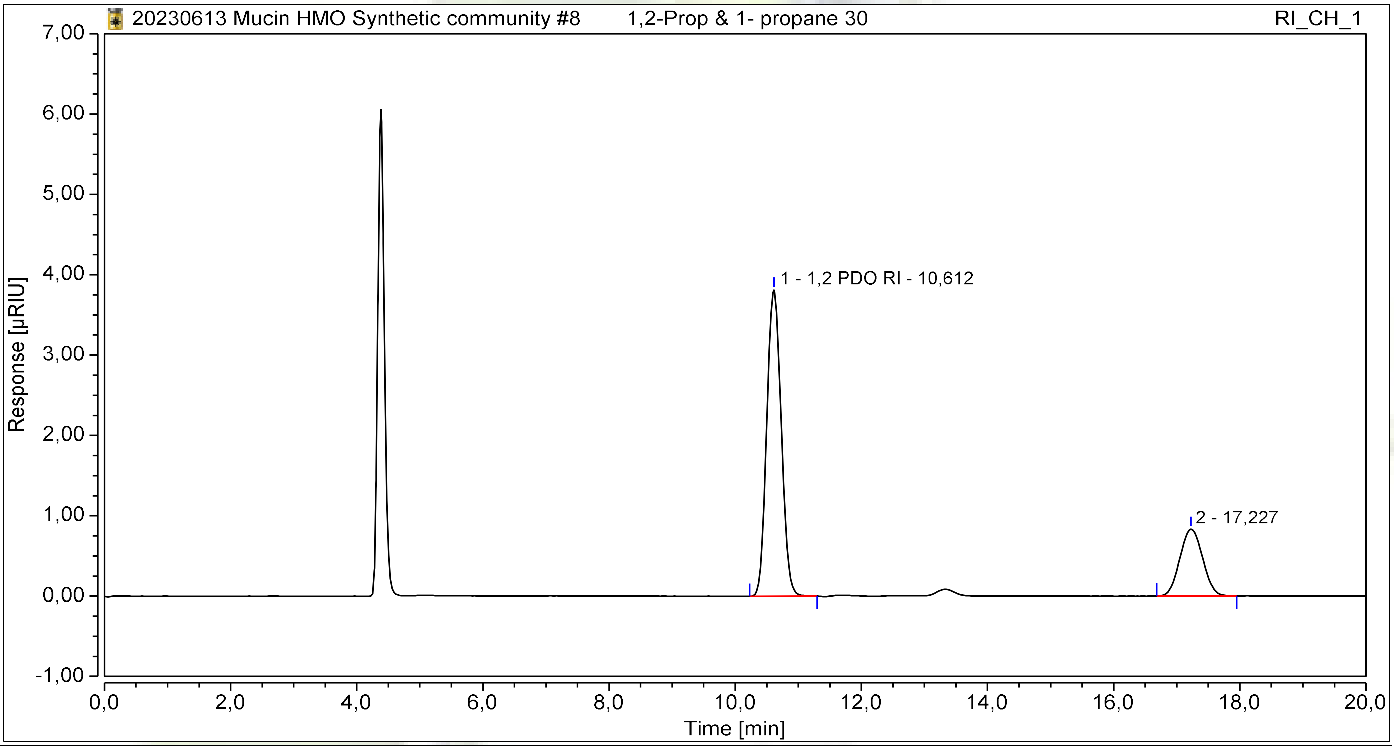

Integration Results

No.	Peak Name	Retention Time min	Area µRIU*min	Height µRIU	Relative Area %	Relative Height %	Amount
n.a.	GlcNAc	n.a.	n.a.	n.a.	n.a.	n.a.	n.a.
n.a.	Citrate	n.a.	n.a.	n.a.	n.a.	n.a.	n.a.
n.a.	Glucose	n.a.	n.a.	n.a.	n.a.	n.a.	n.a.
n.a.	Galactose	n.a.	n.a.	n.a.	n.a.	n.a.	n.a.
n.a.	Fucose	n.a.	n.a.	n.a.	n.a.	n.a.	n.a.
n.a.	Succinate RI	n.a.	n.a.	n.a.	n.a.	n.a.	n.a.
n.a.	Lactate RI	n.a.	n.a.	n.a.	n.a.	n.a.	n.a.
n.a.	glycerol	n.a.	n.a.	n.a.	n.a.	n.a.	n.a.
n.a.	Formate RI	n.a.	n.a.	n.a.	n.a.	n.a.	n.a.
n.a.	Acetate RI	n.a.	n.a.	n.a.	n.a.	n.a.	n.a.
1	1,2 PDO RI	10,612	1,014	3,809	74,47	82,09	30,1530
n.a.	1,3-PDO	n.a.	n.a.	n.a.	n.a.	n.a.	n.a.
n.a.	Propionate RI	n.a.	n.a.	n.a.	n.a.	n.a.	n.a.
n.a.	1,3-PDO	n.a.	n.a.	n.a.	n.a.	n.a.	n.a.
n.a.	2-3 BDO	n.a.	n.a.	n.a.	n.a.	n.a.	n.a.
n.a.	Ethanol	n.a.	n.a.	n.a.	n.a.	n.a.	n.a.
n.a.	Isobutyrate RI	n.a.	n.a.	n.a.	n.a.	n.a.	n.a.
n.a.	Butyrate RI	n.a.	n.a.	n.a.	n.a.	n.a.	n.a.
2		17,227	0,348	0,831	25,53	17,91	n.a.
**Total:**			**1,362**	**4,640**	**100,00**	**100,00**	

Peak Analysis

Injection Details

Injection Name:	**1,2-Prop & 1- propane 30**	*Run Time (min):*	**20,00**
Vial Number:	**3:A3**	*Injection Volume:*	**3,00**
Injection Type:	**Calibration Standard**	*Channel:*	**RI_CH_1**
Calibration Level:	**1**	*Wavelength:*	**n.a.**
Instrument Method:	**Default method LC2030C 45 gr 20 min**	*Bandwidth:*	**n.a.**
Processing Method:	**Processing Method LC2030 45 gr**	*Dilution Factor:*	**1,0000**
Injection Date/Time:	**13-jun-23 14:19**	*Sample Weight:*	**1,0000**

Chromatogram

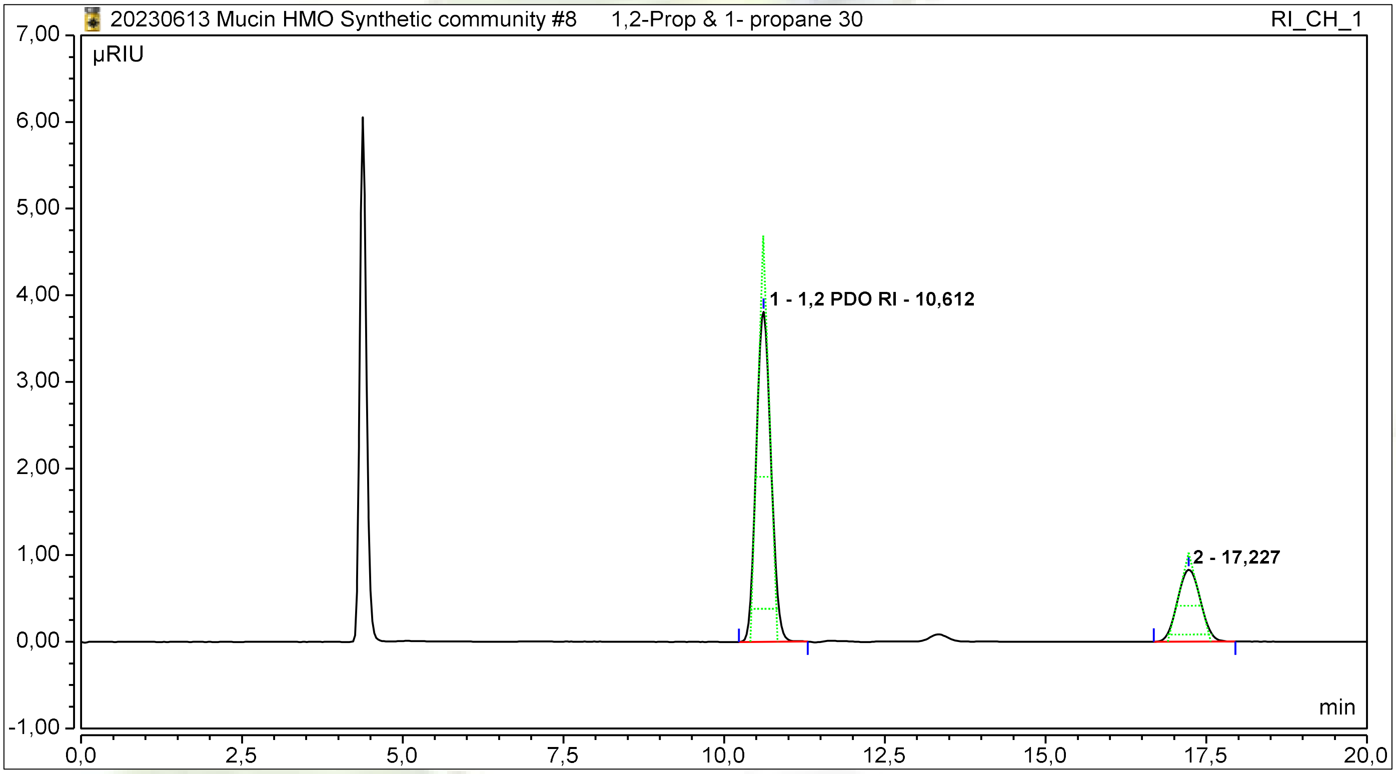

Peak Results

No.	Peak Name	Retention Time min	Width (50%) min	Type	Resolution (EP)	Asymmetry (EP)	Plates (EP)
n.a.	GlcNAc	n.a.	n.a.	n.a.	n.a.	n.a.	n.a.
n.a.	Citrate	n.a.	n.a.	n.a.	n.a.	n.a.	n.a.
n.a.	Glucose	n.a.	n.a.	n.a.	n.a.	n.a.	n.a.
n.a.	Galactose	n.a.	n.a.	n.a.	n.a.	n.a.	n.a.
n.a.	Fucose	n.a.	n.a.	n.a.	n.a.	n.a.	n.a.
n.a.	Succinate RI	n.a.	n.a.	n.a.	n.a.	n.a.	n.a.
n.a.	Lactate RI	n.a.	n.a.	n.a.	n.a.	n.a.	n.a.
n.a.	glycerol	n.a.	n.a.	n.a.	n.a.	n.a.	n.a.
n.a.	Formate RI	n.a.	n.a.	n.a.	n.a.	n.a.	n.a.
n.a.	Acetate RI	n.a.	n.a.	n.a.	n.a.	n.a.	n.a.
1	1,2 PDO RI	10,612	0,252	BMB	12,01	1,08	9861
n.a.	1,3-PDO	n.a.	n.a.	n.a.	n.a.	n.a.	n.a.
n.a.	Propionate RI	n.a.	n.a.	n.a.	n.a.	n.a.	n.a.
n.a.	1,3-PDO	n.a.	n.a.	n.a.	n.a.	n.a.	n.a.
n.a.	2-3 BDO	n.a.	n.a.	n.a.	n.a.	n.a.	n.a.
n.a.	Ethanol	n.a.	n.a.	n.a.	n.a.	n.a.	n.a.
n.a.	Isobutyrate RI	n.a.	n.a.	n.a.	n.a.	n.a.	n.a.
n.a.	Butyrate RI	n.a.	n.a.	n.a.	n.a.	n.a.	n.a.

Chromeleon (c) Dionex
Version 7.2.10.25868

2		17,227	0,398	BMB	n.a.	1,05	10363

Chromatogram and SST Results

Injection Details

Injection Name:	**1,2-Prop & 1- propane 30**	*Run Time (min):*	**20,00**
Vial Number:	**3:A3**	*Injection Volume:*	**3,00**
Injection Type:	**Calibration Standard**	*Channel:*	**RI_CH_1**
Calibration Level:	**1**	*Wavelength:*	**n.a.**
Instrument Method:	**Default method LC2030C 45 gr 20 min**	*Bandwidth:*	**n.a.**
Processing Method:	**Processing Method LC2030 45 gr**	*Dilution Factor:*	**1,0000**
Injection Date/Time:	**13-jun-23 14:19**	*Sample Weight:*	**1,0000**

Chromatogram

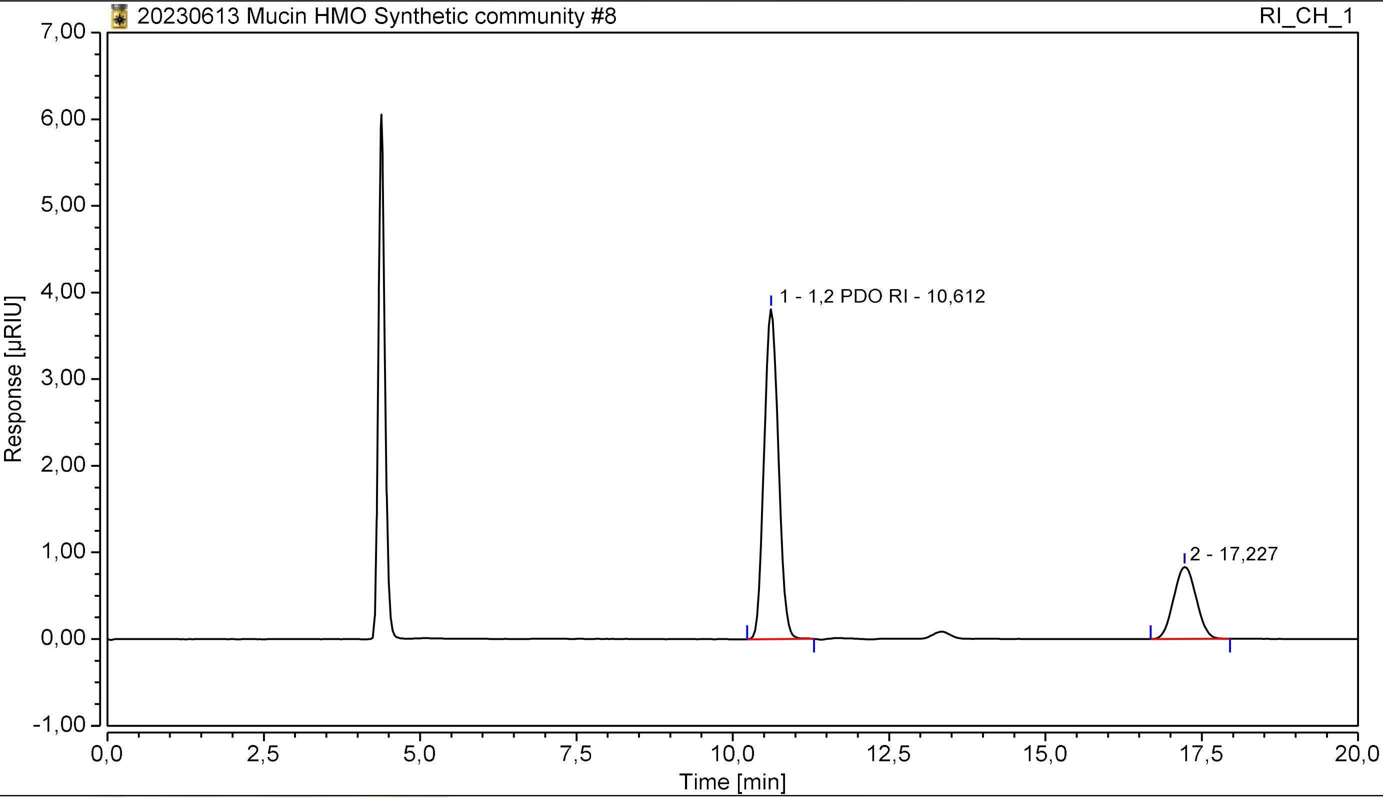

SST Results

No.	Name	Inj.Condition	Peak	Test Result	Injection
**Number of executed test cases:**	**n.a.**		**Total Result:**	**Passed**	

Chromeleon (c) Dionex
Version 7.2.10.25868

Chromatogram

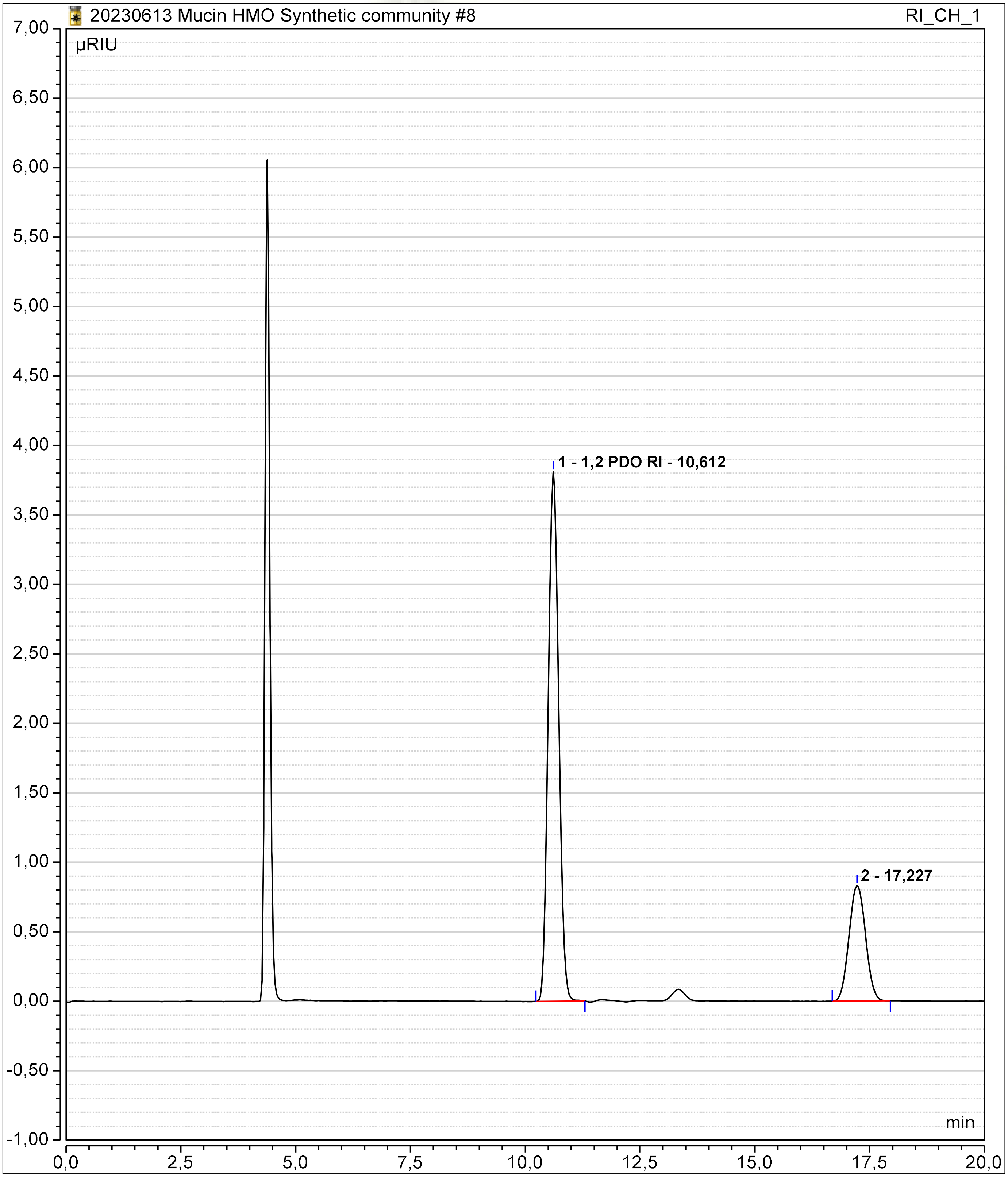

Chromatogram and Results

Injection Details

Injection Name:	**Meth & Eth 100**	*Run Time (min):*	**20,00**
Vial Number:	**3:A4**	*Injection Volume:*	**1,00**
Injection Type:	**Calibration Standard**	*Channel:*	**RI_CH_1**
Calibration Level:	**1**	*Wavelength:*	**n.a.**
Instrument Method:	**Default method LC2030C 45 gr 20 min**	*Bandwidth:*	**n.a.**
Processing Method:	**Processing Method LC2030 45 gr**	*Dilution Factor:*	**1,0000**
Injection Date/Time:	**13-jun-23 14:40**	*Sample Weight:*	**1,0000**

Chromatogram

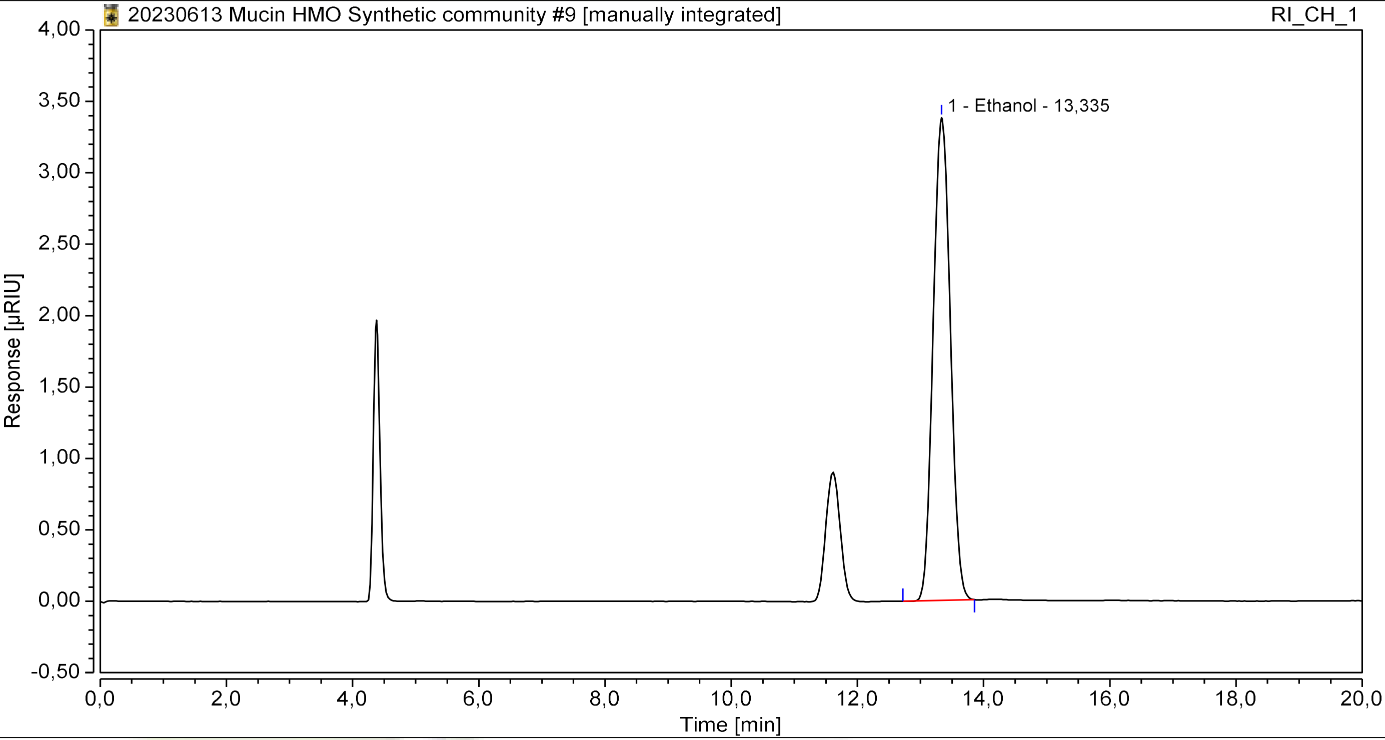

Integration Results

No.	Peak Name	Retention Time min	Area µRIU*min	Height µRIU	Relative Area %	Relative Height %	Amount
n.a.	GlcNAc	n.a.	n.a.	n.a.	n.a.	n.a.	n.a.
n.a.	Citrate	n.a.	n.a.	n.a.	n.a.	n.a.	n.a.
n.a.	Glucose	n.a.	n.a.	n.a.	n.a.	n.a.	n.a.
n.a.	Galactose	n.a.	n.a.	n.a.	n.a.	n.a.	n.a.
n.a.	Fucose	n.a.	n.a.	n.a.	n.a.	n.a.	n.a.
n.a.	Succinate RI	n.a.	n.a.	n.a.	n.a.	n.a.	n.a.
n.a.	Lactate RI	n.a.	n.a.	n.a.	n.a.	n.a.	n.a.
n.a.	glycerol	n.a.	n.a.	n.a.	n.a.	n.a.	n.a.
n.a.	Formate RI	n.a.	n.a.	n.a.	n.a.	n.a.	n.a.
n.a.	Acetate RI	n.a.	n.a.	n.a.	n.a.	n.a.	n.a.
n.a.	1,2 PDO RI	n.a.	n.a.	n.a.	n.a.	n.a.	n.a.
n.a.	1,3-PDO	n.a.	n.a.	n.a.	n.a.	n.a.	n.a.
n.a.	Propionate RI	n.a.	n.a.	n.a.	n.a.	n.a.	n.a.
n.a.	1,3-PDO	n.a.	n.a.	n.a.	n.a.	n.a.	n.a.
n.a.	2-3 BDO	n.a.	n.a.	n.a.	n.a.	n.a.	n.a.
1	Ethanol	13,335	1,066	3,380	100,00	100,00	9,8207
n.a.	Isobutyrate RI	n.a.	n.a.	n.a.	n.a.	n.a.	n.a.
n.a.	Butyrate RI	n.a.	n.a.	n.a.	n.a.	n.a.	n.a.
**Total:**			**1,066**	**3,380**	**100,00**	**100,00**	

Peak Analysis

Injection Details

Injection Name:	**Meth & Eth 100**	*Run Time (min):*	**20,00**
Vial Number:	**3:A4**	*Injection Volume:*	**1,00**
Injection Type:	**Calibration Standard**	*Channel:*	**RI_CH_1**
Calibration Level:	**1**	*Wavelength:*	**n.a.**
Instrument Method:	**Default method LC2030C 45 gr 20 min**	*Bandwidth:*	**n.a.**
Processing Method:	**Processing Method LC2030 45 gr**	*Dilution Factor:*	**1,0000**
Injection Date/Time:	**13-jun-23 14:40**	*Sample Weight:*	**1,0000**

Chromatogram

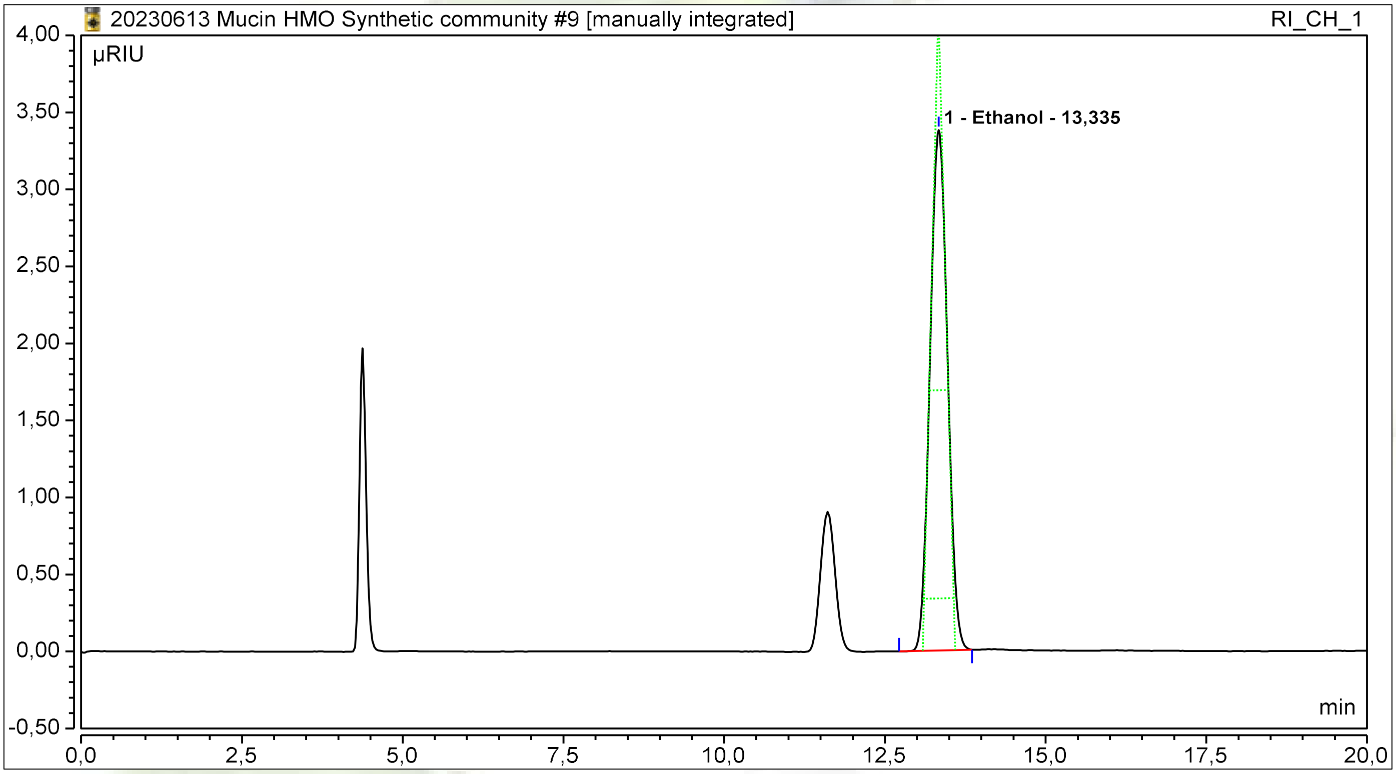

Peak Results

No.	Peak Name	Retention Time<br>min	Width (50%)<br>min	Type	Resolution (EP)	Asymmetry (EP)	Plates (EP)
n.a.	GlcNAc	n.a.	n.a.	n.a.	n.a.	n.a.	n.a.
n.a.	Citrate	n.a.	n.a.	n.a.	n.a.	n.a.	n.a.
n.a.	Glucose	n.a.	n.a.	n.a.	n.a.	n.a.	n.a.
n.a.	Galactose	n.a.	n.a.	n.a.	n.a.	n.a.	n.a.
n.a.	Fucose	n.a.	n.a.	n.a.	n.a.	n.a.	n.a.
n.a.	Succinate RI	n.a.	n.a.	n.a.	n.a.	n.a.	n.a.
n.a.	Lactate RI	n.a.	n.a.	n.a.	n.a.	n.a.	n.a.
n.a.	glycerol	n.a.	n.a.	n.a.	n.a.	n.a.	n.a.
n.a.	Formate RI	n.a.	n.a.	n.a.	n.a.	n.a.	n.a.
n.a.	Acetate RI	n.a.	n.a.	n.a.	n.a.	n.a.	n.a.
n.a.	1,2 PDO RI	n.a.	n.a.	n.a.	n.a.	n.a.	n.a.
n.a.	1,3-PDO	n.a.	n.a.	n.a.	n.a.	n.a.	n.a.
n.a.	Propionate RI	n.a.	n.a.	n.a.	n.a.	n.a.	n.a.
n.a.	1,3-PDO	n.a.	n.a.	n.a.	n.a.	n.a.	n.a.
n.a.	2-3 BDO	n.a.	n.a.	n.a.	n.a.	n.a.	n.a.
1	Ethanol	13,335	0,300	BMB*	n.a.	1,06	10918
n.a.	Isobutyrate RI	n.a.	n.a.	n.a.	n.a.	n.a.	n.a.
n.a.	Butyrate RI	n.a.	n.a.	n.a.	n.a.	n.a.	n.a.

Chromatogram and SST Results

Injection Details

Injection Name:	**Meth & Eth 100**	*Run Time (min):*	**20,00**
Vial Number:	**3:A4**	*Injection Volume:*	**1,00**
Injection Type:	**Calibration Standard**	*Channel:*	**RI_CH_1**
Calibration Level:	**1**	*Wavelength:*	**n.a.**
Instrument Method:	**Default method LC2030C 45 gr 20 min**	*Bandwidth:*	**n.a.**
Processing Method:	**Processing Method LC2030 45 gr**	*Dilution Factor:*	**1,0000**
Injection Date/Time:	**13-jun-23 14:40**	*Sample Weight:*	**1,0000**

Chromatogram

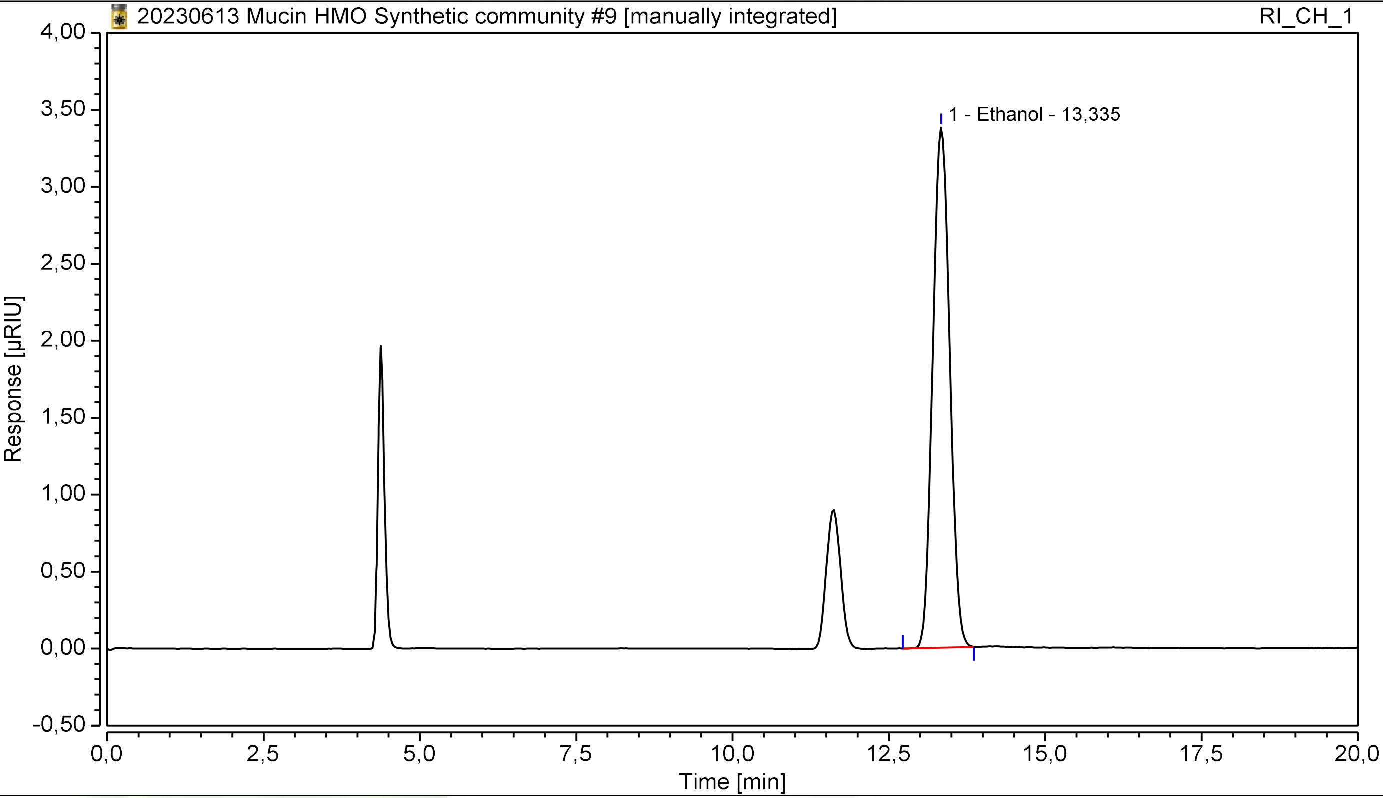

SST Results

No.	Name	Inj.Condition	Peak	Test Result	Injection
**Number of executed test cases:**	**n.a.**		**Total Result:**	**Passed**	

Chromatogram

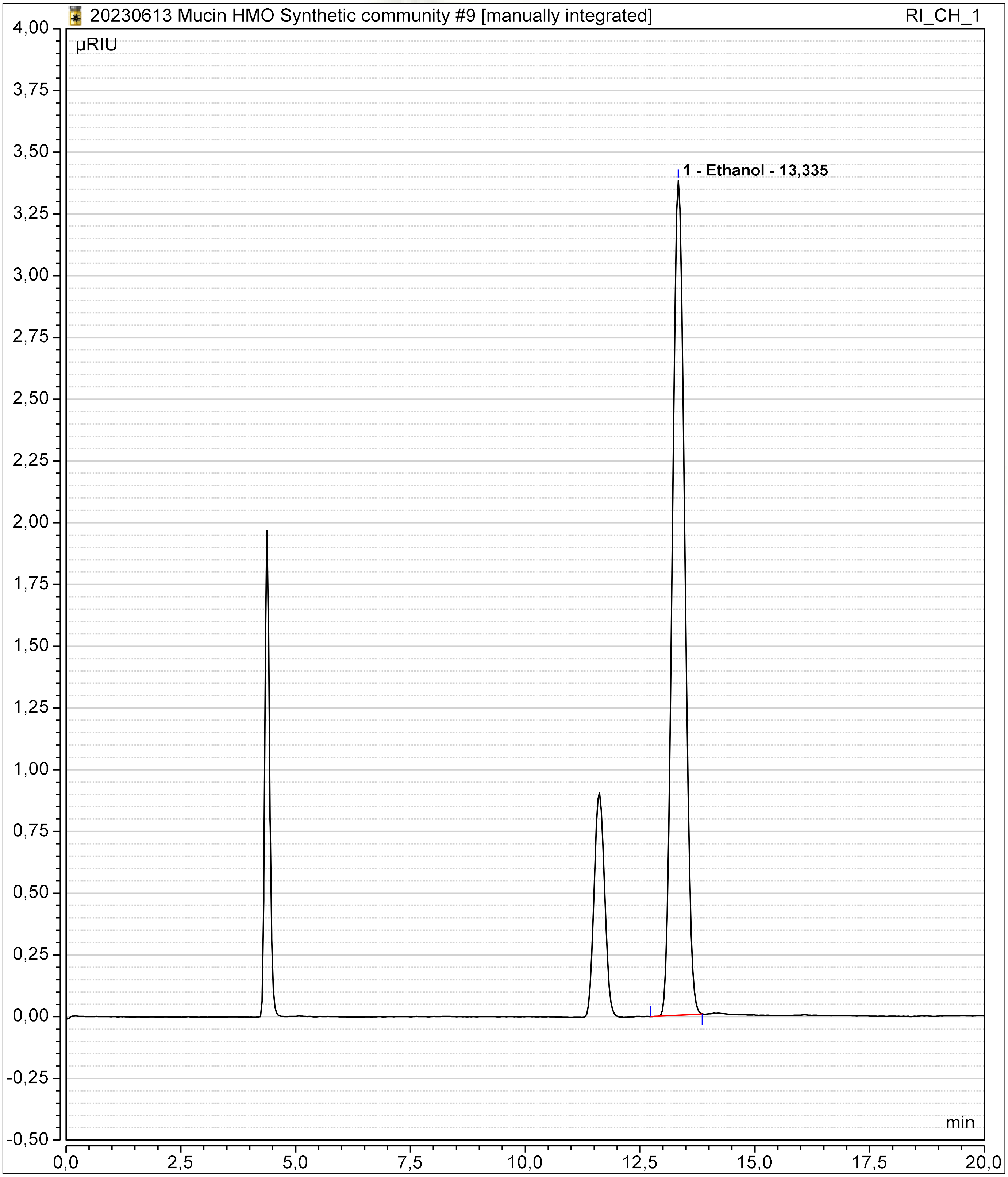

Chromatogram and Results

Injection Details

Injection Name:	**Meth & Eth 200**	*Run Time (min):*	**20,00**
Vial Number:	**3:A4**	*Injection Volume:*	**2,00**
Injection Type:	**Calibration Standard**	*Channel:*	**RI_CH_1**
Calibration Level:	**1**	*Wavelength:*	**n.a.**
Instrument Method:	**Default method LC2030C 45 gr 20 min**	*Bandwidth:*	**n.a.**
Processing Method:	**Processing Method LC2030 45 gr**	*Dilution Factor:*	**1,0000**
Injection Date/Time:	**13-jun-23 15:00**	*Sample Weight:*	**1,0000**

Chromatogram

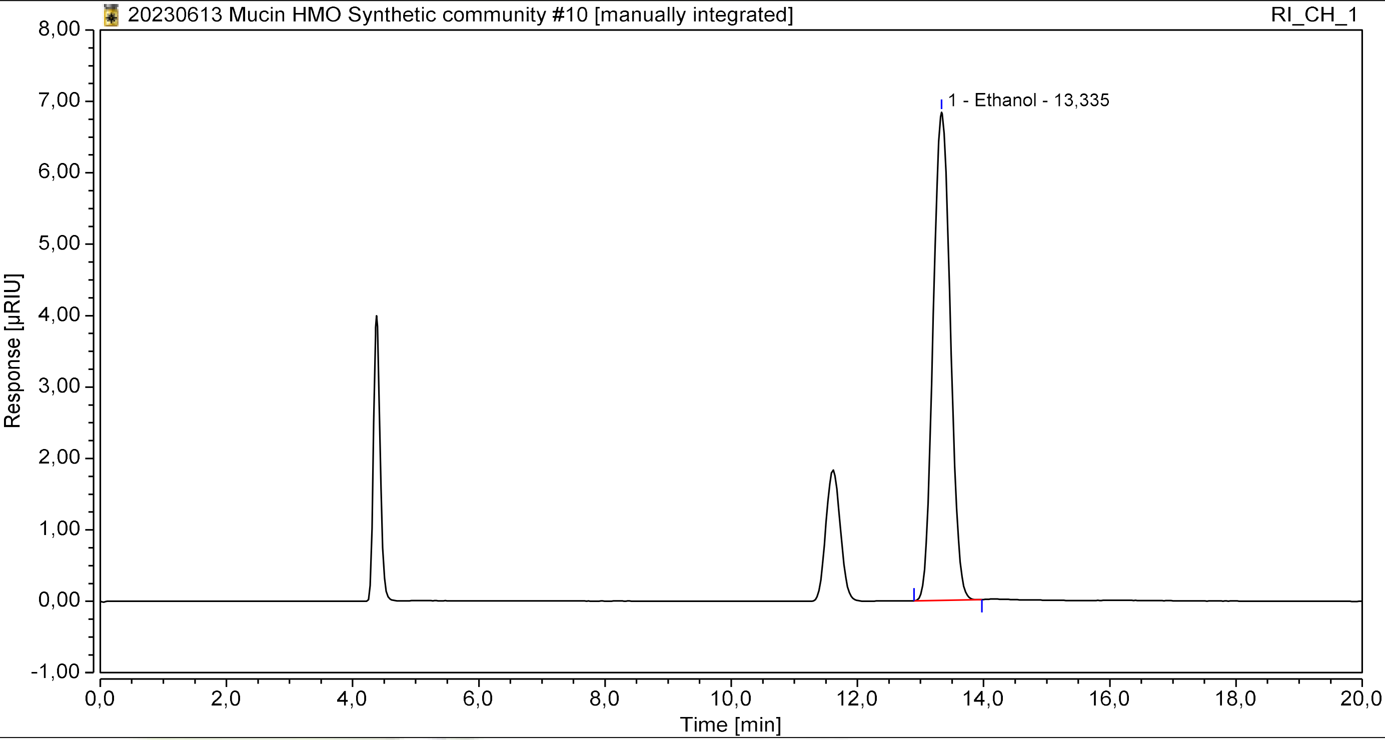

Integration Results

No.	Peak Name	Retention Time min	Area µRIU*min	Height µRIU	Relative Area %	Relative Height %	Amount
n.a.	GlcNAc	n.a.	n.a.	n.a.	n.a.	n.a.	n.a.
n.a.	Citrate	n.a.	n.a.	n.a.	n.a.	n.a.	n.a.
n.a.	Glucose	n.a.	n.a.	n.a.	n.a.	n.a.	n.a.
n.a.	Galactose	n.a.	n.a.	n.a.	n.a.	n.a.	n.a.
n.a.	Fucose	n.a.	n.a.	n.a.	n.a.	n.a.	n.a.
n.a.	Succinate RI	n.a.	n.a.	n.a.	n.a.	n.a.	n.a.
n.a.	Lactate RI	n.a.	n.a.	n.a.	n.a.	n.a.	n.a.
n.a.	glycerol	n.a.	n.a.	n.a.	n.a.	n.a.	n.a.
n.a.	Formate RI	n.a.	n.a.	n.a.	n.a.	n.a.	n.a.
n.a.	Acetate RI	n.a.	n.a.	n.a.	n.a.	n.a.	n.a.
n.a.	1,2 PDO RI	n.a.	n.a.	n.a.	n.a.	n.a.	n.a.
n.a.	1,3-PDO	n.a.	n.a.	n.a.	n.a.	n.a.	n.a.
n.a.	Propionate RI	n.a.	n.a.	n.a.	n.a.	n.a.	n.a.
n.a.	1,3-PDO	n.a.	n.a.	n.a.	n.a.	n.a.	n.a.
n.a.	2-3 BDO	n.a.	n.a.	n.a.	n.a.	n.a.	n.a.
1	Ethanol	13,335	2,163	6,834	100,00	100,00	19,9217
n.a.	Isobutyrate RI	n.a.	n.a.	n.a.	n.a.	n.a.	n.a.
n.a.	Butyrate RI	n.a.	n.a.	n.a.	n.a.	n.a.	n.a.
**Total:**			**2,163**	**6,834**	**100,00**	**100,00**	

Peak Analysis

Injection Details

Injection Name:	**Meth & Eth 200**	*Run Time (min):*	**20,00**
Vial Number:	**3:A4**	*Injection Volume:*	**2,00**
Injection Type:	**Calibration Standard**	*Channel:*	**RI_CH_1**
Calibration Level:	**1**	*Wavelength:*	**n.a.**
Instrument Method:	**Default method LC2030C 45 gr 20 min**	*Bandwidth:*	**n.a.**
Processing Method:	**Processing Method LC2030 45 gr**	*Dilution Factor:*	**1,0000**
Injection Date/Time:	**13-jun-23 15:00**	*Sample Weight:*	**1,0000**

Chromatogram

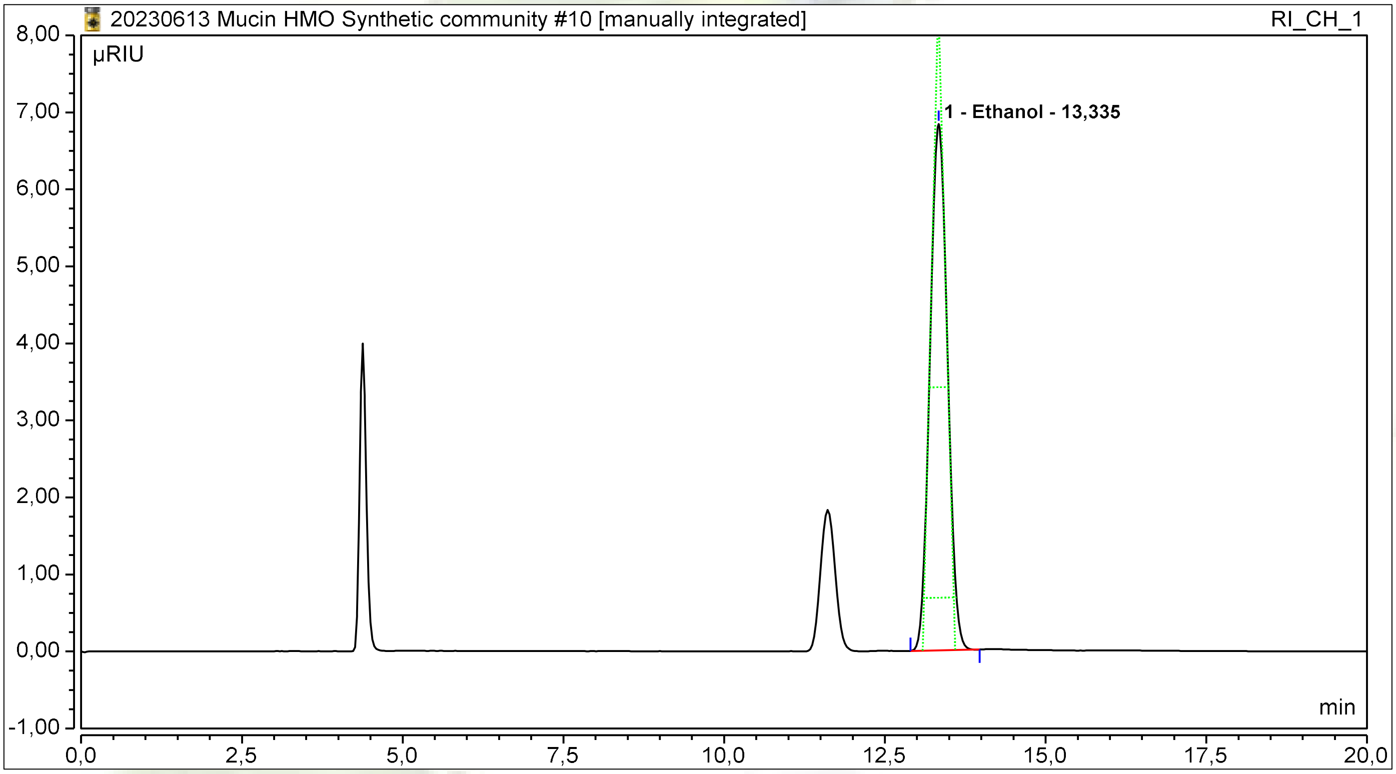

Peak Results

No.	Peak Name	Retention Time min	Width (50%) min	Type	Resolution (EP)	Asymmetry (EP)	Plates (EP)
n.a.	GlcNAc	n.a.	n.a.	n.a.	n.a.	n.a.	n.a.
n.a.	Citrate	n.a.	n.a.	n.a.	n.a.	n.a.	n.a.
n.a.	Glucose	n.a.	n.a.	n.a.	n.a.	n.a.	n.a.
n.a.	Galactose	n.a.	n.a.	n.a.	n.a.	n.a.	n.a.
n.a.	Fucose	n.a.	n.a.	n.a.	n.a.	n.a.	n.a.
n.a.	Succinate RI	n.a.	n.a.	n.a.	n.a.	n.a.	n.a.
n.a.	Lactate RI	n.a.	n.a.	n.a.	n.a.	n.a.	n.a.
n.a.	glycerol	n.a.	n.a.	n.a.	n.a.	n.a.	n.a.
n.a.	Formate RI	n.a.	n.a.	n.a.	n.a.	n.a.	n.a.
n.a.	Acetate RI	n.a.	n.a.	n.a.	n.a.	n.a.	n.a.
n.a.	1,2 PDO RI	n.a.	n.a.	n.a.	n.a.	n.a.	n.a.
n.a.	1,3-PDO	n.a.	n.a.	n.a.	n.a.	n.a.	n.a.
n.a.	Propionate RI	n.a.	n.a.	n.a.	n.a.	n.a.	n.a.
n.a.	1,3-PDO	n.a.	n.a.	n.a.	n.a.	n.a.	n.a.
n.a.	2-3 BDO	n.a.	n.a.	n.a.	n.a.	n.a.	n.a.
1	Ethanol	13,335	0,301	BMB*	n.a.	1,06	10864
n.a.	Isobutyrate RI	n.a.	n.a.	n.a.	n.a.	n.a.	n.a.
n.a.	Butyrate RI	n.a.	n.a.	n.a.	n.a.	n.a.	n.a.

Chromatogram and SST Results

Injection Details

Injection Name:	**Meth & Eth 200**	*Run Time (min):*	**20,00**
Vial Number:	**3:A4**	*Injection Volume:*	**2,00**
Injection Type:	**Calibration Standard**	*Channel:*	**RI_CH_1**
Calibration Level:	**1**	*Wavelength:*	**n.a.**
Instrument Method:	**Default method LC2030C 45 gr 20 min**	*Bandwidth:*	**n.a.**
Processing Method:	**Processing Method LC2030 45 gr**	*Dilution Factor:*	**1,0000**
Injection Date/Time:	**13-jun-23 15:00**	*Sample Weight:*	**1,0000**

Chromatogram

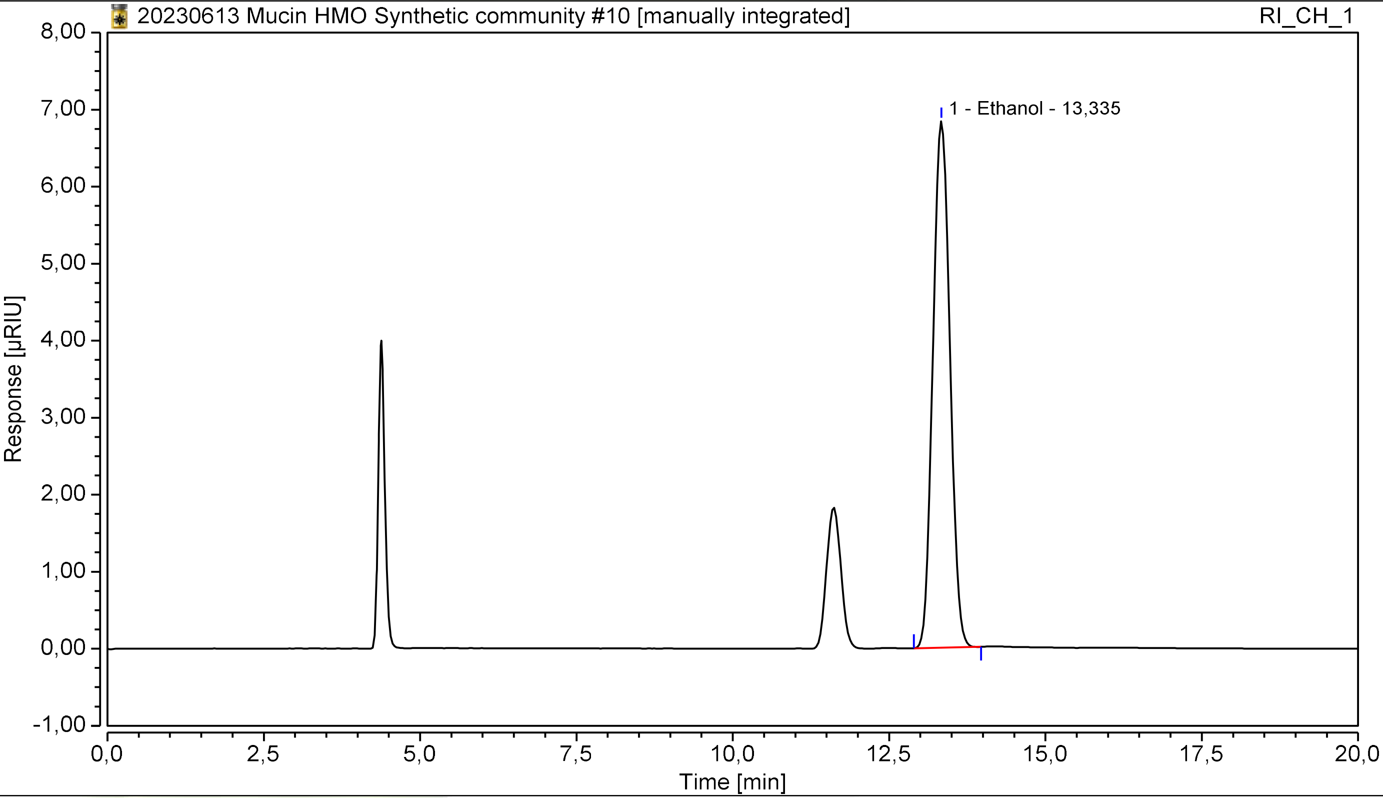

SST Results

No.	Name	Inj.Condition	Peak	Test Result	Injection
**Number of executed test cases:**	**n.a.**		**Total Result:**	**Passed**	

Chromatogram

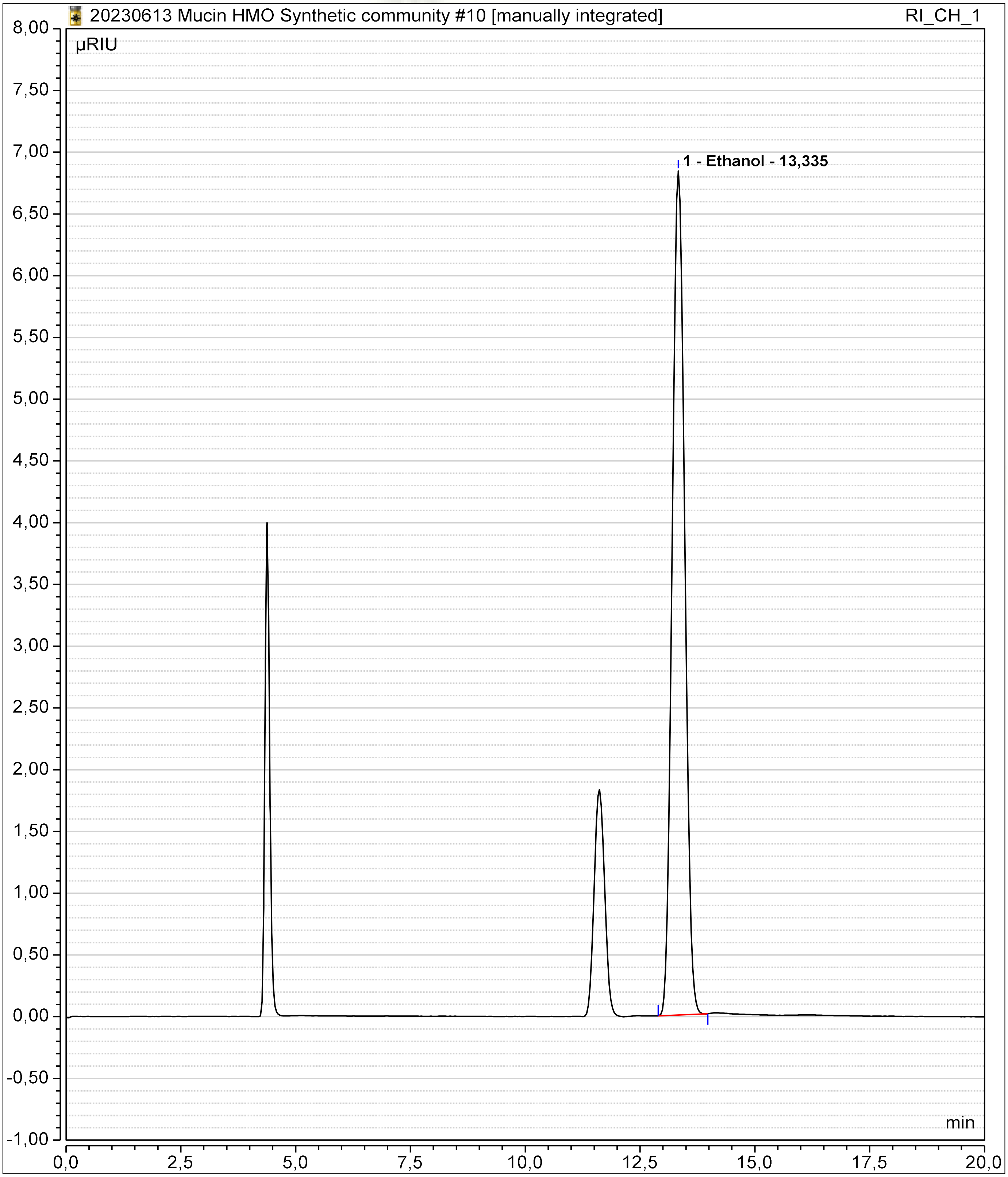

Chromeleon (c) Dionex
Version 7.2.10.25868

Chromatogram and Results

Injection Details

Injection Name:	**Meth & Eth 300**	*Run Time (min):*	**20,00**
Vial Number:	**3:A4**	*Injection Volume:*	**3,00**
Injection Type:	**Calibration Standard**	*Channel:*	**RI_CH_1**
Calibration Level:	**1**	*Wavelength:*	**n.a.**
Instrument Method:	**Default method LC2030C 45 gr 20 min**	*Bandwidth:*	**n.a.**
Processing Method:	**Processing Method LC2030 45 gr**	*Dilution Factor:*	**1,0000**
Injection Date/Time:	**13-jun-23 15:20**	*Sample Weight:*	**1,0000**

Chromatogram

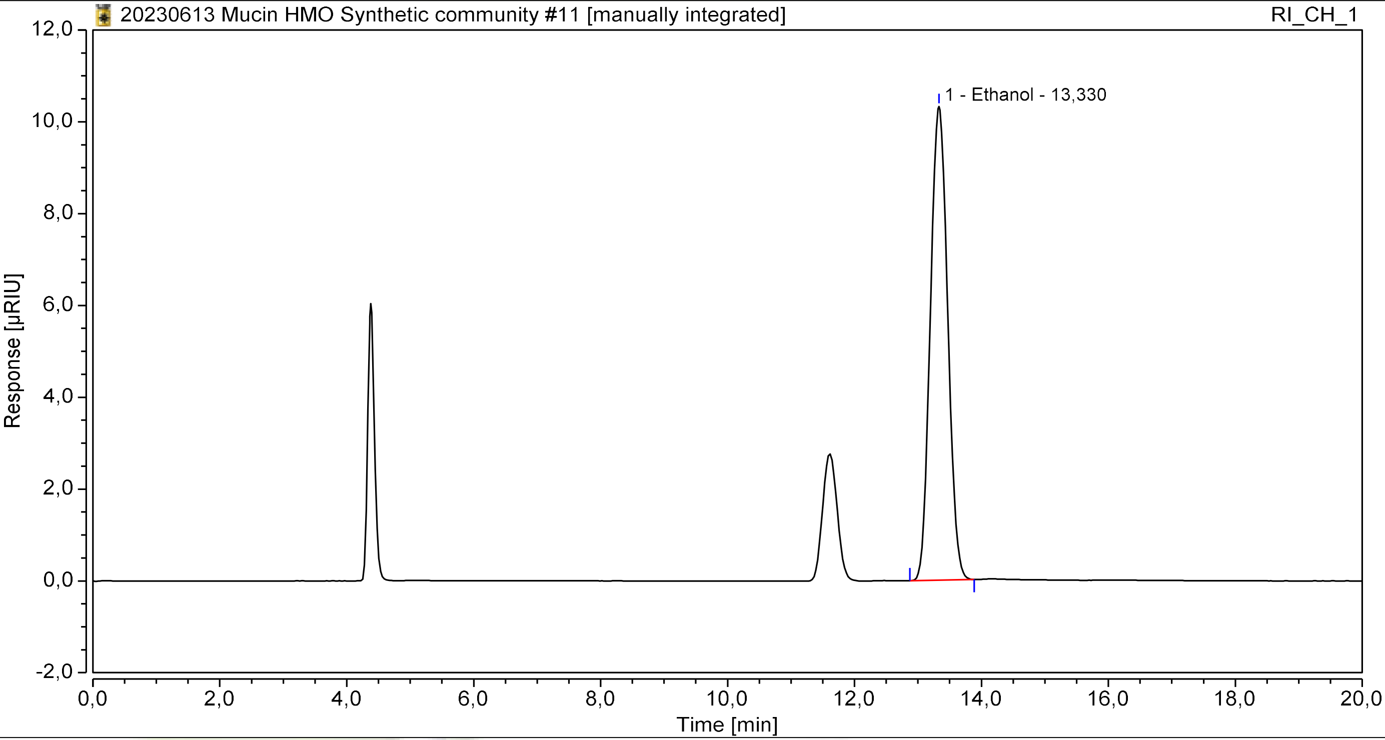

Integration Results

No.	Peak Name	Retention Time<br>min	Area<br>µRIU*min	Height<br>µRIU	Relative Area<br>%	Relative Height<br>%	Amount
n.a.	GlcNAc	n.a.	n.a.	n.a.	n.a.	n.a.	n.a.
n.a.	Citrate	n.a.	n.a.	n.a.	n.a.	n.a.	n.a.
n.a.	Glucose	n.a.	n.a.	n.a.	n.a.	n.a.	n.a.
n.a.	Galactose	n.a.	n.a.	n.a.	n.a.	n.a.	n.a.
n.a.	Fucose	n.a.	n.a.	n.a.	n.a.	n.a.	n.a.
n.a.	Succinate RI	n.a.	n.a.	n.a.	n.a.	n.a.	n.a.
n.a.	Lactate RI	n.a.	n.a.	n.a.	n.a.	n.a.	n.a.
n.a.	glycerol	n.a.	n.a.	n.a.	n.a.	n.a.	n.a.
n.a.	Formate RI	n.a.	n.a.	n.a.	n.a.	n.a.	n.a.
n.a.	Acetate RI	n.a.	n.a.	n.a.	n.a.	n.a.	n.a.
n.a.	1,2 PDO RI	n.a.	n.a.	n.a.	n.a.	n.a.	n.a.
n.a.	1,3-PDO	n.a.	n.a.	n.a.	n.a.	n.a.	n.a.
n.a.	Propionate RI	n.a.	n.a.	n.a.	n.a.	n.a.	n.a.
n.a.	1,3-PDO	n.a.	n.a.	n.a.	n.a.	n.a.	n.a.
n.a.	2-3 BDO	n.a.	n.a.	n.a.	n.a.	n.a.	n.a.
1	Ethanol	13,330	3,269	10,316	100,00	100,00	30,1120
n.a.	Isobutyrate RI	n.a.	n.a.	n.a.	n.a.	n.a.	n.a.
n.a.	Butyrate RI	n.a.	n.a.	n.a.	n.a.	n.a.	n.a.
**Total:**			**3,269**	**10,316**	**100,00**	**100,00**	

Calibration

Calibration Details	Lactate RI		
Calibration Type	Lin	*Offset (C0)*	0,0000
Evaluation Type	Area	*Slope (C1)*	0,0344
Number of Calibration Points	3	*Curve (C2)*	0,0000
Number of disabled Calibration Points	0	*R-Square*	0,9990

Calibration Plot — Lactate RI

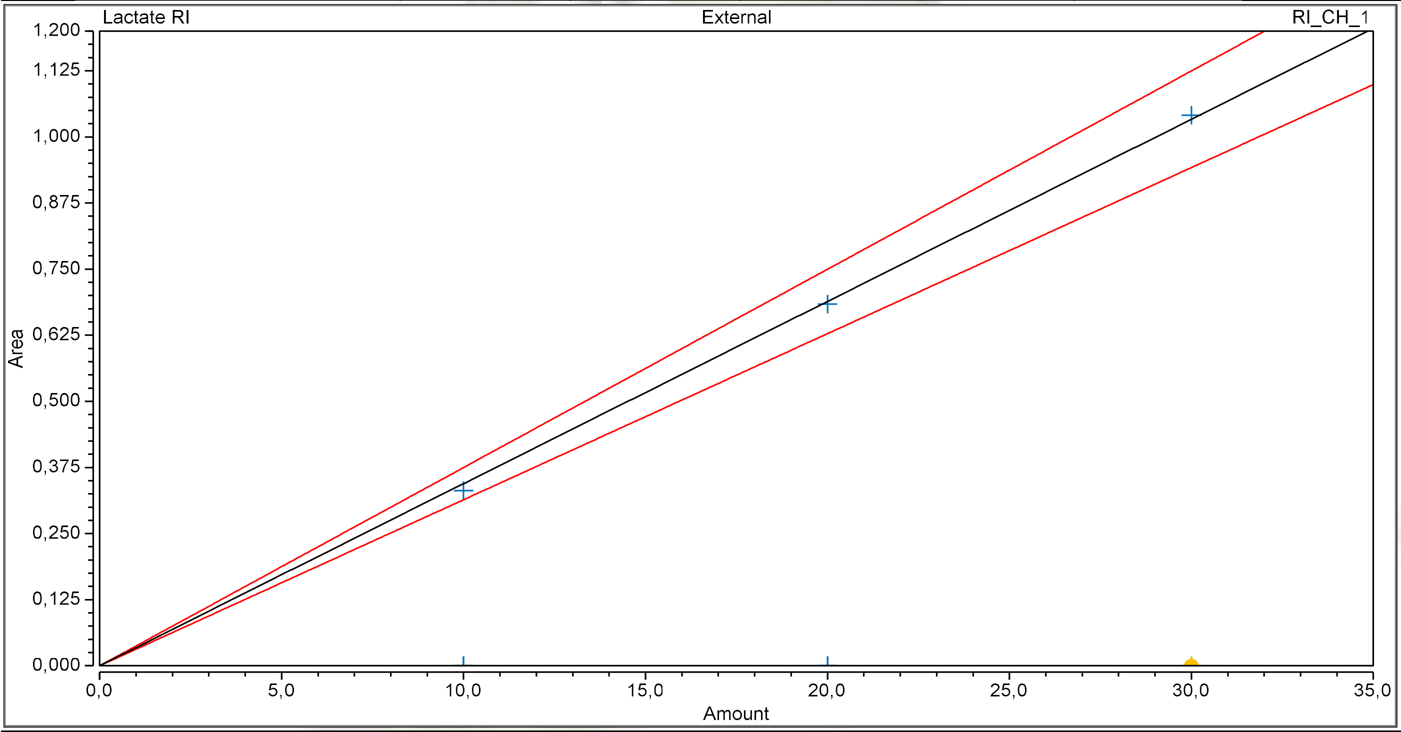

Calibration Results — Lactate RI

No.	Injection Name	Calibration Level	X Value<br>RI_CH_1<br>Lactate RI	Y Value<br>RI_CH_1<br>Lactate RI	Y Value<br>RI_CH_1<br>Lactate RI	Area<br>µRIU*min<br>RI_CH_1<br>Lactate RI	Height<br>µRIU<br>RI_CH_1<br>Lactate RI
3	VFA 10	1	10,0000	0,3309	0,3309	0,331	1,545
4	VFA 20	1	20,0000	0,6841	0,6841	0,684	3,162
5	VFA 30	1	30,0000	1,0410	1,0410	1,041	4,810
6	1,2-Prop & 1- propane 10	1	10,0000	n.a.	n.a.	n.a.	n.a.
7	1,2-Prop & 1- propane 20	1	20,0000	n.a.	n.a.	n.a.	n.a.
8	1,2-Prop & 1- propane 30	1	30,0000	n.a.	n.a.	n.a.	n.a.
9	Meth & Eth 100	1	10,0000	n.a.	n.a.	n.a.	n.a.
10	Meth & Eth 200	1	20,0000	n.a.	n.a.	n.a.	n.a.
11	Meth & Eth 300	1	30,0000	n.a.	n.a.	n.a.	n.a.

Peak Analysis

Injection Details

Injection Name:	**Meth & Eth 300**	*Run Time (min):*	**20,00**
Vial Number:	**3:A4**	*Injection Volume:*	**3,00**
Injection Type:	**Calibration Standard**	*Channel:*	**RI_CH_1**
Calibration Level:	**1**	*Wavelength:*	**n.a.**
Instrument Method:	**Default method LC2030C 45 gr 20 min**	*Bandwidth:*	**n.a.**
Processing Method:	**Processing Method LC2030 45 gr**	*Dilution Factor:*	**1,0000**
Injection Date/Time:	**13-jun-23 15:20**	*Sample Weight:*	**1,0000**

Chromatogram

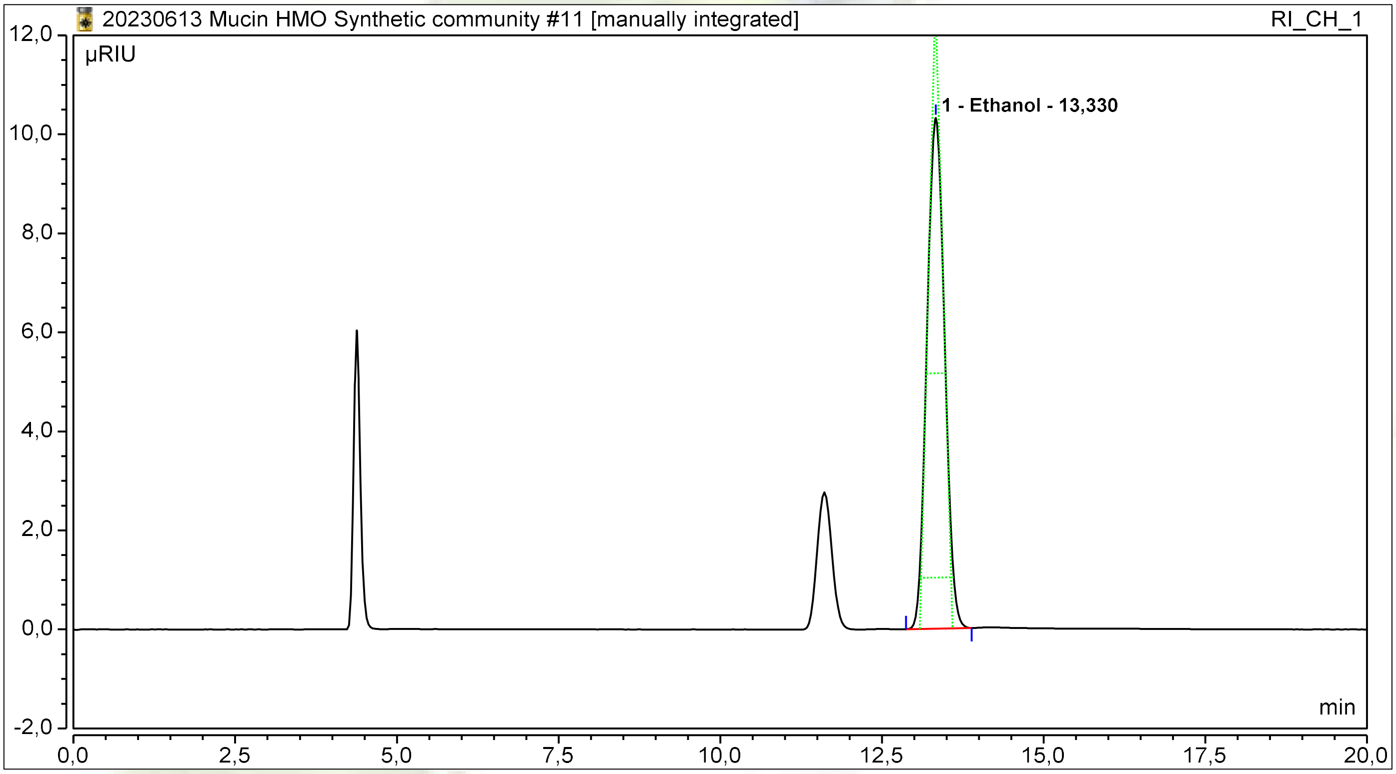

Peak Results

No.	Peak Name	Retention Time min	Width (50%) min	Type	Resolution (EP)	Asymmetry (EP)	Plates (EP)
n.a.	GlcNAc	n.a.	n.a.	n.a.	n.a.	n.a.	n.a.
n.a.	Citrate	n.a.	n.a.	n.a.	n.a.	n.a.	n.a.
n.a.	Glucose	n.a.	n.a.	n.a.	n.a.	n.a.	n.a.
n.a.	Galactose	n.a.	n.a.	n.a.	n.a.	n.a.	n.a.
n.a.	Fucose	n.a.	n.a.	n.a.	n.a.	n.a.	n.a.
n.a.	Succinate RI	n.a.	n.a.	n.a.	n.a.	n.a.	n.a.
n.a.	Lactate RI	n.a.	n.a.	n.a.	n.a.	n.a.	n.a.
n.a.	glycerol	n.a.	n.a.	n.a.	n.a.	n.a.	n.a.
n.a.	Formate RI	n.a.	n.a.	n.a.	n.a.	n.a.	n.a.
n.a.	Acetate RI	n.a.	n.a.	n.a.	n.a.	n.a.	n.a.
n.a.	1,2 PDO RI	n.a.	n.a.	n.a.	n.a.	n.a.	n.a.
n.a.	1,3-PDO	n.a.	n.a.	n.a.	n.a.	n.a.	n.a.
n.a.	Propionate RI	n.a.	n.a.	n.a.	n.a.	n.a.	n.a.
n.a.	1,3-PDO	n.a.	n.a.	n.a.	n.a.	n.a.	n.a.
n.a.	2-3 BDO	n.a.	n.a.	n.a.	n.a.	n.a.	n.a.
1	Ethanol	13,330	0,302	BMB*	n.a.	1,07	10825
n.a.	Isobutyrate RI	n.a.	n.a.	n.a.	n.a.	n.a.	n.a.
n.a.	Butyrate RI	n.a.	n.a.	n.a.	n.a.	n.a.	n.a.

Chromeleon (c) Dionex
Version 7.2.10.25868

Chromatogram and SST Results

Injection Details

Injection Name:	**Meth & Eth 300**	*Run Time (min):*	**20,00**
Vial Number:	**3:A4**	*Injection Volume:*	**3,00**
Injection Type:	**Calibration Standard**	*Channel:*	**RI_CH_1**
Calibration Level:	**1**	*Wavelength:*	**n.a.**
Instrument Method:	**Default method LC2030C 45 gr 20 min**	*Bandwidth:*	**n.a.**
Processing Method:	**Processing Method LC2030 45 gr**	*Dilution Factor:*	**1,0000**
Injection Date/Time:	**13-jun-23 15:20**	*Sample Weight:*	**1,0000**

Chromatogram

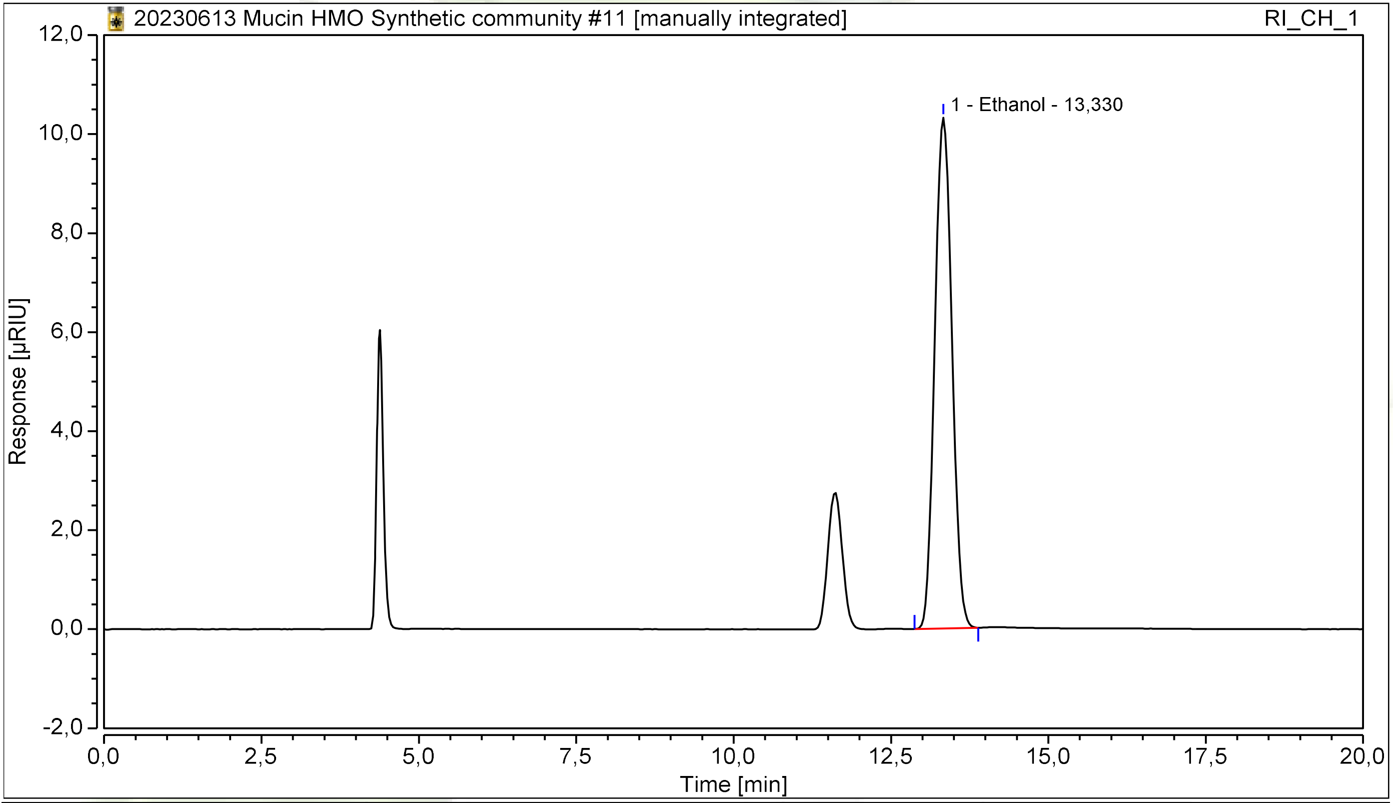

SST Results

No.	Name	Inj.Condition	Peak	Test Result	Injection
**Number of executed test cases:**	**n.a.**		**Total Result:**	**Passed**	

Chromeleon (c) Dionex
Version 7.2.10.25868

Chromatogram

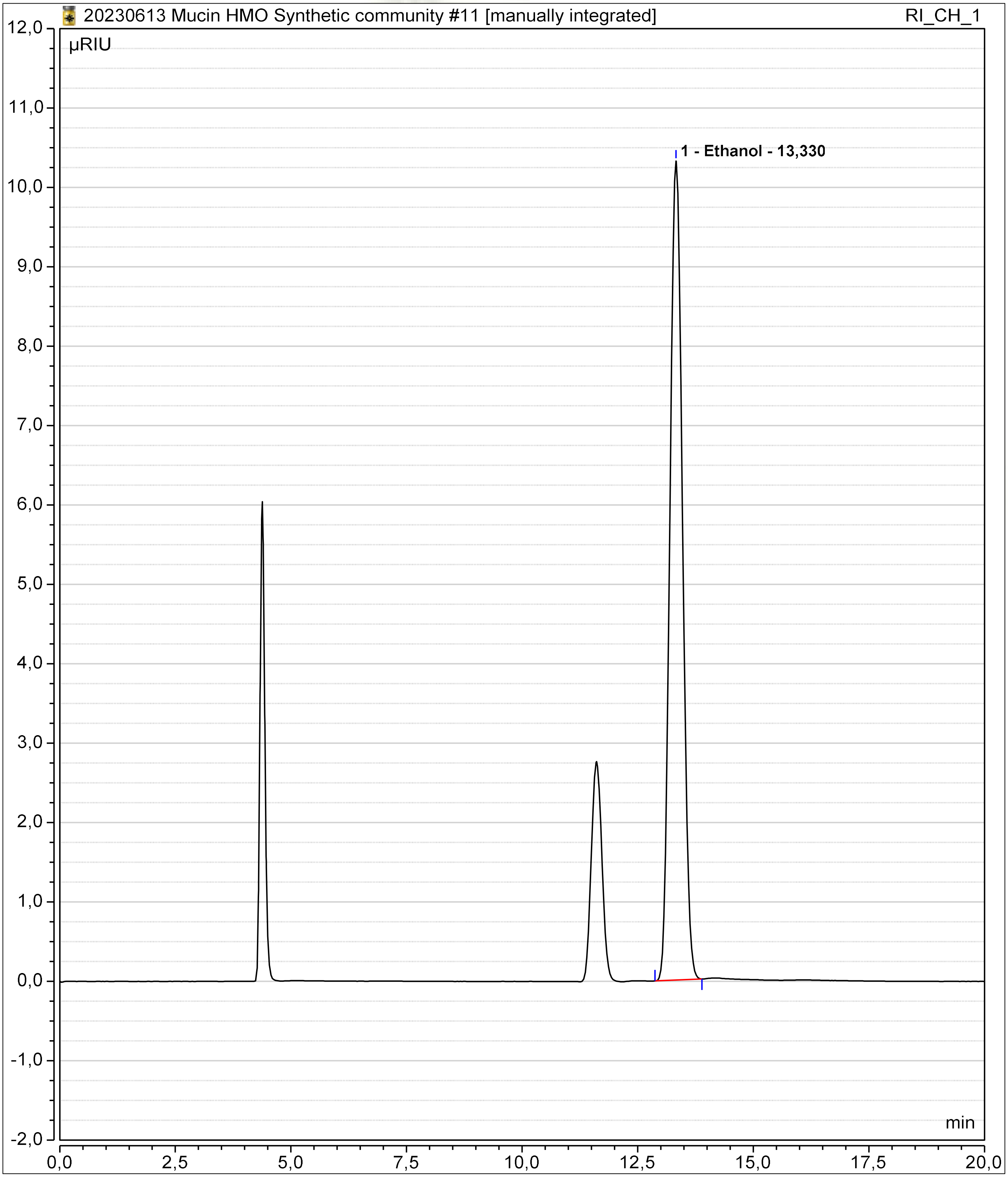

Chromeleon (c) Dionex
Version 7.2.10.25868

Chromatogram and Results

Injection Details

Injection Name:	**16.93 acetate**	*Run Time (min):*	**20,00**
Vial Number:	**3:A5**	*Injection Volume:*	**10,00**
Injection Type:	**Unknown**	*Channel:*	**RI_CH_1**
Calibration Level:		*Wavelength:*	**n.a.**
Instrument Method:	**Default method LC2030C 45 gr 20 min**	*Bandwidth:*	**n.a.**
Processing Method:	**Processing Method LC2030 45 gr**	*Dilution Factor:*	**1,0000**
Injection Date/Time:	**13-jun-23 15:41**	*Sample Weight:*	**1,0000**

Chromatogram

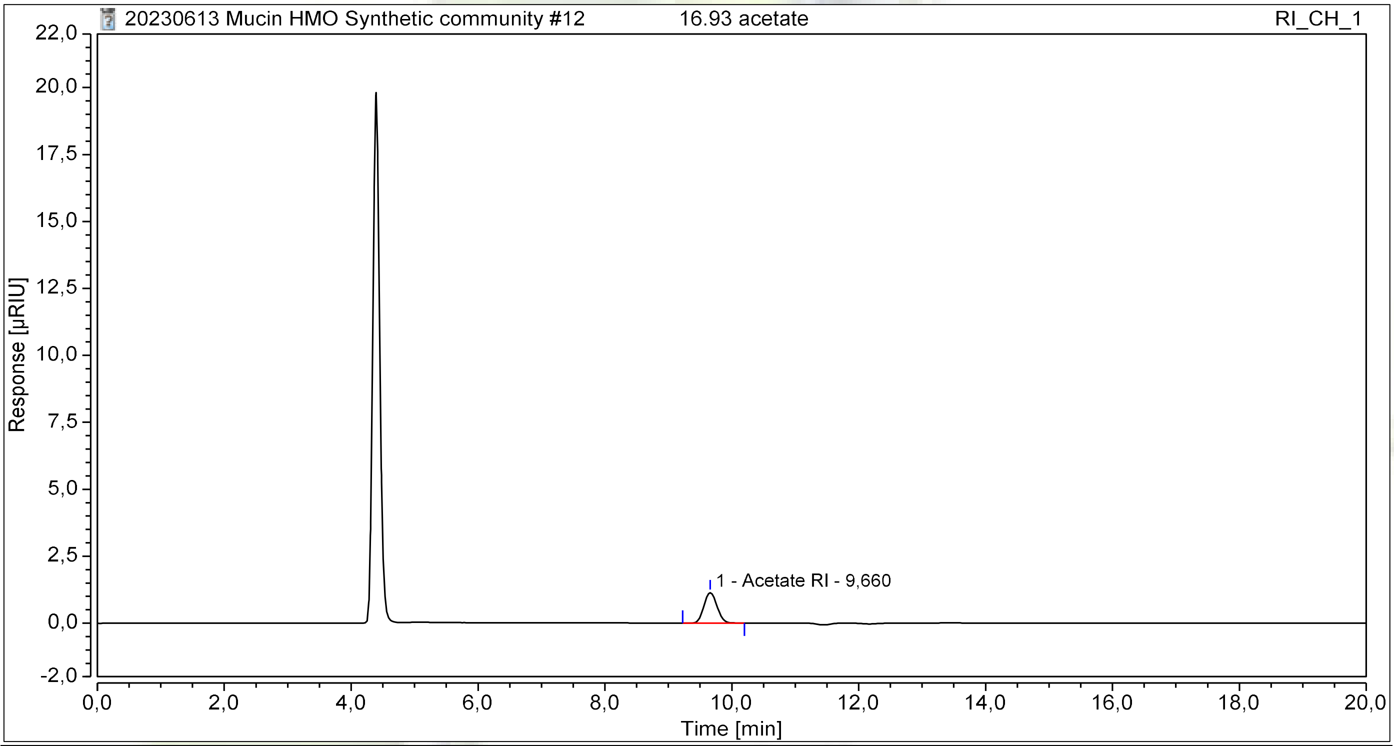

Integration Results

No.	Peak Name	Retention Time min	Area µRIU*min	Height µRIU	Relative Area %	Relative Height %	Amount
n.a.	GlcNAc	n.a.	n.a.	n.a.	n.a.	n.a.	n.a.
n.a.	Citrate	n.a.	n.a.	n.a.	n.a.	n.a.	n.a.
n.a.	Glucose	n.a.	n.a.	n.a.	n.a.	n.a.	n.a.
n.a.	Galactose	n.a.	n.a.	n.a.	n.a.	n.a.	n.a.
n.a.	Fucose	n.a.	n.a.	n.a.	n.a.	n.a.	n.a.
n.a.	Succinate RI	n.a.	n.a.	n.a.	n.a.	n.a.	n.a.
n.a.	Lactate RI	n.a.	n.a.	n.a.	n.a.	n.a.	n.a.
n.a.	glycerol	n.a.	n.a.	n.a.	n.a.	n.a.	n.a.
n.a.	Formate RI	n.a.	n.a.	n.a.	n.a.	n.a.	n.a.
1	Acetate RI	9,660	0,272	1,137	100,00	100,00	16,7091
n.a.	1,2 PDO RI	n.a.	n.a.	n.a.	n.a.	n.a.	n.a.
n.a.	1,3-PDO	n.a.	n.a.	n.a.	n.a.	n.a.	n.a.
n.a.	Propionate RI	n.a.	n.a.	n.a.	n.a.	n.a.	n.a.
n.a.	1,3-PDO	n.a.	n.a.	n.a.	n.a.	n.a.	n.a.
n.a.	2-3 BDO	n.a.	n.a.	n.a.	n.a.	n.a.	n.a.
n.a.	Ethanol	n.a.	n.a.	n.a.	n.a.	n.a.	n.a.
n.a.	Isobutyrate RI	n.a.	n.a.	n.a.	n.a.	n.a.	n.a.
n.a.	Butyrate RI	n.a.	n.a.	n.a.	n.a.	n.a.	n.a.
**Total:**			**0,272**	**1,137**	**100,00**	**100,00**	

Chromeleon (c) Dionex
Version 7.2.10.25868

Peak Analysis

Injection Details

Injection Name:	**16.93 acetate**	*Run Time (min):*	**20,00**
Vial Number:	**3:A5**	*Injection Volume:*	**10,00**
Injection Type:	**Unknown**	*Channel:*	**RI_CH_1**
Calibration Level:		*Wavelength:*	**n.a.**
Instrument Method:	**Default method LC2030C 45 gr 20 min**	*Bandwidth:*	**n.a.**
Processing Method:	**Processing Method LC2030 45 gr**	*Dilution Factor:*	**1,0000**
Injection Date/Time:	**13-jun-23 15:41**	*Sample Weight:*	**1,0000**

Chromatogram

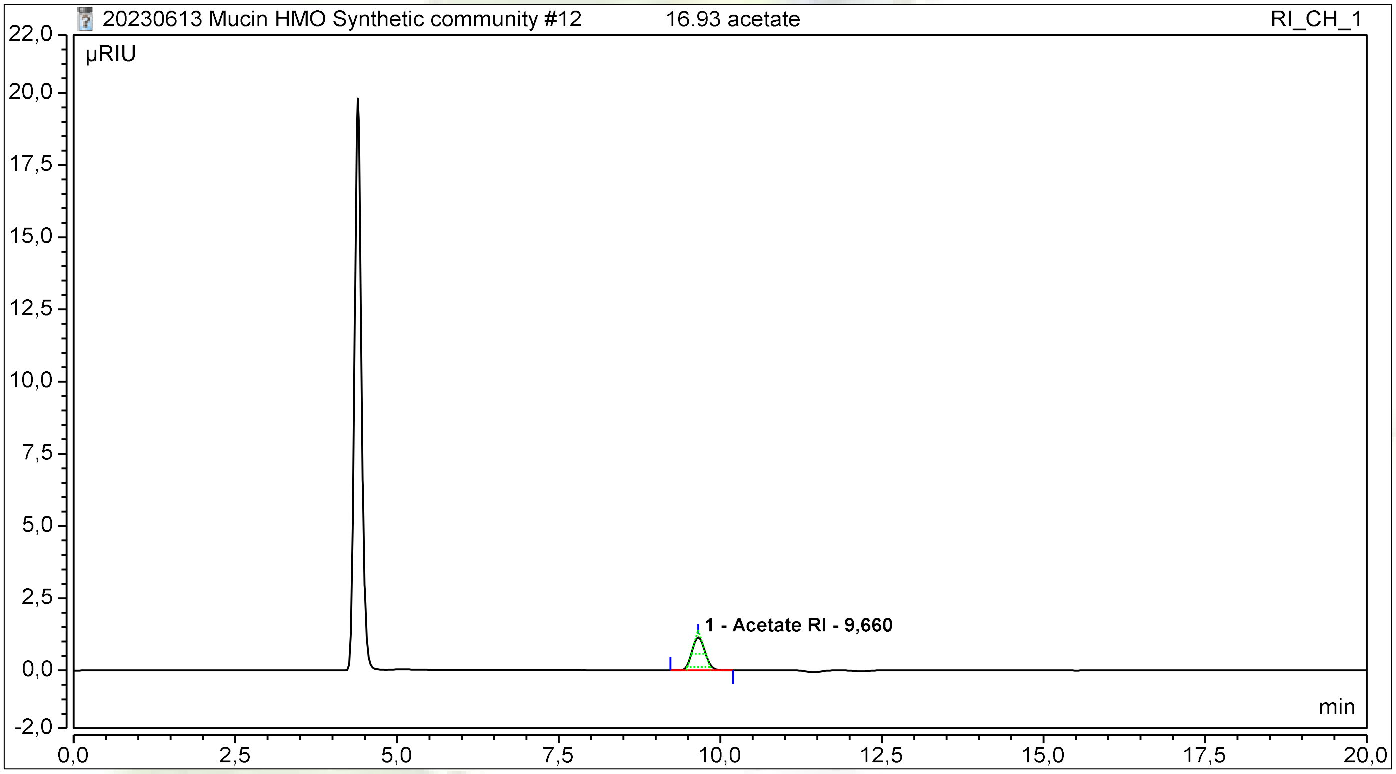

Peak Results

No.	Peak Name	Retention Time min	Width (50%) min	Type	Resolution (EP)	Asymmetry (EP)	Plates (EP)
n.a.	GlcNAc	n.a.	n.a.	n.a.	n.a.	n.a.	n.a.
n.a.	Citrate	n.a.	n.a.	n.a.	n.a.	n.a.	n.a.
n.a.	Glucose	n.a.	n.a.	n.a.	n.a.	n.a.	n.a.
n.a.	Galactose	n.a.	n.a.	n.a.	n.a.	n.a.	n.a.
n.a.	Fucose	n.a.	n.a.	n.a.	n.a.	n.a.	n.a.
n.a.	Succinate RI	n.a.	n.a.	n.a.	n.a.	n.a.	n.a.
n.a.	Lactate RI	n.a.	n.a.	n.a.	n.a.	n.a.	n.a.
n.a.	glycerol	n.a.	n.a.	n.a.	n.a.	n.a.	n.a.
n.a.	Formate RI	n.a.	n.a.	n.a.	n.a.	n.a.	n.a.
1	Acetate RI	9,660	0,226	BMB	n.a.	1,09	10161
n.a.	1,2 PDO RI	n.a.	n.a.	n.a.	n.a.	n.a.	n.a.
n.a.	1,3-PDO	n.a.	n.a.	n.a.	n.a.	n.a.	n.a.
n.a.	Propionate RI	n.a.	n.a.	n.a.	n.a.	n.a.	n.a.
n.a.	1,3-PDO	n.a.	n.a.	n.a.	n.a.	n.a.	n.a.
n.a.	2-3 BDO	n.a.	n.a.	n.a.	n.a.	n.a.	n.a.
n.a.	Ethanol	n.a.	n.a.	n.a.	n.a.	n.a.	n.a.
n.a.	Isobutyrate RI	n.a.	n.a.	n.a.	n.a.	n.a.	n.a.
n.a.	Butyrate RI	n.a.	n.a.	n.a.	n.a.	n.a.	n.a.

Chromatogram and SST Results

Injection Details

Injection Name:	**16.93 acetate**	*Run Time (min):*	**20,00**
Vial Number:	**3:A5**	*Injection Volume:*	**10,00**
Injection Type:	**Unknown**	*Channel:*	**RI_CH_1**
Calibration Level:		*Wavelength:*	**n.a.**
Instrument Method:	**Default method LC2030C 45 gr 20 min**	*Bandwidth:*	**n.a.**
Processing Method:	**Processing Method LC2030 45 gr**	*Dilution Factor:*	**1,0000**
Injection Date/Time:	**13-jun-23 15:41**	*Sample Weight:*	**1,0000**

Chromatogram

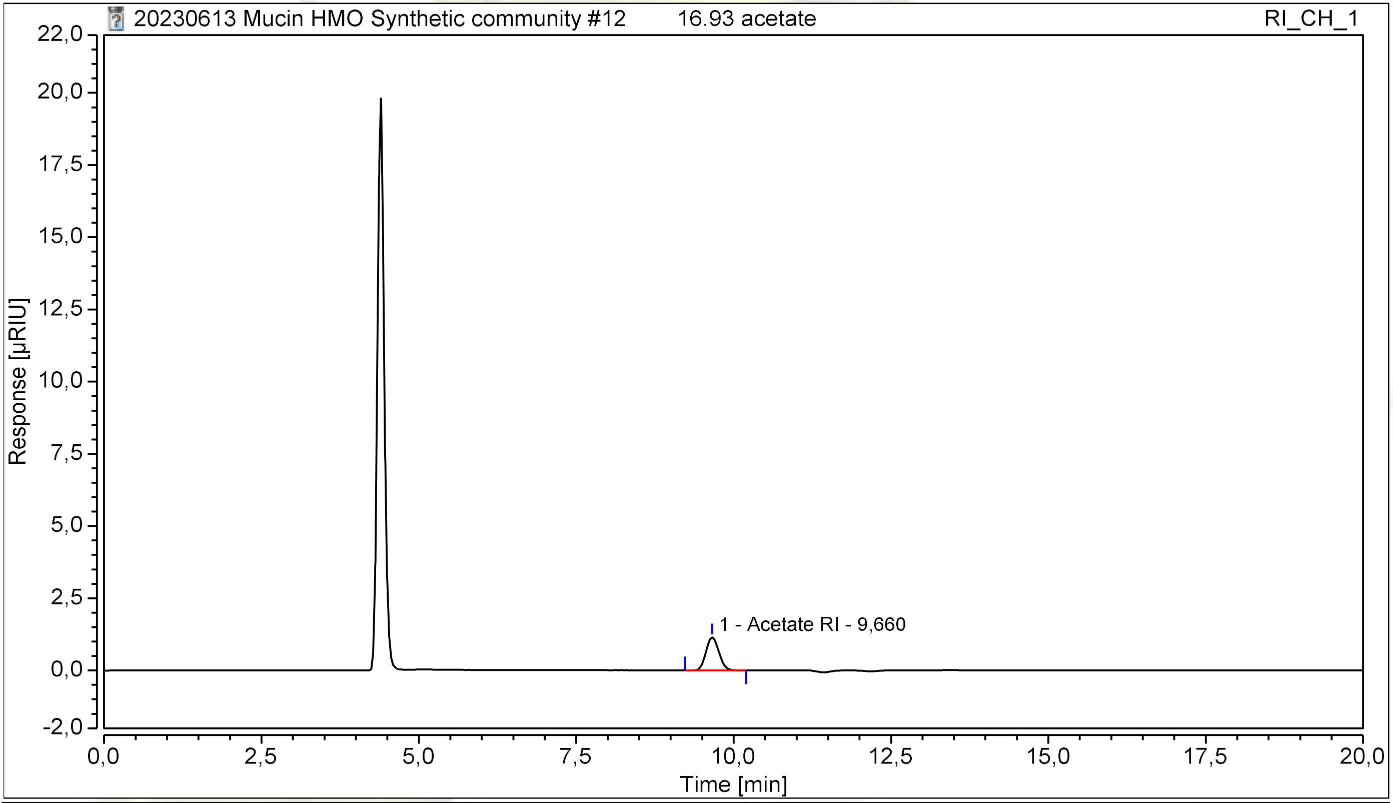

SST Results

No.	Name	Inj.Condition	Peak	Test Result	Injection

Number of executed test cases: **n.a.** **Total Result:** **Passed**

Chromeleon (c) Dionex
Version 7.2.10.25868

Chromatogram

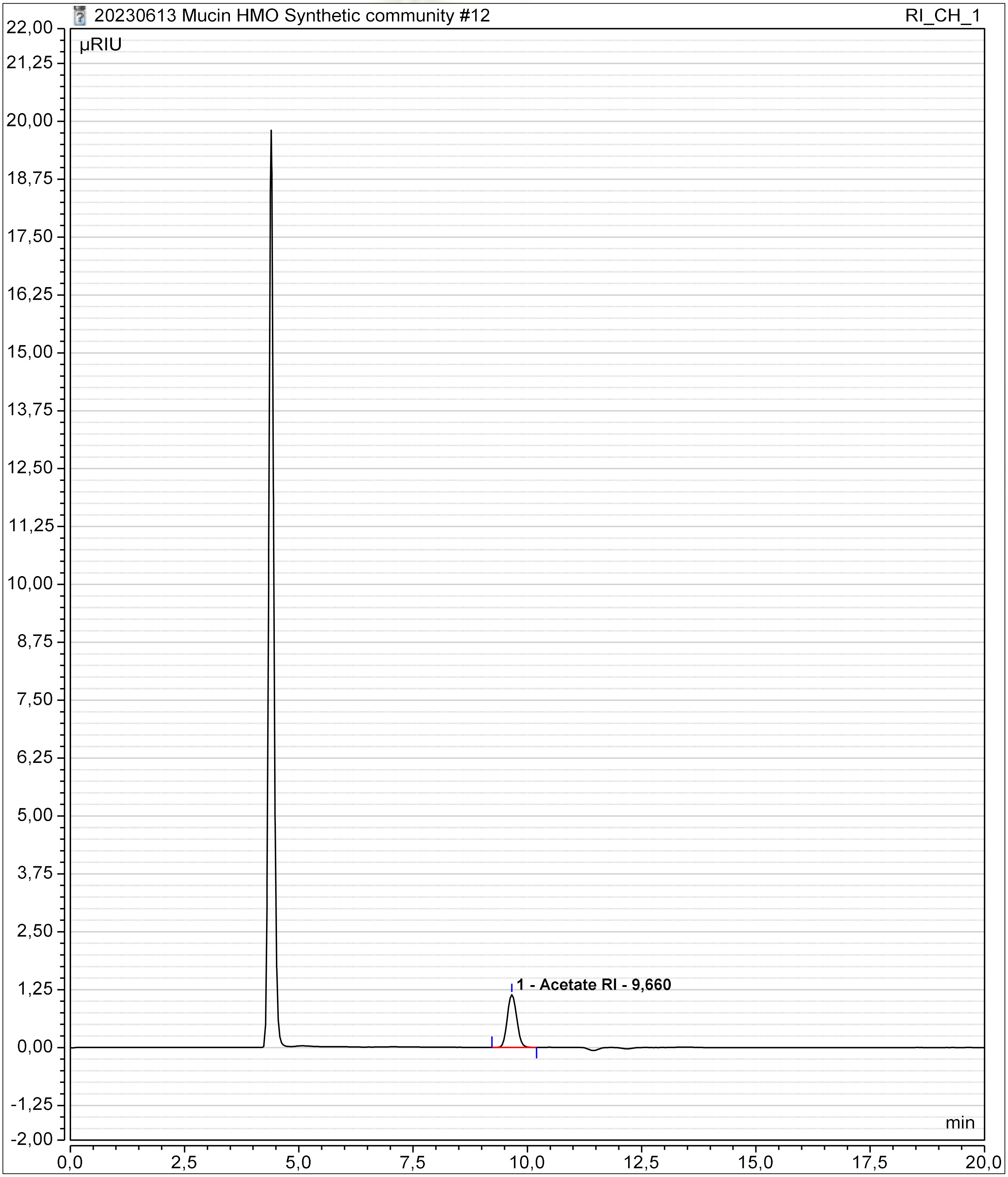

Chromatogram and Results

Injection Details

Injection Name:	**13.68 propionate**	*Run Time (min):*	**20,00**
Vial Number:	**3:A6**	*Injection Volume:*	**10,00**
Injection Type:	**Unknown**	*Channel:*	**RI_CH_1**
Calibration Level:		*Wavelength:*	**n.a.**
Instrument Method:	**Default method LC2030C 45 gr 20 min**	*Bandwidth:*	**n.a.**
Processing Method:	**Processing Method LC2030 45 gr**	*Dilution Factor:*	**1,0000**
Injection Date/Time:	**13-jun-23 16:01**	*Sample Weight:*	**1,0000**

Chromatogram

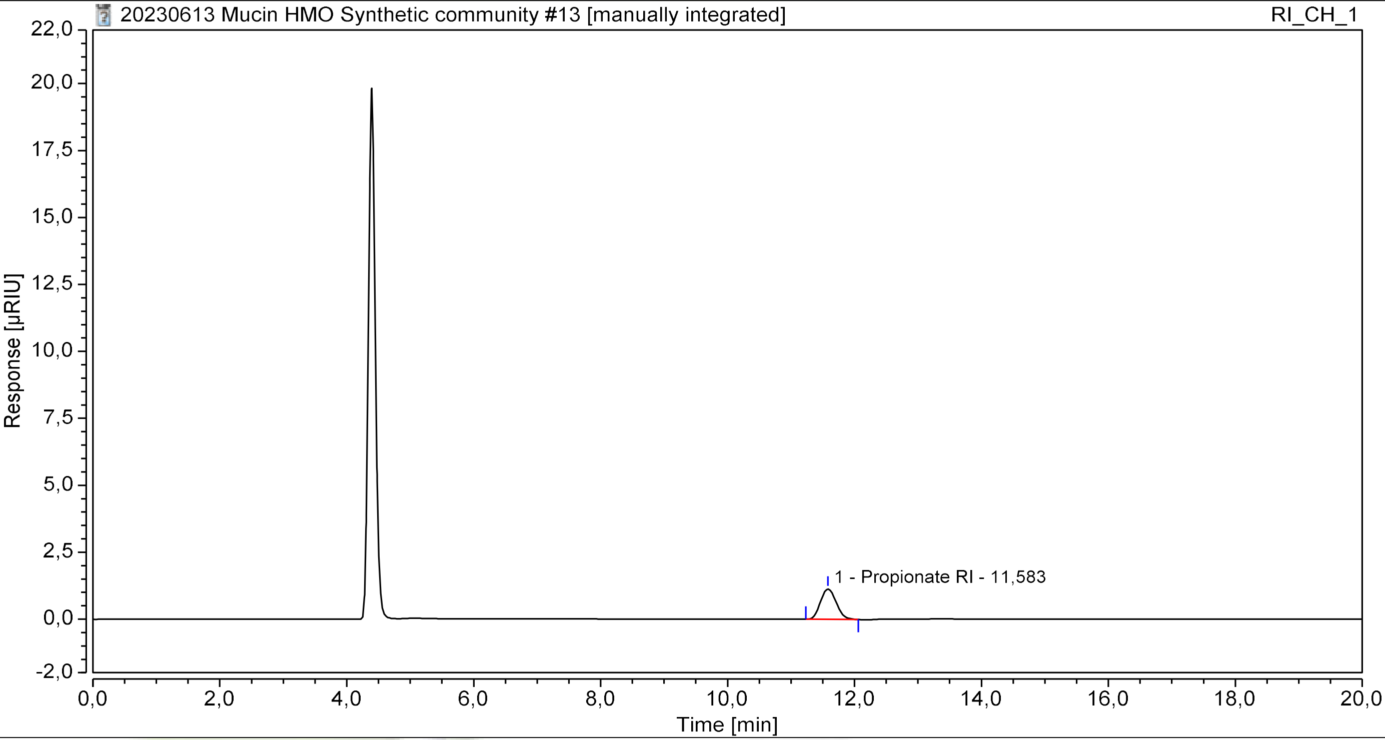

Integration Results

No.	Peak Name	Retention Time min	Area µRIU*min	Height µRIU	Relative Area %	Relative Height %	Amount
n.a.	GlcNAc	n.a.	n.a.	n.a.	n.a.	n.a.	n.a.
n.a.	Citrate	n.a.	n.a.	n.a.	n.a.	n.a.	n.a.
n.a.	Glucose	n.a.	n.a.	n.a.	n.a.	n.a.	n.a.
n.a.	Galactose	n.a.	n.a.	n.a.	n.a.	n.a.	n.a.
n.a.	Fucose	n.a.	n.a.	n.a.	n.a.	n.a.	n.a.
n.a.	Succinate RI	n.a.	n.a.	n.a.	n.a.	n.a.	n.a.
n.a.	Lactate RI	n.a.	n.a.	n.a.	n.a.	n.a.	n.a.
n.a.	glycerol	n.a.	n.a.	n.a.	n.a.	n.a.	n.a.
n.a.	Formate RI	n.a.	n.a.	n.a.	n.a.	n.a.	n.a.
n.a.	Acetate RI	n.a.	n.a.	n.a.	n.a.	n.a.	n.a.
n.a.	1,2 PDO RI	n.a.	n.a.	n.a.	n.a.	n.a.	n.a.
n.a.	1,3-PDO	n.a.	n.a.	n.a.	n.a.	n.a.	n.a.
1	Propionate RI	11,583	0,327	1,134	100,00	100,00	13,1602
n.a.	1,3-PDO	n.a.	n.a.	n.a.	n.a.	n.a.	n.a.
n.a.	2-3 BDO	n.a.	n.a.	n.a.	n.a.	n.a.	n.a.
n.a.	Ethanol	n.a.	n.a.	n.a.	n.a.	n.a.	n.a.
n.a.	Isobutyrate RI	n.a.	n.a.	n.a.	n.a.	n.a.	n.a.
n.a.	Butyrate RI	n.a.	n.a.	n.a.	n.a.	n.a.	n.a.
**Total:**			**0,327**	**1,134**	**100,00**	**100,00**	

Peak Analysis

Injection Details

Injection Name:	**13.68 propionate**	*Run Time (min):*	**20,00**
Vial Number:	**3:A6**	*Injection Volume:*	**10,00**
Injection Type:	**Unknown**	*Channel:*	**RI_CH_1**
Calibration Level:		*Wavelength:*	**n.a.**
Instrument Method:	**Default method LC2030C 45 gr 20 min**	*Bandwidth:*	**n.a.**
Processing Method:	**Processing Method LC2030 45 gr**	*Dilution Factor:*	**1,0000**
Injection Date/Time:	**13-jun-23 16:01**	*Sample Weight:*	**1,0000**

Chromatogram

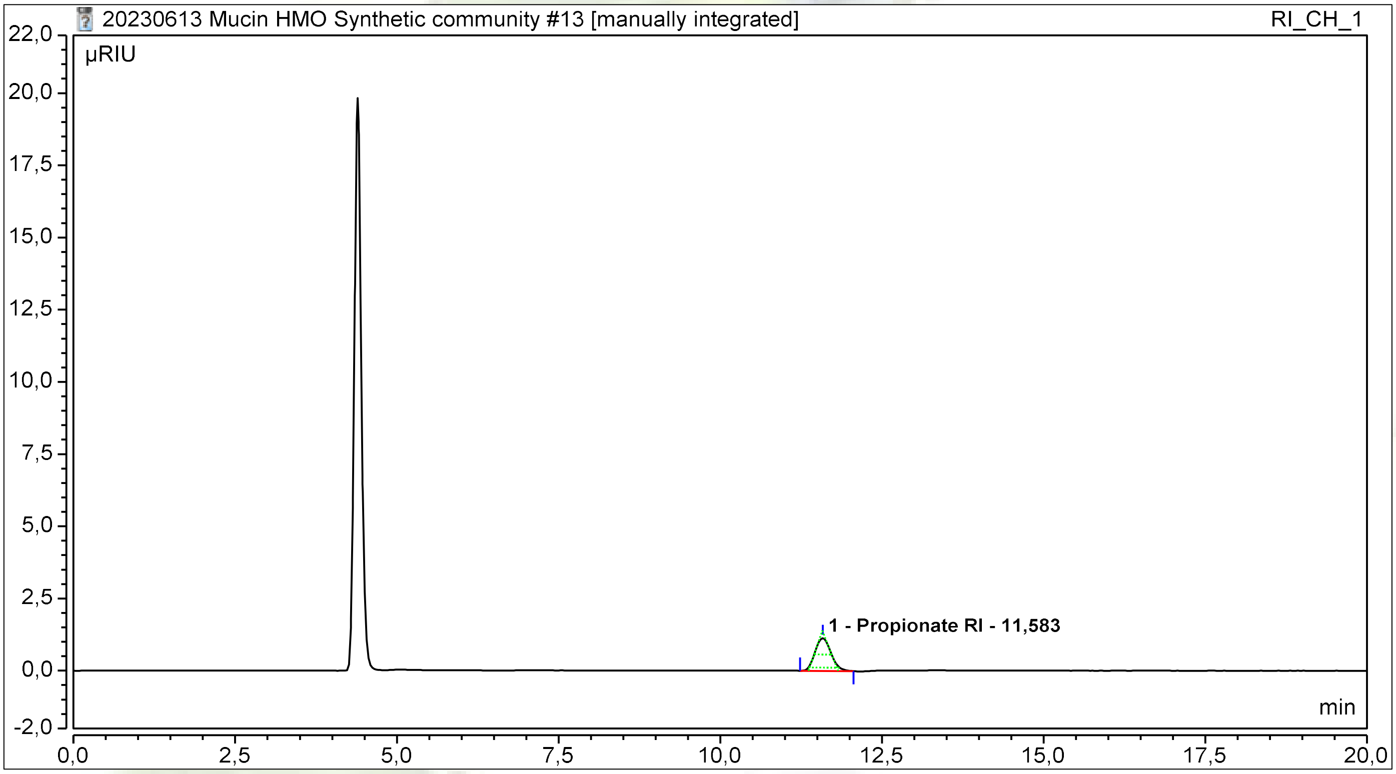

Peak Results

No.	Peak Name	Retention Time min	Width (50%) min	Type	Resolution (EP)	Asymmetry (EP)	Plates (EP)
n.a.	GlcNAc	n.a.	n.a.	n.a.	n.a.	n.a.	n.a.
n.a.	Citrate	n.a.	n.a.	n.a.	n.a.	n.a.	n.a.
n.a.	Glucose	n.a.	n.a.	n.a.	n.a.	n.a.	n.a.
n.a.	Galactose	n.a.	n.a.	n.a.	n.a.	n.a.	n.a.
n.a.	Fucose	n.a.	n.a.	n.a.	n.a.	n.a.	n.a.
n.a.	Succinate RI	n.a.	n.a.	n.a.	n.a.	n.a.	n.a.
n.a.	Lactate RI	n.a.	n.a.	n.a.	n.a.	n.a.	n.a.
n.a.	glycerol	n.a.	n.a.	n.a.	n.a.	n.a.	n.a.
n.a.	Formate RI	n.a.	n.a.	n.a.	n.a.	n.a.	n.a.
n.a.	Acetate RI	n.a.	n.a.	n.a.	n.a.	n.a.	n.a.
n.a.	1,2 PDO RI	n.a.	n.a.	n.a.	n.a.	n.a.	n.a.
n.a.	1,3-PDO	n.a.	n.a.	n.a.	n.a.	n.a.	n.a.
1	Propionate RI	11,583	0,273	BMB*	n.a.	1,12	10001
n.a.	1,3-PDO	n.a.	n.a.	n.a.	n.a.	n.a.	n.a.
n.a.	2-3 BDO	n.a.	n.a.	n.a.	n.a.	n.a.	n.a.
n.a.	Ethanol	n.a.	n.a.	n.a.	n.a.	n.a.	n.a.
n.a.	Isobutyrate RI	n.a.	n.a.	n.a.	n.a.	n.a.	n.a.
n.a.	Butyrate RI	n.a.	n.a.	n.a.	n.a.	n.a.	n.a.

Chromatogram and SST Results

Injection Details

Injection Name:	**13.68 propionate**	*Run Time (min):*	**20,00**
Vial Number:	**3:A6**	*Injection Volume:*	**10,00**
Injection Type:	**Unknown**	*Channel:*	**RI_CH_1**
Calibration Level:		*Wavelength:*	**n.a.**
Instrument Method:	**Default method LC2030C 45 gr 20 min**	*Bandwidth:*	**n.a.**
Processing Method:	**Processing Method LC2030 45 gr**	*Dilution Factor:*	**1,0000**
Injection Date/Time:	**13-jun-23 16:01**	*Sample Weight:*	**1,0000**

Chromatogram

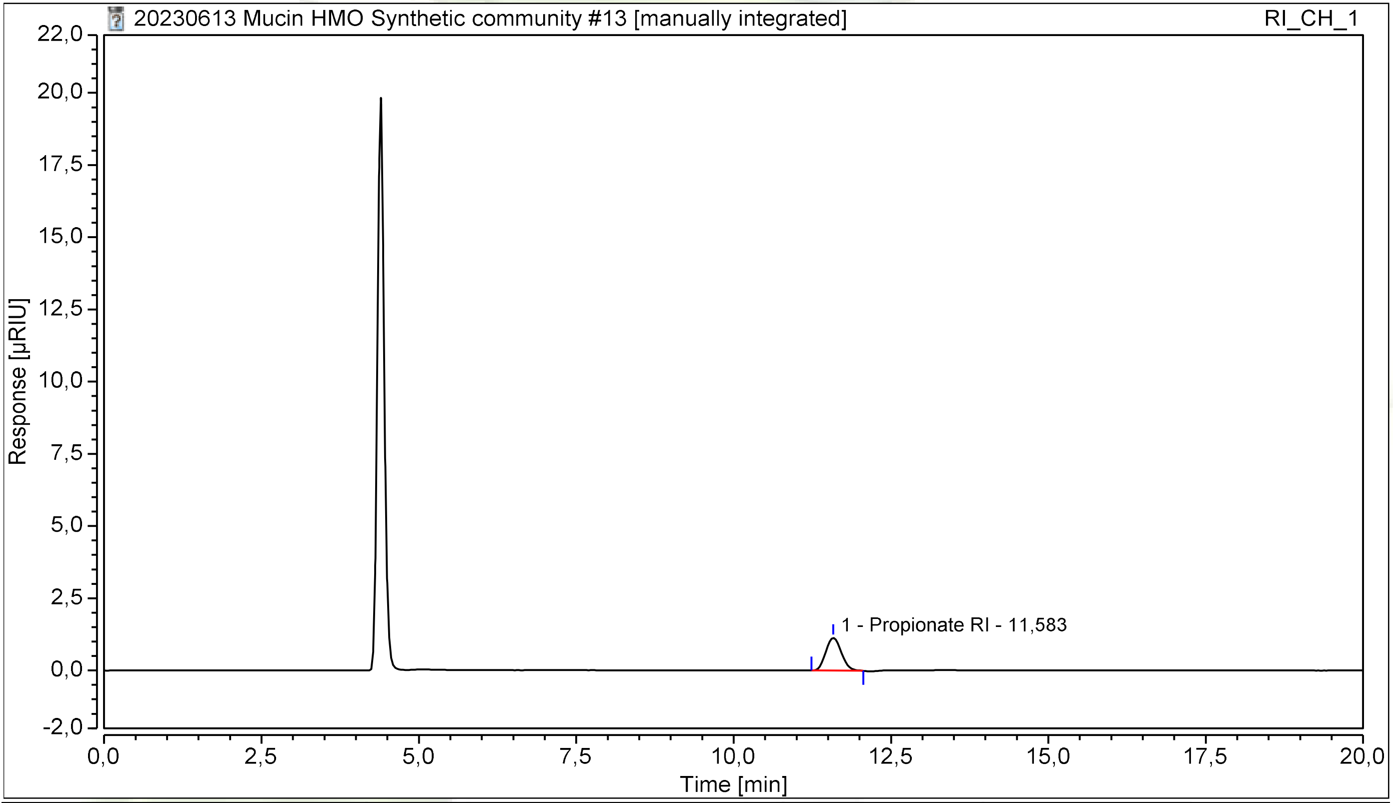

SST Results

No.	Name	Inj.Condition	Peak	Test Result	Injection

Number of executed test cases: **n.a.** **Total Result:** **Passed**

Chromeleon (c) Dionex
Version 7.2.10.25868

Chromatogram

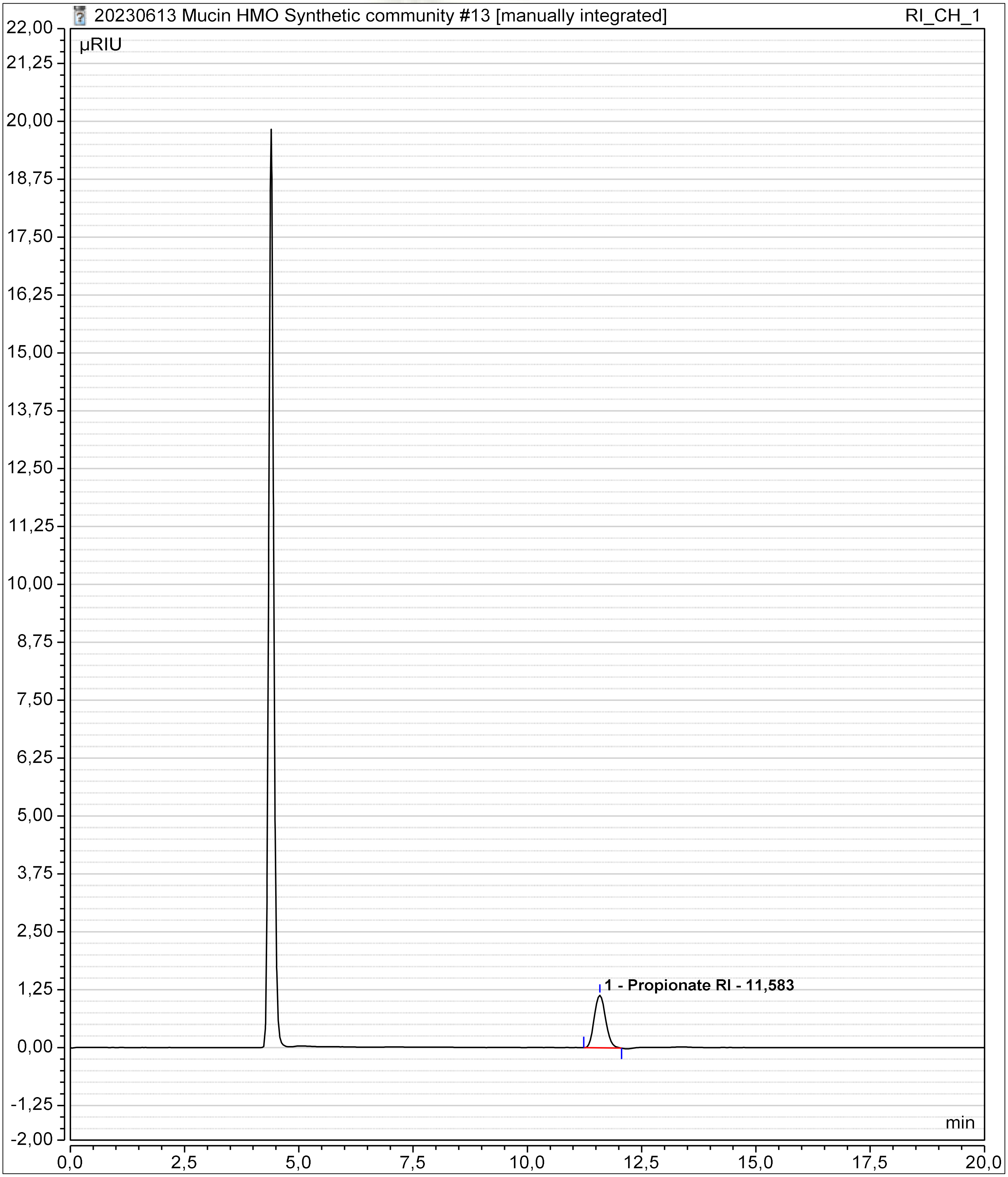

Chromatogram and Results

Injection Details

Injection Name:	**40 5HMO1 t24 r1**	*Run Time (min):*	**20,00**
Vial Number:	**3:A7**	*Injection Volume:*	**10,00**
Injection Type:	**Unknown**	*Channel:*	**RI_CH_1**
Calibration Level:		*Wavelength:*	**n.a.**
Instrument Method:	**Default method LC2030C 45 gr 20 min**	*Bandwidth:*	**n.a.**
Processing Method:	**Processing Method LC2030 45 gr**	*Dilution Factor:*	**1,0000**
Injection Date/Time:	**13-jun-23 16:22**	*Sample Weight:*	**1,0000**

Chromatogram

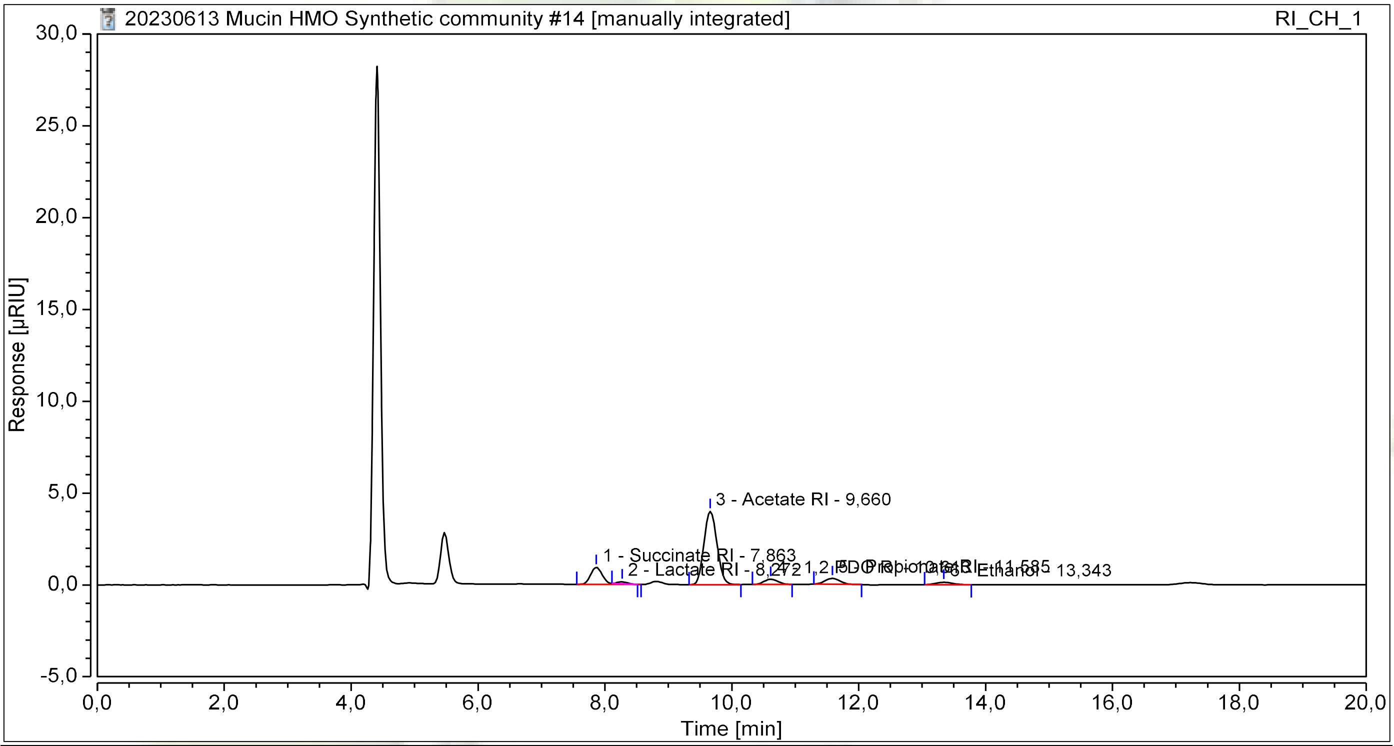

Integration Results

No.	Peak Name	Retention Time<br>min	Area<br>µRIU*min	Height<br>µRIU	Relative Area<br>%	Relative Height<br>%	Amount
n.a.	GlcNAc	n.a.	n.a.	n.a.	n.a.	n.a.	n.a.
n.a.	Citrate	n.a.	n.a.	n.a.	n.a.	n.a.	n.a.
n.a.	Glucose	n.a.	n.a.	n.a.	n.a.	n.a.	n.a.
n.a.	Galactose	n.a.	n.a.	n.a.	n.a.	n.a.	n.a.
n.a.	Fucose	n.a.	n.a.	n.a.	n.a.	n.a.	n.a.
1	Succinate RI	7,863	0,210	0,925	15,19	16,08	n.a.
2	Lactate RI	8,272	0,020	0,111	1,45	1,93	0,5831
n.a.	glycerol	n.a.	n.a.	n.a.	n.a.	n.a.	n.a.
n.a.	Formate RI	n.a.	n.a.	n.a.	n.a.	n.a.	n.a.
3	Acetate RI	9,660	0,950	3,990	68,85	69,37	58,4970
4	1,2 PDO RI	10,613	0,074	0,282	5,34	4,90	2,1914
n.a.	1,3-PDO	n.a.	n.a.	n.a.	n.a.	n.a.	n.a.
5	Propionate RI	11,585	0,086	0,312	6,21	5,42	3,4489
n.a.	1,3-PDO	n.a.	n.a.	n.a.	n.a.	n.a.	n.a.
n.a.	2-3 BDO	n.a.	n.a.	n.a.	n.a.	n.a.	n.a.
6	Ethanol	13,343	0,041	0,132	2,96	2,30	0,3758
n.a.	Isobutyrate RI	n.a.	n.a.	n.a.	n.a.	n.a.	n.a.
n.a.	Butyrate RI	n.a.	n.a.	n.a.	n.a.	n.a.	n.a.
**Total:**			**1,380**	**5,751**	**100,00**	**100,00**	

Peak Analysis

Injection Details

Injection Name:	**40 5HMO1 t24 r1**	*Run Time (min):*	**20,00**
Vial Number:	**3:A7**	*Injection Volume:*	**10,00**
Injection Type:	**Unknown**	*Channel:*	**RI_CH_1**
Calibration Level:		*Wavelength:*	**n.a.**
Instrument Method:	**Default method LC2030C 45 gr 20 min**	*Bandwidth:*	**n.a.**
Processing Method:	**Processing Method LC2030 45 gr**	*Dilution Factor:*	**1,0000**
Injection Date/Time:	**13-jun-23 16:22**	*Sample Weight:*	**1,0000**

Chromatogram

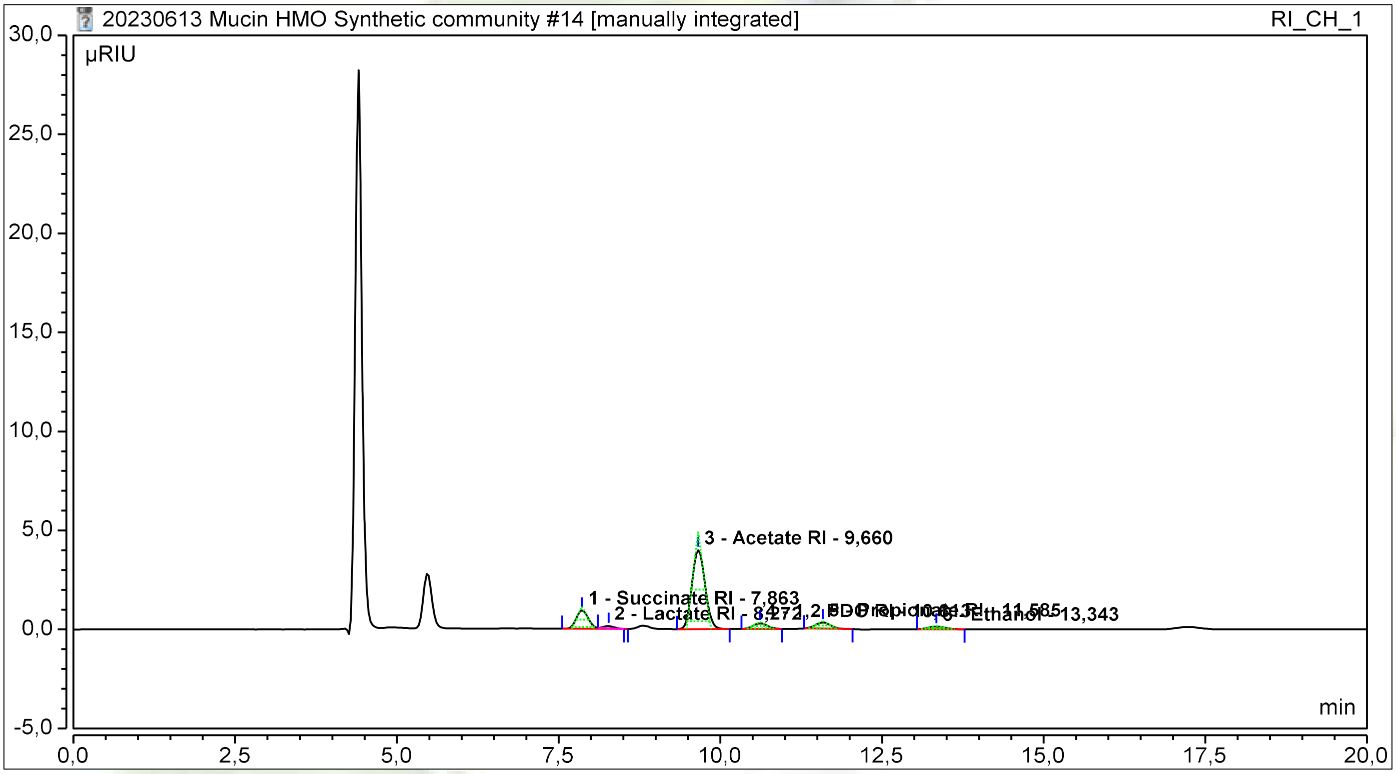

Peak Results

No.	Peak Name	Retention Time<br>min	Width (50%)<br>min	Type	Resolution (EP)	Asymmetry (EP)	Plates (EP)
n.a.	GlcNAc	n.a.	n.a.	n.a.	n.a.	n.a.	n.a.
n.a.	Citrate	n.a.	n.a.	n.a.	n.a.	n.a.	n.a.
n.a.	Glucose	n.a.	n.a.	n.a.	n.a.	n.a.	n.a.
n.a.	Galactose	n.a.	n.a.	n.a.	n.a.	n.a.	n.a.
n.a.	Fucose	n.a.	n.a.	n.a.	n.a.	n.a.	n.a.
1	Succinate RI	7,863	0,203	BMB	4,95	1,26	8337
2	Lactate RI	8,272	n.a.	Rd	n.a.	n.a.	n.a.
n.a.	glycerol	n.a.	n.a.	n.a.	n.a.	n.a.	n.a.
n.a.	Formate RI	n.a.	n.a.	n.a.	n.a.	n.a.	n.a.
3	Acetate RI	9,660	0,225	BMB	2,37	1,09	10185
4	1,2 PDO RI	10,613	0,250	BMB*	2,23	1,08	10022
n.a.	1,3-PDO	n.a.	n.a.	n.a.	n.a.	n.a.	n.a.
5	Propionate RI	11,585	0,264	BMB*	3,70	1,03	10684
n.a.	1,3-PDO	n.a.	n.a.	n.a.	n.a.	n.a.	n.a.
n.a.	2-3 BDO	n.a.	n.a.	n.a.	n.a.	n.a.	n.a.
6	Ethanol	13,343	0,297	BMB*	n.a.	1,09	11168
n.a.	Isobutyrate RI	n.a.	n.a.	n.a.	n.a.	n.a.	n.a.
n.a.	Butyrate RI	n.a.	n.a.	n.a.	n.a.	n.a.	n.a.

Chromatogram and SST Results

Injection Details

Injection Name:	**40 5HMO1 t24 r1**	*Run Time (min):*	**20,00**
Vial Number:	**3:A7**	*Injection Volume:*	**10,00**
Injection Type:	**Unknown**	*Channel:*	**RI_CH_1**
Calibration Level:		*Wavelength:*	**n.a.**
Instrument Method:	**Default method LC2030C 45 gr 20 min**	*Bandwidth:*	**n.a.**
Processing Method:	**Processing Method LC2030 45 gr**	*Dilution Factor:*	**1,0000**
Injection Date/Time:	**13-jun-23 16:22**	*Sample Weight:*	**1,0000**

Chromatogram

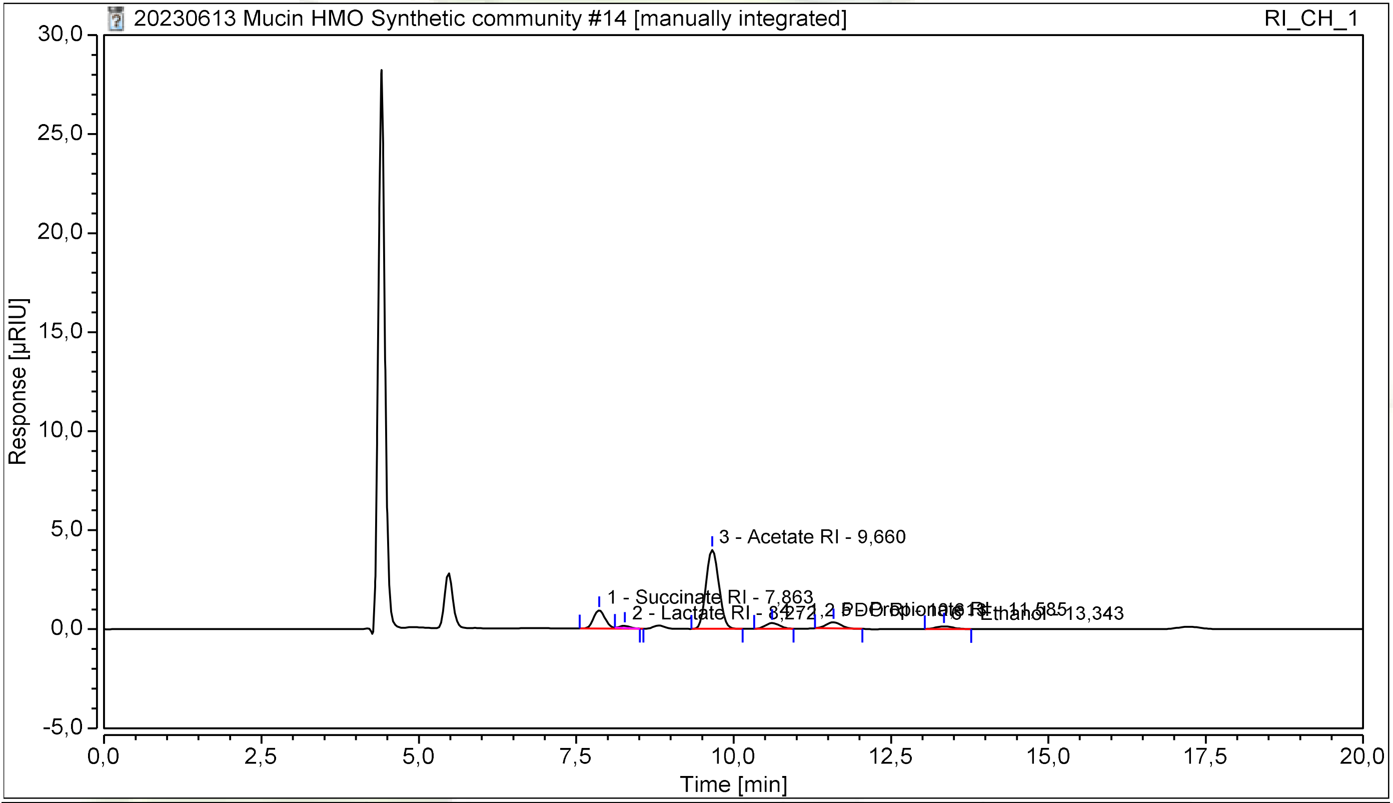

SST Results

No.	Name	Inj.Condition	Peak	Test Result	Injection

Number of executed test cases: **n.a.** **Total Result:** **Passed**

Chromeleon (c) Dionex
Version 7.2.10.25868

Chromatogram

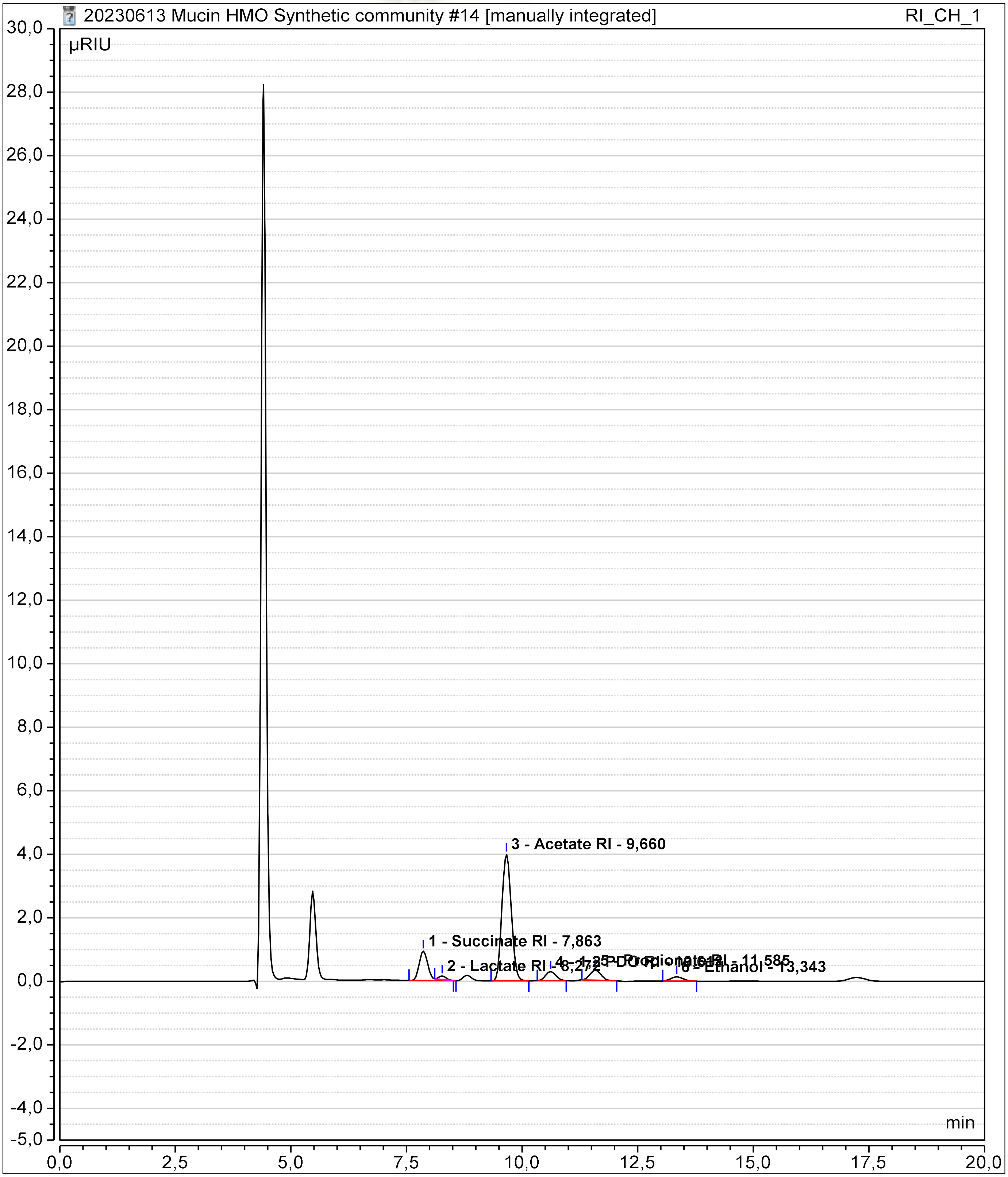

Chromeleon (c) Dionex
Version 7.2.10.25868

Chromatogram and Results

Injection Details

Injection Name:	**41 5HMO1 t24 r2**	*Run Time (min):*	**20,00**
Vial Number:	**3:A8**	*Injection Volume:*	**10,00**
Injection Type:	**Unknown**	*Channel:*	**RI_CH_1**
Calibration Level:		*Wavelength:*	**n.a.**
Instrument Method:	**Default method LC2030C 45 gr 20 min**	*Bandwidth:*	**n.a.**
Processing Method:	**Processing Method LC2030 45 gr**	*Dilution Factor:*	**1,0000**
Injection Date/Time:	**13-jun-23 16:42**	*Sample Weight:*	**1,0000**

Chromatogram

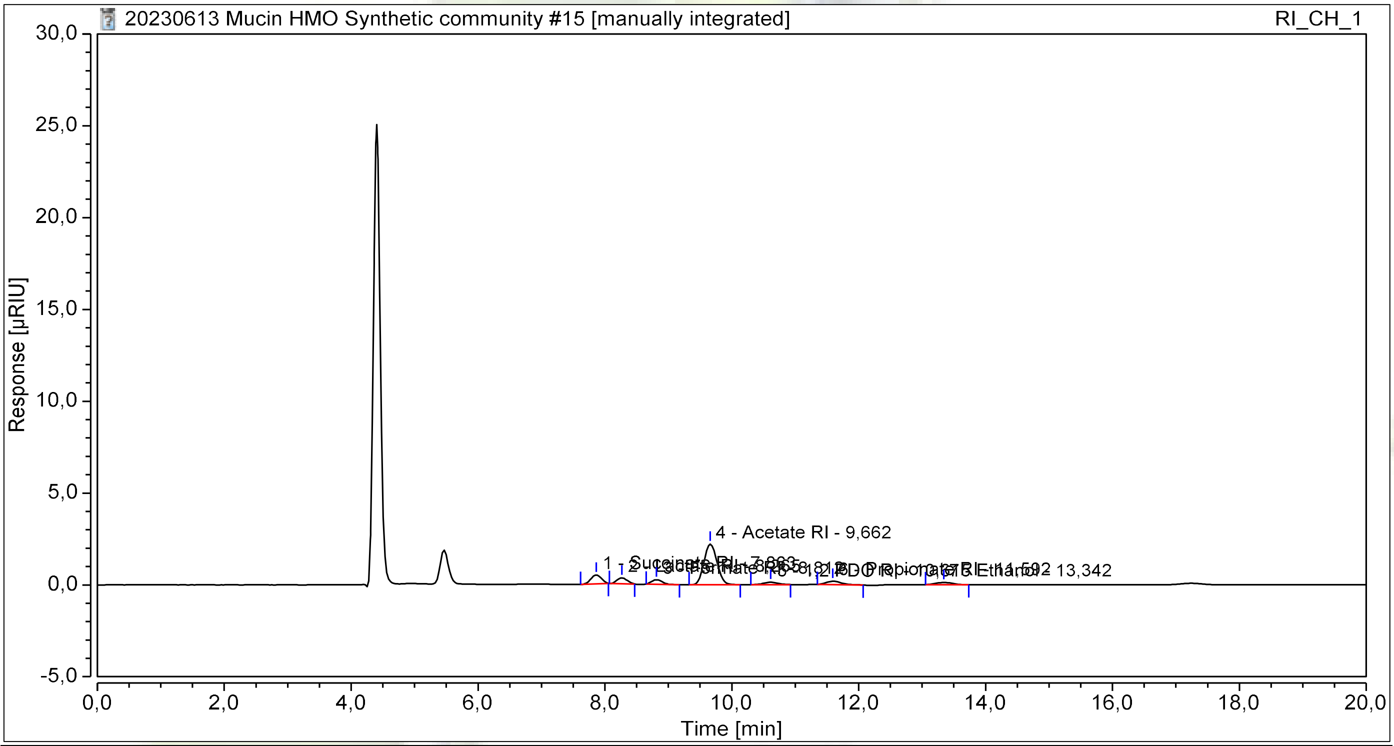

Integration Results

No.	Peak Name	Retention Time min	Area µRIU*min	Height µRIU	Relative Area %	Relative Height %	Amount
n.a.	GlcNAc	n.a.	n.a.	n.a.	n.a.	n.a.	n.a.
n.a.	Citrate	n.a.	n.a.	n.a.	n.a.	n.a.	n.a.
n.a.	Glucose	n.a.	n.a.	n.a.	n.a.	n.a.	n.a.
n.a.	Galactose	n.a.	n.a.	n.a.	n.a.	n.a.	n.a.
n.a.	Fucose	n.a.	n.a.	n.a.	n.a.	n.a.	n.a.
1	Succinate RI	7,863	0,093	0,476	11,00	12,94	n.a.
2	Lactate RI	8,265	0,060	0,315	7,06	8,56	1,7376
n.a.	glycerol	n.a.	n.a.	n.a.	n.a.	n.a.	n.a.
3	Formate RI	8,812	0,046	0,242	5,46	6,59	4,8521
4	Acetate RI	9,662	0,525	2,207	61,97	60,05	32,3148
5	1,2 PDO RI	10,615	0,033	0,127	3,88	3,46	0,9788
n.a.	1,3-PDO	n.a.	n.a.	n.a.	n.a.	n.a.	n.a.
6	Propionate RI	11,592	0,053	0,187	6,27	5,10	2,1360
n.a.	1,3-PDO	n.a.	n.a.	n.a.	n.a.	n.a.	n.a.
n.a.	2-3 BDO	n.a.	n.a.	n.a.	n.a.	n.a.	n.a.
7	Ethanol	13,342	0,037	0,122	4,36	3,31	0,3400
n.a.	Isobutyrate RI	n.a.	n.a.	n.a.	n.a.	n.a.	n.a.
n.a.	Butyrate RI	n.a.	n.a.	n.a.	n.a.	n.a.	n.a.
**Total:**			**0,847**	**3,676**	**100,00**	**100,00**	

Peak Analysis

Injection Details

Injection Name:	**41 5HMO1 t24 r2**	*Run Time (min):*	**20,00**
Vial Number:	**3:A8**	*Injection Volume:*	**10,00**
Injection Type:	**Unknown**	*Channel:*	**RI_CH_1**
Calibration Level:		*Wavelength:*	**n.a.**
Instrument Method:	**Default method LC2030C 45 gr 20 min**	*Bandwidth:*	**n.a.**
Processing Method:	**Processing Method LC2030 45 gr**	*Dilution Factor:*	**1,0000**
Injection Date/Time:	**13-jun-23 16:42**	*Sample Weight:*	**1,0000**

Chromatogram

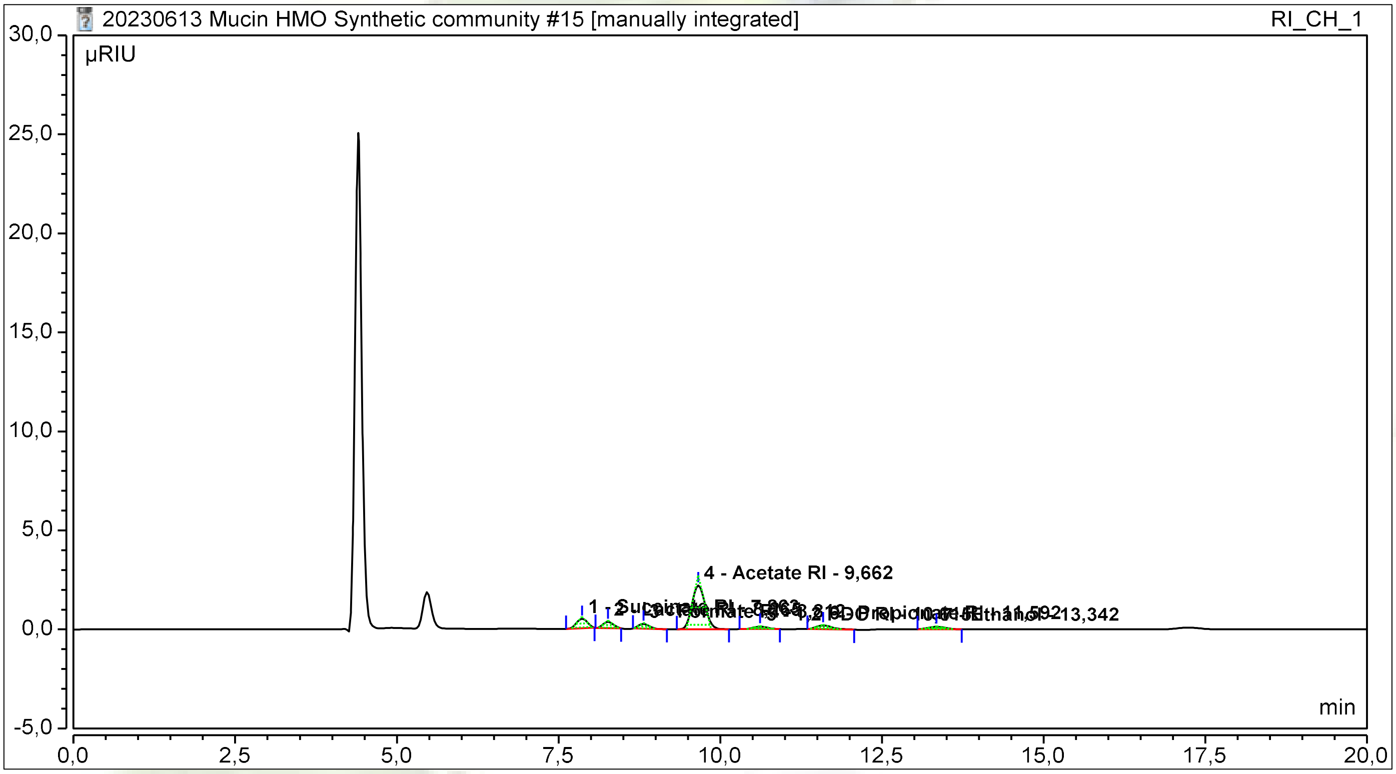

Peak Results

No.	Peak Name	Retention Time min	Width (50%) min	Type	Resolution (EP)	Asymmetry (EP)	Plates (EP)
n.a.	GlcNAc	n.a.	n.a.	n.a.	n.a.	n.a.	n.a.
n.a.	Citrate	n.a.	n.a.	n.a.	n.a.	n.a.	n.a.
n.a.	Glucose	n.a.	n.a.	n.a.	n.a.	n.a.	n.a.
n.a.	Galactose	n.a.	n.a.	n.a.	n.a.	n.a.	n.a.
n.a.	Fucose	n.a.	n.a.	n.a.	n.a.	n.a.	n.a.
1	Succinate RI	7,863	0,191	BMB*	1,25	0,98	9352
2	Lactate RI	8,265	0,187	BMB*	1,72	1,09	10815
n.a.	glycerol	n.a.	n.a.	n.a.	n.a.	n.a.	n.a.
3	Formate RI	8,812	0,188	BMB*	2,43	1,15	12120
4	Acetate RI	9,662	0,225	BMB	2,38	1,08	10208
5	1,2 PDO RI	10,615	0,247	BMB*	2,28	1,05	10215
n.a.	1,3-PDO	n.a.	n.a.	n.a.	n.a.	n.a.	n.a.
6	Propionate RI	11,592	0,259	BMB*	3,73	1,50	11102
n.a.	1,3-PDO	n.a.	n.a.	n.a.	n.a.	n.a.	n.a.
n.a.	2-3 BDO	n.a.	n.a.	n.a.	n.a.	n.a.	n.a.
7	Ethanol	13,342	0,294	BMB*	n.a.	1,08	11400
n.a.	Isobutyrate RI	n.a.	n.a.	n.a.	n.a.	n.a.	n.a.
n.a.	Butyrate RI	n.a.	n.a.	n.a.	n.a.	n.a.	n.a.

Chromeleon (c) Dionex
Version 7.2.10.25868

Chromatogram and SST Results

Injection Details

Injection Name:	**41 5HMO1 t24 r2**	*Run Time (min):*	**20,00**
Vial Number:	**3:A8**	*Injection Volume:*	**10,00**
Injection Type:	**Unknown**	*Channel:*	**RI_CH_1**
Calibration Level:		*Wavelength:*	**n.a.**
Instrument Method:	**Default method LC2030C 45 gr 20 min**	*Bandwidth:*	**n.a.**
Processing Method:	**Processing Method LC2030 45 gr**	*Dilution Factor:*	**1,0000**
Injection Date/Time:	**13-jun-23 16:42**	*Sample Weight:*	**1,0000**

Chromatogram

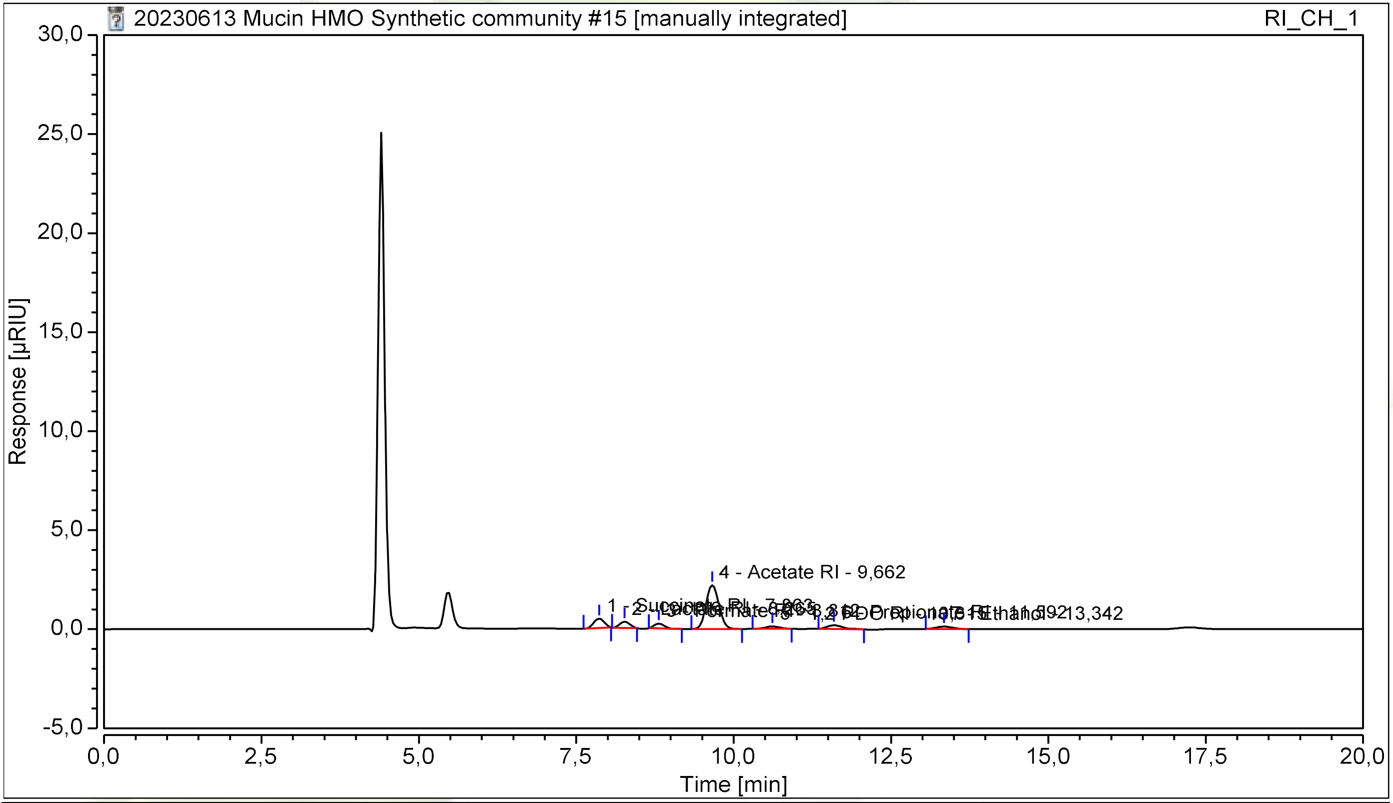

SST Results

No.	Name	Inj.Condition	Peak	Test Result	Injection

Number of executed test cases: **n.a.**   **Total Result:** **Passed**

Chromatogram

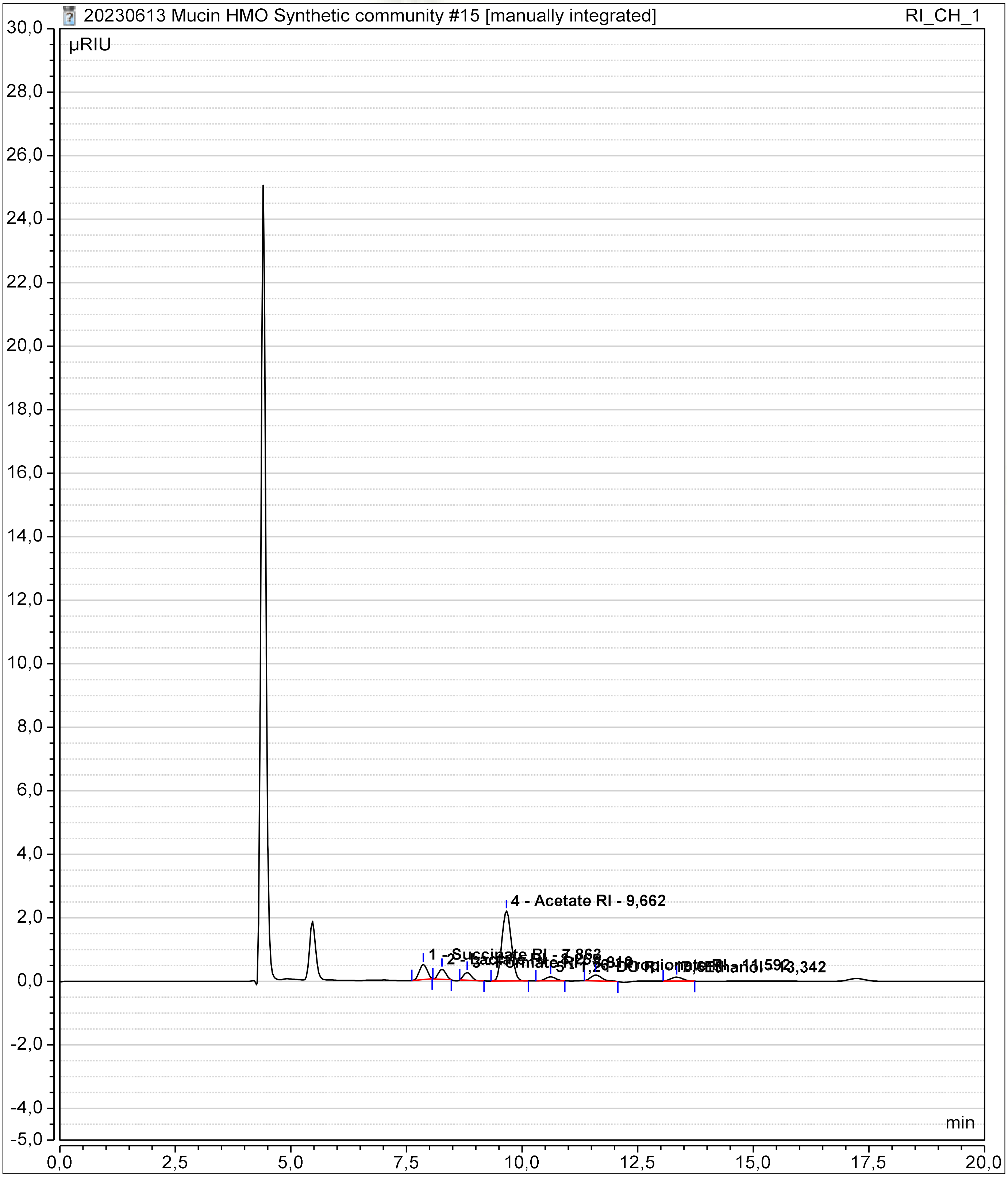

Chromatogram and Results

Injection Details

Injection Name:	**42 5HMO1 t24 r3**	*Run Time (min):*	**20,00**
Vial Number:	**3:A9**	*Injection Volume:*	**10,00**
Injection Type:	**Unknown**	*Channel:*	**RI_CH_1**
Calibration Level:		*Wavelength:*	**n.a.**
Instrument Method:	**Default method LC2030C 45 gr 20 min**	*Bandwidth:*	**n.a.**
Processing Method:	**Processing Method LC2030 45 gr**	*Dilution Factor:*	**1,0000**
Injection Date/Time:	**13-jun-23 17:03**	*Sample Weight:*	**1,0000**

Chromatogram

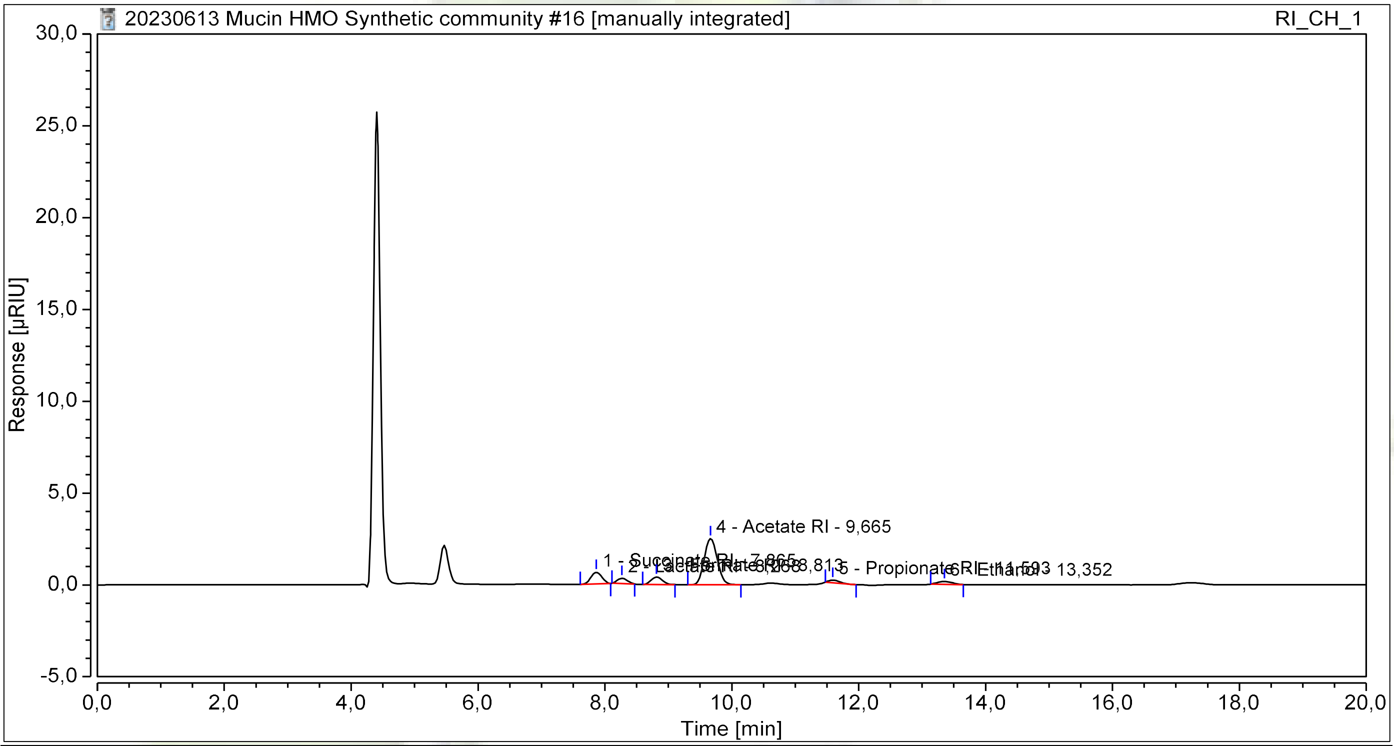

Integration Results

No.	Peak Name	Retention Time min	Area µRIU*min	Height µRIU	Relative Area %	Relative Height %	Amount
n.a.	GlcNAc	n.a.	n.a.	n.a.	n.a.	n.a.	n.a.
n.a.	Citrate	n.a.	n.a.	n.a.	n.a.	n.a.	n.a.
n.a.	Glucose	n.a.	n.a.	n.a.	n.a.	n.a.	n.a.
n.a.	Galactose	n.a.	n.a.	n.a.	n.a.	n.a.	n.a.
n.a.	Fucose	n.a.	n.a.	n.a.	n.a.	n.a.	n.a.
1	Succinate RI	7,865	0,127	0,629	13,65	15,29	n.a.
2	Lactate RI	8,268	0,053	0,285	5,69	6,91	1,5323
n.a.	glycerol	n.a.	n.a.	n.a.	n.a.	n.a.	n.a.
3	Formate RI	8,813	0,084	0,403	9,05	9,79	8,8139
4	Acetate RI	9,665	0,598	2,511	64,47	61,00	36,8202
n.a.	1,2 PDO RI	n.a.	n.a.	n.a.	n.a.	n.a.	n.a.
n.a.	1,3-PDO	n.a.	n.a.	n.a.	n.a.	n.a.	n.a.
5	Propionate RI	11,593	0,025	0,138	2,71	3,35	1,0129
n.a.	1,3-PDO	n.a.	n.a.	n.a.	n.a.	n.a.	n.a.
n.a.	2-3 BDO	n.a.	n.a.	n.a.	n.a.	n.a.	n.a.
6	Ethanol	13,352	0,041	0,151	4,42	3,67	0,3780
n.a.	Isobutyrate RI	n.a.	n.a.	n.a.	n.a.	n.a.	n.a.
n.a.	Butyrate RI	n.a.	n.a.	n.a.	n.a.	n.a.	n.a.
**Total:**			**0,928**	**4,117**	**100,00**	**100,00**	

Peak Analysis

Injection Details

Injection Name:	**42 5HMO1 t24 r3**	*Run Time (min):*	**20,00**
Vial Number:	**3:A9**	*Injection Volume:*	**10,00**
Injection Type:	**Unknown**	*Channel:*	**RI_CH_1**
Calibration Level:		*Wavelength:*	**n.a.**
Instrument Method:	**Default method LC2030C 45 gr 20 min**	*Bandwidth:*	**n.a.**
Processing Method:	**Processing Method LC2030 45 gr**	*Dilution Factor:*	**1,0000**
Injection Date/Time:	**13-jun-23 17:03**	*Sample Weight:*	**1,0000**

Chromatogram

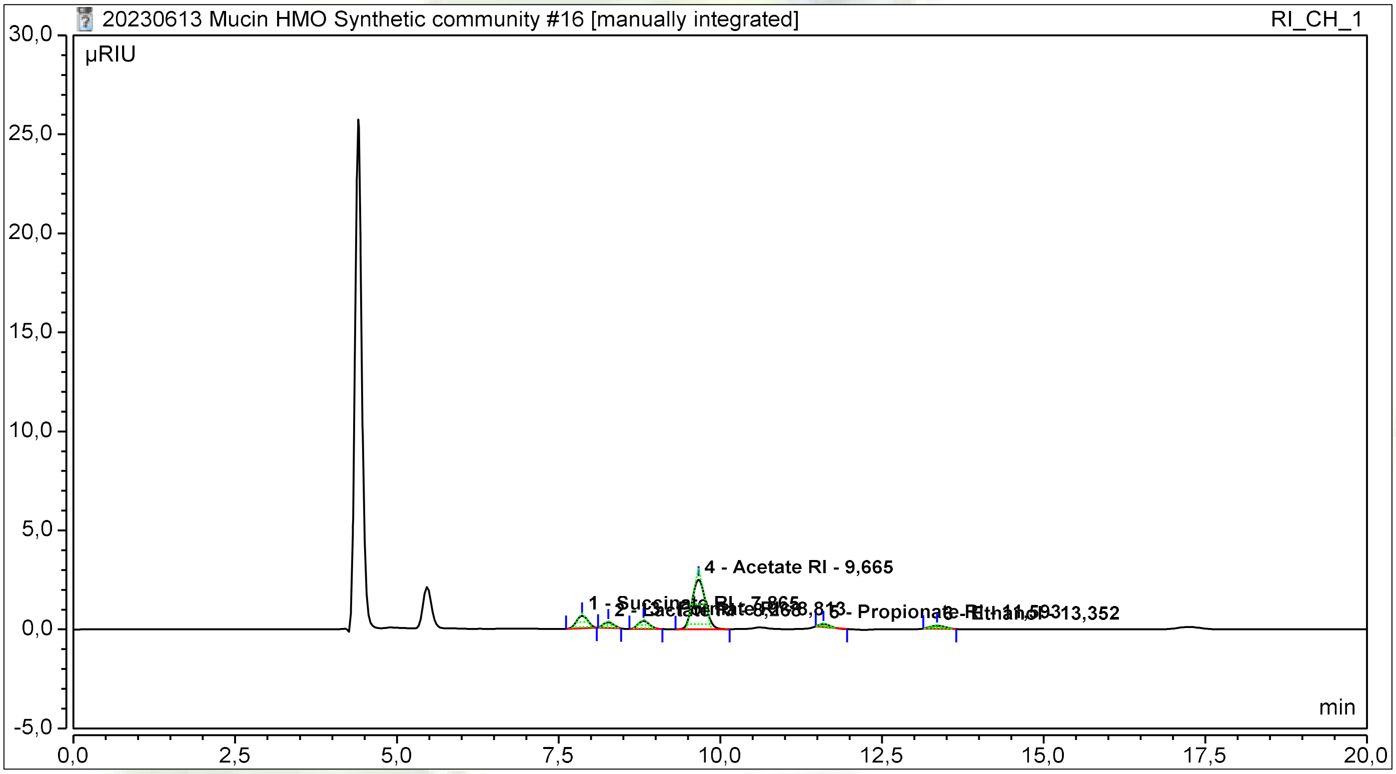

Peak Results

No.	Peak Name	Retention Time min	Width (50%) min	Type	Resolution (EP)	Asymmetry (EP)	Plates (EP)
n.a.	GlcNAc	n.a.	n.a.	n.a.	n.a.	n.a.	n.a.
n.a.	Citrate	n.a.	n.a.	n.a.	n.a.	n.a.	n.a.
n.a.	Glucose	n.a.	n.a.	n.a.	n.a.	n.a.	n.a.
n.a.	Galactose	n.a.	n.a.	n.a.	n.a.	n.a.	n.a.
n.a.	Fucose	n.a.	n.a.	n.a.	n.a.	n.a.	n.a.
1	Succinate RI	7,865	0,195	BMB	1,26	1,01	9021
2	Lactate RI	8,268	0,184	BMB*	1,68	1,12	11186
n.a.	glycerol	n.a.	n.a.	n.a.	n.a.	n.a.	n.a.
3	Formate RI	8,813	0,199	BMB*	2,37	1,10	10870
4	Acetate RI	9,665	0,225	BMB	5,41	1,08	10186
n.a.	1,2 PDO RI	n.a.	n.a.	n.a.	n.a.	n.a.	n.a.
n.a.	1,3-PDO	n.a.	n.a.	n.a.	n.a.	n.a.	n.a.
5	Propionate RI	11,593	0,195	BMB*	4,45	1,37	19509
n.a.	1,3-PDO	n.a.	n.a.	n.a.	n.a.	n.a.	n.a.
n.a.	2-3 BDO	n.a.	n.a.	n.a.	n.a.	n.a.	n.a.
6	Ethanol	13,352	0,271	BMB*	n.a.	1,15	13484
n.a.	Isobutyrate RI	n.a.	n.a.	n.a.	n.a.	n.a.	n.a.
n.a.	Butyrate RI	n.a.	n.a.	n.a.	n.a.	n.a.	n.a.

Chromatogram and SST Results

Injection Details

Injection Name:	**42 5HMO1 t24 r3**	*Run Time (min):*	**20,00**
Vial Number:	**3:A9**	*Injection Volume:*	**10,00**
Injection Type:	**Unknown**	*Channel:*	**RI_CH_1**
Calibration Level:		*Wavelength:*	**n.a.**
Instrument Method:	**Default method LC2030C 45 gr 20 min**	*Bandwidth:*	**n.a.**
Processing Method:	**Processing Method LC2030 45 gr**	*Dilution Factor:*	**1,0000**
Injection Date/Time:	**13-jun-23 17:03**	*Sample Weight:*	**1,0000**

Chromatogram

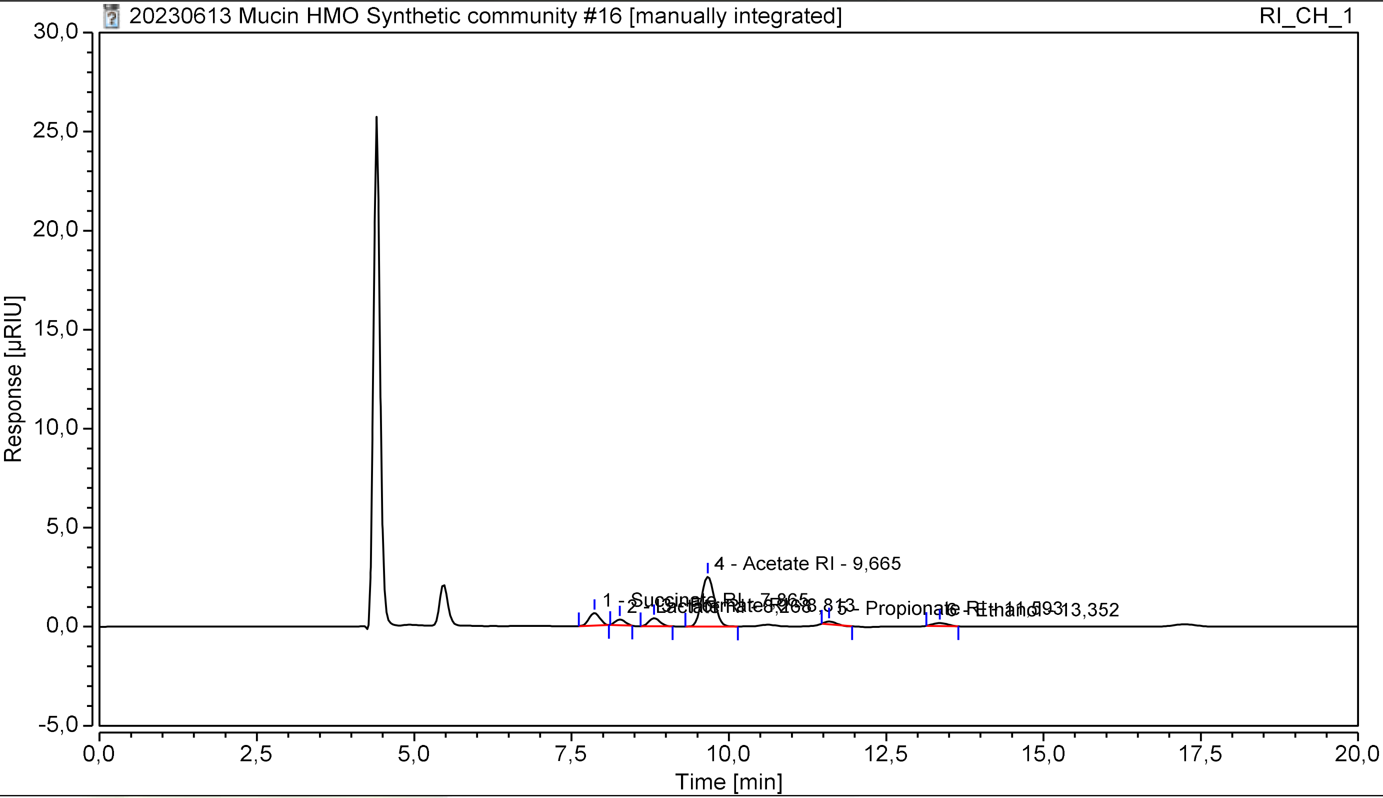

SST Results

No.	Name	Inj.Condition	Peak	Test Result	Injection

Number of executed test cases: **n.a.** **Total Result:** **Passed**

Chromatogram

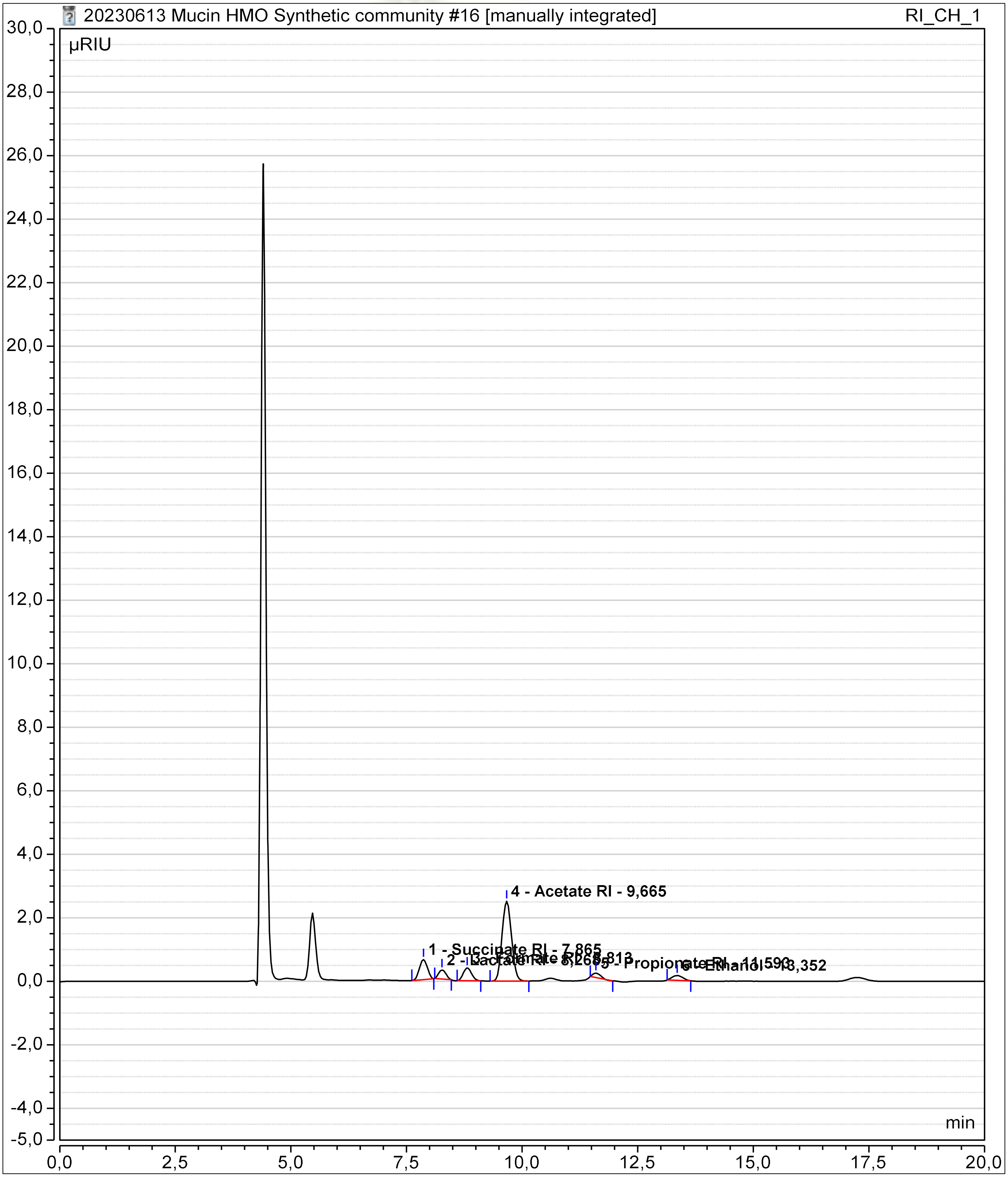

Chromatogram and Results

Injection Details

Injection Name:	**43 5HMO1 t48 r1**	*Run Time (min):*	**20,00**
Vial Number:	**3:A10**	*Injection Volume:*	**10,00**
Injection Type:	**Unknown**	*Channel:*	**RI_CH_1**
Calibration Level:		*Wavelength:*	**n.a.**
Instrument Method:	**Default method LC2030C 45 gr 20 min**	*Bandwidth:*	**n.a.**
Processing Method:	**Processing Method LC2030 45 gr**	*Dilution Factor:*	**1,0000**
Injection Date/Time:	**13-jun-23 17:23**	*Sample Weight:*	**1,0000**

Chromatogram

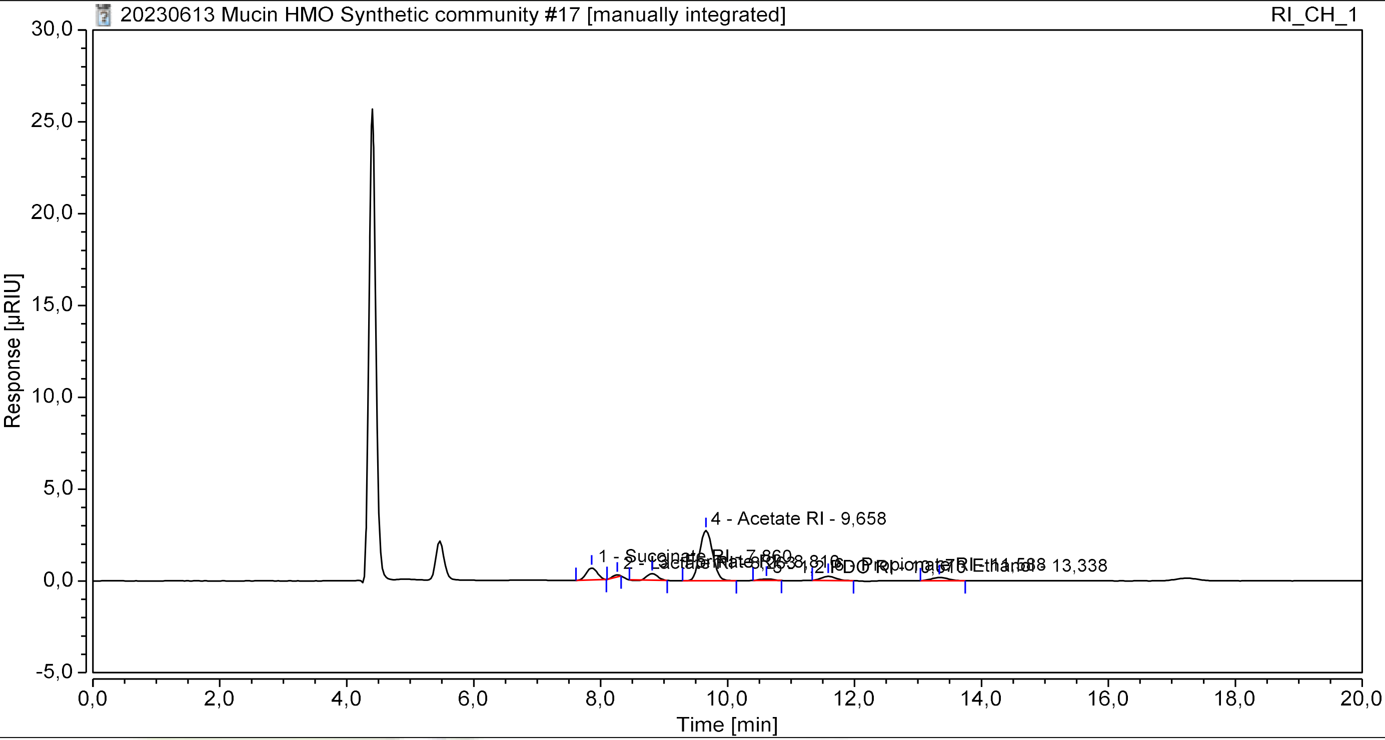

Integration Results

No.	Peak Name	Retention Time min	Area µRIU*min	Height µRIU	Relative Area %	Relative Height %	Amount
n.a.	GlcNAc	n.a.	n.a.	n.a.	n.a.	n.a.	n.a.
n.a.	Citrate	n.a.	n.a.	n.a.	n.a.	n.a.	n.a.
n.a.	Glucose	n.a.	n.a.	n.a.	n.a.	n.a.	n.a.
n.a.	Galactose	n.a.	n.a.	n.a.	n.a.	n.a.	n.a.
n.a.	Fucose	n.a.	n.a.	n.a.	n.a.	n.a.	n.a.
1	Succinate RI	7,860	0,131	0,646	13,15	14,94	n.a.
2	Lactate RI	8,263	0,014	0,114	1,44	2,63	0,4145
n.a.	glycerol	n.a.	n.a.	n.a.	n.a.	n.a.	n.a.
3	Formate RI	8,810	0,067	0,362	6,78	8,36	7,0693
4	Acetate RI	9,658	0,650	2,728	65,46	63,07	40,0062
5	1,2 PDO RI	10,610	0,019	0,083	1,96	1,91	0,5787
n.a.	1,3-PDO	n.a.	n.a.	n.a.	n.a.	n.a.	n.a.
6	Propionate RI	11,588	0,059	0,221	5,91	5,12	2,3632
n.a.	1,3-PDO	n.a.	n.a.	n.a.	n.a.	n.a.	n.a.
n.a.	2-3 BDO	n.a.	n.a.	n.a.	n.a.	n.a.	n.a.
7	Ethanol	13,338	0,053	0,172	5,30	3,97	0,4848
n.a.	Isobutyrate RI	n.a.	n.a.	n.a.	n.a.	n.a.	n.a.
n.a.	Butyrate RI	n.a.	n.a.	n.a.	n.a.	n.a.	n.a.
**Total:**			**0,993**	**4,326**	**100,00**	**100,00**	

Peak Analysis

Injection Details

Injection Name:	**43 5HMO1 t48 r1**	*Run Time (min):*	**20,00**
Vial Number:	**3:A10**	*Injection Volume:*	**10,00**
Injection Type:	**Unknown**	*Channel:*	**RI_CH_1**
Calibration Level:		*Wavelength:*	**n.a.**
Instrument Method:	**Default method LC2030C 45 gr 20 min**	*Bandwidth:*	**n.a.**
Processing Method:	**Processing Method LC2030 45 gr**	*Dilution Factor:*	**1,0000**
Injection Date/Time:	**13-jun-23 17:23**	*Sample Weight:*	**1,0000**

Chromatogram

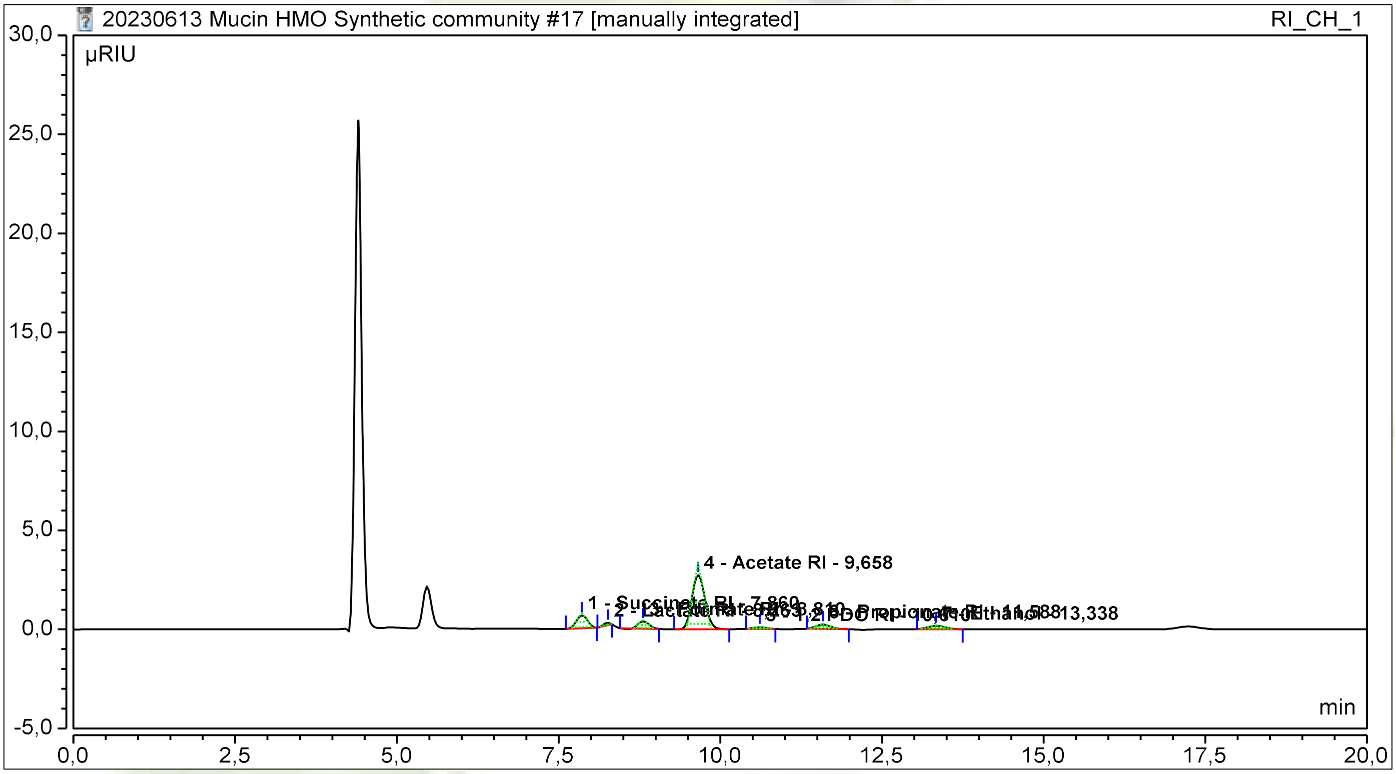

Peak Results

No.	Peak Name	Retention Time min	Width (50%) min	Type	Resolution (EP)	Asymmetry (EP)	Plates (EP)
n.a.	GlcNAc	n.a.	n.a.	n.a.	n.a.	n.a.	n.a.
n.a.	Citrate	n.a.	n.a.	n.a.	n.a.	n.a.	n.a.
n.a.	Glucose	n.a.	n.a.	n.a.	n.a.	n.a.	n.a.
n.a.	Galactose	n.a.	n.a.	n.a.	n.a.	n.a.	n.a.
n.a.	Fucose	n.a.	n.a.	n.a.	n.a.	n.a.	n.a.
1	Succinate RI	7,860	0,196	BMB	1,48	1,02	8942
2	Lactate RI	8,263	0,125	BMB*	2,03	0,73	24137
n.a.	glycerol	n.a.	n.a.	n.a.	n.a.	n.a.	n.a.
3	Formate RI	8,810	0,192	BMB*	2,40	1,12	11627
4	Acetate RI	9,658	0,225	BMB	2,45	1,09	10183
5	1,2 PDO RI	10,610	0,234	BMB*	2,36	1,06	11425
n.a.	1,3-PDO	n.a.	n.a.	n.a.	n.a.	n.a.	n.a.
6	Propionate RI	11,588	0,255	BMB*	3,75	1,10	11400
n.a.	1,3-PDO	n.a.	n.a.	n.a.	n.a.	n.a.	n.a.
n.a.	2-3 BDO	n.a.	n.a.	n.a.	n.a.	n.a.	n.a.
7	Ethanol	13,338	0,296	BMB*	n.a.	1,09	11283
n.a.	Isobutyrate RI	n.a.	n.a.	n.a.	n.a.	n.a.	n.a.
n.a.	Butyrate RI	n.a.	n.a.	n.a.	n.a.	n.a.	n.a.

Chromatogram and SST Results

Injection Details

Injection Name:	**43 5HMO1 t48 r1**	*Run Time (min):*	**20,00**
Vial Number:	**3:A10**	*Injection Volume:*	**10,00**
Injection Type:	**Unknown**	*Channel:*	**RI_CH_1**
Calibration Level:		*Wavelength:*	**n.a.**
Instrument Method:	**Default method LC2030C 45 gr 20 min**	*Bandwidth:*	**n.a.**
Processing Method:	**Processing Method LC2030 45 gr**	*Dilution Factor:*	**1,0000**
Injection Date/Time:	**13-jun-23 17:23**	*Sample Weight:*	**1,0000**

Chromatogram

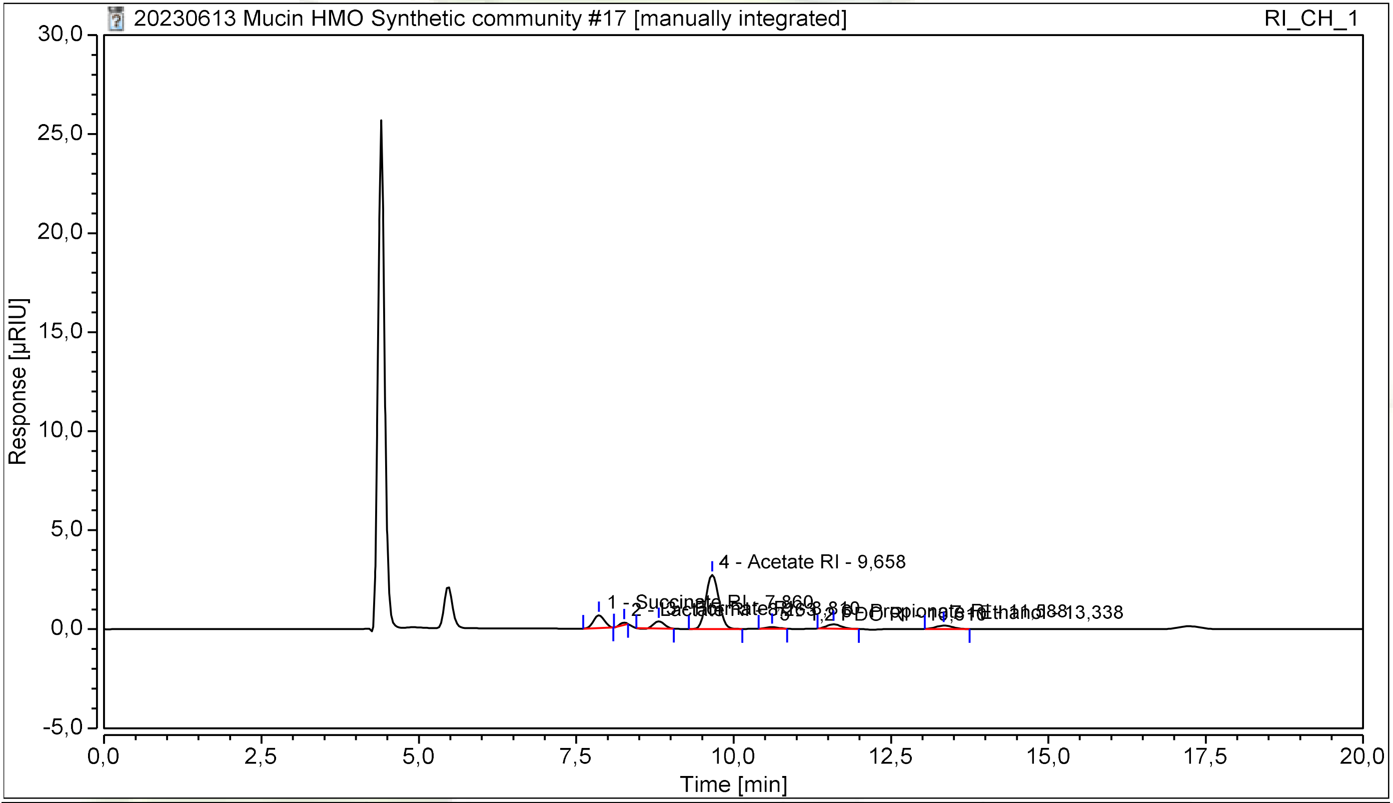

SST Results

No.	Name	Inj.Condition	Peak	Test Result	Injection
**Number of executed test cases:**	**n.a.**		**Total Result:**	**Passed**	

Chromeleon (c) Dionex
Version 7.2.10.25868

Chromatogram

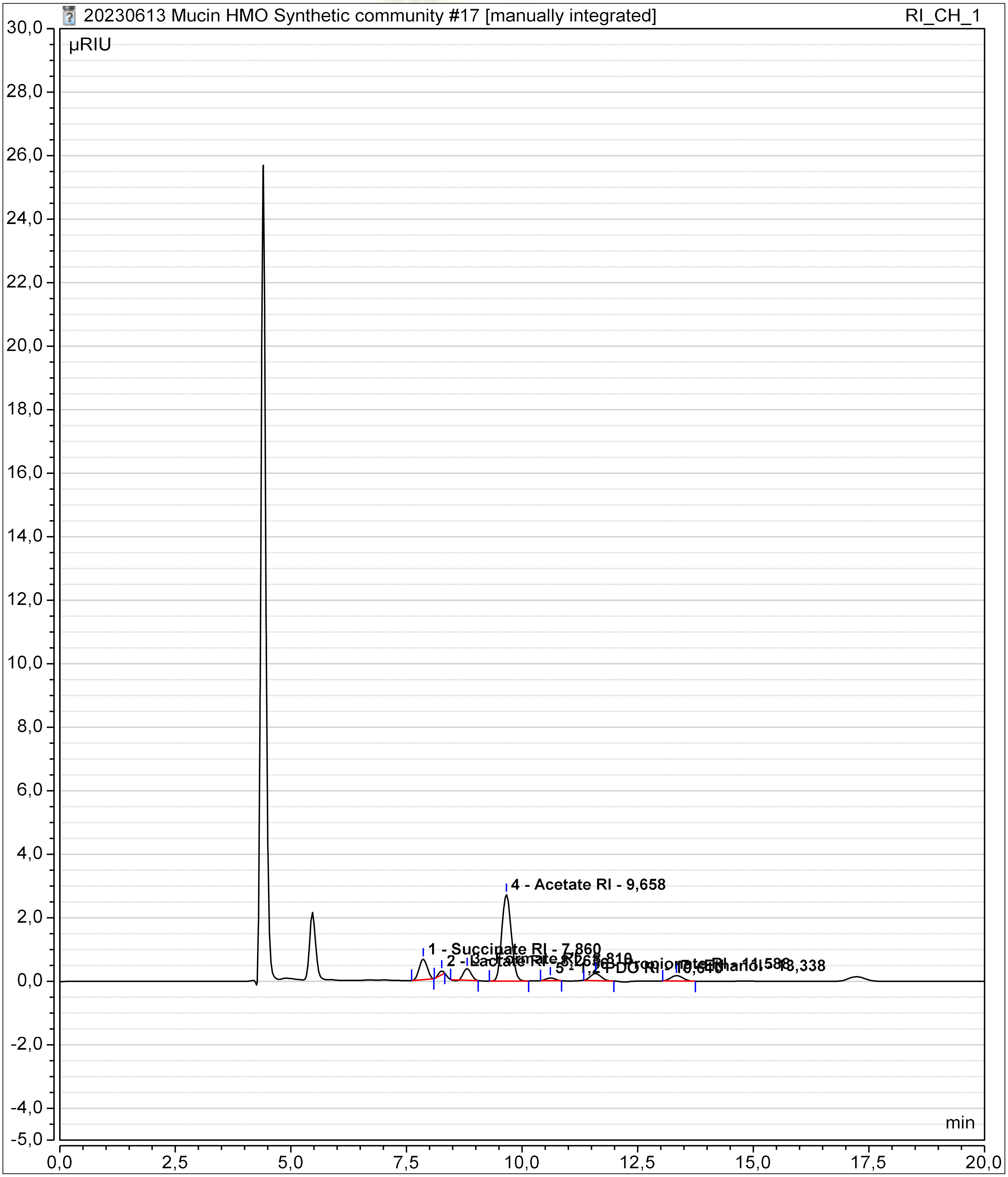

Chromatogram and Results

Injection Details

Injection Name:	**44 5HMO1 t48 r2**	*Run Time (min):*	**20,00**
Vial Number:	**3:A11**	*Injection Volume:*	**10,00**
Injection Type:	**Unknown**	*Channel:*	**RI_CH_1**
Calibration Level:		*Wavelength:*	**n.a.**
Instrument Method:	**Default method LC2030C 45 gr 20 min**	*Bandwidth:*	**n.a.**
Processing Method:	**Processing Method LC2030 45 gr**	*Dilution Factor:*	**1,0000**
Injection Date/Time:	**13-jun-23 17:44**	*Sample Weight:*	**1,0000**

Chromatogram

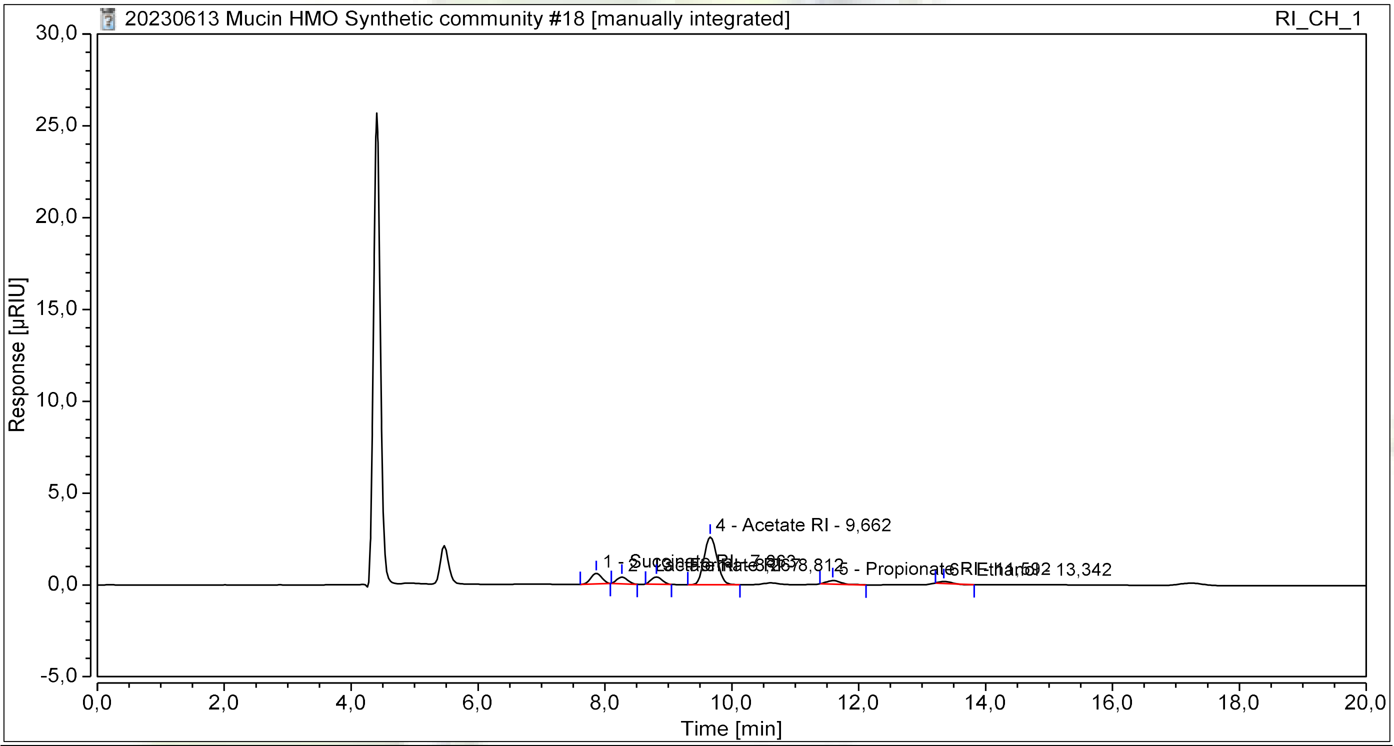

Integration Results

No.	Peak Name	Retention Time<br>min	Area<br>µRIU*min	Height<br>µRIU	Relative Area<br>%	Relative Height<br>%	Amount
n.a.	GlcNAc	n.a.	n.a.	n.a.	n.a.	n.a.	n.a.
n.a.	Citrate	n.a.	n.a.	n.a.	n.a.	n.a.	n.a.
n.a.	Glucose	n.a.	n.a.	n.a.	n.a.	n.a.	n.a.
n.a.	Galactose	n.a.	n.a.	n.a.	n.a.	n.a.	n.a.
n.a.	Fucose	n.a.	n.a.	n.a.	n.a.	n.a.	n.a.
1	Succinate RI	7,863	0,113	0,565	11,93	13,44	n.a.
2	Lactate RI	8,267	0,070	0,359	7,40	8,54	2,0349
n.a.	glycerol	n.a.	n.a.	n.a.	n.a.	n.a.	n.a.
3	Formate RI	8,812	0,078	0,394	8,21	9,38	8,1585
4	Acetate RI	9,662	0,617	2,594	65,17	61,68	37,9957
n.a.	1,2 PDO RI	n.a.	n.a.	n.a.	n.a.	n.a.	n.a.
n.a.	1,3-PDO	n.a.	n.a.	n.a.	n.a.	n.a.	n.a.
5	Propionate RI	11,592	0,050	0,194	5,32	4,61	2,0268
n.a.	1,3-PDO	n.a.	n.a.	n.a.	n.a.	n.a.	n.a.
n.a.	2-3 BDO	n.a.	n.a.	n.a.	n.a.	n.a.	n.a.
6	Ethanol	13,342	0,019	0,099	1,98	2,35	0,1726
n.a.	Isobutyrate RI	n.a.	n.a.	n.a.	n.a.	n.a.	n.a.
n.a.	Butyrate RI	n.a.	n.a.	n.a.	n.a.	n.a.	n.a.
**Total:**			**0,947**	**4,205**	**100,00**	**100,00**	

Peak Analysis

Injection Details

Injection Name:	**44 5HMO1 t48 r2**	*Run Time (min):*	**20,00**
Vial Number:	**3:A11**	*Injection Volume:*	**10,00**
Injection Type:	**Unknown**	*Channel:*	**RI_CH_1**
Calibration Level:		*Wavelength:*	**n.a.**
Instrument Method:	**Default method LC2030C 45 gr 20 min**	*Bandwidth:*	**n.a.**
Processing Method:	**Processing Method LC2030 45 gr**	*Dilution Factor:*	**1,0000**
Injection Date/Time:	**13-jun-23 17:44**	*Sample Weight:*	**1,0000**

Chromatogram

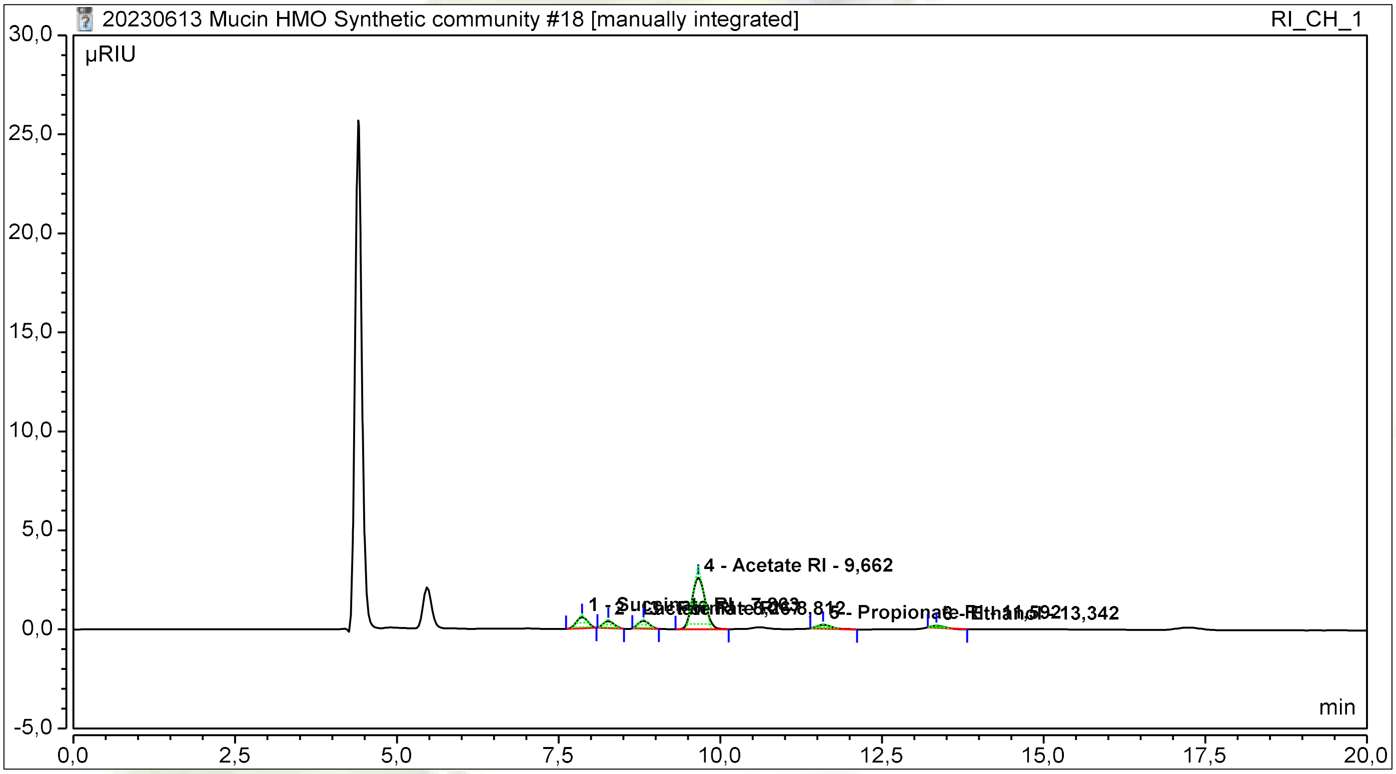

Peak Results

No.	Peak Name	Retention Time min	Width (50%) min	Type	Resolution (EP)	Asymmetry (EP)	Plates (EP)
n.a.	GlcNAc	n.a.	n.a.	n.a.	n.a.	n.a.	n.a.
n.a.	Citrate	n.a.	n.a.	n.a.	n.a.	n.a.	n.a.
n.a.	Glucose	n.a.	n.a.	n.a.	n.a.	n.a.	n.a.
n.a.	Galactose	n.a.	n.a.	n.a.	n.a.	n.a.	n.a.
n.a.	Fucose	n.a.	n.a.	n.a.	n.a.	n.a.	n.a.
1	Succinate RI	7,863	0,194	BMB	1,24	1,01	9111
2	Lactate RI	8,267	0,190	BMB*	1,68	1,16	10469
n.a.	glycerol	n.a.	n.a.	n.a.	n.a.	n.a.	n.a.
3	Formate RI	8,812	0,192	BMB*	2,40	1,12	11631
4	Acetate RI	9,662	0,225	BMB	4,85	1,09	10197
n.a.	1,2 PDO RI	n.a.	n.a.	n.a.	n.a.	n.a.	n.a.
n.a.	1,3-PDO	n.a.	n.a.	n.a.	n.a.	n.a.	n.a.
5	Propionate RI	11,592	0,245	BMB*	4,44	1,24	12427
n.a.	1,3-PDO	n.a.	n.a.	n.a.	n.a.	n.a.	n.a.
n.a.	2-3 BDO	n.a.	n.a.	n.a.	n.a.	n.a.	n.a.
6	Ethanol	13,342	0,221	BMB*	n.a.	1,33	20280
n.a.	Isobutyrate RI	n.a.	n.a.	n.a.	n.a.	n.a.	n.a.
n.a.	Butyrate RI	n.a.	n.a.	n.a.	n.a.	n.a.	n.a.

Chromeleon (c) Dionex
Version 7.2.10.25868

Chromatogram and SST Results

Injection Details

Injection Name:	**44 5HMO1 t48 r2**	*Run Time (min):*	**20,00**
Vial Number:	**3:A11**	*Injection Volume:*	**10,00**
Injection Type:	**Unknown**	*Channel:*	**RI_CH_1**
Calibration Level:		*Wavelength:*	**n.a.**
Instrument Method:	**Default method LC2030C 45 gr 20 min**	*Bandwidth:*	**n.a.**
Processing Method:	**Processing Method LC2030 45 gr**	*Dilution Factor:*	**1,0000**
Injection Date/Time:	**13-jun-23 17:44**	*Sample Weight:*	**1,0000**

Chromatogram

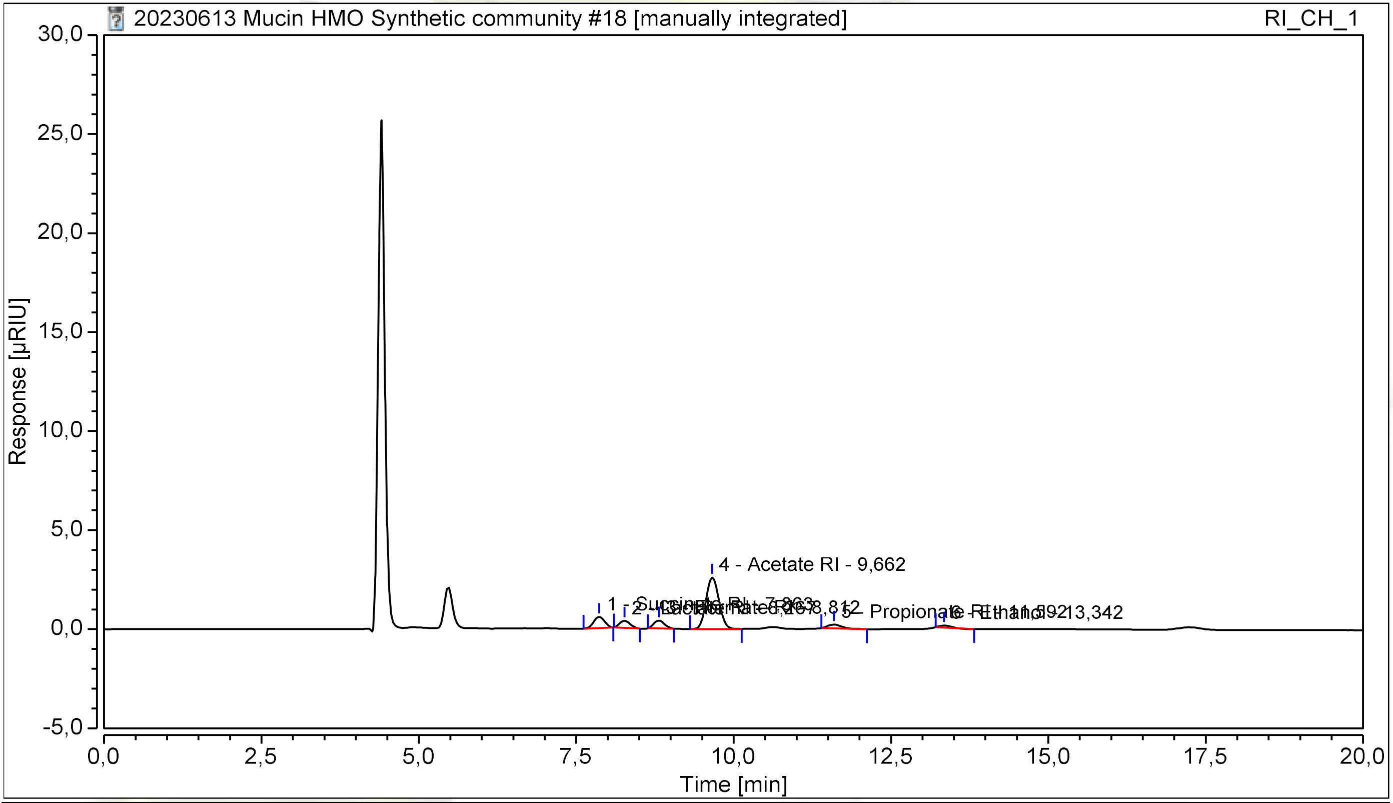

SST Results

No.	Name	Inj.Condition	Peak	Test Result	Injection

Number of executed test cases: **n.a.**   **Total Result:** **Passed**

Chromeleon (c) Dionex
Version 7.2.10.25868

Chromatogram

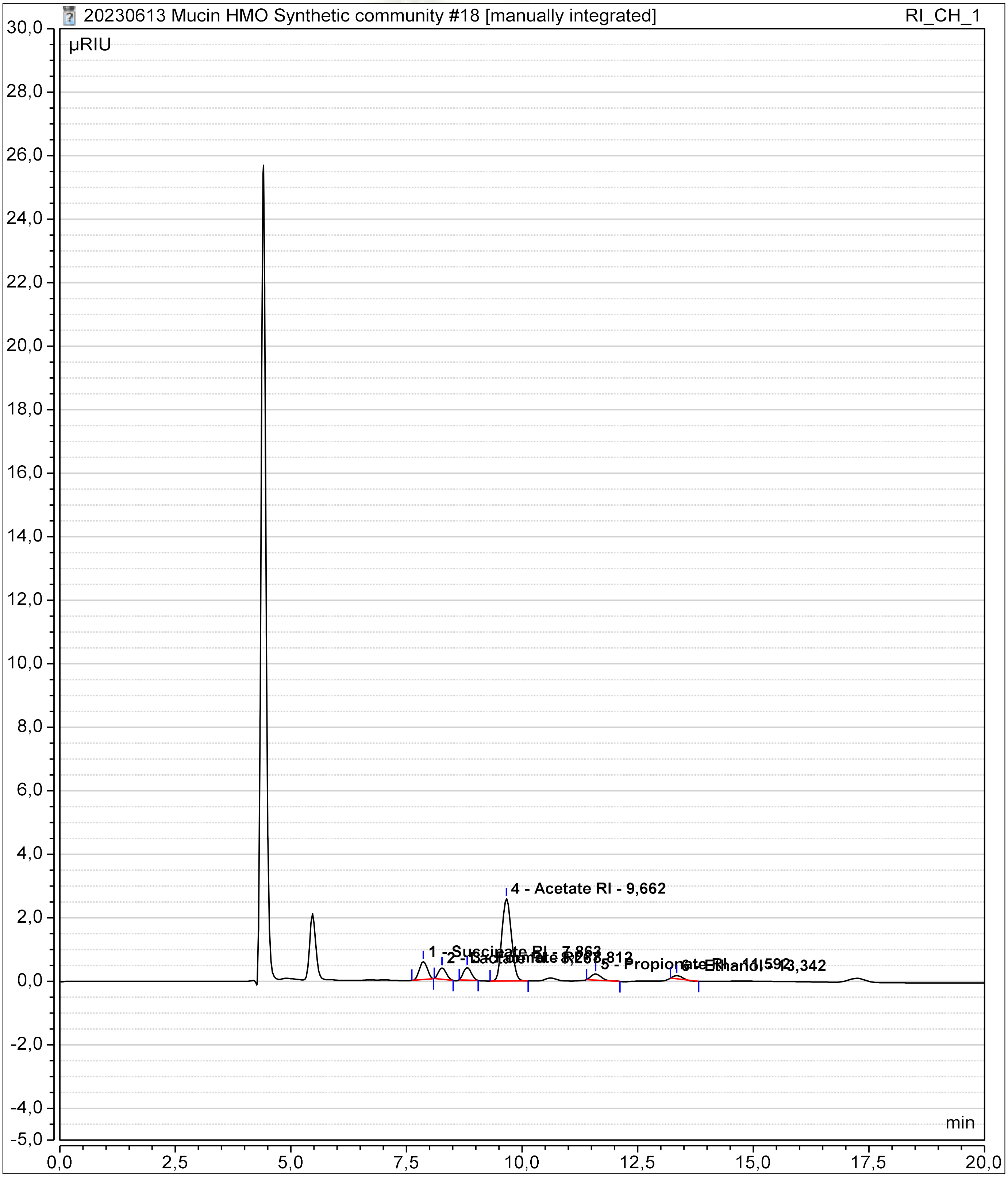

Chromatogram and Results

Injection Details

Injection Name:	**45 5HMO1 t48 r3**	*Run Time (min):*	**20,00**
Vial Number:	**3:A12**	*Injection Volume:*	**10,00**
Injection Type:	**Unknown**	*Channel:*	**RI_CH_1**
Calibration Level:		*Wavelength:*	**n.a.**
Instrument Method:	**Default method LC2030C 45 gr 20 min**	*Bandwidth:*	**n.a.**
Processing Method:	**Processing Method LC2030 45 gr**	*Dilution Factor:*	**1,0000**
Injection Date/Time:	**13-jun-23 18:04**	*Sample Weight:*	**1,0000**

Chromatogram

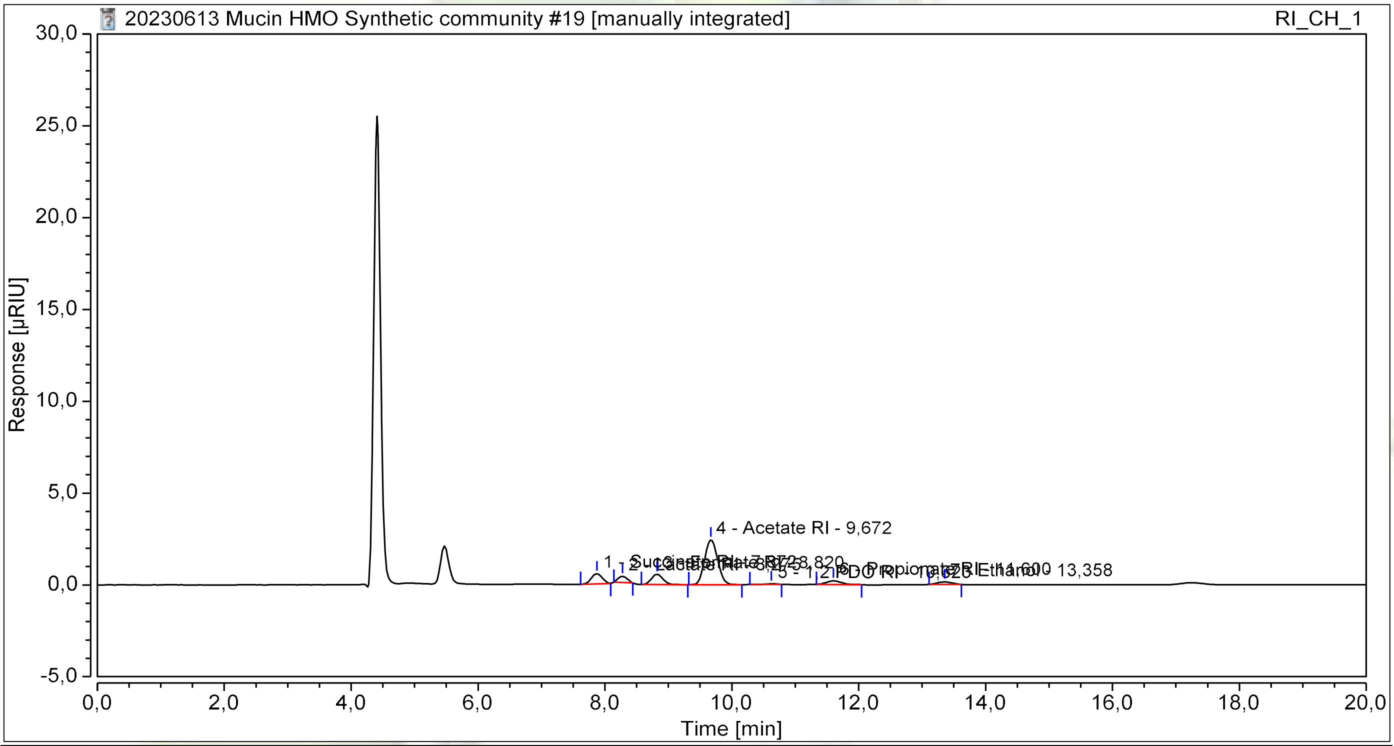

Integration Results

No.	Peak Name	Retention Time<br>min	Area<br>µRIU*min	Height<br>µRIU	Relative Area<br>%	Relative Height<br>%	Amount
n.a.	GlcNAc	n.a.	n.a.	n.a.	n.a.	n.a.	n.a.
n.a.	Citrate	n.a.	n.a.	n.a.	n.a.	n.a.	n.a.
n.a.	Glucose	n.a.	n.a.	n.a.	n.a.	n.a.	n.a.
n.a.	Galactose	n.a.	n.a.	n.a.	n.a.	n.a.	n.a.
n.a.	Fucose	n.a.	n.a.	n.a.	n.a.	n.a.	n.a.
1	Succinate RI	7,872	0,109	0,548	11,39	12,94	n.a.
2	Lactate RI	8,275	0,057	0,336	5,92	7,95	1,6499
n.a.	glycerol	n.a.	n.a.	n.a.	n.a.	n.a.	n.a.
3	Formate RI	8,820	0,117	0,554	12,22	13,09	12,3071
4	Acetate RI	9,672	0,580	2,435	60,47	57,51	35,6954
5	1,2 PDO RI	10,623	0,006	0,031	0,67	0,74	0,1911
n.a.	1,3-PDO	n.a.	n.a.	n.a.	n.a.	n.a.	n.a.
6	Propionate RI	11,600	0,052	0,194	5,40	4,57	2,0840
n.a.	1,3-PDO	n.a.	n.a.	n.a.	n.a.	n.a.	n.a.
n.a.	2-3 BDO	n.a.	n.a.	n.a.	n.a.	n.a.	n.a.
7	Ethanol	13,358	0,038	0,136	3,93	3,21	0,3468
n.a.	Isobutyrate RI	n.a.	n.a.	n.a.	n.a.	n.a.	n.a.
n.a.	Butyrate RI	n.a.	n.a.	n.a.	n.a.	n.a.	n.a.
**Total:**			**0,959**	**4,234**	**100,00**	**100,00**	

Peak Analysis

Injection Details

Injection Name:	**45 5HMO1 t48 r3**	*Run Time (min):*	**20,00**
Vial Number:	**3:A12**	*Injection Volume:*	**10,00**
Injection Type:	**Unknown**	*Channel:*	**RI_CH_1**
Calibration Level:		*Wavelength:*	**n.a.**
Instrument Method:	**Default method LC2030C 45 gr 20 min**	*Bandwidth:*	**n.a.**
Processing Method:	**Processing Method LC2030 45 gr**	*Dilution Factor:*	**1,0000**
Injection Date/Time:	**13-jun-23 18:04**	*Sample Weight:*	**1,0000**

Chromatogram

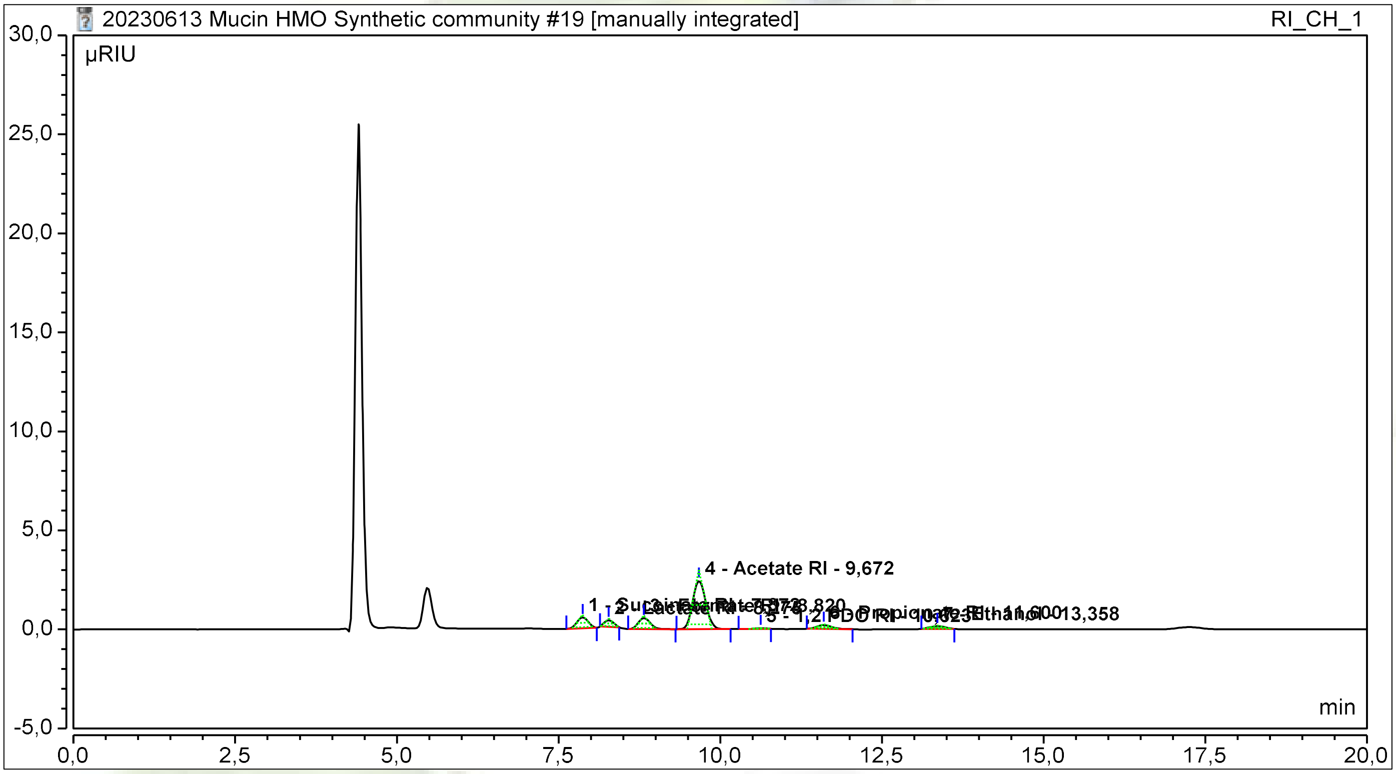

Peak Results

No.	Peak Name	Retention Time min	Width (50%) min	Type	Resolution (EP)	Asymmetry (EP)	Plates (EP)
n.a.	GlcNAc	n.a.	n.a.	n.a.	n.a.	n.a.	n.a.
n.a.	Citrate	n.a.	n.a.	n.a.	n.a.	n.a.	n.a.
n.a.	Glucose	n.a.	n.a.	n.a.	n.a.	n.a.	n.a.
n.a.	Galactose	n.a.	n.a.	n.a.	n.a.	n.a.	n.a.
n.a.	Fucose	n.a.	n.a.	n.a.	n.a.	n.a.	n.a.
1	Succinate RI	7,872	0,194	BMB	1,31	1,00	9157
2	Lactate RI	8,275	0,171	BMB*	1,74	1,10	12996
n.a.	glycerol	n.a.	n.a.	n.a.	n.a.	n.a.	n.a.
3	Formate RI	8,820	0,200	BMB	2,36	1,11	10797
4	Acetate RI	9,672	0,225	BMB	2,57	1,08	10201
5	1,2 PDO RI	10,623	0,212	BMB*	2,47	0,90	13914
n.a.	1,3-PDO	n.a.	n.a.	n.a.	n.a.	n.a.	n.a.
6	Propionate RI	11,600	0,255	BMB*	3,90	1,11	11474
n.a.	1,3-PDO	n.a.	n.a.	n.a.	n.a.	n.a.	n.a.
n.a.	2-3 BDO	n.a.	n.a.	n.a.	n.a.	n.a.	n.a.
7	Ethanol	13,358	0,278	BMB*	n.a.	1,02	12820
n.a.	Isobutyrate RI	n.a.	n.a.	n.a.	n.a.	n.a.	n.a.
n.a.	Butyrate RI	n.a.	n.a.	n.a.	n.a.	n.a.	n.a.

Chromatogram and SST Results

Injection Details

Injection Name:	**45 5HMO1 t48 r3**	*Run Time (min):*	**20,00**
Vial Number:	**3:A12**	*Injection Volume:*	**10,00**
Injection Type:	**Unknown**	*Channel:*	**RI_CH_1**
Calibration Level:		*Wavelength:*	**n.a.**
Instrument Method:	**Default method LC2030C 45 gr 20 min**	*Bandwidth:*	**n.a.**
Processing Method:	**Processing Method LC2030 45 gr**	*Dilution Factor:*	**1,0000**
Injection Date/Time:	**13-jun-23 18:04**	*Sample Weight:*	**1,0000**

Chromatogram

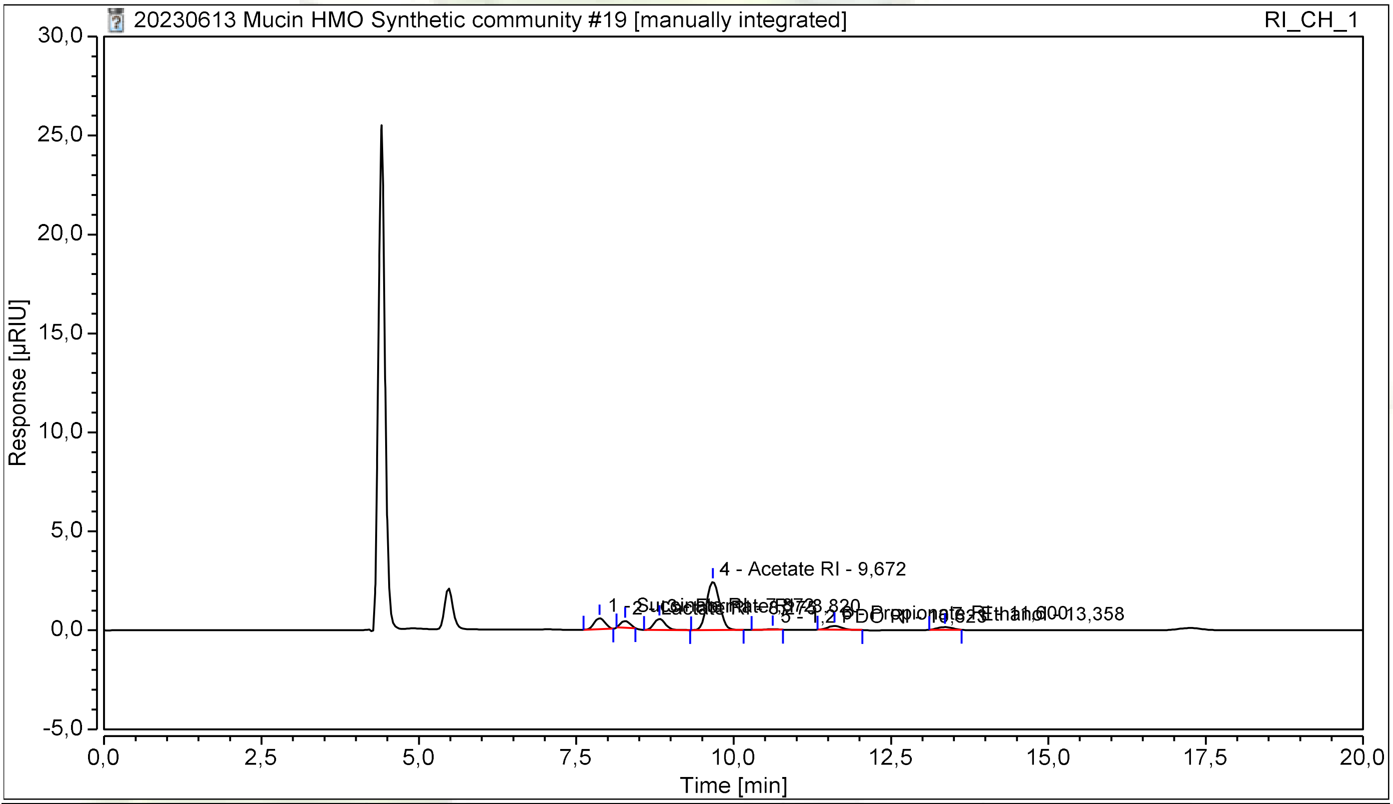

SST Results

No.	Name	Inj.Condition	Peak	Test Result	Injection

Number of executed test cases: **n.a.** **Total Result:** **Passed**

Chromeleon (c) Dionex
Version 7.2.10.25868

Chromatogram

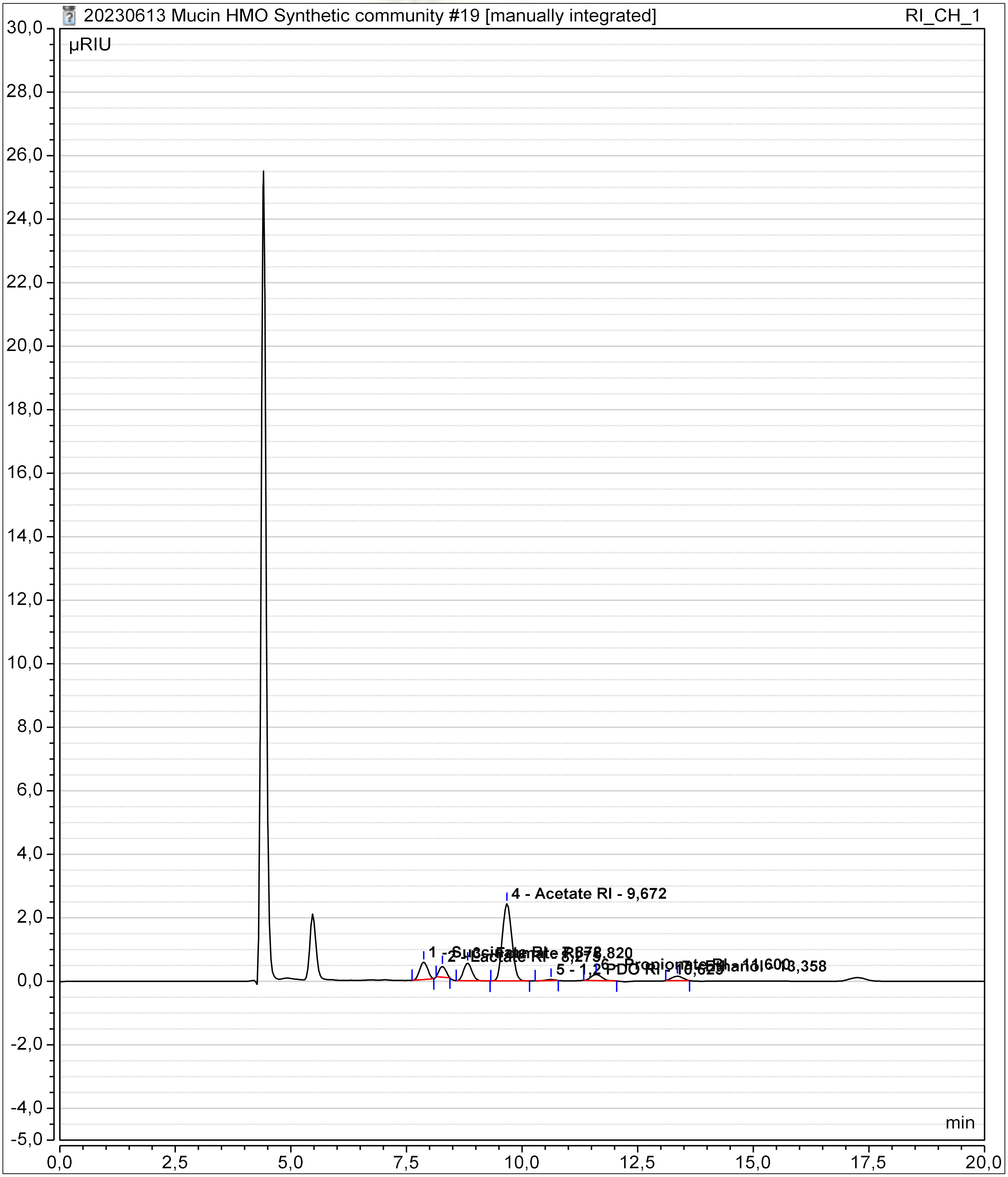

Chromatogram and Results

Injection Details

Injection Name:	**46 5HMO1 t72 r1**	*Run Time (min):*	**20,00**
Vial Number:	**3:B1**	*Injection Volume:*	**10,00**
Injection Type:	**Unknown**	*Channel:*	**RI_CH_1**
Calibration Level:		*Wavelength:*	**n.a.**
Instrument Method:	**Default method LC2030C 45 gr 20 min**	*Bandwidth:*	**n.a.**
Processing Method:	**Processing Method LC2030 45 gr**	*Dilution Factor:*	**1,0000**
Injection Date/Time:	**13-jun-23 18:24**	*Sample Weight:*	**1,0000**

Chromatogram

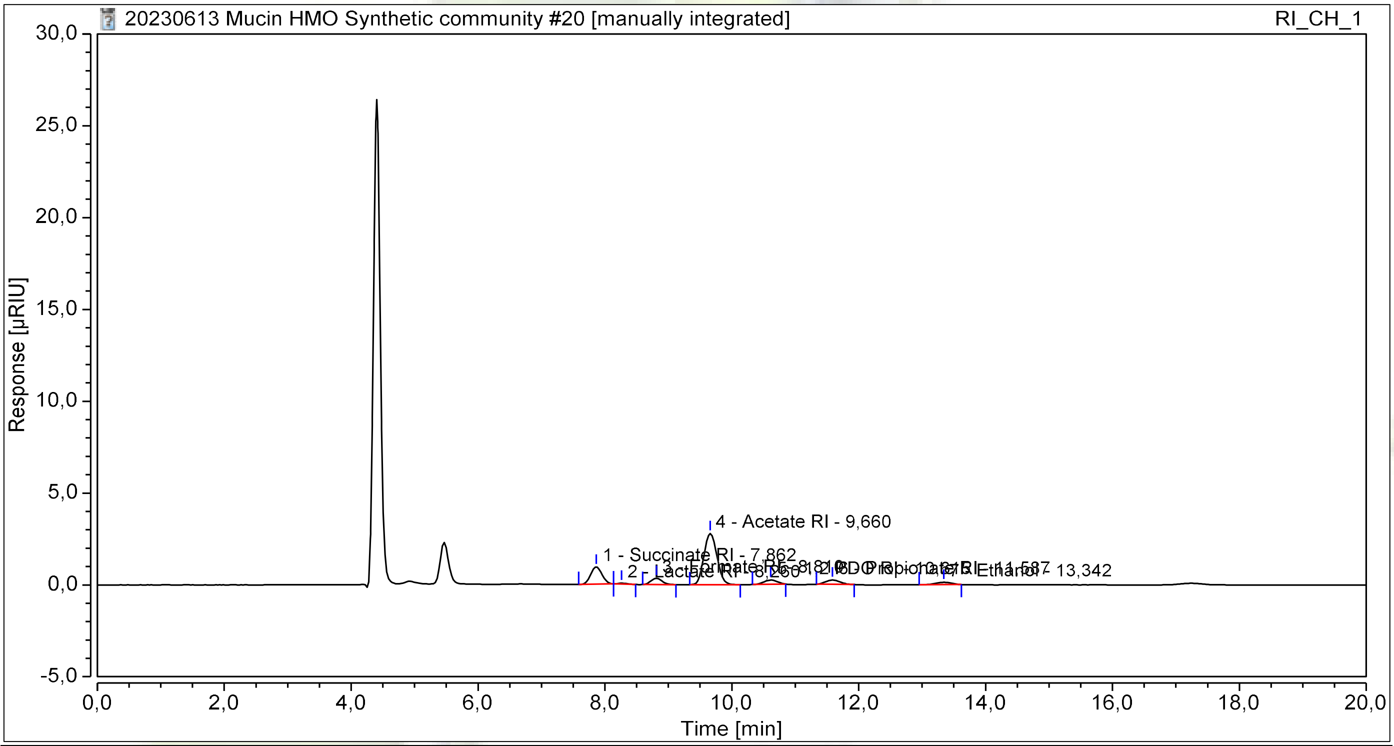

Integration Results

No.	Peak Name	Retention Time min	Area µRIU*min	Height µRIU	Relative Area %	Relative Height %	Amount
n.a.	GlcNAc	n.a.	n.a.	n.a.	n.a.	n.a.	n.a.
n.a.	Citrate	n.a.	n.a.	n.a.	n.a.	n.a.	n.a.
n.a.	Glucose	n.a.	n.a.	n.a.	n.a.	n.a.	n.a.
n.a.	Galactose	n.a.	n.a.	n.a.	n.a.	n.a.	n.a.
n.a.	Fucose	n.a.	n.a.	n.a.	n.a.	n.a.	n.a.
1	Succinate RI	7,862	0,195	0,934	17,81	19,82	n.a.
2	Lactate RI	8,260	0,008	0,047	0,73	0,99	0,2318
n.a.	glycerol	n.a.	n.a.	n.a.	n.a.	n.a.	n.a.
3	Formate RI	8,810	0,070	0,337	6,40	7,14	7,3501
4	Acetate RI	9,660	0,662	2,791	60,50	59,24	40,7550
5	1,2 PDO RI	10,615	0,058	0,235	5,26	4,98	1,7127
n.a.	1,3-PDO	n.a.	n.a.	n.a.	n.a.	n.a.	n.a.
6	Propionate RI	11,587	0,064	0,240	5,80	5,09	2,5552
n.a.	1,3-PDO	n.a.	n.a.	n.a.	n.a.	n.a.	n.a.
n.a.	2-3 BDO	n.a.	n.a.	n.a.	n.a.	n.a.	n.a.
7	Ethanol	13,342	0,038	0,129	3,50	2,75	0,3530
n.a.	Isobutyrate RI	n.a.	n.a.	n.a.	n.a.	n.a.	n.a.
n.a.	Butyrate RI	n.a.	n.a.	n.a.	n.a.	n.a.	n.a.
**Total:**			**1,095**	**4,711**	**100,00**	**100,00**	

Peak Analysis

Injection Details

Injection Name:	**46 5HMO1 t72 r1**	*Run Time (min):*	**20,00**
Vial Number:	**3:B1**	*Injection Volume:*	**10,00**
Injection Type:	**Unknown**	*Channel:*	**RI_CH_1**
Calibration Level:		*Wavelength:*	**n.a.**
Instrument Method:	**Default method LC2030C 45 gr 20 min**	*Bandwidth:*	**n.a.**
Processing Method:	**Processing Method LC2030 45 gr**	*Dilution Factor:*	**1,0000**
Injection Date/Time:	**13-jun-23 18:24**	*Sample Weight:*	**1,0000**

Chromatogram

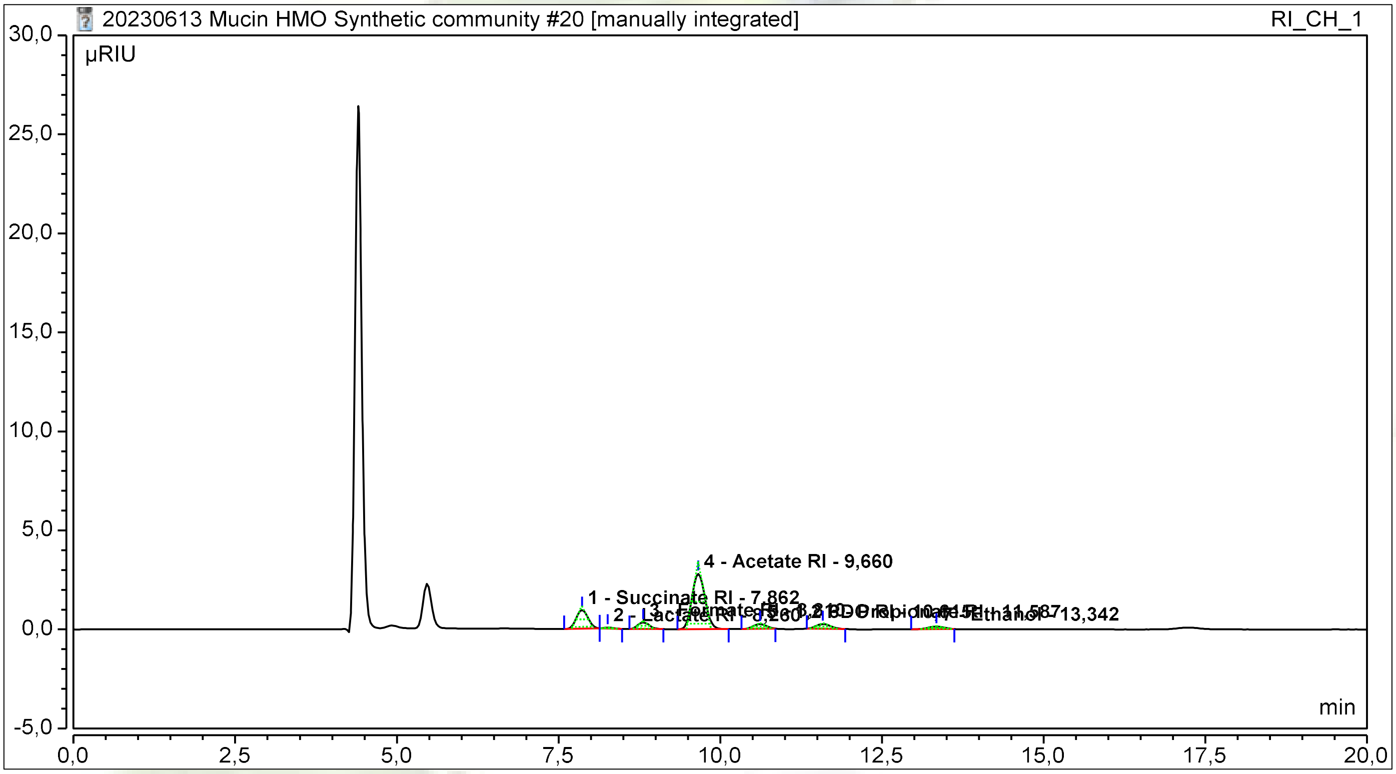

Peak Results

No.	Peak Name	Retention Time min	Width (50%) min	Type	Resolution (EP)	Asymmetry (EP)	Plates (EP)
n.a.	GlcNAc	n.a.	n.a.	n.a.	n.a.	n.a.	n.a.
n.a.	Citrate	n.a.	n.a.	n.a.	n.a.	n.a.	n.a.
n.a.	Glucose	n.a.	n.a.	n.a.	n.a.	n.a.	n.a.
n.a.	Galactose	n.a.	n.a.	n.a.	n.a.	n.a.	n.a.
n.a.	Fucose	n.a.	n.a.	n.a.	n.a.	n.a.	n.a.
1	Succinate RI	7,862	0,199	BMB	1,28	1,06	8612
2	Lactate RI	8,260	0,167	BMB*	1,78	1,35	13557
n.a.	glycerol	n.a.	n.a.	n.a.	n.a.	n.a.	n.a.
3	Formate RI	8,810	0,198	BMB*	2,37	1,11	10936
4	Acetate RI	9,660	0,225	BMB	2,43	1,09	10250
5	1,2 PDO RI	10,615	0,239	BMB*	2,31	0,98	10890
n.a.	1,3-PDO	n.a.	n.a.	n.a.	n.a.	n.a.	n.a.
6	Propionate RI	11,587	0,257	BMB*	3,79	1,09	11280
n.a.	1,3-PDO	n.a.	n.a.	n.a.	n.a.	n.a.	n.a.
n.a.	2-3 BDO	n.a.	n.a.	n.a.	n.a.	n.a.	n.a.
7	Ethanol	13,342	0,290	BMB*	n.a.	0,97	11762
n.a.	Isobutyrate RI	n.a.	n.a.	n.a.	n.a.	n.a.	n.a.
n.a.	Butyrate RI	n.a.	n.a.	n.a.	n.a.	n.a.	n.a.

Chromatogram and SST Results

Injection Details

Injection Name:	**46 5HMO1 t72 r1**	*Run Time (min):*	**20,00**
Vial Number:	**3:B1**	*Injection Volume:*	**10,00**
Injection Type:	**Unknown**	*Channel:*	**RI_CH_1**
Calibration Level:		*Wavelength:*	**n.a.**
Instrument Method:	**Default method LC2030C 45 gr 20 min**	*Bandwidth:*	**n.a.**
Processing Method:	**Processing Method LC2030 45 gr**	*Dilution Factor:*	**1,0000**
Injection Date/Time:	**13-jun-23 18:24**	*Sample Weight:*	**1,0000**

Chromatogram

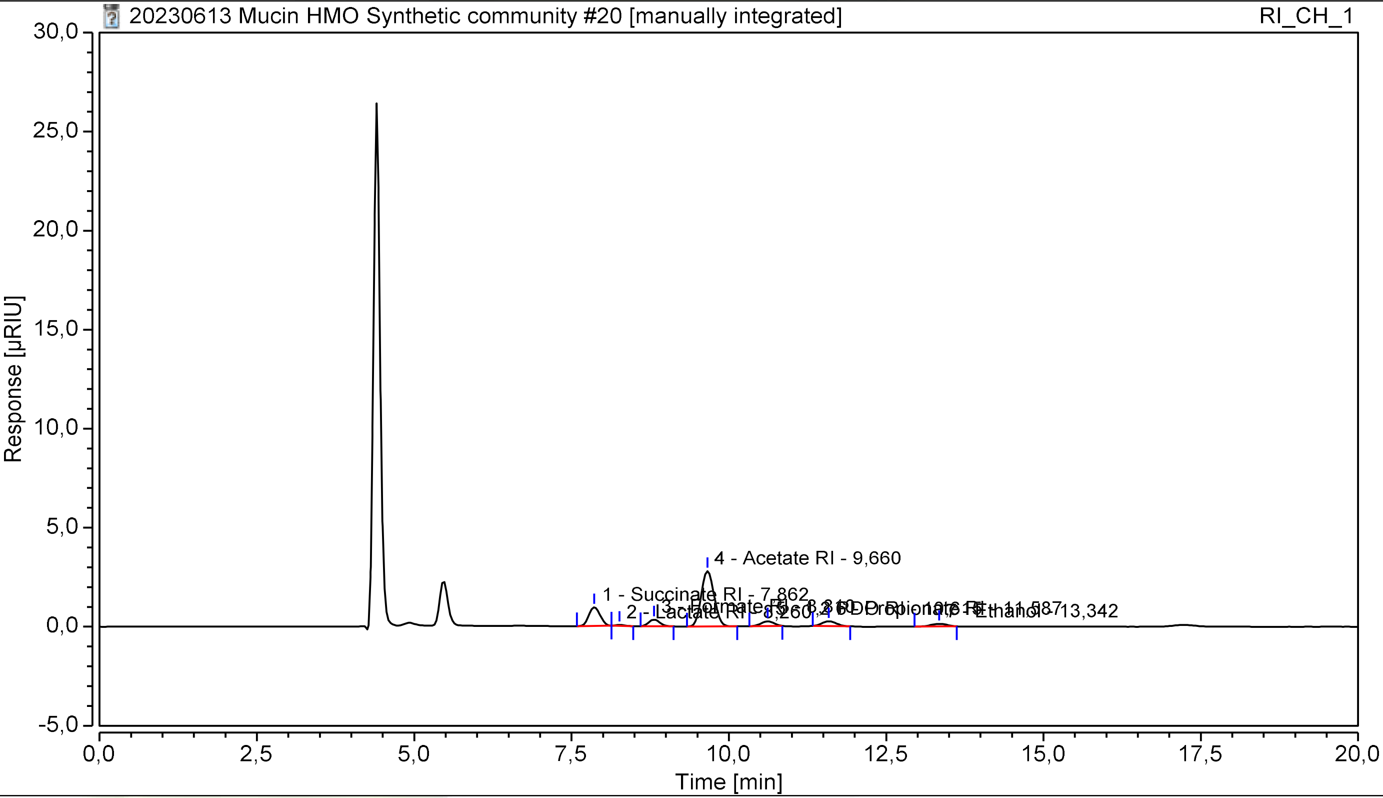

SST Results

No.	Name	Inj.Condition	Peak	Test Result	Injection
**Number of executed test cases:**	**n.a.**		**Total Result:**	**Passed**	

Chromatogram

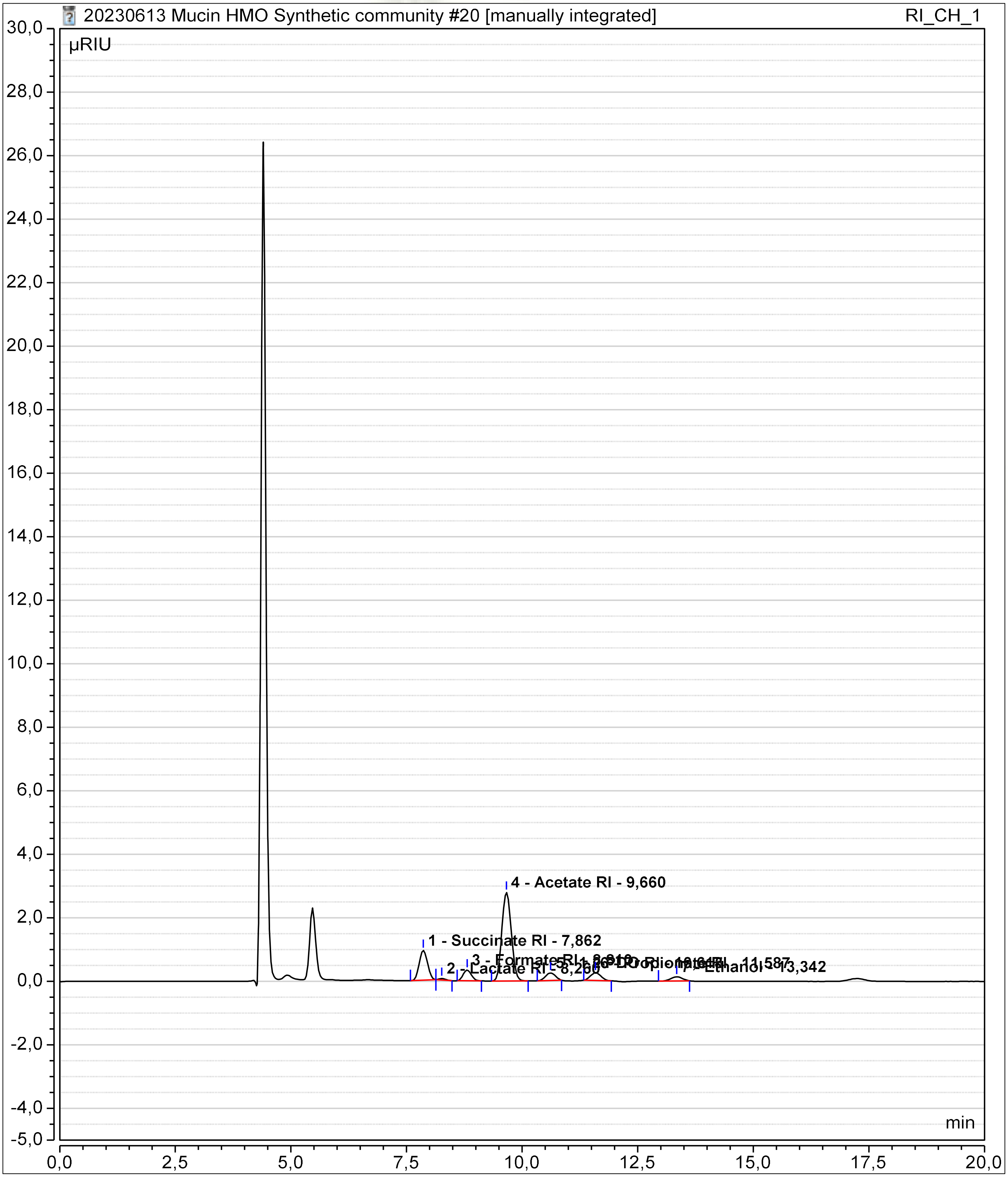

Chromatogram and Results

Injection Details

Injection Name:	**47 5HMO1 t72 r2**	*Run Time (min):*	**20,00**
Vial Number:	**3:B2**	*Injection Volume:*	**10,00**
Injection Type:	**Unknown**	*Channel:*	**RI_CH_1**
Calibration Level:		*Wavelength:*	**n.a.**
Instrument Method:	**Default method LC2030C 45 gr 20 min**	*Bandwidth:*	**n.a.**
Processing Method:	**Processing Method LC2030 45 gr**	*Dilution Factor:*	**1,0000**
Injection Date/Time:	**13-jun-23 18:45**	*Sample Weight:*	**1,0000**

Chromatogram

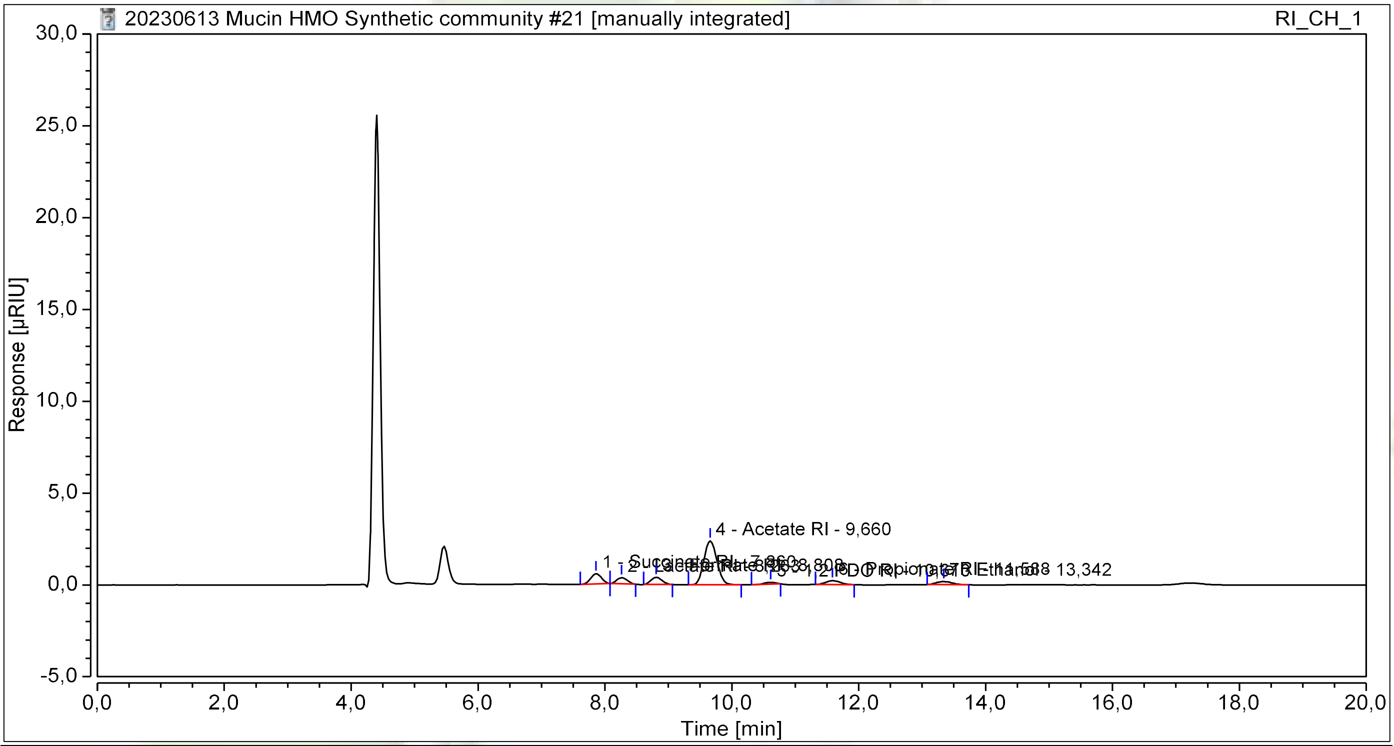

Integration Results

No.	Peak Name	Retention Time min	Area µRIU*min	Height µRIU	Relative Area %	Relative Height %	Amount
n.a.	GlcNAc	n.a.	n.a.	n.a.	n.a.	n.a.	n.a.
n.a.	Citrate	n.a.	n.a.	n.a.	n.a.	n.a.	n.a.
n.a.	Glucose	n.a.	n.a.	n.a.	n.a.	n.a.	n.a.
n.a.	Galactose	n.a.	n.a.	n.a.	n.a.	n.a.	n.a.
n.a.	Fucose	n.a.	n.a.	n.a.	n.a.	n.a.	n.a.
1	Succinate RI	7,860	0,110	0,552	11,69	13,36	n.a.
2	Lactate RI	8,263	0,064	0,331	6,78	8,01	1,8591
n.a.	glycerol	n.a.	n.a.	n.a.	n.a.	n.a.	n.a.
3	Formate RI	8,808	0,079	0,390	8,39	9,44	8,3151
4	Acetate RI	9,660	0,566	2,384	60,00	57,77	34,8635
5	1,2 PDO RI	10,610	0,020	0,095	2,08	2,30	0,5834
n.a.	1,3-PDO	n.a.	n.a.	n.a.	n.a.	n.a.	n.a.
6	Propionate RI	11,588	0,055	0,210	5,84	5,09	2,2163
n.a.	1,3-PDO	n.a.	n.a.	n.a.	n.a.	n.a.	n.a.
n.a.	2-3 BDO	n.a.	n.a.	n.a.	n.a.	n.a.	n.a.
7	Ethanol	13,342	0,049	0,167	5,22	4,03	0,4539
n.a.	Isobutyrate RI	n.a.	n.a.	n.a.	n.a.	n.a.	n.a.
n.a.	Butyrate RI	n.a.	n.a.	n.a.	n.a.	n.a.	n.a.
**Total:**			**0,944**	**4,128**	**100,00**	**100,00**	

Peak Analysis

Injection Details

Injection Name:	**47 5HMO1 t72 r2**	*Run Time (min):*	**20,00**
Vial Number:	**3:B2**	*Injection Volume:*	**10,00**
Injection Type:	**Unknown**	*Channel:*	**RI_CH_1**
Calibration Level:		*Wavelength:*	**n.a.**
Instrument Method:	**Default method LC2030C 45 gr 20 min**	*Bandwidth:*	**n.a.**
Processing Method:	**Processing Method LC2030 45 gr**	*Dilution Factor:*	**1,0000**
Injection Date/Time:	**13-jun-23 18:45**	*Sample Weight:*	**1,0000**

Chromatogram

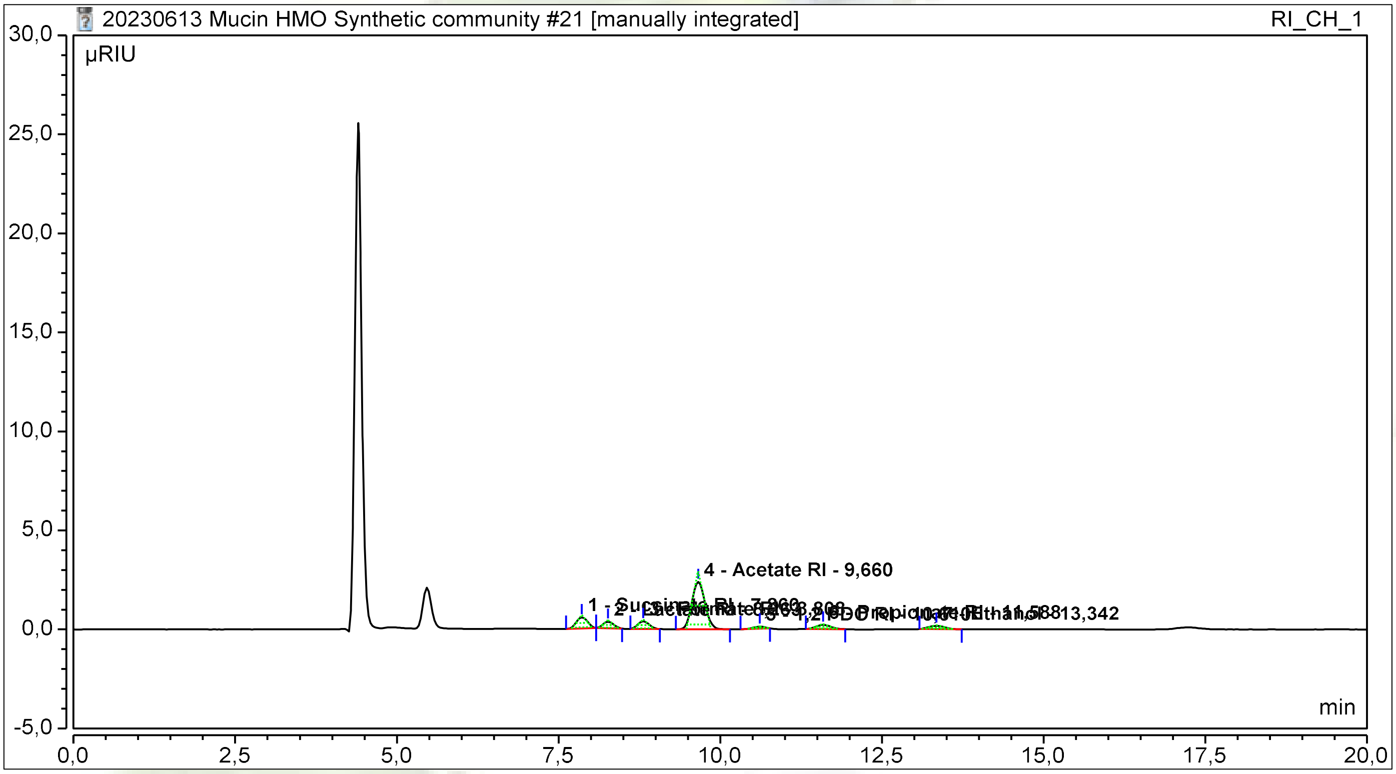

Peak Results

No.	Peak Name	Retention Time min	Width (50%) min	Type	Resolution (EP)	Asymmetry (EP)	Plates (EP)
n.a.	GlcNAc	n.a.	n.a.	n.a.	n.a.	n.a.	n.a.
n.a.	Citrate	n.a.	n.a.	n.a.	n.a.	n.a.	n.a.
n.a.	Glucose	n.a.	n.a.	n.a.	n.a.	n.a.	n.a.
n.a.	Galactose	n.a.	n.a.	n.a.	n.a.	n.a.	n.a.
n.a.	Fucose	n.a.	n.a.	n.a.	n.a.	n.a.	n.a.
1	Succinate RI	7,860	0,194	BMB	1,24	1,01	9094
2	Lactate RI	8,263	0,190	BMB*	1,67	1,12	10534
n.a.	glycerol	n.a.	n.a.	n.a.	n.a.	n.a.	n.a.
3	Formate RI	8,808	0,196	BMB*	2,39	1,10	11162
4	Acetate RI	9,660	0,225	BMB	2,58	1,08	10234
5	1,2 PDO RI	10,610	0,209	BMB*	2,49	0,88	14267
n.a.	1,3-PDO	n.a.	n.a.	n.a.	n.a.	n.a.	n.a.
6	Propionate RI	11,588	0,254	BMB*	3,81	1,09	11555
n.a.	1,3-PDO	n.a.	n.a.	n.a.	n.a.	n.a.	n.a.
n.a.	2-3 BDO	n.a.	n.a.	n.a.	n.a.	n.a.	n.a.
7	Ethanol	13,342	0,289	BMB*	n.a.	1,12	11825
n.a.	Isobutyrate RI	n.a.	n.a.	n.a.	n.a.	n.a.	n.a.
n.a.	Butyrate RI	n.a.	n.a.	n.a.	n.a.	n.a.	n.a.

Chromeleon (c) Dionex
Version 7.2.10.25868

Chromatogram and SST Results

Injection Details

Injection Name:	**47 5HMO1 t72 r2**	*Run Time (min):*	**20,00**
Vial Number:	**3:B2**	*Injection Volume:*	**10,00**
Injection Type:	**Unknown**	*Channel:*	**RI_CH_1**
Calibration Level:		*Wavelength:*	**n.a.**
Instrument Method:	**Default method LC2030C 45 gr 20 min**	*Bandwidth:*	**n.a.**
Processing Method:	**Processing Method LC2030 45 gr**	*Dilution Factor:*	**1,0000**
Injection Date/Time:	**13-jun-23 18:45**	*Sample Weight:*	**1,0000**

Chromatogram

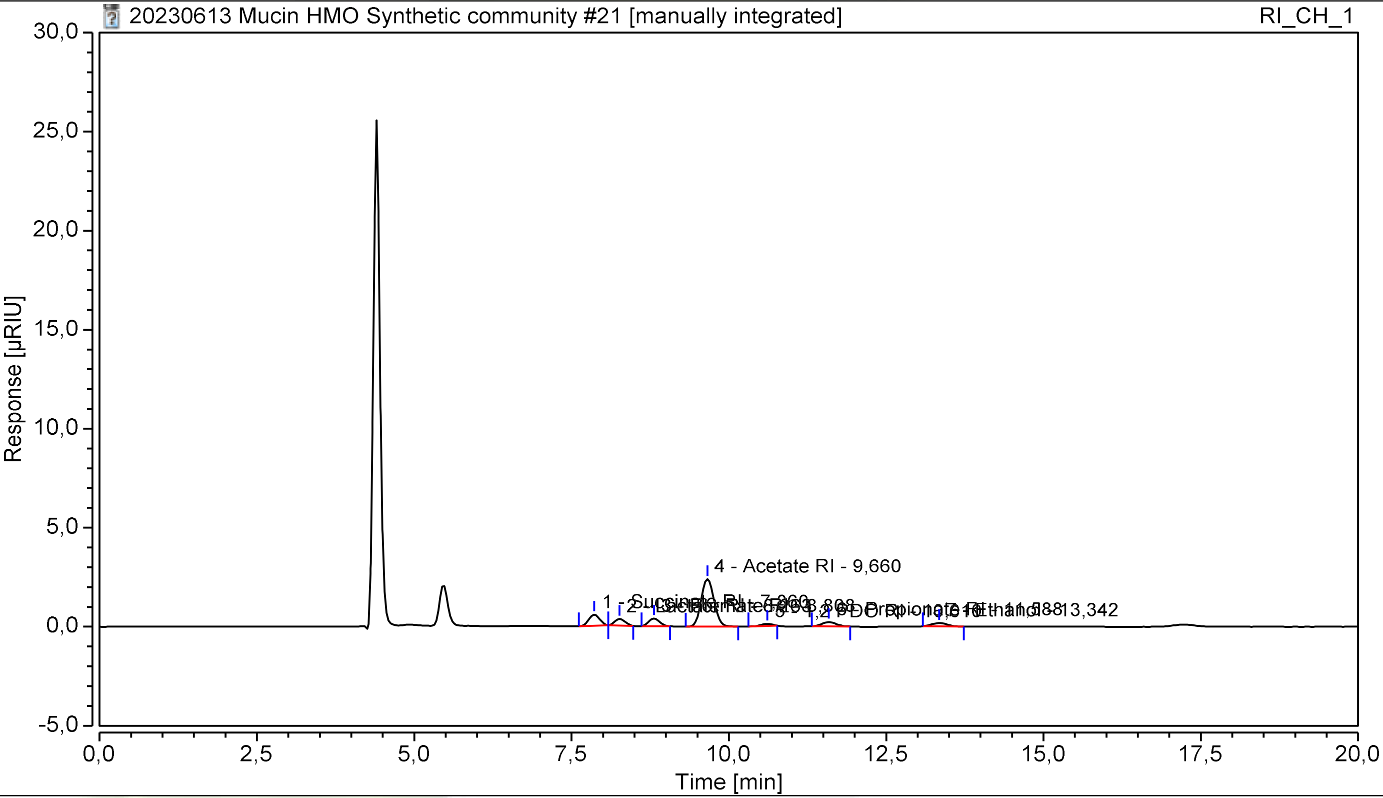

SST Results

No.	Name	Inj.Condition	Peak	Test Result	Injection
**Number of executed test cases:**	**n.a.**		**Total Result:**	**Passed**	

Chromatogram

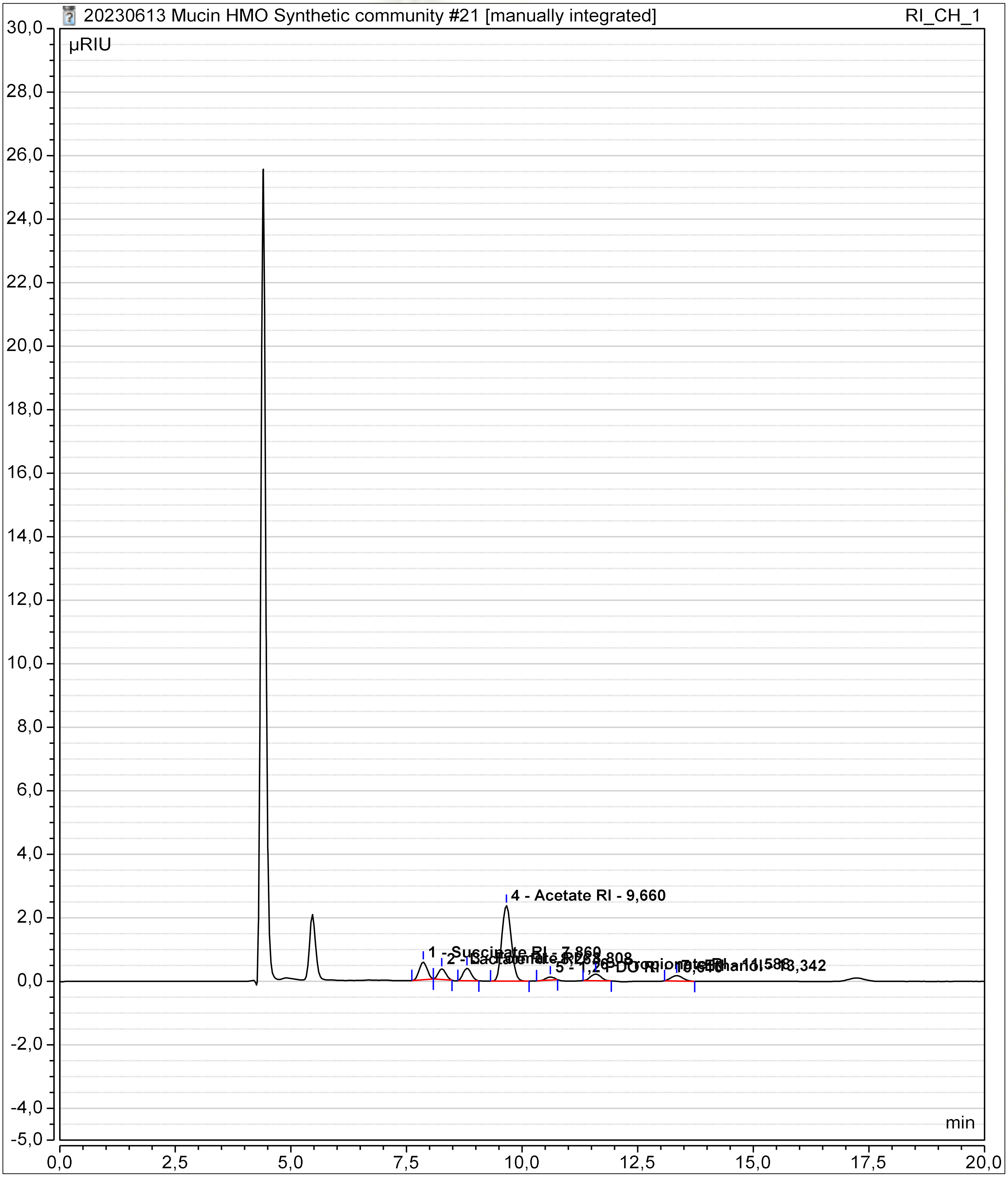

Chromeleon (c) Dionex
Version 7.2.10.25868

Chromatogram and Results

Injection Details

Injection Name:	**48 5HMO1 t72 r3**	*Run Time (min):*	**20,00**
Vial Number:	**3:B3**	*Injection Volume:*	**10,00**
Injection Type:	**Unknown**	*Channel:*	**RI_CH_1**
Calibration Level:		*Wavelength:*	**n.a.**
Instrument Method:	**Default method LC2030C 45 gr 20 min**	*Bandwidth:*	**n.a.**
Processing Method:	**Processing Method LC2030 45 gr**	*Dilution Factor:*	**1,0000**
Injection Date/Time:	**13-jun-23 19:05**	*Sample Weight:*	**1,0000**

Chromatogram

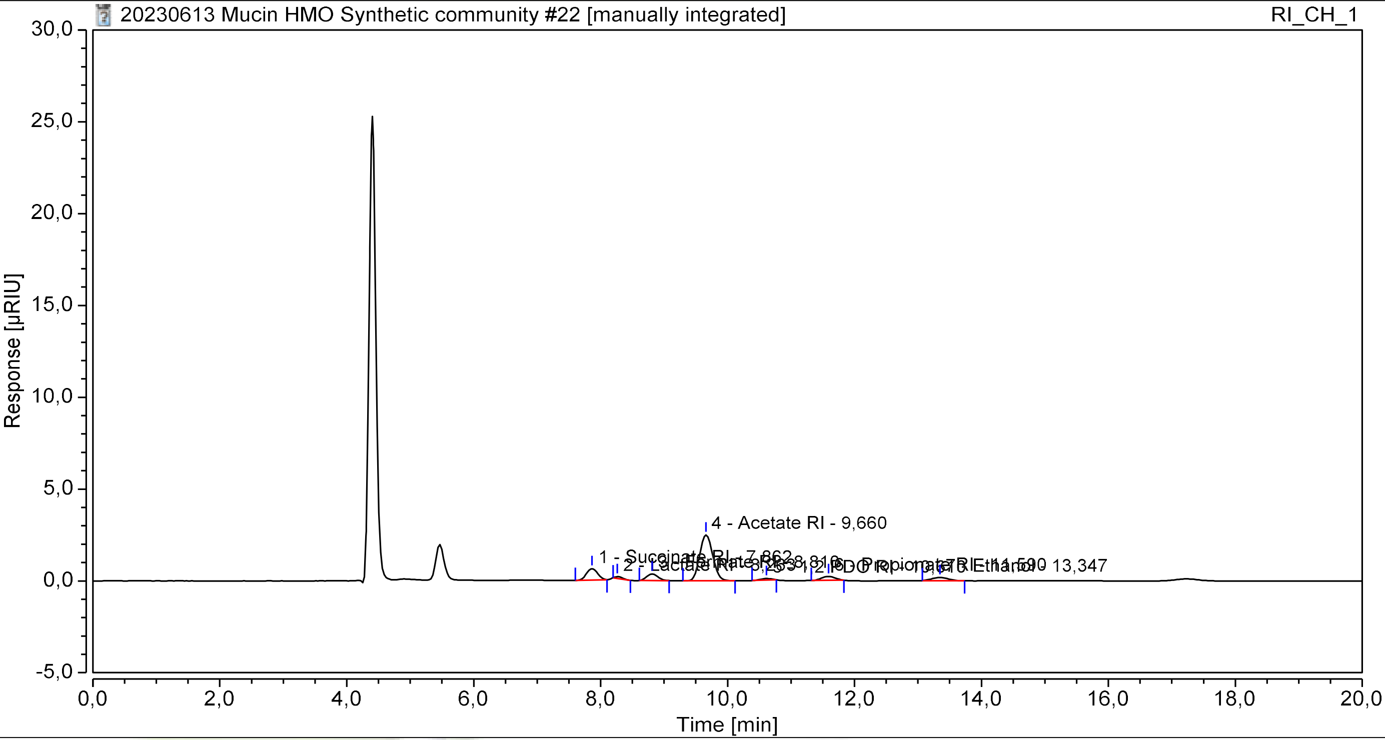

Integration Results

No.	Peak Name	Retention Time<br>min	Area<br>µRIU*min	Height<br>µRIU	Relative Area<br>%	Relative Height<br>%	Amount
n.a.	GlcNAc	n.a.	n.a.	n.a.	n.a.	n.a.	n.a.
n.a.	Citrate	n.a.	n.a.	n.a.	n.a.	n.a.	n.a.
n.a.	Glucose	n.a.	n.a.	n.a.	n.a.	n.a.	n.a.
n.a.	Galactose	n.a.	n.a.	n.a.	n.a.	n.a.	n.a.
n.a.	Fucose	n.a.	n.a.	n.a.	n.a.	n.a.	n.a.
1	Succinate RI	7,862	0,126	0,622	13,64	15,43	n.a.
2	Lactate RI	8,263	0,013	0,089	1,37	2,21	0,3688
n.a.	glycerol	n.a.	n.a.	n.a.	n.a.	n.a.	n.a.
3	Formate RI	8,810	0,074	0,361	7,96	8,95	7,7467
4	Acetate RI	9,660	0,592	2,493	63,86	61,84	36,4180
5	1,2 PDO RI	10,610	0,018	0,085	1,92	2,12	0,5293
n.a.	1,3-PDO	n.a.	n.a.	n.a.	n.a.	n.a.	n.a.
6	Propionate RI	11,590	0,053	0,211	5,69	5,23	2,1228
n.a.	1,3-PDO	n.a.	n.a.	n.a.	n.a.	n.a.	n.a.
n.a.	2-3 BDO	n.a.	n.a.	n.a.	n.a.	n.a.	n.a.
7	Ethanol	13,347	0,051	0,171	5,55	4,23	0,4736
n.a.	Isobutyrate RI	n.a.	n.a.	n.a.	n.a.	n.a.	n.a.
n.a.	Butyrate RI	n.a.	n.a.	n.a.	n.a.	n.a.	n.a.
**Total:**			**0,927**	**4,031**	**100,00**	**100,00**	

Peak Analysis

Injection Details

Injection Name:	**48 5HMO1 t72 r3**	*Run Time (min):*	**20,00**
Vial Number:	**3:B3**	*Injection Volume:*	**10,00**
Injection Type:	**Unknown**	*Channel:*	**RI_CH_1**
Calibration Level:		*Wavelength:*	**n.a.**
Instrument Method:	**Default method LC2030C 45 gr 20 min**	*Bandwidth:*	**n.a.**
Processing Method:	**Processing Method LC2030 45 gr**	*Dilution Factor:*	**1,0000**
Injection Date/Time:	**13-jun-23 19:05**	*Sample Weight:*	**1,0000**

Chromatogram

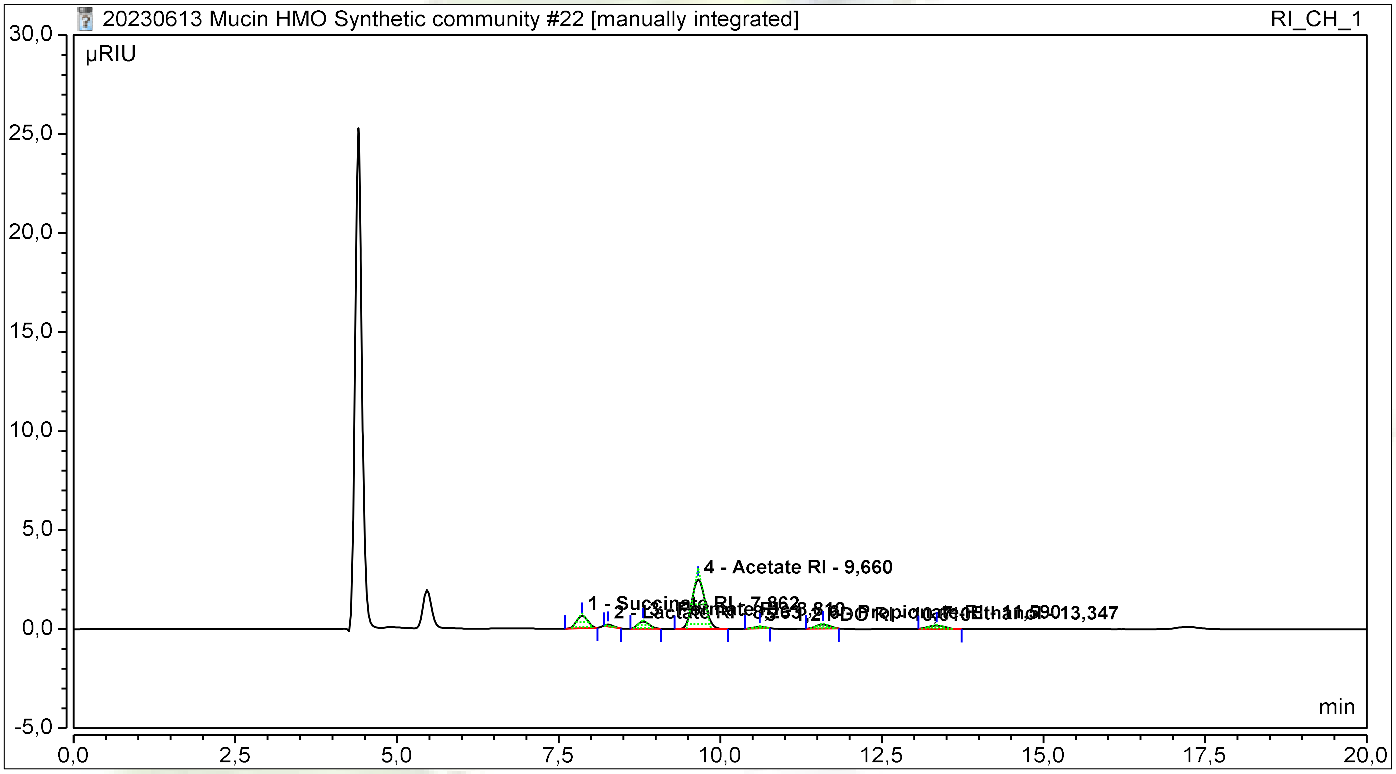

Peak Results

No.	Peak Name	Retention Time min	Width (50%) min	Type	Resolution (EP)	Asymmetry (EP)	Plates (EP)
n.a.	GlcNAc	n.a.	n.a.	n.a.	n.a.	n.a.	n.a.
n.a.	Citrate	n.a.	n.a.	n.a.	n.a.	n.a.	n.a.
n.a.	Glucose	n.a.	n.a.	n.a.	n.a.	n.a.	n.a.
n.a.	Galactose	n.a.	n.a.	n.a.	n.a.	n.a.	n.a.
n.a.	Fucose	n.a.	n.a.	n.a.	n.a.	n.a.	n.a.
1	Succinate RI	7,862	0,196	BMB	1,41	1,03	8901
2	Lactate RI	8,263	0,140	BMB*	1,91	1,79	19242
n.a.	glycerol	n.a.	n.a.	n.a.	n.a.	n.a.	n.a.
3	Formate RI	8,810	0,197	BMB*	2,38	1,11	11086
4	Acetate RI	9,660	0,225	BMB	2,57	1,09	10246
5	1,2 PDO RI	10,610	0,211	BMB*	2,53	0,88	13995
n.a.	1,3-PDO	n.a.	n.a.	n.a.	n.a.	n.a.	n.a.
6	Propionate RI	11,590	0,246	BMB*	3,85	1,00	12292
n.a.	1,3-PDO	n.a.	n.a.	n.a.	n.a.	n.a.	n.a.
n.a.	2-3 BDO	n.a.	n.a.	n.a.	n.a.	n.a.	n.a.
7	Ethanol	13,347	0,293	BMB*	n.a.	1,09	11525
n.a.	Isobutyrate RI	n.a.	n.a.	n.a.	n.a.	n.a.	n.a.
n.a.	Butyrate RI	n.a.	n.a.	n.a.	n.a.	n.a.	n.a.

Chromatogram and SST Results

Injection Details

Injection Name:	**48 5HMO1 t72 r3**	*Run Time (min):*	**20,00**
Vial Number:	**3:B3**	*Injection Volume:*	**10,00**
Injection Type:	**Unknown**	*Channel:*	**RI_CH_1**
Calibration Level:		*Wavelength:*	**n.a.**
Instrument Method:	**Default method LC2030C 45 gr 20 min**	*Bandwidth:*	**n.a.**
Processing Method:	**Processing Method LC2030 45 gr**	*Dilution Factor:*	**1,0000**
Injection Date/Time:	**13-jun-23 19:05**	*Sample Weight:*	**1,0000**

Chromatogram

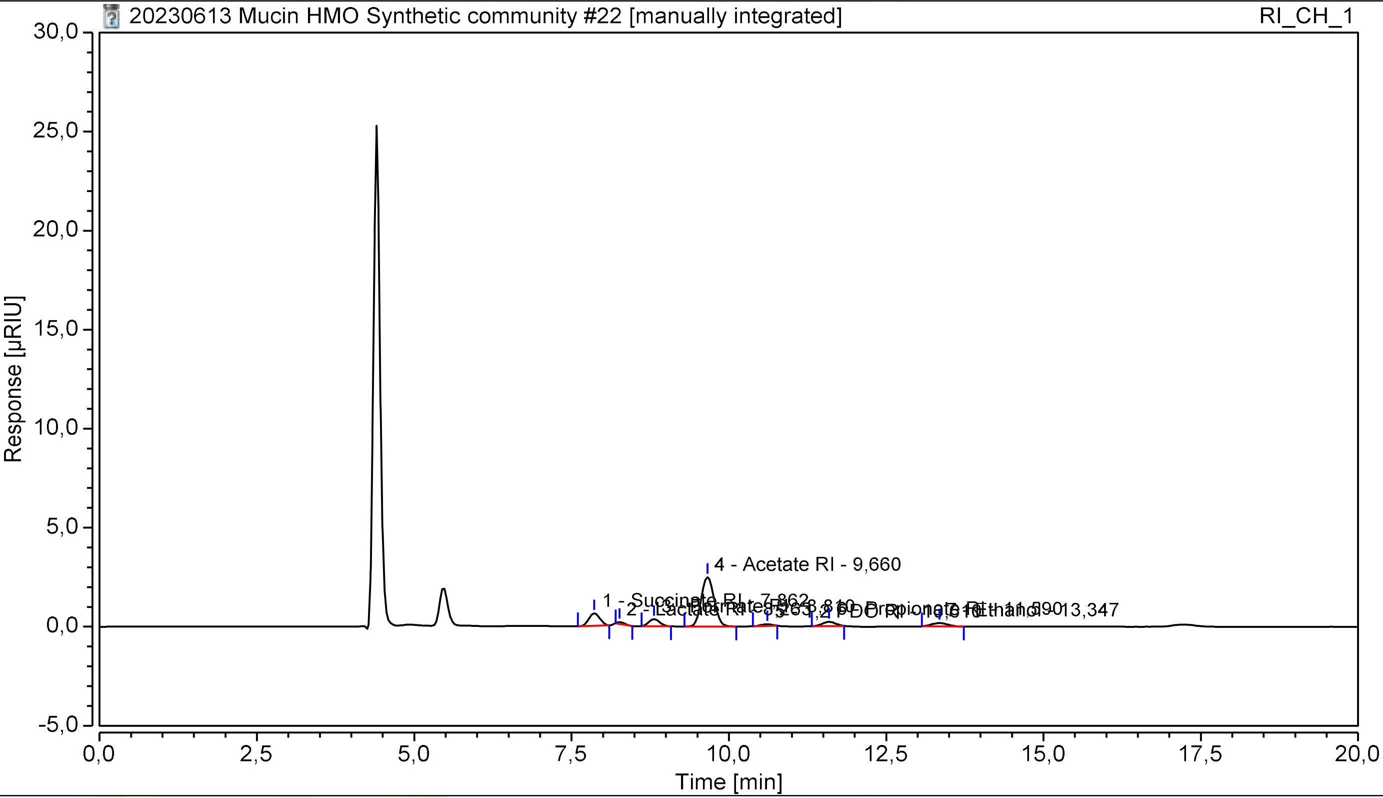

SST Results

No.	Name	Inj.Condition	Peak	Test Result	Injection
**Number of executed test cases:**	**n.a.**		**Total Result:**	**Passed**	

Chromatogram

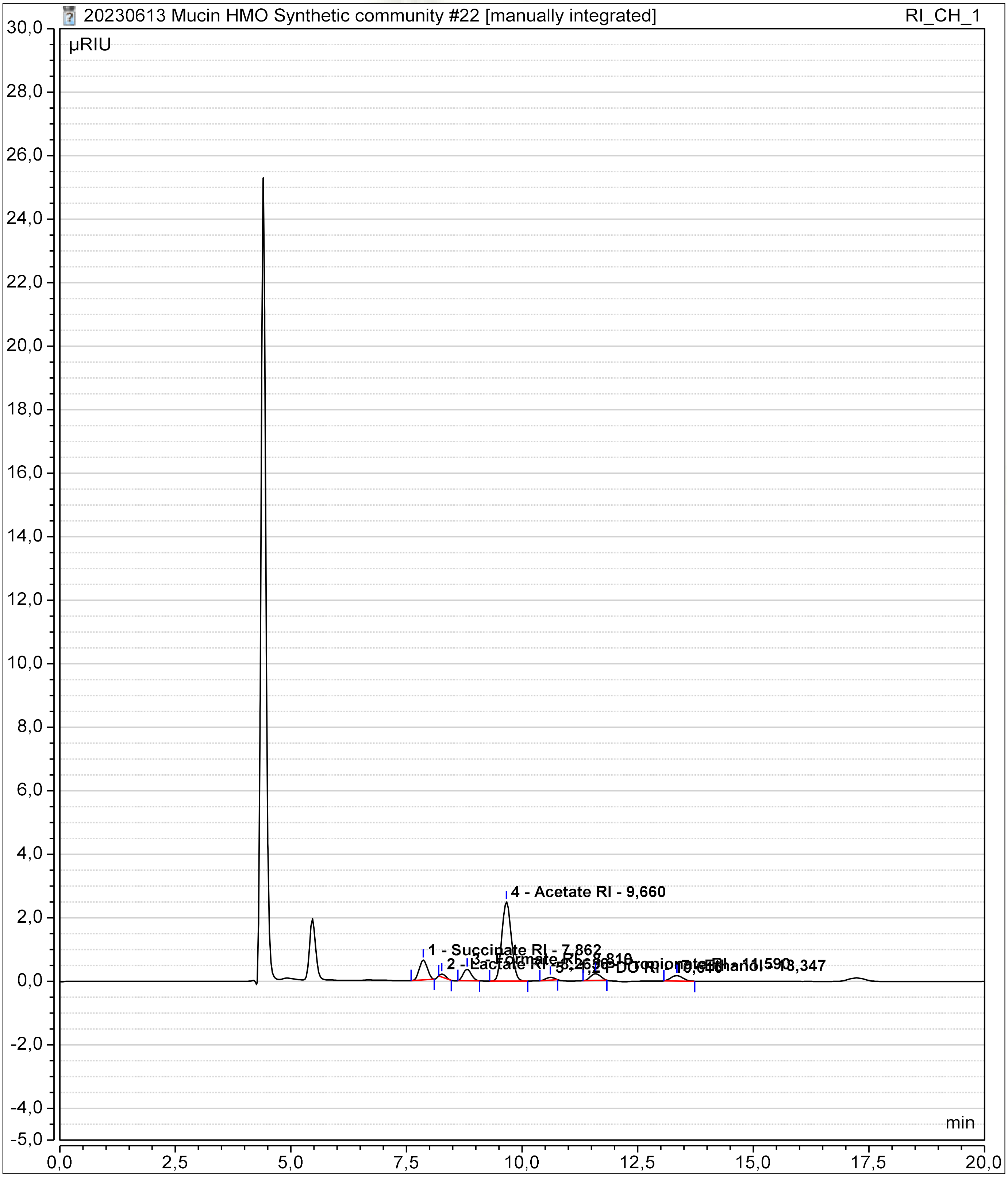

Chromeleon (c) Dionex
Version 7.2.10.25868

Chromatogram and Results

Injection Details

Injection Name:	**49 5HMO1 t96 r1**	*Run Time (min):*	**20,00**
Vial Number:	**3:B4**	*Injection Volume:*	**10,00**
Injection Type:	**Unknown**	*Channel:*	**RI_CH_1**
Calibration Level:		*Wavelength:*	**n.a.**
Instrument Method:	**Default method LC2030C 45 gr 20 min**	*Bandwidth:*	**n.a.**
Processing Method:	**Processing Method LC2030 45 gr**	*Dilution Factor:*	**1,0000**
Injection Date/Time:	**13-jun-23 19:26**	*Sample Weight:*	**1,0000**

Chromatogram

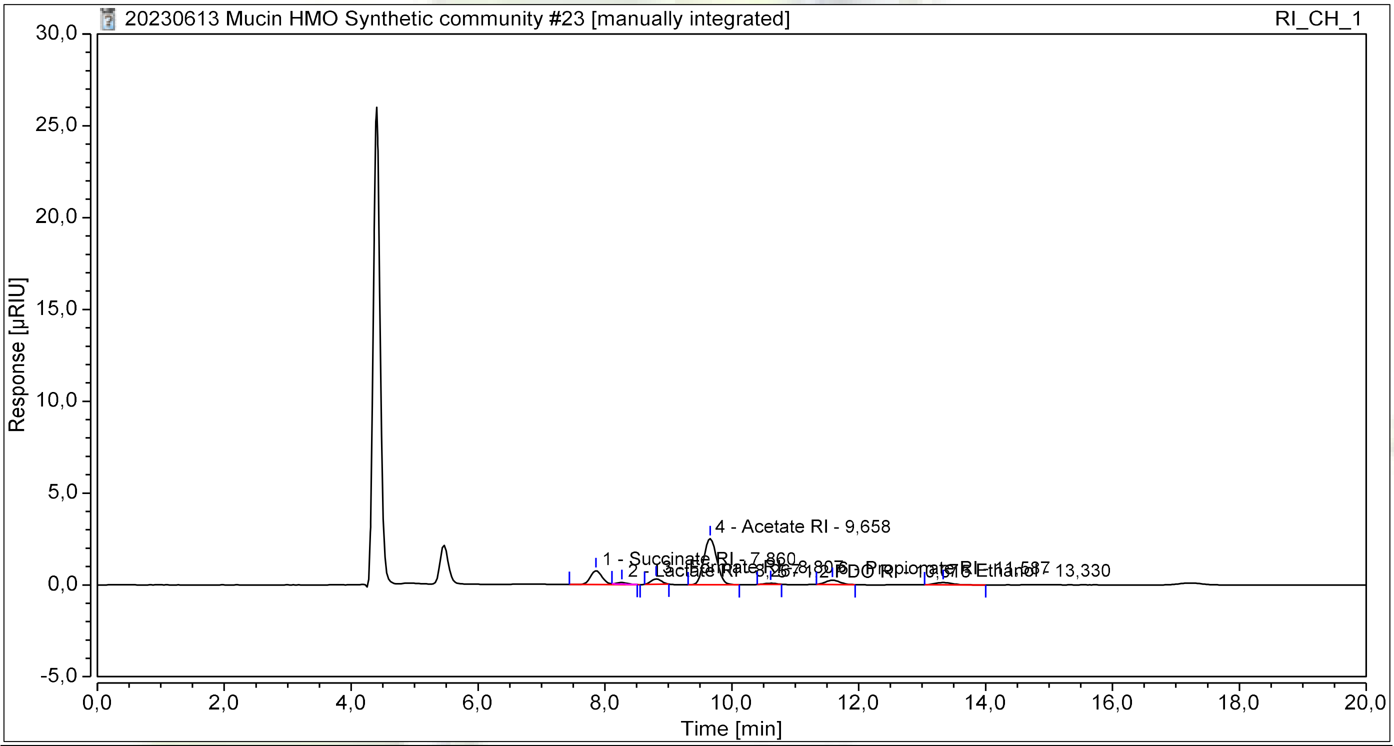

Integration Results

No.	Peak Name	Retention Time min	Area µRIU*min	Height µRIU	Relative Area %	Relative Height %	Amount
n.a.	GlcNAc	n.a.	n.a.	n.a.	n.a.	n.a.	n.a.
n.a.	Citrate	n.a.	n.a.	n.a.	n.a.	n.a.	n.a.
n.a.	Glucose	n.a.	n.a.	n.a.	n.a.	n.a.	n.a.
n.a.	Galactose	n.a.	n.a.	n.a.	n.a.	n.a.	n.a.
n.a.	Fucose	n.a.	n.a.	n.a.	n.a.	n.a.	n.a.
1	Succinate RI	7,860	0,170	0,751	17,95	18,58	n.a.
2	Lactate RI	8,267	0,015	0,084	1,61	2,09	0,4436
n.a.	glycerol	n.a.	n.a.	n.a.	n.a.	n.a.	n.a.
3	Formate RI	8,807	0,055	0,285	5,83	7,04	5,7901
4	Acetate RI	9,658	0,595	2,508	62,92	62,07	36,6490
5	1,2 PDO RI	10,615	0,013	0,060	1,34	1,48	0,3776
n.a.	1,3-PDO	n.a.	n.a.	n.a.	n.a.	n.a.	n.a.
6	Propionate RI	11,587	0,061	0,231	6,40	5,73	2,4387
n.a.	1,3-PDO	n.a.	n.a.	n.a.	n.a.	n.a.	n.a.
n.a.	2-3 BDO	n.a.	n.a.	n.a.	n.a.	n.a.	n.a.
7	Ethanol	13,330	0,037	0,121	3,94	3,01	0,3436
n.a.	Isobutyrate RI	n.a.	n.a.	n.a.	n.a.	n.a.	n.a.
n.a.	Butyrate RI	n.a.	n.a.	n.a.	n.a.	n.a.	n.a.
**Total:**			**0,946**	**4,040**	**100,00**	**100,00**	

Peak Analysis

Injection Details

Injection Name:	**49 5HMO1 t96 r1**	*Run Time (min):*	**20,00**
Vial Number:	**3:B4**	*Injection Volume:*	**10,00**
Injection Type:	**Unknown**	*Channel:*	**RI_CH_1**
Calibration Level:		*Wavelength:*	**n.a.**
Instrument Method:	**Default method LC2030C 45 gr 20 min**	*Bandwidth:*	**n.a.**
Processing Method:	**Processing Method LC2030 45 gr**	*Dilution Factor:*	**1,0000**
Injection Date/Time:	**13-jun-23 19:26**	*Sample Weight:*	**1,0000**

Chromatogram

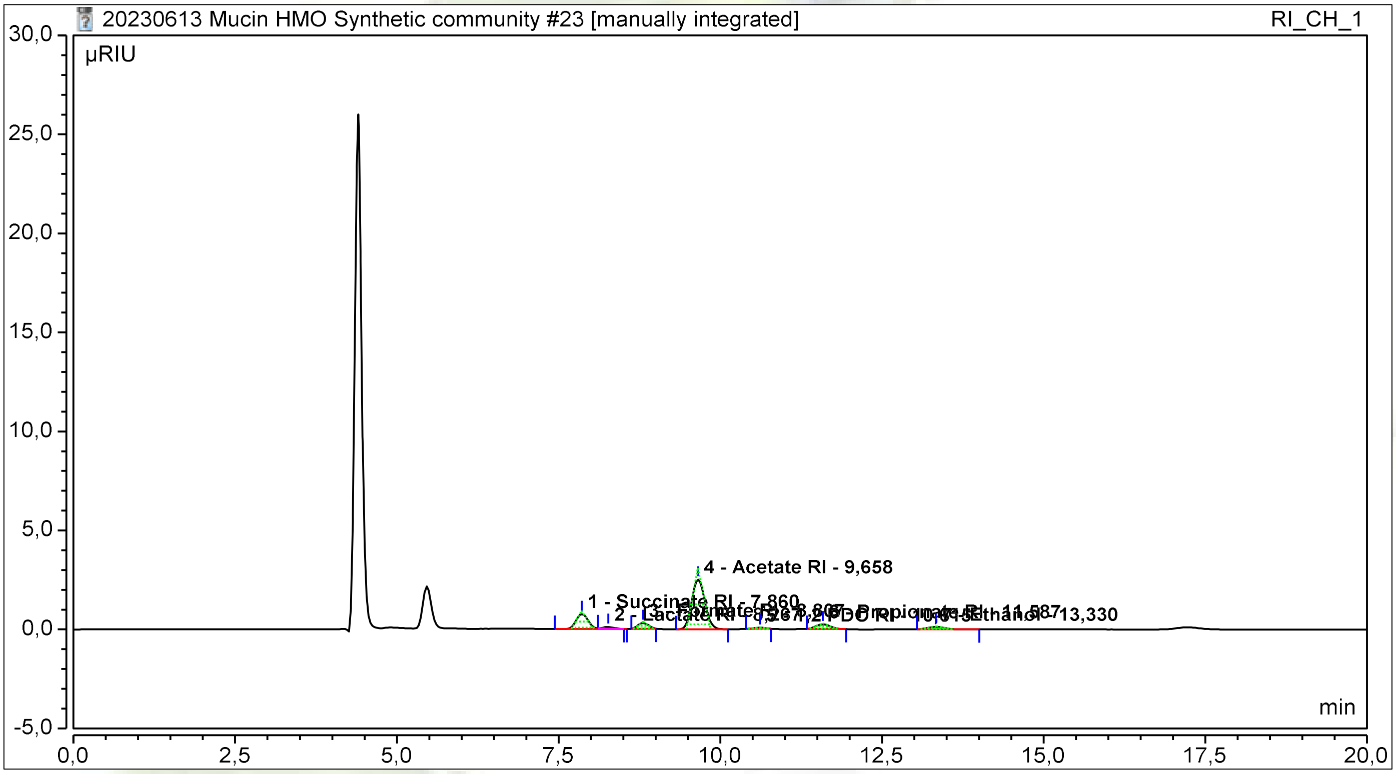

Peak Results

No.	Peak Name	Retention Time min	Width (50%) min	Type	Resolution (EP)	Asymmetry (EP)	Plates (EP)
n.a.	GlcNAc	n.a.	n.a.	n.a.	n.a.	n.a.	n.a.
n.a.	Citrate	n.a.	n.a.	n.a.	n.a.	n.a.	n.a.
n.a.	Glucose	n.a.	n.a.	n.a.	n.a.	n.a.	n.a.
n.a.	Galactose	n.a.	n.a.	n.a.	n.a.	n.a.	n.a.
n.a.	Fucose	n.a.	n.a.	n.a.	n.a.	n.a.	n.a.
1	Succinate RI	7,860	0,202	BMB	2,84	1,21	8351
2	Lactate RI	8,267	n.a.	Rd	n.a.	n.a.	n.a.
n.a.	glycerol	n.a.	n.a.	n.a.	n.a.	n.a.	n.a.
3	Formate RI	8,807	0,190	BMB*	2,42	1,04	11857
4	Acetate RI	9,658	0,225	BMB	2,58	1,08	10232
5	1,2 PDO RI	10,615	0,213	BMB*	2,45	0,90	13712
n.a.	1,3-PDO	n.a.	n.a.	n.a.	n.a.	n.a.	n.a.
6	Propionate RI	11,587	0,254	BMB*	3,73	1,09	11552
n.a.	1,3-PDO	n.a.	n.a.	n.a.	n.a.	n.a.	n.a.
n.a.	2-3 BDO	n.a.	n.a.	n.a.	n.a.	n.a.	n.a.
7	Ethanol	13,330	0,298	BMB*	n.a.	1,12	11053
n.a.	Isobutyrate RI	n.a.	n.a.	n.a.	n.a.	n.a.	n.a.
n.a.	Butyrate RI	n.a.	n.a.	n.a.	n.a.	n.a.	n.a.

Chromatogram and SST Results

Injection Details

Injection Name:	**49 5HMO1 t96 r1**	*Run Time (min):*	**20,00**
Vial Number:	**3:B4**	*Injection Volume:*	**10,00**
Injection Type:	**Unknown**	*Channel:*	**RI_CH_1**
Calibration Level:		*Wavelength:*	**n.a.**
Instrument Method:	**Default method LC2030C 45 gr 20 min**	*Bandwidth:*	**n.a.**
Processing Method:	**Processing Method LC2030 45 gr**	*Dilution Factor:*	**1,0000**
Injection Date/Time:	**13-jun-23 19:26**	*Sample Weight:*	**1,0000**

Chromatogram

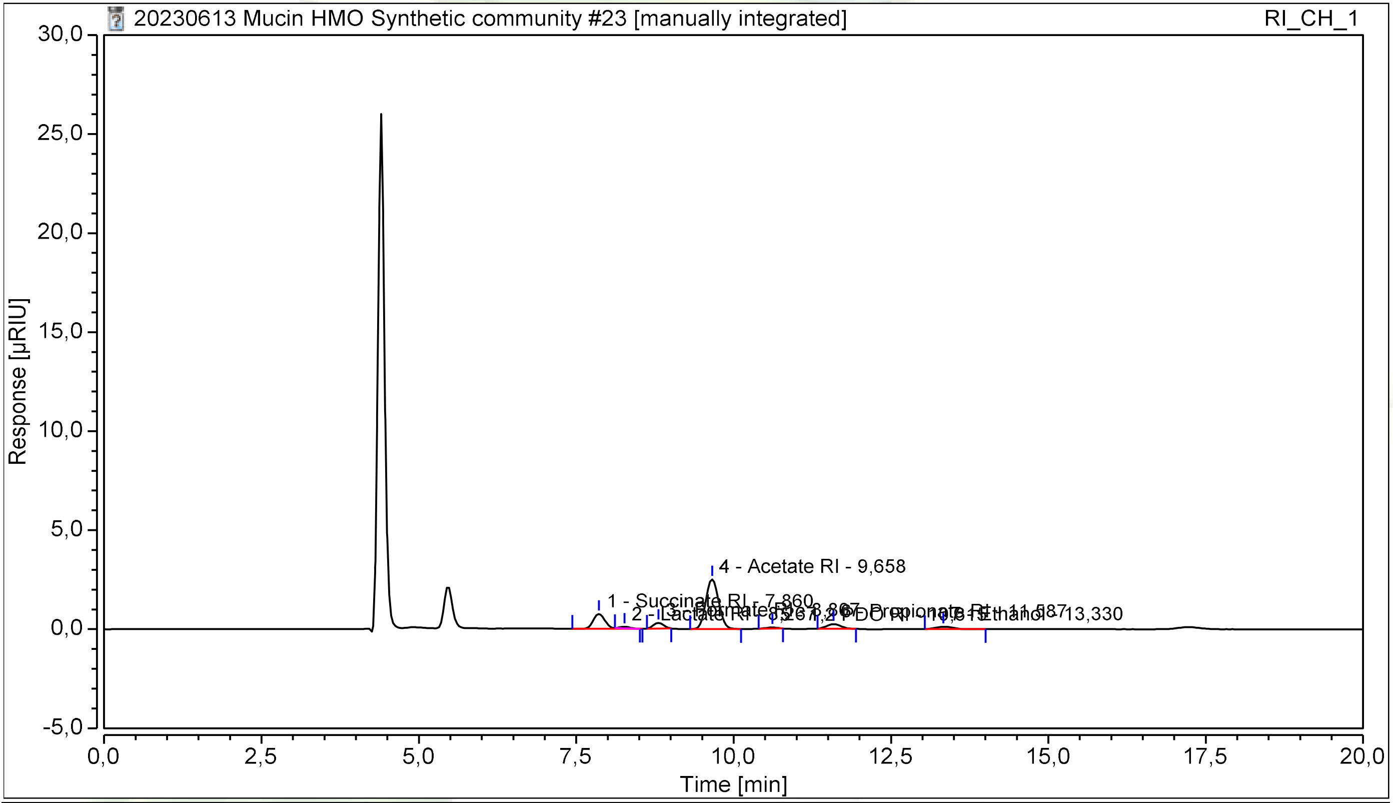

SST Results

No.	Name	Inj.Condition	Peak	Test Result	Injection
**Number of executed test cases:**	**n.a.**		**Total Result:**	**Passed**	

Chromatogram

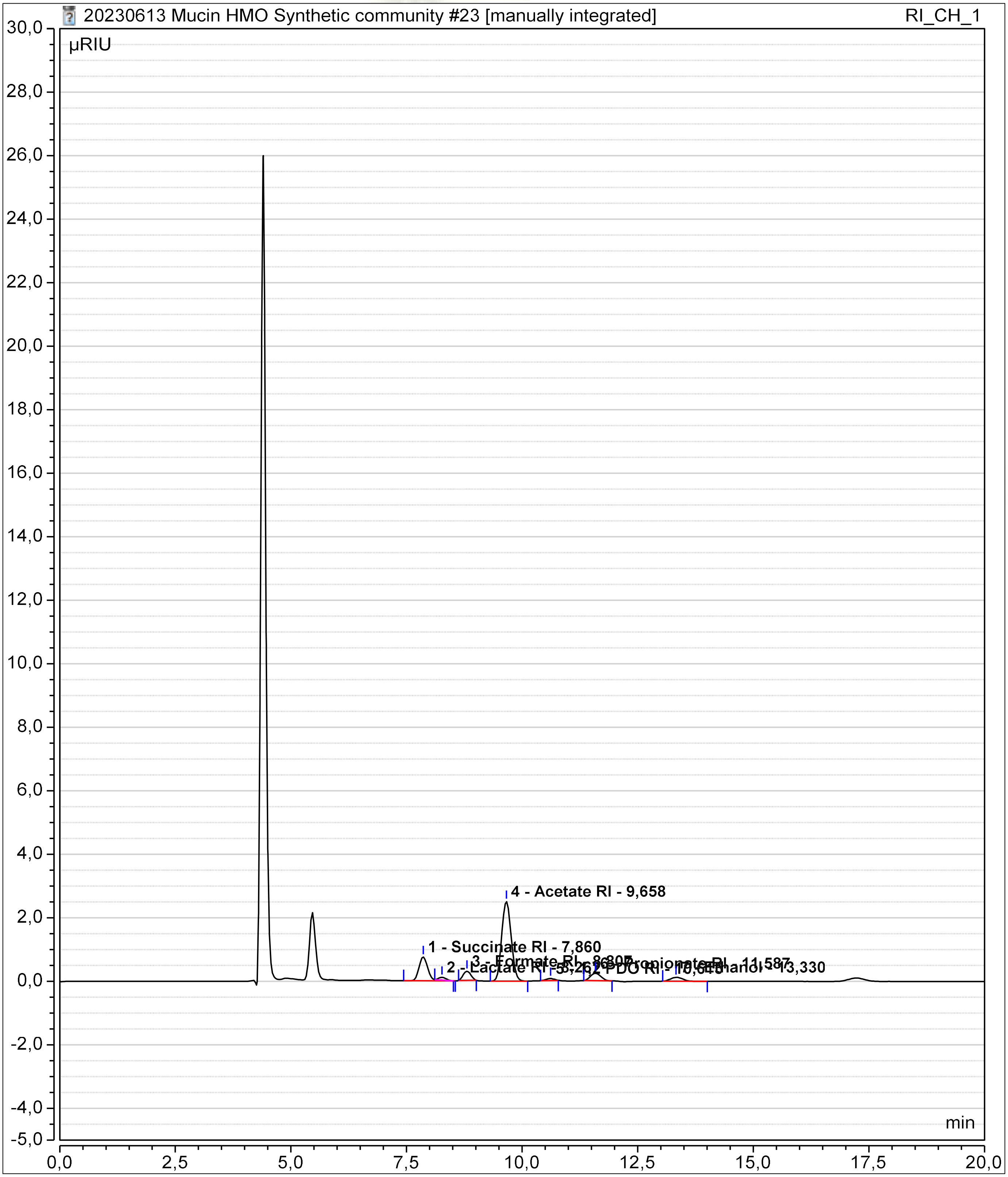

Chromeleon (c) Dionex
Version 7.2.10.25868

Chromatogram and Results

Injection Details

Injection Name:	**50 5HMO1 t96 r2**	*Run Time (min):*	**20,00**
Vial Number:	**3:B5**	*Injection Volume:*	**10,00**
Injection Type:	**Unknown**	*Channel:*	**RI_CH_1**
Calibration Level:		*Wavelength:*	**n.a.**
Instrument Method:	**Default method LC2030C 45 gr 20 min**	*Bandwidth:*	**n.a.**
Processing Method:	**Processing Method LC2030 45 gr**	*Dilution Factor:*	**1,0000**
Injection Date/Time:	**13-jun-23 19:46**	*Sample Weight:*	**1,0000**

Chromatogram

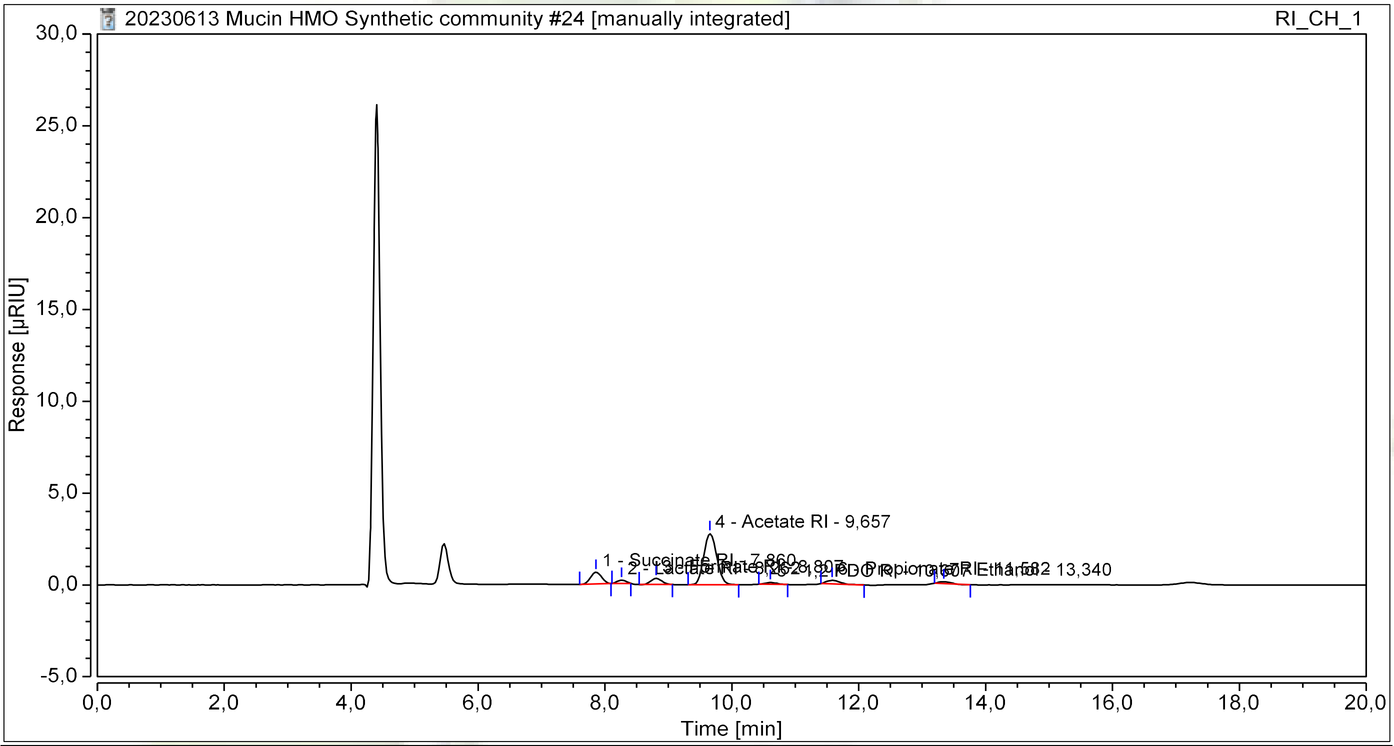

Integration Results

No.	Peak Name	Retention Time min	Area µRIU*min	Height µRIU	Relative Area %	Relative Height %	Amount
n.a.	GlcNAc	n.a.	n.a.	n.a.	n.a.	n.a.	n.a.
n.a.	Citrate	n.a.	n.a.	n.a.	n.a.	n.a.	n.a.
n.a.	Glucose	n.a.	n.a.	n.a.	n.a.	n.a.	n.a.
n.a.	Galactose	n.a.	n.a.	n.a.	n.a.	n.a.	n.a.
n.a.	Fucose	n.a.	n.a.	n.a.	n.a.	n.a.	n.a.
1	Succinate RI	7,860	0,130	0,640	13,26	14,82	n.a.
2	Lactate RI	8,262	0,029	0,174	2,97	4,02	0,8424
n.a.	glycerol	n.a.	n.a.	n.a.	n.a.	n.a.	n.a.
3	Formate RI	8,807	0,068	0,330	6,91	7,65	7,0980
4	Acetate RI	9,657	0,660	2,779	67,44	64,37	40,5969
5	1,2 PDO RI	10,607	0,023	0,098	2,31	2,26	0,6716
n.a.	1,3-PDO	n.a.	n.a.	n.a.	n.a.	n.a.	n.a.
6	Propionate RI	11,582	0,047	0,195	4,83	4,51	1,9013
n.a.	1,3-PDO	n.a.	n.a.	n.a.	n.a.	n.a.	n.a.
n.a.	2-3 BDO	n.a.	n.a.	n.a.	n.a.	n.a.	n.a.
7	Ethanol	13,340	0,022	0,103	2,28	2,38	0,2053
n.a.	Isobutyrate RI	n.a.	n.a.	n.a.	n.a.	n.a.	n.a.
n.a.	Butyrate RI	n.a.	n.a.	n.a.	n.a.	n.a.	n.a.
**Total:**			**0,978**	**4,318**	**100,00**	**100,00**	

Peak Analysis

Injection Details

Injection Name:	**50 5HMO1 t96 r2**	*Run Time (min):*	**20,00**
Vial Number:	**3:B5**	*Injection Volume:*	**10,00**
Injection Type:	**Unknown**	*Channel:*	**RI_CH_1**
Calibration Level:		*Wavelength:*	**n.a.**
Instrument Method:	**Default method LC2030C 45 gr 20 min**	*Bandwidth:*	**n.a.**
Processing Method:	**Processing Method LC2030 45 gr**	*Dilution Factor:*	**1,0000**
Injection Date/Time:	**13-jun-23 19:46**	*Sample Weight:*	**1,0000**

Chromatogram

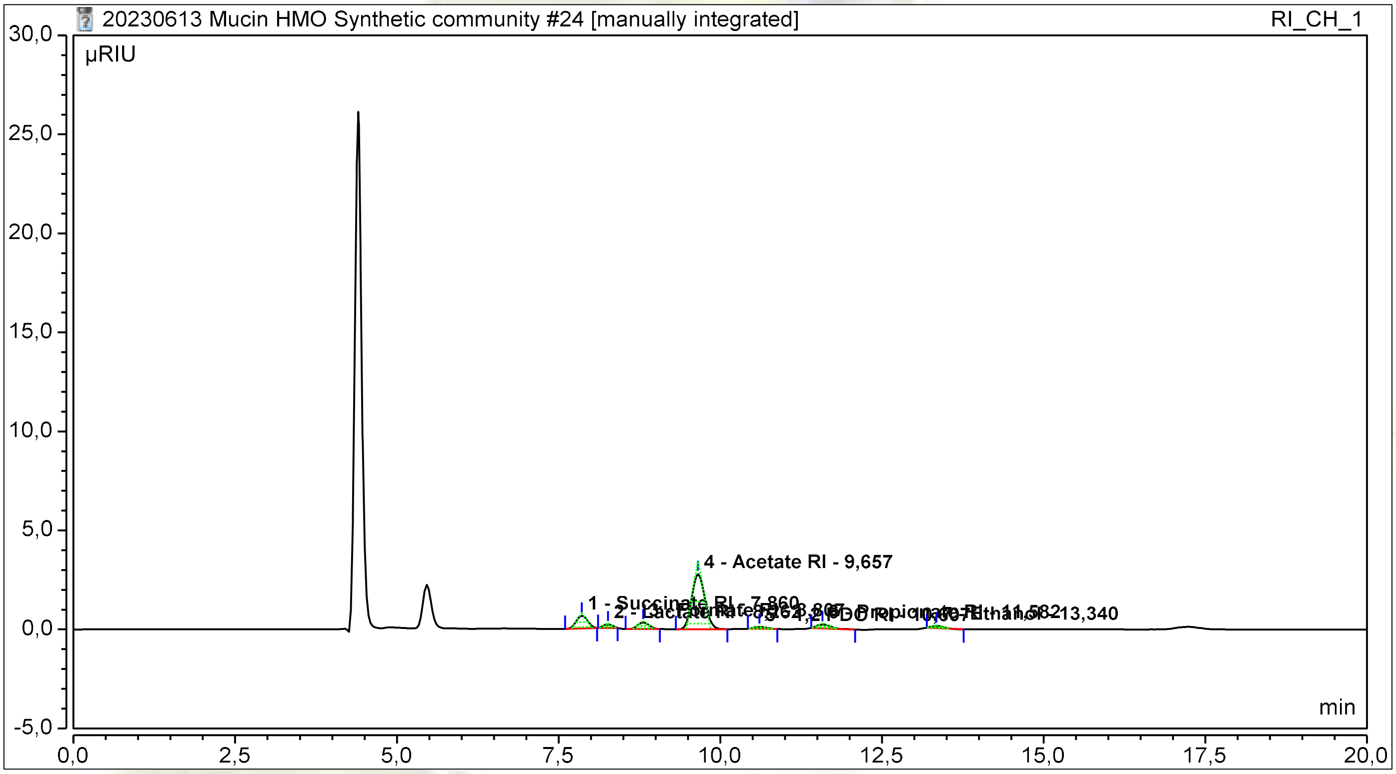

Peak Results

No.	Peak Name	Retention Time min	Width (50%) min	Type	Resolution (EP)	Asymmetry (EP)	Plates (EP)
n.a.	GlcNAc	n.a.	n.a.	n.a.	n.a.	n.a.	n.a.
n.a.	Citrate	n.a.	n.a.	n.a.	n.a.	n.a.	n.a.
n.a.	Glucose	n.a.	n.a.	n.a.	n.a.	n.a.	n.a.
n.a.	Galactose	n.a.	n.a.	n.a.	n.a.	n.a.	n.a.
n.a.	Fucose	n.a.	n.a.	n.a.	n.a.	n.a.	n.a.
1	Succinate RI	7,860	0,196	BMB	1,30	1,02	8946
2	Lactate RI	8,262	0,169	BMB*	1,76	1,01	13236
n.a.	glycerol	n.a.	n.a.	n.a.	n.a.	n.a.	n.a.
3	Formate RI	8,807	0,197	BMB*	2,38	1,08	11062
4	Acetate RI	9,657	0,225	BMB	2,48	1,09	10244
5	1,2 PDO RI	10,607	0,228	BMB*	2,48	1,20	12007
n.a.	1,3-PDO	n.a.	n.a.	n.a.	n.a.	n.a.	n.a.
6	Propionate RI	11,582	0,235	BMB*	4,47	1,25	13428
n.a.	1,3-PDO	n.a.	n.a.	n.a.	n.a.	n.a.	n.a.
n.a.	2-3 BDO	n.a.	n.a.	n.a.	n.a.	n.a.	n.a.
7	Ethanol	13,340	0,229	BMB*	n.a.	1,29	18845
n.a.	Isobutyrate RI	n.a.	n.a.	n.a.	n.a.	n.a.	n.a.
n.a.	Butyrate RI	n.a.	n.a.	n.a.	n.a.	n.a.	n.a.

Chromatogram and SST Results

Injection Details

Injection Name:	**50 5HMO1 t96 r2**	*Run Time (min):*	**20,00**
Vial Number:	**3:B5**	*Injection Volume:*	**10,00**
Injection Type:	**Unknown**	*Channel:*	**RI_CH_1**
Calibration Level:		*Wavelength:*	**n.a.**
Instrument Method:	**Default method LC2030C 45 gr 20 min**	*Bandwidth:*	**n.a.**
Processing Method:	**Processing Method LC2030 45 gr**	*Dilution Factor:*	**1,0000**
Injection Date/Time:	**13-jun-23 19:46**	*Sample Weight:*	**1,0000**

Chromatogram

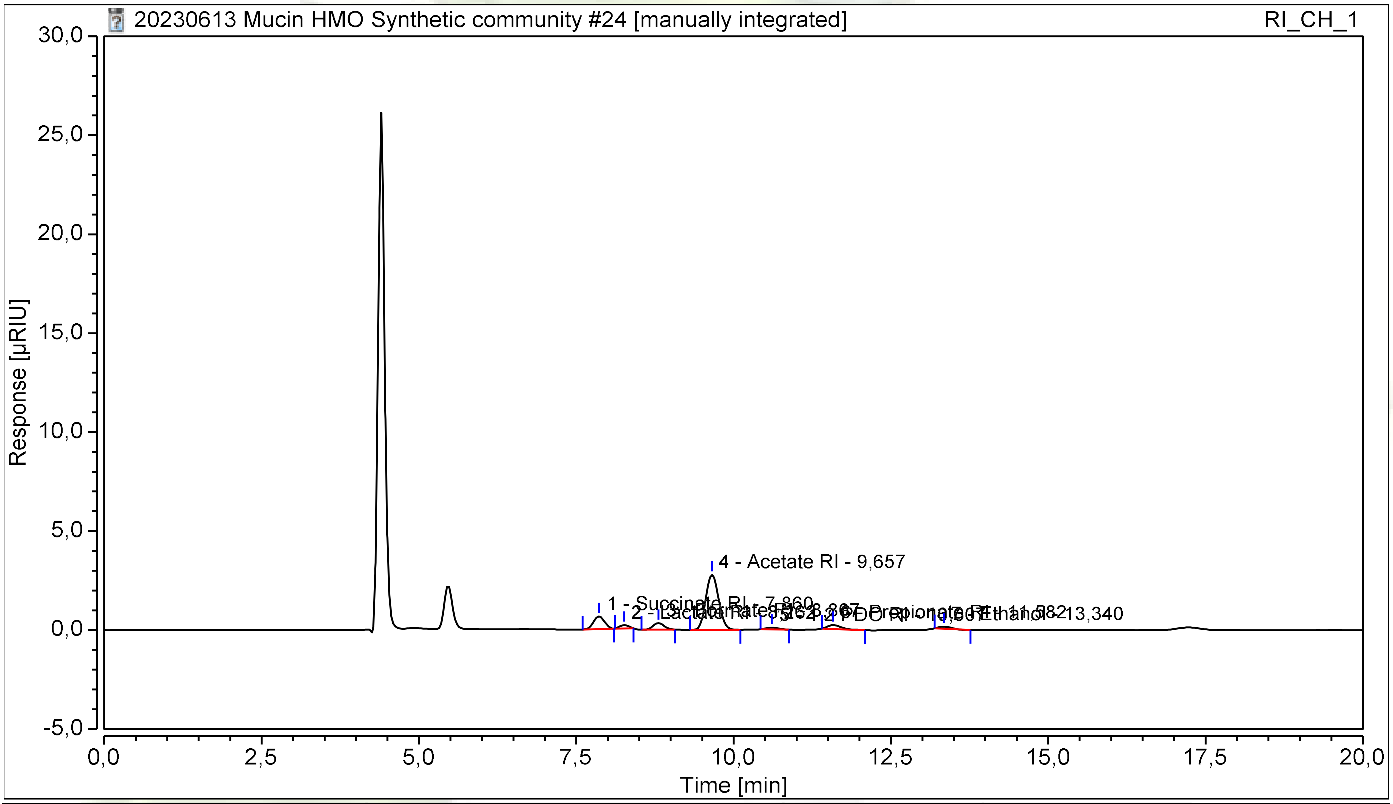

SST Results

No.	Name	Inj.Condition	Peak	Test Result	Injection

Number of executed test cases: **n.a.** **Total Result:** **Passed**

Chromatogram

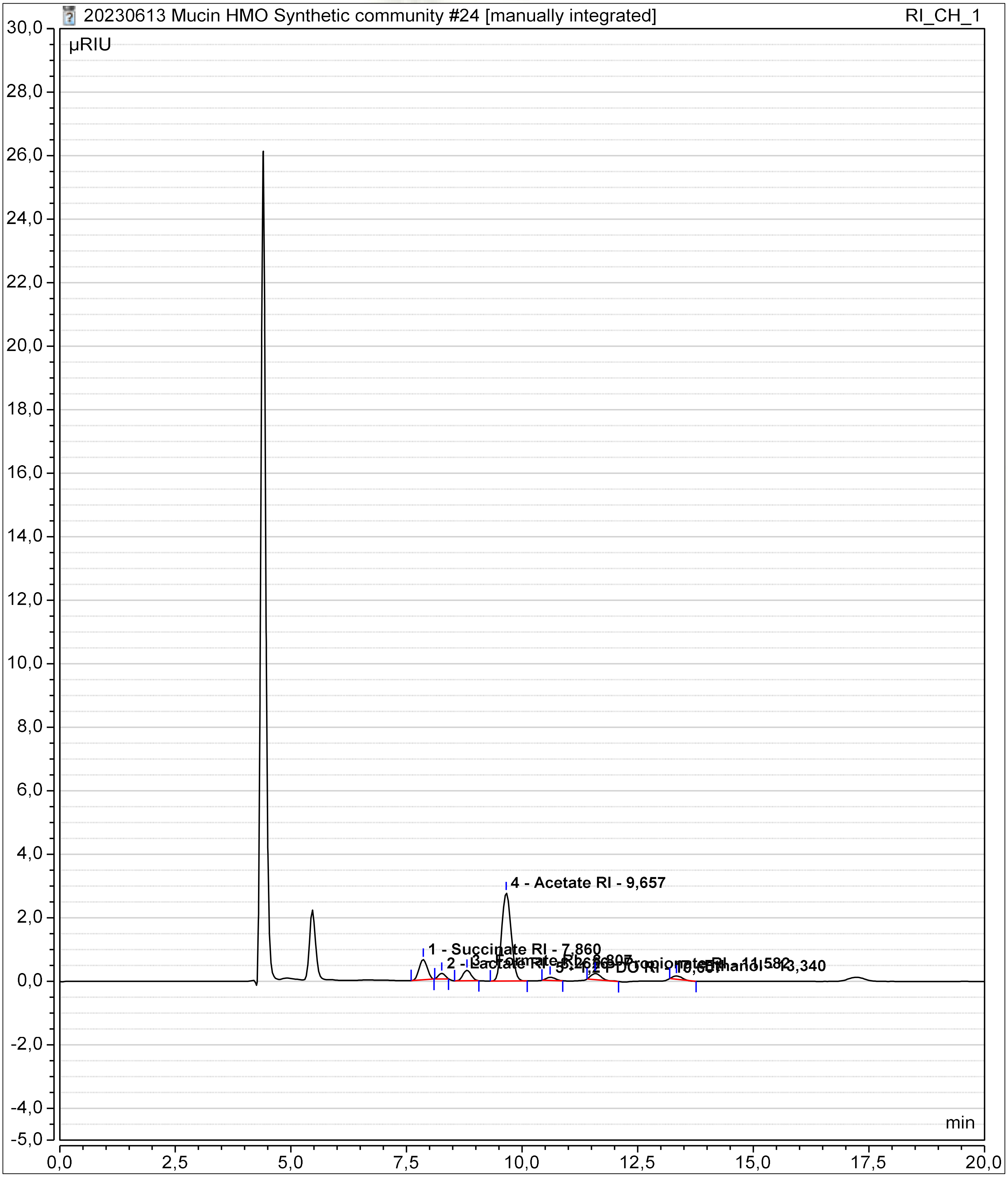

Chromeleon (c) Dionex
Version 7.2.10.25868

Chromatogram and Results

Injection Details

Injection Name:	**51 5HMO1 t96 r3**	*Run Time (min):*	**20,00**
Vial Number:	**3:B6**	*Injection Volume:*	**10,00**
Injection Type:	**Unknown**	*Channel:*	**RI_CH_1**
Calibration Level:		*Wavelength:*	**n.a.**
Instrument Method:	**Default method LC2030C 45 gr 20 min**	*Bandwidth:*	**n.a.**
Processing Method:	**Processing Method LC2030 45 gr**	*Dilution Factor:*	**1,0000**
Injection Date/Time:	**13-jun-23 20:07**	*Sample Weight:*	**1,0000**

Chromatogram

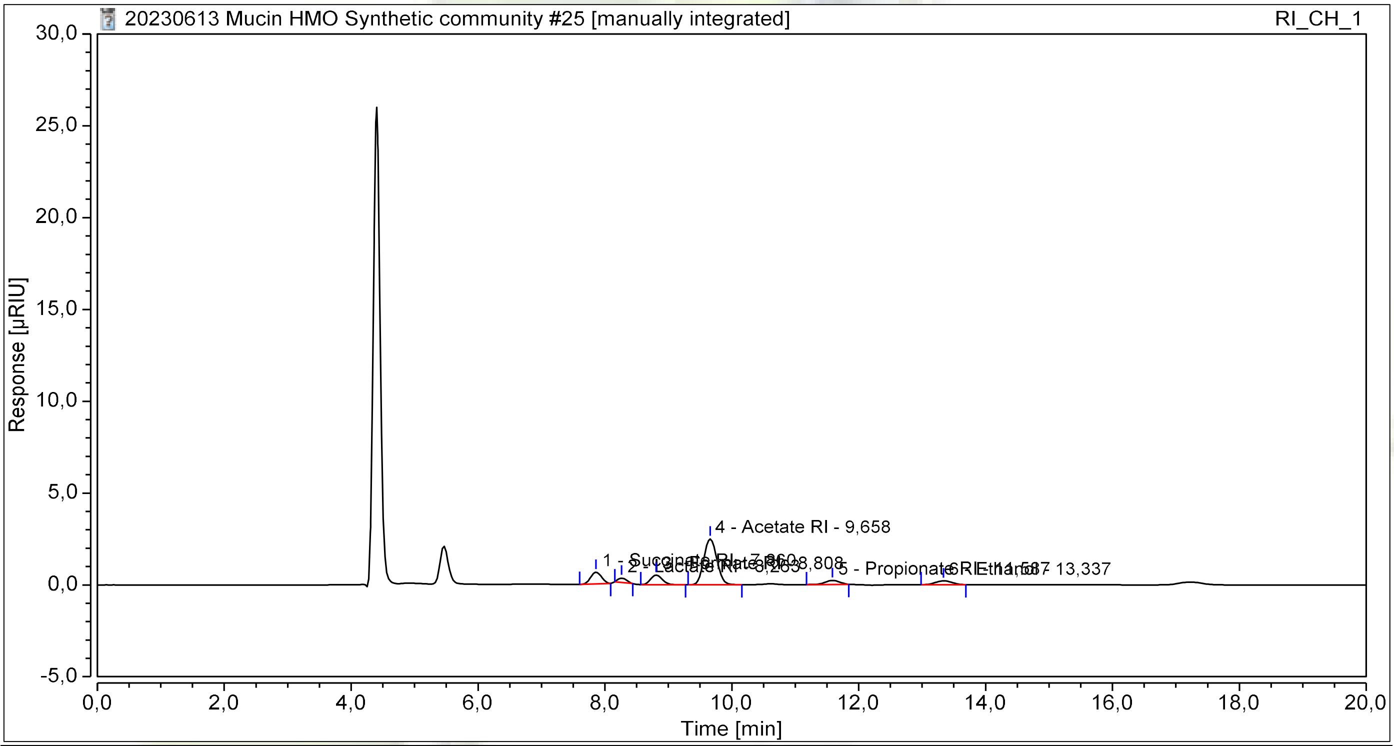

Integration Results

No.	Peak Name	Retention Time min	Area µRIU*min	Height µRIU	Relative Area %	Relative Height %	Amount
n.a.	GlcNAc	n.a.	n.a.	n.a.	n.a.	n.a.	n.a.
n.a.	Citrate	n.a.	n.a.	n.a.	n.a.	n.a.	n.a.
n.a.	Glucose	n.a.	n.a.	n.a.	n.a.	n.a.	n.a.
n.a.	Galactose	n.a.	n.a.	n.a.	n.a.	n.a.	n.a.
n.a.	Fucose	n.a.	n.a.	n.a.	n.a.	n.a.	n.a.
1	Succinate RI	7,860	0,127	0,634	12,84	14,68	n.a.
2	Lactate RI	8,263	0,038	0,234	3,80	5,42	1,0943
n.a.	glycerol	n.a.	n.a.	n.a.	n.a.	n.a.	n.a.
3	Formate RI	8,808	0,110	0,522	11,12	12,10	11,5806
4	Acetate RI	9,658	0,593	2,494	59,78	57,77	36,4948
n.a.	1,2 PDO RI	n.a.	n.a.	n.a.	n.a.	n.a.	n.a.
n.a.	1,3-PDO	n.a.	n.a.	n.a.	n.a.	n.a.	n.a.
5	Propionate RI	11,587	0,058	0,219	5,84	5,07	2,3321
n.a.	1,3-PDO	n.a.	n.a.	n.a.	n.a.	n.a.	n.a.
n.a.	2-3 BDO	n.a.	n.a.	n.a.	n.a.	n.a.	n.a.
6	Ethanol	13,337	0,066	0,214	6,61	4,95	0,6037
n.a.	Isobutyrate RI	n.a.	n.a.	n.a.	n.a.	n.a.	n.a.
n.a.	Butyrate RI	n.a.	n.a.	n.a.	n.a.	n.a.	n.a.
**Total:**			**0,992**	**4,317**	**100,00**	**100,00**	

Peak Analysis

Injection Details

Injection Name:	**51 5HMO1 t96 r3**	*Run Time (min):*	**20,00**
Vial Number:	**3:B6**	*Injection Volume:*	**10,00**
Injection Type:	**Unknown**	*Channel:*	**RI_CH_1**
Calibration Level:		*Wavelength:*	**n.a.**
Instrument Method:	**Default method LC2030C 45 gr 20 min**	*Bandwidth:*	**n.a.**
Processing Method:	**Processing Method LC2030 45 gr**	*Dilution Factor:*	**1,0000**
Injection Date/Time:	**13-jun-23 20:07**	*Sample Weight:*	**1,0000**

Chromatogram

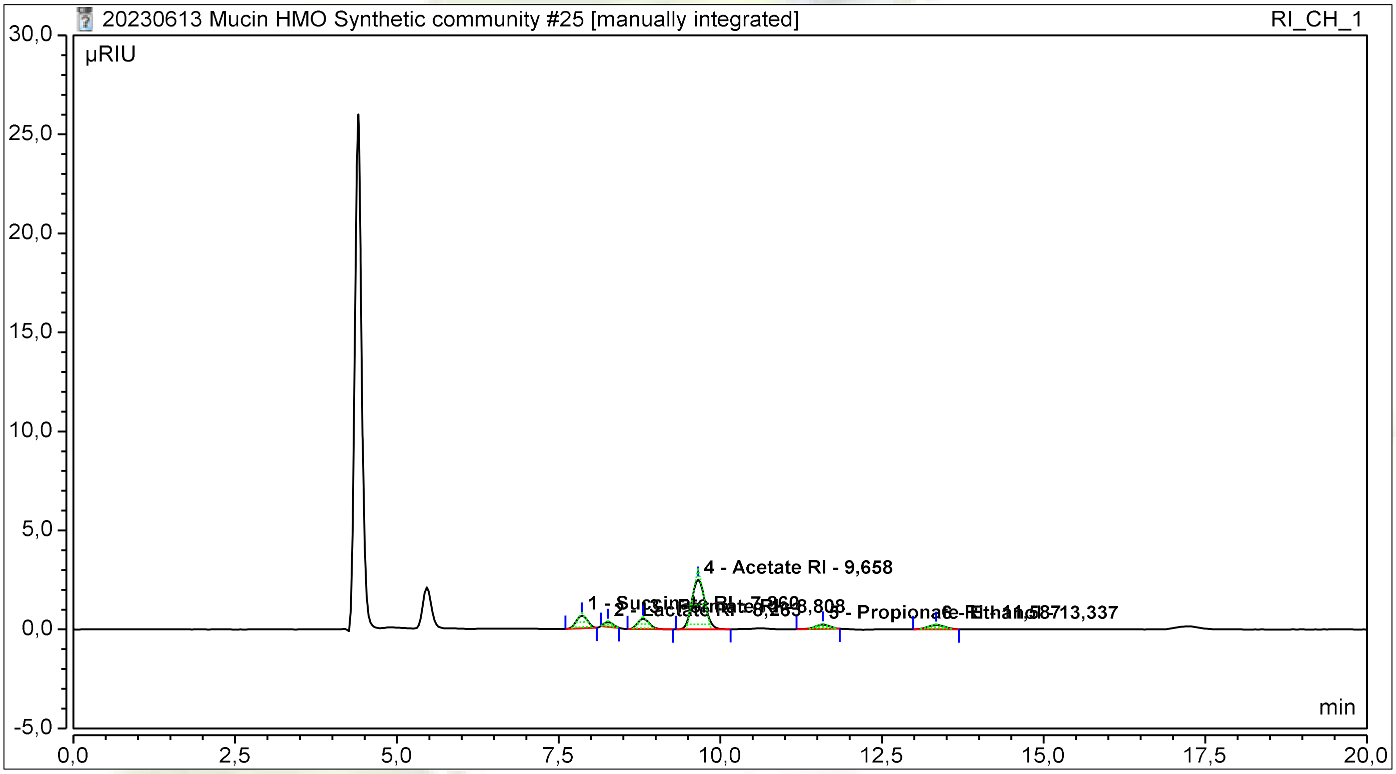

Peak Results

No.	Peak Name	Retention Time min	Width (50%) min	Type	Resolution (EP)	Asymmetry (EP)	Plates (EP)
n.a.	GlcNAc	n.a.	n.a.	n.a.	n.a.	n.a.	n.a.
n.a.	Citrate	n.a.	n.a.	n.a.	n.a.	n.a.	n.a.
n.a.	Glucose	n.a.	n.a.	n.a.	n.a.	n.a.	n.a.
n.a.	Galactose	n.a.	n.a.	n.a.	n.a.	n.a.	n.a.
n.a.	Fucose	n.a.	n.a.	n.a.	n.a.	n.a.	n.a.
1	Succinate RI	7,860	0,195	BMB	1,33	1,02	9038
2	Lactate RI	8,263	0,163	BMB*	1,78	1,28	14323
n.a.	glycerol	n.a.	n.a.	n.a.	n.a.	n.a.	n.a.
3	Formate RI	8,808	0,200	BMB	2,36	1,11	10784
4	Acetate RI	9,658	0,225	BMB	4,75	1,09	10227
n.a.	1,2 PDO RI	n.a.	n.a.	n.a.	n.a.	n.a.	n.a.
n.a.	1,3-PDO	n.a.	n.a.	n.a.	n.a.	n.a.	n.a.
5	Propionate RI	11,587	0,255	BMB*	3,75	0,95	11471
n.a.	1,3-PDO	n.a.	n.a.	n.a.	n.a.	n.a.	n.a.
n.a.	2-3 BDO	n.a.	n.a.	n.a.	n.a.	n.a.	n.a.
6	Ethanol	13,337	0,296	BMB*	n.a.	1,04	11283
n.a.	Isobutyrate RI	n.a.	n.a.	n.a.	n.a.	n.a.	n.a.
n.a.	Butyrate RI	n.a.	n.a.	n.a.	n.a.	n.a.	n.a.

Chromatogram and SST Results

Injection Details

Injection Name:	**51 5HMO1 t96 r3**	*Run Time (min):*	**20,00**
Vial Number:	**3:B6**	*Injection Volume:*	**10,00**
Injection Type:	**Unknown**	*Channel:*	**RI_CH_1**
Calibration Level:		*Wavelength:*	**n.a.**
Instrument Method:	**Default method LC2030C 45 gr 20 min**	*Bandwidth:*	**n.a.**
Processing Method:	**Processing Method LC2030 45 gr**	*Dilution Factor:*	**1,0000**
Injection Date/Time:	**13-jun-23 20:07**	*Sample Weight:*	**1,0000**

Chromatogram

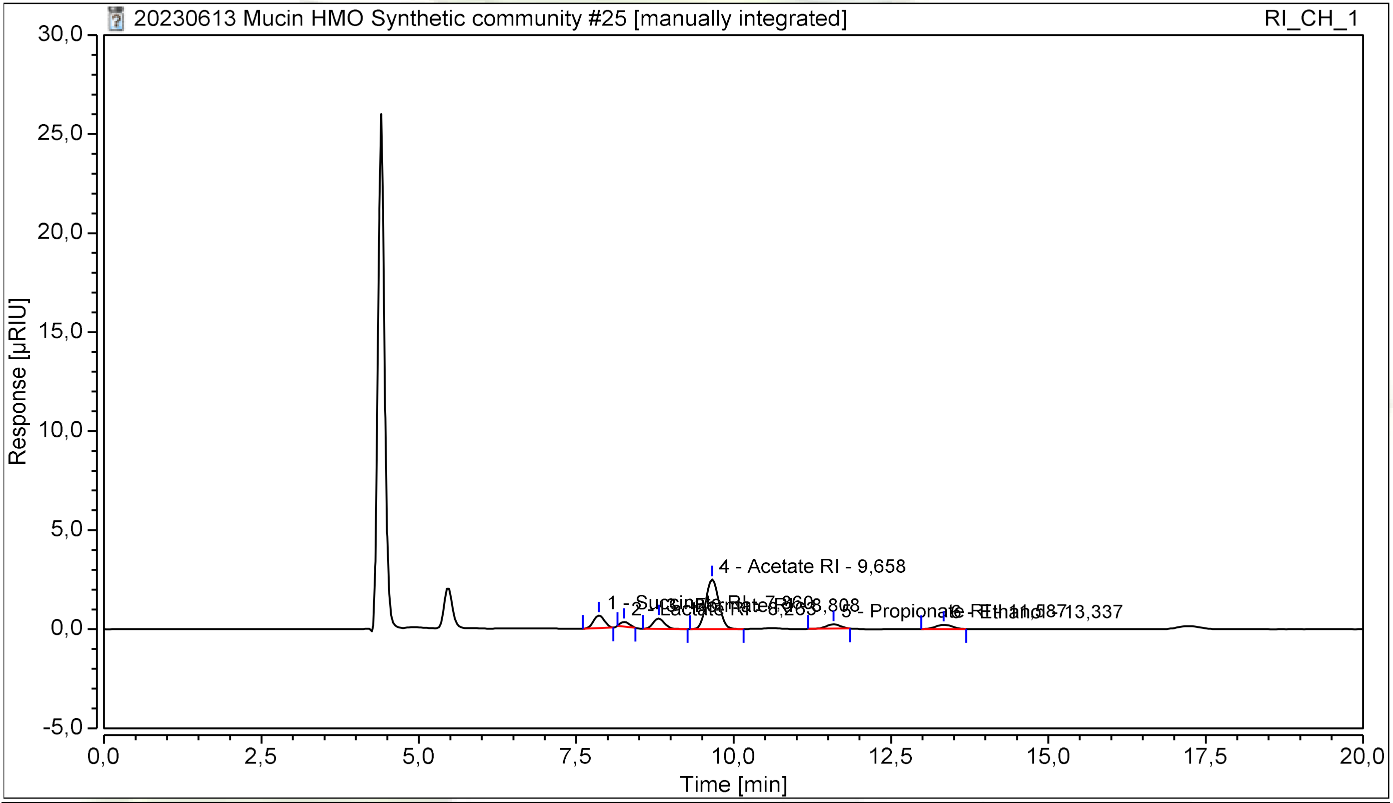

SST Results

No.	Name	Inj.Condition	Peak	Test Result	Injection
**Number of executed test cases:**	**n.a.**		**Total Result:**	**Passed**	

Chromatogram

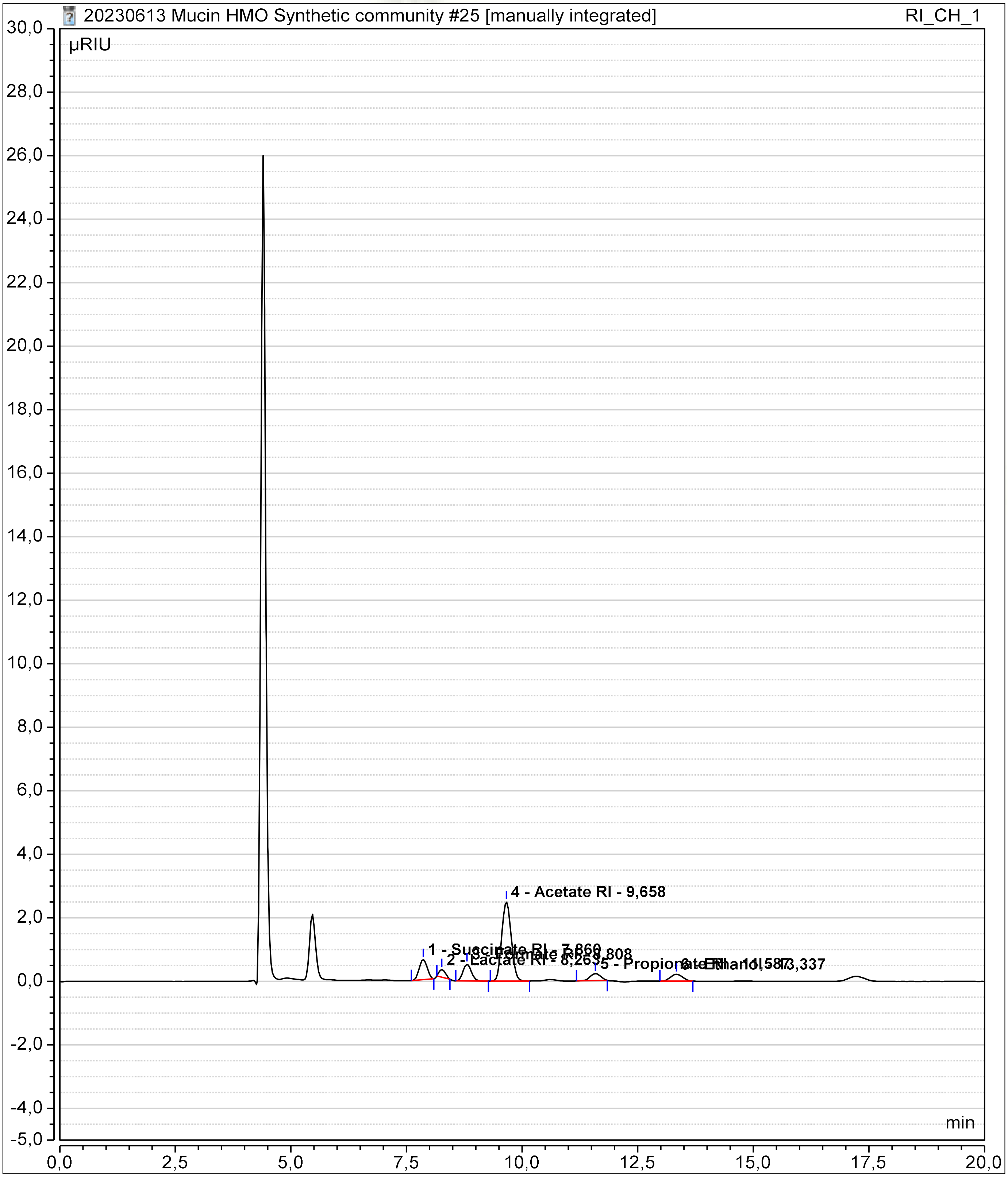

Chromatogram and Results

Injection Details

Injection Name:	**52 5HMO1 t120 r1**	*Run Time (min):*	**20,00**
Vial Number:	**3:B7**	*Injection Volume:*	**10,00**
Injection Type:	**Unknown**	*Channel:*	**RI_CH_1**
Calibration Level:		*Wavelength:*	**n.a.**
Instrument Method:	**Default method LC2030C 45 gr 20 min**	*Bandwidth:*	**n.a.**
Processing Method:	**Processing Method LC2030 45 gr**	*Dilution Factor:*	**1,0000**
Injection Date/Time:	**13-jun-23 20:27**	*Sample Weight:*	**1,0000**

Chromatogram

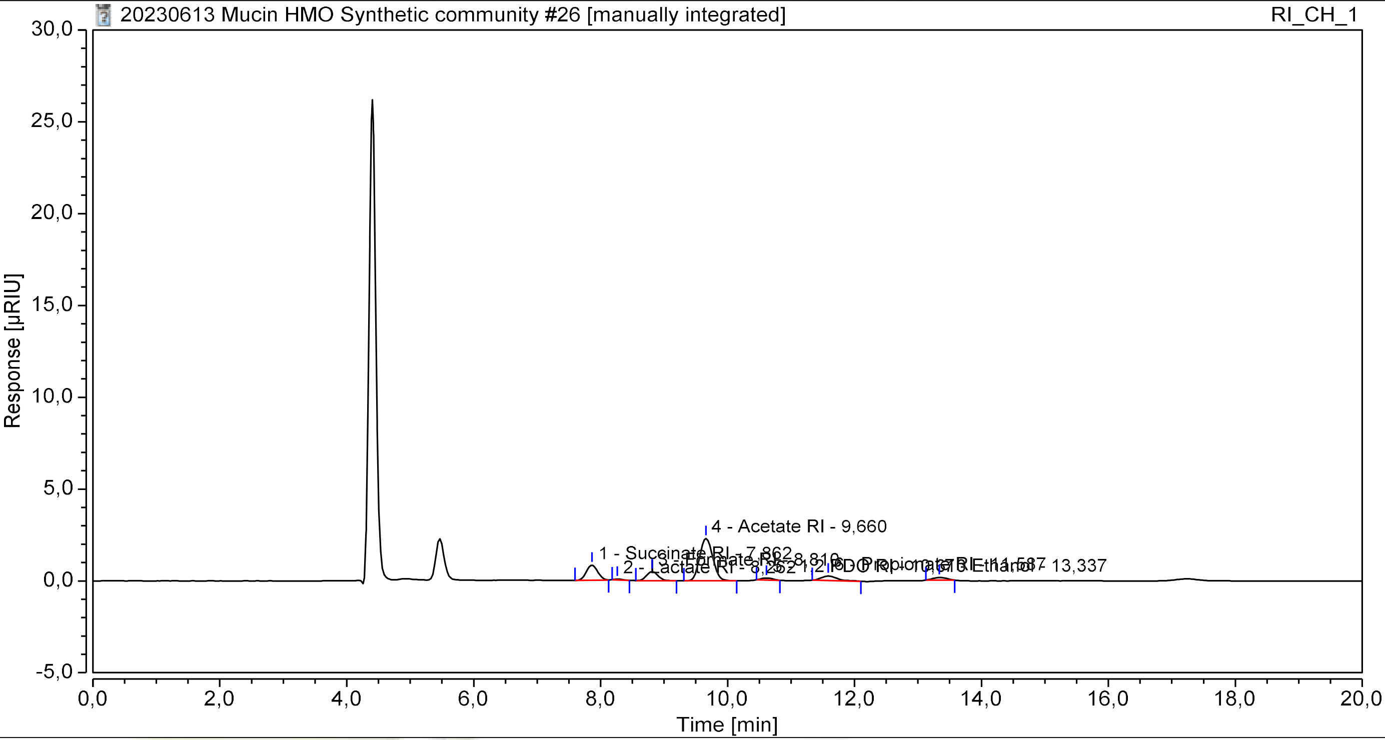

Integration Results

No.	Peak Name	Retention Time min	Area µRIU*min	Height µRIU	Relative Area %	Relative Height %	Amount
n.a.	GlcNAc	n.a.	n.a.	n.a.	n.a.	n.a.	n.a.
n.a.	Citrate	n.a.	n.a.	n.a.	n.a.	n.a.	n.a.
n.a.	Glucose	n.a.	n.a.	n.a.	n.a.	n.a.	n.a.
n.a.	Galactose	n.a.	n.a.	n.a.	n.a.	n.a.	n.a.
n.a.	Fucose	n.a.	n.a.	n.a.	n.a.	n.a.	n.a.
1	Succinate RI	7,862	0,171	0,821	17,74	19,73	n.a.
2	Lactate RI	8,262	0,006	0,038	0,60	0,90	0,1669
n.a.	glycerol	n.a.	n.a.	n.a.	n.a.	n.a.	n.a.
3	Formate RI	8,810	0,103	0,489	10,74	11,76	10,8588
4	Acetate RI	9,660	0,549	2,308	56,97	55,45	33,7790
5	1,2 PDO RI	10,610	0,023	0,108	2,36	2,60	0,6748
n.a.	1,3-PDO	n.a.	n.a.	n.a.	n.a.	n.a.	n.a.
6	Propionate RI	11,587	0,074	0,251	7,65	6,02	2,9633
n.a.	1,3-PDO	n.a.	n.a.	n.a.	n.a.	n.a.	n.a.
n.a.	2-3 BDO	n.a.	n.a.	n.a.	n.a.	n.a.	n.a.
7	Ethanol	13,337	0,038	0,148	3,95	3,54	0,3508
n.a.	Isobutyrate RI	n.a.	n.a.	n.a.	n.a.	n.a.	n.a.
n.a.	Butyrate RI	n.a.	n.a.	n.a.	n.a.	n.a.	n.a.
**Total:**			**0,963**	**4,163**	**100,00**	**100,00**	

Peak Analysis

Injection Details

Injection Name:	**52 5HMO1 t120 r1**	*Run Time (min):*	**20,00**
Vial Number:	**3:B7**	*Injection Volume:*	**10,00**
Injection Type:	**Unknown**	*Channel:*	**RI_CH_1**
Calibration Level:		*Wavelength:*	**n.a.**
Instrument Method:	**Default method LC2030C 45 gr 20 min**	*Bandwidth:*	**n.a.**
Processing Method:	**Processing Method LC2030 45 gr**	*Dilution Factor:*	**1,0000**
Injection Date/Time:	**13-jun-23 20:27**	*Sample Weight:*	**1,0000**

Chromatogram

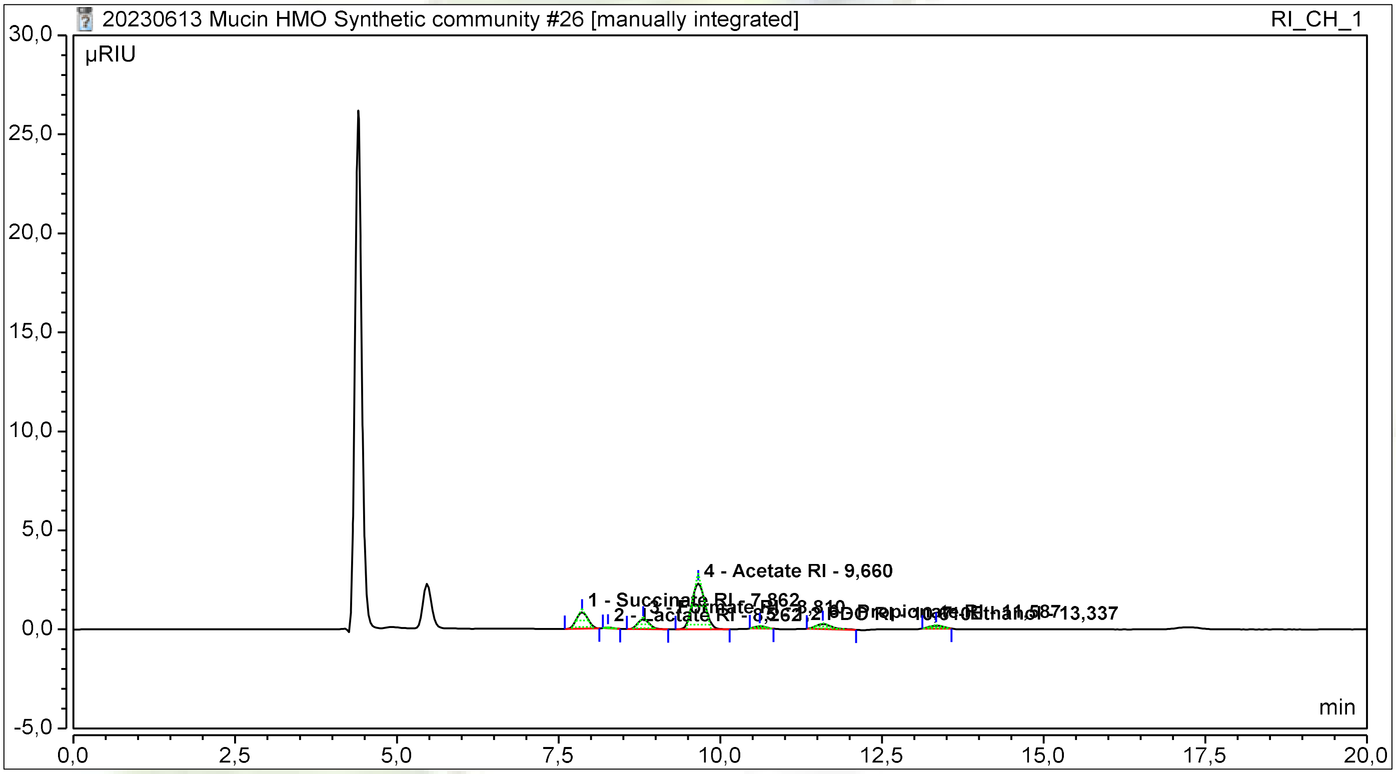

Peak Results

No.	Peak Name	Retention Time min	Width (50%) min	Type	Resolution (EP)	Asymmetry (EP)	Plates (EP)
n.a.	GlcNAc	n.a.	n.a.	n.a.	n.a.	n.a.	n.a.
n.a.	Citrate	n.a.	n.a.	n.a.	n.a.	n.a.	n.a.
n.a.	Glucose	n.a.	n.a.	n.a.	n.a.	n.a.	n.a.
n.a.	Galactose	n.a.	n.a.	n.a.	n.a.	n.a.	n.a.
n.a.	Fucose	n.a.	n.a.	n.a.	n.a.	n.a.	n.a.
1	Succinate RI	7,862	0,199	BMB	1,35	1,05	8647
2	Lactate RI	8,262	0,152	BMB*	1,84	1,62	16444
n.a.	glycerol	n.a.	n.a.	n.a.	n.a.	n.a.	n.a.
3	Formate RI	8,810	0,200	BMB	2,36	1,10	10743
4	Acetate RI	9,660	0,225	BMB	2,56	1,08	10222
5	1,2 PDO RI	10,610	0,213	BMB*	2,41	1,17	13713
n.a.	1,3-PDO	n.a.	n.a.	n.a.	n.a.	n.a.	n.a.
6	Propionate RI	11,587	0,265	BMB*	3,92	1,53	10553
n.a.	1,3-PDO	n.a.	n.a.	n.a.	n.a.	n.a.	n.a.
n.a.	2-3 BDO	n.a.	n.a.	n.a.	n.a.	n.a.	n.a.
7	Ethanol	13,337	0,262	BMB*	n.a.	1,06	14401
n.a.	Isobutyrate RI	n.a.	n.a.	n.a.	n.a.	n.a.	n.a.
n.a.	Butyrate RI	n.a.	n.a.	n.a.	n.a.	n.a.	n.a.

Chromatogram and SST Results

Injection Details

Injection Name:	**52 5HMO1 t120 r1**	*Run Time (min):*	**20,00**
Vial Number:	**3:B7**	*Injection Volume:*	**10,00**
Injection Type:	**Unknown**	*Channel:*	**RI_CH_1**
Calibration Level:		*Wavelength:*	**n.a.**
Instrument Method:	**Default method LC2030C 45 gr 20 min**	*Bandwidth:*	**n.a.**
Processing Method:	**Processing Method LC2030 45 gr**	*Dilution Factor:*	**1,0000**
Injection Date/Time:	**13-jun-23 20:27**	*Sample Weight:*	**1,0000**

Chromatogram

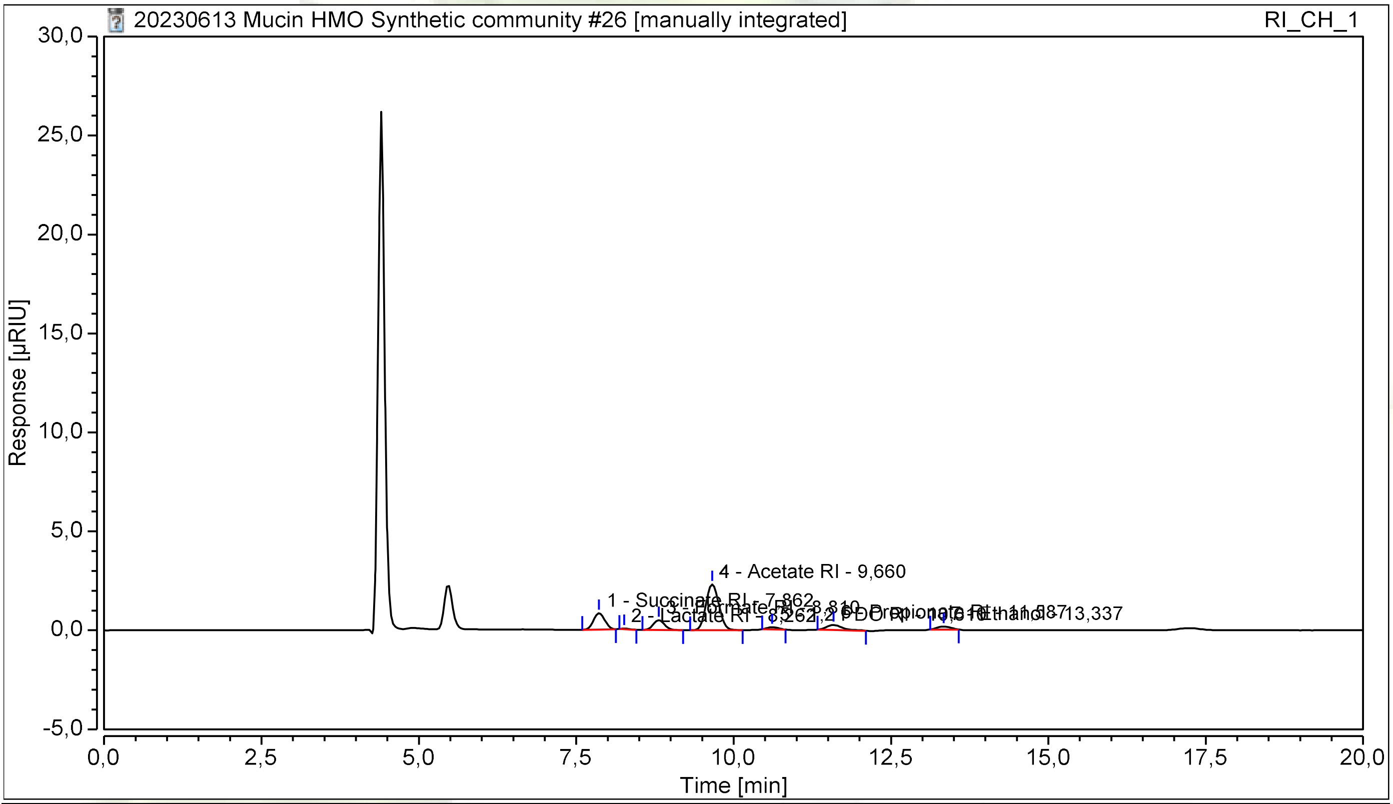

SST Results

No.	Name	Inj.Condition	Peak	Test Result	Injection
**Number of executed test cases:**	**n.a.**		**Total Result:**	**Passed**	

Chromatogram

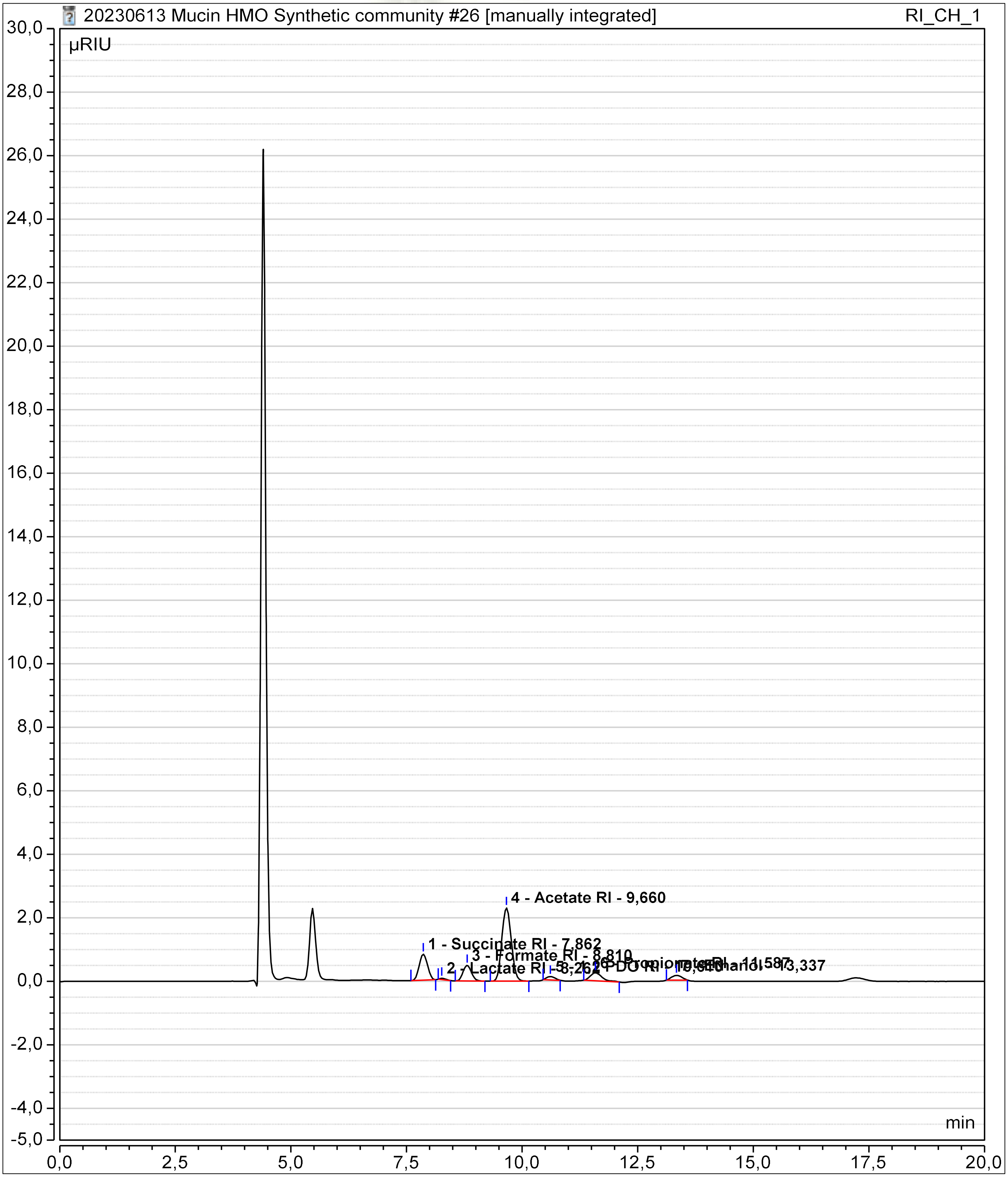

Chromatogram and Results

Injection Details

Injection Name:	**53 5HMO1 t120 r2**	*Run Time (min):*	**20,00**
Vial Number:	**3:B8**	*Injection Volume:*	**10,00**
Injection Type:	**Unknown**	*Channel:*	**RI_CH_1**
Calibration Level:		*Wavelength:*	**n.a.**
Instrument Method:	**Default method LC2030C 45 gr 20 min**	*Bandwidth:*	**n.a.**
Processing Method:	**Processing Method LC2030 45 gr**	*Dilution Factor:*	**1,0000**
Injection Date/Time:	**13-jun-23 20:48**	*Sample Weight:*	**1,0000**

Chromatogram

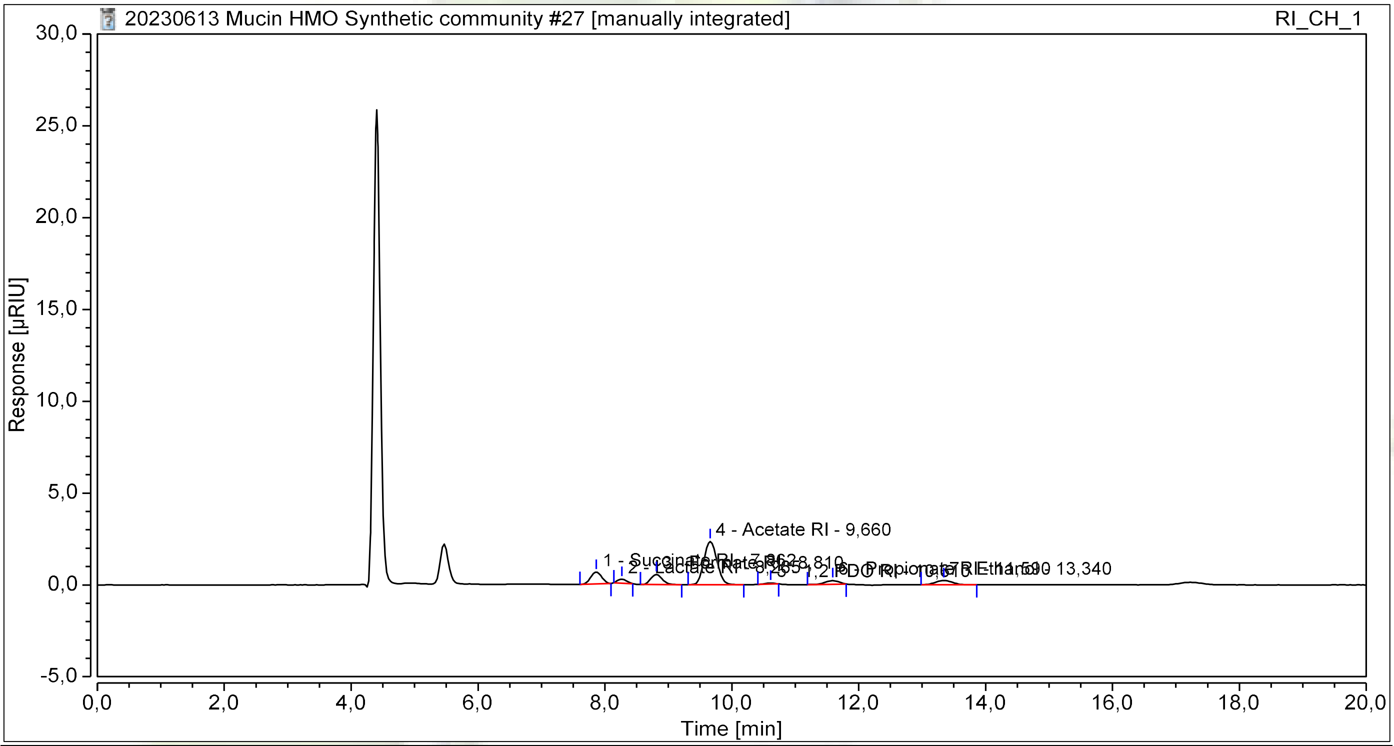

Integration Results

No.	Peak Name	Retention Time min	Area µRIU*min	Height µRIU	Relative Area %	Relative Height %	Amount
n.a.	GlcNAc	n.a.	n.a.	n.a.	n.a.	n.a.	n.a.
n.a.	Citrate	n.a.	n.a.	n.a.	n.a.	n.a.	n.a.
n.a.	Glucose	n.a.	n.a.	n.a.	n.a.	n.a.	n.a.
n.a.	Galactose	n.a.	n.a.	n.a.	n.a.	n.a.	n.a.
n.a.	Fucose	n.a.	n.a.	n.a.	n.a.	n.a.	n.a.
1	Succinate RI	7,862	0,129	0,637	13,30	15,10	n.a.
2	Lactate RI	8,265	0,035	0,207	3,57	4,90	1,0030
n.a.	glycerol	n.a.	n.a.	n.a.	n.a.	n.a.	n.a.
3	Formate RI	8,810	0,116	0,548	11,94	12,97	12,1354
4	Acetate RI	9,660	0,559	2,349	57,69	55,65	34,3839
5	1,2 PDO RI	10,610	0,010	0,053	1,02	1,25	0,2923
n.a.	1,3-PDO	n.a.	n.a.	n.a.	n.a.	n.a.	n.a.
6	Propionate RI	11,590	0,048	0,194	4,94	4,59	1,9227
n.a.	1,3-PDO	n.a.	n.a.	n.a.	n.a.	n.a.	n.a.
n.a.	2-3 BDO	n.a.	n.a.	n.a.	n.a.	n.a.	n.a.
7	Ethanol	13,340	0,073	0,234	7,55	5,54	0,6733
n.a.	Isobutyrate RI	n.a.	n.a.	n.a.	n.a.	n.a.	n.a.
n.a.	Butyrate RI	n.a.	n.a.	n.a.	n.a.	n.a.	n.a.
**Total:**			**0,968**	**4,221**	**100,00**	**100,00**	

Chromeleon (c) Dionex
Version 7.2.10.25868

Peak Analysis

Injection Details

Injection Name:	**53 5HMO1 t120 r2**	*Run Time (min):*	**20,00**
Vial Number:	**3:B8**	*Injection Volume:*	**10,00**
Injection Type:	**Unknown**	*Channel:*	**RI_CH_1**
Calibration Level:		*Wavelength:*	**n.a.**
Instrument Method:	**Default method LC2030C 45 gr 20 min**	*Bandwidth:*	**n.a.**
Processing Method:	**Processing Method LC2030 45 gr**	*Dilution Factor:*	**1,0000**
Injection Date/Time:	**13-jun-23 20:48**	*Sample Weight:*	**1,0000**

Chromatogram

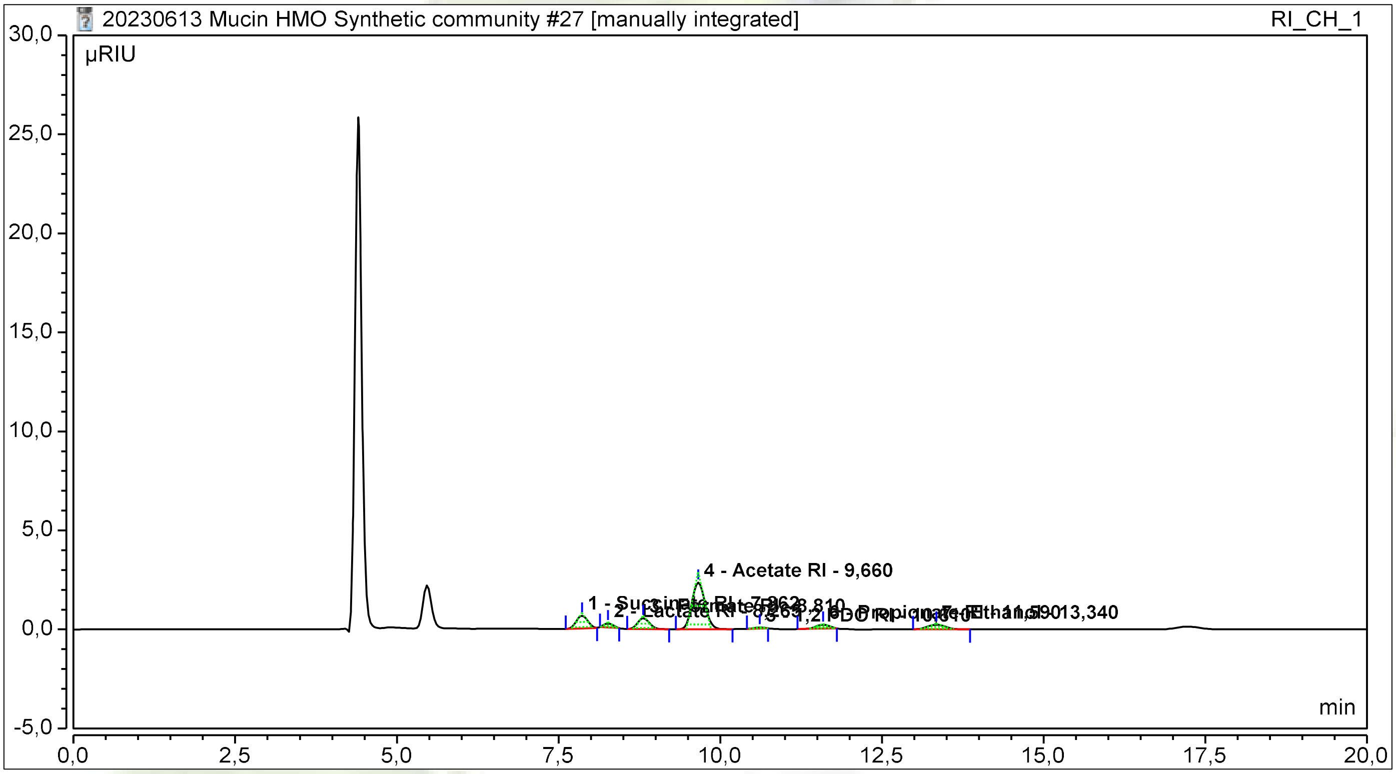

Peak Results

No.	Peak Name	Retention Time min	Width (50%) min	Type	Resolution (EP)	Asymmetry (EP)	Plates (EP)
n.a.	GlcNAc	n.a.	n.a.	n.a.	n.a.	n.a.	n.a.
n.a.	Citrate	n.a.	n.a.	n.a.	n.a.	n.a.	n.a.
n.a.	Glucose	n.a.	n.a.	n.a.	n.a.	n.a.	n.a.
n.a.	Galactose	n.a.	n.a.	n.a.	n.a.	n.a.	n.a.
n.a.	Fucose	n.a.	n.a.	n.a.	n.a.	n.a.	n.a.
1	Succinate RI	7,862	0,196	BMB	1,31	1,02	8954
2	Lactate RI	8,265	0,169	BMB*	1,75	1,18	13275
n.a.	glycerol	n.a.	n.a.	n.a.	n.a.	n.a.	n.a.
3	Formate RI	8,810	0,200	BMB	2,36	1,10	10784
4	Acetate RI	9,660	0,225	BMB	2,71	1,08	10216
5	1,2 PDO RI	10,610	0,188	BMB*	2,69	0,84	17582
n.a.	1,3-PDO	n.a.	n.a.	n.a.	n.a.	n.a.	n.a.
6	Propionate RI	11,590	0,241	BMB*	3,83	0,92	12789
n.a.	1,3-PDO	n.a.	n.a.	n.a.	n.a.	n.a.	n.a.
n.a.	2-3 BDO	n.a.	n.a.	n.a.	n.a.	n.a.	n.a.
7	Ethanol	13,340	0,298	BMB*	n.a.	1,05	11090
n.a.	Isobutyrate RI	n.a.	n.a.	n.a.	n.a.	n.a.	n.a.
n.a.	Butyrate RI	n.a.	n.a.	n.a.	n.a.	n.a.	n.a.

Chromatogram and SST Results

Injection Details

Injection Name:	**53 5HMO1 t120 r2**	*Run Time (min):*	**20,00**
Vial Number:	**3:B8**	*Injection Volume:*	**10,00**
Injection Type:	**Unknown**	*Channel:*	**RI_CH_1**
Calibration Level:		*Wavelength:*	**n.a.**
Instrument Method:	**Default method LC2030C 45 gr 20 min**	*Bandwidth:*	**n.a.**
Processing Method:	**Processing Method LC2030 45 gr**	*Dilution Factor:*	**1,0000**
Injection Date/Time:	**13-jun-23 20:48**	*Sample Weight:*	**1,0000**

Chromatogram

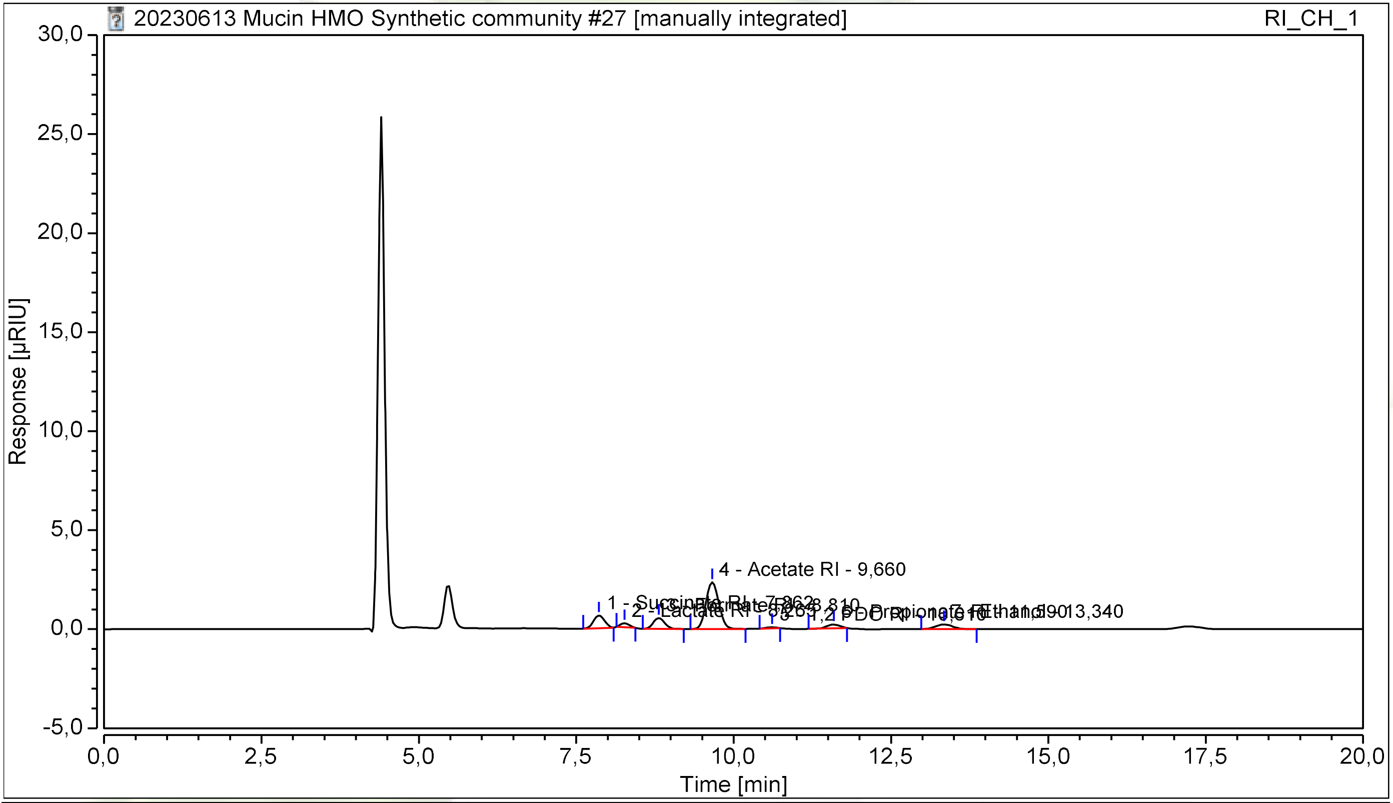

SST Results

No.	Name	Inj.Condition	Peak	Test Result	Injection
**Number of executed test cases:**	**n.a.**		**Total Result:**	**Passed**	

Chromatogram

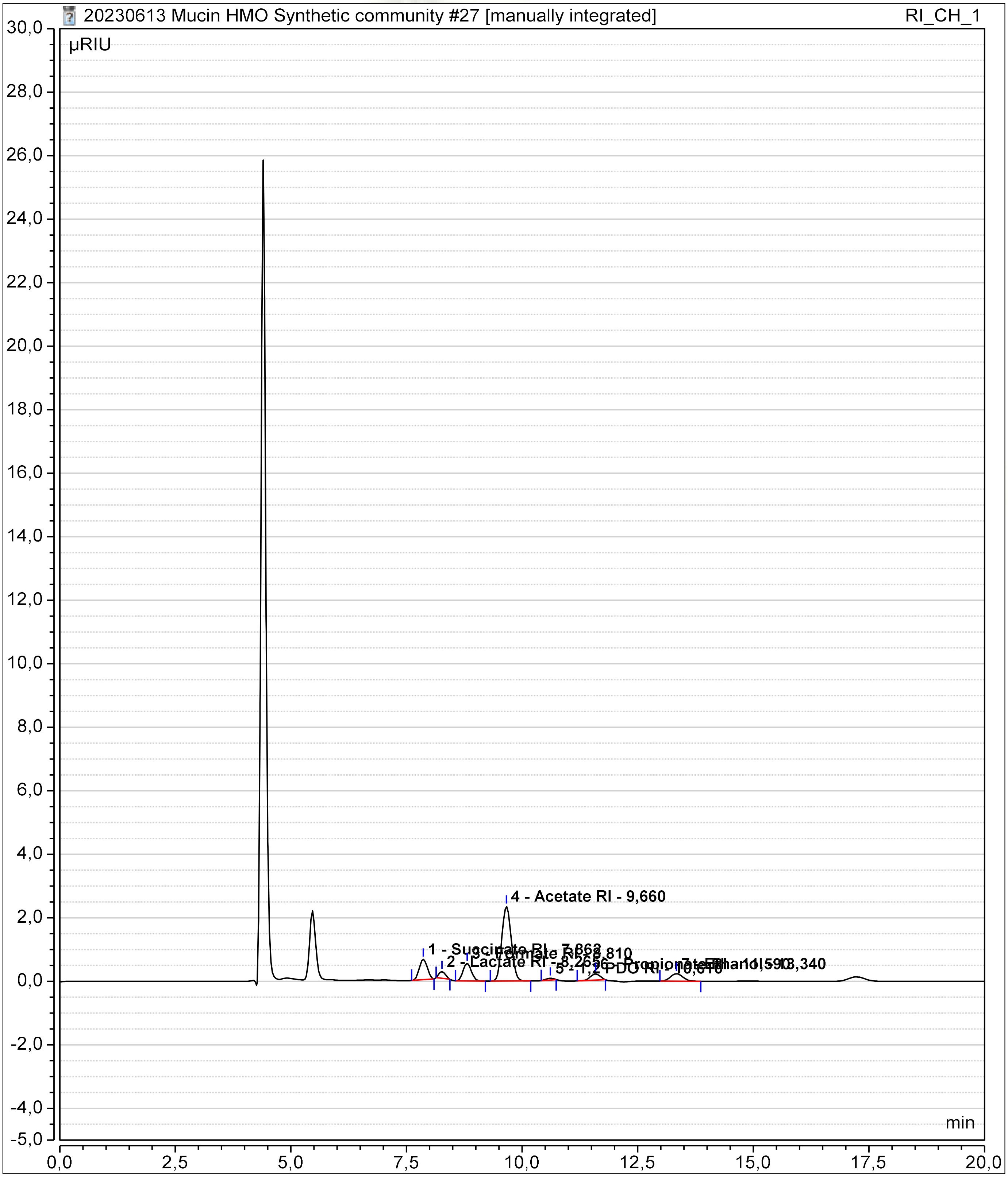

Chromatogram and Results

Injection Details

Injection Name:	**54 5HMO1 t120 r3**	*Run Time (min):*	**20,00**
Vial Number:	**3:B9**	*Injection Volume:*	**10,00**
Injection Type:	**Unknown**	*Channel:*	**RI_CH_1**
Calibration Level:		*Wavelength:*	**n.a.**
Instrument Method:	**Default method LC2030C 45 gr 20 min**	*Bandwidth:*	**n.a.**
Processing Method:	**Processing Method LC2030 45 gr**	*Dilution Factor:*	**1,0000**
Injection Date/Time:	**13-jun-23 21:08**	*Sample Weight:*	**1,0000**

Chromatogram

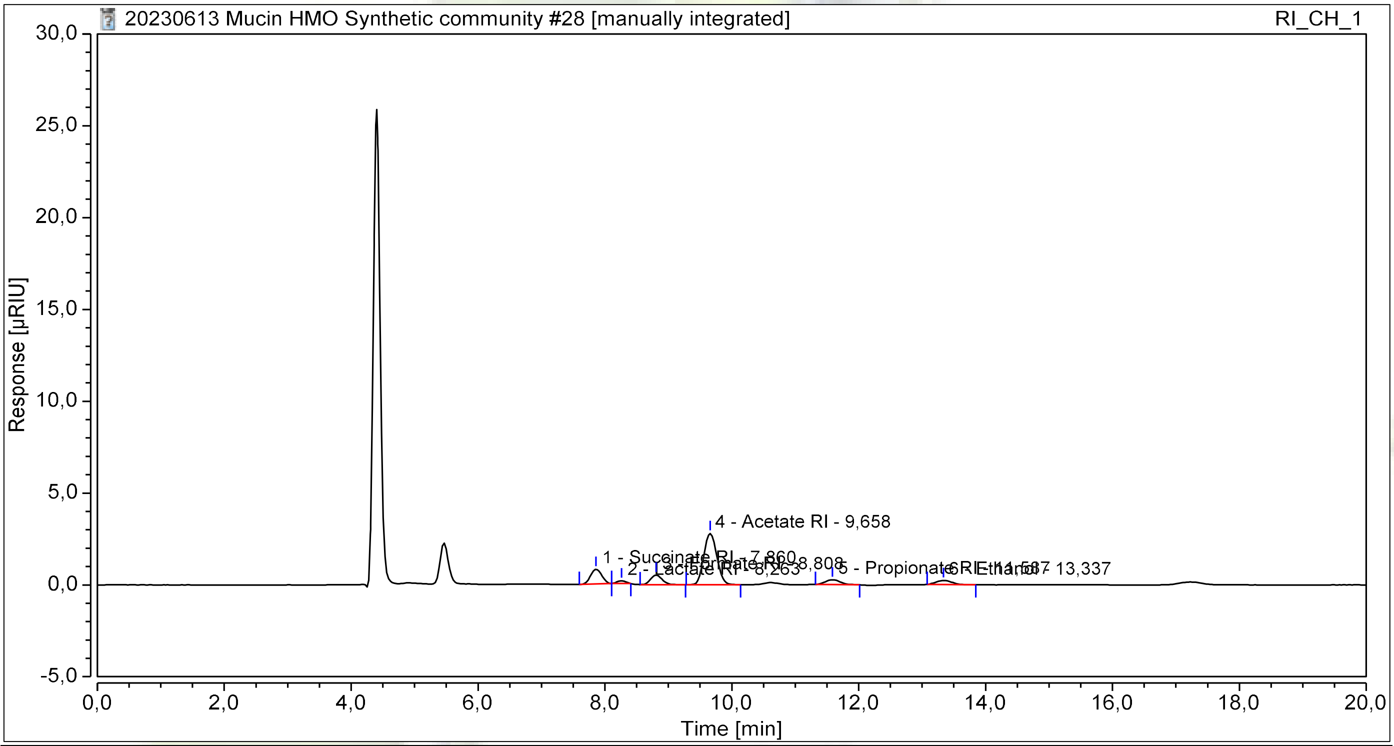

Integration Results

No.	Peak Name	Retention Time min	Area µRIU*min	Height µRIU	Relative Area %	Relative Height %	Amount
n.a.	GlcNAc	n.a.	n.a.	n.a.	n.a.	n.a.	n.a.
n.a.	Citrate	n.a.	n.a.	n.a.	n.a.	n.a.	n.a.
n.a.	Glucose	n.a.	n.a.	n.a.	n.a.	n.a.	n.a.
n.a.	Galactose	n.a.	n.a.	n.a.	n.a.	n.a.	n.a.
n.a.	Fucose	n.a.	n.a.	n.a.	n.a.	n.a.	n.a.
1	Succinate RI	7,860	0,163	0,799	14,90	16,88	n.a.
2	Lactate RI	8,263	0,025	0,152	2,31	3,21	0,7347
n.a.	glycerol	n.a.	n.a.	n.a.	n.a.	n.a.	n.a.
3	Formate RI	8,808	0,109	0,513	9,90	10,84	11,3989
4	Acetate RI	9,658	0,664	2,790	60,50	58,94	40,8510
n.a.	1,2 PDO RI	n.a.	n.a.	n.a.	n.a.	n.a.	n.a.
n.a.	1,3-PDO	n.a.	n.a.	n.a.	n.a.	n.a.	n.a.
5	Propionate RI	11,587	0,071	0,258	6,46	5,45	2,8513
n.a.	1,3-PDO	n.a.	n.a.	n.a.	n.a.	n.a.	n.a.
n.a.	2-3 BDO	n.a.	n.a.	n.a.	n.a.	n.a.	n.a.
6	Ethanol	13,337	0,065	0,221	5,94	4,67	0,5999
n.a.	Isobutyrate RI	n.a.	n.a.	n.a.	n.a.	n.a.	n.a.
n.a.	Butyrate RI	n.a.	n.a.	n.a.	n.a.	n.a.	n.a.
**Total:**			**1,097**	**4,733**	**100,00**	**100,00**	

Peak Analysis

Injection Details

Injection Name:	**54 5HMO1 t120 r3**	*Run Time (min):*	**20,00**
Vial Number:	**3:B9**	*Injection Volume:*	**10,00**
Injection Type:	**Unknown**	*Channel:*	**RI_CH_1**
Calibration Level:		*Wavelength:*	**n.a.**
Instrument Method:	**Default method LC2030C 45 gr 20 min**	*Bandwidth:*	**n.a.**
Processing Method:	**Processing Method LC2030 45 gr**	*Dilution Factor:*	**1,0000**
Injection Date/Time:	**13-jun-23 21:08**	*Sample Weight:*	**1,0000**

Chromatogram

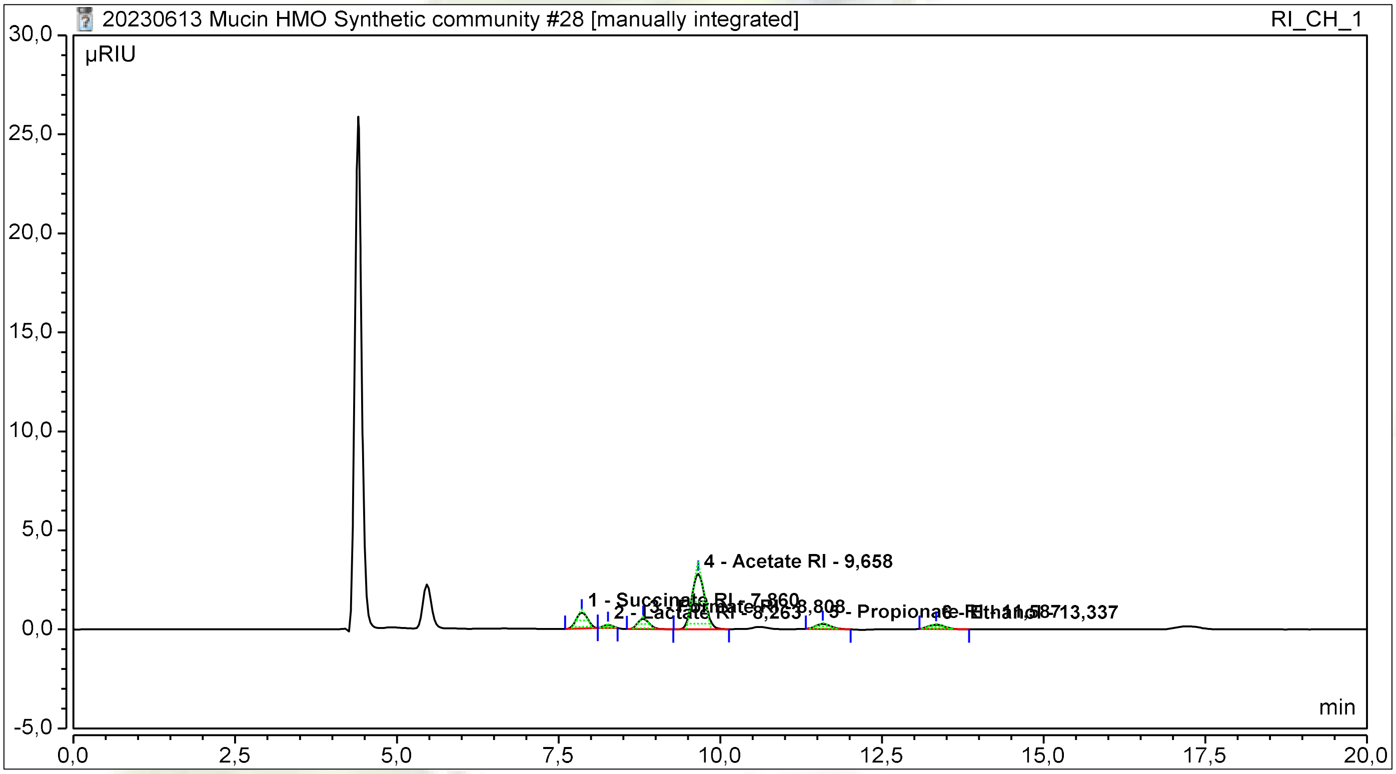

Peak Results

No.	Peak Name	Retention Time min	Width (50%) min	Type	Resolution (EP)	Asymmetry (EP)	Plates (EP)
n.a.	GlcNAc	n.a.	n.a.	n.a.	n.a.	n.a.	n.a.
n.a.	Citrate	n.a.	n.a.	n.a.	n.a.	n.a.	n.a.
n.a.	Glucose	n.a.	n.a.	n.a.	n.a.	n.a.	n.a.
n.a.	Galactose	n.a.	n.a.	n.a.	n.a.	n.a.	n.a.
n.a.	Fucose	n.a.	n.a.	n.a.	n.a.	n.a.	n.a.
1	Succinate RI	7,860	0,197	BMB	1,30	1,03	8820
2	Lactate RI	8,263	0,168	BMB*	1,75	1,00	13347
n.a.	glycerol	n.a.	n.a.	n.a.	n.a.	n.a.	n.a.
3	Formate RI	8,808	0,200	BMB	2,36	1,11	10767
4	Acetate RI	9,658	0,225	BMB	4,69	1,08	10207
n.a.	1,2 PDO RI	n.a.	n.a.	n.a.	n.a.	n.a.	n.a.
n.a.	1,3-PDO	n.a.	n.a.	n.a.	n.a.	n.a.	n.a.
5	Propionate RI	11,587	0,260	BMB*	3,77	1,15	10969
n.a.	1,3-PDO	n.a.	n.a.	n.a.	n.a.	n.a.	n.a.
n.a.	2-3 BDO	n.a.	n.a.	n.a.	n.a.	n.a.	n.a.
6	Ethanol	13,337	0,288	BMB*	n.a.	1,14	11903
n.a.	Isobutyrate RI	n.a.	n.a.	n.a.	n.a.	n.a.	n.a.
n.a.	Butyrate RI	n.a.	n.a.	n.a.	n.a.	n.a.	n.a.

Chromatogram and SST Results

Injection Details

Injection Name:	**54 5HMO1 t120 r3**	*Run Time (min):*	**20,00**
Vial Number:	**3:B9**	*Injection Volume:*	**10,00**
Injection Type:	**Unknown**	*Channel:*	**RI_CH_1**
Calibration Level:		*Wavelength:*	**n.a.**
Instrument Method:	**Default method LC2030C 45 gr 20 min**	*Bandwidth:*	**n.a.**
Processing Method:	**Processing Method LC2030 45 gr**	*Dilution Factor:*	**1,0000**
Injection Date/Time:	**13-jun-23 21:08**	*Sample Weight:*	**1,0000**

Chromatogram

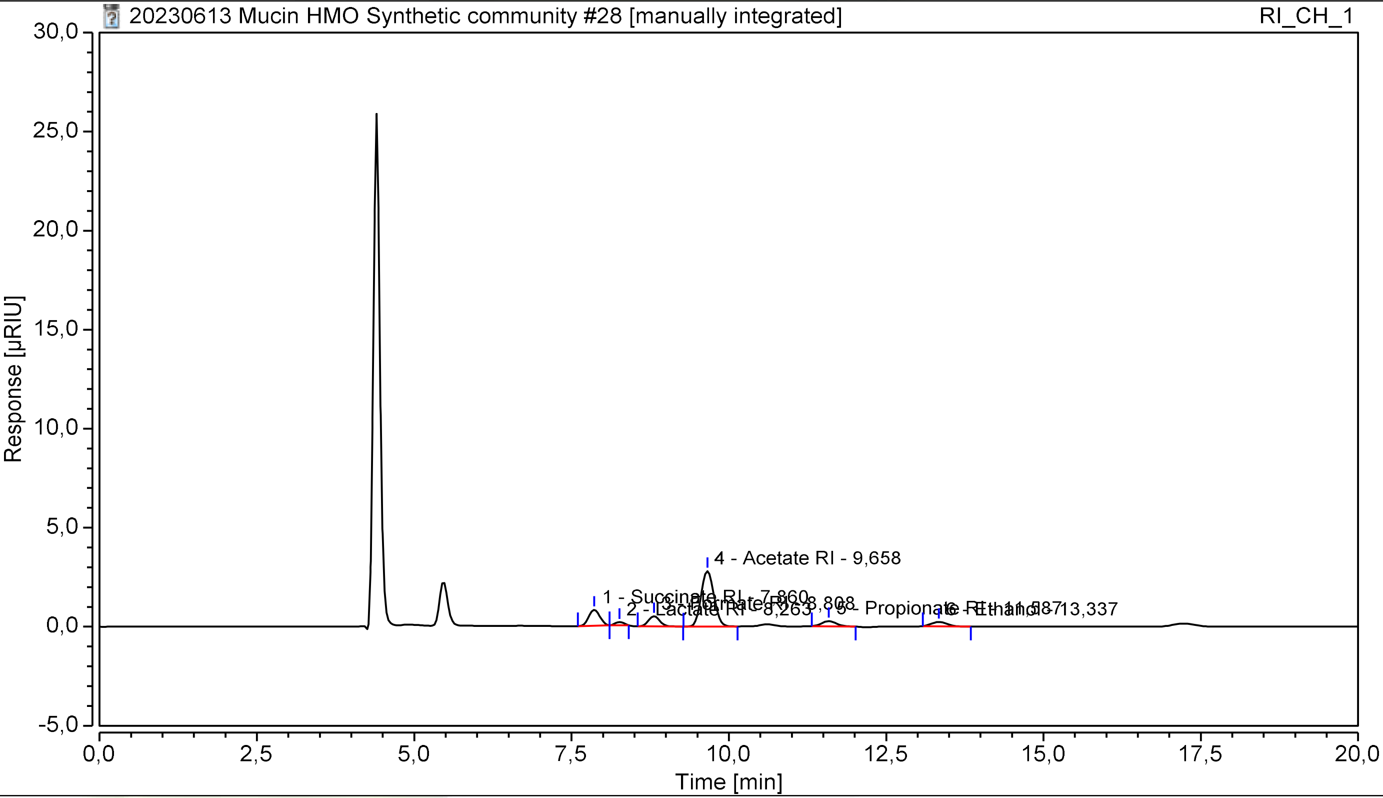

SST Results

No.	Name	Inj.Condition	Peak	Test Result	Injection

Number of executed test cases: **n.a.** **Total Result:** **Passed**

Chromatogram

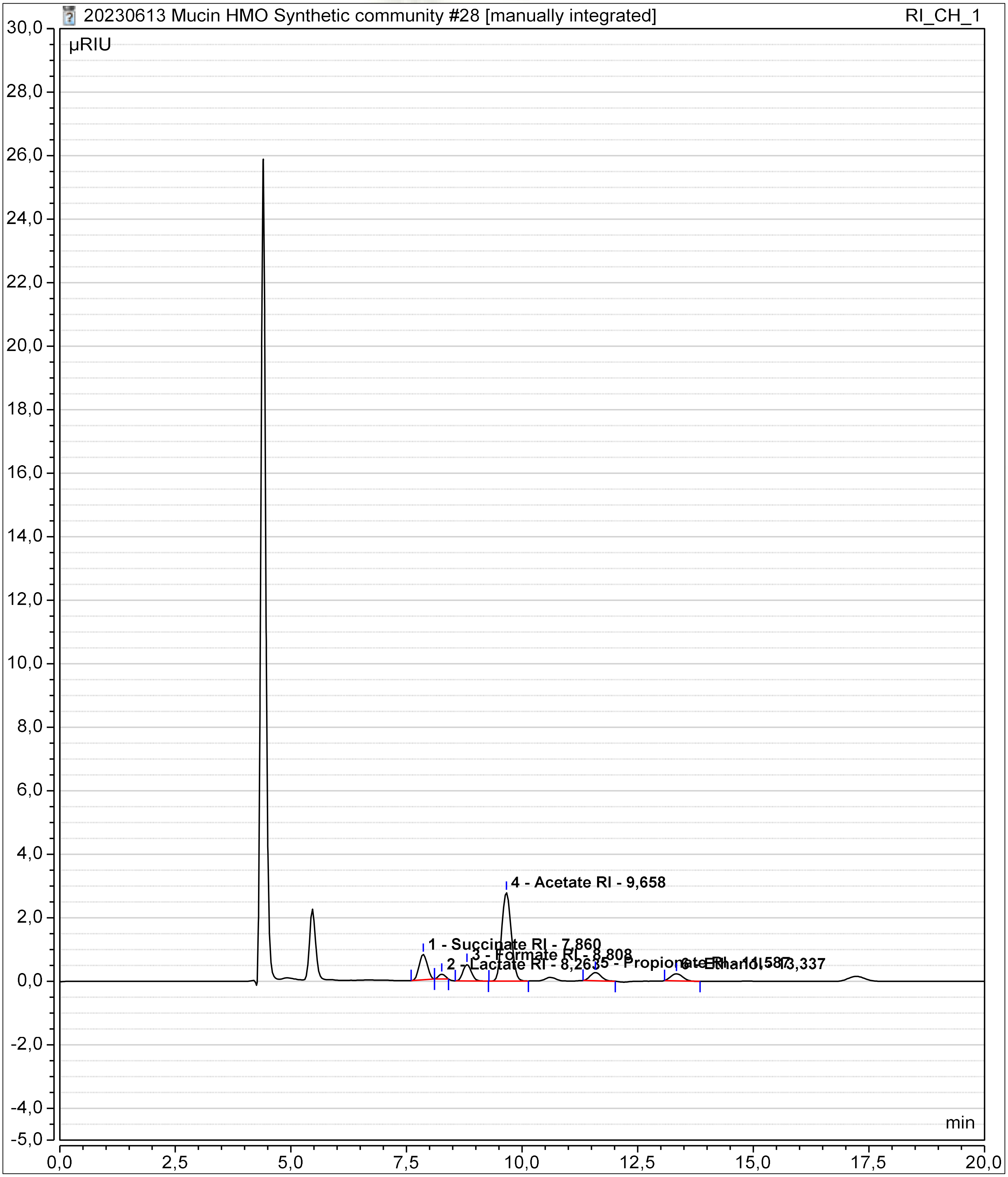

Chromatogram and Results

Injection Details

Injection Name:	**10 GOSFOS t24 r1**	*Run Time (min):*	**20,00**
Vial Number:	**3:B10**	*Injection Volume:*	**10,00**
Injection Type:	**Unknown**	*Channel:*	**RI_CH_1**
Calibration Level:		*Wavelength:*	**n.a.**
Instrument Method:	**Default method LC2030C 45 gr 20 min**	*Bandwidth:*	**n.a.**
Processing Method:	**Processing Method LC2030 45 gr**	*Dilution Factor:*	**1,0000**
Injection Date/Time:	**13-jun-23 21:28**	*Sample Weight:*	**1,0000**

Chromatogram

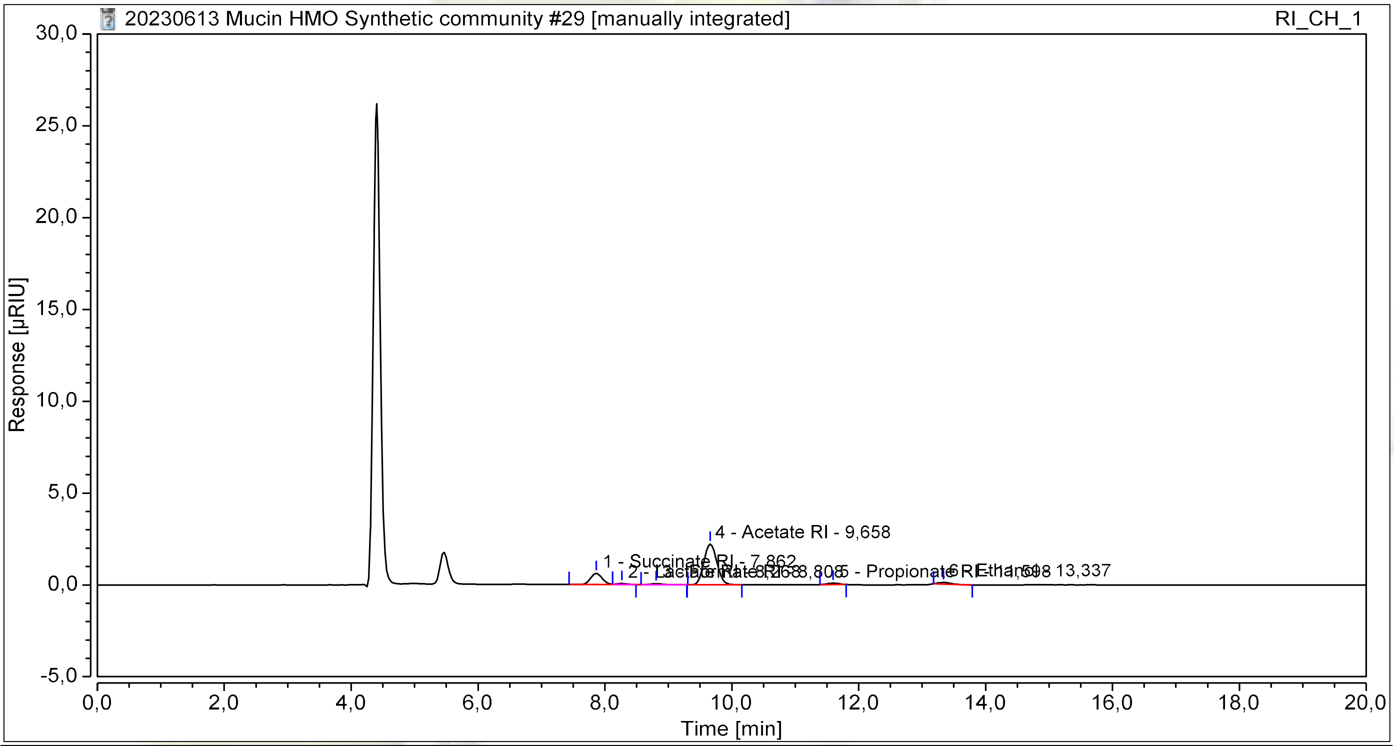

Integration Results

No.	Peak Name	Retention Time min	Area µRIU*min	Height µRIU	Relative Area %	Relative Height %	Amount
n.a.	GlcNAc	n.a.	n.a.	n.a.	n.a.	n.a.	n.a.
n.a.	Citrate	n.a.	n.a.	n.a.	n.a.	n.a.	n.a.
n.a.	Glucose	n.a.	n.a.	n.a.	n.a.	n.a.	n.a.
n.a.	Galactose	n.a.	n.a.	n.a.	n.a.	n.a.	n.a.
n.a.	Fucose	n.a.	n.a.	n.a.	n.a.	n.a.	n.a.
1	Succinate RI	7,862	0,138	0,611	19,03	19,74	n.a.
2	Lactate RI	8,268	0,008	0,046	1,12	1,49	0,2340
n.a.	glycerol	n.a.	n.a.	n.a.	n.a.	n.a.	n.a.
3	Formate RI	8,808	0,014	0,061	1,88	1,97	1,4247
4	Acetate RI	9,658	0,527	2,216	72,97	71,63	32,4619
n.a.	1,2 PDO RI	n.a.	n.a.	n.a.	n.a.	n.a.	n.a.
n.a.	1,3-PDO	n.a.	n.a.	n.a.	n.a.	n.a.	n.a.
5	Propionate RI	11,598	0,016	0,072	2,18	2,32	0,6345
n.a.	1,3-PDO	n.a.	n.a.	n.a.	n.a.	n.a.	n.a.
n.a.	2-3 BDO	n.a.	n.a.	n.a.	n.a.	n.a.	n.a.
6	Ethanol	13,337	0,020	0,088	2,83	2,85	0,1882
n.a.	Isobutyrate RI	n.a.	n.a.	n.a.	n.a.	n.a.	n.a.
n.a.	Butyrate RI	n.a.	n.a.	n.a.	n.a.	n.a.	n.a.
**Total:**			**0,723**	**3,093**	**100,00**	**100,00**	

Peak Analysis

Injection Details

Injection Name:	**10 GOSFOS t24 r1**	*Run Time (min):*	**20,00**
Vial Number:	**3:B10**	*Injection Volume:*	**10,00**
Injection Type:	**Unknown**	*Channel:*	**RI_CH_1**
Calibration Level:		*Wavelength:*	**n.a.**
Instrument Method:	**Default method LC2030C 45 gr 20 min**	*Bandwidth:*	**n.a.**
Processing Method:	**Processing Method LC2030 45 gr**	*Dilution Factor:*	**1,0000**
Injection Date/Time:	**13-jun-23 21:28**	*Sample Weight:*	**1,0000**

Chromatogram

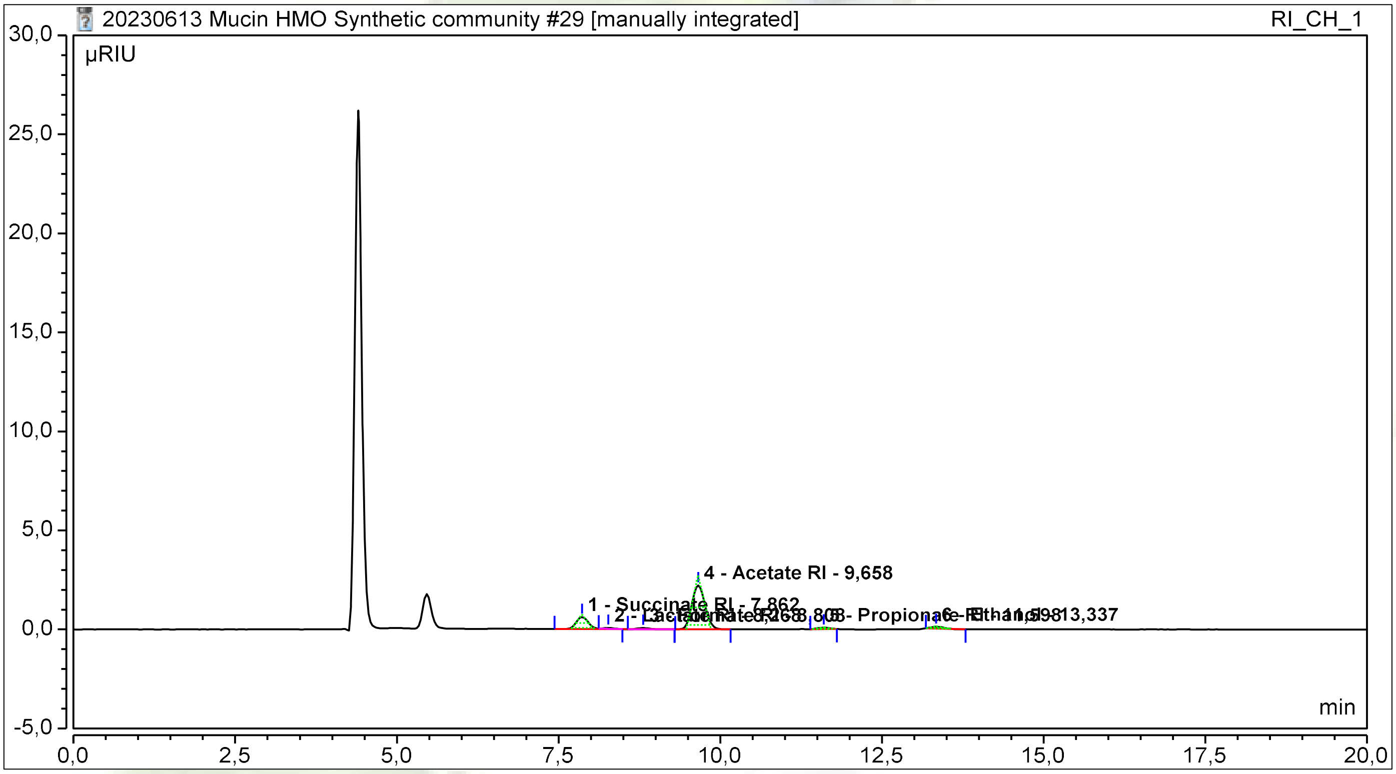

Peak Results

No.	Peak Name	Retention Time min	Width (50%) min	Type	Resolution (EP)	Asymmetry (EP)	Plates (EP)
n.a.	GlcNAc	n.a.	n.a.	n.a.	n.a.	n.a.	n.a.
n.a.	Citrate	n.a.	n.a.	n.a.	n.a.	n.a.	n.a.
n.a.	Glucose	n.a.	n.a.	n.a.	n.a.	n.a.	n.a.
n.a.	Galactose	n.a.	n.a.	n.a.	n.a.	n.a.	n.a.
n.a.	Fucose	n.a.	n.a.	n.a.	n.a.	n.a.	n.a.
1	Succinate RI	7,862	0,203	BMB	4,96	1,12	8344
2	Lactate RI	8,268	n.a.	Rd	n.a.	n.a.	n.a.
n.a.	glycerol	n.a.	n.a.	n.a.	n.a.	n.a.	n.a.
3	Formate RI	8,808	n.a.	Rd	n.a.	n.a.	n.a.
4	Acetate RI	9,658	0,225	BMB	5,17	1,09	10203
n.a.	1,2 PDO RI	n.a.	n.a.	n.a.	n.a.	n.a.	n.a.
n.a.	1,3-PDO	n.a.	n.a.	n.a.	n.a.	n.a.	n.a.
5	Propionate RI	11,598	0,218	BMB*	4,47	1,02	15670
n.a.	1,3-PDO	n.a.	n.a.	n.a.	n.a.	n.a.	n.a.
n.a.	2-3 BDO	n.a.	n.a.	n.a.	n.a.	n.a.	n.a.
6	Ethanol	13,337	0,241	BMB*	n.a.	1,30	16938
n.a.	Isobutyrate RI	n.a.	n.a.	n.a.	n.a.	n.a.	n.a.
n.a.	Butyrate RI	n.a.	n.a.	n.a.	n.a.	n.a.	n.a.

Chromatogram and SST Results

Injection Details

Injection Name:	**10 GOSFOS t24 r1**	*Run Time (min):*	**20,00**
Vial Number:	**3:B10**	*Injection Volume:*	**10,00**
Injection Type:	**Unknown**	*Channel:*	**RI_CH_1**
Calibration Level:		*Wavelength:*	**n.a.**
Instrument Method:	**Default method LC2030C 45 gr 20 min**	*Bandwidth:*	**n.a.**
Processing Method:	**Processing Method LC2030 45 gr**	*Dilution Factor:*	**1,0000**
Injection Date/Time:	**13-jun-23 21:28**	*Sample Weight:*	**1,0000**

Chromatogram

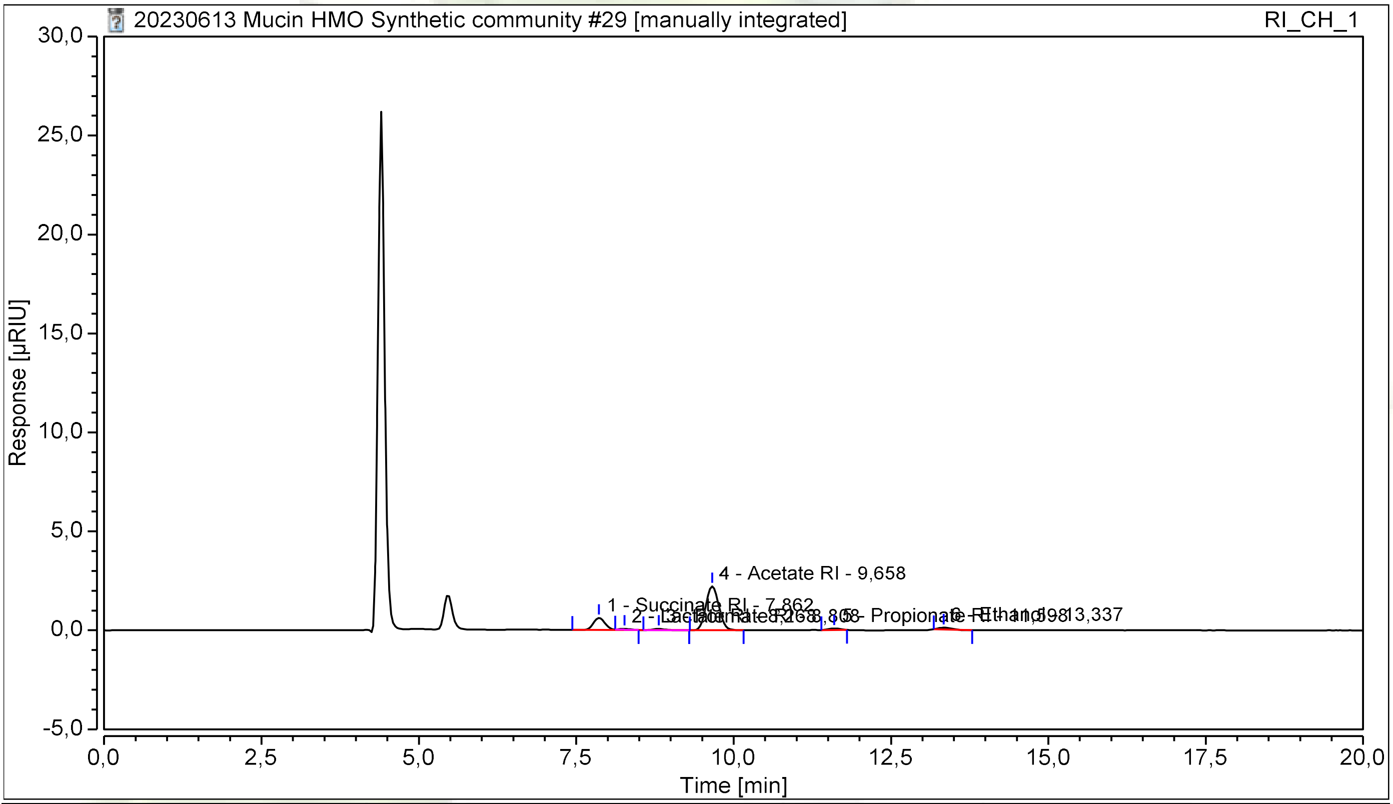

SST Results

No.	Name	Inj.Condition	Peak	Test Result	Injection

Number of executed test cases: **n.a.** **Total Result:** **Passed**

Chromatogram

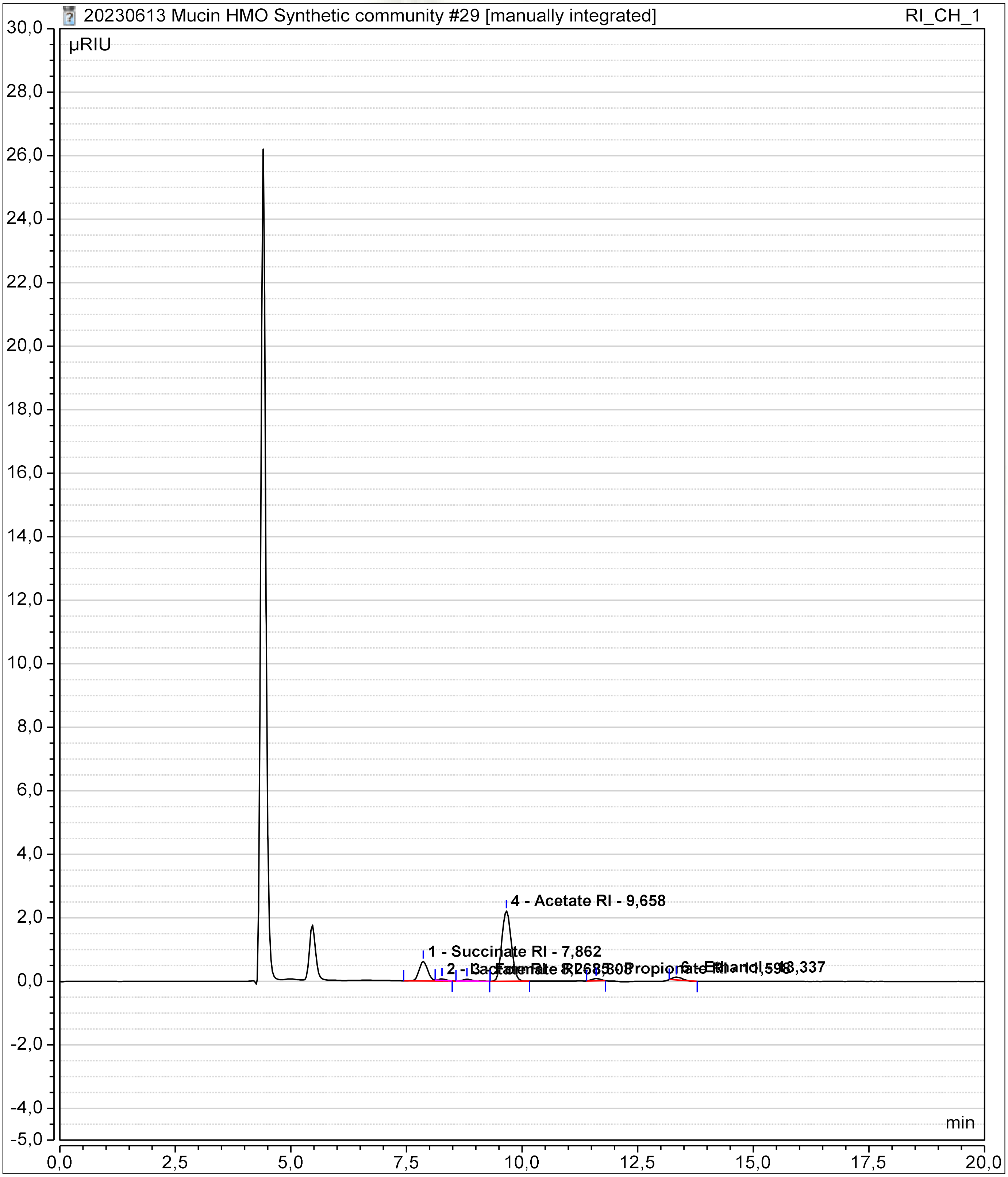

Chromatogram and Results

Injection Details

Injection Name:	**11 GOSFOS t24 r2**	*Run Time (min):*	**20,00**
Vial Number:	**3:B11**	*Injection Volume:*	**10,00**
Injection Type:	**Unknown**	*Channel:*	**RI_CH_1**
Calibration Level:		*Wavelength:*	**n.a.**
Instrument Method:	**Default method LC2030C 45 gr 20 min**	*Bandwidth:*	**n.a.**
Processing Method:	**Processing Method LC2030 45 gr**	*Dilution Factor:*	**1,0000**
Injection Date/Time:	**13-jun-23 21:49**	*Sample Weight:*	**1,0000**

Chromatogram

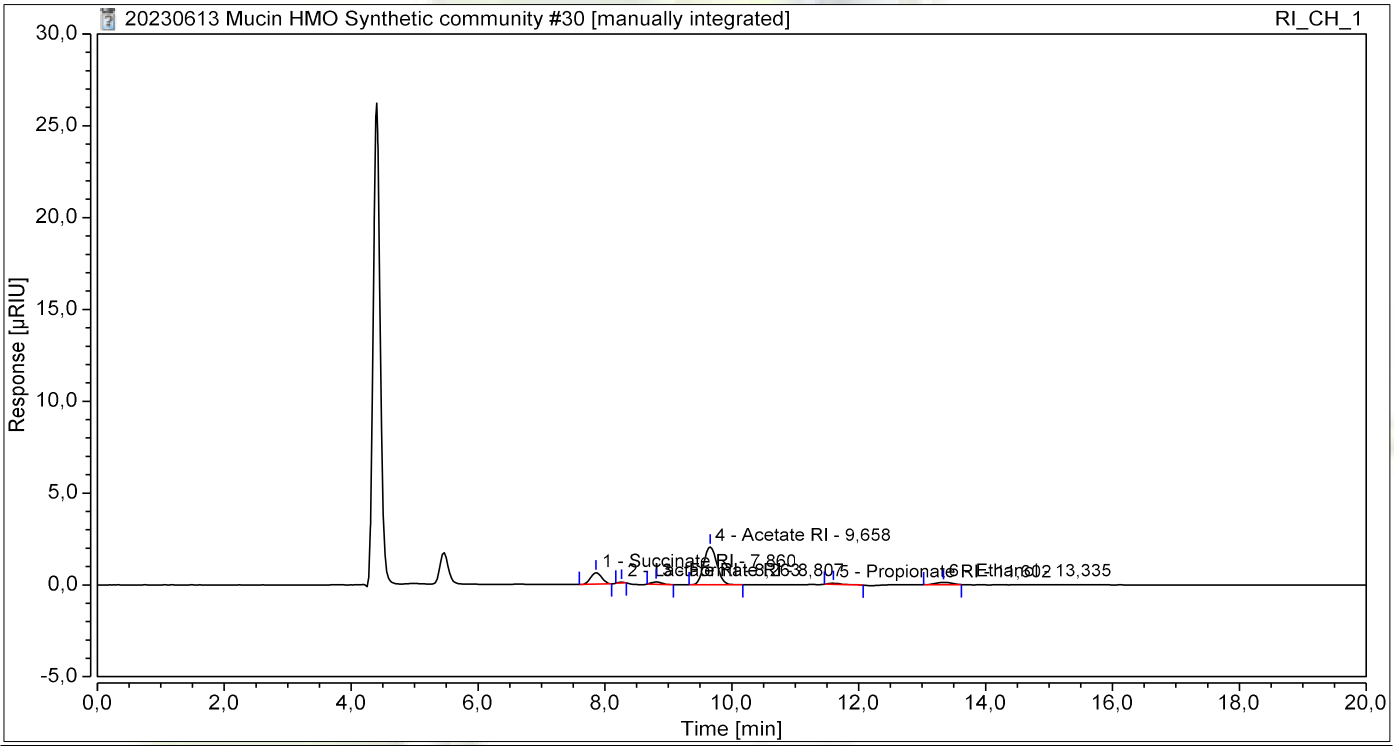

Integration Results

No.	Peak Name	Retention Time<br>min	Area<br>µRIU*min	Height<br>µRIU	Relative Area<br>%	Relative Height<br>%	Amount
n.a.	GlcNAc	n.a.	n.a.	n.a.	n.a.	n.a.	n.a.
n.a.	Citrate	n.a.	n.a.	n.a.	n.a.	n.a.	n.a.
n.a.	Glucose	n.a.	n.a.	n.a.	n.a.	n.a.	n.a.
n.a.	Galactose	n.a.	n.a.	n.a.	n.a.	n.a.	n.a.
n.a.	Fucose	n.a.	n.a.	n.a.	n.a.	n.a.	n.a.
1	Succinate RI	7,860	0,127	0,619	18,08	20,26	n.a.
2	Lactate RI	8,263	0,005	0,050	0,78	1,64	0,1584
n.a.	glycerol	n.a.	n.a.	n.a.	n.a.	n.a.	n.a.
3	Formate RI	8,807	0,024	0,131	3,47	4,30	2,5577
4	Acetate RI	9,658	0,492	2,066	70,15	67,65	30,3054
n.a.	1,2 PDO RI	n.a.	n.a.	n.a.	n.a.	n.a.	n.a.
n.a.	1,3-PDO	n.a.	n.a.	n.a.	n.a.	n.a.	n.a.
5	Propionate RI	11,602	0,016	0,062	2,24	2,02	0,6337
n.a.	1,3-PDO	n.a.	n.a.	n.a.	n.a.	n.a.	n.a.
n.a.	2-3 BDO	n.a.	n.a.	n.a.	n.a.	n.a.	n.a.
6	Ethanol	13,335	0,037	0,126	5,29	4,13	0,3418
n.a.	Isobutyrate RI	n.a.	n.a.	n.a.	n.a.	n.a.	n.a.
n.a.	Butyrate RI	n.a.	n.a.	n.a.	n.a.	n.a.	n.a.
**Total:**			**0,702**	**3,054**	**100,00**	**100,00**	

Chromeleon (c) Dionex
Version 7.2.10.25868

Peak Analysis

Injection Details

Injection Name:	**11 GOSFOS t24 r2**	*Run Time (min):*	**20,00**
Vial Number:	**3:B11**	*Injection Volume:*	**10,00**
Injection Type:	**Unknown**	*Channel:*	**RI_CH_1**
Calibration Level:		*Wavelength:*	**n.a.**
Instrument Method:	**Default method LC2030C 45 gr 20 min**	*Bandwidth:*	**n.a.**
Processing Method:	**Processing Method LC2030 45 gr**	*Dilution Factor:*	**1,0000**
Injection Date/Time:	**13-jun-23 21:49**	*Sample Weight:*	**1,0000**

Chromatogram

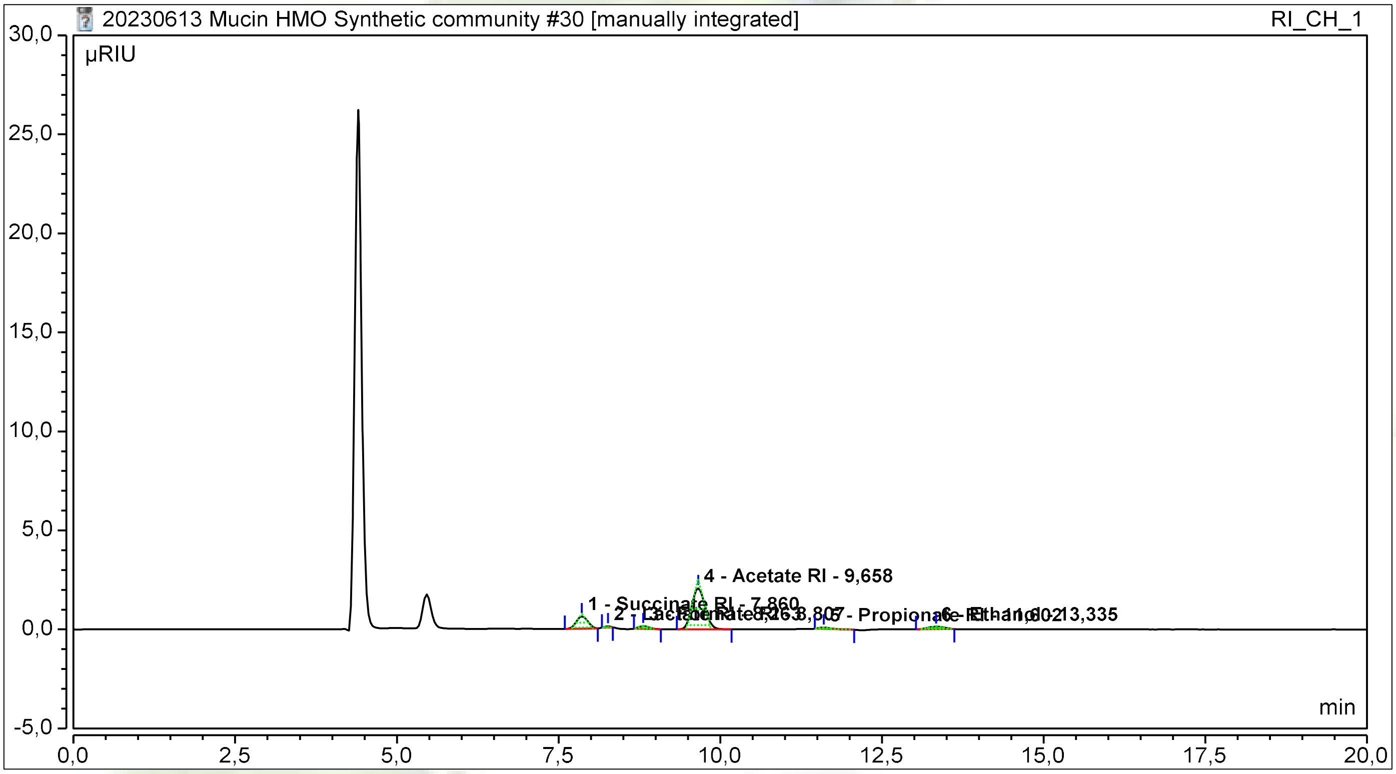

Peak Results

No.	Peak Name	Retention Time min	Width (50%) min	Type	Resolution (EP)	Asymmetry (EP)	Plates (EP)
n.a.	GlcNAc	n.a.	n.a.	n.a.	n.a.	n.a.	n.a.
n.a.	Citrate	n.a.	n.a.	n.a.	n.a.	n.a.	n.a.
n.a.	Glucose	n.a.	n.a.	n.a.	n.a.	n.a.	n.a.
n.a.	Galactose	n.a.	n.a.	n.a.	n.a.	n.a.	n.a.
n.a.	Fucose	n.a.	n.a.	n.a.	n.a.	n.a.	n.a.
1	Succinate RI	7,860	0,197	BMB	1,53	1,04	8789
2	Lactate RI	8,263	0,114	BMB*	2,16	0,91	29171
n.a.	glycerol	n.a.	n.a.	n.a.	n.a.	n.a.	n.a.
3	Formate RI	8,807	0,183	BMB*	2,46	1,23	12897
4	Acetate RI	9,658	0,225	BMB	5,20	1,09	10185
n.a.	1,2 PDO RI	n.a.	n.a.	n.a.	n.a.	n.a.	n.a.
n.a.	1,3-PDO	n.a.	n.a.	n.a.	n.a.	n.a.	n.a.
5	Propionate RI	11,602	0,216	BMB*	4,06	2,19	16038
n.a.	1,3-PDO	n.a.	n.a.	n.a.	n.a.	n.a.	n.a.
n.a.	2-3 BDO	n.a.	n.a.	n.a.	n.a.	n.a.	n.a.
6	Ethanol	13,335	0,288	BMB*	n.a.	1,00	11866
n.a.	Isobutyrate RI	n.a.	n.a.	n.a.	n.a.	n.a.	n.a.
n.a.	Butyrate RI	n.a.	n.a.	n.a.	n.a.	n.a.	n.a.

Chromatogram and SST Results

Injection Details

Injection Name:	**11 GOSFOS t24 r2**	*Run Time (min):*	**20,00**
Vial Number:	**3:B11**	*Injection Volume:*	**10,00**
Injection Type:	**Unknown**	*Channel:*	**RI_CH_1**
Calibration Level:		*Wavelength:*	**n.a.**
Instrument Method:	**Default method LC2030C 45 gr 20 min**	*Bandwidth:*	**n.a.**
Processing Method:	**Processing Method LC2030 45 gr**	*Dilution Factor:*	**1,0000**
Injection Date/Time:	**13-jun-23 21:49**	*Sample Weight:*	**1,0000**

Chromatogram

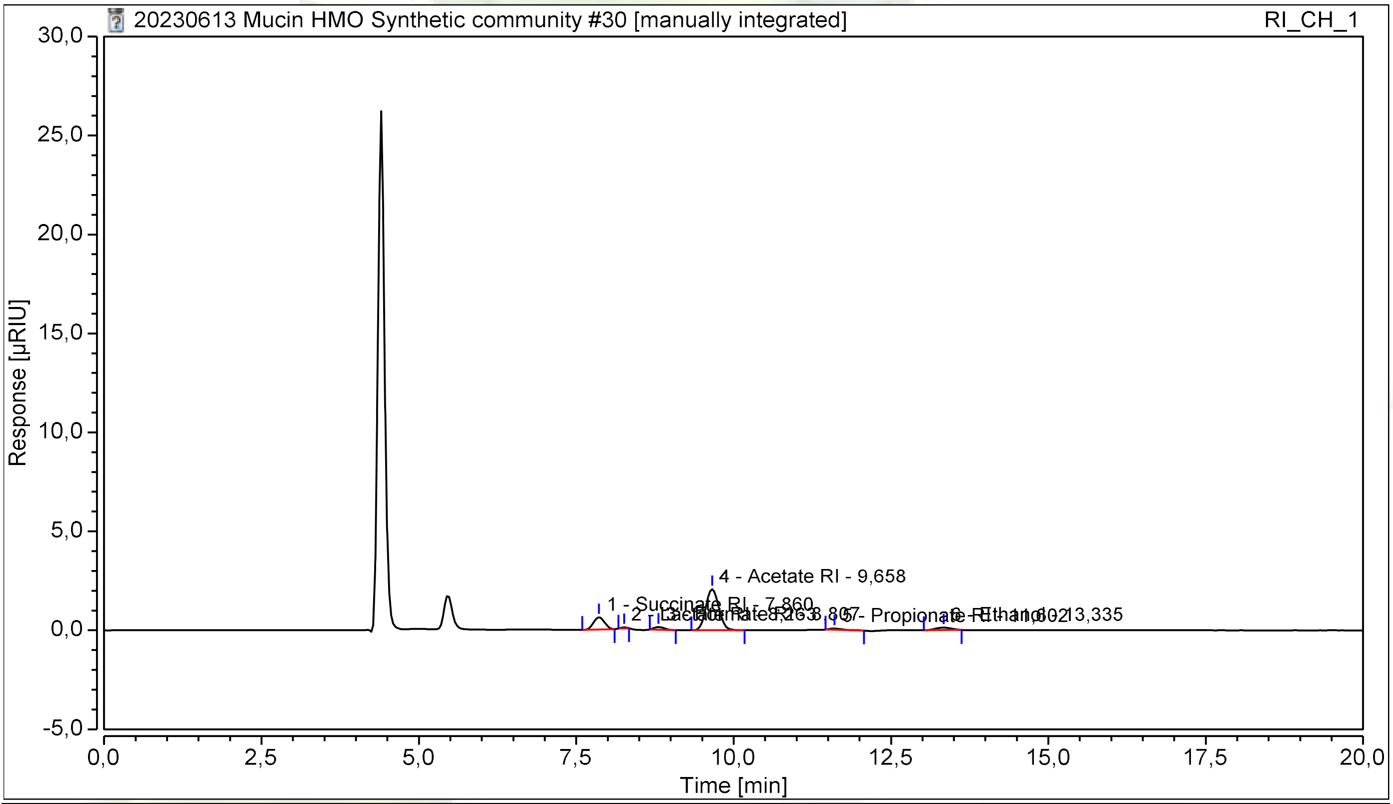

SST Results

No.	Name	Inj.Condition	Peak	Test Result	Injection
**Number of executed test cases:**	**n.a.**		**Total Result:**	**Passed**	

Chromatogram

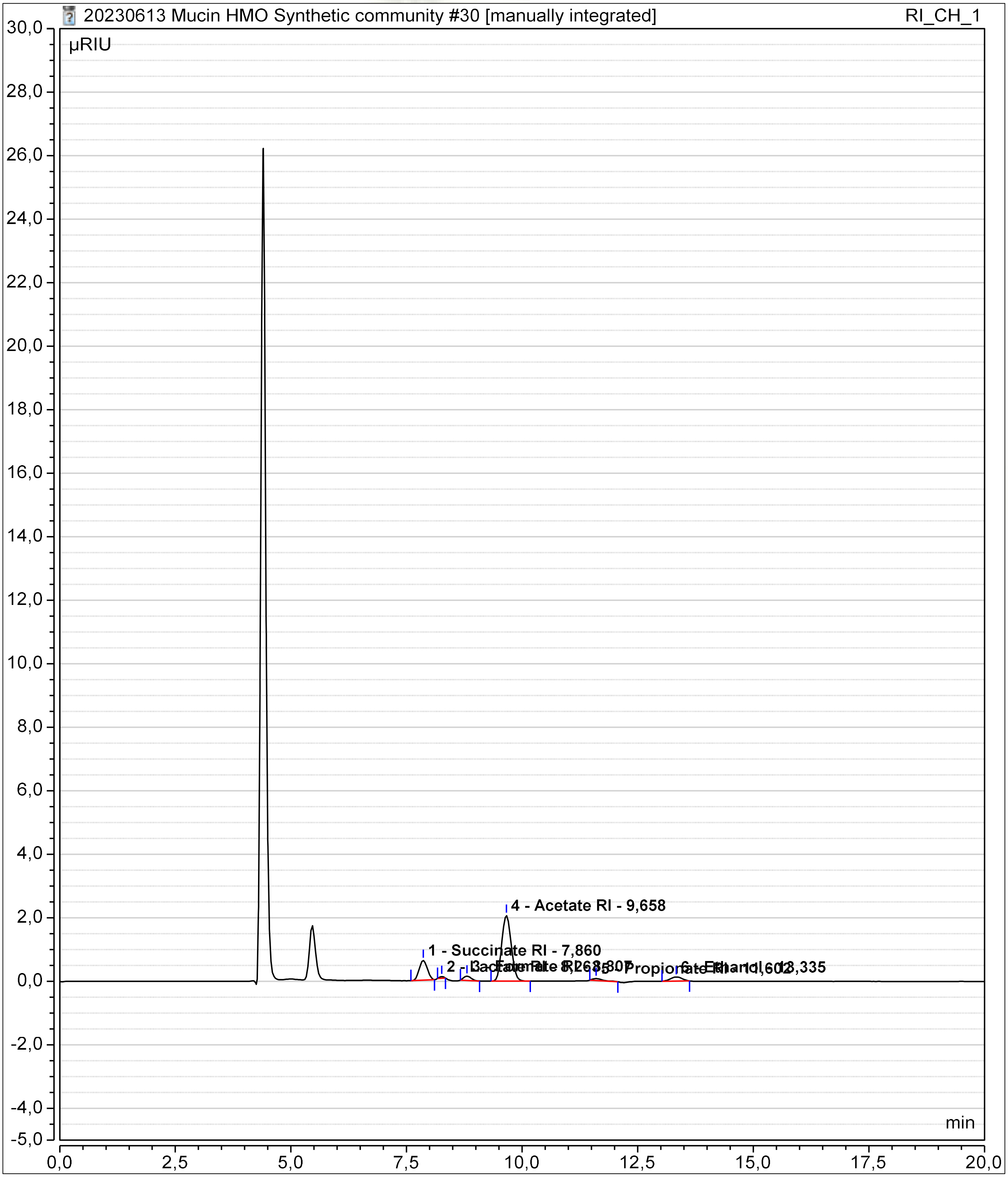

Chromatogram and Results

Injection Details

Injection Name:	**12 GOSFOS t24 r3**	*Run Time (min):*	**20,00**
Vial Number:	**3:B12**	*Injection Volume:*	**10,00**
Injection Type:	**Unknown**	*Channel:*	**RI_CH_1**
Calibration Level:		*Wavelength:*	**n.a.**
Instrument Method:	**Default method LC2030C 45 gr 20 min**	*Bandwidth:*	**n.a.**
Processing Method:	**Processing Method LC2030 45 gr**	*Dilution Factor:*	**1,0000**
Injection Date/Time:	**13-jun-23 22:09**	*Sample Weight:*	**1,0000**

Chromatogram

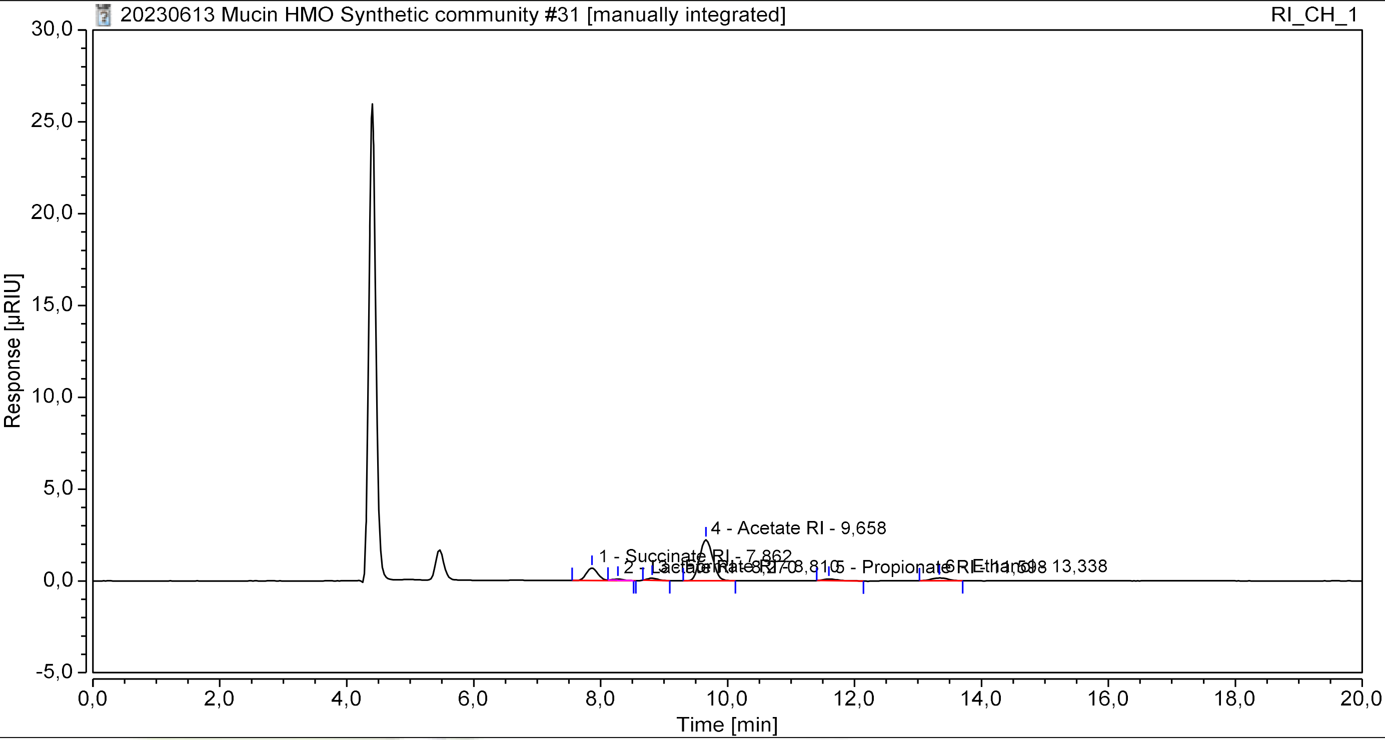

Integration Results

No.	Peak Name	Retention Time min	Area µRIU*min	Height µRIU	Relative Area %	Relative Height %	Amount
n.a.	GlcNAc	n.a.	n.a.	n.a.	n.a.	n.a.	n.a.
n.a.	Citrate	n.a.	n.a.	n.a.	n.a.	n.a.	n.a.
n.a.	Glucose	n.a.	n.a.	n.a.	n.a.	n.a.	n.a.
n.a.	Galactose	n.a.	n.a.	n.a.	n.a.	n.a.	n.a.
n.a.	Fucose	n.a.	n.a.	n.a.	n.a.	n.a.	n.a.
1	Succinate RI	7,862	0,152	0,679	19,31	20,39	n.a.
2	Lactate RI	8,270	0,011	0,063	1,42	1,89	0,3251
n.a.	glycerol	n.a.	n.a.	n.a.	n.a.	n.a.	n.a.
3	Formate RI	8,810	0,022	0,117	2,77	3,51	2,2918
4	Acetate RI	9,658	0,530	2,227	67,27	66,88	32,6182
n.a.	1,2 PDO RI	n.a.	n.a.	n.a.	n.a.	n.a.	n.a.
n.a.	1,3-PDO	n.a.	n.a.	n.a.	n.a.	n.a.	n.a.
5	Propionate RI	11,598	0,026	0,092	3,32	2,76	1,0517
n.a.	1,3-PDO	n.a.	n.a.	n.a.	n.a.	n.a.	n.a.
n.a.	2-3 BDO	n.a.	n.a.	n.a.	n.a.	n.a.	n.a.
6	Ethanol	13,338	0,047	0,152	5,90	4,57	0,4284
n.a.	Isobutyrate RI	n.a.	n.a.	n.a.	n.a.	n.a.	n.a.
n.a.	Butyrate RI	n.a.	n.a.	n.a.	n.a.	n.a.	n.a.
**Total:**			**0,788**	**3,329**	**100,00**	**100,00**	

Peak Analysis

Injection Details

Injection Name:	**12 GOSFOS t24 r3**	*Run Time (min):*	**20,00**
Vial Number:	**3:B12**	*Injection Volume:*	**10,00**
Injection Type:	**Unknown**	*Channel:*	**RI_CH_1**
Calibration Level:		*Wavelength:*	**n.a.**
Instrument Method:	**Default method LC2030C 45 gr 20 min**	*Bandwidth:*	**n.a.**
Processing Method:	**Processing Method LC2030 45 gr**	*Dilution Factor:*	**1,0000**
Injection Date/Time:	**13-jun-23 22:09**	*Sample Weight:*	**1,0000**

Chromatogram

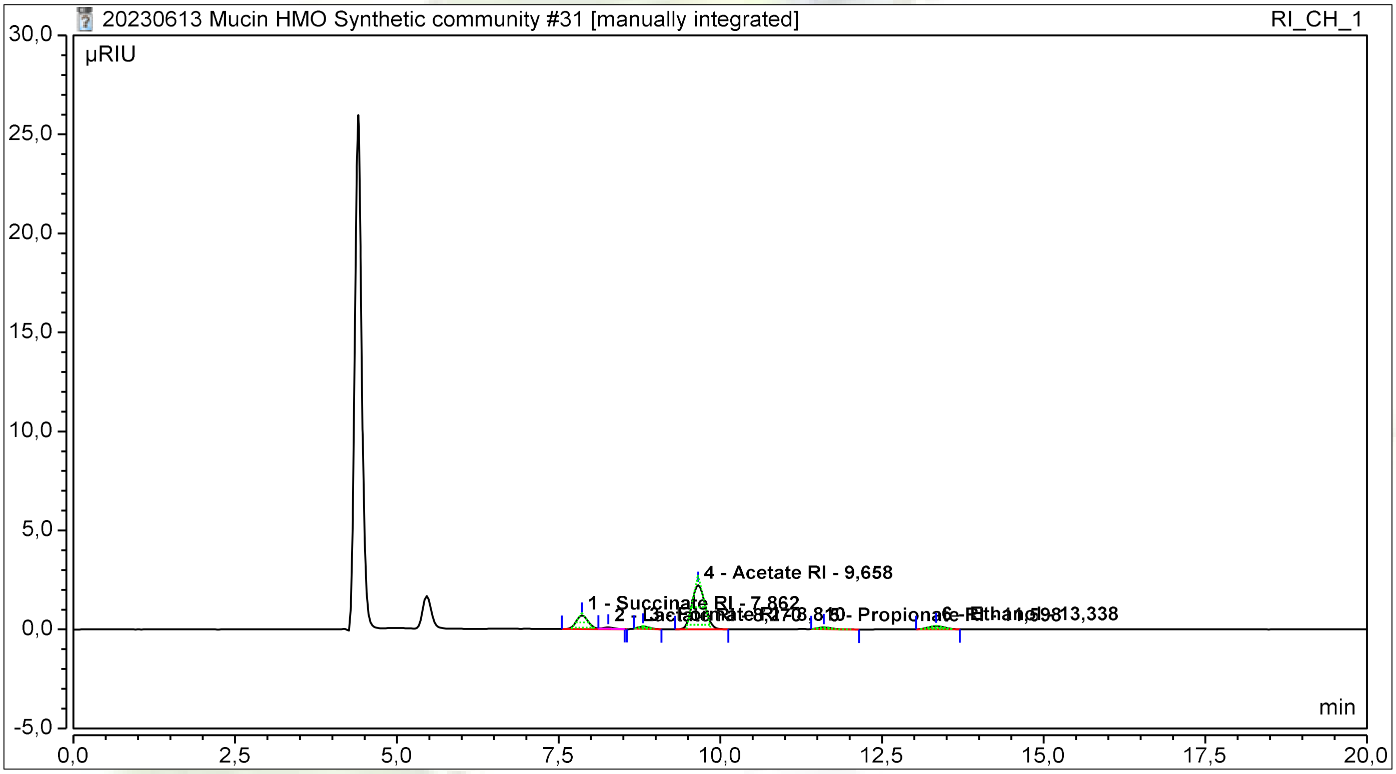

Peak Results

No.	Peak Name	Retention Time min	Width (50%) min	Type	Resolution (EP)	Asymmetry (EP)	Plates (EP)
n.a.	GlcNAc	n.a.	n.a.	n.a.	n.a.	n.a.	n.a.
n.a.	Citrate	n.a.	n.a.	n.a.	n.a.	n.a.	n.a.
n.a.	Glucose	n.a.	n.a.	n.a.	n.a.	n.a.	n.a.
n.a.	Galactose	n.a.	n.a.	n.a.	n.a.	n.a.	n.a.
n.a.	Fucose	n.a.	n.a.	n.a.	n.a.	n.a.	n.a.
1	Succinate RI	7,862	0,202	BMB	2,90	1,13	8371
2	Lactate RI	8,270	n.a.	Rd	n.a.	n.a.	n.a.
n.a.	glycerol	n.a.	n.a.	n.a.	n.a.	n.a.	n.a.
3	Formate RI	8,810	0,184	BMB*	2,45	1,20	12735
4	Acetate RI	9,658	0,225	BMB	4,88	1,09	10193
n.a.	1,2 PDO RI	n.a.	n.a.	n.a.	n.a.	n.a.	n.a.
n.a.	1,3-PDO	n.a.	n.a.	n.a.	n.a.	n.a.	n.a.
5	Propionate RI	11,598	0,244	BMB*	3,81	1,88	12508
n.a.	1,3-PDO	n.a.	n.a.	n.a.	n.a.	n.a.	n.a.
n.a.	2-3 BDO	n.a.	n.a.	n.a.	n.a.	n.a.	n.a.
6	Ethanol	13,338	0,295	BMB*	n.a.	1,05	11305
n.a.	Isobutyrate RI	n.a.	n.a.	n.a.	n.a.	n.a.	n.a.
n.a.	Butyrate RI	n.a.	n.a.	n.a.	n.a.	n.a.	n.a.

Chromeleon (c) Dionex
Version 7.2.10.25868

Chromatogram and SST Results

Injection Details

Injection Name:	**12 GOSFOS t24 r3**	*Run Time (min):*	**20,00**
Vial Number:	**3:B12**	*Injection Volume:*	**10,00**
Injection Type:	**Unknown**	*Channel:*	**RI_CH_1**
Calibration Level:		*Wavelength:*	**n.a.**
Instrument Method:	**Default method LC2030C 45 gr 20 min**	*Bandwidth:*	**n.a.**
Processing Method:	**Processing Method LC2030 45 gr**	*Dilution Factor:*	**1,0000**
Injection Date/Time:	**13-jun-23 22:09**	*Sample Weight:*	**1,0000**

Chromatogram

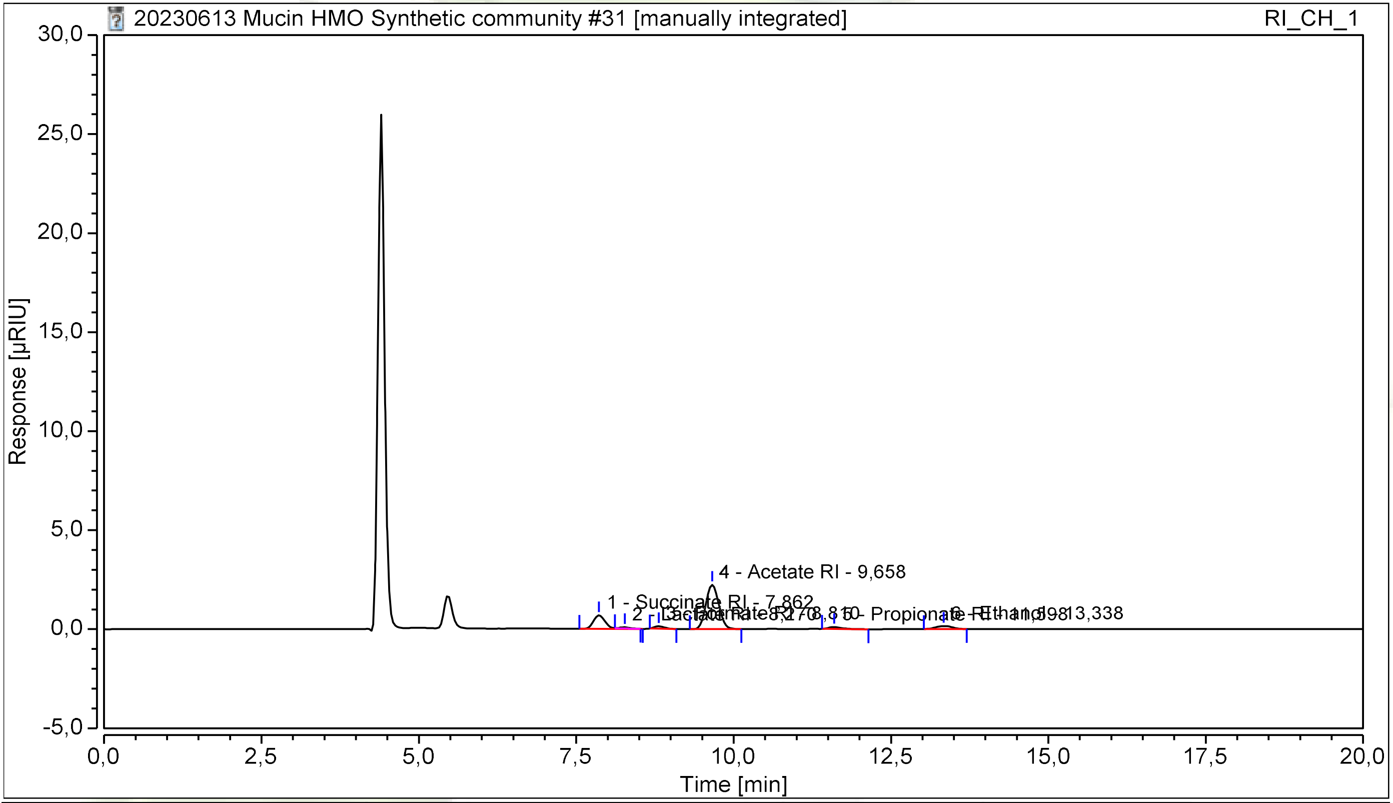

SST Results

No.	Name	Inj.Condition	Peak	Test Result	Injection
**Number of executed test cases:**	**n.a.**		**Total Result:**	**Passed**	

Chromatogram

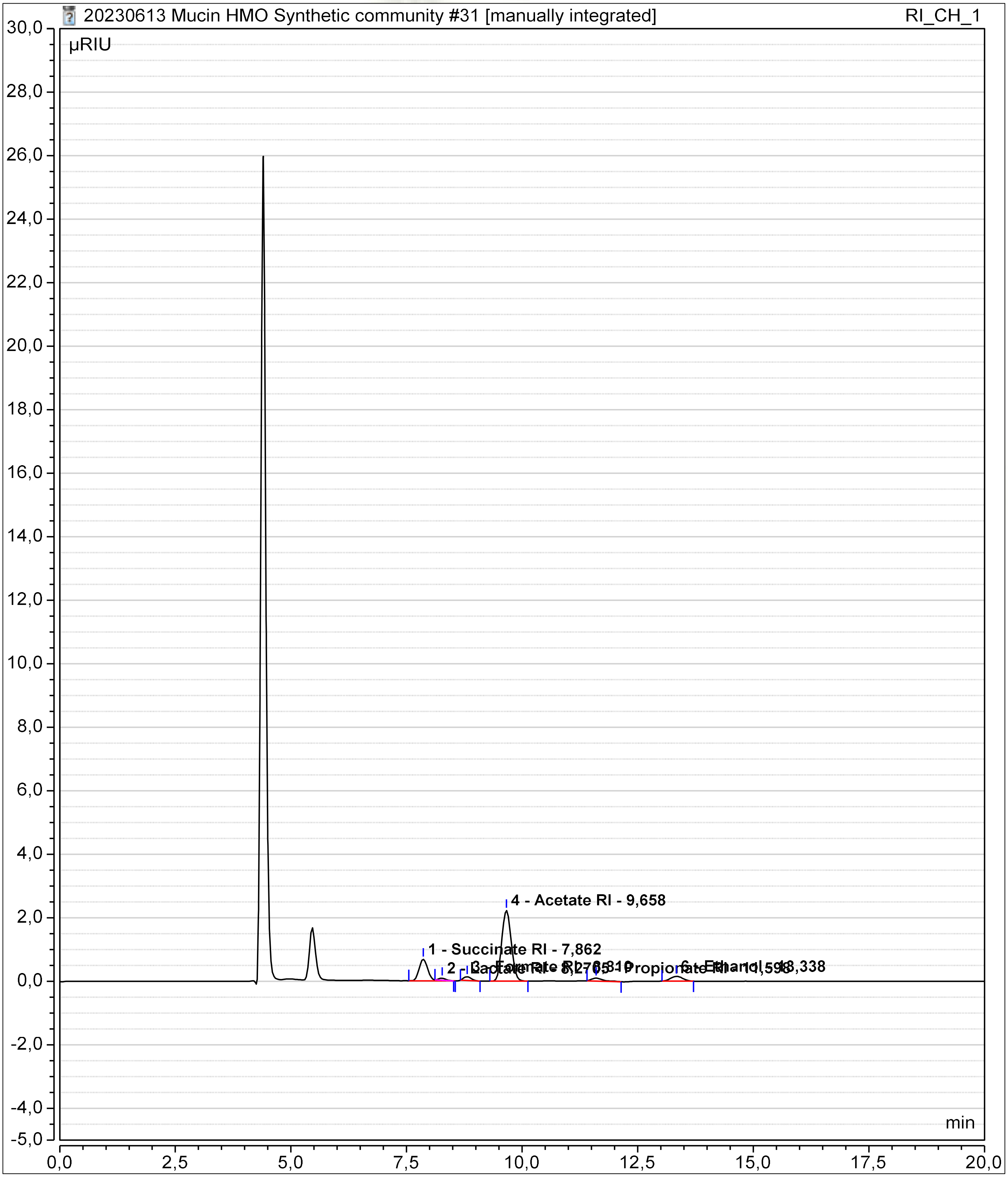

Chromatogram and Results

Injection Details

Injection Name:	**16 GOSFOSEXTR t24 r1**	*Run Time (min):*	**20,00**
Vial Number:	**3:C1**	*Injection Volume:*	**10,00**
Injection Type:	**Unknown**	*Channel:*	**RI_CH_1**
Calibration Level:		*Wavelength:*	**n.a.**
Instrument Method:	**Default method LC2030C 45 gr 20 min**	*Bandwidth:*	**n.a.**
Processing Method:	**Processing Method LC2030 45 gr**	*Dilution Factor:*	**1,0000**
Injection Date/Time:	**13-jun-23 22:30**	*Sample Weight:*	**1,0000**

Chromatogram

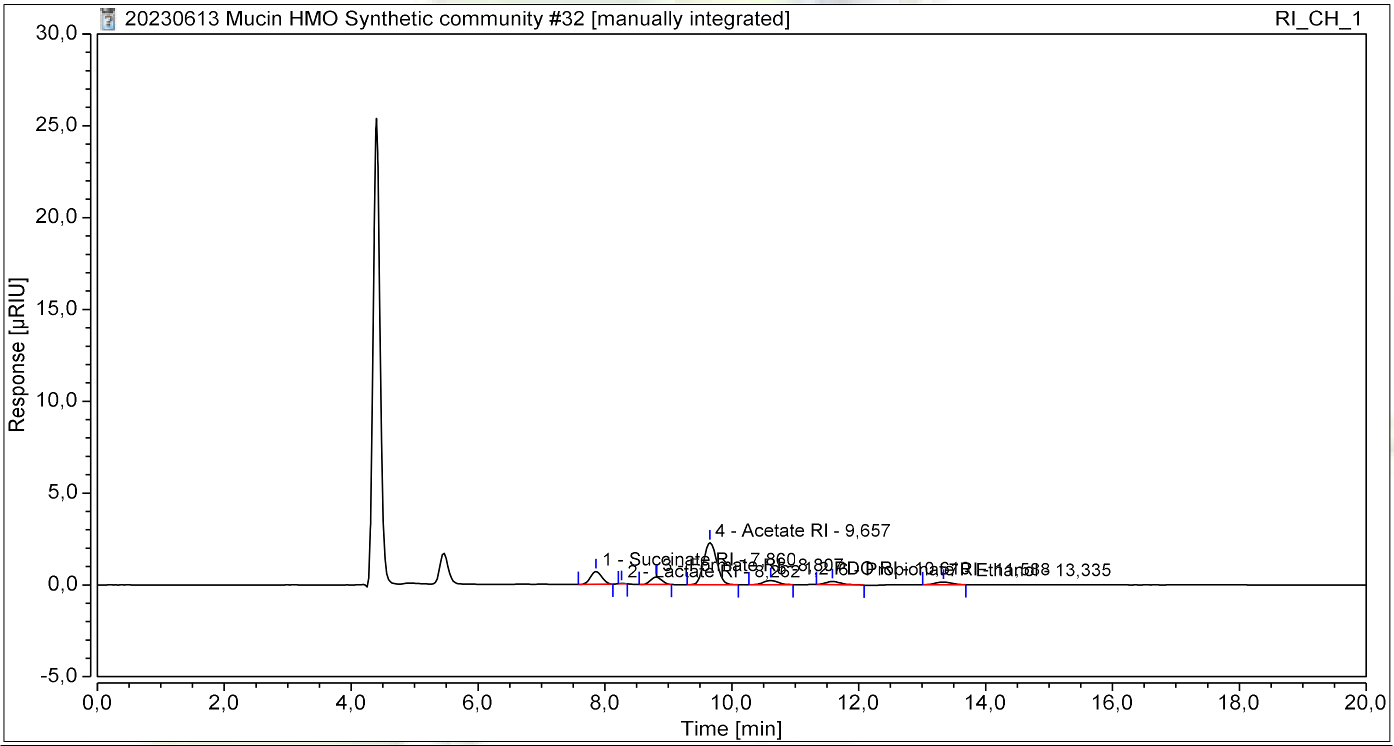

Integration Results

No.	Peak Name	Retention Time<br>min	Area<br>µRIU*min	Height<br>µRIU	Relative Area<br>%	Relative Height<br>%	Amount
n.a.	GlcNAc	n.a.	n.a.	n.a.	n.a.	n.a.	n.a.
n.a.	Citrate	n.a.	n.a.	n.a.	n.a.	n.a.	n.a.
n.a.	Glucose	n.a.	n.a.	n.a.	n.a.	n.a.	n.a.
n.a.	Galactose	n.a.	n.a.	n.a.	n.a.	n.a.	n.a.
n.a.	Fucose	n.a.	n.a.	n.a.	n.a.	n.a.	n.a.
1	Succinate RI	7,860	0,144	0,691	15,55	17,59	n.a.
2	Lactate RI	8,262	0,001	0,012	0,13	0,32	0,0355
n.a.	glycerol	n.a.	n.a.	n.a.	n.a.	n.a.	n.a.
3	Formate RI	8,807	0,081	0,396	8,75	10,09	8,4827
4	Acetate RI	9,657	0,544	2,286	58,97	58,23	33,5020
5	1,2 PDO RI	10,610	0,059	0,225	6,40	5,73	1,7572
n.a.	1,3-PDO	n.a.	n.a.	n.a.	n.a.	n.a.	n.a.
6	Propionate RI	11,588	0,050	0,169	5,36	4,30	1,9919
n.a.	1,3-PDO	n.a.	n.a.	n.a.	n.a.	n.a.	n.a.
n.a.	2-3 BDO	n.a.	n.a.	n.a.	n.a.	n.a.	n.a.
7	Ethanol	13,335	0,045	0,147	4,84	3,74	0,4112
n.a.	Isobutyrate RI	n.a.	n.a.	n.a.	n.a.	n.a.	n.a.
n.a.	Butyrate RI	n.a.	n.a.	n.a.	n.a.	n.a.	n.a.
**Total:**			**0,923**	**3,926**	**100,00**	**100,00**	

Chromeleon (c) Dionex
Version 7.2.10.25868

Peak Analysis

Injection Details

Injection Name:	**16 GOSFOSEXTR t24 r1**	*Run Time (min):*	**20,00**
Vial Number:	**3:C1**	*Injection Volume:*	**10,00**
Injection Type:	**Unknown**	*Channel:*	**RI_CH_1**
Calibration Level:		*Wavelength:*	**n.a.**
Instrument Method:	**Default method LC2030C 45 gr 20 min**	*Bandwidth:*	**n.a.**
Processing Method:	**Processing Method LC2030 45 gr**	*Dilution Factor:*	**1,0000**
Injection Date/Time:	**13-jun-23 22:30**	*Sample Weight:*	**1,0000**

Chromatogram

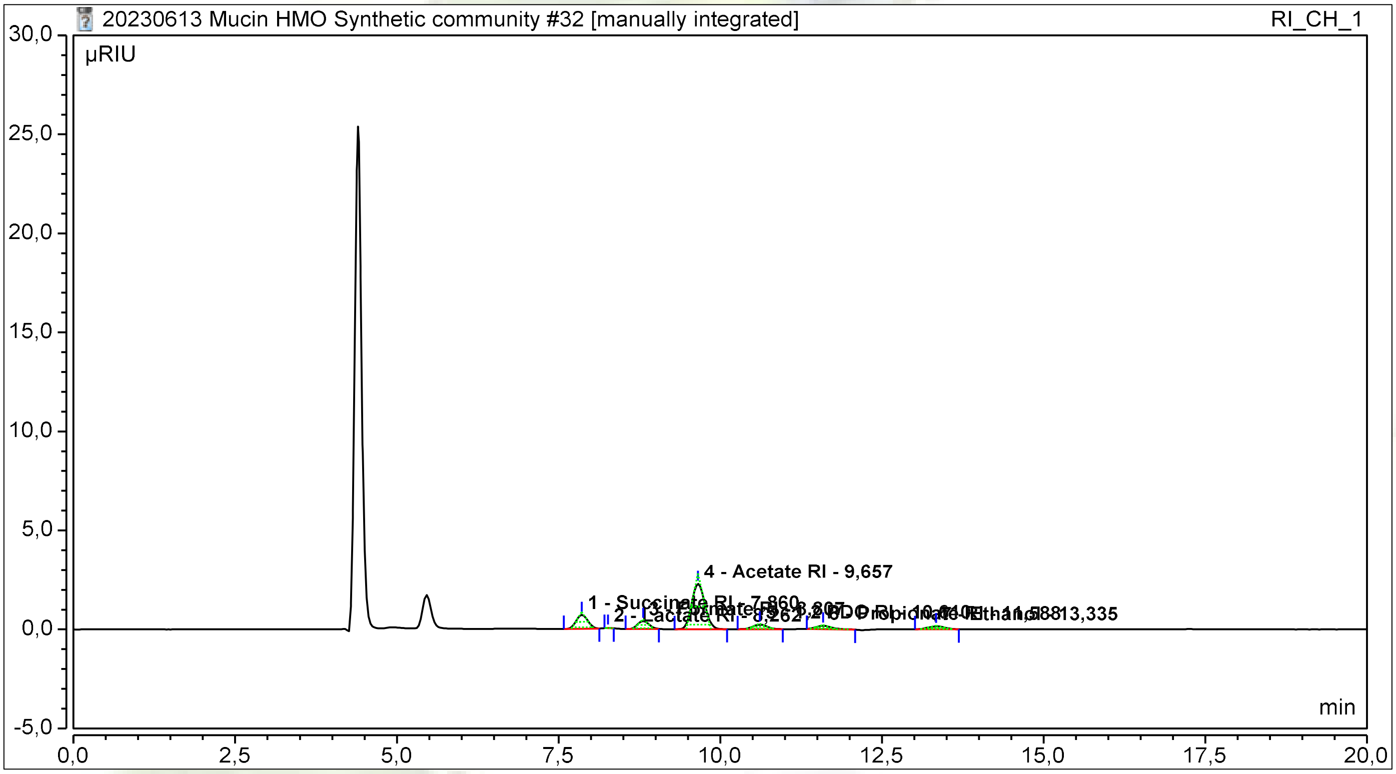

Peak Results

No.	Peak Name	Retention Time min	Width (50%) min	Type	Resolution (EP)	Asymmetry (EP)	Plates (EP)
n.a.	GlcNAc	n.a.	n.a.	n.a.	n.a.	n.a.	n.a.
n.a.	Citrate	n.a.	n.a.	n.a.	n.a.	n.a.	n.a.
n.a.	Glucose	n.a.	n.a.	n.a.	n.a.	n.a.	n.a.
n.a.	Galactose	n.a.	n.a.	n.a.	n.a.	n.a.	n.a.
n.a.	Fucose	n.a.	n.a.	n.a.	n.a.	n.a.	n.a.
1	Succinate RI	7,860	0,199	BMB	1,59	1,05	8654
2	Lactate RI	8,262	0,099	BMB*	2,17	1,42	38583
n.a.	glycerol	n.a.	n.a.	n.a.	n.a.	n.a.	n.a.
3	Formate RI	8,807	0,197	BMB*	2,38	1,06	11103
4	Acetate RI	9,657	0,225	BMB	2,37	1,08	10200
5	1,2 PDO RI	10,610	0,249	BMB*	2,26	1,06	10047
n.a.	1,3-PDO	n.a.	n.a.	n.a.	n.a.	n.a.	n.a.
6	Propionate RI	11,588	0,261	BMB*	3,71	1,54	10950
n.a.	1,3-PDO	n.a.	n.a.	n.a.	n.a.	n.a.	n.a.
n.a.	2-3 BDO	n.a.	n.a.	n.a.	n.a.	n.a.	n.a.
7	Ethanol	13,335	0,295	BMB*	n.a.	1,06	11306
n.a.	Isobutyrate RI	n.a.	n.a.	n.a.	n.a.	n.a.	n.a.
n.a.	Butyrate RI	n.a.	n.a.	n.a.	n.a.	n.a.	n.a.

Chromatogram and SST Results

Injection Details

Injection Name:	**16 GOSFOSEXTR t24 r1**	*Run Time (min):*	**20,00**
Vial Number:	**3:C1**	*Injection Volume:*	**10,00**
Injection Type:	**Unknown**	*Channel:*	**RI_CH_1**
Calibration Level:		*Wavelength:*	**n.a.**
Instrument Method:	**Default method LC2030C 45 gr 20 min**	*Bandwidth:*	**n.a.**
Processing Method:	**Processing Method LC2030 45 gr**	*Dilution Factor:*	**1,0000**
Injection Date/Time:	**13-jun-23 22:30**	*Sample Weight:*	**1,0000**

Chromatogram

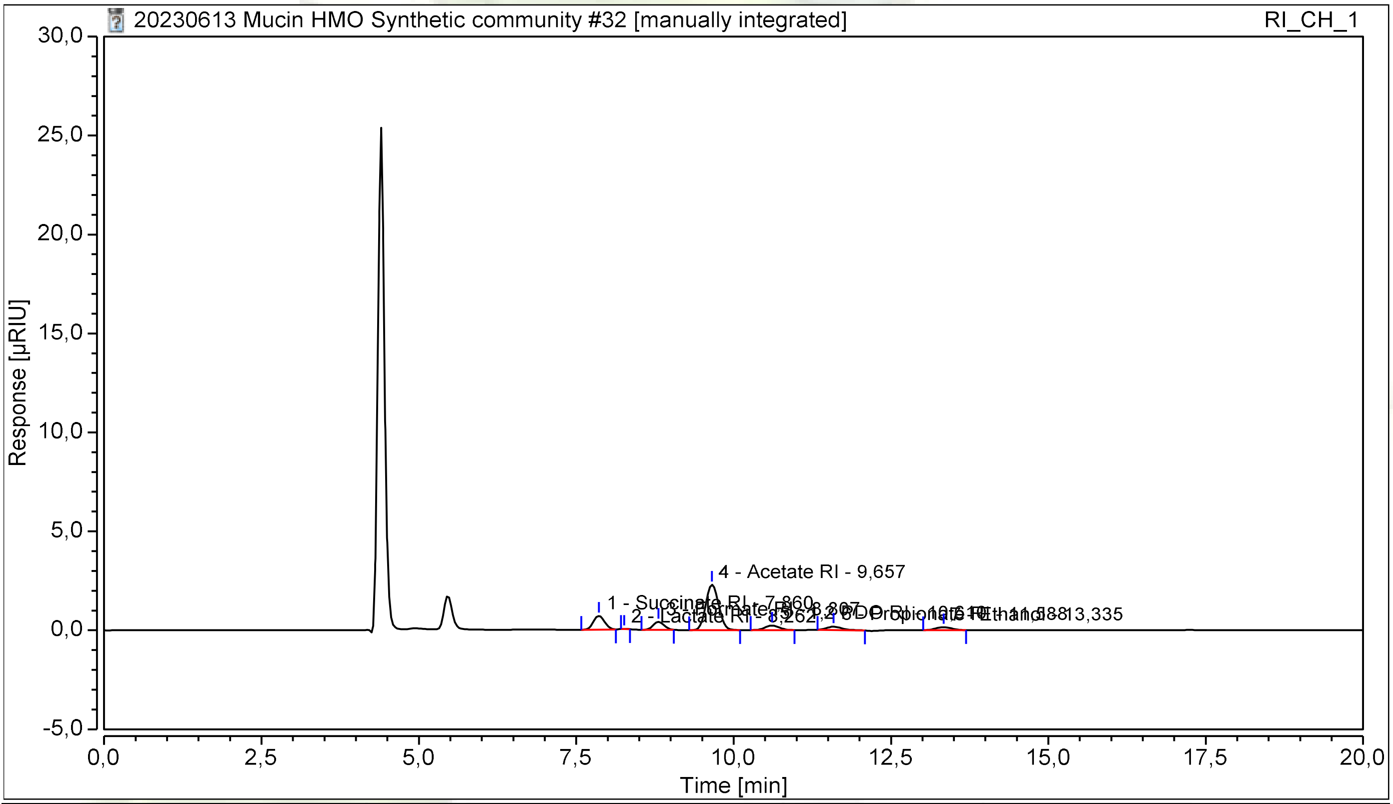

SST Results

No.	Name	Inj.Condition	Peak	Test Result	Injection
**Number of executed test cases:**	**n.a.**		**Total Result:**	**Passed**	

Chromatogram

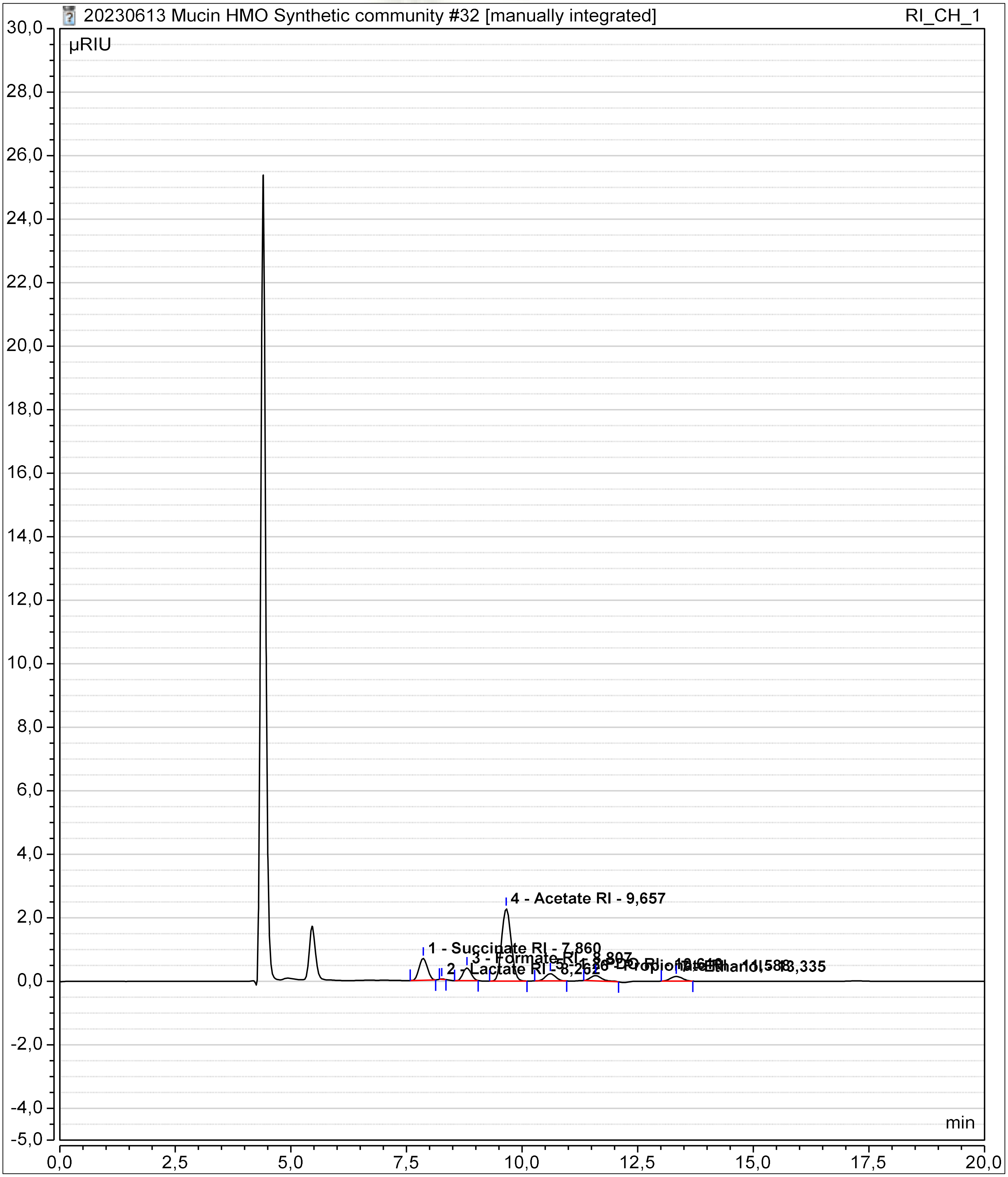

Chromatogram and Results

Injection Details

Injection Name:	**17 GOSFOSEXTR t24 r2**	*Run Time (min):*	**20,00**
Vial Number:	**3:C2**	*Injection Volume:*	**10,00**
Injection Type:	**Unknown**	*Channel:*	**RI_CH_1**
Calibration Level:		*Wavelength:*	**n.a.**
Instrument Method:	**Default method LC2030C 45 gr 20 min**	*Bandwidth:*	**n.a.**
Processing Method:	**Processing Method LC2030 45 gr**	*Dilution Factor:*	**1,0000**
Injection Date/Time:	**13-jun-23 22:50**	*Sample Weight:*	**1,0000**

Chromatogram

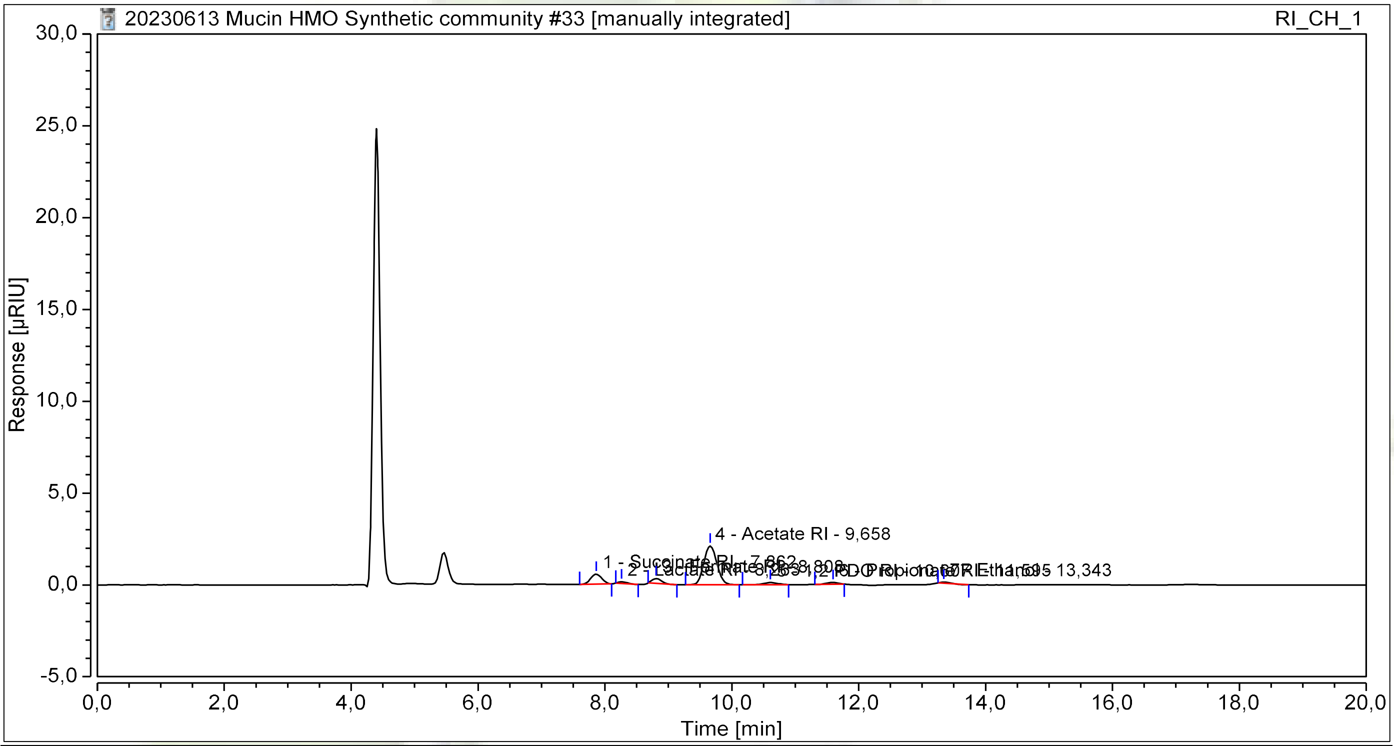

Integration Results

No.	Peak Name	Retention Time<br>min	Area<br>µRIU*min	Height<br>µRIU	Relative Area<br>%	Relative Height<br>%	Amount
n.a.	GlcNAc	n.a.	n.a.	n.a.	n.a.	n.a.	n.a.
n.a.	Citrate	n.a.	n.a.	n.a.	n.a.	n.a.	n.a.
n.a.	Glucose	n.a.	n.a.	n.a.	n.a.	n.a.	n.a.
n.a.	Galactose	n.a.	n.a.	n.a.	n.a.	n.a.	n.a.
n.a.	Fucose	n.a.	n.a.	n.a.	n.a.	n.a.	n.a.
1	Succinate RI	7,862	0,110	0,542	15,12	16,60	n.a.
2	Lactate RI	8,263	0,014	0,087	1,89	2,67	0,4007
n.a.	glycerol	n.a.	n.a.	n.a.	n.a.	n.a.	n.a.
3	Formate RI	8,808	0,045	0,263	6,20	8,05	4,7515
4	Acetate RI	9,658	0,504	2,112	68,97	64,70	31,0175
5	1,2 PDO RI	10,607	0,029	0,112	3,91	3,43	0,8490
n.a.	1,3-PDO	n.a.	n.a.	n.a.	n.a.	n.a.	n.a.
6	Propionate RI	11,595	0,021	0,096	2,87	2,94	0,8422
n.a.	1,3-PDO	n.a.	n.a.	n.a.	n.a.	n.a.	n.a.
n.a.	2-3 BDO	n.a.	n.a.	n.a.	n.a.	n.a.	n.a.
7	Ethanol	13,343	0,008	0,052	1,05	1,60	0,0708
n.a.	Isobutyrate RI	n.a.	n.a.	n.a.	n.a.	n.a.	n.a.
n.a.	Butyrate RI	n.a.	n.a.	n.a.	n.a.	n.a.	n.a.
**Total:**			**0,731**	**3,264**	**100,00**	**100,00**	

Peak Analysis

Injection Details

Injection Name:	**17 GOSFOSEXTR t24 r2**	*Run Time (min):*	**20,00**
Vial Number:	**3:C2**	*Injection Volume:*	**10,00**
Injection Type:	**Unknown**	*Channel:*	**RI_CH_1**
Calibration Level:		*Wavelength:*	**n.a.**
Instrument Method:	**Default method LC2030C 45 gr 20 min**	*Bandwidth:*	**n.a.**
Processing Method:	**Processing Method LC2030 45 gr**	*Dilution Factor:*	**1,0000**
Injection Date/Time:	**13-jun-23 22:50**	*Sample Weight:*	**1,0000**

Chromatogram

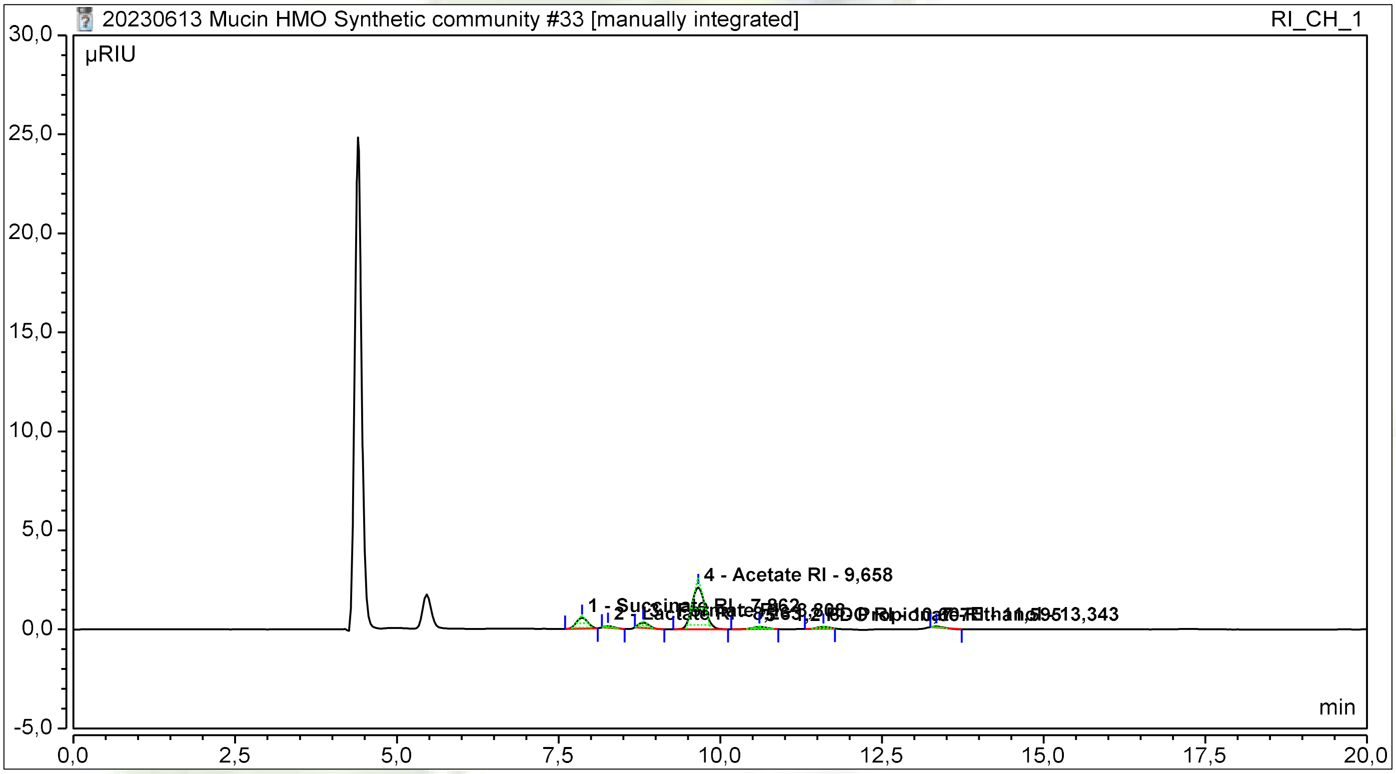

Peak Results

No.	Peak Name	Retention Time min	Width (50%) min	Type	Resolution (EP)	Asymmetry (EP)	Plates (EP)
n.a.	GlcNAc	n.a.	n.a.	n.a.	n.a.	n.a.	n.a.
n.a.	Citrate	n.a.	n.a.	n.a.	n.a.	n.a.	n.a.
n.a.	Glucose	n.a.	n.a.	n.a.	n.a.	n.a.	n.a.
n.a.	Galactose	n.a.	n.a.	n.a.	n.a.	n.a.	n.a.
n.a.	Fucose	n.a.	n.a.	n.a.	n.a.	n.a.	n.a.
1	Succinate RI	7,862	0,197	BMB	1,34	1,02	8853
2	Lactate RI	8,263	0,158	BMB*	1,94	1,49	15207
n.a.	glycerol	n.a.	n.a.	n.a.	n.a.	n.a.	n.a.
3	Formate RI	8,808	0,174	BMB*	2,51	1,22	14191
4	Acetate RI	9,658	0,225	BMB	2,38	1,08	10177
5	1,2 PDO RI	10,607	0,246	BMB*	2,51	1,05	10336
n.a.	1,3-PDO	n.a.	n.a.	n.a.	n.a.	n.a.	n.a.
6	Propionate RI	11,595	0,218	BMB*	5,12	0,93	15602
n.a.	1,3-PDO	n.a.	n.a.	n.a.	n.a.	n.a.	n.a.
n.a.	2-3 BDO	n.a.	n.a.	n.a.	n.a.	n.a.	n.a.
7	Ethanol	13,343	0,184	BMB*	n.a.	1,49	29090
n.a.	Isobutyrate RI	n.a.	n.a.	n.a.	n.a.	n.a.	n.a.
n.a.	Butyrate RI	n.a.	n.a.	n.a.	n.a.	n.a.	n.a.

Chromeleon (c) Dionex
Version 7.2.10.25868

Chromatogram and SST Results

Injection Details

Injection Name:	**17 GOSFOSEXTR t24 r2**	*Run Time (min):*	**20,00**
Vial Number:	**3:C2**	*Injection Volume:*	**10,00**
Injection Type:	**Unknown**	*Channel:*	**RI_CH_1**
Calibration Level:		*Wavelength:*	**n.a.**
Instrument Method:	**Default method LC2030C 45 gr 20 min**	*Bandwidth:*	**n.a.**
Processing Method:	**Processing Method LC2030 45 gr**	*Dilution Factor:*	**1,0000**
Injection Date/Time:	**13-jun-23 22:50**	*Sample Weight:*	**1,0000**

Chromatogram

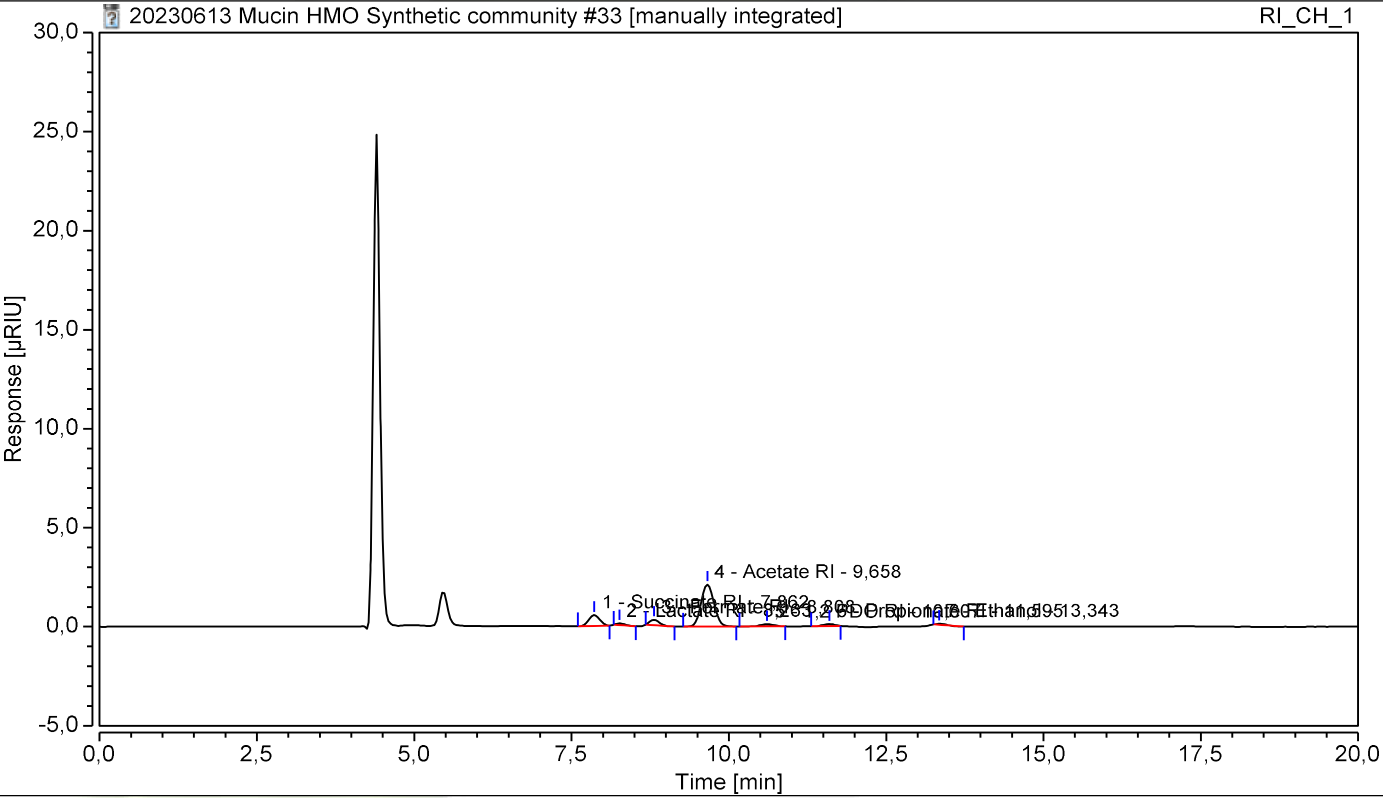

SST Results

No.	Name	Inj.Condition	Peak	Test Result	Injection
**Number of executed test cases:**	**n.a.**		**Total Result:**	**Passed**	

Chromeleon (c) Dionex
Version 7.2.10.25868

Chromatogram

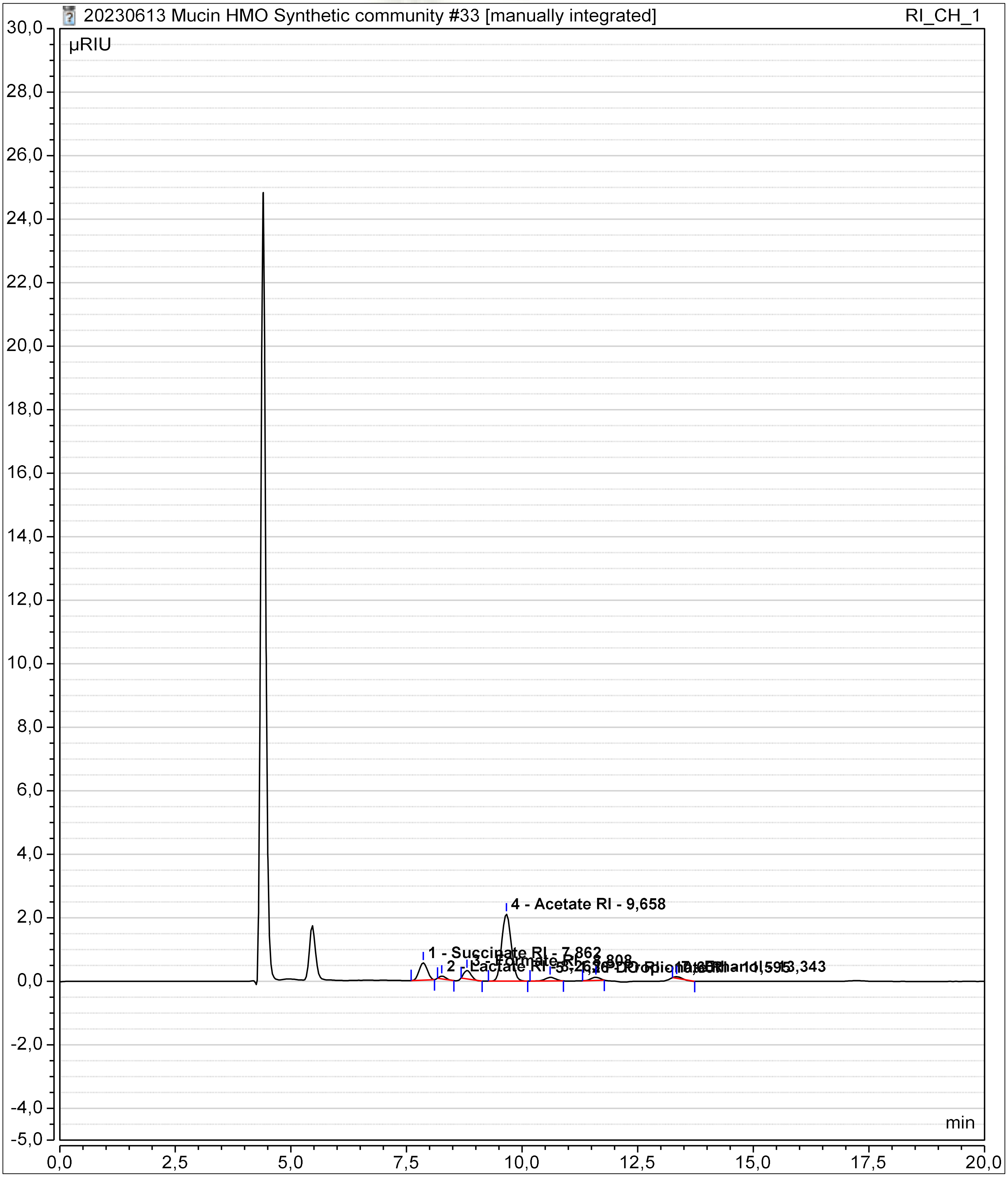

Chromeleon (c) Dionex
Version 7.2.10.25868

Chromatogram and Results

Injection Details

Injection Name:	**18 GOSFOSEXTR t24 r3**	*Run Time (min):*	**20,00**
Vial Number:	**3:C3**	*Injection Volume:*	**10,00**
Injection Type:	**Unknown**	*Channel:*	**RI_CH_1**
Calibration Level:		*Wavelength:*	**n.a.**
Instrument Method:	**Default method LC2030C 45 gr 20 min**	*Bandwidth:*	**n.a.**
Processing Method:	**Processing Method LC2030 45 gr**	*Dilution Factor:*	**1,0000**
Injection Date/Time:	**13-jun-23 23:11**	*Sample Weight:*	**1,0000**

Chromatogram

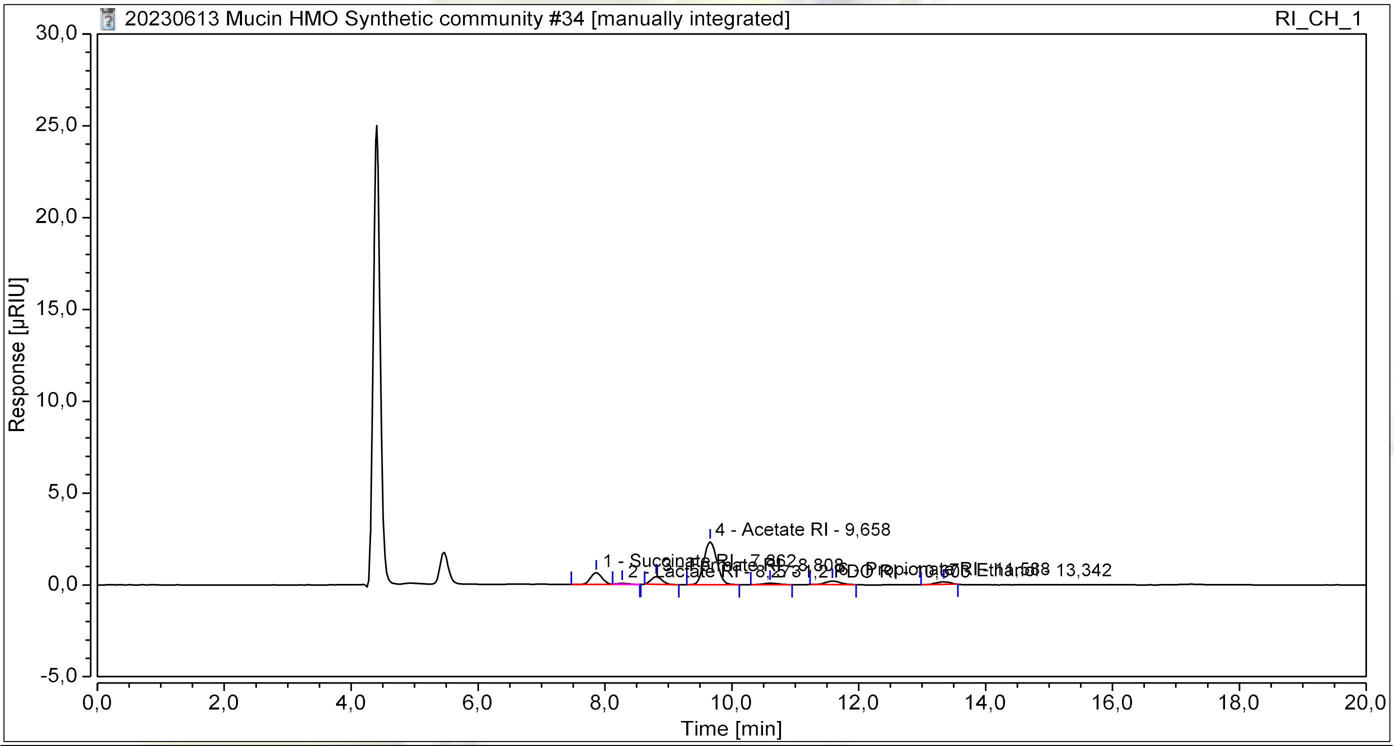

Integration Results

No.	Peak Name	Retention Time min	Area µRIU*min	Height µRIU	Relative Area %	Relative Height %	Amount
n.a.	GlcNAc	n.a.	n.a.	n.a.	n.a.	n.a.	n.a.
n.a.	Citrate	n.a.	n.a.	n.a.	n.a.	n.a.	n.a.
n.a.	Glucose	n.a.	n.a.	n.a.	n.a.	n.a.	n.a.
n.a.	Galactose	n.a.	n.a.	n.a.	n.a.	n.a.	n.a.
n.a.	Fucose	n.a.	n.a.	n.a.	n.a.	n.a.	n.a.
1	Succinate RI	7,862	0,142	0,636	15,78	16,59	n.a.
2	Lactate RI	8,273	0,009	0,050	1,03	1,30	0,2697
n.a.	glycerol	n.a.	n.a.	n.a.	n.a.	n.a.	n.a.
3	Formate RI	8,808	0,084	0,412	9,31	10,75	8,7738
4	Acetate RI	9,658	0,555	2,332	61,84	60,86	34,1868
5	1,2 PDO RI	10,603	0,020	0,079	2,28	2,05	0,6089
n.a.	1,3-PDO	n.a.	n.a.	n.a.	n.a.	n.a.	n.a.
6	Propionate RI	11,588	0,051	0,189	5,64	4,92	2,0366
n.a.	1,3-PDO	n.a.	n.a.	n.a.	n.a.	n.a.	n.a.
n.a.	2-3 BDO	n.a.	n.a.	n.a.	n.a.	n.a.	n.a.
7	Ethanol	13,342	0,037	0,135	4,13	3,52	0,3413
n.a.	Isobutyrate RI	n.a.	n.a.	n.a.	n.a.	n.a.	n.a.
n.a.	Butyrate RI	n.a.	n.a.	n.a.	n.a.	n.a.	n.a.
**Total:**			**0,898**	**3,832**	**100,00**	**100,00**	

Peak Analysis

Injection Details

Injection Name:	**18 GOSFOSEXTR t24 r3**	*Run Time (min):*	**20,00**
Vial Number:	**3:C3**	*Injection Volume:*	**10,00**
Injection Type:	**Unknown**	*Channel:*	**RI_CH_1**
Calibration Level:		*Wavelength:*	**n.a.**
Instrument Method:	**Default method LC2030C 45 gr 20 min**	*Bandwidth:*	**n.a.**
Processing Method:	**Processing Method LC2030 45 gr**	*Dilution Factor:*	**1,0000**
Injection Date/Time:	**13-jun-23 23:11**	*Sample Weight:*	**1,0000**

Chromatogram

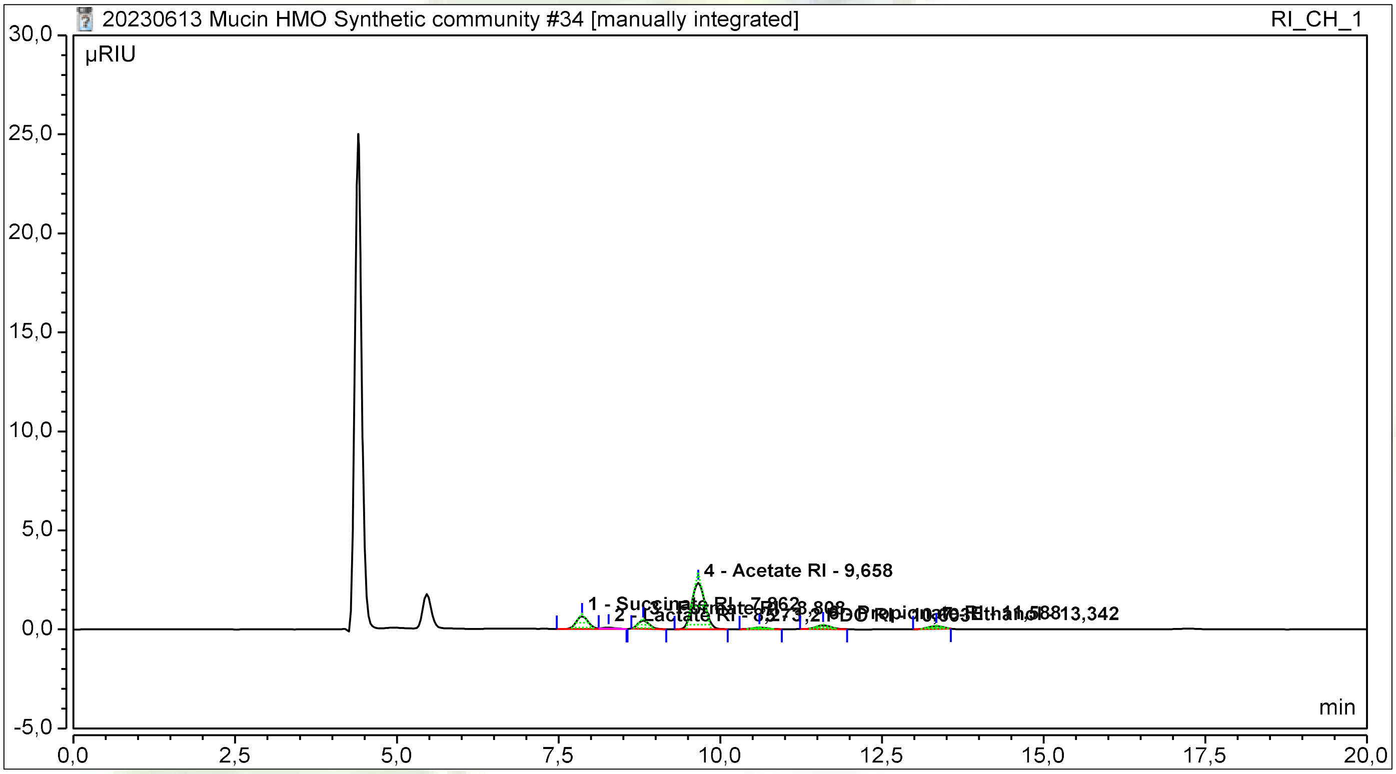

Peak Results

No.	Peak Name	Retention Time min	Width (50%) min	Type	Resolution (EP)	Asymmetry (EP)	Plates (EP)
n.a.	GlcNAc	n.a.	n.a.	n.a.	n.a.	n.a.	n.a.
n.a.	Citrate	n.a.	n.a.	n.a.	n.a.	n.a.	n.a.
n.a.	Glucose	n.a.	n.a.	n.a.	n.a.	n.a.	n.a.
n.a.	Galactose	n.a.	n.a.	n.a.	n.a.	n.a.	n.a.
n.a.	Fucose	n.a.	n.a.	n.a.	n.a.	n.a.	n.a.
1	Succinate RI	7,862	0,202	BMB	2,81	1,10	8408
2	Lactate RI	8,273	n.a.	Rd	n.a.	n.a.	n.a.
n.a.	glycerol	n.a.	n.a.	n.a.	n.a.	n.a.	n.a.
3	Formate RI	8,808	0,195	BMB*	2,38	1,13	11246
4	Acetate RI	9,658	0,225	BMB	2,35	1,08	10201
5	1,2 PDO RI	10,603	0,248	BMB*	2,31	1,10	10091
n.a.	1,3-PDO	n.a.	n.a.	n.a.	n.a.	n.a.	n.a.
6	Propionate RI	11,588	0,256	BMB*	3,90	1,04	11394
n.a.	1,3-PDO	n.a.	n.a.	n.a.	n.a.	n.a.	n.a.
n.a.	2-3 BDO	n.a.	n.a.	n.a.	n.a.	n.a.	n.a.
7	Ethanol	13,342	0,275	BMB*	n.a.	0,90	13082
n.a.	Isobutyrate RI	n.a.	n.a.	n.a.	n.a.	n.a.	n.a.
n.a.	Butyrate RI	n.a.	n.a.	n.a.	n.a.	n.a.	n.a.

Chromeleon (c) Dionex
Version 7.2.10.25868

Chromatogram and SST Results

Injection Details

Injection Name:	**18 GOSFOSEXTR t24 r3**	*Run Time (min):*	**20,00**
Vial Number:	**3:C3**	*Injection Volume:*	**10,00**
Injection Type:	**Unknown**	*Channel:*	**RI_CH_1**
Calibration Level:		*Wavelength:*	**n.a.**
Instrument Method:	**Default method LC2030C 45 gr 20 min**	*Bandwidth:*	**n.a.**
Processing Method:	**Processing Method LC2030 45 gr**	*Dilution Factor:*	**1,0000**
Injection Date/Time:	**13-jun-23 23:11**	*Sample Weight:*	**1,0000**

Chromatogram

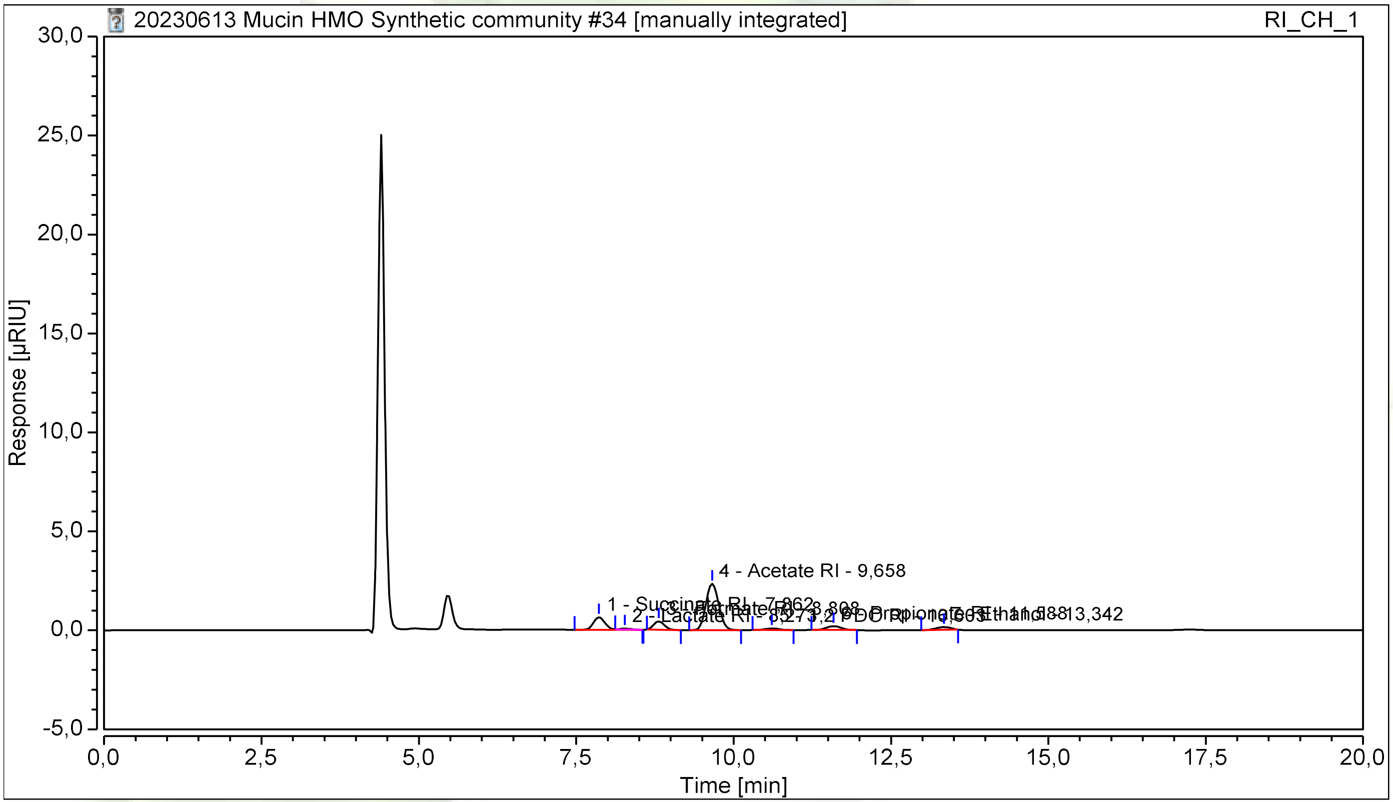

SST Results

No.	Name	Inj.Condition	Peak	Test Result	Injection
**Number of executed test cases:**	**n.a.**		**Total Result:**	**Passed**	

Chromatogram

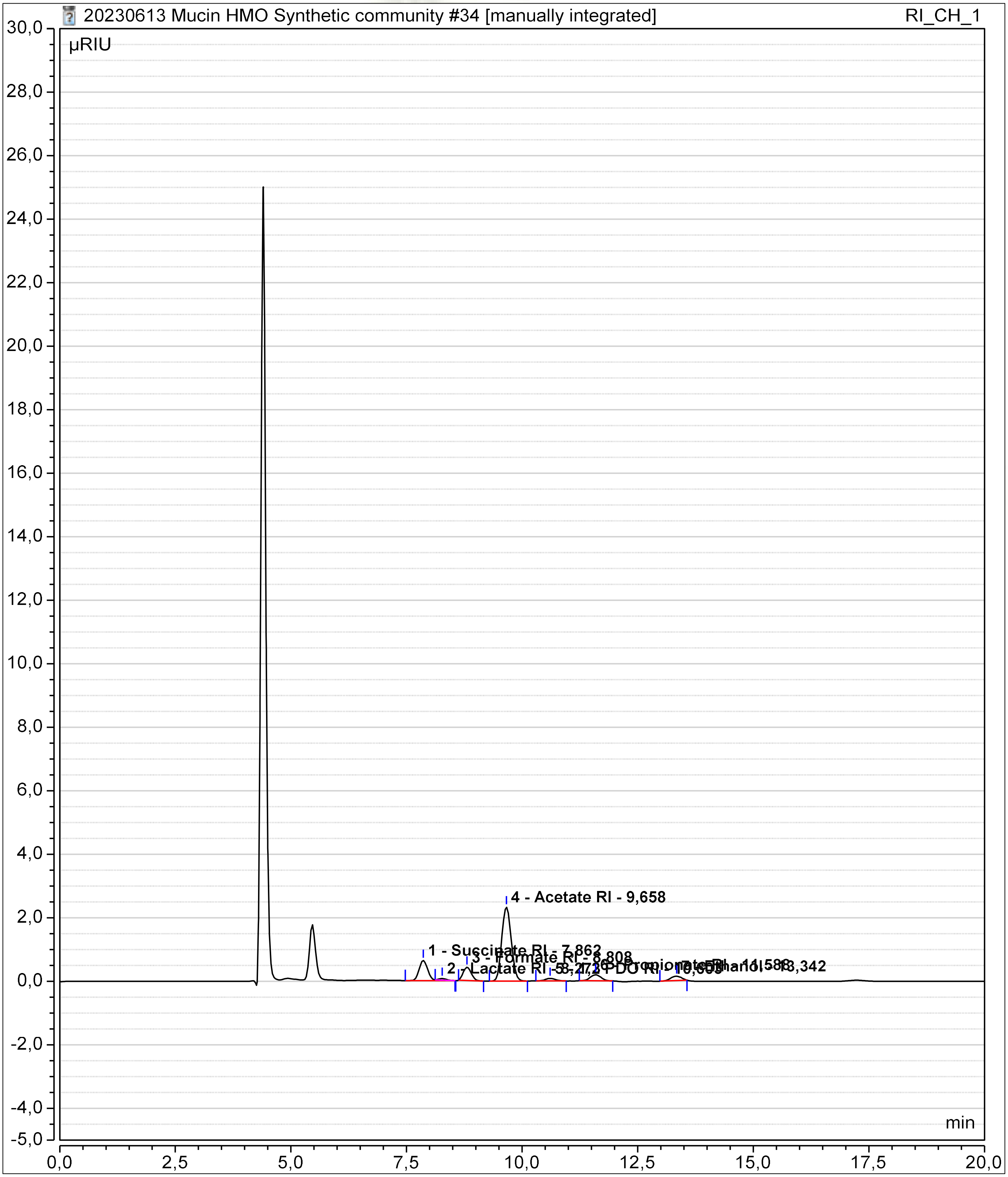

Chromatogram and Results

Injection Details

Injection Name:	**25 GOSFOS t48 r1**	*Run Time (min):*	**20,00**
Vial Number:	**3:C4**	*Injection Volume:*	**10,00**
Injection Type:	**Unknown**	*Channel:*	**RI_CH_1**
Calibration Level:		*Wavelength:*	**n.a.**
Instrument Method:	**Default method LC2030C 45 gr 20 min**	*Bandwidth:*	**n.a.**
Processing Method:	**Processing Method LC2030 45 gr**	*Dilution Factor:*	**1,0000**
Injection Date/Time:	**13-jun-23 23:31**	*Sample Weight:*	**1,0000**

Chromatogram

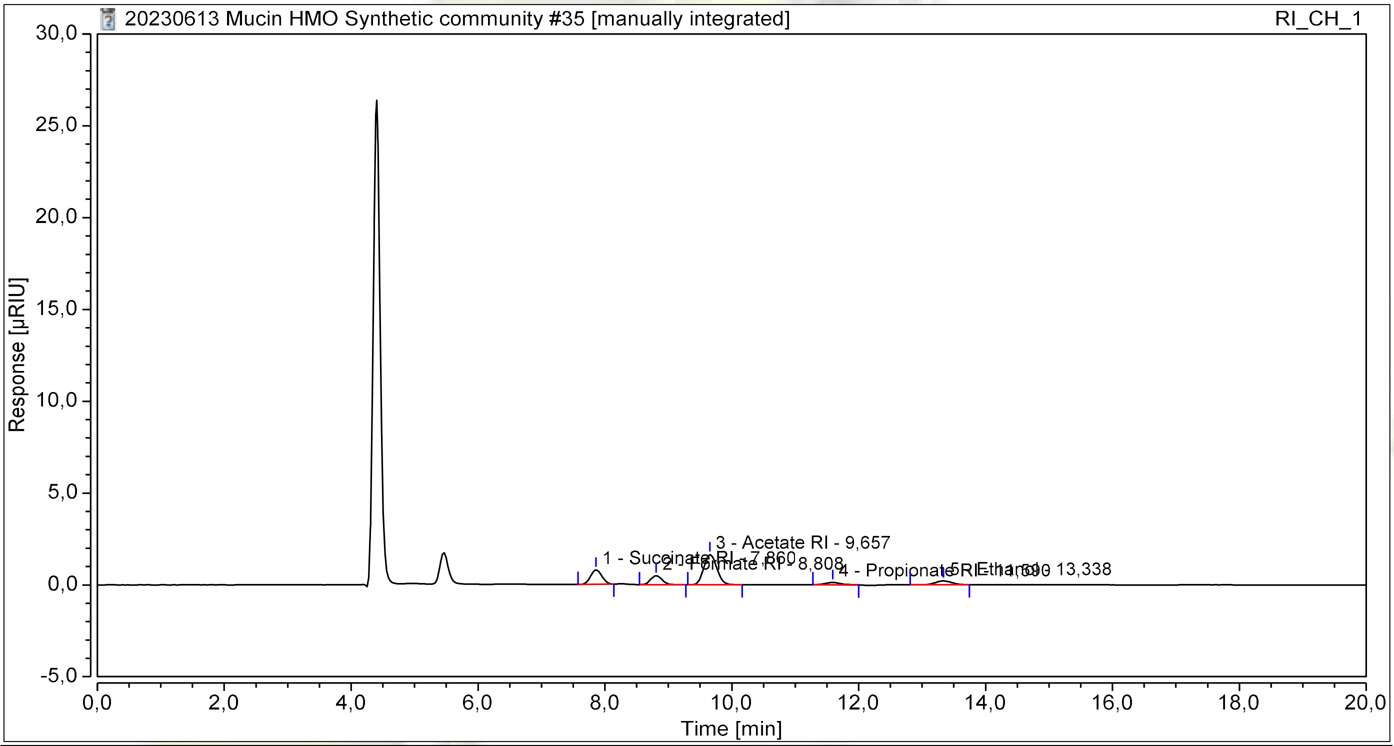

Integration Results

No.	Peak Name	Retention Time min	Area µRIU*min	Height µRIU	Relative Area %	Relative Height %	Amount
n.a.	GlcNAc	n.a.	n.a.	n.a.	n.a.	n.a.	n.a.
n.a.	Citrate	n.a.	n.a.	n.a.	n.a.	n.a.	n.a.
n.a.	Glucose	n.a.	n.a.	n.a.	n.a.	n.a.	n.a.
n.a.	Galactose	n.a.	n.a.	n.a.	n.a.	n.a.	n.a.
n.a.	Fucose	n.a.	n.a.	n.a.	n.a.	n.a.	n.a.
1	Succinate RI	7,860	0,164	0,782	21,72	24,15	n.a.
n.a.	Lactate RI	n.a.	n.a.	n.a.	n.a.	n.a.	n.a.
n.a.	glycerol	n.a.	n.a.	n.a.	n.a.	n.a.	n.a.
2	Formate RI	8,808	0,102	0,483	13,57	14,91	10,7339
3	Acetate RI	9,657	0,389	1,640	51,62	50,66	23,9475
n.a.	1,2 PDO RI	n.a.	n.a.	n.a.	n.a.	n.a.	n.a.
n.a.	1,3-PDO	n.a.	n.a.	n.a.	n.a.	n.a.	n.a.
4	Propionate RI	11,590	0,033	0,123	4,41	3,79	1,3379
n.a.	1,3-PDO	n.a.	n.a.	n.a.	n.a.	n.a.	n.a.
n.a.	2-3 BDO	n.a.	n.a.	n.a.	n.a.	n.a.	n.a.
5	Ethanol	13,338	0,065	0,210	8,67	6,49	0,6021
n.a.	Isobutyrate RI	n.a.	n.a.	n.a.	n.a.	n.a.	n.a.
n.a.	Butyrate RI	n.a.	n.a.	n.a.	n.a.	n.a.	n.a.
**Total:**			**0,754**	**3,238**	**100,00**	**100,00**	

Chromeleon (c) Dionex
Version 7.2.10.25868

Peak Analysis

Injection Details

Injection Name:	**25 GOSFOS t48 r1**	*Run Time (min):*	**20,00**
Vial Number:	**3:C4**	*Injection Volume:*	**10,00**
Injection Type:	**Unknown**	*Channel:*	**RI_CH_1**
Calibration Level:		*Wavelength:*	**n.a.**
Instrument Method:	**Default method LC2030C 45 gr 20 min**	*Bandwidth:*	**n.a.**
Processing Method:	**Processing Method LC2030 45 gr**	*Dilution Factor:*	**1,0000**
Injection Date/Time:	**13-jun-23 23:31**	*Sample Weight:*	**1,0000**

Chromatogram

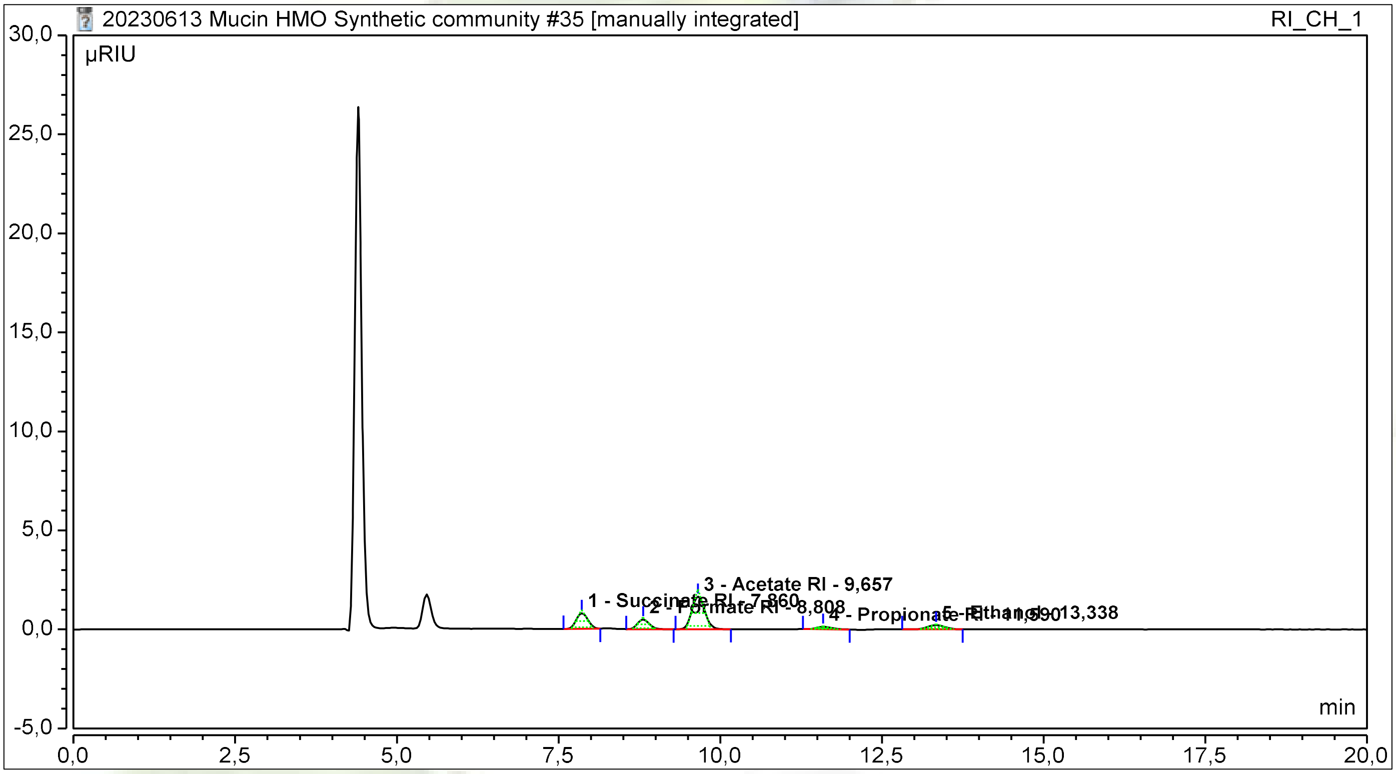

Peak Results

No.	Peak Name	Retention Time min	Width (50%) min	Type	Resolution (EP)	Asymmetry (EP)	Plates (EP)
n.a.	GlcNAc	n.a.	n.a.	n.a.	n.a.	n.a.	n.a.
n.a.	Citrate	n.a.	n.a.	n.a.	n.a.	n.a.	n.a.
n.a.	Glucose	n.a.	n.a.	n.a.	n.a.	n.a.	n.a.
n.a.	Galactose	n.a.	n.a.	n.a.	n.a.	n.a.	n.a.
n.a.	Fucose	n.a.	n.a.	n.a.	n.a.	n.a.	n.a.
1	Succinate RI	7,860	0,199	BMB	2,80	1,06	8601
n.a.	Lactate RI	n.a.	n.a.	n.a.	n.a.	n.a.	n.a.
n.a.	glycerol	n.a.	n.a.	n.a.	n.a.	n.a.	n.a.
2	Formate RI	8,808	0,200	BMB	2,36	1,10	10776
3	Acetate RI	9,657	0,224	BMB	4,78	1,09	10268
n.a.	1,2 PDO RI	n.a.	n.a.	n.a.	n.a.	n.a.	n.a.
n.a.	1,3-PDO	n.a.	n.a.	n.a.	n.a.	n.a.	n.a.
4	Propionate RI	11,590	0,253	BMB*	3,75	1,33	11591
n.a.	1,3-PDO	n.a.	n.a.	n.a.	n.a.	n.a.	n.a.
n.a.	2-3 BDO	n.a.	n.a.	n.a.	n.a.	n.a.	n.a.
5	Ethanol	13,338	0,297	BMB*	n.a.	1,05	11158
n.a.	Isobutyrate RI	n.a.	n.a.	n.a.	n.a.	n.a.	n.a.
n.a.	Butyrate RI	n.a.	n.a.	n.a.	n.a.	n.a.	n.a.

Chromatogram and SST Results

Injection Details

Injection Name:	**25 GOSFOS t48 r1**	*Run Time (min):*	**20,00**
Vial Number:	**3:C4**	*Injection Volume:*	**10,00**
Injection Type:	**Unknown**	*Channel:*	**RI_CH_1**
Calibration Level:		*Wavelength:*	**n.a.**
Instrument Method:	**Default method LC2030C 45 gr 20 min**	*Bandwidth:*	**n.a.**
Processing Method:	**Processing Method LC2030 45 gr**	*Dilution Factor:*	**1,0000**
Injection Date/Time:	**13-jun-23 23:31**	*Sample Weight:*	**1,0000**

Chromatogram

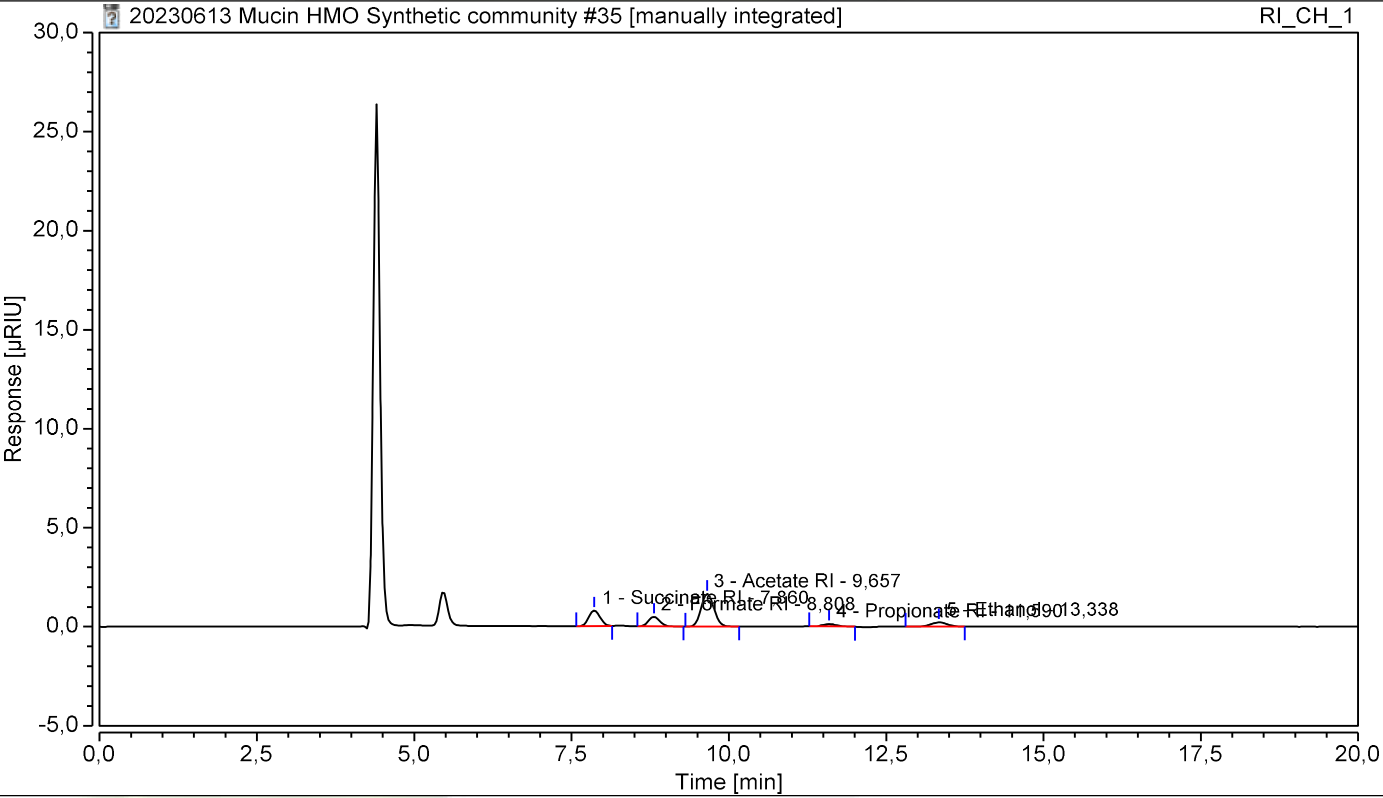

SST Results

No.	Name	Inj.Condition	Peak	Test Result	Injection

Number of executed test cases: **n.a.** **Total Result:** **Passed**

Chromatogram

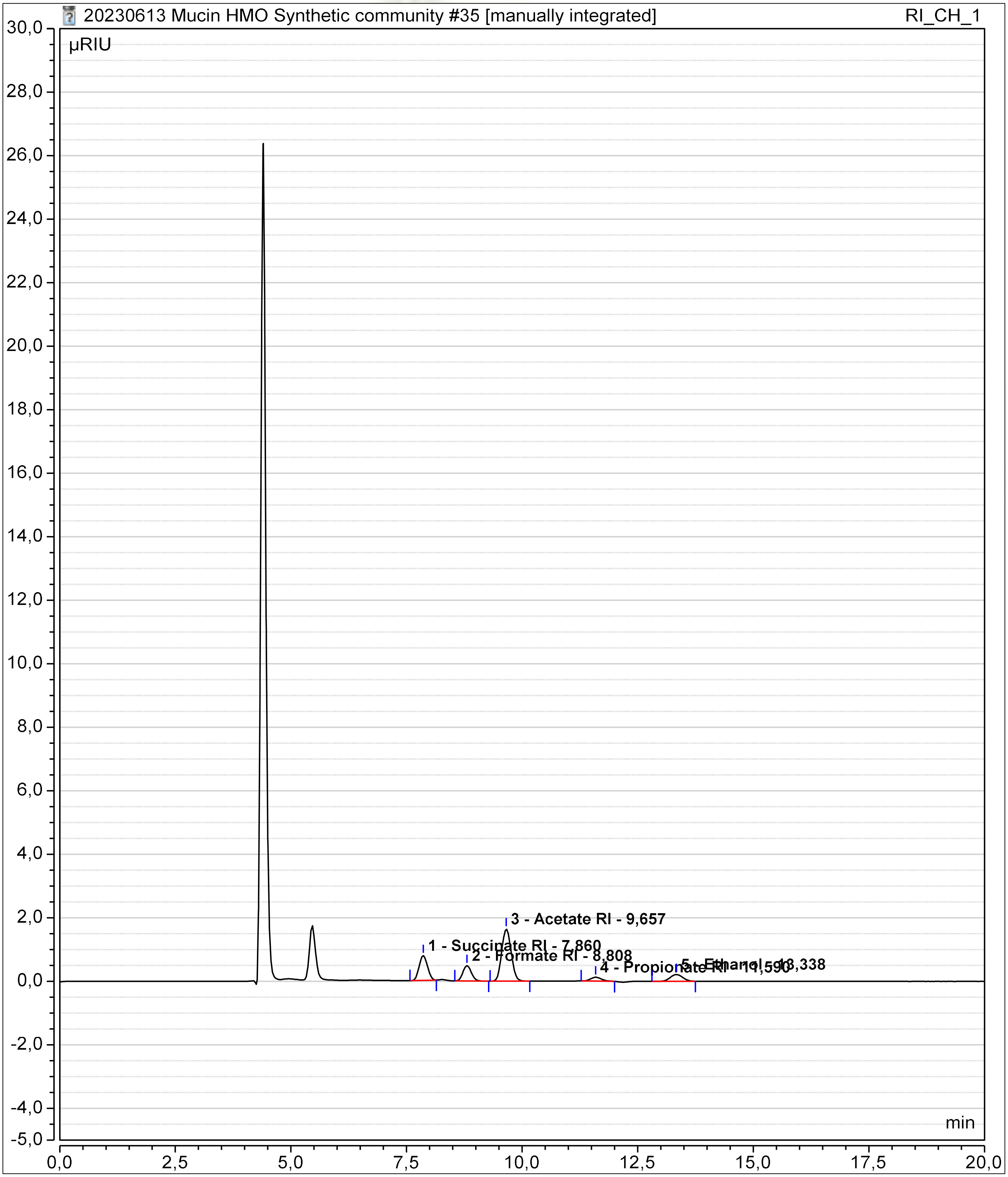

Chromatogram and Results

Injection Details

Injection Name:	**26 GOSFOS t48 r2**	*Run Time (min):*	**20,00**
Vial Number:	**3:C5**	*Injection Volume:*	**10,00**
Injection Type:	**Unknown**	*Channel:*	**RI_CH_1**
Calibration Level:		*Wavelength:*	**n.a.**
Instrument Method:	**Default method LC2030C 45 gr 20 min**	*Bandwidth:*	**n.a.**
Processing Method:	**Processing Method LC2030 45 gr**	*Dilution Factor:*	**1,0000**
Injection Date/Time:	**13-jun-23 23:51**	*Sample Weight:*	**1,0000**

Chromatogram

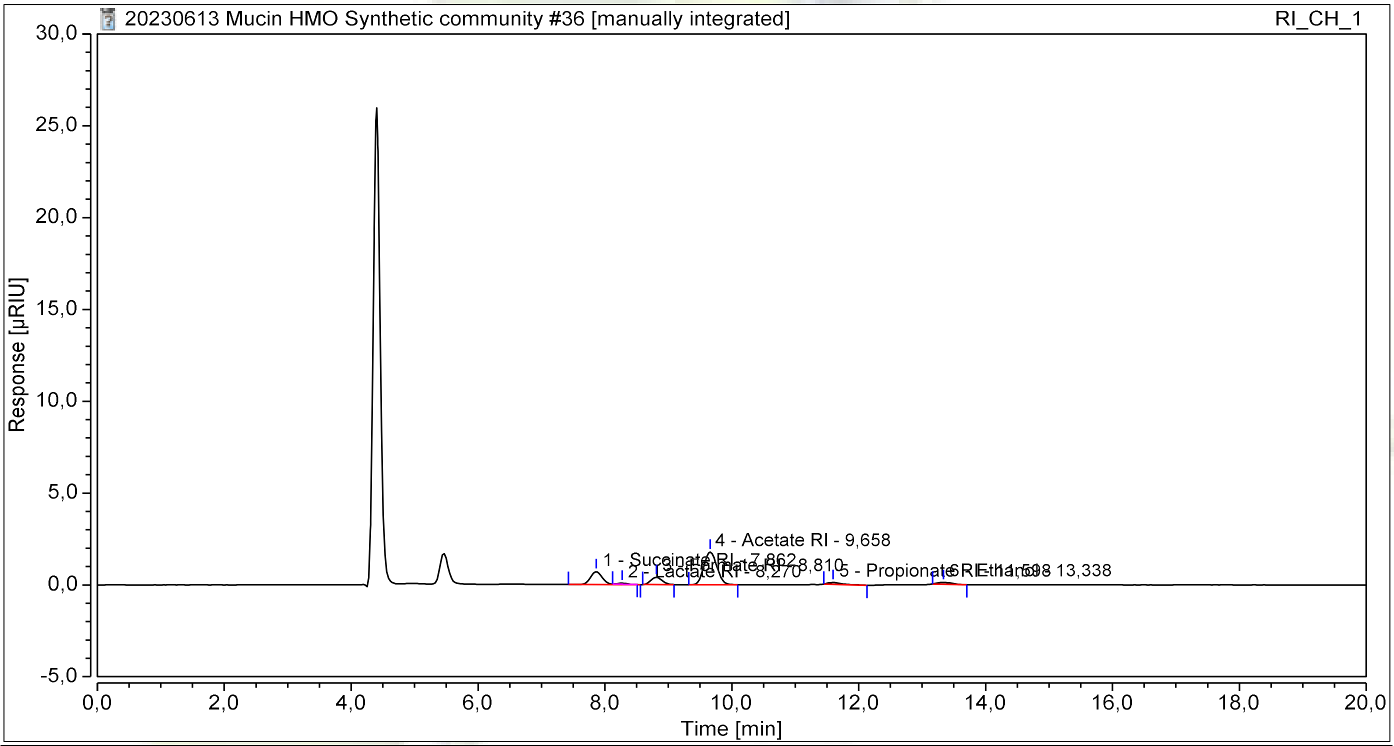

Integration Results

No.	Peak Name	Retention Time min	Area µRIU*min	Height µRIU	Relative Area %	Relative Height %	Amount
n.a.	GlcNAc	n.a.	n.a.	n.a.	n.a.	n.a.	n.a.
n.a.	Citrate	n.a.	n.a.	n.a.	n.a.	n.a.	n.a.
n.a.	Glucose	n.a.	n.a.	n.a.	n.a.	n.a.	n.a.
n.a.	Galactose	n.a.	n.a.	n.a.	n.a.	n.a.	n.a.
n.a.	Fucose	n.a.	n.a.	n.a.	n.a.	n.a.	n.a.
1	Succinate RI	7,862	0,156	0,699	21,78	22,42	n.a.
2	Lactate RI	8,270	0,010	0,055	1,36	1,78	0,2828
n.a.	glycerol	n.a.	n.a.	n.a.	n.a.	n.a.	n.a.
3	Formate RI	8,810	0,081	0,393	11,33	12,60	8,5436
4	Acetate RI	9,658	0,423	1,781	58,86	57,08	26,0153
n.a.	1,2 PDO RI	n.a.	n.a.	n.a.	n.a.	n.a.	n.a.
n.a.	1,3-PDO	n.a.	n.a.	n.a.	n.a.	n.a.	n.a.
5	Propionate RI	11,598	0,023	0,094	3,21	3,00	0,9269
n.a.	1,3-PDO	n.a.	n.a.	n.a.	n.a.	n.a.	n.a.
n.a.	2-3 BDO	n.a.	n.a.	n.a.	n.a.	n.a.	n.a.
6	Ethanol	13,338	0,025	0,098	3,46	3,13	0,2292
n.a.	Isobutyrate RI	n.a.	n.a.	n.a.	n.a.	n.a.	n.a.
n.a.	Butyrate RI	n.a.	n.a.	n.a.	n.a.	n.a.	n.a.
**Total:**			**0,718**	**3,120**	**100,00**	**100,00**	

Chromeleon (c) Dionex
Version 7.2.10.25868

Peak Analysis

Injection Details

Injection Name:	**26 GOSFOS t48 r2**	*Run Time (min):*	**20,00**
Vial Number:	**3:C5**	*Injection Volume:*	**10,00**
Injection Type:	**Unknown**	*Channel:*	**RI_CH_1**
Calibration Level:		*Wavelength:*	**n.a.**
Instrument Method:	**Default method LC2030C 45 gr 20 min**	*Bandwidth:*	**n.a.**
Processing Method:	**Processing Method LC2030 45 gr**	*Dilution Factor:*	**1,0000**
Injection Date/Time:	**13-jun-23 23:51**	*Sample Weight:*	**1,0000**

Chromatogram

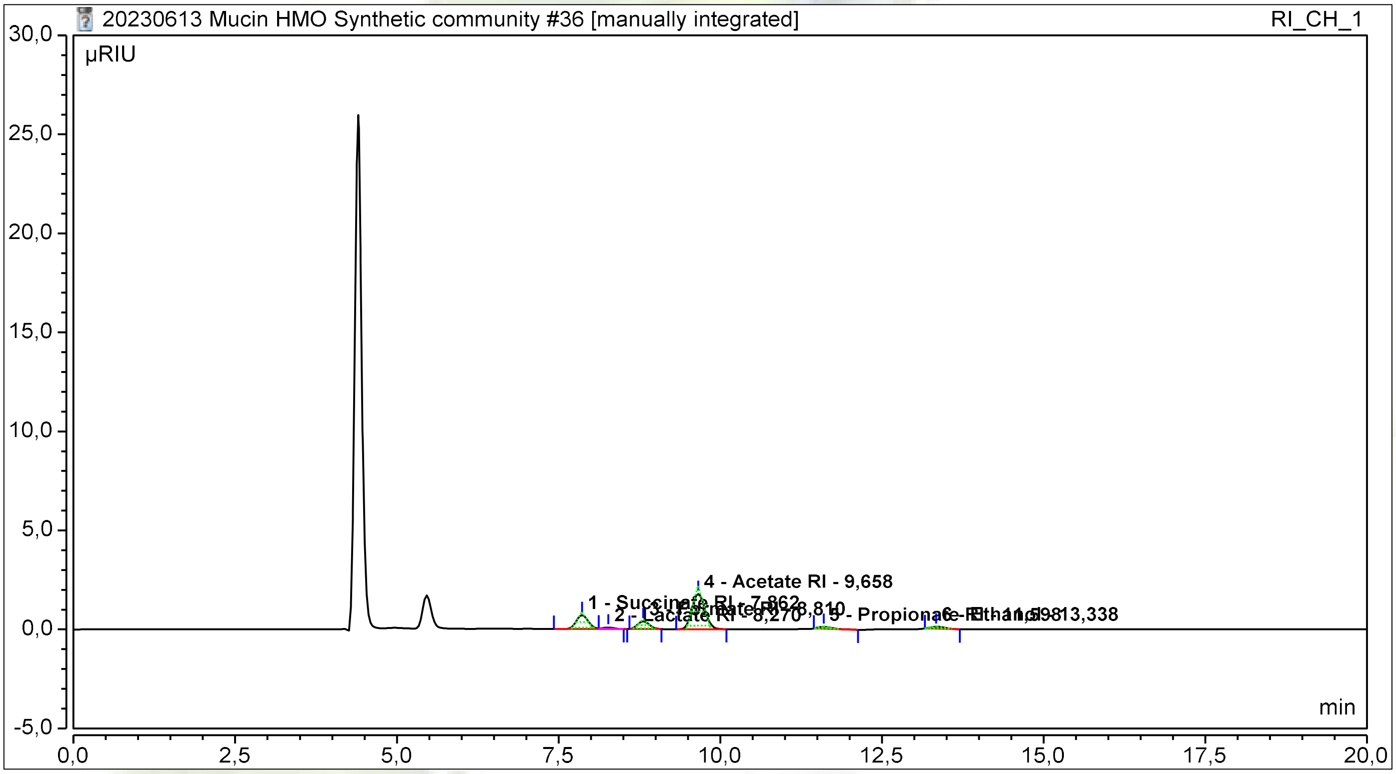

Peak Results

No.	Peak Name	Retention Time min	Width (50%) min	Type	Resolution (EP)	Asymmetry (EP)	Plates (EP)
n.a.	GlcNAc	n.a.	n.a.	n.a.	n.a.	n.a.	n.a.
n.a.	Citrate	n.a.	n.a.	n.a.	n.a.	n.a.	n.a.
n.a.	Glucose	n.a.	n.a.	n.a.	n.a.	n.a.	n.a.
n.a.	Galactose	n.a.	n.a.	n.a.	n.a.	n.a.	n.a.
n.a.	Fucose	n.a.	n.a.	n.a.	n.a.	n.a.	n.a.
1	Succinate RI	7,862	0,202	BMB	2,80	1,12	8401
2	Lactate RI	8,270	n.a.	Rd	n.a.	n.a.	n.a.
n.a.	glycerol	n.a.	n.a.	n.a.	n.a.	n.a.	n.a.
3	Formate RI	8,810	0,198	BMB*	2,37	1,09	10983
4	Acetate RI	9,658	0,225	BMB	5,14	1,09	10242
n.a.	1,2 PDO RI	n.a.	n.a.	n.a.	n.a.	n.a.	n.a.
n.a.	1,3-PDO	n.a.	n.a.	n.a.	n.a.	n.a.	n.a.
5	Propionate RI	11,598	0,221	BMB*	4,32	2,08	15245
n.a.	1,3-PDO	n.a.	n.a.	n.a.	n.a.	n.a.	n.a.
n.a.	2-3 BDO	n.a.	n.a.	n.a.	n.a.	n.a.	n.a.
6	Ethanol	13,338	0,254	BMB*	n.a.	1,31	15222
n.a.	Isobutyrate RI	n.a.	n.a.	n.a.	n.a.	n.a.	n.a.
n.a.	Butyrate RI	n.a.	n.a.	n.a.	n.a.	n.a.	n.a.

Chromeleon (c) Dionex
Version 7.2.10.25868

Chromatogram and SST Results

Injection Details

Injection Name:	**26 GOSFOS t48 r2**	*Run Time (min):*	**20,00**
Vial Number:	**3:C5**	*Injection Volume:*	**10,00**
Injection Type:	**Unknown**	*Channel:*	**RI_CH_1**
Calibration Level:		*Wavelength:*	**n.a.**
Instrument Method:	**Default method LC2030C 45 gr 20 min**	*Bandwidth:*	**n.a.**
Processing Method:	**Processing Method LC2030 45 gr**	*Dilution Factor:*	**1,0000**
Injection Date/Time:	**13-jun-23 23:51**	*Sample Weight:*	**1,0000**

Chromatogram

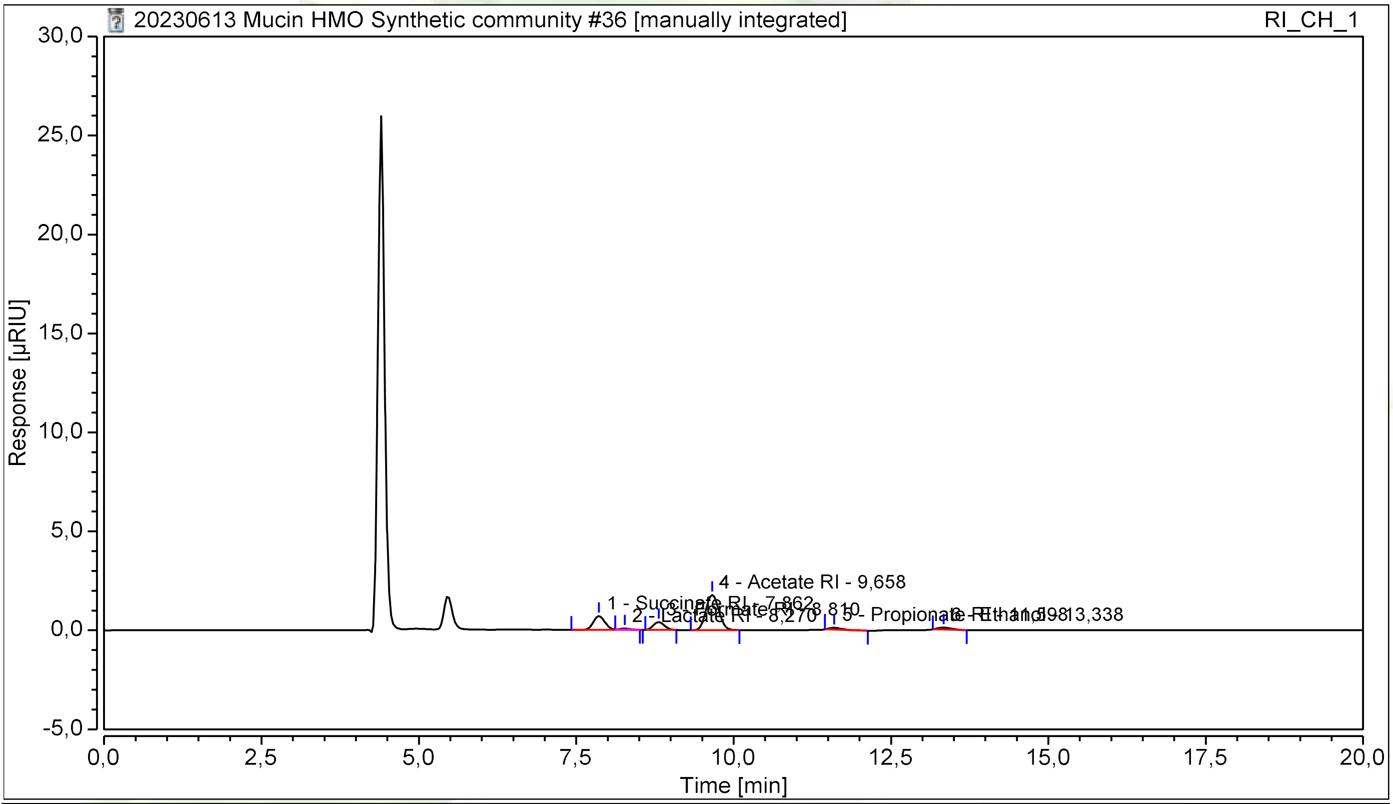

SST Results

No.	Name	Inj.Condition	Peak	Test Result	Injection
**Number of executed test cases:**	**n.a.**		**Total Result:**	**Passed**	

Chromatogram

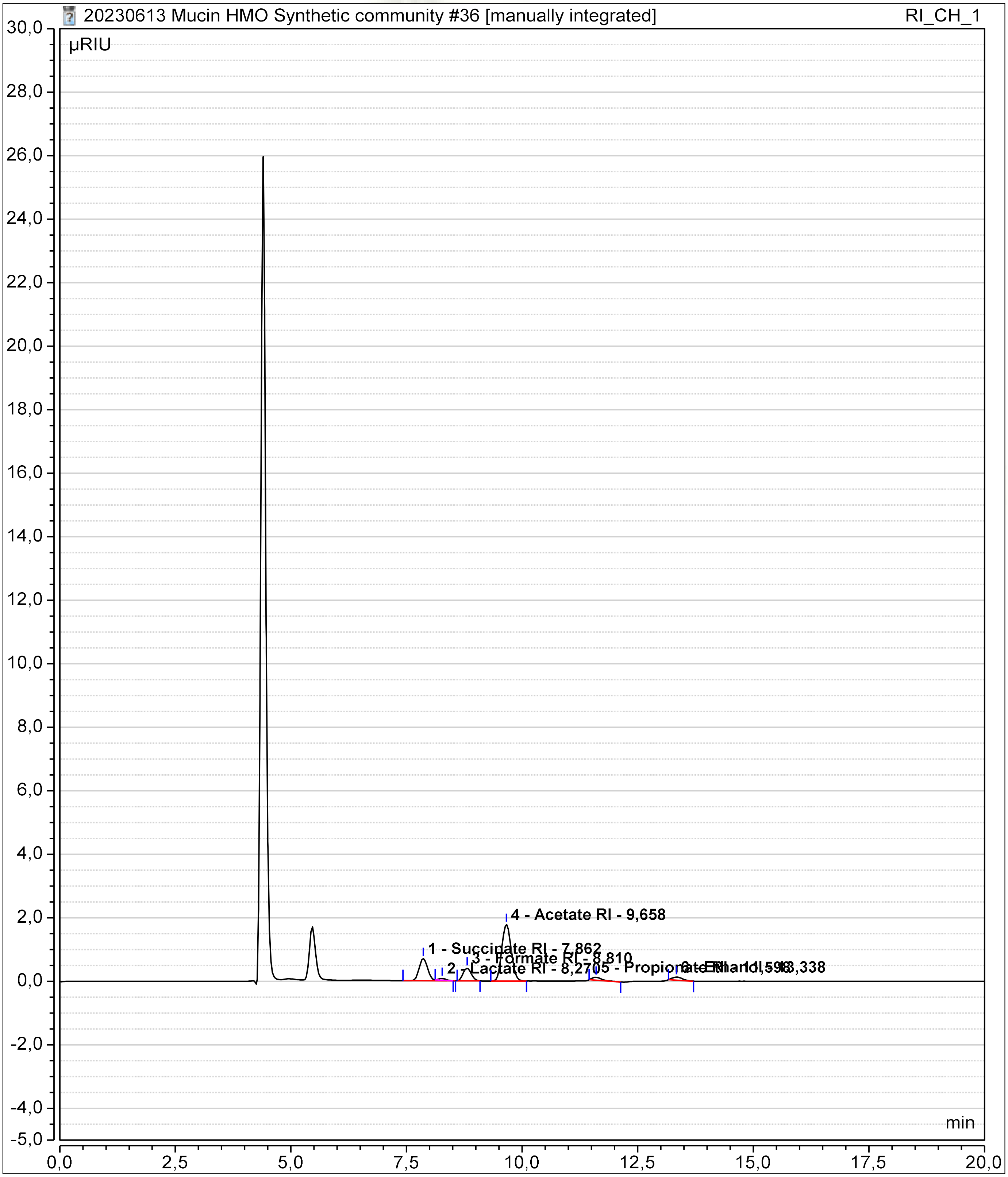

Chromeleon (c) Dionex
Version 7.2.10.25868

Chromatogram and Results

Injection Details

Injection Name:	**27 GOSFOS t48 r3**	*Run Time (min):*	**20,00**
Vial Number:	**3:C6**	*Injection Volume:*	**10,00**
Injection Type:	**Unknown**	*Channel:*	**RI_CH_1**
Calibration Level:		*Wavelength:*	**n.a.**
Instrument Method:	**Default method LC2030C 45 gr 20 min**	*Bandwidth:*	**n.a.**
Processing Method:	**Processing Method LC2030 45 gr**	*Dilution Factor:*	**1,0000**
Injection Date/Time:	**14-jun-23 00:12**	*Sample Weight:*	**1,0000**

Chromatogram

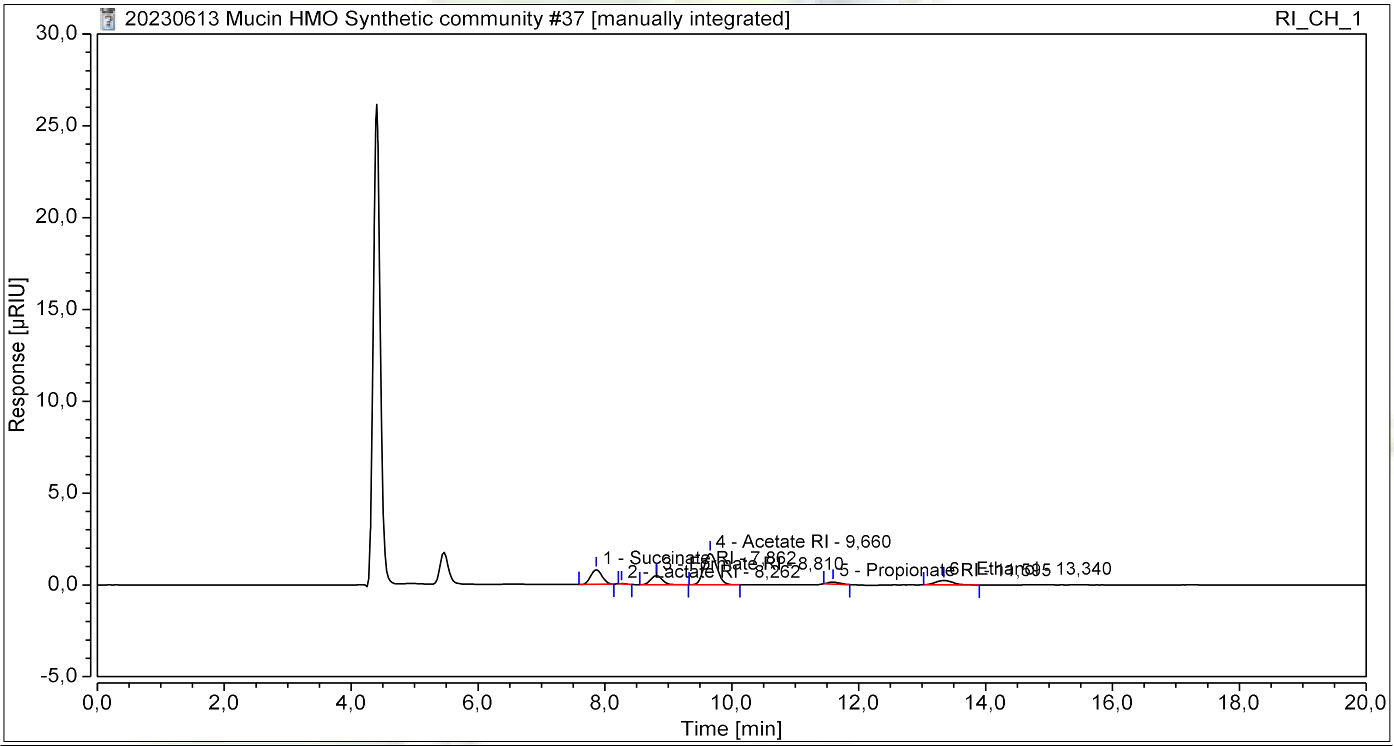

Integration Results

No.	Peak Name	Retention Time min	Area µRIU*min	Height µRIU	Relative Area %	Relative Height %	Amount
n.a.	GlcNAc	n.a.	n.a.	n.a.	n.a.	n.a.	n.a.
n.a.	Citrate	n.a.	n.a.	n.a.	n.a.	n.a.	n.a.
n.a.	Glucose	n.a.	n.a.	n.a.	n.a.	n.a.	n.a.
n.a.	Galactose	n.a.	n.a.	n.a.	n.a.	n.a.	n.a.
n.a.	Fucose	n.a.	n.a.	n.a.	n.a.	n.a.	n.a.
1	Succinate RI	7,862	0,165	0,793	21,43	23,72	n.a.
2	Lactate RI	8,262	0,003	0,018	0,33	0,55	0,0728
n.a.	glycerol	n.a.	n.a.	n.a.	n.a.	n.a.	n.a.
3	Formate RI	8,810	0,105	0,493	13,55	14,74	10,9689
4	Acetate RI	9,660	0,403	1,698	52,28	50,79	24,8061
n.a.	1,2 PDO RI	n.a.	n.a.	n.a.	n.a.	n.a.	n.a.
n.a.	1,3-PDO	n.a.	n.a.	n.a.	n.a.	n.a.	n.a.
5	Propionate RI	11,595	0,023	0,106	2,96	3,18	0,9168
n.a.	1,3-PDO	n.a.	n.a.	n.a.	n.a.	n.a.	n.a.
n.a.	2-3 BDO	n.a.	n.a.	n.a.	n.a.	n.a.	n.a.
6	Ethanol	13,340	0,073	0,235	9,46	7,03	0,6715
n.a.	Isobutyrate RI	n.a.	n.a.	n.a.	n.a.	n.a.	n.a.
n.a.	Butyrate RI	n.a.	n.a.	n.a.	n.a.	n.a.	n.a.
**Total:**			**0,771**	**3,344**	**100,00**	**100,00**	

Chromeleon (c) Dionex
Version 7.2.10.25868

Peak Analysis

Injection Details

Injection Name:	**27 GOSFOS t48 r3**	*Run Time (min):*	**20,00**
Vial Number:	**3:C6**	*Injection Volume:*	**10,00**
Injection Type:	**Unknown**	*Channel:*	**RI_CH_1**
Calibration Level:		*Wavelength:*	**n.a.**
Instrument Method:	**Default method LC2030C 45 gr 20 min**	*Bandwidth:*	**n.a.**
Processing Method:	**Processing Method LC2030 45 gr**	*Dilution Factor:*	**1,0000**
Injection Date/Time:	**14-jun-23 00:12**	*Sample Weight:*	**1,0000**

Chromatogram

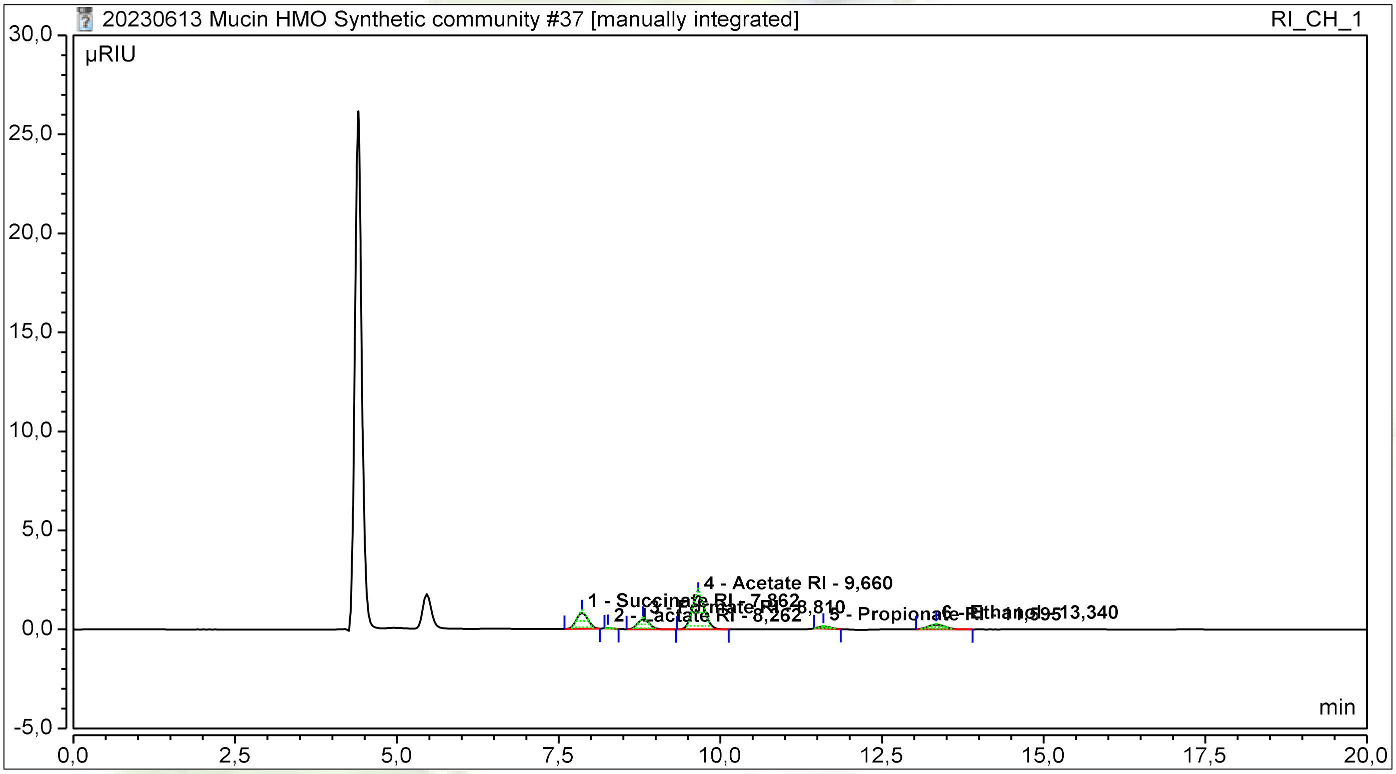

Peak Results

No.	Peak Name	Retention Time min	Width (50%) min	Type	Resolution (EP)	Asymmetry (EP)	Plates (EP)
n.a.	GlcNAc	n.a.	n.a.	n.a.	n.a.	n.a.	n.a.
n.a.	Citrate	n.a.	n.a.	n.a.	n.a.	n.a.	n.a.
n.a.	Glucose	n.a.	n.a.	n.a.	n.a.	n.a.	n.a.
n.a.	Galactose	n.a.	n.a.	n.a.	n.a.	n.a.	n.a.
n.a.	Fucose	n.a.	n.a.	n.a.	n.a.	n.a.	n.a.
1	Succinate RI	7,862	0,199	BMB	1,42	1,06	8645
2	Lactate RI	8,262	0,133	BMB*	1,94	2,06	21361
n.a.	glycerol	n.a.	n.a.	n.a.	n.a.	n.a.	n.a.
3	Formate RI	8,810	0,200	BMB	2,36	1,10	10763
4	Acetate RI	9,660	0,225	BMB	5,20	1,08	10246
n.a.	1,2 PDO RI	n.a.	n.a.	n.a.	n.a.	n.a.	n.a.
n.a.	1,3-PDO	n.a.	n.a.	n.a.	n.a.	n.a.	n.a.
5	Propionate RI	11,595	0,214	BMB*	4,03	1,30	16194
n.a.	1,3-PDO	n.a.	n.a.	n.a.	n.a.	n.a.	n.a.
n.a.	2-3 BDO	n.a.	n.a.	n.a.	n.a.	n.a.	n.a.
6	Ethanol	13,340	0,297	BMB*	n.a.	1,09	11185
n.a.	Isobutyrate RI	n.a.	n.a.	n.a.	n.a.	n.a.	n.a.
n.a.	Butyrate RI	n.a.	n.a.	n.a.	n.a.	n.a.	n.a.

Chromeleon (c) Dionex
Version 7.2.10.25868

Chromatogram and SST Results

Injection Details

Injection Name:	**27 GOSFOS t48 r3**	*Run Time (min):*	**20,00**
Vial Number:	**3:C6**	*Injection Volume:*	**10,00**
Injection Type:	**Unknown**	*Channel:*	**RI_CH_1**
Calibration Level:		*Wavelength:*	**n.a.**
Instrument Method:	**Default method LC2030C 45 gr 20 min**	*Bandwidth:*	**n.a.**
Processing Method:	**Processing Method LC2030 45 gr**	*Dilution Factor:*	**1,0000**
Injection Date/Time:	**14-jun-23 00:12**	*Sample Weight:*	**1,0000**

Chromatogram

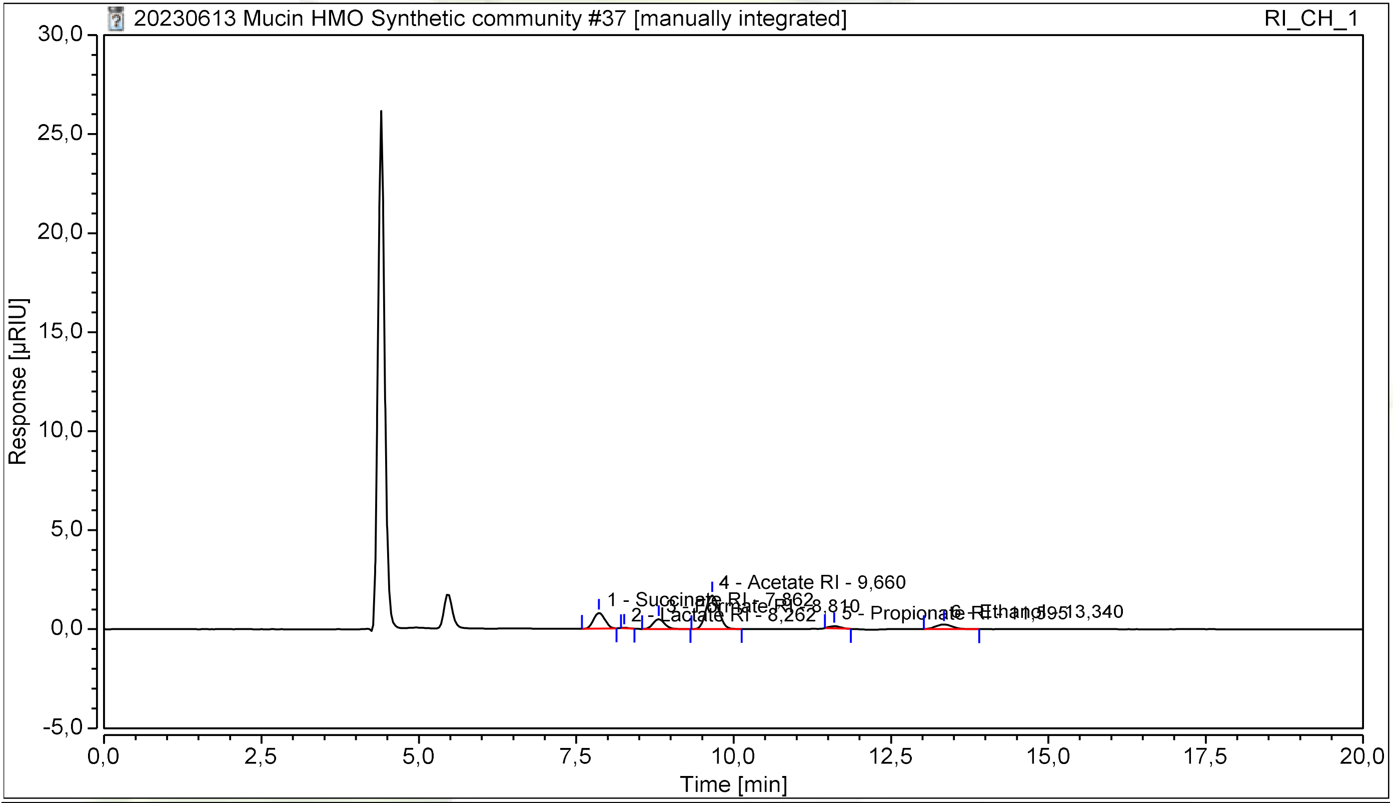

SST Results

No.	Name	Inj.Condition	Peak	Test Result	Injection

Number of executed test cases: **n.a.** **Total Result:** **Passed**

Chromatogram

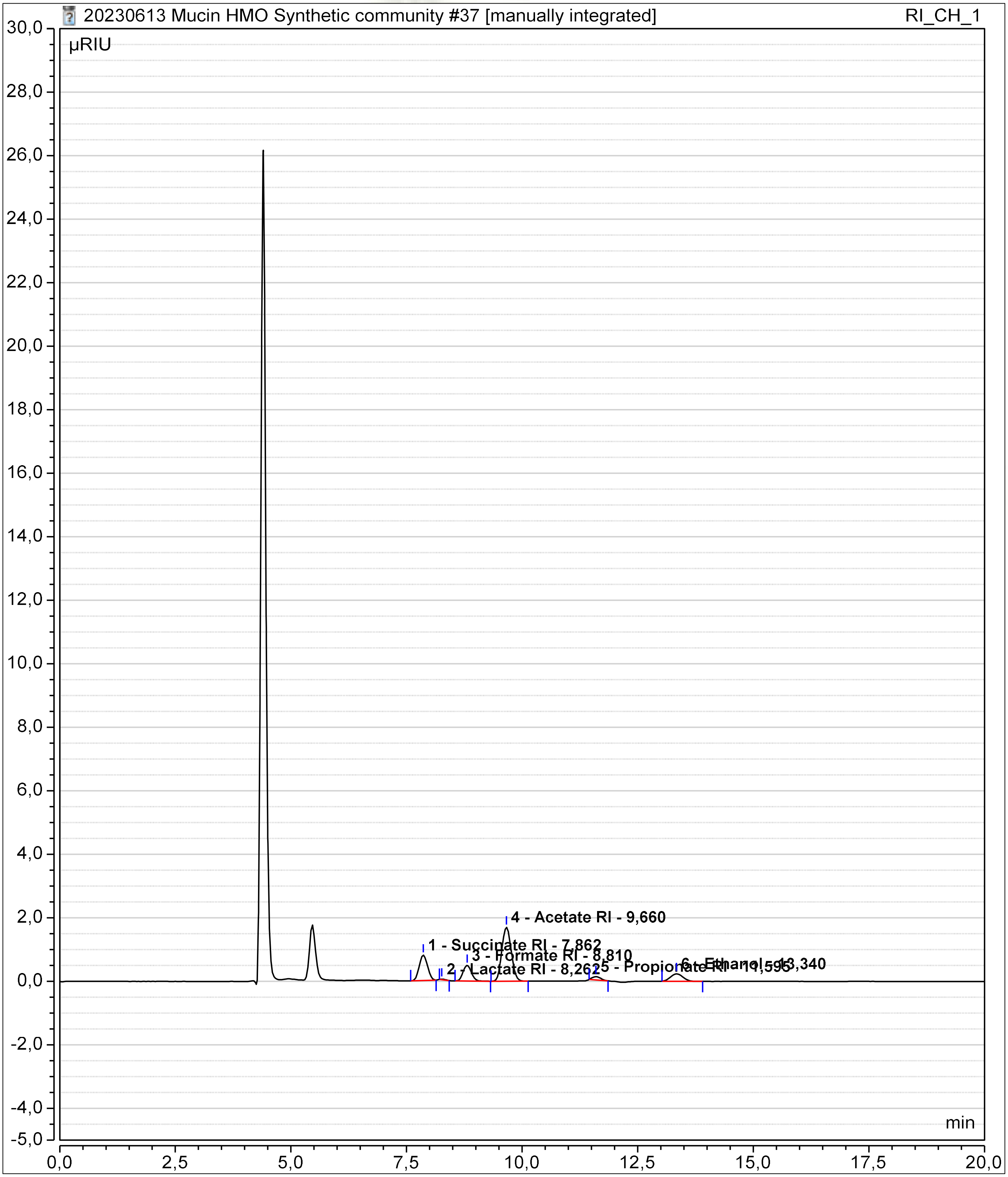

Chromatogram and Results

Injection Details

Injection Name:	**31 GOSFOSEXTR t48 r1**	*Run Time (min):*	**20,00**
Vial Number:	**3:C7**	*Injection Volume:*	**10,00**
Injection Type:	**Unknown**	*Channel:*	**RI_CH_1**
Calibration Level:		*Wavelength:*	**n.a.**
Instrument Method:	**Default method LC2030C 45 gr 20 min**	*Bandwidth:*	**n.a.**
Processing Method:	**Processing Method LC2030 45 gr**	*Dilution Factor:*	**1,0000**
Injection Date/Time:	**14-jun-23 00:32**	*Sample Weight:*	**1,0000**

Chromatogram

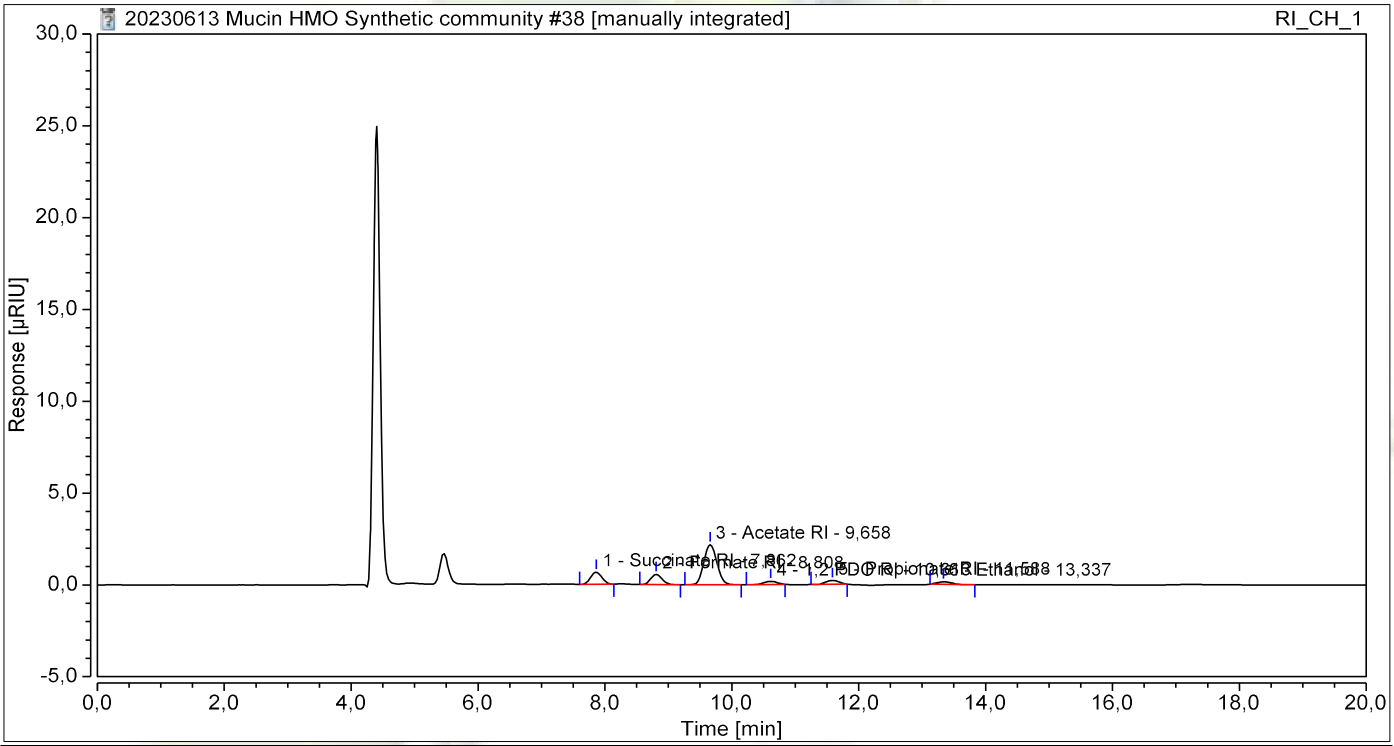

Integration Results

No.	Peak Name	Retention Time min	Area µRIU*min	Height µRIU	Relative Area %	Relative Height %	Amount
n.a.	GlcNAc	n.a.	n.a.	n.a.	n.a.	n.a.	n.a.
n.a.	Citrate	n.a.	n.a.	n.a.	n.a.	n.a.	n.a.
n.a.	Glucose	n.a.	n.a.	n.a.	n.a.	n.a.	n.a.
n.a.	Galactose	n.a.	n.a.	n.a.	n.a.	n.a.	n.a.
n.a.	Fucose	n.a.	n.a.	n.a.	n.a.	n.a.	n.a.
1	Succinate RI	7,862	0,135	0,653	15,09	16,77	n.a.
n.a.	Lactate RI	n.a.	n.a.	n.a.	n.a.	n.a.	n.a.
n.a.	glycerol	n.a.	n.a.	n.a.	n.a.	n.a.	n.a.
2	Formate RI	8,808	0,115	0,549	12,87	14,11	12,1159
3	Acetate RI	9,658	0,518	2,178	57,75	55,99	31,8789
4	1,2 PDO RI	10,613	0,040	0,166	4,43	4,28	1,1806
n.a.	1,3-PDO	n.a.	n.a.	n.a.	n.a.	n.a.	n.a.
5	Propionate RI	11,588	0,051	0,206	5,66	5,29	2,0416
n.a.	1,3-PDO	n.a.	n.a.	n.a.	n.a.	n.a.	n.a.
n.a.	2-3 BDO	n.a.	n.a.	n.a.	n.a.	n.a.	n.a.
6	Ethanol	13,337	0,038	0,138	4,20	3,56	0,3467
n.a.	Isobutyrate RI	n.a.	n.a.	n.a.	n.a.	n.a.	n.a.
n.a.	Butyrate RI	n.a.	n.a.	n.a.	n.a.	n.a.	n.a.
**Total:**			**0,897**	**3,891**	**100,00**	**100,00**	

Peak Analysis

Injection Details

Injection Name:	**31 GOSFOSEXTR t48 r1**	*Run Time (min):*	**20,00**
Vial Number:	**3:C7**	*Injection Volume:*	**10,00**
Injection Type:	**Unknown**	*Channel:*	**RI_CH_1**
Calibration Level:		*Wavelength:*	**n.a.**
Instrument Method:	**Default method LC2030C 45 gr 20 min**	*Bandwidth:*	**n.a.**
Processing Method:	**Processing Method LC2030 45 gr**	*Dilution Factor:*	**1,0000**
Injection Date/Time:	**14-jun-23 00:32**	*Sample Weight:*	**1,0000**

Chromatogram

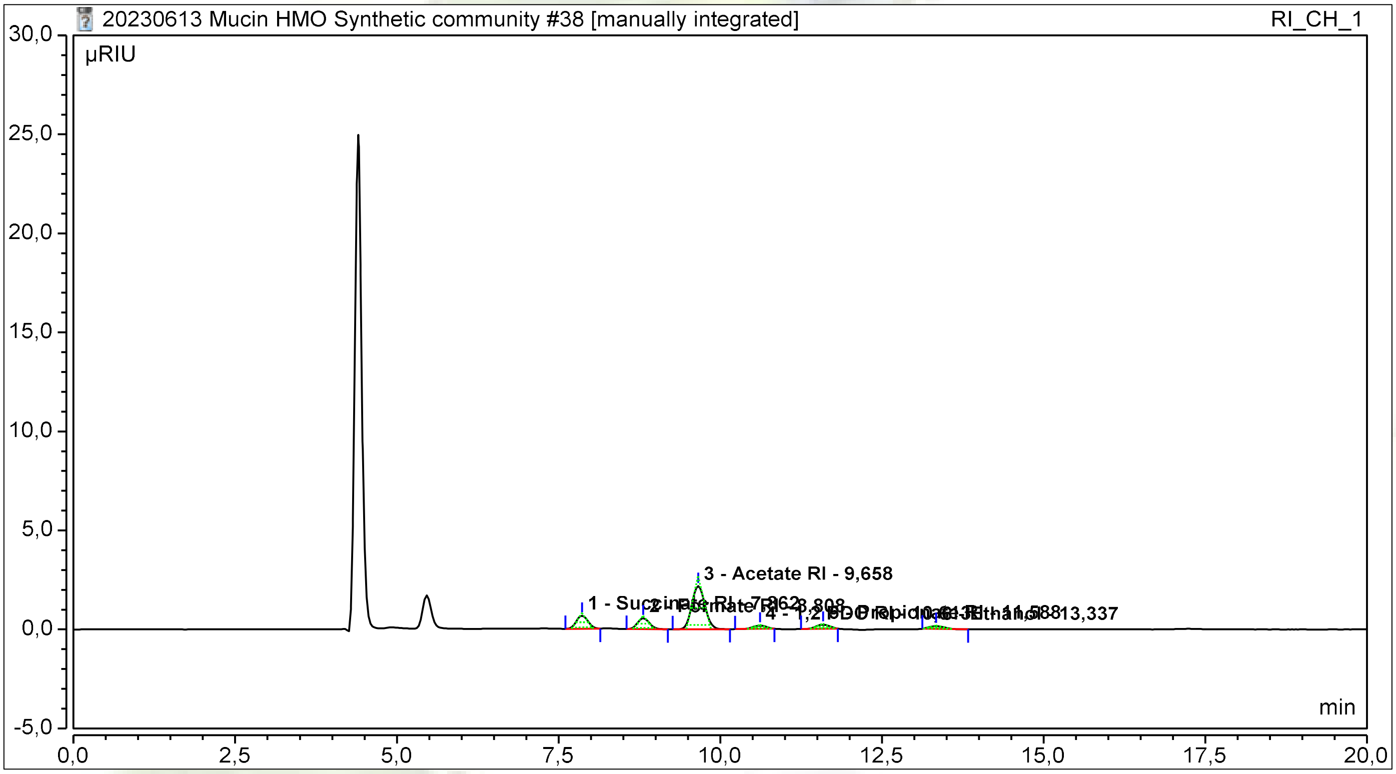

Peak Results

No.	Peak Name	Retention Time min	Width (50%) min	Type	Resolution (EP)	Asymmetry (EP)	Plates (EP)
n.a.	GlcNAc	n.a.	n.a.	n.a.	n.a.	n.a.	n.a.
n.a.	Citrate	n.a.	n.a.	n.a.	n.a.	n.a.	n.a.
n.a.	Glucose	n.a.	n.a.	n.a.	n.a.	n.a.	n.a.
n.a.	Galactose	n.a.	n.a.	n.a.	n.a.	n.a.	n.a.
n.a.	Fucose	n.a.	n.a.	n.a.	n.a.	n.a.	n.a.
1	Succinate RI	7,862	0,198	BMB	2,81	1,06	8705
n.a.	Lactate RI	n.a.	n.a.	n.a.	n.a.	n.a.	n.a.
n.a.	glycerol	n.a.	n.a.	n.a.	n.a.	n.a.	n.a.
2	Formate RI	8,808	0,199	BMB	2,37	1,10	10837
3	Acetate RI	9,658	0,225	BMB	2,45	1,08	10230
4	1,2 PDO RI	10,613	0,236	BMB*	2,40	0,98	11208
n.a.	1,3-PDO	n.a.	n.a.	n.a.	n.a.	n.a.	n.a.
5	Propionate RI	11,588	0,243	BMB*	4,00	0,98	12608
n.a.	1,3-PDO	n.a.	n.a.	n.a.	n.a.	n.a.	n.a.
n.a.	2-3 BDO	n.a.	n.a.	n.a.	n.a.	n.a.	n.a.
6	Ethanol	13,337	0,273	BMB*	n.a.	1,18	13187
n.a.	Isobutyrate RI	n.a.	n.a.	n.a.	n.a.	n.a.	n.a.
n.a.	Butyrate RI	n.a.	n.a.	n.a.	n.a.	n.a.	n.a.

Chromatogram and SST Results

Injection Details

Injection Name:	**31 GOSFOSEXTR t48 r1**	*Run Time (min):*	**20,00**
Vial Number:	**3:C7**	*Injection Volume:*	**10,00**
Injection Type:	**Unknown**	*Channel:*	**RI_CH_1**
Calibration Level:		*Wavelength:*	**n.a.**
Instrument Method:	**Default method LC2030C 45 gr 20 min**	*Bandwidth:*	**n.a.**
Processing Method:	**Processing Method LC2030 45 gr**	*Dilution Factor:*	**1,0000**
Injection Date/Time:	**14-jun-23 00:32**	*Sample Weight:*	**1,0000**

Chromatogram

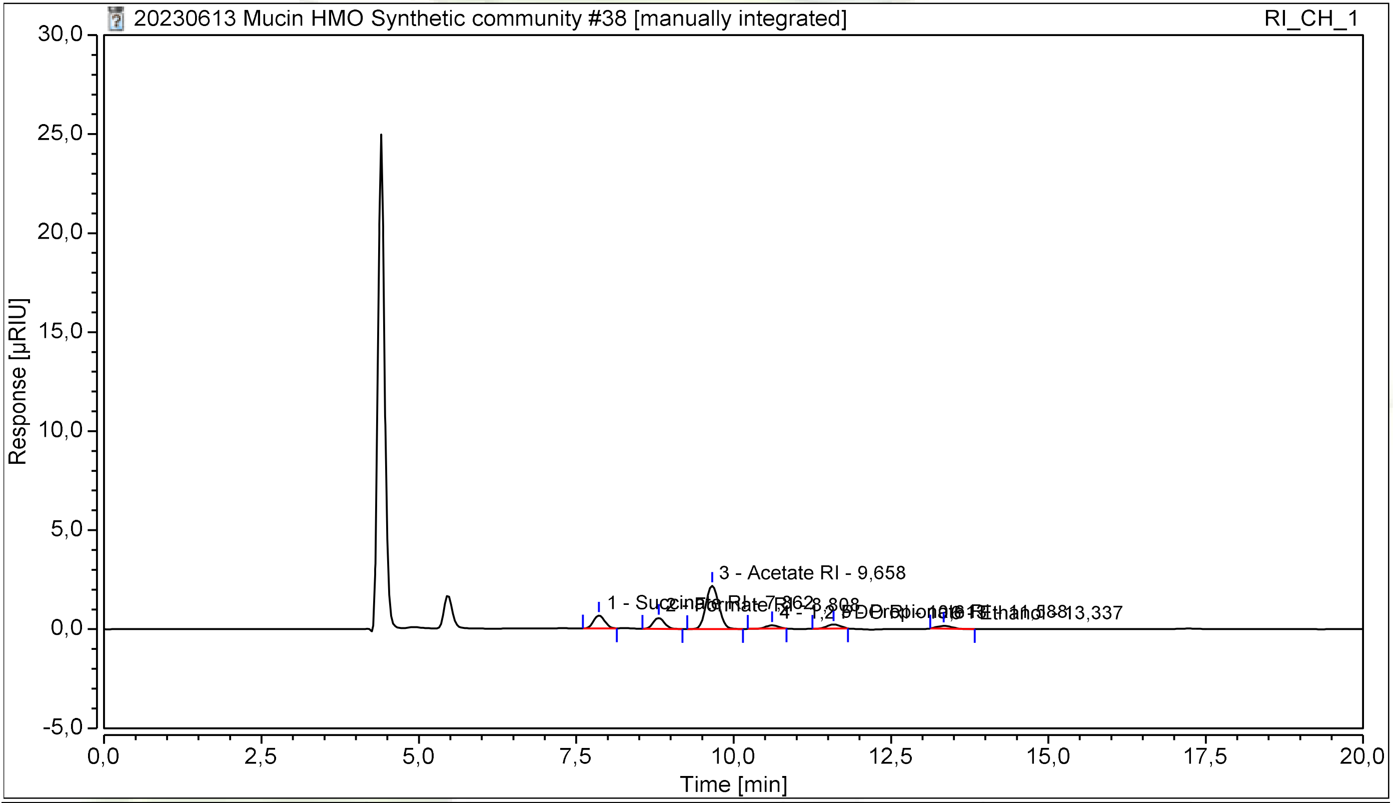

SST Results

No.	Name	Inj.Condition	Peak	Test Result	Injection
**Number of executed test cases:**	**n.a.**		**Total Result:**	**Passed**	

Chromatogram

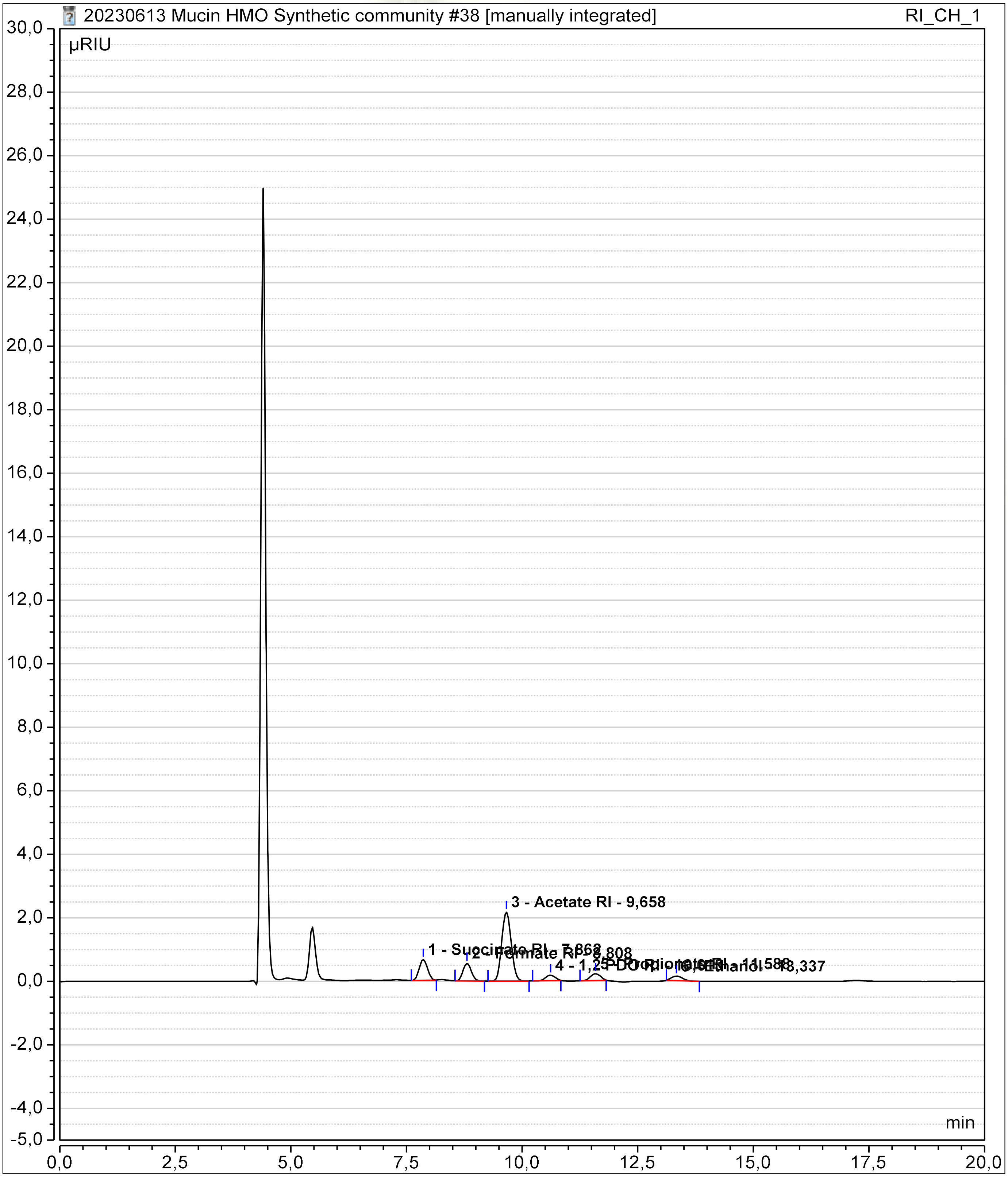

Chromeleon (c) Dionex
Version 7.2.10.25868

Chromatogram and Results

Injection Details

Injection Name:	**32 GOSFOSEXTR t48 r2**	*Run Time (min):*	**20,00**
Vial Number:	**3:C8**	*Injection Volume:*	**10,00**
Injection Type:	**Unknown**	*Channel:*	**RI_CH_1**
Calibration Level:		*Wavelength:*	**n.a.**
Instrument Method:	**Default method LC2030C 45 gr 20 min**	*Bandwidth:*	**n.a.**
Processing Method:	**Processing Method LC2030 45 gr**	*Dilution Factor:*	**1,0000**
Injection Date/Time:	**14-jun-23 00:53**	*Sample Weight:*	**1,0000**

Chromatogram

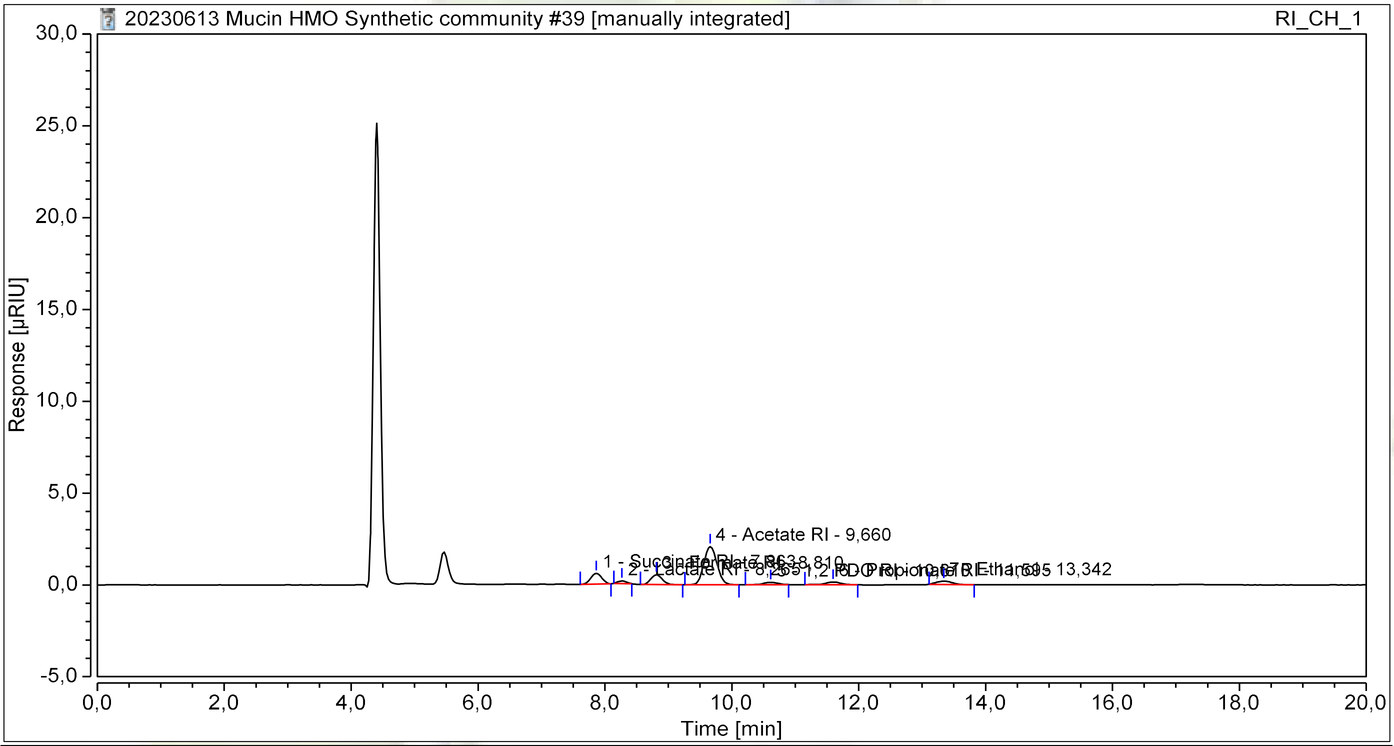

Integration Results

No.	Peak Name	Retention Time min	Area µRIU*min	Height µRIU	Relative Area %	Relative Height %	Amount
n.a.	GlcNAc	n.a.	n.a.	n.a.	n.a.	n.a.	n.a.
n.a.	Citrate	n.a.	n.a.	n.a.	n.a.	n.a.	n.a.
n.a.	Glucose	n.a.	n.a.	n.a.	n.a.	n.a.	n.a.
n.a.	Galactose	n.a.	n.a.	n.a.	n.a.	n.a.	n.a.
n.a.	Fucose	n.a.	n.a.	n.a.	n.a.	n.a.	n.a.
1	Succinate RI	7,863	0,117	0,578	13,48	15,35	n.a.
2	Lactate RI	8,265	0,022	0,134	2,51	3,56	0,6329
n.a.	glycerol	n.a.	n.a.	n.a.	n.a.	n.a.	n.a.
3	Formate RI	8,810	0,112	0,532	12,85	14,11	11,7132
4	Acetate RI	9,660	0,494	2,073	56,93	55,01	30,4219
5	1,2 PDO RI	10,610	0,031	0,121	3,55	3,21	0,9174
n.a.	1,3-PDO	n.a.	n.a.	n.a.	n.a.	n.a.	n.a.
6	Propionate RI	11,595	0,041	0,147	4,69	3,89	1,6395
n.a.	1,3-PDO	n.a.	n.a.	n.a.	n.a.	n.a.	n.a.
n.a.	2-3 BDO	n.a.	n.a.	n.a.	n.a.	n.a.	n.a.
7	Ethanol	13,342	0,052	0,184	5,98	4,87	0,4781
n.a.	Isobutyrate RI	n.a.	n.a.	n.a.	n.a.	n.a.	n.a.
n.a.	Butyrate RI	n.a.	n.a.	n.a.	n.a.	n.a.	n.a.
**Total:**			**0,868**	**3,768**	**100,00**	**100,00**	

Chromeleon (c) Dionex
Version 7.2.10.25868

Peak Analysis

Injection Details

Injection Name:	**32 GOSFOSEXTR t48 r2**	*Run Time (min):*	**20,00**
Vial Number:	**3:C8**	*Injection Volume:*	**10,00**
Injection Type:	**Unknown**	*Channel:*	**RI_CH_1**
Calibration Level:		*Wavelength:*	**n.a.**
Instrument Method:	**Default method LC2030C 45 gr 20 min**	*Bandwidth:*	**n.a.**
Processing Method:	**Processing Method LC2030 45 gr**	*Dilution Factor:*	**1,0000**
Injection Date/Time:	**14-jun-23 00:53**	*Sample Weight:*	**1,0000**

Chromatogram

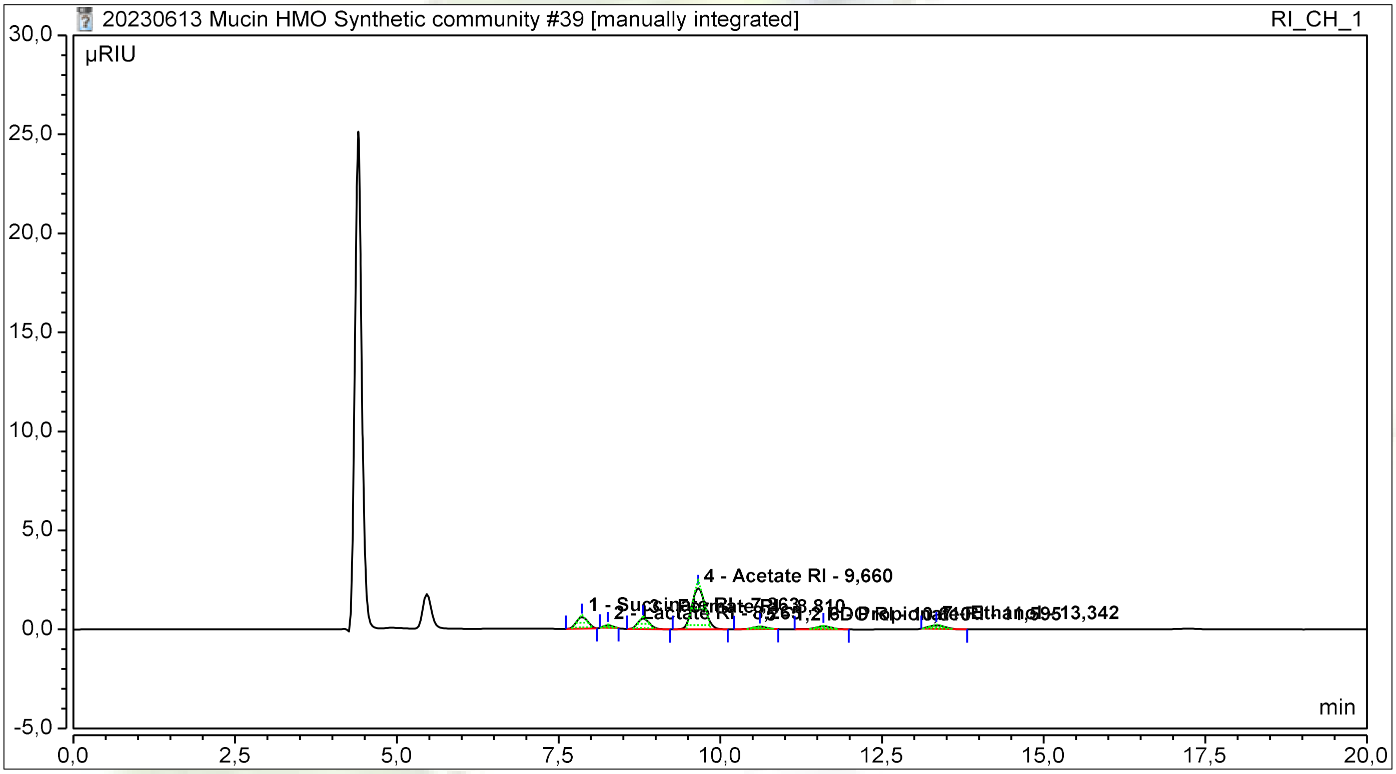

Peak Results

No.	Peak Name	Retention Time min	Width (50%) min	Type	Resolution (EP)	Asymmetry (EP)	Plates (EP)
n.a.	GlcNAc	n.a.	n.a.	n.a.	n.a.	n.a.	n.a.
n.a.	Citrate	n.a.	n.a.	n.a.	n.a.	n.a.	n.a.
n.a.	Glucose	n.a.	n.a.	n.a.	n.a.	n.a.	n.a.
n.a.	Galactose	n.a.	n.a.	n.a.	n.a.	n.a.	n.a.
n.a.	Fucose	n.a.	n.a.	n.a.	n.a.	n.a.	n.a.
1	Succinate RI	7,863	0,195	BMB	1,32	1,02	8968
2	Lactate RI	8,265	0,165	BMB*	1,77	1,14	13975
n.a.	glycerol	n.a.	n.a.	n.a.	n.a.	n.a.	n.a.
3	Formate RI	8,810	0,199	BMB	2,36	1,10	10832
4	Acetate RI	9,660	0,225	BMB	2,38	1,08	10205
5	1,2 PDO RI	10,610	0,246	BMB*	2,33	1,03	10296
n.a.	1,3-PDO	n.a.	n.a.	n.a.	n.a.	n.a.	n.a.
6	Propionate RI	11,595	0,254	BMB*	3,88	0,90	11566
n.a.	1,3-PDO	n.a.	n.a.	n.a.	n.a.	n.a.	n.a.
n.a.	2-3 BDO	n.a.	n.a.	n.a.	n.a.	n.a.	n.a.
7	Ethanol	13,342	0,278	BMB*	n.a.	1,15	12753
n.a.	Isobutyrate RI	n.a.	n.a.	n.a.	n.a.	n.a.	n.a.
n.a.	Butyrate RI	n.a.	n.a.	n.a.	n.a.	n.a.	n.a.

Chromatogram and SST Results

Injection Details

Injection Name:	**32 GOSFOSEXTR t48 r2**	*Run Time (min):*	**20,00**
Vial Number:	**3:C8**	*Injection Volume:*	**10,00**
Injection Type:	**Unknown**	*Channel:*	**RI_CH_1**
Calibration Level:		*Wavelength:*	**n.a.**
Instrument Method:	**Default method LC2030C 45 gr 20 min**	*Bandwidth:*	**n.a.**
Processing Method:	**Processing Method LC2030 45 gr**	*Dilution Factor:*	**1,0000**
Injection Date/Time:	**14-jun-23 00:53**	*Sample Weight:*	**1,0000**

Chromatogram

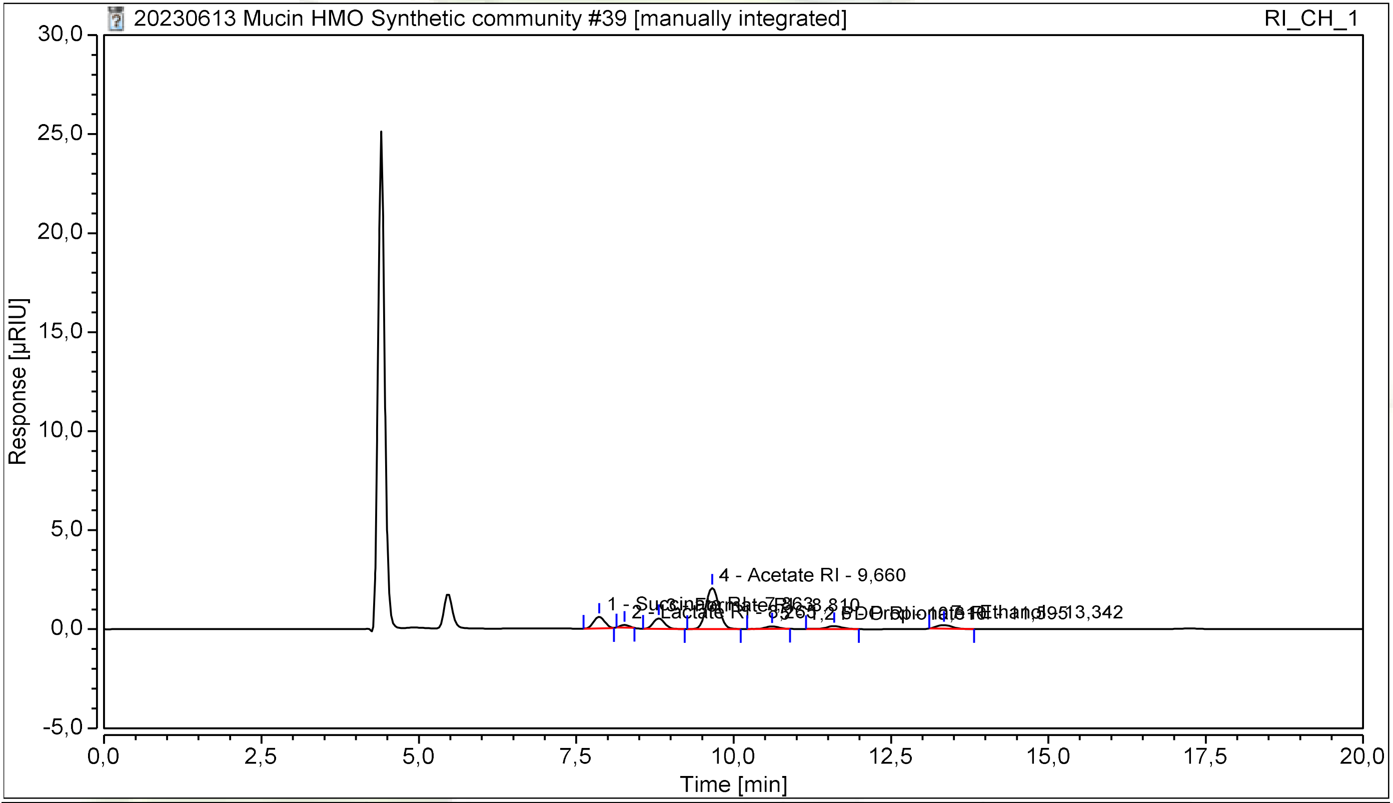

SST Results

No.	Name	Inj.Condition	Peak	Test Result	Injection
**Number of executed test cases:**	**n.a.**		**Total Result:**	**Passed**	

Chromatogram

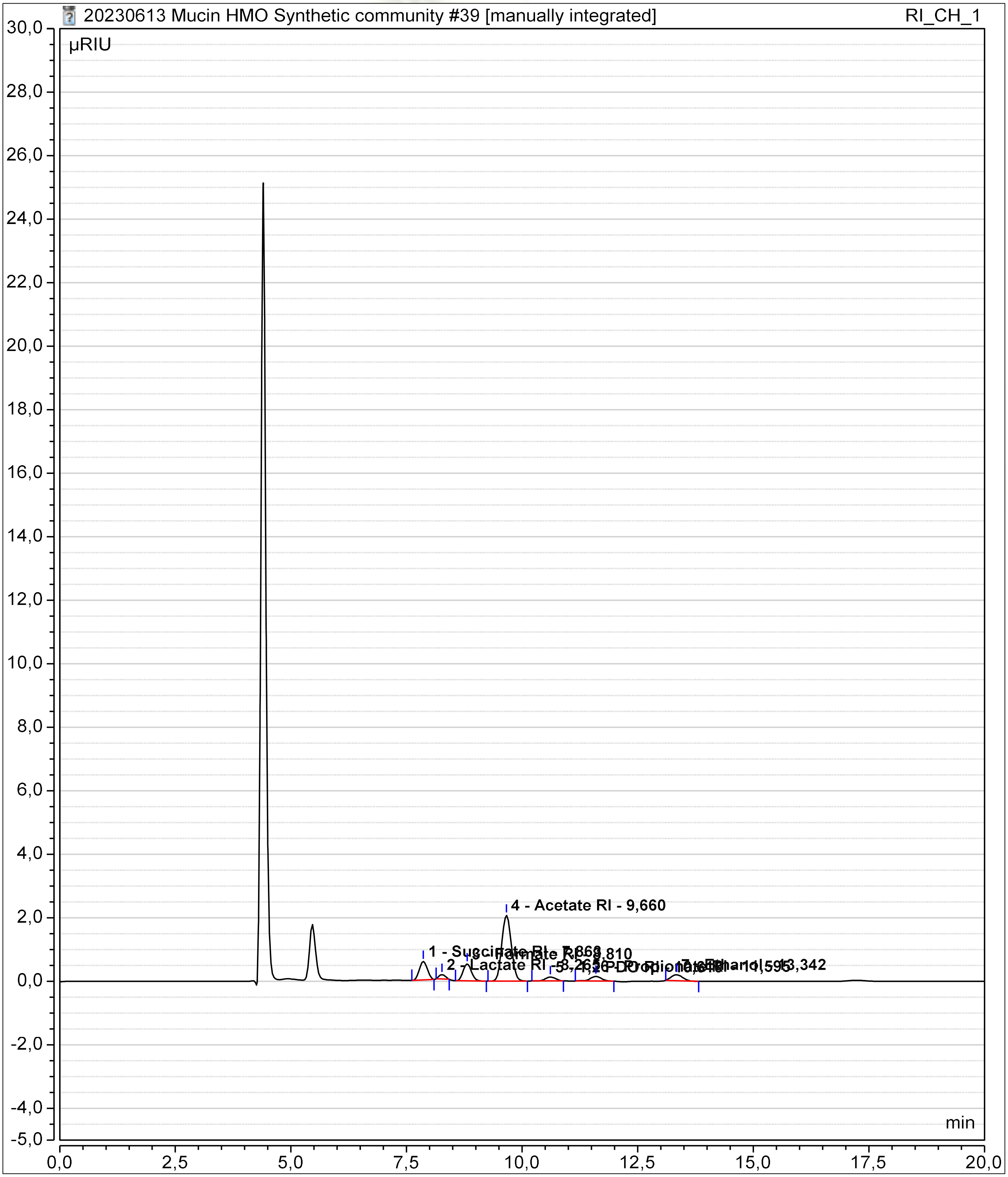

Chromatogram and Results

Injection Details

Injection Name:	**33 GOSFOSEXTR t48 r3**	*Run Time (min):*	**20,00**
Vial Number:	**3:C9**	*Injection Volume:*	**10,00**
Injection Type:	**Unknown**	*Channel:*	**RI_CH_1**
Calibration Level:		*Wavelength:*	**n.a.**
Instrument Method:	**Default method LC2030C 45 gr 20 min**	*Bandwidth:*	**n.a.**
Processing Method:	**Processing Method LC2030 45 gr**	*Dilution Factor:*	**1,0000**
Injection Date/Time:	**14-jun-23 01:13**	*Sample Weight:*	**1,0000**

Chromatogram

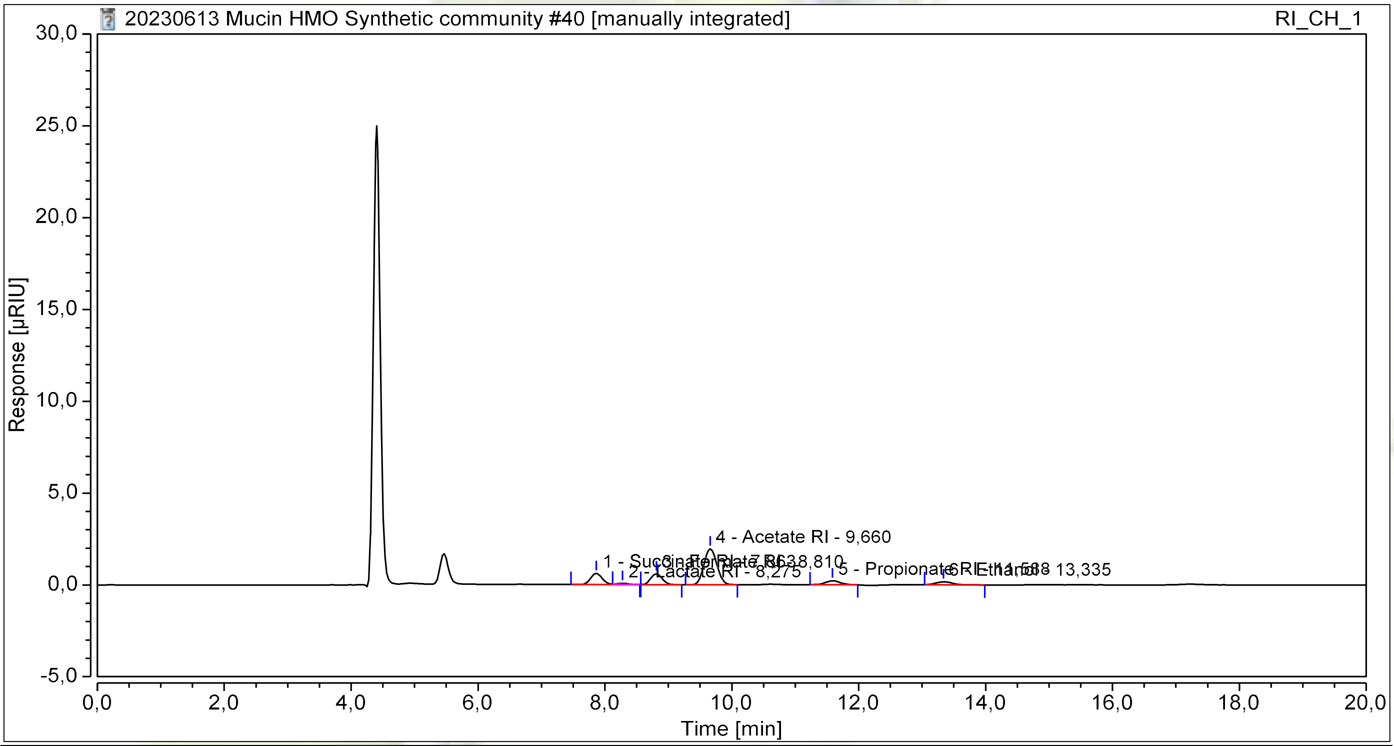

Integration Results

No.	Peak Name	Retention Time min	Area µRIU*min	Height µRIU	Relative Area %	Relative Height %	Amount
n.a.	GlcNAc	n.a.	n.a.	n.a.	n.a.	n.a.	n.a.
n.a.	Citrate	n.a.	n.a.	n.a.	n.a.	n.a.	n.a.
n.a.	Glucose	n.a.	n.a.	n.a.	n.a.	n.a.	n.a.
n.a.	Galactose	n.a.	n.a.	n.a.	n.a.	n.a.	n.a.
n.a.	Fucose	n.a.	n.a.	n.a.	n.a.	n.a.	n.a.
1	Succinate RI	7,863	0,136	0,604	16,19	16,93	n.a.
2	Lactate RI	8,275	0,009	0,051	1,12	1,43	0,2735
n.a.	glycerol	n.a.	n.a.	n.a.	n.a.	n.a.	n.a.
3	Formate RI	8,810	0,125	0,590	14,86	16,55	13,1378
4	Acetate RI	9,660	0,465	1,953	55,16	54,77	28,5904
n.a.	1,2 PDO RI	n.a.	n.a.	n.a.	n.a.	n.a.	n.a.
n.a.	1,3-PDO	n.a.	n.a.	n.a.	n.a.	n.a.	n.a.
5	Propionate RI	11,588	0,058	0,208	6,85	5,84	2,3206
n.a.	1,3-PDO	n.a.	n.a.	n.a.	n.a.	n.a.	n.a.
n.a.	2-3 BDO	n.a.	n.a.	n.a.	n.a.	n.a.	n.a.
6	Ethanol	13,335	0,049	0,160	5,82	4,49	0,4511
n.a.	Isobutyrate RI	n.a.	n.a.	n.a.	n.a.	n.a.	n.a.
n.a.	Butyrate RI	n.a.	n.a.	n.a.	n.a.	n.a.	n.a.
**Total:**			**0,842**	**3,566**	**100,00**	**100,00**	

Peak Analysis

Injection Details

Injection Name:	**33 GOSFOSEXTR t48 r3**	*Run Time (min):*	**20,00**
Vial Number:	**3:C9**	*Injection Volume:*	**10,00**
Injection Type:	**Unknown**	*Channel:*	**RI_CH_1**
Calibration Level:		*Wavelength:*	**n.a.**
Instrument Method:	**Default method LC2030C 45 gr 20 min**	*Bandwidth:*	**n.a.**
Processing Method:	**Processing Method LC2030 45 gr**	*Dilution Factor:*	**1,0000**
Injection Date/Time:	**14-jun-23 01:13**	*Sample Weight:*	**1,0000**

Chromatogram

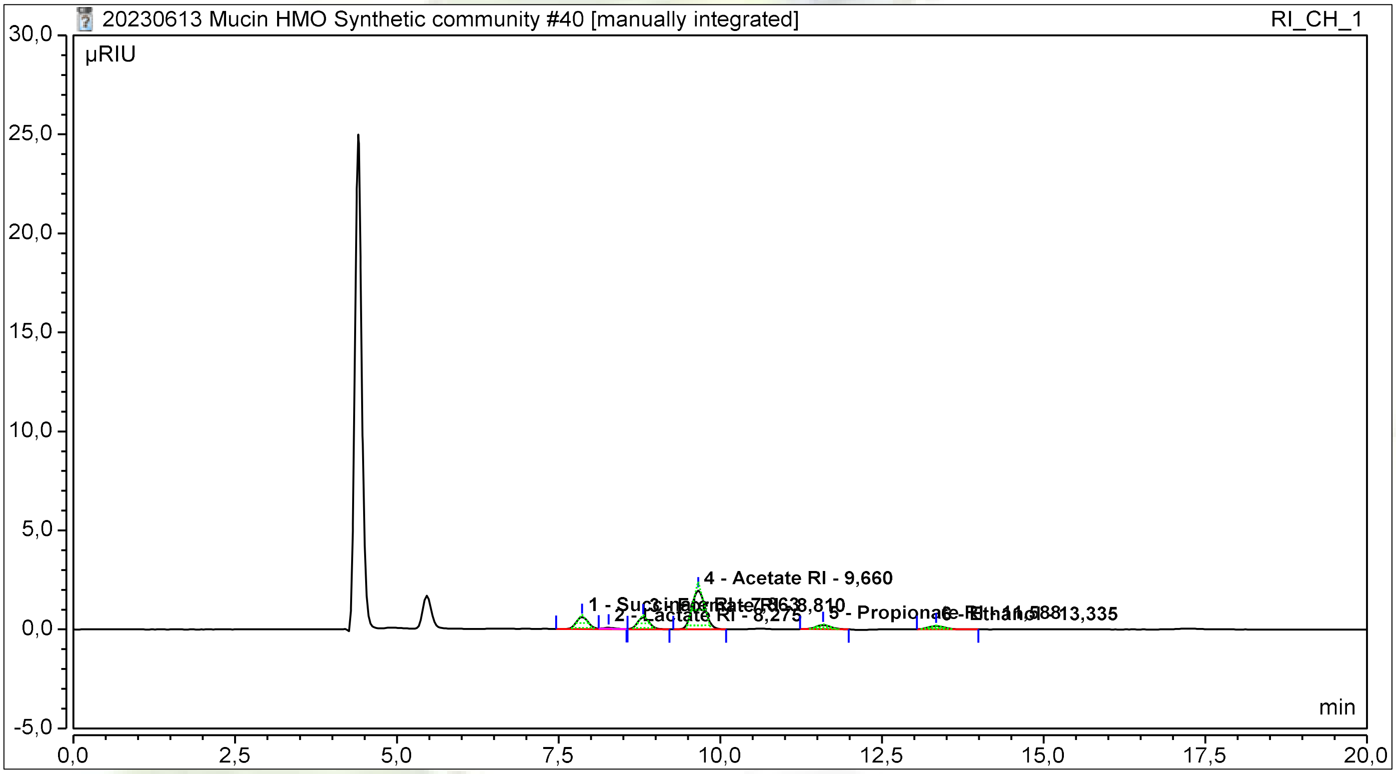

Peak Results

No.	Peak Name	Retention Time min	Width (50%) min	Type	Resolution (EP)	Asymmetry (EP)	Plates (EP)
n.a.	GlcNAc	n.a.	n.a.	n.a.	n.a.	n.a.	n.a.
n.a.	Citrate	n.a.	n.a.	n.a.	n.a.	n.a.	n.a.
n.a.	Glucose	n.a.	n.a.	n.a.	n.a.	n.a.	n.a.
n.a.	Galactose	n.a.	n.a.	n.a.	n.a.	n.a.	n.a.
n.a.	Fucose	n.a.	n.a.	n.a.	n.a.	n.a.	n.a.
1	Succinate RI	7,863	0,202	BM	2,78	1,11	8411
2	Lactate RI	8,275	n.a.	Rd	n.a.	n.a.	n.a.
n.a.	glycerol	n.a.	n.a.	n.a.	n.a.	n.a.	n.a.
3	Formate RI	8,810	0,200	MB	2,36	1,09	10734
4	Acetate RI	9,660	0,225	BMB	4,70	1,08	10230
n.a.	1,2 PDO RI	n.a.	n.a.	n.a.	n.a.	n.a.	n.a.
n.a.	1,3-PDO	n.a.	n.a.	n.a.	n.a.	n.a.	n.a.
5	Propionate RI	11,588	0,260	BMB*	3,72	1,11	11047
n.a.	1,3-PDO	n.a.	n.a.	n.a.	n.a.	n.a.	n.a.
n.a.	2-3 BDO	n.a.	n.a.	n.a.	n.a.	n.a.	n.a.
6	Ethanol	13,335	0,295	BMB*	n.a.	1,09	11328
n.a.	Isobutyrate RI	n.a.	n.a.	n.a.	n.a.	n.a.	n.a.
n.a.	Butyrate RI	n.a.	n.a.	n.a.	n.a.	n.a.	n.a.

Chromatogram and SST Results

Injection Details

Injection Name:	**33 GOSFOSEXTR t48 r3**	*Run Time (min):*	**20,00**
Vial Number:	**3:C9**	*Injection Volume:*	**10,00**
Injection Type:	**Unknown**	*Channel:*	**RI_CH_1**
Calibration Level:		*Wavelength:*	**n.a.**
Instrument Method:	**Default method LC2030C 45 gr 20 min**	*Bandwidth:*	**n.a.**
Processing Method:	**Processing Method LC2030 45 gr**	*Dilution Factor:*	**1,0000**
Injection Date/Time:	**14-jun-23 01:13**	*Sample Weight:*	**1,0000**

Chromatogram

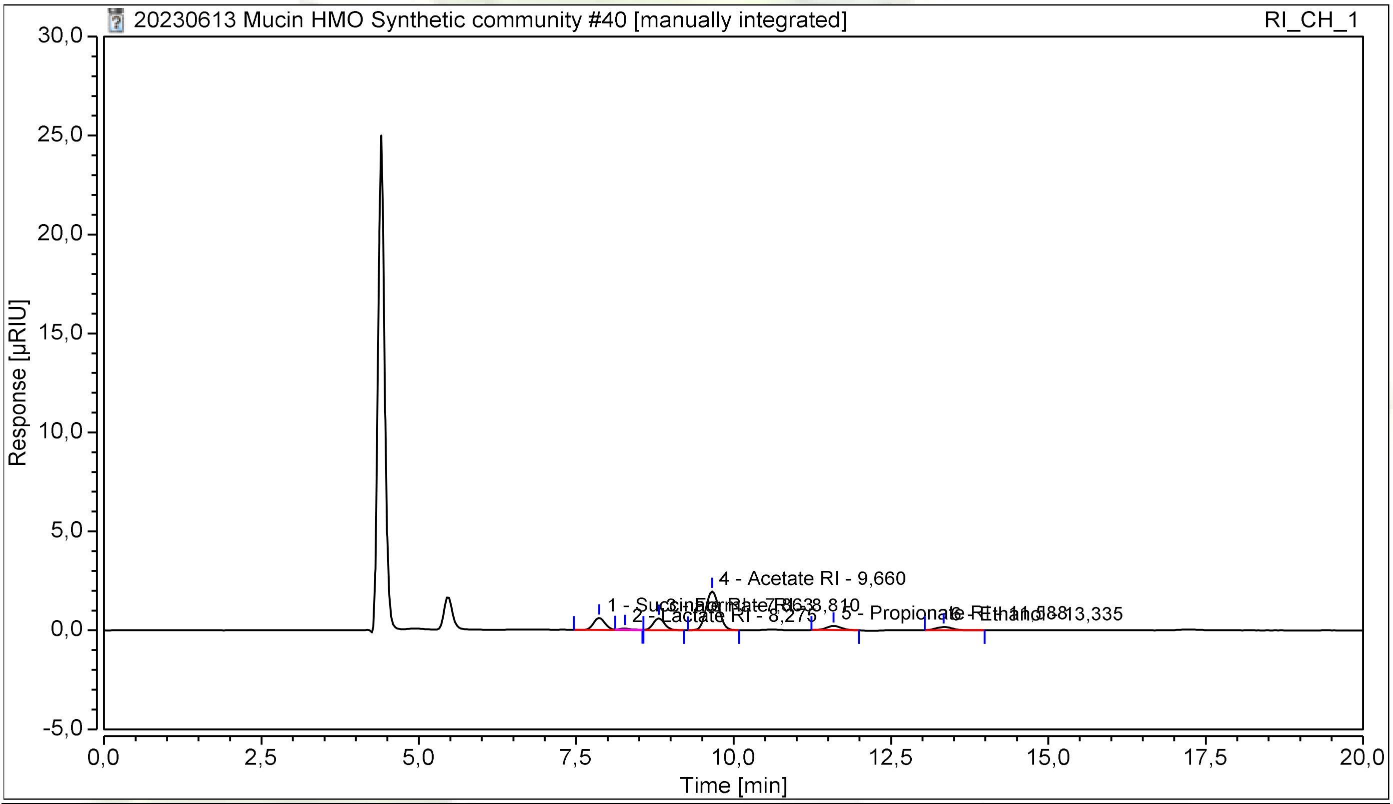

SST Results

No.	Name	Inj.Condition	Peak	Test Result	Injection

**Number of executed test cases:**	**n.a.**	**Total Result:**	**Passed**	

Chromatogram

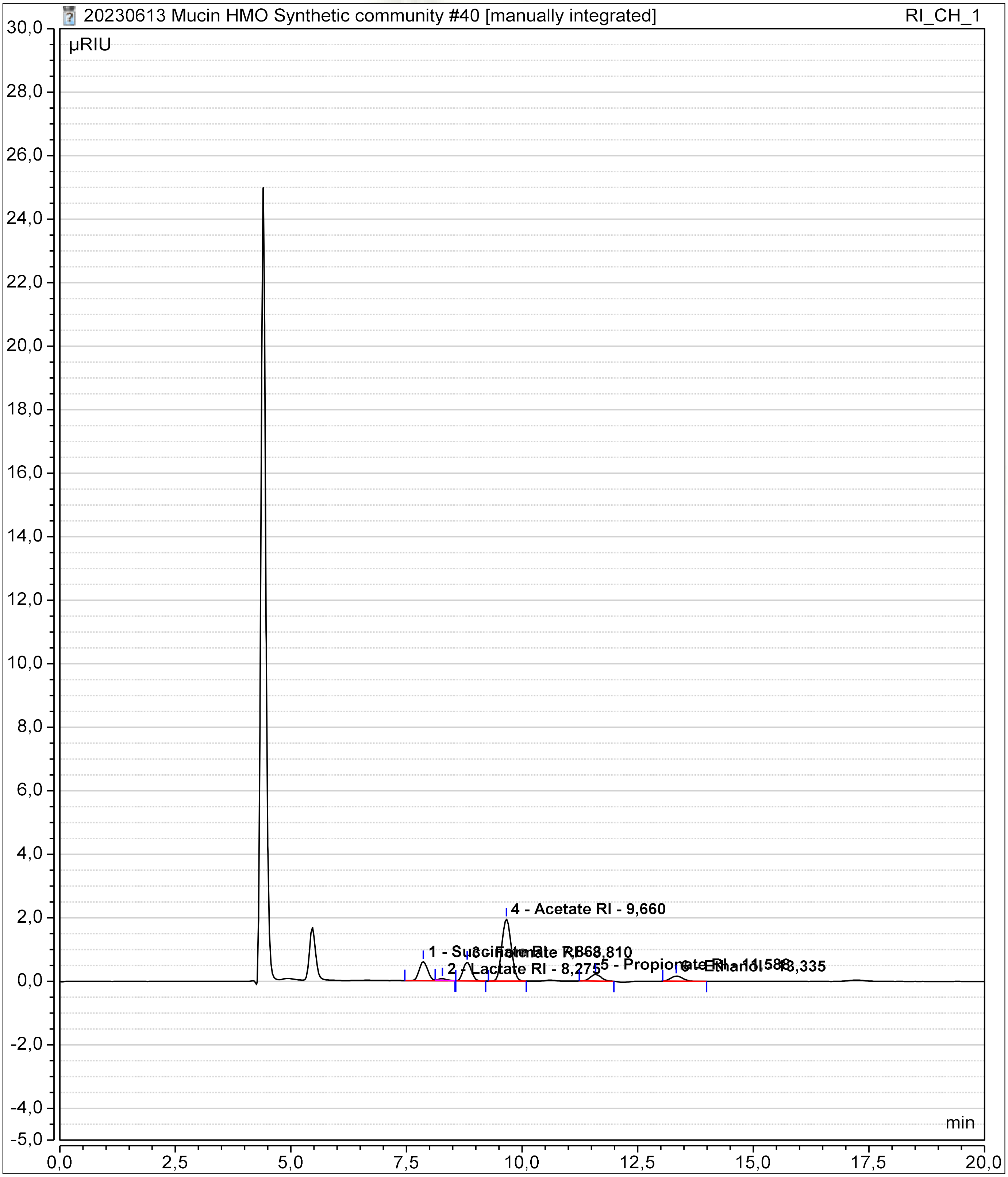

Chromeleon (c) Dionex
Version 7.2.10.25868

Chromatogram and Results

Injection Details

Injection Name:	**1 MUCHMO1 t48 r1**	*Run Time (min):*	**20,00**
Vial Number:	**3:C10**	*Injection Volume:*	**20,00**
Injection Type:	**Unknown**	*Channel:*	**RI_CH_1**
Calibration Level:		*Wavelength:*	**n.a.**
Instrument Method:	**Default method LC2030C 45 gr 20 min**	*Bandwidth:*	**n.a.**
Processing Method:	**Processing Method LC2030 45 gr**	*Dilution Factor:*	**1,0000**
Injection Date/Time:	**14-jun-23 01:34**	*Sample Weight:*	**1,0000**

Chromatogram

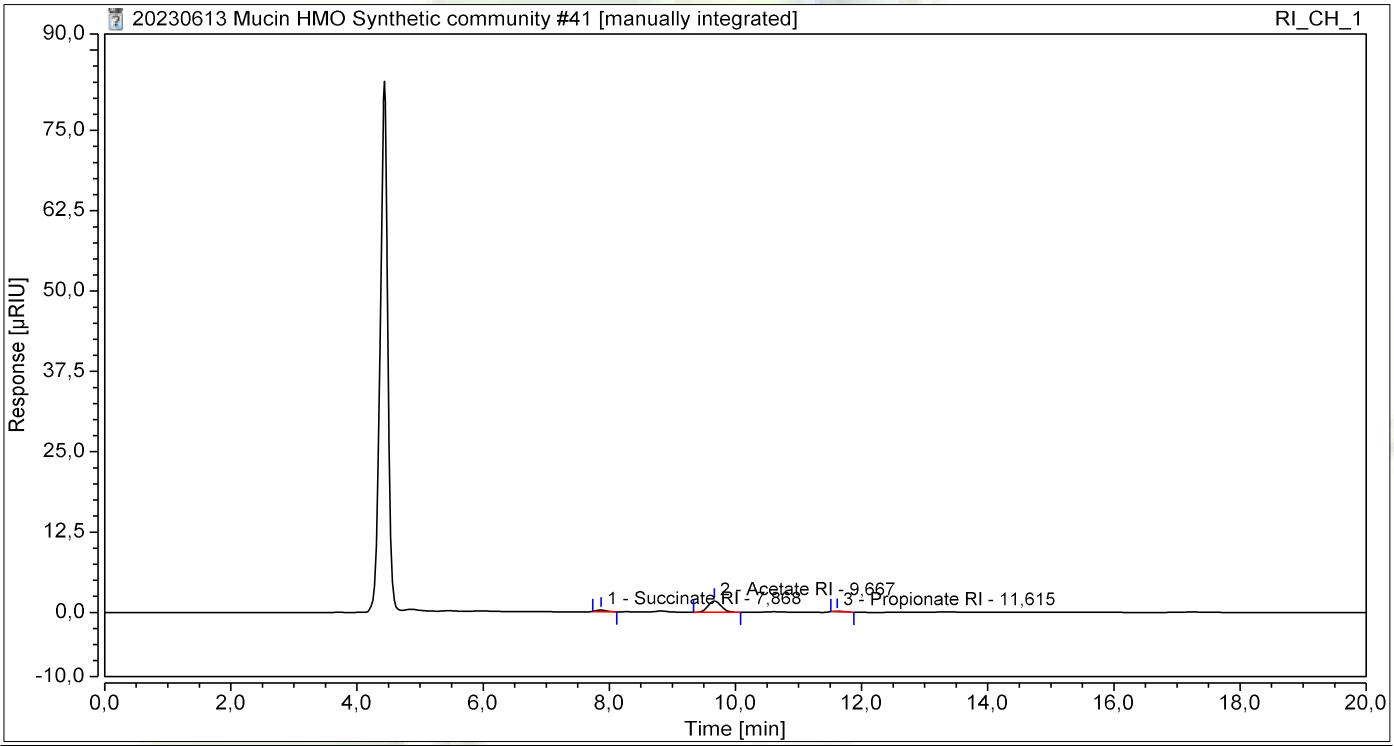

Integration Results

No.	Peak Name	Retention Time<br>min	Area<br>µRIU*min	Height<br>µRIU	Relative Area<br>%	Relative Height<br>%	Amount
n.a.	GlcNAc	n.a.	n.a.	n.a.	n.a.	n.a.	n.a.
n.a.	Citrate	n.a.	n.a.	n.a.	n.a.	n.a.	n.a.
n.a.	Glucose	n.a.	n.a.	n.a.	n.a.	n.a.	n.a.
n.a.	Galactose	n.a.	n.a.	n.a.	n.a.	n.a.	n.a.
n.a.	Fucose	n.a.	n.a.	n.a.	n.a.	n.a.	n.a.
1	Succinate RI	7,868	0,043	0,238	9,00	11,35	n.a.
n.a.	Lactate RI	n.a.	n.a.	n.a.	n.a.	n.a.	n.a.
n.a.	glycerol	n.a.	n.a.	n.a.	n.a.	n.a.	n.a.
n.a.	Formate RI	n.a.	n.a.	n.a.	n.a.	n.a.	n.a.
2	Acetate RI	9,667	0,415	1,747	86,79	83,46	25,5218
n.a.	1,2 PDO RI	n.a.	n.a.	n.a.	n.a.	n.a.	n.a.
n.a.	1,3-PDO	n.a.	n.a.	n.a.	n.a.	n.a.	n.a.
3	Propionate RI	11,615	0,020	0,108	4,21	5,18	0,8086
n.a.	1,3-PDO	n.a.	n.a.	n.a.	n.a.	n.a.	n.a.
n.a.	2-3 BDO	n.a.	n.a.	n.a.	n.a.	n.a.	n.a.
n.a.	Ethanol	n.a.	n.a.	n.a.	n.a.	n.a.	n.a.
n.a.	Isobutyrate RI	n.a.	n.a.	n.a.	n.a.	n.a.	n.a.
n.a.	Butyrate RI	n.a.	n.a.	n.a.	n.a.	n.a.	n.a.
**Total:**			**0,478**	**2,094**	**100,00**	**100,00**	

Chromeleon (c) Dionex
Version 7.2.10.25868

Peak Analysis

Injection Details

Injection Name:	**1 MUCHMO1 t48 r1**	*Run Time (min):*	**20,00**
Vial Number:	**3:C10**	*Injection Volume:*	**20,00**
Injection Type:	**Unknown**	*Channel:*	**RI_CH_1**
Calibration Level:		*Wavelength:*	**n.a.**
Instrument Method:	**Default method LC2030C 45 gr 20 min**	*Bandwidth:*	**n.a.**
Processing Method:	**Processing Method LC2030 45 gr**	*Dilution Factor:*	**1,0000**
Injection Date/Time:	**14-jun-23 01:34**	*Sample Weight:*	**1,0000**

Chromatogram

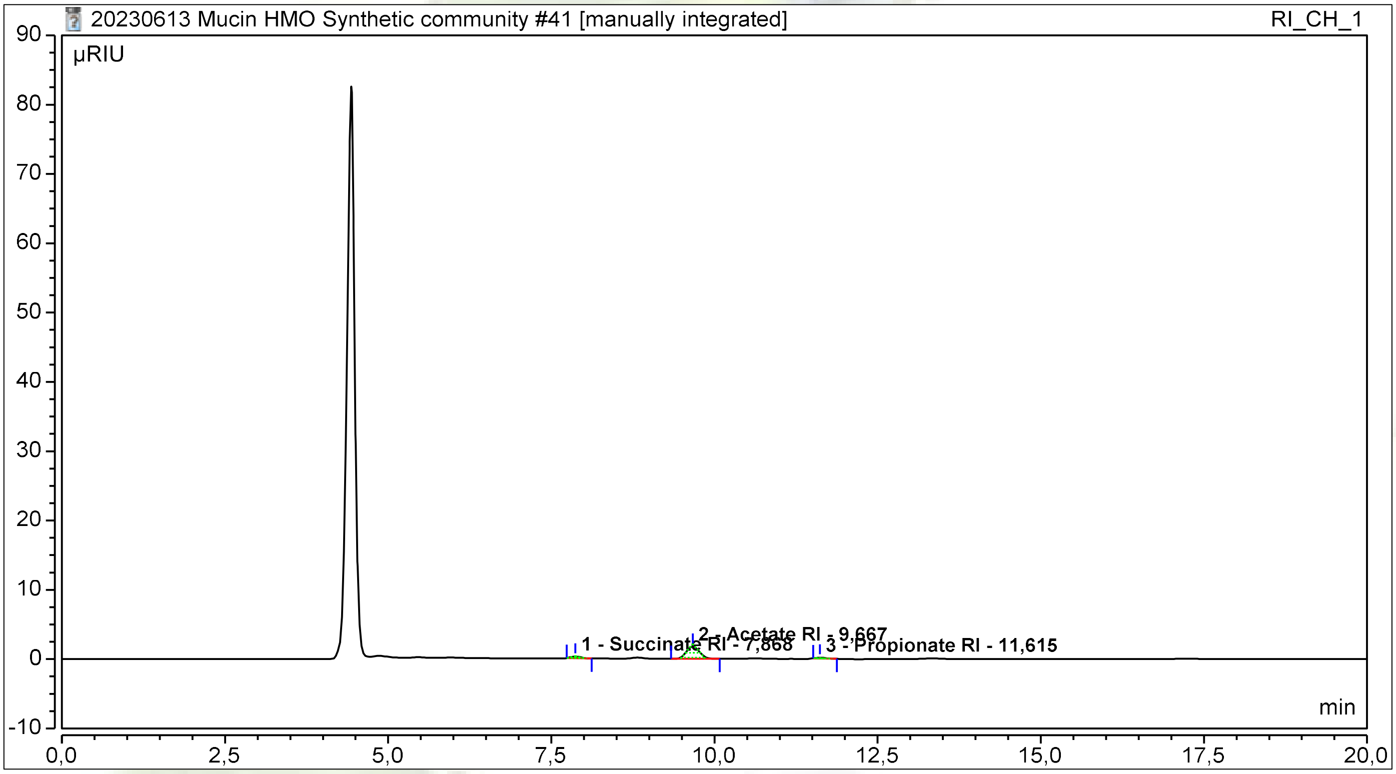

Peak Results

No.	Peak Name	Retention Time min	Width (50%) min	Type	Resolution (EP)	Asymmetry (EP)	Plates (EP)
n.a.	GlcNAc	n.a.	n.a.	n.a.	n.a.	n.a.	n.a.
n.a.	Citrate	n.a.	n.a.	n.a.	n.a.	n.a.	n.a.
n.a.	Glucose	n.a.	n.a.	n.a.	n.a.	n.a.	n.a.
n.a.	Galactose	n.a.	n.a.	n.a.	n.a.	n.a.	n.a.
n.a.	Fucose	n.a.	n.a.	n.a.	n.a.	n.a.	n.a.
1	Succinate RI	7,868	0,180	BMB*	5,24	1,24	10595
n.a.	Lactate RI	n.a.	n.a.	n.a.	n.a.	n.a.	n.a.
n.a.	glycerol	n.a.	n.a.	n.a.	n.a.	n.a.	n.a.
n.a.	Formate RI	n.a.	n.a.	n.a.	n.a.	n.a.	n.a.
2	Acetate RI	9,667	0,225	BMB	5,59	1,07	10195
n.a.	1,2 PDO RI	n.a.	n.a.	n.a.	n.a.	n.a.	n.a.
n.a.	1,3-PDO	n.a.	n.a.	n.a.	n.a.	n.a.	n.a.
3	Propionate RI	11,615	0,186	BMB*	n.a.	1,54	21642
n.a.	1,3-PDO	n.a.	n.a.	n.a.	n.a.	n.a.	n.a.
n.a.	2-3 BDO	n.a.	n.a.	n.a.	n.a.	n.a.	n.a.
n.a.	Ethanol	n.a.	n.a.	n.a.	n.a.	n.a.	n.a.
n.a.	Isobutyrate RI	n.a.	n.a.	n.a.	n.a.	n.a.	n.a.
n.a.	Butyrate RI	n.a.	n.a.	n.a.	n.a.	n.a.	n.a.

Chromatogram and SST Results

Injection Details

Injection Name:	**1 MUCHMO1 t48 r1**	*Run Time (min):*	**20,00**
Vial Number:	**3:C10**	*Injection Volume:*	**20,00**
Injection Type:	**Unknown**	*Channel:*	**RI_CH_1**
Calibration Level:		*Wavelength:*	**n.a.**
Instrument Method:	**Default method LC2030C 45 gr 20 min**	*Bandwidth:*	**n.a.**
Processing Method:	**Processing Method LC2030 45 gr**	*Dilution Factor:*	**1,0000**
Injection Date/Time:	**14-jun-23 01:34**	*Sample Weight:*	**1,0000**

Chromatogram

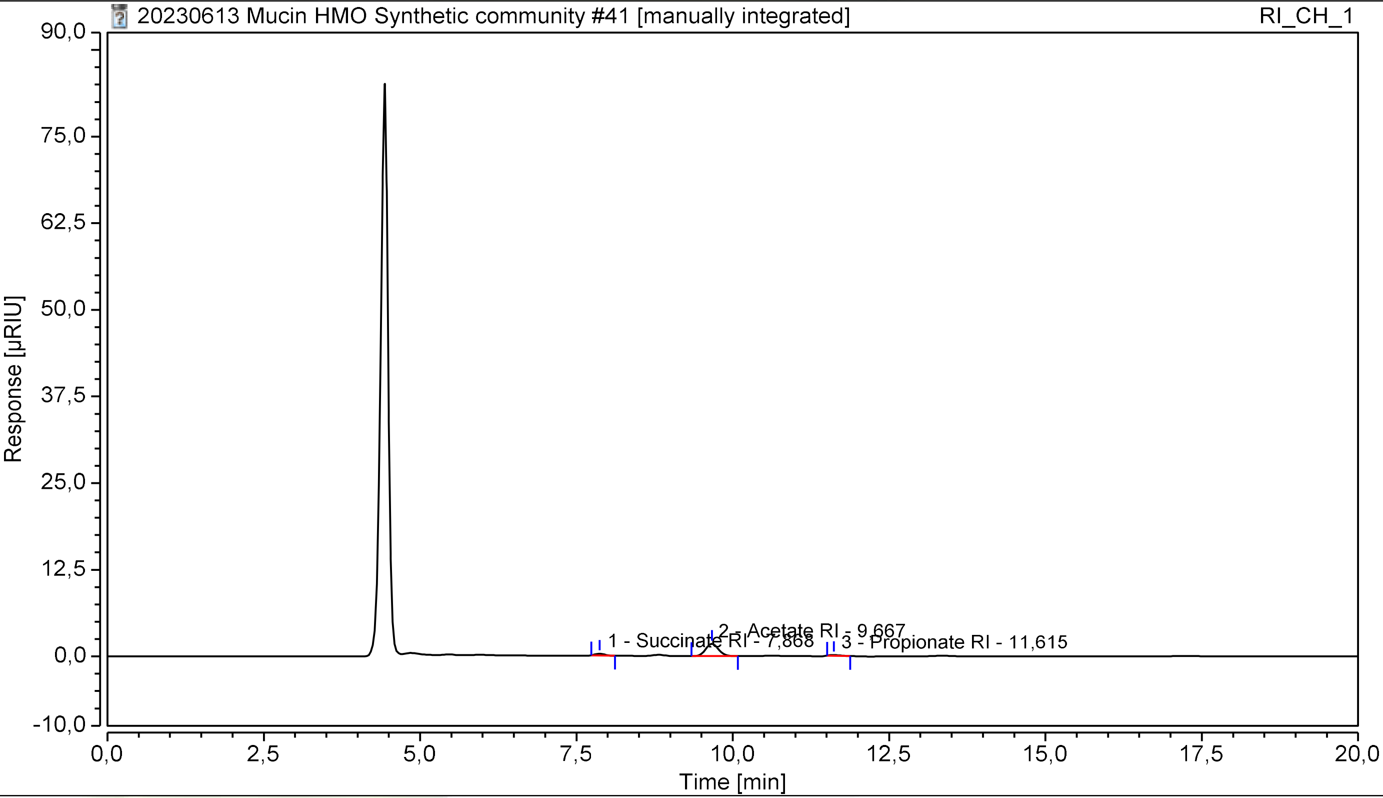

SST Results

No.	Name	Inj.Condition	Peak	Test Result	Injection

Number of executed test cases: **n.a.** **Total Result:** **Passed**

Chromatogram

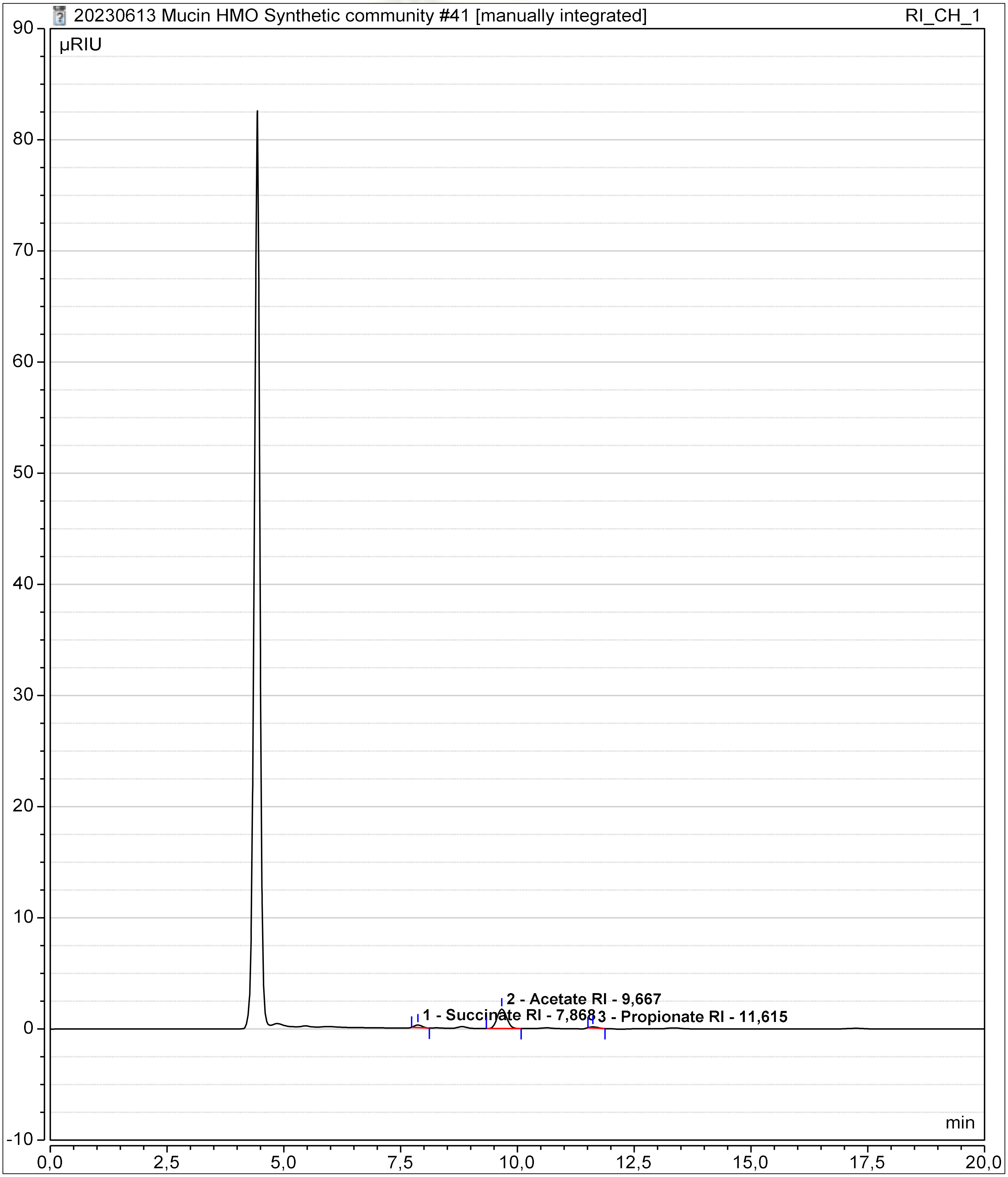

Chromeleon (c) Dionex
Version 7.2.10.25868

Chromatogram and Results

Injection Details

Injection Name:	**2 MUCHMO1 t48 r2**	*Run Time (min):*	**20,00**
Vial Number:	**3:C11**	*Injection Volume:*	**20,00**
Injection Type:	**Unknown**	*Channel:*	**RI_CH_1**
Calibration Level:		*Wavelength:*	**n.a.**
Instrument Method:	**Default method LC2030C 45 gr 20 min**	*Bandwidth:*	**n.a.**
Processing Method:	**Processing Method LC2030 45 gr**	*Dilution Factor:*	**1,0000**
Injection Date/Time:	**14-jun-23 01:54**	*Sample Weight:*	**1,0000**

Chromatogram

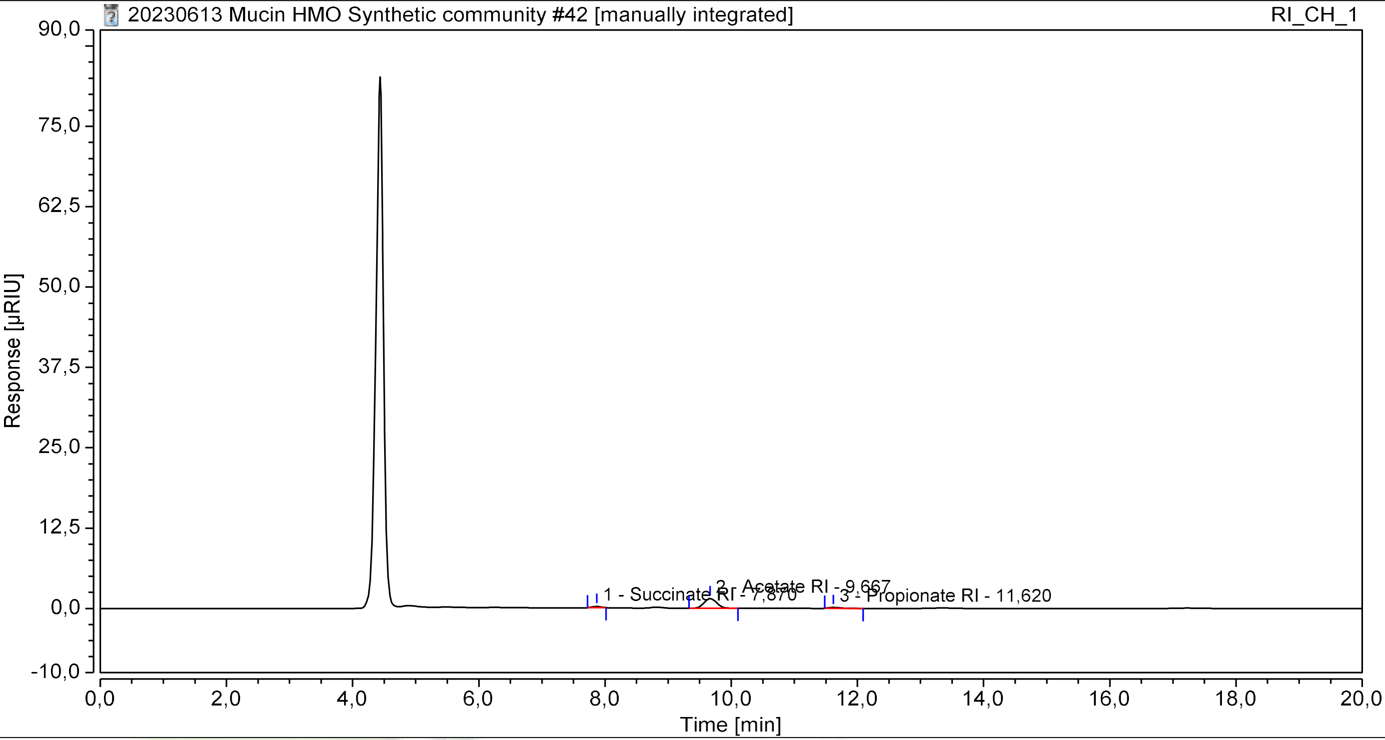

Integration Results

No.	Peak Name	Retention Time min	Area µRIU*min	Height µRIU	Relative Area %	Relative Height %	Amount
n.a.	GlcNAc	n.a.	n.a.	n.a.	n.a.	n.a.	n.a.
n.a.	Citrate	n.a.	n.a.	n.a.	n.a.	n.a.	n.a.
n.a.	Glucose	n.a.	n.a.	n.a.	n.a.	n.a.	n.a.
n.a.	Galactose	n.a.	n.a.	n.a.	n.a.	n.a.	n.a.
n.a.	Fucose	n.a.	n.a.	n.a.	n.a.	n.a.	n.a.
1	Succinate RI	7,870	0,033	0,193	7,74	10,56	n.a.
n.a.	Lactate RI	n.a.	n.a.	n.a.	n.a.	n.a.	n.a.
n.a.	glycerol	n.a.	n.a.	n.a.	n.a.	n.a.	n.a.
n.a.	Formate RI	n.a.	n.a.	n.a.	n.a.	n.a.	n.a.
2	Acetate RI	9,667	0,358	1,506	84,90	82,37	22,0151
n.a.	1,2 PDO RI	n.a.	n.a.	n.a.	n.a.	n.a.	n.a.
n.a.	1,3-PDO	n.a.	n.a.	n.a.	n.a.	n.a.	n.a.
3	Propionate RI	11,620	0,031	0,129	7,37	7,07	1,2485
n.a.	1,3-PDO	n.a.	n.a.	n.a.	n.a.	n.a.	n.a.
n.a.	2-3 BDO	n.a.	n.a.	n.a.	n.a.	n.a.	n.a.
n.a.	Ethanol	n.a.	n.a.	n.a.	n.a.	n.a.	n.a.
n.a.	Isobutyrate RI	n.a.	n.a.	n.a.	n.a.	n.a.	n.a.
n.a.	Butyrate RI	n.a.	n.a.	n.a.	n.a.	n.a.	n.a.
**Total:**			**0,421**	**1,829**	**100,00**	**100,00**	

Peak Analysis

Injection Details

Injection Name:	**2 MUCHMO1 t48 r2**	*Run Time (min):*	**20,00**
Vial Number:	**3:C11**	*Injection Volume:*	**20,00**
Injection Type:	**Unknown**	*Channel:*	**RI_CH_1**
Calibration Level:		*Wavelength:*	**n.a.**
Instrument Method:	**Default method LC2030C 45 gr 20 min**	*Bandwidth:*	**n.a.**
Processing Method:	**Processing Method LC2030 45 gr**	*Dilution Factor:*	**1,0000**
Injection Date/Time:	**14-jun-23 01:54**	*Sample Weight:*	**1,0000**

Chromatogram

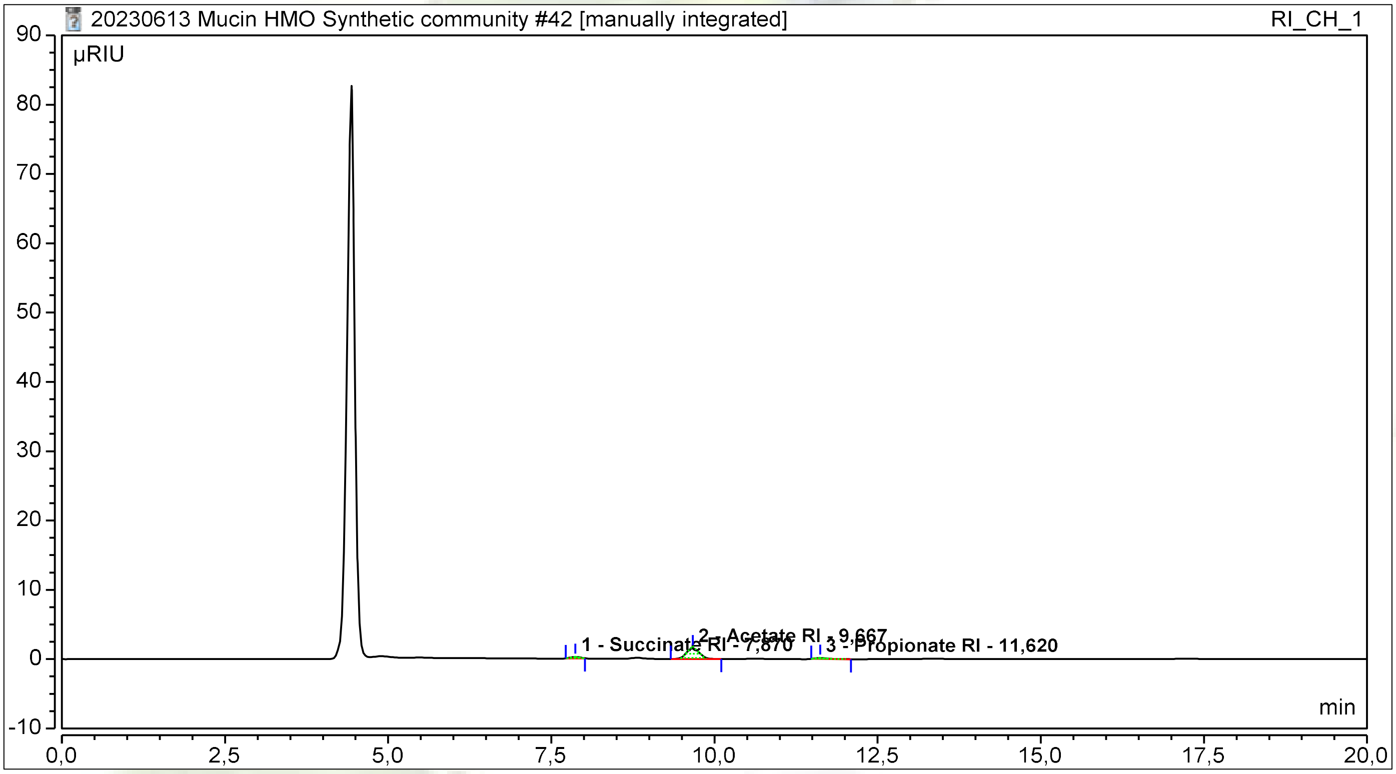

Peak Results

No.	Peak Name	Retention Time min	Width (50%) min	Type	Resolution (EP)	Asymmetry (EP)	Plates (EP)
n.a.	GlcNAc	n.a.	n.a.	n.a.	n.a.	n.a.	n.a.
n.a.	Citrate	n.a.	n.a.	n.a.	n.a.	n.a.	n.a.
n.a.	Glucose	n.a.	n.a.	n.a.	n.a.	n.a.	n.a.
n.a.	Galactose	n.a.	n.a.	n.a.	n.a.	n.a.	n.a.
n.a.	Fucose	n.a.	n.a.	n.a.	n.a.	n.a.	n.a.
1	Succinate RI	7,870	0,171	BMB*	5,36	0,99	11765
n.a.	Lactate RI	n.a.	n.a.	n.a.	n.a.	n.a.	n.a.
n.a.	glycerol	n.a.	n.a.	n.a.	n.a.	n.a.	n.a.
n.a.	Formate RI	n.a.	n.a.	n.a.	n.a.	n.a.	n.a.
2	Acetate RI	9,667	0,225	BMB	5,27	1,08	10223
n.a.	1,2 PDO RI	n.a.	n.a.	n.a.	n.a.	n.a.	n.a.
n.a.	1,3-PDO	n.a.	n.a.	n.a.	n.a.	n.a.	n.a.
3	Propionate RI	11,620	0,212	BMB*	n.a.	2,15	16571
n.a.	1,3-PDO	n.a.	n.a.	n.a.	n.a.	n.a.	n.a.
n.a.	2-3 BDO	n.a.	n.a.	n.a.	n.a.	n.a.	n.a.
n.a.	Ethanol	n.a.	n.a.	n.a.	n.a.	n.a.	n.a.
n.a.	Isobutyrate RI	n.a.	n.a.	n.a.	n.a.	n.a.	n.a.
n.a.	Butyrate RI	n.a.	n.a.	n.a.	n.a.	n.a.	n.a.

Chromatogram and SST Results

Injection Details

Injection Name:	**2 MUCHMO1 t48 r2**	*Run Time (min):*	**20,00**
Vial Number:	**3:C11**	*Injection Volume:*	**20,00**
Injection Type:	**Unknown**	*Channel:*	**RI_CH_1**
Calibration Level:		*Wavelength:*	**n.a.**
Instrument Method:	**Default method LC2030C 45 gr 20 min**	*Bandwidth:*	**n.a.**
Processing Method:	**Processing Method LC2030 45 gr**	*Dilution Factor:*	**1,0000**
Injection Date/Time:	**14-jun-23 01:54**	*Sample Weight:*	**1,0000**

Chromatogram

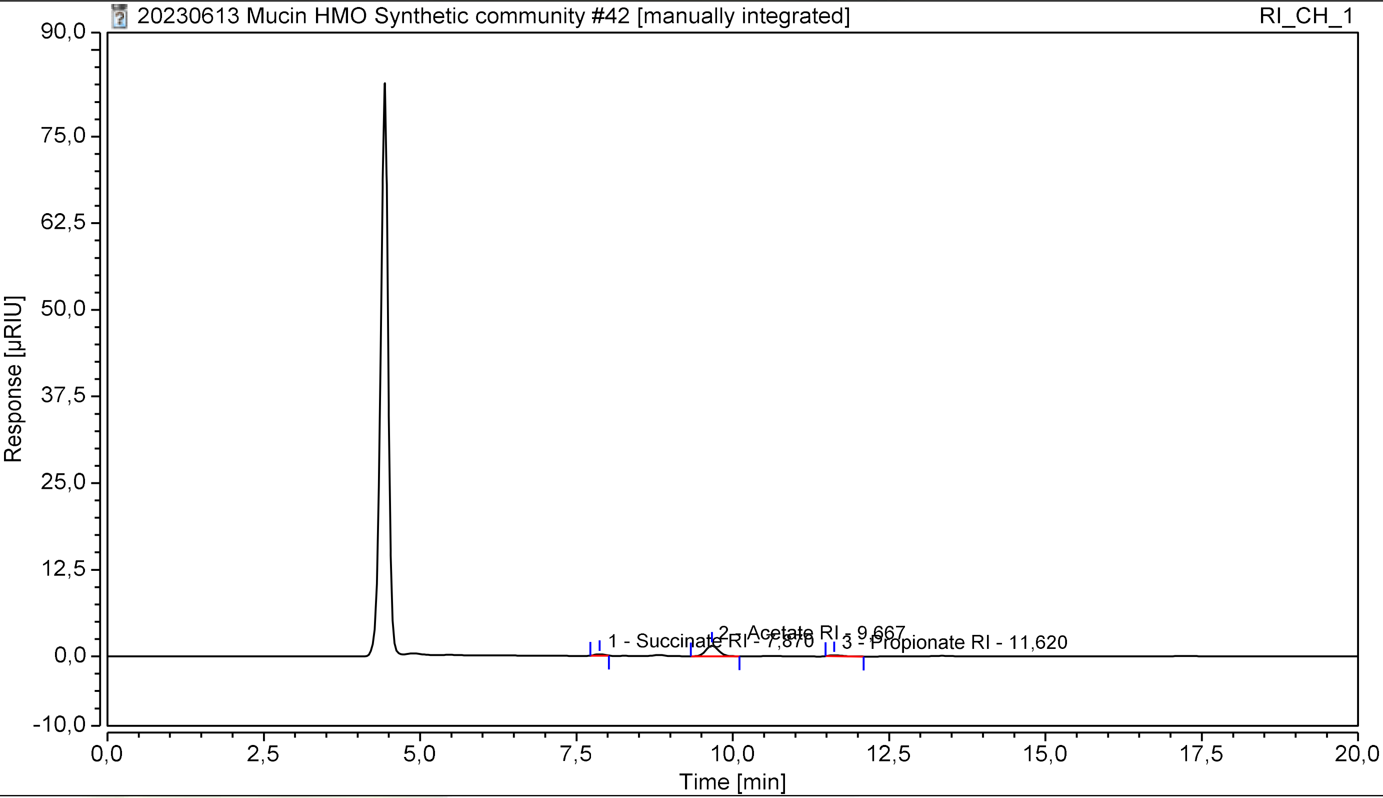

SST Results

No.	Name	Inj.Condition	Peak	Test Result	Injection

Number of executed test cases: **n.a.** **Total Result:** **Passed**

Chromatogram

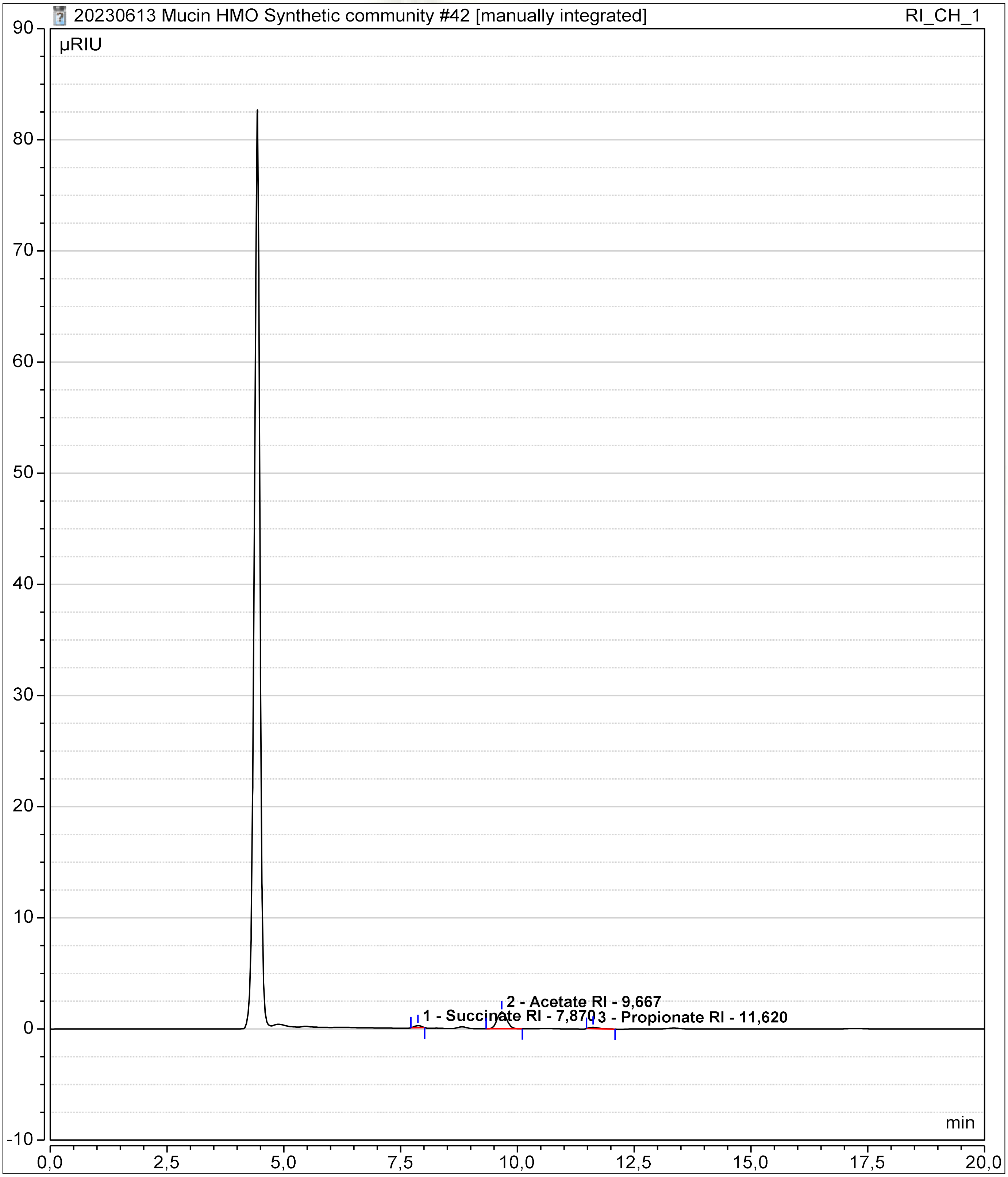

Chromeleon (c) Dionex
Version 7.2.10.25868

Chromatogram and Results

Injection Details

Injection Name:	**3 MUCHMO1 t48 r3**	*Run Time (min):*	**20,00**
Vial Number:	**3:C12**	*Injection Volume:*	**20,00**
Injection Type:	**Unknown**	*Channel:*	**RI_CH_1**
Calibration Level:		*Wavelength:*	**n.a.**
Instrument Method:	**Default method LC2030C 45 gr 20 min**	*Bandwidth:*	**n.a.**
Processing Method:	**Processing Method LC2030 45 gr**	*Dilution Factor:*	**1,0000**
Injection Date/Time:	**14-jun-23 02:15**	*Sample Weight:*	**1,0000**

Chromatogram

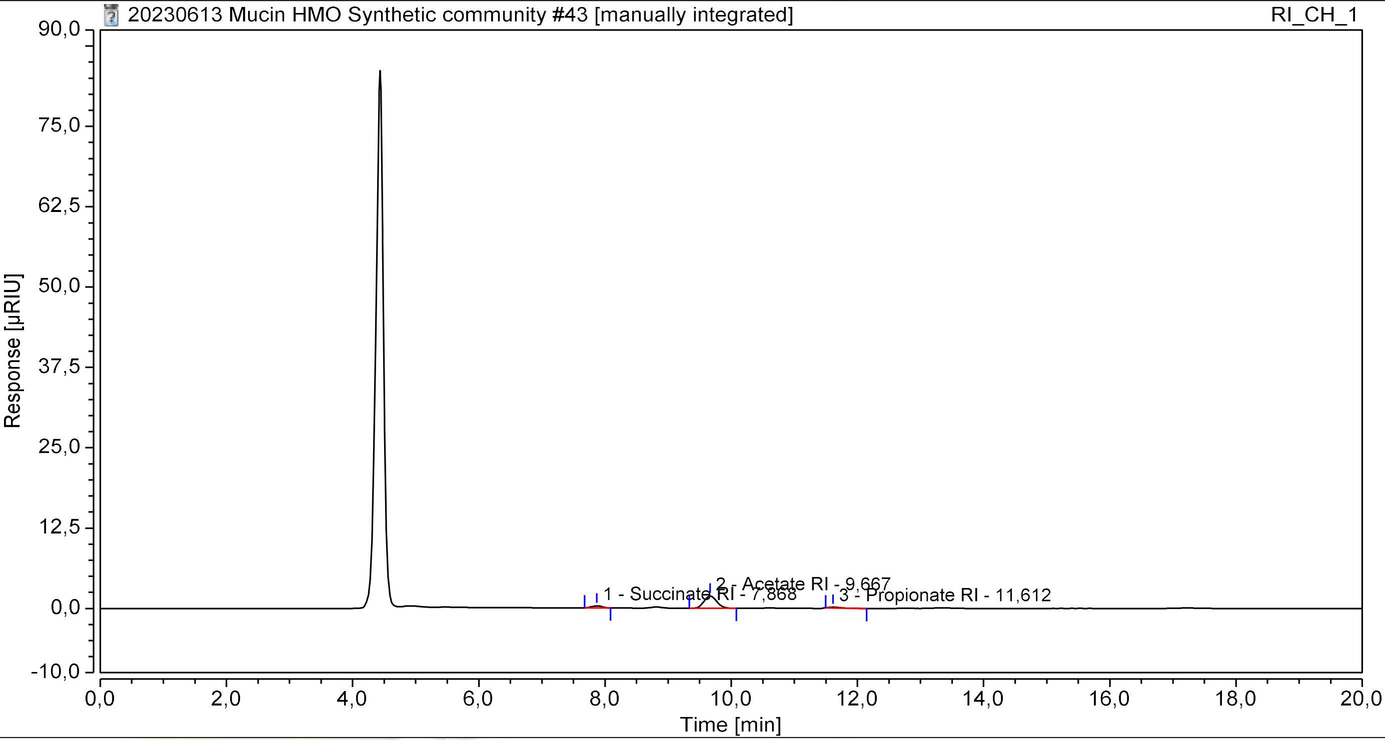

Integration Results

No.	Peak Name	Retention Time min	Area µRIU*min	Height µRIU	Relative Area %	Relative Height %	Amount
n.a.	GlcNAc	n.a.	n.a.	n.a.	n.a.	n.a.	n.a.
n.a.	Citrate	n.a.	n.a.	n.a.	n.a.	n.a.	n.a.
n.a.	Glucose	n.a.	n.a.	n.a.	n.a.	n.a.	n.a.
n.a.	Galactose	n.a.	n.a.	n.a.	n.a.	n.a.	n.a.
n.a.	Fucose	n.a.	n.a.	n.a.	n.a.	n.a.	n.a.
1	Succinate RI	7,868	0,063	0,315	11,65	13,47	n.a.
n.a.	Lactate RI	n.a.	n.a.	n.a.	n.a.	n.a.	n.a.
n.a.	glycerol	n.a.	n.a.	n.a.	n.a.	n.a.	n.a.
n.a.	Formate RI	n.a.	n.a.	n.a.	n.a.	n.a.	n.a.
2	Acetate RI	9,667	0,450	1,893	83,38	80,88	27,7045
n.a.	1,2 PDO RI	n.a.	n.a.	n.a.	n.a.	n.a.	n.a.
n.a.	1,3-PDO	n.a.	n.a.	n.a.	n.a.	n.a.	n.a.
3	Propionate RI	11,612	0,027	0,132	4,97	5,65	1,0791
n.a.	1,3-PDO	n.a.	n.a.	n.a.	n.a.	n.a.	n.a.
n.a.	2-3 BDO	n.a.	n.a.	n.a.	n.a.	n.a.	n.a.
n.a.	Ethanol	n.a.	n.a.	n.a.	n.a.	n.a.	n.a.
n.a.	Isobutyrate RI	n.a.	n.a.	n.a.	n.a.	n.a.	n.a.
n.a.	Butyrate RI	n.a.	n.a.	n.a.	n.a.	n.a.	n.a.
**Total:**			**0,540**	**2,341**	**100,00**	**100,00**	

Chromeleon (c) Dionex
Version 7.2.10.25868

Peak Analysis

Injection Details

Injection Name:	**3 MUCHMO1 t48 r3**	*Run Time (min):*	**20,00**
Vial Number:	**3:C12**	*Injection Volume:*	**20,00**
Injection Type:	**Unknown**	*Channel:*	**RI_CH_1**
Calibration Level:		*Wavelength:*	**n.a.**
Instrument Method:	**Default method LC2030C 45 gr 20 min**	*Bandwidth:*	**n.a.**
Processing Method:	**Processing Method LC2030 45 gr**	*Dilution Factor:*	**1,0000**
Injection Date/Time:	**14-jun-23 02:15**	*Sample Weight:*	**1,0000**

Chromatogram

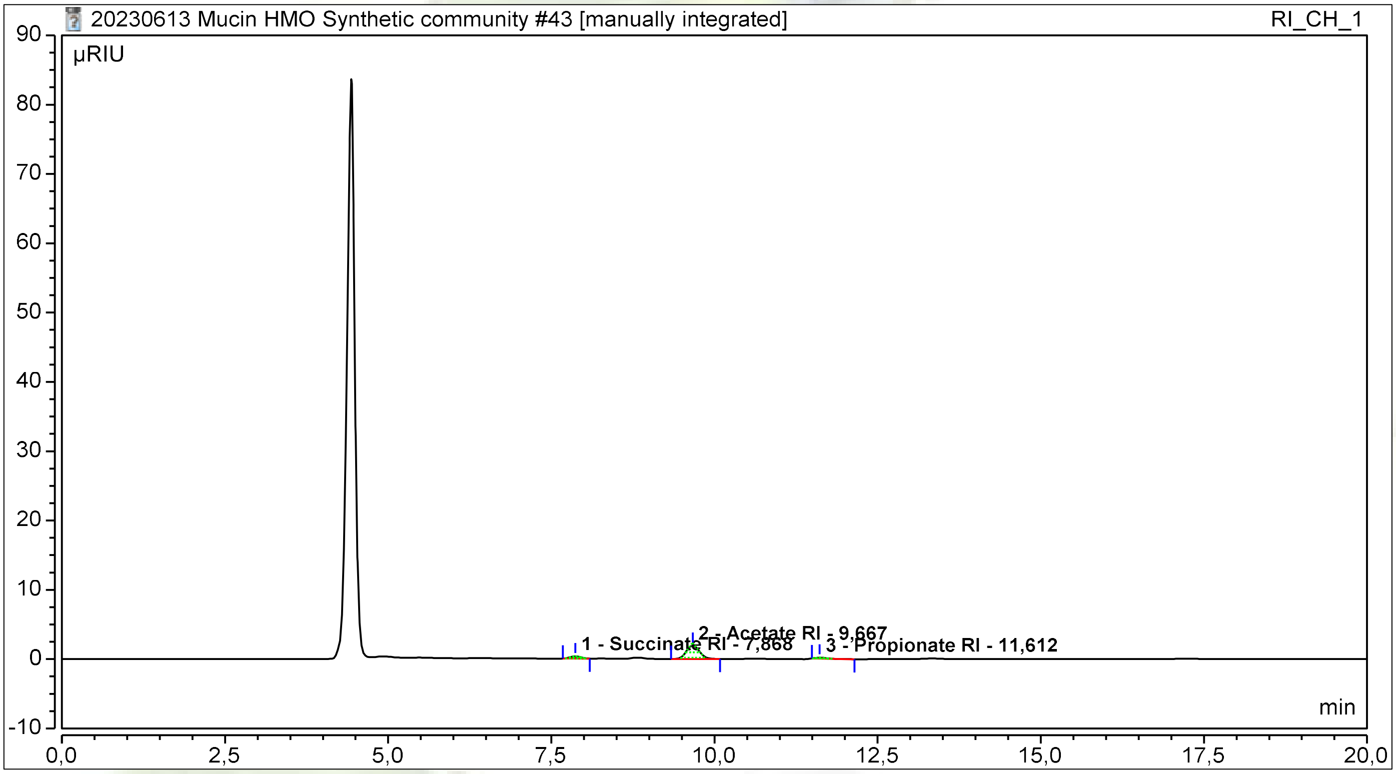

Peak Results

No.	Peak Name	Retention Time min	Width (50%) min	Type	Resolution (EP)	Asymmetry (EP)	Plates (EP)
n.a.	GlcNAc	n.a.	n.a.	n.a.	n.a.	n.a.	n.a.
n.a.	Citrate	n.a.	n.a.	n.a.	n.a.	n.a.	n.a.
n.a.	Glucose	n.a.	n.a.	n.a.	n.a.	n.a.	n.a.
n.a.	Galactose	n.a.	n.a.	n.a.	n.a.	n.a.	n.a.
n.a.	Fucose	n.a.	n.a.	n.a.	n.a.	n.a.	n.a.
1	Succinate RI	7,868	0,194	BMB*	5,06	1,07	9071
n.a.	Lactate RI	n.a.	n.a.	n.a.	n.a.	n.a.	n.a.
n.a.	glycerol	n.a.	n.a.	n.a.	n.a.	n.a.	n.a.
n.a.	Formate RI	n.a.	n.a.	n.a.	n.a.	n.a.	n.a.
2	Acetate RI	9,667	0,225	BMB	5,49	1,08	10202
n.a.	1,2 PDO RI	n.a.	n.a.	n.a.	n.a.	n.a.	n.a.
n.a.	1,3-PDO	n.a.	n.a.	n.a.	n.a.	n.a.	n.a.
3	Propionate RI	11,612	0,193	BMB*	n.a.	1,46	20131
n.a.	1,3-PDO	n.a.	n.a.	n.a.	n.a.	n.a.	n.a.
n.a.	2-3 BDO	n.a.	n.a.	n.a.	n.a.	n.a.	n.a.
n.a.	Ethanol	n.a.	n.a.	n.a.	n.a.	n.a.	n.a.
n.a.	Isobutyrate RI	n.a.	n.a.	n.a.	n.a.	n.a.	n.a.
n.a.	Butyrate RI	n.a.	n.a.	n.a.	n.a.	n.a.	n.a.

Chromeleon (c) Dionex
Version 7.2.10.25868

Chromatogram and SST Results

Injection Details

Injection Name:	**3 MUCHMO1 t48 r3**	*Run Time (min):*	**20,00**
Vial Number:	**3:C12**	*Injection Volume:*	**20,00**
Injection Type:	**Unknown**	*Channel:*	**RI_CH_1**
Calibration Level:		*Wavelength:*	**n.a.**
Instrument Method:	**Default method LC2030C 45 gr 20 min**	*Bandwidth:*	**n.a.**
Processing Method:	**Processing Method LC2030 45 gr**	*Dilution Factor:*	**1,0000**
Injection Date/Time:	**14-jun-23 02:15**	*Sample Weight:*	**1,0000**

Chromatogram

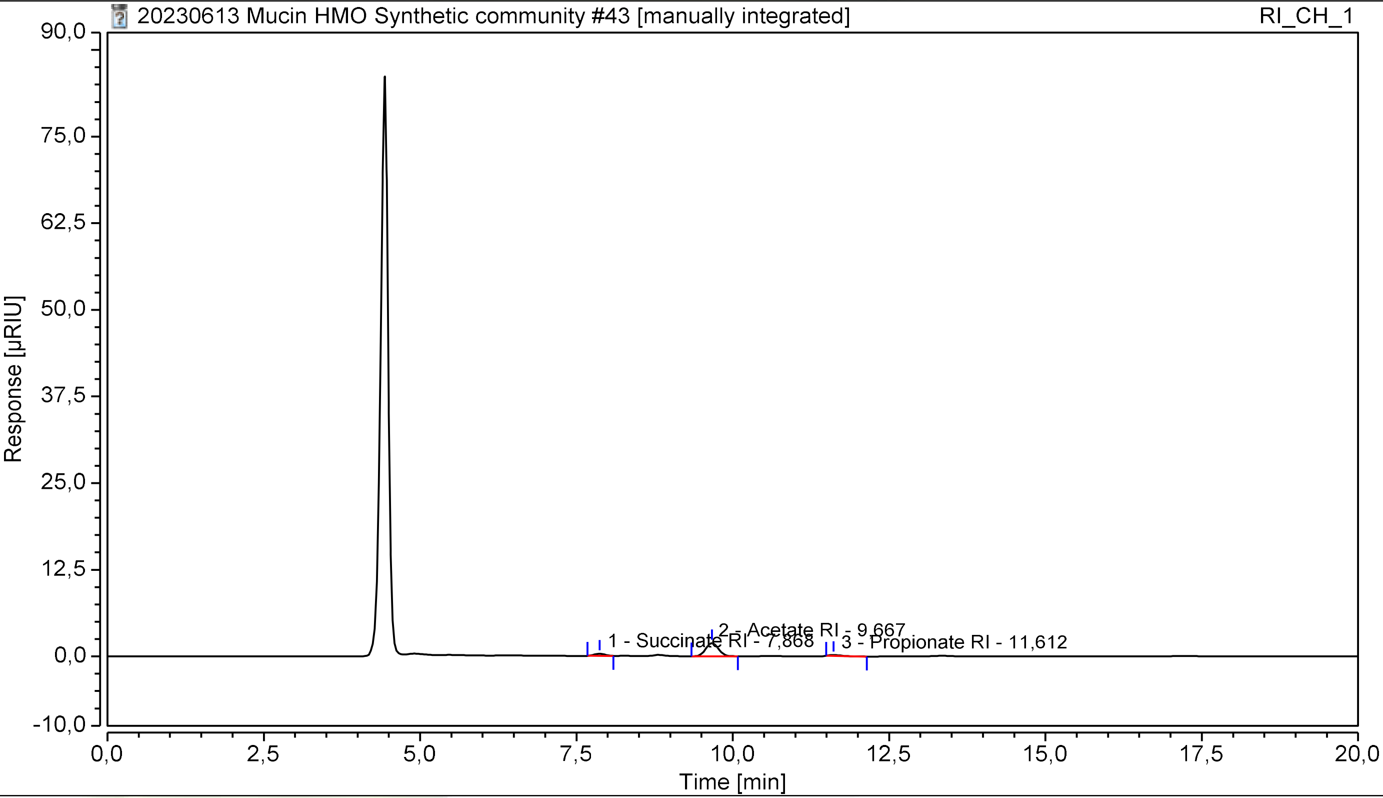

SST Results

No.	Name	Inj.Condition	Peak	Test Result	Injection

Number of executed test cases: **n.a.** **Total Result:** **Passed**

Chromeleon (c) Dionex
Version 7.2.10.25868

Chromatogram

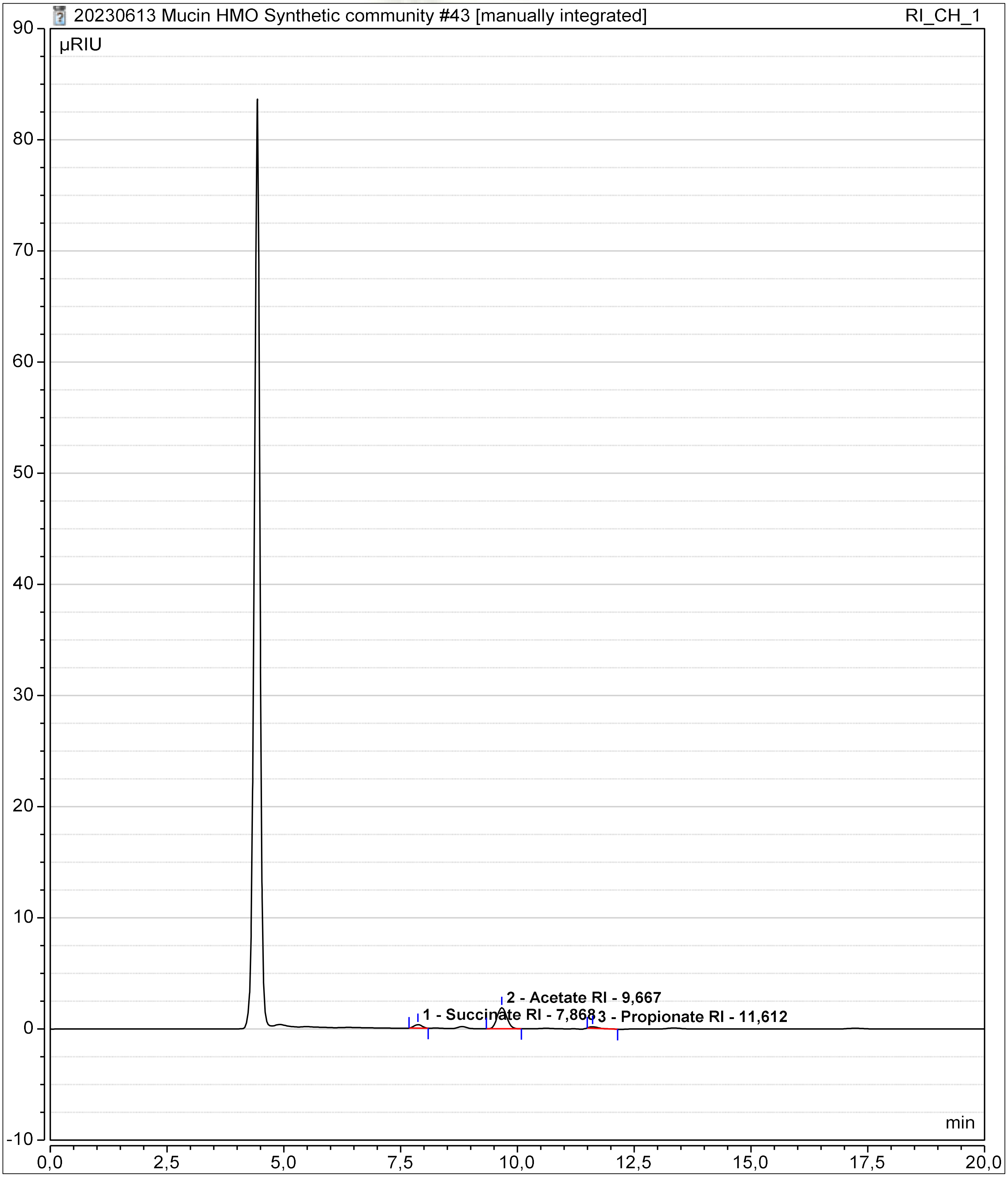

Chromeleon (c) Dionex
Version 7.2.10.25868

Chromatogram and Results

Injection Details

Injection Name:	**4 MUCHMO2 t24 r1**	*Run Time (min):*	**20,00**
Vial Number:	**3:D1**	*Injection Volume:*	**20,00**
Injection Type:	**Unknown**	*Channel:*	**RI_CH_1**
Calibration Level:		*Wavelength:*	**n.a.**
Instrument Method:	**Default method LC2030C 45 gr 20 min**	*Bandwidth:*	**n.a.**
Processing Method:	**Processing Method LC2030 45 gr**	*Dilution Factor:*	**1,0000**
Injection Date/Time:	**14-jun-23 02:35**	*Sample Weight:*	**1,0000**

Chromatogram

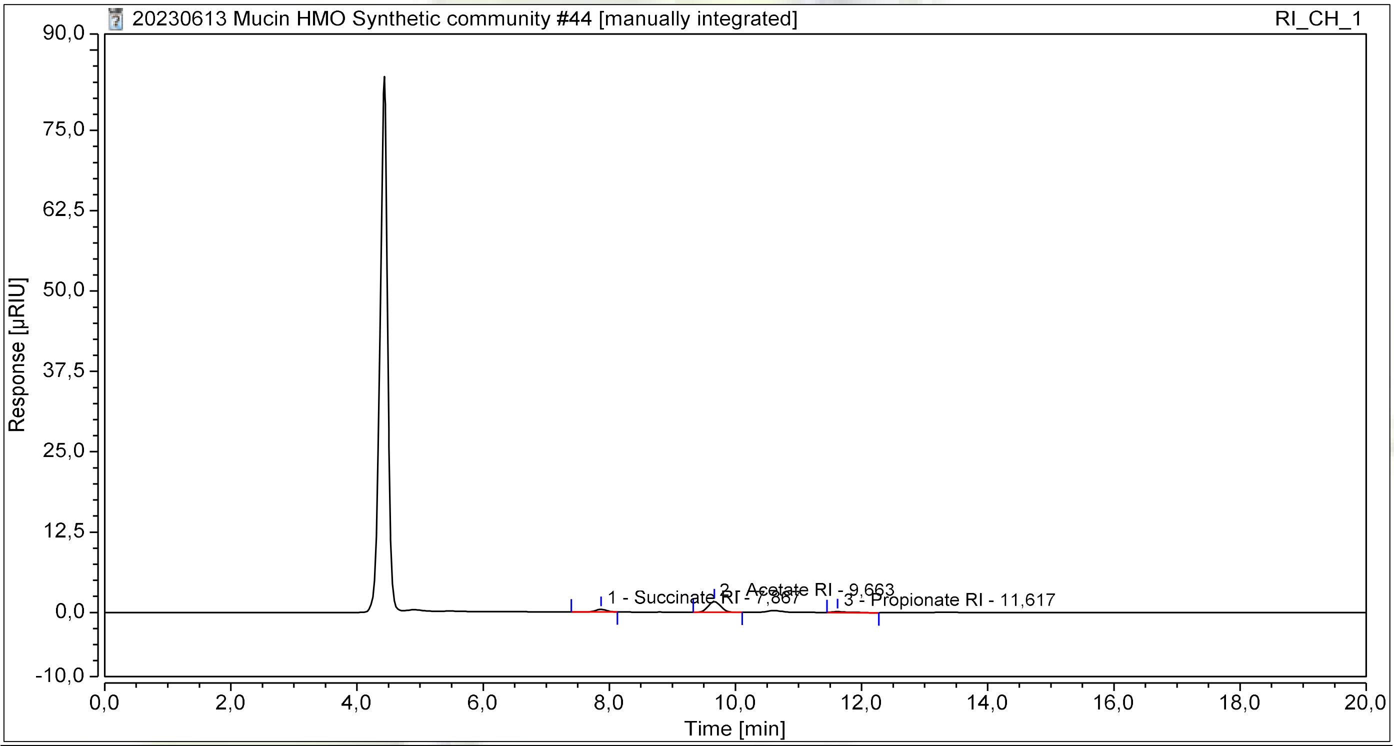

Integration Results

No.	Peak Name	Retention Time min	Area µRIU*min	Height µRIU	Relative Area %	Relative Height %	Amount
n.a.	GlcNAc	n.a.	n.a.	n.a.	n.a.	n.a.	n.a.
n.a.	Citrate	n.a.	n.a.	n.a.	n.a.	n.a.	n.a.
n.a.	Glucose	n.a.	n.a.	n.a.	n.a.	n.a.	n.a.
n.a.	Galactose	n.a.	n.a.	n.a.	n.a.	n.a.	n.a.
n.a.	Fucose	n.a.	n.a.	n.a.	n.a.	n.a.	n.a.
1	Succinate RI	7,867	0,085	0,411	16,52	18,83	n.a.
n.a.	Lactate RI	n.a.	n.a.	n.a.	n.a.	n.a.	n.a.
n.a.	glycerol	n.a.	n.a.	n.a.	n.a.	n.a.	n.a.
n.a.	Formate RI	n.a.	n.a.	n.a.	n.a.	n.a.	n.a.
2	Acetate RI	9,663	0,390	1,643	75,73	75,26	24,0275
n.a.	1,2 PDO RI	n.a.	n.a.	n.a.	n.a.	n.a.	n.a.
n.a.	1,3-PDO	n.a.	n.a.	n.a.	n.a.	n.a.	n.a.
3	Propionate RI	11,617	0,040	0,129	7,76	5,91	1,6088
n.a.	1,3-PDO	n.a.	n.a.	n.a.	n.a.	n.a.	n.a.
n.a.	2-3 BDO	n.a.	n.a.	n.a.	n.a.	n.a.	n.a.
n.a.	Ethanol	n.a.	n.a.	n.a.	n.a.	n.a.	n.a.
n.a.	Isobutyrate RI	n.a.	n.a.	n.a.	n.a.	n.a.	n.a.
n.a.	Butyrate RI	n.a.	n.a.	n.a.	n.a.	n.a.	n.a.
**Total:**			**0,516**	**2,183**	**100,00**	**100,00**	

Peak Analysis

Injection Details

Injection Name:	**4 MUCHMO2 t24 r1**	*Run Time (min):*	**20,00**
Vial Number:	**3:D1**	*Injection Volume:*	**20,00**
Injection Type:	**Unknown**	*Channel:*	**RI_CH_1**
Calibration Level:		*Wavelength:*	**n.a.**
Instrument Method:	**Default method LC2030C 45 gr 20 min**	*Bandwidth:*	**n.a.**
Processing Method:	**Processing Method LC2030 45 gr**	*Dilution Factor:*	**1,0000**
Injection Date/Time:	**14-jun-23 02:35**	*Sample Weight:*	**1,0000**

Chromatogram

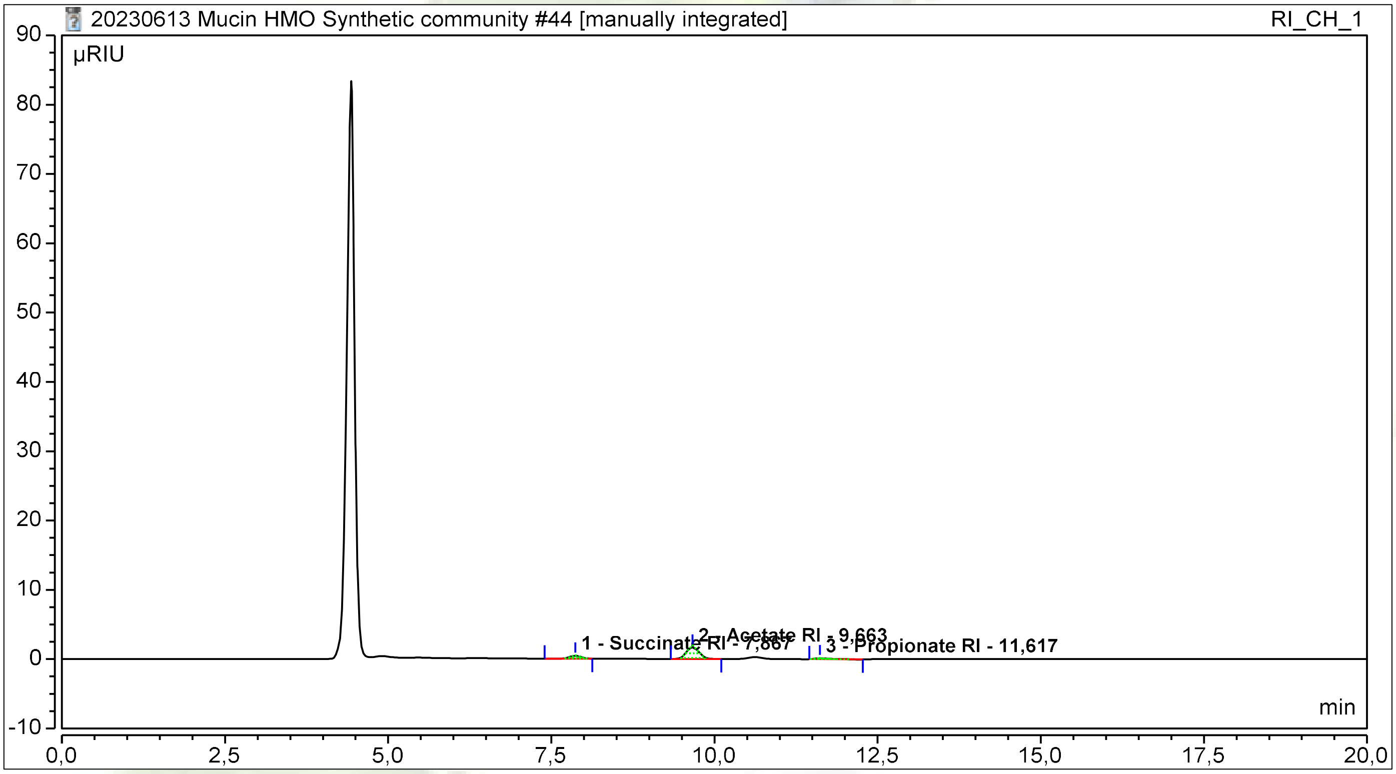

Peak Results

No.	Peak Name	Retention Time min	Width (50%) min	Type	Resolution (EP)	Asymmetry (EP)	Plates (EP)
n.a.	GlcNAc	n.a.	n.a.	n.a.	n.a.	n.a.	n.a.
n.a.	Citrate	n.a.	n.a.	n.a.	n.a.	n.a.	n.a.
n.a.	Glucose	n.a.	n.a.	n.a.	n.a.	n.a.	n.a.
n.a.	Galactose	n.a.	n.a.	n.a.	n.a.	n.a.	n.a.
n.a.	Fucose	n.a.	n.a.	n.a.	n.a.	n.a.	n.a.
1	Succinate RI	7,867	0,200	BMB*	4,99	1,07	8567
n.a.	Lactate RI	n.a.	n.a.	n.a.	n.a.	n.a.	n.a.
n.a.	glycerol	n.a.	n.a.	n.a.	n.a.	n.a.	n.a.
n.a.	Formate RI	n.a.	n.a.	n.a.	n.a.	n.a.	n.a.
2	Acetate RI	9,663	0,225	BMB	4,75	1,08	10203
n.a.	1,2 PDO RI	n.a.	n.a.	n.a.	n.a.	n.a.	n.a.
n.a.	1,3-PDO	n.a.	n.a.	n.a.	n.a.	n.a.	n.a.
3	Propionate RI	11,617	0,260	BMB*	n.a.	2,05	11053
n.a.	1,3-PDO	n.a.	n.a.	n.a.	n.a.	n.a.	n.a.
n.a.	2-3 BDO	n.a.	n.a.	n.a.	n.a.	n.a.	n.a.
n.a.	Ethanol	n.a.	n.a.	n.a.	n.a.	n.a.	n.a.
n.a.	Isobutyrate RI	n.a.	n.a.	n.a.	n.a.	n.a.	n.a.
n.a.	Butyrate RI	n.a.	n.a.	n.a.	n.a.	n.a.	n.a.

Chromeleon (c) Dionex
Version 7.2.10.25868

Chromatogram and SST Results

Injection Details

Injection Name:	**4 MUCHMO2 t24 r1**	*Run Time (min):*	**20,00**
Vial Number:	**3:D1**	*Injection Volume:*	**20,00**
Injection Type:	**Unknown**	*Channel:*	**RI_CH_1**
Calibration Level:		*Wavelength:*	**n.a.**
Instrument Method:	**Default method LC2030C 45 gr 20 min**	*Bandwidth:*	**n.a.**
Processing Method:	**Processing Method LC2030 45 gr**	*Dilution Factor:*	**1,0000**
Injection Date/Time:	**14-jun-23 02:35**	*Sample Weight:*	**1,0000**

Chromatogram

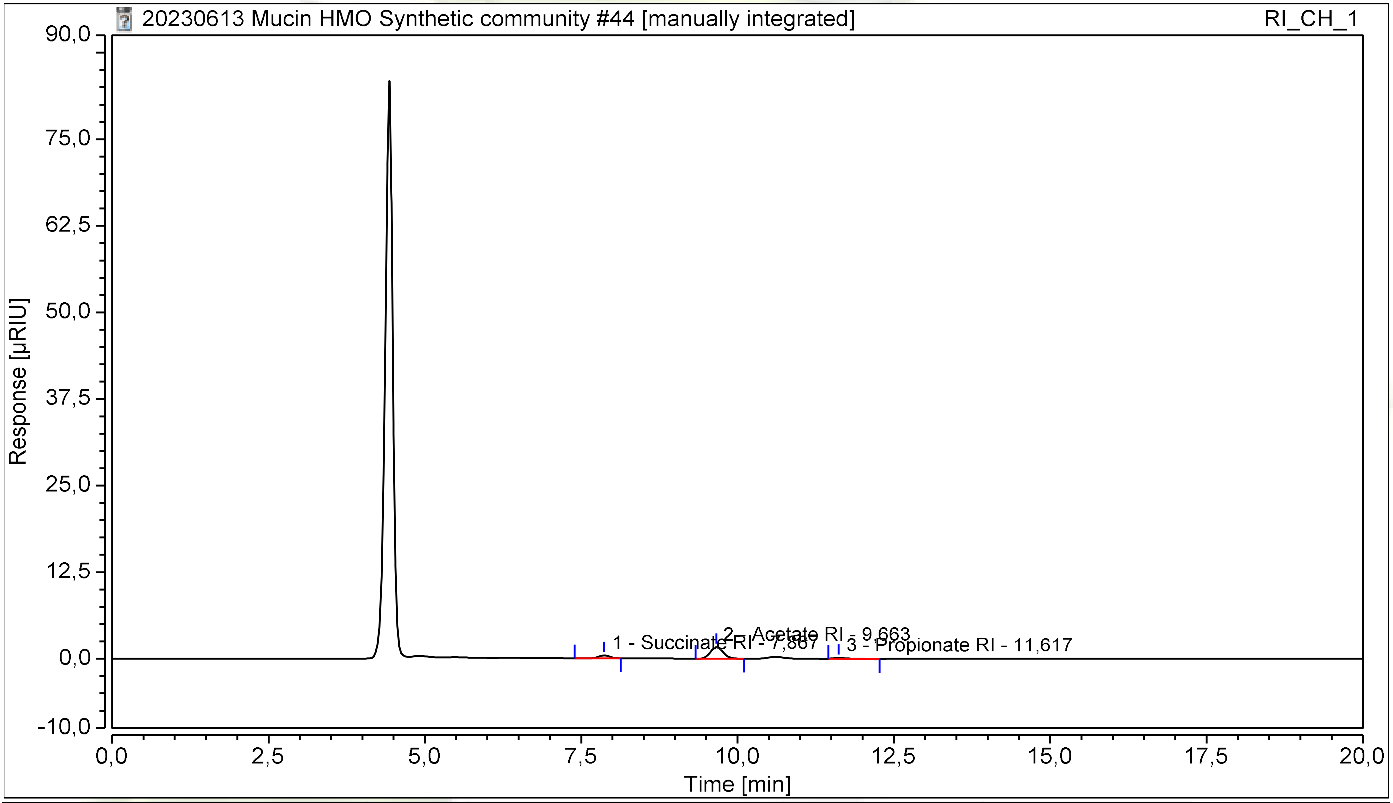

SST Results

No.	Name	Inj.Condition	Peak	Test Result	Injection

Number of executed test cases: **n.a.** **Total Result:** **Passed**

Chromatogram

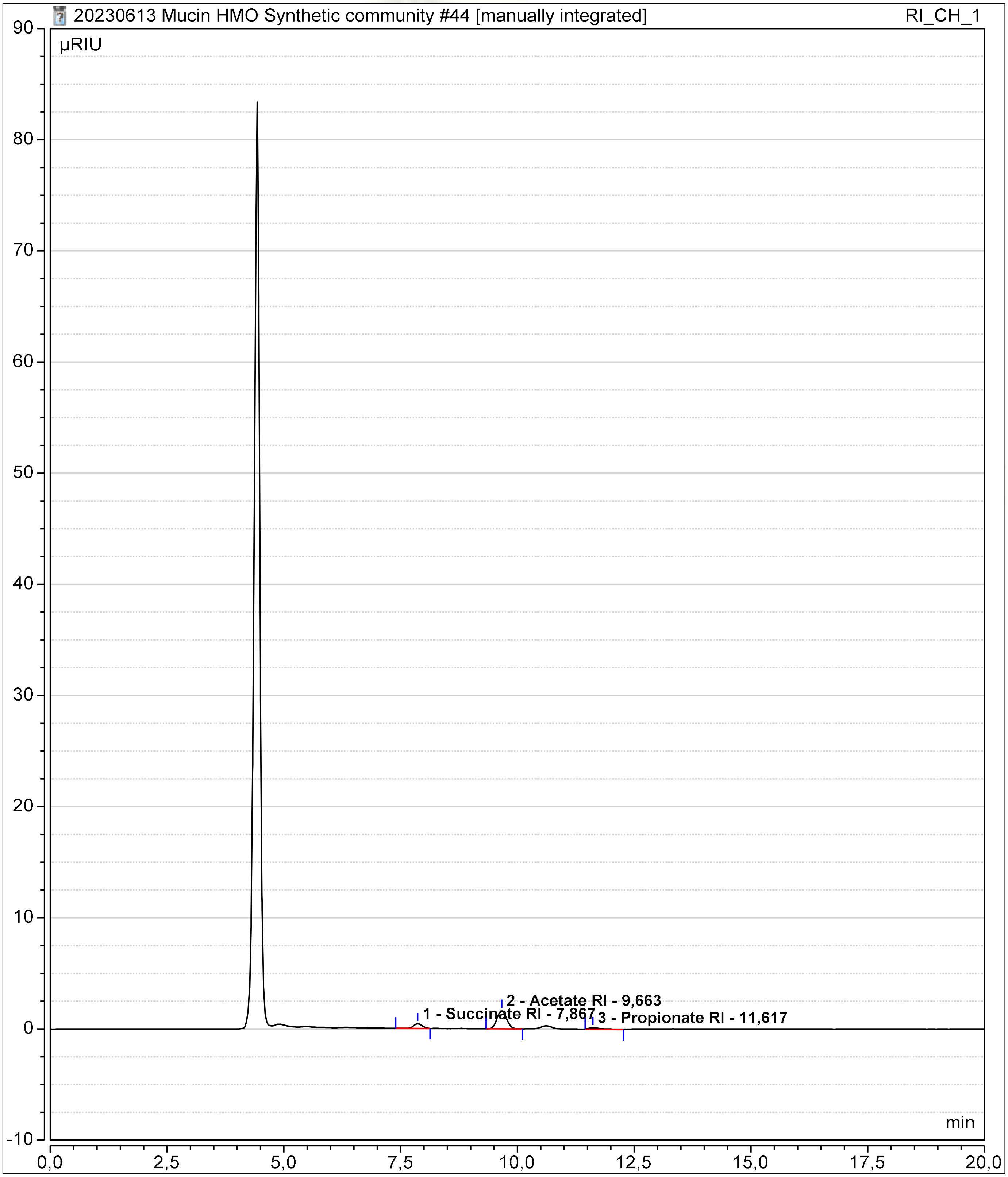

Chromeleon (c) Dionex
Version 7.2.10.25868

Chromatogram and Results

Injection Details

Injection Name:	**5 MUCHMO2 t24 r2**	*Run Time (min):*	**20,00**
Vial Number:	**3:D2**	*Injection Volume:*	**20,00**
Injection Type:	**Unknown**	*Channel:*	**RI_CH_1**
Calibration Level:		*Wavelength:*	**n.a.**
Instrument Method:	**Default method LC2030C 45 gr 20 min**	*Bandwidth:*	**n.a.**
Processing Method:	**Processing Method LC2030 45 gr**	*Dilution Factor:*	**1,0000**
Injection Date/Time:	**14-jun-23 02:55**	*Sample Weight:*	**1,0000**

Chromatogram

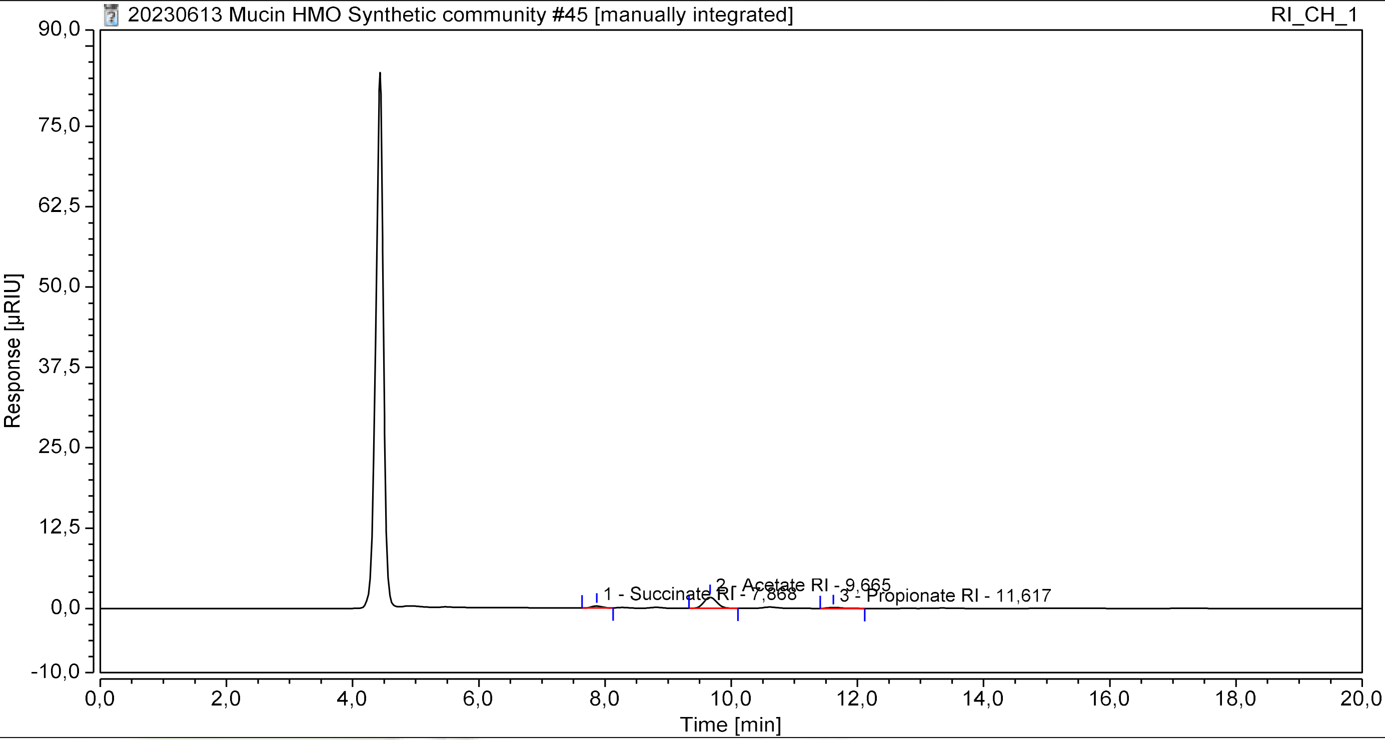

Integration Results

No.	Peak Name	Retention Time min	Area µRIU*min	Height µRIU	Relative Area %	Relative Height %	Amount
n.a.	GlcNAc	n.a.	n.a.	n.a.	n.a.	n.a.	n.a.
n.a.	Citrate	n.a.	n.a.	n.a.	n.a.	n.a.	n.a.
n.a.	Glucose	n.a.	n.a.	n.a.	n.a.	n.a.	n.a.
n.a.	Galactose	n.a.	n.a.	n.a.	n.a.	n.a.	n.a.
n.a.	Fucose	n.a.	n.a.	n.a.	n.a.	n.a.	n.a.
1	Succinate RI	7,868	0,062	0,308	11,76	13,95	n.a.
n.a.	Lactate RI	n.a.	n.a.	n.a.	n.a.	n.a.	n.a.
n.a.	glycerol	n.a.	n.a.	n.a.	n.a.	n.a.	n.a.
n.a.	Formate RI	n.a.	n.a.	n.a.	n.a.	n.a.	n.a.
2	Acetate RI	9,665	0,412	1,731	78,57	78,49	25,3543
n.a.	1,2 PDO RI	n.a.	n.a.	n.a.	n.a.	n.a.	n.a.
n.a.	1,3-PDO	n.a.	n.a.	n.a.	n.a.	n.a.	n.a.
3	Propionate RI	11,617	0,051	0,167	9,66	7,56	2,0380
n.a.	1,3-PDO	n.a.	n.a.	n.a.	n.a.	n.a.	n.a.
n.a.	2-3 BDO	n.a.	n.a.	n.a.	n.a.	n.a.	n.a.
n.a.	Ethanol	n.a.	n.a.	n.a.	n.a.	n.a.	n.a.
n.a.	Isobutyrate RI	n.a.	n.a.	n.a.	n.a.	n.a.	n.a.
n.a.	Butyrate RI	n.a.	n.a.	n.a.	n.a.	n.a.	n.a.
**Total:**			**0,524**	**2,205**	**100,00**	**100,00**	

Chromeleon (c) Dionex
Version 7.2.10.25868

Peak Analysis

Injection Details

Injection Name:	**5 MUCHMO2 t24 r2**	*Run Time (min):*	**20,00**
Vial Number:	**3:D2**	*Injection Volume:*	**20,00**
Injection Type:	**Unknown**	*Channel:*	**RI_CH_1**
Calibration Level:		*Wavelength:*	**n.a.**
Instrument Method:	**Default method LC2030C 45 gr 20 min**	*Bandwidth:*	**n.a.**
Processing Method:	**Processing Method LC2030 45 gr**	*Dilution Factor:*	**1,0000**
Injection Date/Time:	**14-jun-23 02:55**	*Sample Weight:*	**1,0000**

Chromatogram

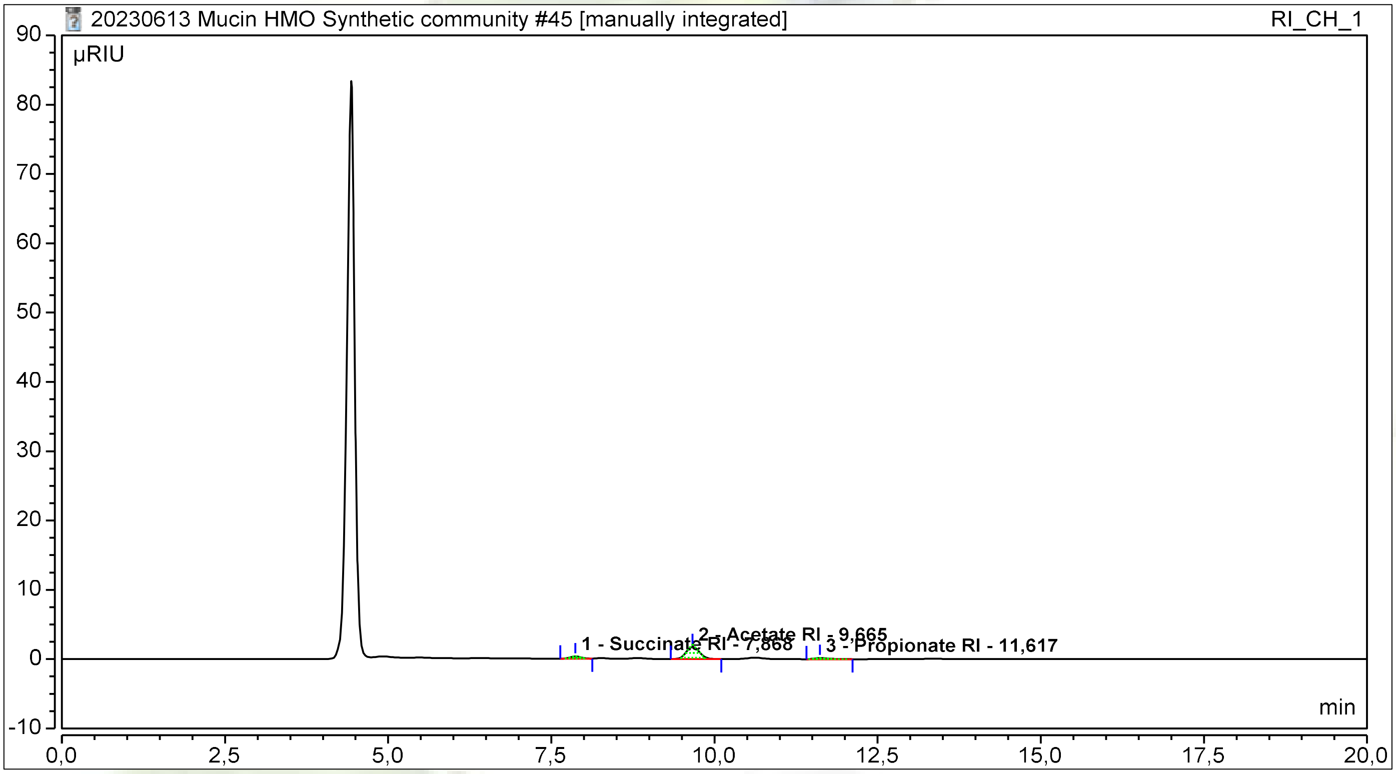

Peak Results

No.	Peak Name	Retention Time min	Width (50%) min	Type	Resolution (EP)	Asymmetry (EP)	Plates (EP)
n.a.	GlcNAc	n.a.	n.a.	n.a.	n.a.	n.a.	n.a.
n.a.	Citrate	n.a.	n.a.	n.a.	n.a.	n.a.	n.a.
n.a.	Glucose	n.a.	n.a.	n.a.	n.a.	n.a.	n.a.
n.a.	Galactose	n.a.	n.a.	n.a.	n.a.	n.a.	n.a.
n.a.	Fucose	n.a.	n.a.	n.a.	n.a.	n.a.	n.a.
1	Succinate RI	7,868	0,195	BMB*	5,05	1,01	9045
n.a.	Lactate RI	n.a.	n.a.	n.a.	n.a.	n.a.	n.a.
n.a.	glycerol	n.a.	n.a.	n.a.	n.a.	n.a.	n.a.
n.a.	Formate RI	n.a.	n.a.	n.a.	n.a.	n.a.	n.a.
2	Acetate RI	9,665	0,225	BMB	4,76	1,08	10190
n.a.	1,2 PDO RI	n.a.	n.a.	n.a.	n.a.	n.a.	n.a.
n.a.	1,3-PDO	n.a.	n.a.	n.a.	n.a.	n.a.	n.a.
3	Propionate RI	11,617	0,258	BMB*	n.a.	1,76	11224
n.a.	1,3-PDO	n.a.	n.a.	n.a.	n.a.	n.a.	n.a.
n.a.	2-3 BDO	n.a.	n.a.	n.a.	n.a.	n.a.	n.a.
n.a.	Ethanol	n.a.	n.a.	n.a.	n.a.	n.a.	n.a.
n.a.	Isobutyrate RI	n.a.	n.a.	n.a.	n.a.	n.a.	n.a.
n.a.	Butyrate RI	n.a.	n.a.	n.a.	n.a.	n.a.	n.a.

Chromeleon (c) Dionex
Version 7.2.10.25868

Chromatogram and SST Results

Injection Details

Injection Name:	**5 MUCHMO2 t24 r2**	*Run Time (min):*	**20,00**
Vial Number:	**3:D2**	*Injection Volume:*	**20,00**
Injection Type:	**Unknown**	*Channel:*	**RI_CH_1**
Calibration Level:		*Wavelength:*	**n.a.**
Instrument Method:	**Default method LC2030C 45 gr 20 min**	*Bandwidth:*	**n.a.**
Processing Method:	**Processing Method LC2030 45 gr**	*Dilution Factor:*	**1,0000**
Injection Date/Time:	**14-jun-23 02:55**	*Sample Weight:*	**1,0000**

Chromatogram

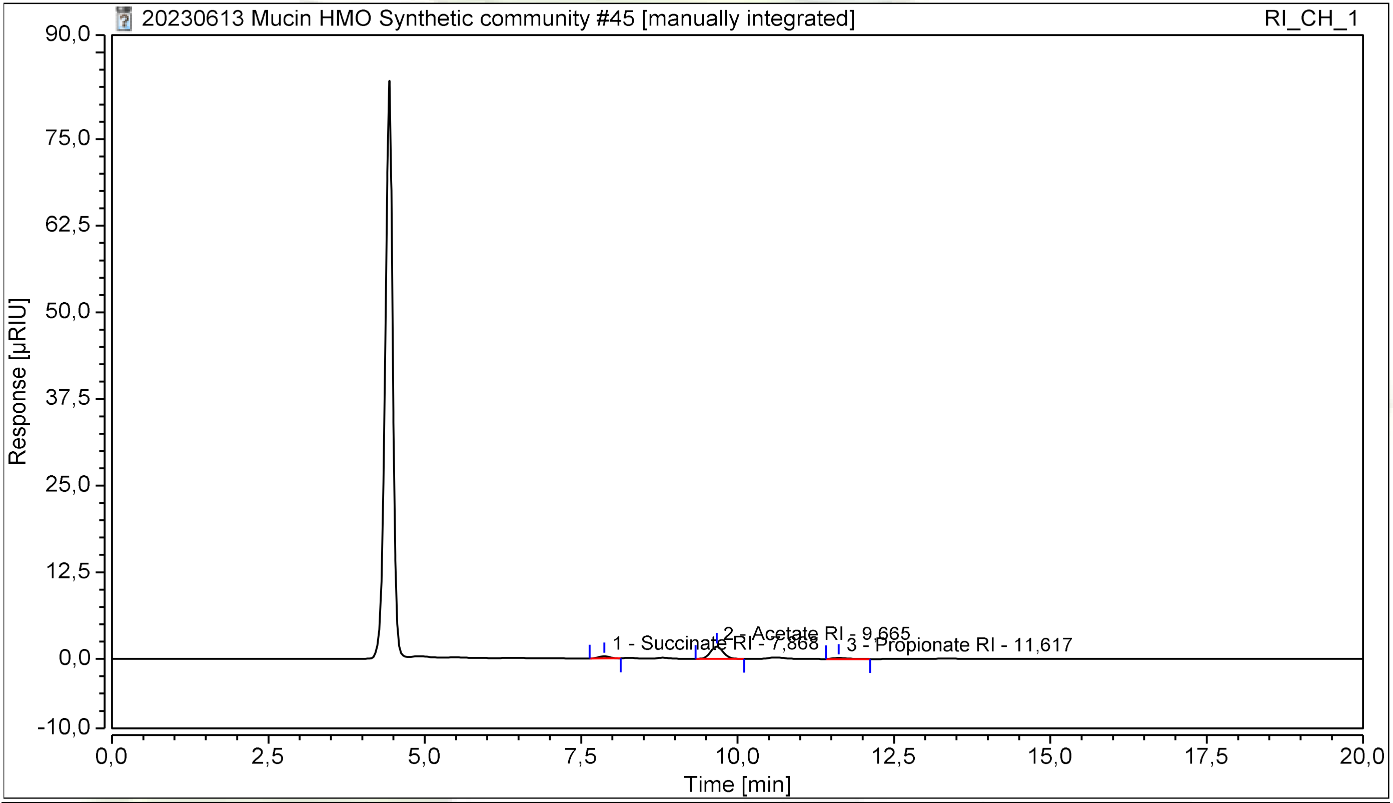

SST Results

No.	Name	Inj.Condition	Peak	Test Result	Injection

Number of executed test cases: **n.a.** **Total Result:** **Passed**

Chromatogram

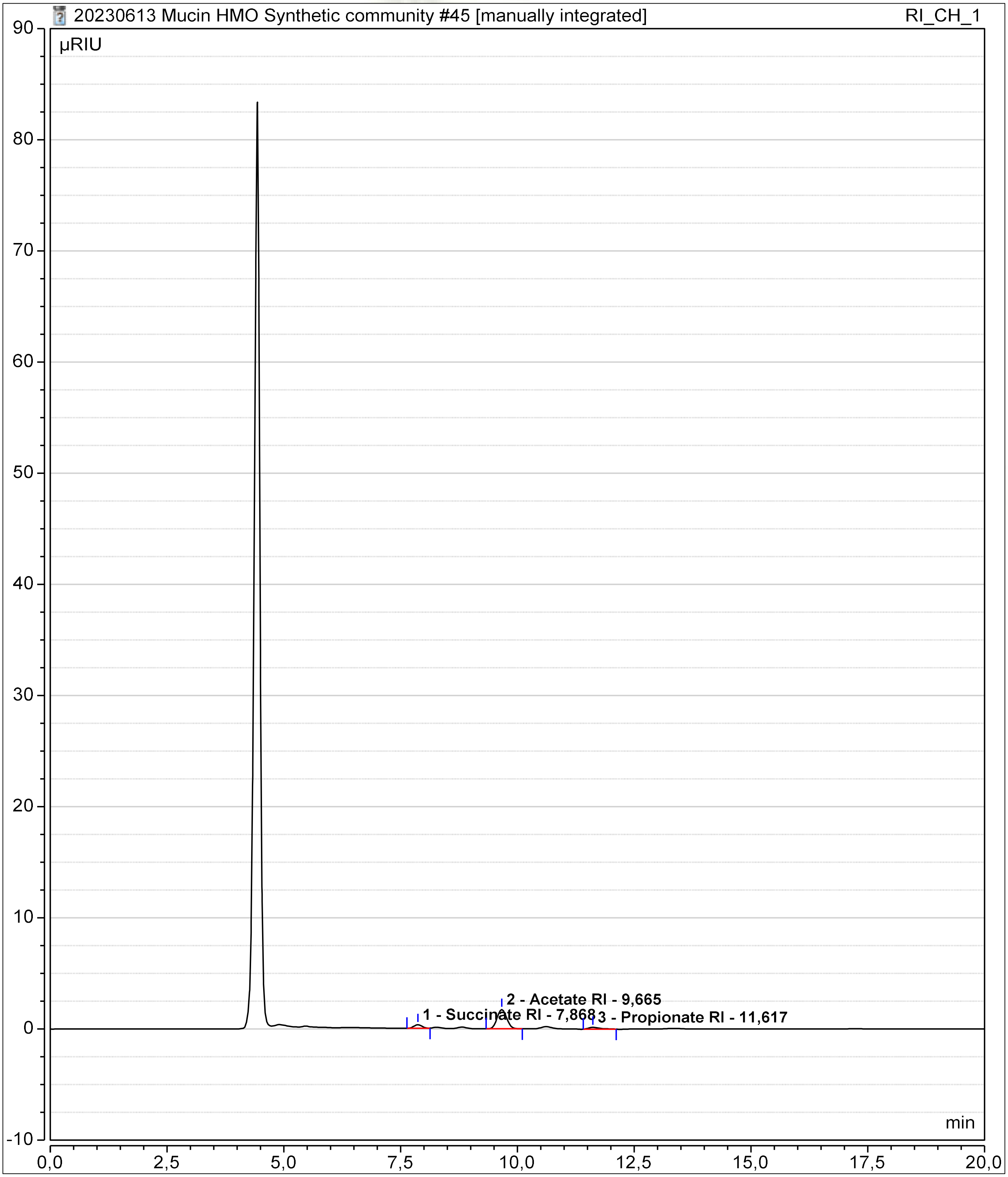

Chromatogram and Results

Injection Details

Injection Name:	**6 MUCHMO2 t24 r3**	*Run Time (min):*	**20,00**
Vial Number:	**3:D3**	*Injection Volume:*	**20,00**
Injection Type:	**Unknown**	*Channel:*	**RI_CH_1**
Calibration Level:		*Wavelength:*	**n.a.**
Instrument Method:	**Default method LC2030C 45 gr 20 min**	*Bandwidth:*	**n.a.**
Processing Method:	**Processing Method LC2030 45 gr**	*Dilution Factor:*	**1,0000**
Injection Date/Time:	**14-jun-23 03:16**	*Sample Weight:*	**1,0000**

Chromatogram

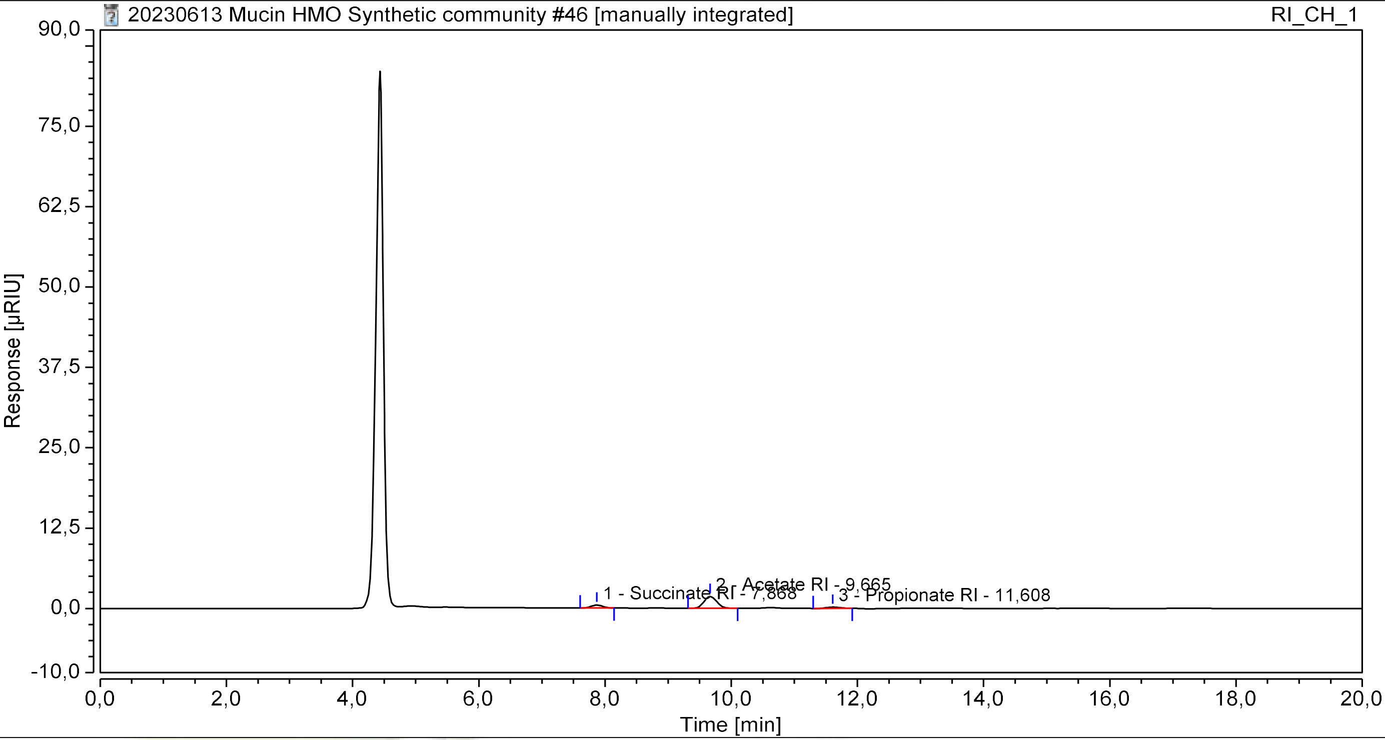

Integration Results

No.	Peak Name	Retention Time min	Area µRIU*min	Height µRIU	Relative Area %	Relative Height %	Amount
n.a.	GlcNAc	n.a.	n.a.	n.a.	n.a.	n.a.	n.a.
n.a.	Citrate	n.a.	n.a.	n.a.	n.a.	n.a.	n.a.
n.a.	Glucose	n.a.	n.a.	n.a.	n.a.	n.a.	n.a.
n.a.	Galactose	n.a.	n.a.	n.a.	n.a.	n.a.	n.a.
n.a.	Fucose	n.a.	n.a.	n.a.	n.a.	n.a.	n.a.
1	Succinate RI	7,868	0,096	0,458	16,50	18,39	n.a.
n.a.	Lactate RI	n.a.	n.a.	n.a.	n.a.	n.a.	n.a.
n.a.	glycerol	n.a.	n.a.	n.a.	n.a.	n.a.	n.a.
n.a.	Formate RI	n.a.	n.a.	n.a.	n.a.	n.a.	n.a.
2	Acetate RI	9,665	0,437	1,834	75,38	73,67	26,8702
n.a.	1,2 PDO RI	n.a.	n.a.	n.a.	n.a.	n.a.	n.a.
n.a.	1,3-PDO	n.a.	n.a.	n.a.	n.a.	n.a.	n.a.
3	Propionate RI	11,608	0,047	0,198	8,12	7,94	1,8926
n.a.	1,3-PDO	n.a.	n.a.	n.a.	n.a.	n.a.	n.a.
n.a.	2-3 BDO	n.a.	n.a.	n.a.	n.a.	n.a.	n.a.
n.a.	Ethanol	n.a.	n.a.	n.a.	n.a.	n.a.	n.a.
n.a.	Isobutyrate RI	n.a.	n.a.	n.a.	n.a.	n.a.	n.a.
n.a.	Butyrate RI	n.a.	n.a.	n.a.	n.a.	n.a.	n.a.
**Total:**			**0,579**	**2,490**	**100,00**	**100,00**	

Chromeleon (c) Dionex
Version 7.2.10.25868

Peak Analysis

Injection Details

Injection Name:	**6 MUCHMO2 t24 r3**	*Run Time (min):*	**20,00**
Vial Number:	**3:D3**	*Injection Volume:*	**20,00**
Injection Type:	**Unknown**	*Channel:*	**RI_CH_1**
Calibration Level:		*Wavelength:*	**n.a.**
Instrument Method:	**Default method LC2030C 45 gr 20 min**	*Bandwidth:*	**n.a.**
Processing Method:	**Processing Method LC2030 45 gr**	*Dilution Factor:*	**1,0000**
Injection Date/Time:	**14-jun-23 03:16**	*Sample Weight:*	**1,0000**

Chromatogram

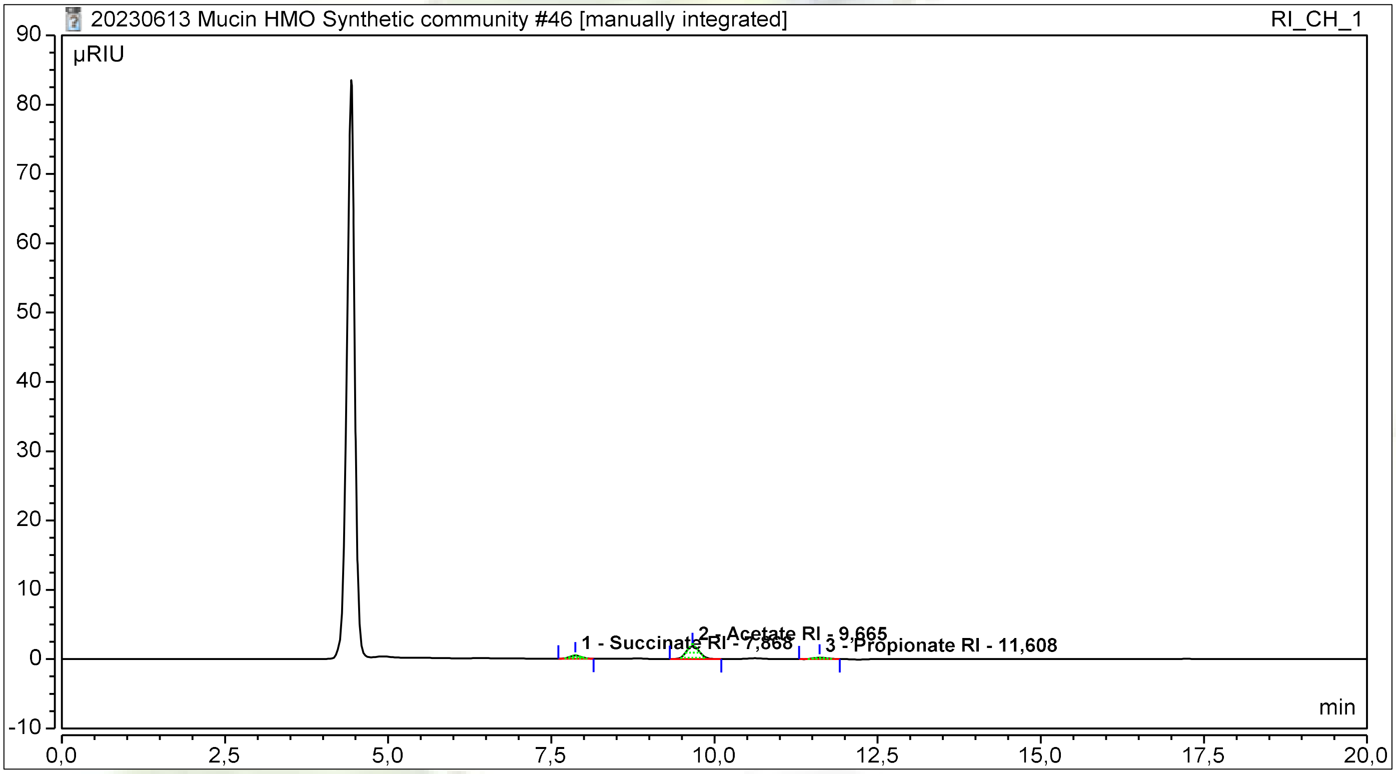

Peak Results

No.	Peak Name	Retention Time<br>min	Width (50%)<br>min	Type	Resolution (EP)	Asymmetry (EP)	Plates (EP)
n.a.	GlcNAc	n.a.	n.a.	n.a.	n.a.	n.a.	n.a.
n.a.	Citrate	n.a.	n.a.	n.a.	n.a.	n.a.	n.a.
n.a.	Glucose	n.a.	n.a.	n.a.	n.a.	n.a.	n.a.
n.a.	Galactose	n.a.	n.a.	n.a.	n.a.	n.a.	n.a.
n.a.	Fucose	n.a.	n.a.	n.a.	n.a.	n.a.	n.a.
1	Succinate RI	7,868	0,200	BMB*	4,99	1,06	8602
n.a.	Lactate RI	n.a.	n.a.	n.a.	n.a.	n.a.	n.a.
n.a.	glycerol	n.a.	n.a.	n.a.	n.a.	n.a.	n.a.
n.a.	Formate RI	n.a.	n.a.	n.a.	n.a.	n.a.	n.a.
2	Acetate RI	9,665	0,225	BMB	4,96	1,09	10183
n.a.	1,2 PDO RI	n.a.	n.a.	n.a.	n.a.	n.a.	n.a.
n.a.	1,3-PDO	n.a.	n.a.	n.a.	n.a.	n.a.	n.a.
3	Propionate RI	11,608	0,237	BMB*	n.a.	1,21	13257
n.a.	1,3-PDO	n.a.	n.a.	n.a.	n.a.	n.a.	n.a.
n.a.	2-3 BDO	n.a.	n.a.	n.a.	n.a.	n.a.	n.a.
n.a.	Ethanol	n.a.	n.a.	n.a.	n.a.	n.a.	n.a.
n.a.	Isobutyrate RI	n.a.	n.a.	n.a.	n.a.	n.a.	n.a.
n.a.	Butyrate RI	n.a.	n.a.	n.a.	n.a.	n.a.	n.a.

Chromatogram and SST Results

Injection Details

Injection Name:	**6 MUCHMO2 t24 r3**	*Run Time (min):*	**20,00**
Vial Number:	**3:D3**	*Injection Volume:*	**20,00**
Injection Type:	**Unknown**	*Channel:*	**RI_CH_1**
Calibration Level:		*Wavelength:*	**n.a.**
Instrument Method:	**Default method LC2030C 45 gr 20 min**	*Bandwidth:*	**n.a.**
Processing Method:	**Processing Method LC2030 45 gr**	*Dilution Factor:*	**1,0000**
Injection Date/Time:	**14-jun-23 03:16**	*Sample Weight:*	**1,0000**

Chromatogram

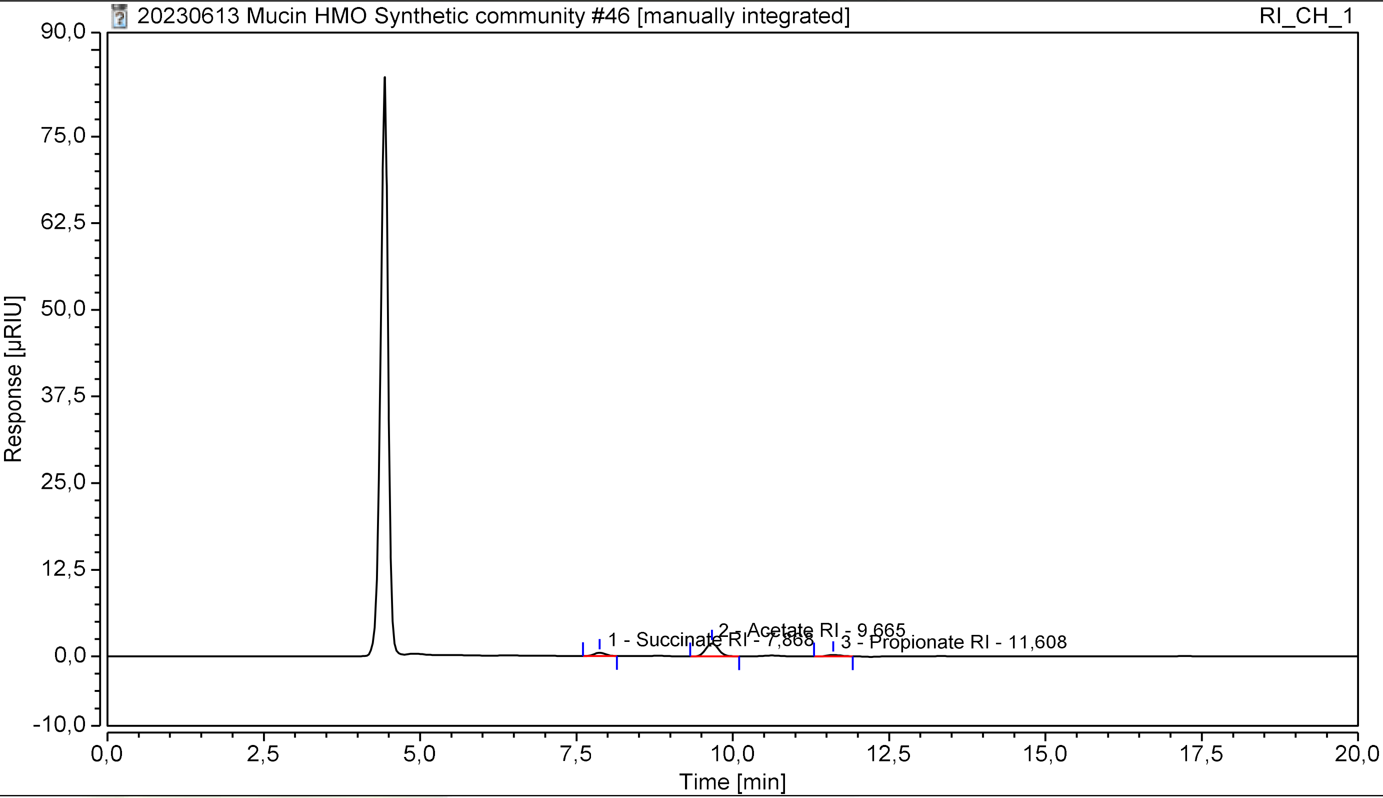

SST Results

No.	Name	Inj.Condition	Peak	Test Result	Injection

Number of executed test cases: **n.a.** **Total Result:** **Passed**

Chromatogram

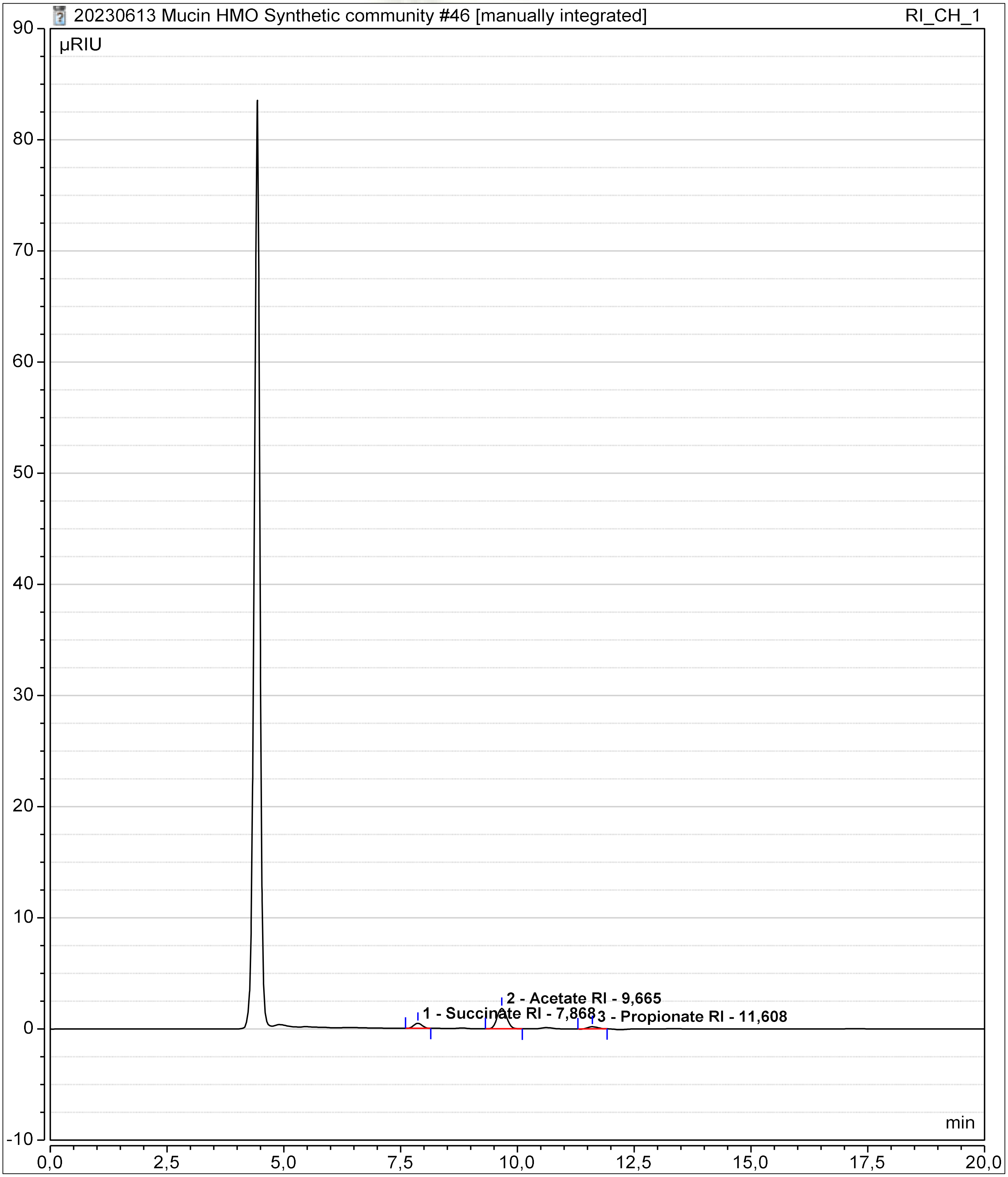

Chromeleon (c) Dionex
Version 7.2.10.25868

Chromatogram and Results

Injection Details

Injection Name:	**7 MUC t24 r1**	*Run Time (min):*	**20,00**
Vial Number:	**3:D4**	*Injection Volume:*	**20,00**
Injection Type:	**Unknown**	*Channel:*	**RI_CH_1**
Calibration Level:		*Wavelength:*	**n.a.**
Instrument Method:	**Default method LC2030C 45 gr 20 min**	*Bandwidth:*	**n.a.**
Processing Method:	**Processing Method LC2030 45 gr**	*Dilution Factor:*	**1,0000**
Injection Date/Time:	**14-jun-23 03:36**	*Sample Weight:*	**1,0000**

Chromatogram

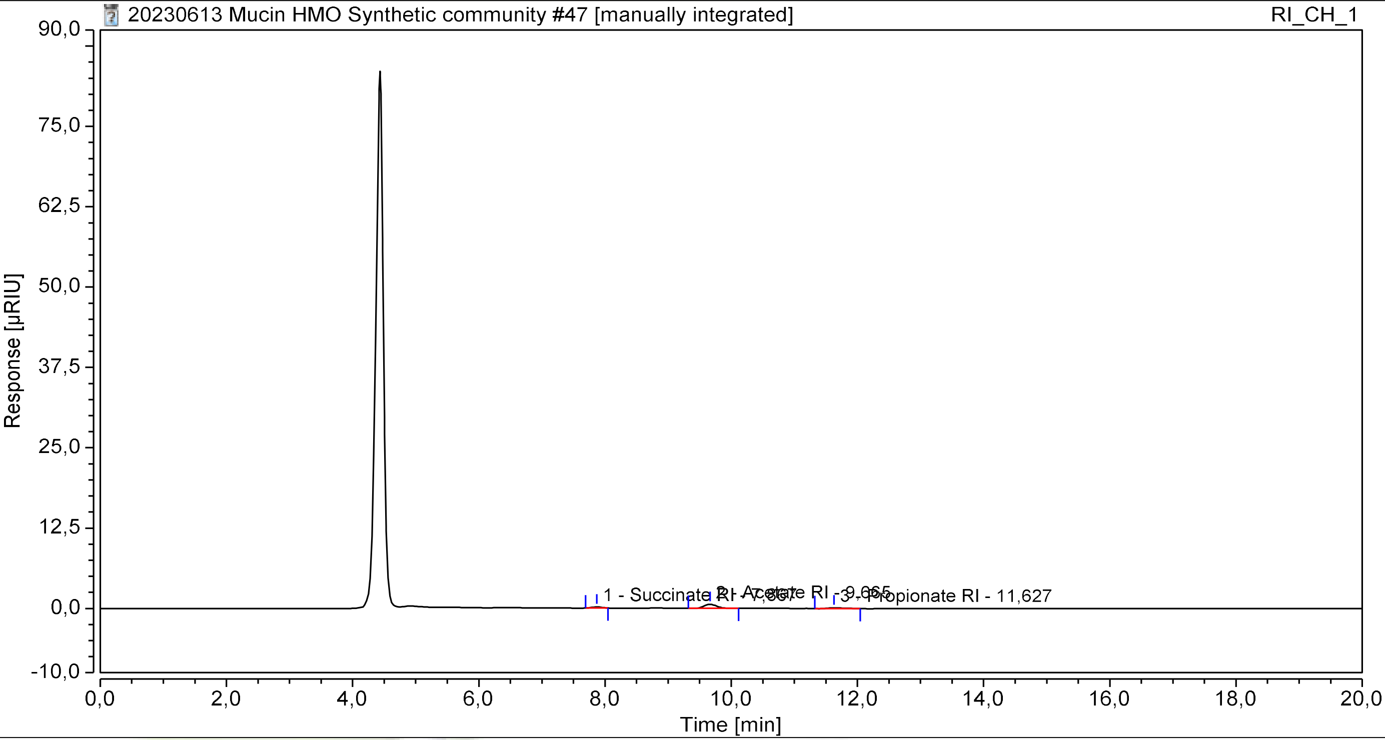

Integration Results

No.	Peak Name	Retention Time min	Area µRIU*min	Height µRIU	Relative Area %	Relative Height %	Amount
n.a.	GlcNAc	n.a.	n.a.	n.a.	n.a.	n.a.	n.a.
n.a.	Citrate	n.a.	n.a.	n.a.	n.a.	n.a.	n.a.
n.a.	Glucose	n.a.	n.a.	n.a.	n.a.	n.a.	n.a.
n.a.	Galactose	n.a.	n.a.	n.a.	n.a.	n.a.	n.a.
n.a.	Fucose	n.a.	n.a.	n.a.	n.a.	n.a.	n.a.
1	Succinate RI	7,867	0,031	0,167	14,86	18,40	n.a.
n.a.	Lactate RI	n.a.	n.a.	n.a.	n.a.	n.a.	n.a.
n.a.	glycerol	n.a.	n.a.	n.a.	n.a.	n.a.	n.a.
n.a.	Formate RI	n.a.	n.a.	n.a.	n.a.	n.a.	n.a.
2	Acetate RI	9,665	0,150	0,629	70,98	69,07	9,2084
n.a.	1,2 PDO RI	n.a.	n.a.	n.a.	n.a.	n.a.	n.a.
n.a.	1,3-PDO	n.a.	n.a.	n.a.	n.a.	n.a.	n.a.
3	Propionate RI	11,627	0,030	0,114	14,16	12,53	1,2010
n.a.	1,3-PDO	n.a.	n.a.	n.a.	n.a.	n.a.	n.a.
n.a.	2-3 BDO	n.a.	n.a.	n.a.	n.a.	n.a.	n.a.
n.a.	Ethanol	n.a.	n.a.	n.a.	n.a.	n.a.	n.a.
n.a.	Isobutyrate RI	n.a.	n.a.	n.a.	n.a.	n.a.	n.a.
n.a.	Butyrate RI	n.a.	n.a.	n.a.	n.a.	n.a.	n.a.
**Total:**			**0,211**	**0,910**	**100,00**	**100,00**	

Peak Analysis

Injection Details

Injection Name:	**7 MUC t24 r1**	*Run Time (min):*	**20,00**
Vial Number:	**3:D4**	*Injection Volume:*	**20,00**
Injection Type:	**Unknown**	*Channel:*	**RI_CH_1**
Calibration Level:		*Wavelength:*	**n.a.**
Instrument Method:	**Default method LC2030C 45 gr 20 min**	*Bandwidth:*	**n.a.**
Processing Method:	**Processing Method LC2030 45 gr**	*Dilution Factor:*	**1,0000**
Injection Date/Time:	**14-jun-23 03:36**	*Sample Weight:*	**1,0000**

Chromatogram

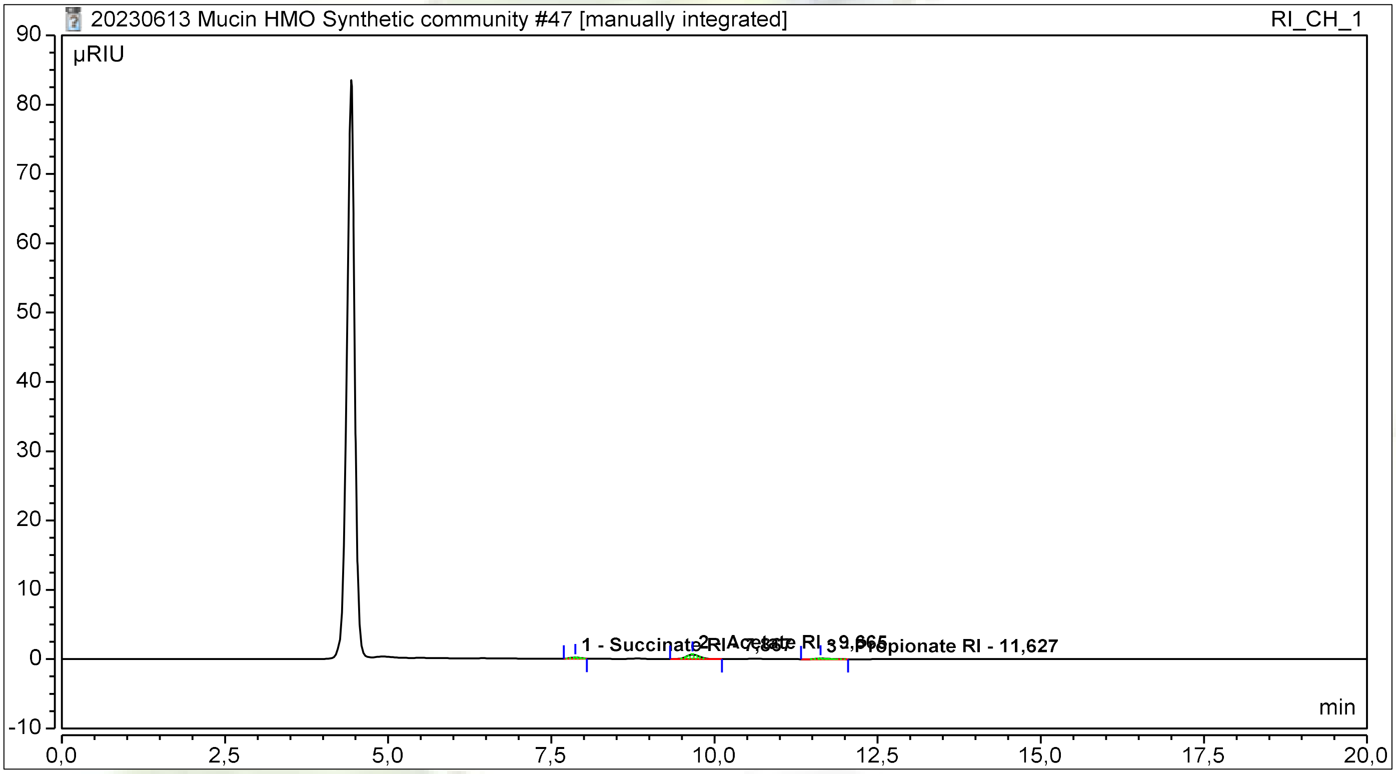

Peak Results

No.	Peak Name	Retention Time min	Width (50%) min	Type	Resolution (EP)	Asymmetry (EP)	Plates (EP)
n.a.	GlcNAc	n.a.	n.a.	n.a.	n.a.	n.a.	n.a.
n.a.	Citrate	n.a.	n.a.	n.a.	n.a.	n.a.	n.a.
n.a.	Glucose	n.a.	n.a.	n.a.	n.a.	n.a.	n.a.
n.a.	Galactose	n.a.	n.a.	n.a.	n.a.	n.a.	n.a.
n.a.	Fucose	n.a.	n.a.	n.a.	n.a.	n.a.	n.a.
1	Succinate RI	7,867	0,186	BMB*	5,17	1,02	9959
n.a.	Lactate RI	n.a.	n.a.	n.a.	n.a.	n.a.	n.a.
n.a.	glycerol	n.a.	n.a.	n.a.	n.a.	n.a.	n.a.
n.a.	Formate RI	n.a.	n.a.	n.a.	n.a.	n.a.	n.a.
2	Acetate RI	9,665	0,225	BMB	4,93	1,09	10202
n.a.	1,2 PDO RI	n.a.	n.a.	n.a.	n.a.	n.a.	n.a.
n.a.	1,3-PDO	n.a.	n.a.	n.a.	n.a.	n.a.	n.a.
3	Propionate RI	11,627	0,245	BMB*	n.a.	1,76	12518
n.a.	1,3-PDO	n.a.	n.a.	n.a.	n.a.	n.a.	n.a.
n.a.	2-3 BDO	n.a.	n.a.	n.a.	n.a.	n.a.	n.a.
n.a.	Ethanol	n.a.	n.a.	n.a.	n.a.	n.a.	n.a.
n.a.	Isobutyrate RI	n.a.	n.a.	n.a.	n.a.	n.a.	n.a.
n.a.	Butyrate RI	n.a.	n.a.	n.a.	n.a.	n.a.	n.a.

Chromatogram and SST Results

Injection Details

Injection Name:	**7 MUC t24 r1**	*Run Time (min):*	**20,00**
Vial Number:	**3:D4**	*Injection Volume:*	**20,00**
Injection Type:	**Unknown**	*Channel:*	**RI_CH_1**
Calibration Level:		*Wavelength:*	**n.a.**
Instrument Method:	**Default method LC2030C 45 gr 20 min**	*Bandwidth:*	**n.a.**
Processing Method:	**Processing Method LC2030 45 gr**	*Dilution Factor:*	**1,0000**
Injection Date/Time:	**14-jun-23 03:36**	*Sample Weight:*	**1,0000**

Chromatogram

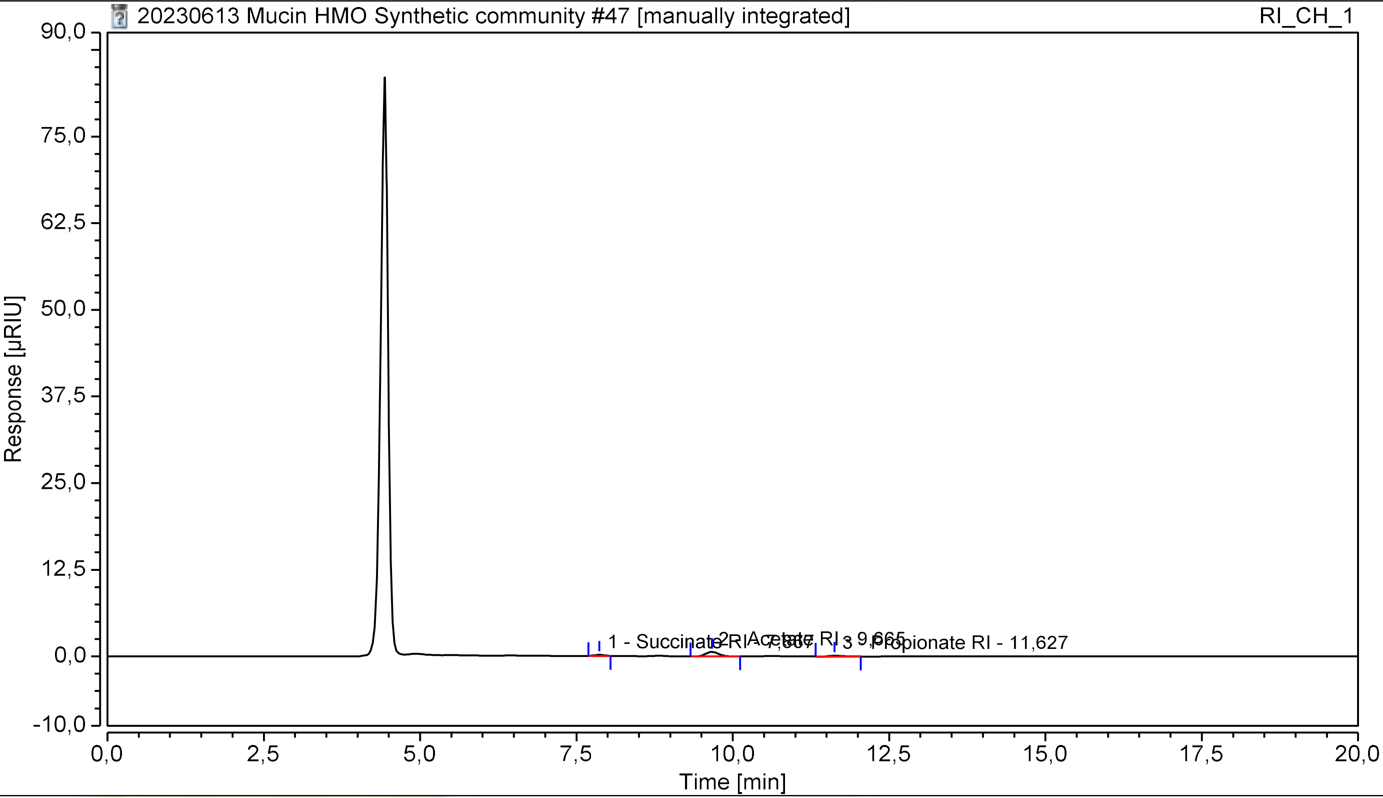

SST Results

No.	Name	Inj.Condition	Peak	Test Result	Injection

Number of executed test cases: **n.a.** **Total Result:** **Passed**

Chromatogram

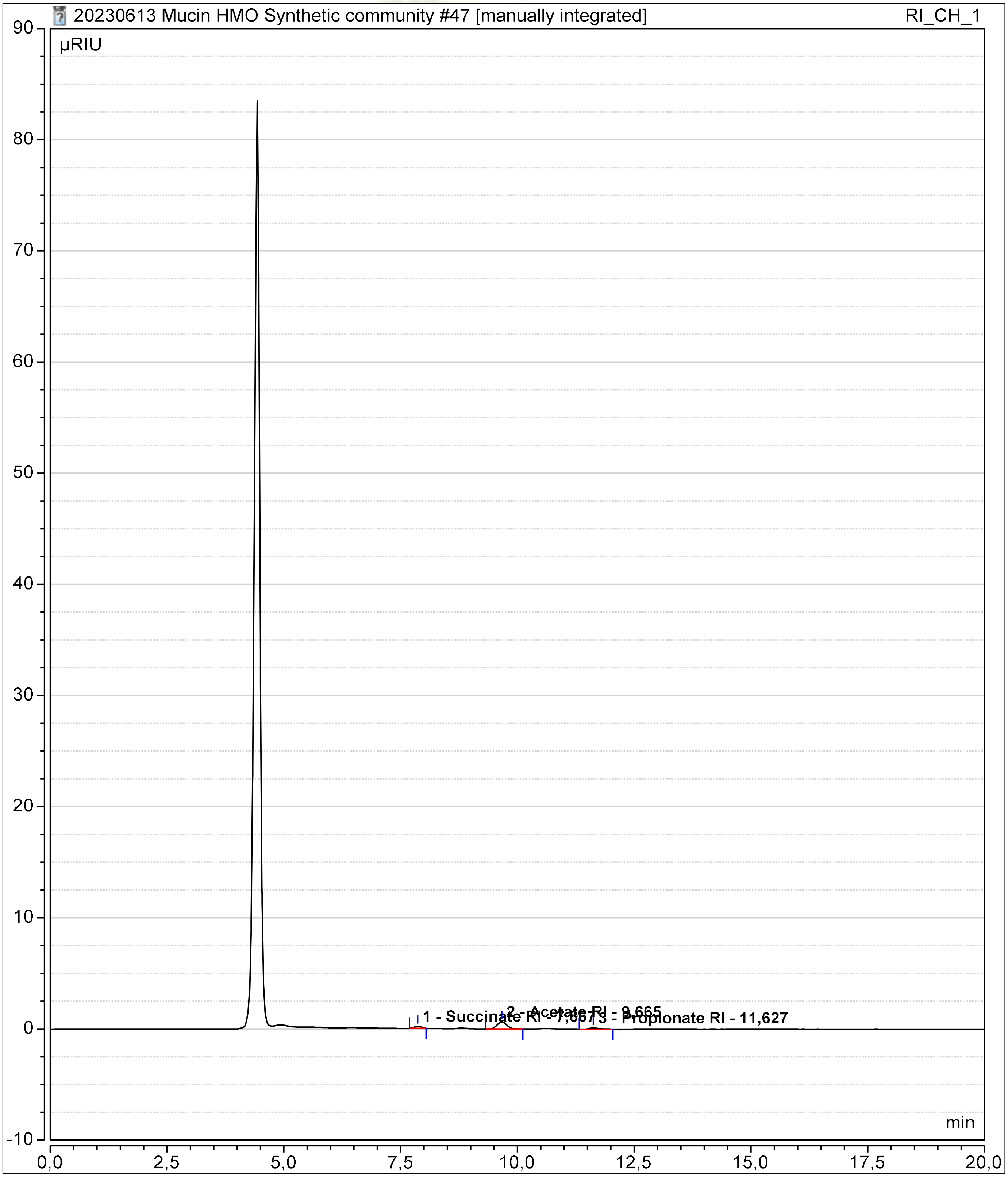

Chromeleon (c) Dionex
Version 7.2.10.25868

Chromatogram and Results

Injection Details

Injection Name:	**8 MUC t24 r2**	*Run Time (min):*	**20,00**
Vial Number:	**3:D5**	*Injection Volume:*	**20,00**
Injection Type:	**Unknown**	*Channel:*	**RI_CH_1**
Calibration Level:		*Wavelength:*	**n.a.**
Instrument Method:	**Default method LC2030C 45 gr 20 min**	*Bandwidth:*	**n.a.**
Processing Method:	**Processing Method LC2030 45 gr**	*Dilution Factor:*	**1,0000**
Injection Date/Time:	**14-jun-23 03:57**	*Sample Weight:*	**1,0000**

Chromatogram

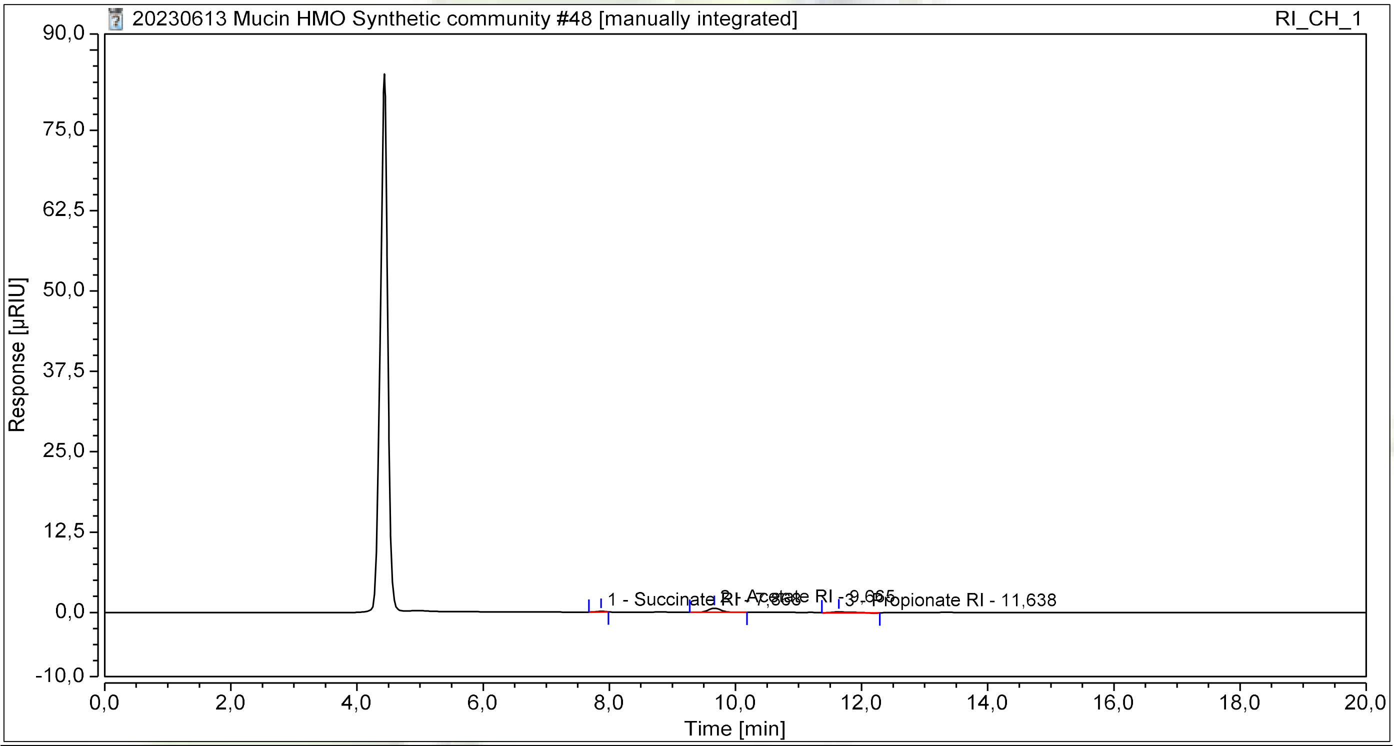

Integration Results

No.	Peak Name	Retention Time min	Area µRIU*min	Height µRIU	Relative Area %	Relative Height %	Amount
n.a.	GlcNAc	n.a.	n.a.	n.a.	n.a.	n.a.	n.a.
n.a.	Citrate	n.a.	n.a.	n.a.	n.a.	n.a.	n.a.
n.a.	Glucose	n.a.	n.a.	n.a.	n.a.	n.a.	n.a.
n.a.	Galactose	n.a.	n.a.	n.a.	n.a.	n.a.	n.a.
n.a.	Fucose	n.a.	n.a.	n.a.	n.a.	n.a.	n.a.
1	Succinate RI	7,868	0,016	0,099	7,79	11,49	n.a.
n.a.	Lactate RI	n.a.	n.a.	n.a.	n.a.	n.a.	n.a.
n.a.	glycerol	n.a.	n.a.	n.a.	n.a.	n.a.	n.a.
n.a.	Formate RI	n.a.	n.a.	n.a.	n.a.	n.a.	n.a.
2	Acetate RI	9,665	0,153	0,643	72,79	74,27	9,4216
n.a.	1,2 PDO RI	n.a.	n.a.	n.a.	n.a.	n.a.	n.a.
n.a.	1,3-PDO	n.a.	n.a.	n.a.	n.a.	n.a.	n.a.
3	Propionate RI	11,638	0,041	0,123	19,42	14,24	1,6434
n.a.	1,3-PDO	n.a.	n.a.	n.a.	n.a.	n.a.	n.a.
n.a.	2-3 BDO	n.a.	n.a.	n.a.	n.a.	n.a.	n.a.
n.a.	Ethanol	n.a.	n.a.	n.a.	n.a.	n.a.	n.a.
n.a.	Isobutyrate RI	n.a.	n.a.	n.a.	n.a.	n.a.	n.a.
n.a.	Butyrate RI	n.a.	n.a.	n.a.	n.a.	n.a.	n.a.
**Total:**			**0,210**	**0,865**	**100,00**	**100,00**	

Peak Analysis

Injection Details

Injection Name:	**8 MUC t24 r2**	*Run Time (min):*	**20,00**
Vial Number:	**3:D5**	*Injection Volume:*	**20,00**
Injection Type:	**Unknown**	*Channel:*	**RI_CH_1**
Calibration Level:		*Wavelength:*	**n.a.**
Instrument Method:	**Default method LC2030C 45 gr 20 min**	*Bandwidth:*	**n.a.**
Processing Method:	**Processing Method LC2030 45 gr**	*Dilution Factor:*	**1,0000**
Injection Date/Time:	**14-jun-23 03:57**	*Sample Weight:*	**1,0000**

Chromatogram

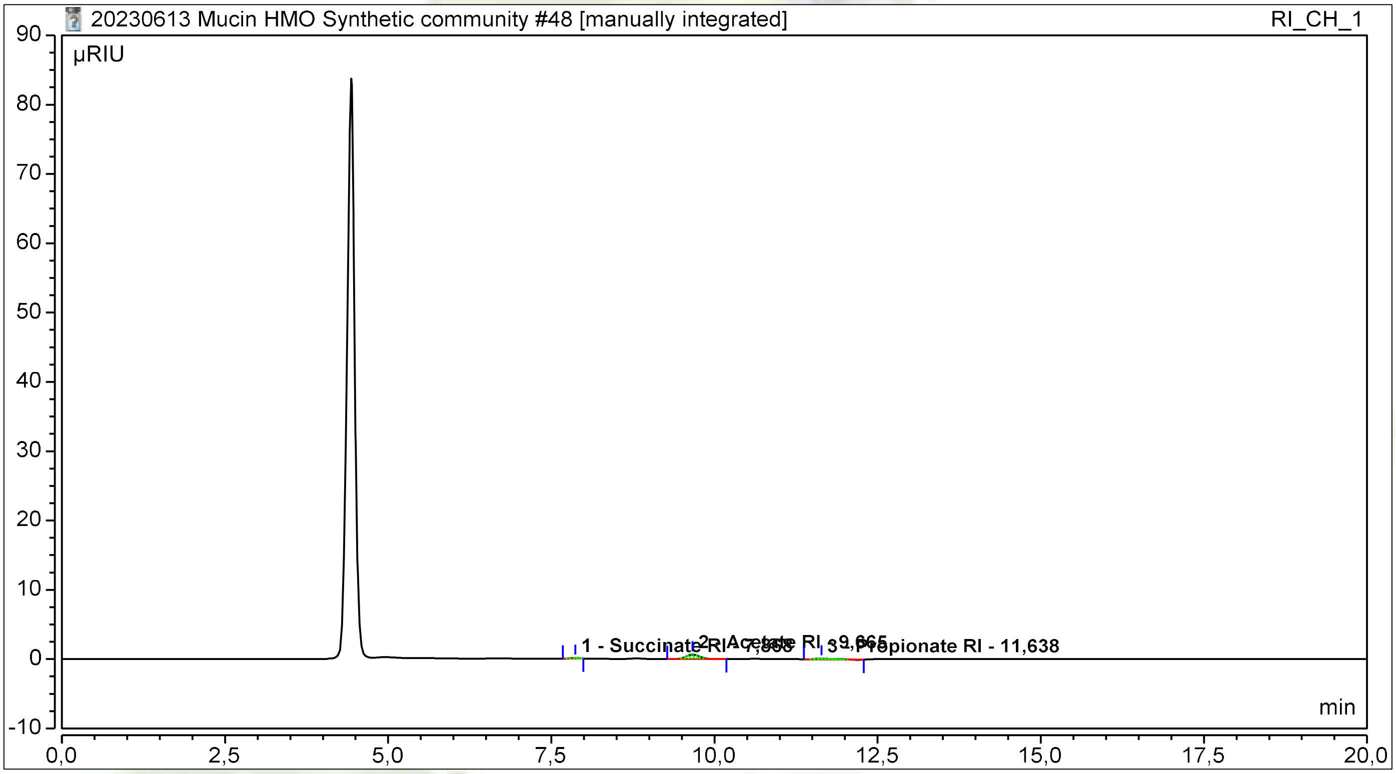

Peak Results

No.	Peak Name	Retention Time<br>min	Width (50%)<br>min	Type	Resolution (EP)	Asymmetry (EP)	Plates (EP)
n.a.	GlcNAc	n.a.	n.a.	n.a.	n.a.	n.a.	n.a.
n.a.	Citrate	n.a.	n.a.	n.a.	n.a.	n.a.	n.a.
n.a.	Glucose	n.a.	n.a.	n.a.	n.a.	n.a.	n.a.
n.a.	Galactose	n.a.	n.a.	n.a.	n.a.	n.a.	n.a.
n.a.	Fucose	n.a.	n.a.	n.a.	n.a.	n.a.	n.a.
1	Succinate RI	7,868	0,166	BMB*	5,43	0,85	12519
n.a.	Lactate RI	n.a.	n.a.	n.a.	n.a.	n.a.	n.a.
n.a.	glycerol	n.a.	n.a.	n.a.	n.a.	n.a.	n.a.
n.a.	Formate RI	n.a.	n.a.	n.a.	n.a.	n.a.	n.a.
2	Acetate RI	9,665	0,225	BMB	3,55	1,08	10236
n.a.	1,2 PDO RI	n.a.	n.a.	n.a.	n.a.	n.a.	n.a.
n.a.	1,3-PDO	n.a.	n.a.	n.a.	n.a.	n.a.	n.a.
3	Propionate RI	11,638	0,431	BMB*	n.a.	1,66	4035
n.a.	1,3-PDO	n.a.	n.a.	n.a.	n.a.	n.a.	n.a.
n.a.	2-3 BDO	n.a.	n.a.	n.a.	n.a.	n.a.	n.a.
n.a.	Ethanol	n.a.	n.a.	n.a.	n.a.	n.a.	n.a.
n.a.	Isobutyrate RI	n.a.	n.a.	n.a.	n.a.	n.a.	n.a.
n.a.	Butyrate RI	n.a.	n.a.	n.a.	n.a.	n.a.	n.a.

Chromatogram and SST Results

Injection Details

Injection Name:	**8 MUC t24 r2**	*Run Time (min):*	**20,00**
Vial Number:	**3:D5**	*Injection Volume:*	**20,00**
Injection Type:	**Unknown**	*Channel:*	**RI_CH_1**
Calibration Level:		*Wavelength:*	**n.a.**
Instrument Method:	**Default method LC2030C 45 gr 20 min**	*Bandwidth:*	**n.a.**
Processing Method:	**Processing Method LC2030 45 gr**	*Dilution Factor:*	**1,0000**
Injection Date/Time:	**14-jun-23 03:57**	*Sample Weight:*	**1,0000**

Chromatogram

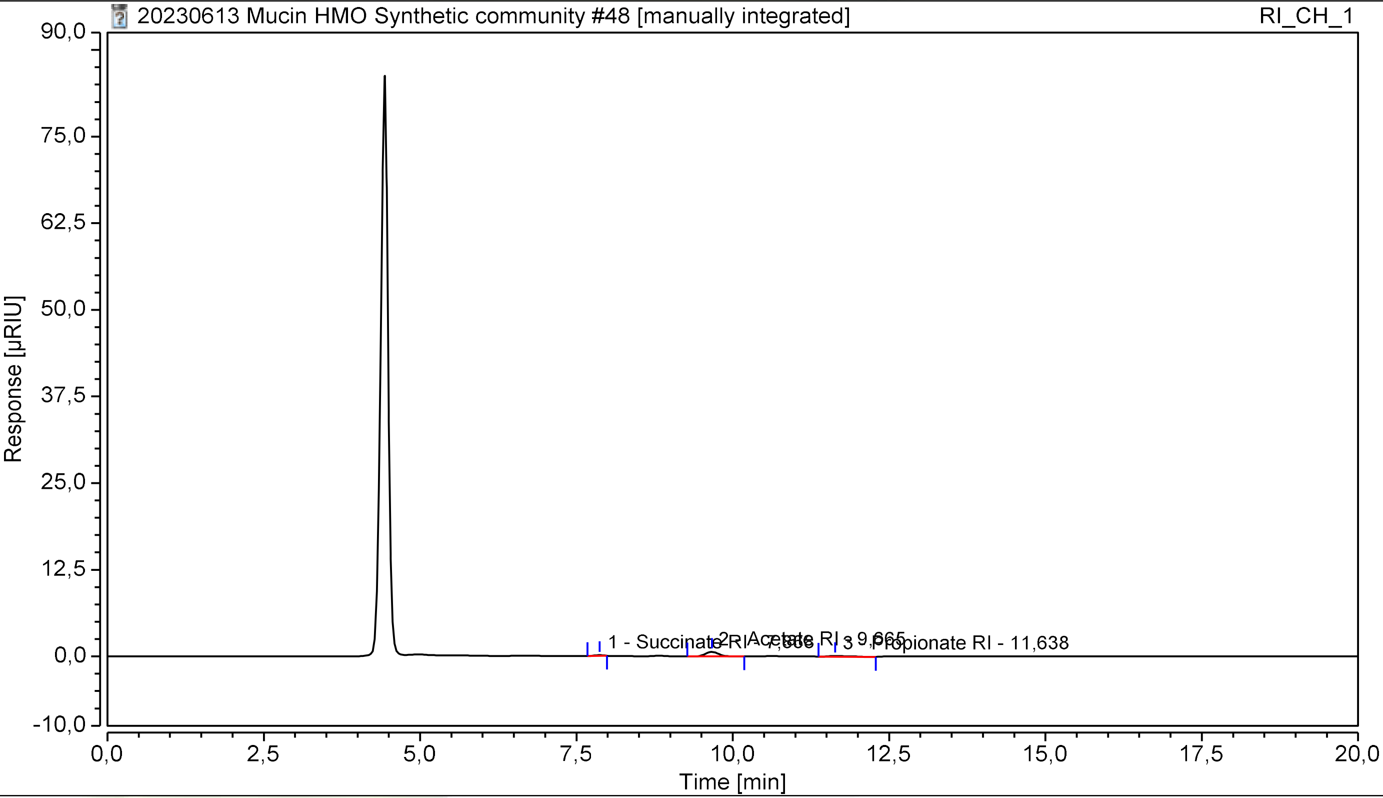

SST Results

No.	Name	Inj.Condition	Peak	Test Result	Injection

Number of executed test cases: **n.a.** **Total Result:** **Passed**

Chromatogram

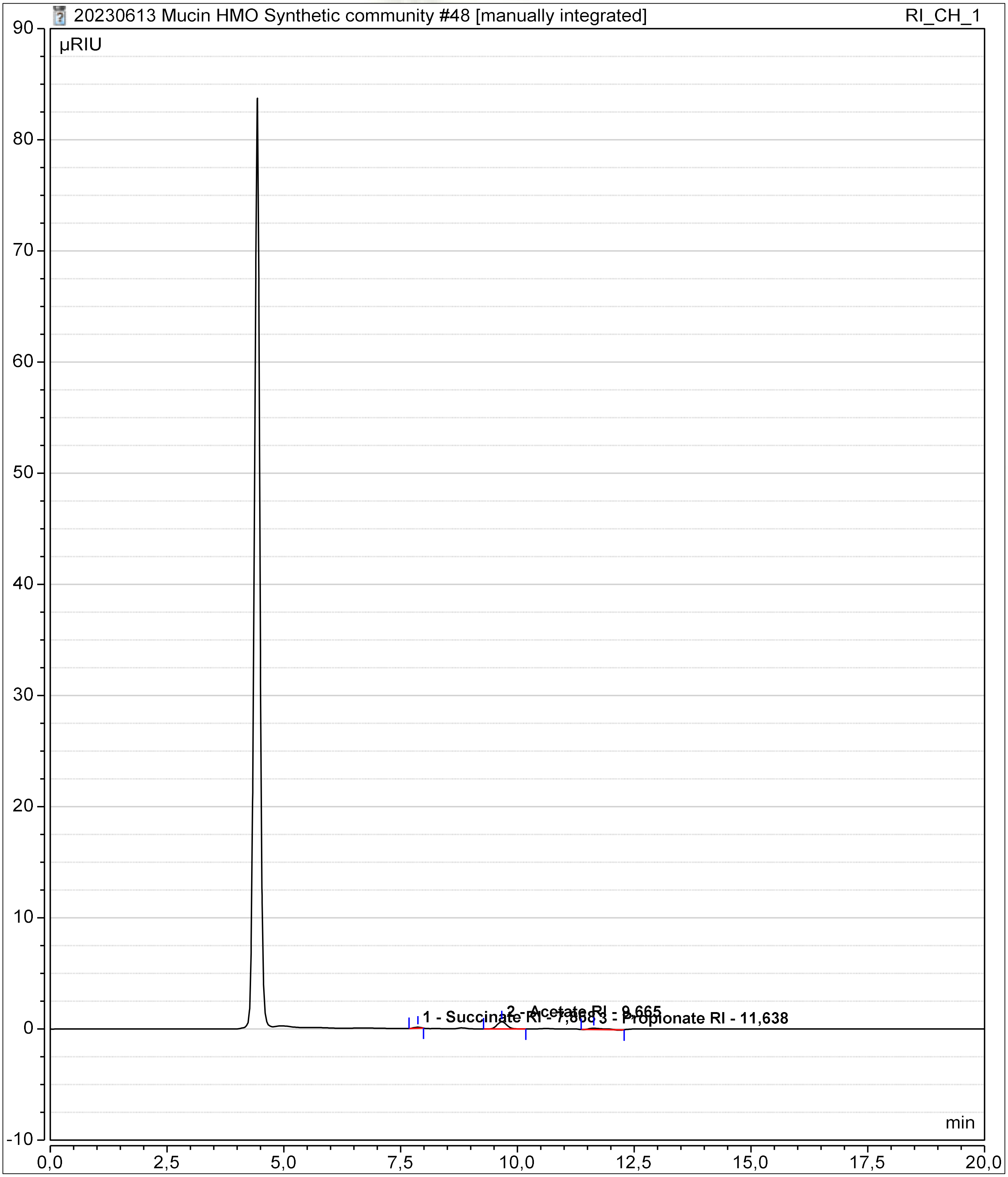

Chromeleon (c) Dionex
Version 7.2.10.25868

Chromatogram and Results

Injection Details

Injection Name:	**9 MUC t24 r3**	*Run Time (min):*	**20,00**
Vial Number:	**3:D6**	*Injection Volume:*	**20,00**
Injection Type:	**Unknown**	*Channel:*	**RI_CH_1**
Calibration Level:		*Wavelength:*	**n.a.**
Instrument Method:	**Default method LC2030C 45 gr 20 min**	*Bandwidth:*	**n.a.**
Processing Method:	**Processing Method LC2030 45 gr**	*Dilution Factor:*	**1,0000**
Injection Date/Time:	**14-jun-23 04:17**	*Sample Weight:*	**1,0000**

Chromatogram

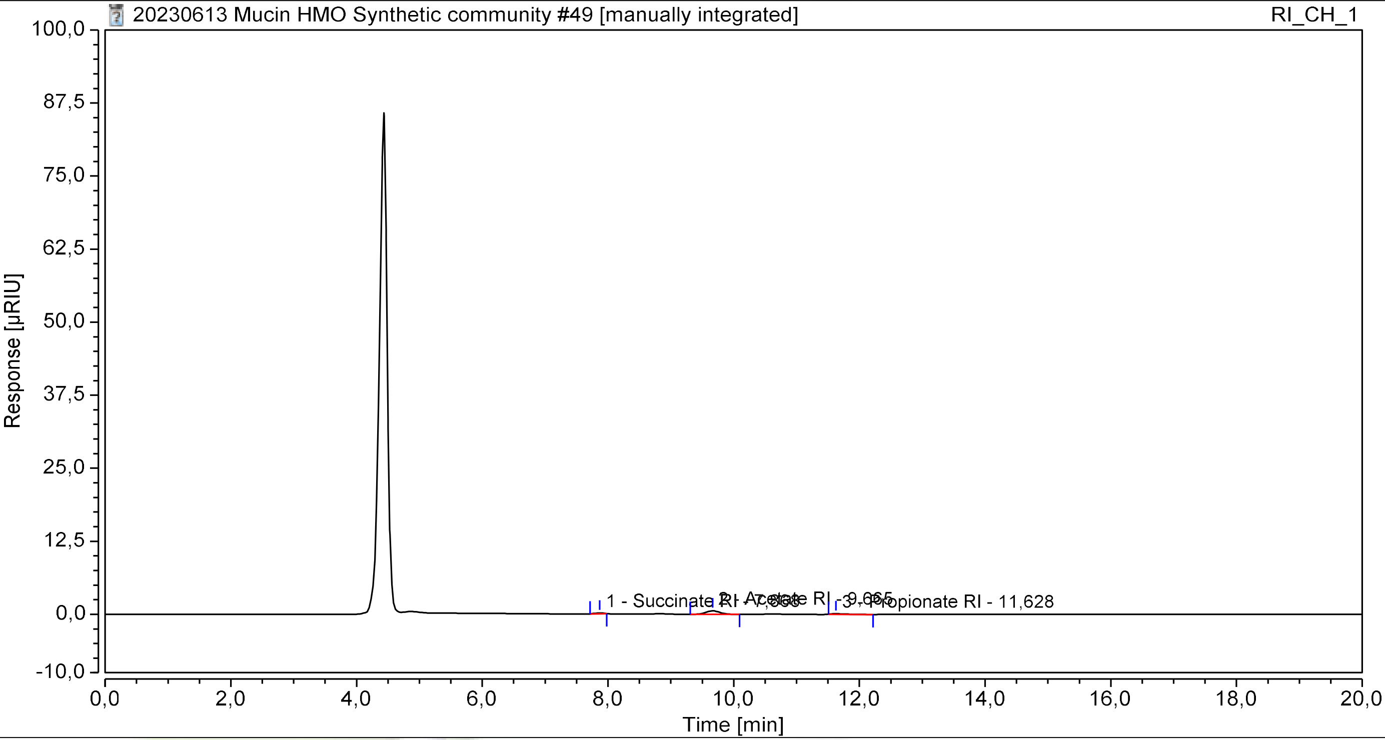

Integration Results

No.	Peak Name	Retention Time min	Area µRIU*min	Height µRIU	Relative Area %	Relative Height %	Amount
n.a.	GlcNAc	n.a.	n.a.	n.a.	n.a.	n.a.	n.a.
n.a.	Citrate	n.a.	n.a.	n.a.	n.a.	n.a.	n.a.
n.a.	Glucose	n.a.	n.a.	n.a.	n.a.	n.a.	n.a.
n.a.	Galactose	n.a.	n.a.	n.a.	n.a.	n.a.	n.a.
n.a.	Fucose	n.a.	n.a.	n.a.	n.a.	n.a.	n.a.
1	Succinate RI	7,868	0,019	0,121	10,27	15,27	n.a.
n.a.	Lactate RI	n.a.	n.a.	n.a.	n.a.	n.a.	n.a.
n.a.	glycerol	n.a.	n.a.	n.a.	n.a.	n.a.	n.a.
n.a.	Formate RI	n.a.	n.a.	n.a.	n.a.	n.a.	n.a.
2	Acetate RI	9,665	0,137	0,582	75,07	73,11	8,4565
n.a.	1,2 PDO RI	n.a.	n.a.	n.a.	n.a.	n.a.	n.a.
n.a.	1,3-PDO	n.a.	n.a.	n.a.	n.a.	n.a.	n.a.
3	Propionate RI	11,628	0,027	0,092	14,66	11,62	1,0798
n.a.	1,3-PDO	n.a.	n.a.	n.a.	n.a.	n.a.	n.a.
n.a.	2-3 BDO	n.a.	n.a.	n.a.	n.a.	n.a.	n.a.
n.a.	Ethanol	n.a.	n.a.	n.a.	n.a.	n.a.	n.a.
n.a.	Isobutyrate RI	n.a.	n.a.	n.a.	n.a.	n.a.	n.a.
n.a.	Butyrate RI	n.a.	n.a.	n.a.	n.a.	n.a.	n.a.
**Total:**			**0,183**	**0,796**	**100,00**	**100,00**	

Peak Analysis

Injection Details

Injection Name:	**9 MUC t24 r3**	*Run Time (min):*	**20,00**
Vial Number:	**3:D6**	*Injection Volume:*	**20,00**
Injection Type:	**Unknown**	*Channel:*	**RI_CH_1**
Calibration Level:		*Wavelength:*	**n.a.**
Instrument Method:	**Default method LC2030C 45 gr 20 min**	*Bandwidth:*	**n.a.**
Processing Method:	**Processing Method LC2030 45 gr**	*Dilution Factor:*	**1,0000**
Injection Date/Time:	**14-jun-23 04:17**	*Sample Weight:*	**1,0000**

Chromatogram

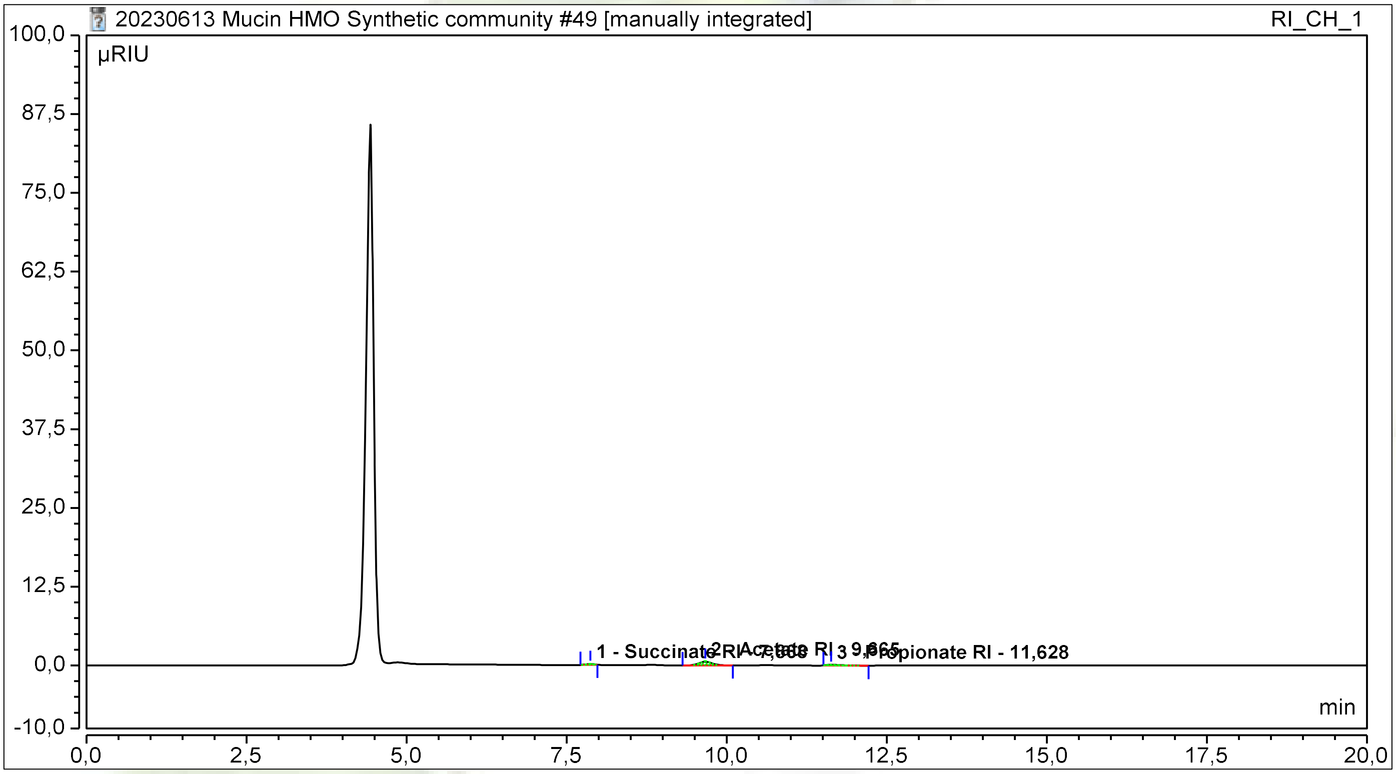

Peak Results

No.	Peak Name	Retention Time min	Width (50%) min	Type	Resolution (EP)	Asymmetry (EP)	Plates (EP)
n.a.	GlcNAc	n.a.	n.a.	n.a.	n.a.	n.a.	n.a.
n.a.	Citrate	n.a.	n.a.	n.a.	n.a.	n.a.	n.a.
n.a.	Glucose	n.a.	n.a.	n.a.	n.a.	n.a.	n.a.
n.a.	Galactose	n.a.	n.a.	n.a.	n.a.	n.a.	n.a.
n.a.	Fucose	n.a.	n.a.	n.a.	n.a.	n.a.	n.a.
1	Succinate RI	7,868	0,157	BMB*	5,57	0,88	13921
n.a.	Lactate RI	n.a.	n.a.	n.a.	n.a.	n.a.	n.a.
n.a.	glycerol	n.a.	n.a.	n.a.	n.a.	n.a.	n.a.
n.a.	Formate RI	n.a.	n.a.	n.a.	n.a.	n.a.	n.a.
2	Acetate RI	9,665	0,224	BMB	5,13	1,07	10350
n.a.	1,2 PDO RI	n.a.	n.a.	n.a.	n.a.	n.a.	n.a.
n.a.	1,3-PDO	n.a.	n.a.	n.a.	n.a.	n.a.	n.a.
3	Propionate RI	11,628	0,228	BMB*	n.a.	2,55	14377
n.a.	1,3-PDO	n.a.	n.a.	n.a.	n.a.	n.a.	n.a.
n.a.	2-3 BDO	n.a.	n.a.	n.a.	n.a.	n.a.	n.a.
n.a.	Ethanol	n.a.	n.a.	n.a.	n.a.	n.a.	n.a.
n.a.	Isobutyrate RI	n.a.	n.a.	n.a.	n.a.	n.a.	n.a.
n.a.	Butyrate RI	n.a.	n.a.	n.a.	n.a.	n.a.	n.a.

Chromatogram and SST Results

Injection Details

Injection Name:	**9 MUC t24 r3**	*Run Time (min):*	**20,00**
Vial Number:	**3:D6**	*Injection Volume:*	**20,00**
Injection Type:	**Unknown**	*Channel:*	**RI_CH_1**
Calibration Level:		*Wavelength:*	**n.a.**
Instrument Method:	**Default method LC2030C 45 gr 20 min**	*Bandwidth:*	**n.a.**
Processing Method:	**Processing Method LC2030 45 gr**	*Dilution Factor:*	**1,0000**
Injection Date/Time:	**14-jun-23 04:17**	*Sample Weight:*	**1,0000**

Chromatogram

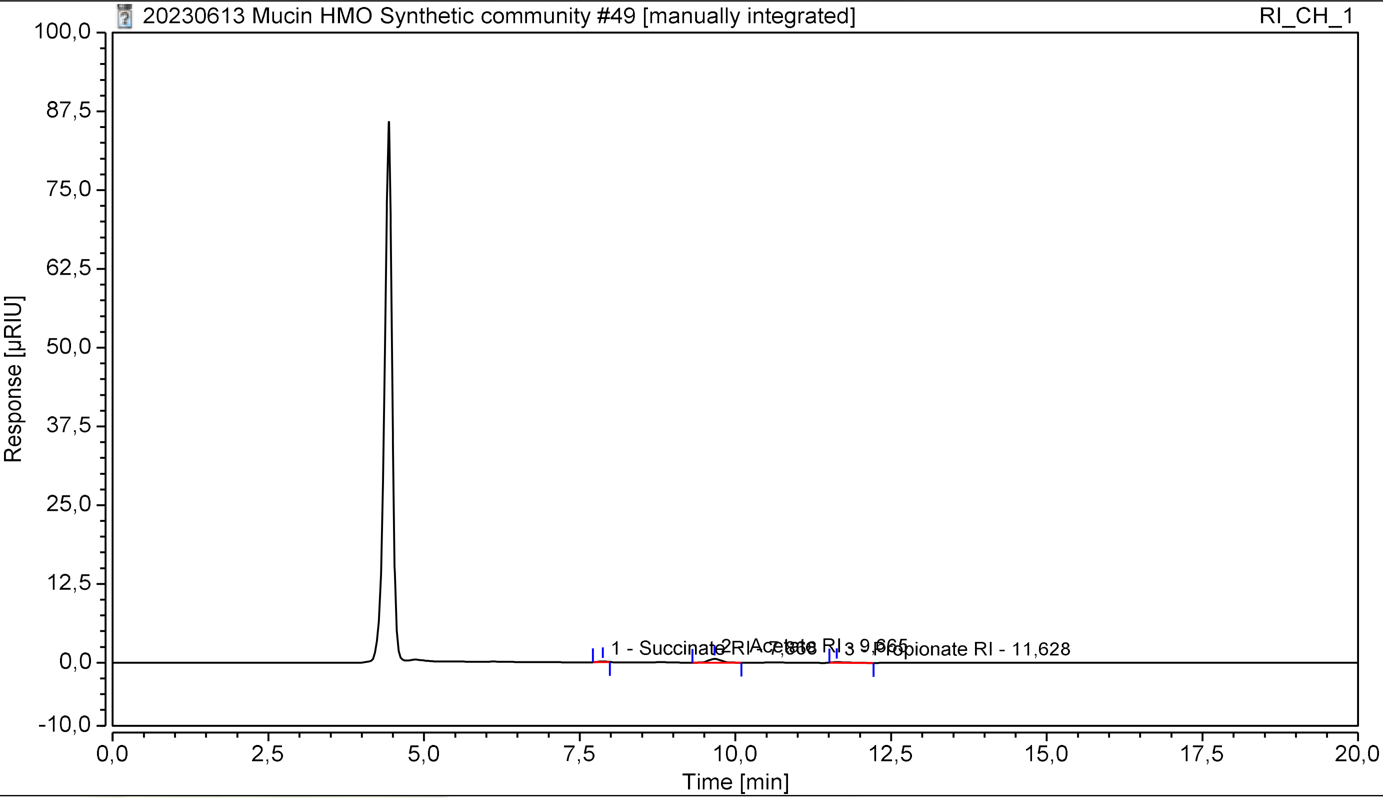

SST Results

No.	Name	Inj.Condition	Peak	Test Result	Injection

Number of executed test cases: **n.a.** **Total Result:** **Passed**

Chromatogram

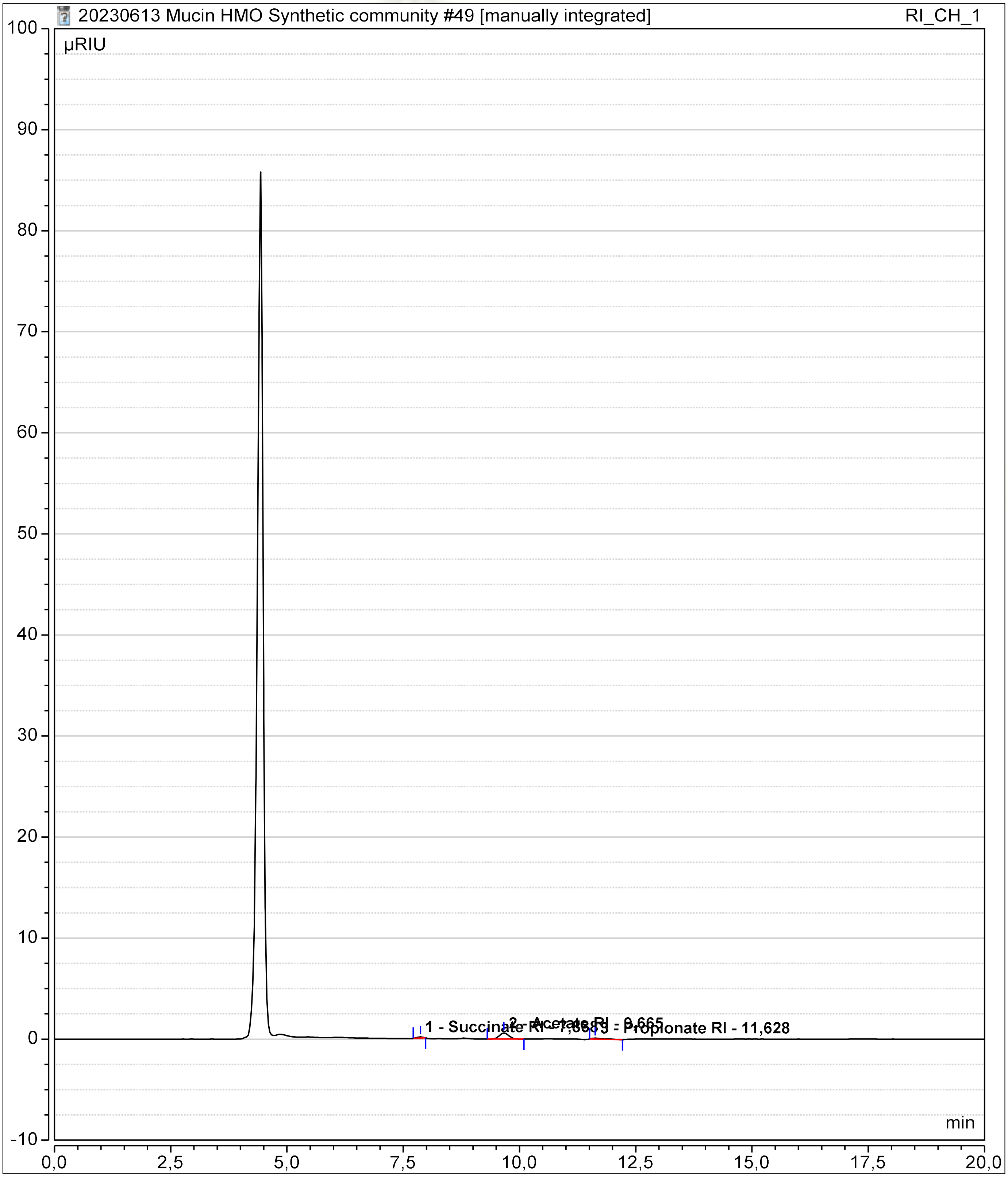

Chromeleon (c) Dionex
Version 7.2.10.25868

Chromatogram and Results

Injection Details

Injection Name:	**13 GOSFOSMUC t24 r1**	*Run Time (min):*	**20,00**
Vial Number:	**3:D7**	*Injection Volume:*	**20,00**
Injection Type:	**Unknown**	*Channel:*	**RI_CH_1**
Calibration Level:		*Wavelength:*	**n.a.**
Instrument Method:	**Default method LC2030C 45 gr 20 min**	*Bandwidth:*	**n.a.**
Processing Method:	**Processing Method LC2030 45 gr**	*Dilution Factor:*	**1,0000**
Injection Date/Time:	**14-jun-23 04:38**	*Sample Weight:*	**1,0000**

Chromatogram

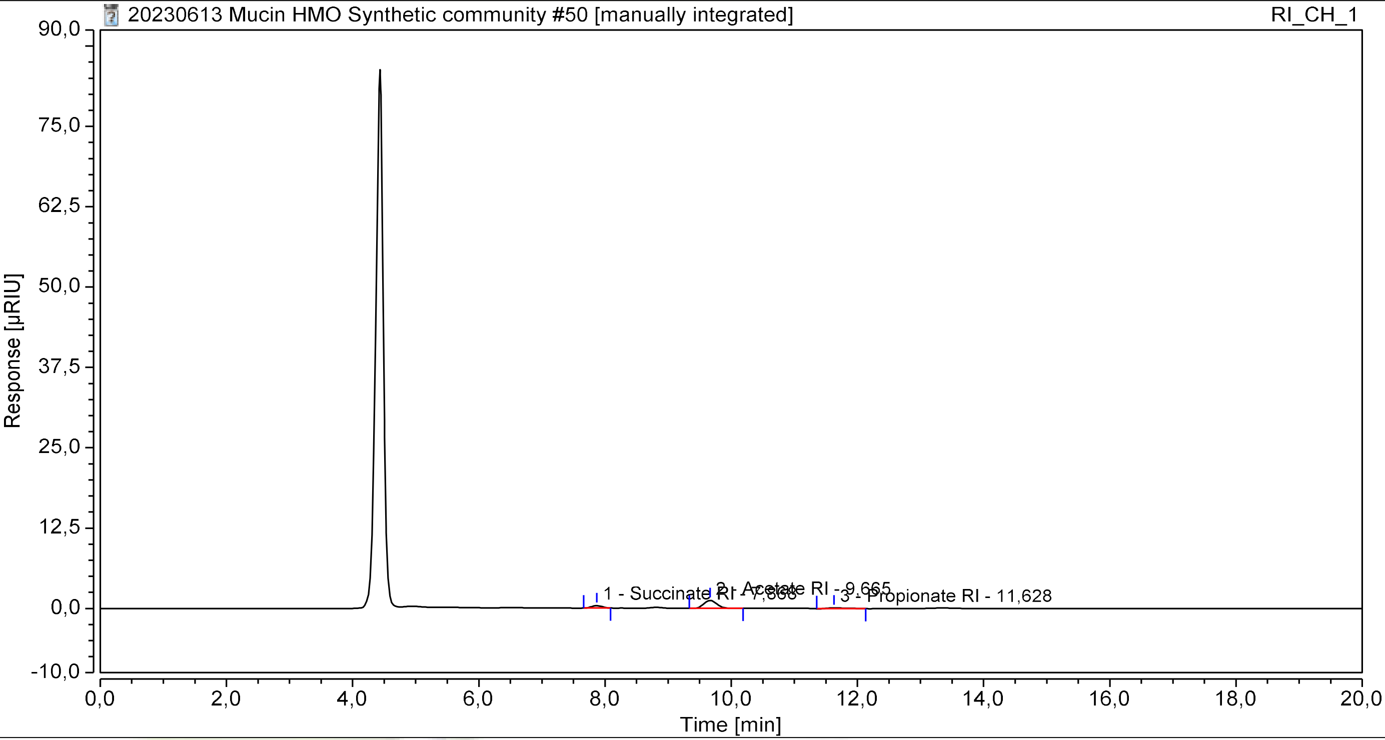

Integration Results

No.	Peak Name	Retention Time min	Area µRIU*min	Height µRIU	Relative Area %	Relative Height %	Amount
n.a.	GlcNAc	n.a.	n.a.	n.a.	n.a.	n.a.	n.a.
n.a.	Citrate	n.a.	n.a.	n.a.	n.a.	n.a.	n.a.
n.a.	Glucose	n.a.	n.a.	n.a.	n.a.	n.a.	n.a.
n.a.	Galactose	n.a.	n.a.	n.a.	n.a.	n.a.	n.a.
n.a.	Fucose	n.a.	n.a.	n.a.	n.a.	n.a.	n.a.
1	Succinate RI	7,868	0,074	0,368	17,98	21,19	n.a.
n.a.	Lactate RI	n.a.	n.a.	n.a.	n.a.	n.a.	n.a.
n.a.	glycerol	n.a.	n.a.	n.a.	n.a.	n.a.	n.a.
n.a.	Formate RI	n.a.	n.a.	n.a.	n.a.	n.a.	n.a.
2	Acetate RI	9,665	0,293	1,230	70,84	70,75	18,0328
n.a.	1,2 PDO RI	n.a.	n.a.	n.a.	n.a.	n.a.	n.a.
n.a.	1,3-PDO	n.a.	n.a.	n.a.	n.a.	n.a.	n.a.
3	Propionate RI	11,628	0,046	0,140	11,17	8,06	1,8594
n.a.	1,3-PDO	n.a.	n.a.	n.a.	n.a.	n.a.	n.a.
n.a.	2-3 BDO	n.a.	n.a.	n.a.	n.a.	n.a.	n.a.
n.a.	Ethanol	n.a.	n.a.	n.a.	n.a.	n.a.	n.a.
n.a.	Isobutyrate RI	n.a.	n.a.	n.a.	n.a.	n.a.	n.a.
n.a.	Butyrate RI	n.a.	n.a.	n.a.	n.a.	n.a.	n.a.
**Total:**			**0,414**	**1,739**	**100,00**	**100,00**	

Chromeleon (c) Dionex
Version 7.2.10.25868

Peak Analysis

Injection Details

Injection Name:	**13 GOSFOSMUC t24 r1**	*Run Time (min):*	**20,00**
Vial Number:	**3:D7**	*Injection Volume:*	**20,00**
Injection Type:	**Unknown**	*Channel:*	**RI_CH_1**
Calibration Level:		*Wavelength:*	**n.a.**
Instrument Method:	**Default method LC2030C 45 gr 20 min**	*Bandwidth:*	**n.a.**
Processing Method:	**Processing Method LC2030 45 gr**	*Dilution Factor:*	**1,0000**
Injection Date/Time:	**14-jun-23 04:38**	*Sample Weight:*	**1,0000**

Chromatogram

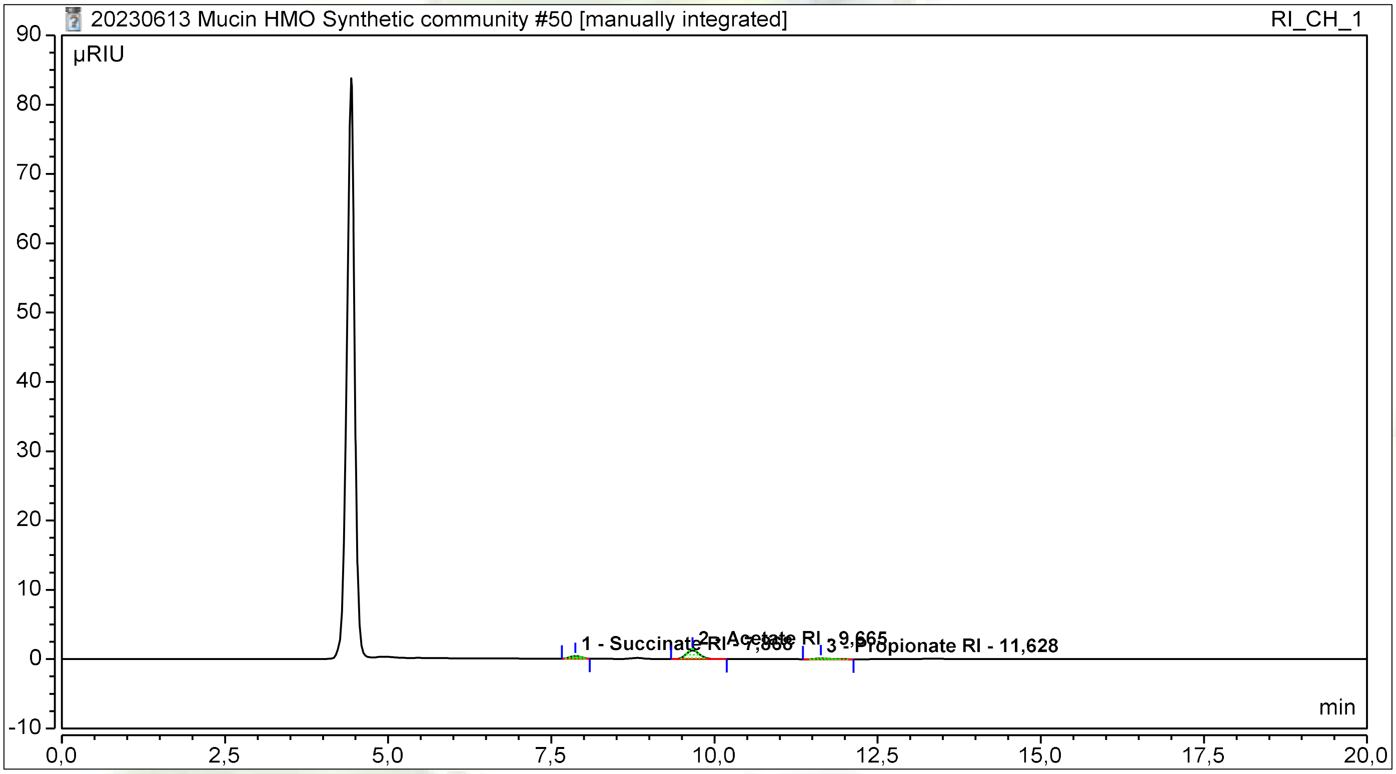

Peak Results

No.	Peak Name	Retention Time min	Width (50%) min	Type	Resolution (EP)	Asymmetry (EP)	Plates (EP)
n.a.	GlcNAc	n.a.	n.a.	n.a.	n.a.	n.a.	n.a.
n.a.	Citrate	n.a.	n.a.	n.a.	n.a.	n.a.	n.a.
n.a.	Glucose	n.a.	n.a.	n.a.	n.a.	n.a.	n.a.
n.a.	Galactose	n.a.	n.a.	n.a.	n.a.	n.a.	n.a.
n.a.	Fucose	n.a.	n.a.	n.a.	n.a.	n.a.	n.a.
1	Succinate RI	7,868	0,196	BMB*	5,03	1,05	8907
n.a.	Lactate RI	n.a.	n.a.	n.a.	n.a.	n.a.	n.a.
n.a.	glycerol	n.a.	n.a.	n.a.	n.a.	n.a.	n.a.
n.a.	Formate RI	n.a.	n.a.	n.a.	n.a.	n.a.	n.a.
2	Acetate RI	9,665	0,225	BMB	4,62	1,08	10205
n.a.	1,2 PDO RI	n.a.	n.a.	n.a.	n.a.	n.a.	n.a.
n.a.	1,3-PDO	n.a.	n.a.	n.a.	n.a.	n.a.	n.a.
3	Propionate RI	11,628	0,277	BMB*	n.a.	1,83	9784
n.a.	1,3-PDO	n.a.	n.a.	n.a.	n.a.	n.a.	n.a.
n.a.	2-3 BDO	n.a.	n.a.	n.a.	n.a.	n.a.	n.a.
n.a.	Ethanol	n.a.	n.a.	n.a.	n.a.	n.a.	n.a.
n.a.	Isobutyrate RI	n.a.	n.a.	n.a.	n.a.	n.a.	n.a.
n.a.	Butyrate RI	n.a.	n.a.	n.a.	n.a.	n.a.	n.a.

Chromatogram and SST Results

Injection Details

Injection Name:	**13 GOSFOSMUC t24 r1**	*Run Time (min):*	**20,00**
Vial Number:	**3:D7**	*Injection Volume:*	**20,00**
Injection Type:	**Unknown**	*Channel:*	**RI_CH_1**
Calibration Level:		*Wavelength:*	**n.a.**
Instrument Method:	**Default method LC2030C 45 gr 20 min**	*Bandwidth:*	**n.a.**
Processing Method:	**Processing Method LC2030 45 gr**	*Dilution Factor:*	**1,0000**
Injection Date/Time:	**14-jun-23 04:38**	*Sample Weight:*	**1,0000**

Chromatogram

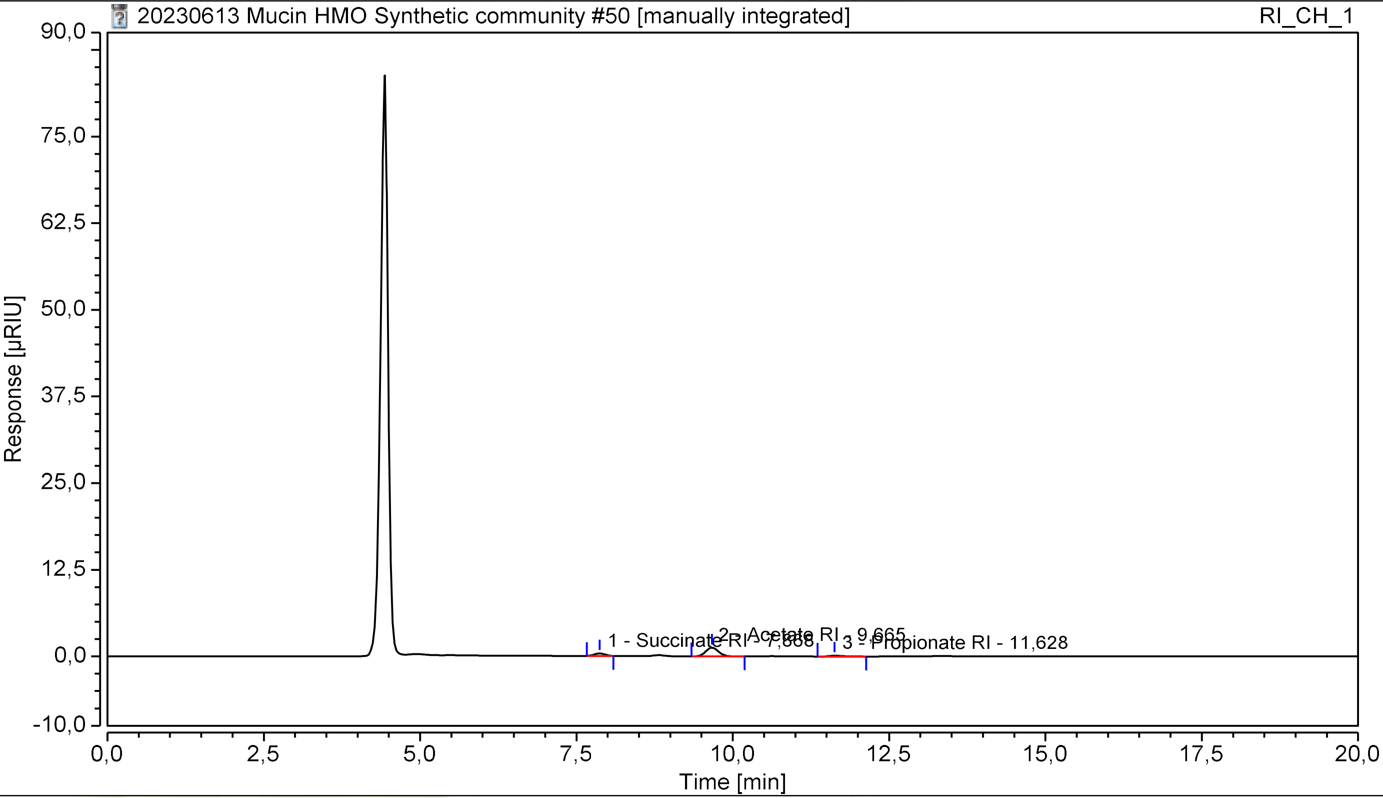

SST Results

No.	Name	Inj.Condition	Peak	Test Result	Injection

**Number of executed test cases:**	**n.a.**	**Total Result:**	**Passed**

Chromatogram

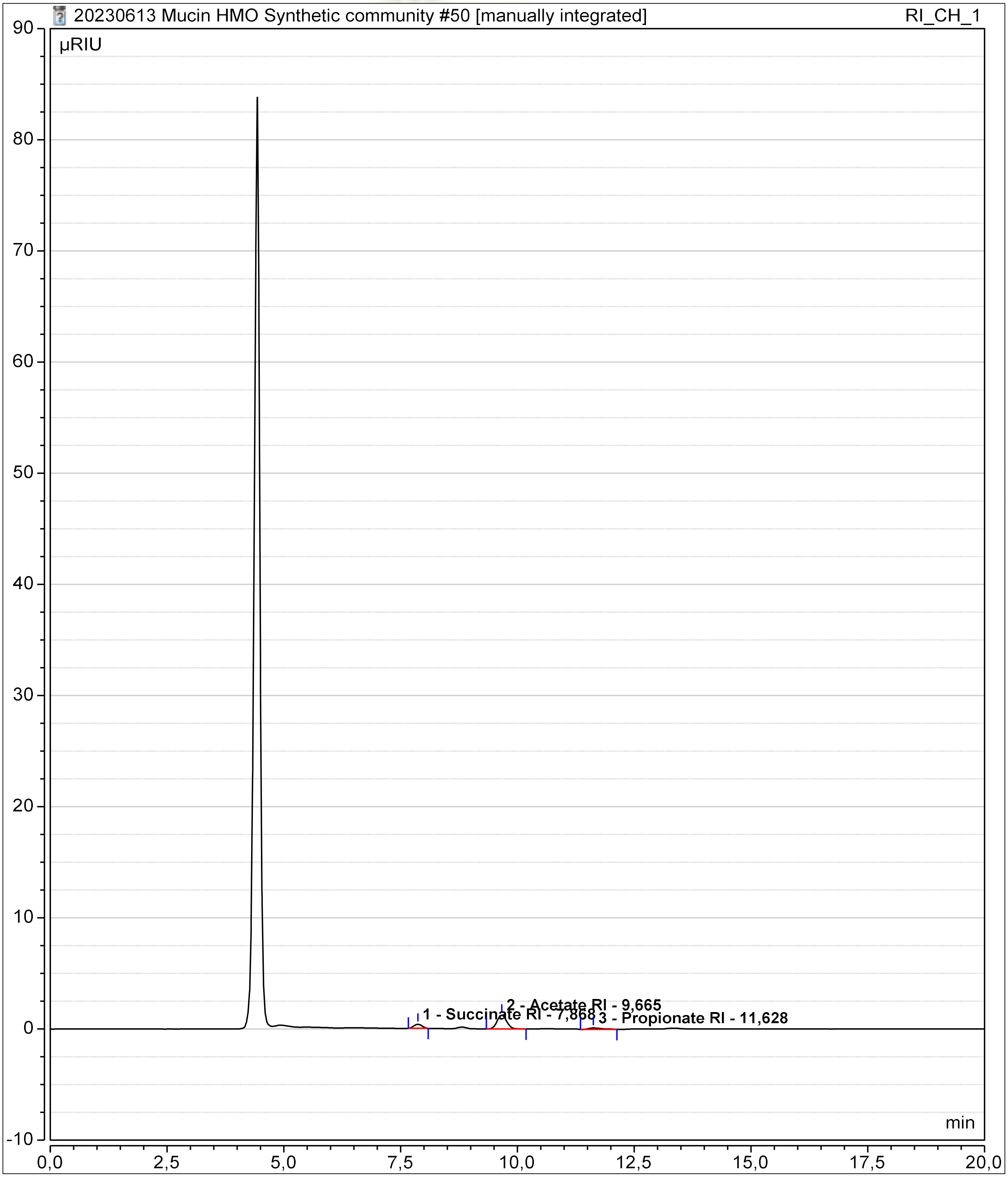

Chromeleon (c) Dionex
Version 7.2.10.25868

Chromatogram and Results

Injection Details

Injection Name:	**14 GOSFOSMUC t24 r2**	*Run Time (min):*	**20,00**
Vial Number:	**3:D8**	*Injection Volume:*	**20,00**
Injection Type:	**Unknown**	*Channel:*	**RI_CH_1**
Calibration Level:		*Wavelength:*	**n.a.**
Instrument Method:	**Default method LC2030C 45 gr 20 min**	*Bandwidth:*	**n.a.**
Processing Method:	**Processing Method LC2030 45 gr**	*Dilution Factor:*	**1,0000**
Injection Date/Time:	**14-jun-23 04:58**	*Sample Weight:*	**1,0000**

Chromatogram

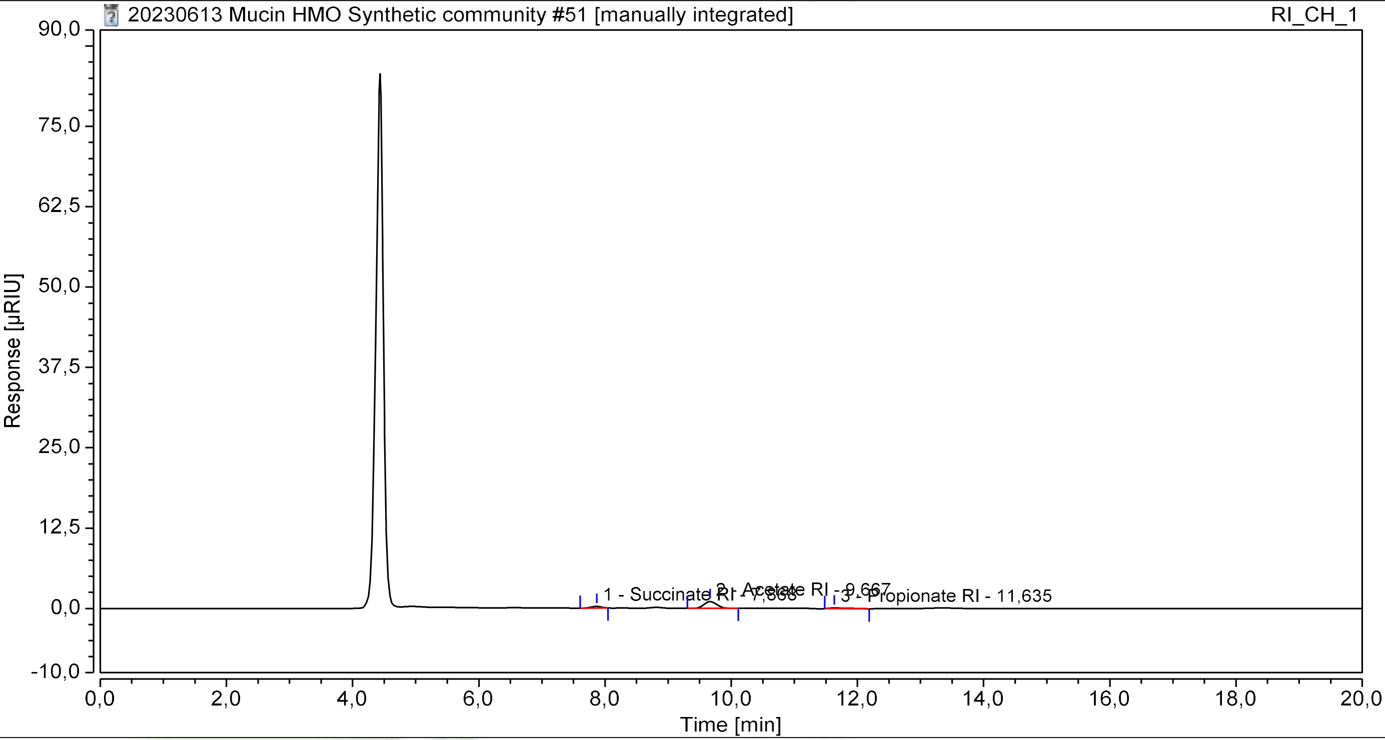

Integration Results

No.	Peak Name	Retention Time min	Area µRIU*min	Height µRIU	Relative Area %	Relative Height %	Amount
n.a.	GlcNAc	n.a.	n.a.	n.a.	n.a.	n.a.	n.a.
n.a.	Citrate	n.a.	n.a.	n.a.	n.a.	n.a.	n.a.
n.a.	Glucose	n.a.	n.a.	n.a.	n.a.	n.a.	n.a.
n.a.	Galactose	n.a.	n.a.	n.a.	n.a.	n.a.	n.a.
n.a.	Fucose	n.a.	n.a.	n.a.	n.a.	n.a.	n.a.
1	Succinate RI	7,868	0,047	0,243	13,80	17,24	n.a.
n.a.	Lactate RI	n.a.	n.a.	n.a.	n.a.	n.a.	n.a.
n.a.	glycerol	n.a.	n.a.	n.a.	n.a.	n.a.	n.a.
n.a.	Formate RI	n.a.	n.a.	n.a.	n.a.	n.a.	n.a.
2	Acetate RI	9,667	0,252	1,057	74,79	75,05	15,5158
n.a.	1,2 PDO RI	n.a.	n.a.	n.a.	n.a.	n.a.	n.a.
n.a.	1,3-PDO	n.a.	n.a.	n.a.	n.a.	n.a.	n.a.
3	Propionate RI	11,635	0,038	0,109	11,41	7,71	1,5476
n.a.	1,3-PDO	n.a.	n.a.	n.a.	n.a.	n.a.	n.a.
n.a.	2-3 BDO	n.a.	n.a.	n.a.	n.a.	n.a.	n.a.
n.a.	Ethanol	n.a.	n.a.	n.a.	n.a.	n.a.	n.a.
n.a.	Isobutyrate RI	n.a.	n.a.	n.a.	n.a.	n.a.	n.a.
n.a.	Butyrate RI	n.a.	n.a.	n.a.	n.a.	n.a.	n.a.
**Total:**			**0,337**	**1,409**	**100,00**	**100,00**	

Peak Analysis

Injection Details

Injection Name:	**14 GOSFOSMUC t24 r2**	*Run Time (min):*	**20,00**
Vial Number:	**3:D8**	*Injection Volume:*	**20,00**
Injection Type:	**Unknown**	*Channel:*	**RI_CH_1**
Calibration Level:		*Wavelength:*	**n.a.**
Instrument Method:	**Default method LC2030C 45 gr 20 min**	*Bandwidth:*	**n.a.**
Processing Method:	**Processing Method LC2030 45 gr**	*Dilution Factor:*	**1,0000**
Injection Date/Time:	**14-jun-23 04:58**	*Sample Weight:*	**1,0000**

Chromatogram

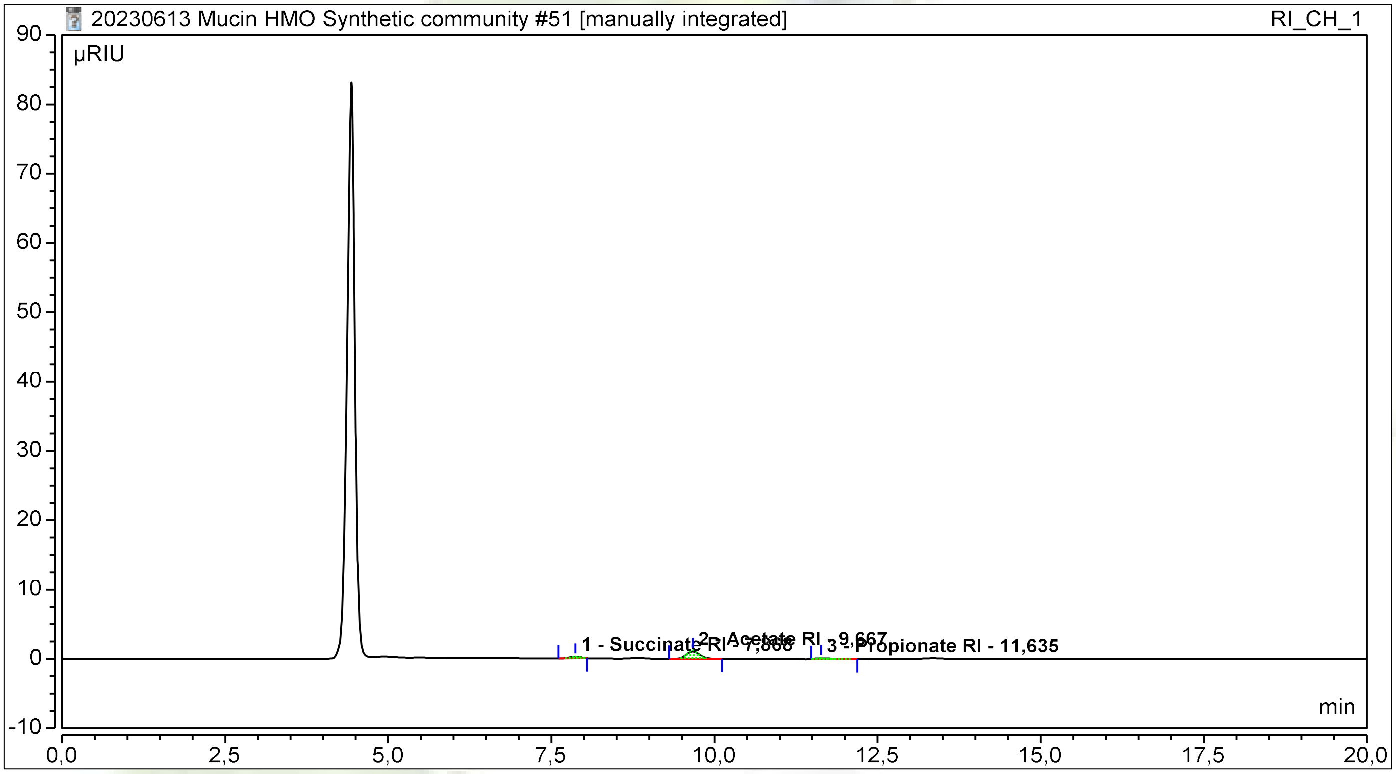

Peak Results

No.	Peak Name	Retention Time min	Width (50%) min	Type	Resolution (EP)	Asymmetry (EP)	Plates (EP)
n.a.	GlcNAc	n.a.	n.a.	n.a.	n.a.	n.a.	n.a.
n.a.	Citrate	n.a.	n.a.	n.a.	n.a.	n.a.	n.a.
n.a.	Glucose	n.a.	n.a.	n.a.	n.a.	n.a.	n.a.
n.a.	Galactose	n.a.	n.a.	n.a.	n.a.	n.a.	n.a.
n.a.	Fucose	n.a.	n.a.	n.a.	n.a.	n.a.	n.a.
1	Succinate RI	7,868	0,189	BMB*	5,13	0,97	9644
n.a.	Lactate RI	n.a.	n.a.	n.a.	n.a.	n.a.	n.a.
n.a.	glycerol	n.a.	n.a.	n.a.	n.a.	n.a.	n.a.
n.a.	Formate RI	n.a.	n.a.	n.a.	n.a.	n.a.	n.a.
2	Acetate RI	9,667	0,225	BMB	4,28	1,08	10198
n.a.	1,2 PDO RI	n.a.	n.a.	n.a.	n.a.	n.a.	n.a.
n.a.	1,3-PDO	n.a.	n.a.	n.a.	n.a.	n.a.	n.a.
3	Propionate RI	11,635	0,317	BMB*	n.a.	2,20	7465
n.a.	1,3-PDO	n.a.	n.a.	n.a.	n.a.	n.a.	n.a.
n.a.	2-3 BDO	n.a.	n.a.	n.a.	n.a.	n.a.	n.a.
n.a.	Ethanol	n.a.	n.a.	n.a.	n.a.	n.a.	n.a.
n.a.	Isobutyrate RI	n.a.	n.a.	n.a.	n.a.	n.a.	n.a.
n.a.	Butyrate RI	n.a.	n.a.	n.a.	n.a.	n.a.	n.a.

Chromatogram and SST Results

Injection Details

Injection Name:	**14 GOSFOSMUC t24 r2**	*Run Time (min):*	**20,00**
Vial Number:	**3:D8**	*Injection Volume:*	**20,00**
Injection Type:	**Unknown**	*Channel:*	**RI_CH_1**
Calibration Level:		*Wavelength:*	**n.a.**
Instrument Method:	**Default method LC2030C 45 gr 20 min**	*Bandwidth:*	**n.a.**
Processing Method:	**Processing Method LC2030 45 gr**	*Dilution Factor:*	**1,0000**
Injection Date/Time:	**14-jun-23 04:58**	*Sample Weight:*	**1,0000**

Chromatogram

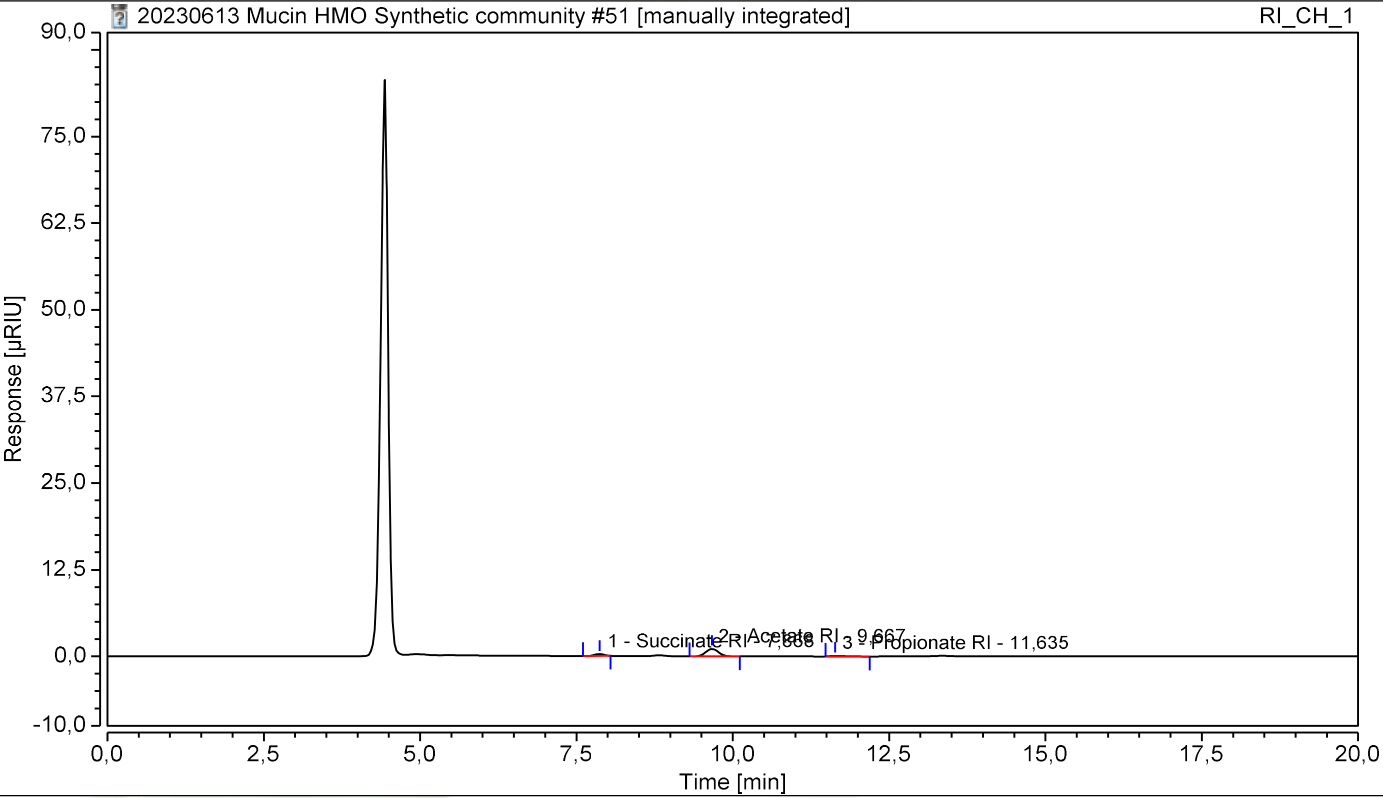

SST Results

No.	Name	Inj.Condition	Peak	Test Result	Injection

Number of executed test cases: **n.a.** **Total Result:** **Passed**

Chromatogram

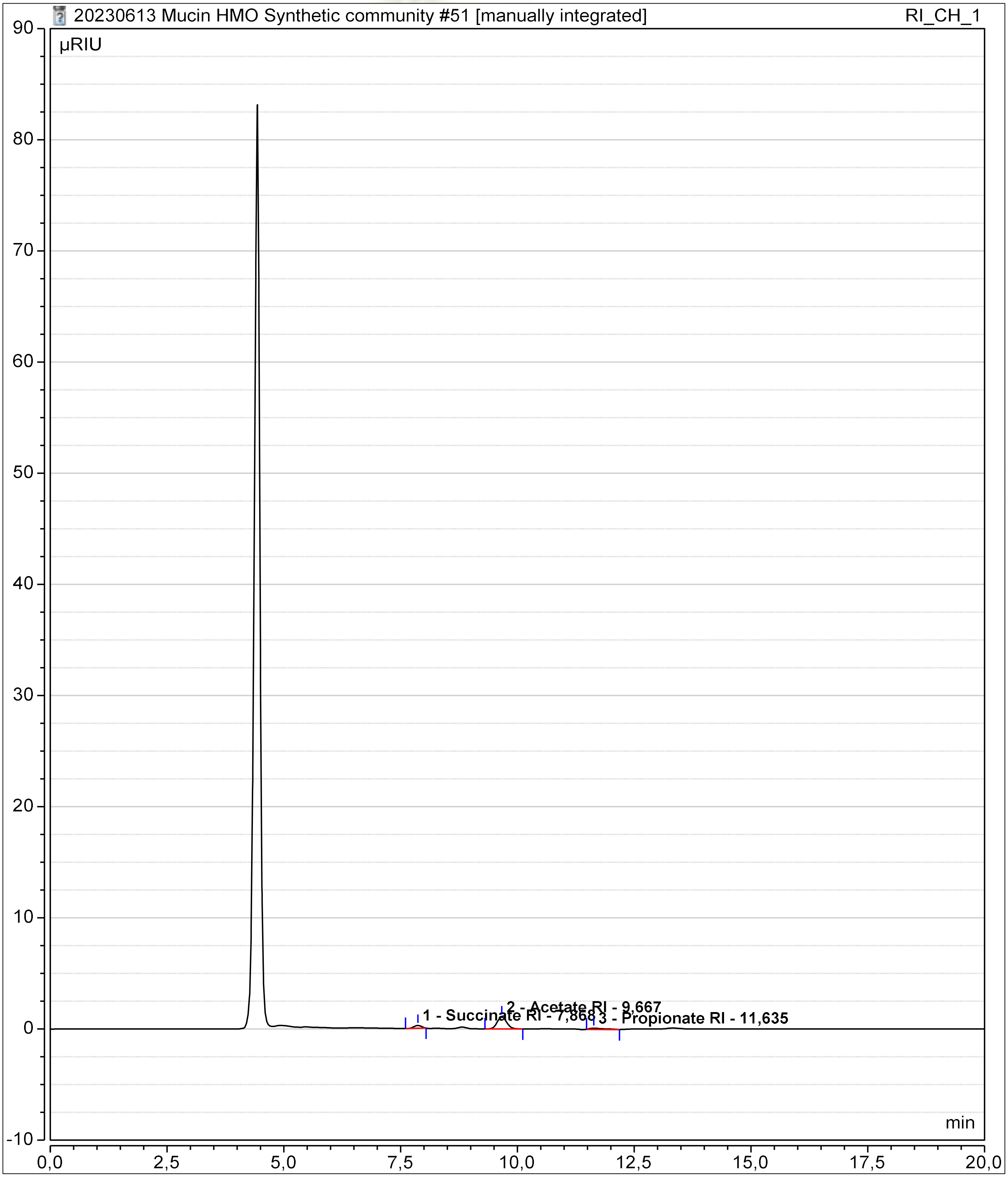

Chromeleon (c) Dionex
Version 7.2.10.25868

Chromatogram and Results

Injection Details

Injection Name:	**15 GOSFOSMUC t24 r3**	*Run Time (min):*	**20,00**
Vial Number:	**3:D9**	*Injection Volume:*	**20,00**
Injection Type:	**Unknown**	*Channel:*	**RI_CH_1**
Calibration Level:		*Wavelength:*	**n.a.**
Instrument Method:	**Default method LC2030C 45 gr 20 min**	*Bandwidth:*	**n.a.**
Processing Method:	**Processing Method LC2030 45 gr**	*Dilution Factor:*	**1,0000**
Injection Date/Time:	**14-jun-23 05:19**	*Sample Weight:*	**1,0000**

Chromatogram

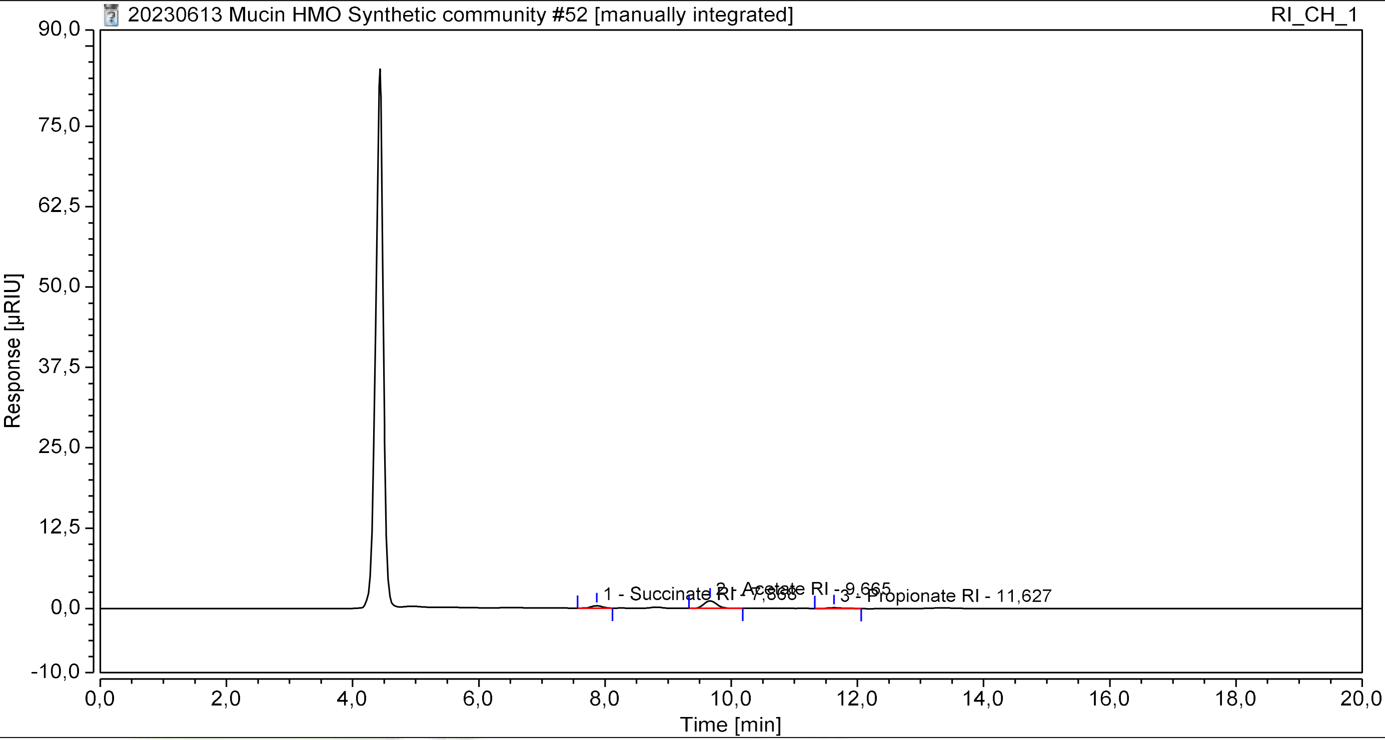

Integration Results

No.	Peak Name	Retention Time min	Area µRIU*min	Height µRIU	Relative Area %	Relative Height %	Amount
n.a.	GlcNAc	n.a.	n.a.	n.a.	n.a.	n.a.	n.a.
n.a.	Citrate	n.a.	n.a.	n.a.	n.a.	n.a.	n.a.
n.a.	Glucose	n.a.	n.a.	n.a.	n.a.	n.a.	n.a.
n.a.	Galactose	n.a.	n.a.	n.a.	n.a.	n.a.	n.a.
n.a.	Fucose	n.a.	n.a.	n.a.	n.a.	n.a.	n.a.
1	Succinate RI	7,868	0,075	0,358	19,02	21,43	n.a.
n.a.	Lactate RI	n.a.	n.a.	n.a.	n.a.	n.a.	n.a.
n.a.	glycerol	n.a.	n.a.	n.a.	n.a.	n.a.	n.a.
n.a.	Formate RI	n.a.	n.a.	n.a.	n.a.	n.a.	n.a.
2	Acetate RI	9,665	0,281	1,179	71,46	70,50	17,2822
n.a.	1,2 PDO RI	n.a.	n.a.	n.a.	n.a.	n.a.	n.a.
n.a.	1,3-PDO	n.a.	n.a.	n.a.	n.a.	n.a.	n.a.
3	Propionate RI	11,627	0,037	0,135	9,52	8,07	1,5057
n.a.	1,3-PDO	n.a.	n.a.	n.a.	n.a.	n.a.	n.a.
n.a.	2-3 BDO	n.a.	n.a.	n.a.	n.a.	n.a.	n.a.
n.a.	Ethanol	n.a.	n.a.	n.a.	n.a.	n.a.	n.a.
n.a.	Isobutyrate RI	n.a.	n.a.	n.a.	n.a.	n.a.	n.a.
n.a.	Butyrate RI	n.a.	n.a.	n.a.	n.a.	n.a.	n.a.
**Total:**			**0,393**	**1,672**	**100,00**	**100,00**	

Chromeleon (c) Dionex
Version 7.2.10.25868

Peak Analysis

Injection Details

Injection Name:	**15 GOSFOSMUC t24 r3**	*Run Time (min):*	**20,00**
Vial Number:	**3:D9**	*Injection Volume:*	**20,00**
Injection Type:	**Unknown**	*Channel:*	**RI_CH_1**
Calibration Level:		*Wavelength:*	**n.a.**
Instrument Method:	**Default method LC2030C 45 gr 20 min**	*Bandwidth:*	**n.a.**
Processing Method:	**Processing Method LC2030 45 gr**	*Dilution Factor:*	**1,0000**
Injection Date/Time:	**14-jun-23 05:19**	*Sample Weight:*	**1,0000**

Chromatogram

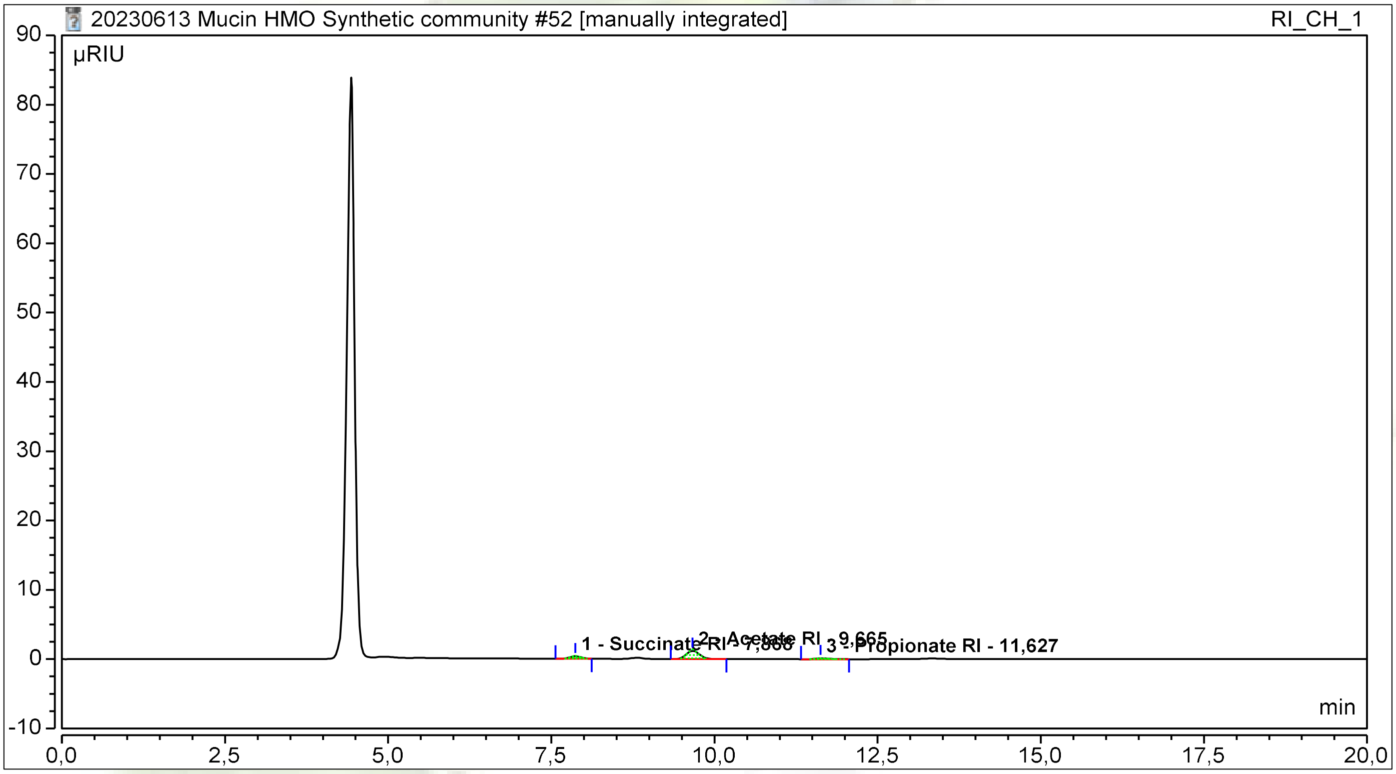

Peak Results

No.	Peak Name	Retention Time min	Width (50%) min	Type	Resolution (EP)	Asymmetry (EP)	Plates (EP)
n.a.	GlcNAc	n.a.	n.a.	n.a.	n.a.	n.a.	n.a.
n.a.	Citrate	n.a.	n.a.	n.a.	n.a.	n.a.	n.a.
n.a.	Glucose	n.a.	n.a.	n.a.	n.a.	n.a.	n.a.
n.a.	Galactose	n.a.	n.a.	n.a.	n.a.	n.a.	n.a.
n.a.	Fucose	n.a.	n.a.	n.a.	n.a.	n.a.	n.a.
1	Succinate RI	7,868	0,200	BMB*	4,99	1,04	8613
n.a.	Lactate RI	n.a.	n.a.	n.a.	n.a.	n.a.	n.a.
n.a.	glycerol	n.a.	n.a.	n.a.	n.a.	n.a.	n.a.
n.a.	Formate RI	n.a.	n.a.	n.a.	n.a.	n.a.	n.a.
2	Acetate RI	9,665	0,225	BMB	4,85	1,08	10185
n.a.	1,2 PDO RI	n.a.	n.a.	n.a.	n.a.	n.a.	n.a.
n.a.	1,3-PDO	n.a.	n.a.	n.a.	n.a.	n.a.	n.a.
3	Propionate RI	11,627	0,252	BMB*	n.a.	1,70	11821
n.a.	1,3-PDO	n.a.	n.a.	n.a.	n.a.	n.a.	n.a.
n.a.	2-3 BDO	n.a.	n.a.	n.a.	n.a.	n.a.	n.a.
n.a.	Ethanol	n.a.	n.a.	n.a.	n.a.	n.a.	n.a.
n.a.	Isobutyrate RI	n.a.	n.a.	n.a.	n.a.	n.a.	n.a.
n.a.	Butyrate RI	n.a.	n.a.	n.a.	n.a.	n.a.	n.a.

Chromeleon (c) Dionex
Version 7.2.10.25868

Chromatogram and SST Results

Injection Details

Injection Name:	**15 GOSFOSMUC t24 r3**	*Run Time (min):*	**20,00**
Vial Number:	**3:D9**	*Injection Volume:*	**20,00**
Injection Type:	**Unknown**	*Channel:*	**RI_CH_1**
Calibration Level:		*Wavelength:*	**n.a.**
Instrument Method:	**Default method LC2030C 45 gr 20 min**	*Bandwidth:*	**n.a.**
Processing Method:	**Processing Method LC2030 45 gr**	*Dilution Factor:*	**1,0000**
Injection Date/Time:	**14-jun-23 05:19**	*Sample Weight:*	**1,0000**

Chromatogram

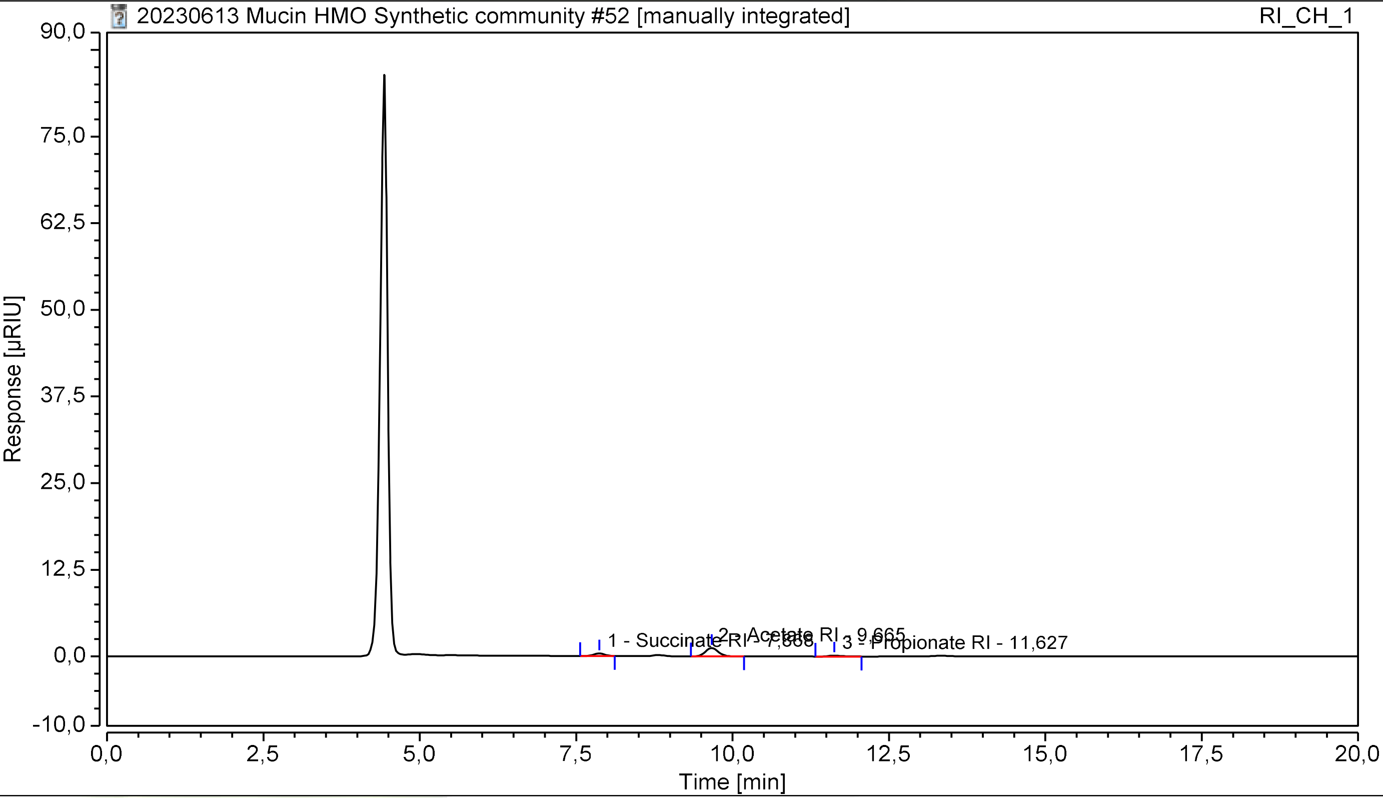

SST Results

No.	Name	Inj.Condition	Peak	Test Result	Injection

Number of executed test cases: **n.a.** **Total Result:** **Passed**

Chromeleon (c) Dionex
Version 7.2.10.25868

Chromatogram

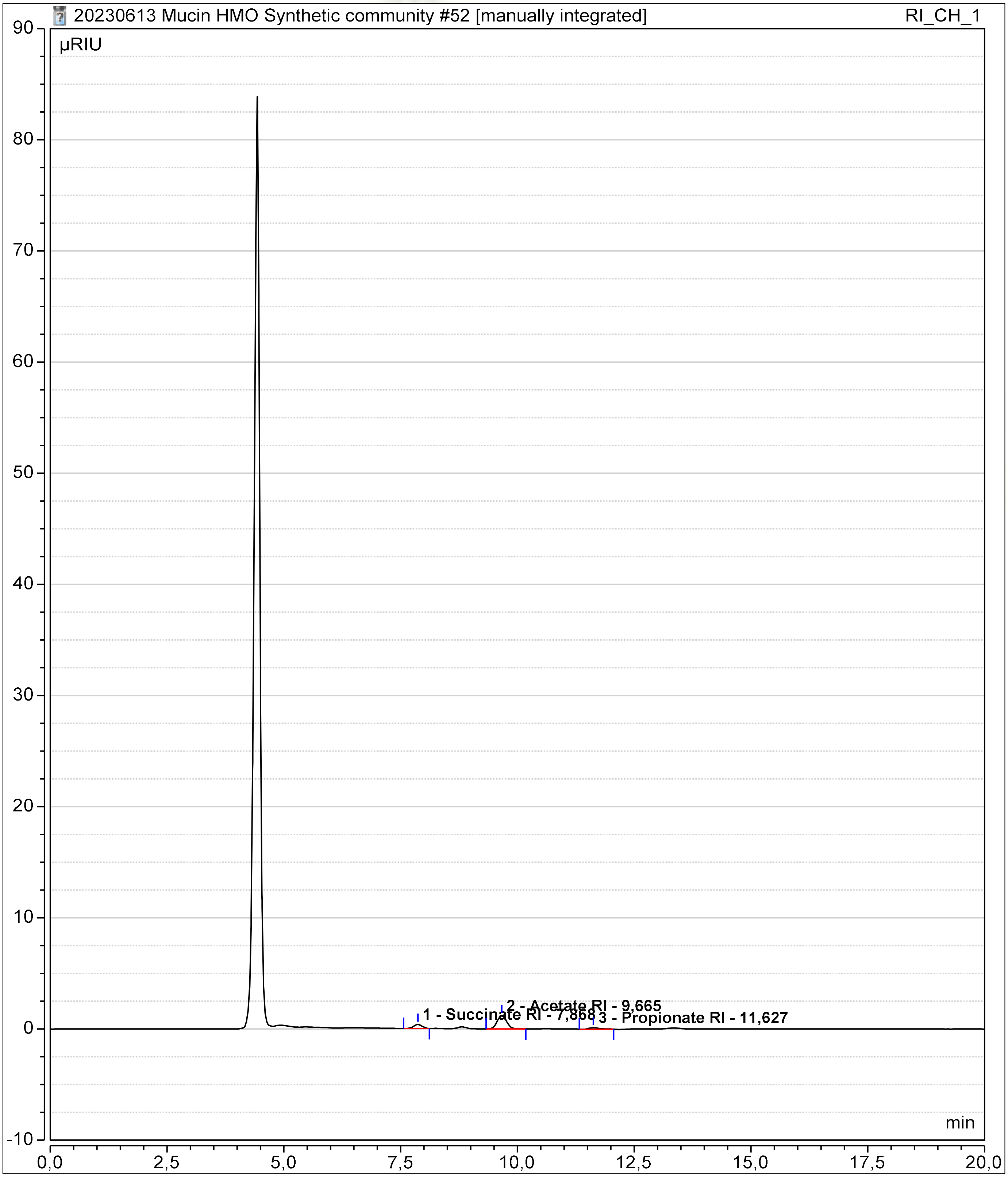

Chromatogram and Results

Injection Details

Injection Name:	**19 MUCHMO2 t48 r1**	*Run Time (min):*	**20,00**
Vial Number:	**3:D10**	*Injection Volume:*	**20,00**
Injection Type:	**Unknown**	*Channel:*	**RI_CH_1**
Calibration Level:		*Wavelength:*	**n.a.**
Instrument Method:	**Default method LC2030C 45 gr 20 min**	*Bandwidth:*	**n.a.**
Processing Method:	**Processing Method LC2030 45 gr**	*Dilution Factor:*	**1,0000**
Injection Date/Time:	**14-jun-23 05:39**	*Sample Weight:*	**1,0000**

Chromatogram

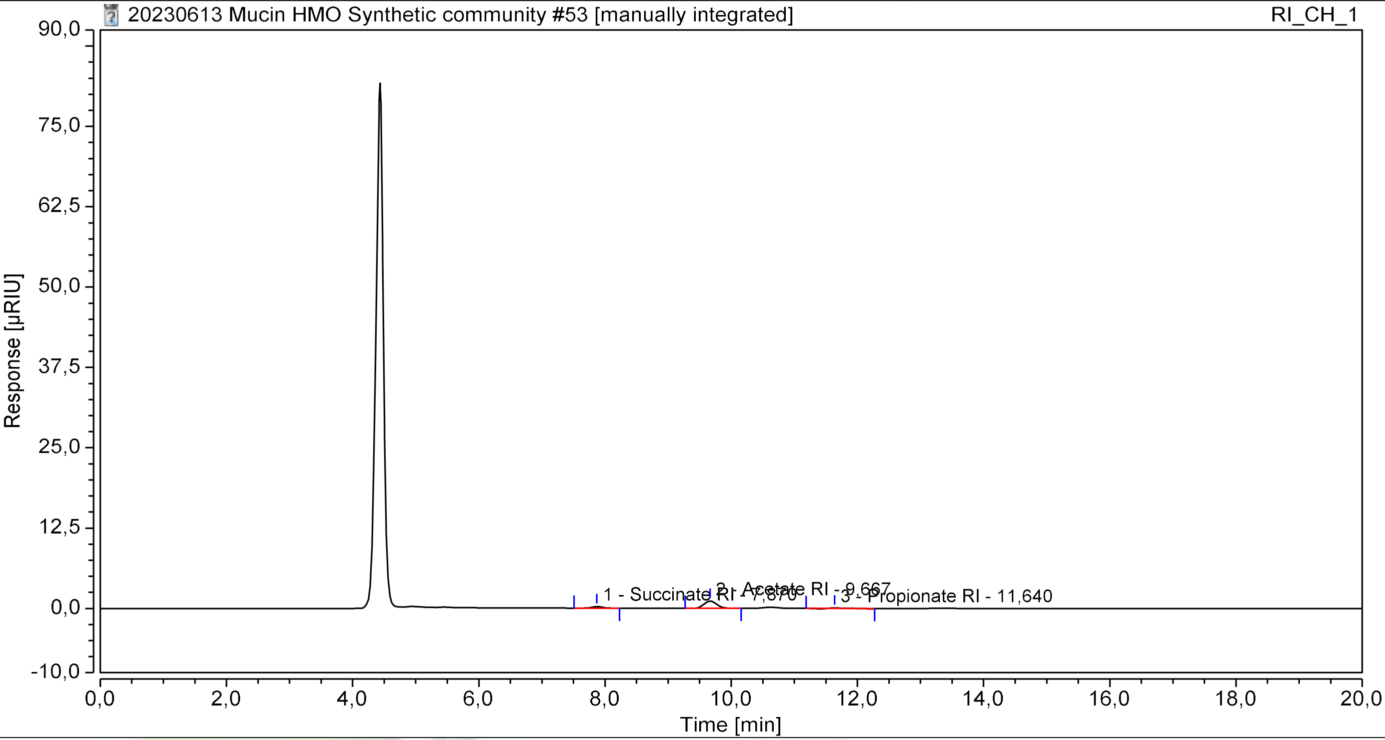

Integration Results

No.	Peak Name	Retention Time min	Area µRIU*min	Height µRIU	Relative Area %	Relative Height %	Amount
n.a.	GlcNAc	n.a.	n.a.	n.a.	n.a.	n.a.	n.a.
n.a.	Citrate	n.a.	n.a.	n.a.	n.a.	n.a.	n.a.
n.a.	Glucose	n.a.	n.a.	n.a.	n.a.	n.a.	n.a.
n.a.	Galactose	n.a.	n.a.	n.a.	n.a.	n.a.	n.a.
n.a.	Fucose	n.a.	n.a.	n.a.	n.a.	n.a.	n.a.
1	Succinate RI	7,870	0,046	0,228	13,93	15,81	n.a.
n.a.	Lactate RI	n.a.	n.a.	n.a.	n.a.	n.a.	n.a.
n.a.	glycerol	n.a.	n.a.	n.a.	n.a.	n.a.	n.a.
n.a.	Formate RI	n.a.	n.a.	n.a.	n.a.	n.a.	n.a.
2	Acetate RI	9,667	0,268	1,127	81,62	78,24	16,5124
n.a.	1,2 PDO RI	n.a.	n.a.	n.a.	n.a.	n.a.	n.a.
n.a.	1,3-PDO	n.a.	n.a.	n.a.	n.a.	n.a.	n.a.
3	Propionate RI	11,640	0,015	0,086	4,45	5,94	0,5886
n.a.	1,3-PDO	n.a.	n.a.	n.a.	n.a.	n.a.	n.a.
n.a.	2-3 BDO	n.a.	n.a.	n.a.	n.a.	n.a.	n.a.
n.a.	Ethanol	n.a.	n.a.	n.a.	n.a.	n.a.	n.a.
n.a.	Isobutyrate RI	n.a.	n.a.	n.a.	n.a.	n.a.	n.a.
n.a.	Butyrate RI	n.a.	n.a.	n.a.	n.a.	n.a.	n.a.
**Total:**			**0,329**	**1,440**	**100,00**	**100,00**	

Peak Analysis

Injection Details

Injection Name:	**19 MUCHMO2 t48 r1**	*Run Time (min):*	**20,00**
Vial Number:	**3:D10**	*Injection Volume:*	**20,00**
Injection Type:	**Unknown**	*Channel:*	**RI_CH_1**
Calibration Level:		*Wavelength:*	**n.a.**
Instrument Method:	**Default method LC2030C 45 gr 20 min**	*Bandwidth:*	**n.a.**
Processing Method:	**Processing Method LC2030 45 gr**	*Dilution Factor:*	**1,0000**
Injection Date/Time:	**14-jun-23 05:39**	*Sample Weight:*	**1,0000**

Chromatogram

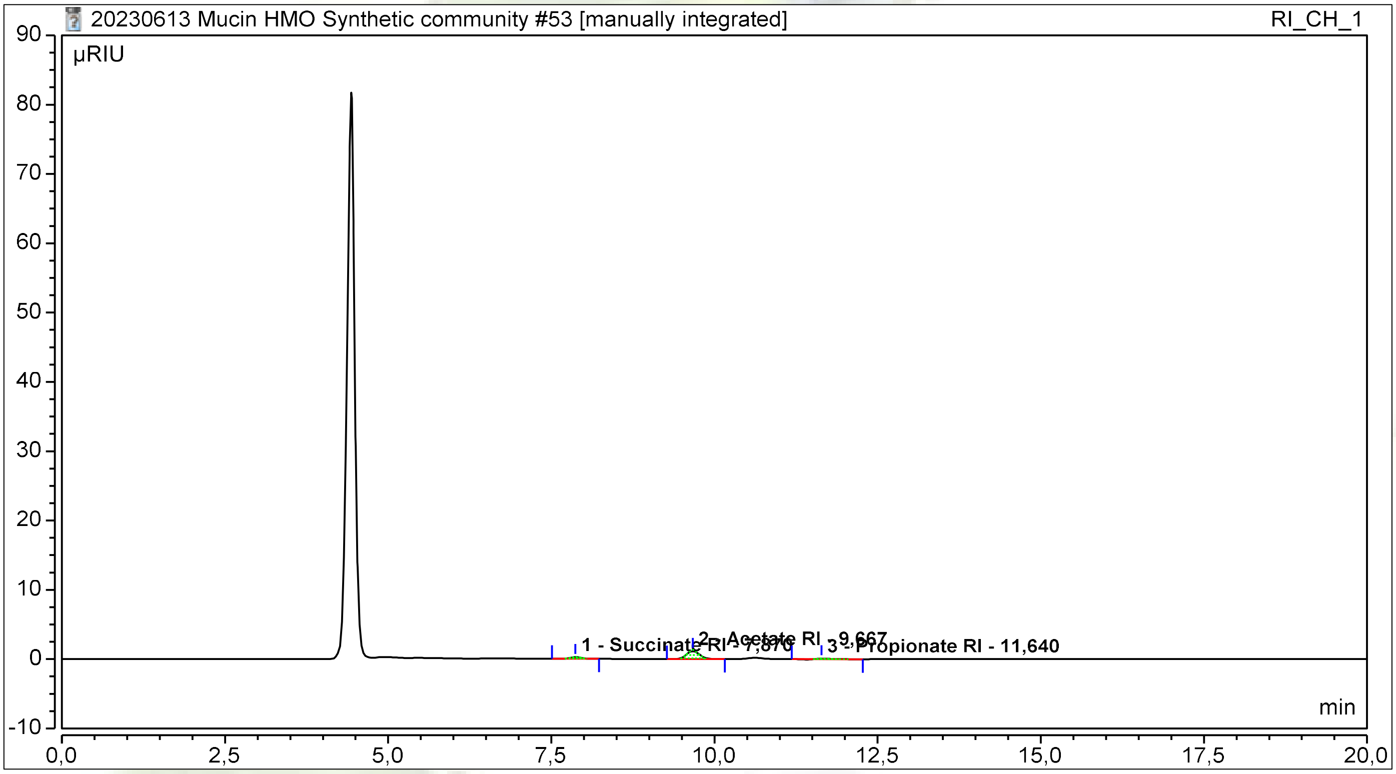

Peak Results

No.	Peak Name	Retention Time<br>min	Width (50%)<br>min	Type	Resolution (EP)	Asymmetry (EP)	Plates (EP)
n.a.	GlcNAc	n.a.	n.a.	n.a.	n.a.	n.a.	n.a.
n.a.	Citrate	n.a.	n.a.	n.a.	n.a.	n.a.	n.a.
n.a.	Glucose	n.a.	n.a.	n.a.	n.a.	n.a.	n.a.
n.a.	Galactose	n.a.	n.a.	n.a.	n.a.	n.a.	n.a.
n.a.	Fucose	n.a.	n.a.	n.a.	n.a.	n.a.	n.a.
1	Succinate RI	7,870	0,197	BMB*	5,02	1,05	8840
n.a.	Lactate RI	n.a.	n.a.	n.a.	n.a.	n.a.	n.a.
n.a.	glycerol	n.a.	n.a.	n.a.	n.a.	n.a.	n.a.
n.a.	Formate RI	n.a.	n.a.	n.a.	n.a.	n.a.	n.a.
2	Acetate RI	9,667	0,225	BMB	4,84	1,08	10228
n.a.	1,2 PDO RI	n.a.	n.a.	n.a.	n.a.	n.a.	n.a.
n.a.	1,3-PDO	n.a.	n.a.	n.a.	n.a.	n.a.	n.a.
3	Propionate RI	11,640	0,256	BMB*	n.a.	2,26	11415
n.a.	1,3-PDO	n.a.	n.a.	n.a.	n.a.	n.a.	n.a.
n.a.	2-3 BDO	n.a.	n.a.	n.a.	n.a.	n.a.	n.a.
n.a.	Ethanol	n.a.	n.a.	n.a.	n.a.	n.a.	n.a.
n.a.	Isobutyrate RI	n.a.	n.a.	n.a.	n.a.	n.a.	n.a.
n.a.	Butyrate RI	n.a.	n.a.	n.a.	n.a.	n.a.	n.a.

Chromatogram and SST Results

Injection Details

Injection Name:	**19 MUCHMO2 t48 r1**	*Run Time (min):*	**20,00**
Vial Number:	**3:D10**	*Injection Volume:*	**20,00**
Injection Type:	**Unknown**	*Channel:*	**RI_CH_1**
Calibration Level:		*Wavelength:*	**n.a.**
Instrument Method:	**Default method LC2030C 45 gr 20 min**	*Bandwidth:*	**n.a.**
Processing Method:	**Processing Method LC2030 45 gr**	*Dilution Factor:*	**1,0000**
Injection Date/Time:	**14-jun-23 05:39**	*Sample Weight:*	**1,0000**

Chromatogram

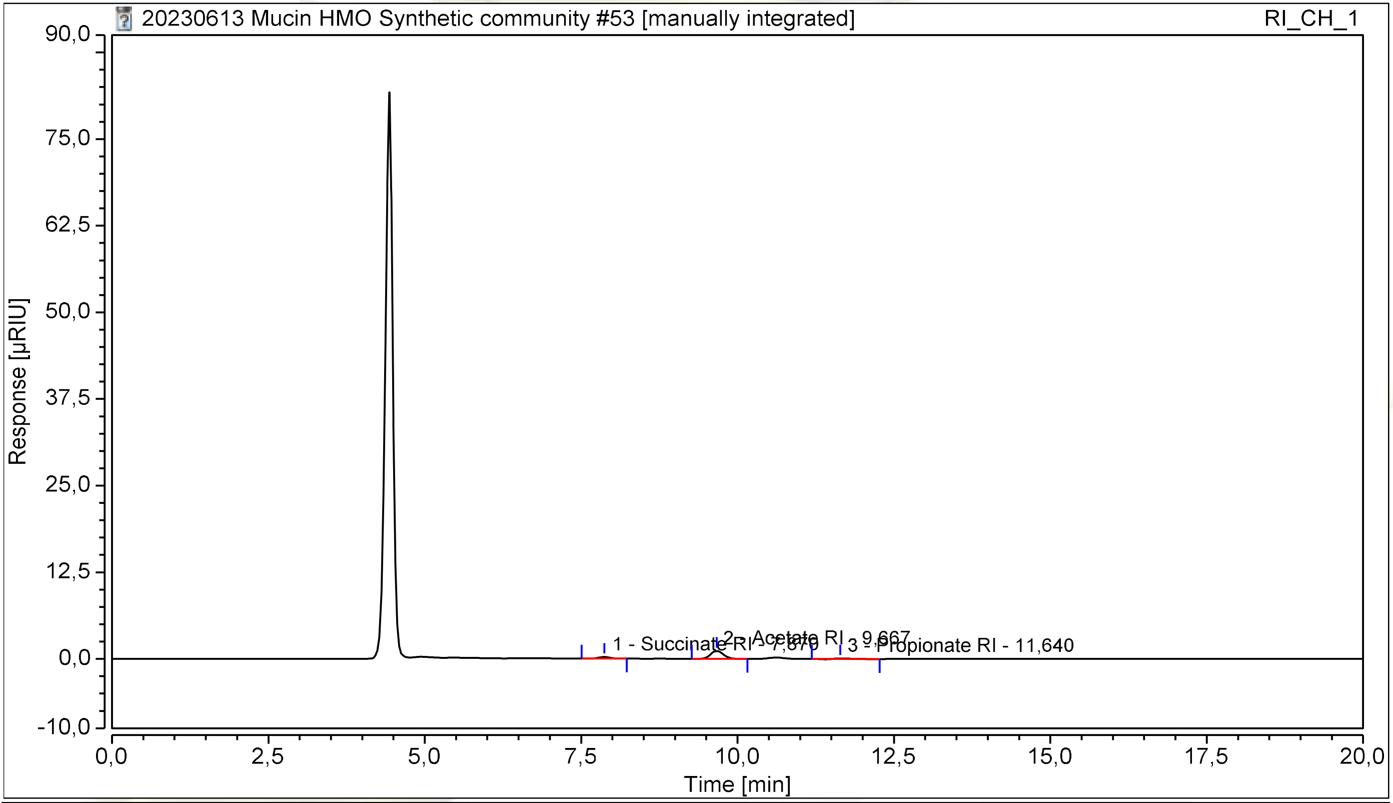

SST Results

No.	Name	Inj.Condition	Peak	Test Result	Injection

Number of executed test cases: **n.a.** **Total Result:** **Passed**

Chromatogram

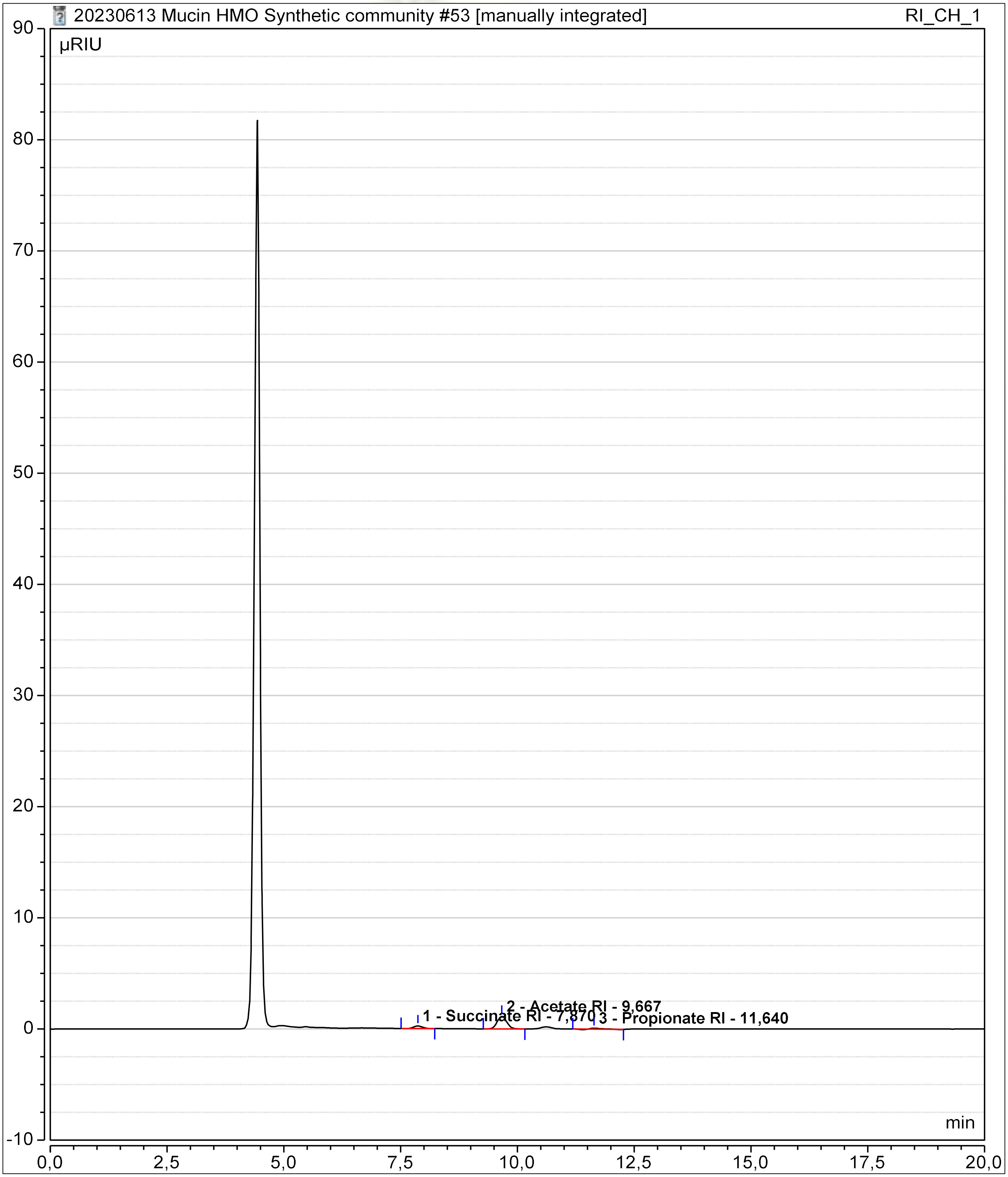

Chromatogram and Results

Injection Details

Injection Name:	**20 MUCHMO2 t48 r2**	*Run Time (min):*	**20,00**
Vial Number:	**3:D11**	*Injection Volume:*	**20,00**
Injection Type:	**Unknown**	*Channel:*	**RI_CH_1**
Calibration Level:		*Wavelength:*	**n.a.**
Instrument Method:	**Default method LC2030C 45 gr 20 min**	*Bandwidth:*	**n.a.**
Processing Method:	**Processing Method LC2030 45 gr**	*Dilution Factor:*	**1,0000**
Injection Date/Time:	**14-jun-23 05:59**	*Sample Weight:*	**1,0000**

Chromatogram

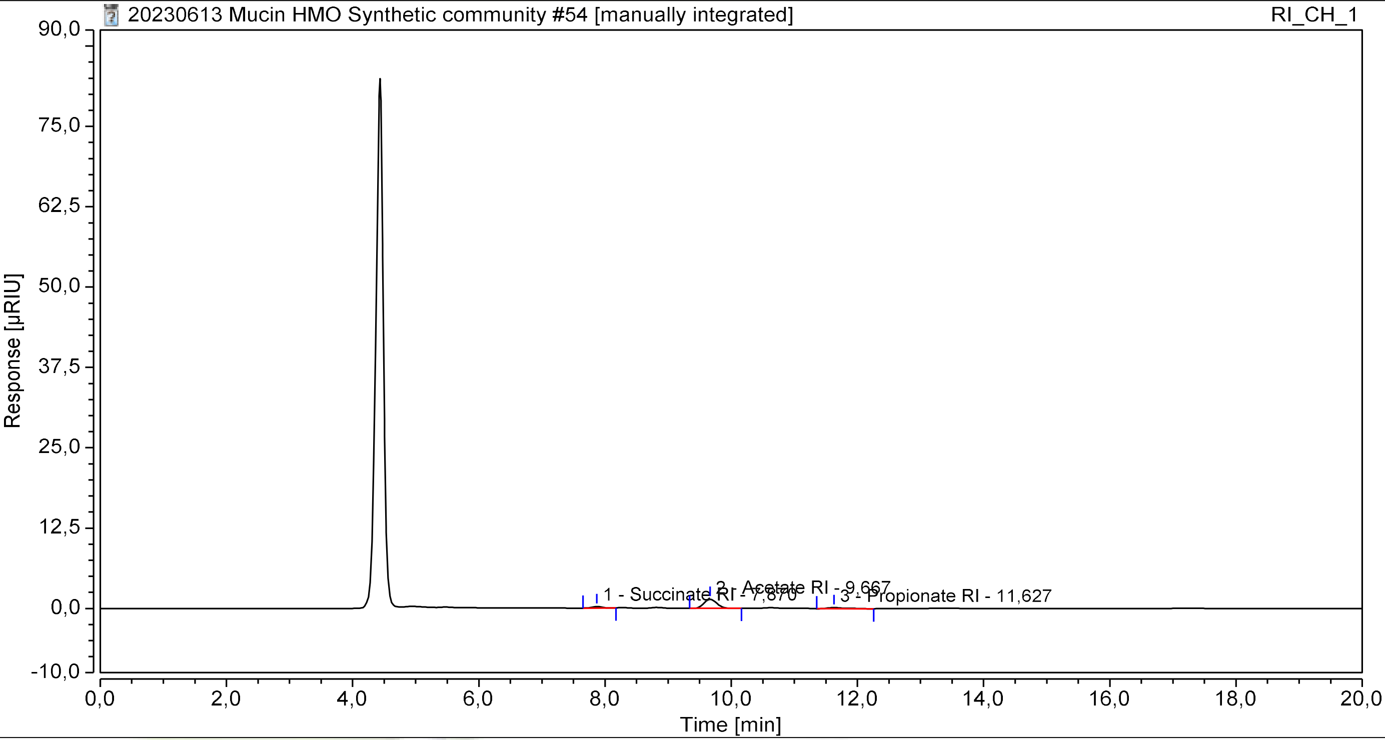

Integration Results

No.	Peak Name	Retention Time min	Area µRIU*min	Height µRIU	Relative Area %	Relative Height %	Amount
n.a.	GlcNAc	n.a.	n.a.	n.a.	n.a.	n.a.	n.a.
n.a.	Citrate	n.a.	n.a.	n.a.	n.a.	n.a.	n.a.
n.a.	Glucose	n.a.	n.a.	n.a.	n.a.	n.a.	n.a.
n.a.	Galactose	n.a.	n.a.	n.a.	n.a.	n.a.	n.a.
n.a.	Fucose	n.a.	n.a.	n.a.	n.a.	n.a.	n.a.
1	Succinate RI	7,870	0,036	0,208	8,57	11,71	n.a.
n.a.	Lactate RI	n.a.	n.a.	n.a.	n.a.	n.a.	n.a.
n.a.	glycerol	n.a.	n.a.	n.a.	n.a.	n.a.	n.a.
n.a.	Formate RI	n.a.	n.a.	n.a.	n.a.	n.a.	n.a.
2	Acetate RI	9,667	0,335	1,406	78,74	79,20	20,6130
n.a.	1,2 PDO RI	n.a.	n.a.	n.a.	n.a.	n.a.	n.a.
n.a.	1,3-PDO	n.a.	n.a.	n.a.	n.a.	n.a.	n.a.
3	Propionate RI	11,627	0,054	0,162	12,69	9,10	2,1710
n.a.	1,3-PDO	n.a.	n.a.	n.a.	n.a.	n.a.	n.a.
n.a.	2-3 BDO	n.a.	n.a.	n.a.	n.a.	n.a.	n.a.
n.a.	Ethanol	n.a.	n.a.	n.a.	n.a.	n.a.	n.a.
n.a.	Isobutyrate RI	n.a.	n.a.	n.a.	n.a.	n.a.	n.a.
n.a.	Butyrate RI	n.a.	n.a.	n.a.	n.a.	n.a.	n.a.
**Total:**			**0,425**	**1,776**	**100,00**	**100,00**	

Peak Analysis

Injection Details

Injection Name:	**20 MUCHMO2 t48 r2**	*Run Time (min):*	**20,00**
Vial Number:	**3:D11**	*Injection Volume:*	**20,00**
Injection Type:	**Unknown**	*Channel:*	**RI_CH_1**
Calibration Level:		*Wavelength:*	**n.a.**
Instrument Method:	**Default method LC2030C 45 gr 20 min**	*Bandwidth:*	**n.a.**
Processing Method:	**Processing Method LC2030 45 gr**	*Dilution Factor:*	**1,0000**
Injection Date/Time:	**14-jun-23 05:59**	*Sample Weight:*	**1,0000**

Chromatogram

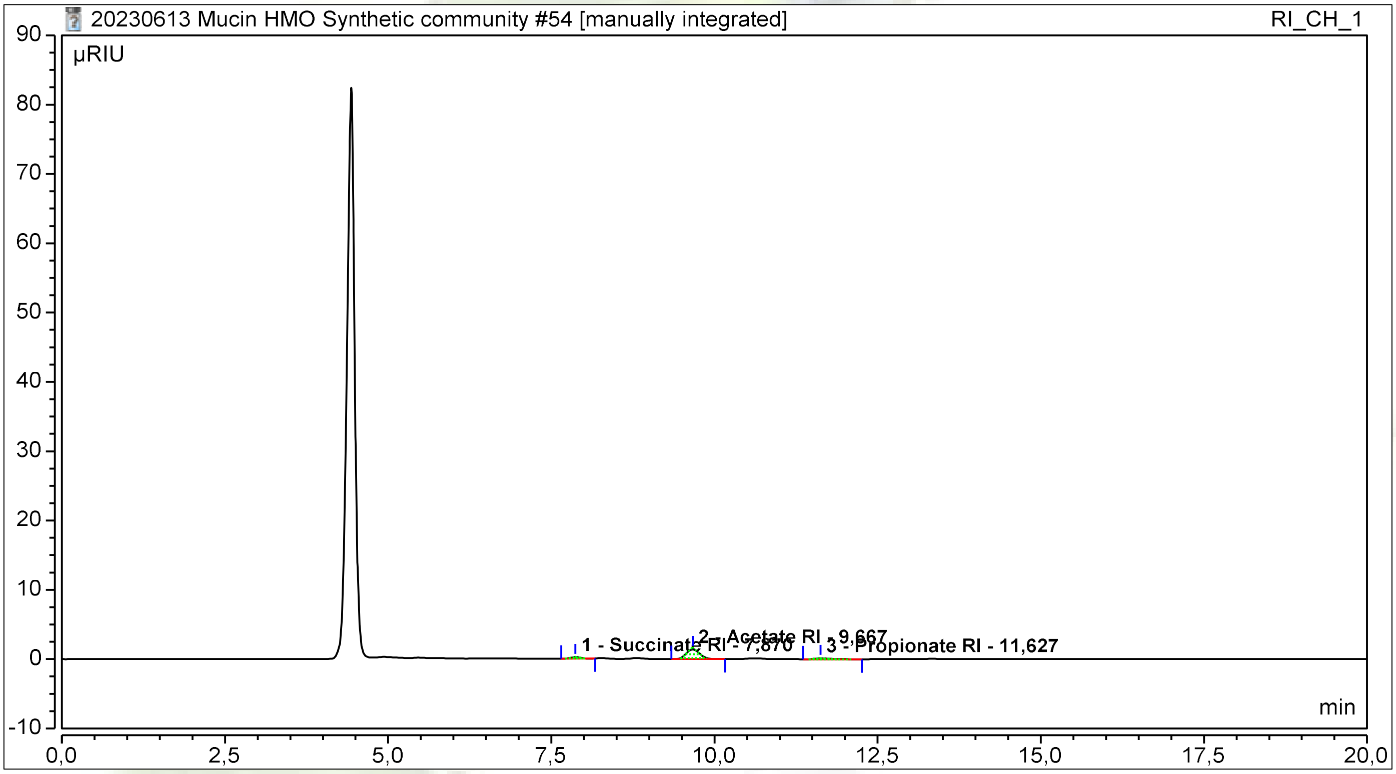

Peak Results

No.	Peak Name	Retention Time min	Width (50%) min	Type	Resolution (EP)	Asymmetry (EP)	Plates (EP)
n.a.	GlcNAc	n.a.	n.a.	n.a.	n.a.	n.a.	n.a.
n.a.	Citrate	n.a.	n.a.	n.a.	n.a.	n.a.	n.a.
n.a.	Glucose	n.a.	n.a.	n.a.	n.a.	n.a.	n.a.
n.a.	Galactose	n.a.	n.a.	n.a.	n.a.	n.a.	n.a.
n.a.	Fucose	n.a.	n.a.	n.a.	n.a.	n.a.	n.a.
1	Succinate RI	7,870	0,185	BMB*	5,16	0,94	9978
n.a.	Lactate RI	n.a.	n.a.	n.a.	n.a.	n.a.	n.a.
n.a.	glycerol	n.a.	n.a.	n.a.	n.a.	n.a.	n.a.
n.a.	Formate RI	n.a.	n.a.	n.a.	n.a.	n.a.	n.a.
2	Acetate RI	9,667	0,225	BMB	4,56	1,08	10213
n.a.	1,2 PDO RI	n.a.	n.a.	n.a.	n.a.	n.a.	n.a.
n.a.	1,3-PDO	n.a.	n.a.	n.a.	n.a.	n.a.	n.a.
3	Propionate RI	11,627	0,282	BMB*	n.a.	1,79	9403
n.a.	1,3-PDO	n.a.	n.a.	n.a.	n.a.	n.a.	n.a.
n.a.	2-3 BDO	n.a.	n.a.	n.a.	n.a.	n.a.	n.a.
n.a.	Ethanol	n.a.	n.a.	n.a.	n.a.	n.a.	n.a.
n.a.	Isobutyrate RI	n.a.	n.a.	n.a.	n.a.	n.a.	n.a.
n.a.	Butyrate RI	n.a.	n.a.	n.a.	n.a.	n.a.	n.a.

Chromeleon (c) Dionex
Version 7.2.10.25868

Chromatogram and SST Results

Injection Details

Injection Name:	**20 MUCHMO2 t48 r2**	*Run Time (min):*	**20,00**
Vial Number:	**3:D11**	*Injection Volume:*	**20,00**
Injection Type:	**Unknown**	*Channel:*	**RI_CH_1**
Calibration Level:		*Wavelength:*	**n.a.**
Instrument Method:	**Default method LC2030C 45 gr 20 min**	*Bandwidth:*	**n.a.**
Processing Method:	**Processing Method LC2030 45 gr**	*Dilution Factor:*	**1,0000**
Injection Date/Time:	**14-jun-23 05:59**	*Sample Weight:*	**1,0000**

Chromatogram

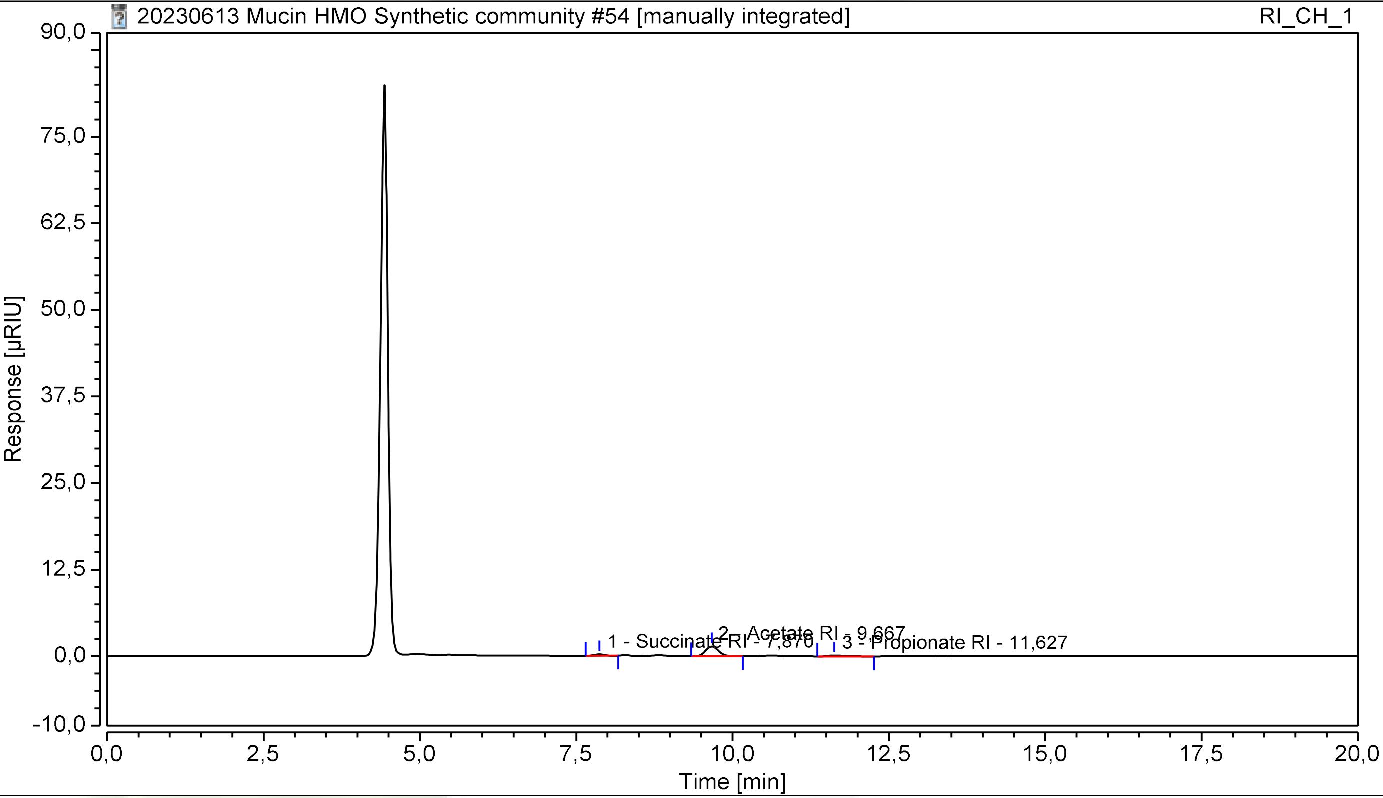

SST Results

No.	Name	Inj.Condition	Peak	Test Result	Injection

Number of executed test cases: **n.a.** **Total Result:** **Passed**

Chromatogram

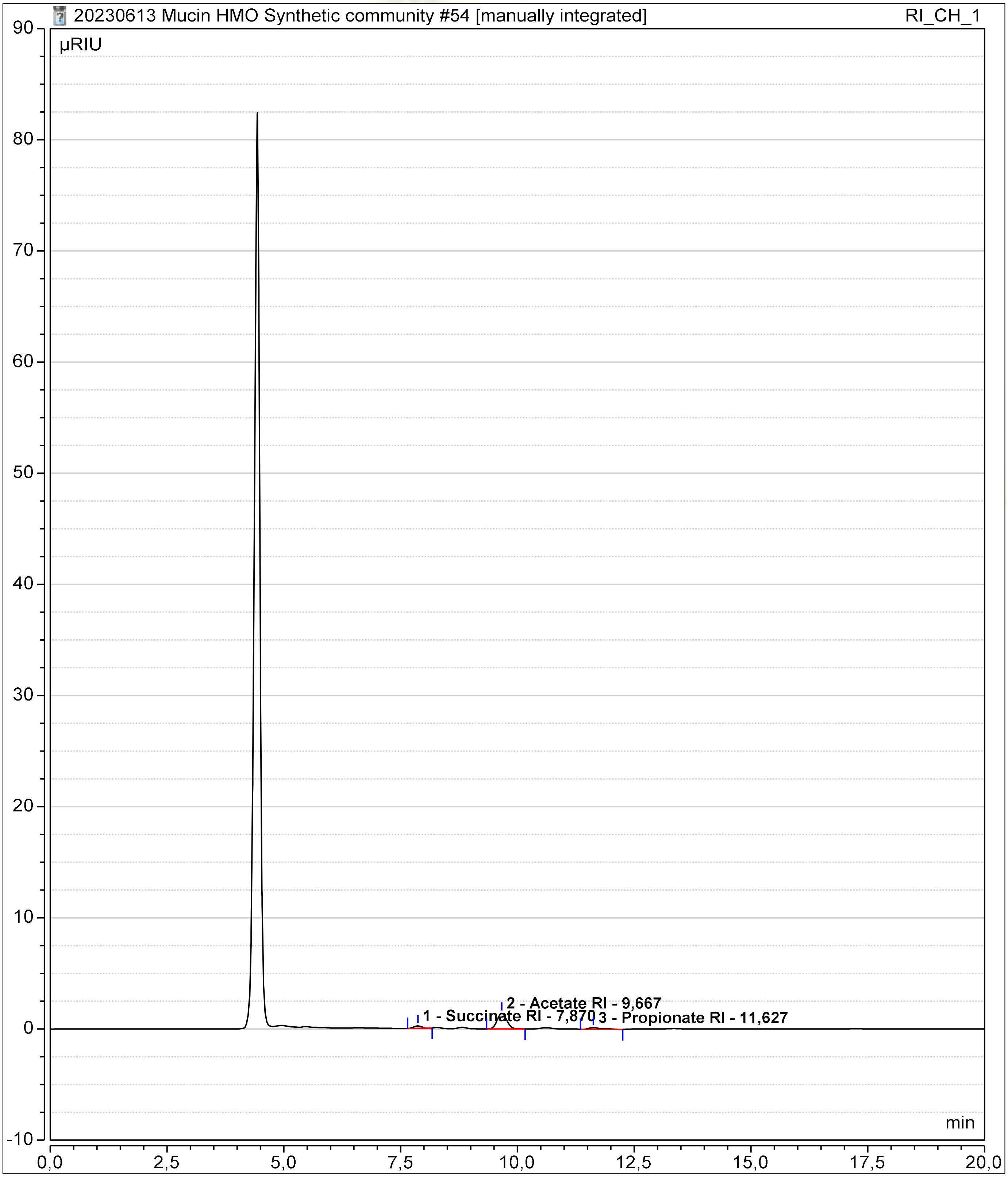

Chromeleon (c) Dionex
Version 7.2.10.25868

Chromatogram and Results

Injection Details

Injection Name:	**21 MUCHMO2 t48 r3**	*Run Time (min):*	**20,00**
Vial Number:	**3:D12**	*Injection Volume:*	**20,00**
Injection Type:	**Unknown**	*Channel:*	**RI_CH_1**
Calibration Level:		*Wavelength:*	**n.a.**
Instrument Method:	**Default method LC2030C 45 gr 20 min**	*Bandwidth:*	**n.a.**
Processing Method:	**Processing Method LC2030 45 gr**	*Dilution Factor:*	**1,0000**
Injection Date/Time:	**14-jun-23 06:20**	*Sample Weight:*	**1,0000**

Chromatogram

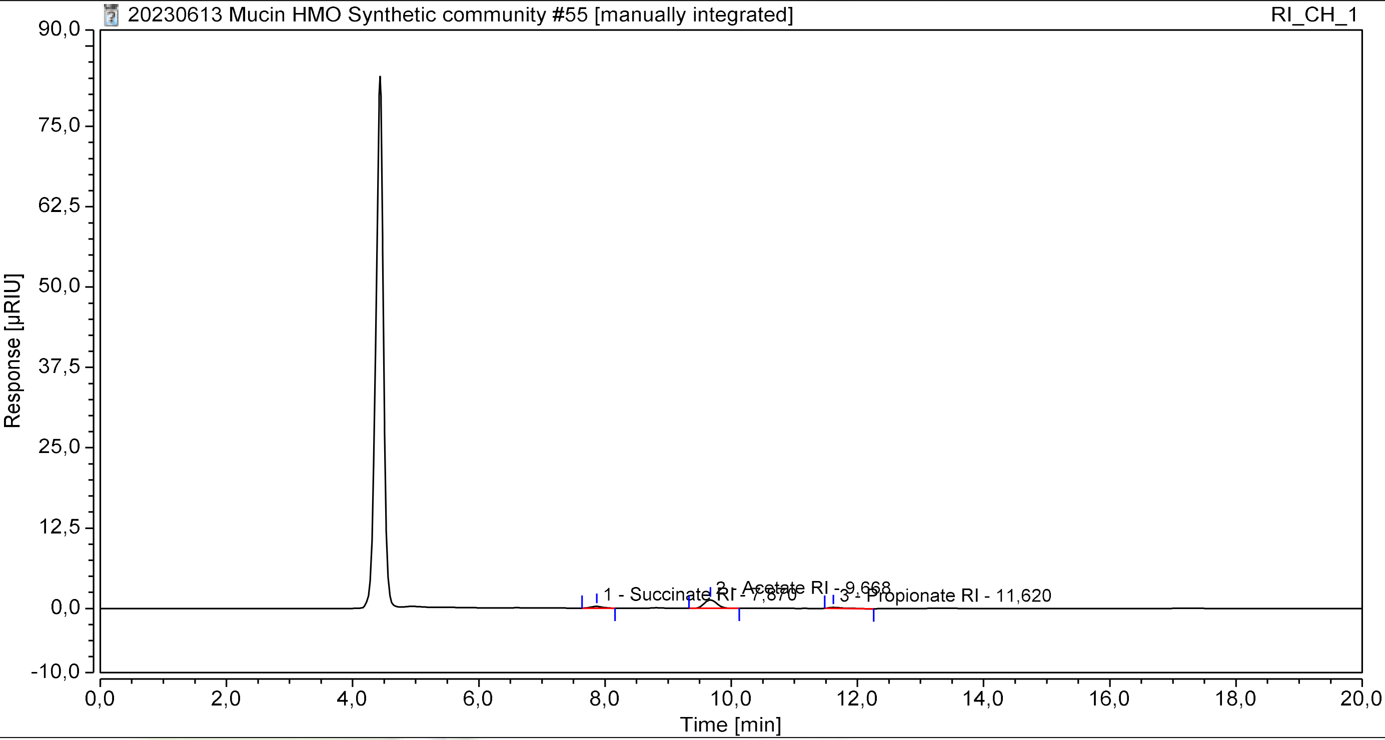

Integration Results

No.	Peak Name	Retention Time min	Area µRIU*min	Height µRIU	Relative Area %	Relative Height %	Amount
n.a.	GlcNAc	n.a.	n.a.	n.a.	n.a.	n.a.	n.a.
n.a.	Citrate	n.a.	n.a.	n.a.	n.a.	n.a.	n.a.
n.a.	Glucose	n.a.	n.a.	n.a.	n.a.	n.a.	n.a.
n.a.	Galactose	n.a.	n.a.	n.a.	n.a.	n.a.	n.a.
n.a.	Fucose	n.a.	n.a.	n.a.	n.a.	n.a.	n.a.
1	Succinate RI	7,870	0,056	0,268	13,72	15,52	n.a.
n.a.	Lactate RI	n.a.	n.a.	n.a.	n.a.	n.a.	n.a.
n.a.	glycerol	n.a.	n.a.	n.a.	n.a.	n.a.	n.a.
n.a.	Formate RI	n.a.	n.a.	n.a.	n.a.	n.a.	n.a.
2	Acetate RI	9,668	0,313	1,313	76,97	76,09	19,2456
n.a.	1,2 PDO RI	n.a.	n.a.	n.a.	n.a.	n.a.	n.a.
n.a.	1,3-PDO	n.a.	n.a.	n.a.	n.a.	n.a.	n.a.
3	Propionate RI	11,620	0,038	0,145	9,31	8,39	1,5213
n.a.	1,3-PDO	n.a.	n.a.	n.a.	n.a.	n.a.	n.a.
n.a.	2-3 BDO	n.a.	n.a.	n.a.	n.a.	n.a.	n.a.
n.a.	Ethanol	n.a.	n.a.	n.a.	n.a.	n.a.	n.a.
n.a.	Isobutyrate RI	n.a.	n.a.	n.a.	n.a.	n.a.	n.a.
n.a.	Butyrate RI	n.a.	n.a.	n.a.	n.a.	n.a.	n.a.
**Total:**			**0,406**	**1,725**	**100,00**	**100,00**	

Chromeleon (c) Dionex
Version 7.2.10.25868

Peak Analysis

Injection Details

Injection Name:	**21 MUCHMO2 t48 r3**	*Run Time (min):*	**20,00**
Vial Number:	**3:D12**	*Injection Volume:*	**20,00**
Injection Type:	**Unknown**	*Channel:*	**RI_CH_1**
Calibration Level:		*Wavelength:*	**n.a.**
Instrument Method:	**Default method LC2030C 45 gr 20 min**	*Bandwidth:*	**n.a.**
Processing Method:	**Processing Method LC2030 45 gr**	*Dilution Factor:*	**1,0000**
Injection Date/Time:	**14-jun-23 06:20**	*Sample Weight:*	**1,0000**

Chromatogram

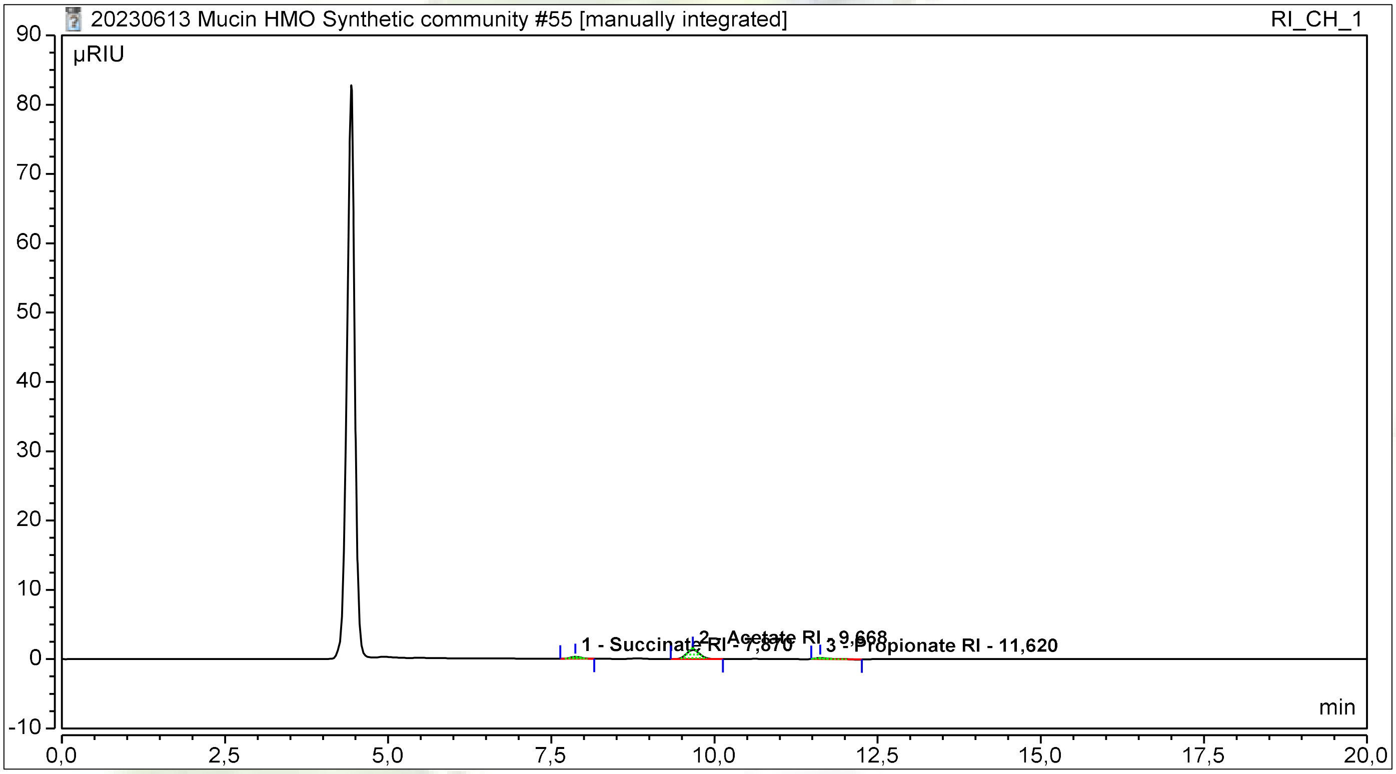

Peak Results

No.	Peak Name	Retention Time min	Width (50%) min	Type	Resolution (EP)	Asymmetry (EP)	Plates (EP)
n.a.	GlcNAc	n.a.	n.a.	n.a.	n.a.	n.a.	n.a.
n.a.	Citrate	n.a.	n.a.	n.a.	n.a.	n.a.	n.a.
n.a.	Glucose	n.a.	n.a.	n.a.	n.a.	n.a.	n.a.
n.a.	Galactose	n.a.	n.a.	n.a.	n.a.	n.a.	n.a.
n.a.	Fucose	n.a.	n.a.	n.a.	n.a.	n.a.	n.a.
1	Succinate RI	7,870	0,199	BMB*	5,00	1,07	8625
n.a.	Lactate RI	n.a.	n.a.	n.a.	n.a.	n.a.	n.a.
n.a.	glycerol	n.a.	n.a.	n.a.	n.a.	n.a.	n.a.
n.a.	Formate RI	n.a.	n.a.	n.a.	n.a.	n.a.	n.a.
2	Acetate RI	9,668	0,225	BMB	5,09	1,08	10202
n.a.	1,2 PDO RI	n.a.	n.a.	n.a.	n.a.	n.a.	n.a.
n.a.	1,3-PDO	n.a.	n.a.	n.a.	n.a.	n.a.	n.a.
3	Propionate RI	11,620	0,227	BMB*	n.a.	2,22	14459
n.a.	1,3-PDO	n.a.	n.a.	n.a.	n.a.	n.a.	n.a.
n.a.	2-3 BDO	n.a.	n.a.	n.a.	n.a.	n.a.	n.a.
n.a.	Ethanol	n.a.	n.a.	n.a.	n.a.	n.a.	n.a.
n.a.	Isobutyrate RI	n.a.	n.a.	n.a.	n.a.	n.a.	n.a.
n.a.	Butyrate RI	n.a.	n.a.	n.a.	n.a.	n.a.	n.a.

Chromatogram and SST Results

Injection Details

Injection Name:	**21 MUCHMO2 t48 r3**	*Run Time (min):*	**20,00**
Vial Number:	**3:D12**	*Injection Volume:*	**20,00**
Injection Type:	**Unknown**	*Channel:*	**RI_CH_1**
Calibration Level:		*Wavelength:*	**n.a.**
Instrument Method:	**Default method LC2030C 45 gr 20 min**	*Bandwidth:*	**n.a.**
Processing Method:	**Processing Method LC2030 45 gr**	*Dilution Factor:*	**1,0000**
Injection Date/Time:	**14-jun-23 06:20**	*Sample Weight:*	**1,0000**

Chromatogram

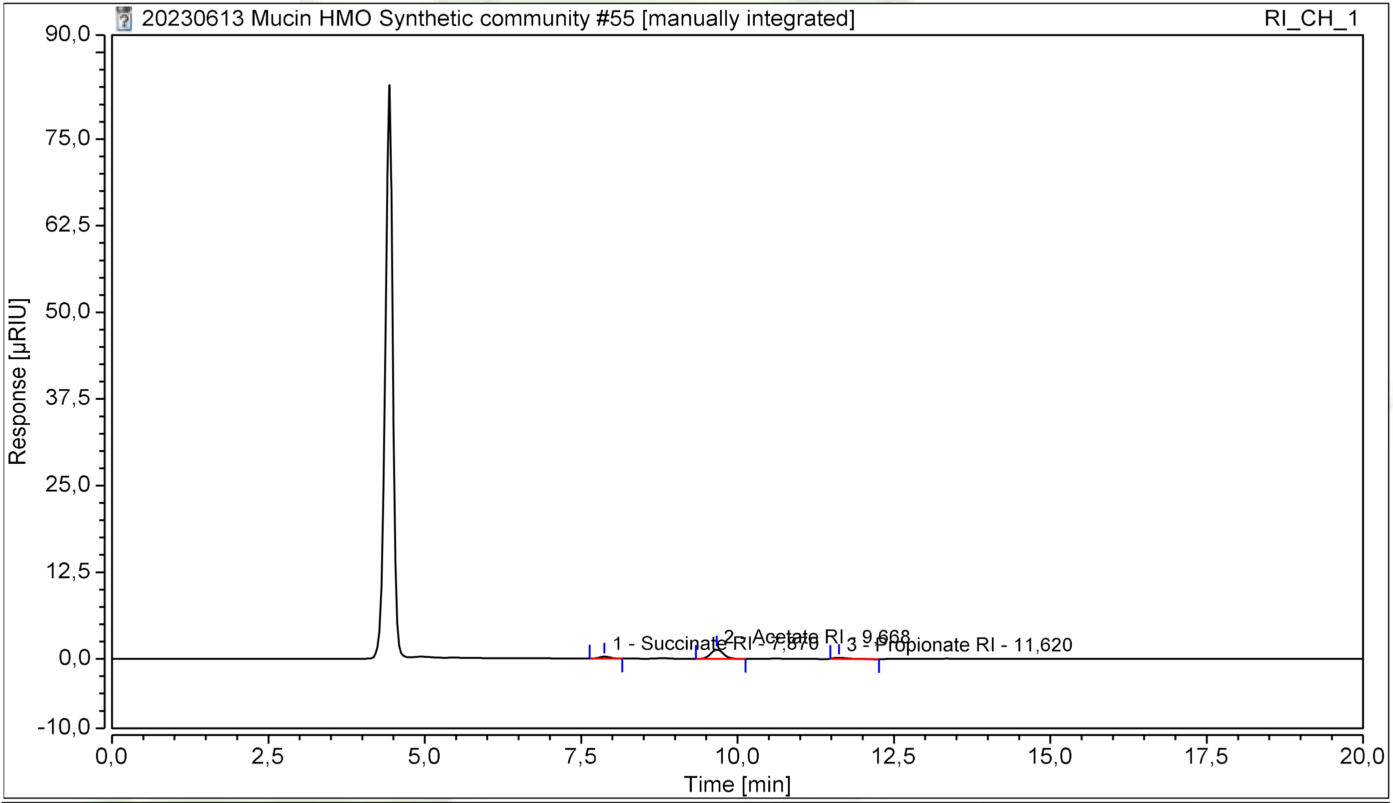

SST Results

No.	Name	Inj.Condition	Peak	Test Result	Injection
**Number of executed test cases:**	**n.a.**		**Total Result:**	**Passed**	

Chromatogram

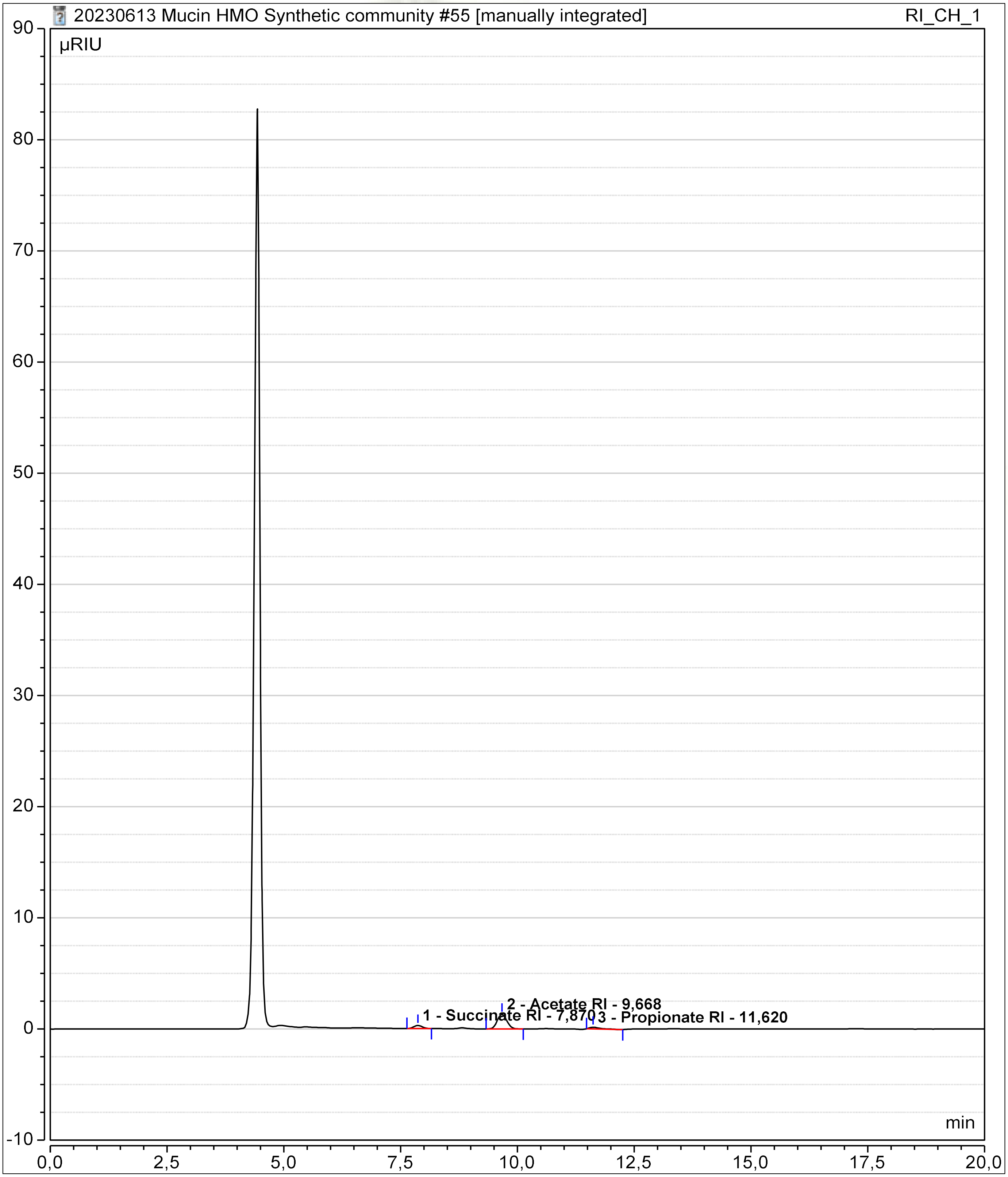

Chromeleon (c) Dionex
Version 7.2.10.25868

Chromatogram and Results

Injection Details

Injection Name:	**22 MUC t48 r1**	*Run Time (min):*	**20,00**
Vial Number:	**3:E1**	*Injection Volume:*	**20,00**
Injection Type:	**Unknown**	*Channel:*	**RI_CH_1**
Calibration Level:		*Wavelength:*	**n.a.**
Instrument Method:	**Default method LC2030C 45 gr 20 min**	*Bandwidth:*	**n.a.**
Processing Method:	**Processing Method LC2030 45 gr**	*Dilution Factor:*	**1,0000**
Injection Date/Time:	**14-jun-23 06:40**	*Sample Weight:*	**1,0000**

Chromatogram

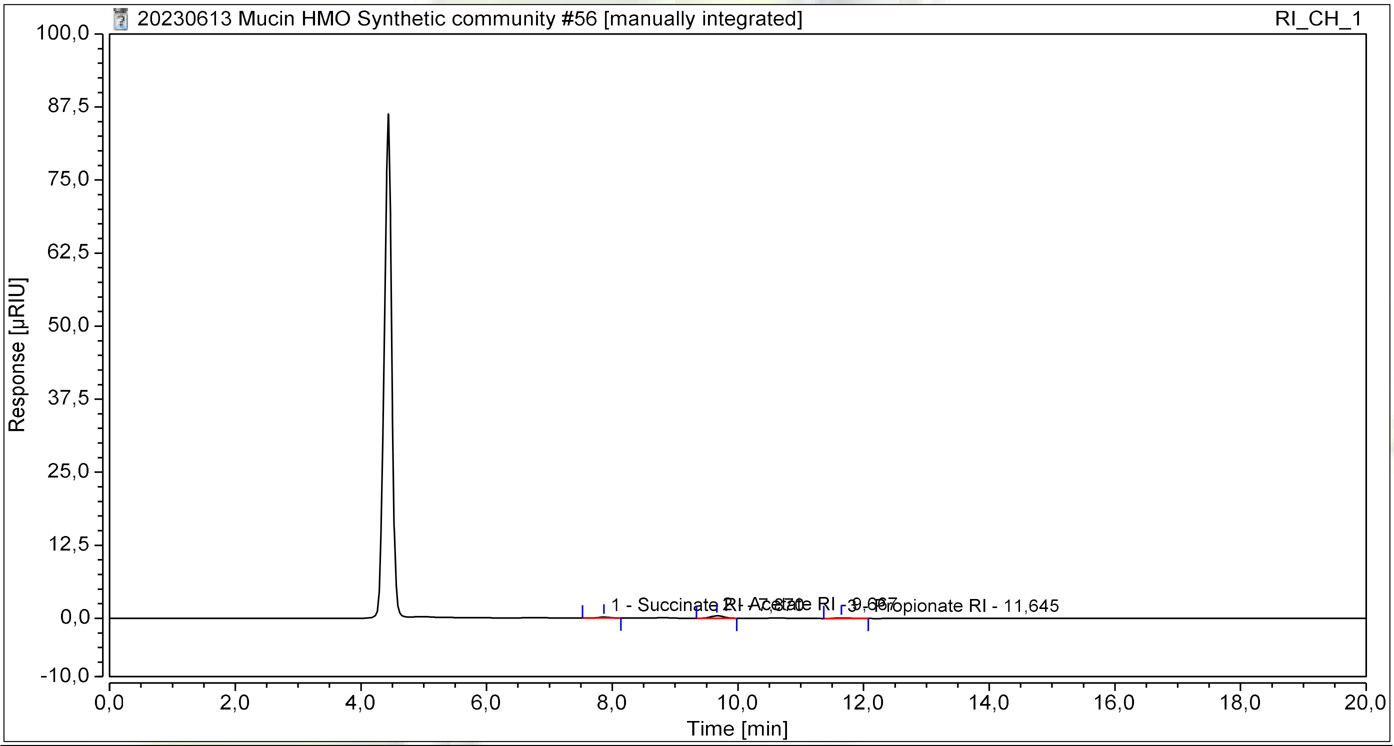

Integration Results

No.	Peak Name	Retention Time<br>min	Area<br>µRIU*min	Height<br>µRIU	Relative Area<br>%	Relative Height<br>%	Amount
n.a.	GlcNAc	n.a.	n.a.	n.a.	n.a.	n.a.	n.a.
n.a.	Citrate	n.a.	n.a.	n.a.	n.a.	n.a.	n.a.
n.a.	Glucose	n.a.	n.a.	n.a.	n.a.	n.a.	n.a.
n.a.	Galactose	n.a.	n.a.	n.a.	n.a.	n.a.	n.a.
n.a.	Fucose	n.a.	n.a.	n.a.	n.a.	n.a.	n.a.
1	Succinate RI	7,870	0,035	0,168	21,13	24,30	n.a.
n.a.	Lactate RI	n.a.	n.a.	n.a.	n.a.	n.a.	n.a.
n.a.	glycerol	n.a.	n.a.	n.a.	n.a.	n.a.	n.a.
n.a.	Formate RI	n.a.	n.a.	n.a.	n.a.	n.a.	n.a.
2	Acetate RI	9,667	0,097	0,412	58,69	59,74	5,9679
n.a.	1,2 PDO RI	n.a.	n.a.	n.a.	n.a.	n.a.	n.a.
n.a.	1,3-PDO	n.a.	n.a.	n.a.	n.a.	n.a.	n.a.
3	Propionate RI	11,645	0,033	0,110	20,18	15,96	1,3413
n.a.	1,3-PDO	n.a.	n.a.	n.a.	n.a.	n.a.	n.a.
n.a.	2-3 BDO	n.a.	n.a.	n.a.	n.a.	n.a.	n.a.
n.a.	Ethanol	n.a.	n.a.	n.a.	n.a.	n.a.	n.a.
n.a.	Isobutyrate RI	n.a.	n.a.	n.a.	n.a.	n.a.	n.a.
n.a.	Butyrate RI	n.a.	n.a.	n.a.	n.a.	n.a.	n.a.
**Total:**			**0,165**	**0,690**	**100,00**	**100,00**	

Peak Analysis

Injection Details

Injection Name:	**22 MUC t48 r1**	*Run Time (min):*	**20,00**
Vial Number:	**3:E1**	*Injection Volume:*	**20,00**
Injection Type:	**Unknown**	*Channel:*	**RI_CH_1**
Calibration Level:		*Wavelength:*	**n.a.**
Instrument Method:	**Default method LC2030C 45 gr 20 min**	*Bandwidth:*	**n.a.**
Processing Method:	**Processing Method LC2030 45 gr**	*Dilution Factor:*	**1,0000**
Injection Date/Time:	**14-jun-23 06:40**	*Sample Weight:*	**1,0000**

Chromatogram

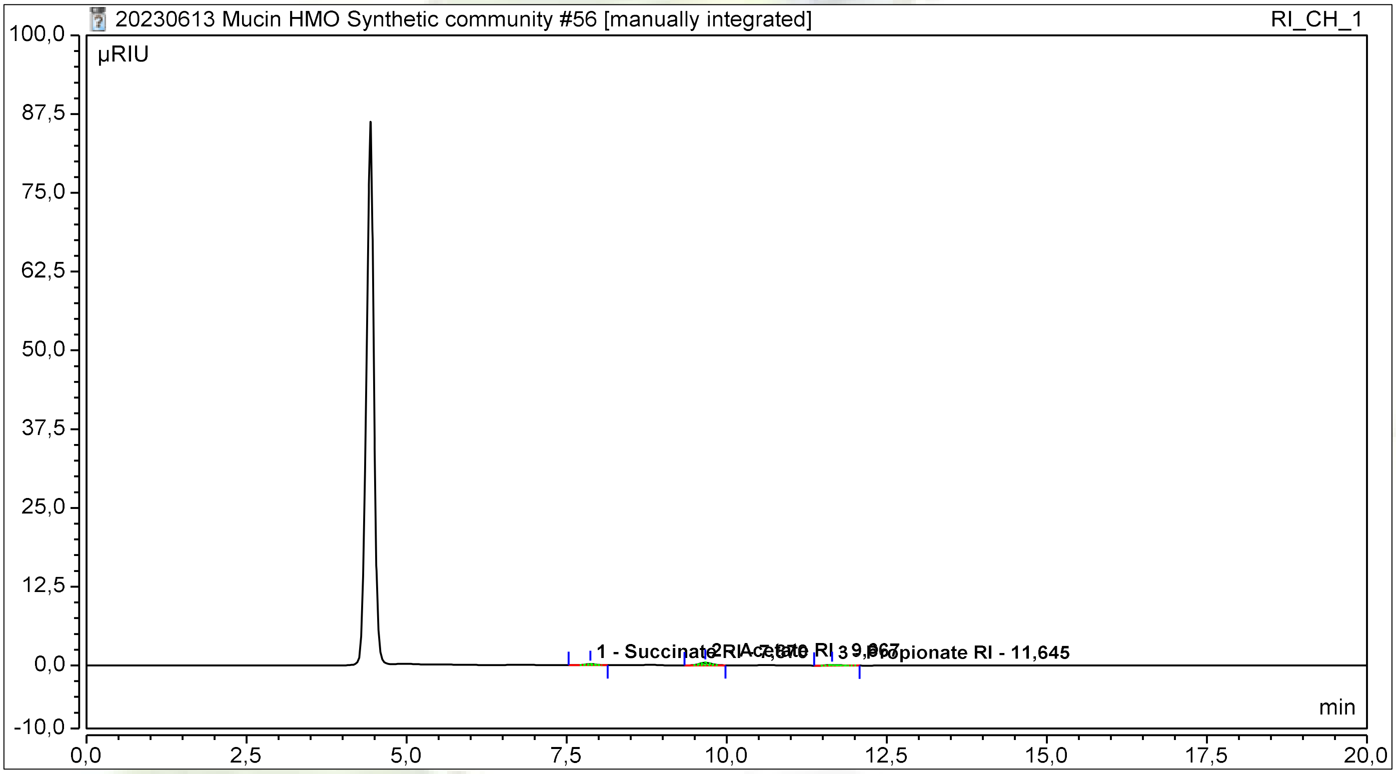

Peak Results

No.	Peak Name	Retention Time<br>min	Width (50%)<br>min	Type	Resolution (EP)	Asymmetry (EP)	Plates (EP)
n.a.	GlcNAc	n.a.	n.a.	n.a.	n.a.	n.a.	n.a.
n.a.	Citrate	n.a.	n.a.	n.a.	n.a.	n.a.	n.a.
n.a.	Glucose	n.a.	n.a.	n.a.	n.a.	n.a.	n.a.
n.a.	Galactose	n.a.	n.a.	n.a.	n.a.	n.a.	n.a.
n.a.	Fucose	n.a.	n.a.	n.a.	n.a.	n.a.	n.a.
1	Succinate RI	7,870	0,199	BMB*	5,02	1,06	8672
n.a.	Lactate RI	n.a.	n.a.	n.a.	n.a.	n.a.	n.a.
n.a.	glycerol	n.a.	n.a.	n.a.	n.a.	n.a.	n.a.
n.a.	Formate RI	n.a.	n.a.	n.a.	n.a.	n.a.	n.a.
2	Acetate RI	9,667	0,224	BMB*	4,70	1,07	10338
n.a.	1,2 PDO RI	n.a.	n.a.	n.a.	n.a.	n.a.	n.a.
n.a.	1,3-PDO	n.a.	n.a.	n.a.	n.a.	n.a.	n.a.
3	Propionate RI	11,645	0,273	BMB*	n.a.	1,66	10104
n.a.	1,3-PDO	n.a.	n.a.	n.a.	n.a.	n.a.	n.a.
n.a.	2-3 BDO	n.a.	n.a.	n.a.	n.a.	n.a.	n.a.
n.a.	Ethanol	n.a.	n.a.	n.a.	n.a.	n.a.	n.a.
n.a.	Isobutyrate RI	n.a.	n.a.	n.a.	n.a.	n.a.	n.a.
n.a.	Butyrate RI	n.a.	n.a.	n.a.	n.a.	n.a.	n.a.

Chromatogram and SST Results

Injection Details

Injection Name:	**22 MUC t48 r1**	*Run Time (min):*	**20,00**
Vial Number:	**3:E1**	*Injection Volume:*	**20,00**
Injection Type:	**Unknown**	*Channel:*	**RI_CH_1**
Calibration Level:		*Wavelength:*	**n.a.**
Instrument Method:	**Default method LC2030C 45 gr 20 min**	*Bandwidth:*	**n.a.**
Processing Method:	**Processing Method LC2030 45 gr**	*Dilution Factor:*	**1,0000**
Injection Date/Time:	**14-jun-23 06:40**	*Sample Weight:*	**1,0000**

Chromatogram

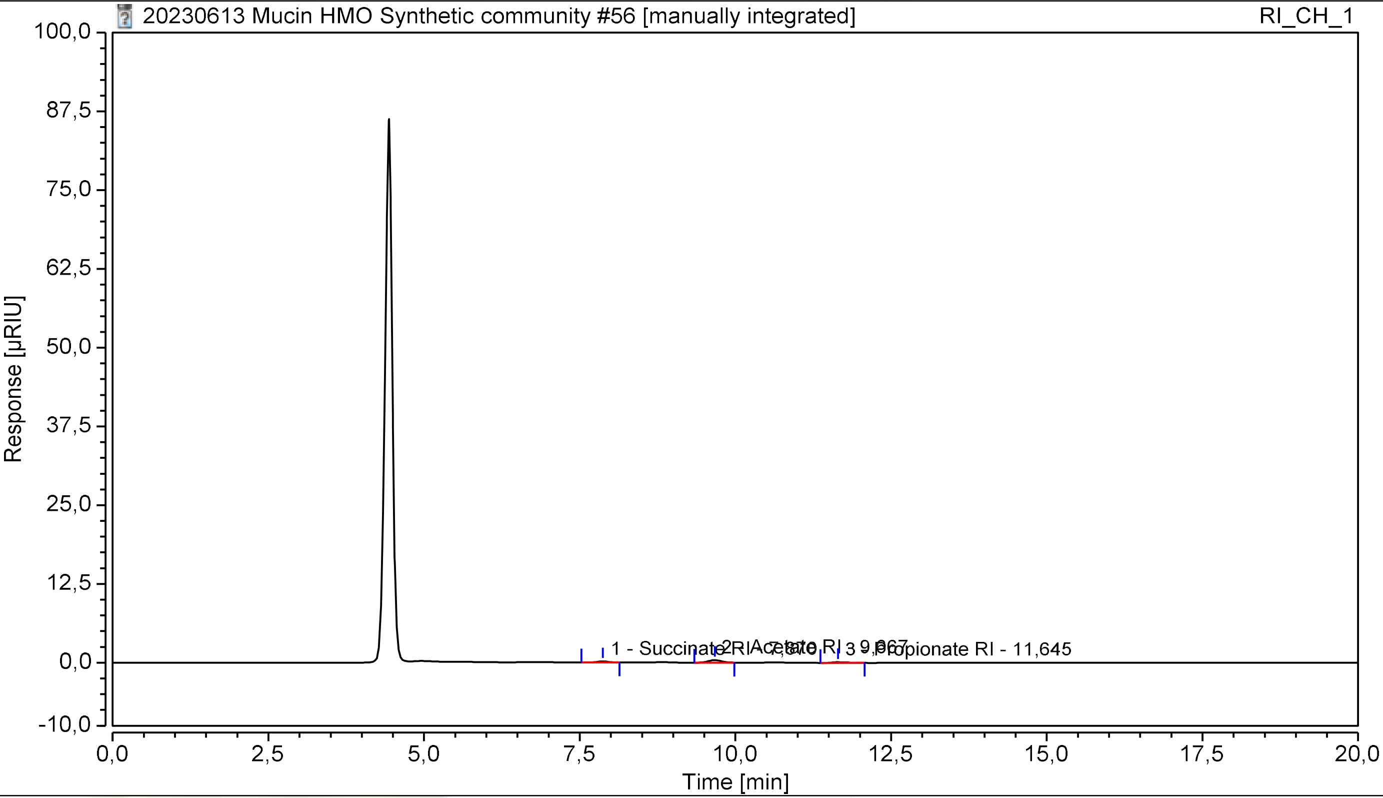

SST Results

No.	Name	Inj.Condition	Peak	Test Result	Injection
**Number of executed test cases:**	**n.a.**		**Total Result:**	**Passed**	

Chromatogram

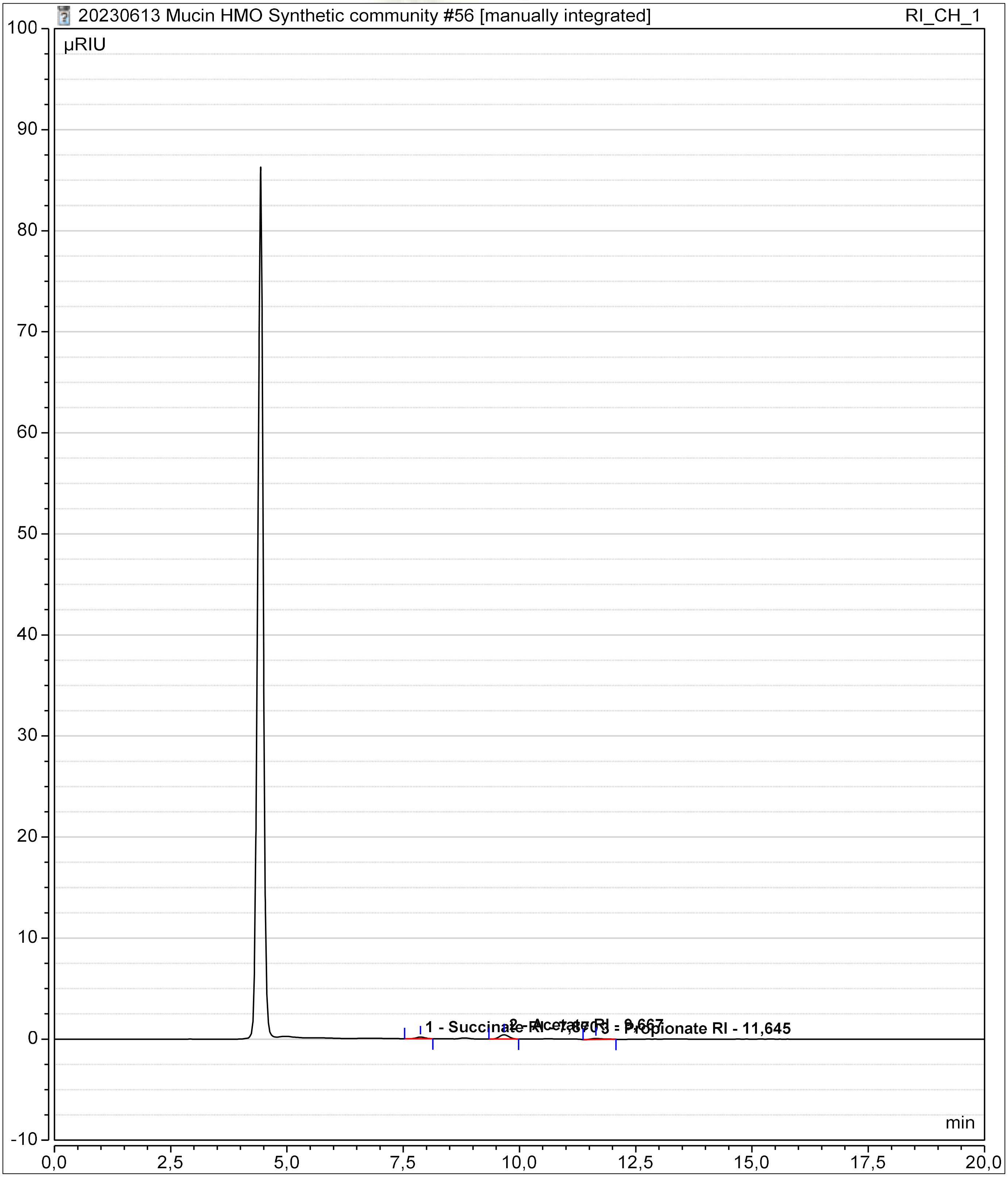

Chromeleon (c) Dionex
Version 7.2.10.25868

Chromatogram and Results

Injection Details

Injection Name:	**23 MUC t48 r2**	*Run Time (min):*	**20,00**
Vial Number:	**3:E2**	*Injection Volume:*	**20,00**
Injection Type:	**Unknown**	*Channel:*	**RI_CH_1**
Calibration Level:		*Wavelength:*	**n.a.**
Instrument Method:	**Default method LC2030C 45 gr 20 min**	*Bandwidth:*	**n.a.**
Processing Method:	**Processing Method LC2030 45 gr**	*Dilution Factor:*	**1,0000**
Injection Date/Time:	**14-jun-23 07:01**	*Sample Weight:*	**1,0000**

Chromatogram

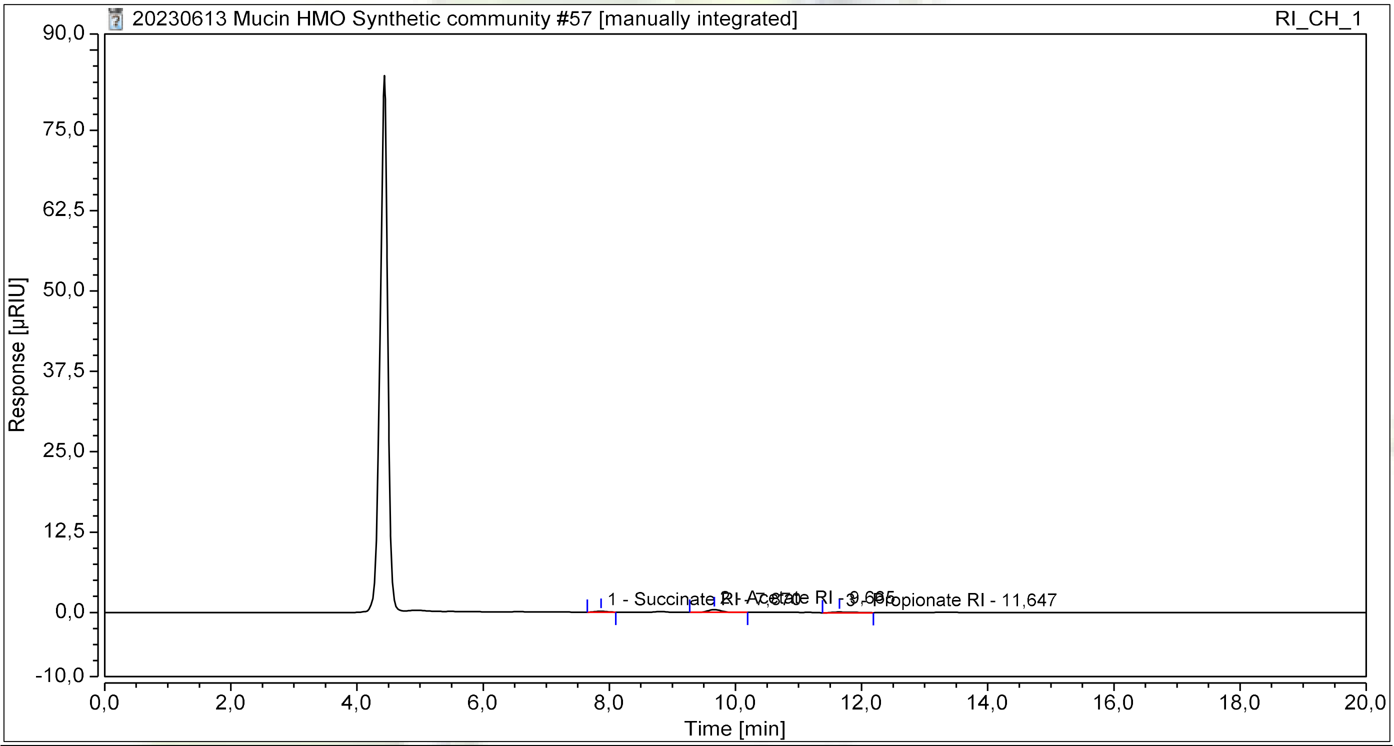

Integration Results

No.	Peak Name	Retention Time min	Area µRIU*min	Height µRIU	Relative Area %	Relative Height %	Amount
n.a.	GlcNAc	n.a.	n.a.	n.a.	n.a.	n.a.	n.a.
n.a.	Citrate	n.a.	n.a.	n.a.	n.a.	n.a.	n.a.
n.a.	Glucose	n.a.	n.a.	n.a.	n.a.	n.a.	n.a.
n.a.	Galactose	n.a.	n.a.	n.a.	n.a.	n.a.	n.a.
n.a.	Fucose	n.a.	n.a.	n.a.	n.a.	n.a.	n.a.
1	Succinate RI	7,870	0,028	0,140	15,92	19,96	n.a.
n.a.	Lactate RI	n.a.	n.a.	n.a.	n.a.	n.a.	n.a.
n.a.	glycerol	n.a.	n.a.	n.a.	n.a.	n.a.	n.a.
n.a.	Formate RI	n.a.	n.a.	n.a.	n.a.	n.a.	n.a.
2	Acetate RI	9,665	0,106	0,443	59,09	63,02	6,5053
n.a.	1,2 PDO RI	n.a.	n.a.	n.a.	n.a.	n.a.	n.a.
n.a.	1,3-PDO	n.a.	n.a.	n.a.	n.a.	n.a.	n.a.
3	Propionate RI	11,647	0,045	0,120	24,99	17,01	1,7984
n.a.	1,3-PDO	n.a.	n.a.	n.a.	n.a.	n.a.	n.a.
n.a.	2-3 BDO	n.a.	n.a.	n.a.	n.a.	n.a.	n.a.
n.a.	Ethanol	n.a.	n.a.	n.a.	n.a.	n.a.	n.a.
n.a.	Isobutyrate RI	n.a.	n.a.	n.a.	n.a.	n.a.	n.a.
n.a.	Butyrate RI	n.a.	n.a.	n.a.	n.a.	n.a.	n.a.
**Total:**			**0,179**	**0,703**	**100,00**	**100,00**	

Peak Analysis

Injection Details

Injection Name:	**23 MUC t48 r2**	*Run Time (min):*	**20,00**
Vial Number:	**3:E2**	*Injection Volume:*	**20,00**
Injection Type:	**Unknown**	*Channel:*	**RI_CH_1**
Calibration Level:		*Wavelength:*	**n.a.**
Instrument Method:	**Default method LC2030C 45 gr 20 min**	*Bandwidth:*	**n.a.**
Processing Method:	**Processing Method LC2030 45 gr**	*Dilution Factor:*	**1,0000**
Injection Date/Time:	**14-jun-23 07:01**	*Sample Weight:*	**1,0000**

Chromatogram

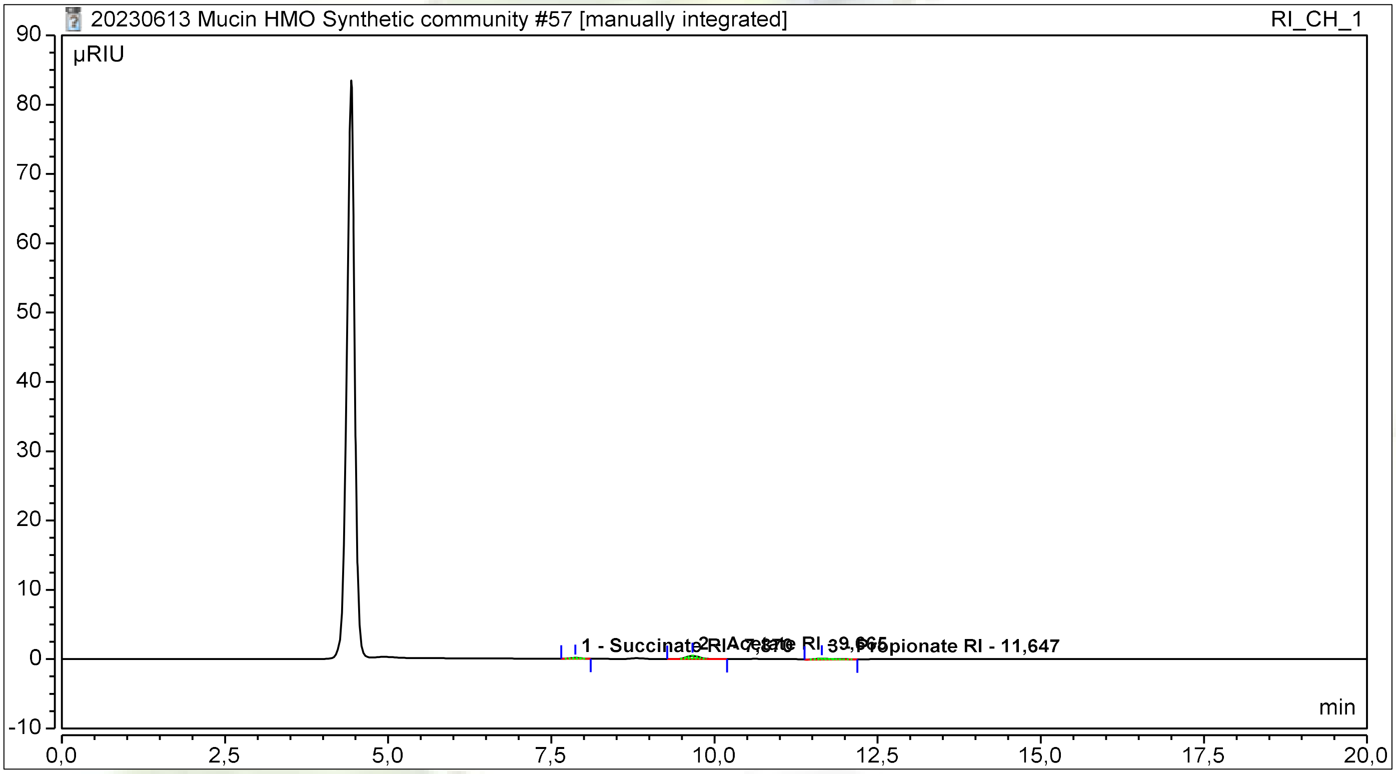

Peak Results

No.	Peak Name	Retention Time min	Width (50%) min	Type	Resolution (EP)	Asymmetry (EP)	Plates (EP)
n.a.	GlcNAc	n.a.	n.a.	n.a.	n.a.	n.a.	n.a.
n.a.	Citrate	n.a.	n.a.	n.a.	n.a.	n.a.	n.a.
n.a.	Glucose	n.a.	n.a.	n.a.	n.a.	n.a.	n.a.
n.a.	Galactose	n.a.	n.a.	n.a.	n.a.	n.a.	n.a.
n.a.	Fucose	n.a.	n.a.	n.a.	n.a.	n.a.	n.a.
1	Succinate RI	7,870	0,197	BMB*	5,02	1,04	8878
n.a.	Lactate RI	n.a.	n.a.	n.a.	n.a.	n.a.	n.a.
n.a.	glycerol	n.a.	n.a.	n.a.	n.a.	n.a.	n.a.
n.a.	Formate RI	n.a.	n.a.	n.a.	n.a.	n.a.	n.a.
2	Acetate RI	9,665	0,225	BMB	4,10	1,08	10199
n.a.	1,2 PDO RI	n.a.	n.a.	n.a.	n.a.	n.a.	n.a.
n.a.	1,3-PDO	n.a.	n.a.	n.a.	n.a.	n.a.	n.a.
3	Propionate RI	11,647	0,346	BMB*	n.a.	1,77	6289
n.a.	1,3-PDO	n.a.	n.a.	n.a.	n.a.	n.a.	n.a.
n.a.	2-3 BDO	n.a.	n.a.	n.a.	n.a.	n.a.	n.a.
n.a.	Ethanol	n.a.	n.a.	n.a.	n.a.	n.a.	n.a.
n.a.	Isobutyrate RI	n.a.	n.a.	n.a.	n.a.	n.a.	n.a.
n.a.	Butyrate RI	n.a.	n.a.	n.a.	n.a.	n.a.	n.a.

Chromatogram and SST Results

Injection Details

Injection Name:	**23 MUC t48 r2**	*Run Time (min):*	**20,00**
Vial Number:	**3:E2**	*Injection Volume:*	**20,00**
Injection Type:	**Unknown**	*Channel:*	**RI_CH_1**
Calibration Level:		*Wavelength:*	**n.a.**
Instrument Method:	**Default method LC2030C 45 gr 20 min**	*Bandwidth:*	**n.a.**
Processing Method:	**Processing Method LC2030 45 gr**	*Dilution Factor:*	**1,0000**
Injection Date/Time:	**14-jun-23 07:01**	*Sample Weight:*	**1,0000**

Chromatogram

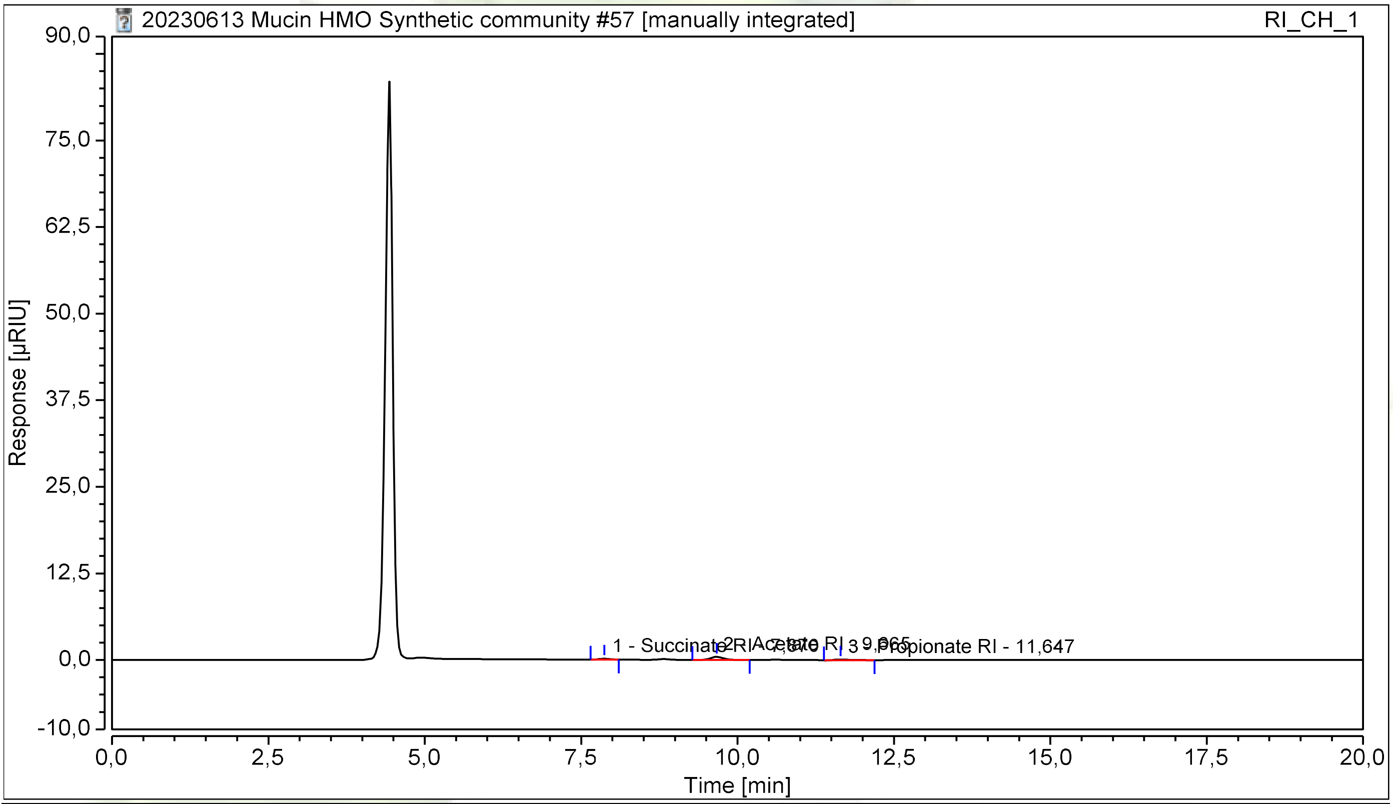

SST Results

No.	Name	Inj.Condition	Peak	Test Result	Injection
**Number of executed test cases:**	**n.a.**		**Total Result:**	**Passed**	

Chromatogram

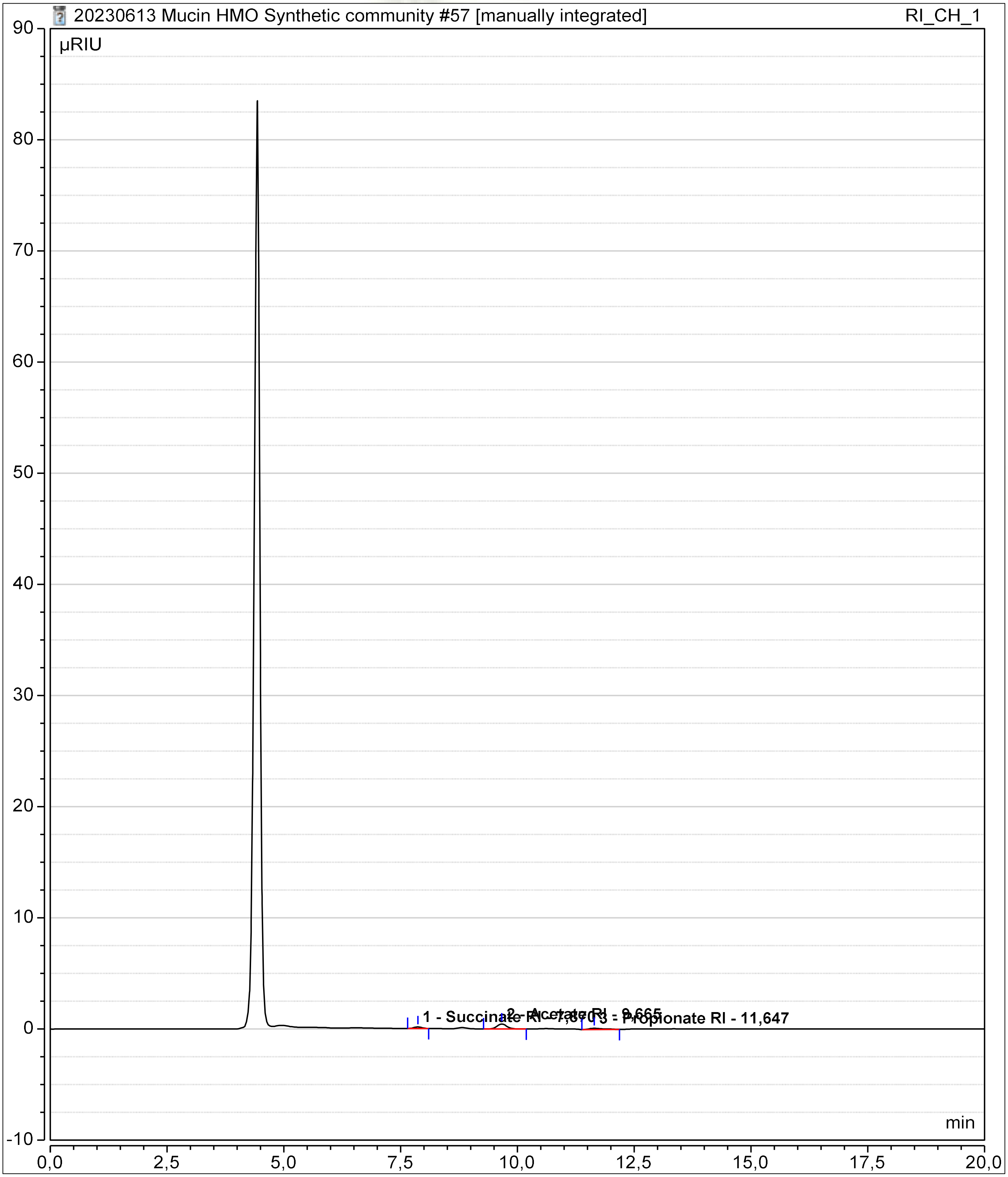

Chromeleon (c) Dionex
Version 7.2.10.25868

Chromatogram and Results

Injection Details

Injection Name:	**24 MUC t48 r3**	*Run Time (min):*	**20,00**
Vial Number:	**3:E3**	*Injection Volume:*	**20,00**
Injection Type:	**Unknown**	*Channel:*	**RI_CH_1**
Calibration Level:		*Wavelength:*	**n.a.**
Instrument Method:	**Default method LC2030C 45 gr 20 min**	*Bandwidth:*	**n.a.**
Processing Method:	**Processing Method LC2030 45 gr**	*Dilution Factor:*	**1,0000**
Injection Date/Time:	**14-jun-23 07:21**	*Sample Weight:*	**1,0000**

Chromatogram

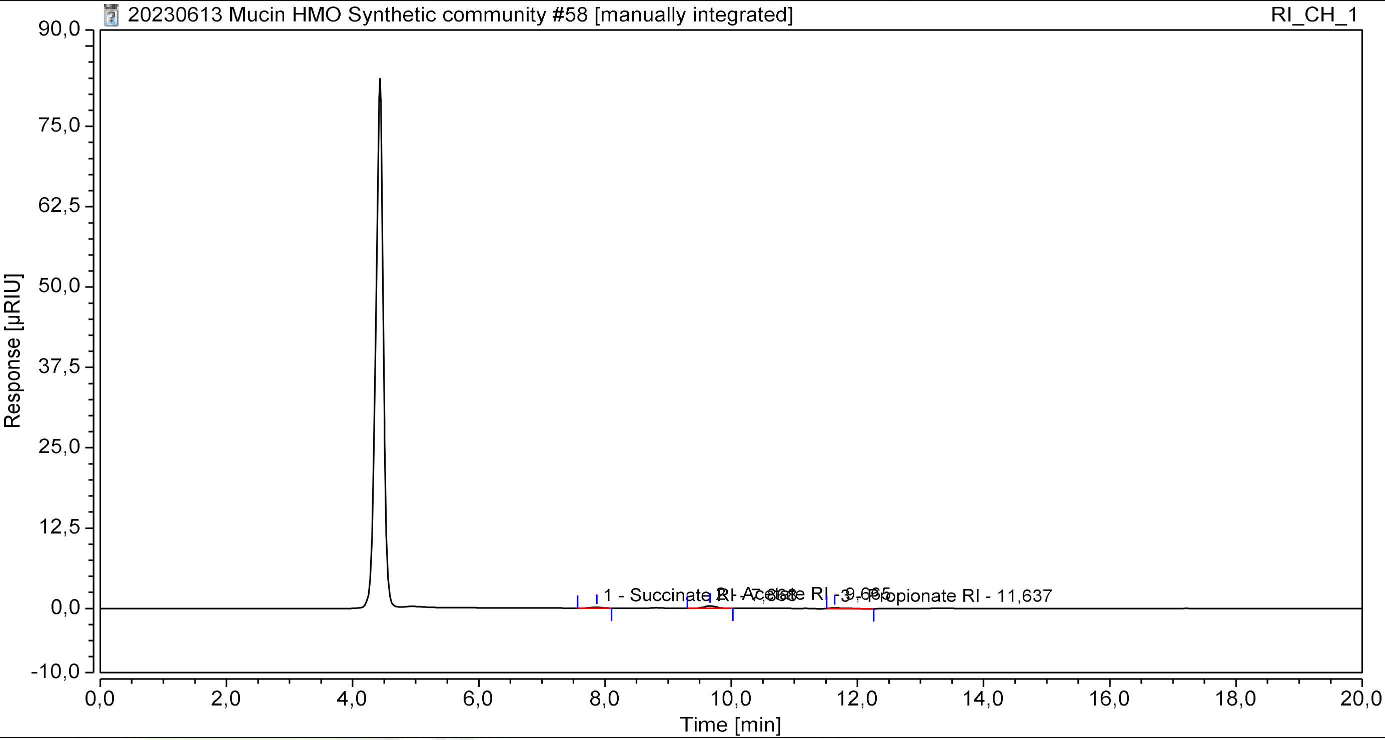

Integration Results

No.	Peak Name	Retention Time min	Area µRIU*min	Height µRIU	Relative Area %	Relative Height %	Amount
n.a.	GlcNAc	n.a.	n.a.	n.a.	n.a.	n.a.	n.a.
n.a.	Citrate	n.a.	n.a.	n.a.	n.a.	n.a.	n.a.
n.a.	Glucose	n.a.	n.a.	n.a.	n.a.	n.a.	n.a.
n.a.	Galactose	n.a.	n.a.	n.a.	n.a.	n.a.	n.a.
n.a.	Fucose	n.a.	n.a.	n.a.	n.a.	n.a.	n.a.
1	Succinate RI	7,868	0,030	0,148	20,77	24,11	n.a.
n.a.	Lactate RI	n.a.	n.a.	n.a.	n.a.	n.a.	n.a.
n.a.	glycerol	n.a.	n.a.	n.a.	n.a.	n.a.	n.a.
n.a.	Formate RI	n.a.	n.a.	n.a.	n.a.	n.a.	n.a.
2	Acetate RI	9,665	0,090	0,380	61,58	62,05	5,5447
n.a.	1,2 PDO RI	n.a.	n.a.	n.a.	n.a.	n.a.	n.a.
n.a.	1,3-PDO	n.a.	n.a.	n.a.	n.a.	n.a.	n.a.
3	Propionate RI	11,637	0,026	0,085	17,65	13,84	1,0387
n.a.	1,3-PDO	n.a.	n.a.	n.a.	n.a.	n.a.	n.a.
n.a.	2-3 BDO	n.a.	n.a.	n.a.	n.a.	n.a.	n.a.
n.a.	Ethanol	n.a.	n.a.	n.a.	n.a.	n.a.	n.a.
n.a.	Isobutyrate RI	n.a.	n.a.	n.a.	n.a.	n.a.	n.a.
n.a.	Butyrate RI	n.a.	n.a.	n.a.	n.a.	n.a.	n.a.
**Total:**			**0,146**	**0,612**	**100,00**	**100,00**	

Chromeleon (c) Dionex
Version 7.2.10.25868

Peak Analysis

Injection Details

Injection Name:	**24 MUC t48 r3**	*Run Time (min):*	**20,00**
Vial Number:	**3:E3**	*Injection Volume:*	**20,00**
Injection Type:	**Unknown**	*Channel:*	**RI_CH_1**
Calibration Level:		*Wavelength:*	**n.a.**
Instrument Method:	**Default method LC2030C 45 gr 20 min**	*Bandwidth:*	**n.a.**
Processing Method:	**Processing Method LC2030 45 gr**	*Dilution Factor:*	**1,0000**
Injection Date/Time:	**14-jun-23 07:21**	*Sample Weight:*	**1,0000**

Chromatogram

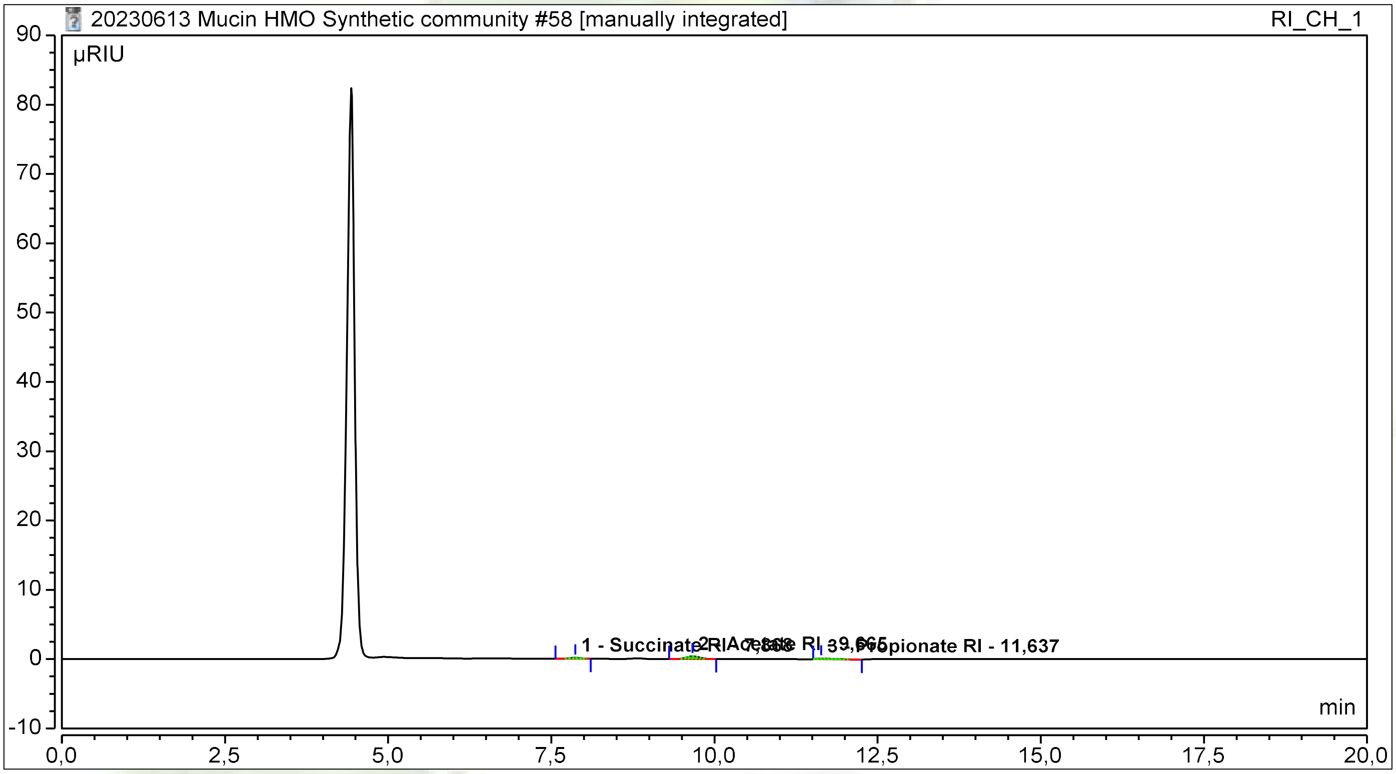

Peak Results

No.	Peak Name	Retention Time<br>min	Width (50%)<br>min	Type	Resolution (EP)	Asymmetry (EP)	Plates (EP)
n.a.	GlcNAc	n.a.	n.a.	n.a.	n.a.	n.a.	n.a.
n.a.	Citrate	n.a.	n.a.	n.a.	n.a.	n.a.	n.a.
n.a.	Glucose	n.a.	n.a.	n.a.	n.a.	n.a.	n.a.
n.a.	Galactose	n.a.	n.a.	n.a.	n.a.	n.a.	n.a.
n.a.	Fucose	n.a.	n.a.	n.a.	n.a.	n.a.	n.a.
1	Succinate RI	7,868	0,197	BMB*	5,02	1,04	8806
n.a.	Lactate RI	n.a.	n.a.	n.a.	n.a.	n.a.	n.a.
n.a.	glycerol	n.a.	n.a.	n.a.	n.a.	n.a.	n.a.
n.a.	Formate RI	n.a.	n.a.	n.a.	n.a.	n.a.	n.a.
2	Acetate RI	9,665	0,225	BMB*	3,66	1,08	10243
n.a.	1,2 PDO RI	n.a.	n.a.	n.a.	n.a.	n.a.	n.a.
n.a.	1,3-PDO	n.a.	n.a.	n.a.	n.a.	n.a.	n.a.
3	Propionate RI	11,637	0,410	BMB*	n.a.	2,29	4462
n.a.	1,3-PDO	n.a.	n.a.	n.a.	n.a.	n.a.	n.a.
n.a.	2-3 BDO	n.a.	n.a.	n.a.	n.a.	n.a.	n.a.
n.a.	Ethanol	n.a.	n.a.	n.a.	n.a.	n.a.	n.a.
n.a.	Isobutyrate RI	n.a.	n.a.	n.a.	n.a.	n.a.	n.a.
n.a.	Butyrate RI	n.a.	n.a.	n.a.	n.a.	n.a.	n.a.

Chromatogram and SST Results

Injection Details

Injection Name:	**24 MUC t48 r3**	*Run Time (min):*	**20,00**
Vial Number:	**3:E3**	*Injection Volume:*	**20,00**
Injection Type:	**Unknown**	*Channel:*	**RI_CH_1**
Calibration Level:		*Wavelength:*	**n.a.**
Instrument Method:	**Default method LC2030C 45 gr 20 min**	*Bandwidth:*	**n.a.**
Processing Method:	**Processing Method LC2030 45 gr**	*Dilution Factor:*	**1,0000**
Injection Date/Time:	**14-jun-23 07:21**	*Sample Weight:*	**1,0000**

Chromatogram

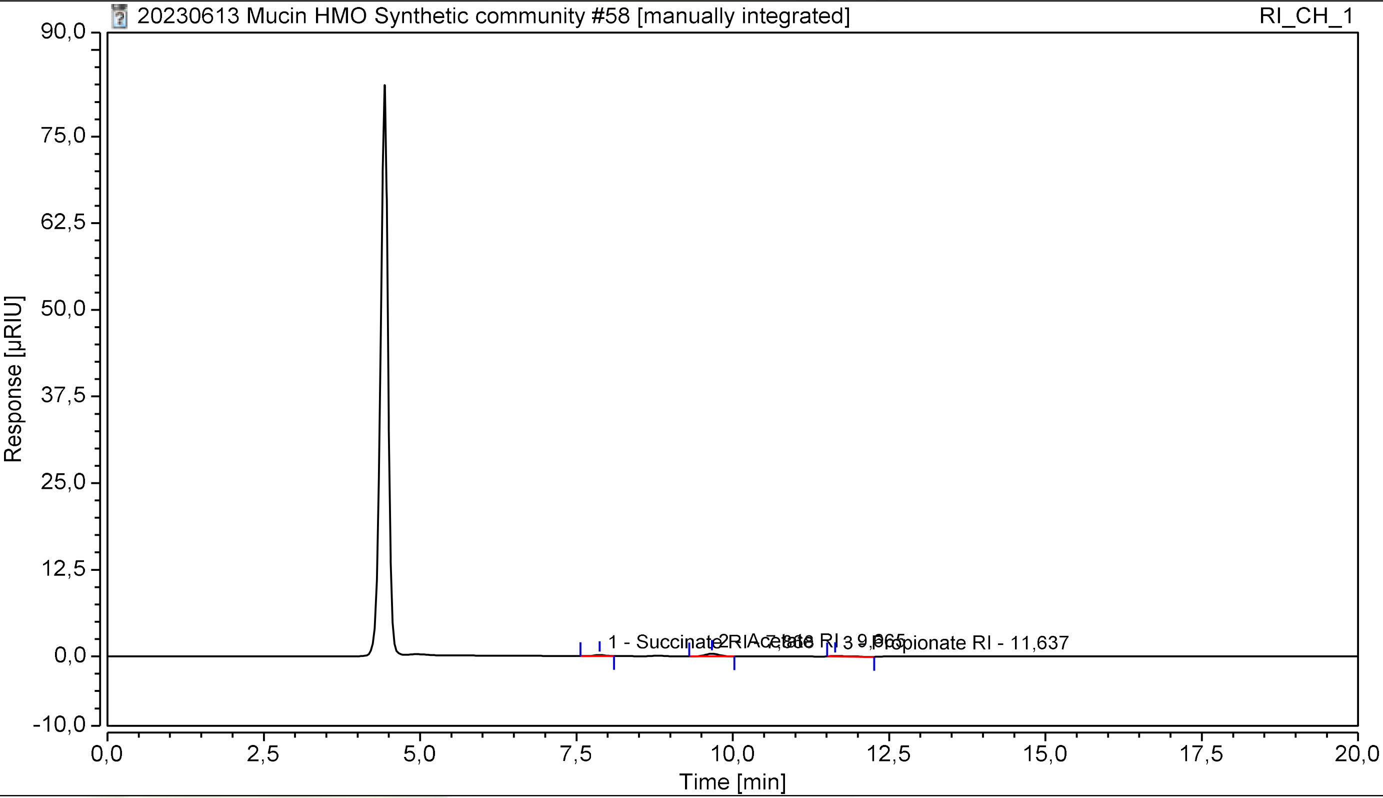

SST Results

No.	Name	Inj.Condition	Peak	Test Result	Injection

**Number of executed test cases:**	**n.a.**	**Total Result:**	**Passed**

Chromatogram

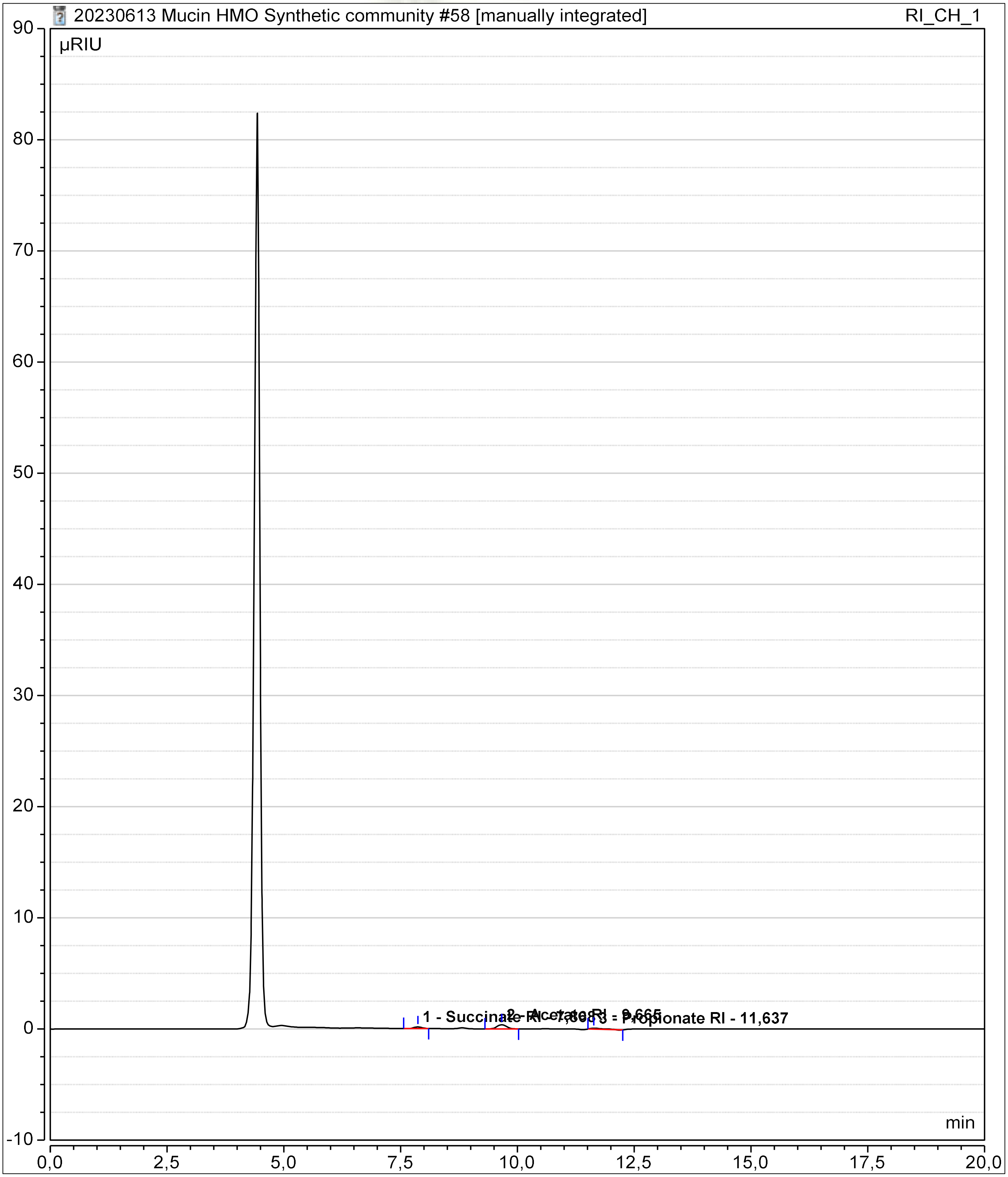

Chromeleon (c) Dionex
Version 7.2.10.25868

Chromatogram and Results

Injection Details

Injection Name:	**28 GOSFOSMUC t48 r1**	*Run Time (min):*	**20,00**
Vial Number:	**3:E4**	*Injection Volume:*	**20,00**
Injection Type:	**Unknown**	*Channel:*	**RI_CH_1**
Calibration Level:		*Wavelength:*	**n.a.**
Instrument Method:	**Default method LC2030C 45 gr 20 min**	*Bandwidth:*	**n.a.**
Processing Method:	**Processing Method LC2030 45 gr**	*Dilution Factor:*	**1,0000**
Injection Date/Time:	**14-jun-23 07:42**	*Sample Weight:*	**1,0000**

Chromatogram

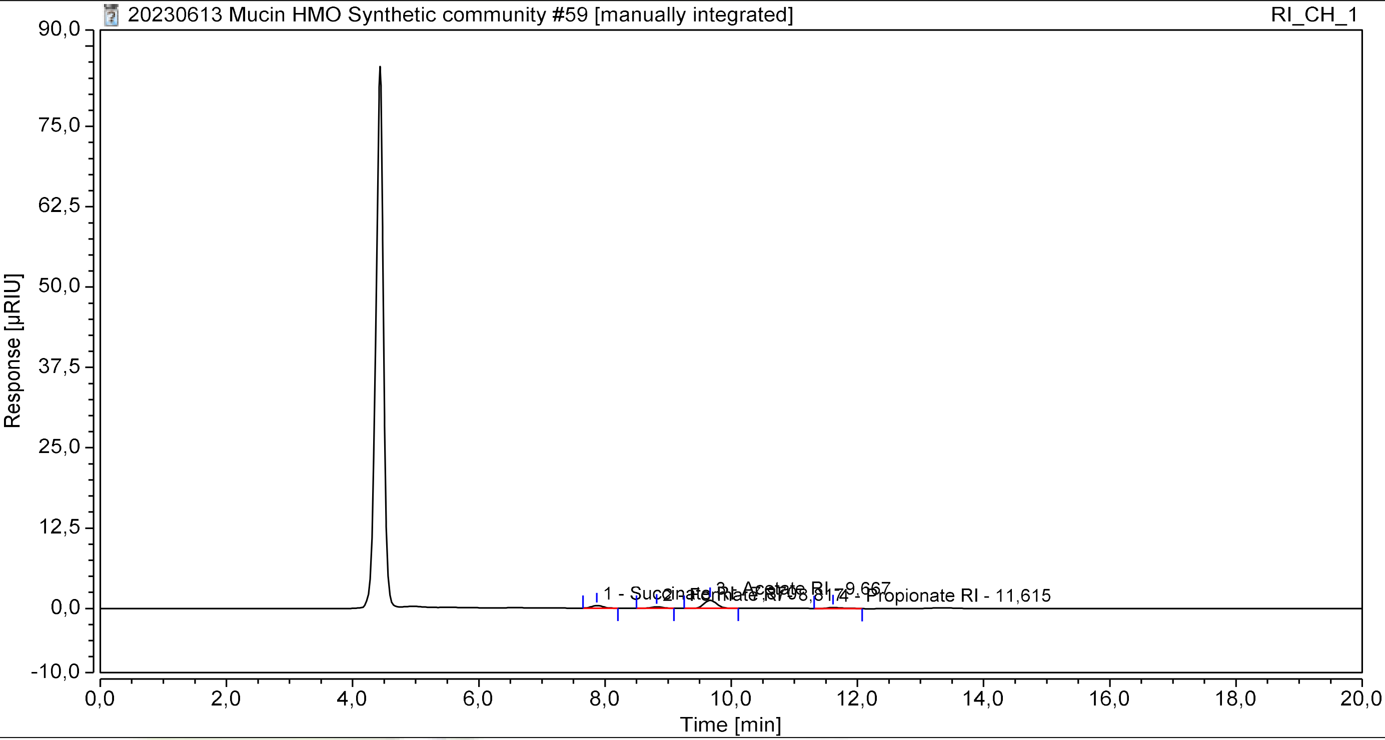

Integration Results

No.	Peak Name	Retention Time min	Area µRIU*min	Height µRIU	Relative Area %	Relative Height %	Amount
n.a.	GlcNAc	n.a.	n.a.	n.a.	n.a.	n.a.	n.a.
n.a.	Citrate	n.a.	n.a.	n.a.	n.a.	n.a.	n.a.
n.a.	Glucose	n.a.	n.a.	n.a.	n.a.	n.a.	n.a.
n.a.	Galactose	n.a.	n.a.	n.a.	n.a.	n.a.	n.a.
n.a.	Fucose	n.a.	n.a.	n.a.	n.a.	n.a.	n.a.
1	Succinate RI	7,870	0,082	0,397	17,45	19,60	n.a.
n.a.	Lactate RI	n.a.	n.a.	n.a.	n.a.	n.a.	n.a.
n.a.	glycerol	n.a.	n.a.	n.a.	n.a.	n.a.	n.a.
2	Formate RI	8,817	0,046	0,222	9,82	10,96	4,8664
3	Acetate RI	9,667	0,298	1,251	63,06	61,72	18,3191
n.a.	1,2 PDO RI	n.a.	n.a.	n.a.	n.a.	n.a.	n.a.
n.a.	1,3-PDO	n.a.	n.a.	n.a.	n.a.	n.a.	n.a.
4	Propionate RI	11,615	0,046	0,156	9,67	7,72	1,8373
n.a.	1,3-PDO	n.a.	n.a.	n.a.	n.a.	n.a.	n.a.
n.a.	2-3 BDO	n.a.	n.a.	n.a.	n.a.	n.a.	n.a.
n.a.	Ethanol	n.a.	n.a.	n.a.	n.a.	n.a.	n.a.
n.a.	Isobutyrate RI	n.a.	n.a.	n.a.	n.a.	n.a.	n.a.
n.a.	Butyrate RI	n.a.	n.a.	n.a.	n.a.	n.a.	n.a.
**Total:**			**0,472**	**2,026**	**100,00**	**100,00**	

Peak Analysis

Injection Details

Injection Name:	**28 GOSFOSMUC t48 r1**	*Run Time (min):*	**20,00**
Vial Number:	**3:E4**	*Injection Volume:*	**20,00**
Injection Type:	**Unknown**	*Channel:*	**RI_CH_1**
Calibration Level:		*Wavelength:*	**n.a.**
Instrument Method:	**Default method LC2030C 45 gr 20 min**	*Bandwidth:*	**n.a.**
Processing Method:	**Processing Method LC2030 45 gr**	*Dilution Factor:*	**1,0000**
Injection Date/Time:	**14-jun-23 07:42**	*Sample Weight:*	**1,0000**

Chromatogram

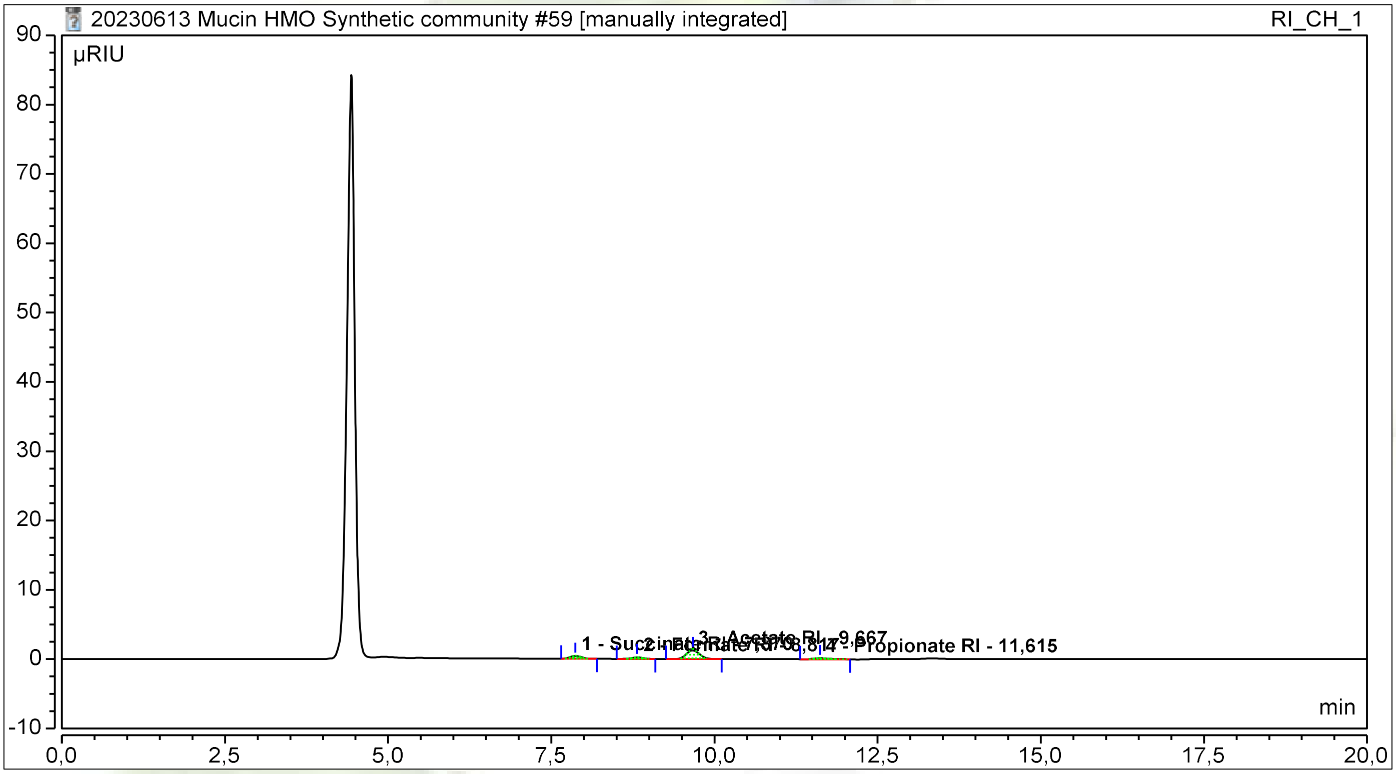

Peak Results

No.	Peak Name	Retention Time min	Width (50%) min	Type	Resolution (EP)	Asymmetry (EP)	Plates (EP)
n.a.	GlcNAc	n.a.	n.a.	n.a.	n.a.	n.a.	n.a.
n.a.	Citrate	n.a.	n.a.	n.a.	n.a.	n.a.	n.a.
n.a.	Glucose	n.a.	n.a.	n.a.	n.a.	n.a.	n.a.
n.a.	Galactose	n.a.	n.a.	n.a.	n.a.	n.a.	n.a.
n.a.	Fucose	n.a.	n.a.	n.a.	n.a.	n.a.	n.a.
1	Succinate RI	7,870	0,199	BMB*	2,81	1,08	8644
n.a.	Lactate RI	n.a.	n.a.	n.a.	n.a.	n.a.	n.a.
n.a.	glycerol	n.a.	n.a.	n.a.	n.a.	n.a.	n.a.
2	Formate RI	8,817	0,198	BMB*	2,37	1,05	10953
3	Acetate RI	9,667	0,225	BMB	4,76	1,08	10218
n.a.	1,2 PDO RI	n.a.	n.a.	n.a.	n.a.	n.a.	n.a.
n.a.	1,3-PDO	n.a.	n.a.	n.a.	n.a.	n.a.	n.a.
4	Propionate RI	11,615	0,258	BMB*	n.a.	1,77	11218
n.a.	1,3-PDO	n.a.	n.a.	n.a.	n.a.	n.a.	n.a.
n.a.	2-3 BDO	n.a.	n.a.	n.a.	n.a.	n.a.	n.a.
n.a.	Ethanol	n.a.	n.a.	n.a.	n.a.	n.a.	n.a.
n.a.	Isobutyrate RI	n.a.	n.a.	n.a.	n.a.	n.a.	n.a.
n.a.	Butyrate RI	n.a.	n.a.	n.a.	n.a.	n.a.	n.a.

Chromeleon (c) Dionex
Version 7.2.10.25868

Chromatogram and SST Results

Injection Details

Injection Name:	**28 GOSFOSMUC t48 r1**	*Run Time (min):*	**20,00**
Vial Number:	**3:E4**	*Injection Volume:*	**20,00**
Injection Type:	**Unknown**	*Channel:*	**RI_CH_1**
Calibration Level:		*Wavelength:*	**n.a.**
Instrument Method:	**Default method LC2030C 45 gr 20 min**	*Bandwidth:*	**n.a.**
Processing Method:	**Processing Method LC2030 45 gr**	*Dilution Factor:*	**1,0000**
Injection Date/Time:	**14-jun-23 07:42**	*Sample Weight:*	**1,0000**

Chromatogram

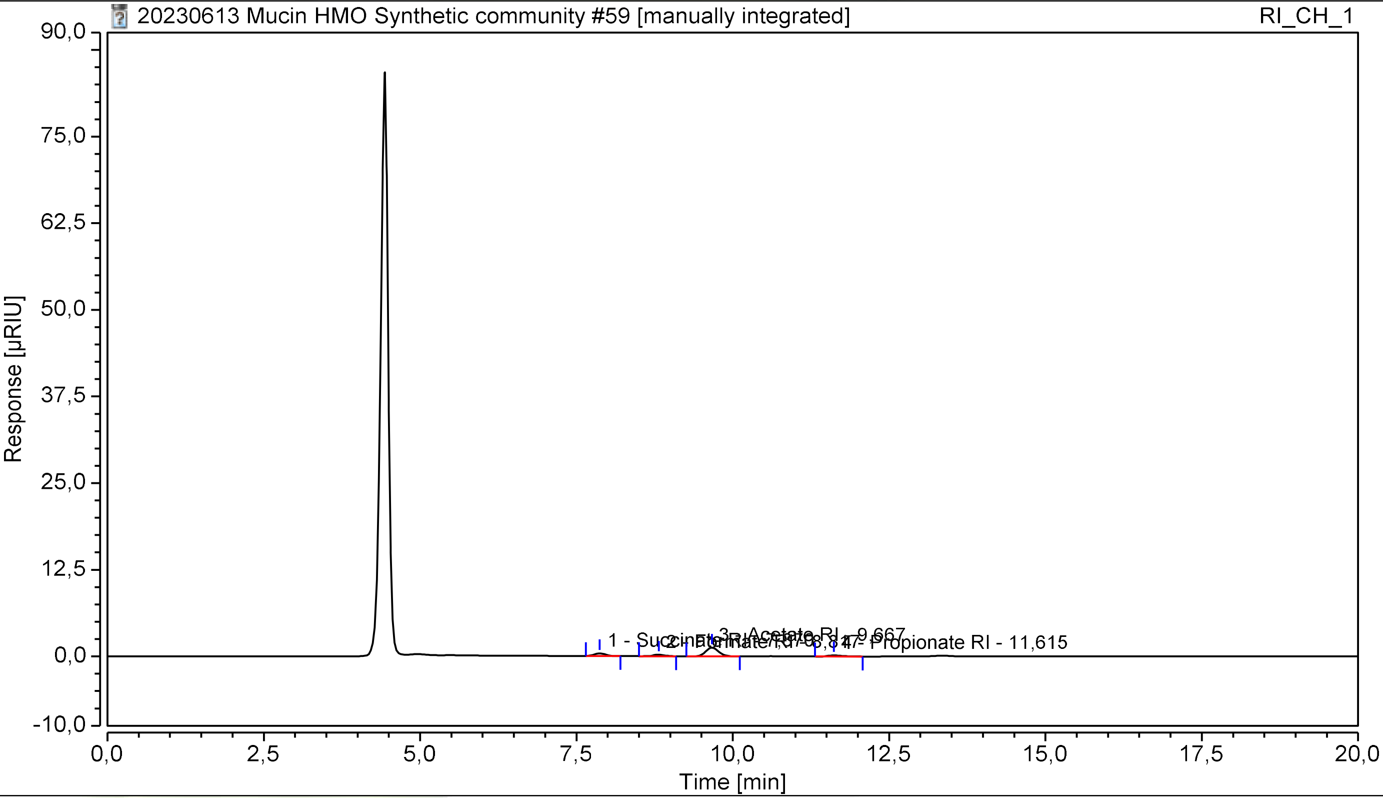

SST Results

No.	Name	Inj.Condition	Peak	Test Result	Injection
**Number of executed test cases:**	**n.a.**		**Total Result:**	**Passed**	

Chromeleon (c) Dionex
Version 7.2.10.25868

Chromatogram

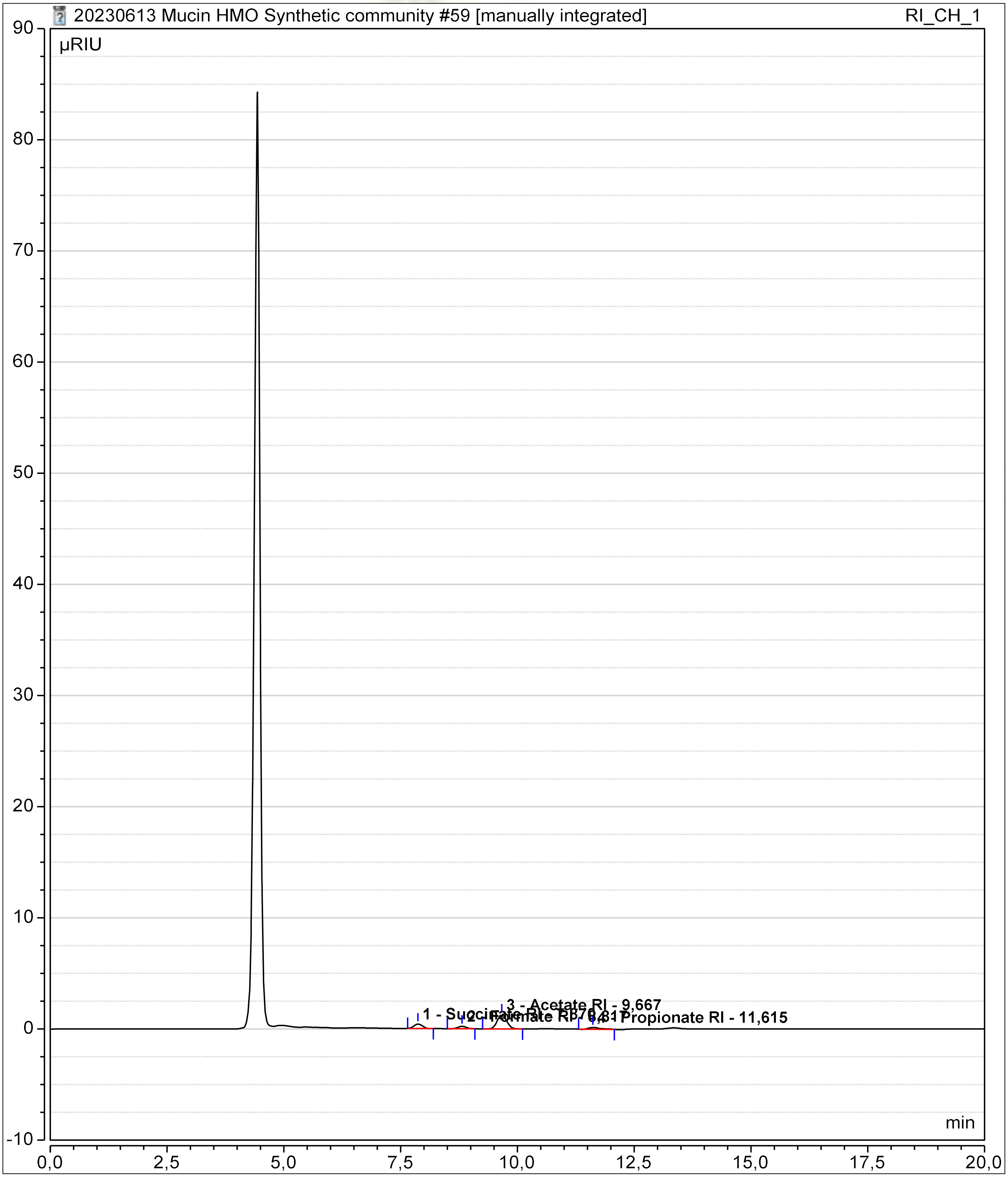

Chromeleon (c) Dionex
Version 7.2.10.25868

Chromatogram and Results

Injection Details

Injection Name:	**29 GOSFOSMUC t48 r2**	*Run Time (min):*	**20,00**
Vial Number:	**3:E5**	*Injection Volume:*	**20,00**
Injection Type:	**Unknown**	*Channel:*	**RI_CH_1**
Calibration Level:		*Wavelength:*	**n.a.**
Instrument Method:	**Default method LC2030C 45 gr 20 min**	*Bandwidth:*	**n.a.**
Processing Method:	**Processing Method LC2030 45 gr**	*Dilution Factor:*	**1,0000**
Injection Date/Time:	**14-jun-23 08:02**	*Sample Weight:*	**1,0000**

Chromatogram

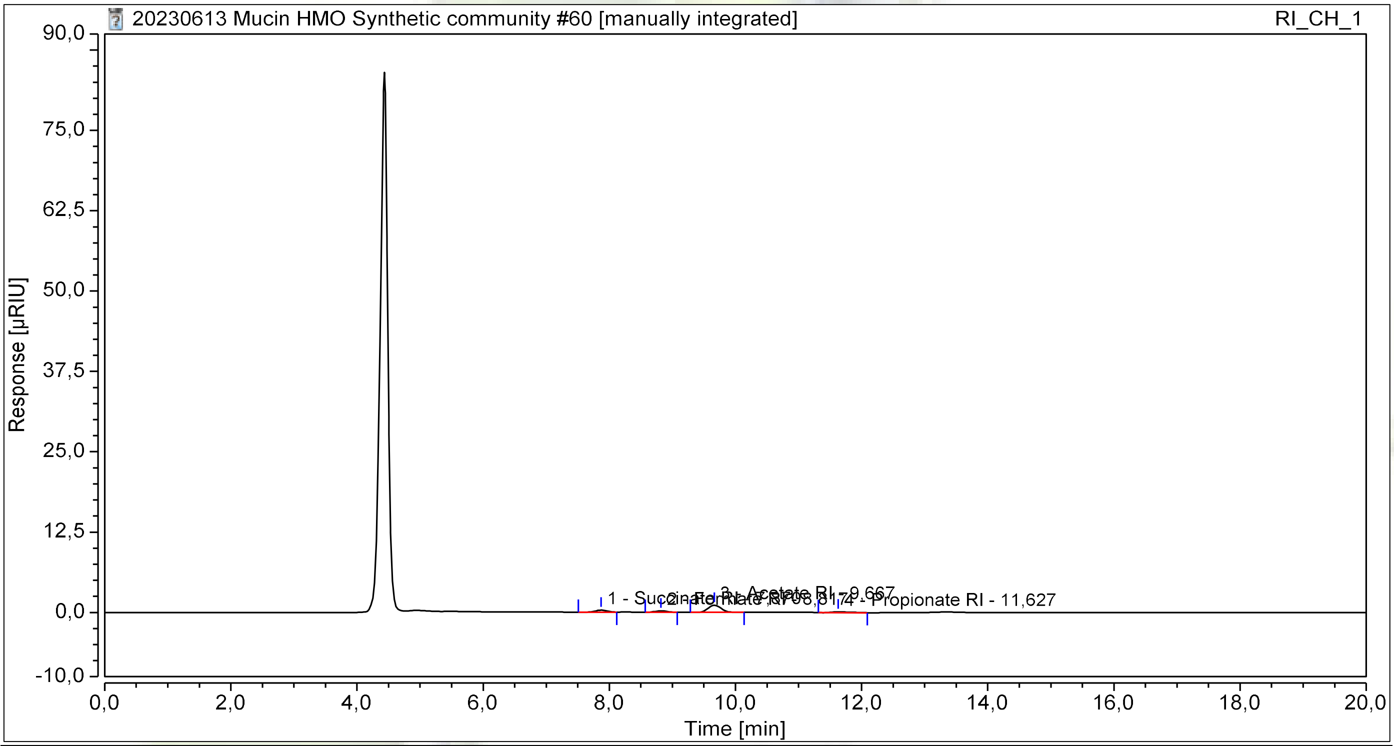

Integration Results

No.	Peak Name	Retention Time min	Area µRIU*min	Height µRIU	Relative Area %	Relative Height %	Amount
n.a.	GlcNAc	n.a.	n.a.	n.a.	n.a.	n.a.	n.a.
n.a.	Citrate	n.a.	n.a.	n.a.	n.a.	n.a.	n.a.
n.a.	Glucose	n.a.	n.a.	n.a.	n.a.	n.a.	n.a.
n.a.	Galactose	n.a.	n.a.	n.a.	n.a.	n.a.	n.a.
n.a.	Fucose	n.a.	n.a.	n.a.	n.a.	n.a.	n.a.
1	Succinate RI	7,870	0,064	0,313	15,40	17,48	n.a.
n.a.	Lactate RI	n.a.	n.a.	n.a.	n.a.	n.a.	n.a.
n.a.	glycerol	n.a.	n.a.	n.a.	n.a.	n.a.	n.a.
2	Formate RI	8,817	0,049	0,233	11,61	13,00	5,0966
3	Acetate RI	9,667	0,266	1,118	63,68	62,42	16,3888
n.a.	1,2 PDO RI	n.a.	n.a.	n.a.	n.a.	n.a.	n.a.
n.a.	1,3-PDO	n.a.	n.a.	n.a.	n.a.	n.a.	n.a.
4	Propionate RI	11,627	0,039	0,127	9,31	7,11	1,5658
n.a.	1,3-PDO	n.a.	n.a.	n.a.	n.a.	n.a.	n.a.
n.a.	2-3 BDO	n.a.	n.a.	n.a.	n.a.	n.a.	n.a.
n.a.	Ethanol	n.a.	n.a.	n.a.	n.a.	n.a.	n.a.
n.a.	Isobutyrate RI	n.a.	n.a.	n.a.	n.a.	n.a.	n.a.
n.a.	Butyrate RI	n.a.	n.a.	n.a.	n.a.	n.a.	n.a.
**Total:**			**0,418**	**1,791**	**100,00**	**100,00**	

Peak Analysis

Injection Details

Injection Name:	**29 GOSFOSMUC t48 r2**	*Run Time (min):*	**20,00**
Vial Number:	**3:E5**	*Injection Volume:*	**20,00**
Injection Type:	**Unknown**	*Channel:*	**RI_CH_1**
Calibration Level:		*Wavelength:*	**n.a.**
Instrument Method:	**Default method LC2030C 45 gr 20 min**	*Bandwidth:*	**n.a.**
Processing Method:	**Processing Method LC2030 45 gr**	*Dilution Factor:*	**1,0000**
Injection Date/Time:	**14-jun-23 08:02**	*Sample Weight:*	**1,0000**

Chromatogram

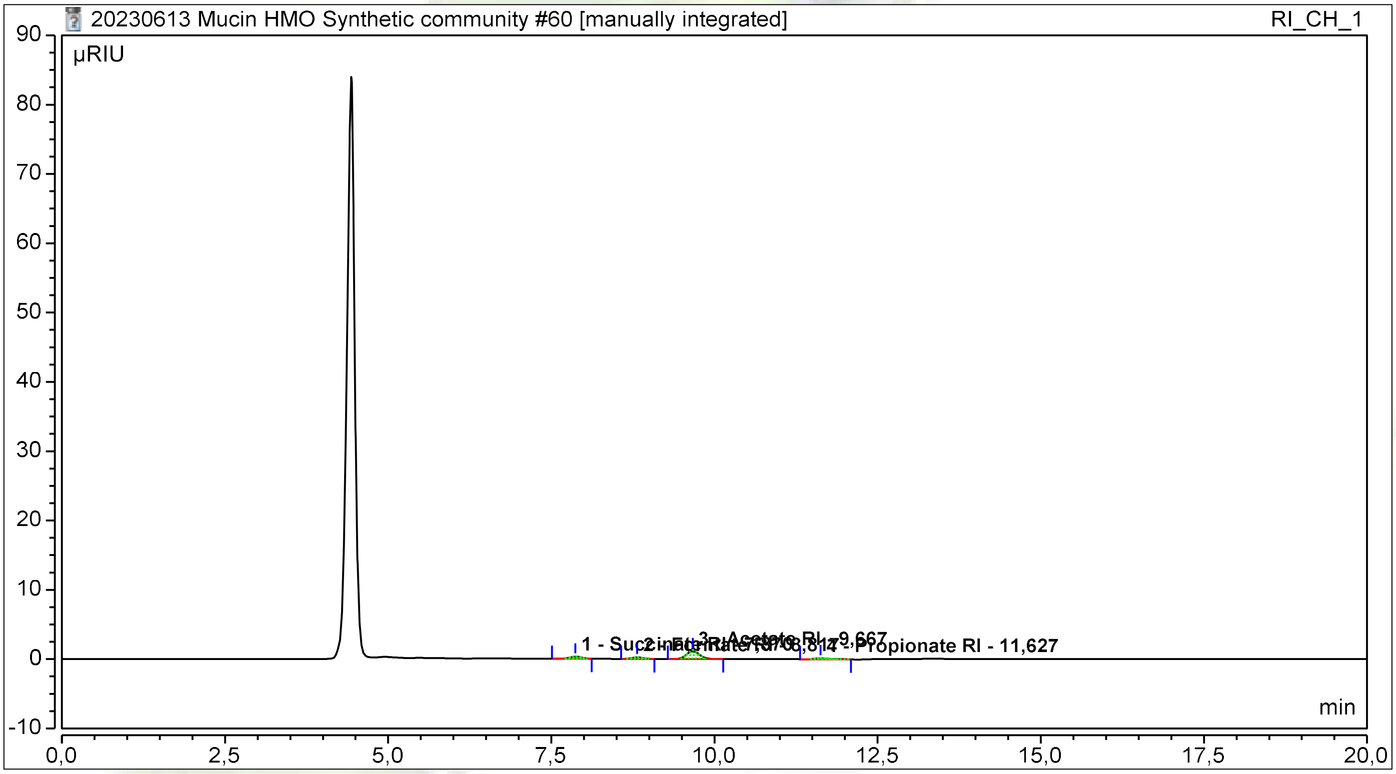

Peak Results

No.	Peak Name	Retention Time min	Width (50%) min	Type	Resolution (EP)	Asymmetry (EP)	Plates (EP)
n.a.	GlcNAc	n.a.	n.a.	n.a.	n.a.	n.a.	n.a.
n.a.	Citrate	n.a.	n.a.	n.a.	n.a.	n.a.	n.a.
n.a.	Glucose	n.a.	n.a.	n.a.	n.a.	n.a.	n.a.
n.a.	Galactose	n.a.	n.a.	n.a.	n.a.	n.a.	n.a.
n.a.	Fucose	n.a.	n.a.	n.a.	n.a.	n.a.	n.a.
1	Succinate RI	7,870	0,198	BMB*	2,81	1,06	8743
n.a.	Lactate RI	n.a.	n.a.	n.a.	n.a.	n.a.	n.a.
n.a.	glycerol	n.a.	n.a.	n.a.	n.a.	n.a.	n.a.
2	Formate RI	8,817	0,199	BMB*	2,36	1,06	10867
3	Acetate RI	9,667	0,225	BMB	4,63	1,08	10198
n.a.	1,2 PDO RI	n.a.	n.a.	n.a.	n.a.	n.a.	n.a.
n.a.	1,3-PDO	n.a.	n.a.	n.a.	n.a.	n.a.	n.a.
4	Propionate RI	11,627	0,275	BMB*	n.a.	1,87	9935
n.a.	1,3-PDO	n.a.	n.a.	n.a.	n.a.	n.a.	n.a.
n.a.	2-3 BDO	n.a.	n.a.	n.a.	n.a.	n.a.	n.a.
n.a.	Ethanol	n.a.	n.a.	n.a.	n.a.	n.a.	n.a.
n.a.	Isobutyrate RI	n.a.	n.a.	n.a.	n.a.	n.a.	n.a.
n.a.	Butyrate RI	n.a.	n.a.	n.a.	n.a.	n.a.	n.a.

Chromeleon (c) Dionex
Version 7.2.10.25868

Chromatogram and SST Results

Injection Details

Injection Name:	**29 GOSFOSMUC t48 r2**	*Run Time (min):*	**20,00**
Vial Number:	**3:E5**	*Injection Volume:*	**20,00**
Injection Type:	**Unknown**	*Channel:*	**RI_CH_1**
Calibration Level:		*Wavelength:*	**n.a.**
Instrument Method:	**Default method LC2030C 45 gr 20 min**	*Bandwidth:*	**n.a.**
Processing Method:	**Processing Method LC2030 45 gr**	*Dilution Factor:*	**1,0000**
Injection Date/Time:	**14-jun-23 08:02**	*Sample Weight:*	**1,0000**

Chromatogram

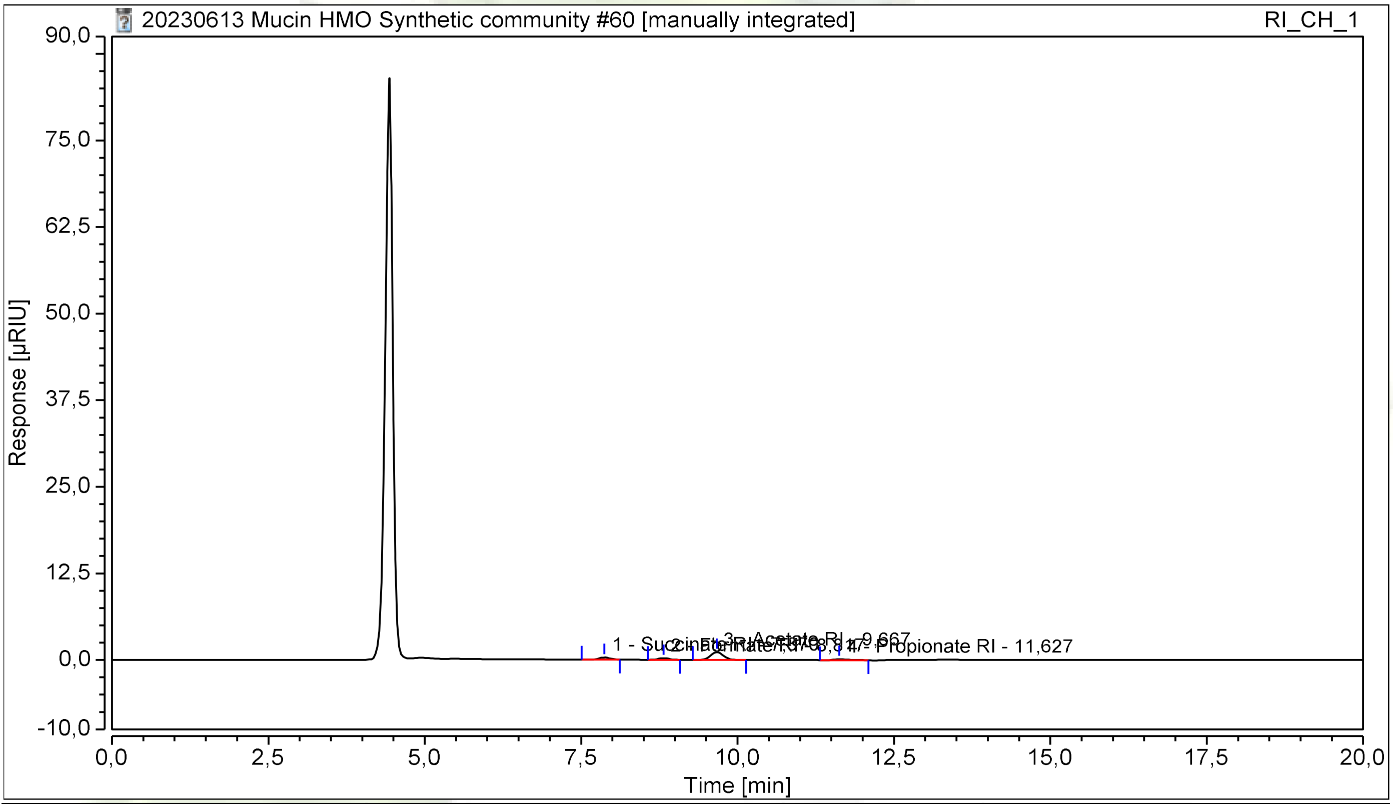

SST Results

No.	Name	Inj.Condition	Peak	Test Result	Injection

Number of executed test cases: **n.a.**   **Total Result:** **Passed**

Chromatogram

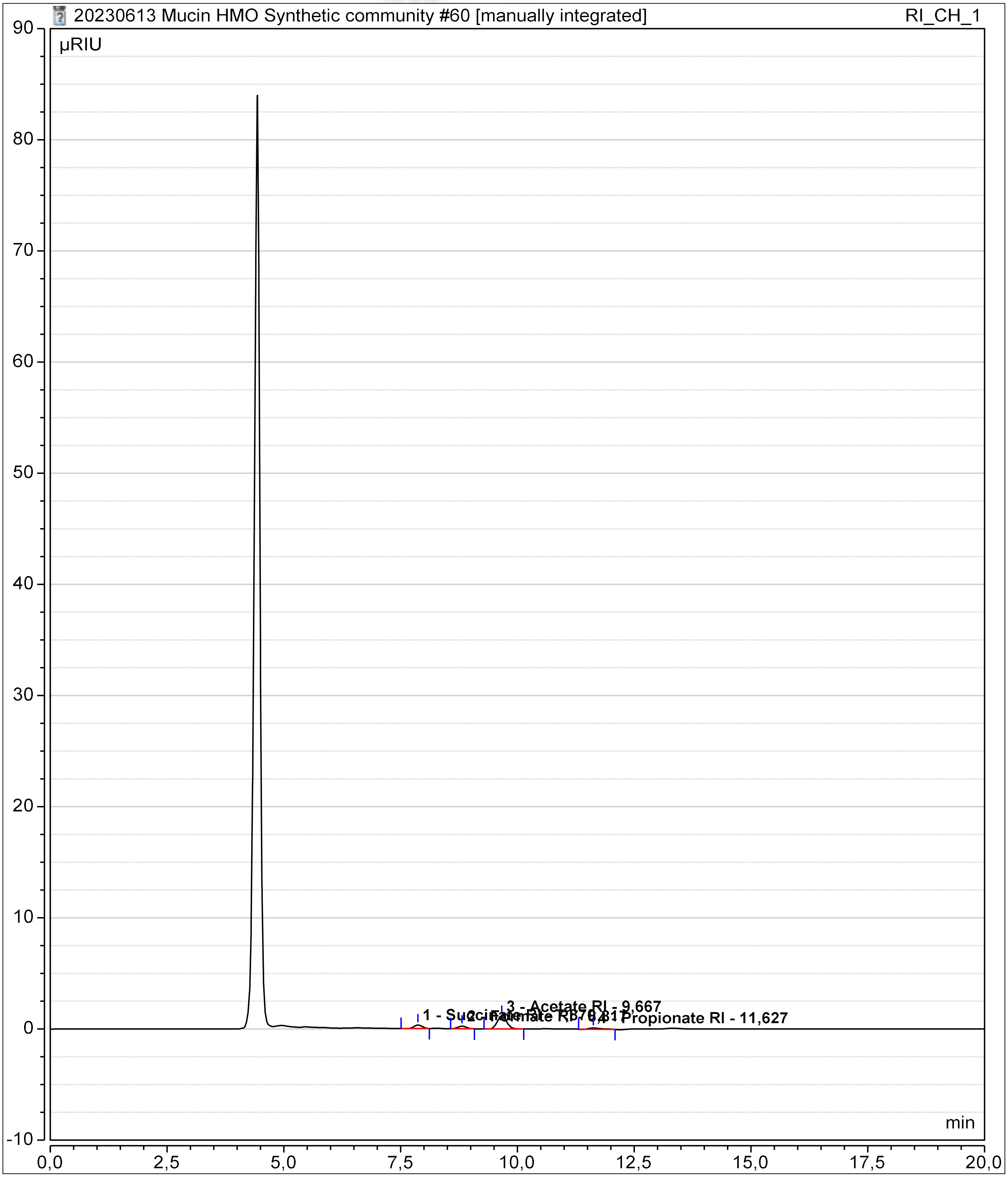

Chromatogram and Results

Injection Details

Injection Name:	**30 GOSFOSMUC t48 r3**	*Run Time (min):*	**20,00**
Vial Number:	**3:E6**	*Injection Volume:*	**20,00**
Injection Type:	**Unknown**	*Channel:*	**RI_CH_1**
Calibration Level:		*Wavelength:*	**n.a.**
Instrument Method:	**Default method LC2030C 45 gr 20 min**	*Bandwidth:*	**n.a.**
Processing Method:	**Processing Method LC2030 45 gr**	*Dilution Factor:*	**1,0000**
Injection Date/Time:	**14-jun-23 08:23**	*Sample Weight:*	**1,0000**

Chromatogram

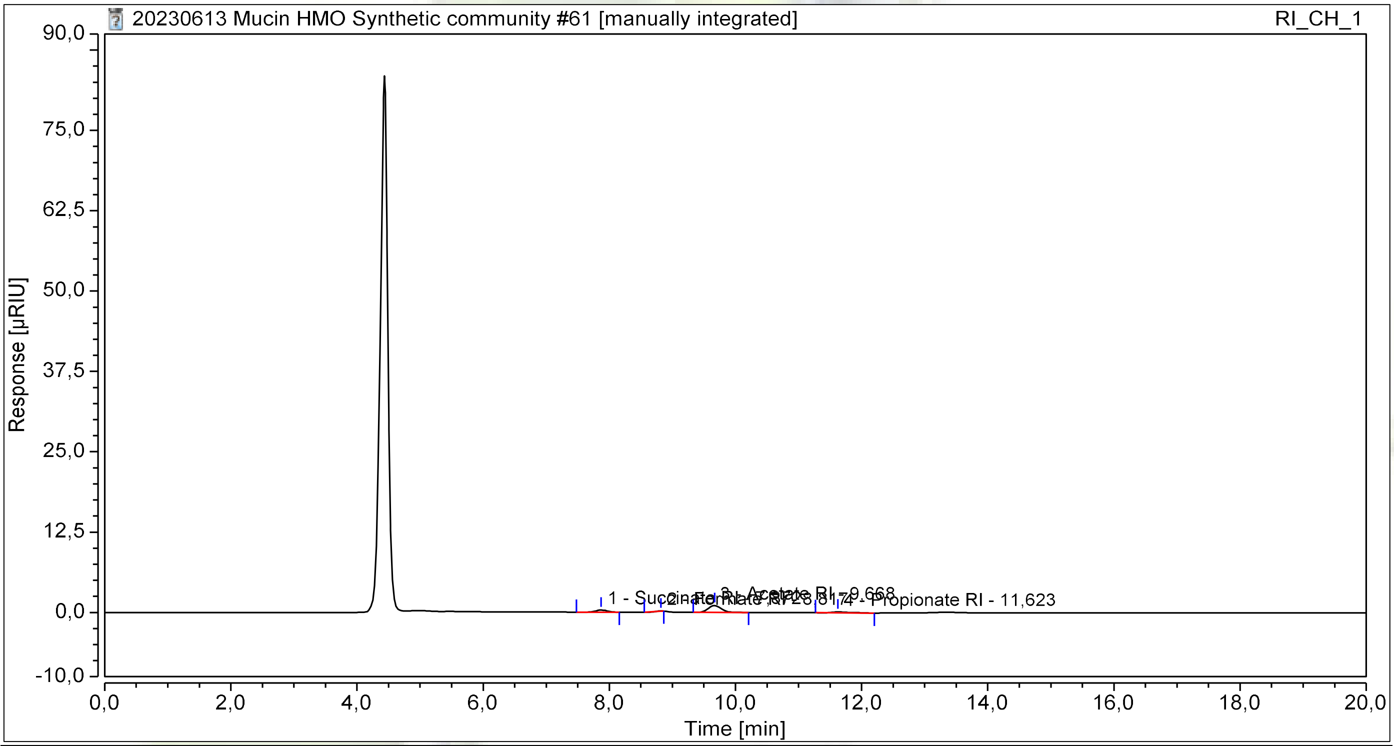

Integration Results

No.	Peak Name	Retention Time min	Area µRIU*min	Height µRIU	Relative Area %	Relative Height %	Amount
n.a.	GlcNAc	n.a.	n.a.	n.a.	n.a.	n.a.	n.a.
n.a.	Citrate	n.a.	n.a.	n.a.	n.a.	n.a.	n.a.
n.a.	Glucose	n.a.	n.a.	n.a.	n.a.	n.a.	n.a.
n.a.	Galactose	n.a.	n.a.	n.a.	n.a.	n.a.	n.a.
n.a.	Fucose	n.a.	n.a.	n.a.	n.a.	n.a.	n.a.
1	Succinate RI	7,872	0,070	0,333	18,79	20,88	n.a.
n.a.	Lactate RI	n.a.	n.a.	n.a.	n.a.	n.a.	n.a.
n.a.	glycerol	n.a.	n.a.	n.a.	n.a.	n.a.	n.a.
2	Formate RI	8,817	0,003	0,058	0,69	3,62	0,2687
3	Acetate RI	9,668	0,254	1,066	68,69	66,93	15,6349
n.a.	1,2 PDO RI	n.a.	n.a.	n.a.	n.a.	n.a.	n.a.
n.a.	1,3-PDO	n.a.	n.a.	n.a.	n.a.	n.a.	n.a.
4	Propionate RI	11,623	0,044	0,137	11,82	8,58	1,7585
n.a.	1,3-PDO	n.a.	n.a.	n.a.	n.a.	n.a.	n.a.
n.a.	2-3 BDO	n.a.	n.a.	n.a.	n.a.	n.a.	n.a.
n.a.	Ethanol	n.a.	n.a.	n.a.	n.a.	n.a.	n.a.
n.a.	Isobutyrate RI	n.a.	n.a.	n.a.	n.a.	n.a.	n.a.
n.a.	Butyrate RI	n.a.	n.a.	n.a.	n.a.	n.a.	n.a.
**Total:**			**0,370**	**1,593**	**100,00**	**100,00**	

Peak Analysis

Injection Details

Injection Name:	**30 GOSFOSMUC t48 r3**	*Run Time (min):*	**20,00**
Vial Number:	**3:E6**	*Injection Volume:*	**20,00**
Injection Type:	**Unknown**	*Channel:*	**RI_CH_1**
Calibration Level:		*Wavelength:*	**n.a.**
Instrument Method:	**Default method LC2030C 45 gr 20 min**	*Bandwidth:*	**n.a.**
Processing Method:	**Processing Method LC2030 45 gr**	*Dilution Factor:*	**1,0000**
Injection Date/Time:	**14-jun-23 08:23**	*Sample Weight:*	**1,0000**

Chromatogram

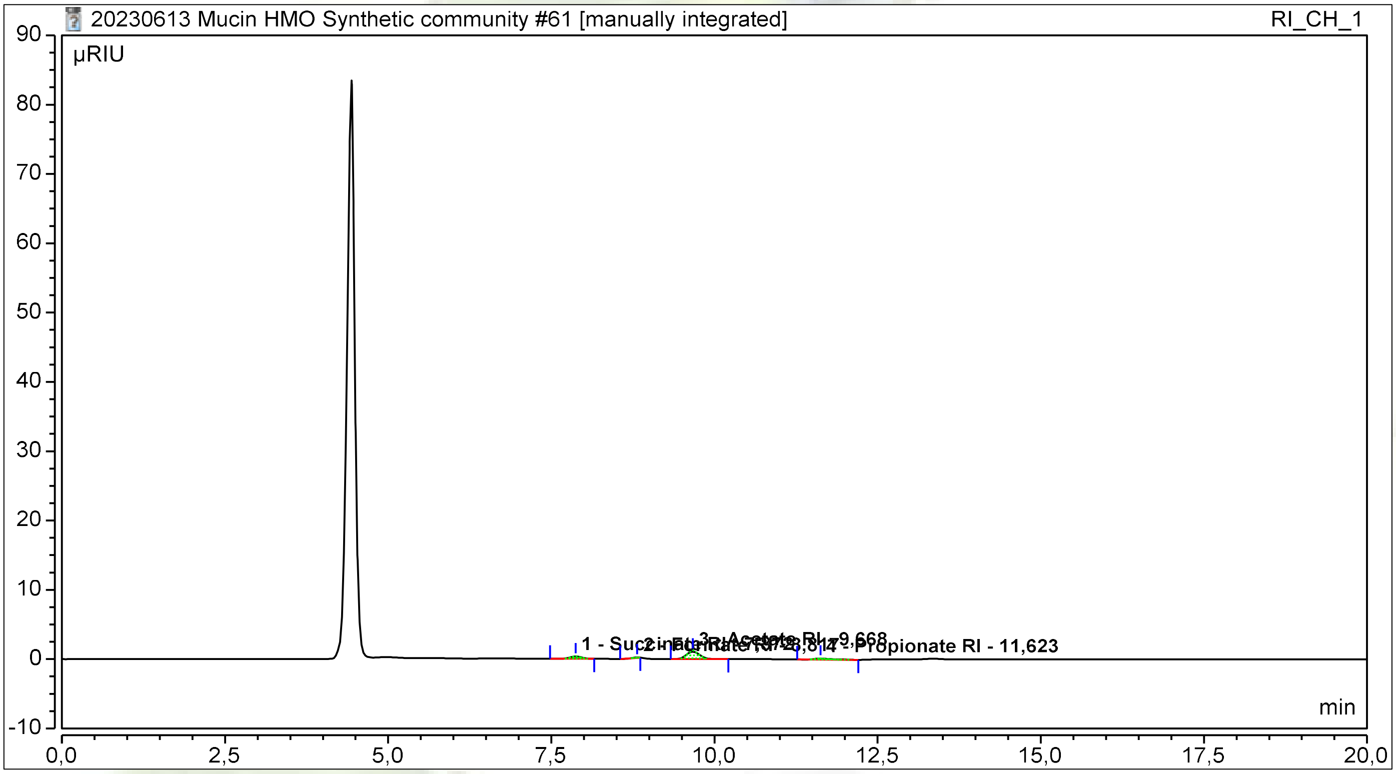

Peak Results

No.	Peak Name	Retention Time<br>min	Width (50%)<br>min	Type	Resolution (EP)	Asymmetry (EP)	Plates (EP)
n.a.	GlcNAc	n.a.	n.a.	n.a.	n.a.	n.a.	n.a.
n.a.	Citrate	n.a.	n.a.	n.a.	n.a.	n.a.	n.a.
n.a.	Glucose	n.a.	n.a.	n.a.	n.a.	n.a.	n.a.
n.a.	Galactose	n.a.	n.a.	n.a.	n.a.	n.a.	n.a.
n.a.	Fucose	n.a.	n.a.	n.a.	n.a.	n.a.	n.a.
1	Succinate RI	7,872	0,200	BMB*	3,65	1,07	8569
n.a.	Lactate RI	n.a.	n.a.	n.a.	n.a.	n.a.	n.a.
n.a.	glycerol	n.a.	n.a.	n.a.	n.a.	n.a.	n.a.
2	Formate RI	8,817	0,105	BMB*	3,04	0,73	38751
3	Acetate RI	9,668	0,225	BMB	4,50	1,08	10211
n.a.	1,2 PDO RI	n.a.	n.a.	n.a.	n.a.	n.a.	n.a.
n.a.	1,3-PDO	n.a.	n.a.	n.a.	n.a.	n.a.	n.a.
4	Propionate RI	11,623	0,288	BMB*	n.a.	2,09	9054
n.a.	1,3-PDO	n.a.	n.a.	n.a.	n.a.	n.a.	n.a.
n.a.	2-3 BDO	n.a.	n.a.	n.a.	n.a.	n.a.	n.a.
n.a.	Ethanol	n.a.	n.a.	n.a.	n.a.	n.a.	n.a.
n.a.	Isobutyrate RI	n.a.	n.a.	n.a.	n.a.	n.a.	n.a.
n.a.	Butyrate RI	n.a.	n.a.	n.a.	n.a.	n.a.	n.a.

Chromatogram and SST Results

Injection Details

Injection Name:	**30 GOSFOSMUC t48 r3**	*Run Time (min):*	**20,00**
Vial Number:	**3:E6**	*Injection Volume:*	**20,00**
Injection Type:	**Unknown**	*Channel:*	**RI_CH_1**
Calibration Level:		*Wavelength:*	**n.a.**
Instrument Method:	**Default method LC2030C 45 gr 20 min**	*Bandwidth:*	**n.a.**
Processing Method:	**Processing Method LC2030 45 gr**	*Dilution Factor:*	**1,0000**
Injection Date/Time:	**14-jun-23 08:23**	*Sample Weight:*	**1,0000**

Chromatogram

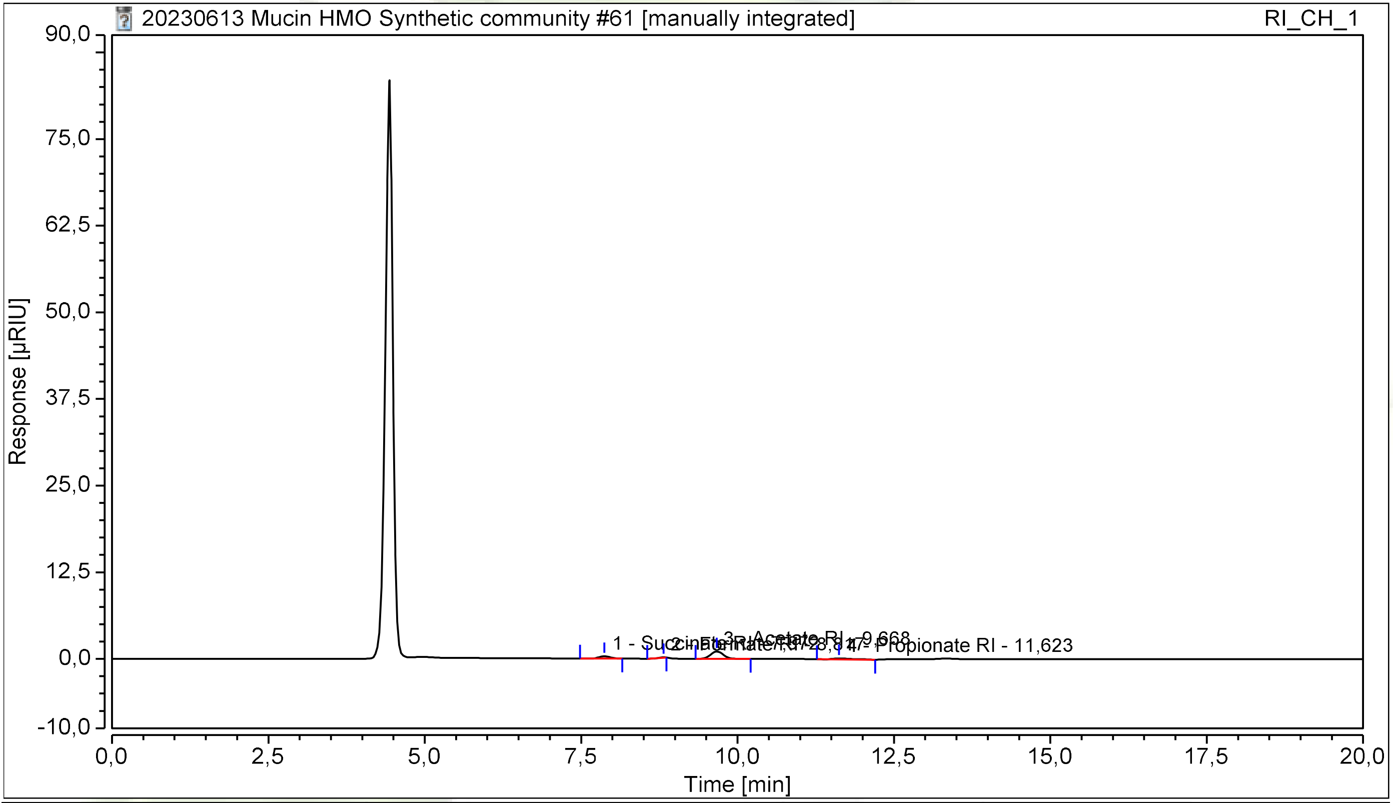

SST Results

No.	Name	Inj.Condition	Peak	Test Result	Injection

**Number of executed test cases:**	**n.a.**	**Total Result:**	**Passed**

Chromatogram

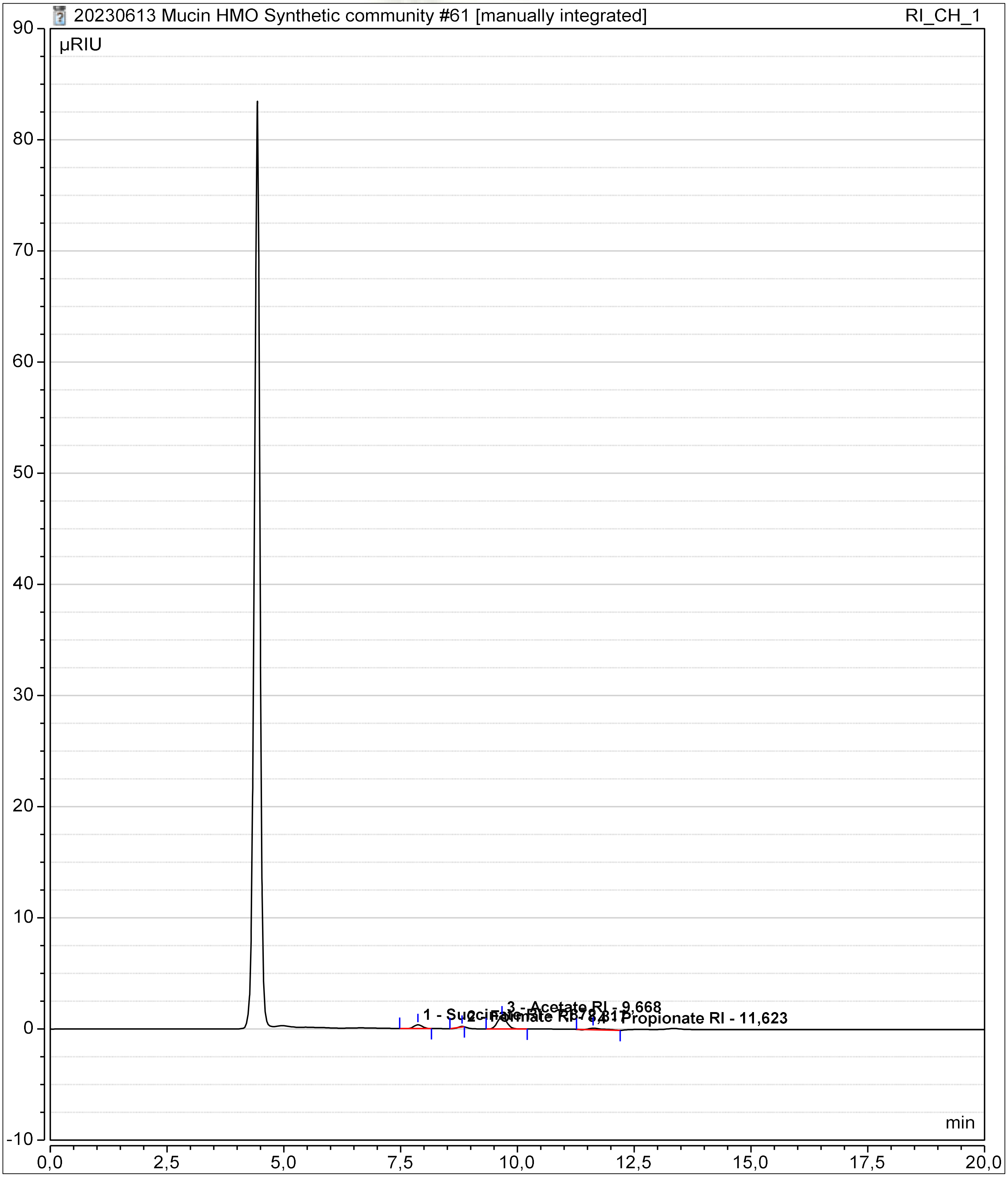

Chromeleon (c) Dionex
Version 7.2.10.25868

Chromatogram and Results

Injection Details

Injection Name:	**34 MUCHMO3 t24 r1**	*Run Time (min):*	**20,00**
Vial Number:	**3:E7**	*Injection Volume:*	**20,00**
Injection Type:	**Unknown**	*Channel:*	**RI_CH_1**
Calibration Level:		*Wavelength:*	**n.a.**
Instrument Method:	**Default method LC2030C 45 gr 20 min**	*Bandwidth:*	**n.a.**
Processing Method:	**Processing Method LC2030 45 gr**	*Dilution Factor:*	**1,0000**
Injection Date/Time:	**14-jun-23 08:43**	*Sample Weight:*	**1,0000**

Chromatogram

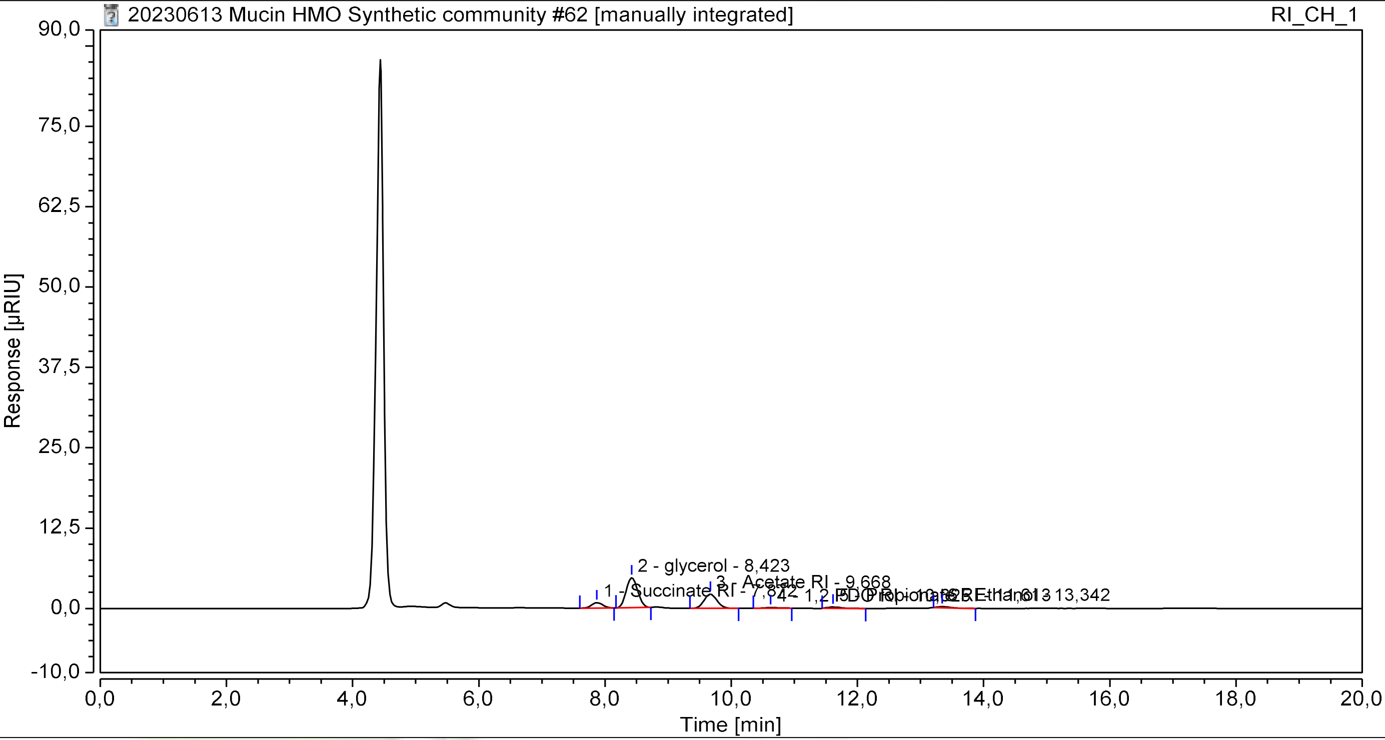

Integration Results

No.	Peak Name	Retention Time min	Area µRIU*min	Height µRIU	Relative Area %	Relative Height %	Amount
n.a.	GlcNAc	n.a.	n.a.	n.a.	n.a.	n.a.	n.a.
n.a.	Citrate	n.a.	n.a.	n.a.	n.a.	n.a.	n.a.
n.a.	Glucose	n.a.	n.a.	n.a.	n.a.	n.a.	n.a.
n.a.	Galactose	n.a.	n.a.	n.a.	n.a.	n.a.	n.a.
n.a.	Fucose	n.a.	n.a.	n.a.	n.a.	n.a.	n.a.
1	Succinate RI	7,872	0,175	0,834	9,79	10,28	n.a.
n.a.	Lactate RI	n.a.	n.a.	n.a.	n.a.	n.a.	n.a.
2	glycerol	8,423	0,979	4,640	54,89	57,21	n.a.
n.a.	Formate RI	n.a.	n.a.	n.a.	n.a.	n.a.	n.a.
3	Acetate RI	9,668	0,520	2,190	29,14	27,00	31,9754
4	1,2 PDO RI	10,625	0,028	0,094	1,54	1,16	0,8192
n.a.	1,3-PDO	n.a.	n.a.	n.a.	n.a.	n.a.	n.a.
5	Propionate RI	11,613	0,058	0,206	3,27	2,54	2,3443
n.a.	1,3-PDO	n.a.	n.a.	n.a.	n.a.	n.a.	n.a.
n.a.	2-3 BDO	n.a.	n.a.	n.a.	n.a.	n.a.	n.a.
6	Ethanol	13,342	0,024	0,146	1,37	1,81	0,2256
n.a.	Isobutyrate RI	n.a.	n.a.	n.a.	n.a.	n.a.	n.a.
n.a.	Butyrate RI	n.a.	n.a.	n.a.	n.a.	n.a.	n.a.
**Total:**			**1,783**	**8,109**	**100,00**	**100,00**	

Peak Analysis

Injection Details

Injection Name:	**34 MUCHMO3 t24 r1**	*Run Time (min):*	**20,00**
Vial Number:	**3:E7**	*Injection Volume:*	**20,00**
Injection Type:	**Unknown**	*Channel:*	**RI_CH_1**
Calibration Level:		*Wavelength:*	**n.a.**
Instrument Method:	**Default method LC2030C 45 gr 20 min**	*Bandwidth:*	**n.a.**
Processing Method:	**Processing Method LC2030 45 gr**	*Dilution Factor:*	**1,0000**
Injection Date/Time:	**14-jun-23 08:43**	*Sample Weight:*	**1,0000**

Chromatogram

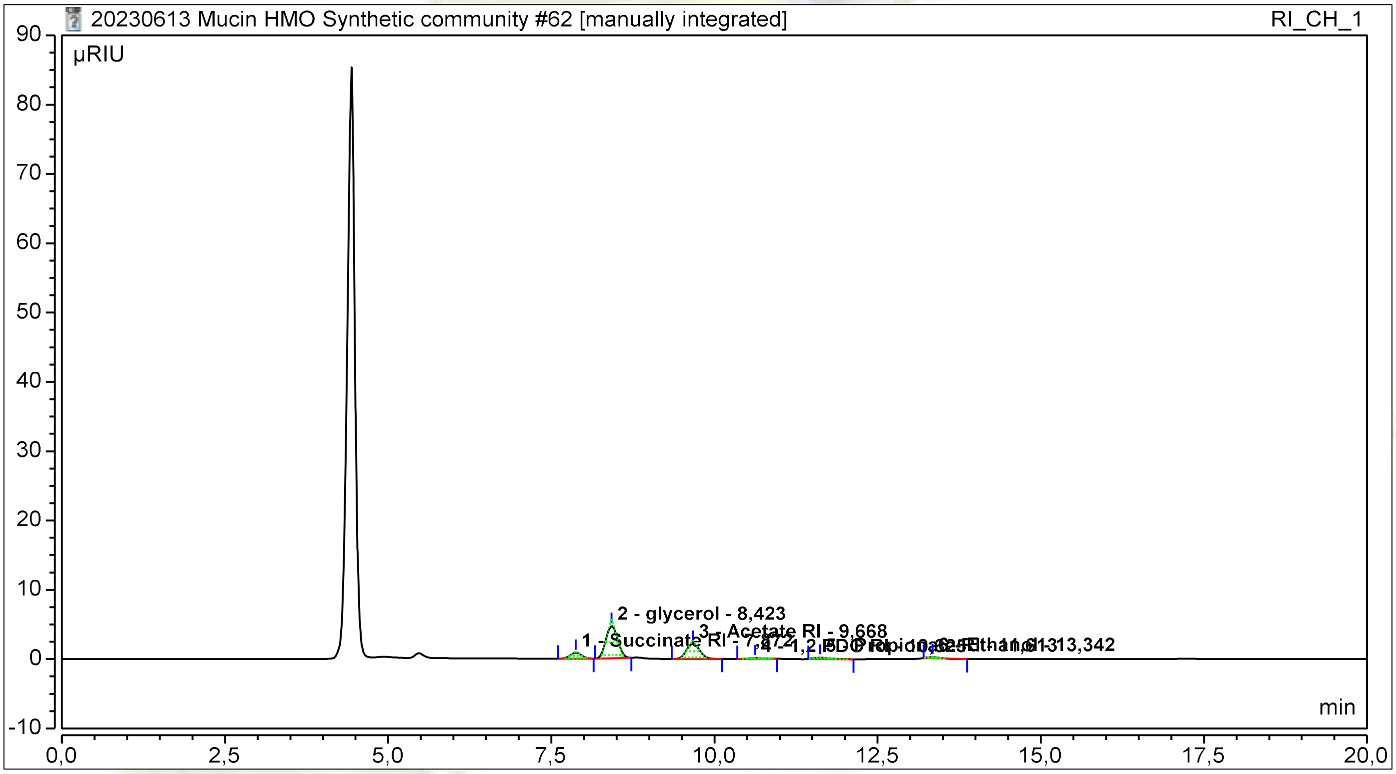

Peak Results

No.	Peak Name	Retention Time min	Width (50%) min	Type	Resolution (EP)	Asymmetry (EP)	Plates (EP)
n.a.	GlcNAc	n.a.	n.a.	n.a.	n.a.	n.a.	n.a.
n.a.	Citrate	n.a.	n.a.	n.a.	n.a.	n.a.	n.a.
n.a.	Glucose	n.a.	n.a.	n.a.	n.a.	n.a.	n.a.
n.a.	Galactose	n.a.	n.a.	n.a.	n.a.	n.a.	n.a.
n.a.	Fucose	n.a.	n.a.	n.a.	n.a.	n.a.	n.a.
1	Succinate RI	7,872	0,200	BMB	1,62	1,06	8601
n.a.	Lactate RI	n.a.	n.a.	n.a.	n.a.	n.a.	n.a.
2	glycerol	8,423	0,201	BMB*	3,45	1,07	9695
n.a.	Formate RI	n.a.	n.a.	n.a.	n.a.	n.a.	n.a.
3	Acetate RI	9,668	0,225	BMB	2,25	1,08	10270
4	1,2 PDO RI	10,625	0,277	BMB*	2,24	1,21	8169
n.a.	1,3-PDO	n.a.	n.a.	n.a.	n.a.	n.a.	n.a.
5	Propionate RI	11,613	0,245	BMB*	4,43	1,93	12490
n.a.	1,3-PDO	n.a.	n.a.	n.a.	n.a.	n.a.	n.a.
n.a.	2-3 BDO	n.a.	n.a.	n.a.	n.a.	n.a.	n.a.
6	Ethanol	13,342	0,216	BMB*	n.a.	1,27	21215
n.a.	Isobutyrate RI	n.a.	n.a.	n.a.	n.a.	n.a.	n.a.
n.a.	Butyrate RI	n.a.	n.a.	n.a.	n.a.	n.a.	n.a.

Chromatogram and SST Results

Injection Details

Injection Name:	**34 MUCHMO3 t24 r1**	*Run Time (min):*	**20,00**
Vial Number:	**3:E7**	*Injection Volume:*	**20,00**
Injection Type:	**Unknown**	*Channel:*	**RI_CH_1**
Calibration Level:		*Wavelength:*	**n.a.**
Instrument Method:	**Default method LC2030C 45 gr 20 min**	*Bandwidth:*	**n.a.**
Processing Method:	**Processing Method LC2030 45 gr**	*Dilution Factor:*	**1,0000**
Injection Date/Time:	**14-jun-23 08:43**	*Sample Weight:*	**1,0000**

Chromatogram

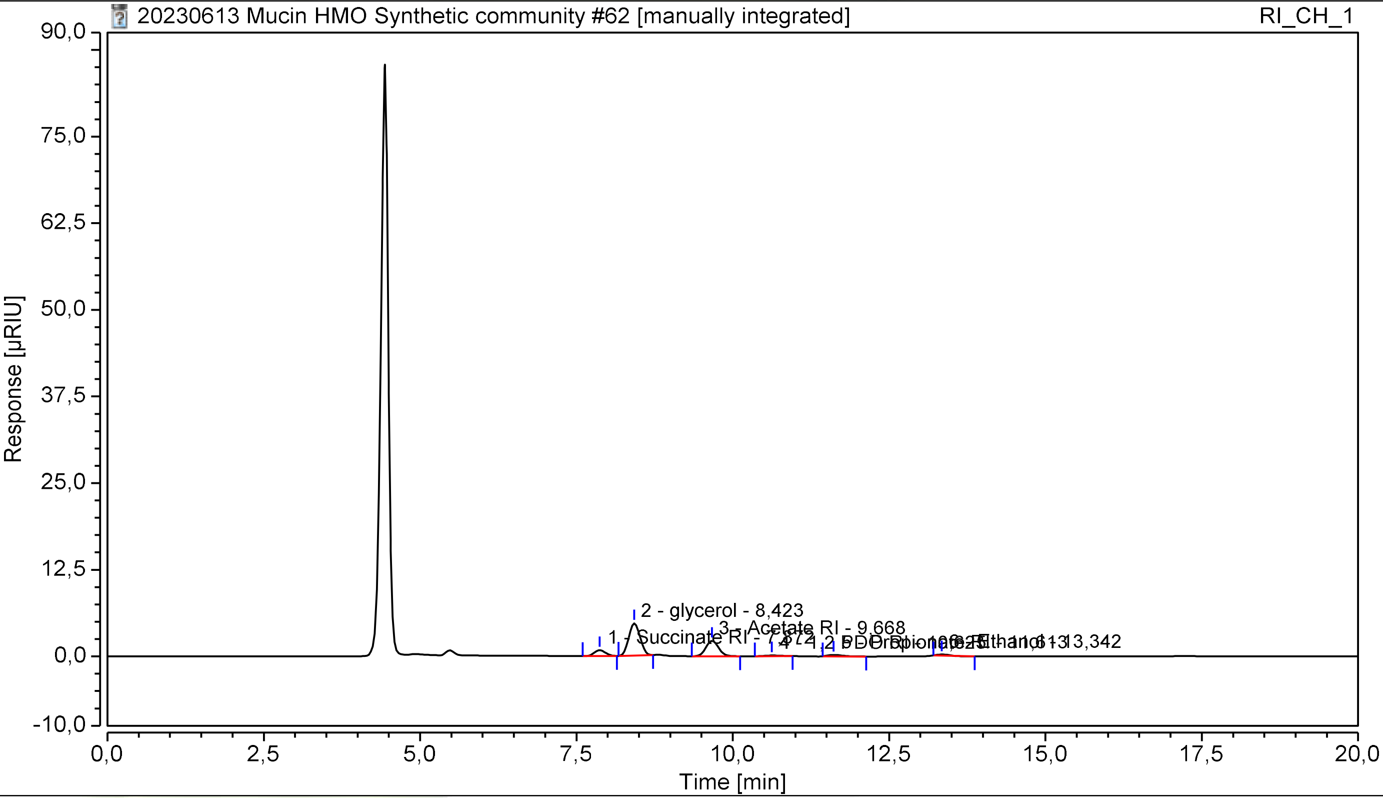

SST Results

No.	Name	Inj.Condition	Peak	Test Result	Injection
**Number of executed test cases:**	**n.a.**		**Total Result:**	**Passed**	

Chromatogram

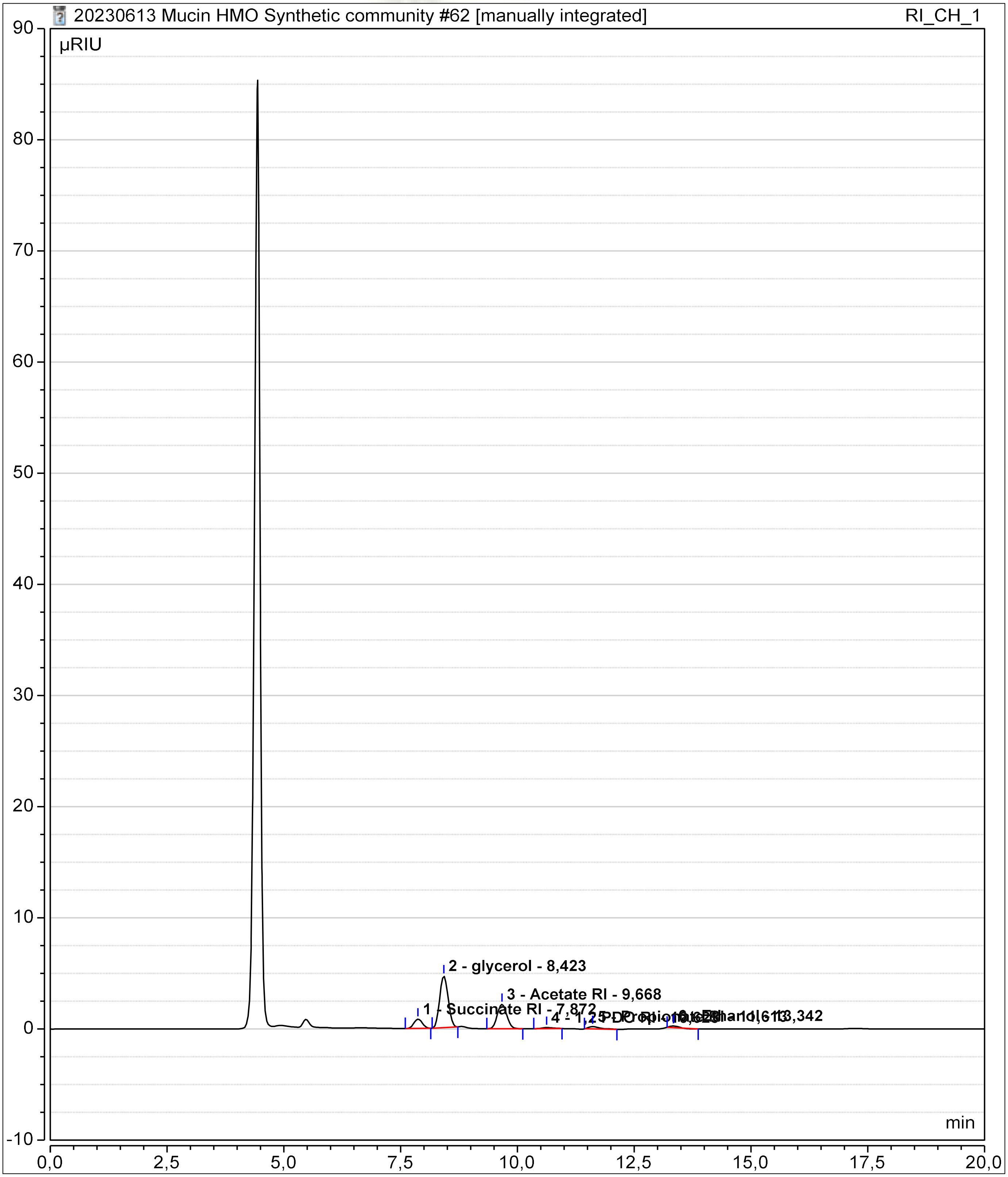

Chromeleon (c) Dionex
Version 7.2.10.25868

Chromatogram and Results

Injection Details

Injection Name:	**35 MUCHMO3 t24 r2**	*Run Time (min):*	**20,00**
Vial Number:	**3:E8**	*Injection Volume:*	**20,00**
Injection Type:	**Unknown**	*Channel:*	**RI_CH_1**
Calibration Level:		*Wavelength:*	**n.a.**
Instrument Method:	**Default method LC2030C 45 gr 20 min**	*Bandwidth:*	**n.a.**
Processing Method:	**Processing Method LC2030 45 gr**	*Dilution Factor:*	**1,0000**
Injection Date/Time:	**14-jun-23 09:03**	*Sample Weight:*	**1,0000**

Chromatogram

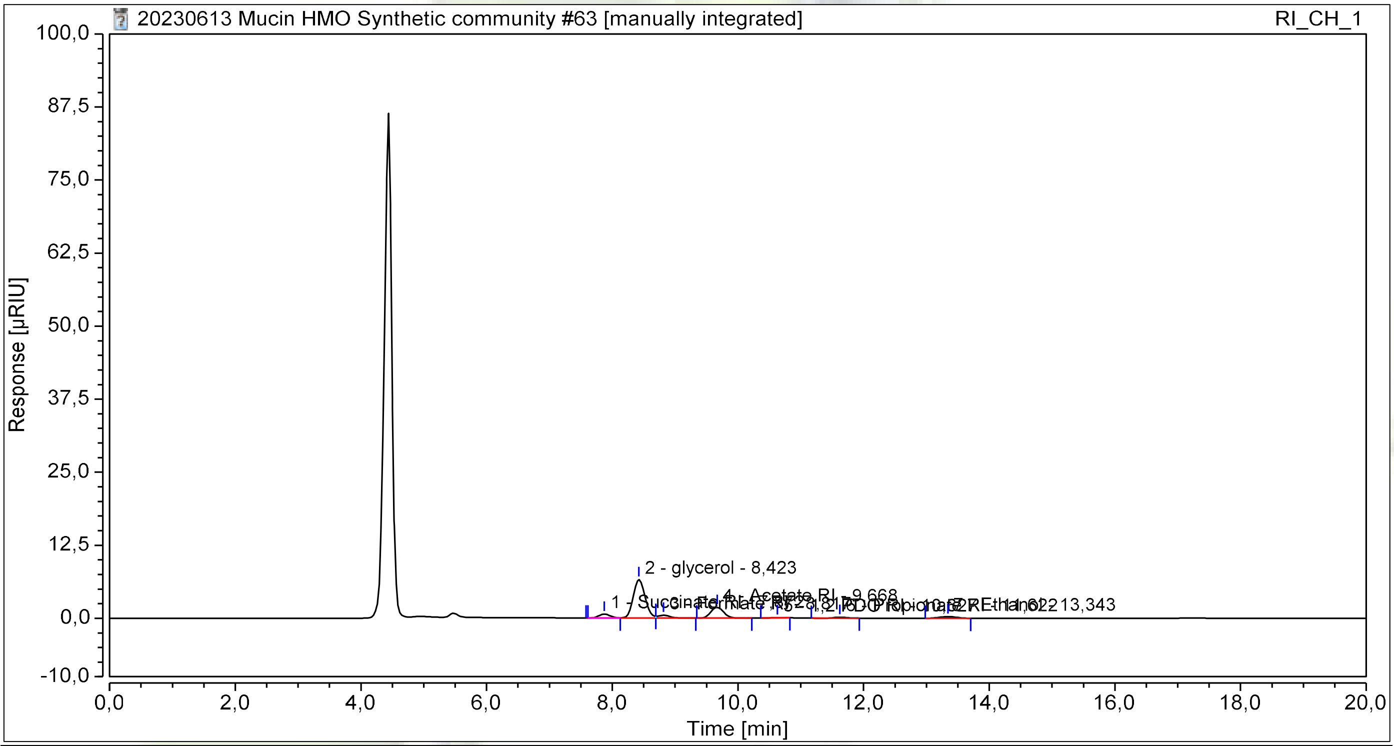

Integration Results

No.	Peak Name	Retention Time<br>min	Area<br>µRIU*min	Height<br>µRIU	Relative Area<br>%	Relative Height<br>%	Amount
n.a.	GlcNAc	n.a.	n.a.	n.a.	n.a.	n.a.	n.a.
n.a.	Citrate	n.a.	n.a.	n.a.	n.a.	n.a.	n.a.
n.a.	Glucose	n.a.	n.a.	n.a.	n.a.	n.a.	n.a.
n.a.	Galactose	n.a.	n.a.	n.a.	n.a.	n.a.	n.a.
n.a.	Fucose	n.a.	n.a.	n.a.	n.a.	n.a.	n.a.
1	Succinate RI	7,872	0,136	0,655	6,02	6,50	n.a.
n.a.	Lactate RI	n.a.	n.a.	n.a.	n.a.	n.a.	n.a.
2	glycerol	8,423	1,448	6,605	64,16	65,47	n.a.
3	Formate RI	8,817	0,107	0,489	4,72	4,85	11,1844
4	Acetate RI	9,668	0,436	1,837	19,34	18,21	26,8630
5	1,2 PDO RI	10,627	0,016	0,069	0,69	0,69	0,4643
n.a.	1,3-PDO	n.a.	n.a.	n.a.	n.a.	n.a.	n.a.
6	Propionate RI	11,622	0,029	0,156	1,29	1,55	1,1715
n.a.	1,3-PDO	n.a.	n.a.	n.a.	n.a.	n.a.	n.a.
n.a.	2-3 BDO	n.a.	n.a.	n.a.	n.a.	n.a.	n.a.
7	Ethanol	13,343	0,085	0,277	3,78	2,74	0,7855
n.a.	Isobutyrate RI	n.a.	n.a.	n.a.	n.a.	n.a.	n.a.
n.a.	Butyrate RI	n.a.	n.a.	n.a.	n.a.	n.a.	n.a.
**Total:**			**2,257**	**10,088**	**100,00**	**100,00**	

Peak Analysis

Injection Details

Injection Name:	**35 MUCHMO3 t24 r2**	*Run Time (min):*	**20,00**
Vial Number:	**3:E8**	*Injection Volume:*	**20,00**
Injection Type:	**Unknown**	*Channel:*	**RI_CH_1**
Calibration Level:		*Wavelength:*	**n.a.**
Instrument Method:	**Default method LC2030C 45 gr 20 min**	*Bandwidth:*	**n.a.**
Processing Method:	**Processing Method LC2030 45 gr**	*Dilution Factor:*	**1,0000**
Injection Date/Time:	**14-jun-23 09:03**	*Sample Weight:*	**1,0000**

Chromatogram

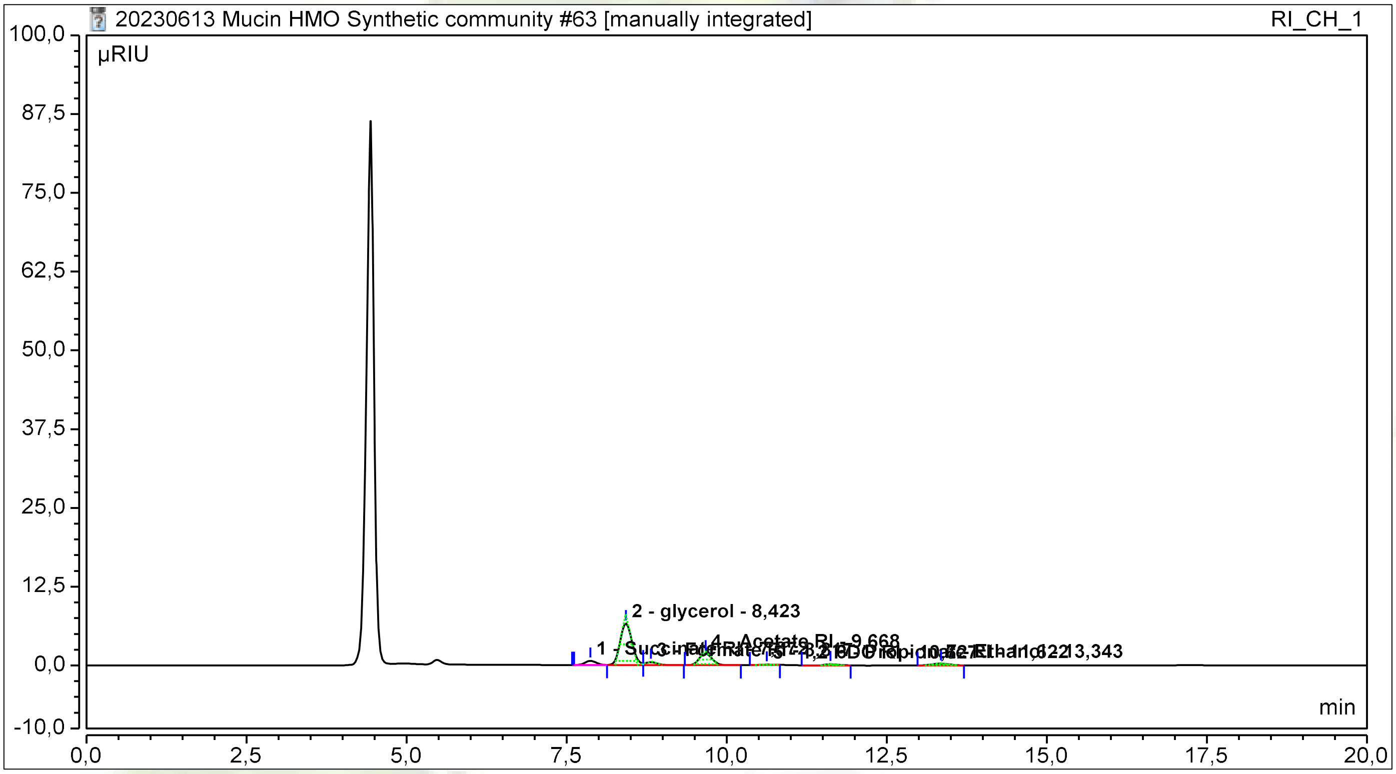

Peak Results

No.	Peak Name	Retention Time min	Width (50%) min	Type	Resolution (EP)	Asymmetry (EP)	Plates (EP)
n.a.	GlcNAc	n.a.	n.a.	n.a.	n.a.	n.a.	n.a.
n.a.	Citrate	n.a.	n.a.	n.a.	n.a.	n.a.	n.a.
n.a.	Glucose	n.a.	n.a.	n.a.	n.a.	n.a.	n.a.
n.a.	Galactose	n.a.	n.a.	n.a.	n.a.	n.a.	n.a.
n.a.	Fucose	n.a.	n.a.	n.a.	n.a.	n.a.	n.a.
1	Succinate RI	7,872	n.a.	Ru	n.a.	n.a.	n.a.
n.a.	Lactate RI	n.a.	n.a.	n.a.	n.a.	n.a.	n.a.
2	glycerol	8,423	0,205	BM	n.a.	1,13	9382
3	Formate RI	8,817	n.a.	MB	n.a.	n.a.	n.a.
4	Acetate RI	9,668	0,225	BMB	2,53	1,08	10250
5	1,2 PDO RI	10,627	0,223	BMB*	2,65	0,97	12593
n.a.	1,3-PDO	n.a.	n.a.	n.a.	n.a.	n.a.	n.a.
6	Propionate RI	11,622	0,220	BMB*	3,93	1,30	15457
n.a.	1,3-PDO	n.a.	n.a.	n.a.	n.a.	n.a.	n.a.
n.a.	2-3 BDO	n.a.	n.a.	n.a.	n.a.	n.a.	n.a.
7	Ethanol	13,343	0,297	BMB*	n.a.	1,03	11159
n.a.	Isobutyrate RI	n.a.	n.a.	n.a.	n.a.	n.a.	n.a.
n.a.	Butyrate RI	n.a.	n.a.	n.a.	n.a.	n.a.	n.a.

Chromatogram and SST Results

Injection Details

Injection Name:	**35 MUCHMO3 t24 r2**	*Run Time (min):*	**20,00**
Vial Number:	**3:E8**	*Injection Volume:*	**20,00**
Injection Type:	**Unknown**	*Channel:*	**RI_CH_1**
Calibration Level:		*Wavelength:*	**n.a.**
Instrument Method:	**Default method LC2030C 45 gr 20 min**	*Bandwidth:*	**n.a.**
Processing Method:	**Processing Method LC2030 45 gr**	*Dilution Factor:*	**1,0000**
Injection Date/Time:	**14-jun-23 09:03**	*Sample Weight:*	**1,0000**

Chromatogram

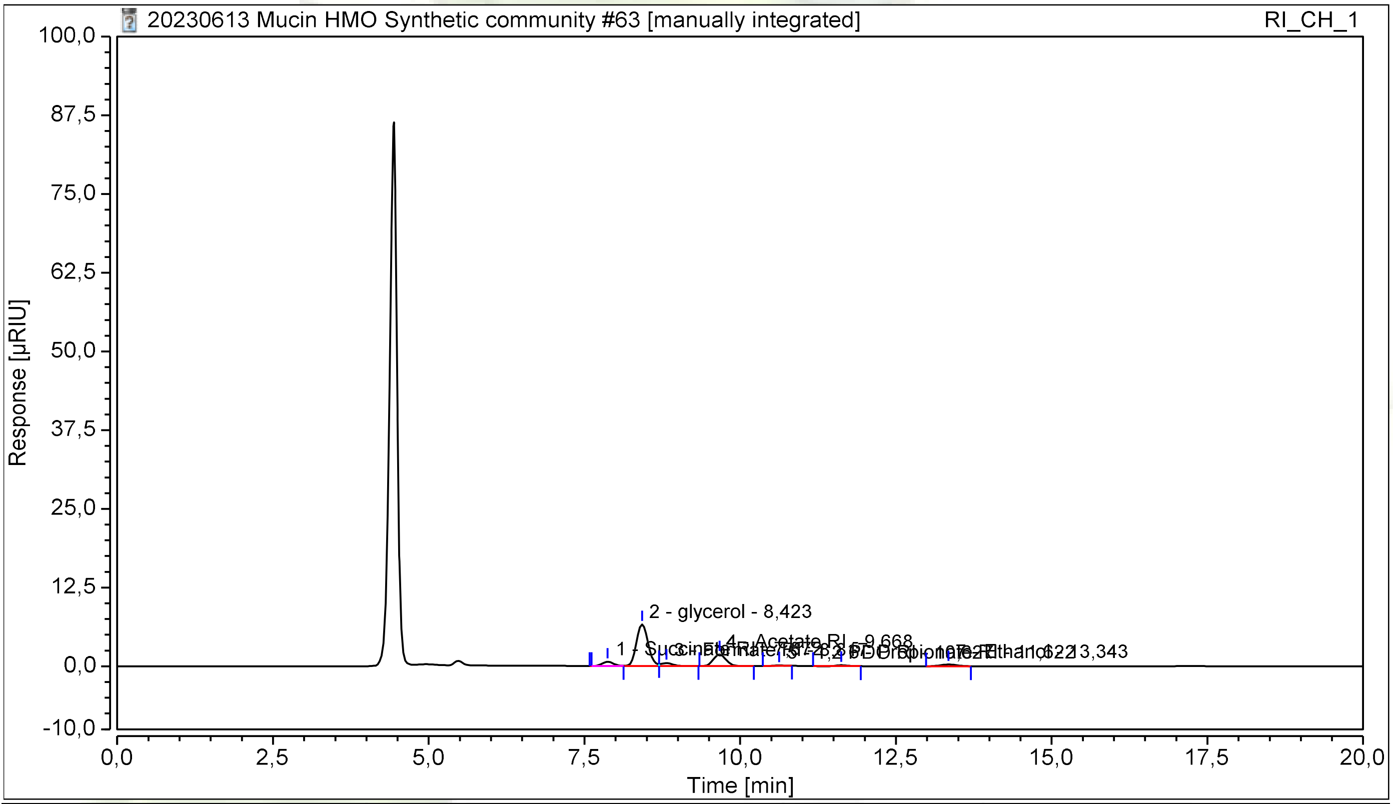

SST Results

No.	Name	Inj.Condition	Peak	Test Result	Injection

**Number of executed test cases:**	**n.a.**	**Total Result:**	**Passed**

Chromatogram

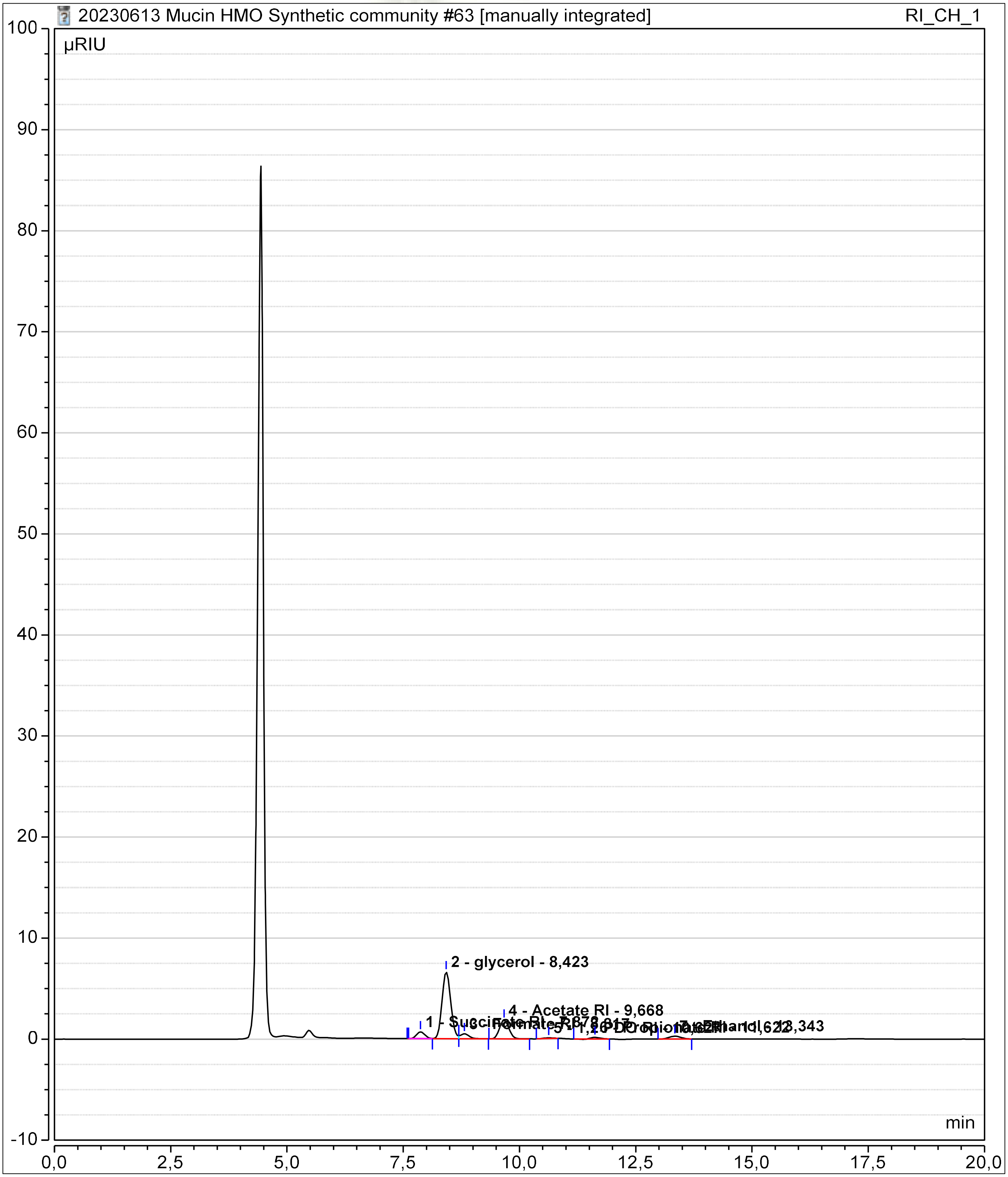

Chromatogram and Results

Injection Details

Injection Name:	**36 MUCHMO3 t24 r3**	*Run Time (min):*	**20,00**
Vial Number:	**3:E9**	*Injection Volume:*	**20,00**
Injection Type:	**Unknown**	*Channel:*	**RI_CH_1**
Calibration Level:		*Wavelength:*	**n.a.**
Instrument Method:	**Default method LC2030C 45 gr 20 min**	*Bandwidth:*	**n.a.**
Processing Method:	**Processing Method LC2030 45 gr**	*Dilution Factor:*	**1,0000**
Injection Date/Time:	**14-jun-23 09:24**	*Sample Weight:*	**1,0000**

Chromatogram

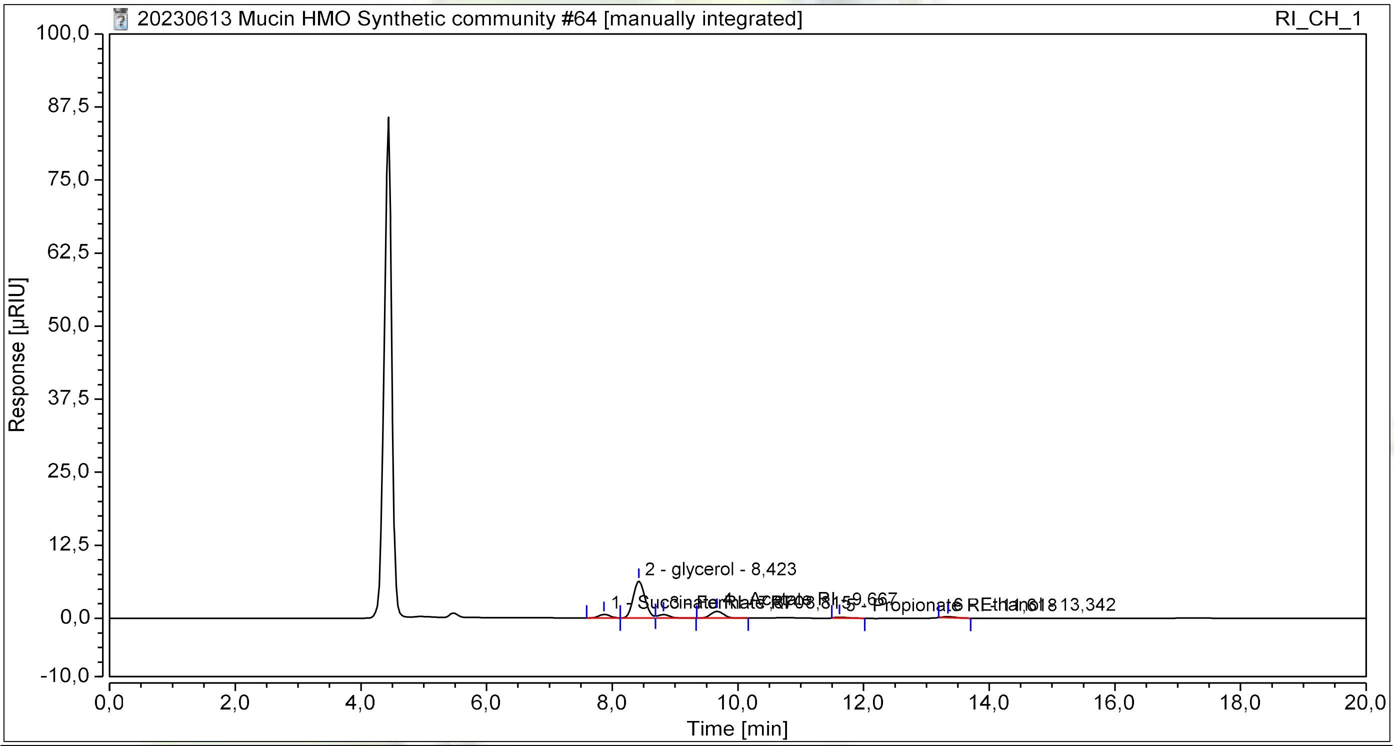

Integration Results

No.	Peak Name	Retention Time min	Area µRIU*min	Height µRIU	Relative Area %	Relative Height %	Amount
n.a.	GlcNAc	n.a.	n.a.	n.a.	n.a.	n.a.	n.a.
n.a.	Citrate	n.a.	n.a.	n.a.	n.a.	n.a.	n.a.
n.a.	Glucose	n.a.	n.a.	n.a.	n.a.	n.a.	n.a.
n.a.	Galactose	n.a.	n.a.	n.a.	n.a.	n.a.	n.a.
n.a.	Fucose	n.a.	n.a.	n.a.	n.a.	n.a.	n.a.
1	Succinate RI	7,870	0,133	0,623	6,69	6,92	n.a.
n.a.	Lactate RI	n.a.	n.a.	n.a.	n.a.	n.a.	n.a.
2	glycerol	8,423	1,377	6,327	69,57	70,35	n.a.
3	Formate RI	8,815	0,129	0,589	6,50	6,55	13,5032
4	Acetate RI	9,667	0,277	1,166	14,00	12,97	17,0629
n.a.	1,2 PDO RI	n.a.	n.a.	n.a.	n.a.	n.a.	n.a.
n.a.	1,3-PDO	n.a.	n.a.	n.a.	n.a.	n.a.	n.a.
5	Propionate RI	11,618	0,026	0,128	1,34	1,42	1,0658
n.a.	1,3-PDO	n.a.	n.a.	n.a.	n.a.	n.a.	n.a.
n.a.	2-3 BDO	n.a.	n.a.	n.a.	n.a.	n.a.	n.a.
6	Ethanol	13,342	0,038	0,161	1,90	1,79	0,3464
n.a.	Isobutyrate RI	n.a.	n.a.	n.a.	n.a.	n.a.	n.a.
n.a.	Butyrate RI	n.a.	n.a.	n.a.	n.a.	n.a.	n.a.
**Total:**			**1,980**	**8,994**	**100,00**	**100,00**	

Peak Analysis

Injection Details

Injection Name:	**36 MUCHMO3 t24 r3**	*Run Time (min):*	**20,00**
Vial Number:	**3:E9**	*Injection Volume:*	**20,00**
Injection Type:	**Unknown**	*Channel:*	**RI_CH_1**
Calibration Level:		*Wavelength:*	**n.a.**
Instrument Method:	**Default method LC2030C 45 gr 20 min**	*Bandwidth:*	**n.a.**
Processing Method:	**Processing Method LC2030 45 gr**	*Dilution Factor:*	**1,0000**
Injection Date/Time:	**14-jun-23 09:24**	*Sample Weight:*	**1,0000**

Chromatogram

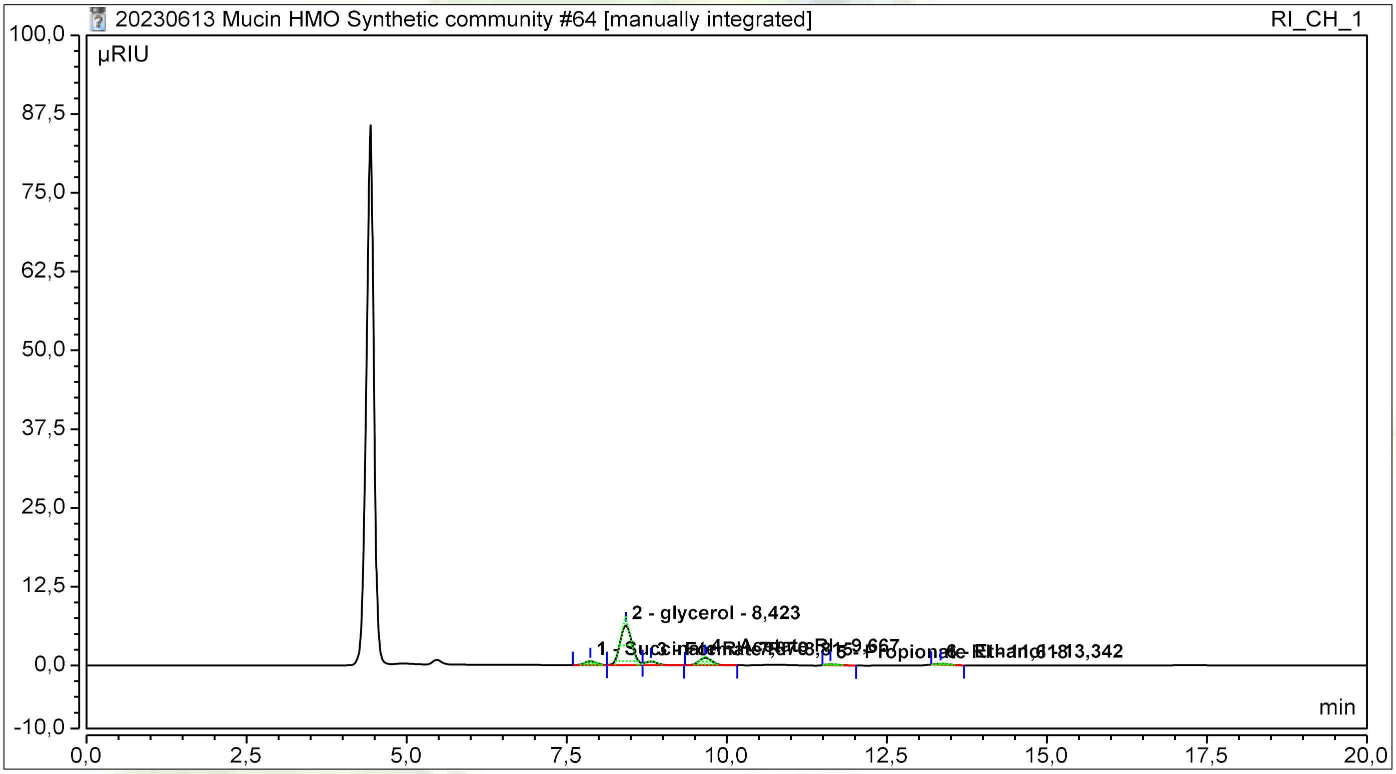

Peak Results

No.	Peak Name	Retention Time min	Width (50%) min	Type	Resolution (EP)	Asymmetry (EP)	Plates (EP)
n.a.	GlcNAc	n.a.	n.a.	n.a.	n.a.	n.a.	n.a.
n.a.	Citrate	n.a.	n.a.	n.a.	n.a.	n.a.	n.a.
n.a.	Glucose	n.a.	n.a.	n.a.	n.a.	n.a.	n.a.
n.a.	Galactose	n.a.	n.a.	n.a.	n.a.	n.a.	n.a.
n.a.	Fucose	n.a.	n.a.	n.a.	n.a.	n.a.	n.a.
1	Succinate RI	7,870	0,202	BM	1,61	1,10	8443
n.a.	Lactate RI	n.a.	n.a.	n.a.	n.a.	n.a.	n.a.
2	glycerol	8,423	0,205	M	n.a.	1,15	9396
3	Formate RI	8,815	n.a.	MB	n.a.	n.a.	n.a.
4	Acetate RI	9,667	0,225	BMB	5,42	1,09	10239
n.a.	1,2 PDO RI	n.a.	n.a.	n.a.	n.a.	n.a.	n.a.
n.a.	1,3-PDO	n.a.	n.a.	n.a.	n.a.	n.a.	n.a.
5	Propionate RI	11,618	0,200	BMB*	4,67	1,56	18696
n.a.	1,3-PDO	n.a.	n.a.	n.a.	n.a.	n.a.	n.a.
n.a.	2-3 BDO	n.a.	n.a.	n.a.	n.a.	n.a.	n.a.
6	Ethanol	13,342	0,235	BMB*	n.a.	1,37	17806
n.a.	Isobutyrate RI	n.a.	n.a.	n.a.	n.a.	n.a.	n.a.
n.a.	Butyrate RI	n.a.	n.a.	n.a.	n.a.	n.a.	n.a.

Chromeleon (c) Dionex
Version 7.2.10.25868

Chromatogram and SST Results

Injection Details

Injection Name:	**36 MUCHMO3 t24 r3**	*Run Time (min):*	**20,00**
Vial Number:	**3:E9**	*Injection Volume:*	**20,00**
Injection Type:	**Unknown**	*Channel:*	**RI_CH_1**
Calibration Level:		*Wavelength:*	**n.a.**
Instrument Method:	**Default method LC2030C 45 gr 20 min**	*Bandwidth:*	**n.a.**
Processing Method:	**Processing Method LC2030 45 gr**	*Dilution Factor:*	**1,0000**
Injection Date/Time:	**14-jun-23 09:24**	*Sample Weight:*	**1,0000**

Chromatogram

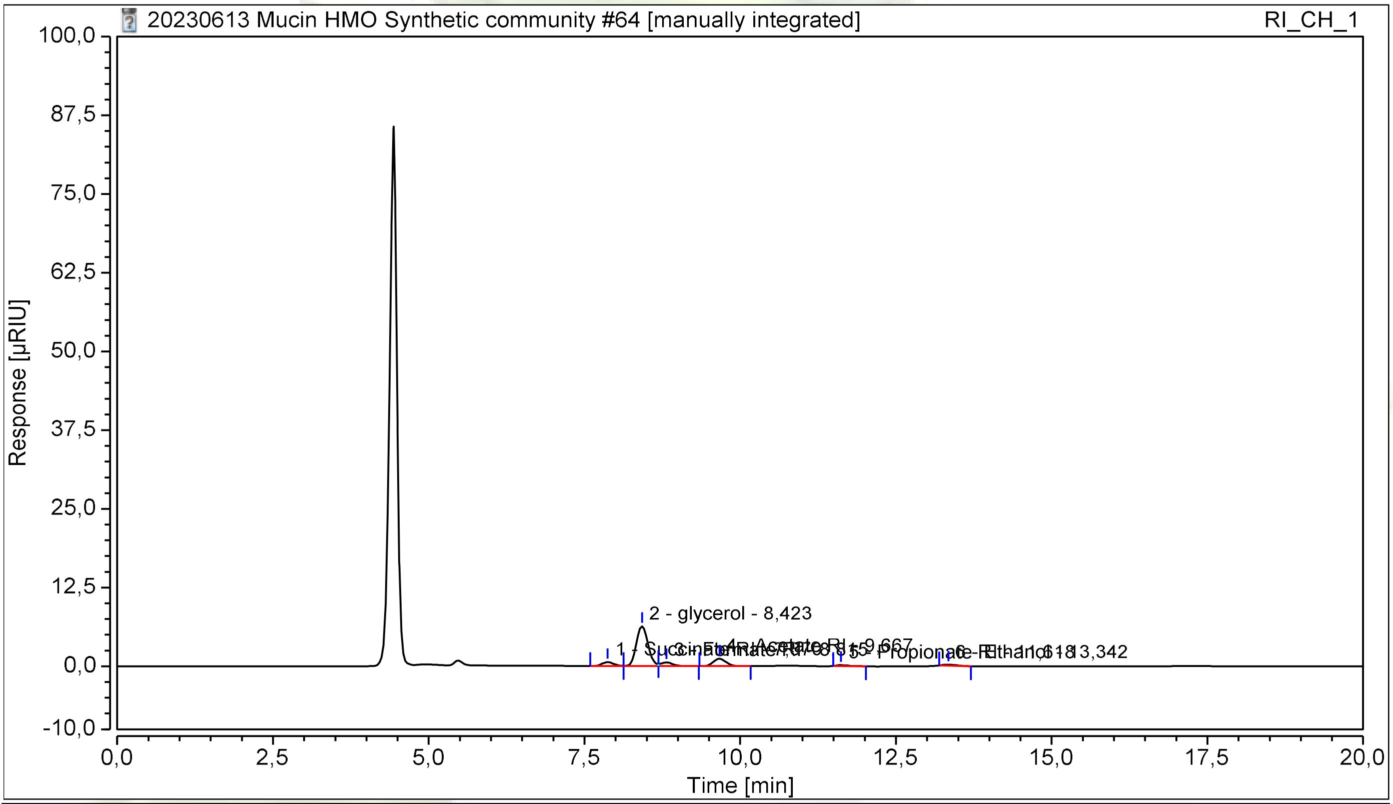

SST Results

No.	Name	Inj.Condition	Peak	Test Result	Injection
**Number of executed test cases:**	**n.a.**		**Total Result:**	**Passed**	

Chromatogram

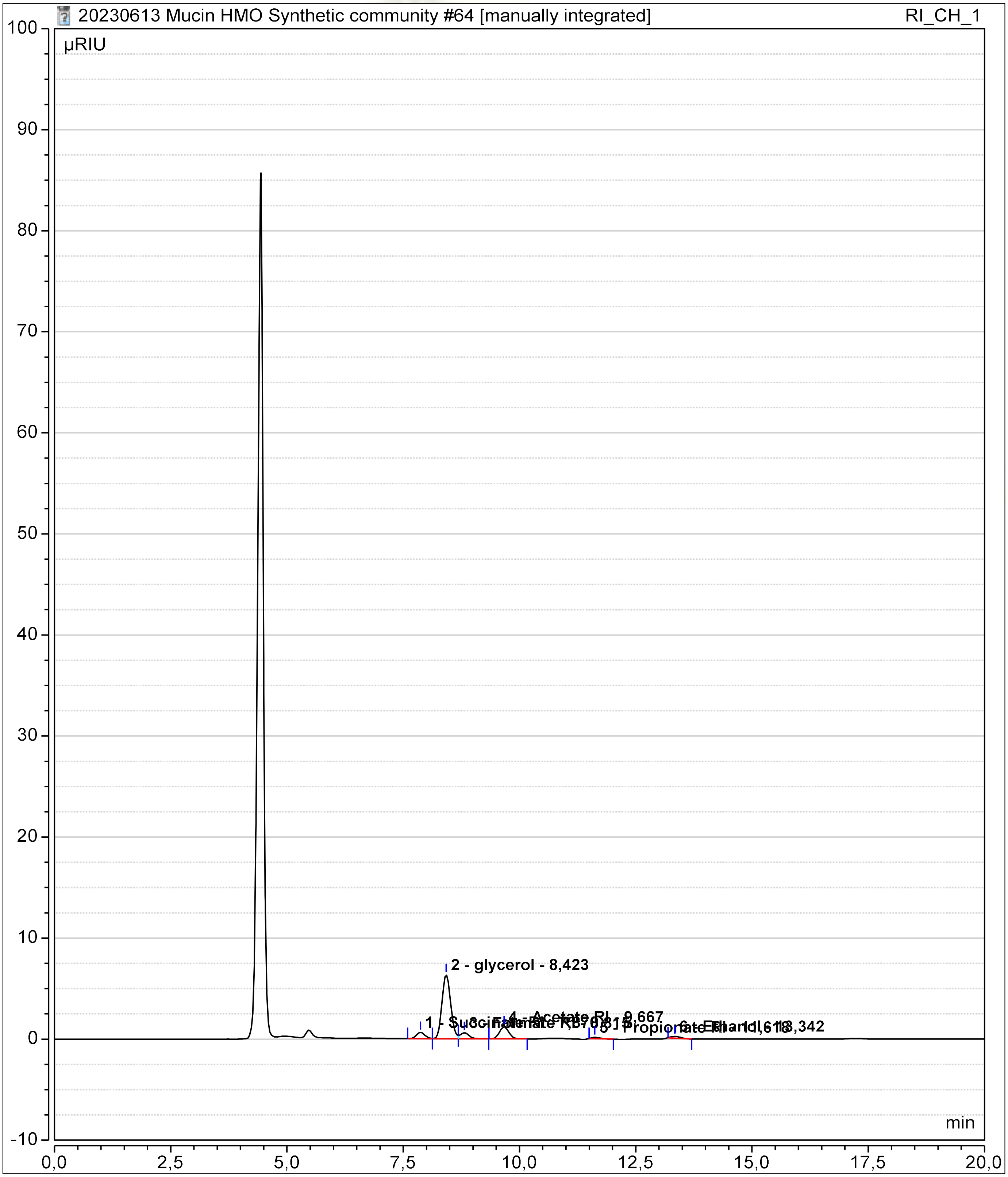

Chromatogram and Results

Injection Details

Injection Name:	**37 MUCHMO3 t48 r1**	*Run Time (min):*	**20,00**
Vial Number:	**3:E10**	*Injection Volume:*	**20,00**
Injection Type:	**Unknown**	*Channel:*	**RI_CH_1**
Calibration Level:		*Wavelength:*	**n.a.**
Instrument Method:	**Default method LC2030C 45 gr 20 min**	*Bandwidth:*	**n.a.**
Processing Method:	**Processing Method LC2030 45 gr**	*Dilution Factor:*	**1,0000**
Injection Date/Time:	**14-jun-23 09:44**	*Sample Weight:*	**1,0000**

Chromatogram

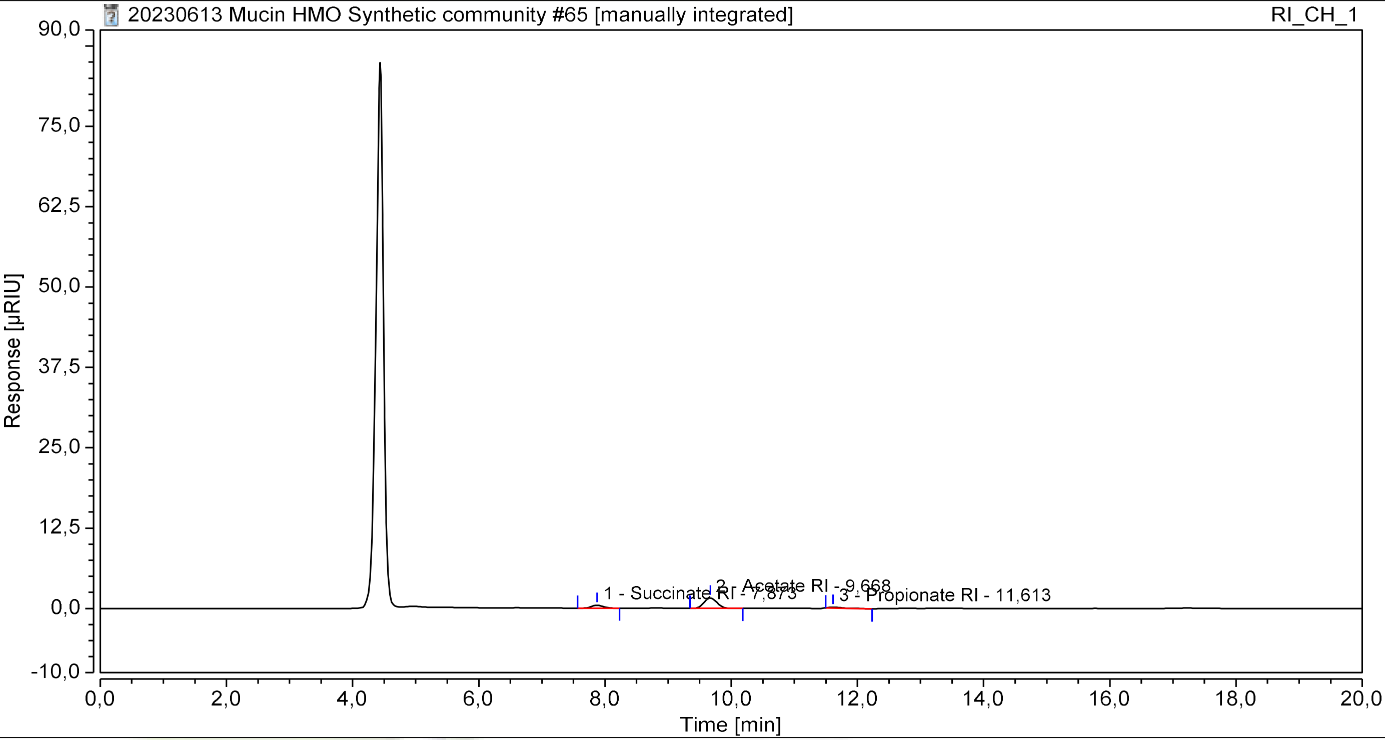

Integration Results

No.	Peak Name	Retention Time<br>min	Area<br>µRIU*min	Height<br>µRIU	Relative Area<br>%	Relative Height<br>%	Amount
n.a.	GlcNAc	n.a.	n.a.	n.a.	n.a.	n.a.	n.a.
n.a.	Citrate	n.a.	n.a.	n.a.	n.a.	n.a.	n.a.
n.a.	Glucose	n.a.	n.a.	n.a.	n.a.	n.a.	n.a.
n.a.	Galactose	n.a.	n.a.	n.a.	n.a.	n.a.	n.a.
n.a.	Fucose	n.a.	n.a.	n.a.	n.a.	n.a.	n.a.
1	Succinate RI	7,873	0,093	0,448	18,27	20,43	n.a.
n.a.	Lactate RI	n.a.	n.a.	n.a.	n.a.	n.a.	n.a.
n.a.	glycerol	n.a.	n.a.	n.a.	n.a.	n.a.	n.a.
n.a.	Formate RI	n.a.	n.a.	n.a.	n.a.	n.a.	n.a.
2	Acetate RI	9,668	0,382	1,605	75,15	73,21	23,5189
n.a.	1,2 PDO RI	n.a.	n.a.	n.a.	n.a.	n.a.	n.a.
n.a.	1,3-PDO	n.a.	n.a.	n.a.	n.a.	n.a.	n.a.
3	Propionate RI	11,613	0,033	0,139	6,58	6,36	1,3470
n.a.	1,3-PDO	n.a.	n.a.	n.a.	n.a.	n.a.	n.a.
n.a.	2-3 BDO	n.a.	n.a.	n.a.	n.a.	n.a.	n.a.
n.a.	Ethanol	n.a.	n.a.	n.a.	n.a.	n.a.	n.a.
n.a.	Isobutyrate RI	n.a.	n.a.	n.a.	n.a.	n.a.	n.a.
n.a.	Butyrate RI	n.a.	n.a.	n.a.	n.a.	n.a.	n.a.
**Total:**			**0,509**	**2,192**	**100,00**	**100,00**	

Peak Analysis

Injection Details

Injection Name:	**37 MUCHMO3 t48 r1**	*Run Time (min):*	**20,00**
Vial Number:	**3:E10**	*Injection Volume:*	**20,00**
Injection Type:	**Unknown**	*Channel:*	**RI_CH_1**
Calibration Level:		*Wavelength:*	**n.a.**
Instrument Method:	**Default method LC2030C 45 gr 20 min**	*Bandwidth:*	**n.a.**
Processing Method:	**Processing Method LC2030 45 gr**	*Dilution Factor:*	**1,0000**
Injection Date/Time:	**14-jun-23 09:44**	*Sample Weight:*	**1,0000**

Chromatogram

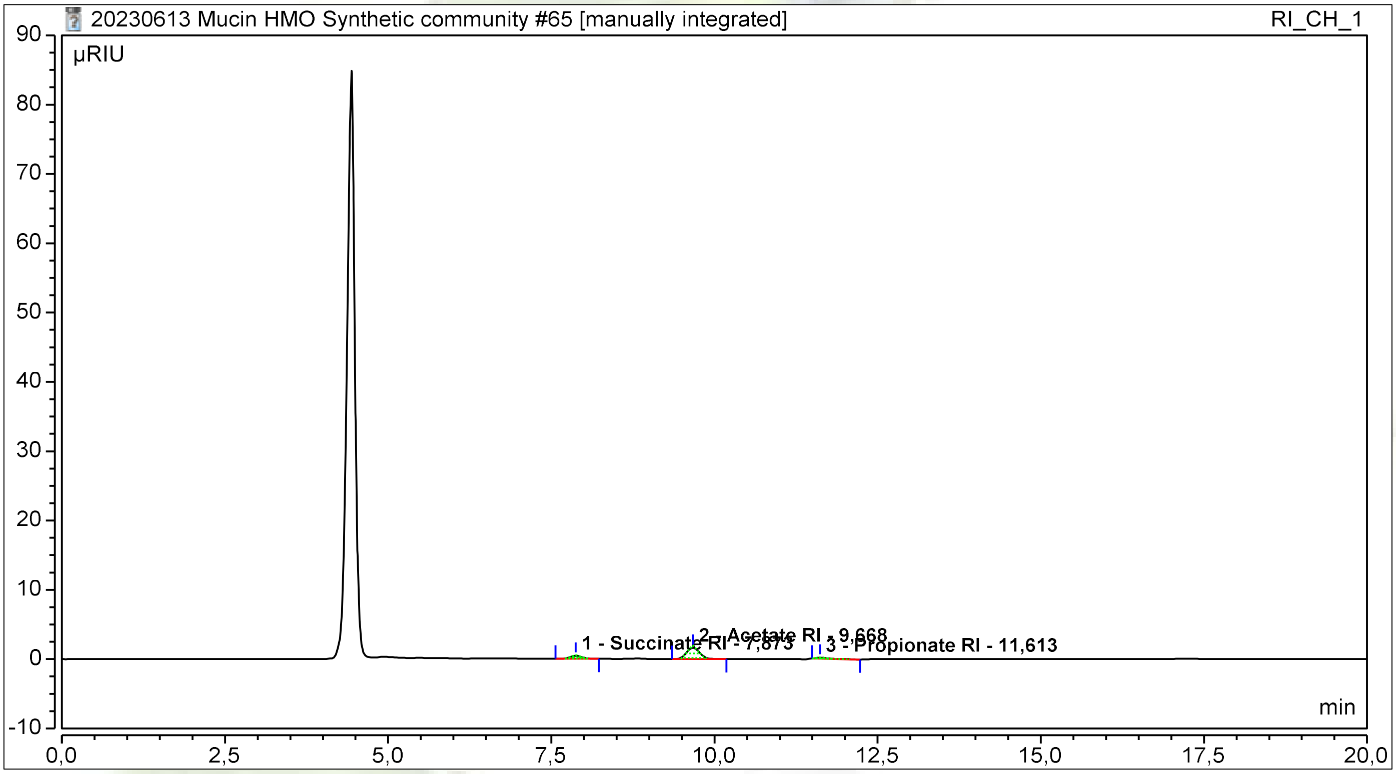

Peak Results

No.	Peak Name	Retention Time min	Width (50%) min	Type	Resolution (EP)	Asymmetry (EP)	Plates (EP)
n.a.	GlcNAc	n.a.	n.a.	n.a.	n.a.	n.a.	n.a.
n.a.	Citrate	n.a.	n.a.	n.a.	n.a.	n.a.	n.a.
n.a.	Glucose	n.a.	n.a.	n.a.	n.a.	n.a.	n.a.
n.a.	Galactose	n.a.	n.a.	n.a.	n.a.	n.a.	n.a.
n.a.	Fucose	n.a.	n.a.	n.a.	n.a.	n.a.	n.a.
1	Succinate RI	7,873	0,199	BMB*	4,99	1,05	8632
n.a.	Lactate RI	n.a.	n.a.	n.a.	n.a.	n.a.	n.a.
n.a.	glycerol	n.a.	n.a.	n.a.	n.a.	n.a.	n.a.
n.a.	Formate RI	n.a.	n.a.	n.a.	n.a.	n.a.	n.a.
2	Acetate RI	9,668	0,225	BMB	5,35	1,08	10198
n.a.	1,2 PDO RI	n.a.	n.a.	n.a.	n.a.	n.a.	n.a.
n.a.	1,3-PDO	n.a.	n.a.	n.a.	n.a.	n.a.	n.a.
3	Propionate RI	11,613	0,204	BMB*	n.a.	2,63	17963
n.a.	1,3-PDO	n.a.	n.a.	n.a.	n.a.	n.a.	n.a.
n.a.	2-3 BDO	n.a.	n.a.	n.a.	n.a.	n.a.	n.a.
n.a.	Ethanol	n.a.	n.a.	n.a.	n.a.	n.a.	n.a.
n.a.	Isobutyrate RI	n.a.	n.a.	n.a.	n.a.	n.a.	n.a.
n.a.	Butyrate RI	n.a.	n.a.	n.a.	n.a.	n.a.	n.a.

Chromeleon (c) Dionex
Version 7.2.10.25868

Chromatogram and SST Results

Injection Details

Injection Name:	**37 MUCHMO3 t48 r1**	*Run Time (min):*	**20,00**
Vial Number:	**3:E10**	*Injection Volume:*	**20,00**
Injection Type:	**Unknown**	*Channel:*	**RI_CH_1**
Calibration Level:		*Wavelength:*	**n.a.**
Instrument Method:	**Default method LC2030C 45 gr 20 min**	*Bandwidth:*	**n.a.**
Processing Method:	**Processing Method LC2030 45 gr**	*Dilution Factor:*	**1,0000**
Injection Date/Time:	**14-jun-23 09:44**	*Sample Weight:*	**1,0000**

Chromatogram

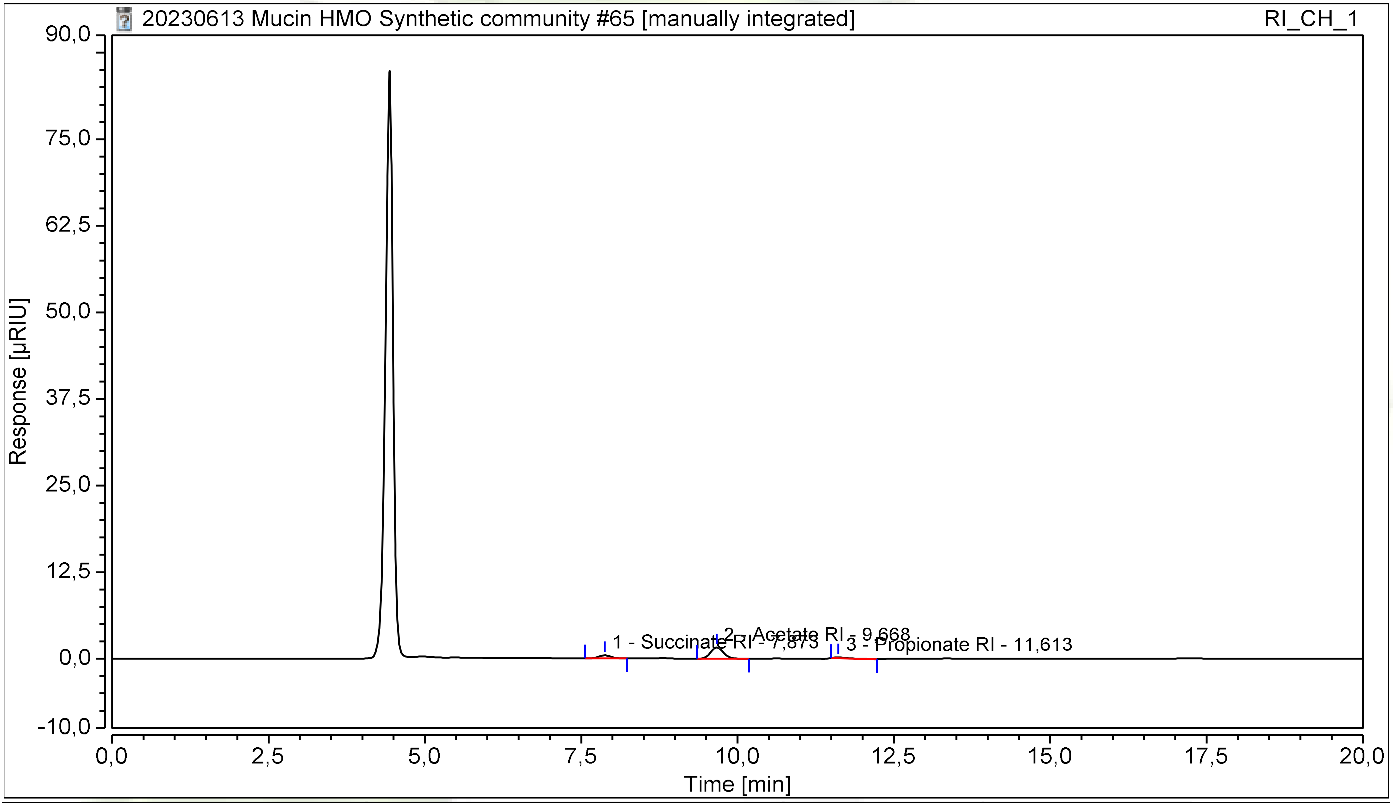

SST Results

No.	Name	Inj.Condition	Peak	Test Result	Injection

Number of executed test cases: **n.a.** **Total Result:** **Passed**

Chromeleon (c) Dionex
Version 7.2.10.25868

Chromatogram

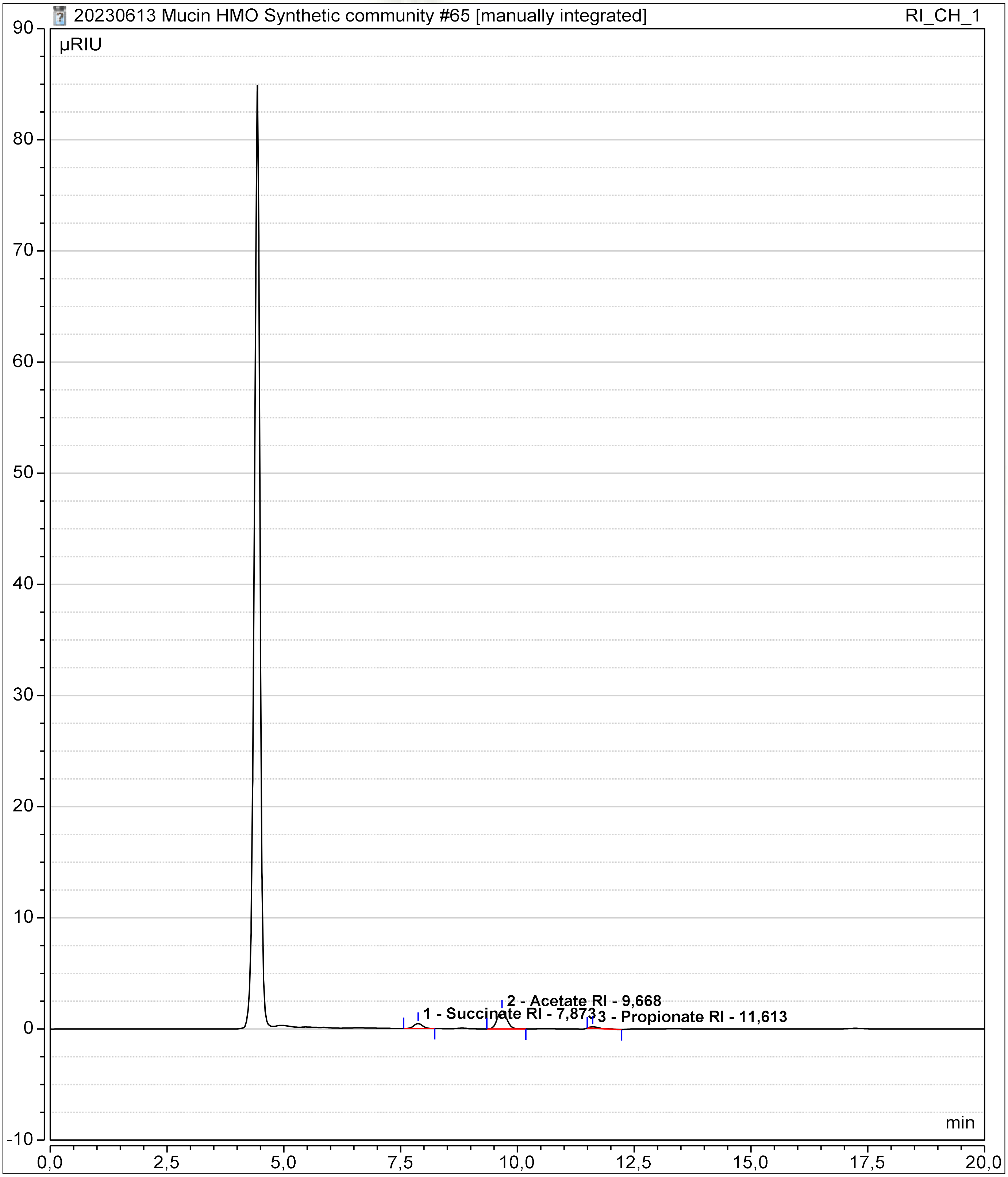

Chromeleon (c) Dionex
Version 7.2.10.25868

Chromatogram and Results

Injection Details

Injection Name:	**38 MUCHMO3 t48 r2**	*Run Time (min):*	**20,00**
Vial Number:	**3:E11**	*Injection Volume:*	**20,00**
Injection Type:	**Unknown**	*Channel:*	**RI_CH_1**
Calibration Level:		*Wavelength:*	**n.a.**
Instrument Method:	**Default method LC2030C 45 gr 20 min**	*Bandwidth:*	**n.a.**
Processing Method:	**Processing Method LC2030 45 gr**	*Dilution Factor:*	**1,0000**
Injection Date/Time:	**14-jun-23 10:05**	*Sample Weight:*	**1,0000**

Chromatogram

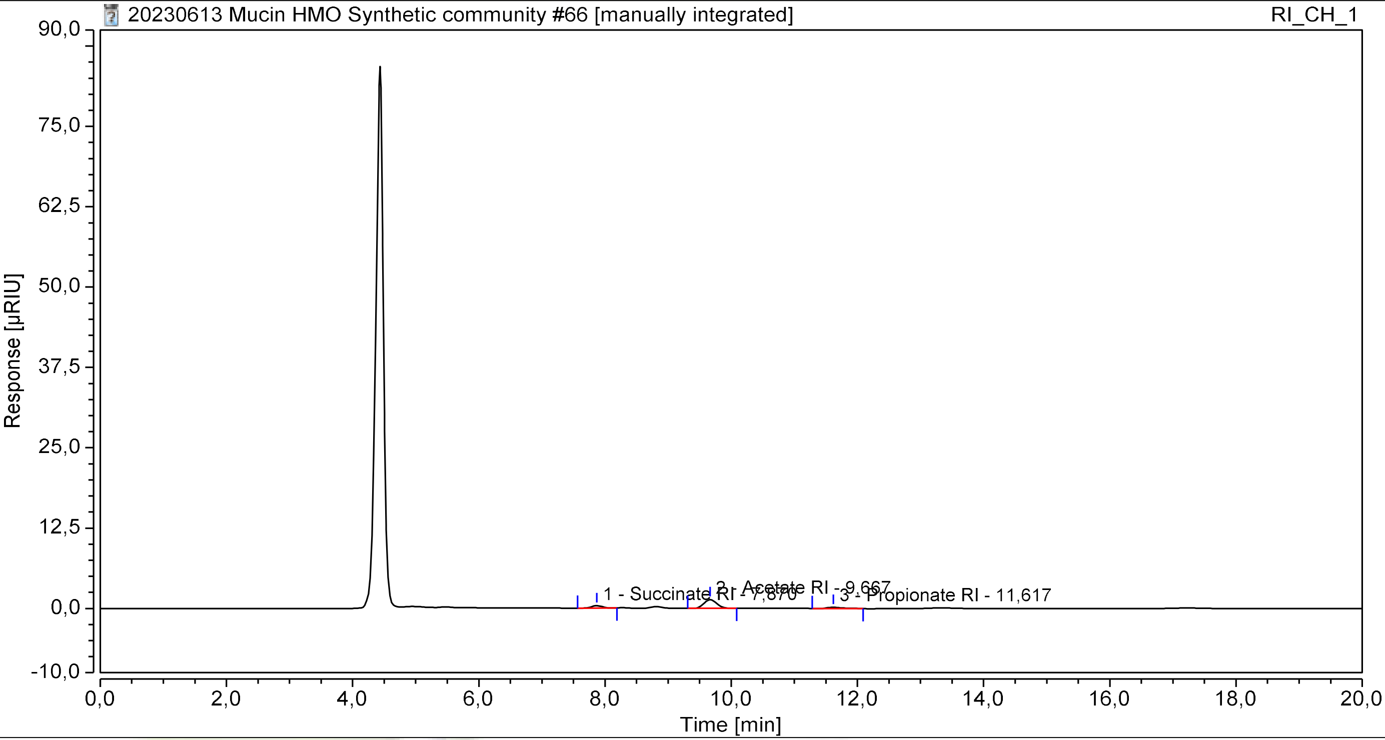

Integration Results

No.	Peak Name	Retention Time min	Area µRIU*min	Height µRIU	Relative Area %	Relative Height %	Amount
n.a.	GlcNAc	n.a.	n.a.	n.a.	n.a.	n.a.	n.a.
n.a.	Citrate	n.a.	n.a.	n.a.	n.a.	n.a.	n.a.
n.a.	Glucose	n.a.	n.a.	n.a.	n.a.	n.a.	n.a.
n.a.	Galactose	n.a.	n.a.	n.a.	n.a.	n.a.	n.a.
n.a.	Fucose	n.a.	n.a.	n.a.	n.a.	n.a.	n.a.
1	Succinate RI	7,870	0,067	0,352	15,11	18,52	n.a.
n.a.	Lactate RI	n.a.	n.a.	n.a.	n.a.	n.a.	n.a.
n.a.	glycerol	n.a.	n.a.	n.a.	n.a.	n.a.	n.a.
n.a.	Formate RI	n.a.	n.a.	n.a.	n.a.	n.a.	n.a.
2	Acetate RI	9,667	0,323	1,358	72,87	71,34	19,8781
n.a.	1,2 PDO RI	n.a.	n.a.	n.a.	n.a.	n.a.	n.a.
n.a.	1,3-PDO	n.a.	n.a.	n.a.	n.a.	n.a.	n.a.
3	Propionate RI	11,617	0,053	0,193	12,02	10,14	2,1427
n.a.	1,3-PDO	n.a.	n.a.	n.a.	n.a.	n.a.	n.a.
n.a.	2-3 BDO	n.a.	n.a.	n.a.	n.a.	n.a.	n.a.
n.a.	Ethanol	n.a.	n.a.	n.a.	n.a.	n.a.	n.a.
n.a.	Isobutyrate RI	n.a.	n.a.	n.a.	n.a.	n.a.	n.a.
n.a.	Butyrate RI	n.a.	n.a.	n.a.	n.a.	n.a.	n.a.
**Total:**			**0,443**	**1,904**	**100,00**	**100,00**	

Peak Analysis

Injection Details

Injection Name:	**38 MUCHMO3 t48 r2**	*Run Time (min):*	**20,00**
Vial Number:	**3:E11**	*Injection Volume:*	**20,00**
Injection Type:	**Unknown**	*Channel:*	**RI_CH_1**
Calibration Level:		*Wavelength:*	**n.a.**
Instrument Method:	**Default method LC2030C 45 gr 20 min**	*Bandwidth:*	**n.a.**
Processing Method:	**Processing Method LC2030 45 gr**	*Dilution Factor:*	**1,0000**
Injection Date/Time:	**14-jun-23 10:05**	*Sample Weight:*	**1,0000**

Chromatogram

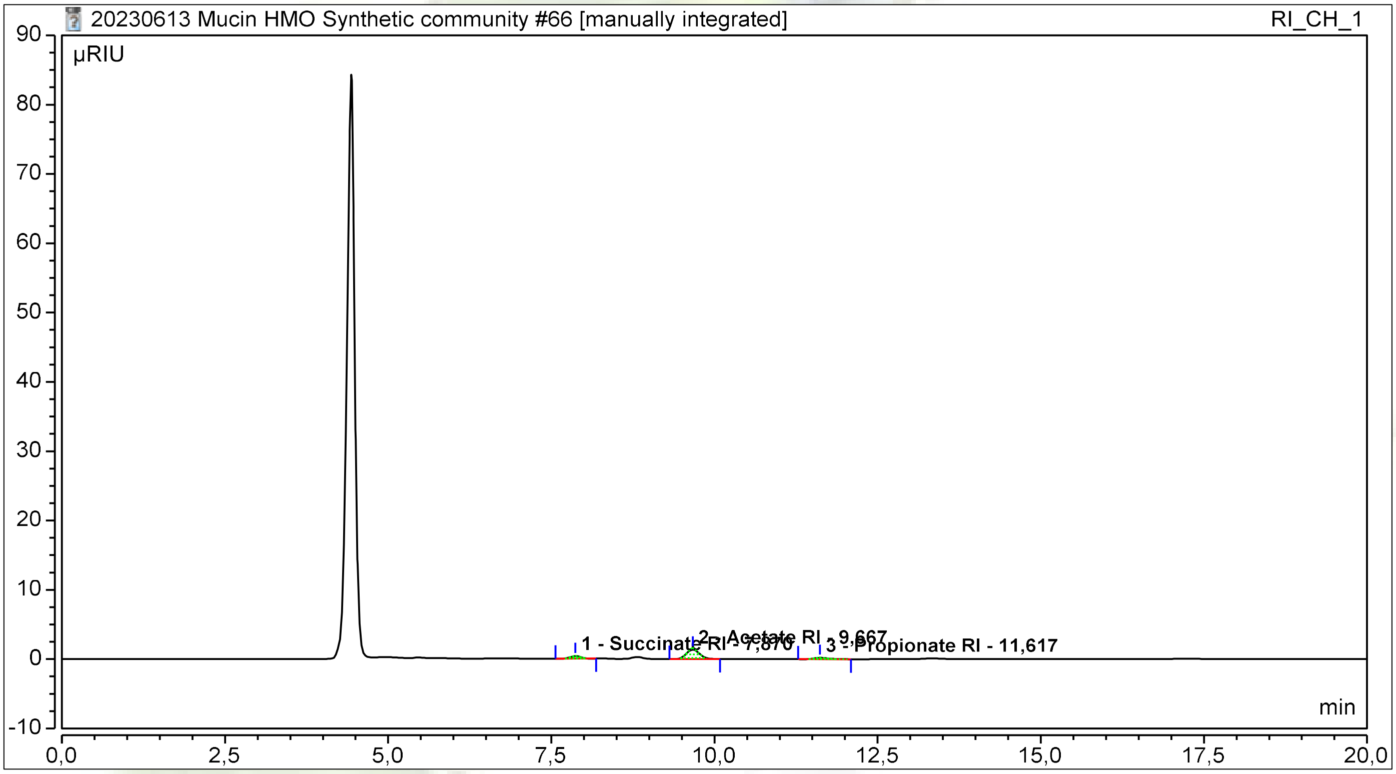

Peak Results

No.	Peak Name	Retention Time min	Width (50%) min	Type	Resolution (EP)	Asymmetry (EP)	Plates (EP)
n.a.	GlcNAc	n.a.	n.a.	n.a.	n.a.	n.a.	n.a.
n.a.	Citrate	n.a.	n.a.	n.a.	n.a.	n.a.	n.a.
n.a.	Glucose	n.a.	n.a.	n.a.	n.a.	n.a.	n.a.
n.a.	Galactose	n.a.	n.a.	n.a.	n.a.	n.a.	n.a.
n.a.	Fucose	n.a.	n.a.	n.a.	n.a.	n.a.	n.a.
1	Succinate RI	7,870	0,192	BMB*	5,08	1,00	9317
n.a.	Lactate RI	n.a.	n.a.	n.a.	n.a.	n.a.	n.a.
n.a.	glycerol	n.a.	n.a.	n.a.	n.a.	n.a.	n.a.
n.a.	Formate RI	n.a.	n.a.	n.a.	n.a.	n.a.	n.a.
2	Acetate RI	9,667	0,225	BMB	4,85	1,08	10217
n.a.	1,2 PDO RI	n.a.	n.a.	n.a.	n.a.	n.a.	n.a.
n.a.	1,3-PDO	n.a.	n.a.	n.a.	n.a.	n.a.	n.a.
3	Propionate RI	11,617	0,249	BMB*	n.a.	1,79	12036
n.a.	1,3-PDO	n.a.	n.a.	n.a.	n.a.	n.a.	n.a.
n.a.	2-3 BDO	n.a.	n.a.	n.a.	n.a.	n.a.	n.a.
n.a.	Ethanol	n.a.	n.a.	n.a.	n.a.	n.a.	n.a.
n.a.	Isobutyrate RI	n.a.	n.a.	n.a.	n.a.	n.a.	n.a.
n.a.	Butyrate RI	n.a.	n.a.	n.a.	n.a.	n.a.	n.a.

Chromatogram and SST Results

Injection Details

Injection Name:	**38 MUCHMO3 t48 r2**	*Run Time (min):*	**20,00**
Vial Number:	**3:E11**	*Injection Volume:*	**20,00**
Injection Type:	**Unknown**	*Channel:*	**RI_CH_1**
Calibration Level:		*Wavelength:*	**n.a.**
Instrument Method:	**Default method LC2030C 45 gr 20 min**	*Bandwidth:*	**n.a.**
Processing Method:	**Processing Method LC2030 45 gr**	*Dilution Factor:*	**1,0000**
Injection Date/Time:	**14-jun-23 10:05**	*Sample Weight:*	**1,0000**

Chromatogram

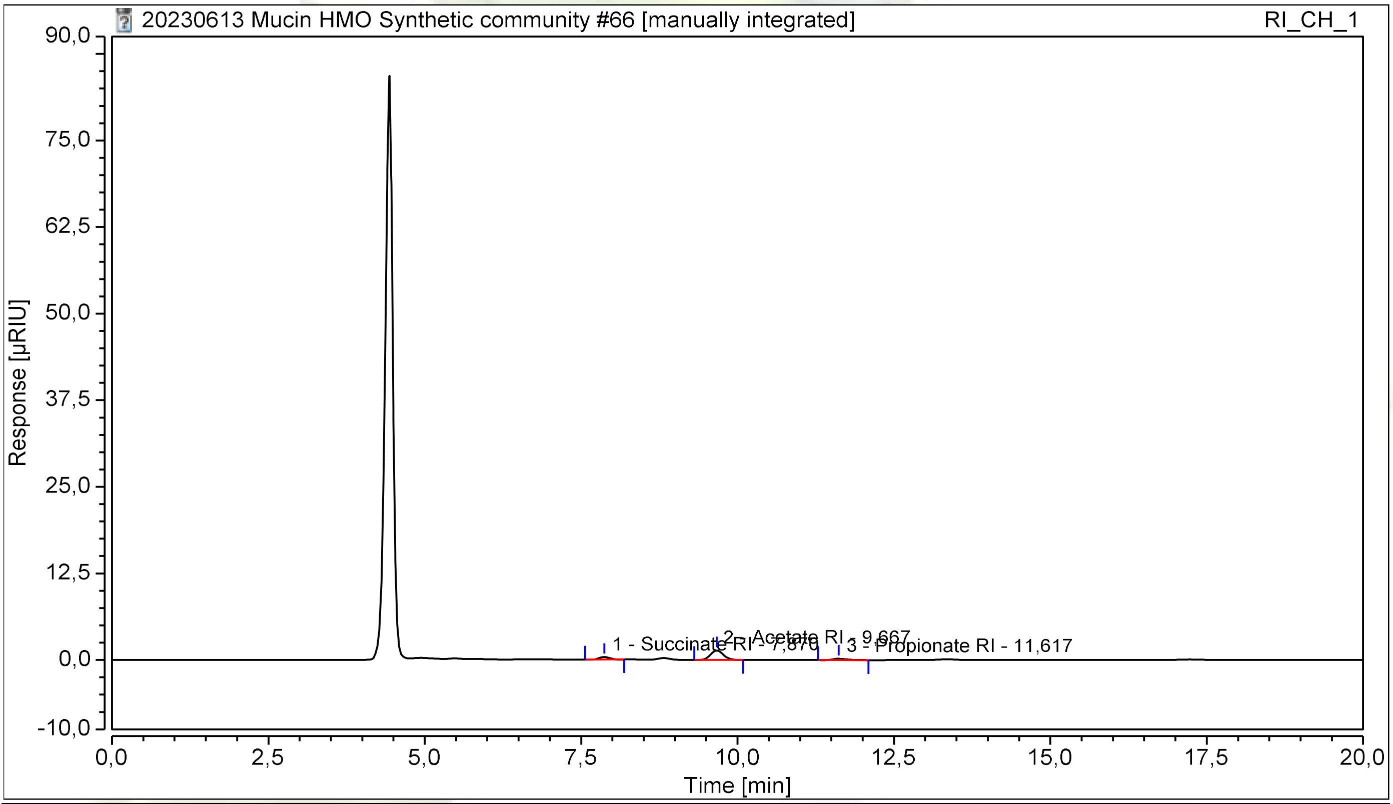

SST Results

No.	Name	Inj.Condition	Peak	Test Result	Injection

Number of executed test cases: **n.a.** **Total Result:** **Passed**

Chromatogram

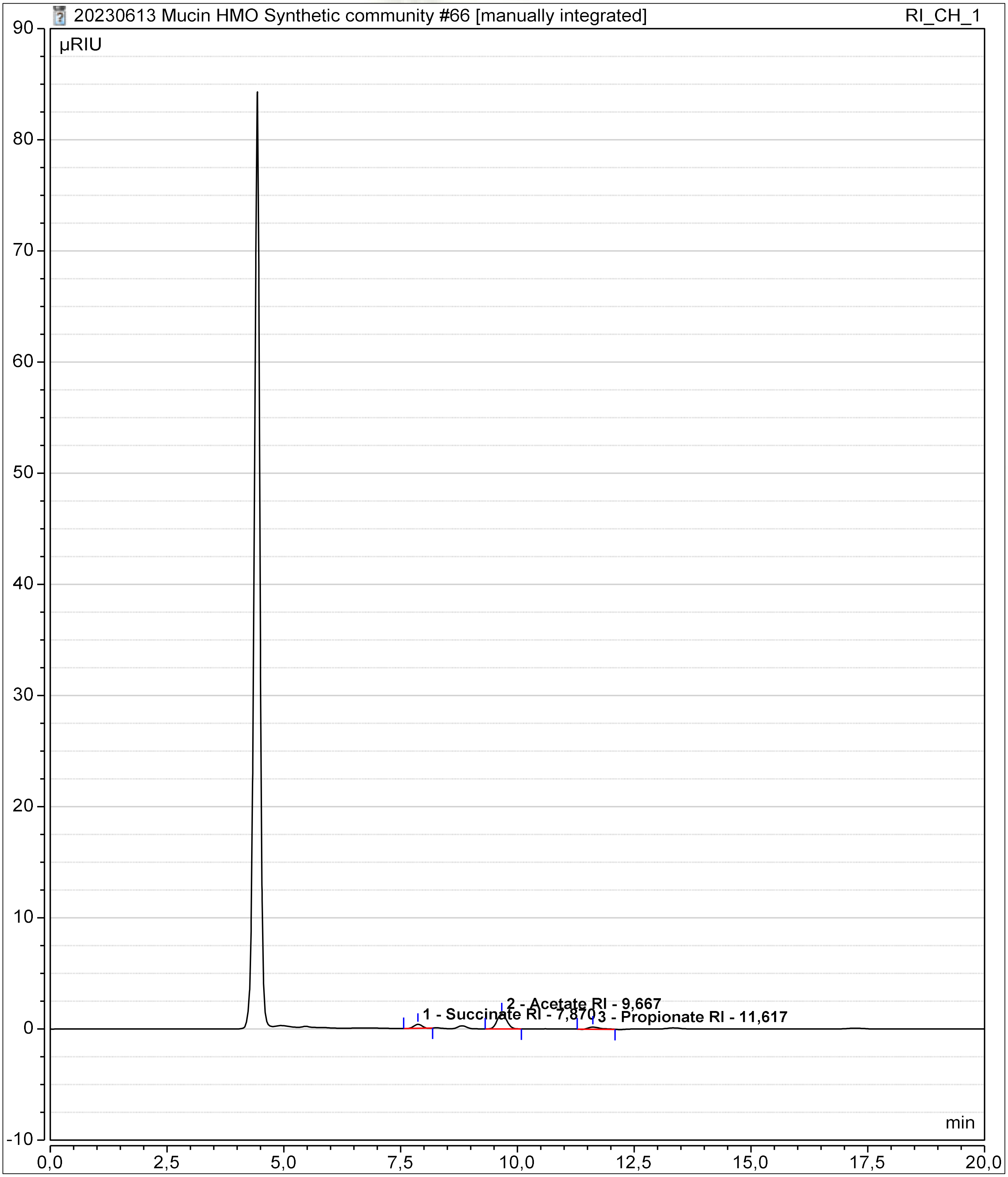

Chromatogram and Results

Injection Details

Injection Name:	**39 MUCHMO3 t48 r3**	*Run Time (min):*	**20,00**
Vial Number:	**3:E12**	*Injection Volume:*	**20,00**
Injection Type:	**Unknown**	*Channel:*	**RI_CH_1**
Calibration Level:		*Wavelength:*	**n.a.**
Instrument Method:	**Default method LC2030C 45 gr 20 min**	*Bandwidth:*	**n.a.**
Processing Method:	**Processing Method LC2030 45 gr**	*Dilution Factor:*	**1,0000**
Injection Date/Time:	**14-jun-23 10:25**	*Sample Weight:*	**1,0000**

Chromatogram

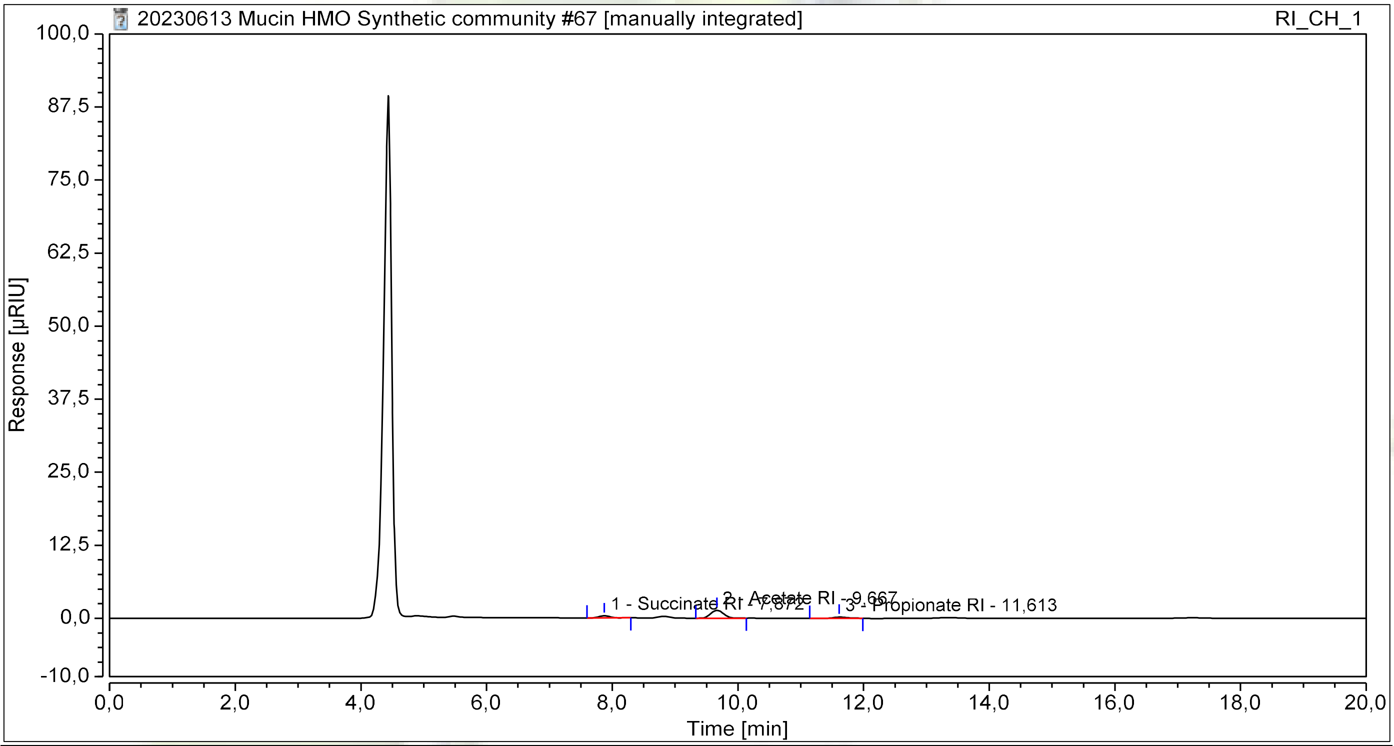

Integration Results

No.	Peak Name	Retention Time min	Area µRIU*min	Height µRIU	Relative Area %	Relative Height %	Amount
n.a.	GlcNAc	n.a.	n.a.	n.a.	n.a.	n.a.	n.a.
n.a.	Citrate	n.a.	n.a.	n.a.	n.a.	n.a.	n.a.
n.a.	Glucose	n.a.	n.a.	n.a.	n.a.	n.a.	n.a.
n.a.	Galactose	n.a.	n.a.	n.a.	n.a.	n.a.	n.a.
n.a.	Fucose	n.a.	n.a.	n.a.	n.a.	n.a.	n.a.
1	Succinate RI	7,872	0,065	0,362	15,42	19,19	n.a.
n.a.	Lactate RI	n.a.	n.a.	n.a.	n.a.	n.a.	n.a.
n.a.	glycerol	n.a.	n.a.	n.a.	n.a.	n.a.	n.a.
n.a.	Formate RI	n.a.	n.a.	n.a.	n.a.	n.a.	n.a.
2	Acetate RI	9,667	0,316	1,340	75,04	71,10	19,4182
n.a.	1,2 PDO RI	n.a.	n.a.	n.a.	n.a.	n.a.	n.a.
n.a.	1,3-PDO	n.a.	n.a.	n.a.	n.a.	n.a.	n.a.
3	Propionate RI	11,613	0,040	0,183	9,54	9,71	1,6141
n.a.	1,3-PDO	n.a.	n.a.	n.a.	n.a.	n.a.	n.a.
n.a.	2-3 BDO	n.a.	n.a.	n.a.	n.a.	n.a.	n.a.
n.a.	Ethanol	n.a.	n.a.	n.a.	n.a.	n.a.	n.a.
n.a.	Isobutyrate RI	n.a.	n.a.	n.a.	n.a.	n.a.	n.a.
n.a.	Butyrate RI	n.a.	n.a.	n.a.	n.a.	n.a.	n.a.
**Total:**			**0,420**	**1,884**	**100,00**	**100,00**	

Peak Analysis

Injection Details

Injection Name:	**39 MUCHMO3 t48 r3**	*Run Time (min):*	**20,00**
Vial Number:	**3:E12**	*Injection Volume:*	**20,00**
Injection Type:	**Unknown**	*Channel:*	**RI_CH_1**
Calibration Level:		*Wavelength:*	**n.a.**
Instrument Method:	**Default method LC2030C 45 gr 20 min**	*Bandwidth:*	**n.a.**
Processing Method:	**Processing Method LC2030 45 gr**	*Dilution Factor:*	**1,0000**
Injection Date/Time:	**14-jun-23 10:25**	*Sample Weight:*	**1,0000**

Chromatogram

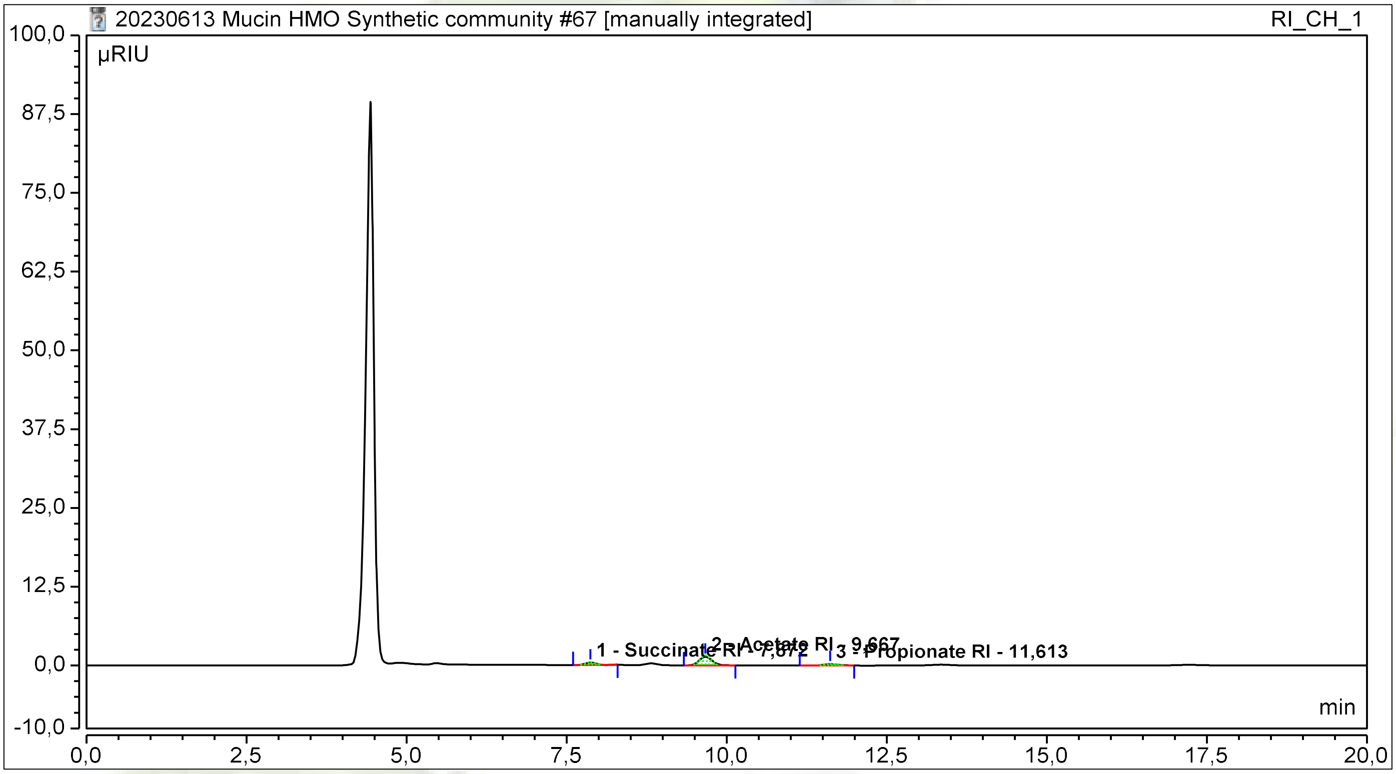

Peak Results

No.	Peak Name	Retention Time min	Width (50%) min	Type	Resolution (EP)	Asymmetry (EP)	Plates (EP)
n.a.	GlcNAc	n.a.	n.a.	n.a.	n.a.	n.a.	n.a.
n.a.	Citrate	n.a.	n.a.	n.a.	n.a.	n.a.	n.a.
n.a.	Glucose	n.a.	n.a.	n.a.	n.a.	n.a.	n.a.
n.a.	Galactose	n.a.	n.a.	n.a.	n.a.	n.a.	n.a.
n.a.	Fucose	n.a.	n.a.	n.a.	n.a.	n.a.	n.a.
1	Succinate RI	7,872	0,188	BMB*	5,15	0,97	9693
n.a.	Lactate RI	n.a.	n.a.	n.a.	n.a.	n.a.	n.a.
n.a.	glycerol	n.a.	n.a.	n.a.	n.a.	n.a.	n.a.
n.a.	Formate RI	n.a.	n.a.	n.a.	n.a.	n.a.	n.a.
2	Acetate RI	9,667	0,223	BMB	5,06	1,08	10422
n.a.	1,2 PDO RI	n.a.	n.a.	n.a.	n.a.	n.a.	n.a.
n.a.	1,3-PDO	n.a.	n.a.	n.a.	n.a.	n.a.	n.a.
3	Propionate RI	11,613	0,231	BMB*	n.a.	1,47	13983
n.a.	1,3-PDO	n.a.	n.a.	n.a.	n.a.	n.a.	n.a.
n.a.	2-3 BDO	n.a.	n.a.	n.a.	n.a.	n.a.	n.a.
n.a.	Ethanol	n.a.	n.a.	n.a.	n.a.	n.a.	n.a.
n.a.	Isobutyrate RI	n.a.	n.a.	n.a.	n.a.	n.a.	n.a.
n.a.	Butyrate RI	n.a.	n.a.	n.a.	n.a.	n.a.	n.a.

Chromatogram and SST Results

Injection Details

Injection Name:	**39 MUCHMO3 t48 r3**	*Run Time (min):*	**20,00**
Vial Number:	**3:E12**	*Injection Volume:*	**20,00**
Injection Type:	**Unknown**	*Channel:*	**RI_CH_1**
Calibration Level:		*Wavelength:*	**n.a.**
Instrument Method:	**Default method LC2030C 45 gr 20 min**	*Bandwidth:*	**n.a.**
Processing Method:	**Processing Method LC2030 45 gr**	*Dilution Factor:*	**1,0000**
Injection Date/Time:	**14-jun-23 10:25**	*Sample Weight:*	**1,0000**

Chromatogram

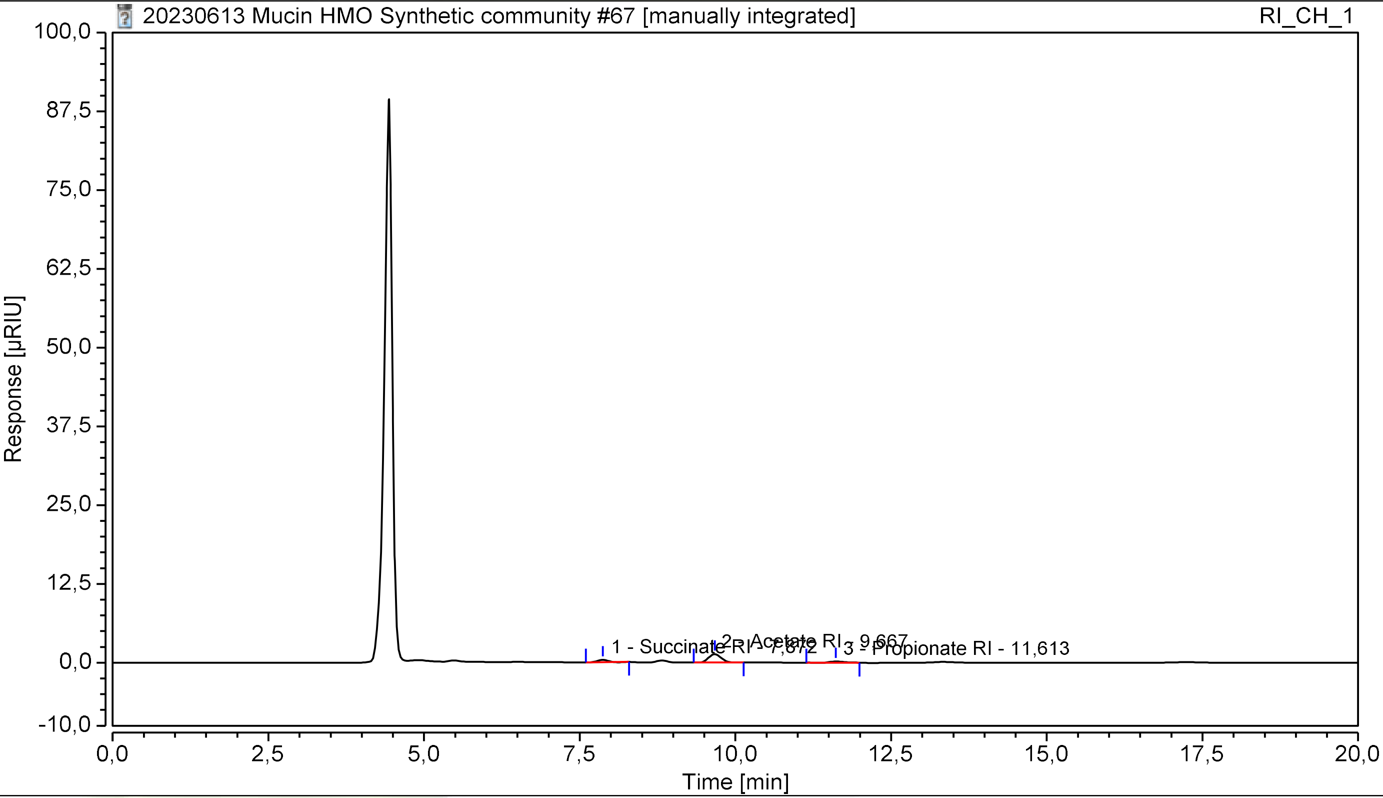

SST Results

No.	Name	Inj.Condition	Peak	Test Result	Injection

Number of executed test cases: **n.a.** **Total Result:** **Passed**

Chromeleon (c) Dionex
Version 7.2.10.25868

Chromatogram

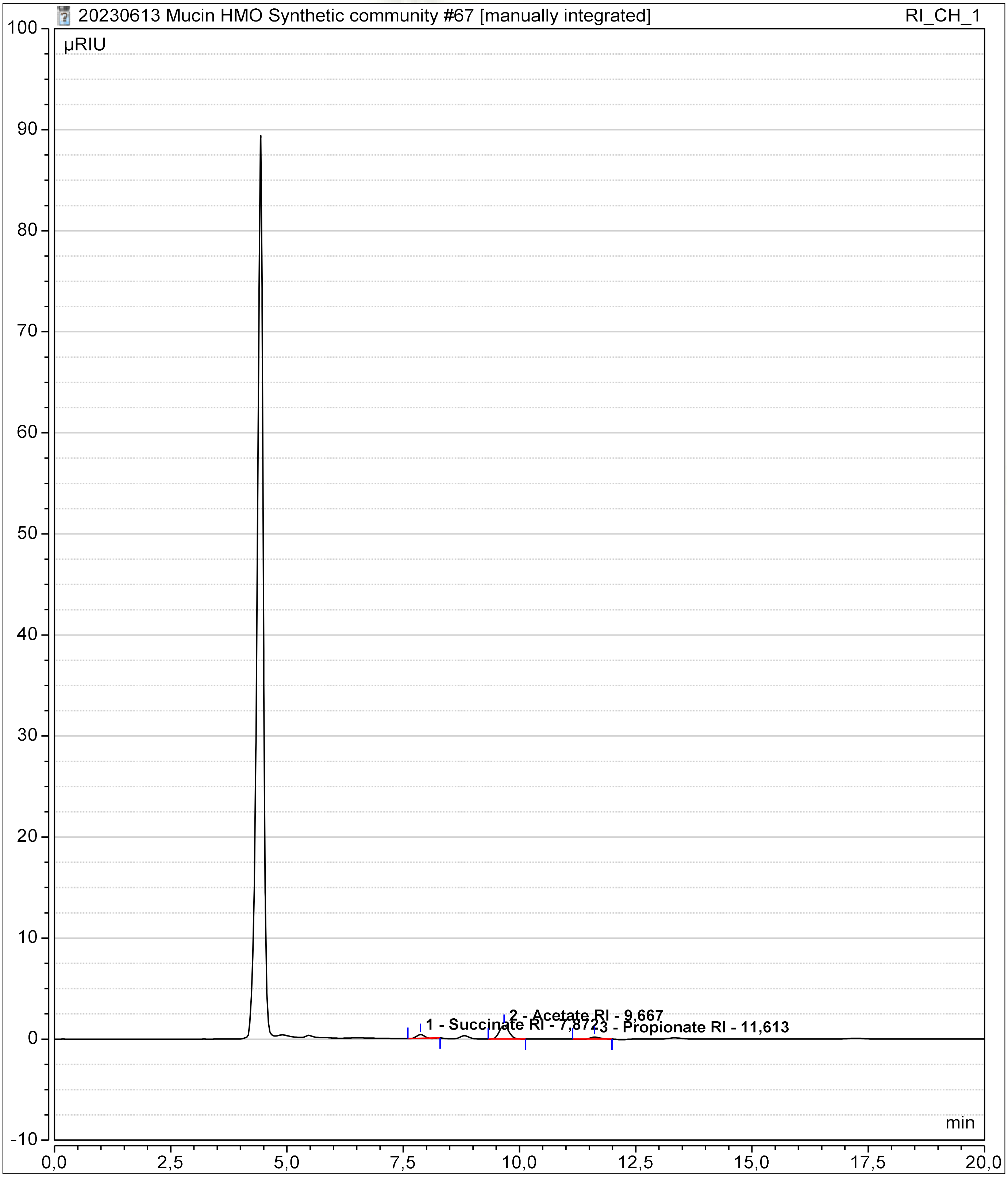

Chromeleon (c) Dionex
Version 7.2.10.25868

Chromatogram and Results

Injection Details

Injection Name:	**Shutdown**	*Run Time (min):*	**10,00**
Vial Number:	**3:F1**	*Injection Volume:*	**20,00**
Injection Type:	**Blank**	*Channel:*	**RI_CH_1**
Calibration Level:		*Wavelength:*	**n.a.**
Instrument Method:	**Default shutdown method LC2030C - 0.1 mLmin**	*Bandwidth:*	**n.a.**
Processing Method:	**Processing Method LC2030 45 gr**	*Dilution Factor:*	**1,0000**
Injection Date/Time:	**14-jun-23 10:49**	*Sample Weight:*	**1,0000**

Chromatogram

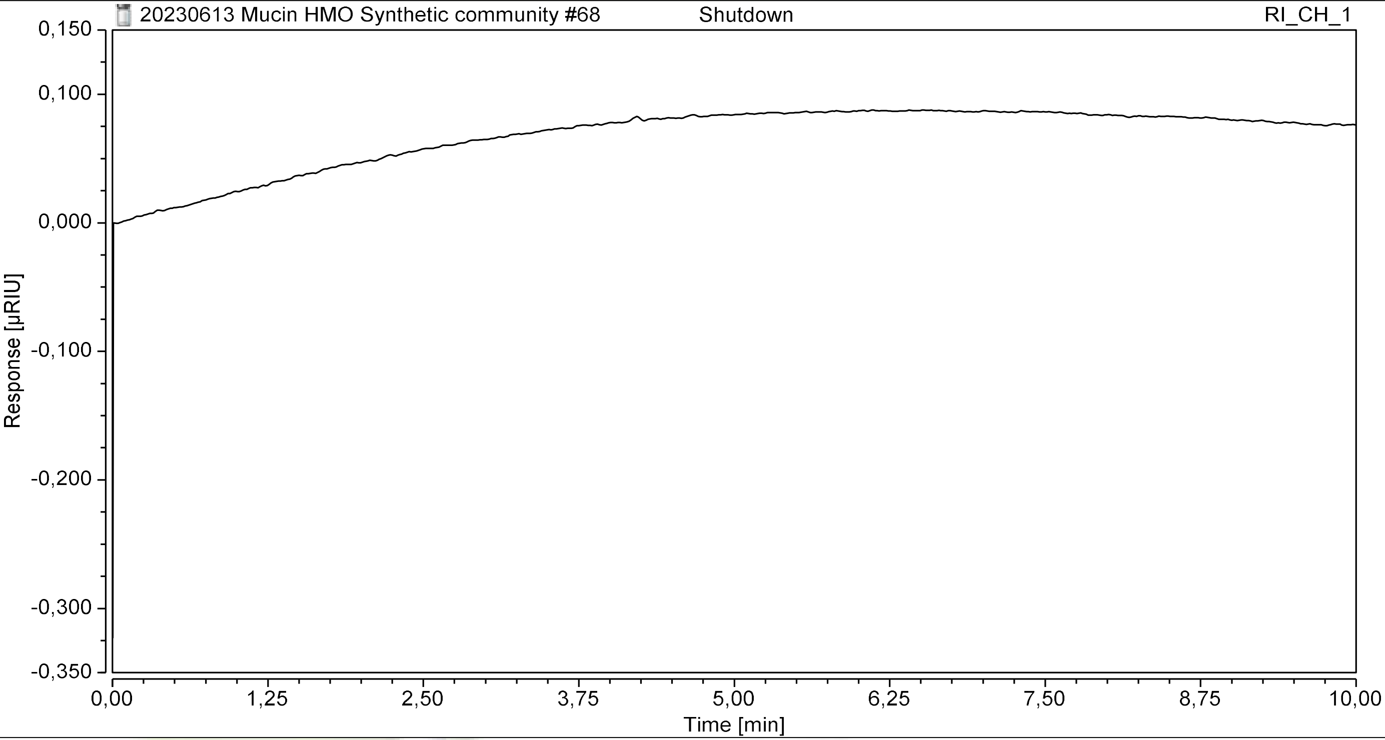

Integration Results

No.	Peak Name	Retention Time min	Area µRIU*min	Height µRIU	Relative Area %	Relative Height %	Amount
n.a.	GlcNAc	n.a.	n.a.	n.a.	n.a.	n.a.	n.a.
n.a.	Citrate	n.a.	n.a.	n.a.	n.a.	n.a.	n.a.
n.a.	Glucose	n.a.	n.a.	n.a.	n.a.	n.a.	n.a.
n.a.	Galactose	n.a.	n.a.	n.a.	n.a.	n.a.	n.a.
n.a.	Fucose	n.a.	n.a.	n.a.	n.a.	n.a.	n.a.
n.a.	Succinate RI	n.a.	n.a.	n.a.	n.a.	n.a.	n.a.
n.a.	Lactate RI	n.a.	n.a.	n.a.	n.a.	n.a.	n.a.
n.a.	glycerol	n.a.	n.a.	n.a.	n.a.	n.a.	n.a.
n.a.	Formate RI	n.a.	n.a.	n.a.	n.a.	n.a.	n.a.
n.a.	Acetate RI	n.a.	n.a.	n.a.	n.a.	n.a.	n.a.
n.a.	1,2 PDO RI	n.a.	n.a.	n.a.	n.a.	n.a.	n.a.
n.a.	1,3-PDO	n.a.	n.a.	n.a.	n.a.	n.a.	n.a.
n.a.	Propionate RI	n.a.	n.a.	n.a.	n.a.	n.a.	n.a.
n.a.	1,3-PDO	n.a.	n.a.	n.a.	n.a.	n.a.	n.a.
n.a.	2-3 BDO	n.a.	n.a.	n.a.	n.a.	n.a.	n.a.
n.a.	Ethanol	n.a.	n.a.	n.a.	n.a.	n.a.	n.a.
n.a.	Isobutyrate RI	n.a.	n.a.	n.a.	n.a.	n.a.	n.a.
n.a.	Butyrate RI	n.a.	n.a.	n.a.	n.a.	n.a.	n.a.
**Total:**			**0,000**	**0,000**	**0,00**	**0,00**	

Peak Analysis

Injection Details

Injection Name:	**Shutdown**	*Run Time (min):*	**10,00**
Vial Number:	**3:F1**	*Injection Volume:*	**20,00**
Injection Type:	**Blank**	*Channel:*	**RI_CH_1**
Calibration Level:		*Wavelength:*	**n.a.**
Instrument Method:	**Default shutdown method LC2030C - 0.1 m**	*Bandwidth:*	**n.a.**
Processing Method:	**Processing Method LC2030 45 gr**	*Dilution Factor:*	**1,0000**
Injection Date/Time:	**14-jun-23 10:49**	*Sample Weight:*	**1,0000**

Chromatogram

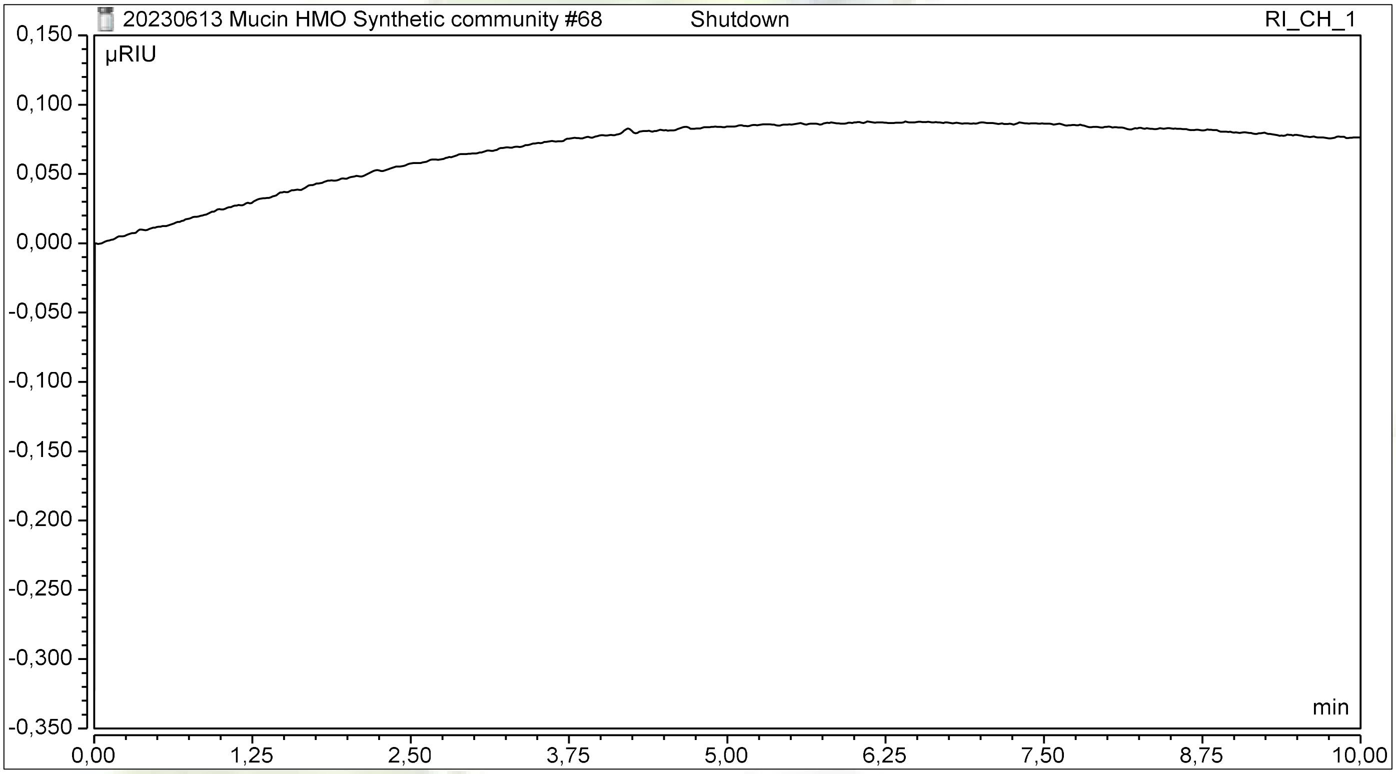

Peak Results

No.	Peak Name	Retention Time min	Width (50%) min	Type	Resolution (EP)	Asymmetry (EP)	Plates (EP)
n.a.	GlcNAc	n.a.	n.a.	n.a.	n.a.	n.a.	n.a.
n.a.	Citrate	n.a.	n.a.	n.a.	n.a.	n.a.	n.a.
n.a.	Glucose	n.a.	n.a.	n.a.	n.a.	n.a.	n.a.
n.a.	Galactose	n.a.	n.a.	n.a.	n.a.	n.a.	n.a.
n.a.	Fucose	n.a.	n.a.	n.a.	n.a.	n.a.	n.a.
n.a.	Succinate RI	n.a.	n.a.	n.a.	n.a.	n.a.	n.a.
n.a.	Lactate RI	n.a.	n.a.	n.a.	n.a.	n.a.	n.a.
n.a.	glycerol	n.a.	n.a.	n.a.	n.a.	n.a.	n.a.
n.a.	Formate RI	n.a.	n.a.	n.a.	n.a.	n.a.	n.a.
n.a.	Acetate RI	n.a.	n.a.	n.a.	n.a.	n.a.	n.a.
n.a.	1,2 PDO RI	n.a.	n.a.	n.a.	n.a.	n.a.	n.a.
n.a.	1,3-PDO	n.a.	n.a.	n.a.	n.a.	n.a.	n.a.
n.a.	Propionate RI	n.a.	n.a.	n.a.	n.a.	n.a.	n.a.
n.a.	1,3-PDO	n.a.	n.a.	n.a.	n.a.	n.a.	n.a.
n.a.	2-3 BDO	n.a.	n.a.	n.a.	n.a.	n.a.	n.a.
n.a.	Ethanol	n.a.	n.a.	n.a.	n.a.	n.a.	n.a.
n.a.	Isobutyrate RI	n.a.	n.a.	n.a.	n.a.	n.a.	n.a.
n.a.	Butyrate RI	n.a.	n.a.	n.a.	n.a.	n.a.	n.a.

Chromatogram and SST Results

Injection Details

Injection Name:	**Shutdown**	*Run Time (min):*	**10,00**
Vial Number:	**3:F1**	*Injection Volume:*	**20,00**
Injection Type:	**Blank**	*Channel:*	**RI_CH_1**
Calibration Level:		*Wavelength:*	**n.a.**
Instrument Method:	**Default shutdown method LC2030C - 0.1 mLmin**	*Bandwidth:*	**n.a.**
Processing Method:	**Processing Method LC2030 45 gr**	*Dilution Factor:*	**1,0000**
Injection Date/Time:	**14-jun-23 10:49**	*Sample Weight:*	**1,0000**

Chromatogram

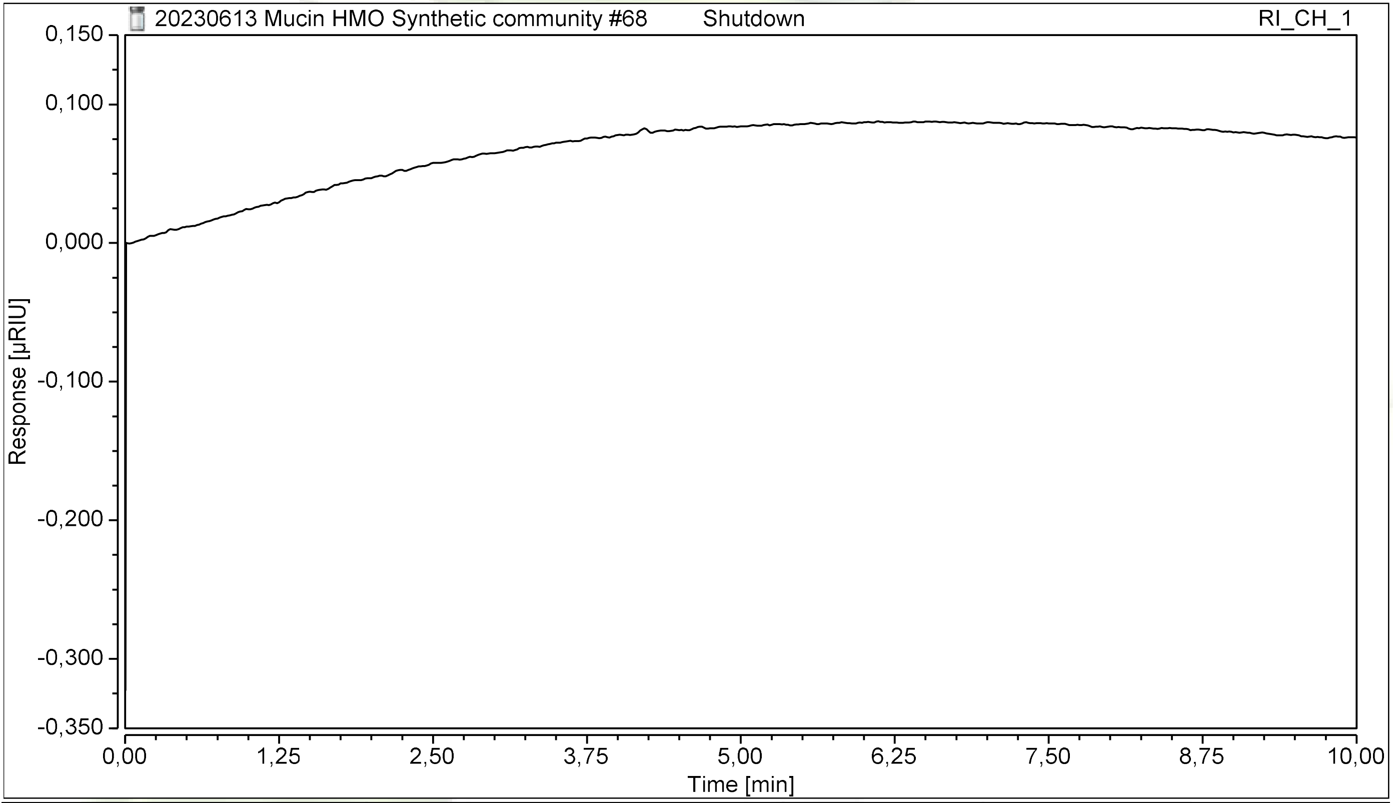

SST Results

No.	Name	Inj.Condition	Peak	Test Result	Injection

Number of executed test cases: n.a. **Total Result:** Passed

Summary

Sequence Details

Name:	**20230613 Mucin HMO Synthetic community**	*Created On:*	**08-dec-17 07:28:29**
Directory:	**HPLC-DATA\8_LC-2030C\Data2023\Maryse\20**	*Created By:*	**HPLC install**
Data Vault:	**AZR_CHROM_DATA_MIB-SSB**	*Updated On:*	**13-sep-23 13:52:19**
No. of Injections:	**68**	*Updated By:*	**mib007**

By Component **Lactate RI**

No.	Injection Name	Ret.Time min RI_CH_1 Lactate RI	Area µRIU*min RI_CH_1 Lactate RI	Height µRIU RI_CH_1 Lactate RI	Amount RI_CH_1 Lactate RI	Rel.Area % RI_CH_1 Lactate RI	Peak Type RI_CH_1 Lactate RI
1	water 1	n.a.	n.a.	n.a.	n.a.	n.a.	n.a.
2	no injection	n.a.	n.a.	n.a.	n.a.	n.a.	n.a.
3	VFA 10	8,257	0,331	1,545	9,609	23,00	BMB
4	VFA 20	8,260	0,684	3,162	19,861	23,03	BM
5	VFA 30	8,260	1,041	4,810	30,223	22,99	BM
6	1,2-Prop & 1- propane 10	n.a.	n.a.	n.a.	n.a.	n.a.	n.a.
7	1,2-Prop & 1- propane 20	n.a.	n.a.	n.a.	n.a.	n.a.	n.a.
8	1,2-Prop & 1- propane 30	n.a.	n.a.	n.a.	n.a.	n.a.	n.a.
9	Meth & Eth 100	n.a.	n.a.	n.a.	n.a.	n.a.	n.a.
10	Meth & Eth 200	n.a.	n.a.	n.a.	n.a.	n.a.	n.a.
11	Meth & Eth 300	n.a.	n.a.	n.a.	n.a.	n.a.	n.a.
12	16.93 acetate	n.a.	n.a.	n.a.	n.a.	n.a.	n.a.
13	13.68 propionate	n.a.	n.a.	n.a.	n.a.	n.a.	n.a.
14	40 5HMO1 t24 r1	8,272	0,020	0,111	0,583	1,45	Rd
15	41 5HMO1 t24 r2	8,265	0,060	0,315	1,738	7,06	BMB*
16	42 5HMO1 t24 r3	8,268	0,053	0,285	1,532	5,69	BMB*
17	43 5HMO1 t48 r1	8,263	0,014	0,114	0,415	1,44	BMB*
18	44 5HMO1 t48 r2	8,267	0,070	0,359	2,035	7,40	BMB*
19	45 5HMO1 t48 r3	8,275	0,057	0,336	1,650	5,92	BMB*
20	46 5HMO1 t72 r1	8,260	0,008	0,047	0,232	0,73	BMB*
21	47 5HMO1 t72 r2	8,263	0,064	0,331	1,859	6,78	BMB*
22	48 5HMO1 t72 r3	8,263	0,013	0,089	0,369	1,37	BMB*
23	49 5HMO1 t96 r1	8,267	0,015	0,084	0,444	1,61	Rd
24	50 5HMO1 t96 r2	8,262	0,029	0,174	0,842	2,97	BMB*
25	51 5HMO1 t96 r3	8,263	0,038	0,234	1,094	3,80	BMB*
26	52 5HMO1 t120 r1	8,262	0,006	0,038	0,167	0,60	BMB*
27	53 5HMO1 t120 r2	8,265	0,035	0,207	1,003	3,57	BMB*
28	54 5HMO1 t120 r3	8,263	0,025	0,152	0,735	2,31	BMB*
29	10 GOSFOS t24 r1	8,268	0,008	0,046	0,234	1,12	Rd
30	11 GOSFOS t24 r2	8,263	0,005	0,050	0,158	0,78	BMB*
31	12 GOSFOS t24 r3	8,270	0,011	0,063	0,325	1,42	Rd
32	16 GOSFOSEXTR t24 r1	8,262	0,001	0,012	0,035	0,13	BMB*
33	17 GOSFOSEXTR t24 r2	8,263	0,014	0,087	0,401	1,89	BMB*
34	18 GOSFOSEXTR t24 r3	8,273	0,009	0,050	0,270	1,03	Rd
35	25 GOSFOS t48 r1	n.a.	n.a.	n.a.	n.a.	n.a.	n.a.
36	26 GOSFOS t48 r2	8,270	0,010	0,055	0,283	1,36	Rd
37	27 GOSFOS t48 r3	8,262	0,003	0,018	0,073	0,33	BMB*
38	31 GOSFOSEXTR t48 r1	n.a.	n.a.	n.a.	n.a.	n.a.	n.a.
39	32 GOSFOSEXTR t48 r2	8,265	0,022	0,134	0,633	2,51	BMB*
40	33 GOSFOSEXTR t48 r3	8,275	0,009	0,051	0,274	1,12	Rd
41	1 MUCHMO1 t48 r1	n.a.	n.a.	n.a.	n.a.	n.a.	n.a.
42	2 MUCHMO1 t48 r2	n.a.	n.a.	n.a.	n.a.	n.a.	n.a.
43	3 MUCHMO1 t48 r3	n.a.	n.a.	n.a.	n.a.	n.a.	n.a.

44	4 MUCHMO2 t24 r1	n.a.	n.a.	n.a.	n.a.	n.a.	n.a.
45	5 MUCHMO2 t24 r2	n.a.	n.a.	n.a.	n.a.	n.a.	n.a.
46	6 MUCHMO2 t24 r3	n.a.	n.a.	n.a.	n.a.	n.a.	n.a.
47	7 MUC t24 r1	n.a.	n.a.	n.a.	n.a.	n.a.	n.a.
48	8 MUC t24 r2	n.a.	n.a.	n.a.	n.a.	n.a.	n.a.
49	9 MUC t24 r3	n.a.	n.a.	n.a.	n.a.	n.a.	n.a.
50	13 GOSFOSMUC t24 r1	n.a.	n.a.	n.a.	n.a.	n.a.	n.a.
51	14 GOSFOSMUC t24 r2	n.a.	n.a.	n.a.	n.a.	n.a.	n.a.
52	15 GOSFOSMUC t24 r3	n.a.	n.a.	n.a.	n.a.	n.a.	n.a.
53	19 MUCHMO2 t48 r1	n.a.	n.a.	n.a.	n.a.	n.a.	n.a.
54	20 MUCHMO2 t48 r2	n.a.	n.a.	n.a.	n.a.	n.a.	n.a.
55	21 MUCHMO2 t48 r3	n.a.	n.a.	n.a.	n.a.	n.a.	n.a.
56	22 MUC t48 r1	n.a.	n.a.	n.a.	n.a.	n.a.	n.a.
57	23 MUC t48 r2	n.a.	n.a.	n.a.	n.a.	n.a.	n.a.
58	24 MUC t48 r3	n.a.	n.a.	n.a.	n.a.	n.a.	n.a.
59	28 GOSFOSMUC t48 r1	n.a.	n.a.	n.a.	n.a.	n.a.	n.a.
60	29 GOSFOSMUC t48 r2	n.a.	n.a.	n.a.	n.a.	n.a.	n.a.
61	30 GOSFOSMUC t48 r3	n.a.	n.a.	n.a.	n.a.	n.a.	n.a.
62	34 MUCHMO3 t24 r1	n.a.	n.a.	n.a.	n.a.	n.a.	n.a.
63	35 MUCHMO3 t24 r2	n.a.	n.a.	n.a.	n.a.	n.a.	n.a.
64	36 MUCHMO3 t24 r3	n.a.	n.a.	n.a.	n.a.	n.a.	n.a.
65	37 MUCHMO3 t48 r1	n.a.	n.a.	n.a.	n.a.	n.a.	n.a.
66	38 MUCHMO3 t48 r2	n.a.	n.a.	n.a.	n.a.	n.a.	n.a.
67	39 MUCHMO3 t48 r3	n.a.	n.a.	n.a.	n.a.	n.a.	n.a.
68	Shutdown	n.a.	n.a.	n.a.	n.a.	n.a.	n.a.

Chromatogram

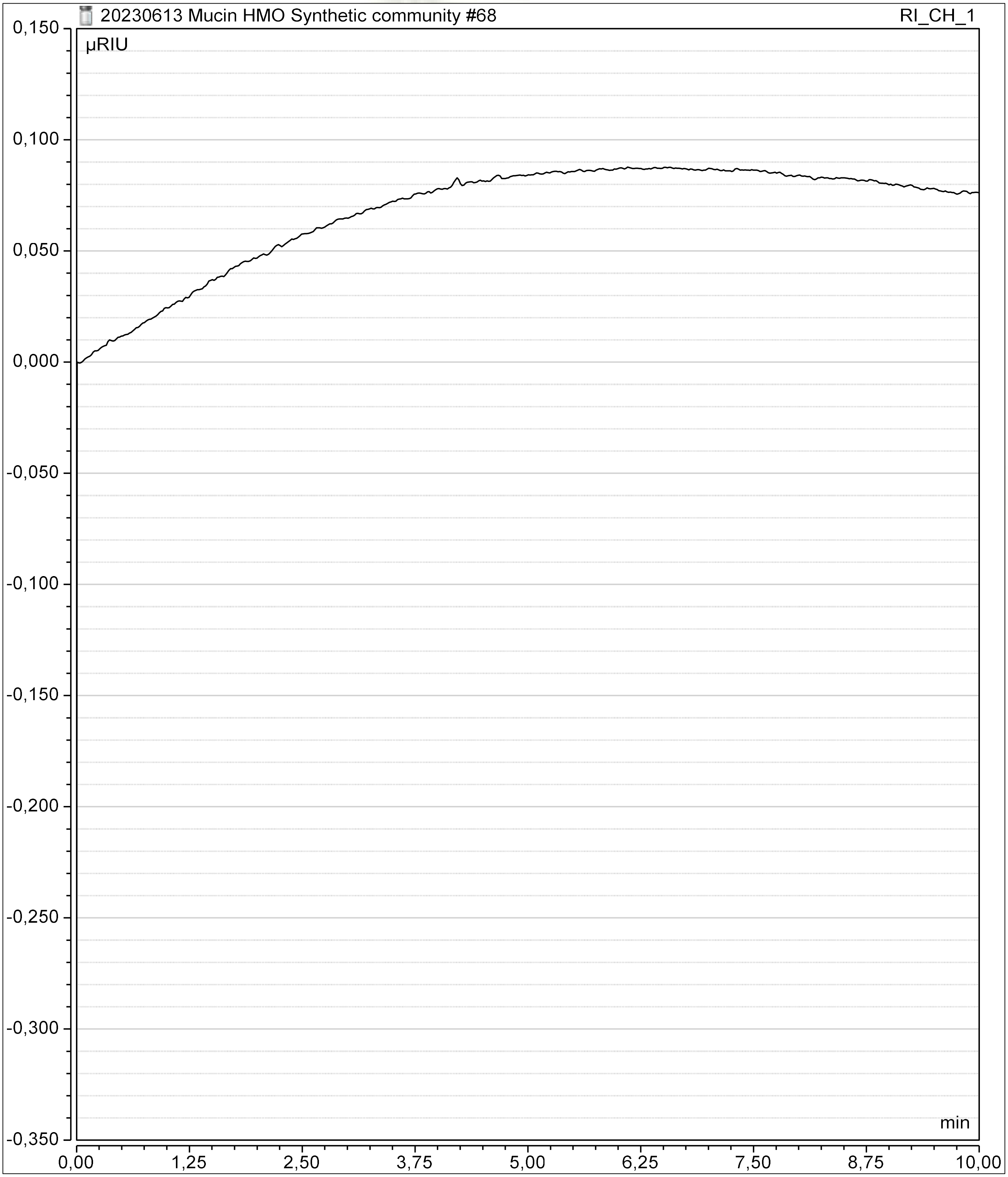

Chromeleon (c) Dionex
Version 7.2.10.25868
